# Genome resequencing and transcriptome profiling reveal structural diversity and expression patterns of constitutive disease resistance genes in Huanglongbing-tolerant *Poncirus trifoliata* and its hybrids

**DOI:** 10.1038/hortres.2017.64

**Published:** 2017-11-15

**Authors:** Nidhi Rawat, Brajendra Kumar, Ute Albrecht, Dongliang Du, Ming Huang, Qibin Yu, Yi Zhang, Yong-Ping Duan, Kim D Bowman, Fred G Gmitter, Zhanao Deng

**Affiliations:** 1 University of Florida, IFAS, Gulf Coast Research and Education Center, Wimauma, FL, USA; 2 Ocimum BioSolutions Ltd., Royal Demeure, Plot no. 12/2, Sector- 1, HUDA Techno Enclave, Madhapur, Hyderabad, India; 3 University of Florida, IFAS, Southwest Florida Research and Education Center, Immokalee, FL, USA; 4 University of Florida, IFAS, Citrus Research and Education Center, Lake Alfred, FL, USA; 5 U.S. Horticultural Research Laboratory, Agricultural Research Service, U.S. Department of Agriculture, Fort Pierce, FL, USA

## Abstract

Huanglongbing (HLB) is the most destructive bacterial disease of citrus worldwide. While most citrus varieties are susceptible to HLB, *Poncirus trifoliata,* a close relative of *Citrus*, and some of its hybrids with *Citrus* are tolerant to HLB. No specific HLB tolerance genes have been identified in *P. trifoliata* but recent studies have shown that constitutive disease resistance *(CDR)* genes were expressed at much higher levels in HLB-tolerant *Poncirus* hybrids and the expression of *CDR* genes was modulated by *Candidatus* Liberibacter asiaticus (*C*Las), the pathogen of HLB. The current study was undertaken to mine and characterize the *CDR* gene family in *Citrus* and *Poncirus* and to understand its association with HLB tolerance in *Poncirus*. We identified 17 *CDR* genes in two citrus genomes, deduced their structures, and investigated their phylogenetic relationships. We revealed that the expansion of the *CDR* family in *Citrus* seems to be due to segmental and tandem duplication events. Through genome resequencing and transcriptome sequencing, we identified eight *CDR* genes in the *Poncirus* genome (*PtCDR1*-*PtCDR8*). The number of SNPs was the highest in *PtCDR2* and the lowest in *PtCDR7*. Most of the deletion and insertion events were observed in the UTR regions of *Citrus* and *Poncirus CDR* genes. *PtCDR2* and *PtCDR8* were in abundance in the leaf transcriptomes of two HLB-tolerant *Poncirus* genotypes and were also upregulated in HLB-tolerant, *Poncirus* hybrids as revealed by real-time PCR analysis. These two *CDR* genes seem to be good candidate genes for future studies of their role in citrus-*C*Las interactions.

## Introduction

Citrus (*Citrus* L.) is a widely grown and nutritious fruit, with oranges, grapefruit, lemons, limes, and tangerines among the well-known citrus varieties worldwide.^1^ China, Brazil, United States (U.S.), India, Mexico, and Spain are the world's leading citrus fruit-producing countries, representing close to two-thirds of the global citrus production.^2^ In the U.S., a total of 9.02 million metric tons of citrus production was reported in the 2014–2015 season, with Florida as the leading citrus-producing state (56%), followed by California (41%), Texas, and Arizona.^3^

Huanglongbing (HLB) has become the most serious recent disease threat to the U.S. citrus industry.^4,5^ HLB was first reported in Florida in 2005 and has caused a cumulative loss of more than $2.9 billion in grower revenues between 2006–2007 and 2013–2014 (Florida Citrus Commission; http://www.floridacitrus.org/). HLB resulted in an average annual loss of more than $975 million in the Florida citrus industry output. HLB, also known as citrus greening, is presumably caused by the phloem-limited, gram-negative bacterium *Candidatus* Liberibacter asiaticus (*C*Las).^6^ Three Liberibacter species are known to cause HLB; among them, *C*Las is the most widespread species. While *C*Las is heat-tolerant, the African and American forms of Liberibacter are heat-sensitive. *Ca.* L. africanus is presently primarily distributed in Africa,^7^ and *Ca.* L. americanus has been found only in Brazil.^8^ The bacterium *C*Las is vectored by the Asian citrus psyllid (ACP; *Diaphorina citri*).^9^ ACPs also damage citrus directly by feeding on new citrus growth, resulting in twisted and curled leaves and shoots.^10^ Typical symptom of HLB in citrus is the asymmetrical pattern of blotchy yellowing or mottling on leaves. As the disease progresses, citrus fruit becomes smaller, and juice quality declines. Diseased mature fruit may remain partially green, which is why HLB is also called citrus greening. The fruit can become lopsided with dark aborted seeds.^11,12^ At present, there are no effective control methods for HLB. *C*Las attacks all important commercial varieties of citrus, including oranges, grapefruit, lemons, and tangerines. Sweet oranges, grapefruit, and mandarins are highly susceptible to HLB.^4,13,14^

Several studies have indicated that *Poncirus trifoliata* (L.) Raf., a close relative of *Citrus,* and some of its hybrids are tolerant to HLB.^15–18^ *Poncirus trifoliata* is sexually compatible with *Citrus* and has been widely used as a breeding parent in developing new citrus rootstock cultivars.^19,20^ It carries valuable genes for improving the resistance of citrus scion cultivars to biotic (for example, citrus tristeza virus) and abiotic stresses.^21,22^ In recent studies, several previously released *Poncirus* and *Citrus* hybrids, such as ‘US-897’ (*C. reticulata* L. Blanco Cleopatra × *P. trifoliata* Flying Dragon) and ‘US-942’ (*C. reticulata* Sunki×*P. trifoliata* Flying Dragon), have proven to be HLB-tolerant, even though these hybrids were not initially selected for HLB tolerance.^15–17^ It is expected that *P. trifoliata* will become a major source of valuable genes for use in sexual hybridization, somatic hybridization, and cisgenic approaches to improving *Citrus* toward resistance to HLB.^20^

Creating HLB-resistant/tolerant citrus cultivars is considered the most economic, environment-friendly approach for long-term management of HLB.^23^ However, it is very challenging to achieve this goal solely via conventional breeding due to the complex reproductive biology and long periods of juvenility.^24^ As has been demonstrated in major agronomic crops, integrating genomics, transcriptomics, proteomics, and metabolomics can facilitate the identification of differentially expressed genes as candidates for genetic transformation and for use in marker-assisted early selection of disease-resistant progeny in conventional breeding, and can provide much deeper insight about the complex interactions between the host plant citrus and the pathogen *C*Las. Mining large datasets from whole genome sequencing and transcriptome sequencing projects is an invaluable tool that can assist in the identification of plant resistance and defense-related genes. Several reports had been published on the identification of HLB-responsive genes in citrus using transcriptomic, proteomic, and metabolomic approaches.^25–30^ Recently, a meta-analysis and a gene-network co-expression analysis were conducted with published reports, suggesting constitutive disease resistance 1 genes (*CDR1*) as potential candidate genes for HLB resistance/tolerance in *Poncirus*.^15,31,32^ *CDR1* was first identified and cloned in *Arabidopsis*. Its product has been implicated in disease resistance signaling.^33^ Overexpression of a rice (*Oryza sativa* L.) *CDR1* gene, *OsCDR1*, led to constitutive activation of defense response and enhanced resistance in rice and *Arabidopsis* against bacterial and fungal pathogens.^34^

*CDR* genes belong to the plant aspartic proteinase (APs) gene family. Plant APs are widely distributed in the plant kingdom,^35^ and the majority of plant APs identified so far are translated as single-chain preproenzymes, which are subsequently converted to mature enzymes with single or two chains.^36^ Plant APs have a ‘typical’ precursor of ~100 amino acids in the protein, also known as the plant-specific insert, which is removed upon activation into the mature form of the enzymes.^37^ Plant APs are classified into three groups: typical APs, nucellin-like APs, and atypical APs.^38^ CDRs are apoplastic enzymes which belong to the ‘atypical’ plant APs. Atypical APs do not contain plant-specific inserts and display intermediate features between the typical and nucellin-like sequences.^38^ Thus far, only a small number of atypical and nucellin-like APs have been studied, such as nucellin and PCS1 involved in cell death regulation,^39^ CND41 involved in nitrogen remobilization,^40^ and CDR1 involved in disease resistance.^33,34^ Recently PvNod41, an AP from common bean (*Phaseolus vulgaris*) and closely related to CDR1, has been identified as a major player during plant defense and nodule development.^41^

The potential roles of *CDR1* genes in HLB resistance/tolerance were first recognized in a microarray-based genome-wide gene expression study.^15^ In a subsequent study comparing *CDR1* expression in *C*Las or citrus tristeza virus infection,^17^ one *CDR1* ortholog was constitutively expressed at much higher levels in the HLB-tolerant *Poncirus* hybrids ‘US-897’ and ‘US-942’ than in HLB-susceptible Cleopatra mandarin (*C. reticulata*), while a second form of *CDR1* was induced in Cleopatra after *C*Las infection to a level similar to that found in non-inoculated ‘US-897’ and ‘US-942’.

The present study is focused on genome-wide identification and analysis of *CDR* genes in *Citrus* and *Poncirus* genome and transcriptome sequences. The genomic organization, phylogenetic relationship, protein domain architecture and exon/intron junctions of the identified citrus *CDR* genes were comprehensively analyzed. Given the critical roles of plant *CDR* genes in defense, *CDR* gene variants in HLB-susceptible and HLB-tolerant *Citrus* and *Poncirus* genotypes are of great interest. Publicly available genome sequences are only available for HLB-susceptible sweet orange (*C. sinensis*) and Clementine mandarin (*C. ×clementina*), therefore we resequenced the genome of two *P. trifoliata* accessions (DPI 50-7 and Flying Dragon (FD)) and two *Poncirus* hybrid cultivars ‘US-897’ and ‘US-812’, sequenced the leaf transcriptome of the two *P. trifoliata* accessions DPI 50-7 and Flying Dragon, and identified the genetic variation in *Citrus* and *Poncirus CDR* genes. We analyzed the expression of the entire *CDR* gene family in HLB-susceptible and HLB-tolerant genotypes after *C*Las infection and obtained information about the response of each copy of *CDR* genes to HLB.

## Materials and methods

### Identification of *CDR* genes in *Citrus* genomes

We created an in-house MySQL database of the two citrus genomes: *Citrus *×*clementina v 1.0* and *Citrus sinensis* at https://phytozome.jgi.doe.gov/. *CDR* genes were identified using keyword searches such as ‘constitutive disease resistance’ and ‘aspartic protease family protein’, and the identified *CDR* gene sequences were retrieved. BLASTP (http://blast.ncbi.nlm.nih.gov/Blast.cgi) was conducted to identify *CDR* gene family members using the amino-acid sequence of previously characterized *Arabidopsis CDR1* (NP_198319).^33^ We also downloaded the *Poncirus* EST unigene database (https://www.citrusgenomedb.org/species/trifoliata/unigene1.0) and searched for *CDR* gene-related ESTs using BLASTP. *CDR* genes with BLASTP search score values⩾100 and e-value⩽e ^-10^ were used for further analysis. The aspartic protease domain (ASP) (PF00026) was downloaded from the Pfam database (http://pfam.sanger.ac.uk/). To exclude overlapping genes, all putative *CDR* genes were aligned using ClustalW.^42^ The ASP domain was then manually checked for each identified *CDR* gene.

### *CDR* sequence alignment and phylogenetic analysis

The structural divergence among the identified *CDR* genes was examined by investigating the conserved motif in the encoded CDR proteins. The complete amino-acid sequences of *CDR* genes were subjected to MEME analysis online (http://meme.nbcr.net/meme). The subcellular localization of CDR proteins was predicted by subCELlular LOcalization predictor (CELLO v.2.5; http://cello.Life.nctu.edu.tw/). *CDR* genes were aligned using ClustalW.^42^ Phylogenetic trees were constructed with the PhyML software plugin in Geneious software (http://www.geneious.com/) using the neighbor-joining (NJ) method, and the bootstrap test was replicated 1000 times.

### Domain and gene structure analysis

The Pfam domain and signal peptide of *CDR* genes were predicted using the conserved domain database (http://www.ncbi.nlm.nih.gov/Structure/cdd/wrpsb.cgi) with default settings. The results were confirmed using CDART search (http://www.ncbi.nlm.nih.gov/Structure/lexington/lexington.cgi). Diagrams of protein structures were constructed with the DOG 1.0 software (http://dog.biocuckoo.org/). The cDNA and DNA sequences of *CDR* genes were retrieved from the citrus genome databases (see above) and diagrams of exon/intron structures of *CDR* genes were illustrated using the online Gene Structure Display Server (http://gsds.cbi.pku.edu.cn/). Physical locations (scaffold) of these *CDR* genes in the citrus genomes were identified.

### Plant material, DNA and RNA isolation and HiSeq sequencing

Two HLB-tolerant *P. trifoliata* accessions DPI 50-7 and Flying Dragon were selected for whole genome resequencing and leaf transcriptome sequencing. The accession Flying Dragon was the source of HLB tolerance in *Poncirus* hybrids including ‘US-897’ and ‘US-942’.^15–17^ The accession DPI 50-7 (previously labeled as DPI 9-6) is similar to Flying Dragon in HLB tolerance, but it is morphologically and isozymically distinct from Flying Dragon.^21^ Two HLB-tolerant *Poncirus* hybrids ‘US-897’ and ‘US-812’ (*C. reticulata* Sunki×*P. trifoliata* Benecke) were also selected for whole genome resequencing. Leaf samples were collected from two different sources. The first source of plants was grown under disease-free conditions in the greenhouse at the Chiefland Budwood Foundation, Bureau of Citrus Budwood Registration, Division of Plant Industry, Florida Department of Agriculture and Consumer Services, Chiefland, Florida. The second source of DPI 50-7 and Flying Dragon plants was grown in the orchard at the University of Florida’s Citrus Research and Education Center in Lake Alfred, Florida, and had been exposed to *C*Las through natural psyllid transmission. Leaves from both plant sources were collected in April 2015. Leaf tissues were immediately frozen in liquid nitrogen and stored at −80 °C. Genomic DNA was extracted from the leaves using the CTAB method^43^ and treated with RNase I (Qiagen, Hilden, Germany). The isolated DNA was shipped to Novogene Corporation (Beijing, China) where the DNA was fragmented and sequenced on an Illumina HiSeq 2500 platform (Illumina, Inc., San Diego, CA, USA) following Illumina protocols, to produce a paired-end library of sequence reads for each sample. Total RNA was isolated from leaves using the RNeasy plant isolation kit (Qiagen, Germany). RNA samples were shipped to Novogene Corporation where mRNA were enriched and converted into cDNAs, which were sequenced on an Illumina HiSeq 2500 using the Illumina ‘TruSeq RNA-Seq Sample Prep kit.’

### Identification of *CDR* genes in *Poncirus* genomes and variant calling in resequenced genomic and transcriptomic sequencing data

Illumina shotgun DNA sequence reads were mapped to the annotated citrus genome assembly *C. ×clementina v1.0* using Burrows-Wheeler Aligner (BWA) v0.7.12 software (for resequenced genomes) (bio-bwa.sourceforge.net; La Jolla, CA, USA) and TopHat v2.1.0 (for transcriptomes), respectively. Prior to read mapping, sequence reads were trimmed to remove sequence tags and filtered for low-quality reads based on the distribution of Phred-like scores in each sequencing cycle using trim galore (http://www.bioinformatics.babraham.ac.uk/projects/trim_galore/). Sequence reads of the four resequenced genomes were separately mapped to the annotated reference citrus genome assembly using BWA-mem software with default parameters.^44^ TopHat v2.1.0 (ref. 45) was run on RNA-Seq reads with default parameters; a citrus gene model annotation file in the GFF3 format was used to enable Bowtie2 v2.2.8 to first align transcript sequences to the transcriptome and then to map only unmapped reads to the genome. Single-nucleotide polymorphisms (SNP) in the assembled reads (DNA-Seq and RNA-Seq) were identified using SAMtools^46^ and Geneious software, with a minimum Phred quality score of 20. DNA resequencing data are available in the NCBI Sequence Read Archive repository (Acc. #SRP096286).

### Identification of *CDR-*related ESTs from the *Poncirus* EST database

*Poncirus* ESTs were downloaded from the citrus genome database (https://www.citrusgenomedb.org/species/trifoliata/unigene1.0). The *Arabidopsis CDR1* gene sequence was used in BLASTP searches against the EST database to identify *CDR* genes-related ESTs with an *E* value<1e^−10^. *Poncirus* ESTs (contigs) matching with *CDR* genes were manually checked for the presence of the ASP domain. RNA-Seq reads of DPI 50-7 and Flying Dragon were mapped to the identified *CDR-*related contigs using the TopHat (v2.1.0) and Bowtie2 (2.2.8) programs with default parameters.^47^

### Estimation of the expression level of *CDR* genes in RNA-Seq data

The expression level and transcript abundance of *CDR* genes in the *Poncirus* transcriptomes were determined with Cufflinks v2.0.2. Changes in the abundance of *CDR* transcripts between two sources of *Poncirus* samples were estimated using the Cuffdiff program. FPKM (Fragments per Kilobase of exon per Million fragments mapped) were calculated for each *CDR* gene, but only transcripts showing an FPKM>1 were reported (Supplementary Table 1).

### RNA isolation and real-time PCR

For real-time PCR analysis of *C*Las infected and non-infected plants, three plants each of greenhouse-grown two year-old seedlings of six genotypes were used: ‘US-812’, ‘US-897’, and ‘US-942’ (HLB-tolerant), and Valencia sweet orange (*C*. *sinensis*), Duncan grapefruit (*C*. *paradisi*), and Ruby Red grapefruit (*C*. *paradisi*) (HLB-susceptible). Plants were inoculated in June 2013, by grafting with *C*Las-infected budwood and were maintained in the U.S. Horticultural Research Laboratory greenhouses in Fort Pierce, Florida. The inoculum source used for *C*Las infection was tested to be negative for citrus tristeza virus (CTV) infection. Infection with *C*Las was verified via PCR analysis as described.^29^ Total RNA was isolated from leaves using the RNeasy plant kit (Qiagen). Total RNA was treated with DNase to remove potential residual DNA and converted to cDNA using the Superscript III kit (Invitrogen, Carlsbad, CA, USA). We designed 16 sets of gene-specific primers (Table 1) and also used the previously reported CDR primers^15^ for PCR amplification which we named UaCDR1 and UaCDR2 in the present study. Real-time PCR was carried out using the AriaMx system (Agilent Technologies, Santa Clara, CA, USA) and the SYBR green chemistry. Relative gene expression levels were calculated using the 2^−ΔΔCT^ method.^48^ All real-time PCR experiments were conducted with three biological and two technical replicates. Each reaction was carried out in triplicate with a reaction volume of 20 μL containing 0.5 μL (10 μm) of each gene-specific primers (Table 1), 1.0 μl of cDNA (20 ng/μl), 10 μl of SYBR green (1X buffer), and 8 μl autoclaved deionized water. The PCR cycling parameters were 95 °C for 30 s, followed by 40 cycles of 95 °C for 5 s and 60 °C for 30 s.

## Results

### Identification of 17 *CDR* genes in *Citrus* genomes

A total of 17 *CDR* genes were identified in two *Citrus* genomes: nine from the *C. ×clementina* genome and eight from the *C. sinensis* genome. The nomenclature system for *CDR* genes in the present study provisionally uses the names *CcCDR1 to CcCDR9* for *C. ×clementina CDR* genes and *CsCDR1 to CsCDR8* for *C. sinensis* CDR genes, to distinguish each of the *CDR* genes based on the homology between the citrus databases. The predicted CDR proteins varied in length from 185 (CcCDR2) to 480 (CcCDR1 and CsCDR1) amino acids. The predicted relative molecular mass varied from 20.4 kDa (CcCDR2) to 51.7 kDa (CcCDR1 and CsCDR1), and the pIs ranged from 4.67 (CcCDR8) to 9.71 (CcCDR1), with eight members having a pI<7 and nine CDR members having a pI>7 (Supplementary Table 2). Pair-wise analysis of predicted CDR protein sequences indicated that the overall amino-acid identity was 53.4%.

### Phylogenetic and physical relationships among *Citrus CDR* genes

**Figure 1:**
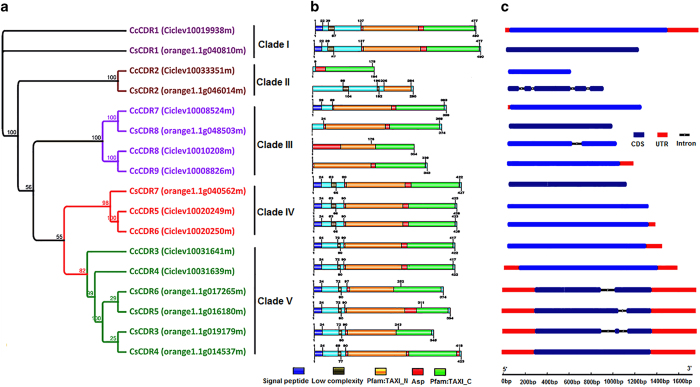
Phylogenetic analysis, distribution of conserved domains, and gene structure of *Citrus CDR* genes. (**a**) Neighbor-joining tree was created using PhyML software with 1000 bootstrap after aligning conserved ASP domain of *CDR* genes with ClustalW. A total of nine *CDR* genes from *C*. *×clementina* (*CcCDR1- CcCDR9*) and eight *CDR* genes from *C. sinensis* genome (*CsCDR1- CsCDR8*) were used to construct the tree. Names in the bracket represent respective gene IDs from *Citrus* genome databases. Five different clades were color labeled. Number above and below branches indicate bootstrap values. (**b**) Conserved domains in the deduced protein sequences from *CcCDR* (*C.*×*clementina*) and *CsCDR* (*C. sinensis*) genes. The relative positions of each domain within each protein are marked with different colors. (**c**) The structures of *CcCDR* genes are presented from *C.*×*clementina* (light blue) from *C. sinensis* (dark blue). Gene lengths are displayed proportionally. The blue regions represent exons. Interrupted black lines are introns and red regions are UTRs.

The 17 *CDR* genes were grouped into five major clades, clade I, II, III, IV and V, with well-supported bootstrap values (Figure 1a). Clade I and II had two members, one from *C*. *×clementina,* and one from *C*. *sinensis*. Four members were clustered into clade III, three genes from *C*. *×clementina* and one from *C*. *sinensis*. Three members were present in clade IV, with two genes from *C*. *×clementina* and one from *C*. *sinensis*. Six members fell into clade V, two genes from *C*. *×clementina* and four from *C*. *sinensis*. Fifteen of the predicted CDR proteins would be localized in the outer membrane of citrus cells while the predicted CcCDR8 and CcCDR9 proteins would be present extracellularly (Supplementary Table 3).

Three CDR genes (*CcCDR*2, *CcCDR3*, and *CcCDR*4) are located within 50 kb on Scaffold 3 in the *C.*×*clementina* genome assembly. The predicted protein structure of *CcCDR3* and *CcCDR4* is almost identical*. CcCDR5* and *CcCDR6* are present at the same genomic scaffold location and have similar predicted protein structures, indicating a likely tandem duplication event. *CsCDR3, CsCDR5* and *CsCDR6* in the *C. sinensis* genome represent different alternate splicing isoforms of the *CsCDR4.*

### Domains and structures of *Citrus CDR* genes

CDR proteins are characterized by the following domains: a signal peptide, a propeptide, and an ASP domain with two active sites.^49^ All 17 predicted citrus CDR proteins contain at least one ASP domain (Figure 1b). Except for CcCDR2, CcCDR8, CcCDR9, CsCDR2 and CsCDR8, 12 CDR proteins each contain a signal peptide of 24 amino acids. In addition, the 12 CDR proteins each carry one low complexity domain. All 17 CDR proteins contain a TAXI_N and TAXI_C domain along with the ASP domain.

The divergence of exon/intron structure often plays a key role in the evolution of gene families.^50^ To understand the structural components of *Citrus CDR* genes, their exon and intron organization were inferred by comparing the cDNA sequences with the corresponding genomic DNA sequences (Figure 1c). Among the nine *CcCDR* genes, one gene (*CcCDR8*) contains one intron, and five genes (*CcCDR1*, *CcCDR3*, *CcCDR4*, *CcCDR6* and *CcCDR7*) have either 5’ or 3’ or both the untranslated regions (UTR) present in their transcripts. Among eight *CsCDR* genes, one gene (*CsCDR4)* has three transcription isoforms, three genes (*CsCDR3*, *CsCDR5* and *CsCDR6*) each contains one or two introns and both 5′ and 3′ UTRs. The protein structures of these four *CsCDR* genes (*CsCDR3-6*) are highly similar. The other four *CsCDR* genes (*CsCDR1-2* and *CsCDR7-8*) have no UTRs in their respective transcripts. *CsCDR2* contained the largest number of introns (four introns) among all the *CDR* genes in this study.

### *CDR* genes and SNPs in resequenced *Poncirus* genomes

**Figure 2:**
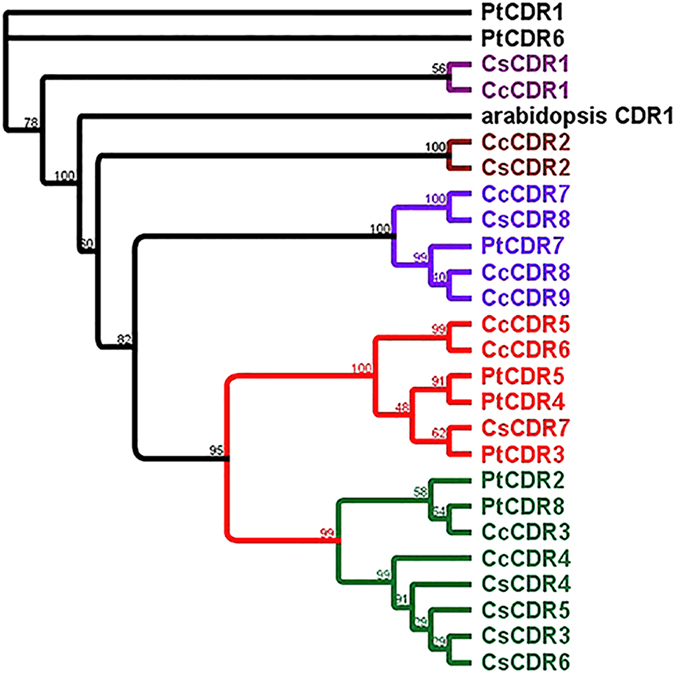
Phylogenetic tree of *CDR* genes of *Citrus* and *Poncirus*. The phylogenetic tree was created using eight *Poncirus CDR* genes (*PtCDR1*-*PtCDR8*), *Arabidopsis CDR1* gene and 17 *Citrus CDR* genes. The ASP domain was first aligned with ClustalW and a neighbor-joining tree was created using PHYML software with 1000 bootstrap value.

We obtained 21 gigabases (Gb) and 10.9 Gb of clean sequence data for *Poncirus* accessions DPI 50-7 and Flying Dragon, respectively, which represent 55× and 25× depth of coverage of their genomes (Table 2). A total of 5411 sequence reads of DPI 50-7 and 2665 sequence reads of Flying Dragon matched to 15 *Citrus CDR* genes. We identified eight copies of *CDR* genes (named as *PtCDR1* to *PtCDR8*) in the resequenced *Poncirus* samples. SNPs in the *PtCDR* genes were called with a read-depth level of 3 to 50 and a Phred quality score>20. The type of SNP (transition, transversion, substitution, deletion, and insertion) and location of the SNPs (5′-UTR, exon, intron and 3′-UTR) were characterized for the eight *PtCDR* genes (Supplementary Table 4). Most of deletions and insertions were present in the UTR regions. The ratio of transition/transversion was>1 for four *PtCDR* genes (*PtCDR1*, *PtCDR2*, *PtCDR6* and *PtCDR7*), suggesting the presence of larger numbers of non-synonymous SNPs (nsSNPs) in these genes. The predicted proteins of all *PtCDR* genes contain the ASP domain. *Poncirus CDR* genes were clustered with their *Citrus CDR* orthologs (Figure 2).

There are various numbers of amino-acid (aa) changes in *PtCDR* genes: *PtCDR1* (4 aa), *PtCDR2* (27 aa), *PtCDR3* (21 aa), *PtCDR4* (21 aa), *PtCDR5* (19 aa), *PtCDR6* (5 aa), *PtCDR7* (7 aa), and *PtCDR8* (16 aa). We observed insertion/deletion of amino acids in four *PtCDR* genes: *PtCDR2* (insertion of 4 aa), *PtCDR4* (deletion of 1 aa), *PtCDR6* (insertion of 2 aa), and *PtCDR8* (insertion of 7 aa). Overall, substitution and insertion/deletion of amino acids were more abundant in *PtCDR2* and *PtCDR8* compared to other *PtCDR* genes.

We obtained ~11 and 13 Gb of clean DNA sequences for the ‘US-897’ and ‘US-812’ genome, respectively, equivalent to an average of 26× depth of coverage per hybrid genotype. A total of 3142 sequence reads from ‘US-897’ and 4335 reads from ‘US-812’ matched to the 17 *Citrus CDR* genes, respectively. Most of the sequence variants found in ‘US-897’ and ‘US-812’ were heterozygous in nature. The numbers of homozygous SNPs present in these *Poncirus* hybrids for a given *CDR* gene were about half of that in DPI 50-7 and Flying Dragon, as would be expected due to the hybrid origin of ‘US-812’ and ‘US-897’.

Consensus DNA sequences were generated from the sequence reads for all *PtCDR* genes (Supplementary Table 5). ‘US-812’ carries two different alleles (one from *Citrus* and one from *P*. *trifoliata*) at *PtCDR1*, *PtCDR2, PtCDR3, PtCDR4, PtCDR6* and *PtCDR8* loci and only one allele from *P. trifoliata* at *PtCDR5* and *PtCDR7* loci. On the other hand, ‘US-897’ carries two different alleles at all *PtCDR* gene loci, except for *PtCDR3*, at which ‘US-897’ carries only the *P. trifoliata* allele. The absence of a *Citrus* allele at the *PtCDR5* and *PtCDR7* loci in ‘US-812’ and at the *PtCDR3* locus in ‘US-897’ may suggest that these loci are hemizygous in ‘US-812’ and ‘US-897’.

### *CDR* genes in *Poncirus* transcriptomes

We obtained ~20 million sequence reads for each of the four leaf transcriptomes (two *Poncirus* accessions (DPI 50-7 and Flying Dragon) grown under two different conditions). The FPKM values for each *CDR* gene were calculated (Supplementary Table 1). Two *Poncirus* CDR genes, *PtCDR1* and *PtCDR4*, were in lower abundance in *C*Las-exposed, field-grown DPI 50-7 than in non-inoculated, greenhouse-grown DPI 50-7. *PtCDR2* (for DPI 50-7 and Flying Dragon) and *PtCDR8* (for DPI-50-7) were in higher abundance in *C*Las-exposed, field-grown plants than in non-inoculated, greenhouse-grown plants.

### *CDR-*related ESTs from the *Poncirus* EST database

Out of the 61 874 *Poncirus* ESTs from the citrus genome database, 10 ESTs (contigs) match with the *Arabidopsis CDR* genes and contain the ASP domain. Mapping RNA-Seq reads of DPI 50-7 and Flying Dragon with the 10 identified *CDR-*related contigs allowed for retrieval of 7032 reads from the RNA-Seq data that have 98% identity with the 10 *CDR*-related contigs. The 10 *Poncirus* ESTs correspond to two *Poncirus CDR* genes, *PtCDR2* and *PtCDR8*, indicating that these two genes are expressed in *Poncirus* leaves.

### Real-time PCR amplification of *CDR* genes in HLB-susceptible and tolerant genotypes

**Figure 3:**
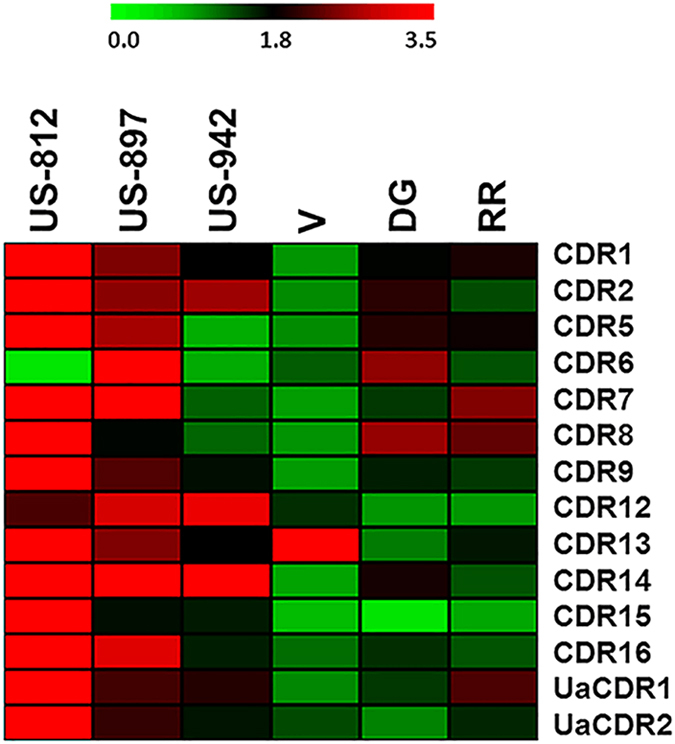
Relative expression of *CDR* genes in HLB-susceptible and HLB-tolerant genotypes. The results of real-time PCR of each *CDR* gene with three HLB-tolerant (US-812, US-897 and US-942) and three HLB-susceptible (V, DG and RR) genotypes are presented as a heat map. ‘Green’ blocks represent down-regulation while ‘red’ blocks represent up-regulation in *C*Las-infected plant when compared with its respective non-inoculated plant. *Citrus* and *Poncirus* genotypes are represented horizontally and primers used for Real-time PCR analysis are shown vertically.

Results for 14 pairs of CDR primers are represented in Figure 3. Expression of most of the *CDR* genes was higher in *C*Las infected ‘US-812’ and ‘US-897’ when compared with their respective non-infected samples (Figure 3, Supplementary Table 6). Moreover, *CDR* transcripts amplified by primer pairs CDR14 *(CsCDR3)*, CDR15 *(CsCDR6)*, CDR16 *(CsCDR5/PtCDR2),* UaCDR1 and UaCDR2 had higher expression levels in the HLB-tolerant genotypes. *PtCDR2* was also found to be upregulated in RNA-Seq data. All upregulated *CDR* genes belong to clade V of the phylogenetic tree and share similar gene structures (Figure 1a).

## Discussion

### *CDR* genes in *Citrus*

Recently, several studies were carried out to identify potential candidate genes in *Citrus* and *Poncirus* associated with HLB tolerance, and *CDR* genes were among those identified.^15^ In this study, 17 *CDR* genes were identified in two *Citrus* genomes, and they fell into five clades. Fifteen of the 17 *CDR* genes contained no or only one intron. Citrus *CDR* genes in the same clades share similar motifs. On the basis of the high similarity at both protein and gene structure levels and their locations in the citrus genome, it appears that *CcCDR5* and *CcCDR6* have arisen from a recent tandem duplication and may have a redundant function in citrus. These two genes have the same gene structure except for the presence of a 57-bp 3′-UTR in *CcCDR6. CsCDR3-CsCDR6* were present within the same phylogenetic clade (Figure 1a), and they may represent paralogs. Based on the results presented above, it is clear that the expansion of the *Citrus CDR* family was the result of segmental and tandem duplication, which could be the result of chromosomal rearrangement and fusions. Similar results were found with the aspartic gene family in rice.^51^

### CDR genes in *Poncirus*

Genome resequencing and analysis led to the identification of eight *CDR* genes in *Poncirus*. Compared to *Citrus CDR* genes, *Poncirus CDR* genes carry a multitude of SNPs. Most of the SNPs were present in exonic regions of *PtCDR* genes, and deletion and insertion-type mutations were prominent in the regulatory and non-coding regions (5′ and 3′-UTR regions). Most of the identified SNPs were of the transition type, and the ratio between transition and transversion was more than one for four *PtCDR* genes. The degeneracy of the genetic code and the selective pressure for gene conservation are likely accountable for the dominance of transitions over transversions. In general, transition mutations are generated at higher frequency than transversions in synonymous substitutions, and there is a stronger selection against replacement substitutions, leading to higher occurrences of transitions. SNPs in protein-coding sequences have potential effects on gene function, especially the nsSNPs that could lead to amino-acid residue changes and altered functional or structural properties of the protein. Results from the present study indicate that the predicted protein structure and function may be altered in some of the *Poncirus CDR* genes, particularly in *PtCDR2* and *PtCDR8,* as they contain the largest numbers of amino-acid changes among all *Poncirus CDR* genes. Li *et al.*^52^ analyzed the contributions of genic and non-genic sequence polymorphisms to natural variation of quantitative traits in maize and revealed that 79% of the explained variation could be attributed to SNPs located in genes or within 5 Kb upstream of genes. The SNPs in the genic and promotor regions of *Poncirus* CDR genes may be useful for genetic dissection of complex traits such as HLB tolerance.

Transcripts of four *PtCDR* genes were detected in *Poncirus* leaf transcriptomes. While *PtCDR1* and *PtCDR4* were in low abundance, *PtCDR*2 and *PtCDR*8 showed high abundance in plants grown in the field and exposed to natural HLB disease pressure. Both *PtCDR2* and *PtCDR8* were grouped in clade V of the phylogenetic tree and have similar gene structures. A total of 70 SNPs were revealed in these four expressed *PtCDR* genes when compared with their *Citrus CDR* orthologs. Among the SNPs, 20% were nsSNPs. Most of these nsSNPs are present in regulatory regions. These nsSNPs should be closely examined in future studies of the roles of *CDR* genes in HLB tolerance.

In a previous study,^17^ the expression of one *CDR* probe set *(Cit.23704.1.S1_at)* was 300-1000 fold higher in ‘US-897’ and ‘US-942’ than in Cleopatra (*Citrus reshni* hort. Ex Tanaka), regardless of *C*Las infection. In that study, another *CDR* probe set *(Cit.28117.1.S1_s_at)* was found to be expressed similarly in the three cultivars without *C*Las infection, and increased moderately in all three cultivars through the 30 weeks following infection. An earlier study^15^ using microarray analysis also found higher expression of these *CDR* probe sets in ‘US-897’ as compared with Cleopatra, independent of *C*Las infection. The present study shows that probe sets *Cit.23704.1.S1_at* and *Cit.28117.1.S1_s_at* bind to *CcCDR3* or *PtCDR8* genes with 90% homology. In contrast to the present study, the primers (UaCDR1 and UaCDR2) used to validate these probe sets bind to multiple *CDR* genes instead of individual *CDR* genes. We observed that the *CDR* genes in clade V were highly expressed in HLB-tolerant genotypes compared with HLB-susceptible genotypes. *PtCDR2* and *PtCDR8* identified with relative high abundance in *Poncirus* transcriptome sequencing data as well in real-time PCR analysis were clustered with clade V, making these genes very interesting candidates for further functional characterization.

It is worth noting that many *CDR* genes (including *CcCDR1*, *7* and *8*) showed low expressions when compared with other *CDR* genes after *C*Las infection. A possible explanation for higher induction for Clade V *CDR* genes could be that these genes have characteristic UTR regions in their gene sequences which are not common in genes present in other clades, which in turn, could result in diverse regulatory mechanisms of clade V genes. The expression patterns of *CDR* genes support our findings that the distribution of specific motifs or specific patterns for a motif in proteins is associated with a specific clade in the phylogram. These motifs may be involved in regulation of HLB-related gene expression.

In summary, we identified 17 *CDR* genes in two citrus genomes and revealed key features of their gene structure. Separation of these *CDR* genes into five clades was mutually supported by their phylogeny and exon/intron structure and the distribution of conserved domains. Whole genome resequencing led to the identification of eight *CDR* genes in *Poncirus*. *PtCDR2* and *PtCDR8* were found in high abundance in *Poncirus* leaf transcriptomes. The expression of *PtCDR2* and *PtCDR8* genes responded to *C*Las infection differently in HLB-tolerant and susceptible genotypes. The information gained in this study provides new insights regarding the potential roles of *CDR* genes in mediating citrus responses to *C*Las infection and HLB development. Gene sequences and sequence variants from this analysis should be very valuable for functional validation of *CDR* genes and potentially for engineering HLB resistance/tolerance in new citrus cultivars.

## Figures and Tables

**Table 1:** Primers designed and used for real-time PCR amplification of *CDR* genes

*Citrus CDR genes*	*PtCDR orthologs*	*Primer name*	*Primer Sequence (5′ to 3′)*	*Product size (bp)*	*Tm (*^*0*^*c)*
*CsCDR7, CcCDR5, CcCDR6*	*PtCDR3, PtCDR4, PtCDR5*	CDR1	F: GTGCAACAAAGCTCAACCAA R: TCCCGGATTTGATCCTGATA	175	58
*CcCDR6*	*PtCDR4*	CDR2	F: TAGTCCGCTACGGAGACCAA R: GCTGGTGGTAAATAGGTTAG	464	58
*CsCDR7*	*PtCDR3*	CDR3	F: CAGTCTCCTACGGAGACGAT R: GCTGGTGGTAAATAGGTAAG	461	58
*CcCDR5*	*PtCDR5*	CDR4	F: TAGTCCGCTACGGAGACCAA R: GCTGGTGGTAAATAGGTACG	464	58
*CcCDR7*	—	CDR5	F: TTGTTCCCCGTCTTTCTTTG R: AGATTGGCGACTTGGGAG	107	58
*CcCDR7, CcCDR8*	—	CDR6	F: CTCCCAAGTCGCCAATCT R: GCATATGCACCTCTTCCATATGG	320	60
*CsCDR2*	—	CDR7	F: GTTAGCCAAATGGGTCCTTC R: TCTCGCAACCAAGGGAGTAG	166	60
*CcCDR2*	—	CDR8	F: TGCCACAAGTGTTTAGCTCAG R: CCTAGTGAAAGTGTTCAAAGGAC	189	60
*CsCDR1, CcCDR1*	*PtCDR1, PtCDR6*	CDR9	F: CTACTCTCAAACCGACCCTGTC R: GTGATAGAACCGTCTCCGTAGG	157	58
*CcCDR9*	*PtCDR7*	CDR10	F: CTTTTGGTGGAATTGTTGC R: CCACCGGACACAAATTCTAGCC	190	58
*CcCDR8, CcCDR9*	*PtCDR7*	CDR11	F: CAGTCGTACTTTATTACCAC R: TGTATTGGCATCACCACCTG	266	55
*CsCDR4, CsCDR5*	*—*	CDR12	F: CCTATTGTTTGGTTCCAGTT R: GGTACCTGTAAAAACAGAACAC	429	55
*CcCDR3*	*PtCDR8*	CDR13	F: CAAGCTGATATAATACCCAATATCGGAG R: GAGGCTCGCACTGCGT	116	58
*CsCDR6*	*—*	CDR14	F: CAATTGCTGGAAACCAAAG R: GTCTGCAACAGGTTGT	147	56
*CsCDR3*	*PtCDR2*	CDR15	F: CAATTGCTGGAAACC R: TTCAAGTGATCCTGTAGGGTCAGA	78	56
*CsCDR3, CsCDR5*	*PtCDR2*	CDR16	F: CAGTCGTACTTTATTACCAC R: TGTATTGGCATCACCACCTG	166	56
*CcCDR3, CcCDR4, CsCDR3,* *CsCDR4, CsCDR5, CsCDR6*	*PtCDR2, PtCDR8*	UaCDR1^a^	F: TCCACTCTTTGATCCTMAAAAGTC R: CTCAAYTTCACATCTGCRYCCYCYT	(Albrecht and Bowman, 2012)	55
*CcCDR3, CcCDR4, CsCDR3,* *CsCDR4, CsCDR5, CsCDR6*	*PtCDR2, PtCDR8*	UaCDR2^a^	F: G AATCGAGGTACTTGATAGCTC R: GCAAGACTTTGATCAAATCCT	(Albrecht and Bowman, 2012)	55
18 S ribosomal rRNA (as an internal reference)		18 S^a^	F: GTGACGGAGAATTAGGGTTCG R: CTGCCTTCCTTGGATGTGGTA	(Kim *et al*.,^26^)	55-59

a Primer sequences from previous published reports.

**Table 2:** Statistics of the DNA-Seq sequence data

*Sample*	*Raw reads*	*Clean reads*	*Raw base (Gb*)	*Clean base (Gb*)	*Effective rate (%)*	*Error rate (%)*	*Q20 (%)*	*Q30 (%)*	*GC content (%)*
DPI 50-7	86 109 356	84 802 048	21.527	21.201	98.48	0.04	94.44	90.22	39.32
Flying Dragon	43 847 801	43 242 390	10.962	10.811	98.62	0.04	94.13	89.48	38.01
US-897	44 253 371	43 720 411	11.063	10.930	98.80	0.04	94.27	89.81	38.57
US-812	54 971 421	54 385 348	13.743	13.596	98.93	0.04	94.25	89.71	38.84

Abbreviation: Gb, Gigabases.
