# Supplementary material for: Genome resequencing and transcriptome profiling reveal structural diversity and expression patterns of constitutive disease resistance genes in Huanglongbing-tolerant Poncirus trifoliata and its hybrids
Source: Hortic Res. 2017 Nov 15;4:17064–. doi: 10.1038/hortres.2017.64 (PMC5686287; doi:10.1038/hortres.2017.64)
Supplement: Supplementary Table 1 [file hortres201764-s1.pdf]

Table S1: Differentially expressed *Poncirus CDR* genes in RNA-Seq data. List of *CDR* genes detected in DPI 50-7 and Flying Dragon transcriptome data along with their FPKM values.

**S.No.**	**Gene_id**	**Genotype**	***FPKM Value (greenhouse-grown)**	***FPKM Value (field-collected)**	**log2(fold_change)**
1	*PtCDR2*	DPI 50-7	241.021	2244.87	3.2194
		Flying Dragon	1532.92	3741.47	1.28732
2	*PtCDR8*	DPI 50-7	105.861	181.57	0.778358
		Flying Dragon	199.797	167.898	-0.250948
3	*PtCDR1*	DPI 50-7	10.2342	3.1363	-1.70627
		Flying Dragon	_	_	_
4	*PtCDR4*	DPI 50-7	1.73627	1.42524	-0.28478
		Flying Dragon	_	_	_

*FPKM Value = fragments per kilo base of exon per million fragments mapped

Supplementry Table 1: Differentially expressed transcripts identified in DPI 50-7 RNA-Seq data

Gene_id	locus	FPKM Value (greenhouse-grown)	FPKM Value (field-collected)	log2(fold_change)
Ciclev10015896m.g	scaffold_2:33303510-33307252	4.13048	0.000296219	-13.7674
Ciclev10016087m.g	scaffold_2:13799455-13802309	9.18351	0.00769328	-10.2212
Ciclev10013139m.g	scaffold_6:17572791-17573553	1222.1	1.79453	-9.41154
Ciclev10033176m.g,Ciclev10033829m.g	scaffold_4:16223614-16233365	56.3628	0.11424	-8.94653
Ciclev10015743m.g	scaffold_2:26076215-26078590	37.662	0.134532	-8.12901
Ciclev10028988m.g	scaffold_8:22184809-22187211	43.5344	0.160729	-8.08138
Ciclev10007321m.g	scaffold_1:28248422-28254232	30.189	0.121132	-7.9613
Ciclev10032948m.g	scaffold_4:19927053-19931264	32.1302	0.130994	-7.93829
Ciclev10022159m.g	scaffold_3:47237821-47239022	88.6278	0.365458	-7.92191
Ciclev10020292m.g	scaffold_3:8410167-8412277	24.303	0.10095	-7.91134
Ciclev10010934m.g	scaffold_6:24923533-24935909	7.60832	0.0331335	-7.84315
Ciclev10021923m.g	scaffold_3:6828480-6829499	27.304	0.129516	-7.71984
Ciclev10014707m.g	scaffold_2:32481548-32484643	111.102	0.588072	-7.56168
Ciclev10002900m.g	scaffold_5:441330-538185	571.694	3.46743	-7.36523
Ciclev10032519m.g	scaffold_4:24820268-24824732	0.614268	0.00421505	-7.18718
Ciclev10022337m.g	scaffold_3:49295580-49296607	22.7832	0.184015	-6.952
Ciclev10022129m.g	scaffold_3:18472966-18487087	942.15	8.30451	-6.82592
Ciclev10022211m.g,Ciclev10022911m.g	scaffold_3:31770209-32016548	729.509	6.45745	-6.81982
Ciclev10031021m.g	scaffold_4:21321026-21325773	10.985	0.112893	-6.60444
Ciclev10001968m.g	scaffold_5:36226059-36227269	47.6087	0.50883	-6.5479
Ciclev10032858m.g	scaffold_4:24521144-24523696	137.793	1.49453	-6.52666
Ciclev10031134m.g	scaffold_4:785463-788251	14.8011	0.160569	-6.52637
Ciclev10002672m.g	scaffold_5:27365221-27366726	13.4289	0.153929	-6.44693
Ciclev10013995m.g	scaffold_13:158978-162675	4.24968	0.0495015	-6.42374
Ciclev10023723m.g	scaffold_3:45988657-45993348	7.58263	0.0931816	-6.34651
Ciclev10019884m.g	scaffold_3:11631021-11635628	5.18592	0.0666631	-6.28157
Ciclev10028930m.g	scaffold_8:3113094-3114407	205.386	2.76749	-6.21362
Ciclev10022989m.g	scaffold_3:45381554-45381989	43.2209	0.590046	-6.19476
-	scaffold_5:19085272-19085568	370.36	5.06058	-6.19348
-	scaffold_1:24209806-24210531	17.9345	0.253918	-6.14223
Ciclev10015707m.g	scaffold_2:34052179-34054960	33.1014	0.481564	-6.10302
Ciclev10005777m.g	scaffold_9:4699069-4700431	24.5777	0.359321	-6.09593
Ciclev10001325m.g	scaffold_5:43048046-43051036	7.58629	0.112201	-6.07923
Ciclev10022124m.g,Ciclev10023287m.g	scaffold_3:18472966-18487087	995.364	15.0824	-6.04428
Ciclev10007068m.g	scaffold_9:28562375-28563020	96.5714	1.47059	-6.03712
Ciclev10026970m.g	scaffold_7:13551378-13553061	6.64907	0.101668	-6.03121
Ciclev10025272m.g	scaffold_7:16553737-16556729	4.69346	0.0720811	-6.02489
Ciclev10005385m.g	scaffold_9:547009-550115	92.6157	1.42357	-6.02367
-	scaffold_1:5008452-5008735	88.471	1.36836	-6.01468
Ciclev10005869m.g	scaffold_9:28569930-28570930	19.8189	0.313573	-5.98193
Ciclev10031286m.g	scaffold_4:9423809-9425662	4.78725	0.0761784	-5.97367
Ciclev10022074m.g	scaffold_3:33714569-33716049	12.0349	0.19201	-5.9699
Ciclev10002675m.g	scaffold_5:36080141-36081013	34.5456	0.58497	-5.88399
Ciclev10031332m.g	scaffold_4:19249944-19251830	4.51199	0.0807994	-5.80328
Ciclev10023013m.g	scaffold_3:45376158-45376887	14.6874	0.26464	-5.7944
-	scaffold_6:6344256-6344759	23.7195	0.430753	-5.78307
Ciclev10000364m.g	scaffold_5:40040263-40042949	6.86254	0.125467	-5.77337

Ciclev10016832m.g	scaffold_2:32829645-32831351	105.597	1.93101	-5.77307
Ciclev10013766m.g	scaffold_6:20981041-20981593	180.106	3.33343	-5.7557
Ciclev10009935m.g	scaffold_1:1472059-1472860	13.7814	0.259583	-5.73039
Ciclev10001308m.g	scaffold_5:34257704-34260311	107.282	2.06059	-5.70221
Ciclev10012698m.g	scaffold_6:20636355-20637195	10.5934	0.20374	-5.7003
-	scaffold_4:7294697-7294854	1591.81	30.9365	-5.68521
Ciclev10020259m.g	scaffold_3:48136987-48153335	9.40662	0.18569	-5.66271
Ciclev10012140m.g	scaffold_6:23349635-23354158	6.64945	0.132605	-5.64803
Ciclev10015955m.g	scaffold_2:33473684-33475325	23.7555	0.481286	-5.62522
Ciclev10005954m.g	scaffold_9:1134875-1136007	135.158	2.75379	-5.61709
Ciclev10015449m.g	scaffold_2:28206320-28209328	13.4623	0.276473	-5.60565
Ciclev10028964m.g	scaffold_8:461188-468899	85.9833	1.77864	-5.59521
Ciclev10007591m.g	scaffold_1:1762846-1768718	2.86112	0.0597127	-5.5824
-	scaffold_4:11523554-11728349	16.2091	0.338317	-5.58229
-	scaffold_1:6248315-6248726	28.0469	0.605207	-5.53427
-	scaffold_5:2534144-2731098	8.69547	0.191032	-5.50837
Ciclev10002840m.g	scaffold_5:38154974-38155769	10.9983	0.244042	-5.49401
Ciclev10011400m.g	scaffold_6:15761840-15764472	6.86514	0.154727	-5.47149
Ciclev10014952m.g	scaffold_2:14657848-14665608	5.46078	0.127093	-5.42515
-	scaffold_1:779311-812949	233.764	5.45639	-5.42096
Ciclev10010063m.g	scaffold_1:5003828-5004439	202.33	4.73443	-5.41737
Ciclev10011300m.g	scaffold_6:11514709-11519693	1.86225	0.0442433	-5.39544
Ciclev10009569m.g	scaffold_1:27514736-27515607	14.418	0.345162	-5.38446
Ciclev10009119m.g	scaffold_1:27422722-27424055	19.719	0.482083	-5.35416
Ciclev10015599m.g	scaffold_2:26745506-26746983	40.7161	0.996358	-5.35279
-	scaffold_6:14886117-14886335	138.578	3.39749	-5.35009
Ciclev10015870m.g	scaffold_2:35334244-35336841	637.438	16.248	-5.29395
Ciclev10009879m.g	scaffold_1:17185031-17189281	67.319	1.76552	-5.25285
Ciclev10011238m.g	scaffold_6:21226626-21229106	4.2711	0.113018	-5.23999
Ciclev10027295m.g	scaffold_7:7202245-7222786	69.3239	1.83835	-5.23687
Ciclev10019452m.g	scaffold_3:46349126-46351795	2.76266	0.0735171	-5.23183
Ciclev10017104m.g	scaffold_2:33601731-33602996	65.6146	1.77229	-5.21033
Ciclev10025952m.g	scaffold_7:2441451-2443391	7.13921	0.194757	-5.19602
Ciclev10028605m.g	scaffold_8:10272177-10273455	4.53092	0.12371	-5.19478
Ciclev10033694m.g	scaffold_4:25155260-25156786	33.5219	0.917994	-5.19048
-	scaffold_2:26287888-26288140	69.9648	1.93973	-5.1727
Ciclev10014929m.g	scaffold_2:21317979-21320849	2.40709	0.0669949	-5.16709
Ciclev10013249m.g	scaffold_6:22622445-22622917	17.1949	0.490919	-5.13036
Ciclev10002142m.g	scaffold_5:1443599-1445440	9.98062	0.285016	-5.13002
-	scaffold_7:1774752-1776831	123.817	3.54293	-5.12712
Ciclev10010728m.g	scaffold_1:8090395-8092825	4.3766	0.125597	-5.12294
Ciclev10010422m.g	scaffold_1:19100681-19101041	28.9416	0.831405	-5.12145
Ciclev10028106m.g	scaffold_8:7862728-7866997	12.3431	0.35678	-5.11253
-	scaffold_8:2274855-2275082	488.954	14.2722	-5.09842
-	scaffold_5:14438512-14438660	1949.97	57.3633	-5.08718
Ciclev10001665m.g	scaffold_5:42957413-42961730	64.6039	1.90064	-5.08706
Ciclev10020552m.g	scaffold_3:31620762-31715816	22541.7	664.44	-5.08431
-	scaffold_1:5008048-5008400	461.407	13.8806	-5.0549
Ciclev10002415m.g	scaffold_5:29742801-29769281	5.98053	0.181194	-5.04467
Ciclev10001946m.g	scaffold_5:41158107-41160518	149.255	4.53827	-5.03949
Ciclev10015163m.g	scaffold_2:7799700-7801894	790.618	24.1834	-5.03089
Ciclev10032218m.g	scaffold_4:24412331-24414624	24.4669	0.751724	-5.02448
-	scaffold_6:18482290-18482442	1385.52	42.8878	-5.01371
Ciclev10013413m.g	scaffold_6:21415823-21418494	4.54455	0.141716	-5.00306
-	scaffold_8:2273110-2274717	126.669	3.9516	-5.00248
Ciclev10021486m.g	scaffold_3:4182370-4183602	9.89159	0.310943	-4.99148

Ciclev10022001m.g	scaffold_3:18341165-18342395	5398.79	170.929	-4.98117
Ciclev10010784m.g	scaffold_1:11024085-11028084	28.8249	0.914655	-4.97795
Ciclev10031320m.g	scaffold_4:25354556-25358068	10.2446	0.333281	-4.94198
Ciclev10023557m.g	scaffold_3:31770209-32016548	347.023	11.3085	-4.93955
-	scaffold_8:19132836-19211350	369.264	12.0875	-4.93306
Ciclev10031364m.g	scaffold_4:19553769-19555593	2.75452	0.0905473	-4.92698
Ciclev10029316m.g	scaffold_8:2006038-2014782	14.1817	0.470587	-4.91343
Ciclev10000437m.g	scaffold_5:34837869-34841284	24.8667	0.828029	-4.90839
Ciclev10021073m.g	scaffold_3:1172384-1173975	3.30919	0.111645	-4.88948
-	scaffold_5:3950333-3951488	6.68169	0.227626	-4.87547
Ciclev10016917m.g	scaffold_2:32720608-32721917	68.3773	2.32977	-4.87526
Ciclev10009011m.g	scaffold_1:3822951-3824251	6.14562	0.209725	-4.87299
Ciclev10022909m.g	scaffold_3:31620762-31715816	466.007	16.5802	-4.81282
Ciclev10016953m.g	scaffold_2:35861294-35862848	20.84	0.743443	-4.80899
Ciclev10004841m.g	scaffold_9:25739036-25740714	2.59868	0.09331	-4.7996
Ciclev10012912m.g	scaffold_6:10341293-10342204	101.781	3.65866	-4.79801
-	scaffold_8:19132836-19211350	82.2585	2.96139	-4.79582
-	scaffold_7:3067895-3068047	1187.58	42.8878	-4.79132
-	scaffold_5:9713649-9935604	14.8807	0.538843	-4.78743
Ciclev10003662m.g	scaffold_5:35059782-35061627	22.2212	0.805544	-4.78583
Ciclev10022835m.g	scaffold_3:49993997-49994799	42.5361	1.54633	-4.78177
Ciclev10029695m.g	scaffold_8:2308971-2309555	9.31293	0.339473	-4.77787
Ciclev10027856m.g	scaffold_8:6659339-6662205	4.62011	0.170337	-4.76147
Ciclev10032960m.g	scaffold_4:16250556-16251633	9.02573	0.332831	-4.76118
Ciclev10018959m.g	scaffold_3:46357699-46364094	4.1299	0.153526	-4.74955
Ciclev10027055m.g	scaffold_7:11850299-12160617	4.54348	0.169172	-4.74724
Ciclev10030000m.g	scaffold_8:6520016-6520453	196.781	7.3728	-4.73824
Ciclev10028046m.g	scaffold_8:22645848-22651938	2.16574	0.0822343	-4.71898
Ciclev10017620m.g	scaffold_2:23858359-23858920	9.82492	0.373966	-4.71547
Ciclev10028881m.g	scaffold_8:22859339-22860462	12.7229	0.487316	-4.70643
Ciclev10028798m.g	scaffold_8:2763024-2764293	3.64174	0.140224	-4.69883
Ciclev10029088m.g	scaffold_8:502051-503766	64.7764	2.51144	-4.68888
-	scaffold_6:9798460-9798845	17.6123	0.683236	-4.68806
Ciclev10004099m.g	scaffold_5:17583731-17584966	9.68256	0.376522	-4.68458
-	scaffold_1:4737054-4737890	5.35322	0.210671	-4.66734
Ciclev10013859m.g	scaffold_6:18569400-18571341	3.58181	0.142567	-4.65098
Ciclev10016770m.g	scaffold_2:36099095-36101018	18.8939	0.752103	-4.65085
Ciclev10011211m.g	scaffold_6:23100559-23105430	7.23062	0.288013	-4.64991
Ciclev10009484m.g	scaffold_1:16724986-16726058	1368.13	54.5155	-4.6494
Ciclev10002979m.g	scaffold_5:34404146-34405161	5.87227	0.236458	-4.63426
Ciclev10026991m.g	scaffold_7:5129293-5133799	1.62787	0.0658547	-4.62756
Ciclev10001552m.g	scaffold_5:35027721-35030201	71.4606	2.89284	-4.62659
Ciclev10013107m.g	scaffold_6:24764197-24767525	9.96493	0.407399	-4.61234
Ciclev10003884m.g	scaffold_5:108928-112283	2.60423	0.107038	-4.60467
Ciclev10016691m.g	scaffold_2:11634676-11635703	7.60273	0.313545	-4.59977
Ciclev10001881m.g	scaffold_5:33632689-33634531	2.81002	0.116075	-4.59745
Ciclev10015460m.g	scaffold_2:22436684-22438478	100.266	4.15171	-4.59399
Ciclev10024602m.g	scaffold_3:5382489-5385009	4.3282	0.179956	-4.58805
-	scaffold_5:953144-1184934	2174.62	92.6922	-4.55217
Ciclev10001077m.g	scaffold_5:35578104-35582361	2.20398	0.0943668	-4.54569
-	scaffold_7:2154405-2154672	75.3041	3.23228	-4.5421
-	scaffold_6:10361359-10361856	40.7699	1.75605	-4.5371
Ciclev10005395m.g	scaffold_9:6864745-6869616	2.89086	0.124636	-4.53571
Ciclev10028348m.g	scaffold_8:4918555-4921989	1.69622	0.0734138	-4.53013
Ciclev10031048m.g	scaffold_4:24060144-24061905	1.73561	0.0755215	-4.52241
Ciclev10006109m.g	scaffold_9:19673321-19676059	5.75074	0.250474	-4.52101

Ciclev10003943m.g	scaffold_5:37963429-37964754	2.7396	0.119374	-4.52041
Ciclev10017518m.g	scaffold_2:34921845-34922800	14.4397	0.629487	-4.51972
Ciclev10014365m.g	scaffold_2:30005619-30011781	2.7988	0.122493	-4.51403
Ciclev10010470m.g	scaffold_1:27224300-27225158	8.92987	0.391864	-4.51021
Ciclev10002294m.g	scaffold_5:37219889-37221066	4.16376	0.184769	-4.49409
Ciclev10015770m.g	scaffold_2:23243925-23246266	35.9252	1.59524	-4.49315
Ciclev10028623m.g	scaffold_8:24554321-24561746	7.09672	0.315731	-4.49039
-	scaffold_1:813322-814694	3.14069	0.141095	-4.47634
-	scaffold_4:7432881-7433901	13.8895	0.624811	-4.47443
Ciclev10014787m.g	scaffold_2:32218212-32220325	32.0079	1.44495	-4.46934
Ciclev10017624m.g	scaffold_2:4025762-4026947	2.329	0.105461	-4.46492
Ciclev10005113m.g	scaffold_9:4742708-4746966	3.98974	0.180781	-4.46398
Ciclev10029850m.g	scaffold_8:75152-78771	0.961125	0.0440022	-4.44908
Ciclev10011673m.g	scaffold_6:1078459-1084125	18.9562	0.878243	-4.43191
Ciclev10013217m.g	scaffold_6:10367818-10368201	2197.07	102.222	-4.42581
-	scaffold_3:28858149-28858623	20.4333	0.952035	-4.42376
Ciclev10017302m.g	scaffold_2:5916118-5916575	40.0799	1.87623	-4.41697
Ciclev10005745m.g	scaffold_9:660356-663410	13.5667	0.63821	-4.40989
Ciclev10016351m.g	scaffold_2:8406644-8407971	161.393	7.719	-4.38602
Ciclev10018492m.g,Ciclev10023093m.g	scaffold_3:27210507-27242516	5.66896	0.272295	-4.37984
Ciclev10032008m.g	scaffold_4:20060786-20063674	5.32155	0.256384	-4.37547
-	scaffold_8:656156-656510	16.6752	0.806984	-4.36902
-	scaffold_5:34092031-34092271	47.4788	2.29961	-4.36782
Ciclev10002984m.g	scaffold_5:41920801-41921611	298.364	14.4907	-4.36387
Ciclev10004905m.g	scaffold_9:30520041-30521726	3.90638	0.189859	-4.36284
-	scaffold_3:21289253-21289618	15.4868	0.758304	-4.35212
Ciclev10002654m.g	scaffold_5:40652360-40653042	12.739	0.62448	-4.35045
Ciclev10018216m.g	scaffold_2:31238921-31240514	1.97748	0.0969738	-4.34992
Ciclev10003017m.g	scaffold_5:26171952-26172322	52.4965	2.57761	-4.34812
-	scaffold_8:17879230-17881953	6.9518	0.341959	-4.34549
Ciclev10000123m.g	scaffold_5:23452527-23456180	3.85425	0.190005	-4.34234
Ciclev10031202m.g	scaffold_4:15371023-15373637	1.79406	0.0885222	-4.34104
-	scaffold_8:3978675-3979769	18.9761	0.936655	-4.34052
Ciclev10022906m.g	scaffold_3:31620762-31715816	1362.43	68.101	-4.32237
Ciclev10020530m.g	scaffold_3:50307771-50309441	1.96275	0.0982492	-4.32029
Ciclev10016312m.g	scaffold_2:23679907-23681698	6.85659	0.34391	-4.31739
Ciclev10006817m.g	scaffold_9:29360866-29361294	61.2048	3.07728	-4.31392
Ciclev10000431m.g	scaffold_5:20550188-20553313	6.98022	0.351748	-4.31066
Ciclev10008936m.g	scaffold_1:22424056-22425274	30.0624	1.51573	-4.30988
Ciclev10032065m.g	scaffold_4:17842211-17846211	2.35725	0.120541	-4.28951
Ciclev10020532m.g	scaffold_3:50242162-50246469	1.61676	0.0831297	-4.2816
Ciclev10031913m.g	scaffold_4:22969823-22972612	7.9042	0.408679	-4.27358
Ciclev10017229m.g	scaffold_2:11497514-11498250	5.00759	0.259371	-4.27103
Ciclev10016504m.g	scaffold_2:31256159-31258762	2.93854	0.15231	-4.27002
Ciclev10019134m.g	scaffold_3:5981065-5985524	27.6986	1.44382	-4.26185
Ciclev10001072m.g	scaffold_5:37105295-37107915	13.2051	0.690101	-4.25814
-	scaffold_6:6373847-6373979	10774	566.879	-4.24837
Ciclev10000849m.g	scaffold_5:41833069-41836812	17.0776	0.899281	-4.24719
Ciclev10018228m.g	scaffold_2:26268587-26269460	451.295	24.2712	-4.21675
Ciclev10015331m.g	scaffold_2:6388612-6390719	1.81633	0.0984971	-4.2048
Ciclev10015351m.g	scaffold_2:6400796-6402311	2.3781	0.129621	-4.19744
Ciclev10019961m.g	scaffold_3:42080453-42083713	11.7393	0.647086	-4.18124
Ciclev10029550m.g	scaffold_8:22063489-22064178	10.0888	0.559211	-4.17322
Ciclev10004574m.g	scaffold_9:28928892-28932535	32.5252	1.80753	-4.16946
Ciclev10008369m.g	scaffold_1:2237003-2346942	1.63399	0.0908512	-4.16875

-	scaffold_5:28539341-28801354	24.8942	1.38709	-4.16568
Ciclev10007910m.g	scaffold_1:1550194-1553253	1.71681	0.0959309	-4.16159
Ciclev10005979m.g	scaffold_9:3384336-3386365	5.63067	0.315128	-4.1593
Ciclev10025349m.g	scaffold_7:16030857-16033528	20.1286	1.13627	-4.14687
Ciclev10001822m.g	scaffold_5:35721730-35723544	62.7859	3.54752	-4.14556
Ciclev10013402m.g	scaffold_6:20672730-20676154	2.02148	0.114368	-4.14366
Ciclev10004499m.g	scaffold_9:11150757-11155385	1.84669	0.1048	-4.13923
Ciclev10027998m.g	scaffold_8:22969361-22971701	9.68526	0.5504	-4.13724
Ciclev10017232m.g	scaffold_2:21031587-21032341	40.911	2.34097	-4.12731
Ciclev10009680m.g	scaffold_1:22093835-22094718	48.4852	2.77852	-4.12516
Ciclev10002433m.g	scaffold_5:39379288-39380137	8.89058	0.512336	-4.11712
Ciclev10007394m.g	scaffold_1:24741259-24748937	0.80478	0.0464487	-4.11488
Ciclev10023251m.g	scaffold_3:31553969-31555056	25.1173	1.45459	-4.11
Ciclev10019427m.g,Ciclev10019611m.g	scaffold_3:6736405-6745205	49.8206	2.90439	-4.10043
Ciclev10017098m.g	scaffold_2:32213298-32215077	3.54239	0.206646	-4.09949
Ciclev10007651m.g	scaffold_1:7114557-7118282	5.15191	0.300627	-4.09906
Ciclev10005992m.g	scaffold_9:5417912-5418877	244.598	14.3144	-4.09487
Ciclev10030884m.g	scaffold_4:23234711-23239227	1.59272	0.0936595	-4.08792
Ciclev10003510m.g	scaffold_5:35894200-35898548	5.61787	0.332548	-4.07839
Ciclev10001854m.g	scaffold_5:36229309-36230518	37.3488	2.21389	-4.07641
Ciclev10022825m.g	scaffold_3:5829458-5830556	6.7554	0.401008	-4.07434
Ciclev10019364m.g	scaffold_3:29351853-29354190	460.528	27.3442	-4.07399
Ciclev10027663m.g	scaffold_8:21712356-21744083	13.1139	0.779982	-4.07151
Ciclev10008076m.g	scaffold_1:23261480-23263107	1.71815	0.102354	-4.06922
Ciclev10010155m.g	scaffold_1:25325244-25331925	1.09859	0.0655544	-4.06681
Ciclev10014941m.g	scaffold_2:35505675-35509846	27.6152	1.65638	-4.05936
-	scaffold_9:6736768-6737101	15.322	0.919374	-4.05881
Ciclev10023363m.g	scaffold_3:8908352-8913829	3.1723	0.191068	-4.05337
Ciclev10029025m.g,Ciclev10029347m.g	scaffold_8:18863193-18917480	64.6402	3.90907	-4.04753
Ciclev10012922m.g	scaffold_6:19708360-19708885	10.8889	0.660815	-4.04246
Ciclev10017613m.g	scaffold_2:32301724-32302396	4.64709	0.283131	-4.03678
Ciclev10005946m.g	scaffold_9:27625898-27626835	7.03114	0.429121	-4.0343
Ciclev10017299m.g	scaffold_2:28030133-28030667	16.9983	1.0375	-4.03421
Ciclev10012971m.g	scaffold_6:22731981-22732700	16.7884	1.02573	-4.03273
Ciclev10028253m.g	scaffold_8:20027849-20049674	22.966	1.40365	-4.03225
Ciclev10006056m.g	scaffold_9:2676492-2677559	50.8465	3.11275	-4.02989
Ciclev10032987m.g	scaffold_4:22161570-22162681	30.9335	1.89875	-4.02605
-	scaffold_8:11457127-11457324	276.313	17.0214	-4.02088
Ciclev10009639m.g	scaffold_1:18140595-18142122	124.73	7.72148	-4.01379
Ciclev10020552m.g	scaffold_3:31620762-31715816	1.44799	0.0898912	-4.00973
Ciclev10015583m.g	scaffold_2:9164803-9166682	4.01814	0.250017	-4.00643
Ciclev10020415m.g	scaffold_3:11687448-12154880	299.326	18.6678	-4.0031
Ciclev10021041m.g	scaffold_3:632881-635612	6.95885	0.434548	-4.00126
Ciclev10013424m.g	scaffold_6:2526698-2527592	7.60691	0.477786	-3.99287
Ciclev10033437m.g	scaffold_4:17794979-17796765	2.59563	0.163555	-3.98824
Ciclev10019202m.g	scaffold_3:42509538-42515993	1.00766	0.0635686	-3.98656
Ciclev10009650m.g	scaffold_1:7296550-7297509	387.327	24.4389	-3.9863
Ciclev10030121m.g	scaffold_8:16047465-16047711	31.8023	2.00738	-3.98574
Ciclev10014632m.g	scaffold_2:32488694-32493305	369.998	23.4051	-3.98262
Ciclev10026400m.g	scaffold_7:9976620-9980176	4.63238	0.29381	-3.9788
Ciclev10029174m.g	scaffold_8:13205352-13206868	74.7181	4.76734	-3.9702
Ciclev10027941m.g	scaffold_8:24702274-24706747	7.33032	0.468358	-3.96819
Ciclev10029863m.g	scaffold_8:1597559-1598760	3.10956	0.198961	-3.96616
Ciclev10020552m.g	scaffold_3:31620762-31715816	140.207	8.97519	-3.96547

Ciclev10028941m.g	scaffold_8:24502069-24504031	4.06455	0.260494	-3.96378
Ciclev10009194m.g	scaffold_1:3986692-3988340	32.0158	2.05202	-3.96367
Ciclev10020935m.g	scaffold_3:40289940-40295349	1.85674	0.119277	-3.96039
Ciclev10031299m.g	scaffold_4:20876051-20880567	4.53098	0.291446	-3.95852
-	scaffold_5:653491-653810	15.67	1.01307	-3.95119
-	scaffold_1:16766047-17038798	13.2177	0.856817	-3.94734
Ciclev10001432m.g	scaffold_5:9941408-9943881	8.72073	0.565686	-3.94638
Ciclev10013304m.g	scaffold_6:1516209-1517464	323.398	20.9908	-3.94548
Ciclev10014639m.g	scaffold_2:35235516-35237892	3.11356	0.202685	-3.94125
Ciclev10005147m.g	scaffold_9:31044658-31047363	14.5939	0.95266	-3.93725
-	scaffold_7:20327950-20328419	29.5266	1.92859	-3.9364
Ciclev10017916m.g	scaffold_2:35210722-35210959	72.9481	4.79548	-3.92712
Ciclev10012352m.g	scaffold_6:24812610-24814243	6.40338	0.423681	-3.91778
Ciclev10005533m.g	scaffold_9:6747773-6752563	4.43078	0.293411	-3.91656
Ciclev10014082m.g	scaffold_2:7507436-7569782	46.7934	3.10924	-3.91167
Ciclev10019411m.g	scaffold_3:42162086-42164051	1.04118	0.0692546	-3.91016
Ciclev10010007m.g	scaffold_1:5013073-5013862	30.898	2.06935	-3.90026
Ciclev10005703m.g	scaffold_9:29174471-29180718	9.70212	0.650537	-3.8986
-	scaffold_3:40504072-40719738	6.73912	0.453592	-3.89309
Ciclev10012170m.g	scaffold_6:11043781-11046075	1.73956	0.117605	-3.8867
Ciclev10008009m.g	scaffold_1:1149149-1151996	1.0223	0.069281	-3.88321
Ciclev10015182m.g	scaffold_2:31277543-31279358	80.3054	5.46895	-3.87616
Ciclev10028254m.g	scaffold_8:20027849-20049674	174.038	11.8657	-3.87453
Ciclev10016513m.g	scaffold_2:34804822-34810333	13.7174	0.941296	-3.86522
-	scaffold_1:11024085-11028084	55.0723	3.78186	-3.86416
Ciclev10032018m.g	scaffold_4:6175118-6178220	82.3179	5.6798	-3.85729
Ciclev10013296m.g	scaffold_6:18180430-18181409	9.53333	0.65788	-3.85708
Ciclev10028754m.g	scaffold_8:21848604-21851737	14.8662	1.02774	-3.85448
-	scaffold_1:11018781-11020843	1053.37	72.9011	-3.85293
Ciclev10012544m.g	scaffold_6:17786943-17788933	1.84553	0.127811	-3.85195
Ciclev10028017m.g	scaffold_8:21771140-21777001	1.68388	0.116754	-3.85025
Ciclev10013751m.g	scaffold_6:17093918-17098803	2.8052	0.194638	-3.84924
Ciclev10030744m.g	scaffold_4:2020042-2025300	2.51874	0.174899	-3.84811
Ciclev10000260m.g	scaffold_5:41432161-41439014	27.2219	1.89598	-3.84375
Ciclev10017020m.g	scaffold_2:34758767-34759899	7.77454	0.542721	-3.84047
Ciclev10031522m.g	scaffold_4:1976174-1977527	1.63726	0.114654	-3.83593
Ciclev10005327m.g,Ciclev10005484m.g	scaffold_9:280843-294262	12.8926	0.9035	-3.83487
-	scaffold_6:1749221-1749526	16.0759	1.12749	-3.83372
Ciclev10031705m.g	scaffold_4:19720290-19721661	2.54871	0.179414	-3.8284
Ciclev10000630m.g	scaffold_5:34230287-34232445	27.3573	1.94214	-3.8162
Ciclev10027138m.g	scaffold_7:8550403-8551979	2.17484	0.154757	-3.81283
Ciclev10028764m.g	scaffold_8:4404811-4408202	1.62817	0.116065	-3.81024
Ciclev10020017m.g	scaffold_3:2073077-2074731	1.24179	0.0885539	-3.80972
-	scaffold_8:13576402-13576818	8.28886	0.592189	-3.80704
Ciclev10022438m.g	scaffold_3:23315528-23316461	7.17284	0.514993	-3.79992
Ciclev10014129m.g	scaffold_2:26050435-26057582	1.10259	0.079523	-3.79338
-	scaffold_2:22087909-22088135	80.4395	5.81404	-3.79029
Ciclev10030368m.g	scaffold_8:5469163-5470417	1.6224	0.11734	-3.78937
Ciclev10006113m.g	scaffold_9:30616497-30621519	2.90377	0.210036	-3.78922
Ciclev10022120m.g	scaffold_3:45622522-45623522	66.8707	4.8551	-3.7838
Ciclev10032972m.g	scaffold_4:9244726-9245718	5.95216	0.432501	-3.78264
Ciclev10023551m.g	scaffold_3:6698813-6702490	0.899776	0.0656293	-3.77715
Ciclev10032304m.g	scaffold_4:24041592-24043549	187.446	13.7235	-3.77176
Ciclev10023060m.g	scaffold_3:13416060-13417219	673.177	50.1577	-3.74644
Ciclev10005374m.g	scaffold_9:3464275-3467426	5.76271	0.430564	-3.74245

Ciclev10010833m.g	scaffold_1:26233402-26235119	1.36932	0.102453	-3.74043
Ciclev10003164m.g	scaffold_5:22919338-23141855	1.33462	0.0999418	-3.7392
Ciclev10019915m.g	scaffold_3:47861998-47863895	36.7237	2.75278	-3.73775
Ciclev10022355m.g	scaffold_3:45800027-45801135	2.38045	0.178469	-3.73749
Ciclev10024940m.g	scaffold_7:6398676-6401404	1.60588	0.120992	-3.73038
Ciclev10032355m.g	scaffold_4:1849347-1851693	2.62001	0.1985	-3.72236
Ciclev10000770m.g	scaffold_5:43153229-43157246	3.99014	0.302474	-3.72156
Ciclev10006357m.g	scaffold_9:647899-648331	209.196	15.8881	-3.71883
Ciclev10019147m.g	scaffold_3:2939619-2941934	40.2366	3.06293	-3.71552
-	scaffold_2:25676129-25676719	4.43912	0.338405	-3.71345
Ciclev10029412m.g	scaffold_8:24293264-24293846	3.40583	0.259781	-3.71264
Ciclev10001469m.g	scaffold_5:13187677-13190472	97.4199	7.43324	-3.71215
Ciclev10001994m.g	scaffold_5:38307996-38311278	9.58091	0.733606	-3.70709
Ciclev10002693m.g	scaffold_5:37891704-37892899	234.726	17.9738	-3.70701
Ciclev10009893m.g	scaffold_1:4752414-4753820	112.172	8.60548	-3.70432
Ciclev10011703m.g	scaffold_6:19628726-19632850	25.7326	1.98021	-3.69987
-	scaffold_5:43057459-43058107	3.8426	0.29608	-3.69803
Ciclev10032995m.g	scaffold_4:16694754-16695286	19.3943	1.5008	-3.69182
Ciclev10008045m.g	scaffold_1:2211677-2213570	1.2567	0.0975814	-3.68689
Ciclev10015737m.g	scaffold_2:29603745-29604981	1.43727	0.111895	-3.68312
Ciclev10013536m.g	scaffold_6:22616978-22619271	14.9376	1.16462	-3.68101
Ciclev10024825m.g	scaffold_7:175846-180673	66.0921	5.15842	-3.67948
Ciclev10025730m.g	scaffold_7:20111321-20115685	36.9448	2.88797	-3.67725
-	scaffold_2:9184392-9185979	61.9242	4.85413	-3.67322
Ciclev10006212m.g	scaffold_9:26596358-26597142	30.3147	2.38292	-3.66921
Ciclev10000909m.g	scaffold_5:101796-108313	9.48	0.745196	-3.6692
Ciclev10028101m.g	scaffold_8:8041480-8043984	145.148	11.41	-3.66915
Ciclev10003989m.g	scaffold_5:5815714-5818239	9.85053	0.776221	-3.66566
Ciclev10020705m.g	scaffold_3:48188237-48190851	2.30216	0.181452	-3.66533
Ciclev10014882m.g	scaffold_2:29616639-29620120	14.5886	1.15428	-3.65977
Ciclev10004530m.g	scaffold_9:25713468-25717244	0.725736	0.0575336	-3.65697
Ciclev10023099m.g	scaffold_3:47255283-47259374	169.266	13.4241	-3.6564
Ciclev10016355m.g	scaffold_2:31808374-31811065	5.98439	0.474682	-3.65617
-	scaffold_5:13212009-13212982	42.6712	3.40156	-3.64899
Ciclev10026734m.g	scaffold_7:585106-585970	3.4594	0.27711	-3.64199
Ciclev10022041m.g	scaffold_3:18363071-18364170	6998.79	565.551	-3.62938
Ciclev10019920m.g	scaffold_3:1478431-1481737	47.678	3.85608	-3.62812
Ciclev10029032m.g	scaffold_8:19288176-19290093	24.6979	2.00034	-3.62607
Ciclev10017922m.g	scaffold_2:18404214-18404869	23.6672	1.92065	-3.62322
Ciclev10032331m.g	scaffold_4:21222709-21223927	6.61115	0.537822	-3.6197
Ciclev10019694m.g	scaffold_3:1582674-1586208	10.3363	0.842194	-3.61742
Ciclev10006916m.g	scaffold_9:27992868-27993378	5.27378	0.429745	-3.61729
Ciclev10001416m.g	scaffold_5:39875927-39877965	6.13625	0.501927	-3.61181
Ciclev10030732m.g	scaffold_4:1206775-1209356	27.3415	2.24039	-3.60927
Ciclev10005075m.g	scaffold_9:13365217-13368617	23.6984	1.9425	-3.6088
Ciclev10016982m.g	scaffold_2:33654375-33655865	34.0189	2.80396	-3.6008
Ciclev10005144m.g	scaffold_9:25192480-25197226	21.5218	1.77427	-3.6005
Ciclev10028720m.g	scaffold_8:548070-550407	3.09389	0.255117	-3.60019
Ciclev10027341m.g	scaffold_7:8900385-8902865	7.06881	0.583024	-3.59984
Ciclev10025810m.g	scaffold_7:181765-184804	1.5455	0.127876	-3.59525
Ciclev10024637m.g	scaffold_3:41697051-41697860	1.88045	0.155632	-3.59487
Ciclev10009942m.g	scaffold_1:27908366-27909139	9.8305	0.813682	-3.59473
Ciclev10018185m.g	scaffold_2:5273993-5337902	51.3857	4.25614	-3.59375
Ciclev10031403m.g	scaffold_4:1303839-1306777	2.4901	0.206582	-3.59141
Ciclev10004832m.g	scaffold_9:28081759-28084212	0.958509	0.0798033	-3.58627
Ciclev10004692m.g	scaffold_9:3892205-3895908	0.929595	0.0775451	-3.58349

Ciclev10012094m.g	scaffold_6:17469066-17471495	1.25777	0.10544	-3.57637
-	scaffold_6:10361042-10361232	669.361	56.2274	-3.57344
Ciclev10033593m.g	scaffold_4:16066332-16068145	1.75735	0.147624	-3.5734
Ciclev10001578m.g	scaffold_5:42170317-42173218	3.08641	0.259704	-3.57099
Ciclev10015595m.g	scaffold_2:12531372-12534397	111.321	9.37239	-3.57016
Ciclev10000913m.g	scaffold_5:36638720-36641298	10.7859	0.909514	-3.56791
-	scaffold_1:6381982-6382151	381.405	32.2553	-3.56372
Ciclev10014739m.g	scaffold_2:32789181-32791906	5.15894	0.43729	-3.56041
Ciclev10023053m.g	scaffold_3:33746265-33746994	27.301	2.31588	-3.55932
Ciclev10001131m.g	scaffold_5:29664370-29670123	7.37481	0.628622	-3.55234
Ciclev10006192m.g	scaffold_9:15045799-15049915	6.37494	0.543628	-3.55172
Ciclev10006470m.g	scaffold_9:29368680-29370684	1.53825	0.131249	-3.55092
Ciclev10023294m.g,Ciclev10023649m.g,Ciclev10024024m.g	scaffold_3:22043475-22177017	16.6173	1.42151	-3.54719
Ciclev10010053m.g	scaffold_1:28853211-28853916	8.99074	0.76918	-3.54705
Ciclev10032958m.g	scaffold_4:22636710-22637581	2.13951	0.183077	-3.54676
Ciclev10030876m.g	scaffold_4:22796096-22798154	21.9372	1.87901	-3.54533
Ciclev10017595m.g	scaffold_2:22426541-22427908	7.52662	0.648526	-3.53677
-	scaffold_9:7493315-7493492	30390.5	2646.16	-3.52165
Ciclev10032489m.g	scaffold_4:18947497-18952548	2.02017	0.176559	-3.51625
Ciclev10020714m.g	scaffold_3:16767-18635	36.0414	3.15003	-3.51621
Ciclev10033318m.g	scaffold_4:21097636-21097846	86.7231	7.57975	-3.51619
Ciclev10026322m.g	scaffold_7:3842848-3844735	13.3589	1.16994	-3.51329
Ciclev10016276m.g	scaffold_2:15671428-15672816	684.809	60.0567	-3.5113
Ciclev10029987m.g	scaffold_8:7909414-7965005	8.73374	0.772193	-3.49956
Ciclev10007183m.g	scaffold_9:902877-903717	7.9486	0.707664	-3.48956
Ciclev10006989m.g	scaffold_9:29415524-29424081	4.83259	0.430738	-3.48791
Ciclev10033111m.g	scaffold_4:22932585-22935026	5.86426	0.524403	-3.4832
Ciclev10026376m.g	scaffold_7:7340883-7342206	289.172	25.9	-3.4809
Ciclev10003015m.g	scaffold_5:17905405-17906193	2.65231	0.237686	-3.48012
Ciclev10009291m.g	scaffold_1:14007945-14009687	1.16012	0.104119	-3.47798
-	scaffold_7:1774752-1776831	275.604	24.7363	-3.4779
Ciclev10011768m.g	scaffold_6:17051740-17053964	3.86039	0.346627	-3.47729
Ciclev10005687m.g	scaffold_9:3453528-3455361	175.566	15.768	-3.47694
Ciclev10016590m.g	scaffold_2:26650149-26651148	16.8514	1.51509	-3.47539
Ciclev10014629m.g	scaffold_2:34070830-34075209	1.23863	0.1114	-3.47493
Ciclev10015968m.g	scaffold_2:32670180-32672307	5.59207	0.505089	-3.46877
-	scaffold_5:1311648-1355130	61.9051	5.59244	-3.46851
-	scaffold_4:16358966-16359507	12.7722	1.15465	-3.46748
Ciclev10030843m.g	scaffold_4:21976522-21979652	2.92149	0.265129	-3.46194
-	scaffold_8:7698015-7862701	357.294	32.5158	-3.4579
Ciclev10006007m.g	scaffold_9:7260018-7261355	1.93001	0.175997	-3.45499
Ciclev10011654m.g	scaffold_6:17735050-17737235	4.81324	0.438939	-3.45492
Ciclev10027413m.g	scaffold_7:7136553-7138237	2.97943	0.272518	-3.45061
Ciclev10030278m.g	scaffold_8:8820250-8820493	121.79	11.1636	-3.44752
Ciclev10027244m.g	scaffold_7:7835540-7836068	3.93488	0.363504	-3.43628
-	scaffold_2:23372723-23373335	8822.25	815.66	-3.43511
Ciclev10007519m.g	scaffold_1:25798689-25801889	141	13.0578	-3.43271
-	scaffold_6:145836-227910	152.95	14.1717	-3.43198
Ciclev10022829m.g	scaffold_3:7714687-7715660	78.1131	7.23916	-3.43167
Ciclev10021277m.g	scaffold_3:47366615-47370779	9.83649	0.913251	-3.42906
Ciclev10017145m.g	scaffold_2:23582148-23583162	52.6681	4.89236	-3.42833
Ciclev10017492m.g	scaffold_2:4340637-4345933	1.1955	0.111608	-3.42111
Ciclev10009420m.g	scaffold_1:21317417-21318453	20.3347	1.90016	-3.41975
Ciclev10005709m.g	scaffold_9:29399277-29401620	2.60007	0.243957	-3.41385

-	scaffold_2:7491286-7491557	411.932	38.6631	-3.41338
Ciclev10005561m.g	scaffold_9:17424109-17425707	15.9026	1.4933	-3.41269
Ciclev10026750m.g	scaffold_7:10736369-10737126	2.64002	0.24796	-3.41237
Ciclev10001511m.g	scaffold_5:33916070-33917739	1.95915	0.18446	-3.40885
Ciclev10028549m.g	scaffold_8:851241-853420	2.46734	0.232374	-3.40844
Ciclev10006286m.g	scaffold_9:2084224-2084935	270.063	25.4495	-3.40759
Ciclev10017541m.g	scaffold_2:13863970-13866058	0.810249	0.0764493	-3.40579
Ciclev10030893m.g	scaffold_4:2709803-2712515	7.84338	0.741102	-3.40373
-	scaffold_5:2868351-2868510	579.642	54.8517	-3.40155
Ciclev10032279m.g	scaffold_4:23695431-23697813	12.9306	1.22365	-3.40153
-	scaffold_2:28637253-28637663	25.6271	2.43152	-3.39774
Ciclev10007843m.g	scaffold_1:20440642-20444068	471.15	44.7903	-3.39493
Ciclev10019844m.g	scaffold_3:5363038-5365631	91.3953	8.70618	-3.39201
Ciclev10011655m.g	scaffold_6:23669284-23671821	8.80034	0.841032	-3.38733
Ciclev10011085m.g	scaffold_6:18048848-18051957	2.21228	0.211797	-3.38478
Ciclev10029932m.g	scaffold_8:8756363-8760308	59.7649	5.76738	-3.37331
Ciclev10002169m.g	scaffold_5:42790656-42792286	12.1333	1.17672	-3.36614
Ciclev10014073m.g	scaffold_2:13473154-13480880	0.859072	0.083633	-3.36064
Ciclev10011115m.g	scaffold_6:12707185-12711139	15.2748	1.49448	-3.35344
Ciclev10009526m.g	scaffold_1:1832788-1834175	2.51068	0.245784	-3.35261
Ciclev10024842m.g	scaffold_7:11850299-12160617	4.6111	0.451473	-3.3524
Ciclev10028831m.g	scaffold_8:461188-468899	165.627	16.2248	-3.35166
Ciclev10007249m.g	scaffold_1:5137336-5146463	16.41	1.60939	-3.34999
-	scaffold_3:18429131-18429278	66264.6	6509.45	-3.34763
-	scaffold_5:322517-322675	886.841	87.3244	-3.34422
Ciclev10002761m.g	scaffold_5:32261505-32263373	3.161	0.311503	-3.34307
Ciclev10026388m.g	scaffold_7:7794727-7795624	7.03359	0.693825	-3.34162
Ciclev10015218m.g	scaffold_2:19525670-19529613	5.40117	0.536344	-3.33204
Ciclev10033078m.g	scaffold_4:25412814-25413493	4.01033	0.39856	-3.33085
Ciclev10000221m.g	scaffold_5:23561670-23564601	1.54942	0.154201	-3.32885
Ciclev10000171m.g	scaffold_5:34929761-34934474	2.78361	0.277066	-3.32865
-	scaffold_8:10966618-10966924	33.6056	3.35546	-3.32412
Ciclev10027321m.g	scaffold_7:1404812-1406356	1.46297	0.146202	-3.32286
Ciclev10029762m.g	scaffold_8:2817340-2822179	2.9954	0.300277	-3.31839
Ciclev10018109m.g	scaffold_2:15837608-15838865	3.60596	0.362653	-3.31372
Ciclev10002881m.g	scaffold_5:34523831-34526367	2.23975	0.225584	-3.3116
Ciclev10033373m.g	scaffold_4:9286940-9288295	2.65262	0.267814	-3.30812
Ciclev10033573m.g	scaffold_4:19960034-19961246	11.3138	1.14738	-3.30167
Ciclev10026538m.g	scaffold_7:20656098-20658251	44.6907	4.55069	-3.29582
Ciclev10026803m.g	scaffold_7:9468244-9468907	10.7371	1.09332	-3.29582
Ciclev10030698m.g	scaffold_4:24651072-24655710	11.2705	1.14812	-3.29521
Ciclev10020222m.g	scaffold_3:43135377-43138351	7.86877	0.801685	-3.29503
Ciclev10013763m.g	scaffold_6:21798178-21801338	1.3137	0.133848	-3.29496
Ciclev10011088m.g	scaffold_6:21745027-21747494	0.615057	0.0627876	-3.29217
Ciclev10004677m.g	scaffold_9:24171226-24173238	14.2827	1.45889	-3.29133
-	scaffold_6:14408773-14408971	376.957	38.5971	-3.28784
Ciclev10004568m.g	scaffold_9:15238224-15241226	0.828684	0.0850935	-3.2837
Ciclev10023965m.g	scaffold_3:39127971-39130179	1.53997	0.158225	-3.28285
Ciclev10008616m.g	scaffold_1:23733464-23736028	2.67559	0.275062	-3.28203
-	scaffold_8:654842-655963	178.785	18.3887	-3.28133
Ciclev10033254m.g	scaffold_4:23892713-23896882	49.5473	5.09658	-3.28121
Ciclev10022025m.g	scaffold_3:50492189-50493037	7.45976	0.770417	-3.27542
Ciclev10029455m.g	scaffold_8:1103305-1104886	4.15794	0.430189	-3.27283
Ciclev10025688m.g	scaffold_7:1498560-1500104	3.64795	0.37774	-3.27162
Ciclev10009168m.g	scaffold_1:6503300-6505091	1.42188	0.147345	-3.27052
Ciclev10027912m.g	scaffold_8:21712356-21744083	4.7752	0.495521	-3.26854

Ciclev10019494m.g	scaffold_3:2210484-2215202	13.2867	1.37899	-3.2683
Ciclev10028087m.g	scaffold_8:24456357-24459375	0.728263	0.0757698	-3.26476
Ciclev10019940m.g	scaffold_3:44205870-44208997	43.5659	4.53441	-3.26421
Ciclev10018481m.g	scaffold_3:50910496-50917526	1.00145	0.104383	-3.26214
Ciclev10018595m.g	scaffold_3:9970358-9975305	10.7954	1.1258	-3.2614
Ciclev10020962m.g	scaffold_3:44822522-44823980	20.66	2.15939	-3.25815
Ciclev10019107m.g	scaffold_3:9414396-9419290	61.315	6.42425	-3.25464
Ciclev10002351m.g	scaffold_5:39619674-39626261	26.362	2.76289	-3.25421
Ciclev10006302m.g	scaffold_9:1067608-1069647	6.50033	0.681995	-3.25268
-	scaffold_8:16702101-16702243	2700.39	283.907	-3.24968
Ciclev10027084m.g	scaffold_7:611716-612950	5.04596	0.531091	-3.2481
Ciclev10007673m.g	scaffold_1:12900391-12905055	11.6528	1.22664	-3.2479
Ciclev10032369m.g	scaffold_4:1591644-1639717	1.32726	0.139765	-3.24738
Ciclev10031957m.g	scaffold_4:19498805-19500222	1.14972	0.12107	-3.24737
Ciclev10012276m.g	scaffold_6:21048051-21049344	1.37114	0.144389	-3.24734
Ciclev10000761m.g	scaffold_5:43016597-43020200	60.3618	6.35848	-3.24688
Ciclev10027102m.g	scaffold_7:13916806-14069972	7.52858	0.793114	-3.24678
Ciclev10015469m.g	scaffold_2:24379680-24381884	17.2622	1.8186	-3.24672
Ciclev10013182m.g	scaffold_6:16349273-16352688	0.568655	0.0599656	-3.24535
Ciclev10009892m.g	scaffold_1:1676367-1677018	37.1822	3.93406	-3.24052
Ciclev10004838m.g	scaffold_9:28042023-28044097	20.4877	2.17046	-3.23868
Ciclev10012197m.g	scaffold_6:13439324-13443911	1.03125	0.109528	-3.23503
Ciclev10011127m.g	scaffold_6:25107388-25111099	0.58184	0.0620785	-3.22846
Ciclev10014681m.g	scaffold_2:36339105-36342902	1.69647	0.181464	-3.22478
Ciclev10003186m.g	scaffold_5:29751-30872	2.36565	0.253326	-3.22317
Ciclev10020744m.g	scaffold_3:48321395-48324697	270.041	28.9341	-3.22234
Ciclev10003202m.g	scaffold_5:22625617-22628186	70.0365	7.50492	-3.2222
Ciclev10028495m.g	scaffold_8:23871220-23873313	47.5795	5.0986	-3.22217
Ciclev10032829m.g,Ciclev10033500m.g	scaffold_4:17127920-17132002	30.7831	3.29889	-3.22208
Ciclev10017209m.g	scaffold_2:35905503-35906345	30.5554	3.27639	-3.22125
Ciclev10028964m.g	scaffold_8:461188-468899	1889.12	202.863	-3.21913
Ciclev10004520m.g	scaffold_9:4058003-4062738	3.21914	0.345694	-3.2191
Ciclev10026130m.g	scaffold_7:2926256-2928054	7.03044	0.757389	-3.21451
Ciclev10033190m.g	scaffold_4:8406631-8407188	3.56563	0.385515	-3.2093
-	scaffold_2:33521059-33522140	46.9973	5.08203	-3.2091
-	scaffold_5:655534-811195	120.506	13.0519	-3.20678
-	scaffold_5:25884729-25885706	4.79688	0.5196	-3.20662
Ciclev10033156m.g	scaffold_4:12415429-12415833	5.45112	0.590796	-3.20582
Ciclev10021106m.g	scaffold_3:50253005-50255392	2.7225	0.295322	-3.20458
Ciclev10022546m.g	scaffold_3:172151-173201	10.5221	1.14301	-3.20251
Ciclev10026270m.g	scaffold_7:4392029-4392845	4.7102	0.512701	-3.1996
Ciclev10007919m.g	scaffold_1:26032708-26035828	7.61744	0.829446	-3.19909
Ciclev10014351m.g	scaffold_2:24921557-24926440	3.80567	0.41543	-3.19547
-	scaffold_3:34344903-34345231	26.1137	2.85246	-3.19453
Ciclev10021103m.g	scaffold_3:44193714-44195060	4.02005	0.439226	-3.19418
-	scaffold_5:4076819-4284428	11.2194	1.22659	-3.19327
Ciclev10013050m.g	scaffold_6:15050805-15054153	7.63129	0.838527	-3.186
Ciclev10008531m.g	scaffold_1:7564442-7569154	27.4149	3.01707	-3.18374
Ciclev10021409m.g	scaffold_3:49354444-49357035	3.69442	0.407148	-3.18173
Ciclev10007179m.g	scaffold_9:11355736-11362565	0.302249	0.0333296	-3.18086
-	scaffold_3:8136868-8137031	1189.8	131.35	-3.17922
Ciclev10002399m.g	scaffold_5:35872808-35892772	61.1898	6.75567	-3.17912
Ciclev10033856m.g	scaffold_4:6454454-6455489	1.62991	0.179992	-3.17879
Ciclev10022299m.g	scaffold_3:38345734-38347292	10.9725	1.21468	-3.17524
Ciclev10025957m.g	scaffold_7:8218470-8221720	15.5787	1.72988	-3.17083

Ciclev10023354m.g	scaffold_3:19936498-19941380	4.90917	0.545363	-3.17019
Ciclev10007289m.g	scaffold_1:23843155-23850135	1.50401	0.167128	-3.16979
-	scaffold_5:14998571-14999005	34.8773	3.8829	-3.16708
Ciclev10021699m.g	scaffold_3:43420186-43422432	7.82988	0.874181	-3.16299
-	scaffold_1:24010530-24012772	3.90134	0.435828	-3.16214
Ciclev10004709m.g	scaffold_9:2994223-2997285	1.39888	0.15645	-3.1605
Ciclev10025820m.g	scaffold_7:2450661-2454470	1.5944	0.17873	-3.15716
Ciclev10031049m.g	scaffold_4:1515155-1517052	1.62097	0.181835	-3.15615
Ciclev10019174m.g	scaffold_3:48026617-48031503	4.07106	0.457698	-3.15294
Ciclev10019457m.g	scaffold_3:49437523-49445402	67.3546	7.57293	-3.15285
Ciclev10020446m.g	scaffold_3:46191686-46193918	5.71848	0.642967	-3.15281
Ciclev10030920m.g	scaffold_4:1277010-1280156	1654.66	186.265	-3.1511
Ciclev10030075m.g	scaffold_8:6944685-7159404	39.5378	4.4566	-3.14922
Ciclev10032937m.g	scaffold_4:19956091-19956932	7.22177	0.816855	-3.1442
Ciclev10026128m.g	scaffold_7:5258044-5259521	209.773	23.7514	-3.14274
Ciclev10009285m.g	scaffold_1:26683179-26687729	64.5607	7.31558	-3.14161
Ciclev10016251m.g	scaffold_2:28080388-28083539	2.88409	0.326887	-3.14125
Ciclev10016244m.g	scaffold_2:35151819-35153284	1.08724	0.12425	-3.12935
Ciclev10016463m.g	scaffold_2:33673906-33675071	420.496	48.0696	-3.1289
-	scaffold_5:1311648-1355130	3.15664	0.361784	-3.12519
Ciclev10022410m.g	scaffold_3:48051030-48051986	58.1431	6.66544	-3.12484
Ciclev10012926m.g	scaffold_6:22794370-22795208	10.3608	1.19443	-3.11674
Ciclev10018378m.g	scaffold_2:28629483-28632491	2.72132	0.313764	-3.11655
-	scaffold_6:18079179-18079713	6.78777	0.78517	-3.11186
Ciclev10008218m.g	scaffold_1:23456082-23459416	21.7492	2.51699	-3.11119
Ciclev10033331m.g	scaffold_4:2408399-2410046	0.811034	0.0938841	-3.11081
Ciclev10033362m.g	scaffold_4:18981047-18982979	0.891241	0.103177	-3.11069
Ciclev10004474m.g	scaffold_9:543443-546746	452.484	52.4099	-3.10995
Ciclev10023752m.g	scaffold_3:43388179-43391091	0.949872	0.110082	-3.10915
Ciclev10000421m.g	scaffold_5:40589993-40595575	1.07403	0.124639	-3.1072
Ciclev10032323m.g	scaffold_4:23897863-23901686	2.30109	0.268612	-3.09872
Ciclev10007784m.g	scaffold_1:27562750-27565535	1.2459	0.145748	-3.09565
Ciclev10027164m.g	scaffold_7:18387836-18390858	2.78378	0.325775	-3.09509
-	scaffold_5:17540360-17540546	16064	1881.89	-3.09358
Ciclev10016240m.g	scaffold_2:32976770-32978433	1.493	0.174957	-3.09314
Ciclev10022800m.g	scaffold_3:45299456-45303593	9.28461	1.08853	-3.09247
Ciclev10000747m.g	scaffold_5:32672661-32675477	18.0483	2.11778	-3.09124
Ciclev10018774m.g	scaffold_3:27807171-27813248	4.12909	0.485807	-3.08737
Ciclev10003594m.g	scaffold_5:37897415-37901654	0.867193	0.102032	-3.08733
Ciclev10030254m.g	scaffold_8:24322023-24324111	0.933854	0.109948	-3.08638
Ciclev10003452m.g	scaffold_5:16198486-16201340	0.51287	0.060427	-3.08533
Ciclev10017975m.g	scaffold_2:7507436-7569782	8.34078	0.983301	-3.08448
Ciclev10030625m.g	scaffold_4:2261149-2266209	3.48651	0.411741	-3.08197
Ciclev10015194m.g	scaffold_2:7812774-7814952	63.0837	7.45176	-3.08161
Ciclev10005038m.g	scaffold_9:3088508-3089928	587.023	69.4289	-3.07981
-	scaffold_8:7698015-7862701	49.788	5.89703	-3.07774
Ciclev10001970m.g	scaffold_5:36219782-36221019	7.88362	0.935817	-3.07456
Ciclev10031707m.g	scaffold_4:20978332-20980853	1.56167	0.185482	-3.07374
Ciclev10013262m.g,Ciclev10013503m.g	scaffold_6:18374365-18380210	306.714	36.5166	-3.07027
Ciclev10010708m.g	scaffold_1:5492296-5494405	24.3605	2.90332	-3.06877
Ciclev10026035m.g	scaffold_7:6230553-6232339	13.9018	1.65707	-3.06856
Ciclev10014832m.g	scaffold_2:21177784-21180989	1.25613	0.149846	-3.06744
-	scaffold_13:59225-59830	13.657	1.6317	-3.06519
-	scaffold_5:326948-328013	42.2584	5.06205	-3.06145
Ciclev10011029m.g	scaffold_6:20309804-20313653	3.60843	0.432917	-3.05921

Ciclev10024516m.g	scaffold_3:32618757-32621932	2.57533	0.30898	-3.05917
Ciclev10004944m.g	scaffold_9:8338761-8342569	0.943854	0.113357	-3.05769
Ciclev10031996m.g	scaffold_4:23089670-23090833	6.81244	0.818531	-3.05706
Ciclev10016684m.g	scaffold_2:27460832-27461914	89.5285	10.8176	-3.04897
Ciclev10028082m.g	scaffold_8:89020-91588	36.5212	4.41703	-3.04759
Ciclev10020894m.g	scaffold_3:8545461-8547035	3.6984	0.447308	-3.04756
-	scaffold_5:28021736-28021909	664.768	80.4919	-3.04594
Ciclev10012707m.g	scaffold_6:10149054-10152999	1.13069	0.136994	-3.04502
Ciclev10001086m.g	scaffold_5:18104382-18105975	2.50496	0.303671	-3.04421
Ciclev10012557m.g	scaffold_6:23093342-23094697	1.12325	0.136223	-3.04363
-	scaffold_5:2534144-2731098	14.3975	1.74621	-3.04352
Ciclev10024697m.g	scaffold_7:10436107-10442709	1.05662	0.128154	-3.04351
Ciclev10016106m.g	scaffold_2:26016188-26017223	4.08146	0.495052	-3.04343
Ciclev10029154m.g	scaffold_8:4466846-4468794	2.76345	0.336189	-3.03912
Ciclev10007699m.g	scaffold_1:24588855-24591166	1.62684	0.198955	-3.03156
-	scaffold_7:18696310-18696436	30182.4	3691.34	-3.03149
-	scaffold_2:6181064-6338878	29.301	3.58397	-3.03132
Ciclev10033110m.g	scaffold_4:25171279-25172219	28.7908	3.5259	-3.02954
Ciclev10000816m.g	scaffold_5:33695911-33697986	3.82995	0.47079	-3.02417
Ciclev10028548m.g	scaffold_8:24610689-24612555	3.46784	0.427399	-3.02038
Ciclev10009180m.g,Ciclev10010303m.g	scaffold_1:27880115-27883611	258.242	31.8546	-3.01915
-	scaffold_6:19934066-19935899	5.65855	0.701779	-3.01134
Ciclev10019351m.g	scaffold_3:44215332-44220917	5.63227	0.699547	-3.00922
Ciclev10011456m.g	scaffold_6:20973975-20977952	3.25225	0.404029	-3.00891
Ciclev10011189m.g	scaffold_6:25014493-25016623	3.79545	0.471861	-3.00784
Ciclev10000098m.g	scaffold_5:26641956-26646157	1.3973	0.174406	-3.00212
Ciclev10011795m.g	scaffold_6:22985432-22987317	6.13884	0.767529	-2.99967
Ciclev10006064m.g	scaffold_9:2981467-2982057	2.17218	0.271956	-2.9977
Ciclev10016386m.g	scaffold_2:11388043-11389521	1.80173	0.226564	-2.99139
-	scaffold_2:14746000-14746162	41691.6	5248.24	-2.98985
Ciclev10016694m.g	scaffold_2:28111468-28112452	24.8955	3.14389	-2.98526
Ciclev10007746m.g	scaffold_1:20039958-20042536	1.13996	0.144	-2.98483
Ciclev10003935m.g	scaffold_5:42084911-42087039	1.51692	0.192349	-2.97934
Ciclev10026710m.g	scaffold_7:7838618-7840273	270.163	34.2895	-2.97799
Ciclev10029149m.g	scaffold_8:23240222-23241308	4.90088	0.622545	-2.97679
Ciclev10003679m.g	scaffold_5:25221155-25545745	4.12972	0.525431	-2.97447
Ciclev10024819m.g,Ciclev10027295m.g	scaffold_7:7202245-7222786	176.657	22.4913	-2.97351
-	scaffold_5:37381117-37384861	18.7958	2.39304	-2.97349
-	scaffold_4:13715807-13716058	46.2317	5.89699	-2.97083
Ciclev10031096m.g	scaffold_4:2382094-2384251	2.10371	0.268348	-2.97076
-	scaffold_3:45176258-45177778	30.564	3.90311	-2.96913
Ciclev10013748m.g	scaffold_6:20517773-20519981	2.02989	0.259675	-2.96663
Ciclev10019616m.g	scaffold_3:1751879-1754219	11.1201	1.42594	-2.96318
Ciclev10016041m.g	scaffold_2:8592978-8594800	6.89221	0.884788	-2.96156
Ciclev10014501m.g	scaffold_2:28890087-28893154	0.478649	0.0614764	-2.96086
Ciclev10031625m.g	scaffold_4:16379986-16381763	22.9958	2.96027	-2.95757
Ciclev10011494m.g	scaffold_6:17100555-17102810	198.842	25.6228	-2.95612
Ciclev10031574m.g	scaffold_4:19662367-19664575	30.0817	3.88713	-2.95211
Ciclev10027587m.g	scaffold_7:8639039-8640373	41.9716	5.42358	-2.9521
Ciclev10028590m.g	scaffold_8:24576172-24578442	447.838	57.8968	-2.95142
Ciclev10004207m.g	scaffold_9:5611494-5618224	0.902751	0.116808	-2.95019
Ciclev10009338m.g	scaffold_1:25369130-25371445	9.02223	1.1684	-2.94895
Ciclev10001658m.g	scaffold_5:40728916-40730477	19.9776	2.58754	-2.94873
Ciclev10025915m.g	scaffold_7:2416432-2419376	264.109	34.2453	-2.94715

Ciclev10020715m.g	scaffold_3:46149264-46153166	11.156	1.44685	-2.94683
Ciclev10009540m.g	scaffold_1:24667692-24668658	1281.51	166.673	-2.94275
Ciclev10009722m.g	scaffold_1:24764471-24765333	5.15278	0.672138	-2.93852
-	scaffold_9:17385183-17385418	9298.85	1214.14	-2.93712
Ciclev10011796m.g	scaffold_6:22186299-22187922	8.98695	1.17382	-2.93661
Ciclev10008063m.g	scaffold_1:2660827-2662929	15.1158	1.97483	-2.93626
Ciclev10002986m.g	scaffold_5:33975036-33978373	796.997	104.198	-2.93525
Ciclev10015478m.g	scaffold_2:10157391-10159312	10.1366	1.32786	-2.9324
Ciclev10022753m.g	scaffold_3:4311922-4312682	78.2579	10.2548	-2.93194
Ciclev10008921m.g	scaffold_1:24175844-24190285	6.29641	0.828191	-2.92649
Ciclev10002947m.g	scaffold_5:33399338-33401428	179.981	23.7521	-2.92172
Ciclev10013256m.g	scaffold_6:17209379-17211516	8.4218	1.11285	-2.91987
Ciclev10020627m.g	scaffold_3:238937-243369	2.73005	0.360818	-2.91959
Ciclev10028947m.g	scaffold_8:21289995-21292877	1.03529	0.136871	-2.91915
Ciclev10033393m.g	scaffold_4:6445702-6447613	8.81286	1.16524	-2.91898
Ciclev10029994m.g	scaffold_8:4178523-4179087	7.33876	0.971223	-2.91766
-	scaffold_3:29715853-29716147	28.0216	3.70999	-2.91705
Ciclev10004755m.g	scaffold_9:28984971-28989373	23.0952	3.06148	-2.91529
Ciclev10009955m.g	scaffold_1:18169934-18176338	6.25113	0.829352	-2.91406
-	scaffold_5:30029371-30029562	153.825	20.417	-2.91345
Ciclev10011932m.g	scaffold_6:15054586-15056184	75.5246	10.0768	-2.90591
Ciclev10003474m.g	scaffold_5:34941722-34942336	5.47196	0.731205	-2.90371
Ciclev10011062m.g	scaffold_6:21404401-21408079	2.88532	0.385998	-2.90206
Ciclev10009740m.g	scaffold_1:14026556-14027565	1082.12	144.804	-2.90169
Ciclev10026573m.g	scaffold_7:4187098-4190237	4.02242	0.539113	-2.8994
Ciclev10009236m.g	scaffold_1:3862912-3866838	10.0441	1.35116	-2.89407
Ciclev10027976m.g	scaffold_8:1203082-1205664	0.439071	0.0590657	-2.89406
Ciclev10015825m.g	scaffold_2:35525984-35527550	2.03982	0.274765	-2.89217
Ciclev10017183m.g	scaffold_2:21493977-21494506	25.8894	3.48861	-2.89164
Ciclev10020006m.g	scaffold_3:8684447-8686118	11.7503	1.58368	-2.89134
Ciclev10017943m.g	scaffold_2:28908048-28909959	1.03494	0.139694	-2.88921
Ciclev10006445m.g	scaffold_9:2440046-2442253	2.40994	0.325722	-2.88728
Ciclev10030157m.g	scaffold_8:23765296-23767237	0.564456	0.0763517	-2.88613
Ciclev10016932m.g	scaffold_2:33707330-33709334	5.67117	0.76873	-2.8831
Ciclev10021153m.g	scaffold_3:26297948-26304739	13.7476	1.87082	-2.87744
Ciclev10025206m.g	scaffold_7:6806473-6808599	1.96759	0.267937	-2.87646
Ciclev10017800m.g	scaffold_2:10961697-10963150	0.94062	0.128337	-2.87368
Ciclev10030933m.g	scaffold_4:23370802-23372734	0.60752	0.0829323	-2.87293
Ciclev10010358m.g	scaffold_1:1627613-1630842	0.625537	0.0854239	-2.87238
Ciclev10032947m.g	scaffold_4:2525408-2526330	6.06246	0.828322	-2.87164
Ciclev10007992m.g	scaffold_1:9912913-9915380	0.659078	0.090198	-2.86928
Ciclev10025332m.g	scaffold_7:7666254-7668285	3.20169	0.438212	-2.86913
-	scaffold_5:2534144-2731098	281.217	38.5026	-2.86865
Ciclev10017057m.g	scaffold_2:10566017-10566892	53.2382	7.29128	-2.86822
Ciclev10031102m.g	scaffold_4:21735793-21738770	7.96794	1.09178	-2.86752
Ciclev10020875m.g	scaffold_3:20132361-20135883	3.45675	0.474081	-2.86621
-	scaffold_9:22711158-22712341	10.0992	1.38546	-2.86581
Ciclev10031540m.g	scaffold_4:23082915-23085609	117.677	16.1703	-2.86341
Ciclev10021701m.g	scaffold_3:41585375-41587019	1.02328	0.140689	-2.86262
Ciclev10026390m.g	scaffold_7:11307058-11308330	7.93783	1.09265	-2.86092
Ciclev10025417m.g	scaffold_7:14844174-14846782	19.0444	2.62167	-2.86081
Ciclev10001467m.g	scaffold_5:29056983-29060046	0.721355	0.0994908	-2.85808
Ciclev10018051m.g	scaffold_2:26811999-26813313	0.92884	0.128125	-2.85788
Ciclev10003491m.g	scaffold_5:35256589-35260160	3.64619	0.504417	-2.8537
-	scaffold_9:17239663-17239819	2612.74	362.207	-2.85068
Ciclev10016699m.g	scaffold_2:11121170-11126401	1.79868	0.249459	-2.85006

Ciclev10015551m.g	scaffold_2:6764624-6767330	2.73422	0.379565	-2.84871
Ciclev10005780m.g	scaffold_9:1539789-1541532	19.1809	2.6631	-2.84849
Ciclev10006929m.g	scaffold_9:29254533-29256893	2.21904	0.308333	-2.84737
Ciclev10027640m.g	scaffold_1723:182-3359	2.81222	0.391429	-2.84489
-	scaffold_8:9325618-9585469	9.95769	1.38712	-2.84372
Ciclev10002179m.g	scaffold_5:38447705-38448556	46.1678	6.43942	-2.84188
Ciclev10024767m.g	scaffold_7:3403053-3409092	5.15098	0.719412	-2.83996
Ciclev10003043m.g	scaffold_5:36023351-36024604	1.12439	0.157076	-2.8396
Ciclev10029617m.g	scaffold_8:19132836-19211350	1.27336	0.178133	-2.83762
Ciclev10000125m.g	scaffold_5:39941855-39952725	4.63609	0.649036	-2.83654
Ciclev10019309m.g	scaffold_3:45385156-45387558	2.70644	0.379186	-2.83542
Ciclev10029198m.g	scaffold_8:2817340-2822179	12.6487	1.77283	-2.83487
Ciclev10020075m.g	scaffold_3:12397236-12399414	1.98148	0.277896	-2.83397
Ciclev10014542m.g	scaffold_2:22661716-22666995	0.485131	0.0680698	-2.83329
Ciclev10030320m.g	scaffold_8:19132836-19211350	67.7269	9.51952	-2.83077
-	scaffold_7:11686788-11771145	56.7397	7.97984	-2.82993
Ciclev10021778m.g	scaffold_3:47931341-47933843	5.84257	0.822065	-2.82928
Ciclev10008414m.g	scaffold_1:3059165-3061483	1.55011	0.218718	-2.82523
Ciclev10013663m.g	scaffold_6:15162436-15162832	23.0512	3.25269	-2.82514
Ciclev10015623m.g	scaffold_2:2945400-2947403	12.7421	1.80077	-2.82292
Ciclev10004178m.g	scaffold_9:27704793-27713963	0.286617	0.0405477	-2.82143
Ciclev10005152m.g	scaffold_9:24624293-24625478	5.23285	0.740685	-2.82067
Ciclev10028711m.g	scaffold_8:8206052-8208375	5.55602	0.786465	-2.8206
Ciclev10007505m.g	scaffold_1:25193363-25195811	14.8734	2.10913	-2.81802
Ciclev10025521m.g	scaffold_7:4296122-4300353	7.01297	0.99677	-2.81469
Ciclev10027526m.g	scaffold_7:18584129-18585731	0.767984	0.109214	-2.81392
Ciclev10016972m.g	scaffold_2:30445448-30446994	12.0787	1.71824	-2.81346
Ciclev10007548m.g	scaffold_1:20535940-20543308	6.88993	0.980168	-2.81339
Ciclev10012045m.g	scaffold_6:15377565-15379102	1.61894	0.230905	-2.80968
Ciclev10009152m.g	scaffold_1:18808739-18811363	15.4249	2.20671	-2.80529
Ciclev10022709m.g	scaffold_3:46088036-46089232	9.57811	1.37278	-2.80264
Ciclev10013236m.g	scaffold_6:20459781-20460154	350.977	50.344	-2.80148
Ciclev10018706m.g	scaffold_3:41026622-41034652	0.296979	0.0426232	-2.80065
-	scaffold_8:21073700-21073919	162.271	23.2997	-2.80002
Ciclev10004558m.g	scaffold_9:15422640-15491415	0.598245	0.0859491	-2.79918
Ciclev10030803m.g	scaffold_4:312145-317203	52.4574	7.53816	-2.79886
Ciclev10018670m.g	scaffold_3:46090114-46095977	3.21704	0.463144	-2.7962
Ciclev10003688m.g	scaffold_5:21041859-21042911	28.5789	4.11668	-2.7954
Ciclev10017435m.g,Ciclev10018311m.g	scaffold_2:9758948-9769130	43.9897	6.34809	-2.79277
Ciclev10014341m.g	scaffold_2:13677327-13685473	50.6751	7.3185	-2.79166
Ciclev10001249m.g	scaffold_5:34809344-34813224	6.41409	0.930051	-2.78586
Ciclev10018160m.g	scaffold_2:4895612-4986690	2.00787	0.291578	-2.78371
Ciclev10027513m.g	scaffold_7:11686788-11771145	0.36907	0.053607	-2.7834
Ciclev10014333m.g	scaffold_2:9592457-9595926	23.8477	3.46434	-2.7832
Ciclev10030947m.g	scaffold_4:22714723-22717388	3.48225	0.506808	-2.78051
Ciclev10025985m.g	scaffold_7:15182949-15186486	9.50629	1.38437	-2.77966
-	scaffold_6:1209630-1209846	72.8336	10.6288	-2.77662
Ciclev10030383m.g	scaffold_8:2927914-2929165	1.21268	0.176969	-2.77662
-	scaffold_9:6090251-6090439	205.629	30.0372	-2.77522
Ciclev10020796m.g	scaffold_3:50711926-50714343	2.06251	0.301894	-2.77228
Ciclev10010310m.g	scaffold_1:472147-472570	4.51292	0.661337	-2.7706
Ciclev10028165m.g	scaffold_8:22936691-22940430	88.7033	12.9992	-2.77056
Ciclev10014177m.g	scaffold_2:33156191-33161018	9.0574	1.33231	-2.76517
-	scaffold_6:16216077-16218020	48.0431	7.08971	-2.76053
Ciclev10013799m.g	scaffold_6:14498798-14500838	3.33491	0.492414	-2.7597

Ciclev10006010m.g	scaffold_9:1547179-1548124	1.43755	0.212384	-2.75887
Ciclev10013383m.g	scaffold_6:16511762-16512380	3.42444	0.505995	-2.75867
Ciclev10003495m.g	scaffold_5:29185686-29186289	3.7005	0.547822	-2.75594
Ciclev10023664m.g	scaffold_3:1888556-1892031	10.2849	1.52289	-2.75564
Ciclev10019779m.g	scaffold_3:49342203-49346373	2.78968	0.413746	-2.75328
-	scaffold_1:28327528-28328444	3.79284	0.562916	-2.75229
Ciclev10021039m.g	scaffold_3:18826019-18831201	143.57	21.3232	-2.75125
Ciclev10022241m.g	scaffold_3:48908163-48909219	4.3859	0.652611	-2.74858
-	scaffold_6:23253457-23253859	16.9194	2.52052	-2.74688
Ciclev10013222m.g	scaffold_6:1378067-1378751	2.23014	0.33248	-2.74579
Ciclev10011917m.g	scaffold_6:18332185-18334838	1.4152	0.21106	-2.74528
Ciclev10004498m.g	scaffold_9:15953831-15959870	2.01729	0.301857	-2.74048
Ciclev10017191m.g	scaffold_2:4679375-4680117	43.1033	6.44989	-2.74045
Ciclev10003786m.g	scaffold_5:24793479-24794136	3.12729	0.470819	-2.73167
Ciclev10028254m.g	scaffold_8:20027849-20049674	123.008	18.5213	-2.73149
Ciclev10018004m.g	scaffold_2:11416284-11418258	8.69608	1.30995	-2.73085
Ciclev10025302m.g	scaffold_7:15496820-15499377	0.961773	0.145269	-2.72697
-	scaffold_8:17866978-17867263	26.5843	4.02733	-2.72268
Ciclev10033932m.g	scaffold_4:1446500-1448564	0.898724	0.136155	-2.72263
Ciclev10000891m.g	scaffold_5:12168883-12172755	13.9578	2.11959	-2.71921
-	scaffold_1:25784698-25785131	14.525	2.20726	-2.71821
Ciclev10012382m.g	scaffold_6:24923533-24935909	80.752	12.2758	-2.71769
Ciclev10002723m.g	scaffold_5:11380620-11381768	2.13976	0.32644	-2.71256
-	scaffold_5:1311648-1355130	6.57077	1.00286	-2.71195
Ciclev10005127m.g	scaffold_9:3024636-3026071	175.204	26.7942	-2.70905
Ciclev10021190m.g	scaffold_3:41286225-41287833	6.86652	1.05076	-2.70814
Ciclev10026545m.g	scaffold_7:4127517-4128342	38.5187	5.90243	-2.70618
Ciclev10011470m.g	scaffold_6:17775710-17778462	6.49632	0.995534	-2.70608
Ciclev10021994m.g	scaffold_3:47882117-47883277	21.5065	3.30005	-2.70422
Ciclev10025400m.g	scaffold_7:20360085-20395370	394.167	60.6717	-2.69971
Ciclev10016129m.g	scaffold_2:21057079-21059316	8.3474	1.28623	-2.69817
-	scaffold_7:17672247-17672895	30.7408	4.73728	-2.69803
Ciclev10000102m.g	scaffold_5:41765024-41772007	25.8448	3.99036	-2.69528
Ciclev10022791m.g	scaffold_3:39666168-39666901	2.39474	0.369832	-2.69493
Ciclev10019376m.g	scaffold_3:11399184-11403123	44.2071	6.83165	-2.69397
Ciclev10024944m.g	scaffold_7:1851369-1855115	1.32946	0.205606	-2.69288
Ciclev10000103m.g	scaffold_5:38805240-38812427	39.0786	6.04729	-2.69202
Ciclev10030984m.g	scaffold_4:18407455-18412059	42.3252	6.55344	-2.69119
Ciclev10013499m.g	scaffold_6:17507773-17511400	0.987911	0.153155	-2.68939
Ciclev10000518m.g	scaffold_5:39934354-39937311	0.457346	0.0710781	-2.68581
Ciclev10028959m.g	scaffold_8:445468-446936	53.858	8.3937	-2.68178
Ciclev10027908m.g	scaffold_8:5489111-5494845	5.30729	0.827765	-2.68068
Ciclev10033314m.g	scaffold_4:83934-86476	1.66114	0.259259	-2.67971
Ciclev10033316m.g	scaffold_4:3395409-3396460	1095.01	171.081	-2.6782
Ciclev10014770m.g	scaffold_2:35895661-35898841	83.0622	12.9875	-2.67707
Ciclev10013470m.g	scaffold_6:15940241-15990100	1455.57	227.773	-2.67592
-	scaffold_2:12544445-12544746	22.3135	3.49485	-2.67461
Ciclev10000768m.g	scaffold_5:30732823-30734566	1.30699	0.2048	-2.67397
Ciclev10016832m.g	scaffold_2:32829645-32831351	67.6917	10.6106	-2.67347
Ciclev10029524m.g	scaffold_8:16214251-16215800	2.05311	0.322208	-2.67174
Ciclev10031813m.g	scaffold_4:20702894-20707125	10.9902	1.72788	-2.66914
Ciclev10000500m.g	scaffold_5:34232841-34237423	115.387	18.1456	-2.66879
Ciclev10029124m.g	scaffold_8:2612943-2614424	8.44779	1.32893	-2.66831
Ciclev10023404m.g	scaffold_3:48463394-48465325	2.05444	0.323421	-2.66726
Ciclev10004863m.g,Ciclev10006910m.g	scaffold_9:29774047-29781905	10.1993	1.60577	-2.66714

Ciclev10003209m.g	scaffold_5:25221155-25545745	8.24809	1.29916	-2.66648
-	scaffold_5:38002256-38002457	354.106	55.8239	-2.66523
Ciclev10002724m.g	scaffold_5:7233934-7235988	6.00608	0.947166	-2.66474
Ciclev10004322m.g	scaffold_9:18502292-18505531	1.35955	0.214413	-2.66466
Ciclev10019855m.g	scaffold_3:781023-784364	2.02037	0.31936	-2.66136
-	scaffold_7:4302976-4303127	1162.83	183.988	-2.65995
Ciclev10029614m.g	scaffold_8:20292559-20294016	8.91548	1.41237	-2.6582
Ciclev10021911m.g	scaffold_3:49496617-49501009	1.80484	0.286408	-2.65573
Ciclev10004387m.g	scaffold_9:28406862-28409839	0.334814	0.053309	-2.65091
-	scaffold_2:9419344-9419655	60.687	9.67957	-2.64837
Ciclev10031451m.g	scaffold_4:24609446-24612338	5.80717	0.928069	-2.64553
Ciclev10027202m.g	scaffold_7:6124386-6126924	0.940059	0.150279	-2.64511
Ciclev10001800m.g	scaffold_5:17564728-17565994	46.546	7.45478	-2.64242
Ciclev10004742m.g	scaffold_9:27526852-27535693	1.19999	0.192199	-2.64234
-	scaffold_1:779311-812949	93.6579	15.0322	-2.63935
Ciclev10033419m.g	scaffold_4:22988925-22990379	11.599	1.86188	-2.63916
Ciclev10025741m.g	scaffold_7:149298-153895	127.297	20.4461	-2.6383
Ciclev10019042m.g	scaffold_3:36363008-36553812	299.815	48.2075	-2.63674
Ciclev10009191m.g	scaffold_1:1016170-1019117	55.0537	8.8528	-2.63663
Ciclev10011338m.g	scaffold_6:21028836-21031557	0.828533	0.133241	-2.63652
Ciclev10033850m.g	scaffold_4:3086846-3088752	2.76868	0.445763	-2.63485
Ciclev10030731m.g	scaffold_4:12328244-12330656	3.57139	0.575201	-2.63435
Ciclev10031442m.g	scaffold_4:22375081-22378853	49.4949	7.97171	-2.63432
Ciclev10004633m.g	scaffold_9:23562-25565	2.42917	0.391343	-2.63396
Ciclev10003160m.g	scaffold_5:4076819-4284428	18.8763	3.05283	-2.62835
Ciclev10009447m.g	scaffold_1:4491275-4492349	3.57554	0.57898	-2.62657
Ciclev10006825m.g	scaffold_9:7735458-7736581	1.50517	0.243755	-2.62642
Ciclev10012202m.g	scaffold_6:17971824-17973787	11.964	1.94102	-2.62381
Ciclev10017881m.g	scaffold_2:29014466-29017706	1.35686	0.22018	-2.62352
Ciclev10018350m.g	scaffold_2:11400151-11401889	0.49709	0.0807702	-2.62161
-	scaffold_5:4076819-4284428	6.54766	1.06509	-2.62
Ciclev10014502m.g	scaffold_2:10695423-10700095	55.8143	9.08485	-2.6191
Ciclev10019666m.g	scaffold_3:4975978-4977714	35.087	5.71859	-2.61721
Ciclev10030942m.g	scaffold_4:3287750-3290005	1.01924	0.166159	-2.61685
Ciclev10010508m.g	scaffold_1:22014946-22015402	578.446	94.3067	-2.61675
Ciclev10001683m.g	scaffold_5:35173757-35175204	0.902204	0.147372	-2.61399
-	scaffold_6:1729894-1730133	57.1806	9.34071	-2.61392
Ciclev10016434m.g	scaffold_2:12818316-12819910	382.198	62.4516	-2.61351
Ciclev10001160m.g	scaffold_5:8745667-8989247	1.96024	0.320308	-2.6135
Ciclev10030403m.g	scaffold_8:4408513-4409614	0.799812	0.130784	-2.61248
Ciclev10025875m.g	scaffold_7:4575502-4577125	2.80306	0.459191	-2.60983
Ciclev10003680m.g	scaffold_5:32917825-32924241	0.711073	0.116707	-2.6071
Ciclev10003189m.g	scaffold_5:3148560-3150216	1.17445	0.192887	-2.60616
Ciclev10026997m.g	scaffold_7:399184-411768	0.72746	0.119567	-2.60504
-	scaffold_2:23400953-23401118	838.437	137.838	-2.60473
Ciclev10013040m.g	scaffold_6:23325023-23326443	1.45519	0.239337	-2.60409
-	scaffold_7:97880-100644	12.3134	2.02698	-2.60283
Ciclev10022487m.g	scaffold_3:46371639-46372839	114.927	18.9515	-2.60034
Ciclev10009906m.g	scaffold_1:21464647-21466099	114.672	18.917	-2.59976
Ciclev10016123m.g	scaffold_2:11604955-11606340	2.40148	0.396388	-2.59894
Ciclev10001440m.g	scaffold_5:38719566-38721176	163.358	26.9687	-2.59868
Ciclev10005239m.g	scaffold_9:15394254-15397493	0.641618	0.106006	-2.59757
-	scaffold_9:13864894-13865084	254.995	42.1706	-2.59616
Ciclev10015527m.g	scaffold_2:35104843-35109655	16.0476	2.65463	-2.59577
Ciclev10023992m.g	scaffold_3:839295-841793	0.91966	0.152628	-2.59108
Ciclev10033091m.g	scaffold_4:7366608-7367250	17.2401	2.86273	-2.5903

Ciclev10015210m.g	scaffold_2:32155294-32158290	303.249	50.3996	-2.58902
Ciclev10004478m.g	scaffold_9:27848858-27851831	1.63053	0.271144	-2.58821
Ciclev10026224m.g	scaffold_7:11513495-11515546	26.9214	4.48592	-2.58528
Ciclev10000479m.g	scaffold_5:2479777-2485129	0.271125	0.0452067	-2.58435
-	scaffold_5:19068394-19071217	5.27163	0.879755	-2.58307
Ciclev10033564m.g	scaffold_4:25578323-25578530	410.532	68.5191	-2.58292
Ciclev10000375m.g	scaffold_5:40932836-40937518	0.312782	0.0523942	-2.57768
-	scaffold_6:10808555-10808741	240.037	40.2113	-2.57758
Ciclev10021322m.g	scaffold_3:3205519-3207758	1.29883	0.217633	-2.57725
Ciclev10001458m.g	scaffold_5:27012428-27017627	10.3386	1.73775	-2.57275
Ciclev10005737m.g	scaffold_9:1667150-1670619	76.0176	12.7781	-2.57266
Ciclev10033445m.g	scaffold_4:19724036-19724306	30.444	5.12256	-2.57122
Ciclev10011408m.g	scaffold_6:24964963-24968843	0.341958	0.0575469	-2.57101
-	scaffold_7:19950882-20044587	43.5733	7.34064	-2.56947
-	scaffold_9:16817321-16817671	14.7014	2.47875	-2.56827
-	scaffold_3:45728945-45729114	572.108	96.7658	-2.56372
-	scaffold_2:23372084-23372557	4663.81	789.288	-2.56289
Ciclev10029887m.g	scaffold_8:824979-828423	0.342463	0.057972	-2.56252
Ciclev10003914m.g	scaffold_5:34072897-34081147	176.368	29.8924	-2.56074
Ciclev10030623m.g	scaffold_4:20142444-20145612	0.710983	0.120614	-2.55942
Ciclev10017076m.g	scaffold_2:25946698-25948070	3.62498	0.616089	-2.55676
Ciclev10001979m.g	scaffold_5:39839983-39841860	102.658	17.4592	-2.55579
Ciclev10030046m.g	scaffold_8:24259555-24261377	0.575852	0.0980484	-2.55413
-	scaffold_3:21296577-21473295	25.5617	4.35662	-2.55271
Ciclev10029391m.g	scaffold_8:19132836-19211350	81.435	13.8828	-2.55235
Ciclev10000216m.g	scaffold_5:2045916-2057989	1.39558	0.238571	-2.54837
Ciclev10009080m.g	scaffold_1:2096894-2101387	26.4944	4.53026	-2.54802
Ciclev10033989m.g	scaffold_4:62661-63517	43.8331	7.50209	-2.54666
Ciclev10013873m.g	scaffold_6:21665844-21666600	3.69072	0.632216	-2.54541
Ciclev10016696m.g	scaffold_2:33746687-33749902	14.8044	2.53986	-2.5432
Ciclev10019207m.g	scaffold_3:44244253-44249010	2.67706	0.459497	-2.54252
Ciclev10026907m.g	scaffold_7:463993-470316	7.19864	1.23564	-2.54246
Ciclev10033519m.g	scaffold_4:1804770-1805424	2659.55	456.593	-2.5422
Ciclev10006506m.g	scaffold_9:27790597-27828145	666.481	114.478	-2.5415
Ciclev10014951m.g	scaffold_2:36008353-36010461	39.7731	6.83532	-2.54071
Ciclev10014808m.g	scaffold_2:29421094-29427746	0.841044	0.144742	-2.5387
Ciclev10010625m.g	scaffold_1:26437730-26441995	6.45388	1.11107	-2.53822
Ciclev10005034m.g	scaffold_9:13934629-13938104	2.47024	0.425367	-2.53787
Ciclev10011186m.g	scaffold_6:11060637-11063233	1.71925	0.296113	-2.53756
Ciclev10028033m.g	scaffold_8:2161904-2167291	2.69528	0.464432	-2.5369
-	scaffold_2:15477859-15615778	121.249	20.92	-2.53502
Ciclev10029306m.g	scaffold_8:3245556-3247054	9.72897	1.67958	-2.53419
Ciclev10023108m.g	scaffold_3:40176470-40209547	415.903	71.8904	-2.53238
Ciclev10000355m.g	scaffold_5:22304299-22309822	2.3803	0.411627	-2.53173
Ciclev10024043m.g	scaffold_3:33659325-33661615	0.614467	0.106377	-2.53015
Ciclev10014275m.g	scaffold_2:6743858-6746825	0.314991	0.0545318	-2.53014
Ciclev10008821m.g	scaffold_1:28782168-28786731	659.248	114.253	-2.52859
Ciclev10010503m.g	scaffold_1:15714853-15716692	2.49496	0.433515	-2.52487
Ciclev10006253m.g	scaffold_9:3789152-3789631	24.3863	4.24077	-2.52367
Ciclev10015097m.g	scaffold_2:4367067-4371625	11.0767	1.93415	-2.51776
-	scaffold_4:11523554-11728349	16.8547	2.94712	-2.51577
Ciclev10004870m.g	scaffold_9:28110540-28112377	2.93849	0.514082	-2.515
Ciclev10019718m.g	scaffold_3:31555090-31557262	13.0805	2.29109	-2.5133
Ciclev10028415m.g	scaffold_8:19885917-19891514	0.504006	0.0883771	-2.51169
Ciclev10001676m.g	scaffold_5:34627882-34630017	96.3091	16.8928	-2.51126
Ciclev10001561m.g	scaffold_5:42162733-42165933	2.25632	0.396075	-2.51013

Ciclev10011915m.g	scaffold_6:23372891-23374463	2.85297	0.501382	-2.50848
Ciclev10004299m.g	scaffold_9:24960880-24963735	0.360756	0.0634769	-2.50672
Ciclev10025238m.g	scaffold_7:7904864-7908319	129.245	22.7443	-2.50653
-	scaffold_8:6944685-7159404	2735.39	481.761	-2.50536
Ciclev10031082m.g	scaffold_4:1940592-1944128	6.91243	1.21799	-2.50469
Ciclev10010727m.g	scaffold_1:496309-496510	491.994	86.6921	-2.50467
Ciclev10022370m.g	scaffold_3:43862439-43863744	35.0996	6.19094	-2.50323
Ciclev10011493m.g	scaffold_6:23946605-23949478	7.23881	1.27791	-2.50196
Ciclev10025415m.g	scaffold_7:20472101-20474601	59.1799	10.4614	-2.50002
Ciclev10023434m.g	scaffold_3:24513140-24521765	0.535909	0.0947452	-2.49986
Ciclev10019448m.g	scaffold_3:41861778-41864121	12.36	2.18581	-2.49944
Ciclev10027351m.g	scaffold_7:463175-463684	3.59533	0.635921	-2.49921
Ciclev10011754m.g	scaffold_6:21271369-21273131	11.5727	2.04858	-2.49802
Ciclev10022349m.g	scaffold_3:30290830-30303977	8.54544	1.51395	-2.49684
Ciclev10026074m.g	scaffold_7:10781695-10787329	26.0701	4.61897	-2.49675
Ciclev10020212m.g	scaffold_3:32982611-32984120	5.84949	1.03772	-2.49489
Ciclev10004130m.g	scaffold_9:3954506-3961401	2.9354	0.520901	-2.49447
Ciclev10029615m.g	scaffold_8:2073735-2074304	51.9306	9.2187	-2.49395
Ciclev10005998m.g	scaffold_9:3839532-3840449	5.0895	0.905129	-2.49133
Ciclev10012390m.g	scaffold_6:24511360-24514034	17.1788	3.05855	-2.48971
Ciclev10023463m.g	scaffold_3:1974522-1986048	1.56698	0.279157	-2.48884
Ciclev10013158m.g	scaffold_6:17009448-17011100	47.11	8.40297	-2.48706
Ciclev10031440m.g	scaffold_4:16652627-16655000	3.19288	0.569669	-2.48666
Ciclev10016278m.g	scaffold_2:27156057-27158380	2.61223	0.466666	-2.48482
Ciclev10024304m.g	scaffold_3:6242973-6244997	3722.07	665.033	-2.48461
Ciclev10016328m.g	scaffold_2:8900284-8901430	5.65337	1.01186	-2.48211
Ciclev10000438m.g	scaffold_5:37407958-37411524	0.565727	0.101338	-2.48093
Ciclev10032357m.g	scaffold_4:3083089-3084520	1.94805	0.349976	-2.47671
Ciclev10031104m.g	scaffold_4:15505583-15508159	1.80469	0.325009	-2.4732
Ciclev10024153m.g	scaffold_3:44926521-44927256	1.04966	0.189266	-2.47143
-	scaffold_7:14910673-14910894	70.8859	12.7906	-2.47042
Ciclev10016232m.g	scaffold_2:15099025-15102592	4.28212	0.774405	-2.46717
Ciclev10011143m.g	scaffold_6:19569459-19577780	23.3465	4.22743	-2.46535
Ciclev10028078m.g	scaffold_8:24554321-24561746	14.9002	2.70198	-2.46324
Ciclev10031811m.g	scaffold_4:1258145-1262635	64.3392	11.6749	-2.46228
Ciclev10027873m.g	scaffold_8:6834486-6837762	614.821	111.647	-2.46123
Ciclev10032317m.g	scaffold_4:1391782-1392640	0.864408	0.156974	-2.46119
Ciclev10000543m.g	scaffold_5:35320977-35325107	0.761511	0.138444	-2.45956
-	scaffold_3:34345591-34346612	62.1859	11.3105	-2.45893
Ciclev10013952m.g	scaffold_10:50719-57047	10.1789	1.85435	-2.45659
Ciclev10002297m.g	scaffold_5:34552215-34553382	2861.96	522.12	-2.45455
Ciclev10027333m.g	scaffold_7:20837581-20839251	208.017	37.9555	-2.45432
Ciclev10000316m.g	scaffold_5:33969879-33974347	20.6275	3.76755	-2.45287
Ciclev10024474m.g	scaffold_3:25558188-25563956	5.00516	0.91434	-2.45262
Ciclev10020321m.g	scaffold_3:40848610-40913406	1.73883	0.318233	-2.44997
Ciclev10031831m.g	scaffold_4:23185702-23187916	18.5825	3.40859	-2.44669
Ciclev10031447m.g	scaffold_4:982499-984170	235.405	43.2259	-2.44517
Ciclev10022509m.g	scaffold_3:44816497-44817321	3.11535	0.572924	-2.44298
Ciclev10017111m.g	scaffold_2:30862924-30863577	2.06774	0.380502	-2.44208
Ciclev10026052m.g	scaffold_7:17497719-17499325	2.55029	0.469349	-2.44193
Ciclev10021856m.g	scaffold_3:46366594-46368992	0.867158	0.159654	-2.44135
Ciclev10019413m.g	scaffold_3:9998252-10001532	1.9061	0.350964	-2.44123
Ciclev10007736m.g	scaffold_1:14601222-14605899	2.39102	0.440291	-2.4411
Ciclev10009640m.g	scaffold_1:27818757-27819890	20.3531	3.75129	-2.43979
Ciclev10021479m.g	scaffold_3:46784682-46785917	0.727592	0.134241	-2.4383
Ciclev10012453m.g	scaffold_6:23135121-23137028	5.1009	0.942012	-2.43693

Ciclev10015614m.g	scaffold_2:5108673-5112244	15.1263	2.79426	-2.43652
Ciclev10011256m.g	scaffold_6:14391607-14396035	3.56173	0.658578	-2.43515
-	scaffold_5:34022861-34023361	19.9587	3.69059	-2.43509
Ciclev10011555m.g	scaffold_6:5754834-5780076	33.9218	6.27286	-2.43502
Ciclev10031089m.g	scaffold_4:19471732-19475589	10.6588	1.97124	-2.43487
Ciclev10028674m.g	scaffold_8:16674700-16678943	14.9381	2.76333	-2.43452
Ciclev10025446m.g	scaffold_7:2117362-2121600	21.4359	3.97746	-2.43011
Ciclev10010491m.g	scaffold_1:28782168-28786731	62.4634	11.5971	-2.42925
Ciclev10029600m.g	scaffold_8:1213736-1214457	6.90625	1.28364	-2.42766
Ciclev10013325m.g	scaffold_6:25526519-25528600	2.96534	0.552667	-2.42372
Ciclev10013204m.g	scaffold_6:23790000-23794926	1.93501	0.360973	-2.42238
Ciclev10020453m.g	scaffold_3:46920513-46923179	71.283	13.3399	-2.41781
-	scaffold_5:35288218-35288584	12.0896	2.26249	-2.41778
Ciclev10005597m.g	scaffold_9:12562561-12570023	24.4208	4.57087	-2.41757
Ciclev10004781m.g	scaffold_9:26790441-26802517	1.00369	0.188508	-2.41262
Ciclev10000269m.g	scaffold_5:42321790-42327438	5.04101	0.947763	-2.41111
Ciclev10033031m.g	scaffold_4:2350769-2351460	2.0574	0.387443	-2.40877
Ciclev10031467m.g	scaffold_4:3297088-3298858	13.5906	2.56104	-2.40781
Ciclev10008213m.g	scaffold_1:25959549-25962463	1.2036	0.226868	-2.40743
Ciclev10010955m.g	scaffold_6:25511427-25520662	3.76218	0.709666	-2.40636
-	scaffold_1:15130502-15130782	44.6978	8.4546	-2.4024
Ciclev10016265m.g	scaffold_2:27247683-27249818	2.60671	0.493465	-2.40121
Ciclev10021984m.g	scaffold_3:2265849-2267575	4.55467	0.863979	-2.39828
Ciclev10009334m.g	scaffold_1:19049995-19054408	190.108	36.1104	-2.39634
-	scaffold_5:25883693-25884613	5.89261	1.11971	-2.39578
Ciclev10033846m.g	scaffold_4:23651506-23652719	4.16772	0.792613	-2.39457
Ciclev10033818m.g	scaffold_4:24544911-24551825	3.59939	0.685	-2.39358
Ciclev10028945m.g	scaffold_8:21325199-21327234	2.22225	0.42405	-2.38971
Ciclev10028667m.g	scaffold_8:22568165-22571151	188.867	36.054	-2.38914
Ciclev10022787m.g	scaffold_3:19834359-19911599	947.015	180.867	-2.38846
Ciclev10014435m.g	scaffold_2:34258298-34262758	1.105	0.211095	-2.38808
-	scaffold_8:18421720-18422064	8.95841	1.71383	-2.38602
Ciclev10019502m.g	scaffold_3:42928892-42932215	19.3907	3.71066	-2.38562
Ciclev10021961m.g	scaffold_3:50277272-50278603	1.14469	0.219202	-2.38462
Ciclev10003463m.g	scaffold_5:17572998-17573997	18.943	3.62804	-2.3844
Ciclev10002388m.g	scaffold_5:41350378-41351840	3.69845	0.708428	-2.38423
-	scaffold_1:15471373-15471595	81.8244	15.6776	-2.38383
Ciclev10033100m.g	scaffold_4:2038851-2039526	3.4397	0.659069	-2.38378
Ciclev10004884m.g	scaffold_9:22730945-22732373	7.26754	1.39313	-2.38313
Ciclev10021747m.g	scaffold_3:4644459-4648658	3.04329	0.584034	-2.38151
Ciclev10032318m.g	scaffold_4:3443339-3447763	1.15199	0.221098	-2.38136
Ciclev10014635m.g	scaffold_2:27964005-27967181	1.22252	0.234741	-2.38072
-	scaffold_1:5170837-5171150	16.5454	3.17762	-2.38041
Ciclev10018942m.g	scaffold_3:229425-233034	14.9376	2.87021	-2.37972
Ciclev10007916m.g	scaffold_1:4989025-4994456	39.1786	7.53617	-2.37816
Ciclev10029566m.g	scaffold_8:17291958-17467945	3.83254	0.737815	-2.37697
Ciclev10008653m.g	scaffold_1:24619125-24621953	789.494	152.266	-2.37433
Ciclev10015726m.g	scaffold_2:36177995-36179730	5.79318	1.11852	-2.37276
Ciclev10030107m.g	scaffold_8:21050066-21051732	1.64208	0.317334	-2.37145
Ciclev10001451m.g	scaffold_5:39905513-39907734	3.66995	0.709274	-2.37135
Ciclev10004752m.g	scaffold_9:3377303-3379417	0.492264	0.0951817	-2.37068
Ciclev10005288m.g	scaffold_9:28641385-28643731	62.9405	12.1701	-2.37064
Ciclev10017459m.g	scaffold_2:8107990-8109028	0.714219	0.138107	-2.37058
Ciclev10018396m.g	scaffold_2:8798483-8799026	13.4327	2.60143	-2.36838
Ciclev10032402m.g	scaffold_4:3875677-3879087	3.19755	0.619297	-2.36826
Ciclev10029563m.g	scaffold_8:2264875-2265853	1.87346	0.363052	-2.36746

-	scaffold_9:8720527-8720831	23.4524	4.54653	-2.3669
Ciclev10002743m.g	scaffold_5:36634134-36636064	274.922	53.3006	-2.3668
Ciclev10022194m.g	scaffold_3:49560909-49561959	1.05168	0.203907	-2.36671
Ciclev10011279m.g	scaffold_6:15404433-15407830	3.42246	0.663584	-2.36668
Ciclev10024181m.g	scaffold_3:39401956-39402901	0.737582	0.143019	-2.36659
Ciclev10004233m.g	scaffold_9:28257932-28261337	0.23569	0.0457102	-2.3663
Ciclev10023959m.g	scaffold_3:42678069-42680841	10.1672	1.97216	-2.36608
-	scaffold_5:38002044-38002187	2675.63	519.154	-2.36565
Ciclev10007083m.g	scaffold_9:1118490-1119771	4.84132	0.940778	-2.36347
Ciclev10018818m.g	scaffold_3:5428605-5435876	149.273	29.008	-2.36343
-	scaffold_18:19604-34335	21.3881	4.1594	-2.36236
Ciclev10024881m.g	scaffold_7:1613689-1620021	12.6046	2.45244	-2.36166
Ciclev10013499m.g	scaffold_6:17507773-17511400	0.913662	0.178368	-2.3568
-	scaffold_2:14028994-14029238	188.527	36.835	-2.35562
Ciclev10010616m.g	scaffold_1:7860507-7863374	0.77655	0.151733	-2.35554
Ciclev10001948m.g	scaffold_5:42001448-42003265	2.39061	0.467409	-2.35462
Ciclev10022349m.g	scaffold_3:30290830-30303977	5.11419	1.00028	-2.3541
Ciclev10024604m.g	scaffold_3:24026647-24169357	0.696783	0.136449	-2.35235
Ciclev10018256m.g	scaffold_2:32445818-32446501	3.66441	0.717885	-2.35176
Ciclev10023449m.g	scaffold_3:3217382-3219499	8.58677	1.6825	-2.35151
Ciclev10016395m.g	scaffold_2:31727877-31729096	0.743073	0.1456	-2.35149
Ciclev10021785m.g	scaffold_3:2278674-2280175	23.8327	4.67146	-2.351
Ciclev10011513m.g	scaffold_6:15348625-15351249	0.536137	0.105102	-2.35081
Ciclev10005200m.g	scaffold_9:30442074-30445262	32.4168	6.35554	-2.35066
Ciclev10004689m.g	scaffold_9:2230933-2233792	368.669	72.3156	-2.34995
Ciclev10025771m.g	scaffold_7:11369605-11373020	1.5732	0.308674	-2.34955
Ciclev10017907m.g	scaffold_2:34421170-34422323	23.1687	4.5476	-2.349
Ciclev10013548m.g	scaffold_6:20808719-20811891	0.312803	0.0614444	-2.3479
Ciclev10018044m.g	scaffold_2:367178-368057	0.968808	0.190379	-2.34734
Ciclev10019672m.g	scaffold_3:47697603-47699546	99.255	19.5047	-2.34732
Ciclev10028264m.g	scaffold_8:4292179-4375670	2.51537	0.495106	-2.34497
-	scaffold_3:25025458-25026632	10.1315	1.99501	-2.34438
Ciclev10025189m.g	scaffold_7:8763666-8766821	8.65223	1.70633	-2.34217
Ciclev10025834m.g	scaffold_7:70854-73880	197.131	38.8869	-2.3418
Ciclev10026847m.g	scaffold_7:13348925-13360491	48.466	9.57103	-2.34023
Ciclev10005947m.g	scaffold_9:3014692-3016211	1.42	0.280463	-2.34001
Ciclev10011056m.g	scaffold_6:21726439-21728989	4.30975	0.851406	-2.33968
Ciclev10008549m.g	scaffold_1:4379880-4381807	8.11499	1.60555	-2.33752
Ciclev10016386m.g	scaffold_2:11388043-11389521	11.8836	2.35131	-2.33743
Ciclev10001513m.g	scaffold_5:36526058-36528589	16.0078	3.16834	-2.33698
Ciclev10026801m.g	scaffold_7:14281893-14282247	5.58519	1.10575	-2.33658
-	scaffold_5:32831038-32831533	8.92649	1.76735	-2.33651
Ciclev10028355m.g	scaffold_8:3095235-3098108	4.09495	0.811369	-2.33542
Ciclev10024477m.g	scaffold_3:13964885-13966801	10.3454	2.05021	-2.33514
Ciclev10005805m.g	scaffold_9:7646164-7647453	0.530157	0.105124	-2.33432
Ciclev10029053m.g	scaffold_8:4712392-4713689	0.926297	0.183684	-2.33425
Ciclev10019211m.g	scaffold_3:1943646-1947641	6.33306	1.25655	-2.33344
-	scaffold_2:5955259-5955435	415.425	82.5167	-2.33183
Ciclev10025287m.g	scaffold_7:1874572-1876984	5.70781	1.13415	-2.33132
Ciclev10004664m.g	scaffold_9:4261825-4263993	0.317459	0.0630876	-2.33114
Ciclev10003255m.g	scaffold_5:39429190-39431053	0.739377	0.147046	-2.33004
Ciclev10008737m.g	scaffold_1:4208204-4209341	0.810022	0.161182	-2.32927
-	scaffold_5:21373464-21374121	11.6687	2.32354	-2.32825
Ciclev10004978m.g	scaffold_9:4956545-4959033	0.294645	0.0586818	-2.32799
Ciclev10023135m.g	scaffold_3:12271368-12272961	42.4611	8.4591	-2.32757
Ciclev10028965m.g	scaffold_8:18824261-18825514	1461.81	291.311	-2.32713

Ciclev10010000m.g	scaffold_1:27397739-27398620	63.8781	12.7586	-2.32385
Ciclev10024111m.g	scaffold_3:48311543-48313181	25.809	5.16881	-2.31997
Ciclev10028738m.g	scaffold_8:23681244-23682676	147.759	29.5996	-2.3196
Ciclev10026552m.g	scaffold_7:1381760-1382875	76.5013	15.3287	-2.31925
Ciclev10025791m.g	scaffold_7:2938058-2940407	0.945367	0.189573	-2.31812
Ciclev10020992m.g	scaffold_3:23142516-23146296	0.567461	0.113963	-2.31595
Ciclev10025640m.g	scaffold_7:4077657-4079648	0.540598	0.108597	-2.31558
Ciclev10006396m.g	scaffold_9:30590131-30590466	10.5764	2.12875	-2.31277
Ciclev10011857m.g	scaffold_6:19119417-19123027	17.0164	3.4265	-2.31211
Ciclev10008384m.g,Ciclev10008398m.g,Ciclev10010634m.g	scaffold_1:2237003-2346942	4.54586	0.917056	-2.30947
Ciclev10032182m.g	scaffold_4:24623710-24627543	30.4897	6.15304	-2.30895
-	scaffold_1:20351610-20354349	246.065	49.6665	-2.3087
-	scaffold_7:9198398-9198641	32.6724	6.59588	-2.30843
Ciclev10016492m.g	scaffold_2:31250925-31254073	52.9754	10.7037	-2.30721
Ciclev10031030m.g	scaffold_4:349655-353012	0.329033	0.0664919	-2.30698
-	scaffold_2:14030632-14030981	12.3392	2.49363	-2.30692
-	scaffold_5:28539341-28801354	6.20059	1.25452	-2.30527
Ciclev10006981m.g	scaffold_9:7804322-8050719	1.46724	0.297046	-2.30435
Ciclev10020328m.g	scaffold_3:49621664-49623875	2.00527	0.406856	-2.3012
Ciclev10011710m.g	scaffold_6:19531984-19534809	0.390346	0.0792954	-2.29944
Ciclev10027853m.g	scaffold_8:2634214-2638475	2.06921	0.420589	-2.2986
Ciclev10021285m.g	scaffold_3:5253280-5254755	523.383	106.462	-2.29752
Ciclev10019584m.g	scaffold_3:50724060-50729319	1.32442	0.269562	-2.29667
Ciclev10017118m.g	scaffold_2:10056013-10056639	17.0877	3.47807	-2.2966
Ciclev10021399m.g	scaffold_3:2402756-2403773	3.18927	0.649179	-2.29654
Ciclev10000599m.g	scaffold_5:29840773-29846742	8.98905	1.83138	-2.29524
Ciclev10032927m.g	scaffold_4:12434969-12439835	4.01931	0.819678	-2.29382
Ciclev10013922m.g	scaffold_2256:13-1735	0.457803	0.0933737	-2.29364
Ciclev10030291m.g	scaffold_8:19132836-19211350	19.969	4.07716	-2.29212
-	scaffold_9:18607614-18607905	55.8516	11.4308	-2.28868
Ciclev10032774m.g	scaffold_4:1797536-1798886	1.93259	0.396127	-2.2865
Ciclev10026590m.g	scaffold_7:1278697-1279888	1.25051	0.256364	-2.28625
Ciclev10008645m.g	scaffold_1:26820626-26825048	6.78261	1.39134	-2.28536
Ciclev10006490m.g	scaffold_9:22368666-22373982	0.95466	0.195862	-2.28515
Ciclev10012533m.g	scaffold_6:18228712-18229800	10.2247	2.0985	-2.28462
-	scaffold_3:9796583-9797058	207.037	42.4986	-2.2844
Ciclev10031460m.g	scaffold_4:20332918-20335419	0.448514	0.0920907	-2.28403
-	scaffold_3:47793389-47794403	6.44567	1.32381	-2.28363
Ciclev10011117m.g	scaffold_6:15425227-15429724	10.4799	2.15344	-2.28291
Ciclev10030540m.g	scaffold_4:17121877-17126289	26.6128	5.48597	-2.2783
Ciclev10024962m.g	scaffold_7:3650108-3653452	0.277149	0.0572786	-2.27459
Ciclev10028131m.g	scaffold_8:535358-537471	0.438083	0.0906181	-2.27333
Ciclev10014347m.g	scaffold_2:34661891-34669768	19.0938	3.94983	-2.27324
Ciclev10001192m.g	scaffold_5:40417197-40420771	18.4659	3.82104	-2.27283
Ciclev10001677m.g	scaffold_5:35974953-35979998	490.782	101.588	-2.27235
Ciclev10026702m.g	scaffold_7:5344867-5345946	46.3106	9.58773	-2.27208
Ciclev10015422m.g	scaffold_2:4840138-4844193	3.90065	0.808232	-2.27087
Ciclev10020881m.g	scaffold_3:855006-857471	4.82311	1.00117	-2.26829
Ciclev10006246m.g	scaffold_9:29549721-29554186	2.90067	0.602276	-2.26789
Ciclev10029402m.g	scaffold_8:20200487-20201896	9.17519	1.90591	-2.26726
Ciclev10033609m.g	scaffold_4:18515567-18675155	67.7843	14.0813	-2.26717
Ciclev10020563m.g	scaffold_3:46248144-46249558	3.73089	0.777321	-2.26294
Ciclev10000083m.g	scaffold_5:41604995-41608538	3.25467	0.678824	-2.2614
Ciclev10016454m.g	scaffold_2:8151969-8154993	37.4477	7.81389	-2.26076

Ciclev10006904m.g	scaffold_9:25367088-25368146	372.25	77.6764	-2.26073
Ciclev10014842m.g	scaffold_2:22362547-22366644	4.31606	0.900738	-2.26054
-	scaffold_9:11576287-11715575	4.39826	0.918815	-2.25909
Ciclev10004929m.g	scaffold_9:21034568-21037653	1.03628	0.216507	-2.25893
-	scaffold_5:28289811-28536377	28.0471	5.86501	-2.25765
Ciclev10018778m.g	scaffold_3:41991277-41994183	9.47369	1.98346	-2.25591
-	scaffold_5:27309049-27309682	4.37906	0.917931	-2.25416
Ciclev10028034m.g	scaffold_8:2175780-2183619	1.26955	0.266209	-2.25369
Ciclev10011663m.g	scaffold_6:22739630-22741028	1.29898	0.272407	-2.25355
-	scaffold_5:321675-322202	13.3473	2.80421	-2.25088
Ciclev10013119m.g	scaffold_6:17541449-17543009	3850.98	809.481	-2.25016
Ciclev10009975m.g	scaffold_1:22384421-22385632	1045.33	219.758	-2.24997
Ciclev10028229m.g	scaffold_8:19389328-19394447	12.0063	2.52412	-2.24994
Ciclev10023758m.g	scaffold_3:50780245-50780941	60.5999	12.7437	-2.24953
Ciclev10023669m.g	scaffold_3:27210507-27242516	6.26093	1.31701	-2.24911
Ciclev10025504m.g	scaffold_7:5659471-5662345	0.773741	0.162811	-2.24865
Ciclev10014060m.g	scaffold_2:29155997-29160335	0.162724	0.0343642	-2.24345
Ciclev10023118m.g	scaffold_3:9744902-9745821	9.21725	1.9487	-2.24183
Ciclev10002066m.g	scaffold_5:11336688-11353774	2.72445	0.576287	-2.2411
Ciclev10004717m.g	scaffold_9:27765514-27767561	0.371769	0.0787126	-2.23974
Ciclev10003146m.g	scaffold_5:22611509-22623399	0.479491	0.101655	-2.23782
-	scaffold_4:9880494-10159290	90.632	19.2217	-2.23728
Ciclev10027140m.g	scaffold_7:3583793-3584499	2.8375	0.6023	-2.23607
Ciclev10011022m.g	scaffold_6:12863237-12867758	1.35683	0.288329	-2.23445
Ciclev10030313m.g	scaffold_8:2770499-2771191	2.44683	0.520498	-2.23295
Ciclev10020261m.g	scaffold_3:49428800-49433203	4.99916	1.06474	-2.23119
Ciclev10000262m.g	scaffold_5:27087973-27094928	8.91823	1.90423	-2.22755
Ciclev10011523m.g	scaffold_6:24394559-24396615	1.47883	0.315837	-2.2272
Ciclev10022253m.g	scaffold_3:446846-449296	65.6967	14.0358	-2.22671
Ciclev10020052m.g	scaffold_3:39444357-39446022	5.34842	1.14298	-2.22631
Ciclev10003129m.g	scaffold_5:19032658-19035265	1.42831	0.30533	-2.22587
Ciclev10015869m.g	scaffold_2:10298470-10299566	0.896208	0.191802	-2.22422
-	scaffold_1:28303017-28323314	105.026	22.4833	-2.22382
Ciclev10024321m.g	scaffold_3:40089417-40091626	0.575724	0.123271	-2.22355
Ciclev10027448m.g	scaffold_7:4861024-4880959	3.65078	0.781695	-2.22353
Ciclev10031210m.g	scaffold_4:24763311-24766319	0.414675	0.0888646	-2.2223
Ciclev10027191m.g	scaffold_7:175846-180673	47.7276	10.2332	-2.22156
Ciclev10031870m.g	scaffold_4:23135569-23137302	35.8971	7.69767	-2.22137
-	scaffold_5:25666639-25666926	36.7951	7.90473	-2.21873
-	scaffold_9:27105978-27106517	855.382	183.85	-2.21804
Ciclev10025127m.g	scaffold_7:8320463-8323153	0.3406	0.0732254	-2.21766
Ciclev10015826m.g	scaffold_2:28714787-28717185	6.95633	1.49631	-2.21692
Ciclev10002237m.g	scaffold_5:40544379-40545372	1.48936	0.320544	-2.2161
Ciclev10025217m.g	scaffold_7:5884672-5887407	1.74171	0.374874	-2.21603
Ciclev10019393m.g	scaffold_3:45594729-45597938	0.324436	0.0698844	-2.21489
Ciclev10022307m.g	scaffold_3:4931883-4932873	144.879	31.2174	-2.21442
Ciclev10032525m.g	scaffold_4:17822401-17824104	72.0046	15.5153	-2.2144
Ciclev10015253m.g	scaffold_2:26750606-26752196	5.08991	1.09677	-2.21438
Ciclev10018030m.g	scaffold_2:14229496-14232187	4.94132	1.06481	-2.2143
Ciclev10027874m.g	scaffold_8:24600340-24604527	0.346407	0.0746609	-2.21404
Ciclev10033345m.g	scaffold_4:24244928-24247855	3.45311	0.744694	-2.21318
Ciclev10006841m.g	scaffold_9:29555915-29562123	0.399208	0.086146	-2.21229
-	scaffold_4:25282092-25283369	5.38823	1.16293	-2.21205
Ciclev10008603m.g	scaffold_1:28506127-28510309	11.0977	2.39884	-2.20986
Ciclev10026618m.g	scaffold_7:16616365-16618215	272.216	58.8532	-2.20956
Ciclev10031212m.g	scaffold_4:25285785-25288711	11.2943	2.44347	-2.2086

Ciclev10013960m.g	scaffold_108:21981-25744	0.253988	0.0549527	-2.2085
Ciclev10026660m.g	scaffold_7:11216727-11218178	74.2869	16.081	-2.20775
Ciclev10018687m.g	scaffold_3:33388539-33592383	0.286165	0.0619745	-2.2071
Ciclev10024374m.g	scaffold_3:7716702-7718193	7.05751	1.52991	-2.20572
Ciclev10031834m.g	scaffold_4:12571525-12575185	4.37996	0.950103	-2.20476
Ciclev10031033m.g	scaffold_4:22480681-22484154	8.37425	1.82101	-2.20123
Ciclev10019265m.g	scaffold_3:50861812-50866146	77.1328	16.7787	-2.20072
Ciclev10030750m.g	scaffold_4:23173822-23176273	0.846537	0.18463	-2.19693
Ciclev10011479m.g	scaffold_6:24177353-24178998	0.453061	0.0988465	-2.19644
Ciclev10017198m.g	scaffold_2:35270273-35271375	465.522	101.566	-2.19643
Ciclev10009946m.g	scaffold_1:7502538-7503470	3.66616	0.80032	-2.19562
-	scaffold_5:9000-9187	284.529	62.1509	-2.19473
-	scaffold_4:15690355-15782388	5.54142	1.2118	-2.19311
Ciclev10010243m.g	scaffold_1:4272010-4274509	1.07761	0.235769	-2.19239
-	scaffold_33:47710-47871	1670.79	366.671	-2.18797
Ciclev10023800m.g	scaffold_3:42757707-42759180	1.54508	0.339369	-2.18676
Ciclev10020438m.g	scaffold_3:41880758-41884653	8.50459	1.87316	-2.18277
Ciclev10008459m.g	scaffold_1:552219-553956	2.7575	0.607791	-2.18171
-	scaffold_4:2076973-2077442	6.56146	1.44644	-2.18151
Ciclev10028185m.g	scaffold_8:24583238-24585924	39.8448	8.78669	-2.181
Ciclev10003218m.g	scaffold_5:31873014-31876484	3.23549	0.714628	-2.17872
Ciclev10014403m.g	scaffold_2:9161512-9164490	4.99414	1.10307	-2.17871
Ciclev10011176m.g	scaffold_6:14811690-14821352	258.691	57.2075	-2.17695
Ciclev10026559m.g	scaffold_7:13022420-13028396	3.896	0.862339	-2.17567
Ciclev10030600m.g	scaffold_4:25389336-25392417	0.605169	0.134041	-2.17466
Ciclev10025289m.g	scaffold_7:7964710-7967426	32.0484	7.10564	-2.17322
Ciclev10012280m.g	scaffold_6:22433771-22434901	9.20071	2.04156	-2.17207
Ciclev10028851m.g	scaffold_8:14789923-14791809	2.61402	0.581449	-2.16855
Ciclev10027913m.g	scaffold_8:21712356-21744083	0.91153	0.202774	-2.16842
Ciclev10032875m.g	scaffold_4:25167373-25170468	8.17765	1.8197	-2.16798
Ciclev10017826m.g	scaffold_2:21754131-21754443	13.0135	2.89688	-2.16744
Ciclev10018822m.g	scaffold_3:46648299-46651560	2.74983	0.61214	-2.16741
Ciclev10014327m.g	scaffold_2:34986880-34989761	0.882972	0.196606	-2.16706
Ciclev10008808m.g	scaffold_1:3793875-3798312	37.0549	8.26139	-2.16521
Ciclev10022592m.g	scaffold_3:910842-912358	2.36165	0.526756	-2.16459
Ciclev10001697m.g	scaffold_5:33068049-33069093	0.874083	0.195331	-2.16185
-	scaffold_7:6191477-6191984	5.70577	1.27625	-2.16051
Ciclev10001782m.g	scaffold_5:4979770-4981645	10.7561	2.41197	-2.15688
Ciclev10011694m.g	scaffold_6:21325527-21328209	132.435	29.6997	-2.15676
Ciclev10024015m.g	scaffold_3:6326456-6328290	8.98405	2.01716	-2.15504
Ciclev10020140m.g	scaffold_3:48348137-48351697	7.29015	1.63699	-2.1549
Ciclev10005547m.g	scaffold_9:6873517-6875806	5.51269	1.23883	-2.15377
Ciclev10005666m.g	scaffold_9:27698502-27699483	9.60314	2.15941	-2.15287
Ciclev10028756m.g	scaffold_8:210020-211568	7.47563	1.68202	-2.152
Ciclev10019810m.g	scaffold_3:23665051-23668159	71.4522	16.0873	-2.15106
-	scaffold_5:17033873-17034729	5.44511	1.22639	-2.15054
Ciclev10004053m.g	scaffold_5:18107913-18109293	3.44127	0.77512	-2.15045
-	scaffold_6:20733565-20734353	151.216	34.1092	-2.14837
Ciclev10014356m.g	scaffold_2:866585-876264	0.540317	0.122001	-2.14691
Ciclev10008355m.g	scaffold_1:27914229-27916509	176.775	39.9659	-2.14507
Ciclev10000760m.g	scaffold_5:42409836-42412693	1.12567	0.254497	-2.14507
Ciclev10027280m.g,Ciclev10027539m.g	scaffold_7:352102-357146	33.4503	7.56652	-2.14432
Ciclev10008287m.g	scaffold_1:7848670-7851354	3.95915	0.897123	-2.14181
Ciclev10020385m.g	scaffold_3:47202108-47206941	26.2367	5.9494	-2.14077
Ciclev10020990m.g	scaffold_3:23182642-23185114	14.3419	3.25946	-2.13753

Ciclev10008046m.g	scaffold_1:24735244-24739117	0.783377	0.178141	-2.13669
Ciclev10022978m.g	scaffold_3:47918836-47919305	3.22596	0.734324	-2.13524
Ciclev10026272m.g	scaffold_7:6629035-6630243	101.649	23.1696	-2.1333
Ciclev10025671m.g,Ciclev10027010m.g	scaffold_7:17948447-18051030	110.058	25.0934	-2.13288
Ciclev10025414m.g	scaffold_7:3302375-3305817	3.04912	0.695359	-2.13257
-	scaffold_1:20148919-20152456	16.4368	3.75547	-2.12986
-	scaffold_2:633363-635529	34.993	8.00031	-2.12894
Ciclev10015568m.g	scaffold_2:12832947-12834878	131.343	30.0486	-2.12797
Ciclev10019690m.g	scaffold_3:16337473-16341706	1.16593	0.267049	-2.1263
Ciclev10022285m.g	scaffold_3:6107860-6109237	3.41815	0.784247	-2.12384
Ciclev10018292m.g	scaffold_2:34936990-34937714	20.6281	4.73371	-2.12357
Ciclev10022260m.g	scaffold_3:6125037-6126933	56.3281	12.9451	-2.12145
-	scaffold_5:27314577-27320454	17.9602	4.12945	-2.12078
Ciclev10022908m.g	scaffold_3:47351428-47352153	16.6583	3.83534	-2.11882
Ciclev10010881m.g	scaffold_483:9442-11246	75.2803	17.337	-2.11842
Ciclev10019693m.g	scaffold_3:6158862-6161953	19.2373	4.43068	-2.11831
Ciclev10028675m.g	scaffold_8:2710628-2712629	0.946062	0.218059	-2.11722
Ciclev10019887m.g	scaffold_3:27378258-27383803	24.937	5.75267	-2.11598
Ciclev10011765m.g	scaffold_6:24945954-24947983	112.633	25.9973	-2.11519
-	scaffold_5:655534-811195	3.48452	0.804854	-2.11416
Ciclev10031496m.g	scaffold_4:16426858-16429926	43.5978	10.0778	-2.11308
Ciclev10029531m.g	scaffold_8:11297753-11299510	9.34579	2.16035	-2.11305
Ciclev10031520m.g	scaffold_4:5052163-5053516	1.01047	0.233764	-2.11191
Ciclev10032765m.g	scaffold_4:666956-667765	12.1486	2.81306	-2.11058
Ciclev10011555m.g,Ciclev10011889m.g	scaffold_6:5754834-5780076	97.9619	22.6985	-2.10963
Ciclev10024134m.g	scaffold_3:1221448-1222315	1.96944	0.456634	-2.10868
-	scaffold_3:29952421-29952732	37.0865	8.60406	-2.10781
Ciclev10029697m.g	scaffold_8:14795152-14795671	25.6145	5.94638	-2.10688
Ciclev10033553m.g	scaffold_4:14957124-14958803	5.93827	1.38004	-2.10534
Ciclev10009509m.g	scaffold_1:21476759-21547573	4.68959	1.0925	-2.10183
Ciclev10013889m.g	scaffold_6:23800860-23803332	1.07194	0.249785	-2.10146
Ciclev10015853m.g	scaffold_2:34718353-34720151	1.63779	0.3817	-2.10124
Ciclev10025726m.g	scaffold_7:13916806-14069972	5.18636	1.20909	-2.1008
Ciclev10005494m.g	scaffold_9:23071000-23074750	12.9983	3.03524	-2.09844
Ciclev10020141m.g	scaffold_3:48560244-48562027	178.89	41.8025	-2.09741
Ciclev10001618m.g	scaffold_5:42228571-42230813	30.6644	7.16884	-2.09675
Ciclev10001139m.g	scaffold_5:39517974-39523266	9.30118	2.17699	-2.09508
Ciclev10013434m.g	scaffold_6:9032386-9034306	2.05131	0.480122	-2.09507
Ciclev10032350m.g	scaffold_4:22656180-22657410	18.5011	4.34735	-2.0894
Ciclev10003669m.g	scaffold_5:28289811-28536377	60.8852	14.3081	-2.08926
Ciclev10033969m.g	scaffold_4:24314605-24316379	2.78224	0.654538	-2.0877
-	scaffold_5:41362348-41363613	2.92153	0.687798	-2.08667
Ciclev10018577m.g	scaffold_3:1906737-1912326	2.17667	0.513211	-2.0845
Ciclev10012594m.g	scaffold_6:23870518-23876303	1.58981	0.375044	-2.08372
Ciclev10014733m.g	scaffold_2:8436109-8440408	57.4918	13.564	-2.08357
Ciclev10025326m.g	scaffold_7:2839617-2843824	9.69825	2.28837	-2.0834
Ciclev10011097m.g	scaffold_6:15037832-15040247	2.28744	0.541245	-2.07938
Ciclev10028710m.g	scaffold_8:23163228-23167352	51.6933	12.2317	-2.07935
Ciclev10022453m.g	scaffold_3:39092284-39094629	320.086	75.7712	-2.07874
Ciclev10009800m.g	scaffold_1:25204978-25205573	6.10526	1.44591	-2.07807
Ciclev10005242m.g	scaffold_9:26642583-26645312	2.13081	0.504834	-2.07752
Ciclev10000991m.g	scaffold_5:37687806-37690745	104.557	24.8261	-2.07437
Ciclev10030291m.g	scaffold_8:19132836-19211350	7.56243	1.79581	-2.07422
Ciclev10019361m.g	scaffold_3:48116570-48124848	0.679176	0.16133	-2.07377

Ciclev10017989m.g	scaffold_2:32091340-32093402	0.964727	0.229611	-2.07093
Ciclev10001332m.g	scaffold_5:40427855-40429578	88.6916	21.1092	-2.07093
Ciclev10011561m.g	scaffold_6:25339560-25341045	0.773251	0.184133	-2.07019
Ciclev10010593m.g	scaffold_1:23346721-23353317	0.629913	0.150036	-2.06984
Ciclev10031793m.g	scaffold_4:19605472-19608059	43.9335	10.4667	-2.06951
Ciclev10024795m.g	scaffold_7:4288204-4292989	47.3267	11.2831	-2.06848
Ciclev10029158m.g	scaffold_8:2874894-2876912	75.5389	18.017	-2.06786
Ciclev10031553m.g	scaffold_4:19133591-19138090	33.1075	7.90146	-2.06697
Ciclev10007213m.g	scaffold_9:3172304-3172987	7.99828	1.91075	-2.06555
Ciclev10032833m.g	scaffold_4:12732810-12733635	2.80428	0.670937	-2.06338
Ciclev10032860m.g	scaffold_4:3098508-3099415	3.89247	0.931919	-2.06241
-	scaffold_4:7295169-7301155	192.37	46.0723	-2.06191
Ciclev10026072m.g	scaffold_7:5769059-5771090	1.90832	0.457066	-2.06183
Ciclev10025408m.g	scaffold_7:15707974-15709919	21.0957	5.05377	-2.06151
Ciclev10033412m.g	scaffold_4:18329919-18330983	48.5566	11.6505	-2.05928
Ciclev10021067m.g	scaffold_3:5360112-5362918	333.724	80.0731	-2.05926
Ciclev10013710m.g	scaffold_6:1507856-1509072	88.5165	21.2445	-2.05886
Ciclev10026210m.g	scaffold_7:4531275-4532990	647.181	155.638	-2.05598
Ciclev10026829m.g	scaffold_7:2625274-2625987	4.23631	1.01925	-2.0553
Ciclev10029937m.g	scaffold_8:19315361-19315979	4.66061	1.12158	-2.05499
Ciclev10027222m.g	scaffold_7:17182106-17182739	1.34628	0.324127	-2.05435
Ciclev10030779m.g	scaffold_4:3209099-3212422	2.45393	0.590856	-2.05421
Ciclev10001563m.g	scaffold_5:36550506-36552800	612.859	147.669	-2.05318
Ciclev10027163m.g	scaffold_7:15449276-15449510	69.4055	16.7327	-2.05238
Ciclev10028206m.g	scaffold_8:338660-340991	4.1842	1.00896	-2.05208
Ciclev10005975m.g	scaffold_9:5562555-5563170	2.93291	0.70826	-2.04998
Ciclev10011937m.g	scaffold_6:24093844-24097113	3.03402	0.732854	-2.04963
Ciclev10028426m.g	scaffold_8:1698324-1700656	55.6116	13.4457	-2.04824
Ciclev10018237m.g	scaffold_2:7223552-7226407	1.07178	0.259141	-2.04819
-	scaffold_5:30358220-30358392	463.848	112.228	-2.04722
Ciclev10017849m.g	scaffold_2:27983235-27984341	8.07941	1.95691	-2.04568
-	scaffold_5:4426570-4426785	44.8241	10.859	-2.04539
Ciclev10010936m.g	scaffold_6:24321996-24326808	1.79806	0.436267	-2.04316
-	scaffold_1:9136968-9137196	115.474	28.0351	-2.04226
Ciclev10010970m.g	scaffold_6:21429204-21435111	0.318202	0.0772835	-2.04171
Ciclev10019333m.g	scaffold_3:6730669-6732517	7.21494	1.75269	-2.04141
Ciclev10005650m.g	scaffold_9:10547514-10549820	4.74685	1.15352	-2.04093
Ciclev10033003m.g	scaffold_4:7368910-7369710	20.2224	4.91535	-2.04059
Ciclev10013661m.g	scaffold_6:15603247-15618715	76.0298	18.486	-2.04013
Ciclev10017797m.g	scaffold_2:4539343-4539919	3.50008	0.851956	-2.03854
Ciclev10005894m.g	scaffold_9:2452852-2455181	146.914	35.8481	-2.035
Ciclev10001851m.g	scaffold_5:40053323-40055053	13.8933	3.39573	-2.03259
Ciclev10017402m.g	scaffold_2:13390080-13390603	12.2835	3.00691	-2.03036
Ciclev10010731m.g	scaffold_1:5129141-5129723	1.32724	0.325198	-2.02903
Ciclev10021329m.g	scaffold_3:3235233-3236815	3.67266	0.901052	-2.02714
Ciclev10004736m.g	scaffold_9:4429888-4432137	5.61285	1.37795	-2.02622
Ciclev10026695m.g	scaffold_7:20199310-20200356	3.42957	0.842174	-2.02584
Ciclev10003051m.g	scaffold_5:38056868-38057449	7.4154	1.82097	-2.02582
Ciclev10021255m.g	scaffold_3:40942973-40944742	1.20935	0.296982	-2.02579
Ciclev10011242m.g	scaffold_6:25225200-25228665	0.880599	0.216301	-2.02544
Ciclev10031483m.g	scaffold_4:708717-710339	58.4354	14.3891	-2.02186
Ciclev10020307m.g	scaffold_3:46296455-46298898	0.979704	0.241316	-2.02142
Ciclev10015908m.g	scaffold_2:28643549-28645682	0.507006	0.124905	-2.02117
Ciclev10009581m.g	scaffold_1:28022653-28025416	1.5043	0.370674	-2.02087
Ciclev10013488m.g	scaffold_6:15867970-15869805	0.926738	0.228427	-2.02043
-	scaffold_4:15296459-15296734	30.0636	7.41202	-2.02008

Ciclev10028412m.g	scaffold_8:24406838-24409538	9.39311	2.31584	-2.02007
Ciclev10024894m.g	scaffold_7:12825929-12831771	3.8203	0.942185	-2.0196
Ciclev10025631m.g	scaffold_7:1800672-1803725	5.72552	1.41215	-2.01952
-	scaffold_1:14847404-14848726	22.6539	5.58795	-2.01937
Ciclev10024535m.g	scaffold_3:48293738-48294416	8.40213	2.07383	-2.01846
Ciclev10005425m.g,Ciclev10005451m.g,Ciclev10006037m.g,Ciclev10006950m.g	scaffold_9:1567920-1599233	12.9743	3.20241	-2.01843
-	scaffold_5:26357418-26357590	397.584	98.1998	-2.01747
Ciclev10025322m.g	scaffold_7:8838601-8841703	2.93551	0.726295	-2.01498
Ciclev10006032m.g	scaffold_9:658783-659509	9.66263	2.39107	-2.01476
Ciclev10007940m.g,Ciclev10007942m.g	scaffold_1:24964825-24981789	6.0181	1.48969	-2.01429
-	scaffold_3:24927516-24927715	129.56	32.1659	-2.01001
Ciclev10031654m.g	scaffold_4:19460014-19462157	7.91279	1.96568	-2.00916
Ciclev10020109m.g	scaffold_3:3358969-3361865	9.94915	2.47384	-2.00782
Ciclev10033497m.g	scaffold_4:13941614-13951313	11.4585	2.84973	-2.00752
Ciclev10011800m.g	scaffold_6:5886743-5892919	11.1777	2.78006	-2.00744
-	scaffold_5:21062886-21063089	135.401	33.6898	-2.00685
Ciclev10031152m.g	scaffold_4:6273404-6278204	0.880703	0.219142	-2.00679
-	scaffold_3:14050517-14052882	1014.75	252.551	-2.00647
Ciclev10013191m.g	scaffold_6:22970177-22976234	4008.71	997.731	-2.00642
Ciclev10020248m.g	scaffold_3:8647528-8652777	8.22767	2.04817	-2.00615
Ciclev10027734m.g	scaffold_8:2848883-2853210	1.59026	0.395932	-2.00594
Ciclev10033728m.g	scaffold_4:2974962-2975469	1.45752	0.363064	-2.00522
Ciclev10032983m.g	scaffold_4:2274701-2276412	1.72403	0.429944	-2.00356
Ciclev10030719m.g	scaffold_4:7885338-7891653	286.036	71.4381	-2.00143
Ciclev10029391m.g	scaffold_8:19132836-19211350	30.5114	7.63356	-1.99892
Ciclev10029587m.g	scaffold_8:18844078-18844621	18.7455	4.69104	-1.99856
Ciclev10017273m.g	scaffold_2:22722919-22723789	11.9984	3.0103	-1.99487
Ciclev10009184m.g	scaffold_1:3921707-3938807	2517.02	631.529	-1.99479
Ciclev10025715m.g	scaffold_7:1880857-1882892	24.6508	6.18651	-1.99444
Ciclev10031199m.g	scaffold_4:23940022-23941837	8.25954	2.08758	-1.98423
Ciclev10016038m.g	scaffold_2:32934831-32936263	24.7419	6.2537	-1.98418
Ciclev10004919m.g	scaffold_9:4044470-4046388	0.336483	0.0851308	-1.98278
Ciclev10006525m.g	scaffold_9:7203919-7204668	1.22932	0.311513	-1.98049
Ciclev10031056m.g	scaffold_4:12795328-12800283	1.81096	0.459318	-1.97919
-	scaffold_3:8138434-8138696	53.9275	13.6959	-1.97727
Ciclev10005384m.g	scaffold_9:28704398-28707148	34.1631	8.67874	-1.97688
Ciclev10023841m.g	scaffold_3:7306771-7311180	2.15067	0.546773	-1.97578
-	scaffold_6:10809172-10809549	30.779	7.82565	-1.97567
Ciclev10021607m.g	scaffold_3:7083372-7084271	5.35817	1.36303	-1.97492
Ciclev10010144m.g	scaffold_1:24108906-24110284	0.83128	0.211528	-1.97448
Ciclev10015277m.g	scaffold_2:34357524-34359311	2.03248	0.517426	-1.97381
Ciclev10011844m.g	scaffold_6:19813517-19815060	15.7647	4.01387	-1.97363
-	scaffold_2:6791038-6792129	25.5162	6.49744	-1.97347
Ciclev10022480m.g	scaffold_3:2969909-2971645	60.0372	15.2927	-1.97301
Ciclev10024497m.g	scaffold_3:4399146-4399683	1.74208	0.444084	-1.97191
Ciclev10024949m.g	scaffold_7:5042507-5046278	93.8657	23.9435	-1.97097
Ciclev10023029m.g	scaffold_3:45895270-45897033	1883.11	480.837	-1.9695
Ciclev10003738m.g	scaffold_5:40017023-40019409	9.19776	2.35174	-1.96756
Ciclev10032181m.g	scaffold_4:24820268-24824732	86.8474	22.2061	-1.96753
Ciclev10000466m.g	scaffold_5:37349892-37356610	22.3036	5.70762	-1.96632
Ciclev10009462m.g	scaffold_1:10536406-10538155	13.1973	3.37879	-1.96567
Ciclev10016486m.g	scaffold_2:24720358-24721501	9.024	2.3109	-1.96531
Ciclev10030216m.g	scaffold_8:7698015-7862701	2.15976	0.553142	-1.96515

Ciclev10011883m.g	scaffold_6:25477064-25480914	19.9267	5.1066	-1.96426
Ciclev10019963m.g	scaffold_3:40950523-40954728	126.389	32.3941	-1.96407
Ciclev10029385m.g	scaffold_8:24102432-24104345	11.7178	3.00619	-1.96269
Ciclev10008151m.g	scaffold_1:1645090-1648238	0.451456	0.116007	-1.96038
Ciclev10001515m.g	scaffold_5:43079827-43083323	1.65065	0.424424	-1.95946
Ciclev10004037m.g	scaffold_5:38004850-38005786	0.889652	0.22884	-1.9589
Ciclev10023143m.g	scaffold_3:12330210-12331677	320.077	82.3581	-1.95844
Ciclev10021135m.g	scaffold_3:47341663-47343185	5.07997	1.30753	-1.95797
-	scaffold_5:3329894-3330220	467.633	120.5	-1.95635
Ciclev10011214m.g	scaffold_6:3129654-3132355	359.297	92.6405	-1.95546
Ciclev10017454m.g	scaffold_2:34672458-34672887	21.9038	5.65216	-1.9543
Ciclev10004733m.g	scaffold_9:27790597-27828145	10.8778	2.81391	-1.95075
Ciclev10006595m.g	scaffold_9:29529932-29534392	1.97987	0.512909	-1.94863
Ciclev10024638m.g	scaffold_3:15993929-16000998	4.36143	1.13107	-1.94711
Ciclev10008225m.g	scaffold_1:1070372-1072218	14.2571	3.69803	-1.94685
Ciclev10004465m.g	scaffold_9:27282486-27287560	31.7245	8.23079	-1.94649
Ciclev10015613m.g	scaffold_2:11920048-11922578	5.41855	1.40589	-1.94642
Ciclev10015924m.g	scaffold_2:21717831-21719535	3.86597	1.00422	-1.94475
Ciclev10004620m.g	scaffold_9:7579899-7583093	1106.77	288.146	-1.94149
Ciclev10009760m.g	scaffold_1:2082633-2083700	0.609533	0.158727	-1.94116
Ciclev10000835m.g	scaffold_5:36196012-36199839	162.422	42.2956	-1.94116
Ciclev10028778m.g	scaffold_8:2220868-2224576	35.0481	9.14117	-1.93889
Ciclev10015566m.g	scaffold_2:26170760-26172226	0.999481	0.260714	-1.93871
Ciclev10011933m.g	scaffold_6:18160532-18164662	7.231	1.88658	-1.93842
Ciclev10024731m.g	scaffold_7:2472536-2477250	2.21343	0.577562	-1.93824
Ciclev10005594m.g	scaffold_9:25161131-25163412	360.565	94.0849	-1.93822
Ciclev10030027m.g	scaffold_8:22354000-22355919	0.498618	0.130156	-1.9377
Ciclev10019935m.g	scaffold_3:7835501-7838656	3.32002	0.86854	-1.93452
Ciclev10013420m.g	scaffold_6:22556767-22558085	0.698233	0.18268	-1.93439
Ciclev10022257m.g	scaffold_3:8825969-8828784	3.01543	0.789497	-1.93336
Ciclev10008536m.g	scaffold_1:5477341-5483267	5.4853	1.43621	-1.9333
Ciclev10004867m.g	scaffold_9:29829158-29830992	23.8659	6.25492	-1.93189
Ciclev10033778m.g	scaffold_4:15584044-15584272	59.3938	15.5743	-1.93114
Ciclev10014091m.g	scaffold_2:23893669-23897580	7.07254	1.85634	-1.92977
Ciclev10021187m.g	scaffold_3:39657612-39660222	87.1446	22.9062	-1.92767
Ciclev10027275m.g	scaffold_7:2895802-2896693	1.46739	0.386121	-1.92613
-	scaffold_3:38078369-38107449	30.2575	7.9621	-1.92607
Ciclev10013346m.g	scaffold_6:17891315-17895884	1.21612	0.320041	-1.92596
-	scaffold_4:19675198-19675985	75.2778	19.8185	-1.92538
Ciclev10019669m.g	scaffold_3:51022516-51024687	1.33038	0.350933	-1.92257
Ciclev10021832m.g	scaffold_3:36924550-37054826	38.1114	10.0643	-1.92097
Ciclev10009957m.g	scaffold_1:19682378-19682942	368.726	97.3883	-1.92073
Ciclev10014860m.g	scaffold_2:7966422-7974085	4.56917	1.20747	-1.91995
Ciclev10002906m.g	scaffold_5:441330-538185	0.803515	0.212533	-1.91864
Ciclev10021119m.g	scaffold_3:7004357-7007866	53.8816	14.2783	-1.91597
Ciclev10001482m.g	scaffold_5:40075098-40076671	0.529513	0.140441	-1.91471
Ciclev10004715m.g	scaffold_9:30970842-30972432	1.13231	0.300399	-1.91431
Ciclev10033117m.g	scaffold_4:19355160-19355952	7.9261	2.1032	-1.91403
Ciclev10017549m.g	scaffold_2:32233591-32235124	0.43956	0.116656	-1.91379
-	scaffold_5:19013997-19014210	527.12	139.929	-1.91344
Ciclev10029439m.g	scaffold_8:19760076-19761961	124.002	32.919	-1.91337
Ciclev10024934m.g	scaffold_7:7826602-7829404	3.22041	0.855603	-1.91223
Ciclev10020214m.g	scaffold_3:41627893-41634431	19.472	5.17475	-1.91184
Ciclev10028824m.g	scaffold_8:21432040-21433970	69.8376	18.5729	-1.9108
-	scaffold_4:5209549-5209696	1397.99	371.969	-1.9101
Ciclev10017032m.g	scaffold_2:24652426-24654825	144.484	38.4442	-1.91007

Ciclev10014884m.g	scaffold_2:22867079-22870230	1.17969	0.313938	-1.90986
-	scaffold_3:7695162-7696250	4.5665	1.21541	-1.90964
Ciclev10027236m.g	scaffold_7:11062213-11079547	0.368613	0.098163	-1.90886
Ciclev10008439m.g,Ciclev10009601m.g	scaffold_1:22443441-22450169	89.3219	23.7889	-1.90873
Ciclev10013274m.g	scaffold_6:17126056-17129828	1.6096	0.429094	-1.90734
Ciclev10020994m.g	scaffold_3:19310539-19608202	0.557158	0.148534	-1.90729
Ciclev10020342m.g	scaffold_3:45233398-45241100	21.8815	5.83438	-1.90706
Ciclev10007444m.g	scaffold_1:6481259-6485491	1.02095	0.272603	-1.90504
Ciclev10004505m.g	scaffold_9:29768401-29770592	0.373555	0.0998163	-1.90397
Ciclev10030143m.g	scaffold_8:5652682-5657571	5.47567	1.46494	-1.9022
Ciclev10002074m.g	scaffold_5:33745670-33747052	1.09208	0.292193	-1.90208
Ciclev10002233m.g	scaffold_5:42243512-42244971	0.471816	0.12628	-1.90159
Ciclev10010809m.g	scaffold_1:15875750-15879724	2.84153	0.760605	-1.90145
Ciclev10022982m.g	scaffold_3:42772686-42773490	37.5757	10.0604	-1.90111
Ciclev10014671m.g	scaffold_2:5905223-5907905	20.4834	5.48482	-1.90094
Ciclev10021075m.g	scaffold_3:50263606-50266309	7.84408	2.10289	-1.89923
Ciclev10001844m.g	scaffold_5:165096-169348	325.856	87.47	-1.89737
Ciclev10022279m.g	scaffold_3:50687205-50688531	1.04071	0.279414	-1.89709
Ciclev10014744m.g	scaffold_2:22836154-22838521	230.436	61.9125	-1.89606
-	scaffold_3:42897297-42897581	40.3496	10.8423	-1.89589
Ciclev10032964m.g	scaffold_4:24505869-24507677	17.5325	4.71603	-1.89438
Ciclev10023207m.g	scaffold_3:8383302-8410026	1.06829	0.287379	-1.89428
Ciclev10024191m.g	scaffold_3:2594993-2596969	0.602807	0.162314	-1.89291
Ciclev10008006m.g	scaffold_1:7729680-7737152	1.76718	0.476193	-1.89183
Ciclev10029464m.g	scaffold_8:23078211-23079067	87.8136	23.6691	-1.89144
-	scaffold_5:39112671-39121491	42.4262	11.4414	-1.89069
Ciclev10033128m.g	scaffold_4:16316208-16317306	8.03724	2.1675	-1.89067
Ciclev10028301m.g	scaffold_8:6681475-6683886	1.19019	0.321396	-1.88877
Ciclev10024419m.g	scaffold_3:38260451-38263224	0.423204	0.114292	-1.88862
Ciclev10032808m.g	scaffold_4:11975876-11976579	4.28637	1.1576	-1.88862
Ciclev10002567m.g	scaffold_5:34971111-34972764	7.06826	1.90979	-1.88794
Ciclev10028003m.g	scaffold_8:286659-289583	5.63958	1.52388	-1.88784
Ciclev10015582m.g	scaffold_2:4441284-4443386	7.27606	1.9669	-1.88724
Ciclev10010591m.g	scaffold_1:24935568-24937990	0.605941	0.163906	-1.88631
Ciclev10018037m.g	scaffold_2:5155107-5159194	3.24633	0.878494	-1.88571
Ciclev10019374m.g	scaffold_3:2648279-2652347	11.3065	3.06067	-1.88523
Ciclev10000131m.g	scaffold_5:33831343-33837397	0.15623	0.0423167	-1.88437
Ciclev10028907m.g	scaffold_8:1710548-1711561	0.677169	0.183471	-1.88396
Ciclev10015249m.g	scaffold_2:7725023-7728058	14.0772	3.8144	-1.88383
Ciclev10027260m.g	scaffold_7:5823311-5823527	401.176	108.719	-1.88364
Ciclev10008811m.g	scaffold_1:14458466-14461177	3.12239	0.846321	-1.88337
Ciclev10016598m.g	scaffold_2:12292132-12293357	1.749	0.474469	-1.88214
Ciclev10022286m.g	scaffold_3:49145486-49149088	151.589	41.1402	-1.88155
Ciclev10021633m.g	scaffold_3:5865765-5866801	7.31181	1.98465	-1.88134
Ciclev10017634m.g	scaffold_2:26441160-26442363	0.52418	0.142298	-1.88115
Ciclev10006943m.g	scaffold_9:30240997-30243403	1.52664	0.414533	-1.8808
Ciclev10020945m.g	scaffold_3:7526226-7527689	2.08491	0.566555	-1.87969
Ciclev10008496m.g	scaffold_1:25940744-25942304	61.8799	16.8233	-1.87901
Ciclev10008801m.g	scaffold_1:8854441-8858300	13.0074	3.53963	-1.87766
Ciclev10024267m.g	scaffold_3:24026647-24169357	0.802734	0.218473	-1.87747
Ciclev10032084m.g	scaffold_4:12308932-12311438	172.113	46.8451	-1.87738
Ciclev10007841m.g	scaffold_1:22175413-22384190	0.623089	0.169592	-1.87737
Ciclev10025900m.g	scaffold_7:678522-685008	15.5897	4.24608	-1.87639
Ciclev10003356m.g	scaffold_5:8438994-8439210	4198.31	1144.54	-1.87504
Ciclev10026269m.g	scaffold_7:20888715-20891804	0.574146	0.156699	-1.87342

Ciclev10018129m.g	scaffold_2:17956818-17957625	0.907572	0.247755	-1.87309
-	scaffold_7:4608865-4609623	24.4969	6.70076	-1.8702
Ciclev10001964m.g	scaffold_5:39852395-39853836	1.87605	0.513756	-1.86855
Ciclev10012776m.g	scaffold_6:17943467-17944691	34.2264	9.374	-1.86837
Ciclev10029120m.g	scaffold_8:4464680-4466432	0.404001	0.110659	-1.86824
Ciclev10015945m.g	scaffold_2:9224783-9226076	37.725	10.3343	-1.86808
Ciclev10014459m.g	scaffold_2:11493088-11497181	1.35402	0.371335	-1.86646
Ciclev10026775m.g	scaffold_7:11294376-11295280	745.734	204.718	-1.86502
Ciclev10012089m.g	scaffold_6:14087180-14090115	17.2883	4.74722	-1.86464
Ciclev10014192m.g	scaffold_2:6777394-6790546	4.85957	1.33543	-1.86353
Ciclev10019368m.g	scaffold_3:13977376-13980070	106.416	29.2739	-1.86203
Ciclev10013984m.g	scaffold_1238:114-1954	1.35378	0.372565	-1.86143
Ciclev10032510m.g	scaffold_4:19622199-19623356	98.7766	27.1989	-1.86062
Ciclev10021524m.g	scaffold_3:49614816-49617330	0.506329	0.139433	-1.8605
Ciclev10019410m.g	scaffold_3:5991280-5995312	7.96713	2.19561	-1.85944
Ciclev10027636m.g	scaffold_1557:5-854	1.12787	0.310955	-1.85882
Ciclev10015993m.g	scaffold_2:25220620-25223548	0.333131	0.091933	-1.85744
Ciclev10022665m.g	scaffold_3:43111075-43112277	37.5522	10.3648	-1.8572
-	scaffold_3:41537757-41537946	710.399	196.094	-1.85708
Ciclev10025204m.g	scaffold_7:16594625-16599371	7.19216	1.9864	-1.85627
Ciclev10026188m.g	scaffold_7:11686788-11771145	0.984138	0.272251	-1.85393
Ciclev10018724m.g	scaffold_3:33748482-33753225	0.138272	0.0382628	-1.8535
Ciclev10012328m.g	scaffold_6:21780333-21781641	2.12195	0.587396	-1.85298
Ciclev10019533m.g	scaffold_3:47390659-47394084	14.9383	4.13597	-1.85272
Ciclev10022679m.g	scaffold_3:46168530-46169395	4.51142	1.24938	-1.85237
Ciclev10014947m.g	scaffold_2:23956661-23961945	12.6878	3.51518	-1.85177
Ciclev10033404m.g	scaffold_4:18127125-18130447	0.193112	0.05351	-1.85156
Ciclev10024479m.g	scaffold_3:43011640-43045787	1.69864	0.470707	-1.85148
Ciclev10014114m.g	scaffold_2:11943426-11947775	1.32089	0.366063	-1.85134
Ciclev10019128m.g	scaffold_3:3816467-3819514	0.203452	0.056411	-1.85064
Ciclev10024475m.g	scaffold_3:41318013-41318352	334.361	92.7263	-1.85036
-	scaffold_8:10945427-10946324	13.8892	3.85281	-1.84998
Ciclev10030504m.g	scaffold_4:21644110-21650532	9.7529	2.70583	-1.84976
Ciclev10006513m.g	scaffold_9:15198916-15202417	0.518784	0.14408	-1.84827
Ciclev10018340m.g	scaffold_2:11478184-11480297	0.406982	0.113059	-1.84789
Ciclev10016836m.g	scaffold_2:32094157-32095159	1.1565	0.321581	-1.84651
-	scaffold_5:5805570-5807385	23.01	6.40808	-1.8443
Ciclev10010546m.g	scaffold_1:1395147-1398082	0.720393	0.200718	-1.84361
Ciclev10000393m.g	scaffold_5:15822059-15834446	2.42918	0.676868	-1.84353
-	scaffold_2:25403703-25404226	23.2564	6.48528	-1.84239
Ciclev10026277m.g	scaffold_7:2721714-2817730	306.732	85.5624	-1.84193
Ciclev10026954m.g	scaffold_7:19842016-19843066	4.57362	1.27824	-1.83918
Ciclev10025174m.g	scaffold_7:20874760-20878272	104.61	29.2411	-1.83895
Ciclev10000600m.g	scaffold_5:113390-116883	104.858	29.3152	-1.83871
-	scaffold_2:34919344-34920599	127.41	35.6333	-1.83818
Ciclev10013035m.g	scaffold_6:17629347-17629878	2.44939	0.685752	-1.83667
-	scaffold_8:23426576-23426791	478.124	133.927	-1.83593
Ciclev10004059m.g	scaffold_5:10427531-10584700	32.4208	9.08221	-1.8358
Ciclev10030941m.g	scaffold_4:2414210-2417518	13.5434	3.81062	-1.82949
Ciclev10008968m.g	scaffold_1:3962618-3964620	23.3409	6.56801	-1.82933
Ciclev10004722m.g	scaffold_9:23371251-23374087	0.609064	0.171558	-1.82789
Ciclev10007018m.g	scaffold_9:298053-299540	32.7162	9.22315	-1.82667
Ciclev10028367m.g	scaffold_8:20273416-20274830	1.9476	0.549068	-1.82664
Ciclev10013653m.g	scaffold_6:18506890-18509157	5.53572	1.56359	-1.82391
Ciclev10021802m.g	scaffold_3:123864-125349	0.538846	0.152221	-1.82371
Ciclev10001909m.g	scaffold_5:25757752-25759654	8.51494	2.40773	-1.82232

-	scaffold_4:6156948-6157148	239.69	67.792	-1.82198
-	scaffold_2:27865390-27865937	44.1428	12.4911	-1.82128
Ciclev10019107m.g	scaffold_3:9414396-9419290	26.12	7.39145	-1.82123
Ciclev10019121m.g	scaffold_3:7621981-7626081	270.193	76.488	-1.82069
Ciclev10001956m.g	scaffold_5:15762363-15766976	1289	364.972	-1.8204
Ciclev10033723m.g	scaffold_4:16817583-16984703	322.662	91.3682	-1.82026
Ciclev10009896m.g	scaffold_1:20546639-20549368	1.67776	0.475425	-1.81925
Ciclev10029482m.g	scaffold_8:17992652-17993246	11.8796	3.36985	-1.81774
Ciclev10016436m.g	scaffold_2:34381654-34382452	11.8132	3.35163	-1.81746
Ciclev10017901m.g	scaffold_2:22353273-22356112	0.206783	0.0587672	-1.81503
Ciclev10006049m.g	scaffold_9:1724964-1727375	23.6485	6.72681	-1.81375
Ciclev10008649m.g	scaffold_1:17363822-17365612	62.8805	17.8918	-1.81331
-	scaffold_6:10808000-10808279	50.0166	14.232	-1.81327
Ciclev10028419m.g	scaffold_8:23919567-23921211	2.27068	0.64615	-1.81318
Ciclev10026743m.g	scaffold_7:7723299-7724124	2.62933	0.748593	-1.81244
Ciclev10030808m.g	scaffold_4:24123582-24127783	0.250585	0.0713684	-1.81194
Ciclev10022668m.g	scaffold_3:46847633-46848378	2.9585	0.843411	-1.81056
Ciclev10026703m.g	scaffold_7:10853486-10855215	1.78407	0.508877	-1.80978
Ciclev10031390m.g	scaffold_4:24362007-24364244	21.4927	6.13359	-1.80905
-	scaffold_5:31333200-31391489	18.4639	5.27182	-1.80834
Ciclev10028053m.g	scaffold_8:2799454-2802342	10.772	3.0772	-1.80759
Ciclev10025703m.g	scaffold_7:662559-667430	14.5666	4.16125	-1.80758
-	scaffold_3:17869049-17869455	192.711	55.0738	-1.807
Ciclev10027805m.g	scaffold_8:22165580-22171683	4.9574	1.41743	-1.8063
Ciclev10012472m.g	scaffold_6:21446970-21449098	7.44619	2.13114	-1.80487
Ciclev10024530m.g	scaffold_3:27320757-27322899	0.246194	0.0704711	-1.80469
Ciclev10017727m.g	scaffold_2:30412455-30416887	0.172053	0.0492828	-1.8037
Ciclev10004050m.g	scaffold_5:39282607-39283237	3.4799	0.99691	-1.80351
Ciclev10032391m.g	scaffold_4:18856877-18860765	2.8026	0.804175	-1.80118
Ciclev10033903m.g	scaffold_4:18515567-18675155	0.322411	0.0925302	-1.8009
Ciclev10013437m.g	scaffold_6:17715195-17716558	0.678588	0.194804	-1.80051
Ciclev10020940m.g	scaffold_3:41848676-41854429	3.47782	0.998602	-1.8002
Ciclev10008918m.g	scaffold_1:21664720-21665886	0.584881	0.168049	-1.79926
Ciclev10030627m.g	scaffold_4:14034228-14040846	3.08985	0.888241	-1.79852
Ciclev10027535m.g	scaffold_7:10507046-10509551	0.194719	0.0559887	-1.79819
Ciclev10020415m.g	scaffold_3:11687448-12154880	26.6868	7.67448	-1.79799
Ciclev10006102m.g	scaffold_9:1205866-1207398	0.605131	0.174127	-1.79711
Ciclev10032559m.g	scaffold_4:16092829-16094966	11.1184	3.20314	-1.79539
Ciclev10032412m.g	scaffold_4:23333288-23337136	2.66571	0.768122	-1.79511
-	scaffold_4:7466197-7466627	7.75115	2.23415	-1.79469
Ciclev10029741m.g	scaffold_8:23095726-23096226	28.8733	8.34036	-1.79155
Ciclev10026302m.g	scaffold_7:13717778-13722266	38.1159	11.0368	-1.78807
Ciclev10025503m.g	scaffold_7:8951805-8954729	1.83307	0.530871	-1.78782
Ciclev10021702m.g	scaffold_3:9046601-9047756	6.1365	1.77766	-1.78744
Ciclev10013017m.g	scaffold_6:21165236-21165939	2.19116	0.635142	-1.78655
Ciclev10023418m.g	scaffold_3:23477657-23482415	0.174706	0.0507027	-1.78479
-	scaffold_7:4325048-4325402	33.3503	9.6838	-1.78406
Ciclev10004881m.g	scaffold_9:17070430-17072209	32.9858	9.57972	-1.78379
Ciclev10009914m.g	scaffold_1:12048207-12049095	52.9562	15.3843	-1.78334
Ciclev10031145m.g	scaffold_4:21537707-21545270	3.70897	1.07923	-1.78102
Ciclev10000363m.g	scaffold_5:34479396-34482201	42.7048	12.4396	-1.77946
Ciclev10025388m.g	scaffold_7:15526234-15537832	19.5307	5.68913	-1.77946
Ciclev10013129m.g	scaffold_6:25568568-25569321	82.4763	24.0282	-1.77925
Ciclev10025367m.g	scaffold_7:4406271-4410156	4.78762	1.39592	-1.7781
-	scaffold_5:30357144-30357670	9.64103	2.81241	-1.77738
Ciclev10010749m.g	scaffold_1:13865892-13872943	36.9999	10.7992	-1.7766

Ciclev10019977m.g	scaffold_3:7777256-7778895	2.73257	0.797555	-1.7766
Ciclev10011709m.g	scaffold_6:24764197-24767525	2.28579	0.66788	-1.77503
-	scaffold_6:8264229-8264686	6.88714	2.01334	-1.77431
Ciclev10009246m.g	scaffold_1:4043015-4044281	24.2371	7.09151	-1.77305
Ciclev10008742m.g	scaffold_1:2784789-2787063	1347.9	394.46	-1.77276
Ciclev10031682m.g	scaffold_4:583973-586423	101.391	29.7091	-1.77096
Ciclev10026527m.g	scaffold_7:7337322-7338669	515.997	151.289	-1.77005
Ciclev10011386m.g	scaffold_6:17113587-17116563	35.1821	10.3208	-1.76928
-	scaffold_9:17699394-17699712	17.3908	5.10244	-1.76907
Ciclev10026073m.g	scaffold_7:5557829-5560118	12.6133	3.70283	-1.76824
Ciclev10031809m.g	scaffold_4:22390118-22394239	9.52353	2.79602	-1.76812
Ciclev10032585m.g	scaffold_4:3102074-3103327	4.99034	1.46551	-1.76774
Ciclev10031060m.g	scaffold_4:19409167-19412705	167.229	49.1328	-1.76707
Ciclev10032154m.g	scaffold_4:24623710-24627543	3.97672	1.17004	-1.76502
Ciclev10003899m.g	scaffold_5:24926144-25131690	259.134	76.3578	-1.76285
-	scaffold_5:27310156-27310539	25.7383	7.5908	-1.76159
-	scaffold_5:40670956-40671733	134.308	39.6107	-1.76158
Ciclev10011382m.g	scaffold_6:21365188-21367260	33.3198	9.83156	-1.76089
Ciclev10005208m.g,Ciclev10005211m.g	scaffold_9:5236115-5256826	119.727	35.411	-1.75748
Ciclev10006201m.g	scaffold_9:1828507-1829243	264.985	78.4274	-1.75648
Ciclev10030930m.g	scaffold_4:22697055-22699336	0.641593	0.189945	-1.75608
Ciclev10013661m.g	scaffold_6:15603247-15618715	1016.75	301.153	-1.75539
Ciclev10029315m.g	scaffold_8:20424286-20425903	415.924	123.303	-1.75412
Ciclev10025280m.g	scaffold_7:15456947-15460803	47.2224	14.0075	-1.75327
Ciclev10003713m.g	scaffold_5:38273885-38275424	2.25945	0.670296	-1.7531
Ciclev10011487m.g	scaffold_6:22531603-22535175	31.6768	9.39841	-1.75294
Ciclev10014329m.g	scaffold_2:5368058-5378200	22.1574	6.57444	-1.75285
-	scaffold_9:28137586-28139112	68.4753	20.3265	-1.75222
Ciclev10002529m.g	scaffold_5:24735873-24738253	1.68284	0.499812	-1.75144
Ciclev10020116m.g	scaffold_3:39072047-39079586	178.81	53.1462	-1.75039
-	scaffold_5:28539341-28801354	10.3766	3.09136	-1.74702
-	scaffold_9:28297044-28297203	644.047	191.981	-1.7462
Ciclev10008127m.g	scaffold_1:25022793-25024741	1.43829	0.428821	-1.74591
Ciclev10010059m.g	scaffold_1:18812069-18812720	12.7031	3.79137	-1.74439
Ciclev10019279m.g	scaffold_3:50868095-50873164	8.83609	2.63946	-1.74317
-	scaffold_5:21797044-21797239	201.239	60.1633	-1.74195
Ciclev10010051m.g	scaffold_1:2954355-2954931	9.10305	2.72427	-1.74048
Ciclev10021652m.g	scaffold_3:5240107-5241300	70.7885	21.1953	-1.73977
Ciclev10021450m.g	scaffold_3:42357954-42361557	239.137	71.6057	-1.73969
Ciclev10020144m.g	scaffold_3:47189458-47191383	1.77444	0.531469	-1.73931
Ciclev10000089m.g	scaffold_5:33758214-33765582	2.02178	0.605801	-1.73871
Ciclev10002813m.g	scaffold_5:36034339-36035011	16.5688	4.97122	-1.7368
-	scaffold_3:45005959-45008639	95.6262	28.7068	-1.73601
Ciclev10013764m.g	scaffold_6:20201379-20214287	1.19627	0.359865	-1.73301
-	scaffold_111:8221-8355	5827.98	1754.09	-1.73227
Ciclev10013805m.g	scaffold_6:22397591-22397792	18.8342	5.67007	-1.73192
Ciclev10019790m.g	scaffold_3:8640419-8644420	14.3489	4.32611	-1.7298
Ciclev10019828m.g	scaffold_3:348559-351141	1.9253	0.580617	-1.72942
Ciclev10027185m.g	scaffold_7:10990910-11007799	94.1406	28.4116	-1.72834
Ciclev10024551m.g	scaffold_3:25026667-25030809	0.756256	0.228256	-1.72822
Ciclev10015421m.g	scaffold_2:13836741-13840427	5.0571	1.52692	-1.72769
Ciclev10030797m.g	scaffold_4:23618423-23625625	2.93485	0.886286	-1.72744
Ciclev10020504m.g	scaffold_3:46981627-46984276	460.008	138.931	-1.72729
Ciclev10017884m.g	scaffold_2:31275695-31277348	2.03203	0.613907	-1.72683
Ciclev10027176m.g	scaffold_7:4670800-4676448	1.58902	0.480548	-1.72538

Ciclev10025939m.g	scaffold_7:17255495-17259980	31.8681	9.63828	-1.72526
Ciclev10016774m.g	scaffold_2:23058500-23059689	1.77284	0.536309	-1.72493
Ciclev10014642m.g	scaffold_2:35513795-35516719	242.766	73.4943	-1.72386
Ciclev10026866m.g	scaffold_7:17841900-17842342	21.7737	6.59763	-1.72257
Ciclev10019326m.g	scaffold_3:47830376-47832891	12.5421	3.80563	-1.72057
Ciclev10005366m.g	scaffold_9:28444511-28446074	2.83115	0.859081	-1.72052
Ciclev10002523m.g	scaffold_5:43186617-43190241	61.8237	18.7643	-1.72017
Ciclev10012753m.g	scaffold_6:15443542-15445283	2.62839	0.79834	-1.7191
Ciclev10016924m.g	scaffold_2:34204472-34205506	79.1711	24.0661	-1.71797
Ciclev10005968m.g	scaffold_9:27640073-27641178	41.3104	12.5591	-1.71777
Ciclev10025311m.g	scaffold_7:1492716-1495961	5.7487	1.74834	-1.71725
-	scaffold_9:11128953-11129218	103.275	31.4113	-1.71714
Ciclev10013012m.g	scaffold_6:22520369-22521865	42.0739	12.7988	-1.71692
Ciclev10011715m.g,Ciclev10013108m.g	scaffold_6:12546929-12554522	207.063	63.011	-1.71639
Ciclev10016648m.g	scaffold_2:22386726-22388706	76.7819	23.3675	-1.71626
Ciclev10015207m.g	scaffold_2:32184972-32187593	545.117	165.96	-1.71573
Ciclev10013886m.g	scaffold_6:14515616-14517074	0.632882	0.192722	-1.71542
Ciclev10034002m.g	scaffold_4:2468133-2469664	2.67542	0.815741	-1.71358
Ciclev10021038m.g	scaffold_3:1054256-1056078	72.5806	22.1431	-1.71273
Ciclev10029216m.g	scaffold_8:309616-317158	3.92193	1.19657	-1.71266
Ciclev10031368m.g	scaffold_4:308083-310275	90.0069	27.4886	-1.7112
Ciclev10004732m.g	scaffold_9:28990093-28993179	57.5786	17.6047	-1.70957
Ciclev10007993m.g	scaffold_1:24986847-24990195	17.7477	5.43156	-1.70819
Ciclev10015578m.g	scaffold_2:13525591-13527457	2.73148	0.836425	-1.70738
Ciclev10023956m.g	scaffold_3:50663969-50666133	3.53447	1.0825	-1.70713
Ciclev10019938m.g	scaffold_3:48089472-48091232	10.2342	3.1363	-1.70627
Ciclev10012794m.g	scaffold_6:23084454-23085332	2.02292	0.62111	-1.70352
Ciclev10001701m.g	scaffold_5:35972716-35974167	6.67355	2.0493	-1.70333
Ciclev10027030m.g,Ciclev10027467m.g	scaffold_7:13916806-14069972	33.9545	10.4337	-1.70235
-	scaffold_6:22782971-22783449	7.60387	2.33694	-1.70211
Ciclev10011736m.g	scaffold_6:22166951-22168833	53.0984	16.325	-1.70158
Ciclev10024670m.g	scaffold_3:8605286-8607326	4.40249	1.35399	-1.70111
Ciclev10033268m.g	scaffold_4:21093688-21094739	4.07364	1.25306	-1.70087
-	scaffold_1:20139401-20139769	1423.66	438.105	-1.70026
Ciclev10031627m.g	scaffold_4:15087930-15266082	0.86001	0.264664	-1.70019
Ciclev10032280m.g	scaffold_4:24497388-24499313	13.4625	4.14355	-1.70001
Ciclev10030385m.g	scaffold_8:15117014-15119555	114.245	35.1753	-1.6995
Ciclev10019467m.g	scaffold_3:8330156-8337179	1274.59	392.494	-1.69929
Ciclev10031232m.g	scaffold_4:12359537-12362422	12.3942	3.8223	-1.69715
Ciclev10024972m.g	scaffold_7:5094560-5100082	10.6815	3.29451	-1.69698
Ciclev10016111m.g	scaffold_2:32948414-32957830	8.42801	2.60017	-1.69659
Ciclev10020489m.g	scaffold_3:45087591-45089434	4.15214	1.28164	-1.69586
Ciclev10008142m.g	scaffold_1:6402442-6406121	25.7725	7.95657	-1.69561
Ciclev10010195m.g	scaffold_1:4053586-4054930	2.28455	0.705358	-1.69549
Ciclev10014670m.g	scaffold_2:17174408-17177145	58.5552	18.1024	-1.69362
Ciclev10018085m.g	scaffold_2:21029510-21030572	4.54542	1.40584	-1.69299
Ciclev10013568m.g	scaffold_6:4405921-4407715	27.6641	8.55842	-1.6926
Ciclev10017073m.g	scaffold_2:33720253-33720869	65.8151	20.3646	-1.69236
Ciclev10032862m.g	scaffold_4:1438583-1439617	3.79489	1.17461	-1.69187
Ciclev10003661m.g	scaffold_5:39592381-39593026	6.34816	1.96704	-1.69031
Ciclev10009966m.g	scaffold_1:18803915-18804969	262.584	81.3926	-1.68981
Ciclev10011864m.g	scaffold_6:8798479-8801299	10.479	3.24837	-1.68971
Ciclev10011707m.g	scaffold_6:22197698-22200838	1.4274	0.442665	-1.6891
Ciclev10019633m.g	scaffold_3:48934414-48937228	6.49776	2.01744	-1.68742

Ciclev10028011m.g	scaffold_8:3117336-3120914	62.7633	19.4965	-1.6867
Ciclev10019379m.g	scaffold_3:2330116-2334317	2.91096	0.905334	-1.68498
Ciclev10011974m.g	scaffold_6:18937352-18940413	5.3999	1.67959	-1.68482
Ciclev10016616m.g	scaffold_2:25723305-25725690	35.6073	11.0839	-1.68371
Ciclev10015299m.g	scaffold_2:30818814-30820876	0.604235	0.188345	-1.68174
Ciclev10001636m.g	scaffold_5:39928704-39930585	67.6135	21.0756	-1.68174
Ciclev10010831m.g	scaffold_1:26059372-26066070	4.16016	1.29711	-1.68134
Ciclev10027494m.g	scaffold_7:15556462-15556847	2010.26	628.269	-1.67793
-	scaffold_2:6042108-6042315	111.236	34.8047	-1.67627
Ciclev10030798m.g	scaffold_4:21982830-21986241	0.642219	0.201103	-1.67513
Ciclev10031648m.g	scaffold_4:24422372-24425478	13.9872	4.38759	-1.67261
Ciclev10026351m.g	scaffold_7:8678321-8680075	56.0517	17.5846	-1.67244
Ciclev10028516m.g	scaffold_8:23168353-23172621	68.5936	21.5214	-1.6723
-	scaffold_4:11522840-11523142	69.9445	21.9518	-1.67187
Ciclev10030371m.g	scaffold_8:5642819-5646489	5.02443	1.57691	-1.67186
Ciclev10019205m.g	scaffold_3:46730992-46737126	103.855	32.6037	-1.67146
Ciclev10004795m.g	scaffold_9:22620067-22622404	1.85017	0.580992	-1.67106
Ciclev10025329m.g	scaffold_7:5159994-5165972	73.3335	23.0444	-1.67005
Ciclev10022290m.g	scaffold_3:47562252-47564186	37.3471	11.7369	-1.66994
Ciclev10033283m.g	scaffold_4:3678191-3678788	339.975	106.911	-1.66902
Ciclev10021160m.g	scaffold_3:40166644-40168022	4.1865	1.31673	-1.66879
Ciclev10019975m.g	scaffold_3:33683416-33687458	2.60166	0.818513	-1.66835
Ciclev10018823m.g	scaffold_3:30878294-30882778	4.76143	1.49806	-1.6683
Ciclev10019339m.g	scaffold_3:48063482-48066386	180.801	56.9082	-1.6677
-	scaffold_2:12106493-12107162	24.3203	7.65672	-1.66736
Ciclev10014440m.g	scaffold_2:35429102-35438566	27.8395	8.78256	-1.66442
Ciclev10004254m.g	scaffold_9:26920857-26925316	1.62164	0.512099	-1.66296
Ciclev10013311m.g	scaffold_6:12933746-12934265	6.15539	1.94397	-1.66284
Ciclev10001166m.g	scaffold_5:42314433-42320000	19.1331	6.04607	-1.662
Ciclev10030541m.g	scaffold_4:18413040-18422725	0.451629	0.143065	-1.65847
-	scaffold_5:2787300-2787760	45.5785	14.4381	-1.65847
Ciclev10009871m.g	scaffold_1:8013459-8015328	33.122	10.501	-1.65726
-	scaffold_2:6776343-6776659	485.534	153.979	-1.65684
Ciclev10004685m.g	scaffold_9:629822-634705	17.3971	5.52096	-1.65586
Ciclev10008010m.g	scaffold_1:14592247-14596429	5.69271	1.80828	-1.6545
Ciclev10005136m.g	scaffold_9:8566657-8568825	7.13532	2.26771	-1.65374
Ciclev10014329m.g	scaffold_2:5368058-5378200	6.23254	1.98309	-1.65207
Ciclev10010050m.g	scaffold_1:17879965-17882892	2.64893	0.843159	-1.65153
Ciclev10025475m.g	scaffold_7:8845919-8853010	49.4936	15.7551	-1.65142
Ciclev10025452m.g	scaffold_7:8340087-8344492	2.43461	0.775735	-1.65005
Ciclev10027727m.g	scaffold_8:20731793-20739112	2.4426	0.778549	-1.64956
Ciclev10018250m.g	scaffold_2:25692667-25693309	3.10927	0.992499	-1.64744
Ciclev10033019m.g	scaffold_4:25166545-25167334	186.196	59.4496	-1.64709
Ciclev10009229m.g	scaffold_1:17879965-17882892	11.4288	3.65218	-1.64584
-	scaffold_2:25384067-25387970	18.8989	6.04238	-1.64512
Ciclev10028876m.g,Ciclev10030431m.g	scaffold_8:22743958-22747581	22.1202	7.07595	-1.64437
Ciclev10023260m.g	scaffold_3:25494655-25499648	0.734695	0.235187	-1.64334
Ciclev10008675m.g	scaffold_1:27187044-27191517	5.36027	1.71595	-1.6433
Ciclev10008077m.g	scaffold_1:5619537-5622746	1.72358	0.552529	-1.64128
Ciclev10026501m.g	scaffold_7:529835-530825	181.68	58.2875	-1.64014
Ciclev10014128m.g	scaffold_2:29637443-29641901	0.486787	0.156219	-1.63972
Ciclev10021877m.g	scaffold_3:47803711-47805628	245.782	78.9411	-1.63853
Ciclev10020410m.g	scaffold_3:5967420-5971786	4.34227	1.39588	-1.63727
Ciclev10021501m.g	scaffold_3:50399570-50401808	1.74197	0.560074	-1.63703
-	scaffold_2:13931314-13931556	62.3867	20.0823	-1.63531

Ciclev10022153m.g	scaffold_3:5280718-5282122	4.67649	1.50541	-1.63527
Ciclev10026364m.g	scaffold_7:16185817-16187223	55.4327	17.8466	-1.63509
Ciclev10014742m.g	scaffold_2:35938046-35940274	0.540498	0.174123	-1.63418
Ciclev10009681m.g	scaffold_1:173462-175858	162.69	52.4208	-1.63392
Ciclev10003131m.g	scaffold_5:35337109-35341788	2.24347	0.722905	-1.63385
-	scaffold_8:6434299-6453817	33.6247	10.8423	-1.63285
Ciclev10013391m.g	scaffold_6:13114620-13114884	143.954	46.4293	-1.6325
Ciclev10008351m.g	scaffold_1:20457914-20462359	6.11186	1.9713	-1.63246
Ciclev10033427m.g	scaffold_4:20708796-20712687	2.47253	0.797525	-1.63238
Ciclev10018906m.g	scaffold_3:5182211-5185070	2.92015	0.9423	-1.63178
-	scaffold_9:1843626-1844249	10.0704	3.25001	-1.63161
Ciclev10011588m.g	scaffold_6:11722969-11726023	0.430067	0.138915	-1.63036
-	scaffold_5:655534-811195	22.3746	7.23163	-1.62947
Ciclev10017731m.g	scaffold_2:35303011-35304493	1.37802	0.445491	-1.62913
Ciclev10006881m.g	scaffold_9:21184270-21186264	19.4344	6.28569	-1.62847
Ciclev10031341m.g,Ciclev10032478m.g	scaffold_4:10959734-10988823	34.1694	11.059	-1.62748
Ciclev10024513m.g	scaffold_3:45486338-45491014	4.13321	1.33984	-1.6252
Ciclev10007907m.g	scaffold_1:21476759-21547573	11.9071	3.86435	-1.62352
Ciclev10024155m.g	scaffold_3:10116691-10117773	2.60256	0.845147	-1.62265
Ciclev10025455m.g	scaffold_7:5359516-5365577	42.5374	13.8166	-1.62233
-	scaffold_2:21693952-21694517	6.66031	2.16375	-1.62206
Ciclev10002288m.g	scaffold_5:40752810-40753702	4.55299	1.47955	-1.62165
Ciclev10020360m.g	scaffold_3:47549852-47553563	4.29468	1.39581	-1.62145
Ciclev10006002m.g	scaffold_9:4524400-4525971	18.0804	5.88394	-1.61957
Ciclev10010628m.g	scaffold_1:25871864-25876939	14.3223	4.66583	-1.61806
Ciclev10006262m.g	scaffold_9:17213186-17213665	91.6758	29.8742	-1.61764
Ciclev10024203m.g	scaffold_3:14120653-14121608	6.59218	2.14862	-1.61734
Ciclev10026650m.g	scaffold_7:4149697-4150513	2.52069	0.821636	-1.61725
Ciclev10025903m.g	scaffold_7:1295838-1297378	30.8918	10.0738	-1.61661
Ciclev10002831m.g	scaffold_5:953144-1184934	17.8105	5.80966	-1.6162
Ciclev10020570m.g	scaffold_3:4009157-4010937	15.9142	5.19486	-1.61516
Ciclev10012929m.g	scaffold_6:25301082-25303163	7.77603	2.54354	-1.61219
Ciclev10028241m.g	scaffold_8:23456629-23459052	17.542	5.74318	-1.61089
Ciclev10002955m.g	scaffold_5:17392923-17476446	93.7897	30.7187	-1.61031
Ciclev10013472m.g	scaffold_6:19098190-19099862	0.840899	0.275424	-1.61028
Ciclev10014531m.g	scaffold_2:8727345-8730038	1.79997	0.589653	-1.61003
Ciclev10000968m.g	scaffold_5:40400643-40405657	9.14078	2.99967	-1.60752
Ciclev10014480m.g	scaffold_2:11054066-11057781	0.304142	0.0999718	-1.60515
Ciclev10011415m.g	scaffold_6:24233895-24235536	3.5072	1.15307	-1.60484
Ciclev10033080m.g,Ciclev10033990m.g	scaffold_4:354241-358913	260.436	85.6288	-1.60476
Ciclev10033635m.g	scaffold_4:22641895-22645779	0.563126	0.185188	-1.60447
Ciclev10023538m.g	scaffold_3:23572802-23614239	199.517	65.6207	-1.60429
Ciclev10028692m.g	scaffold_8:1693878-1698099	0.854482	0.281117	-1.60388
Ciclev10022919m.g	scaffold_3:9743613-9744406	6.66335	2.19231	-1.60379
Ciclev10017949m.g,Ciclev10018161m.g	scaffold_2:33721391-33724286	23.5328	7.74358	-1.6036
Ciclev10001191m.g	scaffold_5:13273930-13275652	67.4321	22.2156	-1.60186
Ciclev10017141m.g	scaffold_2:35391011-35392163	427.028	140.694	-1.60177
Ciclev10031330m.g	scaffold_4:21520993-21522723	1.26177	0.41609	-1.60048
Ciclev10012837m.g	scaffold_6:22365432-22367412	243.811	80.419	-1.60015
Ciclev10021094m.g	scaffold_3:37993755-37999062	102.61	33.8464	-1.60009
Ciclev10010348m.g	scaffold_1:25143730-25144390	8.16212	2.69253	-1.59998
Ciclev10006520m.g,Ciclev10006984m.g	scaffold_9:25829097-25832322	24.6203	8.12517	-1.59938

Ciclev10008771m.g	scaffold_1:7916944-7921026	2.57935	0.85148	-1.59896
Ciclev10016810m.g	scaffold_2:32304436-32305384	10.6559	3.51767	-1.59896
Ciclev10024293m.g	scaffold_3:25657663-25663490	0.230893	0.0762621	-1.59818
Ciclev10001565m.g	scaffold_5:33766086-33769182	17.2247	5.68947	-1.59812
Ciclev10021095m.g	scaffold_3:18466383-18467774	204.282	67.5632	-1.59625
Ciclev10030328m.g	scaffold_8:2788251-2799301	18.2577	6.03944	-1.59602
Ciclev10013382m.g	scaffold_6:19046543-19048141	8.21918	2.71944	-1.59569
Ciclev10004372m.g	scaffold_9:25073692-25080376	125.597	41.7009	-1.59065
Ciclev10011092m.g	scaffold_6:13371257-13375494	3.09595	1.02823	-1.59023
Ciclev10004519m.g	scaffold_9:1144640-1147524	11.1211	3.69589	-1.5893
Ciclev10027562m.g	scaffold_7:13471112-13472798	2.97777	0.990849	-1.5875
Ciclev10019563m.g	scaffold_3:42235927-42239879	3.94004	1.31332	-1.58499
Ciclev10031919m.g	scaffold_4:24631410-24633675	9.22886	3.07629	-1.58496
Ciclev10028428m.g	scaffold_8:19611496-19617508	1.37736	0.459265	-1.5845
Ciclev10033215m.g	scaffold_4:23142594-23143167	33.025	11.015	-1.58408
Ciclev10009498m.g	scaffold_1:23445787-23446632	1.20102	0.400616	-1.58396
Ciclev10004245m.g	scaffold_9:4167808-4176655	2.43524	0.812731	-1.58321
Ciclev10009186m.g	scaffold_1:26473623-26476630	18.8737	6.29897	-1.58319
Ciclev10018754m.g	scaffold_3:22787984-22801272	40.6474	13.5713	-1.5826
Ciclev10000628m.g	scaffold_5:18239993-18242476	0.498606	0.166743	-1.58028
Ciclev10029394m.g	scaffold_8:3007025-3009612	60.406	20.2034	-1.5801
Ciclev10000450m.g	scaffold_5:42121458-42127625	16.421	5.49511	-1.57933
Ciclev10033705m.g	scaffold_4:6950627-6951913	47.0207	15.7503	-1.57792
Ciclev10020087m.g	scaffold_3:46479396-46481959	5.67967	1.90267	-1.57778
Ciclev10023653m.g	scaffold_3:22765321-22770646	1.66229	0.557387	-1.57642
Ciclev10010110m.g	scaffold_1:24121895-24122708	16.4638	5.52267	-1.57586
Ciclev10002405m.g	scaffold_5:42530863-42532444	17.0139	5.7075	-1.57578
Ciclev10019848m.g	scaffold_3:9466070-9468445	66.651	22.3676	-1.57522
-	scaffold_5:1311648-1355130	33.3338	11.194	-1.57426
Ciclev10014695m.g	scaffold_2:23040898-23042671	0.524777	0.17627	-1.57392
Ciclev10001412m.g,Ciclev10001422m.g,Ciclev10003925m.g	scaffold_5:23886542-23992055	828.096	278.16	-1.57388
-	scaffold_2:24737092-24836931	127.836	42.9597	-1.57324
Ciclev10032628m.g	scaffold_4:19790478-19792588	4.63152	1.55664	-1.57305
Ciclev10002618m.g	scaffold_5:34191763-34193830	0.863296	0.290153	-1.57304
Ciclev10029557m.g	scaffold_8:16569183-16570832	3.91222	1.31585	-1.57199
-	scaffold_7:2922892-2923402	119.987	40.3657	-1.57168
Ciclev10016428m.g	scaffold_2:33991109-33993524	3.07762	1.03579	-1.57108
Ciclev10031199m.g	scaffold_4:23940022-23941837	5.99191	2.0167	-1.57102
Ciclev10004621m.g	scaffold_9:9799745-9804569	15.9016	5.35708	-1.56965
Ciclev10001646m.g	scaffold_5:36504700-36506386	1.51928	0.512149	-1.56875
Ciclev10023895m.g	scaffold_3:9876900-9882844	0.673198	0.227183	-1.56718
Ciclev10009213m.g	scaffold_1:19056290-19058412	33.5262	11.3156	-1.56697
Ciclev10008725m.g	scaffold_1:22552500-22555558	203.825	68.8198	-1.56643
Ciclev10006845m.g	scaffold_9:15303471-15308386	1.40941	0.476146	-1.56561
Ciclev10012937m.g	scaffold_6:20683218-20684669	82.3604	27.8264	-1.5655
Ciclev10023327m.g	scaffold_3:8857722-8858955	1.4656	0.495895	-1.56338
Ciclev10030650m.g	scaffold_4:13903685-13910631	11.9893	4.05677	-1.56335
Ciclev10008166m.g	scaffold_1:22053023-22055471	2.11967	0.717247	-1.5633
Ciclev10028037m.g	scaffold_8:24920509-24925230	45.3272	15.3413	-1.56295
Ciclev10030977m.g	scaffold_4:16129998-16136902	32.3136	10.9397	-1.56257
Ciclev10029254m.g	scaffold_8:13288298-13289998	27.8539	9.43185	-1.56226
Ciclev10014576m.g	scaffold_2:21386585-21390439	3.92848	1.33041	-1.5621
Ciclev10019943m.g	scaffold_3:49398912-49401345	1.28262	0.434419	-1.56194
Ciclev10026151m.g	scaffold_7:14282400-14286582	16.0646	5.44248	-1.56154

-	scaffold_5:26289212-26291616	43.7779	14.8527	-1.55948
Ciclev10006791m.g	scaffold_9:657420-657930	24.0631	8.16458	-1.55937
Ciclev10011453m.g	scaffold_6:17915203-17919250	1.25834	0.427312	-1.55816
Ciclev10020418m.g	scaffold_3:48506025-48509461	61.2679	20.8113	-1.55776
Ciclev10025125m.g	scaffold_7:8447413-8453714	128.108	43.5435	-1.55683
Ciclev10010836m.g	scaffold_1:28928260-28932927	2.81744	0.957973	-1.55633
Ciclev10000349m.g	scaffold_5:23717648-23726626	15.6873	5.33997	-1.5547
Ciclev10022623m.g	scaffold_3:50207879-50208921	47.2042	16.075	-1.5541
Ciclev10022683m.g	scaffold_3:42460939-42467955	45.4431	15.4828	-1.5534
Ciclev10009711m.g	scaffold_1:28209000-28211161	10.9241	3.72287	-1.55303
Ciclev10013048m.g	scaffold_6:11835445-11837145	44.3623	15.1196	-1.55291
Ciclev10029854m.g	scaffold_8:7909414-7965005	5.78517	1.97739	-1.54877
Ciclev10012752m.g	scaffold_6:25013158-25014095	356.108	121.767	-1.54819
Ciclev10005234m.g	scaffold_9:12572175-12580302	43.4436	14.8626	-1.54746
Ciclev10013191m.g	scaffold_6:22970177-22976234	14.7701	5.05635	-1.54651
Ciclev10012108m.g	scaffold_6:19634062-19638194	9.66152	3.31123	-1.54488
Ciclev10026898m.g	scaffold_7:17491173-17497237	0.98599	0.338177	-1.5438
Ciclev10024981m.g	scaffold_7:19562244-19564547	0.398425	0.136702	-1.54327
Ciclev10014971m.g	scaffold_2:8906363-8909753	2.38392	0.817957	-1.54324
Ciclev10026347m.g,Ciclev10026731m.g,Ciclev10026918m.g	scaffold_7:11203966-11208816	381.573	131.082	-1.54149
Ciclev10023206m.g	scaffold_3:7976669-7989624	3.15808	1.08491	-1.54148
-	scaffold_5:1552172-1984270	7.90893	2.71827	-1.54079
Ciclev10007470m.g	scaffold_1:26802786-26810465	8.30358	2.85409	-1.5407
Ciclev10016102m.g	scaffold_2:14178448-14179345	1.18586	0.407667	-1.54048
-	scaffold_2:13418751-13418927	720.07	247.55	-1.54042
Ciclev10007490m.g	scaffold_1:19589031-19593020	6.46123	2.223	-1.5393
Ciclev10015096m.g	scaffold_2:21398468-21400348	6.89251	2.3729	-1.53838
Ciclev10013266m.g	scaffold_6:3804583-3806162	6.44676	2.22142	-1.53709
Ciclev10006433m.g	scaffold_9:5749393-5751226	2.53933	0.87525	-1.53668
-	scaffold_1:25681618-25682215	13.5082	4.65732	-1.53627
Ciclev10016142m.g	scaffold_2:12387212-12388669	23.6223	8.15003	-1.53527
-	scaffold_5:25221155-25545745	23.2758	8.03265	-1.53488
Ciclev10013185m.g	scaffold_6:17638205-17638519	3.34392	1.15409	-1.53478
Ciclev10028062m.g	scaffold_8:6922347-6926626	44.5736	15.393	-1.53392
Ciclev10004942m.g	scaffold_9:1320159-1323922	11.6139	4.01105	-1.5338
Ciclev10020812m.g	scaffold_3:17578350-17580588	4.96372	1.71545	-1.53283
Ciclev10026628m.g	scaffold_7:5135979-5139197	192.453	66.5177	-1.5327
Ciclev10011959m.g	scaffold_6:20767620-20771052	15.0492	5.20991	-1.53035
Ciclev10011310m.g	scaffold_6:21794804-21797934	2.15416	0.746838	-1.52826
Ciclev10025354m.g	scaffold_7:1739442-1743149	3.66951	1.2729	-1.52747
Ciclev10033777m.g	scaffold_4:21719885-21721178	1.59074	0.551884	-1.52726
Ciclev10009242m.g	scaffold_1:1430454-1434069	25.0048	8.67648	-1.52702
Ciclev10012515m.g	scaffold_6:25125981-25128114	3.98271	1.38279	-1.52617
Ciclev10019749m.g	scaffold_3:47737983-47739878	83.1671	28.8875	-1.52557
Ciclev10015536m.g	scaffold_2:35737943-35740687	17.9678	6.24597	-1.52442
Ciclev10003731m.g	scaffold_5:7258872-7261994	1.70381	0.593353	-1.5218
Ciclev10008315m.g	scaffold_1:28623623-28626008	104.778	36.5278	-1.52027
Ciclev10001225m.g	scaffold_5:25722021-25731001	4.53234	1.58101	-1.51941
Ciclev10019431m.g	scaffold_3:22301511-22304667	3.61039	1.25986	-1.51889
Ciclev10013224m.g	scaffold_6:11107416-11108496	9.79968	3.42086	-1.51838
Ciclev10013160m.g	scaffold_6:10448884-10449799	5.781	2.02059	-1.51654
Ciclev10032373m.g	scaffold_4:21329512-21333529	5.02717	1.75813	-1.51571
Ciclev10002182m.g	scaffold_5:36068744-36070294	2.35875	0.825754	-1.51424
Ciclev10001371m.g	scaffold_5:29050806-29055963	87.3942	30.6089	-1.51359

Ciclev10018535m.g	scaffold_3:2404291-2411827	5.195	1.82005	-1.51314
Ciclev10005818m.g	scaffold_9:20182668-20185445	1.55693	0.545586	-1.51283
Ciclev10024636m.g	scaffold_3:33722933-33727855	2.68256	0.940297	-1.51242
Ciclev10015040m.g	scaffold_2:29449881-29459747	6.45441	2.26308	-1.512
Ciclev10004284m.g	scaffold_9:24356661-24541601	0.333584	0.116984	-1.51174
Ciclev10031761m.g	scaffold_4:22166699-22169904	39.8573	13.9889	-1.51056
Ciclev10003021m.g	scaffold_5:37336194-37341294	13.6202	4.78282	-1.50982
Ciclev10018693m.g	scaffold_3:9893235-9898231	7.84694	2.75949	-1.50773
Ciclev10008639m.g	scaffold_1:5043066-5044278	1.18755	0.417815	-1.50705
Ciclev10032787m.g	scaffold_4:9880494-10159290	1.79559	0.631915	-1.50665
Ciclev10031237m.g	scaffold_4:20824179-20826164	11.752	4.1376	-1.50604
Ciclev10005992m.g	scaffold_9:5417912-5418877	12.0312	4.23627	-1.50591
Ciclev10010001m.g,Ciclev10010501m.g	scaffold_1:312354-326006	80.7953	28.4667	-1.50499
Ciclev10022593m.g	scaffold_3:13807410-13808572	3.30566	1.16486	-1.50478
Ciclev10027048m.g	scaffold_7:13916806-14069972	8.56536	3.0183	-1.50478
Ciclev10024202m.g	scaffold_3:43154659-43156863	0.881729	0.310793	-1.50438
-	scaffold_9:26501629-26502328	3.78015	1.33372	-1.50299
Ciclev10008846m.g	scaffold_1:16654708-16656876	61.3099	21.6372	-1.5026
Ciclev10012153m.g	scaffold_6:21554199-21556921	3.17237	1.12017	-1.50185
Ciclev10012611m.g	scaffold_6:25033675-25035365	2.36796	0.836256	-1.50163
Ciclev10011960m.g	scaffold_6:12664435-12665581	1.44394	0.510062	-1.50127
Ciclev10026658m.g	scaffold_7:1938074-1939314	20.7877	7.35862	-1.49822
Ciclev10027474m.g	scaffold_7:921137-921932	44.1238	15.6298	-1.49726
Ciclev10024534m.g	scaffold_3:16912543-16917880	6.55134	2.32143	-1.49678
Ciclev10019438m.g	scaffold_3:47990694-47993786	7.47175	2.64894	-1.49603
Ciclev10027006m.g	scaffold_7:4850477-4851233	56.9663	20.1978	-1.49591
Ciclev10008652m.g	scaffold_1:24583051-24584498	1.67637	0.594501	-1.49559
Ciclev10019936m.g	scaffold_3:47876447-47878633	38.4718	13.6438	-1.49556
Ciclev10029914m.g,Ciclev10029983m.g,Ciclev10030279m.g	scaffold_8:6434299-6453817	11.2418	3.98742	-1.49535
Ciclev10011650m.g	scaffold_6:17402876-17405928	257.354	91.2954	-1.49514
Ciclev10023204m.g	scaffold_3:11687448-12154880	65.1796	23.1243	-1.49501
Ciclev10014972m.g	scaffold_2:33319563-33323869	18.4928	6.56397	-1.49432
Ciclev10020848m.g	scaffold_3:2218488-2221303	1.16977	0.415283	-1.49406
Ciclev10005436m.g	scaffold_9:22368666-22373982	66.0874	23.4726	-1.4934
Ciclev10009804m.g	scaffold_1:5413232-5416822	2440.82	867.083	-1.49312
Ciclev10000939m.g	scaffold_5:41895512-41898880	2.83799	1.00851	-1.49265
Ciclev10009875m.g	scaffold_1:1872342-1874936	0.241299	0.0858053	-1.49168
Ciclev10020968m.g	scaffold_3:22870195-22871393	5.95902	2.11937	-1.49144
Ciclev10004632m.g	scaffold_9:29476303-29481526	2.06952	0.73608	-1.49136
Ciclev10005689m.g	scaffold_9:5070631-5073207	1.42118	0.505609	-1.49099
Ciclev10018553m.g	scaffold_3:19816530-19821783	0.594574	0.211556	-1.49081
Ciclev10021357m.g	scaffold_3:8457314-8464161	3.14817	1.1208	-1.48998
Ciclev10005629m.g	scaffold_9:5544553-5546110	14.9039	5.30637	-1.48989
Ciclev10024674m.g	scaffold_3:36924550-37054826	7.65777	2.72751	-1.48934
Ciclev10010559m.g	scaffold_1:18592411-18593458	0.377972	0.134639	-1.48918
Ciclev10010500m.g	scaffold_1:26725787-26727306	69.4823	24.7663	-1.48827
Ciclev10012823m.g	scaffold_6:17111895-17113568	2.96779	1.05793	-1.48815
Ciclev10014658m.g	scaffold_2:29506074-29516708	20.5569	7.33247	-1.48725
Ciclev10028633m.g	scaffold_8:7890298-7895996	7.7653	2.76997	-1.48717
Ciclev10025483m.g	scaffold_7:4275021-4278979	2.53744	0.905487	-1.48661
Ciclev10012250m.g	scaffold_6:15275664-15280221	200.05	71.4054	-1.48625
Ciclev10029398m.g	scaffold_8:24850287-24851105	17.5133	6.25308	-1.48581
Ciclev10027541m.g	scaffold_7:13630863-13631265	2.26221	0.80872	-1.48402

Ciclev10009710m.g	scaffold_1:20063995-20065196	18.6679	6.67381	-1.48397
Ciclev10019544m.g	scaffold_3:42144665-42148483	2.05692	0.735375	-1.48393
Ciclev10019922m.g	scaffold_3:41270984-41274884	14.4946	5.18324	-1.48358
Ciclev10009245m.g	scaffold_1:1727829-1729315	1.93427	0.691854	-1.48325
Ciclev10033298m.g	scaffold_4:6087502-6090753	0.648137	0.231858	-1.48306
Ciclev10016971m.g	scaffold_2:11064376-11065069	2.06525	0.739143	-1.48239
Ciclev10025685m.g	scaffold_7:20624956-20628592	3.77245	1.35118	-1.48129
Ciclev10021781m.g	scaffold_3:50061025-50063698	3.48041	1.2474	-1.48033
Ciclev10008075m.g	scaffold_1:23326836-23328814	24.3752	8.73656	-1.48027
Ciclev10002391m.g	scaffold_5:40812421-40815992	30.6745	10.996	-1.48006
Ciclev10017910m.g	scaffold_2:29389570-29390242	1.55094	0.556017	-1.47994
-	scaffold_2:35637306-35637982	21.273	7.62975	-1.47932
Ciclev10013732m.g	scaffold_6:2542835-2543207	1.48689	0.533724	-1.47813
Ciclev10004026m.g	scaffold_5:3610209-3610560	3.29374	1.18268	-1.47767
-	scaffold_5:35145671-35146744	7.74412	2.78084	-1.47758
Ciclev10019492m.g	scaffold_3:4954029-4959276	4.66913	1.6773	-1.47701
Ciclev10012157m.g	scaffold_6:12236942-12239128	2.65219	0.953952	-1.47519
Ciclev10013294m.g	scaffold_6:22160125-22162288	1.99263	0.717404	-1.47382
Ciclev10026107m.g	scaffold_7:3674155-3676384	185.921	66.9491	-1.47355
Ciclev10023233m.g	scaffold_3:46380263-46381057	7.41221	2.67082	-1.47262
Ciclev10004263m.g	scaffold_9:17340139-17344170	5.05377	1.8233	-1.47081
-	scaffold_2:27905813-27906021	540.682	195.328	-1.46889
Ciclev10012433m.g	scaffold_6:14588998-14590935	16.0414	5.79529	-1.46885
Ciclev10005876m.g	scaffold_9:735924-736731	0.877627	0.317198	-1.46823
Ciclev10031929m.g	scaffold_4:6955111-6956891	4.06369	1.46916	-1.4678
Ciclev10005995m.g	scaffold_9:75347-78181	29.6327	10.728	-1.46581
Ciclev10012553m.g	scaffold_6:5109625-5111097	406.648	147.272	-1.4653
Ciclev10015725m.g	scaffold_2:34966175-34971756	16.5688	6.00176	-1.46501
Ciclev10026638m.g	scaffold_7:532899-534454	0.747604	0.271013	-1.46391
Ciclev10012905m.g	scaffold_6:12840407-12842940	8.22648	2.98253	-1.46374
Ciclev10027967m.g	scaffold_8:4156439-4161350	8.48237	3.0769	-1.46299
Ciclev10032642m.g	scaffold_4:2229954-2231280	139.903	50.761	-1.46263
-	scaffold_1:21476759-21547573	66.0146	23.9729	-1.46138
Ciclev10026028m.g	scaffold_7:6011522-6014015	78.4479	28.4933	-1.46111
Ciclev10016218m.g	scaffold_2:19918573-19923588	14.6954	5.33957	-1.46057
Ciclev10033629m.g	scaffold_4:21710739-21711192	1.0046	0.365177	-1.45995
Ciclev10017582m.g	scaffold_2:33509943-33510787	1.26407	0.459555	-1.45977
Ciclev10013186m.g	scaffold_6:1030935-1033563	9.11348	3.31694	-1.45815
Ciclev10032441m.g	scaffold_4:18997584-18999613	58.6516	21.3491	-1.45799
-	scaffold_5:30702403-30702738	27.4192	9.98097	-1.45794
Ciclev10008862m.g	scaffold_1:23759729-23762184	13.0152	4.73937	-1.45743
-	scaffold_4:11523554-11728349	98.5331	35.9056	-1.4564
Ciclev10000542m.g	scaffold_5:35350023-35354136	0.718542	0.26192	-1.45594
Ciclev10021062m.g	scaffold_3:50433985-50437744	4.9086	1.79237	-1.45344
Ciclev10019677m.g	scaffold_3:36363008-36553812	3.05239	1.11511	-1.45275
Ciclev10032017m.g	scaffold_4:22050292-22051395	0.916122	0.334713	-1.45261
Ciclev10010857m.g	scaffold_1:28303017-28323314	181.296	66.2532	-1.45229
Ciclev10018672m.g	scaffold_3:49116717-49121222	63.6331	23.2557	-1.45219
Ciclev10002349m.g	scaffold_5:42489683-42491501	95.9306	35.0626	-1.45206
Ciclev10008122m.g	scaffold_1:4775563-4777305	3.04145	1.11296	-1.45036
Ciclev10030611m.g	scaffold_4:2484696-2491143	20.1547	7.38775	-1.44791
Ciclev10010745m.g	scaffold_1:3921707-3938807	121.237	44.4822	-1.44653
Ciclev10023096m.g	scaffold_3:1566438-1567256	5.60882	2.05814	-1.44636
Ciclev10015600m.g	scaffold_2:29069901-29071600	75.2755	27.6328	-1.4458
Ciclev10009585m.g	scaffold_1:22732080-22732696	4.55211	1.67127	-1.44559
Ciclev10006027m.g	scaffold_9:14114993-14116304	4.69594	1.7241	-1.44557

Ciclev10021966m.g,Ciclev10022574m.g	scaffold_3:32716415-32805364	14.16	5.19946	-1.44539
Ciclev10005186m.g	scaffold_9:1556090-1558575	10.3006	3.78256	-1.4453
Ciclev10031862m.g,Ciclev10033793m.g	scaffold_4:10934752-10938522	5.40258	1.98513	-1.44442
Ciclev10015468m.g	scaffold_2:29113360-29116904	106.404	39.1327	-1.44311
Ciclev10027298m.g	scaffold_7:12216951-12219033	0.581596	0.214185	-1.44116
Ciclev10006569m.g	scaffold_9:7804322-8050719	1.84391	0.679671	-1.43986
Ciclev10003326m.g	scaffold_5:26086885-26089960	0.471937	0.174058	-1.43903
-	scaffold_8:6944685-7159404	50.9533	18.7942	-1.43889
Ciclev10032490m.g	scaffold_4:20514641-20520202	57.2452	21.1213	-1.43846
Ciclev10025750m.g	scaffold_7:17099556-17100932	2.36221	0.871618	-1.43837
Ciclev10002961m.g	scaffold_5:35354185-35354887	36.6203	13.5144	-1.43815
Ciclev10024886m.g	scaffold_7:6510941-6517380	11.4705	4.23395	-1.43786
Ciclev10029644m.g	scaffold_8:21110363-21111473	158.971	58.6824	-1.43776
Ciclev10016114m.g	scaffold_2:34048018-34051182	7.51286	2.77376	-1.43752
Ciclev10013843m.g	scaffold_6:20300056-20302392	128.049	47.2886	-1.43713
Ciclev10033583m.g	scaffold_4:7532854-7706546	1.56921	0.579724	-1.4366
Ciclev10008690m.g	scaffold_1:3266177-3267636	0.275587	0.101829	-1.43636
-	scaffold_7:11611054-11611298	76.2131	28.1679	-1.43599
Ciclev10019173m.g	scaffold_3:19253239-19255491	5.56143	2.05602	-1.4356
Ciclev10010535m.g	scaffold_1:25259971-25260178	282.659	104.531	-1.43514
Ciclev10009089m.g	scaffold_1:23078359-23080007	25.0638	9.27141	-1.43474
Ciclev10016407m.g	scaffold_2:10569900-10571074	17.5783	6.51119	-1.4328
Ciclev10029664m.g	scaffold_8:5937884-5938541	34.0649	12.6345	-1.43092
Ciclev10005111m.g	scaffold_9:27259812-27263097	15.2407	5.65305	-1.43083
Ciclev10030927m.g	scaffold_4:23426700-23429072	5.67213	2.10417	-1.43064
Ciclev10031789m.g	scaffold_4:1297726-1299898	21.774	8.07977	-1.43022
-	scaffold_5:28289811-28536377	10.6847	3.96582	-1.42985
Ciclev10011957m.g	scaffold_6:20691106-20706907	22.4519	8.33402	-1.42975
Ciclev10009911m.g,Ciclev10009912m.g	scaffold_1:22775176-22783365	41.1934	15.2922	-1.42962
Ciclev10005557m.g	scaffold_9:28665073-28667783	17.178	6.37758	-1.42948
Ciclev10022548m.g	scaffold_3:33172473-33173133	0.614927	0.228321	-1.42935
Ciclev10007787m.g	scaffold_1:13579582-13583749	2.52135	0.936274	-1.4292
Ciclev10023610m.g	scaffold_3:42906090-42915510	0.926537	0.344237	-1.42845
Ciclev10016664m.g	scaffold_2:25672324-25673455	3.29152	1.22305	-1.42827
Ciclev10033238m.g	scaffold_4:1788017-1789949	26.5309	9.86411	-1.42741
Ciclev10032594m.g	scaffold_4:18687282-18688254	0.795726	0.295904	-1.42714
Ciclev10025988m.g	scaffold_7:8712252-8715371	86.8703	32.3067	-1.42703
Ciclev10009649m.g	scaffold_1:982761-983693	0.514934	0.191512	-1.42695
Ciclev10033127m.g	scaffold_4:23117904-23118901	2.51997	0.937405	-1.42666
Ciclev10012636m.g	scaffold_6:17945664-17947610	20.7823	7.73512	-1.42586
Ciclev10001048m.g	scaffold_5:39963242-39965562	6.49056	2.41662	-1.42535
Ciclev10031558m.g	scaffold_4:17682884-17686035	26.1188	9.72958	-1.42464
Ciclev10014736m.g	scaffold_2:23045427-23048317	3.94262	1.46901	-1.42431
Ciclev10031956m.g	scaffold_4:17499129-17500420	7.94523	2.96078	-1.42411
Ciclev10011060m.g	scaffold_6:13305252-13310801	4.84123	1.80453	-1.42375
Ciclev10031989m.g	scaffold_4:12678670-12681681	59.69	22.2541	-1.42342
Ciclev10026444m.g	scaffold_7:9233534-9235297	9.24143	3.44697	-1.42279
Ciclev10022540m.g	scaffold_3:49731177-49731699	1.41317	0.527337	-1.42214
Ciclev10017131m.g	scaffold_2:5957911-5958497	5.28247	1.97171	-1.42176
Ciclev10020517m.g	scaffold_3:10404108-10407777	30.273	11.3086	-1.42061
-	scaffold_2:18800034-18801893	76.9694	28.7774	-1.41935
Ciclev10026105m.g	scaffold_7:14560603-14562711	78.0073	29.176	-1.41883
Ciclev10012879m.g	scaffold_6:22242908-22244665	13.4888	5.0482	-1.41792

Ciclev10013071m.g	scaffold_6:22915615-22916253	41.5571	15.5599	-1.41726
Ciclev10000759m.g	scaffold_5:33894993-33900548	18.232	6.82947	-1.41663
Ciclev10027716m.g	scaffold_8:6255899-6268429	4.81927	1.80566	-1.41629
Ciclev10017228m.g	scaffold_2:9835137-9838004	58.5752	21.9476	-1.41623
Ciclev10016754m.g	scaffold_2:35994367-35996314	56.5962	21.2066	-1.4162
Ciclev10010850m.g	scaffold_1:22983079-22983903	2.46086	0.922161	-1.41607
Ciclev10029638m.g	scaffold_8:24355053-24355871	37.0723	13.9042	-1.41482
Ciclev10017072m.g	scaffold_2:35983386-35984186	5.7218	2.14613	-1.41473
-	scaffold_3:9784877-9785220	52.8626	19.8317	-1.41444
Ciclev10001888m.g	scaffold_5:36449345-36452178	41.1673	15.4607	-1.41289
Ciclev10004951m.g	scaffold_9:4460615-4462312	150.89	56.6986	-1.41211
Ciclev10026724m.g	scaffold_7:15065828-15067505	0.902307	0.339488	-1.41026
Ciclev10010271m.g	scaffold_1:26477936-26480488	0.265869	0.10008	-1.40956
Ciclev10028375m.g	scaffold_8:24832652-24835113	2.33533	0.879368	-1.40909
-	scaffold_5:38857420-38857798	35.6926	13.4477	-1.40826
Ciclev10008929m.g	scaffold_1:592652-595889	4.36136	1.6455	-1.40625
Ciclev10027484m.g	scaffold_7:778697-781029	1.2211	0.461051	-1.40519
Ciclev10019012m.g	scaffold_3:12742965-12746792	4.29291	1.62144	-1.40468
Ciclev10015036m.g	scaffold_2:31706112-31709106	4.02312	1.51957	-1.40466
Ciclev10028137m.g	scaffold_8:24152579-24156447	0.56255	0.212494	-1.40456
Ciclev10022397m.g	scaffold_3:7311581-7314699	23.2122	8.77074	-1.40411
Ciclev10021043m.g	scaffold_3:43316045-43317909	8.343	3.15297	-1.40385
Ciclev10012121m.g	scaffold_6:13586707-13589828	5.6908	2.15128	-1.40344
Ciclev10030953m.g	scaffold_4:25053029-25057026	1.36154	0.514927	-1.4028
Ciclev10020512m.g	scaffold_3:43950064-43952660	95.6556	36.1781	-1.40273
Ciclev10012712m.g	scaffold_6:3654838-3656896	113.595	42.9688	-1.40254
Ciclev10029803m.g	scaffold_8:23429878-23430542	55.1106	20.8517	-1.40217
Ciclev10033189m.g	scaffold_4:22194797-22195651	71.5787	27.086	-1.40198
Ciclev10031490m.g	scaffold_4:21716795-21718205	14.3199	5.4188	-1.40197
Ciclev10024525m.g	scaffold_3:22335208-22336518	11.792	4.46962	-1.39959
Ciclev10015618m.g	scaffold_2:23337227-23339301	0.302866	0.114823	-1.39927
Ciclev10001187m.g	scaffold_5:42265152-42269685	131.74	49.9838	-1.39816
Ciclev10000487m.g	scaffold_5:25805803-25815252	0.671359	0.254838	-1.39751
-	scaffold_7:97880-100644	7.95591	3.02225	-1.3964
Ciclev10011662m.g	scaffold_6:23818782-23821639	1.03343	0.392591	-1.39634
Ciclev10023485m.g	scaffold_3:33388539-33592383	1.21744	0.462825	-1.39532
Ciclev10022925m.g	scaffold_3:2774464-2775592	0.579485	0.22032	-1.39517
Ciclev10030938m.g	scaffold_4:22097089-22102186	20.7556	7.89177	-1.39508
Ciclev10005750m.g	scaffold_9:26053929-26055086	165.367	62.8859	-1.39486
Ciclev10017219m.g	scaffold_2:17347941-17351501	2.27858	0.86694	-1.39413
Ciclev10024643m.g	scaffold_3:35714601-35715279	1.42069	0.540665	-1.39379
Ciclev10010758m.g	scaffold_1:21668275-21668959	2.05587	0.78258	-1.39344
Ciclev10022920m.g	scaffold_3:39483386-39486219	8.41369	3.20342	-1.39312
Ciclev10004868m.g	scaffold_9:17114164-17116036	7.52524	2.86573	-1.39284
Ciclev10006575m.g	scaffold_9:30852158-30856221	0.72441	0.275888	-1.39272
Ciclev10010038m.g	scaffold_1:6240403-6241344	16.8477	6.41904	-1.39212
Ciclev10004597m.g	scaffold_9:31171508-31199360	57.5304	21.929	-1.39149
Ciclev10002811m.g	scaffold_5:40460686-40461620	0.532165	0.202893	-1.39115
Ciclev10023183m.g	scaffold_3:25085744-25086451	231.596	88.3199	-1.3908
Ciclev10013655m.g,Ciclev10013676m.g	scaffold_6:6314337-6318606	2440.65	932.201	-1.38855
Ciclev10014811m.g	scaffold_2:35970576-35973973	120.954	46.2039	-1.38837
Ciclev10016585m.g	scaffold_2:34804822-34810333	760.848	290.645	-1.38835
Ciclev10017424m.g	scaffold_2:15795260-15800378	5.60251	2.14395	-1.3858
Ciclev10025401m.g	scaffold_7:3560793-3565200	26.3636	10.0977	-1.38452
Ciclev10011034m.g	scaffold_6:17744578-17747968	70.6022	27.0564	-1.38374

Ciclev10010003m.g	scaffold_1:26490582-26491432	0.878301	0.337204	-1.3811
Ciclev10015068m.g	scaffold_2:27279579-27284866	31.5305	12.112	-1.3803
Ciclev10003678m.g	scaffold_5:37597365-37598384	0.604214	0.232121	-1.38018
Ciclev10022544m.g	scaffold_3:7643694-7644822	0.584159	0.224434	-1.38007
Ciclev10022303m.g	scaffold_3:5404054-5405090	6.02797	2.31619	-1.37992
Ciclev10006399m.g	scaffold_9:556724-557601	79.3984	30.5166	-1.37952
-	scaffold_6:17647132-17647303	763.358	293.65	-1.37826
-	scaffold_5:2761727-2762568	11.9483	4.59948	-1.37726
-	scaffold_5:2534144-2731098	8.0065	3.0831	-1.37679
Ciclev10025342m.g	scaffold_7:5611185-5614200	2.37075	0.913454	-1.37594
Ciclev10019546m.g	scaffold_3:8431511-8435173	4.32185	1.66524	-1.37592
Ciclev10003859m.g	scaffold_5:37131590-37133048	0.751523	0.289571	-1.3759
Ciclev10011950m.g	scaffold_6:24853105-24855255	9.1947	3.543	-1.37583
Ciclev10026307m.g,Ciclev10027442m.g	scaffold_7:2721714-2817730	4.55994	1.75757	-1.37543
Ciclev10001976m.g	scaffold_5:37586833-37589255	849.159	327.635	-1.37394
Ciclev10021862m.g	scaffold_3:12637866-12639182	13.0739	5.04702	-1.37318
Ciclev10001122m.g	scaffold_5:15762363-15766976	34.3402	13.2677	-1.37198
Ciclev10017107m.g	scaffold_2:11373564-11374539	1.81363	0.700797	-1.37181
Ciclev10003698m.g	scaffold_5:33866523-33867243	1.10652	0.427584	-1.37176
Ciclev10020276m.g	scaffold_3:42109588-42112025	21.3259	8.24308	-1.37135
Ciclev10001638m.g	scaffold_5:24467954-24473569	1.33915	0.517655	-1.37125
Ciclev10019408m.g	scaffold_3:41857257-41859322	10.1331	3.91892	-1.37055
Ciclev10026017m.g	scaffold_7:8647365-8650863	44.067	17.0466	-1.37021
Ciclev10023267m.g	scaffold_3:46707830-46708427	1.01148	0.391343	-1.36996
Ciclev10008171m.g	scaffold_1:3746177-3749903	47.0063	18.1903	-1.36968
Ciclev10021670m.g	scaffold_3:39983813-39986022	4.237	1.64049	-1.36891
Ciclev10030950m.g	scaffold_4:24212535-24217804	5.38458	2.08573	-1.36828
Ciclev10029512m.g	scaffold_8:4117462-4118362	9.61718	3.72752	-1.3674
Ciclev10000097m.g	scaffold_5:41591990-41602791	4.89814	1.90135	-1.36521
Ciclev10018603m.g	scaffold_3:40475280-40479296	0.609773	0.236971	-1.36356
Ciclev10011910m.g	scaffold_6:22136738-22139832	22.2967	8.66935	-1.36283
Ciclev10031068m.g	scaffold_4:22726583-22728688	0.203502	0.0791592	-1.36221
Ciclev10031917m.g	scaffold_4:22882713-22885472	6.81839	2.6524	-1.36213
Ciclev10008268m.g	scaffold_1:2231395-2233831	43.6715	16.9923	-1.36181
Ciclev10004977m.g	scaffold_9:29246789-29250126	6.866	2.67255	-1.36125
Ciclev10010621m.g	scaffold_1:3597177-3598566	0.779823	0.303607	-1.36094
Ciclev10032749m.g	scaffold_4:2173833-2175353	60.8443	23.6963	-1.36046
Ciclev10000709m.g	scaffold_5:41394567-41399755	9.65288	3.75943	-1.36045
Ciclev10029846m.g	scaffold_8:20299753-20300672	0.561444	0.218661	-1.36044
Ciclev10025585m.g	scaffold_7:4161617-4163419	6.48163	2.52537	-1.35986
Ciclev10021132m.g	scaffold_3:50463538-50464855	1.60667	0.626425	-1.35886
Ciclev10006993m.g	scaffold_9:4085474-4087228	1553.02	605.628	-1.35857
-	scaffold_2:18462754-18462996	45.7502	17.8509	-1.35778
Ciclev10015386m.g	scaffold_2:11306353-11307978	26.7858	10.4571	-1.35699
Ciclev10004816m.g	scaffold_9:25313692-25319614	1.12866	0.440688	-1.35678
Ciclev10011672m.g	scaffold_6:10520679-10522783	35.8015	13.987	-1.35593
Ciclev10003627m.g	scaffold_5:2534144-2731098	45.5361	17.7938	-1.35564
-	scaffold_3:8937950-8938274	15.0038	5.86522	-1.35507
Ciclev10017022m.g	scaffold_2:33700448-33702556	16.5399	6.47011	-1.35409
Ciclev10032822m.g	scaffold_4:2772549-2773801	12.392	4.84762	-1.35406
Ciclev10017241m.g	scaffold_2:9720272-9721084	3.91258	1.53098	-1.35366
Ciclev10022618m.g	scaffold_3:49633249-49635677	32.9729	12.9091	-1.35289
Ciclev10013026m.g	scaffold_6:22728225-22728909	5.63453	2.20602	-1.35285
Ciclev10011731m.g	scaffold_6:23121560-23126186	12.3008	4.81683	-1.3526
Ciclev10031336m.g	scaffold_4:608211-615318	37.3613	14.647	-1.35094

Ciclev10000926m.g	scaffold_5:34382123-34384067	2.70211	1.06074	-1.34902
Ciclev10019483m.g	scaffold_3:5530363-5535891	3.80355	1.49323	-1.3489
Ciclev10006331m.g	scaffold_9:29180966-29185849	0.428806	0.168353	-1.34883
Ciclev10003778m.g	scaffold_5:28857882-28859238	0.68949	0.270797	-1.34832
Ciclev10020225m.g	scaffold_3:47162247-47165561	7.64293	3.00271	-1.34786
Ciclev10000465m.g	scaffold_5:35598097-35604350	57.4355	22.5765	-1.34712
Ciclev10026683m.g	scaffold_7:7727634-7729580	6.15385	2.41909	-1.34702
Ciclev10033341m.g	scaffold_4:2686796-2688071	0.271375	0.106698	-1.34676
-	scaffold_5:3980660-4069314	173.084	68.0734	-1.34631
Ciclev10000919m.g	scaffold_5:42339174-42344676	20.7315	8.16148	-1.34492
-	scaffold_8:21423135-21423354	67.6128	26.6282	-1.34434
Ciclev10011217m.g	scaffold_6:17453268-17455623	1.36844	0.53941	-1.34308
Ciclev10017417m.g	scaffold_2:26182548-26186470	1.83503	0.723429	-1.34288
Ciclev10031301m.g	scaffold_4:14385106-14389080	33.1283	13.0654	-1.34231
Ciclev10032834m.g	scaffold_4:18320631-18323702	1.19994	0.473423	-1.34176
Ciclev10016646m.g	scaffold_2:23073190-23078049	16.9879	6.70361	-1.3415
Ciclev10014891m.g	scaffold_2:15781604-15786427	1.38593	0.547123	-1.34092
Ciclev10026873m.g	scaffold_7:16675946-16676725	1.04372	0.412081	-1.34074
Ciclev10032323m.g	scaffold_4:23897863-23901686	18.5956	7.34241	-1.34064
Ciclev10021859m.g	scaffold_3:16348511-16350656	0.758791	0.299743	-1.33998
Ciclev10024766m.g	scaffold_7:17630699-17638750	21.012	8.30562	-1.33905
Ciclev10012623m.g	scaffold_6:19948155-19948958	15.5734	6.15646	-1.33892
Ciclev10030903m.g	scaffold_4:18393185-18399566	57.1256	22.6118	-1.33706
Ciclev10028494m.g	scaffold_8:5153369-5156195	20.4155	8.08383	-1.33656
Ciclev10017360m.g	scaffold_2:10640189-10646119	11.9571	4.73523	-1.33636
Ciclev10020837m.g	scaffold_3:6811844-6815073	23.1658	9.17537	-1.33616
Ciclev10033949m.g	scaffold_4:14948406-14950036	0.265939	0.105337	-1.33608
Ciclev10012678m.g	scaffold_6:19964328-19965315	70.5232	27.9481	-1.33535
-	scaffold_4:8706377-8707320	3.65189	1.44769	-1.33489
Ciclev10013384m.g	scaffold_6:23635469-23637613	0.603454	0.239229	-1.33485
Ciclev10028372m.g	scaffold_8:4903915-4909930	50.6154	20.0747	-1.3342
Ciclev10018269m.g	scaffold_2:25520323-25523768	0.361648	0.143495	-1.33359
Ciclev10031681m.g	scaffold_4:14681256-14684932	7.42963	2.94799	-1.33356
Ciclev10000764m.g	scaffold_5:42516005-42519605	70.6169	28.0232	-1.33339
Ciclev10014837m.g	scaffold_2:36183108-36186773	0.367807	0.146074	-1.33225
Ciclev10026059m.g	scaffold_7:486047-489142	9.74263	3.86938	-1.33221
Ciclev10007064m.g	scaffold_9:9937337-9940309	34.9828	13.8987	-1.3317
Ciclev10023850m.g	scaffold_3:49490106-49492764	0.538929	0.214131	-1.3316
Ciclev10033745m.g	scaffold_4:14537928-14540007	0.30645	0.121797	-1.33118
Ciclev10029376m.g	scaffold_8:18110127-18110971	1.21759	0.48403	-1.33085
Ciclev10033684m.g	scaffold_4:20713679-20716067	0.314804	0.125202	-1.3302
Ciclev10031241m.g	scaffold_4:17518784-17523468	102.531	40.7844	-1.32997
Ciclev10005065m.g	scaffold_9:21593472-21597037	2.16679	0.862214	-1.32944
Ciclev10009511m.g	scaffold_1:24724344-24725778	2.04681	0.814855	-1.32876
Ciclev10014684m.g	scaffold_2:13425109-13428805	9.01359	3.58951	-1.32832
Ciclev10029794m.g	scaffold_8:22160568-22161832	0.289299	0.115245	-1.32785
Ciclev10002738m.g	scaffold_5:41285150-41288221	1.79522	0.715795	-1.32654
Ciclev10031633m.g	scaffold_4:22511997-22515961	31.7867	12.6775	-1.32615
Ciclev10025912m.g	scaffold_7:2832674-2834027	1.46879	0.585812	-1.32612
Ciclev10023705m.g	scaffold_3:6418885-6419353	12.0604	4.81423	-1.3249
Ciclev10009499m.g	scaffold_1:28805244-28807260	39.8878	15.924	-1.32475
-	scaffold_5:641270-642358	7.99138	3.19046	-1.32468
Ciclev10012845m.g	scaffold_6:22805831-22806783	8.1082	3.23785	-1.32434
Ciclev10009229m.g	scaffold_1:17879965-17882892	23.2687	9.29567	-1.32376
Ciclev10009288m.g	scaffold_1:24870546-24873114	0.449621	0.179865	-1.32179
Ciclev10007906m.g	scaffold_1:28132429-28135867	60.3363	24.1511	-1.32094

Ciclev10007809m.g	scaffold_1:16203906-16208234	108.493	43.4572	-1.31994
Ciclev10012620m.g	scaffold_6:16563164-16563910	7.74812	3.10512	-1.3192
Ciclev10006362m.g	scaffold_9:11887965-11889146	107.687	43.1684	-1.31879
-	scaffold_1:19027131-19027316	249.355	99.9638	-1.31872
-	scaffold_8:10684115-10718089	271.136	108.721	-1.31839
Ciclev10031906m.g	scaffold_4:25580547-25582930	0.497614	0.19961	-1.31784
Ciclev10019146m.g	scaffold_3:49512337-49517307	10.9793	4.40445	-1.31775
Ciclev10007170m.g	scaffold_9:11103567-11104929	0.720518	0.2891	-1.31747
Ciclev10003065m.g	scaffold_5:29590361-29592399	1.5996	0.642226	-1.31655
Ciclev10011564m.g	scaffold_6:17126056-17129828	11.56	4.64418	-1.31565
Ciclev10027044m.g	scaffold_7:14243032-14244426	1.8429	0.740686	-1.31504
Ciclev10001403m.g	scaffold_5:42943641-42945230	0.313798	0.126122	-1.31502
Ciclev10029681m.g	scaffold_8:21491314-21491781	35.0455	14.0943	-1.31412
Ciclev10021249m.g	scaffold_3:20556192-20557621	11.9252	4.7961	-1.31408
Ciclev10029495m.g	scaffold_8:24051886-24052549	1.87629	0.755104	-1.31313
Ciclev10011335m.g	scaffold_6:23758343-23760142	0.646876	0.260528	-1.31205
Ciclev10009166m.g	scaffold_1:27544531-27545731	29.7701	11.9906	-1.31195
Ciclev10012737m.g	scaffold_6:17233313-17234365	0.581785	0.234361	-1.31176
Ciclev10028397m.g	scaffold_8:21639623-21642175	2.8944	1.16605	-1.31164
-	scaffold_5:643172-644559	4.53343	1.82652	-1.31151
Ciclev10010158m.g	scaffold_1:17627428-17628097	1.19351	0.481359	-1.31003
Ciclev10014620m.g	scaffold_2:33446561-33451465	15.0641	6.07556	-1.31003
Ciclev10017085m.g	scaffold_2:31396676-31397374	1.2846	0.518181	-1.30979
Ciclev10024509m.g	scaffold_3:19310539-19608202	0.504065	0.203351	-1.30963
Ciclev10018686m.g	scaffold_3:30934733-30943274	31.0617	12.5311	-1.30963
Ciclev10017415m.g	scaffold_2:7712288-7715284	1.15731	0.466936	-1.30948
Ciclev10006124m.g	scaffold_9:22114942-22116474	442.237	178.437	-1.3094
Ciclev10031446m.g	scaffold_4:22743028-22748532	5.28073	2.13085	-1.30931
Ciclev10004145m.g,Ciclev10004149m.g	scaffold_9:29891234-29928223	15.7658	6.36507	-1.30855
Ciclev10000471m.g	scaffold_5:36327602-36331343	178.834	72.2152	-1.30825
Ciclev10017852m.g	scaffold_2:9430328-9435456	16.039	6.47822	-1.30791
Ciclev10025178m.g	scaffold_7:2556147-2562529	3.182	1.28628	-1.30672
Ciclev10032763m.g	scaffold_4:21662555-21664222	2.52076	1.01917	-1.30647
Ciclev10028621m.g	scaffold_8:20607773-20611605	74.1749	30.001	-1.30592
Ciclev10025357m.g	scaffold_7:742147-744098	0.41959	0.169724	-1.30579
Ciclev10026817m.g	scaffold_7:6780432-6781478	1.73344	0.701221	-1.3057
Ciclev10031007m.g	scaffold_4:18202742-18205466	1.14817	0.464535	-1.30548
Ciclev10007670m.g	scaffold_1:6420803-6425294	18.345	7.42885	-1.30417
Ciclev10030577m.g	scaffold_4:22564847-22570721	4.36096	1.76615	-1.30404
Ciclev10026685m.g	scaffold_7:11483881-11485486	0.557842	0.225967	-1.30374
Ciclev10005026m.g	scaffold_9:1313424-1319164	77.3331	31.3322	-1.30344
Ciclev10011683m.g	scaffold_6:5895318-5897382	0.214442	0.0869452	-1.30241
Ciclev10012747m.g	scaffold_6:21782043-21783120	7.49615	3.03967	-1.30223
Ciclev10026788m.g	scaffold_7:17050258-17052811	0.448818	0.18204	-1.30187
Ciclev10008140m.g	scaffold_1:17308565-17311235	2.98905	1.21244	-1.30178
Ciclev10023571m.g	scaffold_3:23725500-23725704	2708.93	1098.95	-1.3016
Ciclev10015392m.g	scaffold_2:6472570-6474273	5.41324	2.19642	-1.30134
Ciclev10032799m.g	scaffold_4:17826101-17829230	0.750988	0.304755	-1.30114
Ciclev10019056m.g	scaffold_3:49802635-49805960	13.2449	5.37625	-1.30077
Ciclev10010133m.g	scaffold_1:196458-196980	6.17264	2.50576	-1.30064
Ciclev10003979m.g	scaffold_5:2969088-2970111	0.510363	0.207324	-1.29964
Ciclev10013905m.g	scaffold_6:64999-67276	1.68284	0.683629	-1.29962
Ciclev10032536m.g	scaffold_4:21766214-21768471	4.17148	1.69513	-1.29917
Ciclev10011448m.g	scaffold_6:15107790-15109918	6.4063	2.60392	-1.29881

Ciclev10031178m.g,Ciclev10031179m.g	scaffold_4:25314586-25344549	16.1325	6.56117	-1.29794
Ciclev10003514m.g	scaffold_5:31115061-31119553	0.389954	0.158614	-1.29778
Ciclev10001687m.g	scaffold_5:42867853-42869593	0.238493	0.0970767	-1.29675
Ciclev10008263m.g	scaffold_1:1406701-1410403	28.7933	11.7209	-1.29665
Ciclev10031170m.g	scaffold_4:25119292-25121199	1.3111	0.533924	-1.29607
Ciclev10017665m.g	scaffold_2:25422296-25492273	22.2801	9.07758	-1.29538
Ciclev10026096m.g	scaffold_7:6287459-6289468	169.019	68.8643	-1.29536
Ciclev10023859m.g	scaffold_3:8365065-8368815	0.744611	0.303469	-1.29494
Ciclev10000808m.g	scaffold_5:29339627-29387910	33.7481	13.7561	-1.29474
Ciclev10033122m.g	scaffold_4:17175040-17177917	26.297	10.7201	-1.29459
Ciclev10011626m.g	scaffold_6:18361274-18364004	0.236707	0.0964975	-1.29454
Ciclev10012847m.g	scaffold_6:22156915-22158030	4.10897	1.67536	-1.2943
Ciclev10029015m.g	scaffold_8:21875916-21880999	8.59527	3.50623	-1.29362
Ciclev10031210m.g	scaffold_4:24763311-24766319	4.63215	1.89058	-1.29285
Ciclev10025237m.g	scaffold_7:19858337-19861017	0.189515	0.0774412	-1.29114
Ciclev10008008m.g	scaffold_1:24893715-24897647	10.2391	4.18474	-1.29087
Ciclev10007975m.g	scaffold_1:20553920-20558851	16.8185	6.87813	-1.28996
Ciclev10021265m.g	scaffold_3:1000832-1002383	14.7535	6.03476	-1.28969
Ciclev10013271m.g	scaffold_6:15539584-15544811	1.53879	0.629675	-1.28912
Ciclev10029633m.g	scaffold_8:7349169-7350212	0.521459	0.213428	-1.2888
Ciclev10016376m.g	scaffold_2:32646916-32649898	25.3216	10.3681	-1.28822
Ciclev10019870m.g	scaffold_3:42667795-42670673	6.44803	2.64245	-1.28698
Ciclev10019138m.g	scaffold_3:4818577-4821597	0.180469	0.0739712	-1.28672
Ciclev10029807m.g	scaffold_8:21118878-21119967	0.436069	0.178761	-1.28652
-	scaffold_8:14421940-14422942	275.381	112.893	-1.28647
Ciclev10017975m.g	scaffold_2:7507436-7569782	7.82565	3.21107	-1.28516
Ciclev10024869m.g,Ciclev10025053m.g	scaffold_7:8034220-8051948	3.52063	1.44493	-1.28483
Ciclev10015674m.g	scaffold_2:7927462-7929639	3.80166	1.56032	-1.28479
Ciclev10022851m.g	scaffold_3:47912700-47913512	27.49	11.2896	-1.28391
Ciclev10002312m.g	scaffold_5:37491104-37493595	31.2426	12.8335	-1.28361
Ciclev10014400m.g	scaffold_2:35497039-35504104	0.906756	0.37261	-1.28305
-	scaffold_9:24356661-24541601	11.0367	4.53539	-1.28301
Ciclev10008419m.g,Ciclev10010436m.g	scaffold_1:4009868-4027074	55.8332	22.9468	-1.28283
Ciclev10002383m.g	scaffold_5:41829633-41831556	0.440777	0.181195	-1.28251
-	scaffold_8:3655720-3656777	12.6264	5.19167	-1.28218
Ciclev10008922m.g	scaffold_1:11932311-11934116	0.656328	0.269899	-1.282
Ciclev10017726m.g	scaffold_2:24945168-24946821	0.232675	0.09573	-1.28127
Ciclev10032920m.g	scaffold_4:14404153-14405075	52.6381	21.6585	-1.28117
Ciclev10009141m.g	scaffold_1:27286386-27290085	6.81561	2.80584	-1.28041
Ciclev10025325m.g	scaffold_7:870924-875490	2.90779	1.19721	-1.28025
Ciclev10033449m.g	scaffold_4:668539-669853	0.688571	0.283589	-1.2798
Ciclev10025216m.g	scaffold_7:2984807-2989107	1.61024	0.663372	-1.27938
Ciclev10001336m.g	scaffold_5:26913090-26919381	0.289081	0.119158	-1.27859
-	scaffold_4:23360739-23361171	5.37593	2.21615	-1.27846
Ciclev10024042m.g,Ciclev10024539m.g	scaffold_3:24352153-24448329	15.9036	6.56289	-1.27695
Ciclev10020219m.g	scaffold_3:36287896-36289645	11.9613	4.93617	-1.27691
Ciclev10023520m.g	scaffold_3:30221017-30223095	0.351633	0.145205	-1.27598
Ciclev10024304m.g	scaffold_3:6242973-6244997	3535.7	1460.08	-1.27595
Ciclev10030485m.g	scaffold_4:13525690-13533032	2.52755	1.04379	-1.27591
Ciclev10026407m.g,Ciclev10027579m.g	scaffold_7:11062213-11079547	7.31148	3.01986	-1.27568
Ciclev10019057m.g	scaffold_3:33308676-33313031	2.63748	1.08937	-1.27566

Ciclev10011303m.g	scaffold_6:1094832-1101079	0.544768	0.22504	-1.27546
Ciclev10014518m.g	scaffold_2:25914012-25918879	1.5905	0.657352	-1.27474
Ciclev10031405m.g	scaffold_4:23670419-23675612	1.61603	0.66794	-1.27466
Ciclev10013925m.g	scaffold_2343:15-1400	13.9773	5.77725	-1.27464
Ciclev10028094m.g	scaffold_8:23963892-23969651	0.169073	0.0698875	-1.27454
Ciclev10026615m.g	scaffold_7:2152754-2153670	49.4778	20.456	-1.27426
Ciclev10013140m.g	scaffold_6:7782778-7784020	4.33702	1.79312	-1.27423
Ciclev10006202m.g	scaffold_9:14748935-14749813	15.0634	6.2293	-1.27391
Ciclev10010254m.g	scaffold_1:7040305-7041553	1.70053	0.703271	-1.27383
Ciclev10008476m.g	scaffold_1:385468-400033	12.296	5.0853	-1.27378
Ciclev10004016m.g	scaffold_5:29131688-29150431	15.4284	6.38225	-1.27345
Ciclev10001901m.g	scaffold_5:21098159-21102608	223.189	92.3704	-1.27276
Ciclev10008141m.g	scaffold_1:26141011-26143051	2.79376	1.15664	-1.27227
Ciclev10012523m.g	scaffold_6:24182173-24183672	4.67153	1.93412	-1.27222
Ciclev10001524m.g	scaffold_5:29481816-29485113	225.886	93.5445	-1.27187
Ciclev10022384m.g	scaffold_3:548047-549062	0.505375	0.20934	-1.27151
Ciclev10020664m.g	scaffold_3:49299543-49302901	0.428194	0.177437	-1.27096
Ciclev10007552m.g	scaffold_1:23694990-23699893	0.119778	0.0496519	-1.27044
Ciclev10017044m.g	scaffold_2:9912899-9913775	9.08631	3.76829	-1.26978
Ciclev10001231m.g	scaffold_5:42595067-42600153	10.2315	4.24588	-1.26888
Ciclev10020518m.g	scaffold_3:42850751-42855583	13.1571	5.46096	-1.26861
Ciclev10011010m.g	scaffold_6:9170310-9176497	0.123551	0.0512865	-1.26845
Ciclev10007723m.g	scaffold_1:4050230-4052482	4.27562	1.77513	-1.26821
-	scaffold_3:23774428-23774966	14.9538	6.21035	-1.26777
Ciclev10026522m.g	scaffold_7:9996369-9997710	18.7294	7.78071	-1.26733
Ciclev10029205m.g	scaffold_8:19276634-19278102	19.9224	8.28084	-1.26654
Ciclev10026714m.g	scaffold_7:8876128-8878922	384.732	159.974	-1.26602
Ciclev10011188m.g	scaffold_6:10089074-10092385	4.2389	1.76279	-1.26583
Ciclev10028054m.g	scaffold_8:24452744-24455738	11.2855	4.69479	-1.26534
Ciclev10032931m.g	scaffold_4:3193706-3196564	1217.8	506.733	-1.26498
Ciclev10001207m.g	scaffold_5:16649434-16653241	10.9626	4.56487	-1.26395
Ciclev10014701m.g	scaffold_2:24698852-24702216	2.30307	0.959173	-1.2637
Ciclev10018349m.g	scaffold_2:13458211-13459012	1.69085	0.704333	-1.26342
Ciclev10002879m.g	scaffold_5:1234640-1237928	5.46897	2.28002	-1.26222
Ciclev10019330m.g	scaffold_3:42308560-42311669	0.865097	0.360851	-1.26146
Ciclev10028389m.g	scaffold_8:24210469-24212406	1.4915	0.622284	-1.26112
Ciclev10009298m.g	scaffold_1:23424277-23425445	0.405564	0.16924	-1.26086
Ciclev10005926m.g	scaffold_9:27621066-27622194	1.62416	0.67776	-1.26085
Ciclev10009482m.g	scaffold_1:24398799-24399607	3.32206	1.38652	-1.26061
-	scaffold_4:11031847-11032228	10.0167	4.18228	-1.26004
Ciclev10029537m.g	scaffold_8:8778538-8780801	10211.5	4265	-1.25958
Ciclev10013526m.g	scaffold_6:22741637-22742989	0.384848	0.160842	-1.25865
Ciclev10005529m.g	scaffold_9:21107893-21109983	71.0206	29.6992	-1.25781
Ciclev10033679m.g	scaffold_4:21319784-21320216	3.1214	1.30553	-1.25756
Ciclev10003927m.g	scaffold_5:39112671-39121491	70.2645	29.4242	-1.25579
Ciclev10026240m.g	scaffold_7:17340784-17343713	0.629219	0.263511	-1.2557
Ciclev10018997m.g	scaffold_3:46325341-46329222	1.94821	0.815967	-1.25556
Ciclev10012394m.g,Ciclev10013468m.g	scaffold_6:23766082-23774904	24.5206	10.2722	-1.25525
Ciclev10009542m.g	scaffold_1:1421601-1422908	238.419	99.8846	-1.25516
Ciclev10022143m.g	scaffold_3:3643441-3647377	71.5926	29.996	-1.25504
Ciclev10032840m.g	scaffold_4:21468738-21472546	29.5515	12.3828	-1.25489
Ciclev10027120m.g	scaffold_7:4242182-4244611	0.582769	0.24427	-1.25444
Ciclev10003526m.g	scaffold_5:34265852-34267142	1.02699	0.430478	-1.25441
-	scaffold_6:10028356-10028490	6276.29	2631.13	-1.25423
Ciclev10003574m.g	scaffold_5:33718326-33722112	0.249641	0.104664	-1.25409

-	scaffold_2:25384067-25387970	4.501	1.88739	-1.25385
Ciclev10000921m.g	scaffold_5:15445700-15450041	286.428	120.107	-1.25385
Ciclev10010023m.g	scaffold_1:6479157-6480196	2.9844	1.25189	-1.25333
Ciclev10004275m.g	scaffold_9:2424914-2429331	0.131284	0.055114	-1.2522
Ciclev10020723m.g	scaffold_3:8427637-8430390	40.6646	17.0731	-1.25205
Ciclev10027416m.g	scaffold_7:13916806-14069972	0.151677	0.0637267	-1.25103
Ciclev10015046m.g	scaffold_2:12341109-12346771	4.57727	1.9232	-1.25098
-	scaffold_4:10989478-10989696	97.0048	40.7699	-1.25055
Ciclev10021807m.g	scaffold_3:46710781-46713632	9.32112	3.9201	-1.24961
Ciclev10020331m.g	scaffold_3:40757640-40758990	0.329998	0.138944	-1.24796
Ciclev10026336m.g	scaffold_7:6261296-6263128	17.2182	7.25479	-1.24693
-	scaffold_8:9325618-9585469	19.6019	8.26255	-1.24633
-	scaffold_6:17646435-17646794	48.3421	20.3869	-1.24564
Ciclev10020015m.g	scaffold_3:7486735-7493083	54.4846	22.9818	-1.24535
Ciclev10000353m.g	scaffold_5:30767754-30771272	0.967879	0.408283	-1.24526
Ciclev10033325m.g	scaffold_4:21402067-21406426	0.175909	0.0742175	-1.245
Ciclev10002005m.g	scaffold_5:43068092-43070554	59.7152	25.2008	-1.24463
Ciclev10022362m.g	scaffold_3:49740453-49745003	23.768	10.0337	-1.24416
-	scaffold_1:23382080-23382657	16.5568	6.99213	-1.24362
Ciclev10007853m.g	scaffold_1:26427305-26431123	6.73976	2.84789	-1.24281
Ciclev10029956m.g	scaffold_8:22461635-22462367	8.6642	3.66197	-1.24245
Ciclev10010367m.g	scaffold_1:3224491-3225583	0.385258	0.162896	-1.24187
Ciclev10032479m.g	scaffold_4:24503837-24505365	22.9658	9.71139	-1.24174
Ciclev10029787m.g	scaffold_8:23136752-23137537	5.80517	2.45565	-1.24123
Ciclev10009963m.g	scaffold_1:19076908-19077664	24.7857	10.4881	-1.24076
Ciclev10030572m.g	scaffold_4:1282349-1292520	1.08295	0.458258	-1.24074
Ciclev10007564m.g	scaffold_1:2742372-2746341	2.29499	0.971226	-1.24061
Ciclev10019237m.g	scaffold_3:48493457-48497265	5.00287	2.11809	-1.23999
Ciclev10008981m.g	scaffold_1:13123279-13126225	309.848	131.274	-1.23898
Ciclev10004021m.g	scaffold_5:32831731-32863079	10.9121	4.62545	-1.23826
Ciclev10017699m.g	scaffold_2:26369706-26373406	0.801103	0.339611	-1.23811
Ciclev10000329m.g	scaffold_5:30096449-30098995	0.11907	0.0504842	-1.23791
Ciclev10004873m.g	scaffold_9:5021287-5065070	12.1041	5.13337	-1.23752
Ciclev10019599m.g	scaffold_3:39181844-39300715	0.214703	0.0910719	-1.23726
Ciclev10007003m.g	scaffold_9:30980415-30984556	67.4963	28.6348	-1.23704
Ciclev10032172m.g	scaffold_4:17155861-17159566	11.0928	4.70662	-1.23685
Ciclev10008000m.g	scaffold_1:4156422-4160412	67.8105	28.7722	-1.23683
Ciclev10020676m.g	scaffold_3:3852140-3855898	24.5516	10.4241	-1.23589
Ciclev10020580m.g	scaffold_3:36291791-36293107	0.27134	0.115257	-1.23524
Ciclev10025755m.g	scaffold_7:4984937-4993217	6.99488	2.97319	-1.23429
Ciclev10025735m.g	scaffold_7:3334668-3337555	2.16187	0.919274	-1.23371
Ciclev10012467m.g	scaffold_6:20072905-20073937	0.473642	0.201437	-1.23347
Ciclev10003078m.g	scaffold_5:16990298-16991250	0.618151	0.262975	-1.23303
Ciclev10008768m.g	scaffold_1:6358029-6359568	8.21337	3.49549	-1.23248
Ciclev10028905m.g	scaffold_8:10894540-10898401	20.3824	8.67625	-1.23218
Ciclev10031169m.g	scaffold_4:10728378-10731133	12.5694	5.35058	-1.23214
Ciclev10015128m.g,Ciclev10016515m.g	scaffold_2:21506878-21515192	81.802	34.8377	-1.23149
Ciclev10003001m.g	scaffold_5:33268943-33270204	2.55266	1.08782	-1.23057
Ciclev10018035m.g	scaffold_2:3069920-3070541	2.43116	1.03616	-1.23039
Ciclev10003685m.g	scaffold_5:31469286-31718393	1.48824	0.634715	-1.22943
Ciclev10000897m.g	scaffold_5:42272191-42276417	6.77868	2.89345	-1.22821
Ciclev10027982m.g	scaffold_8:18931590-18934477	0.918236	0.391955	-1.22818
Ciclev10005387m.g	scaffold_9:532816-535001	5.08156	2.16989	-1.22765
Ciclev10017852m.g	scaffold_2:9430328-9435456	5.51436	2.35475	-1.22762
Ciclev10008365m.g	scaffold_1:25752022-25755476	18.4786	7.89331	-1.22715

Ciclev10017802m.g	scaffold_2:233379-237733	0.164247	0.0701655	-1.22703
Ciclev10020574m.g	scaffold_3:20762973-20770394	18.2336	7.79052	-1.22681
Ciclev10015606m.g	scaffold_2:29550745-29553863	8.33594	3.56167	-1.22679
Ciclev10001869m.g	scaffold_5:32900140-32903124	23.4424	10.0268	-1.22525
Ciclev10029845m.g	scaffold_8:21055116-21055482	15.5265	6.64146	-1.22516
Ciclev10011621m.g	scaffold_6:17323724-17327821	29.8509	12.7692	-1.22511
Ciclev10030736m.g	scaffold_4:21909660-21916503	9.16014	3.91897	-1.22489
Ciclev10012534m.g	scaffold_6:22181963-22184046	6.81241	2.91643	-1.22396
Ciclev10016998m.g	scaffold_2:25930307-25931908	121.032	51.8171	-1.22388
Ciclev10001864m.g	scaffold_5:40835332-40838299	9.05732	3.87788	-1.22382
Ciclev10024702m.g	scaffold_7:212341-220840	5.93103	2.54009	-1.22341
Ciclev10004660m.g	scaffold_9:2732899-2738896	71.4158	30.593	-1.22304
Ciclev10001155m.g	scaffold_5:40597271-40598741	2.62726	1.12649	-1.22172
Ciclev10023164m.g	scaffold_3:41627893-41634431	0.878447	0.376796	-1.22117
-	scaffold_8:11325099-11325516	8.24804	3.53791	-1.22115
Ciclev10000827m.g	scaffold_5:41258613-41263689	4.64514	1.99256	-1.2211
Ciclev10029511m.g	scaffold_8:20065430-20071183	4.68	2.00756	-1.22106
Ciclev10010624m.g	scaffold_1:5566698-5571112	0.0851624	0.0365321	-1.22105
-	scaffold_5:25667195-25667452	67.8436	29.107	-1.22085
Ciclev10025693m.g	scaffold_7:13366113-13368696	0.279475	0.119933	-1.22049
-	scaffold_4:17485820-17486028	405.512	174.096	-1.21986
Ciclev10015144m.g	scaffold_2:17976118-17977981	10.1457	4.35605	-1.21977
Ciclev10010967m.g	scaffold_6:14932984-15010648	0.261961	0.112499	-1.21943
Ciclev10032177m.g	scaffold_4:1166288-1169372	2.19755	0.943856	-1.21925
Ciclev10033918m.g	scaffold_4:25375725-25378032	0.147527	0.0633793	-1.21889
Ciclev10029456m.g	scaffold_8:24384670-24387116	0.235196	0.101087	-1.21827
Ciclev10022288m.g	scaffold_3:34673450-34674900	2.90756	1.25016	-1.2177
Ciclev10031239m.g	scaffold_4:14996039-15002581	94.3674	40.5921	-1.21709
Ciclev10005624m.g	scaffold_9:5516515-5517794	8.31121	3.57654	-1.21649
Ciclev10013864m.g	scaffold_6:22828055-22898575	1.21734	0.523996	-1.2161
Ciclev10007959m.g	scaffold_1:28888520-28892194	36.2524	15.6061	-1.21596
Ciclev10029774m.g	scaffold_8:16726668-16727617	25.6352	11.0362	-1.21587
Ciclev10029583m.g	scaffold_8:7909414-7965005	78.5495	33.8295	-1.21532
Ciclev10010828m.g	scaffold_1:28394517-28402379	0.681595	0.293768	-1.21424
Ciclev10000515m.g	scaffold_5:43170340-43172802	56.9772	24.5653	-1.21376
Ciclev10024454m.g	scaffold_3:27291188-27295420	0.401204	0.173142	-1.21238
-	scaffold_2:9496748-9497041	20.2345	8.73329	-1.21222
Ciclev10007028m.g	scaffold_9:3500323-3515705	12.5119	5.40117	-1.21196
Ciclev10011622m.g	scaffold_6:25349416-25351949	47.66	20.5785	-1.21164
-	scaffold_1:14646183-14647056	131.387	56.7552	-1.211
Ciclev10001291m.g	scaffold_5:41275200-41278829	0.222509	0.0961609	-1.21034
Ciclev10018045m.g	scaffold_2:32460490-32463101	98.7424	42.787	-1.2065
Ciclev10009900m.g	scaffold_1:4930631-4931370	48.5151	21.0227	-1.20648
-	scaffold_5:3330325-3331504	120.819	52.366	-1.20614
Ciclev10003035m.g	scaffold_5:22715571-22716418	0.500866	0.217142	-1.20579
Ciclev10031201m.g	scaffold_4:23066609-23069480	35.8702	15.5536	-1.20553
Ciclev10028371m.g	scaffold_8:1823963-1885505	0.221415	0.0960524	-1.20486
Ciclev10020389m.g	scaffold_3:936910-938678	1.7937	0.778241	-1.20465
Ciclev10020554m.g	scaffold_3:7020441-7024801	49.04	21.2784	-1.20457
Ciclev10009005m.g	scaffold_1:21870010-21873931	107.537	46.6894	-1.20366
Ciclev10032244m.g	scaffold_4:24612462-24616531	65.3286	28.3643	-1.20364
-	scaffold_5:1552172-1984270	15.455	6.71632	-1.20233
-	scaffold_3:4730531-4731612	130.347	56.6719	-1.20165
Ciclev10004543m.g	scaffold_9:28456944-28459088	9.25479	4.02401	-1.20156
Ciclev10028665m.g	scaffold_8:23383309-23386750	11.3924	4.95472	-1.2012
-	scaffold_7:5835696-5838865	28.8286	12.539	-1.20108

Ciclev10024791m.g	scaffold_7:1369083-1375811	2.73404	1.18979	-1.20033
Ciclev10023566m.g	scaffold_3:39720928-39722989	1.01189	0.440528	-1.19975
-	scaffold_5:26783765-26784186	62.7941	27.358	-1.19866
Ciclev10028214m.g	scaffold_8:1080301-1085905	41.3932	18.0448	-1.19781
Ciclev10005865m.g	scaffold_9:4029725-4032006	10.3644	4.5205	-1.19707
Ciclev10023815m.g	scaffold_3:21647477-21652375	11.489	5.01122	-1.19702
Ciclev10015465m.g	scaffold_2:5948965-5955103	49.2595	21.4898	-1.19675
Ciclev10006138m.g	scaffold_9:25677727-25682495	3.60673	1.57349	-1.19672
Ciclev10010742m.g	scaffold_1:2725367-2727898	0.407762	0.177931	-1.19641
Ciclev10009661m.g	scaffold_1:7914716-7915693	51.6699	22.5812	-1.1942
Ciclev10014466m.g	scaffold_2:9262666-9275068	332.019	145.235	-1.19288
Ciclev10003559m.g	scaffold_5:7286507-7287053	5.87342	2.56985	-1.19252
Ciclev10025621m.g	scaffold_7:14880207-14888981	87.6638	38.3722	-1.19192
Ciclev10001660m.g	scaffold_5:30531742-30535211	5.68374	2.48814	-1.19177
Ciclev10007072m.g	scaffold_9:850505-851048	3.05299	1.33661	-1.19164
Ciclev10000706m.g	scaffold_5:36090417-36095771	35.9875	15.7654	-1.19073
Ciclev10016823m.g	scaffold_2:25669057-25670108	9.82746	4.30697	-1.19014
Ciclev10019893m.g	scaffold_3:39133969-39135992	16.2329	7.11425	-1.19014
Ciclev10001183m.g	scaffold_5:28539341-28801354	0.200481	0.0879045	-1.18945
Ciclev10004479m.g	scaffold_9:14323093-14326233	12.5205	5.49002	-1.18941
Ciclev10016828m.g	scaffold_2:4660423-4661352	38.0436	16.6873	-1.1889
Ciclev10025286m.g	scaffold_7:13323582-13328683	59.0588	25.914	-1.18842
Ciclev10009804m.g	scaffold_1:5413232-5416822	82.6228	36.2541	-1.1884
Ciclev10029366m.g	scaffold_8:2782317-2782897	22.604	9.93456	-1.18605
Ciclev10004981m.g	scaffold_9:28166148-28170644	0.741539	0.325963	-1.18582
Ciclev10027751m.g	scaffold_8:18671466-18676601	4.02304	1.76967	-1.1848
-	scaffold_5:24926144-25131690	26.3829	11.6134	-1.18382
Ciclev10001445m.g	scaffold_5:34453873-34455426	25.6691	11.3011	-1.18357
Ciclev10024488m.g	scaffold_3:11462647-11464397	0.335116	0.147678	-1.18221
Ciclev10009362m.g	scaffold_1:1240480-1242589	19.338	8.5291	-1.18098
Ciclev10012384m.g	scaffold_6:15896415-15898044	215.89	95.2471	-1.18055
Ciclev10014584m.g	scaffold_2:27935054-27938445	1.17793	0.520187	-1.17915
Ciclev10020506m.g	scaffold_3:1510186-1511798	1.15456	0.509998	-1.17879
Ciclev10000851m.g	scaffold_5:26790423-26792763	4.74468	2.09586	-1.17877
Ciclev10025717m.g	scaffold_7:15872235-15875783	46.1775	20.4112	-1.17782
Ciclev10030752m.g	scaffold_4:6655445-6658579	0.931818	0.411931	-1.17765
-	scaffold_8:3439514-3439705	307.651	136.113	-1.17648
Ciclev10025011m.g	scaffold_7:7977526-7984425	27.1168	11.9998	-1.17617
Ciclev10023891m.g	scaffold_3:20633596-20638029	0.110404	0.048869	-1.17581
Ciclev10029126m.g	scaffold_8:2639801-2641261	11.7291	5.19291	-1.17548
Ciclev10028051m.g	scaffold_8:24769267-24772915	2.55842	1.13351	-1.17446
Ciclev10010075m.g	scaffold_1:28529271-28534084	9.1363	4.04858	-1.17419
Ciclev10000206m.g	scaffold_5:19378553-19383953	2.83015	1.25593	-1.17212
Ciclev10010135m.g	scaffold_1:753431-755538	20.251	8.9872	-1.17205
Ciclev10031946m.g	scaffold_4:16752151-16753508	13.5164	6.00184	-1.17123
Ciclev10030478m.g	scaffold_4:15783866-15805068	4.35474	1.93518	-1.17012
Ciclev10008858m.g	scaffold_1:2571060-2573956	1.965	0.873244	-1.17007
Ciclev10022504m.g	scaffold_3:48988754-48996232	21.0995	9.37831	-1.16981
Ciclev10014426m.g	scaffold_2:36251079-36255814	35.7078	15.8719	-1.16976
Ciclev10019677m.g	scaffold_3:36363008-36553812	12.3904	5.51819	-1.16695
Ciclev10009827m.g	scaffold_1:24654485-24659667	8.44172	3.76202	-1.16603
Ciclev10013592m.g,Ciclev10013765m.g	scaffold_6:8265976-8269490	4.62149	2.05996	-1.16574
Ciclev10032328m.g	scaffold_4:3770549-3771985	57.0426	25.4413	-1.16487
Ciclev10018741m.g	scaffold_3:1387645-1394177	13.2815	5.92372	-1.16485
Ciclev10021143m.g	scaffold_3:45720034-45723520	9.10397	4.06269	-1.16406

Ciclev10016561m.g	scaffold_2:24692918-24694730	2.54754	1.13802	-1.16257
Ciclev10026114m.g	scaffold_7:1333010-1335044	2.14927	0.960249	-1.16237
Ciclev10020762m.g	scaffold_3:3880733-3883449	389.661	174.173	-1.1617
Ciclev10000464m.g	scaffold_5:5001604-5005153	14.2321	6.36977	-1.15983
Ciclev10029453m.g	scaffold_8:24007860-24043482	8.04606	3.60128	-1.15977
Ciclev10013835m.g	scaffold_6:20720815-20721130	8.28006	3.70687	-1.15944
Ciclev10003845m.g	scaffold_5:42209501-42210342	17.4456	7.8139	-1.15875
Ciclev10026158m.g	scaffold_7:8921794-8925991	24.9729	11.1865	-1.15861
Ciclev10017718m.g	scaffold_2:32303434-32304052	2.68621	1.20351	-1.15832
-	scaffold_5:3980660-4069314	8.82517	3.95505	-1.15793
Ciclev10029378m.g	scaffold_8:19298370-19299813	196.09	87.9175	-1.15729
Ciclev10002620m.g	scaffold_5:11470191-11471157	25.5756	11.4673	-1.15725
-	scaffold_9:11400452-11402014	70.9161	31.7986	-1.15715
Ciclev10011730m.g	scaffold_6:17901617-17906439	66.2567	29.7137	-1.15694
Ciclev10012705m.g,Ciclev10013687m.g	scaffold_6:17239221-17285298	107.27	48.1267	-1.15633
Ciclev10029842m.g	scaffold_8:24105948-24106734	3.35599	1.50571	-1.1563
Ciclev10007129m.g	scaffold_9:28269558-28270506	1.28029	0.574555	-1.15596
Ciclev10033440m.g	scaffold_4:9304098-9304302	28.9132	12.9791	-1.15554
Ciclev10028735m.g	scaffold_8:1924981-1926909	0.775645	0.348209	-1.15544
Ciclev10032572m.g	scaffold_4:20533473-20543182	13.4194	6.02507	-1.15527
Ciclev10029985m.g	scaffold_8:7698015-7862701	14.6137	6.56518	-1.15442
Ciclev10024994m.g	scaffold_7:9843325-9849124	21.6361	9.72033	-1.15437
Ciclev10032886m.g	scaffold_4:17162156-17164814	56.1676	25.2363	-1.15424
Ciclev10000302m.g	scaffold_5:34276104-34281941	1.15685	0.519807	-1.15415
Ciclev10028203m.g,Ciclev10028286m.g	scaffold_8:8221246-8229261	27.3443	12.2901	-1.15374
Ciclev10025133m.g	scaffold_7:12352172-12754529	0.883854	0.397294	-1.1536
Ciclev10004937m.g	scaffold_9:26333674-26336837	1.43712	0.646601	-1.15223
Ciclev10014790m.g	scaffold_2:14461691-14464311	112.038	50.439	-1.15138
Ciclev10021613m.g	scaffold_3:49207143-49209171	8.82627	3.97648	-1.15031
Ciclev10003420m.g	scaffold_5:33984780-33986191	8.24292	3.71368	-1.15031
-	scaffold_5:40671871-40672575	240.722	108.453	-1.1503
Ciclev10009938m.g	scaffold_1:21887855-21892059	5.86252	2.64177	-1.15002
Ciclev10026811m.g	scaffold_7:20464469-20467666	2.71128	1.22187	-1.14988
Ciclev10000850m.g	scaffold_5:39495950-39498826	295.305	133.175	-1.14888
-	scaffold_1:28345516-28346295	234.994	106.001	-1.14854
Ciclev10020176m.g	scaffold_3:2619019-2621609	155.545	70.1763	-1.14828
-	scaffold_3:50367109-50367988	49.6877	22.4299	-1.14746
Ciclev10018848m.g	scaffold_3:3029044-3031684	1.23749	0.558694	-1.14729
Ciclev10016517m.g	scaffold_2:3658542-3663160	113.132	51.0765	-1.14727
Ciclev10033218m.g	scaffold_4:24994951-24995502	2.46746	1.11434	-1.14683
Ciclev10015754m.g	scaffold_2:31316918-31322323	12.1888	5.5047	-1.14682
Ciclev10012022m.g	scaffold_6:23593515-23595492	233.111	105.361	-1.14567
Ciclev10018865m.g	scaffold_3:27182221-27186798	97.5299	44.0854	-1.14554
Ciclev10001885m.g	scaffold_5:41987451-41990041	50.0572	22.631	-1.14527
Ciclev10017755m.g	scaffold_2:32209479-32211571	2.46422	1.1148	-1.14435
Ciclev10005223m.g	scaffold_9:8083003-8084819	2.79878	1.26663	-1.14381
-	scaffold_6:12465304-12465869	225.356	102.007	-1.14354
Ciclev10026716m.g	scaffold_7:1529253-1529846	43.5014	19.7038	-1.14259
Ciclev10028689m.g	scaffold_8:3843789-3891595	5.68303	2.5768	-1.14108
Ciclev10015168m.g	scaffold_2:20084562-20086249	7.0014	3.17461	-1.14106
Ciclev10018488m.g	scaffold_3:4427362-4441203	11.6861	5.29897	-1.14101
-	scaffold_5:2534144-2731098	8.14241	3.69284	-1.14073
Ciclev10014015m.g	scaffold_2:5051726-5070220	11.4276	5.1835	-1.14052
Ciclev10005353m.g	scaffold_9:3342614-3344229	22.7473	10.3233	-1.13978

Ciclev10011289m.g	scaffold_6:22334900-22338068	8.98123	4.07773	-1.13915
Ciclev10031165m.g	scaffold_4:551320-556394	20.2212	9.18584	-1.13838
Ciclev10026820m.g	scaffold_7:18975821-18981398	64.9219	29.5028	-1.13785
Ciclev10004191m.g	scaffold_9:11198279-11201820	1.8654	0.847869	-1.13757
Ciclev10006321m.g	scaffold_9:24788901-24789488	6.95489	3.16216	-1.13712
-	scaffold_1095:15-4666	15.0705	6.85467	-1.13656
Ciclev10008079m.g	scaffold_1:93834-96613	6.54307	2.97673	-1.13624
Ciclev10033819m.g	scaffold_4:24038218-24038924	117.633	53.5348	-1.13574
-	scaffold_8:8066199-8067238	4.24641	1.93327	-1.1352
Ciclev10011717m.g	scaffold_6:22011951-22016247	29.7513	13.5558	-1.13404
Ciclev10016019m.g	scaffold_2:3230741-3233253	7.74527	3.52944	-1.13388
Ciclev10023708m.g	scaffold_3:49393706-49394222	20.358	9.277	-1.13387
Ciclev10033853m.g	scaffold_4:25231974-25233757	15.8163	7.21168	-1.13301
Ciclev10025781m.g	scaffold_7:569164-574397	79.3905	36.2025	-1.13288
Ciclev10025540m.g	scaffold_7:6535521-6540965	9.18585	4.18965	-1.13258
-	scaffold_5:2878322-2878598	32.1538	14.6716	-1.13196
Ciclev10012011m.g	scaffold_6:22425093-22426402	4.78781	2.18532	-1.13152
-	scaffold_2:20451196-20451702	35.4977	16.2161	-1.1303
Ciclev10005563m.g	scaffold_9:20219861-20223557	327.248	149.562	-1.12964
Ciclev10007964m.g	scaffold_1:24942841-24945912	44.303	20.2626	-1.12858
Ciclev10029096m.g	scaffold_8:1935173-1936163	13.0816	5.98511	-1.12809
-	scaffold_3:28065477-28341901	13.754	6.29387	-1.12784
Ciclev10029483m.g	scaffold_8:3040541-3041447	50.5948	23.154	-1.12773
Ciclev10029437m.g	scaffold_8:24044995-24049924	10.8445	4.96479	-1.12716
Ciclev10004882m.g	scaffold_9:2150091-2156177	23.3624	10.7007	-1.12648
Ciclev10010994m.g	scaffold_6:17638531-17643264	2.94186	1.34762	-1.12632
Ciclev10028602m.g	scaffold_8:23908852-23911480	4.1772	1.91391	-1.12602
-	scaffold_2:33694162-33694813	370.89	169.968	-1.12573
Ciclev10007676m.g	scaffold_1:6396026-6400037	6.34341	2.90778	-1.12534
Ciclev10012991m.g	scaffold_6:17347523-17349267	39.7791	18.2357	-1.12525
Ciclev10031171m.g	scaffold_4:21831619-21837361	24.536	11.2494	-1.12505
Ciclev10007844m.g	scaffold_1:22175413-22384190	0.619365	0.2843	-1.12338
Ciclev10017436m.g	scaffold_2:31211055-31225713	77.9535	35.7977	-1.12274
Ciclev10014104m.g	scaffold_2:13909410-13917679	16.542	7.59837	-1.12237
Ciclev10026166m.g	scaffold_7:4174023-4175667	1.44871	0.665468	-1.12233
Ciclev10017728m.g	scaffold_2:28289961-28291356	1.90606	0.875776	-1.12196
Ciclev10000180m.g	scaffold_5:37502223-37507873	4.86952	2.23794	-1.12161
Ciclev10030771m.g,Ciclev10031967m.g,Ciclev10033368m.g,Ciclev10033766m.g,Ciclev10033930m.g	scaffold_4:5378982-5678990	58.5205	26.9026	-1.1212
Ciclev10017932m.g	scaffold_2:31884790-31887350	123.622	56.8316	-1.12117
-	scaffold_5:18197314-18201966	9.75808	4.48681	-1.12091
Ciclev10008860m.g	scaffold_1:4029777-4031597	40.3843	18.5728	-1.1206
Ciclev10017343m.g	scaffold_2:29110324-29112979	20.3423	9.35651	-1.12044
Ciclev10023862m.g	scaffold_3:27243915-27260256	15.1372	6.96373	-1.12017
Ciclev10021081m.g	scaffold_3:22851985-22853202	7.51781	3.459	-1.11996
-	scaffold_4:20935623-20935825	107.334	49.4103	-1.11922
Ciclev10028080m.g	scaffold_8:24448309-24451833	10.9421	5.04116	-1.11806
Ciclev10014611m.g	scaffold_2:8911566-8917638	123.252	56.8315	-1.11685
Ciclev10024601m.g	scaffold_3:28055808-28059361	83.7227	38.6516	-1.11509
Ciclev10010786m.g	scaffold_1:18429618-18429825	24.0262	11.0925	-1.11502
Ciclev10012160m.g	scaffold_6:19021839-19023746	10.2623	4.7405	-1.11424
Ciclev10002326m.g	scaffold_5:38401026-38402507	49.9194	23.0738	-1.11335
Ciclev10021374m.g	scaffold_3:5318873-5321054	41.6916	19.2729	-1.11319
-	scaffold_9:23862298-23862947	3.83372	1.77266	-1.11283

Ciclev10007532m.g,Ciclev10010416m.g	scaffold_1:26260732-26306089	3.41148	1.57768	-1.11259
Ciclev10022884m.g	scaffold_3:18902661-18909071	6.07635	2.81245	-1.11138
Ciclev10030486m.g	scaffold_4:13477039-13482717	0.421903	0.195323	-1.11105
Ciclev10014919m.g	scaffold_2:22995968-23001278	1.21453	0.562334	-1.1109
Ciclev10010636m.g	scaffold_1:1937508-1940005	1.44085	0.667163	-1.11081
Ciclev10028601m.g	scaffold_8:580919-583733	7.1848	3.32766	-1.11044
Ciclev10006337m.g	scaffold_9:9986212-9986984	6.43205	2.97985	-1.11004
Ciclev10020314m.g	scaffold_3:48450782-48455853	35.4869	16.4505	-1.10916
Ciclev10023524m.g	scaffold_3:9192783-9194985	0.206745	0.095999	-1.10676
Ciclev10009701m.g	scaffold_1:3356191-3360411	40.2875	18.7176	-1.10594
Ciclev10001229m.g,Ciclev10002028m.g	scaffold_5:32546088-32620214	25.3284	11.7683	-1.10585
Ciclev10002580m.g	scaffold_5:12812789-12815627	30.4009	14.1529	-1.10301
Ciclev10014149m.g	scaffold_2:8410447-8415480	2.37398	1.10561	-1.10246
Ciclev10020083m.g	scaffold_3:12791629-12795091	47.7546	22.2484	-1.10194
Ciclev10009663m.g	scaffold_1:25072653-25073652	83.5478	38.9258	-1.10188
-	scaffold_7:19671175-19671594	156.817	73.0803	-1.10153
Ciclev10027563m.g	scaffold_7:6775048-6779066	2.48368	1.15841	-1.10034
Ciclev10014748m.g,Ciclev10014826m.g	scaffold_2:31211055-31225713	196.317	91.5995	-1.09978
Ciclev10031448m.g	scaffold_4:22862807-22865758	5.98933	2.79498	-1.09956
Ciclev10032643m.g	scaffold_4:3413130-3417292	9.77282	4.56766	-1.09732
Ciclev10016259m.g	scaffold_2:9987433-9992419	32.2618	15.1002	-1.09526
Ciclev10012027m.g	scaffold_6:13230447-13232993	1.38535	0.648505	-1.09506
Ciclev10015483m.g	scaffold_2:35212690-35214865	28.0362	13.1265	-1.09481
Ciclev10020814m.g	scaffold_3:13749082-13753019	268.349	125.649	-1.09471
Ciclev10000273m.g	scaffold_5:8573280-8583459	88.2883	41.3804	-1.09327
Ciclev10016912m.g	scaffold_2:32236437-32237693	24.2524	11.3674	-1.09323
Ciclev10011834m.g	scaffold_6:11118966-11124267	3.40348	1.59597	-1.09258
Ciclev10003079m.g	scaffold_5:31784684-31786015	2.46222	1.15473	-1.09241
Ciclev10023974m.g	scaffold_3:20664768-20670970	1.66206	0.779727	-1.09193
Ciclev10031584m.g	scaffold_4:16705304-16707489	14.7315	6.91145	-1.09184
Ciclev10018308m.g	scaffold_2:23330798-23334424	11.8669	5.56801	-1.09171
Ciclev10004534m.g	scaffold_9:1351256-1355766	23.3533	10.9591	-1.09151
Ciclev10026755m.g	scaffold_7:5148742-5150333	20.6942	9.71224	-1.09135
Ciclev10009815m.g	scaffold_1:3531557-3532369	1.80425	0.846829	-1.09125
Ciclev10002009m.g	scaffold_5:42121458-42127625	63.1263	29.6292	-1.09122
Ciclev10030878m.g	scaffold_4:177578-182508	54.1375	25.4124	-1.0911
Ciclev10010931m.g	scaffold_6:24774103-24781001	12.7128	5.9693	-1.09064
Ciclev10026543m.g	scaffold_7:11630256-11632121	122.851	57.6956	-1.09038
Ciclev10023316m.g,Ciclev10023616m.g	scaffold_3:45354744-45357821	55.0841	25.8724	-1.09022
Ciclev10011856m.g	scaffold_6:20940869-20943191	2.89543	1.36042	-1.08972
Ciclev10004116m.g	scaffold_9:11303973-11317752	1.81938	0.855273	-1.08899
Ciclev10031489m.g	scaffold_4:17753989-17757513	15.5801	7.32676	-1.08846
Ciclev10025304m.g	scaffold_7:9157872-9161992	3.55387	1.67136	-1.08837
Ciclev10022854m.g	scaffold_3:9062137-9063873	14.4875	6.81476	-1.08807
Ciclev10021551m.g	scaffold_3:47200054-47201893	147.185	69.2401	-1.08795
Ciclev10015580m.g	scaffold_2:22344915-22350916	23.3412	10.9908	-1.08659
-	scaffold_9:12948650-12948962	31.7302	14.9421	-1.08647
Ciclev10016831m.g	scaffold_2:8546087-8548227	33.0142	15.5488	-1.08629
Ciclev10026163m.g	scaffold_7:5676655-5677970	11.5827	5.4565	-1.08592
Ciclev10003893m.g	scaffold_5:38124093-38126631	1.47055	0.693088	-1.08525
Ciclev10026226m.g	scaffold_7:2030550-2032785	4.02835	1.89923	-1.08478
Ciclev10009894m.g	scaffold_1:26237404-26239791	38.3652	18.096	-1.08413

-	scaffold_9:13070523-13070762	79.1731	37.3628	-1.08341
Ciclev10007797m.g,Ciclev10007801m.g	scaffold_1:22175413-22384190	6.82892	3.22274	-1.08337
Ciclev10013368m.g	scaffold_6:23040221-23050285	8.92006	4.20963	-1.08336
Ciclev10007630m.g	scaffold_1:26000428-26003254	8.79378	4.15199	-1.08268
Ciclev10002801m.g	scaffold_5:42963149-42967105	17.877	8.44201	-1.08245
Ciclev10001997m.g	scaffold_5:2317953-2322201	22.7291	10.7362	-1.08206
Ciclev10003724m.g	scaffold_5:4985573-4987190	5.64072	2.66445	-1.08204
Ciclev10025613m.g	scaffold_7:3921279-3924142	2.68192	1.26746	-1.08133
Ciclev10015042m.g	scaffold_2:12377694-12379170	27.4485	12.9772	-1.08075
Ciclev10011912m.g	scaffold_6:497151-499116	150.925	71.3814	-1.08021
-	scaffold_4:2064484-2064693	87.6339	41.4596	-1.07978
Ciclev10001604m.g	scaffold_5:30838247-30839761	598.608	283.415	-1.0787
-	scaffold_4:11027082-11029275	22.3454	10.5801	-1.07862
Ciclev10012265m.g	scaffold_6:22305921-22308021	8.9314	4.22985	-1.07828
Ciclev10009695m.g	scaffold_1:710008-710699	3.12246	1.47911	-1.07796
Ciclev10023541m.g	scaffold_3:1828214-1831739	17.1341	8.12175	-1.07701
Ciclev10019730m.g	scaffold_3:411948-413905	3.07222	1.45628	-1.07699
Ciclev10026528m.g	scaffold_7:13888033-13889844	11.7803	5.58439	-1.0769
Ciclev10026119m.g,Ciclev10026292m.g	scaffold_7:5747603-5753788	12.4195	5.88954	-1.07638
Ciclev10015086m.g	scaffold_2:23021192-23023457	4.99652	2.37137	-1.07521
Ciclev10019412m.g	scaffold_3:5797130-5801358	25.3355	12.031	-1.07441
Ciclev10016890m.g	scaffold_2:12281394-12282562	1.60362	0.761891	-1.07367
Ciclev10015294m.g	scaffold_2:14154095-14157668	2.62577	1.24784	-1.0733
Ciclev10028116m.g	scaffold_8:18296456-18298723	37.8137	17.9724	-1.07313
Ciclev10010532m.g	scaffold_1:19401078-19405837	0.6385	0.303526	-1.07287
Ciclev10014058m.g	scaffold_2:34736069-34742644	3.69486	1.7573	-1.07216
Ciclev10024603m.g	scaffold_3:3620361-3622844	10.7603	5.11783	-1.07211
Ciclev10003746m.g	scaffold_5:34542607-34543274	13.7441	6.53867	-1.07175
Ciclev10017407m.g	scaffold_2:6552251-6553118	0.630747	0.300351	-1.07041
Ciclev10000893m.g	scaffold_5:29125854-29130238	5.4896	2.61581	-1.06944
Ciclev10008348m.g	scaffold_1:22818737-22821775	0.789597	0.376258	-1.0694
Ciclev10021478m.g	scaffold_3:47819238-47821730	9.67723	4.61198	-1.06921
Ciclev10008812m.g	scaffold_1:18270817-18273015	366.332	174.725	-1.06807
Ciclev10007365m.g	scaffold_1:18169934-18176338	3.06871	1.46586	-1.06588
Ciclev10020877m.g	scaffold_3:33331839-33335944	8.22916	3.93094	-1.06587
Ciclev10026855m.g	scaffold_7:778697-781029	9.86911	4.7167	-1.06514
Ciclev10013109m.g	scaffold_6:15817496-15823667	3.13449	1.49948	-1.06376
Ciclev10023646m.g	scaffold_3:18648425-18650443	398.182	190.52	-1.06349
Ciclev10025075m.g	scaffold_7:4559983-4563560	31.5995	15.1257	-1.0629
Ciclev10005458m.g	scaffold_9:30895119-30899324	27.7	13.2621	-1.06258
Ciclev10019425m.g	scaffold_3:40956322-40958089	3.32811	1.5943	-1.06179
Ciclev10021475m.g	scaffold_3:3211557-3213928	216.83	103.881	-1.06163
Ciclev10005076m.g	scaffold_9:1187245-1191347	6.80883	3.26276	-1.06131
Ciclev10009335m.g,Ciclev10009342m.g	scaffold_1:8633372-8673448	52.3167	25.0738	-1.06109
Ciclev10007895m.g	scaffold_1:28807628-28814574	3.84304	1.84296	-1.06023
Ciclev10026117m.g	scaffold_7:1666169-1671258	23.7928	11.4174	-1.05929
Ciclev10022730m.g	scaffold_3:46450125-46451301	395.34	189.799	-1.05862
Ciclev10003485m.g	scaffold_5:36398839-36399851	34.2018	16.4287	-1.05785
Ciclev10005800m.g	scaffold_9:29948086-29950884	22.9056	11.0127	-1.05653
Ciclev10019888m.g	scaffold_3:47046134-47049135	3.63703	1.74912	-1.05613
Ciclev10009529m.g	scaffold_1:22414121-22415456	4.06544	1.95549	-1.05588
Ciclev10008869m.g	scaffold_1:23654784-23658857	42.7716	20.5899	-1.05472
Ciclev10023167m.g	scaffold_3:44707536-44708255	2.50436	1.20621	-1.05396

Ciclev10027898m.g	scaffold_8:10529692-10534512	0.379046	0.182612	-1.05359
Ciclev10006368m.g	scaffold_9:20062924-20064577	0.367953	0.177341	-1.053
Ciclev10018599m.g	scaffold_3:3372882-3377278	4.16996	2.01024	-1.05266
Ciclev10011223m.g	scaffold_6:13722056-13724585	1.21982	0.588081	-1.05258
Ciclev10016399m.g	scaffold_2:32628689-32634194	35.8276	17.2733	-1.05253
Ciclev10012318m.g	scaffold_6:25280630-25282061	6.31467	3.04449	-1.0525
Ciclev10007029m.g	scaffold_9:18493271-18495447	2.377	1.14611	-1.05239
Ciclev10015688m.g	scaffold_2:33126890-33128442	6.41426	3.09295	-1.0523
Ciclev10025336m.g	scaffold_7:3571637-3573361	2.56105	1.23538	-1.05179
Ciclev10011554m.g	scaffold_6:21450810-21454162	50.2712	24.2556	-1.05141
Ciclev10024464m.g	scaffold_3:753161-754704	79.5008	38.3627	-1.05127
Ciclev10003889m.g	scaffold_5:8429091-8434975	5.38334	2.59953	-1.05025
Ciclev10022339m.g	scaffold_3:36096008-36099799	76.3223	36.8596	-1.05007
Ciclev10000156m.g	scaffold_5:42071084-42074749	8.96372	4.33085	-1.04945
Ciclev10020391m.g	scaffold_3:260116-262699	38.1539	18.4463	-1.0485
Ciclev10011265m.g	scaffold_6:18052473-18058151	11.575	5.5969	-1.04831
Ciclev10020034m.g	scaffold_3:208865-211492	8.74497	4.22966	-1.04791
Ciclev10024891m.g	scaffold_7:8508836-8516904	25.1894	12.1859	-1.0476
Ciclev10020130m.g	scaffold_3:16078618-16081702	147.474	71.3673	-1.04712
Ciclev10031824m.g	scaffold_4:9505297-9506517	2.38824	1.15587	-1.04697
-	scaffold_8:21551587-21554549	4.55339	2.20469	-1.04637
Ciclev10015237m.g	scaffold_2:35473404-35474857	1.05854	0.512565	-1.04627
Ciclev10021069m.g	scaffold_3:21579108-21583976	6.88957	3.33637	-1.04613
Ciclev10004385m.g	scaffold_9:4888530-4892972	1.33909	0.648512	-1.04605
Ciclev10009068m.g	scaffold_1:28492007-28494909	5.47217	2.65033	-1.04594
Ciclev10006938m.g	scaffold_9:7804322-8050719	5.88145	2.84859	-1.04593
Ciclev10019320m.g	scaffold_3:49401955-49407598	5.02005	2.43188	-1.04563
Ciclev10019025m.g	scaffold_3:7840951-7847272	18.0849	8.76334	-1.04523
Ciclev10004326m.g	scaffold_9:2655887-2658618	3.27762	1.589	-1.04453
Ciclev10013621m.g	scaffold_6:20303274-20304256	1.21385	0.588696	-1.044
Ciclev10006232m.g	scaffold_9:275896-277516	32.4898	15.7738	-1.04246
Ciclev10033288m.g	scaffold_4:6098881-6099701	2.47017	1.20038	-1.04112
Ciclev10019581m.g	scaffold_3:44914941-44920328	33.0539	16.063	-1.04108
-	scaffold_2:4887931-4890684	93.4027	45.396	-1.0409
Ciclev10011587m.g	scaffold_6:22213543-22219300	222.157	107.975	-1.04088
-	scaffold_9:6142063-6142476	45.7663	22.2546	-1.04018
Ciclev10021514m.g	scaffold_3:2289044-2290757	2.50504	1.21823	-1.04005
Ciclev10016109m.g	scaffold_2:16422651-16423987	1.33485	0.649226	-1.03988
Ciclev10008610m.g	scaffold_1:11229048-11230742	5.52717	2.68857	-1.0397
Ciclev10010159m.g	scaffold_1:577734-581006	5.77619	2.81061	-1.03923
Ciclev10009431m.g	scaffold_1:24192517-24194019	1.89258	0.921011	-1.03907
Ciclev10011596m.g	scaffold_6:15932015-15936176	38.8939	18.9288	-1.03896
Ciclev10014680m.g	scaffold_2:25812550-25816916	1.91653	0.932845	-1.03879
Ciclev10023753m.g	scaffold_3:47565995-47571014	618.574	301.227	-1.0381
Ciclev10027433m.g	scaffold_7:2025963-2027936	41.9414	20.4267	-1.03792
Ciclev10003286m.g	scaffold_5:17805800-17809117	1.11559	0.543331	-1.0379
Ciclev10006899m.g	scaffold_9:11384364-11396906	1.26628	0.616745	-1.03786
Ciclev10020157m.g	scaffold_3:8087241-8092087	5.6102	2.73378	-1.03716
Ciclev10028422m.g	scaffold_8:23925963-23928007	0.640318	0.312017	-1.03716
-	scaffold_3:9362473-9362931	12.3458	6.01799	-1.03667
Ciclev10018339m.g	scaffold_2:21343764-21344515	1.15546	0.563373	-1.03631
Ciclev10027690m.g	scaffold_8:22655621-22665955	12.3518	6.02398	-1.03593
Ciclev10022936m.g,Ciclev10023160m.g	scaffold_3:6631931-6639335	67.5288	32.9436	-1.0355
Ciclev10018571m.g	scaffold_3:16834306-16840129	0.881787	0.430623	-1.034
Ciclev10025611m.g	scaffold_7:17491173-17497237	96.7709	47.2594	-1.03397

Ciclev10021913m.g	scaffold_3:8859429-8863221	20.879	10.1968	-1.03393
Ciclev10025501m.g	scaffold_7:16249522-16255343	16.5664	8.09149	-1.03378
Ciclev10008581m.g	scaffold_1:26509535-26513207	18.1435	8.86409	-1.03341
Ciclev10003014m.g	scaffold_5:27143728-27145511	20.8512	10.1896	-1.03303
Ciclev10023227m.g	scaffold_3:186546-187535	1.67246	0.817341	-1.03296
Ciclev10001832m.g	scaffold_5:41941762-41943552	6.33265	3.0948	-1.03296
Ciclev10015515m.g	scaffold_2:11323428-11326928	0.582664	0.284964	-1.03189
Ciclev10028140m.g	scaffold_8:539940-542765	1.55984	0.763134	-1.03139
Ciclev10002600m.g	scaffold_5:39180931-39182902	7.03251	3.44111	-1.03117
Ciclev10022171m.g	scaffold_3:47460751-47463827	5.26864	2.57877	-1.03075
Ciclev10028984m.g	scaffold_8:3903538-3904780	1.15201	0.563989	-1.03041
Ciclev10022266m.g	scaffold_3:29133570-29136023	197.631	96.7679	-1.03021
Ciclev10002284m.g	scaffold_5:40856372-40860211	12.0254	5.89032	-1.02967
Ciclev10010212m.g	scaffold_1:3802580-3807212	10.528	5.15768	-1.02943
Ciclev10018877m.g,Ciclev10024009m.g	scaffold_3:7528920-7546847	14.2149	6.9649	-1.02923
Ciclev10009025m.g	scaffold_1:25084405-25087091	545.679	267.41	-1.029
Ciclev10015663m.g	scaffold_2:32958088-32970806	44.7595	21.9371	-1.02882
-	scaffold_2:29445582-29448192	26.211	12.8517	-1.02821
Ciclev10032149m.g	scaffold_4:3258854-3262192	23.362	11.4552	-1.02816
Ciclev10024178m.g	scaffold_3:23419573-23420180	149.956	73.5314	-1.02811
Ciclev10029834m.g	scaffold_8:5718912-5719431	32.9801	16.1725	-1.02805
Ciclev10033543m.g	scaffold_4:6025714-6026668	162.403	79.6621	-1.02761
Ciclev10011169m.g	scaffold_6:21905556-21908832	72.5851	35.6183	-1.02705
Ciclev10012618m.g	scaffold_6:21681865-21683202	144.359	70.845	-1.02692
Ciclev10023594m.g	scaffold_3:31121885-31123521	2.69651	1.32355	-1.02669
Ciclev10029705m.g	scaffold_8:3010002-3014264	9.2271	4.52927	-1.0266
Ciclev10002029m.g	scaffold_5:19149137-19154358	57.1444	28.0703	-1.02557
Ciclev10024415m.g	scaffold_3:42030185-42033226	26.6174	13.0759	-1.02546
Ciclev10016863m.g	scaffold_2:10795981-10797667	1.97892	0.972286	-1.02526
Ciclev10013919m.g	scaffold_6:10449825-10452064	5.72023	2.81112	-1.02493
Ciclev10005866m.g	scaffold_9:22996539-22997969	4.50281	2.21294	-1.02486
Ciclev10000759m.g	scaffold_5:33894993-33900548	5.32374	2.61707	-1.02449
Ciclev10019341m.g	scaffold_3:2066498-2071691	3.01801	1.4839	-1.0242
Ciclev10019986m.g	scaffold_3:50954344-50957869	45.4093	22.3289	-1.02407
Ciclev10002925m.g	scaffold_5:34520507-34522509	981.159	482.528	-1.02387
Ciclev10017588m.g	scaffold_2:30523899-30548559	24.6431	12.1197	-1.02383
-	scaffold_4:6263980-6264230	202.493	99.6121	-1.02348
Ciclev10012388m.g	scaffold_6:25370287-25373154	18.2426	8.97749	-1.02293
Ciclev10000096m.g	scaffold_5:16383841-16396185	5.7187	2.81447	-1.02282
Ciclev10000610m.g,Ciclev10000618m.g	scaffold_5:37701505-37709444	18.0484	8.88423	-1.02255
Ciclev10028139m.g	scaffold_8:21381172-21385223	5.36986	2.64441	-1.02194
Ciclev10015452m.g	scaffold_2:24557241-24561827	73.9821	36.4376	-1.02175
Ciclev10031983m.g	scaffold_4:2776988-2781436	32.0972	15.811	-1.02152
Ciclev10011949m.g	scaffold_6:18896230-18902614	113.236	55.7852	-1.02138
Ciclev10021950m.g	scaffold_3:43093260-43096086	4.75038	2.34026	-1.02137
Ciclev10033273m.g	scaffold_4:7885338-7891653	7.76091	3.82365	-1.02127
Ciclev10001159m.g	scaffold_5:40139284-40142825	107.499	52.9683	-1.02112
Ciclev10000533m.g	scaffold_5:39289632-39299263	3.42658	1.68914	-1.02048
Ciclev10017223m.g	scaffold_2:32623248-32623733	5.20959	2.56862	-1.02017
Ciclev10001293m.g	scaffold_5:9983418-9985631	1.40798	0.694289	-1.02002
Ciclev10020064m.g	scaffold_3:39628209-39629734	75.1441	37.0579	-1.01988
Ciclev10008250m.g	scaffold_1:4148092-4154018	5.14462	2.53717	-1.01984
Ciclev10001360m.g	scaffold_5:40827747-40830739	57.44	28.3365	-1.01939
Ciclev10028221m.g	scaffold_8:22069038-22074682	0.600996	0.296491	-1.01937

Ciclev10009510m.g	scaffold_1:20930297-20931158	19.4446	9.60583	-1.01739
Ciclev10008915m.g	scaffold_1:27739952-27741098	4.20216	2.07593	-1.01737
Ciclev10018915m.g	scaffold_3:62156-67054	36.4679	18.0249	-1.01664
Ciclev10030866m.g	scaffold_4:19430314-19434630	96.8361	47.8832	-1.01603
Ciclev10001341m.g	scaffold_5:35989375-35991063	25.1117	12.4189	-1.01582
Ciclev10013693m.g	scaffold_6:15030948-15036408	7.16268	3.54419	-1.01505
Ciclev10033781m.g	scaffold_4:20718509-20723648	0.846619	0.418921	-1.01503
Ciclev10014144m.g,Ciclev10014153m.g	scaffold_2:30523899-30548559	4.58443	2.26891	-1.01475
Ciclev10005589m.g	scaffold_9:729076-730936	47.9175	23.7175	-1.0146
Ciclev10007732m.g	scaffold_1:5518820-5522727	0.634183	0.313907	-1.01456
-	scaffold_8:24940330-24940536	162.158	80.2648	-1.01456
Ciclev10011931m.g	scaffold_6:18933571-18937213	8.84666	4.37979	-1.01427
Ciclev10004458m.g	scaffold_9:378360-382860	28.8895	14.312	-1.01332
Ciclev10013076m.g	scaffold_6:5635751-5636488	1.47656	0.731703	-1.01291
Ciclev10017888m.g	scaffold_2:32407518-32411163	1.06553	0.528075	-1.01275
Ciclev10011302m.g	scaffold_6:23579127-23582757	5.42428	2.68847	-1.01265
Ciclev10021198m.g	scaffold_3:3675198-3677594	35.1089	17.4082	-1.01207
Ciclev10007999m.g	scaffold_1:25355350-25359330	16.5559	8.21096	-1.01172
Ciclev10018964m.g	scaffold_3:47476187-47482822	64.0528	31.7713	-1.01154
Ciclev10005082m.g	scaffold_9:26566368-26570006	23.7436	11.7833	-1.0108
Ciclev10012908m.g	scaffold_6:23161518-23163869	114.307	56.7526	-1.01016
Ciclev10010820m.g	scaffold_1:24498030-24498483	61.4833	30.5565	-1.00871
Ciclev10014246m.g	scaffold_2:29706028-29709011	1.27516	0.633742	-1.00871
Ciclev10009962m.g	scaffold_1:26705758-26710444	128.858	64.0466	-1.00859
Ciclev10021982m.g	scaffold_3:44556802-44560092	11.8651	5.89746	-1.00856
Ciclev10017395m.g	scaffold_2:7832348-7846578	31.7565	15.7997	-1.00715
Ciclev10025588m.g	scaffold_7:4587901-4590419	28.526	14.1945	-1.00694
Ciclev10026542m.g	scaffold_7:8317977-8319191	157.931	78.6304	-1.00613
Ciclev10024941m.g	scaffold_7:41476-45638	5.74949	2.86273	-1.00605
Ciclev10011313m.g	scaffold_6:24361565-24363601	3.37926	1.6826	-1.00602
Ciclev10002744m.g	scaffold_5:26510552-26513762	45.9454	22.884	-1.00558
Ciclev10008181m.g	scaffold_1:3521484-3525524	1.59193	0.793209	-1.005
Ciclev10000339m.g	scaffold_5:42194475-42197926	20.1225	10.0277	-1.00481
Ciclev10015916m.g	scaffold_2:31721816-31726756	3.7984	1.89295	-1.00476
Ciclev10012247m.g	scaffold_6:20655617-20659403	17.14	8.54704	-1.00387
Ciclev10026324m.g	scaffold_7:6402024-6404938	16.6315	8.29636	-1.00336
Ciclev10027660m.g	scaffold_8:701565-713530	4.60462	2.29837	-1.00247
Ciclev10014492m.g	scaffold_2:28826133-28829533	28.63	14.2931	-1.00221
Ciclev10032919m.g	scaffold_4:24884255-24887178	6.37121	3.18165	-1.00179
Ciclev10004959m.g	scaffold_9:1700782-1703800	3.0216	1.50946	-1.00128
Ciclev10004492m.g	scaffold_9:23214434-23218622	0.970944	0.485089	-1.00114
-	scaffold_5:30357813-30358020	426.405	213.179	-1.00016
Ciclev10032029m.g	scaffold_4:1641629-1643936	53.575	26.7958	-0.999555
Ciclev10015899m.g	scaffold_2:7627241-7631796	6.2371	3.12355	-0.997688
Ciclev10005130m.g	scaffold_9:11951187-11955273	5.5194	2.76562	-0.996908
Ciclev10002407m.g	scaffold_5:441330-538185	0.798123	0.399918	-0.996906
Ciclev10009402m.g	scaffold_1:8359140-8362578	2.98574	1.49614	-0.996842
Ciclev10022230m.g	scaffold_3:8078608-8083078	64.535	32.3414	-0.996698
Ciclev10015739m.g	scaffold_2:2801928-2804205	34.7829	17.4321	-0.99663
Ciclev10000694m.g	scaffold_5:17595312-17598089	13.5815	6.81127	-0.995648
Ciclev10031790m.g	scaffold_4:16068388-16075095	3.69855	1.85501	-0.995533
Ciclev10020754m.g	scaffold_3:41769832-41772129	4.8818	2.44862	-0.995442
Ciclev10010890m.g	scaffold_6:7561834-7666028	24.2952	12.1939	-0.99451
Ciclev10026763m.g	scaffold_7:7146601-7147226	6.10224	3.06455	-0.993661
Ciclev10017441m.g	scaffold_2:23141504-23144902	4.03989	2.02938	-0.993275

Ciclev10027848m.g	scaffold_8:23589701-23593390	18.8335	9.46149	-0.993161
Ciclev10012474m.g	scaffold_6:25017022-25019659	14.604	7.33696	-0.993108
Ciclev10002216m.g	scaffold_5:15695397-15699548	16.4662	8.27309	-0.993013
Ciclev10005478m.g	scaffold_9:29145563-29147885	5.93402	2.982	-0.992728
Ciclev10016358m.g	scaffold_2:35758545-35760859	6.59002	3.3122	-0.992491
Ciclev10030565m.g	scaffold_4:6341872-6358140	5.09777	2.56245	-0.992341
Ciclev10026009m.g	scaffold_7:8733486-8736636	7.77607	3.90879	-0.99232
Ciclev10031291m.g	scaffold_4:433344-436839	12.9585	6.51445	-0.992181
Ciclev10021843m.g	scaffold_3:41767744-41769070	0.666404	0.335108	-0.991772
Ciclev10024935m.g	scaffold_7:3832206-3838543	12.1881	6.13186	-0.991075
Ciclev10031367m.g	scaffold_4:3796044-3799940	33.2825	16.7517	-0.990452
Ciclev10021571m.g	scaffold_3:47956194-47957207	0.659785	0.33216	-0.990116
Ciclev10014569m.g	scaffold_2:27849256-27854640	3.12777	1.57541	-0.989406
Ciclev10020275m.g	scaffold_3:2096571-2098539	31.478	15.8568	-0.989246
Ciclev10016285m.g	scaffold_2:4424381-4427034	175.301	88.3149	-0.989103
Ciclev10013657m.g	scaffold_6:23144465-23148874	0.883631	0.445167	-0.989098
-	scaffold_8:22587654-22590958	19.0202	9.58381	-0.988864
Ciclev10016622m.g,Ciclev10017413m.g	scaffold_2:33303510-33307252	3.88357	1.95778	-0.988163
-	scaffold_3:21065990-21066720	5.48941	2.7675	-0.988065
-	scaffold_3:39181844-39300715	209.183	105.481	-0.987773
Ciclev10012501m.g	scaffold_6:22435910-22439416	158.154	79.7503	-0.987773
Ciclev10017011m.g	scaffold_2:7273711-7336406	14.2145	7.16853	-0.987619
-	scaffold_5:25927642-25928698	895.391	451.595	-0.987488
Ciclev10025535m.g	scaffold_7:5905855-5907416	0.439447	0.221783	-0.986542
Ciclev10016149m.g	scaffold_2:3632402-3633350	9.85319	4.97337	-0.986367
Ciclev10019635m.g	scaffold_3:1131355-1134462	4.24581	2.14399	-0.98574
Ciclev10028873m.g	scaffold_8:281618-285300	27.1847	13.7275	-0.985721
Ciclev10022632m.g	scaffold_3:49071925-49078831	5.96474	3.01229	-0.985596
Ciclev10000488m.g	scaffold_5:27585412-27592977	5.82334	2.94419	-0.983976
Ciclev10017054m.g	scaffold_2:29054382-29056627	125.606	63.5057	-0.983947
Ciclev10004687m.g	scaffold_9:2253077-2257283	12.0734	6.10806	-0.983044
Ciclev10005738m.g	scaffold_9:2854991-2857646	3.40341	1.72263	-0.982369
Ciclev10011035m.g	scaffold_6:19440365-19445030	2.05781	1.04164	-0.98225
Ciclev10020137m.g	scaffold_3:1088237-1091889	35.2346	17.8371	-0.982111
Ciclev10009428m.g	scaffold_1:1671834-1674259	47.5629	24.0797	-0.982022
Ciclev10013720m.g	scaffold_6:20460191-20461995	178.676	90.5246	-0.980963
Ciclev10002616m.g	scaffold_5:42850228-42852413	14.0295	7.10847	-0.980858
Ciclev10024844m.g	scaffold_7:6633972-6639609	3.61881	1.83381	-0.980672
Ciclev10031970m.g	scaffold_4:16817583-16984703	4.03056	2.04351	-0.979931
Ciclev10032186m.g	scaffold_4:25520507-25525216	15.8961	8.05986	-0.979841
Ciclev10009593m.g	scaffold_1:1257675-1258596	3.03255	1.53814	-0.979348
Ciclev10002089m.g	scaffold_5:25793407-25795877	2.59374	1.31598	-0.978892
Ciclev10001460m.g	scaffold_5:37254881-37257398	26.3485	13.377	-0.977962
Ciclev10014996m.g	scaffold_2:25779387-25783068	182.663	92.7588	-0.977626
Ciclev10024756m.g	scaffold_7:15585705-15589192	1.56618	0.795485	-0.977348
Ciclev10031998m.g	scaffold_4:19597302-19601621	244.804	124.356	-0.977154
Ciclev10026585m.g	scaffold_7:20453613-20455163	175.986	89.4254	-0.976708
Ciclev10030016m.g	scaffold_8:4006362-4008246	8.75871	4.45113	-0.976546
-	scaffold_978:5044-5312	132.029	67.1237	-0.975962
Ciclev10029135m.g	scaffold_8:177830-178744	1.65207	0.839972	-0.97586
Ciclev10026239m.g	scaffold_7:4267405-4272218	36.9349	18.784	-0.975479
Ciclev10017132m.g	scaffold_2:5917881-5918594	1.89173	0.962226	-0.975262
Ciclev10016257m.g,Ciclev10018363m.g	scaffold_2:11439284-11443490	45.7204	23.2701	-0.974357
Ciclev10030265m.g	scaffold_8:5969534-5970491	2.70021	1.37473	-0.973922

Ciclev10014111m.g	scaffold_2:31325804-31329886	1.69904	0.865174	-0.973656
-	scaffold_7:11314039-11316585	144.792	73.7806	-0.972672
Ciclev10012173m.g	scaffold_6:14318184-14321646	91.5715	46.6668	-0.972501
Ciclev10014494m.g	scaffold_2:9842219-9845321	14.7301	7.50945	-0.971984
Ciclev10028774m.g	scaffold_8:1491361-1556980	143.183	73.0595	-0.97072
Ciclev10031960m.g	scaffold_4:21227063-21228575	28.8723	14.7328	-0.97066
Ciclev10001068m.g	scaffold_5:35538653-35541459	58.9271	30.0759	-0.970322
Ciclev10003917m.g	scaffold_5:31925317-31954322	96.5769	49.3536	-0.968521
Ciclev10023211m.g	scaffold_3:41559329-41560866	2.09458	1.07047	-0.968411
Ciclev10019371m.g	scaffold_3:41326034-41334146	1.76497	0.902199	-0.968129
Ciclev10033047m.g	scaffold_4:24490926-24493186	130.811	66.8757	-0.967933
Ciclev10014169m.g,Ciclev10017419m.g	scaffold_2:27742180-27755869	24.8742	12.7225	-0.96727
Ciclev10025844m.g	scaffold_7:1474246-1475927	16.1338	8.25247	-0.967189
Ciclev10020172m.g	scaffold_3:16772695-16780195	20.2947	10.3819	-0.96703
Ciclev10020583m.g,Ciclev10023335m.g	scaffold_3:33174236-33188683	17.222	8.81204	-0.966706
Ciclev10006214m.g	scaffold_9:16867068-16867712	45.1912	23.1452	-0.965332
Ciclev10033063m.g	scaffold_4:25192542-25194288	20.8984	10.7041	-0.96523
Ciclev10017082m.g	scaffold_2:31801438-31804455	1.66138	0.851549	-0.964217
Ciclev10000738m.g	scaffold_5:39713348-39716184	3.50957	1.80003	-0.96327
Ciclev10007570m.g	scaffold_1:16463444-16466075	12.7256	6.5288	-0.962845
-	scaffold_5:4076819-4284428	26.8981	13.8009	-0.962743
Ciclev10006052m.g	scaffold_9:9489994-9490507	66.5859	34.1825	-0.961957
Ciclev10031536m.g	scaffold_4:20450304-20453019	1.34266	0.689726	-0.960999
Ciclev10019872m.g	scaffold_3:41921729-41924701	3.65219	1.87638	-0.960812
Ciclev10017000m.g	scaffold_2:261439-264627	13.8799	7.1322	-0.960576
Ciclev10010266m.g	scaffold_1:199017-201722	53.5626	27.5239	-0.960538
Ciclev10017702m.g	scaffold_2:23776561-23778976	4.01399	2.06315	-0.960192
Ciclev10012432m.g	scaffold_6:22067348-22069877	3.06975	1.57787	-0.960139
Ciclev10031493m.g	scaffold_4:17895342-17898031	4.49357	2.31035	-0.959751
Ciclev10019747m.g	scaffold_3:3587344-3589777	7.32115	3.76593	-0.959064
Ciclev10019664m.g	scaffold_3:19834359-19911599	30.8871	15.8948	-0.958447
Ciclev10031306m.g	scaffold_4:3612090-3615119	7.36167	3.78845	-0.958425
Ciclev10006005m.g	scaffold_9:6318925-6320915	5.79511	2.98557	-0.956831
Ciclev10004714m.g	scaffold_9:11247817-11252767	2.43595	1.25505	-0.956742
Ciclev10004956m.g	scaffold_9:28693001-28697846	113.737	58.6201	-0.956229
Ciclev10018829m.g	scaffold_3:2387800-2392571	33.705	17.3774	-0.955753
Ciclev10013606m.g	scaffold_6:15940241-15990100	1016.51	524.12	-0.955656
Ciclev10023264m.g	scaffold_3:45128946-45129672	2.10245	1.08407	-0.955617
Ciclev10004507m.g	scaffold_9:566505-571956	34.7595	17.9231	-0.955586
Ciclev10000129m.g	scaffold_5:25999616-26032103	1.06415	0.548762	-0.955449
Ciclev10020148m.g	scaffold_3:43047188-43049129	0.335389	0.173068	-0.9545
Ciclev10024060m.g	scaffold_3:30728988-30732384	6.73233	3.47516	-0.954028
-	scaffold_6:20193774-20195396	7.08314	3.65645	-0.953945
Ciclev10004276m.g	scaffold_9:25603643-25606763	0.361519	0.186628	-0.953909
Ciclev10006381m.g	scaffold_9:28061220-28063450	0.996855	0.514946	-0.952963
Ciclev10001059m.g,Ciclev10001060m.g	scaffold_5:34732987-34754900	25.2371	13.04	-0.952603
Ciclev10016392m.g	scaffold_2:27679706-27683861	28.8519	14.9078	-0.952602
Ciclev10017213m.g	scaffold_2:35991259-35992081	1.76458	0.911963	-0.952275
Ciclev10015984m.g	scaffold_2:30847373-30850171	14.4723	7.48259	-0.951687
Ciclev10008452m.g	scaffold_1:8051630-8053221	4.29701	2.22231	-0.951271
Ciclev10027635m.g	scaffold_1535:2396-3713	4.54648	2.35144	-0.951205
Ciclev10024739m.g,Ciclev10025113m.g	scaffold_7:800199-840184	4.44607	2.29972	-0.951073

Ciclev10025084m.g	scaffold_7:16626934-16631927	4.63449	2.39861	-0.950212
Ciclev10025701m.g	scaffold_7:3297600-3300948	9.79175	5.06895	-0.94988
Ciclev10022731m.g	scaffold_3:14548215-14551367	75.381	39.0366	-0.949375
Ciclev10028975m.g	scaffold_8:22057652-22059731	159.834	82.8184	-0.948546
Ciclev10016692m.g	scaffold_2:35180593-35182774	103.505	53.6327	-0.948509
Ciclev10033672m.g	scaffold_4:3892954-3907743	9.6062	4.97826	-0.948325
-	scaffold_4:14828963-14829525	9.11742	4.72535	-0.948203
Ciclev10014533m.g	scaffold_2:31603940-31623860	1.12933	0.58537	-0.948049
Ciclev10024304m.g	scaffold_3:6242973-6244997	565.843	293.313	-0.947961
Ciclev10012836m.g	scaffold_6:12352194-12353054	26.1727	13.5702	-0.94762
Ciclev10030126m.g	scaffold_8:7909414-7965005	32.3787	16.7888	-0.947544
-	scaffold_8:18172855-18174697	8.82538	4.57952	-0.946462
Ciclev10025018m.g	scaffold_7:9029223-9034916	16.3012	8.46409	-0.945549
Ciclev10023540m.g	scaffold_3:48576831-48579091	12.9171	6.70923	-0.945063
Ciclev10001022m.g	scaffold_5:24019099-24024516	8.32486	4.32412	-0.94502
Ciclev10014463m.g	scaffold_2:7144021-7151188	46.8418	24.3445	-0.944199
Ciclev10020557m.g	scaffold_3:40848610-40913406	0.615647	0.32003	-0.943897
Ciclev10032656m.g	scaffold_4:6937482-6940177	17.6835	9.19286	-0.943816
Ciclev10024373m.g	scaffold_3:42114765-42116061	2.04102	1.06205	-0.942439
Ciclev10021579m.g	scaffold_3:47325508-47328073	18.1614	9.45603	-0.941566
Ciclev10025290m.g	scaffold_7:17832956-17839750	4.70735	2.45145	-0.94128
Ciclev10009282m.g	scaffold_1:3272464-3275971	19.5563	10.1866	-0.940954
-	scaffold_5:35145183-35145548	27.6549	14.4078	-0.940691
-	scaffold_9:15864623-15934825	45.9386	23.948	-0.939803
Ciclev10021815m.g	scaffold_3:48517015-48518527	6.37924	3.3257	-0.939728
Ciclev10000235m.g	scaffold_5:119680-126181	316.728	165.175	-0.939253
Ciclev10001704m.g	scaffold_5:30549142-30550604	1.20929	0.630652	-0.939247
Ciclev10026010m.g	scaffold_7:2872415-2876978	16.0761	8.38675	-0.938735
Ciclev10029507m.g	scaffold_8:1015156-1016406	73.8639	38.5372	-0.93862
Ciclev10021279m.g	scaffold_3:45659221-45663090	83.1352	43.3817	-0.938374
-	scaffold_6:21918875-21919617	32.4151	16.9224	-0.937727
-	scaffold_2:25330243-25332457	9.17715	4.79377	-0.936885
Ciclev10021471m.g	scaffold_3:6876916-6878672	2.67641	1.39916	-0.935736
Ciclev10014199m.g	scaffold_2:5597836-5605362	1509.7	789.42	-0.935401
Ciclev10006217m.g	scaffold_9:28421143-28427765	40.197	21.0236	-0.935082
Ciclev10014824m.g	scaffold_2:34866247-34872606	8.79685	4.60117	-0.934986
-	scaffold_8:13577213-13577405	415.94	217.595	-0.934729
Ciclev10000382m.g	scaffold_5:41902167-41907204	8.6982	4.5508	-0.934597
-	scaffold_4:10990343-10990578	71.3102	37.3198	-0.934167
Ciclev10009223m.g,Ciclev10009333m.g	scaffold_1:17504347-17514220	15.4893	8.10637	-0.934143
Ciclev10028725m.g	scaffold_8:9318033-9320609	1.06947	0.559787	-0.933945
Ciclev10017616m.g	scaffold_2:28621912-28622422	243.759	127.59	-0.933944
Ciclev10001912m.g	scaffold_5:41975363-41977621	11.2527	5.89303	-0.933185
Ciclev10004300m.g	scaffold_9:12845640-12852274	5.33765	2.79569	-0.933004
Ciclev10032946m.g	scaffold_4:1399540-1400335	8.32433	4.36143	-0.932534
Ciclev10022085m.g	scaffold_3:4914762-4919442	8.36214	4.38368	-0.93173
Ciclev10020737m.g	scaffold_3:800626-802128	238.47	125.041	-0.931407
Ciclev10014437m.g	scaffold_2:6861955-6865446	8.73657	4.58127	-0.931319
Ciclev10000338m.g	scaffold_5:24926144-25131690	60.749	31.8609	-0.931072
Ciclev10030737m.g	scaffold_4:19962026-19967998	24.3399	12.7712	-0.930431
Ciclev10000544m.g	scaffold_5:20952024-20954797	0.750586	0.394011	-0.929781
Ciclev10026481m.g	scaffold_7:7118758-7119761	472.865	248.279	-0.929464
Ciclev10029704m.g	scaffold_8:23911932-23914475	3.601	1.89105	-0.929207
Ciclev10001047m.g	scaffold_5:26935331-26947618	76.0047	39.9358	-0.928404
Ciclev10022902m.g	scaffold_3:15689421-15690080	69.3044	36.4261	-0.927976

Ciclev10020289m.g	scaffold_3:32628246-32630110	1.28506	0.675513	-0.927777
Ciclev10033782m.g	scaffold_4:14173842-14176697	0.44163	0.232164	-0.927689
Ciclev10028343m.g	scaffold_8:2287525-2290355	11.1322	5.85434	-0.927158
Ciclev10000975m.g	scaffold_5:12122494-12130994	5.13513	2.70113	-0.926835
Ciclev10017720m.g	scaffold_2:28004792-28005836	0.785079	0.413055	-0.926504
Ciclev10025887m.g	scaffold_7:3567750-3570289	5.59068	2.94152	-0.926461
Ciclev10003642m.g	scaffold_5:40951550-40953130	138.068	72.6486	-0.926367
Ciclev10016491m.g	scaffold_2:31420456-31425299	80.2481	42.2272	-0.926293
Ciclev10019770m.g	scaffold_3:39768344-39771032	53.3122	28.062	-0.925846
Ciclev10019738m.g	scaffold_3:6465379-6471262	10.4116	5.48119	-0.925625
Ciclev10008902m.g	scaffold_1:2987979-2990176	8.43535	4.44134	-0.925453
Ciclev10025648m.g	scaffold_7:20897593-20901012	2.49422	1.31338	-0.925301
Ciclev10015776m.g	scaffold_2:32249164-32251694	0.474682	0.250049	-0.924748
Ciclev10025950m.g	scaffold_7:8438701-8442381	216.373	114.004	-0.924443
Ciclev10011580m.g	scaffold_6:21983043-21991500	3.29015	1.73365	-0.924341
Ciclev10004357m.g	scaffold_9:31244439-31250453	79.4779	41.8911	-0.923908
Ciclev10009100m.g	scaffold_1:4060374-4067203	19.639	10.3528	-0.923697
Ciclev10032959m.g	scaffold_4:12214864-12215433	3.40519	1.79566	-0.923217
Ciclev10028909m.g	scaffold_8:12563101-12564623	30.7149	16.205	-0.9225
Ciclev10024275m.g	scaffold_3:8555643-8555868	12.5139	6.60306	-0.922329
Ciclev10020267m.g	scaffold_3:4077972-4120402	23.2638	12.2756	-0.922298
Ciclev10019109m.g	scaffold_3:3823695-3826499	0.889837	0.469579	-0.922174
Ciclev10001435m.g	scaffold_5:37388292-37391250	23.0756	12.1825	-0.921556
Ciclev10020224m.g	scaffold_3:246167-250029	136.668	72.2357	-0.919897
Ciclev10010946m.g	scaffold_6:11531186-11535434	8.40227	4.4417	-0.919666
Ciclev10002703m.g	scaffold_5:34704104-34705984	1.23948	0.655259	-0.919598
Ciclev10031282m.g	scaffold_4:15277369-15280873	2.63431	1.39316	-0.919069
Ciclev10011771m.g	scaffold_6:17438269-17440122	28.6475	15.1507	-0.919029
Ciclev10030023m.g	scaffold_8:2673134-2674232	9.14195	4.83672	-0.918472
Ciclev10024977m.g	scaffold_7:10467684-10470677	0.366213	0.193772	-0.91832
Ciclev10011468m.g	scaffold_6:25355838-25359520	0.238993	0.126459	-0.9183
Ciclev10000648m.g	scaffold_5:27842975-27853846	40.0558	21.196	-0.918219
Ciclev10002796m.g	scaffold_5:39444680-39445917	7.69412	4.0721	-0.917983
Ciclev10015151m.g	scaffold_2:32418773-32424515	10.4948	5.55559	-0.917657
Ciclev10001024m.g	scaffold_5:17606026-17618469	22.6687	12.0087	-0.916616
Ciclev10030978m.g	scaffold_4:1517400-1521860	4.68282	2.4809	-0.916517
Ciclev10016629m.g	scaffold_2:2175205-2176049	139.58	73.9683	-0.91611
Ciclev10018667m.g	scaffold_3:7674222-7679805	1.70845	0.905547	-0.915829
Ciclev10015031m.g	scaffold_2:36305903-36308724	18.0496	9.57209	-0.915061
Ciclev10024188m.g	scaffold_3:7135108-7136388	2.43465	1.29117	-0.915041
Ciclev10006216m.g	scaffold_9:6697571-6699157	1017.34	539.532	-0.915017
Ciclev10008356m.g	scaffold_1:2512067-2517523	13.4422	7.1299	-0.914814
Ciclev10011252m.g	scaffold_6:19641519-19644834	3.19976	1.69759	-0.914479
Ciclev10009178m.g	scaffold_1:4036128-4038959	6.50961	3.4547	-0.91401
Ciclev10021059m.g	scaffold_3:46054709-46056408	3.68304	1.95468	-0.913968
Ciclev10012019m.g	scaffold_6:21443412-21444863	8.34905	4.43133	-0.913873
Ciclev10008695m.g	scaffold_1:26163915-26169507	5.77027	3.06324	-0.913581
Ciclev10018579m.g	scaffold_3:7719436-7742996	1.6455	0.873804	-0.913144
Ciclev10003304m.g,Ciclev10003672m.g	scaffold_5:40991931-40993992	573.483	304.626	-0.912712
Ciclev10001042m.g	scaffold_5:34414460-34417249	13.9899	7.43197	-0.912563
Ciclev10021413m.g	scaffold_3:24629369-24734809	1.28144	0.681021	-0.911991
Ciclev10011065m.g	scaffold_6:21480161-21488681	4.85943	2.58319	-0.911634
Ciclev10007148m.g	scaffold_9:27994044-27995189	35.4758	18.8598	-0.911525
Ciclev10015222m.g	scaffold_2:21187168-21188518	11.2158	5.96438	-0.911086
Ciclev10020783m.g	scaffold_3:1199308-1203079	3.1253	1.66201	-0.911067

Ciclev10031200m.g	scaffold_4:16210719-16218022	57.0574	30.3433	-0.911037
Ciclev10023022m.g	scaffold_3:40504072-40719738	12.7214	6.76577	-0.910934
Ciclev10011574m.g	scaffold_6:15283058-15290088	13.9858	7.43886	-0.910813
Ciclev10020463m.g	scaffold_3:42021048-42024337	4.17244	2.2206	-0.90994
Ciclev10004836m.g	scaffold_9:22092432-22094070	19.5529	10.407	-0.909828
Ciclev10029783m.g	scaffold_8:22307649-22308456	3.08701	1.64331	-0.909604
Ciclev10030207m.g	scaffold_8:5957669-5958563	3.30283	1.7592	-0.908782
Ciclev10029295m.g	scaffold_8:1589388-1592716	6.99451	3.72571	-0.90871
Ciclev10023139m.g	scaffold_3:253878-254627	79.2691	42.236	-0.908284
Ciclev10007716m.g	scaffold_1:985260-989352	59.8651	31.9304	-0.906787
Ciclev10008935m.g	scaffold_1:4192564-4194432	1.43016	0.762815	-0.906768
Ciclev10029672m.g	scaffold_8:9829744-9831483	35.6632	19.0239	-0.906624
Ciclev10021601m.g	scaffold_3:39352984-39354543	5.90466	3.15022	-0.9064
Ciclev10006014m.g	scaffold_9:8507995-8509091	55.3067	29.5099	-0.906254
Ciclev10011058m.g	scaffold_6:16592768-16602202	71.018	37.9024	-0.905895
Ciclev10028366m.g	scaffold_8:1604459-1606311	2.11521	1.12904	-0.905705
Ciclev10027028m.g	scaffold_7:17486660-17489826	1.40314	0.749504	-0.904652
-	scaffold_9:17239880-17240034	4699	2510.32	-0.904483
Ciclev10007296m.g	scaffold_1:2015893-2023323	23.3763	12.4922	-0.904017
Ciclev10025081m.g	scaffold_7:7280314-7285822	25.7228	13.747	-0.903927
Ciclev10012424m.g	scaffold_6:22748168-22755153	12.4553	6.65689	-0.903846
Ciclev10025379m.g	scaffold_7:7317933-7321602	5.51229	2.94668	-0.903561
Ciclev10000825m.g	scaffold_5:40617690-40619818	66.5507	35.5802	-0.90338
Ciclev10008594m.g,Ciclev10010851m.g	scaffold_1:7696566-7700750	8.40125	4.49291	-0.902956
Ciclev10005508m.g	scaffold_9:1356579-1359310	16.0672	8.59267	-0.90294
Ciclev10018373m.g	scaffold_2:27274971-27275698	127.319	68.0937	-0.902848
Ciclev10009090m.g	scaffold_1:758861-760017	25.4398	13.6076	-0.902672
Ciclev10014219m.g	scaffold_2:32903447-32914855	27.9368	14.944	-0.902594
Ciclev10007842m.g	scaffold_1:9007492-9081493	20.7425	11.0985	-0.902224
Ciclev10015076m.g	scaffold_2:10691117-10694680	24.5124	13.1173	-0.902044
-	scaffold_6:128093-130159	6.81423	3.64825	-0.901345
Ciclev10023317m.g	scaffold_3:47687295-47692118	83.7092	44.8246	-0.901095
Ciclev10016074m.g	scaffold_2:34913865-34915479	3.11772	1.66959	-0.901001
Ciclev10028654m.g	scaffold_8:21839194-21845835	31.0578	16.6336	-0.900853
Ciclev10024786m.g	scaffold_7:3488264-3498732	2.21243	1.18494	-0.900813
Ciclev10002859m.g	scaffold_5:41432161-41439014	38.3843	20.5619	-0.90054
Ciclev10027598m.g	scaffold_7:20780152-20781475	24.3725	13.0567	-0.900463
Ciclev10024870m.g	scaffold_7:9935392-9942601	4.15199	2.22488	-0.900076
Ciclev10022422m.g	scaffold_3:3930686-3931448	20.8636	11.1803	-0.900026
Ciclev10022170m.g	scaffold_3:49949395-49952120	17.3106	9.2849	-0.898702
Ciclev10015235m.g	scaffold_2:34849441-34852700	32.9258	17.6608	-0.898669
Ciclev10008015m.g	scaffold_1:2551919-2555129	8.96429	4.80939	-0.898336
Ciclev10008537m.g	scaffold_1:18068296-18070262	11.0889	5.94944	-0.898295
Ciclev10016511m.g	scaffold_2:32418773-32424515	61.1241	32.797	-0.898177
Ciclev10033920m.g	scaffold_4:3093113-3094015	0.805325	0.432209	-0.897844
Ciclev10006604m.g	scaffold_9:26557905-26560040	0.772665	0.414743	-0.897626
Ciclev10001514m.g	scaffold_5:29581918-29583598	0.360094	0.193346	-0.897189
Ciclev10013115m.g	scaffold_6:5988162-5990923	108.68	58.357	-0.897109
Ciclev10009852m.g	scaffold_1:6984521-6985212	16.4069	8.81197	-0.896768
Ciclev10010298m.g	scaffold_1:24877678-24879992	1.90853	1.02511	-0.896685
Ciclev10033102m.g	scaffold_4:25190681-25192326	146.775	78.8909	-0.895676
Ciclev10027728m.g	scaffold_8:22846816-22852817	7.91748	4.25653	-0.895365
-	scaffold_4:7494591-7496151	12.5531	6.74918	-0.895262
Ciclev10000062m.g	scaffold_5:43093043-43098705	5.68258	3.0574	-0.894239
Ciclev10025636m.g	scaffold_7:11118625-11121713	5.43195	2.92262	-0.894207

Ciclev10011627m.g	scaffold_6:20821513-20825227	29.5695	15.9139	-0.893821
Ciclev10020883m.g	scaffold_3:17470535-17474419	6.32864	3.40616	-0.893749
Ciclev10015211m.g	scaffold_2:31693764-31696846	0.627161	0.337558	-0.893701
Ciclev10030411m.g	scaffold_8:742600-743104	2.37664	1.27997	-0.89281
Ciclev10012786m.g	scaffold_6:24264176-24265636	1.09019	0.587519	-0.891872
Ciclev10006390m.g	scaffold_9:15297806-15298516	103.817	55.9602	-0.891562
Ciclev10020663m.g	scaffold_3:49286522-49291600	29.1708	15.7264	-0.891338
Ciclev10000710m.g,Ciclev10002987m.g	scaffold_5:21802096-21827812	90.4213	48.749	-0.89129
Ciclev10007207m.g	scaffold_9:4448166-4450777	5.41558	2.92107	-0.890618
-	scaffold_9:11576287-11715575	41.2303	22.2728	-0.888423
Ciclev10031484m.g	scaffold_4:6203851-6205425	12.6118	6.81319	-0.888378
Ciclev10011809m.g	scaffold_6:6265277-6272266	32.4773	17.5458	-0.888305
Ciclev10032803m.g	scaffold_4:2167217-2168964	8.8468	4.78223	-0.887472
Ciclev10011175m.g	scaffold_6:10016563-10019369	39.4374	21.3221	-0.887218
Ciclev10029367m.g	scaffold_8:14277630-14278898	18.4108	9.95507	-0.887053
Ciclev10004749m.g	scaffold_9:4708309-4714548	6.78087	3.66739	-0.886719
Ciclev10019841m.g	scaffold_3:7792187-7795727	29.5035	15.96	-0.886427
Ciclev10004403m.g,Ciclev10004405m.g	scaffold_9:6243723-6272016	10.2444	5.54259	-0.88621
Ciclev10003664m.g,Ciclev10004075m.g	scaffold_5:1311648-1355130	14.3531	7.76764	-0.885814
Ciclev10026756m.g	scaffold_7:8310614-8313304	62.14	33.6311	-0.885724
Ciclev10002843m.g	scaffold_5:33411666-33413970	209.039	113.139	-0.885678
Ciclev10026100m.g	scaffold_7:11682560-11684857	4.97134	2.69073	-0.885637
Ciclev10009072m.g	scaffold_1:168022-170638	4.47649	2.42292	-0.885622
Ciclev10017578m.g,Ciclev10017827m.g	scaffold_2:35967952-35970322	12.304	6.66005	-0.885524
-	scaffold_4:9214713-9215066	11.9942	6.49373	-0.885219
Ciclev10011295m.g	scaffold_6:1108259-1111301	9.96172	5.39436	-0.884944
Ciclev10026849m.g	scaffold_7:15353294-15355555	71.2333	38.589	-0.884363
Ciclev10027056m.g	scaffold_7:11686788-11771145	6.95416	3.76735	-0.884328
Ciclev10019600m.g	scaffold_3:45833455-45838349	67.0104	36.3171	-0.883737
Ciclev10015355m.g	scaffold_2:7952692-7954772	4.12893	2.23838	-0.883312
Ciclev10008931m.g	scaffold_1:3874906-3877195	16.4751	8.93332	-0.883017
Ciclev10028295m.g	scaffold_8:24090577-24094232	46.7379	25.3449	-0.882899
Ciclev10025618m.g	scaffold_7:13810685-13814703	7.22837	3.92079	-0.882527
Ciclev10027695m.g	scaffold_8:17844741-17854467	0.124538	0.0675898	-0.88171
Ciclev10011184m.g	scaffold_6:25237871-25242427	35.8188	19.4424	-0.881508
Ciclev10005522m.g	scaffold_9:29880404-29881583	13.041	7.08338	-0.880545
Ciclev10007546m.g	scaffold_1:24553381-24559515	11.6177	6.31049	-0.880502
Ciclev10005301m.g	scaffold_9:30171790-30177202	44.6029	24.2383	-0.879852
Ciclev10014532m.g	scaffold_2:34232452-34235596	7.37499	4.0087	-0.879508
Ciclev10006215m.g	scaffold_9:15324109-15324844	0.835522	0.454176	-0.879425
Ciclev10008087m.g	scaffold_1:28827438-28830021	6.10285	3.31835	-0.879019
Ciclev10009208m.g	scaffold_1:2951319-2954175	13.1583	7.15572	-0.8788
Ciclev10029499m.g	scaffold_8:19546540-19549348	24.0658	13.0902	-0.878498
Ciclev10002717m.g	scaffold_5:37240885-37243414	42.8444	23.3099	-0.878162
Ciclev10030373m.g	scaffold_8:19799300-19802046	0.344052	0.187236	-0.877766
Ciclev10021442m.g	scaffold_3:43115802-43117814	354.495	192.951	-0.877527
Ciclev10001820m.g	scaffold_5:33752933-33756675	19.2044	10.4543	-0.877343
Ciclev10031592m.g	scaffold_4:12834102-12835666	0.393198	0.214082	-0.87709
Ciclev10000601m.g	scaffold_5:38772838-38775306	4.43891	2.41683	-0.877087
Ciclev10022899m.g	scaffold_3:7277472-7278283	17.2811	9.41045	-0.876859
Ciclev10024287m.g	scaffold_3:32716415-32805364	15.5624	8.47721	-0.876405
Ciclev10008543m.g	scaffold_1:14835508-14837797	0.924718	0.503741	-0.87633

Ciclev10002153m.g	scaffold_5:42291779-42292947	19.1511	10.4359	-0.875877
Ciclev10008049m.g	scaffold_1:7045212-7048799	42.7978	23.3224	-0.875818
Ciclev10012164m.g	scaffold_6:19753977-19756116	39.2499	21.393	-0.875552
Ciclev10022804m.g	scaffold_3:47974459-47975237	24.8201	13.529	-0.875451
Ciclev10003143m.g	scaffold_5:39584829-39589357	29.5711	16.1212	-0.875234
Ciclev10014430m.g	scaffold_2:34501500-34504295	45.4427	24.7801	-0.874868
Ciclev10004948m.g	scaffold_9:1817993-1819854	20.1028	10.9631	-0.874739
Ciclev10031758m.g	scaffold_4:997254-1002343	18.5236	10.1058	-0.874185
Ciclev10029179m.g	scaffold_8:17859576-17862892	58.413	31.8705	-0.874067
Ciclev10002160m.g	scaffold_5:38044495-38047499	46.202	25.2165	-0.873588
Ciclev10015394m.g	scaffold_2:16548473-16553062	15.5943	8.51638	-0.872708
Ciclev10004549m.g	scaffold_9:29944607-29947599	0.466245	0.254648	-0.872586
Ciclev10014850m.g	scaffold_2:27551455-27556561	7.28203	3.97838	-0.872159
Ciclev10028468m.g	scaffold_8:20765981-20774157	57.979	31.6816	-0.871883
Ciclev10032697m.g	scaffold_4:3335944-3338632	65.5992	35.8755	-0.870681
Ciclev10015231m.g	scaffold_2:34270986-34272863	80.3001	43.9187	-0.870569
-	scaffold_5:1552172-1984270	5.7076	3.12283	-0.870031
-	scaffold_6:4000990-4001177	1618.26	885.651	-0.869633
Ciclev10012693m.g	scaffold_6:21813743-21815992	89.655	49.0763	-0.869356
Ciclev10016242m.g	scaffold_2:25374181-25378403	90.3029	49.4391	-0.869121
Ciclev10001611m.g	scaffold_5:39241457-39245153	118.669	64.9972	-0.868496
Ciclev10004101m.g	scaffold_26:20537-24556	0.434623	0.238075	-0.868349
Ciclev10027309m.g	scaffold_7:13343315-13343936	8.80178	4.82168	-0.868261
-	scaffold_4:14058309-14058653	42.2325	23.1367	-0.868167
Ciclev10009425m.g	scaffold_1:23820246-23823101	33.7927	18.5159	-0.867946
-	scaffold_3:431162-431546	41.335	22.659	-0.867276
Ciclev10029272m.g	scaffold_8:1689940-1692902	218.444	119.762	-0.867093
Ciclev10008085m.g	scaffold_1:10575666-10577716	0.285739	0.156684	-0.866839
Ciclev10029965m.g	scaffold_8:2427000-2430311	0.687337	0.377043	-0.86629
Ciclev10029068m.g	scaffold_8:24718237-24719703	3.92704	2.15525	-0.865587
Ciclev10033272m.g	scaffold_4:20185185-20200225	8.2853	4.54781	-0.865381
Ciclev10012644m.g	scaffold_6:20285950-20289908	40.1754	22.0623	-0.864729
Ciclev10018521m.g	scaffold_3:50882691-50894255	0.533409	0.293016	-0.864261
Ciclev10025907m.g	scaffold_7:5031820-5034939	6.51629	3.5832	-0.8628
Ciclev10025225m.g	scaffold_7:19613448-19617030	3.21979	1.77052	-0.862792
Ciclev10014421m.g	scaffold_2:24613404-24618480	38.1799	20.9997	-0.862444
Ciclev10000783m.g	scaffold_5:42445213-42449994	1.23553	0.679574	-0.862425
Ciclev10007078m.g	scaffold_9:4605636-4609344	0.294119	0.161774	-0.862423
Ciclev10020789m.g	scaffold_3:31133565-31137650	69.1263	38.0351	-0.861902
Ciclev10014656m.g	scaffold_2:11835630-11838519	1.12634	0.619779	-0.861814
-	scaffold_1:3504722-3506513	51.6413	28.4224	-0.861496
Ciclev10000358m.g	scaffold_5:37849212-37853413	18.7403	10.3222	-0.860393
Ciclev10000741m.g	scaffold_5:20581813-20588381	1.03305	0.569146	-0.860042
Ciclev10032229m.g	scaffold_4:23504841-23506977	2.89233	1.59411	-0.859482
Ciclev10015466m.g	scaffold_2:9074033-9077904	12.6648	6.98081	-0.85936
Ciclev10023952m.g	scaffold_3:803190-806215	65.4696	36.0913	-0.859174
Ciclev10021034m.g	scaffold_3:18593395-18602254	79.5388	43.8487	-0.859126
Ciclev10017362m.g	scaffold_2:10912561-10913785	7.99653	4.40912	-0.858882
Ciclev10004183m.g	scaffold_9:27122643-27132280	0.121625	0.067066	-0.858792
-	scaffold_3:14200066-14202183	154.716	85.3306	-0.858485
Ciclev10007205m.g	scaffold_9:6519554-6521304	0.754146	0.416147	-0.857751
Ciclev10015378m.g	scaffold_2:31954701-31957411	68.1133	37.5945	-0.857414
Ciclev10020159m.g	scaffold_3:6353186-6356406	8.27606	4.56834	-0.857273
Ciclev10014630m.g	scaffold_2:11715434-11718336	1.37287	0.757906	-0.857099
Ciclev10019895m.g	scaffold_3:32101494-32108155	74.3654	41.0579	-0.856971
Ciclev10000748m.g	scaffold_5:42894358-42897956	0.362369	0.200235	-0.855764

-	scaffold_22:39810-40274	26.4004	14.5892	-0.855662
Ciclev10025268m.g	scaffold_7:112220-115241	17.6911	9.77841	-0.855349
Ciclev10033051m.g	scaffold_4:11203437-11207225	32.631	18.0397	-0.855063
Ciclev10031245m.g	scaffold_4:21300401-21302840	1.1619	0.642373	-0.854999
Ciclev10028441m.g	scaffold_8:14621891-14628467	28.6333	15.8324	-0.854816
Ciclev10026049m.g	scaffold_7:5713132-5714728	18.0735	9.9951	-0.854583
-	scaffold_3:2744128-2744770	12.4697	6.8991	-0.853946
Ciclev10012751m.g	scaffold_6:5286319-5290954	10.5559	5.84304	-0.853261
Ciclev10014768m.g	scaffold_2:16833386-16839435	9.72929	5.38595	-0.853133
Ciclev10005618m.g	scaffold_9:383810-387133	3.34565	1.8523	-0.85297
Ciclev10001261m.g	scaffold_5:19027226-19032242	10.1875	5.64042	-0.852929
Ciclev10028649m.g	scaffold_8:2261181-2264145	20.8003	11.5188	-0.852616
Ciclev10027342m.g	scaffold_7:17857142-17860999	0.563416	0.312049	-0.852428
Ciclev10025480m.g	scaffold_7:5377222-5381234	55.8568	30.949	-0.851841
Ciclev10015142m.g	scaffold_2:35174732-35180227	2.37631	1.31676	-0.851729
Ciclev10022521m.g	scaffold_3:1044993-1045951	165.241	91.6671	-0.850094
-	scaffold_4:4825832-5025734	19.6373	10.8947	-0.849975
Ciclev10013514m.g	scaffold_6:20142308-20143274	1.1229	0.623011	-0.849895
Ciclev10022904m.g	scaffold_3:36608196-36611090	38.054	21.1134	-0.849892
Ciclev10013722m.g	scaffold_6:16720503-16724245	11.6007	6.43757	-0.849625
Ciclev10010390m.g	scaffold_1:26795140-26797276	0.93494	0.518985	-0.849182
Ciclev10024518m.g	scaffold_3:21678085-21719797	10.0852	5.59956	-0.848861
-	scaffold_2:32202525-32202887	18.0508	10.0229	-0.848758
Ciclev10020781m.g	scaffold_3:3860874-3862460	2.1109	1.17267	-0.848063
Ciclev10020882m.g	scaffold_3:39023498-39027970	43.3328	24.0814	-0.847537
Ciclev10033952m.g	scaffold_4:25022320-25025779	5.1634	2.87053	-0.847006
Ciclev10008718m.g	scaffold_1:24462316-24463518	6.7674	3.76335	-0.846582
Ciclev10031391m.g	scaffold_4:21170904-21177408	4.75118	2.64248	-0.846393
Ciclev10000060m.g	scaffold_5:27441741-27448756	7.51595	4.18152	-0.84593
Ciclev10016992m.g	scaffold_2:23699696-23700842	22.8736	12.7305	-0.84539
-	scaffold_9:9654972-9655114	3739	2081.99	-0.844691
Ciclev10017089m.g	scaffold_2:13459490-13463120	41.0414	22.8611	-0.844185
Ciclev10023172m.g	scaffold_3:47310123-47325365	1.25011	0.696527	-0.843807
Ciclev10026061m.g	scaffold_7:2709422-2710648	1.84286	1.02694	-0.843597
Ciclev10003527m.g	scaffold_5:38974277-38976945	0.285849	0.159457	-0.842084
Ciclev10019673m.g	scaffold_3:2809301-2815125	95.9465	53.5255	-0.842003
Ciclev10031452m.g	scaffold_4:7379155-7388483	6.32592	3.52925	-0.841915
Ciclev10008875m.g	scaffold_1:28868796-28872798	109.057	60.8494	-0.841763
Ciclev10032941m.g	scaffold_4:1461988-1463323	1.28236	0.715626	-0.841521
Ciclev10032655m.g	scaffold_4:22558326-22560787	11.8234	6.59853	-0.841426
Ciclev10011665m.g	scaffold_6:12779845-12784130	20.7256	11.5681	-0.841258
Ciclev10022568m.g	scaffold_3:49483584-49489946	68.0397	37.98	-0.841136
Ciclev10012367m.g	scaffold_6:23523307-23526773	27.6449	15.4394	-0.840398
Ciclev10017272m.g	scaffold_2:26329480-26329867	9.06292	5.06358	-0.83982
Ciclev10018024m.g	scaffold_2:20347678-20350137	0.41422	0.231459	-0.83964
Ciclev10007694m.g	scaffold_1:5100429-5104636	6.90805	3.86097	-0.839314
Ciclev10033071m.g	scaffold_4:23439180-23445715	264.846	148.025	-0.839313
Ciclev10030976m.g	scaffold_4:23245917-23248132	13.9113	7.781	-0.838226
Ciclev10031266m.g	scaffold_4:5949844-5951969	4.10718	2.29736	-0.83817
Ciclev10013044m.g	scaffold_6:20039906-20042958	38.002	21.2674	-0.837431
Ciclev10008884m.g	scaffold_1:8865235-8868708	27.5169	15.401	-0.837297
Ciclev10001376m.g	scaffold_5:29867748-29870487	0.548732	0.30714	-0.837205
Ciclev10015676m.g	scaffold_2:8720763-8725932	71.3227	39.9266	-0.837011
Ciclev10018759m.g	scaffold_3:38502085-38505211	1.5516	0.869203	-0.835996
Ciclev10027070m.g	scaffold_7:3909921-3913108	7.21331	4.04089	-0.835986
Ciclev10029424m.g	scaffold_8:17836888-17838664	46.8775	26.2623	-0.835901

Ciclev10032232m.g	scaffold_4:23393841-23395020	23.9301	13.4085	-0.835677
-	scaffold_7:15560101-15560539	242.08	135.661	-0.835479
Ciclev10024310m.g	scaffold_3:2310797-2312390	287.144	160.92	-0.835427
Ciclev10009808m.g	scaffold_1:326165-328950	17.3341	9.71662	-0.835085
Ciclev10028539m.g	scaffold_8:232122-235806	13.0416	7.31242	-0.834696
Ciclev10030031m.g	scaffold_8:16550333-16550840	14.3771	8.06128	-0.834689
Ciclev10028107m.g	scaffold_8:13190040-13194829	51.689	28.9937	-0.834117
Ciclev10020889m.g,Ciclev10024506m.g	scaffold_3:20866111-20870269	4.28949	2.40733	-0.833374
Ciclev10002937m.g	scaffold_5:30526755-30528544	45.8389	25.7281	-0.833227
Ciclev10001640m.g	scaffold_5:34472071-34474847	11.7534	6.59753	-0.833075
Ciclev10033714m.g	scaffold_4:22542107-22543097	2.72525	1.5298	-0.833044
-	scaffold_4:9880494-10159290	6.88027	3.8627	-0.832855
Ciclev10025330m.g	scaffold_7:13581066-13583669	616.138	346.094	-0.832089
Ciclev10027754m.g	scaffold_8:18739959-18745697	0.74459	0.418255	-0.832064
Ciclev10015390m.g	scaffold_2:28120146-28126203	29.2301	16.4228	-0.83176
Ciclev10013263m.g	scaffold_6:17663759-17664483	5.37463	3.0205	-0.831379
Ciclev10022082m.g	scaffold_3:46124030-46130078	57.0924	32.0888	-0.831229
Ciclev10029184m.g	scaffold_8:2788251-2799301	19.9369	11.2057	-0.831212
Ciclev10027041m.g	scaffold_7:13916806-14069972	0.208746	0.117331	-0.831156
Ciclev10025061m.g	scaffold_7:5970613-5982576	73.7839	41.4837	-0.830761
Ciclev10001283m.g	scaffold_5:38352720-38355754	15.7972	8.88756	-0.829809
Ciclev10007864m.g	scaffold_1:9581381-9583491	9.02061	5.07714	-0.82921
Ciclev10018896m.g	scaffold_3:42965236-42972484	8.90784	5.01453	-0.828961
Ciclev10010975m.g	scaffold_6:25331672-25338353	9.01643	5.07637	-0.82876
Ciclev10014121m.g	scaffold_2:25179667-25188103	0.156709	0.0882675	-0.828134
Ciclev10012131m.g	scaffold_6:19924433-19926818	0.454109	0.255783	-0.82812
Ciclev10013983m.g	scaffold_1232:2489-4223	149.067	83.9822	-0.827809
Ciclev10016122m.g	scaffold_2:22428192-22431792	64.8922	36.5639	-0.827626
Ciclev10008839m.g	scaffold_1:4851969-4855156	81.2395	45.7848	-0.827312
Ciclev10014761m.g	scaffold_2:12897886-12901322	11.0811	6.24643	-0.826999
Ciclev10011774m.g	scaffold_6:24375408-24378738	16.3159	9.19973	-0.826613
-	scaffold_6:7316678-7318658	5.23515	2.95255	-0.82627
Ciclev10027750m.g,Ciclev10027752m.g,Ciclev10027755m.g,Ciclev10027760m.g,Ciclev10027763m.g	scaffold_8:20789192-20863026	2.09273	1.18081	-0.825607
Ciclev10000812m.g	scaffold_5:37693881-37699178	33.3937	18.8451	-0.825384
-	scaffold_2:20443950-20444122	298.188	168.343	-0.824823
Ciclev10019340m.g	scaffold_3:3654153-3657882	2.89443	1.63439	-0.824524
Ciclev10007435m.g	scaffold_1:3285600-3291534	9.90466	5.59427	-0.824159
Ciclev10026595m.g	scaffold_7:8970861-8972727	18.7423	10.5878	-0.823888
Ciclev10021359m.g	scaffold_3:48113496-48116009	47.5365	26.8548	-0.823857
Ciclev10012482m.g,Ciclev10012795m.g	scaffold_6:25413890-25421486	18.0043	10.172	-0.823746
Ciclev10009612m.g	scaffold_1:4662424-4666008	4.55988	2.57629	-0.823701
Ciclev10021082m.g	scaffold_3:49526004-49528353	8.31486	4.69823	-0.823575
Ciclev10004707m.g	scaffold_9:25094126-25099067	40.5683	22.9303	-0.823097
Ciclev10000234m.g	scaffold_5:33258591-33265332	1.95304	1.10417	-0.822757
Ciclev10000213m.g	scaffold_5:6024578-6027417	4.34875	2.45866	-0.822726
-	scaffold_8:21551587-21554549	25.005	14.1378	-0.82266
Ciclev10028556m.g	scaffold_8:3625135-3628623	55.4232	31.3399	-0.82249
Ciclev10000235m.g	scaffold_5:119680-126181	744.651	421.116	-0.822348
Ciclev10009752m.g	scaffold_1:27724037-27727392	59.7551	33.7953	-0.822237
Ciclev10012659m.g	scaffold_6:18014433-18025689	1.14782	0.649287	-0.821966
Ciclev10018604m.g	scaffold_3:29031693-29035908	2.09978	1.18828	-0.821367

Ciclev10021452m.g	scaffold_3:23701500-23704547	3703.97	2096.18	-0.821311
Ciclev10011471m.g	scaffold_6:16445358-16450818	60.6377	34.3308	-0.820711
Ciclev10002390m.g	scaffold_5:34866518-34875003	3.38381	1.91593	-0.820603
Ciclev10007331m.g	scaffold_1:7390097-7401504	1.79103	1.01413	-0.820544
Ciclev10027564m.g	scaffold_7:5743041-5743449	30.2986	17.1568	-0.820467
Ciclev10030096m.g	scaffold_8:1823963-1885505	360.513	204.201	-0.820065
Ciclev10001371m.g	scaffold_5:29050806-29055963	6.90442	3.91304	-0.819229
Ciclev10000996m.g	scaffold_5:28289811-28536377	3.73055	2.11479	-0.818876
Ciclev10017829m.g	scaffold_2:34511086-34512379	1.1833	0.670903	-0.818639
Ciclev10011688m.g	scaffold_6:17874577-17877758	1.91247	1.08439	-0.818556
Ciclev10022857m.g,Ciclev10024365m.g	scaffold_3:31467767-31474444	45.9433	26.0534	-0.818384
Ciclev10023201m.g	scaffold_3:4670535-4674390	3.62036	2.05309	-0.81834
Ciclev10030303m.g	scaffold_8:20268210-20272901	2.15891	1.22433	-0.81831
Ciclev10009578m.g	scaffold_1:2528277-2531011	33.4474	18.9774	-0.817611
Ciclev10004926m.g	scaffold_9:8504198-8507952	9.29621	5.2778	-0.816706
Ciclev10016506m.g	scaffold_2:36319743-36321247	16.5459	9.39834	-0.815996
Ciclev10015813m.g	scaffold_2:17324417-17328067	64.8938	36.8674	-0.815735
Ciclev10032910m.g	scaffold_4:21573069-21575423	6.9122	3.92731	-0.815604
Ciclev10031775m.g	scaffold_4:17375041-17376468	0.561037	0.318808	-0.815408
Ciclev10017421m.g	scaffold_2:32982212-32985696	44.1511	25.089	-0.815391
Ciclev10002996m.g	scaffold_5:2206325-2209110	58.3115	33.1461	-0.814941
Ciclev10012274m.g	scaffold_6:16393308-16395378	261.568	148.717	-0.814615
Ciclev10008644m.g	scaffold_1:18405769-18409946	1.42253	0.808849	-0.814512
Ciclev10025242m.g	scaffold_7:133318-138971	14.5314	8.26376	-0.814307
Ciclev10004257m.g	scaffold_9:28912262-28917003	4.86732	2.76914	-0.813693
Ciclev10016498m.g	scaffold_2:36114761-36117093	1.67461	0.952844	-0.813512
Ciclev10017411m.g	scaffold_2:36003941-36005055	5.34969	3.04514	-0.812949
Ciclev10030990m.g	scaffold_4:11435795-11445236	13.8902	7.90862	-0.812572
Ciclev10004527m.g	scaffold_9:25578970-25584492	14.6267	8.32858	-0.81246
Ciclev10003334m.g	scaffold_5:34574463-34591686	84.9069	48.3473	-0.812446
Ciclev10005215m.g	scaffold_9:187478-190288	79.9576	45.5305	-0.8124
Ciclev10008822m.g	scaffold_1:4163201-4166973	9.0514	5.15524	-0.812101
Ciclev10007316m.g	scaffold_1:2899662-2905240	0.954425	0.543668	-0.811906
Ciclev10024596m.g	scaffold_3:3315694-3318592	2.98209	1.69902	-0.811616
Ciclev10016078m.g	scaffold_2:23002424-23005703	10.3458	5.89679	-0.811047
Ciclev10018627m.g	scaffold_3:22582145-22586403	2.93276	1.67176	-0.810893
Ciclev10011567m.g	scaffold_6:21352984-21358482	42.7888	24.3981	-0.810467
Ciclev10033036m.g	scaffold_4:11749785-11752497	43.7567	24.9565	-0.810089
Ciclev10016496m.g	scaffold_2:1099014-1099731	2.37644	1.35588	-0.809569
Ciclev10001982m.g	scaffold_5:33338363-33342254	5.14609	2.93628	-0.809489
Ciclev10008448m.g	scaffold_1:16657240-16660189	57.5429	32.8344	-0.80943
Ciclev10006103m.g	scaffold_9:5531435-5535535	28.2252	16.1144	-0.808634
Ciclev10015157m.g	scaffold_2:31851697-31857541	52.7779	30.138	-0.808349
Ciclev10023870m.g	scaffold_3:32937629-32939468	2.31912	1.32431	-0.808332
Ciclev10029717m.g	scaffold_8:15186678-15187280	35.5691	20.3146	-0.808106
Ciclev10025233m.g	scaffold_7:7960152-7964339	64.124	36.6239	-0.808078
Ciclev10022669m.g	scaffold_3:2221594-2224612	149.05	85.1397	-0.807894
Ciclev10006560m.g	scaffold_9:28035354-28036664	204.151	116.739	-0.806342
Ciclev10033212m.g,Ciclev10033455m.g	scaffold_4:19435713-19438071	13.5674	7.76617	-0.804868
Ciclev10009314m.g	scaffold_1:25334136-25337522	51.2354	29.3358	-0.804478
Ciclev10000654m.g	scaffold_5:12677413-12680761	95.2276	54.5309	-0.804304
Ciclev10030742m.g	scaffold_4:25267552-25269940	1.1618	0.665326	-0.804233
Ciclev10028299m.g	scaffold_8:20879068-20883824	10.9026	6.244	-0.804129
-	scaffold_8:5718378-5718783	22.7903	13.0536	-0.803975

Ciclev10029176m.g	scaffold_8:18758592-18762878	30.7096	17.591	-0.803855
Ciclev10010499m.g	scaffold_1:477532-481593	6.9966	4.00805	-0.803754
Ciclev10027047m.g	scaffold_7:13361298-13363974	0.371559	0.212871	-0.80361
Ciclev10027972m.g	scaffold_8:22475921-22481806	0.670982	0.384499	-0.803294
Ciclev10020206m.g	scaffold_3:45205979-45211054	5.12376	2.93736	-0.802683
Ciclev10021645m.g	scaffold_3:7170824-7174786	8.43237	4.83592	-0.802147
Ciclev10027745m.g	scaffold_8:24543-37463	153.534	88.0514	-0.802145
Ciclev10004543m.g	scaffold_9:28456944-28459088	4.81332	2.76081	-0.801944
Ciclev10014120m.g	scaffold_2:25410685-25415425	4.75047	2.72608	-0.801244
Ciclev10018721m.g	scaffold_3:31161658-31168396	1.50351	0.86296	-0.800971
Ciclev10017521m.g	scaffold_2:33314588-33316267	22.0304	12.6451	-0.800924
Ciclev10031014m.g	scaffold_4:25500600-25505812	39.5599	22.7181	-0.800196
Ciclev10032269m.g	scaffold_4:23439180-23445715	4.66236	2.67754	-0.800152
Ciclev10015859m.g	scaffold_2:31402026-31406669	19.2473	11.0545	-0.80003
Ciclev10019338m.g	scaffold_3:1813569-1816531	3.35986	1.92997	-0.799826
Ciclev10008681m.g	scaffold_1:487183-489198	24.1025	13.8497	-0.799325
Ciclev10032455m.g	scaffold_4:22658515-22662302	12.2007	7.01181	-0.799104
Ciclev10031838m.g,Ciclev10032311m.g	scaffold_4:3548157-3560087	6.79905	3.90812	-0.798858
Ciclev10006654m.g	scaffold_9:28427765-28431247	258.703	148.738	-0.798525
Ciclev10010160m.g	scaffold_1:24240285-24244808	22.5834	12.9846	-0.798462
Ciclev10013660m.g	scaffold_6:20416758-20421738	31.5437	18.1427	-0.797961
Ciclev10015940m.g	scaffold_2:20124169-20127095	11.5109	6.62152	-0.797769
Ciclev10027818m.g	scaffold_8:21057786-21063176	23.1993	13.3466	-0.797609
Ciclev10024215m.g	scaffold_3:21873837-21877515	18.2437	10.496	-0.797561
Ciclev10023722m.g	scaffold_3:36363008-36553812	5.29136	3.04453	-0.797417
Ciclev10026745m.g	scaffold_7:14905767-14906752	47.1707	27.1474	-0.797079
Ciclev10000566m.g	scaffold_5:42555316-42559854	3.30093	1.89986	-0.796977
Ciclev10005370m.g	scaffold_9:27734481-27736035	4.44188	2.55665	-0.796914
Ciclev10002226m.g	scaffold_5:42675741-42682366	9.22294	5.30888	-0.796818
Ciclev10024288m.g,Ciclev10024291m.g	scaffold_3:50855159-50859573	156.444	90.0669	-0.796575
Ciclev10016357m.g	scaffold_2:35803511-35805985	16.6415	9.58458	-0.795996
-	scaffold_4:3187218-3188593	4.36084	2.51189	-0.795831
Ciclev10026925m.g	scaffold_7:4819411-4820934	0.39805	0.229326	-0.795551
Ciclev10019396m.g	scaffold_3:43795212-43800518	20.6675	11.91	-0.795189
Ciclev10020426m.g	scaffold_3:39339397-39346894	15.1298	8.72035	-0.794939
-	scaffold_2:11334665-11336191	5.35638	3.0875	-0.794821
Ciclev10007503m.g	scaffold_1:1778447-1780995	2.24707	1.29632	-0.793619
Ciclev10007067m.g	scaffold_9:1823937-1827527	36.6874	21.1652	-0.793594
Ciclev10011951m.g	scaffold_6:12700075-12705209	25.4199	14.6652	-0.793566
Ciclev10002960m.g	scaffold_5:33183579-33184565	34.6657	19.9995	-0.793545
Ciclev10019792m.g	scaffold_3:41716542-41719311	2.24641	1.29611	-0.793436
Ciclev10015659m.g	scaffold_2:35772567-35774822	3.98026	2.29649	-0.793431
Ciclev10016279m.g	scaffold_2:843354-853044	96.1018	55.4483	-0.79342
Ciclev10022588m.g	scaffold_3:50257480-50262357	15.1673	8.75724	-0.792418
Ciclev10008256m.g	scaffold_1:26494069-26497694	8.38735	4.84304	-0.792302
Ciclev10005220m.g	scaffold_9:29969028-29970745	0.429965	0.248316	-0.792044
Ciclev10011697m.g	scaffold_6:25218607-25221458	150.688	87.0335	-0.791926
-	scaffold_1:18387966-18388376	17.9808	10.3855	-0.791887
Ciclev10029211m.g	scaffold_8:5581254-5582415	15.5515	8.98453	-0.791535
Ciclev10015132m.g	scaffold_2:17353079-17356820	7.75847	4.4824	-0.791501
Ciclev10027536m.g	scaffold_7:17222438-17223308	161.705	93.452	-0.79107
Ciclev10029296m.g	scaffold_8:10520203-10524265	77.9074	45.0408	-0.790526
Ciclev10002870m.g	scaffold_5:39412818-39414334	337.954	195.405	-0.790357
Ciclev10031562m.g	scaffold_4:8408632-8414712	2.35639	1.36263	-0.790188

Ciclev10031290m.g	scaffold_4:15917251-15921592	8.26167	4.77753	-0.79017
Ciclev10002424m.g	scaffold_5:42898493-42904653	33.3705	19.3075	-0.789409
Ciclev10023059m.g	scaffold_3:44863346-44865900	134.507	77.8329	-0.78923
Ciclev10032367m.g	scaffold_4:8400377-8404853	60.3923	34.9483	-0.789142
Ciclev10030325m.g	scaffold_8:8700651-8700897	12.2121	7.06811	-0.78892
Ciclev10033991m.g	scaffold_4:19413411-19416331	6.17348	3.5734	-0.788786
Ciclev10021834m.g	scaffold_3:6000265-6002833	5.60499	3.24552	-0.788264
Ciclev10019566m.g	scaffold_3:4633465-4637285	8.01773	4.64636	-0.787094
Ciclev10031630m.g	scaffold_4:20693083-20697495	11.1631	6.47089	-0.786702
Ciclev10022027m.g	scaffold_3:1675854-1679149	22.7226	13.1757	-0.786251
Ciclev10015265m.g,Ciclev10018329m.g	scaffold_2:26303430-26325625	6.6289	3.84399	-0.786165
Ciclev10033572m.g	scaffold_4:2013663-2017905	93.3358	54.1271	-0.786079
Ciclev10016940m.g	scaffold_2:16532094-16532954	17.2695	10.015	-0.786069
Ciclev10011822m.g	scaffold_6:19586147-19590349	29.3929	17.0507	-0.785634
Ciclev10015247m.g	scaffold_2:10050551-10055583	106.037	61.5408	-0.784957
Ciclev10006876m.g	scaffold_9:9543643-9544285	3.7629	2.18401	-0.784865
Ciclev10030967m.g	scaffold_4:22486236-22491982	14.0807	8.17251	-0.784864
Ciclev10012858m.g	scaffold_6:17376854-17377530	3.75926	2.18245	-0.7845
Ciclev10014314m.g	scaffold_2:13969341-13975357	13.6244	7.91277	-0.783939
Ciclev10014902m.g	scaffold_2:23923128-23928086	33.7949	19.629	-0.783819
Ciclev10015930m.g	scaffold_2:8991164-8994650	7.31532	4.24959	-0.783596
Ciclev10002505m.g	scaffold_5:25191868-25193363	68.707	39.9154	-0.783512
Ciclev10033542m.g	scaffold_4:17927956-17930419	714.272	414.984	-0.783416
Ciclev10025232m.g	scaffold_7:7898244-7901186	46.8333	27.2154	-0.783109
-	scaffold_9:28497456-28497713	400.601	232.856	-0.782726
Ciclev10001614m.g,Ciclev10002486m.g,Ciclev10003405m.g	scaffold_5:5916191-5944275	100.531	58.4581	-0.78217
Ciclev10014350m.g	scaffold_2:12688781-12742589	10.4909	6.10425	-0.781249
Ciclev10031358m.g,Ciclev10031503m.g,Ciclev10032448m.g	scaffold_4:16817583-16984703	3.24894	1.89049	-0.781211
-	scaffold_7:19950882-20044587	6.88727	4.00894	-0.78071
Ciclev10004930m.g,Ciclev10005612m.g	scaffold_9:20890036-21029395	4.20388	2.44745	-0.780442
Ciclev10028952m.g	scaffold_8:18724347-18726706	18.9595	11.0412	-0.780024
Ciclev10009475m.g	scaffold_1:4259474-4260548	7.41321	4.31722	-0.779997
Ciclev10010605m.g	scaffold_1:9391681-9396381	37.4576	21.8156	-0.779901
Ciclev10009717m.g	scaffold_1:973125-975277	12.8043	7.4576	-0.77984
Ciclev10004502m.g	scaffold_9:29392884-29395387	28.5319	16.6186	-0.779775
Ciclev10021138m.g	scaffold_3:7751030-7755076	13.456	7.83764	-0.779756
Ciclev10031833m.g	scaffold_4:19127245-19130791	19.1187	11.1377	-0.77954
Ciclev10006705m.g	scaffold_9:30429001-30434566	56.3691	32.8443	-0.779262
Ciclev10016677m.g	scaffold_2:316657-319518	4.69781	2.73729	-0.779244
Ciclev10000310m.g	scaffold_5:19136105-19146196	22.1601	12.9145	-0.778971
Ciclev10026939m.g	scaffold_7:12320804-12323366	0.875183	0.510113	-0.778769
Ciclev10003916m.g	scaffold_5:39616555-39618657	4.08308	2.38049	-0.778398
Ciclev10025539m.g	scaffold_7:3578162-3583653	0.701042	0.408754	-0.778266
Ciclev10015369m.g	scaffold_2:35761243-35766725	13.447	7.84214	-0.777965
Ciclev10001380m.g	scaffold_5:38491114-38492945	11.2762	6.57661	-0.777865
Ciclev10001935m.g	scaffold_5:41842465-41844878	19.1039	11.147	-0.777208
Ciclev10029065m.g	scaffold_8:2145881-2147154	356.13	207.802	-0.777195
Ciclev10002100m.g	scaffold_5:32903812-32907034	35.5824	20.7639	-0.777089
Ciclev10015104m.g	scaffold_2:33617759-33623310	8.56233	4.99706	-0.776923
Ciclev10019406m.g	scaffold_3:24840385-24845254	32.1118	18.7455	-0.776562

Ciclev10028986m.g	scaffold_8:3102685-3107816	31.5138	18.4004	-0.776251
Ciclev10031741m.g	scaffold_4:22131141-22133014	15.8452	9.25369	-0.775946
Ciclev10021647m.g	scaffold_3:19266870-19273247	311.557	181.98	-0.775716
Ciclev10021543m.g	scaffold_3:44483703-44485553	26.9904	15.7652	-0.775697
Ciclev10001564m.g	scaffold_5:40780715-40782937	3.82485	2.23419	-0.775652
Ciclev10009915m.g	scaffold_1:25427130-25431061	2423.9	1415.9	-0.775613
Ciclev10007936m.g	scaffold_1:898373-902695	49.9358	29.1775	-0.775221
Ciclev10017102m.g	scaffold_2:35920185-35922196	72.1227	42.1735	-0.774115
Ciclev10009045m.g	scaffold_1:3665140-3672863	40.7359	23.8217	-0.774027
Ciclev10010092m.g	scaffold_1:6903803-6918085	1.36463	0.798086	-0.773894
Ciclev10003589m.g	scaffold_5:26810282-26812583	2.71295	1.58711	-0.77346
Ciclev10013834m.g,Ciclev10013866m.g	scaffold_6:23555036-23557575	729.64	426.919	-0.773224
Ciclev10025361m.g	scaffold_7:587249-591254	9.02743	5.28259	-0.77307
Ciclev10005759m.g	scaffold_9:2743203-2746131	274.501	160.697	-0.77247
Ciclev10002646m.g	scaffold_5:27814563-27818048	94.0028	55.0358	-0.772334
Ciclev10030659m.g	scaffold_4:18006649-18012850	14.5744	8.53303	-0.772308
Ciclev10029369m.g,Ciclev10029534m.g	scaffold_8:2655906-2660636	1289.69	755.736	-0.771069
Ciclev10017115m.g	scaffold_2:3543305-3544895	2.34423	1.37432	-0.770397
Ciclev10026097m.g	scaffold_7:4667364-4670010	86.2305	50.5558	-0.770321
Ciclev10030589m.g	scaffold_4:21120134-21127140	56.5796	33.1719	-0.770318
Ciclev10022334m.g	scaffold_3:2679436-2681877	25.8374	15.1546	-0.769708
Ciclev10020629m.g	scaffold_3:18619217-18623445	5.56322	3.26403	-0.769264
Ciclev10017087m.g	scaffold_2:33443605-33444972	58.1463	34.1239	-0.768902
Ciclev10015406m.g,Ciclev10015510m.g	scaffold_2:30142886-30148379	13.6023	7.98419	-0.768629
Ciclev10003627m.g	scaffold_5:2534144-2731098	70.469	41.3673	-0.768499
Ciclev10008522m.g	scaffold_1:2664059-2666819	7.78229	4.57011	-0.767964
Ciclev10033764m.g	scaffold_4:21749981-21754015	8.30676	4.87924	-0.76763
Ciclev10021104m.g	scaffold_3:40973422-40976172	39.4187	23.1539	-0.767625
Ciclev10025938m.g	scaffold_7:8210740-8212523	115.189	67.6628	-0.767567
-	scaffold_8:21397943-21398143	79.8965	46.9329	-0.767532
Ciclev10012313m.g	scaffold_6:21720398-21723489	28.5291	16.7662	-0.76688
Ciclev10027618m.g	scaffold_7:20360085-20395370	3.60878	2.12197	-0.766106
Ciclev10015321m.g	scaffold_2:7273711-7336406	181.379	106.66	-0.765986
Ciclev10017900m.g	scaffold_2:8387596-8392103	2.70792	1.59271	-0.765695
Ciclev10023762m.g,Ciclev10024229m.g	scaffold_3:46509964-46513645	32.3496	19.0334	-0.765213
Ciclev10011992m.g	scaffold_6:16820951-16822532	3.4864	2.05137	-0.765154
Ciclev10030821m.g	scaffold_4:6290058-6294163	24.8219	14.6115	-0.764508
Ciclev10002689m.g	scaffold_5:33968149-33969456	239.676	141.115	-0.764219
Ciclev10000008m.g	scaffold_5:12226123-12245736	6.02689	3.5487	-0.764125
Ciclev10031420m.g	scaffold_4:16817583-16984703	2.32515	1.36921	-0.763982
Ciclev10015570m.g	scaffold_2:7250530-7255818	24.9898	14.717	-0.763856
Ciclev10018391m.g	scaffold_2:843354-853044	3.64238	2.14514	-0.763814
Ciclev10005631m.g	scaffold_9:192171-196670	48.1291	28.3505	-0.763534
Ciclev10021295m.g	scaffold_3:44677910-44678922	5.62496	3.31374	-0.763383
Ciclev10001823m.g	scaffold_5:37449700-37453928	46.268	27.2594	-0.763259
Ciclev10033477m.g	scaffold_4:2018900-2019648	1461.17	860.885	-0.763235
Ciclev10024954m.g	scaffold_7:18990942-18994628	0.348056	0.205089	-0.76307
Ciclev10028132m.g	scaffold_8:5985062-5987794	2.6403	1.55588	-0.762973
Ciclev10013062m.g	scaffold_6:18750204-18752251	22.683	13.3671	-0.762927
-	scaffold_4:9880494-10159290	6.92295	4.08511	-0.761012
Ciclev10019816m.g	scaffold_3:43654586-43656842	53.6292	31.6465	-0.76097
Ciclev10019387m.g	scaffold_3:42274795-42279823	1.9034	1.12331	-0.760818

Ciclev10001805m.g	scaffold_5:36845846-36847355	5.3916	3.1846	-0.7596
Ciclev10016134m.g	scaffold_2:35407310-35413897	46046.1	27199.3	-0.75951
Ciclev10019122m.g	scaffold_3:47470897-47475857	25.875	15.287	-0.759253
Ciclev10005151m.g	scaffold_9:27270413-27276140	24.6088	14.5415	-0.759004
Ciclev10031732m.g	scaffold_4:18791127-18793469	2.03498	1.20274	-0.758684
-	scaffold_1232:108-1849	59.0739	34.9168	-0.758599
Ciclev10002303m.g	scaffold_5:43186617-43190241	122.209	72.2442	-0.758394
Ciclev10029703m.g	scaffold_8:2020264-2030440	26.4592	15.6433	-0.758224
Ciclev10030554m.g	scaffold_4:1240598-1244941	31.7789	18.7915	-0.757985
Ciclev10004419m.g	scaffold_9:396489-401539	1.83462	1.08499	-0.757798
-	scaffold_2:5368058-5378200	21.0027	12.4215	-0.757731
Ciclev10007366m.g	scaffold_1:28710366-28713767	1.5059	0.89104	-0.757066
Ciclev10015358m.g	scaffold_2:15477859-15615778	2.14925	1.27184	-0.756921
Ciclev10028916m.g	scaffold_8:24148646-24152511	10.9274	6.46721	-0.756735
Ciclev10002793m.g	scaffold_5:8435122-8435971	4780.26	2829.37	-0.756611
Ciclev10007378m.g	scaffold_1:5539850-5542980	39.4555	23.3546	-0.756518
Ciclev10004686m.g	scaffold_9:23374166-23382174	124.379	73.6408	-0.756166
Ciclev10005680m.g	scaffold_9:8416603-8419944	18.2103	10.7842	-0.755829
Ciclev10023136m.g	scaffold_3:42882908-42885640	12.9423	7.6663	-0.755488
Ciclev10006442m.g	scaffold_9:4001515-4003633	1.61055	0.954243	-0.755125
Ciclev10002853m.g	scaffold_5:41654673-41656808	16.84	9.98153	-0.754563
Ciclev10031549m.g	scaffold_4:17138365-17142326	28.1909	16.7122	-0.754326
Ciclev10033072m.g	scaffold_4:2127320-2129342	89.8039	53.2416	-0.754225
Ciclev10020633m.g	scaffold_3:1540173-1544282	51.2148	30.3669	-0.754059
Ciclev10023082m.g	scaffold_3:20987827-20989845	1.48903	0.883784	-0.752611
Ciclev10031432m.g	scaffold_4:15087930-15266082	0.907199	0.538543	-0.752356
Ciclev10031679m.g	scaffold_4:702796-704974	28.3645	16.8421	-0.752016
Ciclev10026493m.g	scaffold_7:8504089-8504955	3.60225	2.13913	-0.751877
Ciclev10033422m.g	scaffold_4:19340762-19345467	5.36008	3.18349	-0.751648
Ciclev10015858m.g	scaffold_2:10136091-10141548	9.93687	5.9026	-0.751441
Ciclev10001722m.g	scaffold_5:20746474-20747919	29.466	17.5053	-0.751259
Ciclev10007097m.g	scaffold_9:2412444-2414535	36.6099	21.7503	-0.751197
Ciclev10027203m.g	scaffold_7:5218819-5219128	1.82239	1.08285	-0.751004
Ciclev10000751m.g	scaffold_5:35074023-35076069	8.98461	5.33949	-0.750753
Ciclev10003962m.g	scaffold_5:38707302-38707548	10.52	6.2537	-0.750359
Ciclev10020507m.g	scaffold_3:4637681-4641334	4.89176	2.90932	-0.749671
Ciclev10000195m.g	scaffold_5:42861981-42867071	6.49752	3.86612	-0.749002
Ciclev10007510m.g	scaffold_1:24480590-24484723	3.08321	1.83457	-0.748986
-	scaffold_3:46141996-46142496	8.76947	5.21812	-0.748959
Ciclev10001087m.g	scaffold_5:28289811-28536377	2.19539	1.30636	-0.748929
Ciclev10006977m.g	scaffold_9:28791291-28795010	67.2492	40.0167	-0.748914
-	scaffold_9:27223359-27223540	291.765	173.708	-0.748142
Ciclev10001479m.g	scaffold_5:35159205-35164384	53.4538	31.8259	-0.748089
Ciclev10028073m.g	scaffold_8:3533638-3539841	5.69019	3.38798	-0.748053
Ciclev10022516m.g	scaffold_3:36278242-36281963	65.8619	39.2185	-0.747909
Ciclev10023599m.g	scaffold_3:23966309-23970016	2.7251	1.62277	-0.747851
Ciclev10021045m.g	scaffold_3:6313534-6316072	4.84375	2.88542	-0.747341
Ciclev10013275m.g	scaffold_6:1735295-1739942	318.493	189.851	-0.746393
Ciclev10010795m.g	scaffold_1:26079116-26085611	1.15111	0.68626	-0.746202
-	scaffold_3:47409328-47410435	9.17692	5.47131	-0.746123
Ciclev10010704m.g	scaffold_1:2910986-2914626	122.551	73.0979	-0.745486
Ciclev10026079m.g	scaffold_7:10760889-10764456	205.319	122.479	-0.745338
Ciclev10032562m.g	scaffold_4:14331319-14332962	3.84482	2.29447	-0.744754
Ciclev10030573m.g	scaffold_4:6823256-6833861	1.17247	0.699775	-0.744582
Ciclev10032630m.g	scaffold_4:8482508-8483446	5.83717	3.48415	-0.744464
Ciclev10030098m.g	scaffold_8:843952-844828	4.57067	2.72888	-0.744098

Ciclev10000678m.g	scaffold_5:42845471-42848054	13.8606	8.27544	-0.74408
Ciclev10023101m.g	scaffold_3:2718185-2719011	47.0595	28.1007	-0.743881
Ciclev10004634m.g	scaffold_9:28026708-28033994	15.9925	9.55048	-0.743754
Ciclev10010372m.g	scaffold_1:15739884-15740196	14.7039	8.7813	-0.743689
Ciclev10016529m.g	scaffold_2:33695994-33699245	116.289	69.4592	-0.743471
Ciclev10024306m.g	scaffold_3:47886838-47889275	4.0256	2.40514	-0.743082
Ciclev10028331m.g	scaffold_8:5635417-5637514	0.77703	0.464267	-0.743016
Ciclev10012120m.g	scaffold_6:23012479-23017729	36.509	21.826	-0.742204
Ciclev10006131m.g	scaffold_9:3739999-3749213	56.5188	33.7925	-0.742027
Ciclev10003194m.g	scaffold_5:35724002-35734729	3.26759	1.95407	-0.741746
Ciclev10012100m.g	scaffold_6:23907307-23908869	117.451	70.2403	-0.741691
Ciclev10011666m.g	scaffold_6:22117549-22123151	5.51877	3.30055	-0.741638
Ciclev10001493m.g	scaffold_5:40745800-40749765	18.0763	10.8119	-0.741474
-	scaffold_2:5366886-5367958	8.52849	5.10394	-0.740678
Ciclev10026644m.g	scaffold_7:2469747-2471188	20.8341	12.4711	-0.740351
Ciclev10009838m.g	scaffold_1:18052866-18056771	8.3774	5.0158	-0.740024
Ciclev10005468m.g	scaffold_9:133023-136741	10.5676	6.32807	-0.739804
Ciclev10031561m.g	scaffold_4:22500263-22503337	7.21581	4.32292	-0.739153
Ciclev10018477m.g	scaffold_3:11516530-11533212	12.1687	7.2913	-0.738926
Ciclev10000992m.g	scaffold_5:43215116-43218585	7.85442	4.70996	-0.737791
Ciclev10018243m.g	scaffold_2:23956661-23961945	5.90986	3.54414	-0.737691
Ciclev10006328m.g	scaffold_9:27867871-27870843	2.42561	1.45508	-0.737248
Ciclev10029847m.g	scaffold_8:22022370-22024705	5.92309	3.55348	-0.737116
Ciclev10009063m.g	scaffold_1:8871608-8873634	297.572	178.551	-0.736899
-	scaffold_5:1552172-1984270	122.992	73.8116	-0.736641
Ciclev10022733m.g	scaffold_3:37422716-37423440	23.9652	14.3839	-0.736488
Ciclev10019530m.g	scaffold_3:49367995-49371500	4.5071	2.70572	-0.736187
Ciclev10000459m.g	scaffold_5:40007932-40012979	27.3476	16.4212	-0.735858
Ciclev10018956m.g	scaffold_3:1790799-1794620	31.9837	19.2053	-0.735829
Ciclev10032570m.g	scaffold_4:25584052-25588663	14.5182	8.71802	-0.735793
Ciclev10014498m.g	scaffold_2:33680381-33684343	29.9796	18.0046	-0.735617
Ciclev10012598m.g	scaffold_6:22807157-22810575	105.204	63.182	-0.735597
Ciclev10014272m.g	scaffold_2:12125405-12128781	1.54919	0.930459	-0.735503
Ciclev10002151m.g	scaffold_5:25868373-25873791	82.9999	49.851	-0.735488
Ciclev10011149m.g	scaffold_6:18212639-18215181	8.0654	4.84469	-0.735342
Ciclev10015338m.g	scaffold_2:290798-294122	51.2123	30.7654	-0.735184
-	scaffold_1:7990764-7991375	16.8232	10.1075	-0.735024
Ciclev10000513m.g,Ciclev10004066m.g	scaffold_5:15186861-15191417	3.71933	2.23505	-0.734735
Ciclev10016289m.g	scaffold_2:34120383-34123587	5.95786	3.58029	-0.734718
Ciclev10002301m.g	scaffold_5:37372295-37374350	6.84736	4.11731	-0.733846
Ciclev10028352m.g	scaffold_8:24926568-24928297	0.515307	0.309877	-0.733737
Ciclev10004771m.g	scaffold_9:6905474-6909989	8.30363	4.99627	-0.732891
Ciclev10021151m.g	scaffold_3:31352097-31356324	21.8584	13.1639	-0.731596
Ciclev10011179m.g	scaffold_6:22998183-23002700	9.40381	5.66346	-0.731563
Ciclev10032819m.g	scaffold_4:10615004-10617641	282.973	170.455	-0.731274
Ciclev10005039m.g	scaffold_9:21989655-21994381	40.1835	24.2062	-0.73123
Ciclev10016822m.g	scaffold_2:27549659-27551146	121.86	73.4089	-0.731202
Ciclev10022378m.g	scaffold_3:46818816-46822856	46.4004	27.9688	-0.73032
Ciclev10004630m.g	scaffold_9:25255735-25259023	0.435635	0.262605	-0.730225
Ciclev10017392m.g	scaffold_2:13388729-13389316	12.9136	7.78769	-0.729627
Ciclev10003744m.g	scaffold_5:21182817-21187250	3.38418	2.04108	-0.729471
Ciclev10033202m.g	scaffold_4:11064397-11066982	28.0128	16.9	-0.729065
Ciclev10010455m.g	scaffold_1:28000846-28002193	0.680433	0.410664	-0.728493
Ciclev10021991m.g	scaffold_3:1470162-1473304	491.813	296.827	-0.728488
Ciclev10000788m.g	scaffold_5:12881996-12884304	7.27585	4.39219	-0.728176

Ciclev10009617m.g	scaffold_1:3383959-3384623	8.01821	4.84092	-0.727999
Ciclev10014935m.g	scaffold_2:36310751-36313620	4.7661	2.87781	-0.727836
Ciclev10014537m.g	scaffold_2:691426-698055	6.28343	3.79522	-0.72737
Ciclev10019637m.g	scaffold_3:8018776-8022807	12.8316	7.75122	-0.727209
Ciclev10018897m.g	scaffold_3:25633451-25636420	1.89666	1.14585	-0.72704
Ciclev10013376m.g	scaffold_6:145836-227910	79.0121	47.7395	-0.726888
Ciclev10016134m.g,Ciclev10016136m.g	scaffold_2:35407310-35413897	300.058	181.39	-0.726145
Ciclev10017382m.g	scaffold_2:8950660-8953716	38.0578	23.0179	-0.725441
Ciclev10032530m.g	scaffold_4:10755092-10756179	131.308	79.4235	-0.725315
Ciclev10021193m.g	scaffold_3:40124808-40130014	13.4947	8.16273	-0.725265
Ciclev10001358m.g	scaffold_5:42968040-42970829	7.64221	4.6227	-0.725253
Ciclev10022321m.g	scaffold_3:29153424-29156189	173.207	104.809	-0.724734
Ciclev10026558m.g	scaffold_7:14900379-14905059	51.0756	30.914	-0.724374
Ciclev10013031m.g	scaffold_6:18929933-18930610	2.04369	1.23707	-0.724249
Ciclev10004261m.g	scaffold_9:15334113-15343107	7.94059	4.80857	-0.723638
Ciclev10009702m.g	scaffold_1:1817885-1821205	71.964	43.5848	-0.72345
Ciclev10007733m.g	scaffold_1:27308613-27316115	137.975	83.5726	-0.723306
Ciclev10020608m.g	scaffold_3:31620762-31715816	10.9633	6.64059	-0.723295
Ciclev10027379m.g	scaffold_7:6394334-6394889	13.5562	8.21686	-0.722294
Ciclev10001268m.g	scaffold_5:36798371-36806685	3.70802	2.24897	-0.721386
-	scaffold_5:6777321-6914637	9.48753	5.75488	-0.721245
Ciclev10016709m.g	scaffold_2:35949471-35951316	16.6892	10.1274	-0.720649
Ciclev10011126m.g	scaffold_6:19990049-19998344	5.08308	3.08464	-0.720602
Ciclev10002890m.g	scaffold_5:35089436-35093078	0.388798	0.236001	-0.720224
Ciclev10033598m.g	scaffold_4:2459305-2460905	6.11095	3.71079	-0.719672
Ciclev10021953m.g	scaffold_3:6233805-6237064	94.0185	57.099	-0.719479
Ciclev10022762m.g	scaffold_3:50918616-50919693	32.3113	19.6235	-0.719462
Ciclev10017330m.g	scaffold_2:31429785-31430982	1.83093	1.11224	-0.719106
Ciclev10009234m.g	scaffold_1:2495908-2498789	13.138	7.98255	-0.718826
Ciclev10005872m.g	scaffold_9:5305585-5310037	42.8322	26.0333	-0.71834
Ciclev10007059m.g	scaffold_9:14388385-14388901	2.8677	1.74313	-0.718209
Ciclev10025926m.g	scaffold_7:1941563-1944415	6.9771	4.24146	-0.718068
Ciclev10005819m.g	scaffold_9:176954-178079	14.4458	8.78506	-0.717523
Ciclev10013929m.g	scaffold_2428:9-1046	1.80387	1.0972	-0.717264
Ciclev10013023m.g	scaffold_6:13082312-13084012	190.188	115.754	-0.716363
Ciclev10016772m.g	scaffold_2:34189215-34193815	3.5096	2.13709	-0.715658
Ciclev10006076m.g	scaffold_9:744345-745408	350.048	213.246	-0.715033
Ciclev10028946m.g	scaffold_8:23557864-23560993	60.9004	37.1016	-0.714971
Ciclev10019179m.g	scaffold_3:8673983-8677126	2.71683	1.65526	-0.714868
Ciclev10015376m.g	scaffold_2:28195184-28198839	6.04977	3.68681	-0.714508
Ciclev10020104m.g	scaffold_3:1079238-1083975	5.13444	3.12931	-0.714363
Ciclev10020923m.g	scaffold_3:44837224-44841711	21.663	13.209	-0.713711
Ciclev10027610m.g,Ciclev10027629m.g	scaffold_7:18435686-18440558	147.169	89.7429	-0.713601
Ciclev10027608m.g	scaffold_7:11062213-11079547	23.2417	14.1738	-0.713491
Ciclev10021219m.g	scaffold_3:23034602-23041873	8.0063	4.883	-0.713367
Ciclev10020050m.g	scaffold_3:9423197-9428954	16.6902	10.1817	-0.713021
Ciclev10013313m.g	scaffold_6:13227990-13229649	164.027	100.067	-0.712966
Ciclev10021137m.g	scaffold_3:44180162-44185000	11.442	6.98234	-0.712551
Ciclev10022132m.g	scaffold_3:2790740-2793179	111.101	67.8106	-0.712288
Ciclev10028159m.g	scaffold_8:1048441-1053724	57.9084	35.3564	-0.711803
Ciclev10026346m.g	scaffold_7:1840331-1844090	87.0919	53.2187	-0.710605
Ciclev10001248m.g	scaffold_5:33825655-33829181	14.2795	8.72587	-0.710578
Ciclev10024850m.g	scaffold_7:13348925-13360491	0.368328	0.2251	-0.710425
Ciclev10004120m.g	scaffold_9:30456143-30470622	33.5819	20.5299	-0.709959

Ciclev10014812m.g	scaffold_2:419741-422139	4.11407	2.51539	-0.709785
Ciclev10001173m.g	scaffold_5:18398112-18402314	5.99746	3.66854	-0.709146
Ciclev10021230m.g	scaffold_3:47885399-47886501	498.029	304.665	-0.709005
Ciclev10009083m.g	scaffold_1:4505251-4507917	61.4177	37.5747	-0.708895
Ciclev10011993m.g,Ciclev10013899m.g	scaffold_6:23115094-23119251	21.6188	13.2263	-0.708875
Ciclev10000799m.g	scaffold_5:41711565-41715301	27.7748	16.9953	-0.708635
-	scaffold_6:13799189-13801116	95.6719	58.5523	-0.70837
Ciclev10018046m.g	scaffold_2:34897744-34899006	4.62659	2.83244	-0.707902
-	scaffold_3:27295733-27296795	16.2716	9.9634	-0.707647
Ciclev10026838m.g	scaffold_7:7808109-7809246	1.69806	1.03988	-0.707467
Ciclev10007406m.g	scaffold_1:2190406-2194638	0.255237	0.156331	-0.707235
Ciclev10022874m.g	scaffold_3:46960939-46963194	99.9569	61.2493	-0.706612
Ciclev10008093m.g	scaffold_1:24602748-24608633	12.8605	7.88371	-0.705999
Ciclev10024038m.g	scaffold_3:24993844-24995370	3.46074	2.12166	-0.705886
Ciclev10013544m.g	scaffold_6:21081659-21082112	58.1749	35.6694	-0.705707
Ciclev10005029m.g	scaffold_9:30937529-30941878	21.1362	12.9632	-0.705293
Ciclev10014190m.g	scaffold_2:22005758-22011411	122.538	75.223	-0.703979
Ciclev10013013m.g	scaffold_6:15158199-15161529	119.787	73.5496	-0.703684
Ciclev10022088m.g	scaffold_3:50793519-50795382	5.34735	3.28466	-0.703082
Ciclev10012378m.g	scaffold_6:21894321-21896323	33.3079	20.4613	-0.702968
Ciclev10026691m.g	scaffold_7:875987-877566	7.68914	4.72409	-0.702785
Ciclev10004616m.g	scaffold_9:4315417-4320797	30.5517	18.7733	-0.702567
Ciclev10032710m.g	scaffold_4:24472219-24474178	27.7981	17.0931	-0.701577
Ciclev10021108m.g	scaffold_3:18748972-18754625	8.33265	5.1241	-0.701476
Ciclev10031803m.g	scaffold_4:16350388-16352768	79.859	49.1275	-0.700924
Ciclev10005158m.g	scaffold_9:1360322-1362454	105.123	64.6877	-0.700514
Ciclev10021502m.g	scaffold_3:1771667-1774647	94.7785	58.3409	-0.700053
Ciclev10020687m.g	scaffold_3:42933883-42935909	262.033	161.334	-0.699696
Ciclev10000597m.g	scaffold_5:39630110-39635609	15.4549	9.51995	-0.699035
Ciclev10002336m.g	scaffold_5:40443226-40444140	2.79367	1.7215	-0.698499
Ciclev10028081m.g	scaffold_8:4073518-4078028	164.21	101.191	-0.698453
Ciclev10018057m.g	scaffold_2:18150879-18153808	25.8726	15.9444	-0.698376
Ciclev10007776m.g	scaffold_1:6448566-6452516	134.935	83.1851	-0.697871
Ciclev10019053m.g	scaffold_3:48787184-48791360	0.768226	0.473661	-0.697676
-	scaffold_5:25664920-25666038	9.86456	6.08277	-0.697525
Ciclev10009724m.g	scaffold_1:24248880-24257969	21.0181	12.9635	-0.697176
Ciclev10030202m.g	scaffold_8:3155551-3157738	1.70498	1.05192	-0.696738
Ciclev10023400m.g	scaffold_3:40042169-40046590	7.74811	4.78236	-0.696123
Ciclev10018939m.g	scaffold_3:4165513-4171242	40.4056	24.9463	-0.69573
Ciclev10030621m.g	scaffold_4:7425521-7429658	20.8285	12.8608	-0.695582
Ciclev10023009m.g	scaffold_3:43767608-43771217	19.0391	11.7562	-0.695541
Ciclev10025978m.g,Ciclev10026774m.g,Ciclev10027455m.g,Ciclev10027544m.g	scaffold_7:13916806-14069972	40.601	25.0708	-0.69551
Ciclev10015347m.g	scaffold_2:11353148-11358883	29.7302	18.3583	-0.695501
Ciclev10002268m.g	scaffold_5:31877029-31880235	93.0031	57.4328	-0.695404
Ciclev10019359m.g	scaffold_3:22814309-22821425	23.2329	14.3495	-0.695169
Ciclev10025153m.g	scaffold_7:12832106-12834217	1.81457	1.12133	-0.694415
Ciclev10011368m.g	scaffold_6:22109329-22115988	108.897	67.2946	-0.694404
Ciclev10007309m.g	scaffold_1:9669670-9677601	8.19107	5.06208	-0.694322
Ciclev10022105m.g	scaffold_3:5275890-5280289	29.9864	18.5384	-0.693791
Ciclev10005409m.g	scaffold_9:13154049-13158820	1.48805	0.920021	-0.693688
Ciclev10009195m.g	scaffold_1:25087600-25090448	69.4841	42.961	-0.693655
Ciclev10030167m.g	scaffold_8:16338669-16350540	3.09597	1.91423	-0.693628
Ciclev10031371m.g	scaffold_4:23177790-23182020	11.05	6.83227	-0.693615

Ciclev10030665m.g,Ciclev10031845m.g,Ciclev10033507m.g	scaffold_4:7532854-7706546	12.6101	7.79713	-0.69356
Ciclev10010043m.g	scaffold_1:26752333-26756663	190.797	117.984	-0.693441
Ciclev10021000m.g	scaffold_3:2002426-2008126	25.8788	16.0057	-0.693183
Ciclev10015160m.g	scaffold_2:14585186-14588249	12.2495	7.57622	-0.69317
Ciclev10012857m.g	scaffold_6:15341356-15343333	29.0096	17.9466	-0.692822
Ciclev10012717m.g	scaffold_6:15188998-15189889	27.5774	17.0607	-0.692813
Ciclev10031070m.g	scaffold_4:1742588-1745145	0.542775	0.335832	-0.692613
Ciclev10002431m.g	scaffold_5:38812677-38813954	2.50744	1.55144	-0.692612
Ciclev10014840m.g	scaffold_2:443365-446877	343.379	212.601	-0.691651
-	scaffold_4:2050598-2051330	7.94873	4.92341	-0.691065
Ciclev10019419m.g	scaffold_3:34289828-34294357	27.7019	17.1598	-0.690949
Ciclev10026708m.g	scaffold_7:11223134-11226332	47.8229	29.6246	-0.690906
Ciclev10026592m.g	scaffold_7:3635575-3637520	61.4057	38.0391	-0.690889
-	scaffold_7:11850299-12160617	4.59666	2.8476	-0.69084
Ciclev10029501m.g	scaffold_8:18120878-18123765	229.927	142.467	-0.690546
Ciclev10029442m.g	scaffold_8:15293820-15298120	19.9784	12.3798	-0.690456
Ciclev10031703m.g	scaffold_4:16817583-16984703	30.2498	18.7451	-0.690414
Ciclev10010252m.g	scaffold_1:8696277-8697796	7.73592	4.79379	-0.690406
Ciclev10010262m.g	scaffold_1:3815864-3816437	10.6068	6.57371	-0.69021
Ciclev10019592m.g	scaffold_3:51432-54241	1.00596	0.623585	-0.689922
Ciclev10025586m.g	scaffold_7:16080691-16087893	30.0071	18.6039	-0.689702
Ciclev10014603m.g	scaffold_2:34337212-34341162	2.86585	1.77747	-0.689142
Ciclev10002876m.g	scaffold_5:441330-538185	37.7356	23.4085	-0.688896
Ciclev10010228m.g	scaffold_1:17359317-17362789	6.10886	3.78991	-0.688741
Ciclev10014031m.g	scaffold_2:31241079-31249842	20.6187	12.792	-0.688714
Ciclev10023978m.g	scaffold_3:18212175-18214200	330.556	205.097	-0.688586
Ciclev10028806m.g	scaffold_8:8180357-8184904	12.5843	7.80846	-0.688517
Ciclev10024956m.g	scaffold_7:2106115-2113603	0.214629	0.133214	-0.688106
Ciclev10015803m.g	scaffold_2:2671734-2674368	9.18514	5.70151	-0.687959
Ciclev10029943m.g	scaffold_8:23864200-23865173	3.36406	2.08835	-0.687841
Ciclev10032942m.g	scaffold_4:10913138-10914792	1505.06	934.456	-0.687625
Ciclev10000132m.g	scaffold_5:26039587-26042662	0.212811	0.132144	-0.687466
Ciclev10008201m.g	scaffold_1:27518022-27525012	26.7995	16.6459	-0.687046
Ciclev10016714m.g	scaffold_2:6671882-6677481	16.0153	9.94769	-0.687016
Ciclev10008091m.g	scaffold_1:28582728-28586461	467.739	290.596	-0.686687
Ciclev10028591m.g	scaffold_8:369801-373129	48.9084	30.3873	-0.686615
Ciclev10028639m.g	scaffold_8:957971-961225	626.222	389.102	-0.686525
Ciclev10012877m.g	scaffold_6:17805472-17807229	5.55142	3.45209	-0.685386
Ciclev10002355m.g	scaffold_5:35847755-35849138	50.2351	31.2435	-0.685138
Ciclev10026488m.g	scaffold_7:5277964-5281312	219.107	136.297	-0.684882
Ciclev10017206m.g	scaffold_2:11365124-11365913	1755.76	1092.33	-0.684685
Ciclev10011894m.g	scaffold_6:11963740-11966587	155.907	96.9993	-0.68464
Ciclev10030989m.g	scaffold_4:23845536-23850285	43.8045	27.2545	-0.684582
Ciclev10007086m.g	scaffold_9:19877554-19879692	8.00069	4.9783	-0.684471
Ciclev10015850m.g	scaffold_2:31653196-31656008	2.35292	1.46416	-0.684378
Ciclev10020845m.g	scaffold_3:1106426-1109870	179.171	111.499	-0.684309
Ciclev10021936m.g	scaffold_3:44202967-44205663	211.592	131.706	-0.683963
Ciclev10030911m.g,Ciclev10033433m.g	scaffold_4:3489372-3502059	10.7943	6.72024	-0.683681
Ciclev10031190m.g	scaffold_4:23908188-23912723	21.9773	13.6834	-0.683584
Ciclev10017420m.g	scaffold_2:9262666-9275068	1.8181	1.13211	-0.683413
Ciclev10033254m.g	scaffold_4:23892713-23896882	1.5671	0.975853	-0.683365
-	scaffold_9:3120511-3121552	21.9109	13.6443	-0.683353
Ciclev10000139m.g	scaffold_5:40472464-40478799	4.33004	2.69668	-0.683198

Ciclev10019472m.g	scaffold_3:848754-852261	0.727207	0.45302	-0.682794
Ciclev10026904m.g	scaffold_7:16076145-16076620	13.5873	8.46565	-0.682567
Ciclev10029813m.g	scaffold_8:22457362-22460790	7.58617	4.72675	-0.682523
Ciclev10000037m.g	scaffold_5:35583221-35591613	0.162387	0.101201	-0.682213
Ciclev10013010m.g	scaffold_6:21494399-21496765	165.164	102.945	-0.682018
Ciclev10004982m.g	scaffold_9:9827281-9829466	0.856599	0.533949	-0.681918
-	scaffold_5:26782121-26782498	299.429	186.664	-0.681771
-	scaffold_9:2361408-2361826	146.916	91.5934	-0.681678
Ciclev10000880m.g	scaffold_5:35604717-35608649	7.95723	4.96285	-0.681097
Ciclev10009901m.g	scaffold_1:3730832-3732364	7.75469	4.83717	-0.680906
Ciclev10029411m.g	scaffold_8:1944352-1945020	1.94004	1.21044	-0.680557
Ciclev10028346m.g	scaffold_8:23634205-23639792	39.1065	24.4001	-0.680521
Ciclev10006155m.g	scaffold_9:29952004-29956306	133.835	83.5088	-0.68046
Ciclev10031938m.g	scaffold_4:21947337-21948907	1.58463	0.988843	-0.68033
Ciclev10027170m.g	scaffold_7:19117341-19118762	29.2229	18.2379	-0.680162
Ciclev10024624m.g	scaffold_3:12214028-12215007	29.0811	18.1511	-0.680021
Ciclev10003828m.g	scaffold_5:32624373-32632209	4.89719	3.05675	-0.679953
Ciclev10031935m.g	scaffold_4:15690355-15782388	16.7254	10.4407	-0.679824
Ciclev10026511m.g	scaffold_7:2590902-2593669	57.7445	36.0501	-0.679678
Ciclev10010766m.g	scaffold_1:403275-404286	3.4739	2.16892	-0.679578
Ciclev10023279m.g	scaffold_3:4011167-4011670	3.18717	1.99044	-0.679187
Ciclev10011873m.g	scaffold_6:6112646-6115289	11.8975	7.43143	-0.678945
Ciclev10031818m.g	scaffold_4:20759363-20764370	15.2882	9.5498	-0.678871
Ciclev10022798m.g	scaffold_3:43972974-43974813	48.2513	30.1424	-0.678775
Ciclev10026835m.g	scaffold_7:10750079-10751777	4.37642	2.73395	-0.678765
-	scaffold_4:7490467-7494401	3.30286	2.06339	-0.678701
Ciclev10004760m.g	scaffold_9:30040456-30046344	240.797	150.476	-0.67828
Ciclev10031748m.g	scaffold_4:24996016-25000652	7.61252	4.75844	-0.677887
Ciclev10014388m.g	scaffold_2:16610021-16624993	42.3352	26.4638	-0.677835
Ciclev10014481m.g	scaffold_2:24293274-24297396	3.53338	2.20889	-0.677725
-	scaffold_8:19434956-19435194	49.3164	30.8329	-0.677598
Ciclev10029655m.g	scaffold_8:1987727-1990725	157.553	98.5323	-0.677168
Ciclev10012117m.g	scaffold_6:21412453-21415322	28.2083	17.6416	-0.677135
Ciclev10020221m.g	scaffold_3:4625770-4630292	19.4752	12.1869	-0.676311
Ciclev10020415m.g	scaffold_3:11687448-12154880	15.3586	9.6112	-0.676259
Ciclev10025654m.g	scaffold_7:15473584-15481602	0.43671	0.273297	-0.676206
Ciclev10021525m.g	scaffold_3:48812601-48814276	1.60203	1.00258	-0.676185
Ciclev10028594m.g	scaffold_8:6689818-6692205	9146.97	5724.68	-0.676097
Ciclev10009684m.g	scaffold_1:23085509-23087429	23.5683	14.7507	-0.676065
Ciclev10018347m.g	scaffold_2:18185191-18186982	1.04103	0.651791	-0.675529
Ciclev10015514m.g	scaffold_2:8268213-8270804	0.407401	0.255105	-0.675358
Ciclev10033103m.g	scaffold_4:25461401-25463790	92.8647	58.1517	-0.675308
Ciclev10006063m.g	scaffold_9:16155704-16161524	38.7417	24.2697	-0.674733
Ciclev10026517m.g	scaffold_7:3093668-3095978	28.6891	17.9732	-0.674656
Ciclev10004560m.g	scaffold_9:5648638-5654374	35.8682	22.4712	-0.674629
Ciclev10005072m.g	scaffold_9:27749863-27751611	3.47716	2.17881	-0.674367
Ciclev10017003m.g	scaffold_2:36093568-36099013	27.3752	17.1567	-0.674096
Ciclev10022064m.g	scaffold_3:29551632-29563218	429.5	269.204	-0.673958
Ciclev10008638m.g	scaffold_1:3356191-3360411	89.6117	56.2007	-0.6731
Ciclev10029778m.g	scaffold_8:4997777-4999543	1.73415	1.08785	-0.672743
Ciclev10004595m.g	scaffold_9:27505944-27509877	0.436209	0.27364	-0.672742
Ciclev10016330m.g	scaffold_2:14209265-14216265	13.1737	8.26504	-0.672568
Ciclev10023750m.g	scaffold_3:45612658-45613009	1.23775	0.776585	-0.672505
Ciclev10022561m.g	scaffold_3:24750793-24752907	96.3483	60.459	-0.672301
Ciclev10002020m.g	scaffold_5:33225099-33232451	41.5627	26.0852	-0.672057
Ciclev10025865m.g	scaffold_7:6934881-6939477	1.53843	0.966049	-0.671291

Ciclev10032080m.g	scaffold_4:23025674-23028978	5.60641	3.52102	-0.671086
Ciclev10032341m.g	scaffold_4:16418137-16422763	28.0283	17.6031	-0.67105
Ciclev10003207m.g,Ciclev10003500m.g,Ciclev10003621m.g	scaffold_5:26144092-26153435	22.7245	14.2724	-0.671027
Ciclev10013632m.g	scaffold_6:7919985-7922537	43.5017	27.3333	-0.670411
Ciclev10000539m.g	scaffold_5:12689295-12694012	5.84626	3.67361	-0.670315
Ciclev10017305m.g	scaffold_2:33066597-33067199	3.12208	1.96196	-0.670214
Ciclev10012465m.g	scaffold_6:18943087-18947680	18.8125	11.8238	-0.669995
Ciclev10006514m.g	scaffold_9:69036-69837	83.3015	52.3766	-0.66942
Ciclev10007412m.g	scaffold_1:4952748-4958212	15.1895	9.55068	-0.669399
Ciclev10002660m.g	scaffold_5:31900523-31902875	9.07046	5.70337	-0.66936
Ciclev10025728m.g	scaffold_7:5035428-5038431	11.8739	7.46682	-0.66923
Ciclev10002585m.g	scaffold_5:21106735-21110055	59.5378	37.4409	-0.669192
Ciclev10017455m.g	scaffold_2:10576388-10580593	36.5036	22.9565	-0.669132
Ciclev10027892m.g	scaffold_8:21558538-21563671	5.57672	3.50738	-0.669022
Ciclev10025847m.g	scaffold_7:7779859-7782141	0.611825	0.384845	-0.668842
Ciclev10014428m.g	scaffold_2:30161875-30166336	33.5211	21.0857	-0.668803
Ciclev10018578m.g,Ciclev10018612m.g	scaffold_3:21260283-21283985	13.4706	8.47378	-0.668731
Ciclev10030885m.g	scaffold_4:6117333-6125345	37.7853	23.7696	-0.668711
Ciclev10008810m.g	scaffold_1:25691262-25693088	4.47317	2.81415	-0.668597
Ciclev10008109m.g	scaffold_1:940513-942972	9.10626	5.729	-0.668577
Ciclev10012898m.g	scaffold_6:40380-43868	252.047	158.583	-0.668455
Ciclev10028768m.g	scaffold_8:23182128-23187363	19.7076	12.4037	-0.667984
Ciclev10006317m.g	scaffold_9:1953466-1954083	1.75535	1.10568	-0.666829
Ciclev10009604m.g	scaffold_1:28216226-28219482	32	20.16	-0.666577
Ciclev10018992m.g	scaffold_3:43123639-43131063	30.817	19.4164	-0.666452
Ciclev10025247m.g	scaffold_7:8976000-8980710	2.34135	1.47531	-0.666326
Ciclev10016920m.g	scaffold_2:27377594-27378867	4.83706	3.04893	-0.665829
Ciclev10020659m.g	scaffold_3:10415817-10420049	25.6786	16.1871	-0.665725
Ciclev10011719m.g	scaffold_6:22002890-22006522	114.247	72.0297	-0.665494
Ciclev10021055m.g	scaffold_3:12182671-12187107	80.8097	50.9563	-0.665269
Ciclev10013253m.g	scaffold_6:17317521-17318902	5.01146	3.1602	-0.665217
Ciclev10002915m.g	scaffold_5:42495261-42497399	40.0987	25.2888	-0.665057
Ciclev10011483m.g	scaffold_6:22933478-22936068	7.09711	4.47662	-0.664823
Ciclev10008089m.g	scaffold_1:878815-881721	8.57708	5.41019	-0.664808
Ciclev10025373m.g	scaffold_7:7995868-7999139	1.37569	0.86797	-0.664436
Ciclev10022842m.g	scaffold_3:28389624-28390574	12.5093	7.8926	-0.664428
Ciclev10015947m.g	scaffold_2:3029121-3032017	1.59093	1.00381	-0.664393
Ciclev10000148m.g	scaffold_5:5126652-5131092	2.0234	1.27675	-0.664301
Ciclev10025949m.g	scaffold_7:1202439-1204061	0.770403	0.486149	-0.664216
Ciclev10004896m.g	scaffold_9:27015821-27051136	1.63834	1.03385	-0.66421
-	scaffold_2:24401745-24433873	16.3341	10.3104	-0.663792
Ciclev10000532m.g	scaffold_5:34533360-34540306	29.8227	18.8251	-0.663755
Ciclev10026604m.g	scaffold_7:7301377-7304325	7.93242	5.00769	-0.663616
Ciclev10007952m.g	scaffold_1:5184319-5188255	19.0653	12.0374	-0.663419
Ciclev10009996m.g	scaffold_1:26483958-26484701	29.1484	18.4074	-0.663133
Ciclev10026213m.g	scaffold_7:6388670-6392102	21.4408	13.5402	-0.663102
Ciclev10014762m.g	scaffold_2:28060820-28064917	7.24732	4.57732	-0.662945
Ciclev10029052m.g	scaffold_8:23875186-23878850	54.6142	34.4979	-0.662767
Ciclev10028388m.g	scaffold_8:24437768-24440746	14.1934	8.96839	-0.6623
Ciclev10026484m.g	scaffold_7:18547483-18550515	8.05118	5.08931	-0.661729
Ciclev10013226m.g	scaffold_6:18096609-18098601	71.6763	45.3091	-0.661697
Ciclev10009520m.g	scaffold_1:24812068-24813774	81.1668	51.3098	-0.661655
Ciclev10024599m.g	scaffold_3:28345310-28349148	0.448594	0.283585	-0.661631

Ciclev10020624m.g	scaffold_3:33687571-33691590	53.2048	33.6383	-0.66145
Ciclev10022743m.g	scaffold_3:2945186-2945835	1.77726	1.1239	-0.661142
Ciclev10019149m.g	scaffold_3:9572730-9583014	25.9961	16.4406	-0.661033
Ciclev10030725m.g	scaffold_4:1143602-1148807	14.5366	9.19491	-0.660778
Ciclev10017833m.g	scaffold_2:20171232-20173158	9.37059	5.92835	-0.66051
Ciclev10003702m.g	scaffold_5:828570-829515	5.36828	3.39709	-0.660163
Ciclev10012928m.g	scaffold_6:20956325-20958294	173.541	109.819	-0.660151
Ciclev10000392m.g	scaffold_5:34866518-34875003	8.81079	5.5757	-0.660119
Ciclev10020652m.g	scaffold_3:7479693-7484484	6.27612	3.97193	-0.66003
Ciclev10033155m.g	scaffold_4:2463999-2464574	3.45622	2.18773	-0.659764
Ciclev10017501m.g	scaffold_2:35761243-35766725	225.679	142.853	-0.659739
Ciclev10021520m.g	scaffold_3:23388133-23393485	18.2773	11.5698	-0.659689
-	scaffold_5:10427531-10584700	10.9534	6.93543	-0.659328
Ciclev10017243m.g	scaffold_2:21759010-21761073	878.517	556.316	-0.659165
Ciclev10031770m.g	scaffold_4:15551597-15554506	32.9157	20.8464	-0.65898
Ciclev10000225m.g	scaffold_5:14343906-14350359	6.87576	4.3553	-0.658748
Ciclev10000059m.g	scaffold_5:35744243-35755651	3.78519	2.39909	-0.657879
Ciclev10022494m.g	scaffold_3:41437317-41440547	53.6492	34.0038	-0.65786
Ciclev10000192m.g	scaffold_5:35502381-35510013	3.70412	2.34779	-0.657828
Ciclev10000950m.g	scaffold_5:35948623-35951377	29.7774	18.8742	-0.657806
Ciclev10009727m.g	scaffold_1:21331251-21332338	6.55577	4.15561	-0.657705
Ciclev10001853m.g	scaffold_5:36210238-36213202	108.546	68.8402	-0.656989
Ciclev10014849m.g	scaffold_2:8644003-8647788	18.0109	11.4273	-0.656389
Ciclev10023527m.g	scaffold_3:4954029-4959276	178.412	113.243	-0.655789
Ciclev10006242m.g	scaffold_9:477387-478920	131.156	83.2516	-0.655734
Ciclev10025498m.g	scaffold_7:10007336-10013431	0.414711	0.263279	-0.655514
Ciclev10004775m.g	scaffold_9:26790441-26802517	8.51877	5.40813	-0.655514
-	scaffold_5:9671903-9672490	5.37006	3.40926	-0.655479
Ciclev10019240m.g	scaffold_3:8117418-8124099	11.1949	7.10762	-0.655405
Ciclev10004067m.g	scaffold_5:33868736-33872608	42.8675	27.2214	-0.655145
Ciclev10021364m.g	scaffold_3:6299686-6303749	29.0281	18.4332	-0.655144
Ciclev10024684m.g	scaffold_7:20100362-20110034	9.70497	6.16395	-0.654869
Ciclev10010097m.g	scaffold_1:10539238-10539492	224.229	142.452	-0.654489
Ciclev10015472m.g	scaffold_2:34516498-34519050	52.5545	33.4302	-0.652661
-	scaffold_8:3526414-3526802	10.579	6.73229	-0.652041
Ciclev10008624m.g	scaffold_1:1245556-1247064	8.23715	5.24393	-0.651495
Ciclev10014764m.g	scaffold_2:24641958-24644161	24.8724	15.8435	-0.650654
Ciclev10031220m.g	scaffold_4:22577316-22582276	1.07206	0.682947	-0.650544
-	scaffold_3:10423835-10425663	3.62719	2.31084	-0.650436
Ciclev10018305m.g	scaffold_2:18787025-18790398	48.8849	31.1468	-0.650303
Ciclev10030650m.g	scaffold_4:13903685-13910631	3.22744	2.05733	-0.649618
Ciclev10009076m.g	scaffold_1:738190-743742	140.282	89.4452	-0.649255
Ciclev10007256m.g	scaffold_1:23042606-23047230	1.40799	0.897784	-0.649197
Ciclev10022460m.g	scaffold_3:2898683-2900608	30.9221	19.7171	-0.649193
Ciclev10015687m.g	scaffold_2:34075359-34081355	313.72	200.056	-0.649072
Ciclev10017252m.g	scaffold_2:33551468-33556070	9.97183	6.35907	-0.649041
-	scaffold_9:8451669-8452622	17.2191	10.9817	-0.64891
Ciclev10001527m.g	scaffold_5:38120562-38123758	29.6121	18.8863	-0.648846
Ciclev10024893m.g	scaffold_7:7362930-7366344	4.40379	2.80886	-0.64876
Ciclev10030810m.g	scaffold_4:1219998-1223875	5.63012	3.59175	-0.648479
-	scaffold_3:24602583-24602957	13.5844	8.67116	-0.647652
Ciclev10022507m.g	scaffold_3:4066181-4067705	2.35857	1.50557	-0.647603
Ciclev10004501m.g	scaffold_9:624507-627093	3.30434	2.10931	-0.647592
Ciclev10027953m.g	scaffold_8:12800121-12845493	8.43671	5.38578	-0.647524
Ciclev10010582m.g	scaffold_1:23184868-23193259	13.8992	8.87307	-0.647493
Ciclev10010815m.g	scaffold_1:9452152-9455317	0.375595	0.239782	-0.647453

Ciclev10028564m.g	scaffold_8:18342017-18343462	1.20733	0.770807	-0.647383
Ciclev10030703m.g	scaffold_4:21524992-21532610	1.58535	1.01222	-0.647278
Ciclev10002731m.g	scaffold_5:41330381-41331699	82.8983	52.9434	-0.646892
Ciclev10015165m.g	scaffold_2:34460632-34462032	1.28528	0.820905	-0.646793
-	scaffold_5:20904811-20905162	45.0081	28.7472	-0.646762
Ciclev10011280m.g	scaffold_6:17767218-17771514	4.37939	2.79756	-0.64656
Ciclev10029417m.g	scaffold_8:6861438-6864004	180.757	115.506	-0.646084
Ciclev10018921m.g,Ciclev10018965m.g	scaffold_3:41636615-41671116	79.7961	50.9918	-0.646053
Ciclev10001873m.g	scaffold_5:35661716-35664130	205.252	131.174	-0.645913
Ciclev10021390m.g,Ciclev10021404m.g	scaffold_3:44717414-44725059	12.8701	8.22571	-0.645812
Ciclev10025596m.g	scaffold_7:3913364-3918912	11.3563	7.26146	-0.645162
Ciclev10027543m.g	scaffold_7:20203124-20208299	82.2147	52.5717	-0.64511
Ciclev10008930m.g	scaffold_1:1051154-1052947	204.662	130.894	-0.644847
Ciclev10019786m.g	scaffold_3:47157493-47161822	12.1455	7.76819	-0.644775
Ciclev10018038m.g	scaffold_2:30719028-30725506	1.21911	0.779799	-0.644651
Ciclev10026572m.g	scaffold_7:7072698-7074233	8.18505	5.23592	-0.644547
Ciclev10011247m.g	scaffold_6:13750670-13756217	1.66288	1.06381	-0.644448
Ciclev10019836m.g	scaffold_3:3066959-3071658	3.0758	1.96773	-0.644428
Ciclev10011983m.g,Ciclev10013257m.g	scaffold_6:3258421-3266781	60.2718	38.5647	-0.644202
Ciclev10028677m.g	scaffold_8:10499114-10501752	2.7798	1.77866	-0.644196
Ciclev10025910m.g	scaffold_7:3282640-3284143	309.184	197.846	-0.644091
Ciclev10002714m.g	scaffold_5:34219816-34223850	184.496	118.062	-0.644039
Ciclev10018827m.g	scaffold_3:43702133-43706100	0.719718	0.460649	-0.643764
Ciclev10011576m.g	scaffold_6:8029487-8035109	44.3624	28.3944	-0.643732
Ciclev10009505m.g	scaffold_1:6427074-6437114	75.6873	48.4497	-0.643564
Ciclev10012493m.g,Ciclev10012496m.g,Ciclev10012645m.g,Ciclev10012734m.g	scaffold_6:14650331-14687366	46.5079	29.7719	-0.643523
Ciclev10013206m.g	scaffold_6:12896099-12897242	1.62223	1.03856	-0.643393
Ciclev10031445m.g	scaffold_4:24770753-24773948	12.7224	8.14656	-0.643112
Ciclev10030069m.g	scaffold_8:16603088-16604828	1.74905	1.12	-0.64307
Ciclev10009999m.g	scaffold_1:936818-939301	143.557	91.9458	-0.642769
Ciclev10027369m.g	scaffold_7:13436034-13436394	1.2171	0.779654	-0.642541
Ciclev10014447m.g	scaffold_2:33460711-33463106	17.2601	11.0567	-0.642528
Ciclev10026011m.g	scaffold_7:5006488-5007808	2.46692	1.58031	-0.642499
Ciclev10002982m.g	scaffold_5:13193182-13193944	45.9358	29.4271	-0.642475
Ciclev10000856m.g	scaffold_5:35408651-35414270	33.3658	21.3752	-0.642435
Ciclev10023939m.g	scaffold_3:38078369-38107449	57.0856	36.5793	-0.642099
Ciclev10002751m.g	scaffold_5:42187713-42189727	349.33	223.906	-0.641696
Ciclev10010825m.g	scaffold_1:5054550-5056146	12.8618	8.24588	-0.641349
Ciclev10015868m.g	scaffold_2:26397860-26399712	0.706065	0.452739	-0.64112
Ciclev10025057m.g,Ciclev10027466m.g	scaffold_7:7356904-7362279	5.81325	3.72885	-0.640615
Ciclev10018681m.g	scaffold_3:8926863-8933381	51.5155	33.048	-0.640443
Ciclev10002083m.g	scaffold_5:39748753-39753073	46.7369	29.9837	-0.640383
Ciclev10029498m.g	scaffold_8:23137846-23138685	3.30427	2.12028	-0.640073
Ciclev10019996m.g	scaffold_3:6598548-6602993	17.8423	11.4508	-0.639851
Ciclev10019570m.g	scaffold_3:48475807-48479673	23.6115	15.1616	-0.639068
Ciclev10028270m.g	scaffold_8:8861312-8863137	2.40812	1.54634	-0.63905
Ciclev10001128m.g	scaffold_5:33307926-33312897	5.3952	3.4646	-0.638986
Ciclev10001961m.g	scaffold_5:36463101-36467207	10.3259	6.63168	-0.638825
Ciclev10018694m.g	scaffold_3:42655296-42660975	14.1637	9.10075	-0.638142
Ciclev10027924m.g	scaffold_8:23400114-23403555	34.8132	22.3714	-0.637981

Ciclev10009141m.g	scaffold_1:27286386-27290085	54.576	35.0724	-0.63793
Ciclev10001247m.g	scaffold_5:34778784-34781315	8.01269	5.15107	-0.637415
Ciclev10016179m.g	scaffold_2:26271555-26273261	9.19227	5.90963	-0.637352
Ciclev10032126m.g	scaffold_4:9593405-9595710	65.0006	41.7891	-0.637324
Ciclev10032214m.g	scaffold_4:219361-222771	43.9974	28.2866	-0.637302
Ciclev10016152m.g	scaffold_2:33388564-33391944	9.58061	6.16007	-0.637172
Ciclev10019041m.g,Ciclev10020948m.g,Ciclev10023035m.g,Ciclev10023330m.g	scaffold_3:46519382-46595230	64.144	41.2446	-0.637109
Ciclev10011445m.g	scaffold_6:12670836-12673970	37.8744	24.3563	-0.636932
Ciclev10008943m.g	scaffold_1:3957072-3960020	83.279	53.5603	-0.636788
Ciclev10014520m.g	scaffold_2:9507230-9510821	6.23265	4.00906	-0.63658
Ciclev10031422m.g	scaffold_4:5818962-5823599	12.7158	8.18061	-0.63634
Ciclev10019596m.g	scaffold_3:47666935-47669808	1.75252	1.1278	-0.635918
Ciclev10008878m.g	scaffold_1:27259399-27262797	61.9886	39.8987	-0.635664
Ciclev10001756m.g	scaffold_5:34978110-34983399	139.477	89.7767	-0.635615
Ciclev10026490m.g	scaffold_7:11397391-11398623	58.1479	37.4287	-0.635582
Ciclev10011520m.g	scaffold_6:21816067-21819488	40.1497	25.847	-0.635389
Ciclev10004897m.g	scaffold_9:3154815-3158482	6.13323	3.94873	-0.635259
Ciclev10010458m.g	scaffold_1:233491-237597	0.978419	0.630251	-0.634526
Ciclev10026499m.g	scaffold_7:20885778-20887861	11.4138	7.35239	-0.634498
Ciclev10009403m.g	scaffold_1:2844905-2847136	5.27274	3.39657	-0.634474
-	scaffold_8:7159541-7160485	3.64687	2.3494	-0.634367
Ciclev10001743m.g	scaffold_5:39108939-39111981	82.6899	53.2802	-0.634113
Ciclev10008677m.g	scaffold_1:28103918-28118736	79.3932	51.1589	-0.63403
Ciclev10009331m.g	scaffold_1:26373048-26375504	1025.13	660.614	-0.633931
Ciclev10030723m.g	scaffold_4:25527915-25534725	35.9644	23.1787	-0.633772
Ciclev10023249m.g	scaffold_3:9382848-9385275	9.1051	5.86837	-0.633715
Ciclev10000853m.g	scaffold_5:39626614-39629746	19.3681	12.4844	-0.633561
Ciclev10006114m.g	scaffold_9:5144382-5146965	45.9015	29.5977	-0.633058
Ciclev10025098m.g	scaffold_7:6138356-6143883	0.288655	0.186176	-0.632676
Ciclev10002778m.g	scaffold_5:34976703-34977897	287.641	185.559	-0.632389
Ciclev10008762m.g	scaffold_1:23865510-23867371	1485.16	958.242	-0.632157
Ciclev10014753m.g	scaffold_2:34823743-34829162	11.5281	7.43957	-0.631867
Ciclev10028928m.g	scaffold_8:24537197-24542454	16.1222	10.4043	-0.631866
Ciclev10023854m.g	scaffold_3:41303785-41309278	21.4528	13.8461	-0.631688
Ciclev10028659m.g	scaffold_8:3638678-3643879	7.05669	4.55482	-0.631599
Ciclev10011001m.g	scaffold_6:22221947-22225792	1.15677	0.746699	-0.6315
Ciclev10016702m.g	scaffold_2:32919901-32922181	68.4153	44.1733	-0.631146
Ciclev10009736m.g	scaffold_1:2228312-2230527	35.4991	22.9287	-0.630627
Ciclev10020653m.g	scaffold_3:13560845-13564434	7.88155	5.09198	-0.630252
Ciclev10024847m.g	scaffold_7:13370223-13373193	0.476199	0.307655	-0.630251
-	scaffold_4:19450770-19451314	10.6305	6.87013	-0.629794
Ciclev10022405m.g	scaffold_3:49384005-49385802	4.60086	2.97383	-0.62958
Ciclev10003243m.g	scaffold_5:17934315-17935705	3.19373	2.0651	-0.629033
-	scaffold_7:11850299-12160617	12.252	7.92292	-0.628913
Ciclev10032567m.g	scaffold_4:8451957-8453207	17.7292	11.4663	-0.628721
Ciclev10025846m.g	scaffold_7:13006094-13011505	11.6474	7.53381	-0.628556
Ciclev10015602m.g	scaffold_2:35099436-35104194	27.3662	17.7024	-0.628454
Ciclev10017035m.g	scaffold_2:23723587-23725753	26.2498	16.9811	-0.628376
Ciclev10011970m.g	scaffold_6:17523139-17526888	69.9534	45.2555	-0.628301
Ciclev10028678m.g	scaffold_8:14071485-14075854	9.24345	5.98003	-0.62828
Ciclev10018649m.g	scaffold_3:48531470-48539190	13.2689	8.58521	-0.628118
Ciclev10026953m.g	scaffold_7:187885-189999	2.89986	1.87627	-0.628115
Ciclev10031937m.g	scaffold_4:23956994-23959284	17.0456	11.0295	-0.628031
Ciclev10013047m.g	scaffold_6:21717965-21720078	334.241	216.28	-0.627986

Ciclev10020119m.g	scaffold_3:48069399-48074465	20.3509	13.1719	-0.627627
Ciclev10008802m.g	scaffold_1:20468397-20472963	3.02383	1.95725	-0.627546
Ciclev10012128m.g	scaffold_6:9922495-9927448	37.9461	24.5736	-0.626844
Ciclev10005157m.g	scaffold_9:28349669-28351327	24.1035	15.6122	-0.62657
Ciclev10012143m.g	scaffold_6:20715589-20719704	7.57806	4.90987	-0.626144
Ciclev10009662m.g	scaffold_1:27332599-27333762	58.7928	38.1215	-0.625036
Ciclev10019272m.g	scaffold_3:49346936-49350957	15.6547	10.1511	-0.62496
Ciclev10011425m.g	scaffold_6:17632288-17636959	11.0465	7.16497	-0.624555
Ciclev10028297m.g	scaffold_8:12947987-12949765	92.2407	59.8315	-0.624499
-	scaffold_5:8234427-8235655	7.75622	5.03168	-0.624314
Ciclev10014194m.g	scaffold_2:33424943-33432149	22.9218	14.8736	-0.623964
Ciclev10021410m.g	scaffold_3:2975104-2976731	17.1882	11.1551	-0.623715
Ciclev10022315m.g	scaffold_3:43773634-43774869	3.75896	2.4402	-0.623336
Ciclev10033140m.g	scaffold_4:20658464-20660778	297.314	193.017	-0.623258
Ciclev10014962m.g	scaffold_2:7388981-7393977	8.7543	5.68409	-0.623061
Ciclev10010669m.g	scaffold_1:6230232-6239600	23.4971	15.264	-0.622351
Ciclev10021199m.g	scaffold_3:24629369-24734809	18.5204	12.0339	-0.622008
Ciclev10030689m.g	scaffold_4:7324597-7330347	14.4158	9.37061	-0.621438
Ciclev10018899m.g	scaffold_3:45663753-45674700	42.3649	27.5409	-0.621295
Ciclev10000510m.g	scaffold_5:35125018-35129817	0.436492	0.283769	-0.62124
Ciclev10009128m.g	scaffold_1:8998204-8999804	25.1587	16.357	-0.621148
Ciclev10009448m.g	scaffold_1:6903803-6918085	24.0858	15.6632	-0.620804
Ciclev10028977m.g	scaffold_8:3670008-3672109	1.27898	0.831759	-0.620761
Ciclev10021689m.g	scaffold_3:48914345-48916276	223.2	145.165	-0.620644
Ciclev10022179m.g	scaffold_3:9759781-9761116	2.69287	1.75156	-0.620501
Ciclev10004696m.g	scaffold_9:28974336-28977865	1.59849	1.03998	-0.620161
Ciclev10001739m.g	scaffold_5:37302526-37305633	28.3509	18.4461	-0.620082
Ciclev10024783m.g	scaffold_7:240863-250983	2.46892	1.60662	-0.619858
Ciclev10033522m.g	scaffold_4:18383678-18386849	223.611	145.527	-0.619711
Ciclev10030559m.g	scaffold_4:14499669-14508162	2.60167	1.69345	-0.619474
Ciclev10016443m.g	scaffold_2:347633-354348	22.9079	14.9119	-0.619379
Ciclev10016662m.g	scaffold_2:34643676-34647898	7.27197	4.7338	-0.619346
Ciclev10010691m.g	scaffold_1:22768534-22768744	229.288	149.277	-0.619172
Ciclev10006117m.g,Ciclev10006312m.g	scaffold_9:1737891-1745003	7.76044	5.05295	-0.619011
Ciclev10020521m.g	scaffold_3:5475151-5477416	9.50373	6.18892	-0.618806
Ciclev10031396m.g	scaffold_4:2404352-2407969	26.421	17.2074	-0.618658
Ciclev10012288m.g	scaffold_6:17857848-17860063	7.91031	5.15241	-0.618486
Ciclev10022281m.g	scaffold_3:47639536-47640401	47.2366	30.7678	-0.618483
Ciclev10012880m.g	scaffold_6:22229222-22231270	16.3443	10.6474	-0.618281
Ciclev10029688m.g	scaffold_8:19551715-19552158	9.83421	6.40763	-0.618019
Ciclev10016580m.g	scaffold_2:11611057-11615878	6.11515	3.98486	-0.617859
Ciclev10011098m.g	scaffold_6:23892906-23899830	7.6437	4.98328	-0.617176
-	scaffold_5:26419086-26419508	8.85467	5.77285	-0.617154
Ciclev10020669m.g	scaffold_3:988867-992535	4.97103	3.24111	-0.617058
Ciclev10024761m.g	scaffold_7:5844354-5856879	4.41765	2.88152	-0.61645
Ciclev10018715m.g,Ciclev10023873m.g	scaffold_3:24629369-24734809	7.54336	4.92069	-0.616348
Ciclev10007946m.g	scaffold_1:4718454-4721464	0.787566	0.51375	-0.616335
Ciclev10024608m.g	scaffold_3:7648038-7650966	1.98559	1.29537	-0.616206
Ciclev10021319m.g	scaffold_3:6654996-6660313	108.66	70.8918	-0.616132
Ciclev10022028m.g	scaffold_3:2130469-2131672	11.9274	7.78218	-0.616031
Ciclev10023990m.g,Ciclev10024314m.g	scaffold_3:30345182-30357916	36.8826	24.075	-0.615407
Ciclev10001550m.g,Ciclev10004023m.g	scaffold_5:14684155-14691267	8.4312	5.50369	-0.615338

Ciclev10029259m.g	scaffold_8:23087004-23091069	21.1417	13.8025	-0.615155
Ciclev10021762m.g	scaffold_3:3347747-3350257	9.13332	5.96312	-0.615072
Ciclev10023741m.g	scaffold_3:32912232-32920214	7.63387	4.98476	-0.61489
Ciclev10003066m.g	scaffold_5:27835482-27837426	69.7832	45.5711	-0.614761
Ciclev10000462m.g	scaffold_5:31075830-31079709	9.61435	6.27911	-0.614631
Ciclev10031472m.g	scaffold_4:20856340-20862687	14.1824	9.26253	-0.614622
-	scaffold_5:2534144-2731098	5.54736	3.62457	-0.61399
Ciclev10002246m.g	scaffold_5:29196959-29199955	84.0556	54.9333	-0.613663
Ciclev10027911m.g	scaffold_8:20321002-20329872	7.98331	5.21756	-0.613612
Ciclev10014827m.g	scaffold_2:23341053-23346044	11.6031	7.58348	-0.613578
Ciclev10012736m.g	scaffold_6:17949430-17950757	14.8557	9.70957	-0.613542
Ciclev10008310m.g	scaffold_1:20474444-20476909	1.63371	1.06841	-0.612687
Ciclev10032001m.g	scaffold_4:428403-432345	10.1676	6.64987	-0.612583
Ciclev10016200m.g	scaffold_2:33716092-33717587	67.622	44.2276	-0.612544
Ciclev10030655m.g	scaffold_4:4037574-4045007	20.0297	13.1003	-0.612538
Ciclev10015389m.g,Ciclev10017729m.g	scaffold_2:29674805-29687460	13.0972	8.56627	-0.612518
Ciclev10017438m.g	scaffold_2:32982212-32985696	4.97967	3.2572	-0.612417
Ciclev10028259m.g	scaffold_8:22609393-22613382	49.8155	32.5878	-0.612262
Ciclev10026223m.g	scaffold_7:8255045-8256442	9.33358	6.10577	-0.612256
-	scaffold_8:10790138-10791407	46.542	30.4583	-0.611698
Ciclev10013363m.g	scaffold_6:18848221-18849800	1.15966	0.759004	-0.611523
Ciclev10005536m.g	scaffold_9:6529165-6531694	3.51883	2.30313	-0.611496
Ciclev10019537m.g	scaffold_3:43570990-43574886	19.991	13.0862	-0.611307
Ciclev10006891m.g	scaffold_9:13008714-13010186	5.90877	3.86924	-0.610807
Ciclev10012589m.g	scaffold_6:9464787-9471458	22.3678	14.6575	-0.609791
Ciclev10020379m.g	scaffold_3:49168411-49173865	9.46762	6.20474	-0.609631
Ciclev10002836m.g	scaffold_5:34973021-34975275	6.34798	4.16035	-0.609595
Ciclev10012179m.g	scaffold_6:24237295-24238755	3.35136	2.19647	-0.609556
Ciclev10005678m.g	scaffold_9:12763966-12767183	19.3907	12.7094	-0.609469
Ciclev10014391m.g	scaffold_2:33802694-33808119	6.24613	4.09449	-0.60928
Ciclev10021324m.g	scaffold_3:1195177-1198244	84.2806	55.2739	-0.608601
Ciclev10028409m.g	scaffold_8:18806443-18814838	7.63091	5.00462	-0.608594
Ciclev10004330m.g	scaffold_9:1763487-1769451	24.9197	16.3442	-0.608507
Ciclev10011605m.g	scaffold_6:25456437-25460254	10.0655	6.60187	-0.608469
Ciclev10019471m.g	scaffold_3:2767252-2772249	24.1326	15.83	-0.608328
Ciclev10003912m.g	scaffold_5:22562644-22570792	5.02766	3.29902	-0.607847
Ciclev10032913m.g	scaffold_4:5839800-5844298	14.622	9.59459	-0.607846
Ciclev10005724m.g	scaffold_9:2864003-2866006	88.8624	58.3153	-0.607699
Ciclev10026421m.g	scaffold_7:3369181-3371609	42.8075	28.0978	-0.607407
Ciclev10020142m.g	scaffold_3:5928294-5930580	10.4954	6.88914	-0.607361
-	scaffold_4:24836410-24836956	38.742	25.4308	-0.607323
Ciclev10022640m.g	scaffold_3:41811980-41814097	81.3369	53.4028	-0.606996
Ciclev10007227m.g	scaffold_1:23487199-23497316	0.122266	0.0802845	-0.606829
Ciclev10006206m.g	scaffold_9:15593358-15594491	6.47515	4.25232	-0.606664
Ciclev10016262m.g	scaffold_2:35546691-35549811	101.052	66.3649	-0.606604
Ciclev10027852m.g	scaffold_8:1339973-1347274	96.8564	63.6159	-0.606459
Ciclev10000207m.g	scaffold_5:33908954-33913762	4.0002	2.62766	-0.60629
Ciclev10031710m.g	scaffold_4:23591659-23596185	41.6492	27.3623	-0.606099
-	scaffold_1:20050545-20056701	17.3811	11.4233	-0.605536
Ciclev10009830m.g	scaffold_1:25211314-25212784	39.5705	26.0098	-0.60537
Ciclev10032405m.g	scaffold_4:18515567-18675155	2.39632	1.57541	-0.605093
-	scaffold_5:21062006-21062667	10.0716	6.62413	-0.604495
Ciclev10033029m.g	scaffold_4:2844907-2846923	202.561	133.238	-0.604352
Ciclev10000806m.g	scaffold_5:42079047-42083003	0.524787	0.345222	-0.604207
Ciclev10028651m.g	scaffold_8:2599716-2602090	62.4695	41.1064	-0.60379

Ciclev10010486m.g	scaffold_1:2711448-2715349	4.40196	2.89718	-0.603496
Ciclev10024805m.g	scaffold_7:4086279-4091817	4.91751	3.2375	-0.603048
Ciclev10022226m.g	scaffold_3:13591601-13592697	4.47607	2.94786	-0.602567
Ciclev10013354m.g	scaffold_6:14353818-14356639	11.6922	7.70229	-0.602186
Ciclev10021315m.g	scaffold_3:41497498-41503110	26.6793	17.5762	-0.602101
Ciclev10029337m.g	scaffold_8:10525313-10527112	94.5588	62.2953	-0.602088
Ciclev10015100m.g	scaffold_2:35997708-36001681	14.0732	9.27159	-0.602065
Ciclev10018054m.g	scaffold_2:28547150-28548412	1.07236	0.706489	-0.602047
Ciclev10017563m.g	scaffold_2:5650654-5706559	7.98639	5.26215	-0.601891
Ciclev10013092m.g	scaffold_6:21219689-21222183	11.8919	7.83895	-0.601252
Ciclev10015897m.g	scaffold_2:24501334-24503092	5.27301	3.47594	-0.601223
Ciclev10007286m.g	scaffold_1:24674084-24681956	16.9598	11.1808	-0.601101
Ciclev10002637m.g	scaffold_5:26507014-26509938	40.1612	26.478	-0.60101
Ciclev10031692m.g	scaffold_4:23090937-23096274	20.178	13.3032	-0.601003
Ciclev10014602m.g	scaffold_2:28577538-28581137	1.3612	0.897503	-0.600894
Ciclev10031153m.g	scaffold_4:24596915-24602183	7.6858	5.06818	-0.600729
Ciclev10009361m.g	scaffold_1:22810928-22812161	62.1608	40.992	-0.600664
Ciclev10000144m.g	scaffold_5:34063268-34070535	11.07	7.30252	-0.60019
Ciclev10027792m.g	scaffold_8:386835-392585	6.74034	4.44763	-0.599784
Ciclev10025668m.g	scaffold_7:16217360-16226054	8.37935	5.53027	-0.59949
Ciclev10000692m.g	scaffold_5:40112334-40116721	1.12382	0.741728	-0.599449
Ciclev10033108m.g	scaffold_4:17922696-17923859	111.242	73.4309	-0.599248
Ciclev10004432m.g	scaffold_9:2353441-2357780	55.6172	36.7129	-0.599243
Ciclev10015391m.g	scaffold_2:10963877-10968435	45.2618	29.888	-0.598728
Ciclev10026362m.g	scaffold_7:5243476-5245847	27.7776	18.343	-0.598693
Ciclev10025294m.g	scaffold_7:21036452-21040096	9.96704	6.58269	-0.598489
Ciclev10003535m.g	scaffold_5:39680521-39682746	31.6205	20.8908	-0.597989
Ciclev10001389m.g	scaffold_5:40818912-40822889	121.382	80.2232	-0.597458
Ciclev10012506m.g	scaffold_6:22925988-22927889	1.6896	1.11708	-0.596943
Ciclev10022323m.g	scaffold_3:4376923-4378771	1.32823	0.878349	-0.59664
Ciclev10017277m.g	scaffold_2:26182548-26186470	85.6485	56.6524	-0.59629
Ciclev10010873m.g	scaffold_367:8790-11187	5.0892	3.3668	-0.59606
Ciclev10026467m.g	scaffold_7:5438638-5439587	4.56093	3.01747	-0.595991
Ciclev10030268m.g	scaffold_8:4037788-4038178	33.7526	22.334	-0.595754
Ciclev10029488m.g	scaffold_8:18410915-18411414	11.3975	7.54391	-0.595334
Ciclev10027857m.g,Ciclev10030131m.g	scaffold_8:6456432-6468742	18.9999	12.5775	-0.59515
-	scaffold_5:20905239-20905533	28.0216	18.55	-0.595126
Ciclev10033364m.g	scaffold_4:16097597-16098485	14.7178	9.74549	-0.594758
Ciclev10016771m.g	scaffold_2:25203099-25204788	179.616	118.942	-0.594655
Ciclev10015828m.g	scaffold_2:31872091-31877015	10.822	7.17002	-0.59392
Ciclev10023045m.g	scaffold_3:27202251-27203036	4.61358	3.05686	-0.593838
Ciclev10004940m.g	scaffold_9:2324312-2328817	65.5208	43.4162	-0.593719
Ciclev10031329m.g	scaffold_4:19301322-19303451	98.1252	65.0317	-0.593482
Ciclev10027007m.g	scaffold_7:3756834-3758829	23.2006	15.377	-0.593385
Ciclev10031930m.g	scaffold_4:3458900-3461889	17.6889	11.7241	-0.593366
Ciclev10015330m.g,Ciclev10018105m.g,Ciclev10018331m.g	scaffold_2:28845910-28867192	6.06197	4.0179	-0.593345
Ciclev10023078m.g	scaffold_3:42139591-42142276	32.4478	21.5071	-0.59331
Ciclev10002572m.g	scaffold_5:37258471-37261610	40.1734	26.6364	-0.592843
Ciclev10007445m.g	scaffold_1:18454167-18459508	1.15081	0.763136	-0.592645
Ciclev10016666m.g	scaffold_2:34474182-34477395	29.8327	19.7833	-0.592612
Ciclev10010162m.g	scaffold_1:623430-627655	0.265135	0.175838	-0.592476
Ciclev10020393m.g,Ciclev10021980m.g	scaffold_3:38214467-38218474	639.429	424.109	-0.592348

Ciclev10000337m.g	scaffold_5:36452982-36458601	0.314668	0.208733	-0.592175
Ciclev10003923m.g	scaffold_5:33171947-33172895	0.962241	0.638435	-0.591859
Ciclev10025905m.g	scaffold_7:1714250-1717280	9.8532	6.53749	-0.591856
Ciclev10008366m.g	scaffold_1:27554896-27556656	341.191	226.383	-0.591812
Ciclev10027234m.g	scaffold_7:2678932-2679557	144.593	95.945	-0.59172
Ciclev10011137m.g	scaffold_6:23469416-23474907	1.90494	1.26426	-0.591453
Ciclev10014433m.g	scaffold_2:28753961-28756798	10.1034	6.70636	-0.591245
Ciclev10025293m.g	scaffold_7:2014753-2022603	7.30967	4.85249	-0.591082
Ciclev10026500m.g	scaffold_7:453411-454892	97.4493	64.7156	-0.590538
Ciclev10014881m.g	scaffold_2:7029165-7038442	32.0272	21.2701	-0.590468
Ciclev10020026m.g	scaffold_3:49307479-49313032	35.6875	23.7013	-0.590453
Ciclev10014418m.g	scaffold_2:35612409-35618022	7.90951	5.2534	-0.590337
Ciclev10019121m.g	scaffold_3:7621981-7626081	343.832	228.377	-0.590286
Ciclev10005604m.g	scaffold_9:29990608-29993190	11.5324	7.66231	-0.589845
Ciclev10026314m.g	scaffold_7:4361459-4364308	154.438	102.63	-0.589577
Ciclev10025093m.g	scaffold_7:2132854-2136209	1.30513	0.867314	-0.589562
Ciclev10012221m.g,Ciclev10012494m.g	scaffold_6:14604867-14628446	7.5978	5.04961	-0.58941
Ciclev10002687m.g	scaffold_5:17809522-17810742	1381.17	918.024	-0.589286
Ciclev10028201m.g	scaffold_8:4025925-4028221	15.0839	10.0274	-0.589057
Ciclev10007982m.g	scaffold_1:24547302-24552107	12.0637	8.0207	-0.588875
Ciclev10010094m.g	scaffold_1:13902212-13905427	0.372831	0.247959	-0.588417
Ciclev10005406m.g	scaffold_9:486641-488895	5.71687	3.80313	-0.588037
Ciclev10018523m.g	scaffold_3:44497361-44503252	22.3462	14.871	-0.587528
Ciclev10029225m.g	scaffold_8:7886182-7889942	75.9483	50.543	-0.587506
Ciclev10033512m.g	scaffold_4:24633687-24637440	17.775	11.8314	-0.587224
Ciclev10021214m.g	scaffold_3:3496382-3501362	5.36278	3.5698	-0.587136
Ciclev10022979m.g	scaffold_3:44008481-44011798	31.7783	21.1552	-0.587027
Ciclev10012243m.g	scaffold_6:14794014-14796956	47.2609	31.4644	-0.58693
Ciclev10021469m.g	scaffold_3:41043662-41046480	249.862	166.387	-0.586587
Ciclev10007063m.g	scaffold_9:1638769-1641073	77.9229	51.9053	-0.586166
Ciclev10012981m.g	scaffold_6:25466925-25469211	51.9941	34.6356	-0.586091
Ciclev10028096m.g	scaffold_8:523458-526795	17.9638	11.9682	-0.585889
Ciclev10022213m.g	scaffold_3:7425352-7428313	148.744	99.1142	-0.585672
Ciclev10014244m.g	scaffold_2:30853365-30860109	1.01935	0.679249	-0.585637
Ciclev10005345m.g	scaffold_9:24072148-24073547	3.73717	2.49035	-0.585598
Ciclev10012998m.g	scaffold_6:14757181-14759691	23.7147	15.8042	-0.58547
Ciclev10003259m.g	scaffold_5:953144-1184934	2.83145	1.88706	-0.585399
Ciclev10016239m.g	scaffold_2:14644603-14649552	2.29199	1.52764	-0.585294
Ciclev10024845m.g	scaffold_7:10990910-11007799	0.804997	0.536555	-0.585257
Ciclev10017236m.g	scaffold_2:29502998-29504535	109.875	73.2361	-0.585237
Ciclev10020607m.g	scaffold_3:42534420-42536728	134.21	89.4742	-0.584948
Ciclev10021683m.g	scaffold_3:4284296-4286306	2.12238	1.41494	-0.58494
Ciclev10029472m.g	scaffold_8:24413156-24415621	51.7755	34.5246	-0.584647
Ciclev10005764m.g	scaffold_9:314840-317604	13.5977	9.06981	-0.584214
Ciclev10017489m.g	scaffold_2:7935487-7939189	16.0795	10.7264	-0.58405
Ciclev10021606m.g	scaffold_3:2530707-2533942	28.7847	19.2037	-0.583915
Ciclev10031258m.g	scaffold_4:15087930-15266082	1.19087	0.794541	-0.583819
Ciclev10028728m.g	scaffold_8:6744234-6749656	56.2721	37.5452	-0.583792
-	scaffold_3:19834359-19911599	22.4733	14.9949	-0.583743
Ciclev10019450m.g	scaffold_3:6471378-6473755	6.62114	4.41834	-0.583575
Ciclev10008326m.g	scaffold_1:1794519-1800274	29.5474	19.7227	-0.583173
Ciclev10011968m.g	scaffold_6:24895745-24897651	99.6137	66.499	-0.583012
Ciclev10012332m.g	scaffold_6:2969526-2973147	140.239	93.6197	-0.583
Ciclev10017047m.g	scaffold_2:30267183-30270227	30.0538	20.0687	-0.582599
Ciclev10001051m.g	scaffold_5:29469172-29473572	22.933	15.3179	-0.582205

Ciclev10005438m.g	scaffold_9:996030-999921	61.7213	41.2284	-0.582128
-	scaffold_5:12452242-12452435	143.527	95.881	-0.582008
Ciclev10007997m.g	scaffold_1:24383593-24387969	11.3339	7.57257	-0.581785
Ciclev10032747m.g	scaffold_4:16817583-16984703	10.8537	7.25215	-0.581699
Ciclev10002696m.g	scaffold_5:37113170-37113949	71.7937	47.9978	-0.580887
Ciclev10031107m.g	scaffold_4:2461189-2463120	3.95533	2.64489	-0.580592
Ciclev10004902m.g	scaffold_9:16997186-17000071	76.0901	50.8828	-0.58053
-	scaffold_6:17865619-17866117	5.88774	3.93853	-0.580059
Ciclev10012397m.g	scaffold_6:7799873-7806411	43.1389	28.8605	-0.579892
Ciclev10026408m.g	scaffold_7:4707089-4709899	3.79295	2.53793	-0.57967
Ciclev10033657m.g	scaffold_4:21687637-21696046	7.35903	4.9243	-0.579596
Ciclev10001063m.g	scaffold_5:40554895-40558064	1.92486	1.28805	-0.579557
Ciclev10012654m.g	scaffold_6:10316998-10318328	565.196	378.216	-0.57954
Ciclev10011253m.g	scaffold_6:21693584-21696707	0.897156	0.600386	-0.579468
Ciclev10009453m.g	scaffold_1:24739226-24741152	480.594	321.717	-0.579027
Ciclev10031914m.g	scaffold_4:20595785-20597897	18.4277	12.3361	-0.578984
Ciclev10004273m.g	scaffold_9:1000193-1008334	41.9716	28.0979	-0.578949
Ciclev10019937m.g	scaffold_3:45193344-45196511	8.28651	5.54793	-0.578816
Ciclev10021937m.g	scaffold_3:26288658-26290508	27.4981	18.4121	-0.57868
-	scaffold_1:20429717-20430499	18.1795	12.1743	-0.578471
Ciclev10033733m.g	scaffold_4:532172-535848	25.0144	16.7538	-0.578268
Ciclev10029236m.g	scaffold_8:1119868-1122967	19.7866	13.2554	-0.577941
Ciclev10005037m.g	scaffold_9:3993909-3995504	2.64829	1.77414	-0.577941
Ciclev10026555m.g	scaffold_7:1847950-1850801	39.5738	26.5161	-0.577676
Ciclev10016334m.g	scaffold_2:34146750-34151105	33.2206	22.2612	-0.577544
Ciclev10011566m.g	scaffold_6:8097167-8106629	17.727	11.8797	-0.57745
Ciclev10011514m.g	scaffold_6:15539584-15544811	10.5629	7.07985	-0.577217
Ciclev10033223m.g	scaffold_4:367608-368120	42.572	28.5374	-0.577052
Ciclev10005376m.g	scaffold_9:1099025-1100983	3.93694	2.63943	-0.576847
Ciclev10012400m.g	scaffold_6:14363367-14367774	5.12456	3.43577	-0.576796
Ciclev10028228m.g	scaffold_8:924181-928129	67.2707	45.1053	-0.576681
Ciclev10015131m.g	scaffold_2:35401066-35405149	3.24935	2.17939	-0.576229
Ciclev10017354m.g	scaffold_2:6644288-6646611	84.4968	56.6759	-0.57616
Ciclev10000756m.g	scaffold_5:19740132-19745843	16.1563	10.837	-0.576139
Ciclev10030691m.g	scaffold_4:22855856-22860862	8.63763	5.79477	-0.575884
Ciclev10021801m.g	scaffold_3:1352169-1355852	194.969	130.801	-0.575872
Ciclev10016530m.g	scaffold_2:8375772-8378763	48.808	32.7561	-0.575357
Ciclev10019497m.g	scaffold_3:32065918-32071319	4.14019	2.77874	-0.575267
Ciclev10000287m.g	scaffold_5:36445868-36449020	1.61775	1.08631	-0.574545
Ciclev10029608m.g	scaffold_8:4581980-4582776	60.069	40.3391	-0.574441
Ciclev10029701m.g	scaffold_8:22064856-22066844	1306.61	877.528	-0.574311
Ciclev10002442m.g	scaffold_5:40300305-40304768	0.755914	0.507794	-0.573981
Ciclev10005824m.g	scaffold_9:25727826-25731198	5.72239	3.84413	-0.573959
Ciclev10027337m.g	scaffold_7:4093258-4096416	6.65346	4.46987	-0.573873
Ciclev10003384m.g	scaffold_5:43076052-43077027	22.4086	15.0545	-0.573854
Ciclev10004741m.g	scaffold_9:28536671-28539730	42.5052	28.5596	-0.573661
Ciclev10031017m.g	scaffold_4:11152390-11156903	3.78539	2.54434	-0.573152
Ciclev10021791m.g	scaffold_3:3556858-3558332	6.32945	4.25588	-0.572623
Ciclev10023020m.g	scaffold_3:50363247-50364138	24.0912	16.199	-0.572605
-	scaffold_9:17699876-17701330	5.628	3.78468	-0.572451
Ciclev10014491m.g	scaffold_2:23094790-23102366	48.9261	32.9027	-0.572397
Ciclev10002730m.g	scaffold_5:39509508-39511363	22.7948	15.3298	-0.572363
Ciclev10024381m.g	scaffold_3:42596914-42599083	18.4084	12.3805	-0.572298
Ciclev10016614m.g	scaffold_2:30603999-30606238	23.1322	15.5611	-0.571961
Ciclev10010105m.g	scaffold_1:26411427-26413944	122.12	82.1527	-0.571924
Ciclev10012756m.g	scaffold_6:2445029-2447489	3.33893	2.24616	-0.571924

Ciclev10009538m.g	scaffold_1:11005473-11012720	230.506	155.091	-0.571692
Ciclev10004158m.g	scaffold_9:17658352-17667100	0.182961	0.123132	-0.571326
Ciclev10014538m.g	scaffold_2:27730755-27735638	14.2561	9.59932	-0.570576
Ciclev10020291m.g	scaffold_3:43185628-43190699	11.8864	8.00563	-0.57022
Ciclev10032608m.g	scaffold_4:2798142-2799160	9.69362	6.52921	-0.570128
Ciclev10002575m.g	scaffold_5:33996379-33999120	11.8019	7.9502	-0.569955
Ciclev10029826m.g	scaffold_8:4555221-4556238	1.3319	0.897428	-0.569615
Ciclev10027569m.g	scaffold_7:15729787-15735001	0.766166	0.516252	-0.569581
Ciclev10009544m.g	scaffold_1:28521510-28524566	75.2211	50.7029	-0.569069
Ciclev10026159m.g	scaffold_7:19000089-19004615	21.7627	14.6725	-0.568737
Ciclev10021056m.g	scaffold_3:42672042-42676935	66.3955	44.7674	-0.568637
Ciclev10010015m.g	scaffold_1:912722-913512	356.218	240.272	-0.568091
Ciclev10020900m.g	scaffold_3:49414026-49417258	18.7849	12.6757	-0.567513
Ciclev10012667m.g	scaffold_6:24990725-24991982	17.9942	12.1424	-0.567477
Ciclev10031297m.g	scaffold_4:6930078-6933868	177.056	119.483	-0.567404
Ciclev10017814m.g	scaffold_2:31386656-31390943	31.3003	21.1419	-0.566072
Ciclev10020167m.g	scaffold_3:45807728-45812484	36.478	24.6394	-0.56606
Ciclev10017188m.g	scaffold_2:35199442-35200114	24.0143	16.225	-0.56567
-	scaffold_5:27310662-27311014	16.9102	11.4311	-0.564933
Ciclev10002668m.g	scaffold_5:38863413-38865093	11.9982	8.11349	-0.564419
Ciclev10013102m.g	scaffold_6:16199887-16202624	18.7389	12.6723	-0.564357
-	scaffold_448:64-318	111.766	75.5904	-0.5642
Ciclev10006392m.g	scaffold_9:29150202-29150664	12.7055	8.5934	-0.564148
Ciclev10026525m.g	scaffold_7:13322107-13323414	86.5508	58.5544	-0.563771
Ciclev10011277m.g	scaffold_6:19937889-19942725	0.438967	0.297142	-0.562963
Ciclev10022296m.g	scaffold_3:11023750-11026247	20.8452	14.1164	-0.562343
Ciclev10016688m.g	scaffold_2:10097307-10100347	87.5236	59.2793	-0.562145
Ciclev10002041m.g	scaffold_5:40637572-40639211	1.40564	0.95209	-0.562059
Ciclev10031300m.g,Ciclev10032410m.g	scaffold_4:16460022-16475213	20.0752	13.6021	-0.561587
Ciclev10029535m.g	scaffold_8:844843-849930	4.99374	3.38376	-0.561496
Ciclev10004803m.g	scaffold_9:5556608-5562040	19.0112	12.8826	-0.561431
Ciclev10020247m.g	scaffold_3:33005684-33007151	1.90101	1.28836	-0.561235
Ciclev10018341m.g	scaffold_2:25351240-25353303	19.0672	12.9229	-0.561155
Ciclev10032509m.g	scaffold_4:22451689-22454024	15.913	10.7878	-0.560799
Ciclev10013930m.g	scaffold_2451:33-975	1.58932	1.0776	-0.560593
Ciclev10027926m.g	scaffold_8:23603377-23606134	18.4283	12.4968	-0.560363
Ciclev10015440m.g	scaffold_2:35254805-35258303	36.3863	24.6752	-0.560334
-	scaffold_7:13769732-13772489	156.541	106.184	-0.559972
Ciclev10003032m.g,Ciclev10003081m.g	scaffold_5:181773-184391	5.61244	3.80724	-0.559881
Ciclev10003349m.g	scaffold_5:35348250-35348723	131.942	89.5043	-0.559878
Ciclev10012954m.g	scaffold_6:20514945-20517494	30.144	20.4499	-0.559777
Ciclev10031524m.g	scaffold_4:14074639-14076918	2.90915	1.97372	-0.559682
Ciclev10025725m.g	scaffold_7:1677727-1684978	4.54156	3.08143	-0.559587
Ciclev10004541m.g	scaffold_9:13517151-13519780	23.9805	16.2748	-0.559217
Ciclev10029517m.g	scaffold_8:23443331-23445027	135.93	92.2566	-0.559141
Ciclev10021518m.g	scaffold_3:37084726-37087272	16.5215	11.2136	-0.559096
Ciclev10012626m.g	scaffold_6:22095196-22096556	3.82877	2.59884	-0.559014
Ciclev10032864m.g	scaffold_4:12356080-12357713	11.7919	8.004	-0.558999
Ciclev10012931m.g	scaffold_6:22270196-22276195	7.48695	5.08232	-0.55889
Ciclev10004835m.g	scaffold_9:3764738-3769633	3.18645	2.16306	-0.558877
Ciclev10018782m.g,Ciclev10024606m.g	scaffold_3:28065477-28341901	379.661	257.79	-0.558517
Ciclev10006602m.g	scaffold_9:4253668-4255801	0.50702	0.344492	-0.55757
Ciclev10020464m.g	scaffold_3:42874813-42879300	57.338	38.9595	-0.557516

Ciclev10031156m.g	scaffold_4:20487598-20490030	2.25474	1.53216	-0.557396
Ciclev10017816m.g	scaffold_2:5273993-5337902	0.424357	0.288365	-0.557383
Ciclev10015032m.g	scaffold_2:11832033-11834522	3.93389	2.67406	-0.556923
Ciclev10014716m.g	scaffold_2:32792250-32798007	11.619	7.90009	-0.55655
Ciclev10016689m.g	scaffold_2:35790870-35793694	32.2396	21.9251	-0.55625
Ciclev10017817m.g	scaffold_2:18456571-18459966	8.76249	5.96057	-0.555891
Ciclev10023729m.g	scaffold_3:47635843-47636167	3.64616	2.48045	-0.555772
Ciclev10004356m.g	scaffold_9:25619086-25625135	2.70641	1.84154	-0.555467
Ciclev10025284m.g	scaffold_7:1993994-1999805	9.85393	6.70755	-0.554913
Ciclev10007883m.g	scaffold_1:22824478-22829259	10.2941	7.00737	-0.554874
Ciclev10029547m.g	scaffold_8:1895814-1896813	164.192	111.768	-0.554869
Ciclev10011938m.g	scaffold_6:18741180-18744166	5.09964	3.47167	-0.554766
Ciclev10028274m.g	scaffold_8:12967156-13120570	72.8542	49.5982	-0.554725
Ciclev10007095m.g	scaffold_9:6226730-6229337	44.7482	30.4685	-0.554513
Ciclev10020437m.g,Ciclev10021087m.g,Ciclev10021567m.g	scaffold_3:35372249-35712253	3.64569	2.4825	-0.554399
Ciclev10019662m.g	scaffold_3:46124030-46130078	5.4871	3.73759	-0.553935
Ciclev10012574m.g	scaffold_6:25206938-25210092	43.0176	29.3038	-0.553842
Ciclev10028726m.g	scaffold_8:22998842-23000849	11.3338	7.72144	-0.553688
Ciclev10000720m.g	scaffold_5:38180687-38186303	13.4153	9.1404	-0.553553
Ciclev10011691m.g	scaffold_6:16503593-16506386	344.421	234.764	-0.552961
Ciclev10006094m.g	scaffold_9:31217904-31220698	26.5979	18.1368	-0.552396
Ciclev10004329m.g	scaffold_9:21606471-21610383	0.848816	0.578816	-0.552347
Ciclev10000874m.g	scaffold_5:31403726-31408001	4.09895	2.79595	-0.551912
Ciclev10015683m.g	scaffold_2:9291189-9294021	23.5354	16.054	-0.551905
Ciclev10031619m.g	scaffold_4:22231874-22237604	23.1336	15.7827	-0.551648
-	scaffold_5:10869264-10869778	12.8151	8.74418	-0.551452
Ciclev10012936m.g	scaffold_6:19365714-19369272	21.6186	14.7529	-0.551274
Ciclev10017675m.g,Ciclev10018303m.g	scaffold_2:35944544-35948862	15.9461	10.8833	-0.55109
Ciclev10025088m.g	scaffold_7:4710775-4716941	92.0292	62.8107	-0.551081
Ciclev10003670m.g	scaffold_5:33062912-33063962	6.29327	4.29662	-0.55061
Ciclev10017339m.g	scaffold_2:7213321-7218224	13.2106	9.01982	-0.550523
Ciclev10004849m.g	scaffold_9:28934713-28938611	4.04212	2.76	-0.550444
Ciclev10026368m.g	scaffold_7:613118-615369	21.6669	14.7946	-0.550425
Ciclev10030735m.g	scaffold_4:18223085-18225488	0.409586	0.279677	-0.550405
Ciclev10024980m.g	scaffold_7:7295516-7300071	32.2111	21.9982	-0.550171
Ciclev10031458m.g,Ciclev10031461m.g	scaffold_4:14370384-14385042	134.044	91.5557	-0.549984
Ciclev10008473m.g	scaffold_1:2804668-2806860	0.855365	0.584239	-0.549982
Ciclev10021507m.g	scaffold_3:1670019-1673413	48.637	33.2247	-0.549798
Ciclev10014296m.g	scaffold_2:26658861-26663240	26.7646	18.286	-0.549587
Ciclev10002220m.g	scaffold_5:39207052-39212539	13.5129	9.23244	-0.549559
Ciclev10002943m.g	scaffold_5:16030839-16039385	1.06127	0.725203	-0.549335
Ciclev10031645m.g	scaffold_4:23659237-23662888	33.6476	23.0028	-0.548697
Ciclev10010178m.g	scaffold_1:25323905-25324247	5.82748	3.98683	-0.547631
Ciclev10013829m.g	scaffold_6:21288355-21290142	1.73347	1.1863	-0.547189
Ciclev10026363m.g	scaffold_7:4833777-4835148	9.67723	6.62365	-0.546967
Ciclev10020981m.g	scaffold_3:49529027-49531042	15.083	10.3237	-0.546963
Ciclev10029078m.g	scaffold_8:219688-222334	10.3732	7.10184	-0.546589
Ciclev10011721m.g	scaffold_6:23488305-23491728	15.464	10.5885	-0.546416
Ciclev10021186m.g	scaffold_3:44588334-44589503	2.5125	1.72064	-0.546177
Ciclev10020808m.g,Ciclev10024646m.g	scaffold_3:48542258-48545077	8.33888	5.71085	-0.54615
Ciclev10023058m.g	scaffold_3:46411067-46412389	51.316	35.1503	-0.545869

Ciclev10021941m.g	scaffold_3:2106809-2109266	29.7532	20.381	-0.545816
Ciclev10000073m.g	scaffold_5:35118180-35121835	2.47977	1.69878	-0.545704
Ciclev10005408m.g	scaffold_9:9805898-9809222	24.3427	16.677	-0.545629
Ciclev10011141m.g	scaffold_6:14266025-14269088	37.403	25.6321	-0.545202
Ciclev10015371m.g	scaffold_2:15477859-15615778	6.92061	4.7427	-0.54519
Ciclev10002797m.g	scaffold_5:41899221-41901896	42.0777	28.8418	-0.544895
Ciclev10001879m.g	scaffold_5:35644332-35646412	6.21331	4.25992	-0.544537
Ciclev10025594m.g	scaffold_7:849454-854913	6.50152	4.45758	-0.544517
Ciclev10013662m.g	scaffold_6:16378857-16383433	4.33711	2.97372	-0.544468
Ciclev10017361m.g	scaffold_2:19184974-19186538	67.0341	45.9744	-0.544065
Ciclev10022963m.g	scaffold_3:3833460-3834763	21.9292	15.043	-0.54376
Ciclev10002023m.g	scaffold_5:40740373-40741597	1.78312	1.2232	-0.543748
Ciclev10003359m.g	scaffold_5:3980660-4069314	18.4513	12.6577	-0.543704
Ciclev10004463m.g	scaffold_9:29432438-29439510	31.1485	21.3706	-0.543536
Ciclev10011440m.g	scaffold_6:20707457-20713093	24.5961	16.8779	-0.543296
Ciclev10009866m.g	scaffold_1:20170969-20172066	54.8009	37.6056	-0.543252
Ciclev10024304m.g	scaffold_3:6242973-6244997	60.7432	41.6837	-0.543241
Ciclev10016907m.g	scaffold_2:25847350-25849685	5.84028	4.0086	-0.542937
Ciclev10001115m.g	scaffold_5:15776475-15781159	28.6182	19.6433	-0.542893
Ciclev10022237m.g	scaffold_3:9093722-9095707	1.9139	1.31403	-0.542514
Ciclev10000815m.g	scaffold_5:36726565-36731153	3.85889	2.64962	-0.5424
Ciclev10016370m.g	scaffold_2:13437794-13439664	4.32419	2.96988	-0.542027
Ciclev10003265m.g	scaffold_5:37544471-37560859	33.2811	22.8593	-0.54192
Ciclev10008173m.g	scaffold_1:26244008-26247997	22.2202	15.2654	-0.541608
Ciclev10023054m.g	scaffold_3:43647709-43648340	31.5729	21.6966	-0.541215
Ciclev10015883m.g	scaffold_2:33769959-33771318	1.62282	1.11522	-0.541174
Ciclev10032281m.g	scaffold_4:23166504-23168365	133.196	91.544	-0.541011
-	scaffold_6:21667073-21673152	80.8463	55.5679	-0.54093
Ciclev10002990m.g	scaffold_5:36018226-36021021	1.39093	0.956044	-0.540899
Ciclev10032807m.g	scaffold_4:22597164-22601079	222.172	152.72	-0.540792
Ciclev10004281m.g	scaffold_9:3443237-3447919	1.39794	0.961056	-0.540612
Ciclev10031369m.g	scaffold_4:2004065-2007772	8.59235	5.90744	-0.540518
Ciclev10001591m.g	scaffold_5:40526247-40529184	122.469	84.2269	-0.540062
Ciclev10009324m.g	scaffold_1:329070-331103	396.58	272.802	-0.53976
Ciclev10024864m.g	scaffold_7:5245913-5251737	8.46813	5.82522	-0.539731
Ciclev10028757m.g	scaffold_8:19730603-19734413	1.7402	1.19726	-0.539522
Ciclev10028752m.g	scaffold_8:1892226-1894510	6.70218	4.61363	-0.53873
Ciclev10008455m.g	scaffold_1:17487145-17491004	17.3115	11.9171	-0.538699
Ciclev10026827m.g	scaffold_7:647239-648727	24.5444	16.8962	-0.538691
Ciclev10020057m.g	scaffold_3:4944105-4947636	51.7383	35.6176	-0.538644
Ciclev10021657m.g	scaffold_3:2122422-2125916	10.9185	7.51843	-0.538269
Ciclev10006308m.g	scaffold_9:1685406-1687081	181.946	125.293	-0.5382
Ciclev10002251m.g	scaffold_5:38514269-38516995	49.4491	34.0594	-0.53789
Ciclev10012507m.g	scaffold_6:4003604-4005679	27.9378	19.2453	-0.537713
Ciclev10006738m.g	scaffold_9:2876202-2884325	8.96676	6.17736	-0.537595
Ciclev10019215m.g	scaffold_3:2250888-2258522	16.8486	11.6078	-0.537534
Ciclev10015084m.g,Ciclev10017405m.g	scaffold_2:23789833-23796225	12.2734	8.45655	-0.537392
Ciclev10030603m.g	scaffold_4:16331419-16341855	15.8361	10.9113	-0.537392
Ciclev10008821m.g	scaffold_1:28782168-28786731	15.8532	10.9234	-0.537347
Ciclev10004925m.g	scaffold_9:5680840-5687483	48.2259	33.2311	-0.537277
Ciclev10026434m.g	scaffold_7:16614910-16616240	3.56081	2.45423	-0.536933
Ciclev10026989m.g	scaffold_7:5526512-5531444	0.82473	0.568497	-0.536769
Ciclev10015898m.g	scaffold_2:36156489-36160964	18.838	12.9869	-0.53659
Ciclev10016359m.g	scaffold_2:30418999-30425113	45.0772	31.0816	-0.536341
Ciclev10011613m.g	scaffold_6:20005404-20007246	11.4759	7.91358	-0.536207

Ciclev10002472m.g	scaffold_5:32888505-32889497	6.72716	4.6396	-0.535996
Ciclev10011346m.g	scaffold_6:7983787-7991526	20.6224	14.2244	-0.535843
-	scaffold_4:22944705-22960987	9.79417	6.75719	-0.5355
Ciclev10004864m.g	scaffold_9:29500558-29503424	2.28276	1.57503	-0.535402
Ciclev10027830m.g	scaffold_8:22531206-22540728	15.4169	10.6386	-0.535202
Ciclev10014442m.g,Ciclev10014448m.g	scaffold_2:12624190-12635533	6.72624	4.64155	-0.535193
Ciclev10024885m.g	scaffold_7:1729021-1732466	26.3048	18.1526	-0.535151
Ciclev10022345m.g	scaffold_3:48528819-48531316	33.381	23.0362	-0.535123
Ciclev10023445m.g	scaffold_3:25248366-25288105	79.0308	54.5391	-0.535122
Ciclev10003942m.g	scaffold_5:28908467-28909137	1.35191	0.933114	-0.534874
Ciclev10002833m.g	scaffold_5:29475603-29479771	40.9173	28.2457	-0.534681
Ciclev10024407m.g	scaffold_3:10960688-10961096	33.413	23.0654	-0.534677
Ciclev10031839m.g	scaffold_4:24251847-24254781	11.3839	7.86022	-0.534358
Ciclev10027324m.g	scaffold_7:7869668-7878895	4.03683	2.78757	-0.534215
Ciclev10000149m.g	scaffold_5:19692511-19713573	22.5592	15.5818	-0.533855
Ciclev10014991m.g	scaffold_2:22635763-22640239	765.771	529.036	-0.533546
Ciclev10029730m.g	scaffold_8:4461644-4464561	2.17359	1.50165	-0.533527
Ciclev10024036m.g	scaffold_3:39454215-39457279	21.9367	15.1609	-0.532991
Ciclev10015547m.g	scaffold_2:19176888-19181602	35.98	24.8704	-0.532767
Ciclev10031206m.g	scaffold_4:23431991-23438280	22.0799	15.2632	-0.532676
Ciclev10032231m.g	scaffold_4:20863552-20865814	27.3367	18.9016	-0.532333
Ciclev10009353m.g	scaffold_1:24636878-24638600	69.7808	48.2541	-0.532177
Ciclev10026110m.g	scaffold_7:19037369-19047271	51.4497	35.5887	-0.531742
Ciclev10015934m.g	scaffold_2:35693248-35696515	343.843	237.853	-0.531679
Ciclev10026510m.g	scaffold_7:2461705-2462587	7.20175	4.98319	-0.531279
Ciclev10018935m.g	scaffold_3:42800194-42807761	3.29362	2.27937	-0.531039
Ciclev10028894m.g	scaffold_8:5362568-5364774	102.031	70.6174	-0.530917
Ciclev10031023m.g	scaffold_4:22074826-22078969	9.05767	6.27035	-0.530594
Ciclev10008480m.g	scaffold_1:8030493-8032956	1.48981	1.03161	-0.530227
Ciclev10031321m.g	scaffold_4:1761712-1763308	2.4694	1.71024	-0.529962
Ciclev10011435m.g	scaffold_6:21182616-21185511	30.51	21.133	-0.529787
-	scaffold_5:25583143-25583732	4.89614	3.39241	-0.529332
Ciclev10008836m.g	scaffold_1:526812-529150	80.3645	55.6868	-0.529223
Ciclev10029300m.g	scaffold_8:24350875-24353308	85.0216	58.9181	-0.529119
Ciclev10028652m.g	scaffold_8:24198802-24201776	103.177	71.5068	-0.528968
Ciclev10032918m.g	scaffold_4:23936653-23938195	4.00427	2.77531	-0.528892
Ciclev10027791m.g	scaffold_8:18082602-18092803	7.09977	4.92126	-0.528745
Ciclev10027758m.g	scaffold_8:6720458-6728359	58.4062	40.4849	-0.528736
Ciclev10017170m.g	scaffold_2:31668226-31669231	2490.49	1726.48	-0.528595
Ciclev10012093m.g	scaffold_6:25072091-25074076	1.29108	0.89519	-0.528314
Ciclev10025641m.g	scaffold_7:14123946-14154295	17.5385	12.1619	-0.528153
Ciclev10009884m.g	scaffold_1:10687291-10689065	7.28696	5.05328	-0.528096
Ciclev10014384m.g	scaffold_2:455594-457955	1.12552	0.78053	-0.528062
Ciclev10028670m.g	scaffold_8:6141139-6143433	171.581	118.997	-0.527965
Ciclev10014990m.g	scaffold_2:27340085-27344491	3.09244	2.1457	-0.527296
Ciclev10007260m.g	scaffold_1:23211155-23216515	0.191223	0.132703	-0.527056
Ciclev10018381m.g	scaffold_2:34537261-34537814	12.6214	8.7589	-0.52705
Ciclev10021422m.g	scaffold_3:48433759-48435013	2.03481	1.41219	-0.526964
Ciclev10016448m.g	scaffold_2:24336765-24341972	23.7976	16.5198	-0.526626
Ciclev10020074m.g	scaffold_3:9814006-9820633	10.6362	7.38424	-0.526467
Ciclev10032055m.g	scaffold_4:22554705-22557986	18.4456	12.8074	-0.526299
-	scaffold_8:8101150-8101602	14.7682	10.2544	-0.526247
Ciclev10011633m.g	scaffold_6:23876496-23881814	27.3444	18.9872	-0.526215
Ciclev10027537m.g	scaffold_7:12352172-12754529	0.522403	0.362755	-0.526165
Ciclev10002907m.g	scaffold_5:42014992-42015616	29.9052	20.7667	-0.526124

Ciclev10022971m.g	scaffold_3:22179353-22179947	5.16451	3.58645	-0.526072
Ciclev10002099m.g	scaffold_5:37399361-37401504	3871.26	2688.82	-0.525828
Ciclev10033049m.g	scaffold_4:20614543-20616161	16.2727	11.3027	-0.525793
Ciclev10000657m.g	scaffold_5:441330-538185	45.7904	31.8088	-0.52562
Ciclev10028543m.g	scaffold_8:14335179-14340370	98.7155	68.5838	-0.525409
Ciclev10009999m.g	scaffold_1:936818-939301	38.3082	26.6212	-0.52508
Ciclev10032761m.g	scaffold_4:16410255-16414481	74.9862	52.1118	-0.525017
Ciclev10012000m.g	scaffold_6:14402663-14405661	31.1273	21.6322	-0.524996
Ciclev10020033m.g	scaffold_3:1731127-1732943	150.828	104.844	-0.524667
Ciclev10024397m.g	scaffold_3:18676450-18678412	18.1868	12.6428	-0.524576
Ciclev10012860m.g	scaffold_6:18156134-18159134	32.044	22.2762	-0.524554
Ciclev10020604m.g	scaffold_3:5494685-5499081	8.13127	5.65317	-0.524422
Ciclev10001769m.g	scaffold_5:39014888-39043868	12.8252	8.91687	-0.524377
Ciclev10013162m.g	scaffold_6:23843497-23845380	198.872	138.299	-0.524048
Ciclev10028537m.g	scaffold_8:6315012-6322458	39.043	27.1512	-0.524047
Ciclev10016881m.g	scaffold_2:29152040-29154181	73.0517	50.8049	-0.523951
Ciclev10002684m.g	scaffold_5:35558555-35562044	19.9732	13.8907	-0.523943
Ciclev10016841m.g	scaffold_2:15774221-15776937	7.93491	5.51889	-0.523836
Ciclev10014896m.g	scaffold_2:34300503-34304805	2.97569	2.07024	-0.52342
Ciclev10002824m.g	scaffold_5:35769297-35774649	51.5592	35.8715	-0.523391
Ciclev10017197m.g	scaffold_2:24336765-24341972	503.808	350.561	-0.523208
Ciclev10020450m.g	scaffold_3:47429290-47431121	9.58805	6.67193	-0.523133
Ciclev10022553m.g	scaffold_3:2110307-2113166	29.0974	20.25	-0.522971
Ciclev10032564m.g	scaffold_4:2980991-2983619	53.6008	37.306	-0.522847
Ciclev10025569m.g	scaffold_7:10000260-10003641	190.536	132.618	-0.522783
Ciclev10017275m.g	scaffold_2:29726361-29727127	70.0188	48.7529	-0.522255
Ciclev10011592m.g	scaffold_6:10729033-10731466	17.9621	12.5069	-0.52223
Ciclev10026722m.g	scaffold_7:8898625-8899984	3549.62	2471.65	-0.522191
Ciclev10031262m.g	scaffold_4:25299425-25303339	42.1683	29.3653	-0.522045
Ciclev10019939m.g	scaffold_3:5911440-5915726	57.9661	40.3797	-0.521579
Ciclev10008560m.g	scaffold_1:4619627-4622108	18.1732	12.6599	-0.521548
Ciclev10032368m.g	scaffold_4:18423062-18426424	23.8181	16.5928	-0.521506
Ciclev10011724m.g	scaffold_6:20987327-20988808	1.61714	1.12665	-0.521402
Ciclev10005167m.g	scaffold_9:196935-201673	16.4643	11.4713	-0.521313
Ciclev10014112m.g	scaffold_2:33025580-33034988	12.1613	8.47343	-0.521281
Ciclev10022474m.g	scaffold_3:23043180-23046455	45.1398	31.4576	-0.520989
Ciclev10001581m.g	scaffold_5:35791061-35793496	1.9737	1.37548	-0.520966
Ciclev10008817m.g	scaffold_1:25017747-25021583	18.0514	12.5813	-0.520833
Ciclev10008420m.g	scaffold_1:25235928-25240774	4.15959	2.89964	-0.520569
Ciclev10002480m.g	scaffold_5:39797662-39799186	153.768	107.192	-0.52056
Ciclev10014038m.g	scaffold_2:31811546-31820596	0.21346	0.148805	-0.520547
Ciclev10016939m.g	scaffold_2:4471155-4473536	28.1752	19.6429	-0.520416
Ciclev10003424m.g	scaffold_5:22623764-22625441	17.1888	11.9848	-0.520272
Ciclev10024186m.g	scaffold_3:29963167-29978842	3.0844	2.15127	-0.519803
Ciclev10012549m.g	scaffold_6:2365076-2367978	71.7314	50.0402	-0.519518
Ciclev10021726m.g	scaffold_3:42166129-42169227	21.1011	14.7248	-0.519071
Ciclev10020345m.g	scaffold_3:4219474-4224569	35.0829	24.4826	-0.519015
Ciclev10002389m.g	scaffold_5:16056455-16060841	28.2177	19.6918	-0.519007
Ciclev10014293m.g	scaffold_2:11194588-11210308	17.2772	12.0604	-0.518583
Ciclev10022382m.g	scaffold_3:4840946-4864738	1.89516	1.3235	-0.517964
Ciclev10027491m.g,Ciclev10027586m.g	scaffold_7:19600588-19611360	21.0612	14.711	-0.517692
Ciclev10019878m.g	scaffold_3:41780338-41785634	20.1321	14.0653	-0.517358
Ciclev10032192m.g	scaffold_4:2333546-2335111	260.412	181.959	-0.517182
Ciclev10002817m.g	scaffold_5:41651170-41652391	420.817	294.121	-0.516785
Ciclev10001356m.g	scaffold_5:16030839-16039385	18.7901	13.1333	-0.516741

-	scaffold_8:23424035-23426105	12.5648	8.78262	-0.51667
Ciclev10019949m.g	scaffold_3:7286660-7290182	32.2305	22.5288	-0.516655
Ciclev10011405m.g	scaffold_6:17589274-17593051	30.3002	21.1801	-0.516619
Ciclev10028837m.g,Ciclev10029637m.g,Ciclev10029742m.g,Ciclev10029895m.g	scaffold_8:18973864-19114160	67.8715	47.4441	-0.516575
-	scaffold_4:21357267-21358064	170.765	119.376	-0.5165
Ciclev10026041m.g	scaffold_7:3180143-3181724	110.99	77.5997	-0.516304
Ciclev10016372m.g	scaffold_2:35836829-35839763	13.9785	9.77395	-0.516198
Ciclev10005690m.g	scaffold_9:5021287-5065070	85.0012	59.4359	-0.516149
Ciclev10018411m.g	scaffold_2:29949348-29952324	12.9877	9.08166	-0.516113
Ciclev10022818m.g	scaffold_3:42303544-42306061	50.7873	35.514	-0.51608
Ciclev10020822m.g	scaffold_3:17627459-17631734	19.9944	13.9828	-0.515943
Ciclev10005651m.g	scaffold_9:3774211-3777219	40.8358	28.5589	-0.515896
Ciclev10020242m.g,Ciclev10020824m.g	scaffold_3:49062493-49071728	25.0836	17.5427	-0.51587
Ciclev10012512m.g	scaffold_6:20039906-20042958	2.78575	1.94836	-0.515803
Ciclev10027954m.g	scaffold_8:12800121-12845493	393.576	275.336	-0.51545
-	scaffold_9:18071150-18072307	68.2463	47.7437	-0.51544
Ciclev10019729m.g	scaffold_3:42391565-42399506	31.5966	22.1085	-0.515167
Ciclev10009685m.g	scaffold_1:25955509-25957753	10.3601	7.2496	-0.515064
Ciclev10014068m.g	scaffold_2:23840773-23849610	5.57081	3.89969	-0.514528
Ciclev10018623m.g	scaffold_3:1837796-1847421	23.2921	16.307	-0.51435
Ciclev10008608m.g	scaffold_1:5353693-5358135	13.6783	9.57821	-0.514063
Ciclev10016457m.g	scaffold_2:23685042-23687598	9.74788	6.82613	-0.51402
Ciclev10000069m.g	scaffold_5:8532279-8539677	65.3875	45.7944	-0.513844
Ciclev10006706m.g	scaffold_9:16408326-16415084	7.42245	5.19876	-0.513727
-	scaffold_5:26568616-26569250	15.0425	10.5369	-0.513589
Ciclev10032104m.g	scaffold_4:14463530-14465797	8.15681	5.71382	-0.513548
Ciclev10004515m.g	scaffold_9:460017-463975	12.8703	9.01649	-0.513413
Ciclev10018951m.g	scaffold_3:43340239-43345297	5.836	4.08894	-0.513253
Ciclev10013001m.g	scaffold_6:9949966-9952126	147.968	103.673	-0.513238
Ciclev10011237m.g	scaffold_6:6694889-6751336	27.4955	19.275	-0.51246
Ciclev10025467m.g	scaffold_7:1393893-1399491	34.7722	24.3806	-0.512202
Ciclev10009506m.g	scaffold_1:10830570-10831255	3.52016	2.46862	-0.511938
Ciclev10016840m.g	scaffold_2:28156068-28158670	23.8148	16.7039	-0.511672
Ciclev10021719m.g	scaffold_3:31550421-31552673	4.84125	3.39733	-0.510978
Ciclev10026597m.g	scaffold_7:11163358-11167847	282.377	198.164	-0.510926
Ciclev10002194m.g	scaffold_5:34801351-34804440	63.6342	44.6575	-0.5109
Ciclev10003415m.g	scaffold_5:953144-1184934	4.99424	3.50546	-0.510661
-	scaffold_3:48301733-48306301	9.61148	6.74714	-0.510483
Ciclev10005510m.g	scaffold_9:28648621-28651552	47.6638	33.4642	-0.510277
Ciclev10033893m.g	scaffold_4:8205602-8206194	66.484	46.6874	-0.509975
Ciclev10016449m.g	scaffold_2:12110140-12112419	7.31697	5.13857	-0.509879
Ciclev10005116m.g	scaffold_9:2640023-2644313	25.2051	17.7015	-0.509845
Ciclev10007428m.g	scaffold_1:27713331-27716492	19.6625	13.8113	-0.509597
Ciclev10032688m.g	scaffold_4:21601247-21603994	46.393	32.595	-0.509256
Ciclev10012215m.g	scaffold_6:24975343-24986759	34.228	24.0519	-0.509025
Ciclev10016417m.g	scaffold_2:7944031-7949222	101.828	71.5556	-0.508992
Ciclev10025355m.g	scaffold_7:3594985-3597230	22.5378	15.8385	-0.508905
Ciclev10001262m.g	scaffold_5:41201214-41208112	22.6285	15.9043	-0.508724
Ciclev10023737m.g	scaffold_3:5467386-5470407	21.1746	14.8849	-0.508481
Ciclev10007939m.g	scaffold_1:6388569-6391033	0.868261	0.610378	-0.508428
-	scaffold_1:18388978-18389295	17.5473	12.3361	-0.508362
Ciclev10004172m.g	scaffold_9:1732240-1737412	1.07308	0.754471	-0.50822
Ciclev10005534m.g	scaffold_9:4252386-4253603	1.32833	0.93405	-0.508042

Ciclev10031270m.g	scaffold_4:20564205-20569389	5.46248	3.84124	-0.507984
Ciclev10031495m.g	scaffold_4:1421418-1423124	1.2984	0.913285	-0.507601
Ciclev10019904m.g	scaffold_3:5665575-5667630	1.46458	1.03039	-0.507294
Ciclev10022489m.g	scaffold_3:1056115-1057706	53.6748	37.7725	-0.506908
Ciclev10002214m.g	scaffold_5:39200806-39203977	16.0944	11.3265	-0.506861
Ciclev10029363m.g	scaffold_8:19352993-19353751	18.8736	13.283	-0.506789
Ciclev10015122m.g	scaffold_2:33018147-33022811	21.6496	15.237	-0.50676
Ciclev10001528m.g	scaffold_5:6726816-6730833	13.9821	9.84099	-0.506711
Ciclev10017396m.g	scaffold_2:13396980-13397490	30.5565	21.5072	-0.50666
Ciclev10015638m.g	scaffold_2:12308672-12314225	60.5037	42.5927	-0.506419
Ciclev10016625m.g	scaffold_2:3758359-3761134	11.8672	8.35425	-0.5064
Ciclev10008410m.g	scaffold_1:10946315-10961377	69.0848	48.6364	-0.506331
Ciclev10008105m.g	scaffold_1:9328906-9330925	21.2601	14.9674	-0.506326
Ciclev10010600m.g	scaffold_1:24405245-24407298	14.612	10.2882	-0.506152
Ciclev10021886m.g	scaffold_3:38337962-38342205	9.55433	6.72792	-0.505993
Ciclev10014175m.g	scaffold_2:21993084-21998728	12.9917	9.14986	-0.505774
Ciclev10027882m.g	scaffold_8:224463-229899	41.8794	29.4969	-0.505677
Ciclev10020035m.g	scaffold_3:47112185-47115673	9.25294	6.51733	-0.505631
Ciclev10014307m.g	scaffold_2:14509026-14516757	12.784	9.0046	-0.505603
Ciclev10001114m.g	scaffold_5:42875802-42879064	1910.14	1345.64	-0.505388
Ciclev10014804m.g	scaffold_2:14143536-14146003	5.53519	3.89947	-0.505356
Ciclev10019512m.g	scaffold_3:2782535-2788212	21.5315	15.1705	-0.505184
Ciclev10026786m.g	scaffold_7:3885649-3886115	0.474789	0.334585	-0.504916
Ciclev10020735m.g	scaffold_3:5271978-5274369	75.5351	53.2427	-0.504564
Ciclev10014233m.g	scaffold_2:8583245-8588715	40.7455	28.7213	-0.504518
Ciclev10014407m.g	scaffold_2:31781299-31785480	95.0687	67.0154	-0.504477
Ciclev10010703m.g	scaffold_1:2638872-2646073	13.4877	9.50836	-0.504371
Ciclev10026950m.g	scaffold_7:17934378-17935818	0.909591	0.641398	-0.503999
Ciclev10022066m.g	scaffold_3:45556272-45557951	27.7714	19.5847	-0.503874
Ciclev10021819m.g	scaffold_3:2319674-2322962	136.93	96.5748	-0.503717
Ciclev10027876m.g	scaffold_8:21860620-21867477	25.8024	18.1982	-0.503705
Ciclev10019772m.g	scaffold_3:3856394-3859467	7.87903	5.55717	-0.503668
Ciclev10026836m.g	scaffold_7:6719213-6720376	69.114	48.7555	-0.503412
Ciclev10013103m.g	scaffold_6:21787509-21787967	2.41755	1.70544	-0.503396
Ciclev10025991m.g	scaffold_7:4008632-4012745	16.1957	11.4254	-0.503372
Ciclev10018994m.g	scaffold_3:37979247-37984418	8.67786	6.12197	-0.503342
Ciclev10024361m.g	scaffold_3:25649685-25653541	23.4378	16.5352	-0.503302
Ciclev10023109m.g	scaffold_3:10151278-10156572	20.8331	14.6983	-0.503228
Ciclev10015493m.g	scaffold_2:22962785-22966571	19.7021	13.9005	-0.503211
Ciclev10033813m.g	scaffold_4:25250024-25253269	7.47605	5.27482	-0.503154
Ciclev10032760m.g	scaffold_4:1149635-1152481	47.148	33.2706	-0.502946
Ciclev10008774m.g	scaffold_1:23407138-23409883	55.6796	39.2937	-0.50285
Ciclev10014198m.g	scaffold_2:31740430-31747095	36.2805	25.6098	-0.502501
Ciclev10017059m.g	scaffold_2:11065281-11068645	119.001	84.0164	-0.502235
Ciclev10022756m.g	scaffold_3:41909163-41910689	245.19	173.177	-0.501652
Ciclev10030492m.g	scaffold_4:20185185-20200225	25.6972	18.1502	-0.501627
Ciclev10009268m.g	scaffold_1:7008022-7011892	13.4278	9.48827	-0.501004
Ciclev10009207m.g	scaffold_1:14838663-14840710	109.164	77.1462	-0.500835
Ciclev10026403m.g	scaffold_7:6607425-6609875	70.7785	50.0221	-0.500746
Ciclev10020053m.g	scaffold_3:50133836-50139049	111.786	79.0052	-0.500718
Ciclev10031934m.g	scaffold_4:25260540-25262610	3.10939	2.19774	-0.500612
Ciclev10007325m.g,Ciclev10010443m.g	scaffold_1:11524304-11543392	5.46122	3.86084	-0.500307
Ciclev10005415m.g	scaffold_9:12757099-12763590	12.7368	9.00501	-0.500202
Ciclev10007308m.g	scaffold_1:25792926-25798214	17.5422	12.4082	-0.499539
Ciclev10001904m.g	scaffold_5:41005053-41009439	46.0106	32.5518	-0.499231

Ciclev10004945m.g	scaffold_9:24656960-24660764	13.1189	9.28165	-0.499192
Ciclev10033179m.g	scaffold_4:9245806-9247248	9.08089	6.42624	-0.49886
Ciclev10015960m.g	scaffold_2:28014473-28018607	15.8705	11.2326	-0.498654
Ciclev10029235m.g	scaffold_8:21817948-21821252	56.8508	40.2453	-0.498359
Ciclev10002692m.g	scaffold_5:36986112-36987382	358.594	253.88	-0.498205
Ciclev10030036m.g	scaffold_8:24007860-24043482	1.00586	0.712327	-0.497816
Ciclev10030439m.g	scaffold_8:4004366-4005195	1402.48	993.236	-0.49777
Ciclev10029324m.g	scaffold_8:5471135-5474488	91.0272	64.4693	-0.497686
Ciclev10029808m.g	scaffold_8:6686601-6688894	24.8408	17.5952	-0.497531
Ciclev10009322m.g	scaffold_1:4316928-4319059	53.5411	37.9329	-0.497197
Ciclev10006352m.g	scaffold_9:30456143-30470622	52.4672	37.1728	-0.497167
Ciclev10012189m.g	scaffold_6:25295298-25299298	22.1632	15.7026	-0.497162
-	scaffold_5:25221155-25545745	22.6989	16.0825	-0.497127
Ciclev10008752m.g	scaffold_1:22390817-22394165	16.2581	11.5198	-0.497042
Ciclev10022039m.g,Ciclev10023537m.g,Ciclev10024619m.g	scaffold_3:5038746-5181239	15.0843	10.6909	-0.496665
Ciclev10022980m.g	scaffold_3:560834-562540	34.6669	24.5738	-0.49644
Ciclev10013052m.g	scaffold_6:17729553-17730363	1.99696	1.41573	-0.496258
Ciclev10016456m.g	scaffold_2:14226911-14228104	80.299	56.9434	-0.495853
Ciclev10005664m.g	scaffold_9:4126568-4131986	46.1201	32.7078	-0.495758
Ciclev10022881m.g	scaffold_3:8327048-8328901	120.32	85.3382	-0.495613
Ciclev10028960m.g	scaffold_8:18732670-18734959	10.292	7.3005	-0.49545
Ciclev10011806m.g	scaffold_6:16496807-16498396	1.16921	0.829394	-0.495405
Ciclev10029291m.g	scaffold_8:24431063-24433341	294.545	209.009	-0.494924
Ciclev10000328m.g	scaffold_5:16635631-16640424	33.5461	23.8052	-0.494867
Ciclev10021650m.g	scaffold_3:48018377-48020545	5.50956	3.91069	-0.494512
Ciclev10011410m.g	scaffold_6:24157114-24161063	3.47645	2.46785	-0.494359
Ciclev10004985m.g	scaffold_9:1676852-1678628	4.29514	3.04937	-0.494197
Ciclev10024719m.g	scaffold_7:52700-63560	7.63342	5.42126	-0.493703
Ciclev10014853m.g	scaffold_2:31734948-31738843	9.73068	6.9108	-0.493688
Ciclev10008115m.g	scaffold_1:6628387-6634237	69.7666	49.5605	-0.493347
Ciclev10028789m.g	scaffold_8:1670116-1675908	56.5727	40.1889	-0.493308
Ciclev10020187m.g	scaffold_3:49818205-49820365	13.1816	9.3651	-0.49316
Ciclev10010264m.g	scaffold_1:2638872-2646073	10.2896	7.31102	-0.493047
Ciclev10033011m.g	scaffold_4:12717450-12718931	55.6426	39.5359	-0.493029
Ciclev10017707m.g	scaffold_2:11381916-11385787	409.183	290.738	-0.493028
Ciclev10032804m.g	scaffold_4:6460637-6463843	9.31721	6.62019	-0.493025
Ciclev10009503m.g	scaffold_1:12972468-12977393	236.289	167.932	-0.492677
Ciclev10011540m.g	scaffold_6:18299496-18301431	77.4155	55.0197	-0.492674
Ciclev10011452m.g	scaffold_6:13765655-13771161	9.21989	6.55284	-0.492631
Ciclev10030897m.g	scaffold_4:23387547-23393103	4.14818	2.94825	-0.49262
Ciclev10025370m.g	scaffold_7:3722810-3724786	38.2473	27.1847	-0.492564
Ciclev10029427m.g	scaffold_8:23911932-23914475	31.6509	22.4962	-0.492564
Ciclev10030552m.g	scaffold_4:19111713-19126397	60.1026	42.7196	-0.492529
Ciclev10028220m.g	scaffold_8:23006226-23008505	9.98347	7.0966	-0.492414
Ciclev10003557m.g	scaffold_5:24450098-24450524	0.954775	0.678736	-0.49231
Ciclev10011170m.g	scaffold_6:22940928-22945030	16.7416	11.9015	-0.492299
Ciclev10001310m.g	scaffold_5:37275152-37277867	7.8834	5.6057	-0.491925
-	scaffold_5:5600812-5601221	18.0308	12.8221	-0.491835
Ciclev10022809m.g	scaffold_3:3623693-3625333	474.068	337.163	-0.491645
Ciclev10000592m.g	scaffold_5:37876853-37884032	32.2958	22.9693	-0.491639
Ciclev10016100m.g	scaffold_2:30036935-30039324	25.3638	18.0446	-0.4912
Ciclev10008119m.g	scaffold_1:22881966-22884843	52.0407	37.0235	-0.491197
Ciclev10000067m.g	scaffold_5:37280602-37288224	5.81489	4.1372	-0.491097
-	scaffold_4:24775239-24776629	137.066	97.5254	-0.491024

-	scaffold_4:11523554-11728349	44.0593	31.3552	-0.490745
Ciclev10009636m.g	scaffold_1:16696978-16699623	265.426	188.922	-0.490519
Ciclev10014795m.g	scaffold_2:6870821-6877750	13.9464	9.92688	-0.490479
Ciclev10018601m.g	scaffold_3:8291226-8297326	1.78959	1.27418	-0.490061
Ciclev10006441m.g	scaffold_9:29882137-29883799	0.703523	0.500973	-0.489865
Ciclev10005564m.g	scaffold_9:29749062-29751434	132.782	94.5619	-0.489731
Ciclev10002627m.g	scaffold_5:36646218-36647375	24.5052	17.4579	-0.489211
Ciclev10003233m.g	scaffold_5:36407963-36409504	3.64077	2.59388	-0.489135
Ciclev10003293m.g	scaffold_5:7237869-7240251	1.46434	1.0433	-0.48909
Ciclev10020592m.g	scaffold_3:8816783-8821895	31.0375	22.1207	-0.488613
Ciclev10000188m.g	scaffold_5:42710680-42715502	17.8564	12.7305	-0.488152
Ciclev10030399m.g,Ciclev10030432m.g	scaffold_8:22012172-22018223	12.6936	9.05012	-0.488089
Ciclev10009471m.g,Ciclev10009961m.g	scaffold_1:15134042-15140387	116.211	82.8605	-0.487992
Ciclev10032205m.g	scaffold_4:24077584-24079950	12.4593	8.88513	-0.487754
Ciclev10007290m.g	scaffold_1:12622639-12634394	16.438	11.7253	-0.487409
Ciclev10015471m.g	scaffold_2:15059631-15063672	0.495846	0.353763	-0.487109
Ciclev10025391m.g	scaffold_7:7202245-7222786	8.88712	6.34066	-0.487083
Ciclev10013334m.g	scaffold_6:23657255-23657810	0.981563	0.700346	-0.487014
Ciclev10018618m.g	scaffold_3:1110090-1115498	9.43845	6.73438	-0.487005
Ciclev10017164m.g	scaffold_2:35112849-35114799	70.1281	50.049	-0.48665
Ciclev10031039m.g	scaffold_4:25506445-25511133	423.918	302.57	-0.486517
Ciclev10025965m.g	scaffold_7:5893553-5897759	57.2481	40.869	-0.486222
Ciclev10000394m.g	scaffold_5:31411457-31441415	5.32209	3.79972	-0.486099
Ciclev10017117m.g	scaffold_2:27599380-27600941	91.7715	65.5273	-0.48595
Ciclev10021225m.g	scaffold_3:45845448-45847783	13.9446	9.95717	-0.485897
Ciclev10019453m.g	scaffold_3:43966964-43972591	8.26994	5.90545	-0.485831
Ciclev10004410m.g	scaffold_9:3641279-3644796	12.477	8.91048	-0.485694
Ciclev10016916m.g	scaffold_2:9964786-9966216	34.1018	24.3545	-0.48566
Ciclev10019479m.g	scaffold_3:7414968-7418001	19.7767	14.1247	-0.485588
Ciclev10031703m.g	scaffold_4:16817583-16984703	45.7721	32.6909	-0.485581
Ciclev10016177m.g	scaffold_2:10124051-10125415	117.963	84.2591	-0.485436
Ciclev10021159m.g	scaffold_3:48103776-48107542	50.8461	36.3204	-0.48536
Ciclev10000365m.g	scaffold_5:31863795-31870938	18.0921	12.9273	-0.48494
-	scaffold_1:13120198-13120554	22.32	15.9536	-0.484456
Ciclev10014126m.g	scaffold_2:34559760-34586231	125.712	89.8599	-0.484368
Ciclev10000447m.g	scaffold_5:15564479-15571647	2.07459	1.48296	-0.484352
Ciclev10021850m.g	scaffold_3:37893390-37900992	14.758	10.5498	-0.484287
Ciclev10026553m.g	scaffold_7:6134144-6135296	49.4493	35.351	-0.484199
Ciclev10009940m.g	scaffold_1:21078113-21080074	273.777	195.77	-0.483844
Ciclev10033290m.g	scaffold_4:2774149-2775819	37.7344	26.9853	-0.483708
Ciclev10029657m.g	scaffold_8:24294600-24295449	1287.26	920.579	-0.48369
Ciclev10033682m.g	scaffold_4:2941167-2941449	38.4292	27.4827	-0.483682
Ciclev10028435m.g	scaffold_8:3023831-3028487	65.208	46.6355	-0.483619
Ciclev10026355m.g	scaffold_7:1268457-1271678	16.7695	11.9939	-0.483537
-	scaffold_1:20664826-20823973	9.64913	6.90215	-0.483354
Ciclev10027179m.g	scaffold_7:7438223-7441084	0.703153	0.503195	-0.48272
Ciclev10031457m.g	scaffold_4:3866063-3871971	22.146	15.849	-0.48265
Ciclev10014662m.g	scaffold_2:26509928-26515893	11.0469	7.90774	-0.482302
Ciclev10021685m.g	scaffold_3:46750835-46754323	27.4403	19.6487	-0.48186
Ciclev10033397m.g,Ciclev10033444m.g	scaffold_4:2428038-2429956	208.329	149.205	-0.481571
-	scaffold_3:28683923-28684878	10.1025	7.23563	-0.481522
Ciclev10009198m.g	scaffold_1:18719131-18732457	427.563	306.257	-0.481395
-	scaffold_4:3779660-3781446	6.2616	4.4851	-0.481392

Ciclev10016510m.g	scaffold_2:34688498-34691194	26.0375	18.6517	-0.481287
Ciclev10001170m.g	scaffold_5:41242935-41246768	5.23892	3.75306	-0.481201
Ciclev10027901m.g	scaffold_8:21887371-21893128	8.62211	6.17704	-0.481125
Ciclev10012142m.g	scaffold_6:20221207-20222224	2.96197	2.12248	-0.480803
Ciclev10018166m.g	scaffold_2:25703102-25703750	0.440246	0.315522	-0.48057
Ciclev10011262m.g	scaffold_6:7403799-7411226	11.1828	8.01559	-0.480407
Ciclev10004853m.g	scaffold_9:1705205-1710948	34.4936	24.7326	-0.479911
Ciclev10026281m.g	scaffold_7:16273806-16281383	15.5256	11.1325	-0.479868
Ciclev10012875m.g	scaffold_6:18188138-18189060	282.284	202.44	-0.479656
Ciclev10014138m.g	scaffold_2:33763000-33767074	9.09925	6.52626	-0.479491
Ciclev10012844m.g	scaffold_6:12354544-12355995	44.4679	31.894	-0.479478
Ciclev10017084m.g	scaffold_2:30404216-30406520	16.5258	11.8555	-0.479165
Ciclev10028263m.g	scaffold_8:6068943-6075685	13.5331	9.71294	-0.478514
Ciclev10026891m.g	scaffold_7:6121610-6123232	30.2062	21.6862	-0.478066
Ciclev10016549m.g	scaffold_2:29909579-29913341	13.922	9.9954	-0.478028
Ciclev10033118m.g	scaffold_4:3767410-3770036	16.5328	11.8706	-0.477935
Ciclev10014979m.g	scaffold_2:23326559-23330494	10.3766	7.45145	-0.477747
Ciclev10019972m.g	scaffold_3:31304518-31310511	74.2475	53.3335	-0.477301
Ciclev10022935m.g	scaffold_3:32071629-32076377	45.7005	32.8284	-0.477267
Ciclev10025909m.g	scaffold_7:15315229-15321153	20.8266	14.9617	-0.477154
Ciclev10033270m.g	scaffold_4:21151909-21153516	381.994	274.479	-0.476852
Ciclev10015537m.g	scaffold_2:11916419-11918383	4.38634	3.15231	-0.476609
Ciclev10032240m.g	scaffold_4:7746079-7748525	53.1282	38.1833	-0.476539
Ciclev10012021m.g	scaffold_6:9455950-9460264	366.697	263.56	-0.476457
Ciclev10022755m.g	scaffold_3:37381091-37420501	41.4758	29.8106	-0.476447
Ciclev10006077m.g	scaffold_9:6380789-6383011	73.8343	53.0717	-0.476348
Ciclev10010502m.g	scaffold_1:28052329-28053460	0.207887	0.14943	-0.476325
Ciclev10007489m.g	scaffold_1:26459700-26471409	7.31069	5.25531	-0.476232
Ciclev10006188m.g	scaffold_9:26211987-26236468	0.532736	0.383094	-0.475723
Ciclev10001015m.g	scaffold_5:40713634-40719229	6.07508	4.36875	-0.475683
Ciclev10001322m.g	scaffold_5:41035559-41037824	44.6607	32.121	-0.475488
Ciclev10002708m.g	scaffold_5:42259212-42259753	2.49131	1.79195	-0.475379
Ciclev10003605m.g	scaffold_5:40676194-40679319	6.1937	4.45587	-0.475095
Ciclev10028349m.g	scaffold_8:1823963-1885505	71.6388	51.5411	-0.475019
Ciclev10021272m.g	scaffold_3:2459619-2463757	1.52499	1.09722	-0.474944
Ciclev10026169m.g	scaffold_7:20833367-20836799	132.284	95.1807	-0.474895
Ciclev10003279m.g,Ciclev10003329m.g,Ciclev10004001m.g	scaffold_5:22611509-22623399	118.106	84.988	-0.474748
Ciclev10017383m.g	scaffold_2:9511324-9518280	58.971	42.4403	-0.47457
Ciclev10000491m.g,Ciclev10003317m.g	scaffold_5:953144-1184934	42.3292	30.4674	-0.474384
Ciclev10029279m.g	scaffold_8:2332171-2334039	26.8611	19.3354	-0.474271
-	scaffold_3:7848593-7849342	14.266	10.2696	-0.474195
Ciclev10019852m.g	scaffold_3:49090638-49095466	16.8815	12.1565	-0.473717
Ciclev10013916m.g	scaffold_6:18437196-18440541	17.7537	12.7857	-0.473592
Ciclev10029560m.g	scaffold_8:10362236-10362644	286.953	206.687	-0.473367
Ciclev10009952m.g	scaffold_1:24468087-24470417	239.931	172.837	-0.473208
Ciclev10026219m.g	scaffold_7:18975821-18981398	2.67026	1.92387	-0.47297
Ciclev10015114m.g	scaffold_2:20382304-20385049	1.30263	0.938779	-0.472574
Ciclev10023110m.g	scaffold_3:2874466-2875676	74.6064	53.7805	-0.472217
Ciclev10005087m.g	scaffold_9:25662048-25666791	30.607	22.0653	-0.472081
-	scaffold_3:25395464-25396505	4.22344	3.04497	-0.471994
Ciclev10007835m.g	scaffold_1:18042409-18051299	12.5672	9.06189	-0.471774
Ciclev10020793m.g	scaffold_3:42287919-42291209	83.8797	60.497	-0.471458
Ciclev10023497m.g	scaffold_3:6286480-6290433	4.06103	2.92901	-0.471432

Ciclev10022169m.g	scaffold_3:44959565-44964478	229.452	165.515	-0.471227
Ciclev10008979m.g	scaffold_1:24354876-24358736	43.0012	31.0242	-0.470982
-	scaffold_6:24214578-24215337	14.6165	10.5474	-0.470703
Ciclev10000150m.g	scaffold_5:32939570-32945214	9.53031	6.87721	-0.470699
Ciclev10001162m.g	scaffold_5:19180691-19182592	0.216159	0.155992	-0.470621
Ciclev10014589m.g	scaffold_2:35579402-35586357	63.5395	45.8536	-0.470617
Ciclev10008288m.g	scaffold_1:26350558-26354625	8.91081	6.4317	-0.470356
Ciclev10004162m.g	scaffold_9:12494754-12502431	11.5124	8.31035	-0.470207
Ciclev10003715m.g	scaffold_5:42683915-42686550	0.594527	0.429194	-0.470113
Ciclev10029669m.g	scaffold_8:5123744-5125235	2.09109	1.51011	-0.469604
Ciclev10019192m.g	scaffold_3:31325834-31339383	13.8446	9.99948	-0.469403
Ciclev10029601m.g,Ciclev10030428m.g	scaffold_8:6735307-6738952	105.091	75.9106	-0.469272
Ciclev10023234m.g	scaffold_3:24814787-24816626	49.148	35.5015	-0.469252
Ciclev10021521m.g	scaffold_3:606718-609324	22.4403	16.2162	-0.468662
-	scaffold_5:33677967-33678239	33.8727	24.48	-0.468519
Ciclev10028715m.g	scaffold_8:17468873-17471267	3.51536	2.54065	-0.468474
Ciclev10008255m.g	scaffold_1:22758711-22761740	598.034	432.225	-0.468445
Ciclev10015120m.g	scaffold_2:29847209-29851852	27.7514	20.0575	-0.468421
Ciclev10030945m.g	scaffold_4:16324347-16328584	2.05933	1.48876	-0.468061
Ciclev10013072m.g	scaffold_6:1715430-1718390	157.958	114.214	-0.467803
Ciclev10011973m.g	scaffold_6:13666332-13669690	69.8676	50.5193	-0.467788
Ciclev10004221m.g	scaffold_9:15981286-15989471	8.26035	5.97319	-0.467703
Ciclev10018427m.g	scaffold_3:3092289-3130540	13.3969	9.68754	-0.467696
Ciclev10011599m.g	scaffold_6:8958457-8986420	21.7678	15.7419	-0.467584
Ciclev10031076m.g	scaffold_4:22364030-22367299	85.7778	62.0356	-0.467508
Ciclev10024455m.g	scaffold_3:43801280-43801864	2399.86	1735.98	-0.467199
Ciclev10027827m.g	scaffold_8:242707-246711	3.57219	2.58424	-0.467067
Ciclev10019081m.g	scaffold_3:3299618-3311799	6.41883	4.64381	-0.467001
Ciclev10033005m.g	scaffold_4:2678602-2679354	2.67582	1.93605	-0.46686
Ciclev10011741m.g	scaffold_6:7785327-7796808	75.0978	54.3626	-0.466157
Ciclev10000571m.g	scaffold_5:12159642-12164071	2.09748	1.51837	-0.466128
Ciclev10032875m.g	scaffold_4:25167373-25170468	39.8687	28.8623	-0.466073
Ciclev10032657m.g	scaffold_4:12191110-12193951	6.15708	4.45773	-0.465937
Ciclev10021331m.g	scaffold_3:3805878-3809717	120.363	87.1427	-0.465934
Ciclev10002649m.g	scaffold_5:42352016-42353093	6.06124	4.38854	-0.465871
Ciclev10014511m.g	scaffold_2:5615634-5621177	10.3703	7.50852	-0.465853
Ciclev10028843m.g	scaffold_8:4000406-4003956	9.7305	7.0464	-0.465628
Ciclev10028164m.g	scaffold_8:20499093-20504304	5.85386	4.23996	-0.465337
Ciclev10031073m.g	scaffold_4:23404931-23425248	117.956	85.439	-0.465285
Ciclev10030109m.g	scaffold_8:19305845-19307173	7.34154	5.31799	-0.465201
Ciclev10009976m.g	scaffold_1:22837499-22839817	177.713	128.755	-0.464922
Ciclev10004801m.g	scaffold_9:83308-87262	11.0739	8.02341	-0.464877
Ciclev10009394m.g	scaffold_1:25765316-25766713	64.4655	46.709	-0.464827
Ciclev10016440m.g	scaffold_2:36145309-36147106	14.8787	10.7831	-0.464478
Ciclev10026875m.g	scaffold_7:4203875-4205830	17.5434	12.7149	-0.464406
Ciclev10009769m.g	scaffold_1:24271752-24272644	2409.23	1746.17	-0.46438
Ciclev10013378m.g	scaffold_6:16005354-16006587	1.3117	0.950753	-0.464295
Ciclev10020312m.g	scaffold_3:30393564-30398973	79.5145	57.6359	-0.46425
Ciclev10010560m.g	scaffold_1:9592680-9593277	0.825293	0.598288	-0.464066
Ciclev10026007m.g,Ciclev10026012m.g	scaffold_7:14481483-14501384	197.32	143.051	-0.464
Ciclev10025352m.g	scaffold_7:1820469-1822053	1.65208	1.19814	-0.463485
Ciclev10025026m.g	scaffold_7:5773825-5778887	50.3966	36.559	-0.463101
Ciclev10028683m.g	scaffold_8:24442335-24444606	63.4256	46.0117	-0.463064
Ciclev10008229m.g	scaffold_1:4467756-4471325	4.02061	2.91748	-0.462693

Ciclev10009275m.g	scaffold_1:3253345-3254158	0.575471	0.417621	-0.462546
Ciclev10002721m.g	scaffold_5:39674392-39677320	46.7065	33.8952	-0.462541
Ciclev10032024m.g	scaffold_4:18432285-18436809	21.8098	15.8303	-0.462285
Ciclev10020835m.g	scaffold_3:5470586-5474647	37.7193	27.3792	-0.462223
Ciclev10022479m.g	scaffold_3:8477602-8488997	60.6894	44.06	-0.461975
Ciclev10032968m.g	scaffold_4:19567950-19569850	417.582	303.169	-0.46194
Ciclev10030562m.g	scaffold_4:17536454-17545827	0.688213	0.499763	-0.461611
Ciclev10032757m.g	scaffold_4:11964849-11971089	31.9484	23.2018	-0.461509
Ciclev10002404m.g	scaffold_5:38189668-38190638	1115.01	809.774	-0.461466
Ciclev10010072m.g	scaffold_1:17551961-17554435	46.9629	34.1122	-0.461234
Ciclev10012434m.g	scaffold_6:23342708-23346658	140.436	102.008	-0.461233
Ciclev10025822m.g	scaffold_7:456002-459404	126.249	91.7196	-0.460965
Ciclev10018805m.g	scaffold_3:50768515-50772455	4.28634	3.11406	-0.460953
Ciclev10008488m.g	scaffold_1:18113948-18117209	12.8842	9.36104	-0.460861
Ciclev10000182m.g	scaffold_5:39477781-39484776	20.7149	15.0513	-0.460779
Ciclev10000352m.g	scaffold_5:36588686-36592786	5.8449	4.24753	-0.460556
Ciclev10011268m.g	scaffold_6:15070589-15076199	1.15138	0.836833	-0.460353
Ciclev10012909m.g	scaffold_6:10318646-10320818	119.616	86.9476	-0.460191
Ciclev10005388m.g	scaffold_9:2443652-2447321	54.9935	39.976	-0.460127
Ciclev10001965m.g	scaffold_5:40393801-40400154	29.3049	21.3034	-0.460063
Ciclev10015185m.g	scaffold_2:5968187-5973023	1.10316	0.801969	-0.46002
Ciclev10026894m.g	scaffold_7:4565985-4569023	0.877532	0.637959	-0.459989
Ciclev10020648m.g	scaffold_3:39692470-39696185	27.7804	20.1972	-0.459913
Ciclev10028424m.g	scaffold_8:713660-715466	15.751	11.4532	-0.459695
Ciclev10004255m.g	scaffold_9:14687872-14698268	25.6758	18.6705	-0.459651
Ciclev10020502m.g	scaffold_3:44279498-44283658	68.3308	49.6897	-0.45959
Ciclev10021662m.g	scaffold_3:50281511-50287997	93.6351	68.1169	-0.459037
Ciclev10010804m.g	scaffold_1:28909536-28910017	2.003	1.45757	-0.458596
Ciclev10032010m.g	scaffold_4:22284473-22287046	22.5583	16.4173	-0.458437
Ciclev10010140m.g	scaffold_1:17999645-18001343	41.8615	30.4703	-0.458219
Ciclev10011798m.g	scaffold_6:23484090-23485669	6.20571	4.51717	-0.458176
Ciclev10008390m.g	scaffold_1:16098038-16100795	12.3232	8.97149	-0.457961
Ciclev10019555m.g	scaffold_3:10382954-10391443	10.294	7.49447	-0.45791
Ciclev10028985m.g	scaffold_8:24795575-24806623	9.50221	6.91806	-0.457895
Ciclev10010322m.g	scaffold_1:23390415-23394090	1.27036	0.924905	-0.457862
Ciclev10001913m.g	scaffold_5:25788253-25792720	5.57143	4.05675	-0.457726
Ciclev10029409m.g	scaffold_8:23220443-23224438	79.3409	57.7844	-0.457383
Ciclev10018137m.g	scaffold_2:36034475-36035132	0.923966	0.673001	-0.457232
Ciclev10009932m.g	scaffold_1:2457208-2459620	2.67636	1.94942	-0.457225
Ciclev10017375m.g	scaffold_2:30499724-30504591	3.74938	2.73108	-0.457181
Ciclev10024562m.g	scaffold_3:904382-905099	116.658	84.9791	-0.457108
Ciclev10023703m.g	scaffold_3:27274446-27274841	46.8856	34.1659	-0.456588
Ciclev10006361m.g	scaffold_9:4448166-4450777	89.3249	65.0932	-0.456556
Ciclev10004554m.g	scaffold_9:4283234-4286516	9.95551	7.25484	-0.456551
Ciclev10017234m.g	scaffold_2:35785115-35786782	15.0984	11.0047	-0.456273
Ciclev10022158m.g	scaffold_3:49734900-49738269	63.3255	46.1671	-0.45592
Ciclev10005028m.g	scaffold_9:16745130-16748803	21.478	15.6587	-0.455897
Ciclev10011250m.g	scaffold_6:19658507-19663778	19.1268	13.947	-0.45564
Ciclev10005783m.g	scaffold_9:27938112-27941628	54.5155	39.7556	-0.455509
Ciclev10004151m.g	scaffold_9:253574-260425	28.9843	21.1384	-0.455407
Ciclev10021110m.g	scaffold_3:50968439-50971551	55.0087	40.1197	-0.455349
Ciclev10010082m.g	scaffold_1:27500633-27503373	13.9922	10.206	-0.4552
Ciclev10013394m.g	scaffold_6:23797513-23800185	16.0888	11.736	-0.455113
Ciclev10008545m.g	scaffold_1:20890730-20895676	9.66388	7.04992	-0.454996
Ciclev10025879m.g	scaffold_7:16587337-16590811	1.91555	1.39764	-0.454768
Ciclev10013538m.g	scaffold_6:72418-72841	5.96715	4.35447	-0.454544

Ciclev10022488m.g	scaffold_3:46814908-46817550	185.004	135.007	-0.454519
Ciclev10011518m.g	scaffold_6:17665503-17668726	37.0905	27.0698	-0.454369
Ciclev10023098m.g	scaffold_3:89733-90245	0.499621	0.364678	-0.45421
Ciclev10015206m.g	scaffold_2:10231920-10233354	1.26231	0.921555	-0.453929
Ciclev10032884m.g	scaffold_4:15547574-15549138	94.9856	69.3499	-0.453815
Ciclev10015942m.g	scaffold_2:29567198-29570566	20.8137	15.2005	-0.453416
Ciclev10011409m.g	scaffold_6:16154921-16158807	49.3769	36.0664	-0.453179
Ciclev10007654m.g	scaffold_1:22498750-22504815	19.5013	14.2553	-0.452069
Ciclev10027024m.g	scaffold_7:5319072-5319801	0.245274	0.179297	-0.452045
Ciclev10003161m.g	scaffold_5:41932894-41936414	19.6612	14.3727	-0.452017
Ciclev10008249m.g	scaffold_1:275262-280936	56.4133	41.2407	-0.451966
Ciclev10010112m.g	scaffold_1:22907148-22910024	98.9084	72.3127	-0.451845
Ciclev10031670m.g	scaffold_4:22155109-22160150	27.1801	19.8721	-0.451807
Ciclev10027814m.g	scaffold_8:21662822-21667935	0.55161	0.40333	-0.451688
Ciclev10030412m.g	scaffold_8:23674664-23679316	80.0551	58.5374	-0.451635
Ciclev10032882m.g	scaffold_4:25065382-25066587	184.788	135.171	-0.45109
Ciclev10025598m.g	scaffold_7:203838-207450	13.7403	10.0539	-0.45065
Ciclev10032631m.g	scaffold_4:1504684-1505639	0.235814	0.172572	-0.450452
Ciclev10004641m.g	scaffold_9:1027059-1029387	6.99836	5.12175	-0.45038
Ciclev10011612m.g	scaffold_6:24344668-24347411	18.3502	13.4319	-0.450131
Ciclev10033703m.g	scaffold_4:7180976-7183760	7.12631	5.21756	-0.44978
Ciclev10023015m.g	scaffold_3:39993699-39996603	142.891	104.65	-0.449347
Ciclev10006522m.g	scaffold_9:2968846-2971662	0.766953	0.561727	-0.449269
Ciclev10012697m.g	scaffold_6:17491240-17494671	49.5073	36.2659	-0.449028
Ciclev10016896m.g	scaffold_2:553134-556043	29.0048	21.2502	-0.448811
Ciclev10014266m.g	scaffold_2:31431080-31437122	4.33602	3.17702	-0.448698
Ciclev10024851m.g	scaffold_7:230316-235974	3.50176	2.56626	-0.448412
-	scaffold_6:17852079-17855366	191.753	140.542	-0.448252
Ciclev10028267m.g	scaffold_8:1216554-1220646	3.96281	2.90457	-0.4482
Ciclev10010209m.g	scaffold_1:18454167-18459508	0.222743	0.163315	-0.447723
Ciclev10003312m.g	scaffold_5:40463922-40469054	1.55067	1.13701	-0.447643
Ciclev10019155m.g	scaffold_3:37591679-37597425	6.00854	4.40631	-0.447444
Ciclev10002346m.g	scaffold_5:20384811-20387339	121.541	89.1312	-0.447441
Ciclev10016979m.g	scaffold_2:21948037-21948818	3.05952	2.24392	-0.447279
Ciclev10013153m.g	scaffold_6:11407483-11409763	31.5879	23.169	-0.447176
Ciclev10021053m.g	scaffold_3:1149032-1152392	17.7763	13.04	-0.447017
Ciclev10015195m.g	scaffold_2:25205133-25208439	17.4255	12.7827	-0.447003
Ciclev10028291m.g	scaffold_8:1888733-1892204	77.6449	56.9628	-0.446872
Ciclev10017168m.g	scaffold_2:8752627-8755153	20.6503	15.1502	-0.446822
Ciclev10018671m.g	scaffold_3:47892245-47897508	8.47304	6.21709	-0.44664
Ciclev10009557m.g	scaffold_1:3133202-3136004	34.0201	24.9625	-0.446623
Ciclev10015974m.g	scaffold_2:34208421-34209850	47.774	35.0546	-0.446623
Ciclev10028860m.g	scaffold_8:6474194-6475882	39.3204	28.858	-0.446305
Ciclev10016512m.g	scaffold_2:30779574-30780824	15.1519	11.1231	-0.445939
Ciclev10026679m.g	scaffold_7:8894133-8894829	350.724	257.498	-0.445778
Ciclev10016280m.g	scaffold_2:23891813-23893095	4975.62	3653.24	-0.4457
Ciclev10014176m.g	scaffold_2:22175369-22181367	9.93273	7.29293	-0.445692
Ciclev10000841m.g	scaffold_5:40534832-40539098	8.83846	6.48949	-0.445689
Ciclev10002059m.g	scaffold_5:38743042-38746428	10.4103	7.6442	-0.445569
Ciclev10029414m.g	scaffold_8:23977411-23978585	69.7643	51.2306	-0.445483
Ciclev10014119m.g	scaffold_2:27509978-27514016	0.988442	0.725909	-0.445368
Ciclev10014776m.g	scaffold_2:27701171-27707547	7.50289	5.51019	-0.445345
Ciclev10005417m.g	scaffold_9:3202313-3203413	6.90358	5.07022	-0.445297
Ciclev10006197m.g	scaffold_9:2362828-2365557	81.2959	59.7176	-0.445024
Ciclev10022042m.g	scaffold_3:5743178-5747184	22.7299	16.6983	-0.444892

Ciclev10015873m.g,Ciclev10018072m.g	scaffold_2:238743-250675	408.56	300.243	-0.444418
Ciclev10000430m.g	scaffold_5:17812396-17817017	22.9238	16.8473	-0.444332
Ciclev10005830m.g	scaffold_9:29931102-29937516	38.3813	28.2112	-0.444134
Ciclev10023213m.g	scaffold_3:1162274-1163781	8.036	5.90781	-0.443855
Ciclev10028320m.g,Ciclev10028369m.g	scaffold_8:6076635-6128024	65.7272	48.3205	-0.443855
Ciclev10022021m.g,Ciclev10024565m.g	scaffold_3:46519382-46595230	10.7954	7.93722	-0.443711
Ciclev10016989m.g	scaffold_2:28832510-28833017	0.522073	0.383894	-0.443543
Ciclev10017160m.g	scaffold_2:2674699-2675291	0.34865	0.256376	-0.443518
Ciclev10004753m.g	scaffold_9:3688972-3692790	107.992	79.4191	-0.443367
Ciclev10018028m.g	scaffold_2:8310445-8320631	26.8954	19.7804	-0.443289
Ciclev10019638m.g	scaffold_3:2933120-2937460	2677.59	1969.34	-0.443225
Ciclev10006003m.g	scaffold_9:28061220-28063450	128.587	94.5744	-0.443224
Ciclev10026132m.g	scaffold_7:4525787-4529207	11.3058	8.31624	-0.443057
Ciclev10018614m.g	scaffold_3:11078797-11089090	12.4277	9.14294	-0.442835
Ciclev10010079m.g	scaffold_1:23554124-23557055	201.636	148.438	-0.441888
Ciclev10013125m.g	scaffold_6:17007165-17008747	327.617	241.185	-0.44187
Ciclev10017247m.g	scaffold_2:6932306-6933093	1312.15	966.01	-0.441822
Ciclev10005033m.g	scaffold_9:10316806-10320241	15.7218	11.575	-0.441759
Ciclev10022799m.g	scaffold_3:50798604-50800785	166.759	122.782	-0.441669
Ciclev10013219m.g	scaffold_6:2028504-2032253	34.8688	25.6762	-0.441509
Ciclev10005403m.g	scaffold_9:28757273-28759075	18.3792	13.5338	-0.441506
Ciclev10022844m.g	scaffold_3:15799879-15804088	96.8175	71.2943	-0.441481
Ciclev10009786m.g,Ciclev10010066m.g	scaffold_1:14053547-14069954	8.63471	6.35978	-0.441171
Ciclev10022140m.g	scaffold_3:7506702-7511038	52.4688	38.6468	-0.441111
Ciclev10013088m.g	scaffold_6:22724681-22726973	15.2912	11.2632	-0.441086
Ciclev10026274m.g	scaffold_7:3866303-3868206	255.764	188.392	-0.441076
Ciclev10002263m.g	scaffold_5:9979514-9982669	20.5744	15.1549	-0.441068
Ciclev10007969m.g	scaffold_1:18896163-18901623	24.1065	17.7618	-0.440641
Ciclev10019319m.g	scaffold_3:2352856-2355637	11.9622	8.81504	-0.440446
Ciclev10012797m.g	scaffold_6:20270430-20274129	52.4384	38.6447	-0.440354
Ciclev10010509m.g	scaffold_1:6400376-6401210	0.604584	0.445654	-0.440018
Ciclev10016275m.g	scaffold_2:12043968-12046230	5.08457	3.7481	-0.439968
Ciclev10000965m.g,Ciclev10004061m.g	scaffold_5:14944935-14955856	20.0245	14.7619	-0.439888
Ciclev10022664m.g	scaffold_3:12789640-12791258	0.892195	0.657735	-0.439852
Ciclev10031443m.g	scaffold_4:642156-644823	8517.16	6279.1	-0.439813
Ciclev10032669m.g	scaffold_4:21733663-21735787	297.587	219.41	-0.439681
Ciclev10021568m.g	scaffold_3:18925718-18932522	13.059	9.62858	-0.439653
Ciclev10013120m.g	scaffold_6:11292994-11295908	54.3328	40.0612	-0.439618
Ciclev10023008m.g	scaffold_3:17535477-17536101	45.8674	33.8195	-0.439614
Ciclev10005569m.g	scaffold_9:30683786-30686283	6.67433	4.92195	-0.439393
Ciclev10026581m.g	scaffold_7:221279-224106	10.7496	7.92739	-0.439368
Ciclev10026277m.g,Ciclev10026284m.g	scaffold_7:2721714-2817730	17.276	12.7415	-0.439234
Ciclev10008850m.g	scaffold_1:2605752-2609177	18.1813	13.4095	-0.439195
Ciclev10026071m.g	scaffold_7:3472565-3476911	20.7473	15.3032	-0.43909
Ciclev10028052m.g	scaffold_8:1715521-1718932	69.3117	51.1321	-0.438871
Ciclev10017371m.g	scaffold_2:7712288-7715284	46.7767	34.5119	-0.438697
Ciclev10019589m.g	scaffold_3:41791735-41800410	12.7541	9.41075	-0.43858
Ciclev10032268m.g	scaffold_4:2430023-2433146	76.2832	56.2893	-0.438505
Ciclev10011533m.g	scaffold_6:21864841-21866491	28.979	21.3845	-0.438448
Ciclev10027560m.g	scaffold_7:3729181-3730825	4.74364	3.50067	-0.43836

Ciclev10025207m.g	scaffold_7:5515192-5521809	14.9922	11.0643	-0.4383
Ciclev10022734m.g	scaffold_3:50512923-50513791	3.27583	2.41762	-0.438273
Ciclev10032932m.g	scaffold_4:17382496-17384442	256.587	189.373	-0.438219
Ciclev10022601m.g	scaffold_3:7012273-7013949	157.371	116.191	-0.437676
Ciclev10018615m.g	scaffold_3:3251736-3258182	29.707	21.9372	-0.437423
Ciclev10018682m.g	scaffold_3:39411615-39416261	2.12354	1.56829	-0.437273
Ciclev10016632m.g	scaffold_2:475373-478428	20.8124	15.3766	-0.436704
Ciclev10012002m.g	scaffold_6:2571940-2573984	22.8884	16.9137	-0.436427
Ciclev10022245m.g	scaffold_3:2845656-2848886	7.82031	5.77954	-0.436269
Ciclev10002007m.g	scaffold_5:33391428-33396907	140.722	104.017	-0.436023
Ciclev10015412m.g	scaffold_2:33289743-33294803	7.19225	5.31636	-0.436003
Ciclev10012640m.g	scaffold_6:21145702-21148279	172.909	127.821	-0.435887
Ciclev10012884m.g	scaffold_6:24274030-24276658	103.312	76.3742	-0.435854
Ciclev10032076m.g	scaffold_4:2160182-2163554	12.592	9.30918	-0.435777
Ciclev10008816m.g	scaffold_1:1599577-1603604	10.9234	8.07663	-0.435599
Ciclev10017888m.g	scaffold_2:32407518-32411163	14.4871	10.7132	-0.435378
Ciclev10021492m.g	scaffold_3:7248626-7253007	26.728	19.7664	-0.435305
Ciclev10007516m.g	scaffold_1:25441527-25445434	9.23574	6.83093	-0.435146
Ciclev10026261m.g	scaffold_7:1794791-1797256	61.2114	45.2837	-0.434808
Ciclev10032470m.g	scaffold_4:16756747-16759899	22.8758	16.9261	-0.434569
Ciclev10033094m.g	scaffold_4:12900776-12906838	35.9626	26.6113	-0.434455
Ciclev10013953m.g	scaffold_10:324807-325242	25.7757	19.0764	-0.434218
Ciclev10022968m.g	scaffold_3:45812588-45815067	96.471	71.4057	-0.434056
Ciclev10014328m.g	scaffold_2:30012163-30018752	56.0619	41.4962	-0.434043
Ciclev10026291m.g	scaffold_7:12332712-12337401	153.561	113.678	-0.433851
Ciclev10011659m.g	scaffold_6:10648787-10650598	66.4893	49.2248	-0.433735
Ciclev10012606m.g	scaffold_6:17330417-17333481	23.8466	17.6557	-0.433649
Ciclev10021938m.g	scaffold_3:36924550-37054826	41.2388	30.5337	-0.433602
Ciclev10014053m.g	scaffold_2:32096233-32105112	8.89313	6.58816	-0.432816
Ciclev10007839m.g	scaffold_1:2751178-2761967	7.87931	5.83712	-0.432812
Ciclev10017134m.g	scaffold_2:14640952-14643739	16.7711	12.4252	-0.432715
Ciclev10005911m.g	scaffold_9:713852-716708	42.8633	31.7568	-0.432678
Ciclev10007096m.g	scaffold_9:23890154-23890616	22.5731	16.7255	-0.432554
Ciclev10031327m.g	scaffold_4:24801973-24805847	16.4896	12.2195	-0.432374
Ciclev10029098m.g	scaffold_8:13553544-13555568	16.5177	12.2404	-0.432356
Ciclev10008463m.g	scaffold_1:18634787-18636361	14.6159	10.8312	-0.432347
Ciclev10022953m.g	scaffold_3:45792121-45793164	36.3968	26.9732	-0.432282
Ciclev10027615m.g	scaffold_7:1726199-1728709	18.3469	13.6009	-0.431833
Ciclev10017187m.g	scaffold_2:11041472-11042677	0.884349	0.655704	-0.431571
Ciclev10025347m.g	scaffold_7:2721714-2817730	8.69978	6.45143	-0.431361
Ciclev10011732m.g	scaffold_6:18385421-18387448	15.7535	11.6834	-0.431211
Ciclev10015243m.g	scaffold_2:11654897-11658922	10.4445	7.74769	-0.430907
-	scaffold_5:655534-811195	74.674	55.396	-0.430826
Ciclev10005185m.g	scaffold_9:762823-765605	11.1437	8.2682	-0.43059
Ciclev10001083m.g,Ciclev10001615m.g,Ciclev10002197m.g	scaffold_5:33079707-33163140	42.6562	31.6492	-0.430586
Ciclev10016099m.g	scaffold_2:354827-358235	258.696	191.974	-0.430345
Ciclev10033431m.g	scaffold_4:24481764-24485301	13.0111	9.65593	-0.430259
Ciclev10020097m.g,Ciclev10021527m.g	scaffold_3:8383302-8410026	16.5118	12.2544	-0.430203
Ciclev10005290m.g	scaffold_9:3759681-3762674	9.61111	7.13301	-0.430192
Ciclev10029821m.g	scaffold_8:23745327-23746729	0.169992	0.126164	-0.430168
Ciclev10005172m.g	scaffold_9:4339722-4341951	63.1517	46.8722	-0.430088
Ciclev10028136m.g	scaffold_8:22403821-22408174	57.1568	42.4255	-0.429992
Ciclev10011164m.g	scaffold_6:14689943-14700102	6.00394	4.45715	-0.429789

Ciclev10031671m.g	scaffold_4:2447997-2450912	15.5712	11.563	-0.429367
Ciclev10012263m.g	scaffold_6:11826471-11830875	49.4519	36.7275	-0.429164
Ciclev10025050m.g	scaffold_7:12956764-12962208	1.70059	1.26309	-0.429083
Ciclev10001775m.g	scaffold_5:32315343-32320120	20.4182	15.1653	-0.429082
Ciclev10029354m.g	scaffold_8:18064760-18071003	65.5635	48.7008	-0.428948
Ciclev10018542m.g	scaffold_3:49869032-49877681	7.90321	5.87094	-0.428847
Ciclev10008501m.g	scaffold_1:2005590-2009207	7.30159	5.42416	-0.42881
Ciclev10000997m.g	scaffold_5:39589972-39592011	9.41773	6.99743	-0.428553
Ciclev10021301m.g	scaffold_3:7157694-7158781	4.29641	3.19331	-0.428079
Ciclev10013225m.g	scaffold_6:5899521-5904942	16.0459	11.9264	-0.428045
Ciclev10003471m.g	scaffold_5:6255728-6257807	0.476541	0.354229	-0.427916
Ciclev10032190m.g	scaffold_4:17152925-17155643	129.596	96.3509	-0.427653
Ciclev10029599m.g	scaffold_8:3399074-3434977	9.63715	7.16528	-0.427583
-	scaffold_5:19865022-20070734	11.0549	8.22	-0.427471
Ciclev10009222m.g	scaffold_1:8046180-8049306	29.9028	22.2347	-0.427463
Ciclev10024644m.g	scaffold_3:43795212-43800518	6.07058	4.51408	-0.427402
Ciclev10031029m.g	scaffold_4:1189661-1193876	38.7106	28.7874	-0.427291
Ciclev10015772m.g	scaffold_2:7672177-7675700	12.5735	9.35085	-0.427218
Ciclev10032131m.g	scaffold_4:3667579-3673924	26.4485	19.6699	-0.427194
Ciclev10004324m.g	scaffold_9:30572544-30580705	11.8036	8.77941	-0.427031
Ciclev10030232m.g	scaffold_8:22174720-22176483	26.388	19.6273	-0.42702
Ciclev10016045m.g	scaffold_2:4854924-4857997	14.9001	11.0857	-0.426618
Ciclev10022921m.g	scaffold_3:30462421-30463517	1.14571	0.852498	-0.426475
Ciclev10009645m.g	scaffold_1:27540014-27543337	16.9649	12.6235	-0.426449
Ciclev10015574m.g	scaffold_2:10024285-10026423	12.1214	9.02	-0.426356
Ciclev10024826m.g	scaffold_7:3376810-3385942	6.21465	4.62478	-0.42629
Ciclev10029628m.g	scaffold_8:20547179-20549390	26.4618	19.6984	-0.425829
Ciclev10003193m.g	scaffold_5:27263530-27268194	24.4583	18.2101	-0.425581
-	scaffold_2:27792008-27792321	114.819	85.4872	-0.425579
Ciclev10010087m.g	scaffold_1:9666063-9668524	116.611	86.8251	-0.425515
Ciclev10013077m.g	scaffold_6:23639246-23639974	16.5495	12.3236	-0.425366
Ciclev10002818m.g	scaffold_5:36919656-36923034	65.1051	48.4829	-0.425295
Ciclev10021851m.g	scaffold_3:6902897-6907412	18.9648	14.1241	-0.42516
Ciclev10031672m.g	scaffold_4:18782082-18787545	94.8128	70.6166	-0.425074
Ciclev10017086m.g	scaffold_2:27987812-27989086	150.651	112.208	-0.425033
Ciclev10028853m.g	scaffold_8:21141421-21142499	19.1402	14.2562	-0.425012
Ciclev10011447m.g,Ciclev10013213m.g	scaffold_6:21822312-21835198	66.6829	49.6678	-0.425006
Ciclev10005675m.g	scaffold_9:8376736-8414244	51.6325	38.4609	-0.424888
-	scaffold_3:5705428-5705626	170.239	126.819	-0.424788
Ciclev10008038m.g	scaffold_1:21476759-21547573	2.17777	1.62254	-0.424605
Ciclev10031260m.g	scaffold_4:20619170-20622578	18.4422	13.7409	-0.424535
-	scaffold_2:33865827-33868113	16.8966	12.5903	-0.424423
Ciclev10008068m.g	scaffold_1:15829555-15834871	29.5179	21.9962	-0.424337
Ciclev10012630m.g	scaffold_6:15462977-15466398	39.9382	29.7641	-0.424197
Ciclev10005148m.g	scaffold_9:25493377-25500370	69.153	51.5376	-0.424166
Ciclev10001327m.g	scaffold_5:37494368-37498693	242.596	180.804	-0.42413
-	scaffold_1:19721667-19778009	83.1216	61.951	-0.424095
Ciclev10004399m.g	scaffold_9:15125423-15131152	45.687	34.0508	-0.424095
Ciclev10017717m.g	scaffold_2:31340742-31343850	49.6725	37.023	-0.424028
Ciclev10027603m.g	scaffold_7:4154746-4155496	0.514118	0.383222	-0.423918
Ciclev10033263m.g	scaffold_4:21417274-21419616	27.5804	20.5587	-0.423896
Ciclev10012239m.g	scaffold_6:25121918-25125183	112.639	83.9781	-0.423623
Ciclev10003848m.g	scaffold_5:655534-811195	35.2183	26.2573	-0.42361
Ciclev10021060m.g	scaffold_3:793626-796219	522.948	389.931	-0.42345
Ciclev10002874m.g	scaffold_5:36748680-36750848	30.9726	23.0956	-0.423377

Ciclev10012169m.g	scaffold_6:23713310-23717489	31.2959	23.3443	-0.422902
Ciclev10012277m.g,Ciclev10013094m.g	scaffold_6:13420635-13426593	55.3128	41.2655	-0.422679
Ciclev10013118m.g	scaffold_6:2541593-2542087	0.437512	0.326442	-0.422497
Ciclev10018953m.g	scaffold_3:1324137-1329256	7.84358	5.8525	-0.422459
Ciclev10016735m.g	scaffold_2:35841858-35844177	23.7954	17.7568	-0.422315
Ciclev10022723m.g	scaffold_3:37094784-37098257	172.582	128.818	-0.421946
Ciclev10018924m.g	scaffold_3:43309850-43315758	16.4112	12.2498	-0.42193
Ciclev10018802m.g	scaffold_3:6449634-6452593	1.95687	1.46084	-0.421748
Ciclev10014641m.g,Ciclev10017491m.g,Ciclev10017770m.g,Ciclev10018029m.g	scaffold_2:30736179-30773700	25.5924	19.109	-0.421462
-	scaffold_5:23653254-23653902	10.0243	7.48503	-0.421419
Ciclev10006042m.g	scaffold_9:28734051-28735038	0.400749	0.299278	-0.42121
Ciclev10032615m.g	scaffold_4:16355234-16356284	31.5699	23.5815	-0.420896
Ciclev10011876m.g	scaffold_6:23456733-23461541	6.8846	5.14275	-0.420832
Ciclev10033420m.g	scaffold_4:21054392-21068514	103.986	77.6938	-0.420512
Ciclev10031787m.g	scaffold_4:16570651-16572491	3.44883	2.57718	-0.420314
Ciclev10033863m.g	scaffold_4:23612265-23614044	3.62808	2.71149	-0.420119
Ciclev10010006m.g	scaffold_1:24741259-24748937	7.97843	5.96302	-0.420064
Ciclev10029733m.g	scaffold_8:2020264-2030440	0.834714	0.623935	-0.419887
Ciclev10021848m.g	scaffold_3:11677912-11679468	5.01007	3.74572	-0.419589
-	scaffold_2:35021915-35022142	202.326	151.286	-0.419406
Ciclev10020551m.g	scaffold_3:24489554-24491893	8.32207	6.22273	-0.419395
Ciclev10026305m.g	scaffold_7:4436885-4440321	57.3867	42.9111	-0.419368
-	scaffold_3:28684992-28685362	29.1496	21.7978	-0.419295
Ciclev10024228m.g	scaffold_3:6801186-6804260	5.50712	4.1188	-0.419074
Ciclev10014065m.g	scaffold_2:28932640-28942243	17.0113	12.7236	-0.418982
Ciclev10002002m.g	scaffold_5:37270210-37274678	22.7835	17.0423	-0.418872
Ciclev10002321m.g	scaffold_5:35738600-35741550	42.3917	31.7106	-0.418817
Ciclev10011433m.g	scaffold_6:21229810-21233537	17.5566	13.1372	-0.418354
Ciclev10012375m.g,Ciclev10012379m.g,Ciclev10012633m.g	scaffold_6:10374765-10420958	51.1976	38.3158	-0.41814
Ciclev10016815m.g	scaffold_2:10210804-10213389	25.8219	19.3255	-0.418091
-	scaffold_6:18435607-18435912	125.035	93.5816	-0.418034
Ciclev10030630m.g	scaffold_4:19440591-19448615	37.2299	27.8718	-0.417655
Ciclev10008271m.g	scaffold_1:13873758-13877202	7.94248	5.94613	-0.41764
Ciclev10007283m.g	scaffold_1:26692877-26704500	19.7682	14.8015	-0.417439
Ciclev10014084m.g	scaffold_2:31552710-31567003	2.89115	2.16513	-0.41719
Ciclev10001724m.g	scaffold_5:30800766-30803631	11.5574	8.65793	-0.416723
Ciclev10001000m.g	scaffold_5:13183492-13185654	53.1951	39.8506	-0.416692
-	scaffold_9:24263537-24264307	14.08	10.5484	-0.416612
Ciclev10013348m.g	scaffold_6:15484462-15485386	1.84655	1.38344	-0.416572
-	scaffold_2:10130233-10131029	220.441	165.156	-0.416565
Ciclev10007216m.g	scaffold_9:3237867-3238611	22.4244	16.802	-0.416441
Ciclev10012109m.g	scaffold_6:14689943-14700102	67.1556	50.3178	-0.41644
Ciclev10033720m.g	scaffold_4:3049683-3051468	0.419807	0.314575	-0.416321
Ciclev10028056m.g	scaffold_8:21443116-21447306	72.0597	54.0038	-0.416131
Ciclev10017879m.g	scaffold_2:34130088-34130475	68.0896	51.0413	-0.415768
Ciclev10029609m.g	scaffold_8:165914-167748	20.0984	15.0662	-0.415763
Ciclev10020528m.g	scaffold_3:44525265-44527119	0.717844	0.538131	-0.415715
Ciclev10031854m.g	scaffold_4:3477654-3481892	7.35498	5.5145	-0.41549
Ciclev10003302m.g	scaffold_5:11911913-11913031	7.51933	5.63865	-0.415254
Ciclev10021860m.g	scaffold_3:48603076-48605404	37.6875	28.2655	-0.415043
Ciclev10012166m.g	scaffold_6:14281196-14286862	486.033	364.541	-0.414976

Ciclev10002493m.g	scaffold_5:42879562-42882316	386.425	289.864	-0.414811
Ciclev10016098m.g	scaffold_2:20031904-20042011	306.464	229.889	-0.414783
Ciclev10019980m.g	scaffold_3:5615539-5620092	7.60529	5.70647	-0.414405
Ciclev10013918m.g	scaffold_6:19126603-19128455	25.9134	19.4444	-0.414345
Ciclev10017445m.g	scaffold_2:19536650-19539688	7.47348	5.60968	-0.413861
Ciclev10025698m.g	scaffold_7:5434910-5437325	14.2625	10.7058	-0.413835
Ciclev10020939m.g	scaffold_3:48704415-48708817	14.6614	11.0053	-0.413825
Ciclev10006198m.g	scaffold_9:29545502-29547180	143.003	107.344	-0.413806
Ciclev10016420m.g	scaffold_2:35855337-35858338	95.405	71.618	-0.413743
Ciclev10006356m.g	scaffold_9:27790597-27828145	109.654	82.3197	-0.413651
Ciclev10033230m.g	scaffold_4:12945610-12947370	60.3915	45.339	-0.413594
Ciclev10011702m.g	scaffold_6:20117538-20123487	40.6123	30.4899	-0.413586
Ciclev10022763m.g	scaffold_3:20529900-20531846	53.6056	40.251	-0.413357
Ciclev10007983m.g	scaffold_1:22175413-22384190	34.3899	25.8244	-0.413248
Ciclev10000295m.g	scaffold_5:41694481-41700100	28.0878	21.0953	-0.413022
Ciclev10018294m.g	scaffold_2:7347490-7348024	0.41834	0.314199	-0.412998
Ciclev10019263m.g	scaffold_3:43076522-43084747	8.07995	6.06875	-0.412946
Ciclev10028899m.g	scaffold_8:24282482-24287657	7.94118	5.96541	-0.412732
Ciclev10032603m.g	scaffold_4:2146460-2149740	16.775	12.6019	-0.412672
Ciclev10016842m.g	scaffold_2:4481222-4482431	94.3905	70.9286	-0.412275
Ciclev10020398m.g	scaffold_3:17774630-17782729	5.50003	4.13338	-0.412117
Ciclev10005883m.g	scaffold_9:15390157-15393735	2.24215	1.68516	-0.411998
Ciclev10011860m.g	scaffold_6:23515997-23518706	1.90107	1.4289	-0.411907
Ciclev10000457m.g	scaffold_5:39227920-39237745	28.8536	21.6907	-0.411677
Ciclev10022803m.g	scaffold_3:35932197-35935023	29.668	22.303	-0.411672
Ciclev10024054m.g	scaffold_3:806801-808331	261.786	196.819	-0.411522
Ciclev10033098m.g	scaffold_4:25113577-25114198	0.571454	0.429757	-0.411114
Ciclev10000347m.g	scaffold_5:36036989-36048596	22.0801	16.6099	-0.410699
Ciclev10028636m.g	scaffold_8:24638859-24641066	1.23752	0.930971	-0.410638
Ciclev10020839m.g	scaffold_3:7934793-7939524	13.2596	9.9783	-0.410175
Ciclev10010940m.g	scaffold_6:21290860-21300106	81.3512	61.226	-0.410018
Ciclev10001894m.g	scaffold_5:42985724-42990116	131.266	98.8145	-0.409702
Ciclev10001733m.g	scaffold_5:34659784-34662439	2.01133	1.51453	-0.40928
Ciclev10007115m.g	scaffold_9:30012884-30017717	10.2272	7.70125	-0.409248
Ciclev10010765m.g	scaffold_1:344011-344854	0.339486	0.255708	-0.408854
Ciclev10029987m.g	scaffold_8:7909414-7965005	230.863	173.913	-0.408674
Ciclev10012619m.g	scaffold_6:23881967-23889247	53.5158	40.3147	-0.408661
Ciclev10026748m.g	scaffold_7:2041330-2042230	3.27142	2.46461	-0.408555
Ciclev10028129m.g	scaffold_8:365123-369233	1.59337	1.2005	-0.408452
Ciclev10010409m.g	scaffold_1:22175413-22384190	5.64149	4.25066	-0.408389
Ciclev10001348m.g	scaffold_5:42390615-42395712	104.41	78.6707	-0.408363
Ciclev10002566m.g	scaffold_5:33964667-33966527	80.4994	60.6635	-0.408148
Ciclev10011861m.g	scaffold_6:7976197-7982000	163.669	123.339	-0.408147
Ciclev10006657m.g	scaffold_9:2191084-2191819	0.702805	0.529677	-0.40801
Ciclev10010368m.g	scaffold_1:19688665-19692235	2.19969	1.65784	-0.407998
Ciclev10005675m.g,Ciclev10006635m.g	scaffold_9:8376736-8414244	100.559	75.7953	-0.407862
Ciclev10020946m.g	scaffold_3:5284984-5289515	41.7964	31.5129	-0.407438
Ciclev10010421m.g	scaffold_1:1488818-1496062	28.811	21.725	-0.407263
-	scaffold_5:10407338-10407754	44.7599	33.7548	-0.407113
Ciclev10001758m.g	scaffold_5:36616632-36620799	14.7687	11.1383	-0.407018
Ciclev10016944m.g	scaffold_2:6532934-6535930	297.451	224.353	-0.406882
Ciclev10010999m.g	scaffold_6:19900234-19904192	15.7477	11.8798	-0.406634
Ciclev10009588m.g	scaffold_1:26600302-26601217	72.0406	54.3485	-0.406569
Ciclev10013056m.g	scaffold_6:23063663-23065846	15.5694	11.7473	-0.406383
Ciclev10033435m.g	scaffold_4:1171785-1173887	1.50837	1.13826	-0.406158

Ciclev10026431m.g	scaffold_7:14268410-14275412	15.5284	11.7196	-0.405987
Ciclev10028057m.g	scaffold_8:25022488-25027972	75.0488	56.6465	-0.405843
Ciclev10025029m.g	scaffold_7:3058159-3067713	25.6471	19.36	-0.40572
Ciclev10007996m.g	scaffold_1:18203019-18206175	14.8226	11.19	-0.405587
Ciclev10029320m.g	scaffold_8:16433704-16436177	13.7225	10.3598	-0.405546
Ciclev10002622m.g	scaffold_5:34245417-34248236	38.3062	28.922	-0.405413
Ciclev10027766m.g	scaffold_8:24757172-24765538	10.0302	7.57343	-0.405337
Ciclev10005298m.g	scaffold_9:2046955-2050677	50.4872	38.1228	-0.405263
-	scaffold_4:9880494-10159290	4.76063	3.59483	-0.405231
Ciclev10025619m.g	scaffold_7:5935715-5939865	18.5529	14.0121	-0.404977
Ciclev10015887m.g	scaffold_2:10424778-10472483	6.66829	5.03634	-0.404942
Ciclev10022720m.g	scaffold_3:43447941-43449920	173.334	130.914	-0.404939
Ciclev10024760m.g	scaffold_7:3850620-3863167	31.8846	24.0843	-0.40477
Ciclev10002392m.g	scaffold_5:30544606-30548868	35.5298	26.8387	-0.404714
Ciclev10026842m.g	scaffold_7:15252031-15255159	47.1933	35.6551	-0.404473
Ciclev10000387m.g	scaffold_5:30567036-30570413	5.01689	3.79033	-0.404468
Ciclev10008813m.g	scaffold_1:734372-736031	159.54	120.549	-0.404297
Ciclev10016199m.g	scaffold_2:36093568-36099013	27.1306	20.5046	-0.403975
Ciclev10002119m.g	scaffold_5:42348092-42350470	344.407	260.311	-0.403874
Ciclev10020382m.g	scaffold_3:45731066-45735238	14.5458	10.9957	-0.403661
Ciclev10007849m.g	scaffold_1:27167270-27171073	2.92535	2.21153	-0.403569
Ciclev10007658m.g	scaffold_1:598418-604135	14.1866	10.7258	-0.403446
Ciclev10012882m.g	scaffold_6:22327156-22330006	15.3779	11.6266	-0.403436
Ciclev10002528m.g	scaffold_5:33921983-33926087	65.3479	49.4147	-0.403202
Ciclev10030973m.g	scaffold_4:21612918-21619733	10.9227	8.26025	-0.403074
Ciclev10015801m.g	scaffold_2:29506074-29516708	42.9041	32.4541	-0.402715
Ciclev10006724m.g	scaffold_9:2330431-2331669	48.6254	36.7824	-0.402695
Ciclev10000798m.g	scaffold_5:41672134-41678524	35.0061	26.4833	-0.40252
Ciclev10016857m.g	scaffold_2:35480391-35483365	21.5975	16.3405	-0.402416
Ciclev10008867m.g	scaffold_1:3609275-3615258	56.6104	42.8363	-0.402234
Ciclev10006213m.g	scaffold_9:21177410-21180021	4.60036	3.48111	-0.4022
-	scaffold_9:7456968-7457526	8.25316	6.24544	-0.402144
Ciclev10018473m.g	scaffold_3:3953526-3962077	4.99788	3.78313	-0.401735
Ciclev10015244m.g	scaffold_2:6826731-6832001	3.24114	2.45373	-0.401528
Ciclev10002814m.g	scaffold_5:17836086-17837904	28.9551	21.9224	-0.401409
Ciclev10003203m.g	scaffold_5:20777927-20778458	1.05481	0.798637	-0.401377
Ciclev10022148m.g	scaffold_3:11418161-11423840	7.97172	6.03661	-0.401152
Ciclev10033044m.g	scaffold_4:5040356-5042760	60.6311	45.9607	-0.399657
Ciclev10027676m.g	scaffold_8:20376641-20384538	4.98432	3.77843	-0.399609
Ciclev10005984m.g	scaffold_9:19202943-19204062	21.0091	15.93	-0.399266
Ciclev10029224m.g	scaffold_8:1336906-1339200	154.327	117.026	-0.399165
Ciclev10033608m.g	scaffold_4:5155988-5160744	1.17758	0.893156	-0.398841
Ciclev10023690m.g	scaffold_3:32543167-32579333	7.21887	5.47594	-0.398667
Ciclev10025829m.g	scaffold_7:3879583-3881117	5.6272	4.26888	-0.398558
Ciclev10008418m.g	scaffold_1:4404554-4409802	51.2007	38.8418	-0.398552
Ciclev10011946m.g	scaffold_6:20385171-20388381	9.60535	7.28859	-0.398199
Ciclev10015675m.g	scaffold_2:24569574-24572242	5.78784	4.39267	-0.397928
Ciclev10028796m.g	scaffold_8:6897535-6901468	14.3503	10.8916	-0.397869
Ciclev10027449m.g	scaffold_7:11052336-11053224	0.26908	0.204234	-0.39781
Ciclev10031509m.g	scaffold_4:22007450-22012267	10.9003	8.27412	-0.397686
Ciclev10015605m.g	scaffold_2:11910554-11915055	11.1007	8.42738	-0.397499
Ciclev10031709m.g	scaffold_4:22865936-22868335	37.1696	28.2206	-0.397371
Ciclev10007788m.g	scaffold_1:14383196-14392881	25.4354	19.3132	-0.397252
Ciclev10002551m.g,Ciclev10004073m.g	scaffold_5:42109315-42112542	35.2869	26.7953	-0.39715
Ciclev10023439m.g	scaffold_3:32652095-32652674	0.496506	0.377102	-0.396856

Ciclev10013603m.g	scaffold_6:7496063-7497811	5.87247	4.46088	-0.396638
Ciclev10032666m.g	scaffold_4:608211-615318	31.8365	24.1902	-0.396255
-	scaffold_2:32888260-32889405	14.7023	11.1726	-0.396084
Ciclev10007575m.g	scaffold_1:5388449-5396902	12.5494	9.53724	-0.395969
Ciclev10001481m.g	scaffold_5:36028654-36031153	2.4972	1.89805	-0.395795
Ciclev10018134m.g	scaffold_2:34811292-34814689	30.5078	23.1894	-0.395709
Ciclev10018990m.g	scaffold_3:3145372-3149274	12.1429	9.2313	-0.395506
Ciclev10008106m.g	scaffold_1:21866376-21869490	17.9826	13.6714	-0.395441
Ciclev10021969m.g	scaffold_3:894964-897547	80.3722	61.1051	-0.395405
Ciclev10012754m.g	scaffold_6:9789369-9794598	1.59016	1.21019	-0.393942
Ciclev10030628m.g	scaffold_4:14065434-14074014	23.9362	18.2177	-0.39385
Ciclev10027010m.g,Ciclev10027530m.g	scaffold_7:17948447-18051030	11.1715	8.50259	-0.393849
Ciclev10008435m.g	scaffold_1:5194084-5198860	55.3322	42.1152	-0.393777
Ciclev10016184m.g	scaffold_2:27779246-27785501	41.7334	31.7656	-0.393735
Ciclev10032971m.g	scaffold_4:295199-296138	17.1542	13.0571	-0.393723
Ciclev10029093m.g	scaffold_8:22156305-22159272	59.5287	45.3116	-0.393705
Ciclev10017067m.g	scaffold_2:32997288-32998282	7.81582	5.94951	-0.393627
-	scaffold_4:25607556-25609538	2.70477	2.05932	-0.393336
Ciclev10014888m.g	scaffold_2:27474499-27476966	108.848	82.9089	-0.392715
Ciclev10031526m.g	scaffold_4:21548652-21551668	2.59894	1.9797	-0.392643
Ciclev10033183m.g	scaffold_4:729271-729866	2.28905	1.74407	-0.392287
Ciclev10023073m.g	scaffold_3:4944105-4947636	3.15455	2.40436	-0.391786
Ciclev10002826m.g	scaffold_5:21362593-21366180	49.3865	37.6435	-0.391714
Ciclev10020811m.g	scaffold_3:45816253-45820244	1.77344	1.35177	-0.391698
Ciclev10018478m.g	scaffold_3:44400736-44409548	3.74771	2.85719	-0.391411
Ciclev10013168m.g	scaffold_6:10427838-10429894	170.882	130.327	-0.390869
Ciclev10005541m.g	scaffold_9:7200580-7203678	21.8382	16.6564	-0.390777
Ciclev10021907m.g	scaffold_3:45885974-45888854	86.5936	66.0489	-0.390727
Ciclev10006661m.g	scaffold_9:11181404-11191293	17.6292	13.4484	-0.390528
Ciclev10005071m.g	scaffold_9:16838229-16846426	21.7736	16.6103	-0.390507
Ciclev10007425m.g	scaffold_1:26615111-26624993	12.4744	9.51681	-0.390416
Ciclev10031968m.g	scaffold_4:15477116-15478843	95.5686	72.9321	-0.389983
Ciclev10021578m.g	scaffold_3:30465122-30468499	3.38852	2.58595	-0.389959
Ciclev10005820m.g	scaffold_9:5394791-5395790	121.246	92.5298	-0.389944
Ciclev10008193m.g	scaffold_1:4365380-4369697	19.1184	14.5905	-0.389934
Ciclev10024446m.g	scaffold_3:4800388-4817559	4.6705	3.56439	-0.389922
Ciclev10028588m.g	scaffold_8:11549015-11550586	7.81081	5.96297	-0.389441
Ciclev10000792m.g	scaffold_5:16368132-16371600	1.82921	1.39647	-0.389437
Ciclev10025993m.g	scaffold_7:1545356-1547511	3.79291	2.89578	-0.389354
Ciclev10031885m.g	scaffold_4:24881230-24882906	11.1783	8.53503	-0.38923
Ciclev10014172m.g	scaffold_2:26719185-26723009	30.7383	23.4706	-0.389184
Ciclev10011271m.g	scaffold_6:23966038-23969583	15.1339	11.5582	-0.388866
Ciclev10026064m.g	scaffold_7:3967395-3969310	8.94708	6.83349	-0.388795
Ciclev10024183m.g	scaffold_3:15662562-15663341	0.899184	0.686817	-0.388689
Ciclev10004402m.g	scaffold_9:27714382-27727799	13.9637	10.6677	-0.388438
Ciclev10021379m.g	scaffold_3:48689811-48692761	19.863	15.176	-0.388287
Ciclev10008908m.g	scaffold_1:2599956-2603377	80.5587	61.5544	-0.388178
Ciclev10001826m.g	scaffold_5:33854543-33858415	16.0911	12.2957	-0.38811
Ciclev10033753m.g	scaffold_4:20786529-20790368	8.29861	6.34144	-0.388059
Ciclev10003501m.g	scaffold_5:42649976-42652003	8.17373	6.24617	-0.388023
Ciclev10029230m.g	scaffold_8:6554901-6560226	27.4503	20.9769	-0.388022
-	scaffold_5:441330-538185	19.6989	15.0571	-0.387669
Ciclev10020165m.g	scaffold_3:46042915-46044739	4.26467	3.25988	-0.387615
Ciclev10016092m.g	scaffold_2:5135442-5140979	53.4178	40.8333	-0.387574
Ciclev10008633m.g	scaffold_1:8276124-8278135	73.8017	56.4205	-0.387434

-	scaffold_6:16920993-16921387	21.3874	16.3515	-0.387332
Ciclev10006839m.g	scaffold_9:8077796-8081563	7.67185	5.86547	-0.387327
Ciclev10019567m.g	scaffold_3:14897911-14903449	145.764	111.446	-0.387284
Ciclev10031507m.g	scaffold_4:23130215-23131775	6.19636	4.73776	-0.387215
Ciclev10026640m.g	scaffold_7:5492443-5493231	84.724	64.7916	-0.386964
Ciclev10009088m.g	scaffold_1:26970433-26975029	42.4159	32.4395	-0.386857
Ciclev10019681m.g	scaffold_3:9305488-9313725	104.423	79.8752	-0.386619
Ciclev10004272m.g,Ciclev10004467m.g	scaffold_9:29208177-29221979	39.7579	30.4135	-0.386528
Ciclev10021532m.g	scaffold_3:39620222-39625996	35.052	26.8304	-0.385633
Ciclev10010893m.g	scaffold_6:18657956-18672823	11.2711	8.62769	-0.385578
Ciclev10030970m.g	scaffold_4:20099491-20103015	25.6978	19.6713	-0.385547
Ciclev10010138m.g	scaffold_1:1399656-1400369	659.035	504.487	-0.385538
Ciclev10002624m.g	scaffold_5:24800760-24805736	65.5275	50.1649	-0.385424
Ciclev10005068m.g	scaffold_9:5605469-5610873	88.6914	67.9033	-0.385312
Ciclev10015752m.g	scaffold_2:35846187-35849559	25.6127	19.616	-0.384826
Ciclev10007301m.g	scaffold_1:27486048-27491908	35.2685	27.012	-0.384781
Ciclev10003191m.g	scaffold_5:33705152-33709918	3.2374	2.47971	-0.384661
Ciclev10011941m.g,Ciclev10013819m.g	scaffold_6:4067938-4074069	34.9842	26.8046	-0.384221
Ciclev10010021m.g	scaffold_1:4845256-4849815	27.0231	20.7052	-0.384199
Ciclev10032174m.g	scaffold_4:14967253-14973093	328.461	251.701	-0.384013
Ciclev10009934m.g	scaffold_1:21883405-21885741	37.3154	28.5964	-0.383938
Ciclev10005897m.g	scaffold_9:28054213-28056032	244.311	187.229	-0.383916
Ciclev10000407m.g	scaffold_5:40863026-40868561	38.7785	29.7198	-0.383835
Ciclev10020988m.g	scaffold_3:49103622-49109291	40.8667	31.3237	-0.383672
Ciclev10017434m.g	scaffold_2:28605787-28618242	23.6644	18.142	-0.383383
Ciclev10021974m.g	scaffold_3:48551947-48554426	197.812	151.653	-0.383357
Ciclev10029342m.g	scaffold_8:4475086-4477963	20.5687	15.7725	-0.383039
Ciclev10002908m.g	scaffold_5:34194833-34196805	31.6097	24.2416	-0.38288
Ciclev10028234m.g	scaffold_8:21342049-21348646	12.9831	9.9599	-0.382437
Ciclev10002025m.g	scaffold_5:871875-875378	55.3542	42.465	-0.382421
Ciclev10021940m.g	scaffold_3:48621899-48624947	28.1163	21.5704	-0.382352
Ciclev10027395m.g	scaffold_7:18351691-18352605	0.240731	0.184697	-0.382264
Ciclev10002047m.g	scaffold_5:40631967-40634597	39.5392	30.3365	-0.382231
Ciclev10031933m.g	scaffold_4:6368335-6373763	20.11	15.4303	-0.38214
Ciclev10003772m.g	scaffold_5:33319302-33321501	94.0695	72.182	-0.382089
Ciclev10014928m.g	scaffold_2:7863352-7869814	14.013	10.7527	-0.382065
Ciclev10003897m.g	scaffold_5:29169638-29171172	28.9714	22.2339	-0.381869
Ciclev10017351m.g	scaffold_2:30698900-30699397	6.60511	5.06918	-0.38183
Ciclev10019793m.g	scaffold_3:4354538-4356299	0.104126	0.0799213	-0.381678
Ciclev10014598m.g	scaffold_2:4864393-4884787	176.227	135.274	-0.381543
Ciclev10006475m.g	scaffold_9:29158847-29161171	0.349408	0.268234	-0.38142
Ciclev10003512m.g	scaffold_5:39207052-39212539	1.73235	1.32998	-0.381334
Ciclev10032623m.g	scaffold_4:12339807-12341113	66.5197	51.0695	-0.38132
Ciclev10016502m.g	scaffold_2:26026810-26029598	18.458	14.1736	-0.381047
Ciclev10019304m.g	scaffold_3:7744610-7750724	48.6999	37.3998	-0.380889
Ciclev10028878m.g	scaffold_8:24846865-24850089	35.9071	27.5787	-0.380713
Ciclev10025116m.g	scaffold_7:7258155-7260647	22.3215	17.1461	-0.380553
Ciclev10008863m.g	scaffold_1:4731245-4732596	3.10344	2.38421	-0.38036
Ciclev10024724m.g	scaffold_7:5185844-5198750	37.5937	28.8846	-0.380189
Ciclev10031117m.g	scaffold_4:18809376-18811980	2.12011	1.62897	-0.380178
Ciclev10001277m.g	scaffold_5:4607325-4613461	42.1581	32.3955	-0.380018
Ciclev10024281m.g	scaffold_3:44328078-44330075	0.0926591	0.0712072	-0.379908
Ciclev10029469m.g	scaffold_8:24107681-24109725	29.8581	22.948	-0.379754
Ciclev10022441m.g	scaffold_3:449406-451690	92.515	71.1331	-0.379167

Ciclev10021292m.g	scaffold_3:5351245-5355794	14.4746	11.1294	-0.37914
Ciclev10025448m.g	scaffold_7:6436784-6440864	4.99566	3.84166	-0.378943
Ciclev10000711m.g	scaffold_5:31411457-31441415	9.84973	7.5748	-0.378877
Ciclev10002980m.g	scaffold_5:17392923-17476446	0.468072	0.359988	-0.378782
Ciclev10019917m.g	scaffold_3:43719321-43724297	29.6955	22.8398	-0.378696
Ciclev10032432m.g	scaffold_4:23837174-23839644	71.0687	54.6669	-0.378546
Ciclev10008835m.g	scaffold_1:21452440-21455666	57.4945	44.2325	-0.378316
Ciclev10013090m.g	scaffold_6:14397046-14399928	71.663	55.1341	-0.378282
Ciclev10004428m.g	scaffold_9:387770-393501	16.0993	12.3867	-0.378201
Ciclev10019577m.g	scaffold_3:44437465-44442544	32.1932	24.7699	-0.378172
-	scaffold_8:17291958-17467945	31.5818	24.3024	-0.377995
Ciclev10027626m.g	scaffold_7:5170571-5173081	44.7505	34.4379	-0.377908
Ciclev10006067m.g	scaffold_9:29613406-29617590	40.7293	31.3452	-0.377825
Ciclev10033132m.g	scaffold_4:25480982-25481509	27.8287	21.4208	-0.377561
Ciclev10005825m.g	scaffold_9:1178026-1180584	46.0727	35.4665	-0.377457
Ciclev10015876m.g	scaffold_2:7621258-7625957	186.294	143.438	-0.377157
Ciclev10013900m.g	scaffold_6:2089779-2092517	23.4251	18.0366	-0.377133
Ciclev10015490m.g	scaffold_2:7662358-7668170	622.208	479.082	-0.377125
Ciclev10032956m.g	scaffold_4:24237867-24243284	373.005	287.216	-0.37706
-	scaffold_1:20142839-20143172	33.4298	25.7425	-0.376984
Ciclev10015451m.g	scaffold_2:33004824-33011298	34.9939	26.947	-0.376978
Ciclev10020095m.g,Ciclev10024547m.g	scaffold_3:37174506-37260267	12.5268	9.64637	-0.376959
Ciclev10002883m.g	scaffold_5:26949950-26952841	81.1994	62.5403	-0.376683
Ciclev10013142m.g	scaffold_6:22828055-22898575	916.322	705.81	-0.376575
Ciclev10008919m.g	scaffold_1:24273992-24277800	55.8185	42.9953	-0.376563
Ciclev10017235m.g	scaffold_2:22122561-22123287	0.91555	0.705261	-0.376481
Ciclev10033713m.g	scaffold_4:3104604-3105354	0.862779	0.664623	-0.376456
Ciclev10013632m.g	scaffold_6:7919985-7922537	18.0185	13.8803	-0.376439
-	scaffold_2:2181868-2184353	46.4625	35.7942	-0.376341
Ciclev10003615m.g	scaffold_5:36171244-36173637	77.3284	59.5786	-0.376203
Ciclev10015526m.g	scaffold_2:13564285-13570027	74.959	57.7665	-0.375868
Ciclev10001715m.g	scaffold_5:38566191-38571337	35.887	27.6563	-0.375857
Ciclev10014600m.g	scaffold_2:32873929-32878999	20.6717	15.9322	-0.375716
Ciclev10008134m.g	scaffold_1:21318783-21325312	24.78	19.1009	-0.375538
Ciclev10022556m.g	scaffold_3:2893723-2894888	49.288	37.9937	-0.375476
Ciclev10027872m.g	scaffold_8:24613596-24623540	49.3327	38.0373	-0.375129
Ciclev10004215m.g	scaffold_9:4724358-4730943	31.0573	23.9491	-0.37496
Ciclev10008345m.g	scaffold_1:21911566-21914523	40.7054	31.3891	-0.374957
Ciclev10028093m.g	scaffold_8:685244-692374	5.13396	3.95901	-0.374932
Ciclev10022714m.g	scaffold_3:48436922-48441004	192.44	148.432	-0.374611
-	scaffold_2:2181868-2184353	33.0032	25.462	-0.374263
Ciclev10020030m.g	scaffold_3:42025356-42028727	19.2751	14.8707	-0.374261
Ciclev10025132m.g	scaffold_7:2595399-2598112	1.64823	1.27165	-0.374208
Ciclev10018214m.g	scaffold_2:23269514-23272329	4.2703	3.29511	-0.374013
Ciclev10004767m.g	scaffold_9:29665573-29672166	11.4878	8.86448	-0.373991
Ciclev10008721m.g	scaffold_1:16451113-16456491	11.8067	9.11154	-0.373835
Ciclev10017285m.g	scaffold_2:8307579-8309929	683.647	527.661	-0.37364
Ciclev10000652m.g	scaffold_5:37086126-37091907	3.84395	2.96719	-0.373493
Ciclev10032846m.g	scaffold_4:18320631-18323702	71.5552	55.2355	-0.373459
Ciclev10002865m.g	scaffold_5:37721099-37724344	81.7991	63.1431	-0.373459
Ciclev10024800m.g	scaffold_7:856305-865378	7.50782	5.79557	-0.373443
Ciclev10021896m.g	scaffold_3:4260125-4262819	36.3873	28.0912	-0.373318
Ciclev10018077m.g	scaffold_2:25883452-25885407	4.3184	3.33383	-0.373316
-	scaffold_7:5697603-5698567	4.1933	3.23742	-0.373244
Ciclev10010136m.g	scaffold_1:22997199-22998134	16.408	12.6681	-0.373194

Ciclev10004041m.g	scaffold_5:42911507-42912104	83.2104	64.2511	-0.373042
Ciclev10029775m.g	scaffold_8:22197182-22200608	19.7946	15.285	-0.372987
Ciclev10016237m.g	scaffold_2:13906793-13909086	42.5665	32.873	-0.372813
-	scaffold_3:32816686-32817201	81.5052	62.9474	-0.372743
Ciclev10000941m.g	scaffold_5:35065342-35070910	12.9453	9.99866	-0.372617
Ciclev10011811m.g	scaffold_6:18059849-18064527	12.9143	9.97487	-0.372596
Ciclev10000990m.g	scaffold_5:37709913-37713327	6.47378	5.00088	-0.372425
Ciclev10004249m.g	scaffold_9:29130638-29136024	29.829	23.0434	-0.372363
Ciclev10002104m.g	scaffold_5:35187716-35211685	0.203372	0.157109	-0.372353
Ciclev10015323m.g	scaffold_2:9365310-9371079	24.1177	18.6323	-0.372287
Ciclev10001030m.g	scaffold_5:38090538-38095612	24.6297	19.0279	-0.372286
Ciclev10029085m.g	scaffold_8:22209234-22212108	0.192711	0.148896	-0.372135
Ciclev10022969m.g	scaffold_3:4232103-4234557	92.9128	71.7974	-0.371945
Ciclev10002106m.g	scaffold_5:38469257-38470252	3.29783	2.54837	-0.371943
Ciclev10005567m.g	scaffold_9:2723075-2725283	17.6092	13.6075	-0.371928
Ciclev10002548m.g	scaffold_5:19511363-19515330	25.2616	19.5256	-0.371578
Ciclev10015360m.g	scaffold_2:8742984-8745818	26.9758	20.8516	-0.37151
Ciclev10033518m.g	scaffold_4:23587464-23591486	91.6616	70.8526	-0.371498
Ciclev10022263m.g	scaffold_3:42155512-42159500	39.9746	30.9014	-0.37141
Ciclev10003137m.g	scaffold_5:27375152-27377371	50.6974	39.2006	-0.371034
Ciclev10009918m.g	scaffold_1:3750238-3752032	53.6278	41.4695	-0.370929
-	scaffold_5:2902730-2905332	23.5639	18.2218	-0.370914
Ciclev10001975m.g	scaffold_5:31903631-31906548	11.3979	8.81485	-0.370763
Ciclev10025244m.g	scaffold_7:19162656-19167025	12.1075	9.36383	-0.370735
Ciclev10002068m.g	scaffold_5:39300390-39301722	0.863174	0.667591	-0.370687
Ciclev10016899m.g	scaffold_2:27019344-27022514	44.1361	34.1369	-0.370625
Ciclev10031602m.g	scaffold_4:2434487-2438383	21.2285	16.4195	-0.370592
Ciclev10026620m.g	scaffold_7:1233609-1239263	59.1194	45.7277	-0.370563
Ciclev10020680m.g	scaffold_3:4939307-4940643	5.42273	4.19511	-0.37031
Ciclev10022413m.g	scaffold_3:2798608-2801856	29.0636	22.4844	-0.370288
Ciclev10022885m.g	scaffold_3:38639877-38875210	14.7416	11.405	-0.370225
Ciclev10011790m.g	scaffold_6:15414774-15418041	32.9849	25.5205	-0.370151
Ciclev10023364m.g	scaffold_3:48420367-48420695	40.8179	31.5812	-0.370136
Ciclev10028099m.g	scaffold_8:21461942-21467922	14.5645	11.2698	-0.369998
Ciclev10006440m.g	scaffold_9:17162447-17165582	4.33427	3.3541	-0.369863
Ciclev10022327m.g	scaffold_3:907954-909544	6.46721	5.0048	-0.369831
Ciclev10032200m.g,Ciclev10034009m.g	scaffold_4:17417354-17469137	23.5075	18.1923	-0.369796
Ciclev10005804m.g	scaffold_9:27159311-27160462	49.9675	38.6712	-0.369732
Ciclev10016613m.g	scaffold_2:30599334-30602528	323.009	249.998	-0.369659
Ciclev10012259m.g	scaffold_6:19265823-19270947	114.079	88.3037	-0.369483
Ciclev10004559m.g	scaffold_9:14845593-14856063	10.5958	8.20196	-0.369449
Ciclev10002412m.g	scaffold_5:41850269-41852717	51.9017	40.1773	-0.369401
Ciclev10032867m.g	scaffold_4:22997595-23000653	66.8186	51.7299	-0.36925
Ciclev10032423m.g	scaffold_4:19970059-19974163	13.2232	10.2382	-0.369105
Ciclev10027423m.g	scaffold_7:4813262-4813789	350.804	271.622	-0.369064
Ciclev10016814m.g	scaffold_2:20399880-20405870	38.648	29.9292	-0.368839
Ciclev10007350m.g	scaffold_1:7213253-7218074	20.8065	16.1142	-0.368701
Ciclev10001506m.g	scaffold_5:35521571-35526568	10.1156	7.83457	-0.368651
Ciclev10026478m.g	scaffold_7:1274861-1275645	0.393868	0.305131	-0.368283
Ciclev10012198m.g	scaffold_6:16230125-16231785	0.749535	0.580755	-0.368065
Ciclev10008671m.g	scaffold_1:23837941-23840989	7.2597	5.62542	-0.367949
Ciclev10022793m.g	scaffold_3:10425914-10435293	6632.06	5139.21	-0.367911
Ciclev10007882m.g	scaffold_1:23072855-23077664	42.4406	32.8906	-0.36777
Ciclev10031192m.g	scaffold_4:23002373-23006798	6.63781	5.146	-0.367256
Ciclev10018849m.g	scaffold_3:46482148-46489916	51.0747	39.5963	-0.367243

Ciclev10032092m.g	scaffold_4:19280283-19286048	1.67772	1.30102	-0.366861
Ciclev10001706m.g,Ciclev10003426m.g	scaffold_5:35187716-35211685	30.0297	23.2878	-0.366813
Ciclev10009909m.g	scaffold_1:24787599-24788444	0.285597	0.221482	-0.366788
Ciclev10032426m.g	scaffold_4:20967361-20968415	25.1354	19.4941	-0.366681
Ciclev10019811m.g	scaffold_3:23572802-23614239	12.6346	9.80034	-0.366473
Ciclev10026675m.g	scaffold_7:1698933-1700014	0.197318	0.153068	-0.366349
Ciclev10028103m.g	scaffold_8:3010002-3014264	98.5693	76.4703	-0.366238
Ciclev10009926m.g	scaffold_1:22451735-22454183	118.907	92.2578	-0.366087
Ciclev10008332m.g	scaffold_1:26450529-26458332	20.7242	16.0796	-0.366082
Ciclev10031700m.g,Ciclev10033217m.g	scaffold_4:3773993-3776411	15.136	11.7439	-0.366074
Ciclev10007991m.g	scaffold_1:3785660-3789593	10.3321	8.0187	-0.365689
Ciclev10002710m.g	scaffold_5:41618252-41620257	142.617	110.726	-0.365143
Ciclev10006017m.g	scaffold_9:29548143-29548977	281.162	218.344	-0.3648
Ciclev10020458m.g	scaffold_3:39365661-39369964	57.236	44.4513	-0.364695
Ciclev10019912m.g	scaffold_3:25248366-25288105	28.293	21.9768	-0.364466
Ciclev10004391m.g	scaffold_9:24672267-24677081	10.38	8.0645	-0.364154
Ciclev10030904m.g	scaffold_4:569470-574327	7.90054	6.13847	-0.364072
Ciclev10005642m.g	scaffold_9:13427131-13430543	73.0095	56.7423	-0.36366
Ciclev10018610m.g	scaffold_3:1136007-1142628	21.9301	17.045	-0.363568
Ciclev10010921m.g	scaffold_6:16397312-16411495	14.4615	11.2401	-0.363567
Ciclev10017201m.g	scaffold_2:8125926-8128247	26.0315	20.2464	-0.362593
Ciclev10012739m.g	scaffold_6:21389136-21390399	0.232656	0.180957	-0.362552
Ciclev10018911m.g	scaffold_3:43987024-43990983	1.78569	1.38901	-0.362425
Ciclev10026208m.g	scaffold_7:535263-538670	23.3646	18.1791	-0.36204
Ciclev10016889m.g	scaffold_2:7406303-7408138	2.18421	1.69963	-0.361895
Ciclev10029204m.g	scaffold_8:22093002-22098082	18.2392	14.1933	-0.361832
Ciclev10014166m.g	scaffold_2:24644218-24650568	13.6234	10.6029	-0.361625
Ciclev10026461m.g	scaffold_7:15106301-15109977	59.4347	46.2638	-0.361422
Ciclev10026133m.g	scaffold_7:3903231-3907502	261.074	203.241	-0.361269
Ciclev10002290m.g	scaffold_5:29400845-29403554	35.4263	27.5852	-0.360928
Ciclev10024072m.g	scaffold_3:23715297-23716953	1.67329	1.30299	-0.360868
-	scaffold_6:24536187-24537417	33.0748	25.7576	-0.360737
Ciclev10020477m.g,Ciclev10023583m.g,Ciclev10023763m.g,Ciclev10024414m.g	scaffold_3:40848610-40913406	32.2025	25.0788	-0.360704
Ciclev10026319m.g	scaffold_7:6872665-6877291	44.1481	34.3838	-0.360621
Ciclev10031729m.g	scaffold_4:325294-327314	3.09493	2.41046	-0.360598
Ciclev10002116m.g	scaffold_5:24806463-24809020	44.2297	34.4481	-0.360588
Ciclev10007511m.g,Ciclev10007765m.g	scaffold_1:28138396-28149541	4.94332	3.85015	-0.360564
Ciclev10031244m.g	scaffold_4:14688023-14692300	17.4603	13.6027	-0.360178
Ciclev10008868m.g	scaffold_1:1781758-1785065	66.6563	51.9482	-0.359666
Ciclev10011539m.g	scaffold_6:13137213-13139744	63.5231	49.5072	-0.359643
Ciclev10015995m.g	scaffold_2:31821261-31822604	0.703907	0.548666	-0.359456
Ciclev10001824m.g	scaffold_5:41513451-41514893	0.345058	0.268969	-0.3594
Ciclev10005302m.g	scaffold_9:26680724-26683812	5.96632	4.65157	-0.359123
Ciclev10001999m.g	scaffold_5:30522831-30524198	6.87527	5.36034	-0.359093
Ciclev10009573m.g	scaffold_1:18569231-18571013	12.7131	9.91308	-0.358909
Ciclev10013476m.g	scaffold_6:22936545-22938841	2.55299	1.99086	-0.358799
Ciclev10005003m.g	scaffold_9:4733101-4736595	25.8804	20.1832	-0.358702
Ciclev10008969m.g	scaffold_1:25095004-25097485	38.2467	29.8277	-0.358684
Ciclev10032087m.g	scaffold_4:20556553-20557809	10.6589	8.31306	-0.358611
Ciclev10029860m.g	scaffold_8:12925558-12936454	3.2152	2.50768	-0.358556
Ciclev10030847m.g	scaffold_4:3152792-3160873	39.3844	30.72	-0.358446

Ciclev10003709m.g	scaffold_5:41308041-41309940	0.165145	0.128848	-0.358064
Ciclev10017019m.g	scaffold_2:29351435-29352806	428.56	334.374	-0.358033
Ciclev10018685m.g	scaffold_3:1705988-1710077	1.18786	0.927005	-0.357715
Ciclev10002625m.g	scaffold_5:38979502-38982238	144.298	112.616	-0.357636
Ciclev10001509m.g	scaffold_5:41190081-41192832	19.1765	14.9668	-0.357575
Ciclev10026008m.g	scaffold_7:5335234-5337889	2.02477	1.58031	-0.357549
Ciclev10011734m.g	scaffold_6:14472656-14477261	5.04482	3.93796	-0.357355
Ciclev10028444m.g	scaffold_8:23410430-23412632	14.9679	11.6842	-0.35731
Ciclev10020858m.g	scaffold_3:1288174-1294561	64.0727	50.019	-0.357232
Ciclev10018674m.g	scaffold_3:41885839-41888860	0.0488483	0.0381353	-0.35718
Ciclev10020423m.g	scaffold_3:3890332-3893103	8.59252	6.70844	-0.357104
Ciclev10027109m.g	scaffold_7:5823654-5827373	34.4393	26.8887	-0.35706
Ciclev10003160m.g	scaffold_5:4076819-4284428	132.182	103.229	-0.35668
Ciclev10029606m.g	scaffold_8:469565-474874	10.6862	8.34601	-0.356594
Ciclev10018996m.g	scaffold_3:29979422-29987188	6.55412	5.11933	-0.356446
Ciclev10025046m.g	scaffold_7:2328816-2337471	51.7698	40.4371	-0.35643
Ciclev10001111m.g	scaffold_5:17947452-17948817	2.36817	1.84989	-0.356334
Ciclev10031546m.g	scaffold_4:20331059-20332526	0.148068	0.115665	-0.356311
Ciclev10020546m.g	scaffold_3:2953188-2957407	17.4143	13.6041	-0.356234
Ciclev10024179m.g	scaffold_3:21296577-21473295	0.520102	0.406333	-0.35613
Ciclev10005578m.g	scaffold_9:25585704-25588460	8.58913	6.71039	-0.356115
Ciclev10033302m.g	scaffold_4:19982138-19983197	0.46655	0.364511	-0.356068
Ciclev10015570m.g	scaffold_2:7250530-7255818	36.3624	28.4101	-0.356041
Ciclev10033310m.g	scaffold_4:19001055-19040863	11.7219	9.15866	-0.355995
Ciclev10015893m.g	scaffold_2:36211960-36216858	71.6472	55.9875	-0.355807
Ciclev10015596m.g	scaffold_2:29588936-29590279	0.160681	0.125587	-0.355517
Ciclev10030882m.g	scaffold_4:24704710-24710503	1.00261	0.783663	-0.355451
Ciclev10033061m.g	scaffold_4:25058290-25061910	3627.87	2835.81	-0.355362
Ciclev10004939m.g	scaffold_9:16245482-16251934	31.283	24.4574	-0.355109
Ciclev10011546m.g	scaffold_6:22523212-22529556	29.4893	23.0551	-0.355103
Ciclev10018031m.g	scaffold_2:3313126-3315167	2.48263	1.94096	-0.355096
Ciclev10010759m.g,Ciclev10010796m.g	scaffold_1:12802422-12810731	104.999	82.1122	-0.354711
Ciclev10014986m.g	scaffold_2:31940558-31950750	62.7056	49.0435	-0.354531
Ciclev10009010m.g	scaffold_1:26773395-26776856	59.756	46.7369	-0.354521
Ciclev10012695m.g	scaffold_6:8237013-8239040	17.2169	13.4685	-0.354236
Ciclev10012203m.g	scaffold_6:21920374-21922631	39.8077	31.1442	-0.354086
Ciclev10021893m.g	scaffold_3:37098314-37103303	19.4869	15.2471	-0.353964
Ciclev10027493m.g	scaffold_7:11850299-12160617	0.776312	0.607481	-0.353799
Ciclev10032809m.g	scaffold_4:23331664-23333081	27.4872	21.5094	-0.353795
Ciclev10016578m.g	scaffold_2:22019866-22022847	175.013	136.961	-0.353698
Ciclev10031605m.g	scaffold_4:24909949-24913654	33.6862	26.365	-0.353536
Ciclev10021622m.g	scaffold_3:43472093-43473220	67.8093	53.0719	-0.353535
Ciclev10031116m.g	scaffold_4:3849031-3862473	27.5553	21.5666	-0.353534
Ciclev10004814m.g	scaffold_9:2647856-2652393	18.8182	14.7288	-0.353489
Ciclev10026666m.g	scaffold_7:7270100-7271553	69.5389	54.4276	-0.353484
Ciclev10000984m.g	scaffold_5:33028396-33034775	36.4435	28.5258	-0.353392
Ciclev10032356m.g	scaffold_4:1385386-1386410	1.9391	1.51801	-0.353209
Ciclev10022273m.g	scaffold_3:41398175-41402035	13.6686	10.7006	-0.353171
Ciclev10018536m.g	scaffold_3:34654253-34665460	11.8625	9.28707	-0.35311
Ciclev10033492m.g	scaffold_4:22547989-22550074	157.664	123.435	-0.3531
Ciclev10025085m.g	scaffold_7:3501753-3506479	11.6335	9.10937	-0.352863
Ciclev10018387m.g	scaffold_2:5135442-5140979	0.627087	0.491055	-0.352781
Ciclev10027246m.g	scaffold_7:6692687-6700236	0.0631334	0.0494457	-0.35256
Ciclev10018781m.g	scaffold_3:1759052-1765932	24.7138	19.3568	-0.352472
Ciclev10002367m.g	scaffold_5:40735855-40737496	62.8683	49.2415	-0.352458

Ciclev10000782m.g	scaffold_5:8512379-8517973	14.7443	11.5487	-0.352424
Ciclev10022348m.g	scaffold_3:44987718-44991534	45.3893	35.552	-0.352419
Ciclev10028350m.g,Ciclev10028381m.g,Ciclev10028384m.g	scaffold_8:4292179-4375670	604.43	473.481	-0.352269
Ciclev10000458m.g	scaffold_5:35517170-35521219	15.8217	12.395	-0.35215
Ciclev10016231m.g	scaffold_2:9351629-9355095	29.4095	23.0416	-0.352039
Ciclev10029731m.g	scaffold_8:12237358-12240488	0.271599	0.212819	-0.351853
Ciclev10017823m.g	scaffold_2:25999031-26002655	10.4377	8.17929	-0.351751
Ciclev10019700m.g	scaffold_3:43815646-43818118	0.440527	0.345214	-0.351738
Ciclev10027331m.g	scaffold_7:5908416-5915731	41.9652	32.8862	-0.351713
Ciclev10031435m.g	scaffold_4:17888930-17894661	12.7665	10.0051	-0.351627
Ciclev10009474m.g	scaffold_1:2038680-2041094	25.4481	19.9462	-0.351447
Ciclev10005460m.g	scaffold_9:594296-596633	57.1148	44.7713	-0.351291
Ciclev10004572m.g	scaffold_9:836229-842228	25.5446	20.0251	-0.35121
Ciclev10022955m.g	scaffold_3:6218544-6219405	71.1869	55.8072	-0.351158
Ciclev10003039m.g	scaffold_5:19275096-19276356	18.8545	14.7827	-0.351004
Ciclev10030677m.g	scaffold_4:21772521-21777465	15.1168	11.8528	-0.350932
Ciclev10030115m.g	scaffold_8:8422589-8423196	1.80838	1.41801	-0.350826
Ciclev10007243m.g	scaffold_1:28787153-28792998	6.52042	5.1131	-0.350767
Ciclev10030297m.g	scaffold_8:21095288-21102525	63.6712	49.9327	-0.350657
Ciclev10002208m.g	scaffold_5:34896600-34897733	0.217292	0.170427	-0.350478
Ciclev10011044m.g	scaffold_6:17483131-17489738	21.9389	17.21	-0.350246
Ciclev10030007m.g	scaffold_8:3961200-3962790	1.6342	1.28205	-0.350131
Ciclev10025165m.g	scaffold_7:8616533-8625549	15.78	12.382	-0.34985
Ciclev10019395m.g,Ciclev10023057m.g,Ciclev10023853m.g	scaffold_3:35372249-35712253	55.499	43.55	-0.349788
Ciclev10026137m.g	scaffold_7:2674506-2677232	6.8268	5.35699	-0.349787
Ciclev10021307m.g	scaffold_3:40261790-40266885	57.4321	45.069	-0.349722
Ciclev10026310m.g	scaffold_7:2299637-2302863	31.9183	25.0486	-0.349653
Ciclev10002362m.g	scaffold_5:24760556-24762003	231.145	181.406	-0.34958
Ciclev10021057m.g	scaffold_3:50378162-50380916	39.1252	30.7091	-0.349432
Ciclev10032537m.g	scaffold_4:102101-107421	151.06	118.587	-0.349176
Ciclev10019521m.g	scaffold_3:3339666-3344735	3.35336	2.6329	-0.348954
Ciclev10021749m.g	scaffold_3:7808705-7814222	23.7891	18.6782	-0.348944
Ciclev10018957m.g	scaffold_3:36702871-36705959	3.70736	2.91101	-0.348875
-	scaffold_3:27371669-27372192	14.5991	11.468	-0.348272
Ciclev10019458m.g	scaffold_3:495657-500678	22.0385	17.313	-0.348165
Ciclev10008889m.g	scaffold_1:5179147-5182620	27.6791	21.7451	-0.348106
Ciclev10009608m.g	scaffold_1:27535286-27536764	33.1404	26.0374	-0.348006
Ciclev10012654m.g	scaffold_6:10316998-10318328	752.603	591.315	-0.347964
Ciclev10006135m.g	scaffold_9:2431248-2433257	3.57141	2.80615	-0.347899
-	scaffold_4:11523554-11728349	23.9128	18.7894	-0.347862
Ciclev10026121m.g	scaffold_7:1870932-1874304	136.475	107.25	-0.347665
Ciclev10005135m.g	scaffold_9:21599567-21603588	14.182	11.1459	-0.347554
Ciclev10023799m.g	scaffold_3:44702949-44705581	16.5341	12.9955	-0.34744
Ciclev10014668m.g	scaffold_2:19838049-19844296	9.49736	7.46602	-0.347187
Ciclev10008613m.g	scaffold_1:3656492-3659822	12.3706	9.72499	-0.347142
Ciclev10023184m.g	scaffold_3:2942912-2944293	322.367	253.465	-0.346915
Ciclev10028112m.g	scaffold_8:24373946-24377968	143.58	112.92	-0.34656
Ciclev10000463m.g	scaffold_5:36317431-36322995	34.6296	27.2358	-0.346499
Ciclev10032906m.g	scaffold_4:24032080-24033681	36.3666	28.6021	-0.346496
Ciclev10005658m.g	scaffold_9:8638575-8640776	55.6029	43.7342	-0.3464
Ciclev10010088m.g	scaffold_1:28334535-28336598	206.224	162.211	-0.346345
Ciclev10033826m.g	scaffold_4:19163046-19164164	0.336113	0.26439	-0.346276

Ciclev10017998m.g	scaffold_2:7904951-7905653	0.762242	0.599628	-0.346182
Ciclev10021939m.g	scaffold_3:5679929-5683309	25.999	20.4533	-0.346127
Ciclev10027590m.g	scaffold_7:10976435-10979000	0.954784	0.75113	-0.346113
Ciclev10013553m.g	scaffold_6:20294040-20299455	89.256	70.2178	-0.346112
Ciclev10024457m.g	scaffold_3:2191112-2192674	0.394664	0.310533	-0.345878
Ciclev10003826m.g	scaffold_5:4915969-4917216	1.72308	1.3559	-0.345736
Ciclev10022873m.g	scaffold_3:49433712-49434245	1.13076	0.889977	-0.345457
Ciclev10008855m.g	scaffold_1:7264568-7269002	8.98185	7.07089	-0.34512
Ciclev10019763m.g	scaffold_3:49554049-49560198	75.0625	59.0933	-0.345098
Ciclev10012459m.g	scaffold_6:20024369-20027538	954.341	751.356	-0.345007
Ciclev10017034m.g	scaffold_2:35667231-35668414	8.37073	6.59146	-0.344756
Ciclev10027020m.g	scaffold_7:3754022-3756116	45.2163	35.6108	-0.344528
Ciclev10033154m.g	scaffold_4:11771544-11772109	119.789	94.3654	-0.34417
-	scaffold_8:1194827-1195126	115.787	91.2142	-0.344143
Ciclev10017685m.g	scaffold_2:23713588-23714725	0.180161	0.141941	-0.343994
Ciclev10032556m.g	scaffold_4:11918617-11930009	16.0769	12.6671	-0.343894
Ciclev10025780m.g	scaffold_7:6671757-6675798	14.0436	11.067	-0.343649
Ciclev10030418m.g	scaffold_8:23972304-23973115	0.758318	0.597629	-0.343553
Ciclev10001700m.g	scaffold_5:38404014-38407692	3.62344	2.85607	-0.343331
Ciclev10001213m.g,Ciclev10001239m.g	scaffold_5:32737146-32758322	49.1253	38.7249	-0.343206
Ciclev10022689m.g	scaffold_3:3623693-3625333	111.485	87.8863	-0.343133
Ciclev10024788m.g	scaffold_7:5020269-5031631	18.0723	14.2469	-0.343131
Ciclev10029554m.g	scaffold_8:24427858-24429582	5.92136	4.66807	-0.343103
Ciclev10006128m.g	scaffold_9:6330883-6332137	11.9608	9.42951	-0.343063
Ciclev10016172m.g	scaffold_2:29379161-29384201	23.0713	18.1902	-0.34294
Ciclev10031288m.g	scaffold_4:2755399-2757980	52.0935	41.0736	-0.342891
Ciclev10012444m.g	scaffold_6:20135561-20136831	1.42616	1.12471	-0.342577
Ciclev10030144m.g	scaffold_8:1491361-1556980	0.205935	0.16242	-0.342463
Ciclev10014622m.g	scaffold_2:29449881-29459747	22.3805	17.6536	-0.342281
Ciclev10031120m.g	scaffold_4:11412330-11417624	29.1888	23.0257	-0.342166
Ciclev10000480m.g	scaffold_5:16238638-16242339	13.994	11.0408	-0.341967
Ciclev10033233m.g	scaffold_4:13903685-13910631	0.813007	0.641445	-0.341941
Ciclev10013228m.g	scaffold_6:1950120-1950908	89.7459	70.8111	-0.341871
Ciclev10021716m.g	scaffold_3:41576812-41581730	8.52267	6.72616	-0.341523
Ciclev10009255m.g	scaffold_1:4861411-4866144	90.8104	71.6728	-0.341431
Ciclev10002841m.g	scaffold_5:35647841-35649029	51.1035	40.3343	-0.341415
Ciclev10025517m.g	scaffold_7:7883413-7886933	0.206781	0.163206	-0.341405
Ciclev10016721m.g	scaffold_2:25729607-25732435	12.2243	9.65109	-0.340992
Ciclev10021414m.g	scaffold_3:48421596-48426413	8.22278	6.49245	-0.340863
Ciclev10017248m.g	scaffold_2:27141147-27141869	63.6351	50.2444	-0.340858
Ciclev10002244m.g	scaffold_5:17058859-17070837	13.1706	10.3999	-0.34075
Ciclev10033085m.g	scaffold_4:25445604-25446251	188.065	148.507	-0.340695
Ciclev10015379m.g	scaffold_2:35806348-35811690	69.866	55.1757	-0.340558
Ciclev10021411m.g	scaffold_3:23891294-23893475	12.1599	9.60448	-0.340355
Ciclev10012740m.g	scaffold_6:13630037-13632023	1.70566	1.34724	-0.340322
Ciclev10000881m.g	scaffold_5:40550221-40554434	4.14994	3.2782	-0.340186
Ciclev10023834m.g	scaffold_3:48016068-48017656	154.884	122.361	-0.34005
Ciclev10030491m.g	scaffold_4:6325179-6334300	13.339	10.5391	-0.339889
Ciclev10010342m.g	scaffold_1:7787593-7790004	0.460032	0.363592	-0.339415
Ciclev10009643m.g	scaffold_1:2420323-2423040	108.603	85.8567	-0.339068
Ciclev10030221m.g	scaffold_8:808495-811820	39.4064	31.1555	-0.338944
Ciclev10018611m.g	scaffold_3:27976742-27982947	3.21199	2.5395	-0.338923
Ciclev10026857m.g	scaffold_7:19686425-19686911	0.59826	0.473055	-0.338764
Ciclev10017211m.g	scaffold_2:33863105-33865585	27.1514	21.4705	-0.338673
Ciclev10021740m.g	scaffold_3:50333873-50334980	2.85869	2.26076	-0.338545

Ciclev10016320m.g	scaffold_2:28991258-28992928	4.32683	3.42196	-0.338489
Ciclev10011519m.g	scaffold_6:13684424-13691301	6.90586	5.46176	-0.338457
Ciclev10014108m.g	scaffold_2:9232125-9242195	17.1888	13.5947	-0.338424
Ciclev10020127m.g	scaffold_3:49052905-49056242	10.9591	8.6693	-0.338142
Ciclev10005268m.g	scaffold_9:3696391-3698741	1.74545	1.38077	-0.338132
Ciclev10001862m.g	scaffold_5:36344743-36347545	88.2579	69.8182	-0.338123
Ciclev10002707m.g	scaffold_5:35624924-35626992	32.4685	25.6884	-0.337925
Ciclev10009108m.g	scaffold_1:6332706-6334356	0.254224	0.201146	-0.337856
Ciclev10030696m.g	scaffold_4:13941614-13951313	82.6061	65.3655	-0.337717
Ciclev10023570m.g	scaffold_3:754956-758039	21.2615	16.8247	-0.337659
Ciclev10017672m.g	scaffold_2:33405293-33408781	99.4429	78.7039	-0.337433
Ciclev10028615m.g	scaffold_8:12890292-12894971	72.1392	57.1081	-0.337088
Ciclev10001007m.g	scaffold_5:41787407-41790854	41.5612	32.9026	-0.337035
Ciclev10015950m.g	scaffold_2:19530112-19532329	31.2773	24.763	-0.336932
Ciclev10014379m.g	scaffold_2:23230584-23236238	8.16785	6.46675	-0.336915
Ciclev10023468m.g	scaffold_3:32626816-32627452	0.832337	0.658992	-0.336907
Ciclev10020293m.g	scaffold_3:34521797-34529861	9.04974	7.16577	-0.336753
Ciclev10023518m.g,Ciclev10024113m.g	scaffold_3:25204873-25207379	13.3036	10.536	-0.33649
Ciclev10002713m.g	scaffold_5:22711547-22715413	43.1239	34.1538	-0.336442
Ciclev10008714m.g	scaffold_1:24512787-24516864	202.305	160.228	-0.336408
Ciclev10002103m.g	scaffold_5:1311648-1355130	3.17551	2.51506	-0.336394
Ciclev10025677m.g	scaffold_7:190663-192426	93.6795	74.1987	-0.33634
Ciclev10032332m.g	scaffold_4:15807588-15810104	17.3977	13.7837	-0.335934
Ciclev10008925m.g	scaffold_1:4514414-4516096	0.33859	0.268282	-0.33579
Ciclev10007589m.g	scaffold_1:1680898-1687851	10.3731	8.22009	-0.335618
Ciclev10009813m.g	scaffold_1:693214-695933	74.6309	59.1413	-0.335606
Ciclev10028359m.g	scaffold_8:20352231-20354410	14.4521	11.4562	-0.33516
Ciclev10003159m.g	scaffold_5:30507659-30511514	0.877687	0.695742	-0.335154
Ciclev10030724m.g	scaffold_4:24971611-24985270	8.55835	6.78681	-0.334599
Ciclev10031340m.g	scaffold_4:16737522-16740944	2.31733	1.83769	-0.334571
Ciclev10024979m.g	scaffold_7:5150546-5159703	85.371	67.7013	-0.334563
Ciclev10027904m.g	scaffold_8:21470292-21478473	11.1448	8.83968	-0.334306
Ciclev10004446m.g	scaffold_9:12808153-12820311	122.571	97.2225	-0.334255
Ciclev10001617m.g	scaffold_5:42589391-42594049	14.44	11.4544	-0.334175
Ciclev10004852m.g	scaffold_9:16971263-16973312	303.864	241.051	-0.334084
Ciclev10019734m.g	scaffold_3:49821905-49825730	50.1868	39.8167	-0.333935
Ciclev10012951m.g	scaffold_6:14178289-14181908	13.0442	10.349	-0.333923
-	scaffold_7:13676436-13677788	6.83622	5.42372	-0.333917
Ciclev10017480m.g	scaffold_2:34755345-34756943	70.3043	55.7781	-0.333912
Ciclev10018181m.g	scaffold_2:35830654-35832364	8.36305	6.6352	-0.333889
Ciclev10029562m.g	scaffold_8:23561256-23563244	19.045	15.1117	-0.333746
Ciclev10004333m.g	scaffold_9:7699003-7710008	13.4915	10.7076	-0.333422
Ciclev10024998m.g	scaffold_7:5493731-5502436	21.2995	16.9048	-0.33338
Ciclev10010253m.g	scaffold_1:24518936-24521304	0.421035	0.334166	-0.333374
Ciclev10006491m.g	scaffold_9:21186802-21188959	0.778819	0.618154	-0.333323
Ciclev10000855m.g	scaffold_5:39559029-39561472	0.448168	0.355733	-0.333246
Ciclev10021868m.g	scaffold_3:4831750-4834270	272.06	215.958	-0.333176
Ciclev10001712m.g	scaffold_5:37381117-37384861	7.68726	6.10329	-0.332884
Ciclev10014231m.g,Ciclev10014945m.g	scaffold_2:28845910-28867192	6.89897	5.47759	-0.332841
Ciclev10006717m.g	scaffold_9:4548430-4550326	31.7551	25.213	-0.332822
Ciclev10003142m.g,Ciclev10004080m.g	scaffold_5:40297491-40299711	30.3953	24.1341	-0.332777
Ciclev10005174m.g	scaffold_9:5079816-5081252	0.297912	0.236546	-0.332761
Ciclev10002498m.g	scaffold_5:40457008-40460158	24.4135	19.3847	-0.33276

Ciclev10026767m.g	scaffold_7:7313005-7315653	25.6507	20.3673	-0.332749
Ciclev10010392m.g	scaffold_1:9319815-9321373	107.965	85.73	-0.332696
Ciclev10027838m.g	scaffold_8:2407189-2412578	2.17657	1.72845	-0.33258
Ciclev10022697m.g	scaffold_3:23731183-23733880	20.9395	16.6293	-0.332504
Ciclev10005402m.g,Ciclev10005454m.g	scaffold_9:29186957-29194048	26.2088	20.8155	-0.332395
Ciclev10019510m.g	scaffold_3:47151470-47153278	0.100102	0.0795054	-0.332344
Ciclev10031090m.g	scaffold_4:24916682-24919674	22.0351	17.5017	-0.33231
Ciclev10015071m.g	scaffold_2:32892597-32898169	18.3364	14.5653	-0.332181
Ciclev10028145m.g	scaffold_8:1369168-1376108	9.72336	7.72543	-0.331839
Ciclev10012679m.g	scaffold_6:17002903-17003946	199.586	158.592	-0.331686
Ciclev10028288m.g	scaffold_8:21868752-21874117	13.6783	10.87	-0.33154
Ciclev10016785m.g	scaffold_2:16553370-16554188	2.6335	2.09298	-0.331423
Ciclev10010710m.g	scaffold_1:158901-161375	0.177532	0.14112	-0.331158
Ciclev10023907m.g	scaffold_3:21296577-21473295	1.30122	1.03439	-0.331078
Ciclev10011083m.g	scaffold_6:5316526-5324712	14.4619	11.4965	-0.331062
-	scaffold_8:14549880-14551502	73.9362	58.7813	-0.330924
Ciclev10016185m.g	scaffold_2:23313423-23317150	47.1711	37.5025	-0.330919
Ciclev10001966m.g	scaffold_5:20076268-20079754	24.8302	19.7465	-0.330493
Ciclev10021188m.g	scaffold_3:8621740-8623528	12.4743	9.92056	-0.330469
Ciclev10002463m.g	scaffold_5:176649-180570	108.638	86.4139	-0.33019
Ciclev10020139m.g	scaffold_3:13986076-13991027	2.76223	2.1972	-0.330169
Ciclev10021063m.g	scaffold_3:1022283-1024330	0.1438	0.114409	-0.329872
Ciclev10002401m.g	scaffold_5:39421060-39423811	221.154	175.971	-0.329717
Ciclev10004957m.g	scaffold_9:29139659-29144050	7.95331	6.3293	-0.329511
Ciclev10024391m.g	scaffold_3:45941779-45943616	0.120236	0.0956971	-0.329321
Ciclev10025171m.g	scaffold_7:9733633-9808728	0.690089	0.549262	-0.329288
Ciclev10019597m.g	scaffold_3:48762648-48765718	12.0741	9.61233	-0.32896
Ciclev10004051m.g	scaffold_5:29545386-29545962	0.851601	0.677985	-0.328923
Ciclev10018933m.g	scaffold_3:48589734-48601762	8.44407	6.72272	-0.328895
Ciclev10000190m.g	scaffold_5:42870966-42874870	26.8529	21.384	-0.328546
-	scaffold_3:20345793-20346581	11.0996	8.83961	-0.328452
Ciclev10028303m.g	scaffold_8:10864062-10865838	17.7838	14.1638	-0.328362
Ciclev10032260m.g	scaffold_4:20378719-20381917	8.23324	6.55759	-0.328295
Ciclev10008865m.g,Ciclev10008880m.g	scaffold_1:7417421-7441321	48.4239	38.5696	-0.328256
Ciclev10023214m.g	scaffold_3:37941592-37943870	350.015	278.793	-0.328223
Ciclev10014618m.g	scaffold_2:28403936-28488203	5.94389	4.73472	-0.328127
Ciclev10007840m.g	scaffold_1:26174997-26176820	2.58189	2.05677	-0.328049
Ciclev10005129m.g	scaffold_9:21236662-21244381	6.81756	5.43097	-0.328046
Ciclev10030480m.g	scaffold_4:7038098-7048171	6.25209	4.98078	-0.327967
Ciclev10014081m.g	scaffold_2:34169175-34175022	9.96155	7.9361	-0.32794
Ciclev10018518m.g	scaffold_3:1208945-1215347	1.22723	0.977834	-0.327745
Ciclev10022089m.g	scaffold_3:19305598-19309195	30.7478	24.4999	-0.327709
Ciclev10005849m.g	scaffold_9:741796-744144	8.50072	6.77457	-0.327457
Ciclev10022957m.g	scaffold_3:47920329-47921349	20.8787	16.6427	-0.327139
-	scaffold_5:29675211-29675693	73.1966	58.3499	-0.327047
Ciclev10021157m.g	scaffold_3:10140903-10143672	10.8431	8.64457	-0.326916
Ciclev10025428m.g	scaffold_7:13636527-13641737	11.2601	8.97744	-0.326845
Ciclev10012007m.g	scaffold_6:18036337-18039129	7.86056	6.26756	-0.326727
Ciclev10019765m.g	scaffold_3:47092819-47099085	49.318	39.3243	-0.326693
Ciclev10002365m.g	scaffold_5:34822162-34825902	13.8366	11.0333	-0.326624
Ciclev10023193m.g	scaffold_3:13254231-13255708	160.387	127.896	-0.326588
Ciclev10012641m.g	scaffold_6:21546996-21549044	25.4569	20.3036	-0.326321
Ciclev10020210m.g	scaffold_3:47583336-47586652	38.865	30.9999	-0.326209
Ciclev10021020m.g	scaffold_3:32942462-32946682	81.6947	65.1677	-0.326085

Ciclev10004610m.g	scaffold_9:21970856-21985239	15.6853	12.5137	-0.325905
Ciclev10020454m.g	scaffold_3:6209509-6215018	24.9437	19.9048	-0.325558
Ciclev10033546m.g	scaffold_4:1020658-1023241	18.1873	14.5145	-0.325435
Ciclev10020832m.g	scaffold_3:20577773-20598741	21.6203	17.2594	-0.324998
Ciclev10002511m.g	scaffold_5:30730207-30732143	15.2528	12.1768	-0.32494
Ciclev10018733m.g	scaffold_3:38068172-38077968	14.644	11.6914	-0.324866
Ciclev10000961m.g	scaffold_5:28539341-28801354	11.5072	9.18706	-0.324866
Ciclev10011171m.g	scaffold_6:11177533-11180583	1.54254	1.23161	-0.32476
Ciclev10031235m.g	scaffold_4:22119092-22124960	26.4891	21.1514	-0.324646
Ciclev10012229m.g	scaffold_6:24276905-24280452	7.84285	6.26289	-0.32455
Ciclev10028334m.g	scaffold_8:2814140-2815707	59.6977	47.6715	-0.324546
Ciclev10001235m.g	scaffold_5:42675741-42682366	0.672316	0.536967	-0.324307
Ciclev10004318m.g	scaffold_9:13140193-13150856	10.6624	8.51735	-0.324055
Ciclev10031955m.g	scaffold_4:3882717-3885303	55.2424	44.1341	-0.323882
Ciclev10011767m.g	scaffold_6:12902086-12906699	67.8972	54.257	-0.323542
Ciclev10021619m.g	scaffold_3:13316415-13318060	2.61723	2.09169	-0.323375
Ciclev10020713m.g	scaffold_3:6708215-6710187	15.6362	12.4972	-0.323288
Ciclev10026771m.g,Ciclev10026951m.g	scaffold_7:21074575-21091634	31.3715	25.0739	-0.323269
Ciclev10029023m.g	scaffold_8:17651979-17655604	24.2769	19.4046	-0.32318
Ciclev10029750m.g	scaffold_8:20077658-20078589	5.15984	4.12454	-0.323095
Ciclev10025052m.g	scaffold_7:20548587-20557694	13.574	10.8522	-0.32286
Ciclev10019345m.g	scaffold_3:39416582-39419994	0.446645	0.357112	-0.322754
Ciclev10008958m.g	scaffold_1:5262744-5266604	56.2758	45.0005	-0.322573
Ciclev10014326m.g	scaffold_2:9820972-9829072	27.1148	21.6838	-0.322466
Ciclev10014781m.g	scaffold_2:29841903-29846845	13.581	10.8614	-0.322379
Ciclev10033000m.g	scaffold_4:24312107-24313141	634.386	507.407	-0.322219
Ciclev10031750m.g	scaffold_4:21666060-21671693	16.4582	13.1647	-0.322132
Ciclev10016084m.g	scaffold_2:26230654-26234306	0.188517	0.150797	-0.322081
Ciclev10015642m.g	scaffold_2:145508-147469	33.1111	26.487	-0.32203
Ciclev10002352m.g	scaffold_5:41735342-41738372	12.1535	9.72268	-0.321945
Ciclev10032113m.g	scaffold_4:22523091-22528307	13.1621	10.5312	-0.321722
Ciclev10025767m.g	scaffold_7:690119-693985	13.0534	10.4443	-0.321702
Ciclev10001711m.g	scaffold_5:33329875-33334212	9.3575	7.48744	-0.321651
Ciclev10005608m.g	scaffold_9:2974586-2980387	35.4741	28.3861	-0.321582
Ciclev10022375m.g	scaffold_3:43621621-43622288	0.4224	0.338005	-0.321563
Ciclev10021832m.g	scaffold_3:36924550-37054826	89.9992	72.0222	-0.321471
Ciclev10007668m.g	scaffold_1:3407664-3413915	95.4336	76.3717	-0.32146
Ciclev10030641m.g	scaffold_4:21472847-21478103	2.81191	2.25035	-0.321404
Ciclev10031777m.g	scaffold_4:19383823-19390838	83.3024	66.6671	-0.321383
Ciclev10019335m.g	scaffold_3:47208958-47214112	12.1949	9.76063	-0.321234
Ciclev10005271m.g	scaffold_9:16403440-16407875	24.8006	19.8522	-0.321079
Ciclev10026600m.g	scaffold_7:1864077-1866507	25.0118	20.0217	-0.321041
Ciclev10019404m.g	scaffold_3:49331730-49335628	7.79131	6.23736	-0.320931
Ciclev10032495m.g	scaffold_4:18793984-18798509	8.14553	6.52276	-0.320526
Ciclev10029280m.g	scaffold_8:15963291-15967798	66.7885	53.4963	-0.32016
Ciclev10033452m.g	scaffold_4:24294648-24300291	58.4347	46.8077	-0.320078
Ciclev10029696m.g	scaffold_8:21102978-21105476	154.962	124.138	-0.319973
Ciclev10022019m.g	scaffold_3:44900058-44900865	2.93258	2.34928	-0.319951
Ciclev10002053m.g	scaffold_5:28826545-28827698	0.720814	0.577511	-0.319777
Ciclev10009378m.g	scaffold_1:28424575-28427136	14.5822	11.6841	-0.319662
Ciclev10019112m.g	scaffold_3:43062615-43067761	2.13592	1.71161	-0.319498
-	scaffold_2:9053624-9053785	518.521	415.561	-0.319341
Ciclev10015936m.g	scaffold_2:28707339-28713424	14.3179	11.4768	-0.319091
Ciclev10019801m.g	scaffold_3:39100209-39102696	27.1131	21.7342	-0.319022
Ciclev10015904m.g	scaffold_2:26600401-26606954	6.22143	4.98741	-0.318954

Ciclev10028114m.g	scaffold_8:12938329-12942299	94.8911	76.0893	-0.318578
Ciclev10026526m.g	scaffold_7:19048239-19050341	207.673	166.533	-0.318512
Ciclev10012242m.g	scaffold_6:14765914-14777322	15.5834	12.4969	-0.318437
Ciclev10003654m.g	scaffold_5:24456299-24464558	126.235	101.235	-0.31841
Ciclev10015989m.g	scaffold_2:25528399-25533049	13.1927	10.5806	-0.318323
Ciclev10005318m.g	scaffold_9:1300653-1301993	5.21257	4.18106	-0.318124
Ciclev10014294m.g	scaffold_2:29338881-29346713	9.88967	7.93493	-0.317704
Ciclev10012904m.g	scaffold_6:8562430-8564865	20.5808	16.514	-0.317612
Ciclev10021930m.g	scaffold_3:39019361-39023158	28.9389	23.2214	-0.317553
Ciclev10011675m.g	scaffold_6:17725048-17727018	2.89545	2.32343	-0.31753
Ciclev10022587m.g	scaffold_3:49283437-49284914	32.2999	25.919	-0.317519
Ciclev10006443m.g	scaffold_9:24356661-24541601	15.3226	12.2962	-0.317449
Ciclev10031094m.g	scaffold_4:19665199-19670933	84.2662	67.6232	-0.317435
Ciclev10030236m.g	scaffold_8:19224319-19225160	0.802645	0.64416	-0.317342
Ciclev10021239m.g	scaffold_3:30717320-30725003	14.9958	12.035	-0.317331
Ciclev10001290m.g	scaffold_5:40060072-40063681	15.7723	12.6592	-0.317208
Ciclev10026070m.g	scaffold_7:3638783-3641338	0.131894	0.105866	-0.317149
Ciclev10024101m.g	scaffold_3:47754573-47758066	31.4795	25.2701	-0.316984
Ciclev10006012m.g	scaffold_9:10287532-10289558	35.7342	28.6893	-0.31679
Ciclev10029003m.g	scaffold_8:935922-937629	406.66	326.492	-0.316773
Ciclev10022037m.g	scaffold_3:47421545-47423766	12.658	10.1641	-0.316575
Ciclev10002837m.g	scaffold_5:29677633-29680577	74.1248	59.5216	-0.316543
Ciclev10012046m.g	scaffold_6:11848517-11852824	29.2269	23.47	-0.31648
Ciclev10009831m.g	scaffold_1:8136572-8137861	35.4296	28.4529	-0.316379
Ciclev10006167m.g	scaffold_9:29443689-29445638	37.7427	30.3113	-0.316344
Ciclev10026196m.g	scaffold_7:5621857-5625716	6.08913	4.89029	-0.316317
Ciclev10024269m.g	scaffold_3:49202498-49205155	45.3008	36.387	-0.316116
Ciclev10006268m.g	scaffold_9:10002026-10002750	0.351865	0.282648	-0.316016
Ciclev10009210m.g	scaffold_1:3699779-3704588	21.463	17.241	-0.316004
Ciclev10026193m.g	scaffold_7:15224590-15226971	0.195298	0.156896	-0.315875
Ciclev10012082m.g	scaffold_6:24721815-24726045	24.1707	19.4192	-0.315778
Ciclev10010325m.g	scaffold_1:9318618-9319218	1.06152	0.852978	-0.315548
Ciclev10020645m.g	scaffold_3:48758033-48761795	21.4868	17.2662	-0.3155
Ciclev10027588m.g	scaffold_7:4303460-4304729	10.7751	8.65883	-0.315453
Ciclev10000964m.g	scaffold_5:37473063-37478070	5.25302	4.22169	-0.315328
Ciclev10008955m.g	scaffold_1:20094574-20101958	65.0948	52.3147	-0.315326
Ciclev10020162m.g	scaffold_3:1015285-1020829	27.4255	22.0425	-0.315226
Ciclev10021999m.g	scaffold_3:26603125-26608232	16.4921	13.2566	-0.315065
Ciclev10001694m.g	scaffold_5:6464855-6470466	32.6914	26.2823	-0.31482
Ciclev10025816m.g	scaffold_7:7796590-7799786	214.608	172.536	-0.31481
Ciclev10010925m.g	scaffold_6:19759952-19772656	12.0975	9.72624	-0.314759
Ciclev10015327m.g	scaffold_2:23226393-23230298	53.7649	43.2293	-0.314653
Ciclev10002741m.g	scaffold_5:39957970-39960797	39.4531	31.7307	-0.314258
Ciclev10018947m.g	scaffold_3:11411336-11415886	41.4322	33.3351	-0.313712
Ciclev10005481m.g	scaffold_9:29488657-29491181	84.535	68.0156	-0.313684
Ciclev10011218m.g	scaffold_6:14486904-14489292	86.6078	69.6894	-0.313558
Ciclev10031083m.g	scaffold_4:25613153-25618069	10.7062	8.61479	-0.313554
Ciclev10012484m.g	scaffold_6:10785023-10788727	14.7982	11.9079	-0.313505
Ciclev10019126m.g	scaffold_3:9114328-9120895	17.4783	14.0647	-0.313492
Ciclev10015689m.g	scaffold_2:10907223-10911491	19.8318	15.9612	-0.313241
Ciclev10008717m.g	scaffold_1:22940353-22941713	0.373996	0.301016	-0.313183
Ciclev10009294m.g	scaffold_1:3070376-3071739	4.23111	3.40571	-0.313079
Ciclev10028607m.g	scaffold_8:23209598-23213914	8.41059	6.77026	-0.312996
Ciclev10021646m.g	scaffold_3:13392888-13398359	101.51	81.719	-0.312883
Ciclev10017250m.g	scaffold_2:13506079-13508503	42.2335	34.0035	-0.312704
Ciclev10021720m.g	scaffold_3:26660973-26662413	12.2871	9.89288	-0.312677

Ciclev10014404m.g	scaffold_2:538542-545225	7.65399	6.16273	-0.312644
Ciclev10016601m.g	scaffold_2:30893753-30897946	0.662493	0.533418	-0.312638
Ciclev10019282m.g	scaffold_3:181595-185335	21.081	16.9766	-0.312391
Ciclev10003775m.g	scaffold_5:42095626-42097285	12.6045	10.1513	-0.312278
Ciclev10020871m.g	scaffold_3:25087316-25089114	76.3921	61.5288	-0.312161
Ciclev10007747m.g	scaffold_1:4234078-4236501	15.2945	12.3201	-0.311999
Ciclev10027730m.g	scaffold_8:2491365-2502341	3.10807	2.50403	-0.311768
Ciclev10010011m.g	scaffold_1:2941461-2943030	3.59533	2.89694	-0.311596
Ciclev10031661m.g	scaffold_4:20278483-20280732	7.24102	5.8347	-0.311533
Ciclev10033858m.g	scaffold_4:13998495-14001465	1.14681	0.924246	-0.311277
Ciclev10016635m.g	scaffold_2:390462-395075	21.4609	17.2967	-0.311217
Ciclev10030128m.g	scaffold_8:16422153-16433240	6.03341	4.86291	-0.311154
Ciclev10026263m.g	scaffold_7:17424162-17426071	60.7966	49.0103	-0.310907
Ciclev10000740m.g	scaffold_5:18156521-18160252	18.4803	14.8982	-0.310853
Ciclev10012455m.g	scaffold_6:20131173-20132496	21.3117	17.1809	-0.310842
Ciclev10016341m.g	scaffold_2:33274227-33276034	2.87256	2.31629	-0.310521
Ciclev10001246m.g	scaffold_5:38766602-38769204	8.46956	6.82993	-0.310416
Ciclev10023668m.g	scaffold_3:40774996-40777617	0.412414	0.332587	-0.310362
Ciclev10025933m.g	scaffold_7:4997088-4999784	175.531	141.56	-0.310312
Ciclev10011263m.g	scaffold_6:19055517-19058726	18.684	15.069	-0.310222
Ciclev10014769m.g	scaffold_2:6840742-6844063	77.5673	62.5606	-0.310194
Ciclev10032709m.g	scaffold_4:24157479-24159724	29.4291	23.7358	-0.310176
Ciclev10002314m.g	scaffold_5:40275057-40277774	0.176492	0.14235	-0.310164
Ciclev10026746m.g	scaffold_7:10698380-10700868	252.514	203.706	-0.309874
Ciclev10011109m.g	scaffold_6:19699519-19703543	16.1367	13.0182	-0.309815
Ciclev10026118m.g	scaffold_7:8913080-8915021	1.27466	1.02834	-0.3098
Ciclev10007297m.g	scaffold_1:12875667-12897805	35.0855	28.3122	-0.309448
Ciclev10011958m.g	scaffold_6:15712657-15716009	5.71246	4.61002	-0.30934
Ciclev10033912m.g	scaffold_4:18758378-18781489	0.0914615	0.0738142	-0.309267
Ciclev10012938m.g	scaffold_6:22083544-22084946	0.219198	0.176908	-0.309237
Ciclev10026152m.g,Ciclev10026215m.g	scaffold_7:9038417-9044438	13.3943	10.8105	-0.309186
Ciclev10026785m.g	scaffold_7:1272258-1274372	108.21	87.3556	-0.308856
Ciclev10004012m.g	scaffold_5:41218504-41219281	4.50605	3.63792	-0.308747
Ciclev10017199m.g	scaffold_2:23837167-23840360	0.182988	0.147744	-0.308654
Ciclev10005896m.g	scaffold_9:1976731-1979144	20.4749	16.5314	-0.308643
Ciclev10032672m.g	scaffold_4:9412863-9416686	22.2725	17.9849	-0.308474
Ciclev10011219m.g	scaffold_6:7968909-7971042	10.4996	8.4793	-0.308315
-	scaffold_1:8620951-8623079	10.6998	8.64129	-0.308263
Ciclev10028191m.g	scaffold_8:5664168-5670065	27.1822	21.953	-0.308249
Ciclev10016047m.g	scaffold_2:3846077-3850109	11.6008	9.37182	-0.307829
Ciclev10028513m.g,Ciclev10030133m.g	scaffold_8:1491361-1556980	6.78592	5.48221	-0.307787
Ciclev10011299m.g	scaffold_6:19388697-19391611	0.0794961	0.0642296	-0.307646
Ciclev10026687m.g	scaffold_7:3462352-3463444	163.334	131.974	-0.307575
Ciclev10021846m.g	scaffold_3:38332375-38335713	40.445	32.6817	-0.307478
Ciclev10000649m.g	scaffold_5:34121539-34126463	21.9679	17.753	-0.307331
Ciclev10014095m.g	scaffold_2:15692369-15706704	24.217	19.5713	-0.307282
Ciclev10021434m.g	scaffold_3:36730046-36743795	20.3641	16.4576	-0.307273
Ciclev10020672m.g	scaffold_3:35372249-35712253	0.197723	0.159826	-0.306972
Ciclev10028452m.g	scaffold_8:22674394-22676455	7.80384	6.30905	-0.306762
Ciclev10004297m.g	scaffold_9:1909018-1913742	2.91337	2.35602	-0.306335
Ciclev10017128m.g	scaffold_2:12374280-12376220	191.022	154.48	-0.306323
Ciclev10008276m.g	scaffold_1:1167554-1170565	105.346	85.1938	-0.306322
Ciclev10016225m.g	scaffold_2:33984111-33987376	15.5477	12.5745	-0.306206
-	scaffold_9:27947712-27948049	43.1889	34.9304	-0.306177

Ciclev10001424m.g	scaffold_5:29033626-29037975	5.20166	4.20758	-0.305982
Ciclev10002560m.g	scaffold_5:37676182-37679875	3.17552	2.56866	-0.305973
Ciclev10015112m.g	scaffold_2:32991364-32994574	12.1197	9.80647	-0.305546
Ciclev10006386m.g	scaffold_9:2900564-2901139	0.546653	0.442325	-0.30552
Ciclev10033807m.g	scaffold_4:7074803-7078715	4.88646	3.95409	-0.305443
Ciclev10008353m.g	scaffold_1:22386353-22389735	6.10061	4.9378	-0.305084
Ciclev10021958m.g	scaffold_3:6541867-6545524	5.5355	4.48046	-0.305068
Ciclev10024334m.g	scaffold_3:12805805-12807587	4.19542	3.39643	-0.304798
Ciclev10019808m.g	scaffold_3:40253150-40254806	8.98273	7.27243	-0.304715
Ciclev10005987m.g	scaffold_9:12381451-12382508	0.402803	0.326186	-0.304379
Ciclev10033070m.g	scaffold_4:1114644-1117928	58.4731	47.352	-0.304349
Ciclev10005091m.g	scaffold_9:15250698-15256773	26.5937	21.5371	-0.304259
Ciclev10004963m.g	scaffold_9:905622-911210	5.60856	4.54223	-0.304231
Ciclev10032856m.g,Ciclev10033560m.g	scaffold_4:23345065-23349566	9.65742	7.8223	-0.304046
Ciclev10032021m.g	scaffold_4:6297563-6301762	48.7233	39.4703	-0.303844
Ciclev10033354m.g	scaffold_4:632094-638772	7.59513	6.15329	-0.303717
Ciclev10022165m.g	scaffold_3:42180649-42183843	7.7734	6.29811	-0.303626
Ciclev10003667m.g	scaffold_5:6736304-6741789	7.54964	6.11699	-0.303586
Ciclev10003338m.g	scaffold_5:20388534-20389551	0.271616	0.220105	-0.303376
Ciclev10003120m.g	scaffold_5:36814201-36816256	0.206748	0.167565	-0.303147
Ciclev10019342m.g	scaffold_3:880710-890544	122.706	99.4599	-0.303014
Ciclev10005140m.g	scaffold_9:30846188-30849705	17.7897	14.4198	-0.302991
Ciclev10024691m.g	scaffold_7:1426465-1435078	19.8537	16.094	-0.302889
Ciclev10011677m.g	scaffold_6:327365-332748	21.8817	17.7403	-0.302696
Ciclev10019615m.g	scaffold_3:38116518-38126805	28.3787	23.0115	-0.302452
Ciclev10028781m.g	scaffold_8:10587797-10592154	53.2572	43.189	-0.30231
Ciclev10031713m.g	scaffold_4:21285061-21290391	16.8786	13.6888	-0.3022
Ciclev10022379m.g	scaffold_3:46373796-46377587	115.275	93.4909	-0.302188
Ciclev10020943m.g	scaffold_3:48640070-48641944	7.76023	6.2939	-0.302146
Ciclev10017569m.g	scaffold_2:28506098-28507480	1.40376	1.13852	-0.30214
Ciclev10032751m.g	scaffold_4:23725054-23726990	17.7565	14.4059	-0.301689
Ciclev10005661m.g	scaffold_9:10966420-10967873	0.742172	0.602178	-0.301564
Ciclev10019061m.g	scaffold_3:3561267-3564592	11.1792	9.07198	-0.301334
Ciclev10028931m.g	scaffold_8:998266-1001763	36.3043	29.4617	-0.301299
Ciclev10000643m.g	scaffold_5:24399708-24405331	45.5424	36.9595	-0.301264
Ciclev10012309m.g	scaffold_6:16986365-16997689	29.4704	23.9199	-0.301055
Ciclev10020804m.g	scaffold_3:6484725-6486543	6.23495	5.06074	-0.301031
Ciclev10018701m.g	scaffold_3:50613433-50627221	24.4571	19.8521	-0.300962
Ciclev10006054m.g	scaffold_9:10272161-10274584	1.29871	1.05423	-0.300901
Ciclev10003999m.g	scaffold_5:35938507-35945435	4.58759	3.72426	-0.300785
Ciclev10005036m.g	scaffold_9:31105157-31110317	59.4478	48.2642	-0.300672
Ciclev10001318m.g	scaffold_5:35521571-35526568	18.0406	14.6469	-0.300658
Ciclev10020874m.g	scaffold_3:18517468-18520307	0.862299	0.700112	-0.300603
Ciclev10009882m.g	scaffold_1:2011737-2014271	34.5365	28.0424	-0.300514
Ciclev10020662m.g	scaffold_3:48889812-48893678	30.0191	24.3746	-0.300501
Ciclev10031013m.g	scaffold_4:795216-799810	57.1838	46.4422	-0.300169
Ciclev10012897m.g	scaffold_6:18098919-18111094	19.1823	15.5793	-0.300145
Ciclev10009077m.g	scaffold_1:24200102-24206223	60.7514	49.3486	-0.299908
Ciclev10002952m.g	scaffold_5:43021169-43023077	40.0361	32.5226	-0.299858
Ciclev10004938m.g	scaffold_9:650076-654040	20.5441	16.6888	-0.299849
Ciclev10012703m.g	scaffold_6:17983936-17985312	27.3075	22.184	-0.299778
Ciclev10028906m.g	scaffold_8:975146-976388	1.11076	0.902408	-0.299689
Ciclev10003294m.g	scaffold_5:41347083-41349587	3.11807	2.53362	-0.299453
Ciclev10024430m.g	scaffold_3:6420380-6424022	68.3738	55.5646	-0.299278
Ciclev10022710m.g	scaffold_3:1184671-1187488	120.79	98.1671	-0.299192

Ciclev10016033m.g	scaffold_2:4074297-4079001	25.2884	20.5539	-0.299064
Ciclev10010530m.g	scaffold_1:22711667-22716034	42.8796	34.8528	-0.299014
Ciclev10015992m.g	scaffold_2:35259734-35263700	10.6406	8.64922	-0.298941
Ciclev10027767m.g	scaffold_8:693995-698113	0.85799	0.697469	-0.29883
Ciclev10029339m.g	scaffold_8:5037312-5037950	0.497964	0.404915	-0.298421
Ciclev10003992m.g	scaffold_5:20211283-20217483	0.296754	0.241306	-0.298402
Ciclev10002056m.g	scaffold_5:35449778-35453908	59.0785	48.0405	-0.29838
Ciclev10019819m.g	scaffold_3:6259922-6265711	40.0382	32.5633	-0.298127
Ciclev10012269m.g	scaffold_6:11505384-11512462	12.053	9.80408	-0.297939
Ciclev10000775m.g	scaffold_5:38559098-38562819	9.14462	7.439	-0.297815
Ciclev10011818m.g	scaffold_6:23586241-23589193	38.3365	31.1865	-0.297794
Ciclev10032629m.g	scaffold_4:2996224-2998198	148.928	121.156	-0.297744
Ciclev10028471m.g	scaffold_8:111796-114263	3.66068	2.97821	-0.297665
Ciclev10027227m.g	scaffold_7:15032982-15036326	0.141284	0.114953	-0.29755
Ciclev10025215m.g	scaffold_7:273450-278808	10.5175	8.5586	-0.297341
Ciclev10008754m.g	scaffold_1:28439801-28442008	110.619	90.0252	-0.297201
Ciclev10013128m.g	scaffold_6:22758026-22760875	115.408	93.9272	-0.297125
Ciclev10019314m.g	scaffold_3:1188545-1191618	7.24691	5.8981	-0.297117
Ciclev10000080m.g	scaffold_5:37544471-37560859	5.52068	4.4934	-0.297039
Ciclev10017281m.g	scaffold_2:10640189-10646119	39.7659	32.3788	-0.296483
Ciclev10026578m.g	scaffold_7:5503293-5507230	0.365628	0.297749	-0.296281
Ciclev10030854m.g	scaffold_4:651358-653455	2.77984	2.26391	-0.296185
Ciclev10026599m.g	scaffold_7:5763586-5768156	8.86962	7.22497	-0.29588
Ciclev10031081m.g	scaffold_4:22752863-22762487	14.2532	11.6113	-0.295757
Ciclev10029786m.g	scaffold_8:1949280-1951706	0.109499	0.0892056	-0.295707
Ciclev10008725m.g	scaffold_1:22552500-22555558	8.21797	6.69551	-0.295588
Ciclev10031151m.g	scaffold_4:18742364-18749147	13.2454	10.7928	-0.295423
Ciclev10032948m.g	scaffold_4:19927053-19931264	24.7303	20.1516	-0.295386
-	scaffold_3:431685-432174	12.1633	9.91166	-0.295333
Ciclev10016518m.g	scaffold_2:10521621-10523007	21.6106	17.6118	-0.295198
Ciclev10029261m.g	scaffold_8:6628106-6631194	39.4069	32.1155	-0.295178
Ciclev10000289m.g	scaffold_5:19183059-19192077	12.7535	10.394	-0.295145
Ciclev10031413m.g	scaffold_4:1012478-1015971	18.0598	14.7186	-0.295141
Ciclev10009155m.g,Ciclev10010190m.g	scaffold_1:27943662-27965514	25.5644	20.8363	-0.295036
Ciclev10032850m.g	scaffold_4:19222129-19223136	20.7725	16.9343	-0.294727
Ciclev10026742m.g	scaffold_7:5398600-5399282	2.79083	2.27541	-0.294568
Ciclev10031326m.g	scaffold_4:1531261-1535758	13.0901	10.6727	-0.294553
Ciclev10033891m.g	scaffold_4:14719959-14722082	0.270664	0.220697	-0.294435
Ciclev10005743m.g	scaffold_9:15114172-15116741	23.8171	19.4264	-0.293979
Ciclev10024343m.g	scaffold_3:714152-716354	1.09029	0.889337	-0.293916
Ciclev10008125m.g	scaffold_1:26385358-26387485	1.31959	1.07638	-0.293909
Ciclev10032624m.g	scaffold_4:25619922-25621380	91.6374	74.7548	-0.293771
Ciclev10018574m.g	scaffold_3:46143240-46147293	0.185112	0.151012	-0.293736
Ciclev10027025m.g	scaffold_7:16191640-16194242	1.58837	1.29579	-0.293718
Ciclev10021191m.g	scaffold_3:2102635-2106125	23.8933	19.4922	-0.293711
Ciclev10024248m.g	scaffold_3:7443493-7455910	3.02708	2.4697	-0.293592
Ciclev10028898m.g	scaffold_8:438273-441426	15.3994	12.5641	-0.293576
Ciclev10015272m.g	scaffold_2:8518854-8526597	60.9691	49.7461	-0.293495
Ciclev10028534m.g	scaffold_8:1316878-1320611	4.96486	4.05102	-0.293466
Ciclev10016169m.g	scaffold_2:34661891-34669768	22.563	18.4102	-0.293454
Ciclev10023660m.g	scaffold_3:43439731-43442964	7.89988	6.44617	-0.293389
Ciclev10002719m.g	scaffold_5:37444588-37447583	29.3382	23.9395	-0.293386
Ciclev10017146m.g	scaffold_2:2986242-2991381	48.4479	39.5349	-0.293309
Ciclev10021928m.g	scaffold_3:50045013-50052161	43.8527	35.7874	-0.293213
Ciclev10009485m.g	scaffold_1:1687893-1690412	11.7089	9.55582	-0.293157

Ciclev10027948m.g	scaffold_8:2056320-2061243	18.1922	14.8474	-0.293109
Ciclev10001459m.g	scaffold_5:34284004-34286922	0.085327	0.0696402	-0.293084
Ciclev10015980m.g	scaffold_2:104667-108211	95.1494	77.6714	-0.292811
Ciclev10022750m.g	scaffold_3:42672042-42676935	2230.44	1821.08	-0.292537
Ciclev10032815m.g	scaffold_4:13584975-13588353	46.2011	37.7222	-0.292515
Ciclev10020018m.g	scaffold_3:37499135-37505288	41.8103	34.1374	-0.292506
Ciclev10007467m.g	scaffold_1:17428189-17432539	0.0655049	0.053487	-0.292415
Ciclev10005192m.g	scaffold_9:31030856-31033493	56.6231	46.2354	-0.292392
Ciclev10003169m.g	scaffold_5:42066579-42069123	51.8788	42.3618	-0.292382
Ciclev10020154m.g	scaffold_3:42488523-42497198	13.7723	11.2459	-0.292372
Ciclev10009743m.g	scaffold_1:6655986-6657753	224.225	183.094	-0.292362
Ciclev10016894m.g	scaffold_2:11498829-11501800	460.725	376.224	-0.292315
Ciclev10017772m.g	scaffold_2:23815448-23818481	0.092788	0.0757699	-0.292313
Ciclev10032444m.g,Ciclev10033869m.g	scaffold_4:16079268-16089881	8.75269	7.14781	-0.292226
Ciclev10030758m.g	scaffold_4:9236548-9243065	18.6949	15.2682	-0.292116
Ciclev10018748m.g	scaffold_3:30784118-30802152	38.782	31.6734	-0.292115
Ciclev10016219m.g	scaffold_2:13270646-13281464	18.3544	14.9908	-0.292055
Ciclev10019016m.g	scaffold_3:49745052-49750265	4.24544	3.46763	-0.291962
Ciclev10029929m.g	scaffold_8:3257244-3394853	136.61	111.595	-0.2918
Ciclev10008153m.g	scaffold_1:25926884-25938245	49.602	40.53	-0.291409
Ciclev10020367m.g	scaffold_3:32895853-32900578	93.9849	76.7975	-0.291369
Ciclev10021599m.g	scaffold_3:40918147-40922656	24.3068	19.8624	-0.291318
Ciclev10032379m.g	scaffold_4:712740-715475	53.4256	43.6583	-0.291276
Ciclev10024085m.g	scaffold_3:39558394-39559825	0.13227	0.108104	-0.291067
Ciclev10027703m.g	scaffold_8:22445572-22451342	22.9505	18.7581	-0.291013
-	scaffold_7:4745337-4746941	11.8319	9.67089	-0.290961
Ciclev10022845m.g	scaffold_3:46317718-46320168	13.1833	10.7758	-0.290919
Ciclev10008228m.g	scaffold_1:20009949-20012784	5.6035	4.58048	-0.290831
Ciclev10028340m.g	scaffold_8:4911564-4916480	43.4695	35.5337	-0.290813
Ciclev10011081m.g	scaffold_6:16998340-17001430	0.142742	0.11669	-0.290727
Ciclev10002973m.g	scaffold_5:27598225-27600485	181.384	148.297	-0.290557
Ciclev10006013m.g	scaffold_9:2165865-2170588	11.292	9.2324	-0.290524
Ciclev10028892m.g	scaffold_8:6893731-6897309	101.178	82.725	-0.290493
Ciclev10032228m.g	scaffold_4:21793764-21796262	39.5542	32.344	-0.290333
Ciclev10021439m.g	scaffold_3:43258338-43260639	14.4916	11.8536	-0.289888
-	scaffold_2:35918844-35919989	133.827	109.474	-0.289785
Ciclev10032445m.g	scaffold_4:3525465-3529256	17.3587	14.1999	-0.289779
Ciclev10009759m.g	scaffold_1:28442271-28444777	276.421	226.122	-0.289763
Ciclev10010332m.g	scaffold_1:21730278-21730899	0.479059	0.391984	-0.28941
Ciclev10026933m.g	scaffold_7:8911099-8912352	0.182144	0.149058	-0.289202
Ciclev10029260m.g	scaffold_8:6065091-6068908	148.614	121.619	-0.289196
Ciclev10012223m.g	scaffold_6:18461676-18466491	54.6287	44.7146	-0.288911
Ciclev10017589m.g	scaffold_2:29110324-29112979	1.77909	1.45626	-0.288874
Ciclev10007927m.g	scaffold_1:23094967-23098030	33.0891	27.0863	-0.288792
Ciclev10011191m.g	scaffold_6:15170699-15173761	0.827661	0.677554	-0.288703
Ciclev10001198m.g	scaffold_5:5972891-5976639	22.7456	18.6207	-0.288677
Ciclev10022078m.g	scaffold_3:41736667-41739727	8.43143	6.90316	-0.288521
Ciclev10028968m.g	scaffold_8:23614089-23617076	2.97643	2.43721	-0.288352
Ciclev10032000m.g	scaffold_4:20947001-20950286	10.6265	8.70196	-0.288259
Ciclev10009560m.g	scaffold_1:4336653-4340473	55.5642	45.5027	-0.288204
Ciclev10015731m.g	scaffold_2:27084176-27089027	48.1165	39.4039	-0.288194
Ciclev10016847m.g	scaffold_2:22620376-22622795	25.1561	20.6017	-0.288146
Ciclev10004119m.g	scaffold_9:11441031-11462053	1.45254	1.18961	-0.288082
Ciclev10017095m.g,Ciclev10017323m.g	scaffold_2:32749007-32750939	164.532	134.759	-0.287984

Ciclev10029465m.g	scaffold_8:954620-956247	182.554	149.529	-0.287899
Ciclev10019985m.g	scaffold_3:5297682-5300987	4.23674	3.47054	-0.287796
Ciclev10022313m.g	scaffold_3:6100355-6101497	935.177	766.081	-0.287742
Ciclev10012016m.g	scaffold_6:23786914-23789562	39.7493	32.5637	-0.287666
Ciclev10007340m.g	scaffold_1:3230046-3235228	8.69559	7.12393	-0.287612
Ciclev10029273m.g	scaffold_8:24809582-24811672	0.593716	0.48641	-0.2876
Ciclev10018416m.g	scaffold_816:2164-5140	41.0572	33.6402	-0.287447
Ciclev10029572m.g,Ciclev10030226m.g	scaffold_8:9025006-9167585	34.5258	28.2915	-0.287308
Ciclev10020021m.g	scaffold_3:27392620-27394211	0.134983	0.110627	-0.287073
Ciclev10004485m.g	scaffold_9:3189774-3195453	1.14917	0.941975	-0.286828
Ciclev10027001m.g	scaffold_7:443590-448415	13.9576	11.4411	-0.28682
Ciclev10004488m.g	scaffold_9:87544-95218	4.07591	3.34124	-0.286739
Ciclev10015037m.g	scaffold_2:20031904-20042011	9.04136	7.41174	-0.286728
Ciclev10002759m.g	scaffold_5:37158688-37161113	38.9108	31.8977	-0.28672
Ciclev10027826m.g	scaffold_8:12480170-12487647	12.2171	10.0155	-0.286663
Ciclev10023989m.g	scaffold_3:45317361-45318298	5.57819	4.5738	-0.286403
Ciclev10003755m.g	scaffold_5:19400140-19406765	24.6179	20.1869	-0.286287
Ciclev10031954m.g	scaffold_4:21898103-21903480	9.95816	8.16703	-0.286068
Ciclev10008629m.g	scaffold_1:21446505-21451731	20.8912	17.1347	-0.285973
Ciclev10026852m.g	scaffold_7:17420860-17422787	244.883	200.865	-0.285872
Ciclev10019153m.g	scaffold_3:16194519-16198109	0.746347	0.612214	-0.285812
Ciclev10031477m.g	scaffold_4:8364483-8369840	44.7811	36.7388	-0.285585
Ciclev10021529m.g	scaffold_3:4670535-4674390	133.963	109.933	-0.285205
Ciclev10008803m.g	scaffold_1:28388949-28392647	8.72787	7.16244	-0.285179
Ciclev10016466m.g	scaffold_2:16392850-16394263	121.13	99.4039	-0.285177
Ciclev10020250m.g	scaffold_3:20599780-20601125	1.73627	1.42524	-0.28478
Ciclev10005758m.g	scaffold_9:15611277-15614399	7.98274	6.5529	-0.28475
Ciclev10007504m.g	scaffold_1:22876161-22881016	9.06323	7.43987	-0.284748
Ciclev10007449m.g	scaffold_1:5120900-5128999	36.1059	29.6392	-0.284729
Ciclev10010998m.g	scaffold_6:22399071-22414452	32.4016	26.605	-0.284367
Ciclev10022658m.g	scaffold_3:602712-605468	23.845	19.5795	-0.284345
Ciclev10025935m.g	scaffold_7:2390009-2391658	159.495	130.974	-0.284232
Ciclev10026308m.g	scaffold_7:1264964-1266960	489.386	401.876	-0.284222
Ciclev10019830m.g	scaffold_3:45090811-45097273	80.0131	65.7121	-0.284077
Ciclev10016302m.g	scaffold_2:36078500-36081245	342.608	281.383	-0.284025
-	scaffold_3:27208499-27209654	311.755	256.061	-0.283925
Ciclev10022484m.g	scaffold_3:40152132-40155842	5.931	4.87178	-0.283826
-	scaffold_2:9467516-9470154	13.3041	10.9282	-0.283818
Ciclev10011412m.g	scaffold_6:10810244-10814702	47.4555	38.9825	-0.283747
Ciclev10009944m.g	scaffold_1:7737684-7741202	24.5927	20.2055	-0.283476
Ciclev10002021m.g	scaffold_5:19581294-19585262	8.67654	7.12891	-0.283438
Ciclev10002683m.g	scaffold_5:41972942-41973902	138.342	113.682	-0.283244
Ciclev10026080m.g	scaffold_7:10497023-10499186	29.3224	24.0983	-0.28307
Ciclev10022331m.g	scaffold_3:38529596-38531433	17.7245	14.5719	-0.282562
Ciclev10016808m.g	scaffold_2:7208671-7212343	91.594	75.3039	-0.282527
Ciclev10019710m.g	scaffold_3:622221-626208	16.0387	13.1867	-0.282478
Ciclev10024413m.g	scaffold_3:43350018-43353840	25.5396	20.9985	-0.282452
-	scaffold_9:22161913-22162106	186.585	153.41	-0.282447
Ciclev10024633m.g	scaffold_3:27179650-27180616	27.4875	22.6015	-0.282362
Ciclev10002109m.g	scaffold_5:37267094-37269707	56.8009	46.7085	-0.282228
Ciclev10020944m.g	scaffold_3:1331108-1333730	102.519	84.3038	-0.282224
Ciclev10002694m.g	scaffold_5:30751920-30752967	0.417432	0.343277	-0.282169
Ciclev10027052m.g	scaffold_7:17225399-17226338	0.47908	0.393996	-0.282088
Ciclev10007971m.g	scaffold_1:27802689-27806822	7.34388	6.04024	-0.281938
Ciclev10020958m.g	scaffold_3:363597-364689	0.171323	0.140932	-0.281725

Ciclev10029201m.g	scaffold_8:2061651-2063506	40.3778	33.2167	-0.281652
Ciclev10002669m.g	scaffold_5:41208819-41211050	131.037	107.818	-0.281379
Ciclev10032499m.g	scaffold_4:1097135-1099047	58.9289	48.4893	-0.281311
Ciclev10015334m.g	scaffold_2:12893460-12896650	5.44577	4.48131	-0.281214
Ciclev10031415m.g	scaffold_4:18800789-18805604	33.2123	27.3309	-0.281185
Ciclev10014775m.g	scaffold_2:35726267-35730801	55.1995	45.4247	-0.28118
Ciclev10006078m.g	scaffold_9:627372-629617	43.0463	35.4276	-0.281017
Ciclev10019812m.g	scaffold_3:43163059-43169327	10.5344	8.67124	-0.280796
Ciclev10022993m.g	scaffold_3:42229223-42233850	361.235	297.368	-0.280689
Ciclev10030860m.g	scaffold_4:6160861-6164185	9.89982	8.15029	-0.280551
Ciclev10032005m.g	scaffold_4:17799468-17803894	13.947	11.4827	-0.280496
Ciclev10001384m.g	scaffold_5:41352237-41355326	104.033	85.653	-0.28046
Ciclev10031383m.g	scaffold_4:3843377-3848372	6.35864	5.23663	-0.280078
Ciclev10014912m.g	scaffold_2:33276749-33286428	21.0276	17.3204	-0.279812
Ciclev10013130m.g	scaffold_6:22925114-22925778	7.41938	6.11167	-0.279731
-	scaffold_1:20432009-20432258	29.4182	24.2339	-0.279682
Ciclev10012135m.g	scaffold_6:23951226-23954781	26.7556	22.0406	-0.279674
Ciclev10010423m.g	scaffold_1:26729765-26744426	6.5	5.35547	-0.279426
Ciclev10024876m.g	scaffold_7:504676-511474	31.9711	26.3448	-0.27925
Ciclev10021880m.g	scaffold_3:3192158-3195638	21.1795	17.4525	-0.279235
Ciclev10031198m.g	scaffold_4:21841320-21845493	66.0725	54.4463	-0.279216
Ciclev10020951m.g	scaffold_3:44353874-44358046	28.8623	23.7879	-0.278957
Ciclev10027417m.g	scaffold_7:8384758-8387191	0.315041	0.259657	-0.278932
Ciclev10032334m.g	scaffold_4:2365141-2368178	13.524	11.1505	-0.278417
Ciclev10000713m.g	scaffold_5:5309894-5315706	38.0313	31.3579	-0.278356
Ciclev10000637m.g	scaffold_5:5087959-5093839	18.3966	15.1695	-0.278273
Ciclev10012993m.g	scaffold_6:309808-311028	9.04897	7.46185	-0.27822
Ciclev10002050m.g	scaffold_5:19780297-19783698	1.69285	1.39601	-0.278143
Ciclev10014504m.g	scaffold_2:32352768-32363488	69.0019	56.9112	-0.277924
Ciclev10029012m.g	scaffold_8:21272855-21274555	4.07579	3.36195	-0.277782
Ciclev10027189m.g	scaffold_7:1085044-1086475	0.21225	0.175099	-0.277595
Ciclev10009344m.g	scaffold_1:25972368-25974724	27.3671	22.5815	-0.277303
Ciclev10019199m.g	scaffold_3:49549859-49552300	8.48215	6.99891	-0.277298
Ciclev10017915m.g	scaffold_2:27354207-27356162	12.6205	10.4143	-0.27721
Ciclev10007392m.g	scaffold_1:17492478-17500476	47.408	39.121	-0.277186
Ciclev10029827m.g	scaffold_8:3578731-3582321	49.9784	41.2436	-0.277133
Ciclev10011105m.g	scaffold_6:16159559-16167038	14.8279	12.237	-0.277069
Ciclev10019439m.g	scaffold_3:49473118-49478840	42.5993	35.1618	-0.276822
Ciclev10020117m.g	scaffold_3:46755615-46761709	18.0532	14.9014	-0.2768
Ciclev10004681m.g	scaffold_9:21959071-21965155	0.182797	0.150903	-0.276621
Ciclev10025966m.g	scaffold_7:3073051-3078885	36.4877	30.125	-0.276452
Ciclev10019077m.g	scaffold_3:23394730-23406560	15.7374	12.9931	-0.276448
Ciclev10032818m.g	scaffold_4:20512736-20514546	1.45061	1.19775	-0.276334
Ciclev10008143m.g	scaffold_1:845483-849143	21.3087	17.5955	-0.276236
Ciclev10028265m.g	scaffold_8:558873-560883	0.599617	0.495229	-0.275944
Ciclev10013742m.g,Ciclev10013855m.g	scaffold_6:17999855-18000810	251.592	207.847	-0.275562
Ciclev10001582m.g,Ciclev10003487m.g	scaffold_5:24058278-24064539	17.1159	14.1425	-0.2753
Ciclev10001152m.g	scaffold_5:40219020-40224336	20.106	16.6135	-0.27527
Ciclev10025852m.g	scaffold_7:209373-211666	36.3009	29.997	-0.275186
Ciclev10012723m.g	scaffold_6:13032632-13037086	87.8963	72.6355	-0.275127
Ciclev10003668m.g,Ciclev10003825m.g	scaffold_5:2932583-2963992	25.2957	20.904	-0.275111
Ciclev10008258m.g	scaffold_1:27503995-27506104	2.72941	2.25579	-0.274955
Ciclev10008687m.g	scaffold_1:24523399-24526186	101.739	84.0932	-0.274814

Ciclev10002453m.g	scaffold_5:38982704-38986196	41.2313	34.0822	-0.274718
Ciclev10020850m.g	scaffold_3:20757274-20762831	75.7153	62.5946	-0.274547
Ciclev10016918m.g,Ciclev10017041m.g	scaffold_2:27393144-27417687	273.225	225.888	-0.274484
Ciclev10019959m.g	scaffold_3:44125298-44128833	18.9993	15.7092	-0.274332
Ciclev10021674m.g	scaffold_3:8812688-8816485	41.239	34.1017	-0.274167
Ciclev10020595m.g	scaffold_3:48951764-48956546	19.4229	16.0616	-0.274143
Ciclev10007381m.g	scaffold_1:24022020-24025656	0.106843	0.0883547	-0.274115
Ciclev10002378m.g	scaffold_5:20818485-20821238	398.62	329.653	-0.274067
Ciclev10019942m.g	scaffold_3:47687295-47692118	7.26919	6.01154	-0.27406
Ciclev10031209m.g	scaffold_4:25291121-25295311	20.2826	16.7795	-0.273543
Ciclev10012638m.g	scaffold_6:21267036-21270592	205.173	169.738	-0.273533
Ciclev10012072m.g,Ciclev10012463m.g,Ciclev10013865m.g	scaffold_6:18694429-18718926	302.552	250.307	-0.273483
Ciclev10027790m.g	scaffold_8:21953128-21960709	20.4724	16.9383	-0.273394
Ciclev10000585m.g	scaffold_5:17906312-17911447	73.5241	60.8326	-0.273372
Ciclev10011444m.g	scaffold_6:25184098-25188972	12.5943	10.4208	-0.273309
Ciclev10020694m.g	scaffold_3:49521120-49522813	0.16385	0.135576	-0.273273
Ciclev10028827m.g	scaffold_8:2066623-2070221	29.8658	24.7137	-0.273183
Ciclev10022585m.g	scaffold_3:35024362-35026113	9.644	7.98042	-0.273168
Ciclev10009241m.g	scaffold_1:3709735-3711613	14.7586	12.2131	-0.273128
Ciclev10008843m.g	scaffold_1:26404749-26409802	7.76462	6.42558	-0.273089
Ciclev10019080m.g	scaffold_3:18902661-18909071	30.9811	25.6457	-0.272671
Ciclev10032166m.g	scaffold_4:16062725-16065983	15.2101	12.5925	-0.272468
Ciclev10008946m.g	scaffold_1:2652646-2658693	287.659	238.164	-0.272404
Ciclev10001488m.g	scaffold_5:34695462-34698892	38.5893	31.9517	-0.272308
Ciclev10028018m.g	scaffold_8:17659924-17664551	3.26374	2.70245	-0.272258
Ciclev10024750m.g	scaffold_7:9008017-9014427	10.5027	8.6982	-0.271978
Ciclev10013496m.g	scaffold_6:15046031-15048058	0.113257	0.0937983	-0.271963
Ciclev10031381m.g	scaffold_4:22266712-22272740	716.032	593.012	-0.271962
Ciclev10021011m.g,Ciclev10023168m.g	scaffold_3:15993929-16000998	12.8242	10.6209	-0.271958
Ciclev10003057m.g	scaffold_5:39402262-39404061	14.8449	12.2961	-0.271765
Ciclev10016719m.g	scaffold_2:7989391-7991352	63.0857	52.2592	-0.271627
Ciclev10015477m.g	scaffold_2:33925533-33930014	8.73457	7.23618	-0.271508
Ciclev10018836m.g	scaffold_3:45412306-45417781	44.506	36.8732	-0.271425
Ciclev10011725m.g	scaffold_6:21704365-21710334	11.8287	9.80084	-0.27132
Ciclev10008866m.g	scaffold_1:5561613-5563736	23.2008	19.2233	-0.271317
Ciclev10029624m.g	scaffold_8:18856286-18858746	22.268	18.452	-0.271196
Ciclev10024775m.g	scaffold_7:19299710-19305869	6.36427	5.27388	-0.271131
Ciclev10028776m.g	scaffold_8:19639125-19641071	0.318989	0.264345	-0.271086
Ciclev10017106m.g	scaffold_2:33791774-33792471	232.615	192.812	-0.270752
Ciclev10008155m.g	scaffold_1:24248880-24257969	44.2015	36.6425	-0.270578
Ciclev10009395m.g	scaffold_1:25761569-25764926	65.8333	54.5782	-0.270493
Ciclev10013485m.g	scaffold_6:12469610-12471722	0.0939679	0.0779043	-0.270466
Ciclev10024842m.g,Ciclev10027211m.g,Ciclev10027317m.g	scaffold_7:11850299-12160617	6.67581	5.53493	-0.270378
Ciclev10002001m.g	scaffold_5:31873014-31876484	231.131	191.643	-0.270291
Ciclev10013003m.g	scaffold_6:19647674-19649777	82.9797	68.8058	-0.270229
Ciclev10032531m.g	scaffold_4:24854225-24857409	25.5085	21.152	-0.270179
Ciclev10011517m.g	scaffold_6:16359096-16369830	37.8937	31.423	-0.270138
Ciclev10005917m.g	scaffold_9:14857969-14859951	108.028	89.5938	-0.269939
Ciclev10023194m.g	scaffold_3:32063429-32064847	235.117	195	-0.269907
Ciclev10017245m.g	scaffold_2:23622268-23622607	67.988	56.391	-0.269816

Ciclev10018406m.g	scaffold_2:7273711-7336406	0.612009	0.507681	-0.269631
Ciclev10021569m.g	scaffold_3:5420052-5424166	70.4186	58.4224	-0.269435
Ciclev10009806m.g	scaffold_1:24228789-24231495	430.128	356.855	-0.269428
Ciclev10019437m.g	scaffold_3:45328577-45332337	42.8805	35.5819	-0.269179
Ciclev10022476m.g	scaffold_3:5874123-5878839	0.421984	0.350176	-0.269108
Ciclev10011512m.g	scaffold_6:10568125-10572710	5.94194	4.93082	-0.269105
Ciclev10033798m.g	scaffold_4:156887-160109	0.153276	0.127195	-0.269086
Ciclev10026697m.g	scaffold_7:9343804-9346550	19.3331	16.0439	-0.269053
Ciclev10019825m.g	scaffold_3:48944260-48947832	23.07	19.1453	-0.269023
Ciclev10019519m.g	scaffold_3:5593677-5600600	21.6379	17.9571	-0.269005
Ciclev10002272m.g	scaffold_5:39163271-39168399	64.1537	53.246	-0.26886
Ciclev10009916m.g	scaffold_1:28119678-28120450	42.681	35.4308	-0.268591
Ciclev10001866m.g	scaffold_5:13779700-13783960	42.0978	34.9478	-0.268541
Ciclev10014554m.g	scaffold_2:7507436-7569782	0.097975	0.081352	-0.268235
Ciclev10007267m.g	scaffold_1:2971709-2978294	4.41173	3.66328	-0.268207
Ciclev10015304m.g	scaffold_2:28797761-28800039	0.139345	0.115714	-0.26809
Ciclev10020455m.g	scaffold_3:39854715-39858576	9.1978	7.63845	-0.268009
Ciclev10012381m.g	scaffold_6:14274325-14280737	94.233	78.2592	-0.267972
Ciclev10030591m.g	scaffold_4:19254047-19268079	7.57653	6.29232	-0.267946
Ciclev10007272m.g	scaffold_1:20874960-20884864	8.3534	6.93757	-0.267934
Ciclev10019948m.g	scaffold_3:10359412-10365180	8.58599	7.13098	-0.267882
Ciclev10006499m.g	scaffold_9:993822-994323	1549.53	1287.04	-0.267767
Ciclev10023789m.g	scaffold_3:25445567-25448031	0.143982	0.119597	-0.267708
Ciclev10017186m.g	scaffold_2:8060849-8062464	80.6323	66.9776	-0.267679
Ciclev10015502m.g	scaffold_2:25535583-25538608	69.8745	58.0419	-0.267672
Ciclev10006189m.g	scaffold_9:310746-314283	17.4835	14.5258	-0.267373
Ciclev10025963m.g	scaffold_7:2274067-2279911	46.8892	38.959	-0.267298
Ciclev10026911m.g	scaffold_7:13777727-13778471	3.02149	2.51071	-0.267168
Ciclev10009263m.g	scaffold_1:14702051-14706757	23.594	19.6069	-0.26706
Ciclev10001967m.g	scaffold_5:25978504-25981882	12.5162	10.4011	-0.267059
Ciclev10001386m.g	scaffold_5:40912317-40927246	20.124	16.7234	-0.267055
Ciclev10014387m.g	scaffold_2:30693645-30698460	5.8993	4.90243	-0.267046
Ciclev10014013m.g	scaffold_2:30787786-30807383	8.61527	7.16058	-0.266818
Ciclev10002048m.g	scaffold_5:28539341-28801354	19.0116	15.8019	-0.266785
Ciclev10016464m.g	scaffold_2:34939400-34941396	45.5093	37.8288	-0.266678
Ciclev10018398m.g	scaffold_2:6942325-6945068	387.69	322.289	-0.266551
Ciclev10032303m.g	scaffold_4:25044516-25047286	15.4472	12.8419	-0.266477
Ciclev10014201m.g	scaffold_2:23150753-23159075	13.2186	10.9911	-0.26623
Ciclev10005361m.g	scaffold_9:4278945-4282762	70.8638	58.9228	-0.266225
Ciclev10026145m.g	scaffold_7:3415155-3421947	14.6305	12.1671	-0.265995
Ciclev10021865m.g	scaffold_3:49088507-49090570	10.6185	8.83095	-0.265944
-	scaffold_3:21536332-21537446	60.3645	50.2032	-0.265921
Ciclev10021541m.g	scaffold_3:44371080-44373131	5.23535	4.35532	-0.265508
Ciclev10018309m.g	scaffold_2:32708394-32714688	19.4751	16.2016	-0.265491
Ciclev10020430m.g	scaffold_3:42810728-42816514	27.9597	23.2657	-0.265144
Ciclev10022433m.g	scaffold_3:21483184-21484454	6.03091	5.01843	-0.265141
-	scaffold_6:12475117-12475849	79.7482	66.3623	-0.265088
Ciclev10015232m.g	scaffold_2:34139534-34145605	75.8642	63.1453	-0.264744
Ciclev10008981m.g	scaffold_1:13123279-13126225	121.554	101.177	-0.264709
Ciclev10011354m.g	scaffold_6:16698775-16701800	0.940287	0.78268	-0.264678
Ciclev10004720m.g	scaffold_9:28078769-28081418	0.117486	0.0977948	-0.264655
Ciclev10014323m.g	scaffold_2:29353053-29360609	22.1519	18.4394	-0.264638
Ciclev10009329m.g	scaffold_1:23480029-23483437	3.85126	3.20622	-0.264458
Ciclev10012932m.g,Ciclev10013818m.g	scaffold_6:20195909-20200196	48.1932	40.1216	-0.264451
Ciclev10009412m.g	scaffold_1:23809095-23813097	52.5985	43.7897	-0.264429

Ciclev10020809m.g	scaffold_3:33388539-33592383	1.33902	1.1148	-0.264384
Ciclev10028617m.g	scaffold_8:18043132-18047093	14.9833	12.4755	-0.264257
Ciclev10019208m.g	scaffold_3:32716415-32805364	0.101292	0.0843396	-0.264233
Ciclev10030795m.g	scaffold_4:6188061-6196258	9.42092	7.84436	-0.264212
Ciclev10001828m.g	scaffold_5:38879719-38882455	66.1577	55.1003	-0.263849
Ciclev10030666m.g	scaffold_4:18437253-18445158	7.01359	5.8419	-0.263715
Ciclev10028852m.g	scaffold_8:1220779-1222036	19.9279	16.6002	-0.263591
Ciclev10003901m.g	scaffold_5:37866132-37869674	24.0465	20.032	-0.263525
Ciclev10017296m.g	scaffold_2:32252308-32254004	51.2566	42.7012	-0.263462
Ciclev10032902m.g	scaffold_4:19467668-19468803	298.054	248.321	-0.263367
Ciclev10009814m.g	scaffold_1:12500802-12501644	12.1005	10.0818	-0.263315
Ciclev10020205m.g	scaffold_3:3767474-3771747	53.8031	44.8297	-0.263233
Ciclev10002350m.g	scaffold_5:41252803-41257611	0.622037	0.518381	-0.262989
Ciclev10011742m.g	scaffold_6:12594909-12596760	37.463	31.2209	-0.262955
Ciclev10032359m.g	scaffold_4:6527143-6529959	5.86856	4.89113	-0.262839
Ciclev10019120m.g	scaffold_3:30474462-30483527	18.0142	15.0197	-0.262281
Ciclev10025785m.g	scaffold_7:3217136-3218728	11.7374	9.78626	-0.26228
Ciclev10033438m.g	scaffold_4:19171922-19173038	0.185414	0.154606	-0.262154
Ciclev10023968m.g	scaffold_3:8383302-8410026	13.5126	11.2674	-0.262149
Ciclev10023409m.g	scaffold_3:48400224-48402500	0.0865428	0.0721837	-0.261741
Ciclev10003088m.g	scaffold_5:41715561-41718220	104.999	87.584	-0.261643
Ciclev10021724m.g	scaffold_3:379831-381992	200.642	167.366	-0.261612
Ciclev10026489m.g	scaffold_7:17868382-17872357	28.5704	23.8405	-0.261105
Ciclev10003246m.g	scaffold_5:9125380-9131852	0.127072	0.106045	-0.260969
Ciclev10018878m.g	scaffold_3:1801952-1805043	2.20224	1.83784	-0.26096
Ciclev10020042m.g	scaffold_3:6711278-6716416	49.8881	41.6338	-0.260941
Ciclev10015905m.g	scaffold_2:26595971-26598955	65.2635	54.4692	-0.260836
Ciclev10015246m.g	scaffold_2:33211427-33212854	0.318331	0.265698	-0.260743
Ciclev10022079m.g	scaffold_3:41409392-41412064	63.3215	52.856	-0.260629
Ciclev10021083m.g	scaffold_3:40130520-40133133	51.728	43.1817	-0.260523
Ciclev10011935m.g	scaffold_6:19885485-19888972	65.127	54.3687	-0.26048
Ciclev10033104m.g	scaffold_4:14158631-14161064	27.4351	22.9033	-0.260465
Ciclev10008139m.g	scaffold_1:6827020-6832187	17.9533	14.9878	-0.260459
Ciclev10016908m.g	scaffold_2:22667396-22670152	55.3521	46.2136	-0.26032
Ciclev10028014m.g	scaffold_8:8656194-8658247	0.341852	0.285445	-0.260156
Ciclev10008761m.g	scaffold_1:25618962-25621811	20.2502	16.9093	-0.260116
Ciclev10014484m.g	scaffold_2:32824858-32828812	6.62963	5.53619	-0.260035
Ciclev10004933m.g	scaffold_9:22740207-22743885	18.9615	15.8344	-0.260011
Ciclev10019073m.g	scaffold_3:46833041-46836780	15.1468	12.6503	-0.259841
Ciclev10011643m.g	scaffold_6:11401277-11405083	5.09272	4.25343	-0.259811
Ciclev10031847m.g	scaffold_4:2543855-2546646	60.7404	50.7331	-0.259728
Ciclev10030862m.g	scaffold_4:25270619-25274515	15.7099	13.1255	-0.259301
Ciclev10014688m.g	scaffold_2:26384891-26388518	35.5359	29.6939	-0.259111
Ciclev10007979m.g	scaffold_1:22074141-22077662	16.6594	13.9223	-0.258944
Ciclev10021127m.g	scaffold_3:6577359-6582553	960.595	802.781	-0.258922
Ciclev10027201m.g	scaffold_7:6682484-6683930	0.21895	0.182988	-0.25885
Ciclev10001023m.g	scaffold_5:36743352-36746199	31.0793	25.9759	-0.258782
Ciclev10032529m.g	scaffold_4:18429353-18432003	144.974	121.174	-0.258718
Ciclev10021425m.g	scaffold_3:37597623-37600961	143.845	120.235	-0.258666
Ciclev10002525m.g	scaffold_5:35307568-35311024	25.8158	21.5801	-0.258555
Ciclev10009502m.g	scaffold_1:340088-343187	14.7725	12.3487	-0.258551
Ciclev10007706m.g	scaffold_1:1747313-1750072	14.2553	11.9169	-0.258492
-	scaffold_2:24401745-24433873	54.8977	45.8941	-0.258437
Ciclev10015410m.g	scaffold_2:7368568-7371271	6.37374	5.3292	-0.258221
Ciclev10027956m.g	scaffold_8:15384926-15400040	86.2174	72.0895	-0.25819
Ciclev10028186m.g	scaffold_8:2427000-2430311	54.4086	45.4942	-0.258153

Ciclev10017777m.g	scaffold_2:5401277-5408814	0.381352	0.318871	-0.258151
Ciclev10026245m.g	scaffold_7:6760310-6763833	17.0023	14.218	-0.258012
Ciclev10032798m.g	scaffold_4:21113893-21115825	8.69133	7.26843	-0.257935
Ciclev10016522m.g	scaffold_2:13843238-13847481	95.6945	80.0309	-0.257879
Ciclev10026260m.g	scaffold_7:14309893-14311942	89.8405	75.1359	-0.257863
Ciclev10008755m.g	scaffold_1:24426759-24434342	9.64771	8.06876	-0.257839
Ciclev10020704m.g	scaffold_3:48221723-48226897	99.1522	82.9279	-0.257788
Ciclev10010274m.g	scaffold_1:4411736-4415297	0.113494	0.0949245	-0.257763
Ciclev10033281m.g	scaffold_4:2787966-2789736	303.692	254.032	-0.2576
Ciclev10030592m.g	scaffold_4:20015329-20030390	7.6336	6.38628	-0.257388
Ciclev10022317m.g	scaffold_3:44036306-44038264	14.0255	11.7374	-0.256946
Ciclev10028302m.g	scaffold_8:6276517-6280582	18.8793	15.801	-0.256794
Ciclev10031365m.g	scaffold_4:2506550-2510488	47.72	39.9433	-0.256641
Ciclev10007299m.g	scaffold_1:7027968-7037974	18.6404	15.6035	-0.256566
-	scaffold_5:1278349-1280704	709.291	593.737	-0.256553
Ciclev10004440m.g	scaffold_9:3313826-3321104	42.7225	35.7661	-0.256404
Ciclev10001406m.g	scaffold_5:17543856-17545059	2.24367	1.87868	-0.256136
Ciclev10016645m.g	scaffold_2:29701244-29703374	75.3845	63.1231	-0.2561
Ciclev10005357m.g	scaffold_9:4080934-4084753	4.90073	4.1043	-0.255861
Ciclev10002366m.g	scaffold_5:19775138-19779139	0.697212	0.58393	-0.255802
-	scaffold_3:48346838-48348092	139.219	116.63	-0.255412
Ciclev10031035m.g	scaffold_4:7094750-7107350	20.0005	16.7559	-0.255367
Ciclev10009532m.g	scaffold_1:27145165-27146787	2.10761	1.76581	-0.255278
Ciclev10031322m.g	scaffold_4:19542041-19547598	8.15395	6.83195	-0.255202
Ciclev10015063m.g	scaffold_2:33746687-33749902	5.22174	4.37533	-0.255139
Ciclev10002899m.g	scaffold_5:37447648-37449498	133.032	111.48	-0.254984
Ciclev10014490m.g	scaffold_2:23012305-23018997	12.3372	10.3387	-0.254967
Ciclev10029971m.g	scaffold_8:10471533-10472154	0.293226	0.245759	-0.254771
Ciclev10004947m.g	scaffold_9:136879-139697	19.5496	16.3863	-0.25465
Ciclev10029016m.g	scaffold_8:2627267-2630058	102.525	85.9416	-0.254543
Ciclev10004754m.g	scaffold_9:10280259-10286861	17.0796	14.3182	-0.254424
Ciclev10011607m.g	scaffold_6:18138304-18140393	0.161511	0.1354	-0.254398
Ciclev10003151m.g	scaffold_5:27290321-27292059	10.0524	8.42819	-0.254241
Ciclev10016575m.g	scaffold_2:36331572-36338458	11.6077	9.73307	-0.254122
Ciclev10011187m.g	scaffold_6:23195663-23202728	13.6104	11.4124	-0.254105
Ciclev10030217m.g	scaffold_8:3984384-3985368	0.215224	0.180472	-0.254066
Ciclev10004126m.g	scaffold_9:328323-340215	0.0501933	0.0420903	-0.254007
-	scaffold_3:4729347-4729527	514.257	431.26	-0.253934
Ciclev10024667m.g	scaffold_3:32644486-32645513	0.398109	0.333868	-0.253889
Ciclev10022332m.g	scaffold_3:28408854-28413893	28.2585	23.701	-0.253734
Ciclev10017154m.g	scaffold_2:14275513-14279686	61.2304	51.3595	-0.253616
Ciclev10013279m.g	scaffold_6:9261148-9263251	145.277	121.891	-0.253214
Ciclev10027481m.g	scaffold_7:9138390-9140782	8.41193	7.05846	-0.253084
Ciclev10025016m.g	scaffold_7:8669200-8675024	4.2089	3.53211	-0.252915
Ciclev10017928m.g	scaffold_2:7992743-8010810	45.7859	38.4235	-0.252914
Ciclev10007148m.g	scaffold_9:27994044-27995189	32.4124	27.2006	-0.252907
Ciclev10028231m.g	scaffold_8:24304823-24309347	154.546	129.704	-0.252817
Ciclev10016695m.g	scaffold_2:35317326-35318478	2.82431	2.37045	-0.252736
Ciclev10015649m.g	scaffold_2:35551654-35556229	20.7207	17.3917	-0.252679
Ciclev10015639m.g	scaffold_2:29629667-29632222	2.47738	2.0794	-0.252646
Ciclev10012817m.g	scaffold_6:20510147-20512065	41.6598	34.9675	-0.252642
Ciclev10030874m.g	scaffold_4:14003070-14010665	0.707588	0.593933	-0.252609
Ciclev10006263m.g	scaffold_9:13074743-13076567	277.049	232.557	-0.252559
Ciclev10018367m.g	scaffold_2:32241647-32243384	3.64439	3.05962	-0.252323
Ciclev10006723m.g	scaffold_9:6766348-6768124	4.67586	3.92583	-0.252232
Ciclev10014673m.g	scaffold_2:34695247-34699412	59.3178	49.8038	-0.252209

Ciclev10007613m.g	scaffold_1:1094543-1101551	17.9958	15.1098	-0.252178
Ciclev10023332m.g	scaffold_3:46703443-46707584	23.9983	20.1496	-0.252177
Ciclev10009577m.g	scaffold_1:8392027-8398431	37.9302	31.8475	-0.252168
Ciclev10029326m.g	scaffold_8:8074949-8076436	5.63993	4.73615	-0.251963
Ciclev10021338m.g	scaffold_3:50369658-50371345	7.30339	6.13361	-0.25183
Ciclev10020024m.g	scaffold_3:43227841-43234655	19.5414	16.412	-0.251789
Ciclev10030919m.g	scaffold_4:14468927-14474331	0.0994246	0.0835381	-0.251169
Ciclev10026686m.g	scaffold_7:4214422-4217517	61.5751	51.7477	-0.250852
Ciclev10019679m.g	scaffold_3:45304615-45309598	0.396207	0.333001	-0.250729
Ciclev10020826m.g,Ciclev10024045m.g	scaffold_3:35372249-35712253	3.72067	3.12771	-0.250457
Ciclev10004220m.g	scaffold_9:2053820-2062248	60.9184	51.2191	-0.250195
Ciclev10011817m.g	scaffold_6:25117897-25121077	159.162	133.834	-0.250045
Ciclev10008814m.g	scaffold_1:435461-440858	26.6519	22.4165	-0.249677
Ciclev10030958m.g	scaffold_4:22189095-22192952	15.9338	13.4041	-0.249409
Ciclev10024649m.g	scaffold_3:2793364-2795811	114.703	96.4941	-0.249392
Ciclev10026946m.g	scaffold_7:4593349-4597110	1.07944	0.908151	-0.249274
Ciclev10013246m.g	scaffold_6:16811267-16812118	9.38082	7.89277	-0.249181
Ciclev10026940m.g	scaffold_7:1664643-1665104	99.9215	84.0745	-0.249126
Ciclev10030510m.g	scaffold_4:18456750-18473188	13.2452	11.1457	-0.248983
Ciclev10024984m.g	scaffold_7:11637219-11644282	8.23603	6.93062	-0.248965
Ciclev10021185m.g	scaffold_3:39424396-39436836	22.3426	18.808	-0.248453
Ciclev10001182m.g	scaffold_5:42582877-42585184	7.94261	6.68636	-0.248392
Ciclev10011099m.g	scaffold_6:2642806-2648287	45.1105	37.9774	-0.248324
Ciclev10011945m.g	scaffold_6:19618870-19623379	70.2868	59.1746	-0.248275
Ciclev10026425m.g	scaffold_7:14236550-14242054	66.8682	56.3127	-0.247859
Ciclev10021621m.g	scaffold_3:47215628-47221472	7.17341	6.04118	-0.247829
Ciclev10012222m.g	scaffold_6:25105268-25107158	8.05655	6.785	-0.247813
Ciclev10022641m.g	scaffold_3:9040180-9045543	93.6637	78.8974	-0.247513
Ciclev10031554m.g	scaffold_4:20744806-20749779	17.2571	14.5388	-0.247284
Ciclev10028390m.g	scaffold_8:15749729-15754665	48.0853	40.5135	-0.247192
Ciclev10017399m.g	scaffold_2:13440052-13441745	103.09	86.863	-0.247088
Ciclev10032480m.g	scaffold_4:23815551-23819564	19.9644	16.8234	-0.246962
Ciclev10010049m.g	scaffold_1:27086758-27087231	7.83858	6.60551	-0.246922
Ciclev10032215m.g	scaffold_4:23276361-23280491	53.9441	45.4597	-0.246876
Ciclev10031292m.g	scaffold_4:7276568-7281860	12.932	10.8986	-0.246795
Ciclev10005859m.g	scaffold_9:24652831-24656311	24.3593	20.5299	-0.246741
Ciclev10022637m.g	scaffold_3:47940724-47944325	85.9514	72.4484	-0.246568
Ciclev10015200m.g	scaffold_2:35286716-35291781	22.4679	18.9405	-0.246389
Ciclev10012076m.g	scaffold_6:1117496-1122095	51.1159	43.0951	-0.246249
Ciclev10032078m.g	scaffold_4:17231015-17237310	12.8325	10.8196	-0.246155
Ciclev10033458m.g	scaffold_4:22608243-22613244	0.139044	0.117239	-0.246087
Ciclev10009104m.g	scaffold_1:102535-105601	19.2078	16.1968	-0.245979
Ciclev10029286m.g	scaffold_8:10786480-10790086	16.5278	13.9373	-0.245952
Ciclev10017538m.g	scaffold_2:36040902-36042843	187.855	158.413	-0.245931
Ciclev10020263m.g	scaffold_3:38111190-38116265	22.2429	18.7588	-0.245775
Ciclev10024878m.g	scaffold_7:5983951-5989234	7.13311	6.01591	-0.245748
Ciclev10030963m.g	scaffold_4:6787212-6792814	13.2019	11.1351	-0.245629
Ciclev10028544m.g	scaffold_8:22254354-22258398	52.9166	44.6336	-0.245591
Ciclev10015433m.g	scaffold_2:35470077-35472862	1051.29	886.758	-0.245554
Ciclev10005351m.g	scaffold_9:25708842-25712318	27.8534	23.4942	-0.245551
Ciclev10032492m.g	scaffold_4:12446219-12449597	55.723	47.007	-0.245398
Ciclev10029631m.g	scaffold_8:24097205-24099101	23.1272	19.5112	-0.24529
Ciclev10029605m.g	scaffold_8:19405381-19408147	45.0251	37.9855	-0.245281
Ciclev10007981m.g	scaffold_1:11031534-11041945	17.7546	14.9789	-0.245258

Ciclev10024689m.g,Ciclev10024692m.g,Ciclev10024693m.g,Ciclev10024698m.g,Ciclev10024937m.g,Ciclev10027469m.g	scaffold_7:12352172-12754529	46.2969	39.0607	-0.245198
Ciclev10008474m.g	scaffold_1:4075551-4078123	0.180049	0.151922	-0.245054
Ciclev10015729m.g	scaffold_2:32731452-32732986	3.14035	2.6499	-0.244988
Ciclev10025505m.g	scaffold_7:14696987-14698886	0.310983	0.262439	-0.244853
Ciclev10012174m.g	scaffold_6:22711862-22714956	45.7316	38.5938	-0.244822
Ciclev10032419m.g	scaffold_4:485789-489054	31.1233	26.2658	-0.244811
Ciclev10002090m.g	scaffold_5:41844936-41847835	26.0988	22.0259	-0.244783
Ciclev10027800m.g	scaffold_8:7966629-7969209	2.30904	1.94904	-0.24453
Ciclev10031943m.g	scaffold_4:25409034-25412164	26.2133	22.128	-0.244423
Ciclev10029702m.g	scaffold_8:24094524-24097049	45.3983	38.3261	-0.244311
Ciclev10026275m.g	scaffold_7:10862824-10867916	43.9545	37.109	-0.244241
Ciclev10000947m.g	scaffold_5:40691800-40694522	7.88013	6.65343	-0.244122
Ciclev10022532m.g	scaffold_3:3169451-3172178	196.434	165.857	-0.244099
Ciclev10016188m.g	scaffold_2:8114155-8118479	20.495	17.3102	-0.243653
Ciclev10012227m.g	scaffold_6:2198314-2200549	99.7031	84.2172	-0.243523
-	scaffold_122:22865-23434	203.416	171.826	-0.243485
Ciclev10016706m.g	scaffold_2:20153459-20156796	1170.81	988.985	-0.243483
Ciclev10001634m.g	scaffold_5:424432-427199	11.0396	9.32695	-0.243209
Ciclev10024801m.g	scaffold_7:9304911-9314043	0.485523	0.410209	-0.243181
Ciclev10027381m.g	scaffold_7:17002958-17007249	0.0765239	0.0646596	-0.243045
Ciclev10000174m.g	scaffold_5:15086105-15095333	39.7892	33.6221	-0.242967
Ciclev10002945m.g	scaffold_5:37096174-37098369	288.772	244.039	-0.24282
Ciclev10001485m.g	scaffold_5:37652360-37656378	19.8379	16.7664	-0.242688
Ciclev10018840m.g	scaffold_3:14468143-14477222	29.6933	25.0972	-0.242614
Ciclev10027229m.g	scaffold_7:13916806-14069972	0.349846	0.295761	-0.242291
Ciclev10018500m.g	scaffold_3:33346281-33357142	0.784427	0.663317	-0.241941
Ciclev10028969m.g	scaffold_8:24666728-24669708	20.6438	17.4572	-0.241889
Ciclev10024093m.g	scaffold_3:2882370-2886879	0.406621	0.343886	-0.241753
Ciclev10006291m.g	scaffold_9:6882900-6884531	90.7863	76.781	-0.241724
Ciclev10004177m.g	scaffold_9:29863655-29873118	0.0469517	0.0397132	-0.241559
Ciclev10004086m.g	scaffold_5:38603368-38606636	0.485177	0.410396	-0.241493
Ciclev10009304m.g	scaffold_1:947895-950969	346.681	293.289	-0.241285
Ciclev10030438m.g	scaffold_8:22214111-22215352	16.4554	13.9217	-0.241226
-	scaffold_7:7011916-7012404	17.0911	14.4644	-0.24074
Ciclev10009035m.g	scaffold_1:6023155-6156487	14.135	11.9644	-0.240518
Ciclev10025072m.g	scaffold_7:5939879-5945344	5.43846	4.60334	-0.240516
Ciclev10009082m.g	scaffold_1:1529754-1532900	35.6678	30.1913	-0.24049
Ciclev10000085m.g	scaffold_5:18178099-18191595	12.4058	10.5013	-0.240453
Ciclev10016277m.g	scaffold_2:13310843-13314055	12.7788	10.8171	-0.240441
-	scaffold_5:10586129-10586370	50.8379	43.0353	-0.240382
-	scaffold_7:11085565-11086485	23.8062	20.1548	-0.240212
Ciclev10016974m.g	scaffold_2:30435789-30437278	325.992	276.003	-0.240151
Ciclev10016051m.g	scaffold_2:36360248-36364688	31.6459	26.7955	-0.240026
Ciclev10000077m.g	scaffold_5:8520323-8529662	0.235322	0.199257	-0.240001
Ciclev10011132m.g	scaffold_6:23760364-23764900	5.07887	4.30074	-0.239924
Ciclev10021577m.g	scaffold_3:6329132-6332201	287.445	243.429	-0.239784
Ciclev10009862m.g	scaffold_1:5682448-5685827	0.330684	0.280057	-0.239735
Ciclev10007796m.g	scaffold_1:3082252-3085455	6.36634	5.39223	-0.239581
Ciclev10031844m.g	scaffold_4:18515567-18675155	0.106021	0.0898037	-0.239504
Ciclev10031159m.g	scaffold_4:24737049-24741836	20.5467	17.4047	-0.239432
Ciclev10009370m.g	scaffold_1:3152547-3153983	3.87059	3.27924	-0.239191
Ciclev10032302m.g	scaffold_4:6100707-6103243	163.671	138.68	-0.239033

Ciclev10028999m.g	scaffold_8:2253460-2256041	61.5547	52.1696	-0.238659
Ciclev10016228m.g	scaffold_2:30499724-30504591	53.723	45.538	-0.238468
Ciclev10002700m.g	scaffold_5:42907630-42909811	69.5612	58.9641	-0.238446
Ciclev10006429m.g	scaffold_9:883115-885386	33.6327	28.5094	-0.238426
Ciclev10011803m.g	scaffold_6:13714431-13719318	13.9515	11.8299	-0.237988
Ciclev10028974m.g	scaffold_8:23733110-23735863	22.4641	19.048	-0.237982
Ciclev10011776m.g	scaffold_6:17995492-17999289	42.8887	36.3705	-0.237828
Ciclev10028818m.g	scaffold_8:22114548-22117612	23.8001	20.1851	-0.237677
Ciclev10002895m.g	scaffold_5:2461405-2462911	61.8488	52.4571	-0.237607
Ciclev10008612m.g	scaffold_1:28429427-28433154	23.5727	19.9941	-0.23754
Ciclev10018263m.g	scaffold_2:20342544-20346784	1.01866	0.86403	-0.237515
Ciclev10015017m.g	scaffold_2:26262942-26266468	194.937	165.381	-0.237222
Ciclev10018834m.g	scaffold_3:585619-589952	0.250038	0.212139	-0.237139
Ciclev10033033m.g	scaffold_4:22163574-22164234	71.9533	61.0493	-0.237086
Ciclev10021386m.g	scaffold_3:20750711-20753437	162.374	137.769	-0.237066
Ciclev10009610m.g	scaffold_1:9895995-9900184	52.4895	44.5445	-0.236781
Ciclev10021605m.g	scaffold_3:44510199-44514531	106.193	90.1244	-0.236695
Ciclev10023002m.g	scaffold_3:7931525-7933933	68.7902	58.3816	-0.236691
Ciclev10019040m.g	scaffold_3:48775889-48781106	36.8767	31.3028	-0.23642
-	scaffold_4:21270257-21271997	231.467	196.493	-0.236333
Ciclev10021421m.g	scaffold_3:43713163-43718608	7.18467	6.09961	-0.236204
Ciclev10009458m.g	scaffold_1:4267475-4268413	2.17673	1.84824	-0.23601
Ciclev10002475m.g	scaffold_5:42329950-42331110	295.427	250.866	-0.235882
Ciclev10018607m.g	scaffold_3:10050622-10055433	32.3177	27.444	-0.235836
Ciclev10010361m.g	scaffold_1:7173142-7175233	0.256114	0.217491	-0.235826
Ciclev10022949m.g	scaffold_3:20887902-20889521	166.721	141.6	-0.23562
Ciclev10028748m.g	scaffold_8:152384-156478	6.20767	5.27229	-0.23562
Ciclev10001370m.g	scaffold_5:34462922-34464693	218.675	185.74	-0.235498
Ciclev10031101m.g	scaffold_4:14722373-14731300	32.4819	27.5948	-0.235242
Ciclev10006204m.g	scaffold_9:4852517-4856998	4.59342	3.90247	-0.235182
Ciclev10016210m.g	scaffold_2:35466892-35469412	229.72	195.194	-0.234966
Ciclev10009584m.g	scaffold_1:20462859-20465557	189.711	161.204	-0.234909
Ciclev10001744m.g	scaffold_5:34219816-34223850	6.21606	5.28293	-0.234664
Ciclev10028697m.g	scaffold_8:19345062-19347259	39.7685	33.7989	-0.234649
Ciclev10002356m.g	scaffold_5:21354053-21361949	55.3991	47.089	-0.234471
Ciclev10022891m.g	scaffold_3:36924550-37054826	25.8135	21.9427	-0.234384
Ciclev10008259m.g	scaffold_1:57753-61152	18.9154	16.0796	-0.234333
Ciclev10021910m.g	scaffold_3:34260414-34261417	0.206497	0.175547	-0.234266
Ciclev10019352m.g	scaffold_3:859090-864509	24.4007	20.7438	-0.234241
Ciclev10001104m.g	scaffold_5:38278462-38282008	10.9217	9.28488	-0.234238
Ciclev10018695m.g	scaffold_3:41067674-41229006	37.379	31.7777	-0.234211
Ciclev10024822m.g	scaffold_7:9016612-9021744	0.0588349	0.05002	-0.234167
Ciclev10009301m.g	scaffold_1:22698687-22701817	19.5063	16.5853	-0.234034
Ciclev10025755m.g,Ciclev10026185m.g	scaffold_7:4984937-4993217	68.092	57.8969	-0.233997
Ciclev10029293m.g	scaffold_8:4161511-4163718	12.6423	10.75	-0.233924
Ciclev10013038m.g	scaffold_6:23004524-23005537	15.5582	13.2319	-0.233655
Ciclev10015695m.g	scaffold_2:36109512-36111528	5.41723	4.60761	-0.233536
Ciclev10019090m.g	scaffold_3:41364483-41372406	38.8871	33.0838	-0.233168
Ciclev10034000m.g	scaffold_4:4442480-4443708	0.338524	0.288032	-0.233029
Ciclev10015756m.g	scaffold_2:5622193-5627328	5.35275	4.55493	-0.232852
Ciclev10021506m.g	scaffold_3:8863737-8868063	61.8617	52.6424	-0.232823
Ciclev10011632m.g	scaffold_6:20032678-20037208	4.13113	3.51553	-0.232794
Ciclev10019660m.g	scaffold_3:48927020-48930561	4.67822	3.98123	-0.232747
Ciclev10007561m.g	scaffold_1:4343811-4351112	6.95366	5.91784	-0.232704
Ciclev10009646m.g	scaffold_1:729433-733112	25.7136	21.8835	-0.232689

Ciclev10012584m.g	scaffold_6:16338497-16344107	7.77003	6.61328	-0.232554
Ciclev10026818m.g,Ciclev10027192m.g	scaffold_7:17884685-17891357	16.5949	14.125	-0.232489
Ciclev10021983m.g	scaffold_3:12413336-12415802	93.7946	79.8353	-0.232478
Ciclev10027075m.g	scaffold_7:19950882-20044587	9.36853	7.97555	-0.23224
Ciclev10005487m.g	scaffold_9:6896162-6899722	120.077	102.23	-0.232133
Ciclev10021431m.g	scaffold_3:26610746-26615954	8.84483	7.53054	-0.232081
Ciclev10021195m.g	scaffold_3:35349180-35352968	19.365	16.4888	-0.231962
Ciclev10008025m.g	scaffold_1:23858133-23865242	23.2103	19.763	-0.23196
Ciclev10025507m.g	scaffold_7:2602193-2605924	52.235	44.483	-0.231761
Ciclev10006039m.g	scaffold_9:1379847-1380607	36.3511	30.9573	-0.231719
Ciclev10022329m.g	scaffold_3:47963149-47964781	9.9243	8.45192	-0.231686
Ciclev10025265m.g	scaffold_7:16287568-16295373	19.8482	16.9048	-0.231577
Ciclev10026330m.g	scaffold_7:2516642-2520591	27.7989	23.6781	-0.231475
Ciclev10030923m.g	scaffold_4:22000710-22007343	23.2341	19.7903	-0.231451
Ciclev10033289m.g	scaffold_4:21950026-21951065	30.2075	25.7312	-0.231388
Ciclev10012043m.g	scaffold_6:19076229-19077992	37.9006	32.2845	-0.231379
Ciclev10033171m.g	scaffold_4:2274701-2276412	0.302552	0.257731	-0.231319
Ciclev10005240m.g	scaffold_9:11902905-11909432	31.3905	26.7448	-0.23107
Ciclev10005243m.g,Ciclev10005580m.g	scaffold_9:26211987-26236468	18.7258	15.9565	-0.230884
Ciclev10030739m.g	scaffold_4:19921297-19926352	2.50258	2.13259	-0.230813
Ciclev10020777m.g	scaffold_3:49359634-49366836	52.8276	45.0178	-0.230797
Ciclev10020115m.g	scaffold_3:1003168-1008156	0.6652	0.566871	-0.230768
Ciclev10000418m.g	scaffold_5:7288318-7296349	60.657	51.6926	-0.230715
Ciclev10029607m.g	scaffold_8:21533337-21538855	65.2168	55.5849	-0.23055
Ciclev10024732m.g	scaffold_7:5262682-5272835	1.36465	1.16315	-0.230498
Ciclev10026366m.g	scaffold_7:7707533-7711360	74.7448	63.7083	-0.230492
Ciclev10028863m.g	scaffold_8:1165175-1168300	7.51602	6.4065	-0.230431
Ciclev10018850m.g,Ciclev10024514m.g	scaffold_3:41311545-41317333	8.5118	7.25581	-0.230328
Ciclev10016178m.g	scaffold_2:27688773-27692240	31.2543	26.6432	-0.230285
Ciclev10023622m.g	scaffold_3:33701269-33702102	38.5663	32.8772	-0.230251
Ciclev10020487m.g	scaffold_3:35943914-35948556	93.2262	79.4774	-0.23019
Ciclev10007880m.g	scaffold_1:25694515-25699120	16.4979	14.065	-0.230174
Ciclev10019370m.g	scaffold_3:9332235-9343875	46.7926	39.9016	-0.229836
Ciclev10025368m.g	scaffold_7:8770312-8776325	41.2831	35.2093	-0.229596
Ciclev10008957m.g	scaffold_1:27733135-27734134	0.519258	0.44288	-0.229537
Ciclev10021027m.g	scaffold_3:46804585-46806158	4.45926	3.80337	-0.229528
Ciclev10030188m.g	scaffold_8:18702609-18703217	3.23285	2.75769	-0.229347
Ciclev10030042m.g	scaffold_8:23791291-23794338	154.842	132.088	-0.229299
Ciclev10017794m.g	scaffold_2:7091005-7093729	0.396834	0.33852	-0.229293
Ciclev10028439m.g	scaffold_8:23180386-23181882	3.89706	3.32444	-0.229274
Ciclev10008325m.g	scaffold_1:22172744-22175311	0.300763	0.256607	-0.229067
Ciclev10016298m.g	scaffold_2:32754954-32757337	46.4246	39.6097	-0.229034
Ciclev10006059m.g	scaffold_9:1313424-1319164	18.1006	15.4437	-0.229019
Ciclev10021394m.g	scaffold_3:808542-811384	15.3094	13.0632	-0.228915
Ciclev10001100m.g	scaffold_5:38108660-38114412	131.103	111.867	-0.228911
Ciclev10022715m.g	scaffold_3:24801988-24804993	18.4418	15.7367	-0.228844
Ciclev10013007m.g	scaffold_6:19709882-19714583	80.2529	68.4814	-0.228842
Ciclev10032994m.g	scaffold_4:24742453-24746142	25.273	21.5689	-0.228647
Ciclev10017401m.g	scaffold_2:10097307-10100347	0.910759	0.777311	-0.228578
Ciclev10003047m.g	scaffold_5:36703531-36705350	60.5361	51.6669	-0.228555
Ciclev10007013m.g	scaffold_9:28789952-28790567	59.0942	50.4413	-0.228411
Ciclev10013173m.g	scaffold_6:2576664-2578793	25.8348	22.0525	-0.228378
Ciclev10015661m.g	scaffold_2:32541902-32543726	84.4867	72.1231	-0.228262

Ciclev10020813m.g,Ciclev10020816m.g	scaffold_3:39003334-39013340	13.8824	11.8532	-0.227977
Ciclev10018514m.g	scaffold_3:9242232-9262502	14.193	12.1193	-0.22787
Ciclev10025365m.g	scaffold_7:10135304-10139379	20.4006	17.4201	-0.227856
Ciclev10019291m.g	scaffold_3:1864603-1871328	24.8251	21.1995	-0.227767
Ciclev10018507m.g	scaffold_3:27267892-27272240	0.355626	0.303719	-0.227626
Ciclev10011836m.g	scaffold_6:22551668-22554518	10.4074	8.88891	-0.227528
Ciclev10029309m.g	scaffold_8:6188272-6189242	39.3728	33.6309	-0.227412
Ciclev10008048m.g	scaffold_1:10705681-10713493	16.7961	14.347	-0.227381
Ciclev10029109m.g	scaffold_8:2608217-2611946	20.3484	17.3829	-0.227248
Ciclev10021337m.g	scaffold_3:19244782-19252200	47.7589	40.7987	-0.227246
Ciclev10016018m.g	scaffold_2:35798995-35801167	0.108294	0.0925155	-0.227192
Ciclev10033375m.g	scaffold_4:2954331-2955831	0.194918	0.166536	-0.227035
Ciclev10010965m.g	scaffold_6:22368270-22375518	0.536882	0.458731	-0.226956
Ciclev10015608m.g	scaffold_2:34331653-34335809	108.838	93.0241	-0.226508
Ciclev10014398m.g	scaffold_2:29773815-29781129	4.26748	3.64764	-0.226422
Ciclev10014271m.g	scaffold_2:31471037-31482943	36.9975	31.629	-0.226182
Ciclev10033061m.g	scaffold_4:25058290-25061910	105.093	89.8502	-0.226075
Ciclev10015416m.g	scaffold_2:33737074-33741138	460.297	393.572	-0.225938
Ciclev10024939m.g	scaffold_7:6000982-6007795	1.7351	1.48358	-0.225936
Ciclev10022200m.g	scaffold_3:49388058-49389305	6.28393	5.37332	-0.225853
Ciclev10025137m.g,Ciclev10025168m.g	scaffold_7:18743277-18800003	10.1605	8.68819	-0.225844
Ciclev10021998m.g	scaffold_3:4920661-4923421	142.957	122.242	-0.225835
Ciclev10032069m.g	scaffold_4:23666783-23669594	44.3809	37.9539	-0.22569
Ciclev10004493m.g	scaffold_9:22110428-22114889	20.5124	17.542	-0.22569
Ciclev10002763m.g	scaffold_5:41057354-41058811	59.4462	50.8376	-0.225689
Ciclev10011939m.g	scaffold_6:22652065-22657755	12.742	10.8973	-0.225622
Ciclev10029039m.g	scaffold_8:7354520-7359255	19.0392	16.2832	-0.225585
Ciclev10015597m.g	scaffold_2:34609195-34614203	22.2517	19.031	-0.225559
Ciclev10020964m.g	scaffold_3:5289554-5293873	60.3767	51.6396	-0.225513
Ciclev10022503m.g	scaffold_3:18925718-18932522	23.9957	20.5247	-0.225416
Ciclev10023034m.g	scaffold_3:47980805-47985365	15.3779	13.1538	-0.225378
Ciclev10019143m.g	scaffold_3:2504394-2508834	10.6369	9.099	-0.225294
Ciclev10016576m.g	scaffold_2:13857321-13860557	15.2754	13.0676	-0.225214
Ciclev10003025m.g	scaffold_5:34499264-34500918	38.9907	33.3562	-0.225176
Ciclev10020855m.g	scaffold_3:20890247-20896717	17.4161	14.9066	-0.224469
Ciclev10003673m.g,Ciclev10003722m.g,Ciclev10003903m.g	scaffold_5:31333200-31391489	3.04042	2.60255	-0.224345
Ciclev10016988m.g	scaffold_2:1260268-1384822	56.3577	48.2435	-0.224277
Ciclev10011695m.g	scaffold_6:22039490-22042363	8.43302	7.21887	-0.224275
Ciclev10026197m.g	scaffold_7:4513036-4514995	99.788	85.4265	-0.224183
Ciclev10019052m.g	scaffold_3:47921450-47925057	7.72769	6.61613	-0.22405
Ciclev10019675m.g	scaffold_3:42816765-42820382	18.0534	15.4584	-0.223885
Ciclev10020352m.g	scaffold_3:23708082-23711317	24.1572	20.6858	-0.223813
Ciclev10022858m.g	scaffold_3:5735311-5737986	280.559	240.251	-0.223762
Ciclev10002195m.g	scaffold_5:41568008-41571721	18.579	15.91	-0.223735
Ciclev10004237m.g	scaffold_9:27526852-27535693	14.038	12.0216	-0.223709
Ciclev10012214m.g,Ciclev10012267m.g	scaffold_6:14765914-14777322	90.6177	77.6043	-0.223657
Ciclev10017400m.g	scaffold_2:13393864-13394541	5.31828	4.55465	-0.223619
Ciclev10001272m.g	scaffold_5:33351542-33354758	85.4311	73.1685	-0.223539
Ciclev10016525m.g	scaffold_2:32437123-32439365	867.408	742.932	-0.223481
Ciclev10005337m.g	scaffold_9:4121562-4124482	32.0395	27.4421	-0.22346
Ciclev10009570m.g	scaffold_1:3255120-3258139	19.3307	16.5572	-0.223433

Ciclev10020953m.g	scaffold_3:3263222-3265031	4.28902	3.67375	-0.223393
Ciclev10024243m.g	scaffold_3:48937449-48942653	103.788	88.9019	-0.223354
Ciclev10010848m.g	scaffold_1:21975140-22013742	0.442777	0.379276	-0.223332
Ciclev10021447m.g	scaffold_3:42647412-42652459	60.9419	52.214	-0.222998
Ciclev10000557m.g	scaffold_5:17106093-17108296	0.544748	0.466832	-0.222687
-	scaffold_5:4076819-4284428	78.4088	67.202	-0.222511
Ciclev10010475m.g	scaffold_1:22069934-22073130	0.147576	0.126489	-0.222444
Ciclev10032483m.g	scaffold_4:20765280-20767553	29.9342	25.6637	-0.222062
Ciclev10023014m.g	scaffold_3:25605170-25606024	1.17615	1.00838	-0.222026
Ciclev10017858m.g	scaffold_2:34592747-34601529	3.69337	3.16658	-0.222014
Ciclev10018680m.g	scaffold_3:38603363-38615654	18.4031	15.7782	-0.222009
Ciclev10029835m.g	scaffold_8:17291958-17467945	2.9672	2.54408	-0.221959
Ciclev10027742m.g	scaffold_8:18276680-18289119	30.8501	26.4534	-0.221818
Ciclev10020374m.g	scaffold_3:49082984-49085406	20.2976	17.4057	-0.22175
Ciclev10025402m.g,Ciclev10027139m.g	scaffold_7:2160486-2167974	16.3651	14.0366	-0.22143
Ciclev10000198m.g	scaffold_5:23637161-23640316	0.108675	0.0932187	-0.22133
Ciclev10018912m.g	scaffold_3:43070753-43074991	0.0748955	0.064249	-0.221203
Ciclev10025906m.g	scaffold_7:1233609-1239263	12.5439	10.7612	-0.221145
Ciclev10023490m.g	scaffold_3:3764749-3766390	24.2434	20.7987	-0.221097
Ciclev10014449m.g	scaffold_2:9396890-9403785	46.1677	39.6083	-0.221082
Ciclev10031334m.g	scaffold_4:21676380-21680465	22.194	19.0412	-0.221044
Ciclev10017506m.g	scaffold_2:5273993-5337902	0.513985	0.440994	-0.220966
Ciclev10033058m.g	scaffold_4:25364678-25366150	134.732	115.601	-0.220935
Ciclev10022208m.g	scaffold_3:9747774-9750640	170.701	146.47	-0.220859
Ciclev10005993m.g	scaffold_9:3914729-3917631	2.90549	2.49329	-0.220731
Ciclev10004454m.g	scaffold_9:4940947-4944426	3.0863	2.6487	-0.220593
Ciclev10005343m.g	scaffold_9:27867871-27870843	43.737	37.5433	-0.220301
Ciclev10026340m.g	scaffold_7:5554618-5556993	172.374	147.968	-0.22026
Ciclev10024951m.g	scaffold_7:3887009-3890047	22.4063	19.2345	-0.220203
-	scaffold_3:19174945-19176182	1975.58	1695.95	-0.22018
Ciclev10007611m.g	scaffold_1:23511712-23515904	7.26234	6.23469	-0.220116
Ciclev10001058m.g	scaffold_5:41662679-41665662	30.3868	26.0882	-0.220044
Ciclev10020184m.g,Ciclev10023406m.g	scaffold_3:38078369-38107449	20.4269	17.5376	-0.220018
Ciclev10022576m.g	scaffold_3:45906878-45907641	24.7934	21.2886	-0.21988
Ciclev10001547m.g	scaffold_5:9471784-9476073	0.641731	0.551022	-0.21986
Ciclev10032992m.g	scaffold_4:19001055-19040863	46.4143	39.8565	-0.219757
Ciclev10029262m.g	scaffold_8:24742619-24743741	6.17787	5.3052	-0.219702
Ciclev10005994m.g	scaffold_9:1879674-1880523	18.2246	15.6507	-0.219658
Ciclev10016446m.g	scaffold_2:27197771-27199773	4.58333	3.93614	-0.219612
-	scaffold_1:14847404-14848726	8.13306	6.98482	-0.219575
Ciclev10010700m.g	scaffold_1:4989025-4994456	48.6928	41.8184	-0.219571
Ciclev10024490m.g	scaffold_3:37168146-37169082	0.258487	0.221996	-0.219558
Ciclev10016269m.g	scaffold_2:11680329-11683191	6.97193	5.98919	-0.219198
Ciclev10014548m.g	scaffold_2:28942816-28945490	14.4214	12.3889	-0.219164
Ciclev10025934m.g	scaffold_7:2148801-2151505	63.329	54.4055	-0.219115
Ciclev10004850m.g	scaffold_9:13440331-13442904	22.8043	19.5919	-0.21905
-	scaffold_5:32404311-32420675	5.54588	4.76465	-0.219044
Ciclev10019649m.g	scaffold_3:25639010-25644826	93.4904	80.3274	-0.218927
Ciclev10022498m.g	scaffold_3:195777-197622	40.2252	34.5646	-0.218807
Ciclev10001980m.g	scaffold_5:36859868-36862687	28.5197	24.5071	-0.218763
Ciclev10016282m.g	scaffold_2:138850-142756	9.83784	8.45393	-0.21872
Ciclev10006176m.g	scaffold_9:1633687-1634853	12.5702	10.8021	-0.2187
-	scaffold_1:28339863-28345269	13.3924	11.5098	-0.218553
Ciclev10015188m.g	scaffold_2:8287532-8293766	56.9443	48.9426	-0.218461

Ciclev10001693m.g	scaffold_5:28145684-28147060	0.164388	0.141305	-0.218299
Ciclev10013400m.g	scaffold_6:18153291-18154630	0.439975	0.378332	-0.217768
Ciclev10026450m.g	scaffold_7:7656669-7660212	36.3784	31.2852	-0.2176
Ciclev10005889m.g	scaffold_9:70997-74462	1.23389	1.06123	-0.217478
Ciclev10005358m.g	scaffold_9:30662180-30667100	11.9899	10.3125	-0.217417
Ciclev10024730m.g	scaffold_7:4108506-4114955	0.092123	0.0792362	-0.217402
Ciclev10018267m.g	scaffold_2:13869716-13870142	10.8267	9.31258	-0.217342
Ciclev10009283m.g	scaffold_1:21870010-21873931	20.7041	17.8091	-0.217301
Ciclev10002218m.g	scaffold_5:30677509-30679699	3.05196	2.62533	-0.217234
-	scaffold_2:20462080-20462685	3.41424	2.93706	-0.21719
Ciclev10003214m.g	scaffold_5:33986603-33987724	3.83963	3.30314	-0.21713
Ciclev10007284m.g	scaffold_1:28232112-28238509	11.9535	10.2837	-0.217066
Ciclev10007458m.g	scaffold_1:26783039-26787341	20.4981	17.6364	-0.216935
Ciclev10024848m.g	scaffold_7:12181226-12185029	0.32424	0.279012	-0.216734
Ciclev10008605m.g	scaffold_1:5328211-5334513	279.95	240.908	-0.216686
Ciclev10004427m.g	scaffold_9:17255227-17273372	34.5626	29.7451	-0.216559
Ciclev10027764m.g	scaffold_8:21005972-21016063	18.527	15.9456	-0.216468
Ciclev10015856m.g	scaffold_2:3750172-3752653	2.97065	2.55697	-0.216345
Ciclev10015999m.g	scaffold_2:21111678-21116170	37.76	32.5119	-0.215889
Ciclev10011371m.g,Ciclev10011616m.g,Ciclev10013371m.g	scaffold_6:22828055-22898575	10.9973	9.46984	-0.215743
Ciclev10025511m.g	scaffold_7:8962364-8965569	59.8325	51.5248	-0.215663
Ciclev10032872m.g	scaffold_4:23014309-23015851	97.0209	83.5564	-0.215545
Ciclev10020707m.g	scaffold_3:4880918-4882591	7.52165	6.47803	-0.215495
Ciclev10014689m.g	scaffold_2:26821202-26826999	10.4161	8.97248	-0.215236
Ciclev10025317m.g	scaffold_7:1652293-1659213	25.1573	21.673	-0.215082
Ciclev10032520m.g	scaffold_4:6975582-6980919	46.2598	39.8531	-0.215068
Ciclev10001889m.g	scaffold_5:36513674-36515140	2.01687	1.73764	-0.214985
Ciclev10029197m.g	scaffold_8:15411103-15415346	41.6795	35.9093	-0.214978
Ciclev10031676m.g	scaffold_4:19462704-19467432	13.92	11.9933	-0.214929
Ciclev10022560m.g	scaffold_3:49908505-49909729	207.26	178.635	-0.21443
Ciclev10016368m.g	scaffold_2:35013847-35015439	124.396	107.219	-0.214375
Ciclev10025877m.g	scaffold_7:4851826-4855236	21.067	18.1615	-0.214097
Ciclev10011920m.g	scaffold_6:18066636-18071382	9.01497	7.77262	-0.213922
Ciclev10015851m.g	scaffold_2:9655577-9659517	9.77824	8.43093	-0.213884
Ciclev10007587m.g	scaffold_1:26744896-26750726	5.45628	4.70524	-0.213651
Ciclev10012613m.g	scaffold_6:6534835-6539295	68.3225	58.9197	-0.213612
Ciclev10021463m.g	scaffold_3:47717901-47720529	18.2113	15.7051	-0.213599
Ciclev10029418m.g	scaffold_8:25018898-25020459	123.369	106.398	-0.213504
Ciclev10001120m.g	scaffold_5:36564476-36569792	53.721	46.3334	-0.213432
Ciclev10000807m.g	scaffold_5:15623323-15630017	60.7077	52.3607	-0.213393
Ciclev10024816m.g	scaffold_7:6733199-6739641	11.1237	9.5947	-0.213327
Ciclev10033481m.g	scaffold_4:7055580-7057811	3.72217	3.21068	-0.213267
Ciclev10007529m.g	scaffold_1:375270-380303	19.7311	17.0213	-0.213131
Ciclev10015019m.g,Ciclev10017842m.g,Ciclev10017937m.g	scaffold_2:5845644-5878657	7.89762	6.81372	-0.212977
Ciclev10005304m.g	scaffold_9:30527056-30531535	8.32216	7.18079	-0.212816
Ciclev10008891m.g	scaffold_1:25724884-25728563	10.7521	9.27753	-0.212806
Ciclev10026539m.g	scaffold_7:708739-711962	68.7492	59.3277	-0.212637
Ciclev10016017m.g	scaffold_2:33190966-33192493	30.7515	26.5391	-0.212536
Ciclev10022892m.g	scaffold_3:43610650-43613471	52.6659	45.4528	-0.212498
Ciclev10016453m.g	scaffold_2:33067670-33071050	51.0768	44.0832	-0.21244
Ciclev10006115m.g	scaffold_9:5152011-5155157	47.5952	41.0869	-0.212137
Ciclev10024529m.g	scaffold_3:40848610-40913406	0.116508	0.100592	-0.211911

Ciclev10005572m.g	scaffold_9:21052328-21056667	94.013	81.1799	-0.211736
Ciclev10004004m.g	scaffold_5:10590809-10591703	2.7546	2.37872	-0.211659
Ciclev10003646m.g	scaffold_5:28289811-28536377	0.598678	0.517042	-0.211498
Ciclev10010890m.g	scaffold_6:7561834-7666028	53.4294	46.1453	-0.21145
Ciclev10012662m.g	scaffold_6:1341778-1348510	24.385	21.0609	-0.211425
Ciclev10012445m.g	scaffold_6:24518131-24520887	152.726	131.909	-0.211402
Ciclev10026092m.g	scaffold_7:5331032-5334481	11.6484	10.0608	-0.211399
Ciclev10010570m.g	scaffold_1:8681799-8686022	16.1111	13.9164	-0.211268
Ciclev10021173m.g	scaffold_3:49840889-49845522	9.68433	8.36543	-0.211214
Ciclev10008210m.g	scaffold_1:21875021-21879839	40.0163	34.5716	-0.211003
Ciclev10000826m.g	scaffold_5:34386297-34390000	8.5097	7.35228	-0.210917
Ciclev10022616m.g	scaffold_3:9984084-9985794	110.492	95.4869	-0.210566
Ciclev10028533m.g	scaffold_8:350998-354814	4.58161	3.95944	-0.210561
Ciclev10025071m.g	scaffold_7:15855468-15857518	0.0854185	0.0738394	-0.210157
Ciclev10032685m.g	scaffold_4:10900482-10903652	35.2076	30.4376	-0.210034
Ciclev10028891m.g	scaffold_8:18152117-18155413	15.819	13.6787	-0.209727
Ciclev10015303m.g,Ciclev10015356m.g,Ciclev10017643m.g	scaffold_2:15477859-15615778	25.049	21.6639	-0.209464
Ciclev10016837m.g	scaffold_2:30237033-30239532	24.5792	21.2593	-0.209342
Ciclev10012338m.g,Ciclev10013913m.g	scaffold_6:21253669-21258519	60.0691	51.9601	-0.20922
Ciclev10005143m.g	scaffold_9:2983707-2988080	13.6386	11.7992	-0.209008
Ciclev10019580m.g	scaffold_3:6454423-6457125	137.082	118.602	-0.208908
Ciclev10001792m.g	scaffold_5:39828395-39834822	18.8415	16.3018	-0.208884
Ciclev10016641m.g	scaffold_2:34415188-34418709	33.4995	28.9866	-0.208753
Ciclev10020012m.g	scaffold_3:45625451-45629218	6.68562	5.78532	-0.208664
Ciclev10027601m.g	scaffold_7:14425058-14427839	0.118458	0.102513	-0.208575
Ciclev10021564m.g	scaffold_3:7420175-7425027	906.756	784.722	-0.208533
Ciclev10020501m.g	scaffold_3:5972287-5978062	29.2343	25.3047	-0.208254
Ciclev10022912m.g	scaffold_3:13122009-13122890	36.2552	31.3842	-0.208151
Ciclev10004481m.g	scaffold_9:7232958-7238380	11.454	9.91588	-0.208039
Ciclev10019323m.g	scaffold_3:50841050-50845732	20.2976	17.5741	-0.207859
Ciclev10025441m.g	scaffold_7:11621416-11630199	69.6626	60.3331	-0.207434
Ciclev10028685m.g,Ciclev10030375m.g	scaffold_8:7698015-7862701	22.9781	19.9018	-0.207358
Ciclev10001110m.g	scaffold_5:185640-188056	2.96799	2.57071	-0.207317
Ciclev10003965m.g	scaffold_5:17660323-17663665	0.285798	0.247543	-0.207316
Ciclev10011298m.g	scaffold_6:17655685-17661763	108.676	94.1322	-0.20727
Ciclev10016654m.g	scaffold_2:25726333-25729314	10.0013	8.6632	-0.207215
Ciclev10001340m.g	scaffold_5:34936852-34940733	2.82942	2.45092	-0.207181
Ciclev10000348m.g	scaffold_5:29691666-29699867	17.8416	15.455	-0.207176
Ciclev10028655m.g	scaffold_8:4892021-4896871	52.115	45.1443	-0.207154
-	scaffold_9:10622071-10623538	9.53374	8.26073	-0.206773
Ciclev10021814m.g	scaffold_3:50415678-50417877	69.9178	60.5832	-0.206743
Ciclev10016637m.g	scaffold_2:25533784-25535072	2569.07	2226.1	-0.206729
Ciclev10012758m.g	scaffold_6:19316501-19319914	138.697	120.183	-0.206711
Ciclev10025798m.g	scaffold_7:881925-885612	10.5064	9.10455	-0.206605
Ciclev10030733m.g	scaffold_4:20833851-20844809	31.2849	27.1127	-0.206499
Ciclev10011309m.g	scaffold_6:24352518-24356049	5.14039	4.45523	-0.20638
Ciclev10010808m.g	scaffold_1:11516458-11518804	62.2335	53.9384	-0.206378
Ciclev10013069m.g	scaffold_6:22920686-22921383	7.47203	6.47649	-0.206287
Ciclev10018377m.g	scaffold_2:13395446-13396682	25.431	22.0451	-0.206132
Ciclev10001821m.g,Ciclev10003488m.g	scaffold_5:30686645-30697419	43.1355	37.3937	-0.206082
Ciclev10027816m.g	scaffold_8:2412964-2419157	5.39833	4.67977	-0.206076

Ciclev10015586m.g	scaffold_2:10011214-10013704	3.22597	2.79698	-0.205865
Ciclev10005021m.g	scaffold_9:28608334-28612490	111.093	96.3239	-0.205808
Ciclev10024039m.g	scaffold_3:48610551-48613529	3.92859	3.40632	-0.205799
Ciclev10020214m.g	scaffold_3:41627893-41634431	21.0589	18.2607	-0.20569
Ciclev10010992m.g	scaffold_6:15262112-15265789	4.71704	4.09148	-0.205261
-	scaffold_8:60351-61361	38.399	33.3083	-0.205187
Ciclev10005176m.g	scaffold_9:3223562-3228240	21.0045	18.2223	-0.204992
Ciclev10032272m.g	scaffold_4:23290323-23293183	24.3672	21.1411	-0.204893
Ciclev10007396m.g	scaffold_1:1173028-1176194	8.92958	7.7477	-0.204825
Ciclev10013557m.g	scaffold_6:623230-626684	0.0614608	0.0533261	-0.204823
Ciclev10005001m.g	scaffold_9:9809899-9811331	0.143593	0.124606	-0.204622
Ciclev10008192m.g	scaffold_1:28607324-28610917	55.6953	48.3337	-0.204527
Ciclev10031176m.g	scaffold_4:14077258-14082781	7.80183	6.7708	-0.204487
Ciclev10020471m.g	scaffold_3:6131213-6135849	77.9039	67.6137	-0.20438
Ciclev10031832m.g	scaffold_4:12223265-12227881	18.474	16.0351	-0.204266
Ciclev10025890m.g	scaffold_7:3630002-3631869	12.2278	10.6144	-0.204134
Ciclev10009718m.g	scaffold_1:6391431-6393134	91.1372	79.1137	-0.204113
Ciclev10024329m.g	scaffold_3:41014772-41015915	0.15712	0.136392	-0.204106
Ciclev10005544m.g	scaffold_9:6977143-6980019	3.39858	2.95024	-0.2041
Ciclev10000831m.g	scaffold_5:39888653-39890854	20.3108	17.6322	-0.204037
Ciclev10001347m.g	scaffold_5:2077316-2081952	13.8955	12.064	-0.203908
Ciclev10011316m.g,Ciclev10013707m.g	scaffold_6:21578621-21585884	20.9364	18.1795	-0.203701
Ciclev10019094m.g	scaffold_3:50012590-50015448	12.495	10.8504	-0.203607
Ciclev10001938m.g	scaffold_5:36166729-36168991	43.0084	37.3485	-0.203571
Ciclev10026629m.g	scaffold_7:10787932-10805299	26.5309	23.0402	-0.203521
Ciclev10020902m.g	scaffold_3:17557384-17562695	42.7682	37.1428	-0.203455
Ciclev10006492m.g	scaffold_9:28917643-28926634	0.0788211	0.068458	-0.203362
Ciclev10015039m.g	scaffold_2:32520911-32523641	19.2011	16.68	-0.203068
Ciclev10012958m.g	scaffold_6:25201576-25206677	23.8636	20.7311	-0.203013
Ciclev10014116m.g	scaffold_2:30874329-30882715	7.96218	6.91705	-0.203007
Ciclev10008442m.g	scaffold_1:27088283-27095331	39.4494	34.2724	-0.202956
Ciclev10026315m.g	scaffold_7:6161508-6166276	17.6443	15.3295	-0.202888
Ciclev10016673m.g	scaffold_2:23773011-23775918	12.8372	11.154	-0.202771
Ciclev10002698m.g	scaffold_5:42321790-42327438	3513.47	3053.12	-0.202611
Ciclev10022528m.g	scaffold_3:7759856-7761731	9.67663	8.40909	-0.202555
-	scaffold_9:13697904-13698416	30.8518	26.8115	-0.202506
Ciclev10003782m.g	scaffold_5:4288544-4289115	38.0881	33.1013	-0.20245
Ciclev10030868m.g	scaffold_4:19891311-19894519	2.8461	2.47373	-0.202296
Ciclev10028475m.g	scaffold_8:18160844-18166421	10.5021	9.12874	-0.202184
Ciclev10032836m.g	scaffold_4:1828099-1836003	15.0848	13.1127	-0.20213
Ciclev10029390m.g	scaffold_8:3123061-3124906	39.4611	34.306	-0.201972
Ciclev10027617m.g	scaffold_7:1319136-1320165	43.6196	37.9232	-0.201899
Ciclev10015874m.g	scaffold_2:9205108-9212700	2571.63	2235.8	-0.201894
Ciclev10015257m.g	scaffold_2:12876951-12881843	13.1648	11.447	-0.201719
Ciclev10018557m.g	scaffold_3:9688630-9704133	6.73079	5.85312	-0.201569
Ciclev10026298m.g	scaffold_7:11617461-11620409	8.3417	7.2561	-0.201146
Ciclev10010040m.g	scaffold_1:4861411-4866144	0.291929	0.253939	-0.201137
Ciclev10018756m.g,Ciclev10023926m.g	scaffold_3:21296577-21473295	31.4494	27.3605	-0.200939
Ciclev10014582m.g	scaffold_2:33607181-33613245	11.8135	10.2778	-0.200904
Ciclev10032885m.g	scaffold_4:9451174-9452661	20.9922	18.2636	-0.200877
Ciclev10002854m.g	scaffold_5:40189638-40191021	56.3489	49.0253	-0.200863
Ciclev10001735m.g	scaffold_5:21234823-21242004	122.843	106.884	-0.200767
Ciclev10003455m.g	scaffold_5:40680040-40683114	29.3394	25.5292	-0.200689
Ciclev10029311m.g	scaffold_8:1016745-1020014	19.6873	17.1308	-0.200673

Ciclev10026584m.g	scaffold_7:14584869-14587091	20.8561	18.1491	-0.200573
Ciclev10019102m.g	scaffold_3:46951319-46953737	120.947	105.251	-0.200537
Ciclev10013833m.g	scaffold_6:25170423-25172480	30.2298	26.3126	-0.200219
Ciclev10005270m.g	scaffold_9:29174471-29180718	18.1182	15.7724	-0.200034
Ciclev10022287m.g	scaffold_3:11100713-11104279	30.9774	26.9696	-0.199882
Ciclev10020009m.g	scaffold_3:24620622-24627357	4.13488	3.59993	-0.199879
Ciclev10026018m.g	scaffold_7:9380936-9383214	0.171586	0.149404	-0.199705
Ciclev10000865m.g	scaffold_5:40025747-40029164	30.382	26.4562	-0.199608
Ciclev10014280m.g	scaffold_2:11962602-11970518	39.1857	34.1234	-0.199564
Ciclev10024322m.g	scaffold_3:23815144-23818592	0.249939	0.21765	-0.199563
-	scaffold_2:29322538-29325753	17.1691	14.9516	-0.199511
Ciclev10016073m.g	scaffold_2:36221192-36223732	20.0208	17.4375	-0.199305
Ciclev10007455m.g	scaffold_1:24840899-24850939	54.6242	47.5777	-0.199256
Ciclev10025612m.g	scaffold_7:14859446-14865332	19.4877	16.9738	-0.199256
Ciclev10017333m.g	scaffold_2:405429-407503	0.792034	0.690006	-0.198955
Ciclev10005583m.g	scaffold_9:18862911-18869324	0.0956017	0.0832875	-0.198937
Ciclev10032755m.g	scaffold_4:18426456-18428730	63.6213	55.4271	-0.198918
Ciclev10015287m.g	scaffold_2:24333955-24336070	13.6872	11.9247	-0.198872
Ciclev10020970m.g	scaffold_3:49394417-49398156	20.5286	17.8861	-0.198797
Ciclev10000217m.g	scaffold_5:41058941-41064267	10.7395	9.3573	-0.198764
Ciclev10026993m.g	scaffold_7:9055027-9085218	5.3497	4.66154	-0.198651
Ciclev10021136m.g	scaffold_3:39606430-39608387	13.8478	12.0671	-0.19858
Ciclev10009715m.g	scaffold_1:2735835-2738104	50.8085	44.2772	-0.198504
Ciclev10012063m.g	scaffold_6:18622295-18624116	13.2235	11.5261	-0.198202
Ciclev10032067m.g	scaffold_4:960168-964127	9.68033	8.44017	-0.197784
Ciclev10021639m.g	scaffold_3:38218698-38223171	34.8327	30.3722	-0.197689
Ciclev10014649m.g	scaffold_2:30388459-30393717	55.8491	48.7006	-0.197593
Ciclev10004740m.g	scaffold_9:16230776-16234950	7.6958	6.71104	-0.197534
Ciclev10030064m.g	scaffold_8:23726496-23728470	0.625544	0.54552	-0.197479
Ciclev10020400m.g	scaffold_3:45054693-45057571	21.2673	18.5477	-0.197395
Ciclev10011905m.g	scaffold_6:25547741-25551894	35.0324	30.5569	-0.197191
Ciclev10019706m.g,Ciclev10019727m.g	scaffold_3:42061471-42069114	44.3307	38.668	-0.197168
Ciclev10020090m.g	scaffold_3:6805382-6811056	12.3254	10.7517	-0.19707
Ciclev10018815m.g	scaffold_3:50298540-50306907	5.2786	4.60487	-0.196995
Ciclev10030076m.g	scaffold_8:17291958-17467945	5.32342	4.64446	-0.196841
Ciclev10014995m.g	scaffold_2:33339097-33345390	11.1503	9.72852	-0.196791
Ciclev10006132m.g	scaffold_9:3279502-3283446	20.0304	17.4797	-0.196513
Ciclev10032775m.g	scaffold_4:6602433-6606585	24.7587	21.6086	-0.196333
Ciclev10003264m.g	scaffold_5:3440138-3441861	0.945435	0.825198	-0.196238
Ciclev10009355m.g	scaffold_1:24807941-24811326	71.1049	62.064	-0.196192
Ciclev10018866m.g	scaffold_3:42942190-42951889	50.5365	44.113	-0.196122
Ciclev10021722m.g	scaffold_3:5234417-5238376	15.3863	13.4315	-0.196025
Ciclev10008358m.g	scaffold_1:28017371-28019706	9.75866	8.51926	-0.195955
Ciclev10009870m.g	scaffold_1:16730781-16734515	25.8301	22.5561	-0.195532
Ciclev10000342m.g	scaffold_5:9949177-9958602	17.4313	15.2222	-0.195506
Ciclev10009111m.g	scaffold_1:697202-700509	61.659	53.8478	-0.195423
Ciclev10022690m.g	scaffold_3:50637672-50640392	221.945	193.841	-0.195328
Ciclev10029519m.g	scaffold_8:20190329-20192688	35.2192	30.7633	-0.195151
Ciclev10033175m.g	scaffold_4:23283690-23285802	6.66476	5.82155	-0.195148
Ciclev10013116m.g	scaffold_6:23720687-23722143	18.2867	15.9735	-0.195112
Ciclev10012410m.g	scaffold_6:12270371-12271603	31.5858	27.5923	-0.195008
Ciclev10023077m.g	scaffold_3:3811695-3812447	0.761191	0.664961	-0.19499
Ciclev10009364m.g	scaffold_1:22893475-22896534	56.4752	49.3364	-0.194965
Ciclev10032893m.g	scaffold_4:3636552-3641089	41.6792	36.4115	-0.194934
Ciclev10022896m.g	scaffold_3:3836281-3837122	14.8318	12.9588	-0.194764

Ciclev10010415m.g	scaffold_1:20995351-21034862	0.414445	0.362135	-0.194651
Ciclev10028208m.g	scaffold_8:1713329-1715296	19.1282	16.7143	-0.194621
Ciclev10006358m.g,Ciclev10007074m.g	scaffold_9:15422640-15491415	27.5212	24.0504	-0.194478
Ciclev10026335m.g	scaffold_7:6073755-6077566	9.19155	8.03276	-0.194413
Ciclev10000456m.g	scaffold_5:2200675-2205513	18.5325	16.1971	-0.194323
Ciclev10019627m.g	scaffold_3:43451183-43454649	12.2577	10.7141	-0.194182
Ciclev10022764m.g	scaffold_3:44318458-44320484	10.5456	9.21755	-0.194181
Ciclev10026636m.g	scaffold_7:2606458-2607220	166.991	145.974	-0.194066
Ciclev10005246m.g	scaffold_9:16277122-16282255	10.1353	8.85993	-0.194027
Ciclev10019695m.g,Ciclev10024224m.g	scaffold_3:19310539-19608202	16.829	14.7115	-0.194008
Ciclev10026199m.g	scaffold_7:224442-227133	22.0294	19.259	-0.193898
Ciclev10004932m.g	scaffold_9:4360106-4392598	19.6271	17.1597	-0.193827
Ciclev10014855m.g	scaffold_2:27125174-27129923	26.0009	22.7338	-0.193719
Ciclev10026015m.g	scaffold_7:4103298-4105423	0.329873	0.288436	-0.19366
Ciclev10005848m.g,Ciclev10006835m.g,Ciclev10007073m.g	scaffold_9:17709102-17734147	21.83	19.0888	-0.193584
Ciclev10001525m.g	scaffold_5:42764088-42767443	9.64387	8.4334	-0.193497
Ciclev10006079m.g	scaffold_9:361253-363893	136.982	119.791	-0.193469
Ciclev10028436m.g	scaffold_8:24110297-24112688	5.76727	5.04362	-0.193429
Ciclev10028448m.g	scaffold_8:2468814-2470497	0.125835	0.110046	-0.193428
Ciclev10008447m.g	scaffold_1:4885528-4889782	5.99542	5.24373	-0.193268
Ciclev10025281m.g	scaffold_7:6309801-6312668	0.0876149	0.0766318	-0.193233
Ciclev10005844m.g	scaffold_9:1850387-1853743	38.9069	34.0351	-0.193005
Ciclev10025673m.g	scaffold_7:7346025-7350895	62.6039	54.7766	-0.192692
Ciclev10019619m.g	scaffold_3:44561366-44565521	4.78703	4.1889	-0.192562
Ciclev10002209m.g	scaffold_5:39275626-39277055	164.591	144.029	-0.192523
Ciclev10028994m.g	scaffold_8:4571879-4573091	7.35173	6.4339	-0.19239
Ciclev10019343m.g	scaffold_3:18705027-18711046	8.1326	7.11924	-0.191993
Ciclev10030560m.g,Ciclev10030753m.g	scaffold_4:15324005-15348363	9.34411	8.18077	-0.191821
-	scaffold_9:1120538-1124579	48.0392	42.0611	-0.191726
Ciclev10019840m.g	scaffold_3:50936569-50941281	17.1603	15.0274	-0.191481
Ciclev10007795m.g	scaffold_1:6610590-6616627	4.76498	4.17333	-0.191271
Ciclev10013556m.g	scaffold_6:17900009-17901386	9.58058	8.39116	-0.191242
Ciclev10000690m.g	scaffold_5:25999616-26032103	21.4834	18.8165	-0.191225
Ciclev10026147m.g	scaffold_7:6997914-7000603	62.4535	54.7021	-0.191186
Ciclev10031600m.g	scaffold_4:21336022-21339712	10.6489	9.32761	-0.191129
Ciclev10015702m.g	scaffold_2:32272603-32277829	1.37581	1.20513	-0.191095
Ciclev10006930m.g	scaffold_9:24356661-24541601	0.144439	0.126524	-0.191051
Ciclev10027735m.g	scaffold_8:868560-875279	8.44439	7.39713	-0.191027
Ciclev10022137m.g	scaffold_3:39601127-39604586	65.9373	57.7613	-0.190991
Ciclev10031891m.g	scaffold_4:1358791-1360710	3.0219	2.64733	-0.190914
Ciclev10033505m.g	scaffold_4:6737916-6742257	0.149491	0.130969	-0.19084
Ciclev10020230m.g	scaffold_3:5686859-5691269	5.84655	5.12338	-0.190492
Ciclev10016432m.g	scaffold_2:28403936-28488203	0.780992	0.684397	-0.190473
Ciclev10027575m.g	scaffold_7:5738695-5741413	4.90314	4.29678	-0.19045
Ciclev10009613m.g,Ciclev10009794m.g	scaffold_1:10813488-10826523	45.6327	39.9923	-0.190347
Ciclev10026399m.g	scaffold_7:11136740-11138535	23.2988	20.4198	-0.190291
Ciclev10021445m.g	scaffold_3:8027149-8071028	25.8081	22.6256	-0.189867
Ciclev10013227m.g	scaffold_6:15560181-15560933	8.87765	7.78339	-0.189779
Ciclev10030934m.g	scaffold_4:22447692-22451141	5.18181	4.54351	-0.189648
Ciclev10028730m.g	scaffold_8:1398234-1404988	547.95	480.499	-0.189512

Ciclev10028709m.g	scaffold_8:2218260-2220170	18.1386	15.9098	-0.189152
Ciclev10021620m.g	scaffold_3:39331730-39333098	5.28042	4.63192	-0.189041
Ciclev10015437m.g	scaffold_2:6112766-6115811	25.5443	22.4103	-0.188837
Ciclev10012946m.g	scaffold_6:3081769-3084882	24.8238	21.7793	-0.188769
Ciclev10009318m.g	scaffold_1:67027-70390	161.61	141.79	-0.188756
Ciclev10017606m.g	scaffold_2:27565271-27569859	12.4222	10.8991	-0.188711
Ciclev10010257m.g	scaffold_1:19281524-19284088	0.155848	0.136745	-0.188647
Ciclev10004038m.g	scaffold_5:23190128-23193543	0.779074	0.683604	-0.1886
Ciclev10029552m.g	scaffold_8:19132836-19211350	30.1793	26.4824	-0.188525
Ciclev10007997m.g	scaffold_1:24383593-24387969	8.18652	7.18375	-0.188513
Ciclev10002573m.g	scaffold_5:36106049-36109239	56.0858	49.2165	-0.188493
Ciclev10025144m.g	scaffold_7:3806010-3810954	16.6181	14.586	-0.188175
Ciclev10005291m.g	scaffold_9:31010082-31017820	43.0343	37.7787	-0.187913
Ciclev10010381m.g	scaffold_1:26181272-26186058	9.48929	8.33103	-0.187806
Ciclev10001625m.g	scaffold_5:42297978-42300281	23.2183	20.385	-0.187755
Ciclev10022424m.g	scaffold_3:5759570-5761045	22.0651	19.3735	-0.18768
Ciclev10018666m.g	scaffold_3:29551632-29563218	12.1004	10.6245	-0.187661
-	scaffold_5:6470588-6472531	8.50945	7.47216	-0.187542
Ciclev10014737m.g	scaffold_2:27958036-27961375	31.4909	27.6527	-0.187515
Ciclev10004362m.g	scaffold_9:818267-824080	40.6647	35.7131	-0.187325
Ciclev10014282m.g	scaffold_2:33421079-33423773	6.08637	5.34575	-0.187189
Ciclev10017710m.g	scaffold_2:8499283-8501879	39.9576	35.1008	-0.186967
Ciclev10027218m.g	scaffold_7:5633439-5633887	4.00205	3.51561	-0.186964
Ciclev10027664m.g	scaffold_8:4527771-4547179	7.97558	7.00635	-0.186926
Ciclev10019995m.g	scaffold_3:48412348-48418728	14.6935	12.9089	-0.186805
Ciclev10008504m.g,Ciclev10009360m.g,Ciclev10009694m.g,Ciclev10010566m.g	scaffold_1:13032451-13087281	67.3198	59.1442	-0.186795
Ciclev10009720m.g	scaffold_1:2025697-2028188	9.73354	8.55197	-0.186707
Ciclev10000804m.g	scaffold_5:39693591-39696240	47.6451	41.8661	-0.186544
-	scaffold_8:3257244-3394853	3.72159	3.27031	-0.186491
Ciclev10007642m.g	scaffold_1:1697215-1700458	19.8459	17.4408	-0.18637
Ciclev10009139m.g	scaffold_1:363481-366894	4.71693	4.14589	-0.186168
Ciclev10021732m.g	scaffold_3:40110007-40113420	83.5638	73.4573	-0.185972
Ciclev10032377m.g	scaffold_4:1119180-1120988	1234.93	1085.67	-0.185837
Ciclev10018633m.g	scaffold_3:41553655-41556858	0.749599	0.659013	-0.185811
Ciclev10023047m.g	scaffold_3:1465300-1468453	24.4407	21.4901	-0.185614
Ciclev10003454m.g	scaffold_5:42932101-42934392	0.161243	0.141796	-0.185421
Ciclev10031821m.g,Ciclev10033902m.g	scaffold_4:23404931-23425248	29.1175	25.6136	-0.184977
Ciclev10029532m.g	scaffold_8:22482736-22484522	308.017	271	-0.184717
Ciclev10019226m.g	scaffold_3:1614828-1617871	4.1792	3.67703	-0.184684
Ciclev10014409m.g	scaffold_2:27112543-27120945	35.5301	31.2631	-0.184581
Ciclev10023271m.g	scaffold_3:642196-645175	1.83639	1.61585	-0.18458
Ciclev10021389m.g	scaffold_3:45299456-45303593	51.5053	45.3205	-0.184557
Ciclev10032888m.g	scaffold_4:24255612-24257964	5.36595	4.72176	-0.184509
Ciclev10004521m.g	scaffold_9:870925-875408	56.317	49.5569	-0.184483
Ciclev10011207m.g	scaffold_6:18230244-18235193	31.8221	28.0053	-0.18433
Ciclev10008663m.g	scaffold_1:25241367-25245143	31.1426	27.4074	-0.184328
Ciclev10020100m.g	scaffold_3:1370868-1373669	0.750853	0.660804	-0.184309
Ciclev10030905m.g	scaffold_4:18064848-18071232	15.1116	13.2997	-0.184262
Ciclev10006071m.g	scaffold_9:1747173-1748858	372.954	328.241	-0.184243
Ciclev10016095m.g	scaffold_2:32262312-32265373	13.1865	11.6056	-0.184237
Ciclev10029643m.g	scaffold_8:22882123-22883062	45.4984	40.0448	-0.184202
Ciclev10030582m.g	scaffold_4:24931247-24942450	4.8256	4.24733	-0.184154
Ciclev10020852m.g	scaffold_3:46860730-46864523	32.4638	28.5753	-0.18406

Ciclev10014248m.g	scaffold_2:34455610-34459917	38.9956	34.3347	-0.183645
Ciclev10021165m.g	scaffold_3:2947083-2950009	19.7287	17.3711	-0.183605
Ciclev10021382m.g	scaffold_3:38223844-38228544	16.5317	14.5568	-0.183539
Ciclev10028430m.g	scaffold_8:23885102-23890220	18.7072	16.4741	-0.18339
Ciclev10004448m.g	scaffold_9:17930803-17937481	15.0544	13.2576	-0.18337
Ciclev10020201m.g	scaffold_3:33679134-33682824	3.16192	2.78467	-0.183295
Ciclev10007276m.g	scaffold_1:1440555-1447132	14.1515	12.4635	-0.183246
Ciclev10031950m.g	scaffold_4:16167354-16171545	11.6073	10.2231	-0.183197
Ciclev10000549m.g	scaffold_5:15594240-15601189	10.3936	9.15471	-0.183111
Ciclev10016402m.g	scaffold_2:26669730-26674322	33.6165	29.6112	-0.183027
Ciclev10017627m.g	scaffold_2:17347941-17351501	12.2297	10.7726	-0.183012
Ciclev10000238m.g,Ciclev10004084m.g	scaffold_5:655534-811195	19.4693	17.1499	-0.182999
Ciclev10024989m.g	scaffold_7:7149220-7153371	55.4352	48.8325	-0.182959
Ciclev10005686m.g	scaffold_9:29465748-29470163	17.569	15.4782	-0.182789
Ciclev10028143m.g	scaffold_8:20774567-20778268	18.7865	16.5529	-0.182609
Ciclev10002699m.g	scaffold_5:33381134-33382700	12.8039	11.2847	-0.182212
Ciclev10007442m.g	scaffold_1:8834227-8839676	5.74786	5.0663	-0.18209
Ciclev10017176m.g	scaffold_2:35382238-35384696	63.4326	55.9113	-0.182085
Ciclev10020833m.g	scaffold_3:47080117-47081379	13.8213	12.1826	-0.182075
Ciclev10019894m.g	scaffold_3:2035202-2041798	21.8135	19.2282	-0.182002
Ciclev10033978m.g	scaffold_4:17111497-17118007	1.09929	0.969272	-0.181601
Ciclev10020876m.g	scaffold_3:3615906-3620146	31.3163	27.6124	-0.181597
Ciclev10016378m.g	scaffold_2:33438927-33441556	15.9321	14.048	-0.181571
Ciclev10018744m.g	scaffold_3:717768-722169	16.847	14.8557	-0.181474
Ciclev10001734m.g	scaffold_5:38621879-38625968	266.112	234.679	-0.181344
Ciclev10012248m.g	scaffold_6:16637519-16640347	5.31561	4.68861	-0.181075
Ciclev10024733m.g	scaffold_7:192884-201687	17.9714	15.852	-0.181042
Ciclev10025868m.g	scaffold_7:15269015-15273842	21.7695	19.2022	-0.181042
Ciclev10018806m.g	scaffold_3:47230362-47236093	4.26735	3.76458	-0.180851
-	scaffold_7:10731560-10733262	17.6375	15.5599	-0.180819
Ciclev10009421m.g	scaffold_1:28667630-28670696	6.44665	5.68734	-0.180796
Ciclev10028672m.g	scaffold_8:356240-360220	36.4819	32.1863	-0.180732
Ciclev10020161m.g	scaffold_3:48295516-48299459	245.685	216.774	-0.180622
Ciclev10013335m.g	scaffold_6:8093359-8096237	160.674	141.794	-0.180335
Ciclev10020271m.g	scaffold_3:23294401-23299117	11.3191	9.98912	-0.180331
Ciclev10029553m.g	scaffold_8:25009628-25010885	4.42197	3.90257	-0.180267
Ciclev10023111m.g	scaffold_3:49246985-49248965	28.7045	25.3332	-0.180249
Ciclev10026741m.g	scaffold_7:1324163-1325758	61.2471	54.0574	-0.18015
Ciclev10029674m.g	scaffold_8:4948814-4949917	22.2996	19.6833	-0.180043
Ciclev10001264m.g	scaffold_5:34784367-34786120	17.1706	15.1562	-0.180038
Ciclev10024904m.g	scaffold_7:17380315-17385501	12.1139	10.6932	-0.179971
Ciclev10011249m.g	scaffold_6:23410635-23414246	22.0502	19.4647	-0.17993
Ciclev10018879m.g	scaffold_3:13226415-13233923	18.698	16.5059	-0.179898
Ciclev10004594m.g	scaffold_9:892256-896653	13.4723	11.8931	-0.179881
Ciclev10027592m.g	scaffold_7:651059-653230	1.37654	1.2152	-0.179853
Ciclev10012460m.g,Ciclev10013658m.g	scaffold_6:19680061-19686740	13.5574	11.9691	-0.179767
Ciclev10026862m.g	scaffold_7:1924001-1925583	178.233	157.401	-0.17932
Ciclev10003582m.g	scaffold_5:8745667-8989247	30.2265	26.6972	-0.179127
Ciclev10001688m.g	scaffold_5:32925602-32929147	53.346	47.1266	-0.178838
Ciclev10026739m.g	scaffold_7:5523074-5524475	1505.68	1330.18	-0.178793
Ciclev10022492m.g	scaffold_3:1069368-1070035	58.2197	51.4393	-0.178638
Ciclev10025234m.g	scaffold_7:7038918-7044450	8.42139	7.44117	-0.17853
Ciclev10032895m.g	scaffold_4:18207504-18209407	42.9108	37.9193	-0.178408
Ciclev10001344m.g	scaffold_5:25876760-25880898	1250.06	1104.73	-0.178304

Ciclev10021246m.g	scaffold_3:38135687-38139496	15.8473	14.005	-0.178292
Ciclev10002598m.g	scaffold_5:39682995-39692504	24.8191	21.9344	-0.178257
Ciclev10031926m.g	scaffold_4:16180410-16182110	5.93528	5.24551	-0.178232
Ciclev10014998m.g	scaffold_2:16597795-16602257	49.0006	43.3064	-0.178221
Ciclev10031433m.g	scaffold_4:23969751-23972108	1.29462	1.14422	-0.17816
Ciclev10020753m.g	scaffold_3:12376712-12381097	31.9295	28.2244	-0.177946
Ciclev10014482m.g	scaffold_2:23889636-23891706	4.98928	4.41099	-0.177728
Ciclev10002361m.g	scaffold_5:12704907-12708437	41.5913	36.7735	-0.177614
Ciclev10033038m.g	scaffold_4:22685898-22688912	101.937	90.1409	-0.177417
Ciclev10014353m.g	scaffold_2:11522686-11529458	45.2962	40.056	-0.177373
Ciclev10031923m.g	scaffold_4:21389145-21402033	7.43675	6.57716	-0.177208
Ciclev10000923m.g	scaffold_5:26496114-26502834	20.2209	17.8841	-0.177168
Ciclev10003235m.g	scaffold_5:31333200-31391489	1.53484	1.35759	-0.177045
Ciclev10001569m.g	scaffold_5:21545136-21549647	14.0989	12.4709	-0.177016
Ciclev10025319m.g	scaffold_7:10766583-10772072	15.1885	13.4373	-0.176731
Ciclev10009779m.g	scaffold_1:4269207-4270164	55.4332	49.0428	-0.176709
Ciclev10022000m.g	scaffold_3:5339486-5341137	22.7697	20.1448	-0.176708
Ciclev10033336m.g	scaffold_4:4721499-4727886	0.279328	0.247213	-0.176207
Ciclev10004785m.g	scaffold_9:20759792-20767162	80.0499	70.8534	-0.176063
Ciclev10009218m.g	scaffold_1:2155051-2156056	6.6448	5.88163	-0.176009
Ciclev10007180m.g	scaffold_9:25806603-25807599	0.804486	0.712251	-0.17568
Ciclev10016942m.g	scaffold_2:27762133-27765622	80.8539	71.5858	-0.175644
Ciclev10007777m.g	scaffold_1:24285933-24289230	13.5225	11.9746	-0.175377
Ciclev10004955m.g,Ciclev10006320m.g	scaffold_9:25922688-25937792	18.6075	16.4841	-0.174812
Ciclev10002706m.g	scaffold_5:37884978-37885975	24.5772	21.7729	-0.174785
Ciclev10027795m.g	scaffold_8:5950468-5956596	8.07536	7.15423	-0.174731
Ciclev10031324m.g	scaffold_4:250673-254183	45.4057	40.228	-0.174675
Ciclev10009895m.g	scaffold_1:7550601-7551330	10.0767	8.92778	-0.174656
Ciclev10012132m.g,Ciclev10013214m.g	scaffold_6:9034906-9043358	76.3673	67.6606	-0.174638
Ciclev10002639m.g	scaffold_5:31300142-31302469	27.5614	24.4213	-0.174512
Ciclev10020955m.g,Ciclev10020957m.g	scaffold_3:44886488-44897661	36.5179	32.3687	-0.174005
Ciclev10016740m.g	scaffold_2:29051023-29053661	7.66186	6.79151	-0.173961
Ciclev10012616m.g	scaffold_6:19718530-19720586	25.8116	22.8807	-0.173893
Ciclev10012101m.g	scaffold_6:18810580-18812327	49.1315	43.553	-0.173878
Ciclev10012301m.g	scaffold_6:19493227-19496277	0.495996	0.439863	-0.173273
Ciclev10000359m.g	scaffold_5:22227782-22263681	13.4059	11.8898	-0.173149
Ciclev10001686m.g	scaffold_5:42915820-42918272	41.3922	36.7152	-0.172981
Ciclev10002126m.g	scaffold_5:9968943-9975353	42.7643	37.9325	-0.172972
Ciclev10000854m.g	scaffold_5:38085341-38087688	2.75743	2.44589	-0.172966
Ciclev10007420m.g	scaffold_1:22660740-22664920	18.4484	16.3649	-0.172891
Ciclev10002261m.g	scaffold_5:38420274-38423903	92.5153	82.0725	-0.172793
Ciclev10018544m.g	scaffold_3:28544725-28549488	0.0487179	0.0432238	-0.172626
Ciclev10010834m.g	scaffold_1:881841-883786	5.30469	4.70798	-0.172161
Ciclev10008661m.g	scaffold_1:1762846-1768718	1.72075	1.52752	-0.171849
Ciclev10027390m.g,Ciclev10027529m.g	scaffold_7:14835112-14837703	17.103	15.1846	-0.171641
Ciclev10007828m.g	scaffold_1:17529725-17534811	4.84368	4.30122	-0.17136
Ciclev10014527m.g	scaffold_2:27009600-27014648	37.1884	33.0235	-0.171359
Ciclev10030535m.g	scaffold_4:24588405-24594706	11.8852	10.5544	-0.171324
Ciclev10017101m.g	scaffold_2:15432098-15433385	246.887	219.278	-0.171086
Ciclev10005128m.g	scaffold_9:4556262-4561773	26.8073	23.8099	-0.171064
Ciclev10029604m.g	scaffold_8:2031342-2034652	62.909	55.8761	-0.171036
Ciclev10021131m.g	scaffold_3:29442088-29445067	19.6967	17.4952	-0.170996

Ciclev10002562m.g	scaffold_5:41163521-41166013	19.1954	17.0508	-0.170917
Ciclev10000138m.g	scaffold_5:32250437-32256614	10.7056	9.51038	-0.170787
-	scaffold_1:3767269-3771394	249.337	221.533	-0.170577
Ciclev10022301m.g	scaffold_3:7364479-7367864	28.4993	25.3224	-0.170513
Ciclev10026469m.g	scaffold_7:1297658-1300335	10.3416	9.18906	-0.170472
Ciclev10026591m.g	scaffold_7:11131540-11135740	36.3987	32.3438	-0.170395
Ciclev10012570m.g	scaffold_6:20651753-20654352	26.2036	23.2853	-0.170347
Ciclev10009483m.g	scaffold_1:23022568-23025718	89.9179	79.9102	-0.170229
Ciclev10016687m.g	scaffold_2:36277702-36279976	79.9905	71.089	-0.170202
Ciclev10007888m.g	scaffold_1:3603607-3608662	33.6963	29.9483	-0.170115
Ciclev10023407m.g,Ciclev10023745m.g,Ciclev10023805m.g	scaffold_3:46519382-46595230	64.2683	57.1207	-0.170094
Ciclev10018688m.g	scaffold_3:25100777-25104843	1.49935	1.3326	-0.170085
Ciclev10011248m.g	scaffold_6:21804905-21811325	16.2291	14.4256	-0.169951
Ciclev10020322m.g	scaffold_3:48753306-48756857	195.613	173.908	-0.169673
-	scaffold_3:3579467-3581004	138.724	123.337	-0.169612
Ciclev10016987m.g	scaffold_2:12359078-12359874	7.01189	6.23595	-0.169195
Ciclev10010588m.g	scaffold_1:13128080-13135994	39.2615	34.9183	-0.16913
Ciclev10013218m.g	scaffold_6:11070233-11072271	173.176	154.029	-0.16904
-	scaffold_3:28065477-28341901	38.1951	33.9734	-0.16898
Ciclev10026619m.g	scaffold_7:5805229-5807265	30.8636	27.4535	-0.168916
Ciclev10026125m.g	scaffold_7:15740196-15745512	23.8786	21.2432	-0.168717
Ciclev10011307m.g	scaffold_6:17471789-17475553	185.44	164.98	-0.16866
Ciclev10017692m.g	scaffold_2:9275177-9278258	37.3249	33.2114	-0.168459
Ciclev10005222m.g	scaffold_9:28854427-28855863	28.2063	25.1012	-0.168261
Ciclev10011645m.g	scaffold_6:17762613-17767198	10.8421	9.64952	-0.168116
Ciclev10002788m.g	scaffold_5:9976756-9977512	25.0495	22.2951	-0.168055
Ciclev10012329m.g	scaffold_6:12790588-12792391	41.4034	36.8546	-0.167904
Ciclev10026087m.g	scaffold_7:5617821-5621433	20.5023	18.2501	-0.167882
Ciclev10008279m.g	scaffold_1:28026841-28029049	13.341	11.8766	-0.167753
Ciclev10014074m.g	scaffold_2:7409933-7416233	0.0501575	0.0446549	-0.167648
Ciclev10027946m.g	scaffold_8:239206-242555	13.2347	11.7856	-0.167299
Ciclev10000476m.g	scaffold_5:34994036-35002166	16.6253	14.8053	-0.167274
Ciclev10005599m.g	scaffold_9:3660354-3663506	46.5093	41.418	-0.16726
Ciclev10012077m.g	scaffold_6:20524744-20531628	21.3855	19.0448	-0.167235
Ciclev10001434m.g	scaffold_5:40508146-40511842	39.8499	35.4901	-0.167159
Ciclev10033967m.g	scaffold_4:18246158-18250435	2.29078	2.04034	-0.167035
Ciclev10007752m.g	scaffold_1:406500-412982	18.0052	16.0369	-0.167019
Ciclev10018407m.g	scaffold_2:10723490-10725303	29.4504	26.2318	-0.166972
Ciclev10011527m.g	scaffold_6:1854279-1862782	36.8334	32.809	-0.166921
Ciclev10013737m.g	scaffold_6:19159909-19165528	17.3615	15.465	-0.166877
Ciclev10029203m.g	scaffold_8:23002628-23005065	4.30212	3.83268	-0.166695
Ciclev10018931m.g	scaffold_3:20916726-20923032	81.0918	72.2468	-0.166623
Ciclev10028704m.g	scaffold_8:24711490-24717436	10.2796	9.15894	-0.166531
Ciclev10025681m.g	scaffold_7:14641023-14645051	66.0637	58.863	-0.166496
Ciclev10016962m.g	scaffold_2:33930536-33932493	8.72139	7.77179	-0.166311
Ciclev10026979m.g	scaffold_7:9733633-9808728	6.26288	5.58115	-0.166263
Ciclev10017427m.g	scaffold_2:31211055-31225713	8.27193	7.37166	-0.166234
-	scaffold_2:34748940-34752517	19.4951	17.374	-0.166181
Ciclev10022007m.g	scaffold_3:49359634-49366836	102.354	91.2221	-0.166114
Ciclev10011516m.g	scaffold_6:23722796-23725939	6.54444	5.83273	-0.166098
Ciclev10024699m.g	scaffold_7:3310361-3322676	0.608273	0.542182	-0.165941
Ciclev10000593m.g	scaffold_5:38658342-38661581	15.5195	13.8339	-0.16587
Ciclev10031975m.g	scaffold_4:2451344-2457577	41.8564	37.3109	-0.165851
Ciclev10005932m.g	scaffold_9:4708309-4714548	32.4221	28.9026	-0.165776

-	scaffold_2:33308039-33313042	12.4739	11.1209	-0.165632
Ciclev10022653m.g	scaffold_3:39834679-39837971	47.1298	42.0194	-0.165586
Ciclev10019553m.g	scaffold_3:39823221-39829331	38.4204	34.2583	-0.165419
Ciclev10006686m.g	scaffold_9:3422909-3427718	30.7655	27.4329	-0.165407
Ciclev10003036m.g	scaffold_5:40634800-40636508	4.11283	3.66765	-0.165276
Ciclev10029515m.g	scaffold_8:18954401-18956282	687.209	612.891	-0.165119
Ciclev10026656m.g	scaffold_7:3690912-3692053	1.58774	1.41618	-0.164966
Ciclev10016586m.g	scaffold_2:34699890-34702062	93.4073	83.3156	-0.16495
Ciclev10004319m.g	scaffold_9:967687-981800	28.6707	25.5736	-0.164919
Ciclev10009981m.g	scaffold_1:957780-963845	35.1094	31.3216	-0.1647
Ciclev10032127m.g	scaffold_4:11782183-11783387	28.4202	25.3564	-0.164566
Ciclev10031714m.g	scaffold_4:556536-560714	55.2337	49.2804	-0.164536
Ciclev10003616m.g	scaffold_5:847952-855377	18.8201	16.7933	-0.164392
Ciclev10009328m.g	scaffold_1:6230232-6239600	81.0087	72.29	-0.16428
Ciclev10022673m.g	scaffold_3:41762939-41765791	136.201	121.543	-0.164264
Ciclev10007798m.g	scaffold_1:16735859-16738877	20.4656	18.2638	-0.16421
Ciclev10024225m.g	scaffold_3:8626324-8631026	8.78872	7.84444	-0.163982
-	scaffold_8:3526886-3528408	24.2609	21.6564	-0.163841
Ciclev10025253m.g	scaffold_7:17802097-17807441	13.5529	12.0989	-0.163724
Ciclev10008427m.g	scaffold_1:25037027-25040422	16.5976	14.8196	-0.163461
-	scaffold_4:11029739-11031627	6.24325	5.57474	-0.163393
Ciclev10026014m.g	scaffold_7:94154-97631	42.8383	38.254	-0.163289
Ciclev10022174m.g	scaffold_3:3339666-3344735	41.1594	36.7615	-0.163027
Ciclev10017887m.g	scaffold_2:15105671-15106321	2.14618	1.917	-0.162922
Ciclev10030811m.g	scaffold_4:1770346-1774209	17.8026	15.9021	-0.162868
Ciclev10010014m.g	scaffold_1:18709720-18711349	22.3288	19.9459	-0.162813
Ciclev10004858m.g	scaffold_9:26981497-26984277	15.2074	13.5846	-0.1628
Ciclev10027093m.g	scaffold_7:5393033-5397048	2.246	2.00636	-0.16278
Ciclev10026582m.g	scaffold_7:14773830-14775350	9.69241	8.65994	-0.162499
Ciclev10028219m.g	scaffold_8:22098773-22103225	19.126	17.0893	-0.162438
Ciclev10016852m.g	scaffold_2:9321323-9322241	528.547	472.285	-0.162374
Ciclev10005584m.g	scaffold_9:28651828-28654472	17.0026	15.1947	-0.162188
-	scaffold_7:9336417-9336701	51.5578	46.0797	-0.162061
Ciclev10021032m.g	scaffold_3:44018159-44021078	124.226	111.046	-0.161805
Ciclev10026066m.g,Ciclev10026144m.g	scaffold_7:15376817-15382697	21.2518	18.9976	-0.161768
Ciclev10023436m.g	scaffold_3:44834034-44836704	6.88257	6.15319	-0.161613
Ciclev10033145m.g	scaffold_4:18897762-18901255	158.946	142.103	-0.161598
Ciclev10008244m.g	scaffold_1:2508979-2511795	9.44766	8.44677	-0.161557
Ciclev10014079m.g	scaffold_2:28237698-28249210	6.44308	5.76104	-0.161421
Ciclev10020168m.g	scaffold_3:47707384-47711982	129.193	115.522	-0.161355
Ciclev10019979m.g	scaffold_3:3963023-3966628	56.8999	50.8809	-0.161303
Ciclev10031570m.g	scaffold_4:19572950-19576064	4.14871	3.70991	-0.161278
Ciclev10019386m.g	scaffold_3:47767460-47776185	20.1277	18.0002	-0.161167
Ciclev10001275m.g	scaffold_5:41140492-41143801	48.8303	43.6721	-0.161065
Ciclev10003484m.g	scaffold_5:28289811-28536377	47.6279	42.5979	-0.161024
Ciclev10001489m.g	scaffold_5:33805329-33808164	47.1129	42.1375	-0.161018
Ciclev10009657m.g	scaffold_1:27046427-27049188	395.183	353.463	-0.160963
Ciclev10028890m.g	scaffold_8:5459390-5461591	11.9699	10.7072	-0.160826
Ciclev10022359m.g	scaffold_3:2292398-2293655	424.639	379.856	-0.160784
Ciclev10027828m.g	scaffold_8:6146652-6154380	76.0336	68.0175	-0.160732
Ciclev10025066m.g	scaffold_7:8400363-8405866	17.5812	15.728	-0.160698
Ciclev10022090m.g	scaffold_3:46924691-46927004	47.6525	42.6306	-0.160663
Ciclev10011853m.g	scaffold_6:9447684-9454144	14.6833	13.1362	-0.160625
Ciclev10016574m.g	scaffold_2:6880110-6883726	146.972	131.514	-0.16032
Ciclev10029365m.g	scaffold_8:4461644-4464561	199.751	178.755	-0.160221

Ciclev10009311m.g	scaffold_1:26979381-26983460	14.0322	12.5576	-0.160182
Ciclev10008158m.g	scaffold_1:23049612-23053555	38.3119	34.2945	-0.159814
Ciclev10019258m.g	scaffold_3:50570867-50578436	8.5215	7.63113	-0.159211
Ciclev10000847m.g	scaffold_5:38393281-38398923	14.1397	12.6631	-0.159123
Ciclev10029386m.g	scaffold_8:8176370-8177435	1218.95	1091.74	-0.159013
Ciclev10029536m.g	scaffold_8:17529062-17529928	27.6919	24.8038	-0.158904
Ciclev10025974m.g	scaffold_7:6572707-6577937	14.0657	12.5996	-0.158805
Ciclev10020175m.g	scaffold_3:45679580-45685821	22.3057	19.9826	-0.158668
Ciclev10006154m.g	scaffold_9:3708207-3710264	50.2051	44.9771	-0.158644
Ciclev10000240m.g	scaffold_5:42934683-42937662	48.4956	43.4463	-0.158621
Ciclev10009764m.g	scaffold_1:883901-885880	42.5319	38.1039	-0.158606
-	scaffold_4:14859835-14860346	5.62843	5.04253	-0.158585
Ciclev10007404m.g	scaffold_1:21471414-21476261	3.85355	3.4526	-0.158506
Ciclev10031837m.g	scaffold_4:23156234-23160869	9.89007	8.86127	-0.158468
Ciclev10002309m.g	scaffold_5:34843503-34848399	28.3466	25.4039	-0.158123
Ciclev10019362m.g	scaffold_3:30515492-30520783	53.4337	47.8887	-0.158064
Ciclev10018941m.g	scaffold_3:3423785-3427132	1.40061	1.25529	-0.15803
Ciclev10004290m.g	scaffold_9:28658176-28664383	42.0808	37.7182	-0.157902
Ciclev10004647m.g	scaffold_9:28802067-28807915	14.7987	13.2646	-0.157884
Ciclev10015516m.g	scaffold_2:4443845-4450419	105.893	94.9294	-0.157684
Ciclev10005470m.g	scaffold_9:20360609-20494992	4.8967	4.39008	-0.157564
Ciclev10004968m.g,Ciclev10006373m.g,Ciclev10006658m.g	scaffold_9:18155113-18179752	56.5791	50.7261	-0.157543
Ciclev10016100m.g	scaffold_2:30036935-30039324	13.1469	11.7882	-0.157383
Ciclev10011863m.g	scaffold_6:4708318-4713203	24.0734	21.5864	-0.157319
Ciclev10010730m.g	scaffold_1:863426-878303	80.0805	71.8093	-0.15728
Ciclev10014033m.g	scaffold_2:9636152-9651661	21.3692	19.1637	-0.157154
Ciclev10031012m.g	scaffold_4:9871253-9874701	36.9127	33.1042	-0.157102
Ciclev10020585m.g	scaffold_3:2992219-2995911	20.0307	17.9656	-0.156979
Ciclev10026411m.g	scaffold_7:1601261-1603457	41.1343	36.895	-0.156917
Ciclev10014057m.g	scaffold_2:4199571-4207315	19.3844	17.387	-0.15689
Ciclev10006055m.g	scaffold_9:3711079-3711717	45.5263	40.836	-0.156859
Ciclev10033129m.g	scaffold_4:3675936-3677767	20.1752	18.0977	-0.156775
Ciclev10026337m.g	scaffold_7:76590-79898	17.6969	15.8748	-0.156764
Ciclev10031963m.g	scaffold_4:23998362-24003075	5.88349	5.27776	-0.156745
Ciclev10022320m.g	scaffold_3:48839563-48844650	38.9678	34.956	-0.156743
Ciclev10009203m.g	scaffold_1:18813635-18816503	86.1997	77.331	-0.156636
Ciclev10011638m.g	scaffold_6:24825165-24829196	1.78059	1.59747	-0.156568
Ciclev10021128m.g	scaffold_3:40145088-40146670	20.7609	18.6263	-0.156528
Ciclev10001810m.g	scaffold_5:36018226-36021021	1.77405	1.59174	-0.156447
Ciclev10024867m.g	scaffold_7:11567904-11575213	100.136	89.867	-0.156098
Ciclev10025600m.g	scaffold_7:2255288-2259852	21.532	19.3262	-0.155922
Ciclev10025398m.g	scaffold_7:6272363-6281579	18.0259	16.1799	-0.15587
Ciclev10003010m.g	scaffold_5:226515-230191	0.448522	0.402614	-0.155784
Ciclev10015088m.g	scaffold_2:5011911-5016026	36.9689	33.189	-0.155608
Ciclev10028976m.g	scaffold_8:4558900-4567593	15.2051	13.6506	-0.1556
Ciclev10017811m.g	scaffold_2:12745771-12748867	62.8673	56.44	-0.155593
Ciclev10025591m.g	scaffold_7:6127463-6131417	67.5559	60.6508	-0.155556
Ciclev10030556m.g	scaffold_4:138715-145809	11.411	10.2452	-0.155464
Ciclev10032183m.g	scaffold_4:2548168-2551230	16.4527	14.7749	-0.155177
Ciclev10013208m.g	scaffold_6:24881053-24884358	42.8047	38.441	-0.155122
Ciclev10033727m.g	scaffold_4:20682086-20687196	8.75988	7.86806	-0.154904
Ciclev10005935m.g	scaffold_9:19571264-19577393	3.87636	3.48213	-0.154735
Ciclev10002351m.g	scaffold_5:39619674-39626261	318.914	286.512	-0.154574
Ciclev10016552m.g	scaffold_2:11739858-11742644	14.0651	12.6365	-0.154528

Ciclev10032726m.g	scaffold_4:1755430-1757505	31.1	27.9457	-0.154287
Ciclev10002260m.g,Ciclev10004072m.g	scaffold_5:398574-411055	76.5725	68.823	-0.153936
Ciclev10029282m.g	scaffold_8:11113258-11117344	63.4154	57.0002	-0.153867
Ciclev10017332m.g,Ciclev10017335m.g	scaffold_2:11754293-11785844	130.465	117.272	-0.153794
Ciclev10032054m.g	scaffold_4:24289940-24293701	46.6038	41.892	-0.153772
Ciclev10002935m.g	scaffold_5:32441852-32442840	0.659001	0.59243	-0.153634
Ciclev10014755m.g	scaffold_2:23987529-23993442	10.1239	9.10129	-0.153619
Ciclev10033185m.g	scaffold_4:16817583-16984703	1349.79	1213.62	-0.153412
Ciclev10011757m.g,Ciclev10012614m.g	scaffold_6:18112183-18134557	20.9663	18.8525	-0.153316
Ciclev10019372m.g	scaffold_3:10933405-10938651	8.25623	7.42503	-0.153087
Ciclev10013285m.g	scaffold_6:16582498-16587420	186.927	168.114	-0.153038
Ciclev10028238m.g	scaffold_8:22668524-22673498	107.865	97.0102	-0.153012
Ciclev10006280m.g	scaffold_9:30902733-30904789	11.8374	10.6471	-0.15289
Ciclev10004340m.g	scaffold_9:7500356-7510739	12.7847	11.5001	-0.15277
Ciclev10004268m.g	scaffold_9:15624982-15638532	17.3571	15.6136	-0.152725
Ciclev10009624m.g	scaffold_1:17842170-17843617	898.206	808	-0.152692
Ciclev10033174m.g	scaffold_4:21240348-21241064	603.073	542.653	-0.152303
Ciclev10019014m.g	scaffold_3:43097277-43103724	17.7445	15.9675	-0.152225
Ciclev10024911m.g	scaffold_7:4805544-4809377	0.729078	0.656085	-0.15219
Ciclev10028625m.g	scaffold_8:24855941-24859986	52.1373	46.9191	-0.152139
Ciclev10005702m.g	scaffold_9:22712687-22715627	84.2826	75.8478	-0.152127
Ciclev10001084m.g	scaffold_5:39477781-39484776	21.007	18.9051	-0.152092
Ciclev10021441m.g	scaffold_3:6437053-6441117	20.3552	18.319	-0.152054
-	scaffold_3:42119753-42121529	126.466	113.821	-0.151972
Ciclev10010743m.g	scaffold_1:26866471-26869582	1.12519	1.01281	-0.151807
Ciclev10025639m.g	scaffold_7:3372252-3375541	9.73905	8.76652	-0.151778
Ciclev10011102m.g	scaffold_6:14228462-14237028	28.1269	25.3184	-0.151766
Ciclev10025866m.g	scaffold_7:17201235-17204282	152.003	136.83	-0.151722
Ciclev10017607m.g	scaffold_2:35960452-35963542	60.4665	54.4309	-0.151709
Ciclev10015414m.g	scaffold_2:6181064-6338878	17.7925	16.0168	-0.151684
Ciclev10032207m.g	scaffold_4:25514792-25517935	35.1274	31.6235	-0.151599
Ciclev10029448m.g	scaffold_8:37657-40607	22.2051	19.9909	-0.15155
Ciclev10026134m.g	scaffold_7:894835-899277	18.0639	16.2631	-0.151504
Ciclev10030509m.g	scaffold_4:18155356-18168835	8.1009	7.29352	-0.151467
Ciclev10009738m.g	scaffold_1:21862691-21865186	41.5477	37.4105	-0.151325
-	scaffold_3:43194885-43199062	37.1037	33.4105	-0.151261
-	scaffold_8:23840586-23840730	1057.04	951.83	-0.151258
Ciclev10013255m.g,Ciclev10013894m.g	scaffold_6:25255394-25263103	28.1412	25.341	-0.151214
Ciclev10014906m.g	scaffold_2:27641544-27644092	7.49617	6.75027	-0.151209
Ciclev10001494m.g	scaffold_5:34251120-34255285	692.425	623.526	-0.151208
Ciclev10013089m.g	scaffold_6:19676467-19678707	39.7161	35.7715	-0.150914
Ciclev10015057m.g	scaffold_2:36205158-36211643	25.6005	23.0581	-0.150901
Ciclev10021273m.g	scaffold_3:8494351-8498796	22.2561	20.0463	-0.150864
Ciclev10008313m.g	scaffold_1:4732723-4736490	30.845	27.7849	-0.15074
Ciclev10011510m.g	scaffold_6:19536844-19540748	13.3681	12.0426	-0.150649
Ciclev10024352m.g	scaffold_3:3666374-3669279	0.58034	0.522855	-0.150489
Ciclev10022860m.g	scaffold_3:46242494-46243557	32.6499	29.4158	-0.150486
Ciclev10017167m.g	scaffold_2:34841951-34844319	121.99	109.908	-0.150465
Ciclev10028566m.g	scaffold_8:20079719-20084101	136.818	123.273	-0.1504
Ciclev10019901m.g	scaffold_3:49608021-49613129	16.4365	14.8098	-0.150356
Ciclev10001056m.g,Ciclev10002300m.g	scaffold_5:27798445-27812705	11.8678	10.6934	-0.150333

Ciclev10004468m.g	scaffold_9:25135830-25143404	84.7604	76.3775	-0.150244
Ciclev10015641m.g	scaffold_2:34370577-34373817	17.1629	15.4656	-0.150228
Ciclev10020406m.g	scaffold_3:44866567-44869888	13.676	12.3238	-0.150203
Ciclev10015549m.g	scaffold_2:213637-217240	13.2149	11.9102	-0.149966
Ciclev10025496m.g	scaffold_7:5703010-5709503	21.3882	19.2771	-0.149928
Ciclev10019018m.g	scaffold_3:24785317-24795064	20.4817	18.4637	-0.149645
Ciclev10028342m.g	scaffold_8:19493343-19498791	12.8451	11.5797	-0.149624
Ciclev10031543m.g	scaffold_4:291774-294454	4.32443	3.89843	-0.149619
Ciclev10015763m.g	scaffold_2:4036386-4040860	4.0349	3.63747	-0.149596
Ciclev10023573m.g	scaffold_3:2684071-2687087	64.8911	58.5045	-0.149475
Ciclev10031564m.g,Ciclev10033771m.g	scaffold_4:9624052-9633617	19.8888	17.932	-0.14942
Ciclev10030747m.g	scaffold_4:18195036-18200527	7.02758	6.33667	-0.149303
Ciclev10025028m.g	scaffold_7:8488323-8494781	31.1815	28.1201	-0.149088
Ciclev10011396m.g	scaffold_6:23628745-23631556	13.9759	12.6052	-0.14892
Ciclev10000405m.g	scaffold_5:37626148-37632397	29.793	26.8724	-0.148851
Ciclev10009418m.g	scaffold_1:3444866-3446616	44.4178	40.0639	-0.148832
Ciclev10017510m.g	scaffold_2:29644506-29646280	168.81	152.264	-0.148828
Ciclev10026845m.g	scaffold_7:6798934-6802042	40.6089	36.6286	-0.148826
Ciclev10018658m.g	scaffold_3:50353531-50358006	7.22099	6.5138	-0.148698
Ciclev10001914m.g	scaffold_5:41555117-41556812	2.4768	2.23427	-0.14867
Ciclev10015274m.g	scaffold_2:7006668-7012033	10.6582	9.61467	-0.148653
Ciclev10032522m.g	scaffold_4:15082029-15083114	17.2497	15.5616	-0.14858
-	scaffold_4:18510567-18511000	6.1158	5.51815	-0.148357
Ciclev10019719m.g	scaffold_3:45746799-45751646	8.99039	8.11194	-0.148337
Ciclev10009449m.g	scaffold_1:4857159-4860226	6.15648	5.55586	-0.148095
Ciclev10025033m.g	scaffold_7:1797393-1800284	2.17903	1.96667	-0.147937
-	scaffold_5:34103148-34115415	55.6902	50.2689	-0.147758
Ciclev10014295m.g	scaffold_2:14657848-14665608	2.7892	2.51782	-0.147673
Ciclev10018617m.g	scaffold_3:45459831-45467484	22.8875	20.6617	-0.1476
Ciclev10009354m.g	scaffold_1:20550791-20553673	153.35	138.44	-0.14757
Ciclev10002397m.g	scaffold_5:35651950-35654845	8.61737	7.78057	-0.147371
Ciclev10030748m.g	scaffold_4:18337788-18350824	11.6188	10.4922	-0.147149
Ciclev10020287m.g	scaffold_3:49687407-49690302	8.49879	7.67617	-0.146872
Ciclev10009167m.g	scaffold_1:5368174-5370161	198.03	178.866	-0.146834
Ciclev10025374m.g	scaffold_7:593833-598812	18.1323	16.3781	-0.146792
Ciclev10004924m.g	scaffold_9:7691198-7698485	46.744	42.2223	-0.146776
Ciclev10019973m.g	scaffold_3:32093657-32097501	16.4448	14.855	-0.146678
Ciclev10024401m.g	scaffold_3:4348757-4351532	13.871	12.5301	-0.146672
Ciclev10021328m.g	scaffold_3:45215825-45219396	15.7884	14.2633	-0.146559
Ciclev10025285m.g	scaffold_7:3742498-3751655	31.8013	28.7321	-0.146421
Ciclev10011064m.g	scaffold_6:17390300-17397359	102.99	93.0611	-0.146255
Ciclev10003084m.g	scaffold_5:11913693-11914695	63.963	57.8156	-0.145779
Ciclev10019902m.g	scaffold_3:32084280-32089458	18.726	16.9265	-0.145753
Ciclev10020260m.g	scaffold_3:45616183-45621279	22.4006	20.2491	-0.145679
Ciclev10013906m.g	scaffold_6:12631172-12634698	32.2002	29.1113	-0.14549
-	scaffold_16:66506-82324	12.0164	10.8638	-0.145483
Ciclev10022811m.g	scaffold_3:12901462-12904143	87.3339	78.9576	-0.145463
Ciclev10026832m.g	scaffold_7:8693649-8696481	71.6991	64.8246	-0.145413
Ciclev10014592m.g	scaffold_2:8084905-8092367	4.71167	4.26029	-0.145288
Ciclev10029330m.g	scaffold_8:18806443-18814838	262.395	237.275	-0.145179
Ciclev10016736m.g	scaffold_2:25753623-25755610	31.9716	28.9123	-0.145111
Ciclev10014022m.g,Ciclev10015005m.g,Ciclev10017580m.g	scaffold_2:10424778-10472483	12.6459	11.4363	-0.145054
Ciclev10022530m.g	scaffold_3:42680982-42684147	34.1503	30.8844	-0.14502

Ciclev10027412m.g	scaffold_7:8425981-8428053	1.90271	1.72106	-0.144755
Ciclev10011783m.g	scaffold_6:25343400-25347347	11.2361	10.1637	-0.144718
Ciclev10001762m.g	scaffold_5:21071128-21074649	16.4592	14.8894	-0.14461
Ciclev10002549m.g	scaffold_5:42838210-42840133	3.83351	3.46793	-0.144591
Ciclev10031138m.g	scaffold_4:22528413-22532439	69.9898	63.3454	-0.143906
Ciclev10002950m.g	scaffold_5:38639090-38641163	68.9015	62.3837	-0.143366
Ciclev10010044m.g	scaffold_1:6227616-6228389	339.898	307.755	-0.143317
Ciclev10030996m.g	scaffold_4:23705189-23711365	16.1032	14.5809	-0.143266
Ciclev10013338m.g	scaffold_6:23332476-23336262	0.999979	0.905552	-0.1431
Ciclev10007617m.g	scaffold_1:17514389-17522560	34.819	31.5349	-0.142924
Ciclev10004793m.g	scaffold_9:469600-474975	148.054	134.093	-0.14289
Ciclev10015098m.g	scaffold_2:35952188-35956231	30.0017	27.1728	-0.142879
Ciclev10016300m.g	scaffold_2:29598613-29603228	145.525	131.812	-0.142776
Ciclev10031677m.g	scaffold_4:3619706-3623502	27.2166	24.6538	-0.142672
Ciclev10024326m.g	scaffold_3:49198700-49202168	44.0882	39.9392	-0.142587
Ciclev10018555m.g	scaffold_3:8563135-8573549	6.67265	6.04478	-0.14257
Ciclev10005574m.g	scaffold_9:95221-96541	105.371	95.4599	-0.142513
Ciclev10015309m.g	scaffold_2:33997618-33999134	18.6829	16.9256	-0.142505
Ciclev10011669m.g	scaffold_6:24128224-24132186	73.9239	66.9729	-0.142463
Ciclev10022591m.g	scaffold_3:4360528-4364138	35.2836	31.966	-0.14246
Ciclev10009101m.g	scaffold_1:26975378-26978796	22.0541	19.982	-0.142348
Ciclev10018593m.g	scaffold_3:45160202-45169388	18.8636	17.0917	-0.142304
Ciclev10021587m.g,Ciclev10022203m.g	scaffold_3:10467511-10483970	6.52235	5.90989	-0.142259
Ciclev10026914m.g	scaffold_7:9089279-9091276	288.431	261.351	-0.142235
Ciclev10028611m.g	scaffold_8:19744964-19746459	2.22044	2.012	-0.142212
Ciclev10009114m.g	scaffold_1:4522021-4524821	115.613	104.762	-0.142185
Ciclev10012980m.g	scaffold_6:19192423-19195689	71.508	64.8058	-0.141983
Ciclev10023852m.g	scaffold_3:41995020-42002754	33.7608	30.5989	-0.141872
Ciclev10009735m.g	scaffold_1:5863717-5866856	2.95255	2.67629	-0.14173
Ciclev10024907m.g	scaffold_7:2857977-2861876	5.73804	5.20134	-0.141675
Ciclev10009897m.g	scaffold_1:24574963-24582425	18.9444	17.1744	-0.141514
Ciclev10028072m.g	scaffold_8:4083629-4089056	18.7084	16.9619	-0.141383
Ciclev10025895m.g	scaffold_7:20916738-20921801	26.1145	23.6772	-0.141353
Ciclev10007908m.g	scaffold_1:25639465-25643626	67.9395	61.6176	-0.14091
Ciclev10019644m.g	scaffold_3:46690872-46698753	8.59271	7.79352	-0.140838
Ciclev10015463m.g	scaffold_2:27497100-27501650	9.77542	8.86625	-0.140836
Ciclev10021714m.g	scaffold_3:30728988-30732384	111.348	101.028	-0.14032
Ciclev10022699m.g	scaffold_3:35935667-35938071	18.4992	16.7848	-0.140307
Ciclev10018609m.g	scaffold_3:7136635-7145051	31.9171	28.9599	-0.140274
Ciclev10025927m.g	scaffold_7:1594957-1596986	3.22084	2.92259	-0.140193
Ciclev10027374m.g	scaffold_7:11128142-11129372	3.86587	3.50858	-0.139905
Ciclev10019617m.g	scaffold_3:49043874-49052386	6.23383	5.65825	-0.139763
Ciclev10014220m.g	scaffold_2:22371490-22383372	5.46977	4.96499	-0.139688
Ciclev10007954m.g	scaffold_1:26375884-26383450	9.88471	8.97279	-0.139642
Ciclev10032658m.g	scaffold_4:19561294-19567152	90.5739	82.2204	-0.139598
Ciclev10018568m.g	scaffold_3:34622366-34641954	14.1618	12.857	-0.139442
Ciclev10005712m.g	scaffold_9:30926725-30931198	43.496	39.4905	-0.139376
Ciclev10016727m.g	scaffold_2:23184583-23186799	30.61	27.7922	-0.139323
Ciclev10005726m.g	scaffold_9:8466199-8470081	16.5054	14.9864	-0.139284
Ciclev10004623m.g	scaffold_9:12201414-12210457	14.4429	13.117	-0.13892
Ciclev10028647m.g	scaffold_8:725296-727320	2.7719	2.51761	-0.138819
Ciclev10015015m.g	scaffold_2:21400947-21406324	21.5093	19.5369	-0.138755
Ciclev10021268m.g	scaffold_3:49149328-49151661	9.06901	8.23759	-0.138721
Ciclev10031360m.g	scaffold_4:10878164-10883204	98.9955	89.9228	-0.138675
Ciclev10011394m.g	scaffold_6:15741902-15746306	7.36231	6.68763	-0.138662

Ciclev10024364m.g	scaffold_3:10421825-10423612	3.6082	3.27778	-0.138563
Ciclev10008646m.g	scaffold_1:18303957-18309021	2234.7	2030.23	-0.138434
Ciclev10012457m.g	scaffold_6:25221810-25224666	25.4902	23.1617	-0.138206
Ciclev10005974m.g	scaffold_9:4119935-4121030	555.848	505.096	-0.138133
Ciclev10004618m.g	scaffold_9:2218439-2224463	16.6996	15.1761	-0.138009
Ciclev10004438m.g	scaffold_9:19386441-19399612	69.0604	62.769	-0.137807
Ciclev10013372m.g	scaffold_6:19484840-19487134	20.7359	18.8518	-0.137429
Ciclev10020583m.g	scaffold_3:33174236-33188683	5.74279	5.22114	-0.137388
Ciclev10026688m.g	scaffold_7:185146-185751	28.6907	26.0854	-0.137342
Ciclev10012571m.g	scaffold_6:15283058-15290088	7.61803	6.92661	-0.137268
Ciclev10010826m.g	scaffold_1:28510743-28513570	28.3778	25.8147	-0.13657
Ciclev10028912m.g	scaffold_8:22487086-22489505	17.4236	15.8515	-0.136419
Ciclev10020349m.g	scaffold_3:47310123-47325365	58.8153	53.5121	-0.136326
Ciclev10031765m.g,Ciclev10033656m.g	scaffold_4:780579-784430	1228.07	1117.47	-0.136162
Ciclev10024413m.g	scaffold_3:43350018-43353840	25.8412	23.5149	-0.136096
Ciclev10008156m.g	scaffold_1:6644076-6646249	5.81543	5.29223	-0.136012
Ciclev10021561m.g	scaffold_3:2755858-2757382	1.67288	1.5227	-0.135705
Ciclev10009849m.g	scaffold_1:11340405-11344077	7.97414	7.25934	-0.135491
Ciclev10005225m.g	scaffold_9:1945580-1947434	22.7537	20.7195	-0.13511
Ciclev10000767m.g	scaffold_5:42217934-42222257	31.3254	28.5256	-0.135077
Ciclev10017544m.g	scaffold_2:14609235-14613660	11.1233	10.1307	-0.134846
Ciclev10022036m.g	scaffold_3:49589350-49591133	20.559	18.7259	-0.134739
Ciclev10020692m.g	scaffold_3:29292110-29295993	1870.04	1703.44	-0.134619
Ciclev10004728m.g	scaffold_9:27208888-27211614	28.1364	25.638	-0.134156
-	scaffold_4:7123330-7123837	10.2704	9.35917	-0.134038
Ciclev10014550m.g	scaffold_2:12562231-12570957	11.8091	10.7617	-0.133989
Ciclev10018524m.g	scaffold_3:37944756-37949994	2.07708	1.89307	-0.133828
Ciclev10013081m.g	scaffold_6:24491130-24492254	121.931	111.132	-0.133799
Ciclev10001315m.g	scaffold_5:11394533-11398659	78.8918	71.9213	-0.133457
Ciclev10007599m.g	scaffold_1:26070071-26074858	11.4765	10.4671	-0.132818
Ciclev10027862m.g	scaffold_8:19483500-19491585	30.9279	28.2114	-0.132632
Ciclev10000039m.g	scaffold_5:42718714-42734359	37.3192	34.0547	-0.132062
Ciclev10000199m.g	scaffold_5:2303130-2309639	2.84341	2.59491	-0.131938
Ciclev10025929m.g	scaffold_7:11357989-11362560	229.552	209.502	-0.13186
Ciclev10031395m.g	scaffold_4:17952453-17958235	123.604	112.82	-0.131695
Ciclev10019131m.g	scaffold_3:767879-772430	23.8935	21.8091	-0.131686
Ciclev10027687m.g	scaffold_8:24170232-24183146	8.89908	8.12288	-0.131664
Ciclev10021497m.g	scaffold_3:7355187-7357179	4.7366	4.32357	-0.131629
Ciclev10032544m.g	scaffold_4:1381349-1383312	134.727	122.986	-0.131542
Ciclev10015315m.g	scaffold_2:26418292-26420912	11.6556	10.6411	-0.131372
Ciclev10029152m.g	scaffold_8:23464640-23467070	3.1256	2.85387	-0.131212
Ciclev10027698m.g	scaffold_8:21157251-21166003	7.61652	6.95438	-0.131209
Ciclev10029212m.g	scaffold_8:21336262-21340075	31.4555	28.7213	-0.131196
Ciclev10008278m.g	scaffold_1:15478049-15482243	15.1725	13.855	-0.131057
Ciclev10028573m.g	scaffold_8:22505946-22510122	26.631	24.3192	-0.131014
Ciclev10012050m.g	scaffold_6:15057288-15061094	32.0204	29.2475	-0.130679
Ciclev10021635m.g	scaffold_3:38541419-38547555	8.29642	7.57838	-0.130599
Ciclev10014745m.g	scaffold_2:15839407-15847167	23.0603	21.0647	-0.130583
Ciclev10008642m.g	scaffold_1:20995351-21034862	389.629	355.941	-0.130465
Ciclev10007931m.g	scaffold_1:25840518-25845895	45.3519	41.432	-0.130416
Ciclev10008698m.g	scaffold_1:8623591-8627692	19.3827	17.7096	-0.130244
Ciclev10008199m.g,Ciclev10010827m.g	scaffold_1:1970231-1976950	9.71295	8.87798	-0.129678
Ciclev10014488m.g	scaffold_2:3601296-3610993	37.9255	34.6665	-0.129625
Ciclev10003012m.g	scaffold_5:42288916-42290276	26.5851	24.3008	-0.129616

Ciclev10025812m.g	scaffold_7:14647414-14654942	55.4344	50.6814	-0.129325
Ciclev10026612m.g	scaffold_7:11302181-11306553	98.2294	89.8295	-0.128966
Ciclev10016214m.g	scaffold_2:36104523-36107958	35.1158	32.1151	-0.128866
Ciclev10021854m.g	scaffold_3:39738979-39744295	15.8217	14.47	-0.128831
Ciclev10015393m.g	scaffold_2:8271911-8273575	8.31451	7.60437	-0.128802
Ciclev10032498m.g	scaffold_4:25371387-25373340	216.081	197.632	-0.128757
Ciclev10015407m.g	scaffold_2:32030343-32035492	83.9946	76.831	-0.128608
Ciclev10014125m.g,Ciclev10014126m.g,Ciclev10015563m.g,Ciclev10016248m.g	scaffold_2:34559760-34586231	77.0894	70.5165	-0.128572
Ciclev10016383m.g	scaffold_2:8510401-8517461	33.1433	30.3191	-0.128489
Ciclev10026373m.g	scaffold_7:18374450-18378453	114.884	105.099	-0.128433
Ciclev10015055m.g	scaffold_2:32132995-32137975	16.6288	15.2161	-0.12809
Ciclev10026840m.g	scaffold_7:5111277-5112832	14.8206	13.5619	-0.128042
Ciclev10003023m.g	scaffold_5:34657438-34659553	28.8918	26.4399	-0.127941
Ciclev10020702m.g	scaffold_3:49452996-49455979	27.0707	24.7745	-0.127875
Ciclev10021823m.g	scaffold_3:11657370-11658678	12.0567	11.0353	-0.127709
Ciclev10004880m.g	scaffold_9:166431-170727	48.6856	44.5648	-0.127591
Ciclev10015545m.g	scaffold_2:13110834-13117657	17.5674	16.0823	-0.127429
Ciclev10011466m.g	scaffold_6:23228599-23232310	13.3288	12.204	-0.12719
Ciclev10025397m.g	scaffold_7:6660907-6663562	29.6557	27.1552	-0.127083
Ciclev10030593m.g	scaffold_4:24193307-24197268	0.934927	0.856123	-0.127036
Ciclev10009634m.g	scaffold_1:24400221-24402626	23.9096	21.8958	-0.126932
Ciclev10008154m.g	scaffold_1:27529848-27535221	47.118	43.1502	-0.126911
Ciclev10017502m.g	scaffold_2:9347793-9351278	210.081	192.395	-0.126881
Ciclev10003419m.g	scaffold_5:32931029-32934227	7.16664	6.56331	-0.126874
Ciclev10027731m.g	scaffold_8:22595020-22604190	38.0479	34.8477	-0.126754
Ciclev10015520m.g	scaffold_2:28800353-28804551	14.1587	12.9681	-0.126725
Ciclev10031052m.g	scaffold_4:21754311-21759602	14.9569	13.6992	-0.126715
Ciclev10028955m.g	scaffold_8:786306-788424	215.043	196.961	-0.12671
Ciclev10013714m.g	scaffold_6:22678094-22682872	3.95306	3.62076	-0.126679
Ciclev10012371m.g	scaffold_6:17796448-17799874	58.3875	53.4804	-0.126649
Ciclev10012182m.g	scaffold_6:21127983-21132025	50.0996	45.8916	-0.126569
Ciclev10011278m.g	scaffold_6:19545255-19549611	3.30735	3.0302	-0.126259
Ciclev10015519m.g	scaffold_2:28761648-28766750	6.883	6.30631	-0.12624
Ciclev10001978m.g	scaffold_5:40832602-40834625	25.4773	23.3445	-0.126132
Ciclev10007363m.g	scaffold_1:5280768-5289488	6.77612	6.20993	-0.125882
Ciclev10017178m.g	scaffold_2:23905996-23909204	95.2263	87.2796	-0.125715
Ciclev10021806m.g	scaffold_3:532881-536813	8.94204	8.19656	-0.125584
Ciclev10007533m.g	scaffold_1:28716724-28730151	38.0442	34.875	-0.125484
Ciclev10032351m.g	scaffold_4:24946512-24948944	308.665	283.007	-0.125202
Ciclev10004703m.g	scaffold_9:1810004-1815669	23.3947	21.4503	-0.125178
-	scaffold_9:11403370-11403633	29.525	27.0715	-0.125163
Ciclev10012735m.g	scaffold_6:14482284-14485541	35.6369	32.6769	-0.125098
Ciclev10026255m.g	scaffold_7:6405177-6427187	21.9529	20.13	-0.125058
Ciclev10032618m.g	scaffold_4:6335137-6336149	12.6651	11.6136	-0.125049
Ciclev10007426m.g,Ciclev10007475m.g	scaffold_1:19443215-19466195	16.2167	14.8706	-0.125023
Ciclev10018165m.g	scaffold_2:30621080-30623685	20.2606	18.5789	-0.125011
Ciclev10019659m.g	scaffold_3:8509861-8514062	8.87356	8.13731	-0.12496
Ciclev10019238m.g	scaffold_3:49174005-49178355	8.42905	7.72972	-0.124954
Ciclev10015782m.g	scaffold_2:28138080-28141376	27.7591	25.459	-0.124787
Ciclev10001759m.g	scaffold_5:21459893-21463088	18.8962	17.3322	-0.124639
Ciclev10008176m.g	scaffold_1:22792919-22795069	90.2109	82.7513	-0.12452
Ciclev10018426m.g	scaffold_3:44085089-44104660	5.25867	4.82428	-0.124385
Ciclev10018013m.g	scaffold_2:24565856-24567752	134.068	122.995	-0.124364

Ciclev10029673m.g	scaffold_8:23133454-23135190	9.682	8.88253	-0.124335
Ciclev10014807m.g	scaffold_2:1824668-1830700	17.9423	16.4608	-0.124326
Ciclev10001285m.g	scaffold_5:38930336-38936779	14.5655	13.3638	-0.124232
Ciclev10004212m.g	scaffold_9:25349210-25365513	20.7581	19.0519	-0.123736
-	scaffold_5:8745667-8989247	14.5609	13.3674	-0.123381
Ciclev10026139m.g	scaffold_7:901454-902956	2.9735	2.7298	-0.123366
Ciclev10020092m.g,Ciclev10023826m.g	scaffold_3:27395595-27413326	63.2537	58.0817	-0.123068
Ciclev10017430m.g	scaffold_2:11799005-11831046	275.884	253.336	-0.12301
Ciclev10031535m.g	scaffold_4:3803027-3807177	37.4859	34.4262	-0.122841
Ciclev10007279m.g	scaffold_1:26211566-26226910	25.7476	23.6464	-0.12282
Ciclev10026448m.g	scaffold_7:5603545-5608700	131.627	120.895	-0.122701
Ciclev10032722m.g	scaffold_4:14583491-14585675	180.78	166.044	-0.122661
Ciclev10005518m.g	scaffold_9:905622-911210	148.139	136.07	-0.12261
Ciclev10007703m.g	scaffold_1:7069272-7072709	11.4957	10.5594	-0.122565
Ciclev10004652m.g	scaffold_9:7205060-7210887	65.5439	60.221	-0.122194
Ciclev10029423m.g	scaffold_8:21494162-21497281	52.7499	48.4668	-0.122171
Ciclev10001657m.g	scaffold_5:37622886-37624944	34.0332	31.2791	-0.121745
Ciclev10014653m.g	scaffold_2:35620831-35628855	19.0936	17.5486	-0.121738
Ciclev10000708m.g	scaffold_5:42601357-42605843	8.34275	7.66796	-0.121679
Ciclev10011197m.g	scaffold_6:20799705-20807639	8.20606	7.54241	-0.121664
Ciclev10033729m.g	scaffold_4:23609401-23612148	61.0466	56.1124	-0.121592
Ciclev10011997m.g	scaffold_6:10485063-10488724	26.1796	24.074	-0.120965
Ciclev10013301m.g	scaffold_6:14072453-14077679	6.69613	6.15839	-0.120774
Ciclev10007666m.g	scaffold_1:6169468-6178216	13.773	12.6671	-0.120763
Ciclev10001606m.g	scaffold_5:42021422-42023134	28.4401	26.1564	-0.120758
Ciclev10018086m.g	scaffold_2:9374794-9377121	0.948761	0.872598	-0.120728
Ciclev10006298m.g	scaffold_9:22168154-22168867	272.385	250.548	-0.12056
Ciclev10029592m.g	scaffold_8:22981142-22982950	87.5947	80.5724	-0.120558
Ciclev10004131m.g	scaffold_9:943199-955128	10.7995	9.93424	-0.120481
Ciclev10002676m.g	scaffold_5:33426152-33427255	13.7921	12.6918	-0.119942
Ciclev10030014m.g	scaffold_8:7380472-7380769	4.94813	4.55374	-0.119832
Ciclev10016547m.g	scaffold_2:14644603-14649552	194.294	178.82	-0.119736
Ciclev10029598m.g	scaffold_8:17797404-17798119	31.567	29.0542	-0.119669
Ciclev10016373m.g	scaffold_2:14002606-14005144	28.6714	26.3917	-0.119525
Ciclev10014075m.g	scaffold_2:34489415-34497724	2.99019	2.75254	-0.119473
Ciclev10016080m.g	scaffold_2:33978527-33982559	16.1663	14.8821	-0.119414
Ciclev10027306m.g	scaffold_7:16108105-16113463	27.6617	25.4685	-0.119174
Ciclev10031185m.g	scaffold_4:14421313-14424594	145.345	133.834	-0.119045
Ciclev10001736m.g	scaffold_5:42278457-42281250	14.1572	13.0369	-0.118941
Ciclev10001558m.g	scaffold_5:38710106-38715190	6.58987	6.06901	-0.118788
Ciclev10021430m.g	scaffold_3:8186688-8189902	25.8827	23.8389	-0.118669
Ciclev10026577m.g	scaffold_7:459938-462008	388.822	358.121	-0.118662
Ciclev10031784m.g	scaffold_4:17077443-17080325	2.08641	1.92186	-0.118521
Ciclev10029576m.g	scaffold_8:17510925-17513265	58.242	53.6553	-0.11834
Ciclev10014010m.g	scaffold_2:29977828-29994253	11.2964	10.4071	-0.118287
Ciclev10030351m.g	scaffold_8:67586-74380	16.7124	15.3971	-0.118267
Ciclev10001079m.g	scaffold_5:21076262-21082155	28.9313	26.6543	-0.118261
Ciclev10000559m.g	scaffold_5:38753374-38762034	20.3013	18.7063	-0.118051
Ciclev10012237m.g	scaffold_6:15817496-15823667	13.7486	12.6692	-0.117964
Ciclev10011563m.g	scaffold_6:15439329-15443184	26.0003	23.961	-0.117837
Ciclev10007970m.g	scaffold_1:22897217-22901872	11.7455	10.8248	-0.117761
Ciclev10008334m.g	scaffold_1:28385659-28388620	85.1118	78.4596	-0.117409
Ciclev10031276m.g	scaffold_4:24141682-24145765	9.21037	8.49182	-0.117185
Ciclev10005099m.g	scaffold_9:28446117-28453825	4.61723	4.25733	-0.117078
Ciclev10018794m.g	scaffold_3:48903211-48907196	5.23073	4.82347	-0.116939

Ciclev10032036m.g	scaffold_4:22620823-22623543	20.2437	18.6682	-0.116889
Ciclev10016788m.g	scaffold_2:29172771-29175757	20.4098	18.8221	-0.116838
Ciclev10018796m.g	scaffold_3:49653522-49660718	11.197	10.326	-0.116825
Ciclev10023915m.g	scaffold_3:43804383-43807840	30.4061	28.0426	-0.116739
Ciclev10021158m.g	scaffold_3:8338967-8344388	18.3263	16.9022	-0.1167
Ciclev10014955m.g	scaffold_2:14148391-14153982	215.854	199.091	-0.116628
Ciclev10014880m.g	scaffold_2:36223845-36231874	2.87879	2.65558	-0.116436
Ciclev10001223m.g	scaffold_5:40702394-40706121	18.9049	17.4417	-0.116216
Ciclev10013682m.g	scaffold_6:20624885-20628239	140.55	129.681	-0.116115
Ciclev10011865m.g	scaffold_6:18194883-18202571	7.11449	6.5648	-0.116009
Ciclev10022754m.g	scaffold_3:45882707-45885851	27.4075	25.2916	-0.11591
Ciclev10012178m.g	scaffold_6:14868820-14872953	40.3252	37.2204	-0.115591
Ciclev10012893m.g	scaffold_6:23175696-23177025	16702	15416.2	-0.115572
Ciclev10008947m.g	scaffold_1:27779707-27782574	67.2648	62.0881	-0.115536
Ciclev10016597m.g	scaffold_2:28096902-28101024	20.6495	19.063	-0.115327
Ciclev10014332m.g	scaffold_2:12095437-12100826	26.8423	24.7806	-0.115299
Ciclev10004139m.g	scaffold_9:3649696-3659241	11.6513	10.7579	-0.115096
Ciclev10002994m.g	scaffold_5:36764591-36765043	3.3587	3.10145	-0.114962
Ciclev10021604m.g	scaffold_3:47310123-47325365	40.3616	37.2714	-0.114914
Ciclev10022435m.g	scaffold_3:45916862-45919225	169.046	156.104	-0.11491
Ciclev10021681m.g	scaffold_3:47215628-47221472	100.064	92.4034	-0.114904
Ciclev10029649m.g,Ciclev10029972m.g,Ciclev10030138m.g	scaffold_8:17291958-17467945	33.4555	30.8972	-0.114769
Ciclev10020949m.g,Ciclev10021105m.g	scaffold_3:38229220-38249259	15.8573	14.6453	-0.11471
Ciclev10004605m.g	scaffold_9:27224778-27232647	23.8716	22.0483	-0.114631
Ciclev10031372m.g	scaffold_4:20253307-20257747	7.40284	6.83756	-0.114597
Ciclev10010937m.g	scaffold_6:22946197-22954557	17.6449	16.3003	-0.114357
Ciclev10003249m.g	scaffold_5:42669246-42674395	44.6087	41.213	-0.114225
Ciclev10007858m.g	scaffold_1:25949072-25953711	6.29574	5.81676	-0.114161
Ciclev10028282m.g	scaffold_8:24192884-24196652	53.7994	49.716	-0.113879
Ciclev10029452m.g	scaffold_8:822411-824342	31.7783	29.3689	-0.113756
Ciclev10007972m.g,Ciclev10008240m.g	scaffold_1:20995351-21034862	652.639	603.157	-0.113751
Ciclev10029397m.g	scaffold_8:23697050-23698248	12.9547	11.9738	-0.113586
Ciclev10002134m.g	scaffold_5:13886545-13915539	130.064	120.228	-0.113444
Ciclev10016261m.g	scaffold_2:10994553-10996721	7.18839	6.64483	-0.113436
Ciclev10000400m.g	scaffold_5:42918370-42923480	21.0521	19.4612	-0.113369
Ciclev10017045m.g	scaffold_2:35075675-35078521	22.5585	20.8546	-0.113305
Ciclev10004589m.g	scaffold_9:22593942-22600743	4.70242	4.34726	-0.113297
Ciclev10000863m.g	scaffold_5:13198140-13204979	5.95541	5.50598	-0.1132
Ciclev10011701m.g	scaffold_6:25267372-25269976	14.5359	13.4418	-0.112899
Ciclev10003457m.g,Ciclev10003529m.g	scaffold_5:39046740-39085639	14.8239	13.7082	-0.112893
Ciclev10031545m.g	scaffold_4:21923247-21930291	23.6475	21.8682	-0.112852
Ciclev10021260m.g	scaffold_3:13568513-13571796	89.1622	82.4547	-0.11283
Ciclev10010835m.g	scaffold_1:22912694-22913798	354.323	327.669	-0.112823
Ciclev10007524m.g	scaffold_1:25987802-25992258	14.3523	13.2732	-0.112774
Ciclev10014665m.g	scaffold_2:22459165-22461667	4.69864	4.34539	-0.112758
Ciclev10023130m.g	scaffold_3:26577436-26586352	65.6885	60.7503	-0.112749
-	scaffold_4:1394024-1394446	806.954	746.325	-0.112684
Ciclev10030585m.g	scaffold_4:17696275-17700620	1.2384	1.14538	-0.112658
Ciclev10001635m.g	scaffold_5:41932894-41936414	7.7283	7.14802	-0.112608
Ciclev10020358m.g	scaffold_3:37984841-37990529	13.4822	12.4714	-0.112436
Ciclev10012342m.g	scaffold_6:16485915-16487373	2.03667	1.88399	-0.112419

Ciclev10022077m.g	scaffold_3:18239858-18242696	2.34006	2.16469	-0.112383
Ciclev10026787m.g	scaffold_7:15735801-15738126	277.65	256.851	-0.112335
Ciclev10009257m.g	scaffold_1:27922255-27925639	32.052	29.6558	-0.112099
-	scaffold_3:25247209-25247525	65.9964	61.1026	-0.111154
Ciclev10012408m.g	scaffold_6:22060987-22063187	12.0659	11.1713	-0.111138
Ciclev10005073m.g	scaffold_9:30000412-30004107	25.0032	23.1511	-0.111032
Ciclev10001144m.g	scaffold_5:39362177-39365948	41.2393	38.197	-0.110562
Ciclev10016542m.g	scaffold_2:11443859-11446838	23.3238	21.6038	-0.110514
Ciclev10019417m.g	scaffold_3:9718428-9725668	40.0604	37.1087	-0.110419
Ciclev10017122m.g	scaffold_2:12050431-12085984	12.8124	11.8709	-0.110106
Ciclev10023554m.g	scaffold_3:43373433-43377433	30.4208	28.188	-0.109977
Ciclev10016481m.g	scaffold_2:11288575-11294965	35.146	32.571	-0.109773
Ciclev10009592m.g	scaffold_1:24408659-24410261	43.994	40.7763	-0.109576
Ciclev10016926m.g	scaffold_2:9305650-9308520	60.9146	56.4604	-0.109547
Ciclev10033722m.g	scaffold_4:1668709-1676282	32.9718	30.5612	-0.109534
Ciclev10001709m.g	scaffold_5:43275223-43279116	20.8064	19.2867	-0.10942
Ciclev10004815m.g	scaffold_9:664986-669744	15.8493	14.6922	-0.109366
Ciclev10005641m.g	scaffold_9:30913799-30915113	153.803	142.575	-0.109363
Ciclev10032550m.g	scaffold_4:15053223-15056267	56.0014	51.9187	-0.109208
Ciclev10012548m.g	scaffold_6:11100646-11103340	135.892	126.002	-0.109014
Ciclev10007764m.g	scaffold_1:995899-1000158	2.48178	2.30137	-0.108879
Ciclev10025937m.g	scaffold_7:6527504-6530918	59.9719	55.6242	-0.108575
Ciclev10013100m.g	scaffold_6:18217725-18223869	29.7696	27.6115	-0.10857
Ciclev10011508m.g	scaffold_6:14524325-14526459	0.648395	0.601396	-0.108558
Ciclev10008331m.g	scaffold_1:312354-326006	14.2185	13.191	-0.108221
Ciclev10009224m.g	scaffold_1:28346766-28349974	34.4177	31.9315	-0.108171
Ciclev10020120m.g	scaffold_3:5461896-5466529	6.73356	6.24738	-0.108117
Ciclev10004430m.g	scaffold_9:4107400-4114181	12.6438	11.7315	-0.108041
Ciclev10007802m.g	scaffold_1:24115727-24121751	14.0144	13.0032	-0.108039
Ciclev10014047m.g	scaffold_2:30241890-30256637	10.7457	9.9708	-0.107976
Ciclev10007728m.g	scaffold_1:2851334-2854493	16.3225	15.1459	-0.10794
Ciclev10028940m.g	scaffold_8:24358435-24361345	5.66907	5.26211	-0.107471
Ciclev10005364m.g	scaffold_9:494940-503720	281.051	260.889	-0.107399
Ciclev10000970m.g,Ciclev10004068m.g	scaffold_5:41703786-41708871	32.9057	30.546	-0.107352
Ciclev10022350m.g	scaffold_3:45221637-45224185	33.1955	30.816	-0.107309
Ciclev10030286m.g	scaffold_8:24896209-24905432	7.82126	7.26216	-0.107002
Ciclev10027297m.g	scaffold_7:2398201-2400528	3.79305	3.52196	-0.106983
Ciclev10023925m.g	scaffold_3:50700488-50702648	12.5257	11.6306	-0.106964
Ciclev10026334m.g	scaffold_7:14278625-14281637	30.5718	28.3892	-0.106859
Ciclev10012850m.g	scaffold_6:18071783-18075799	21.1531	19.651	-0.106269
Ciclev10020055m.g	scaffold_3:26480613-26486649	11.4999	10.6834	-0.106254
Ciclev10004156m.g	scaffold_9:1711624-1717960	4.09249	3.80203	-0.106209
Ciclev10021217m.g	scaffold_3:43607212-43610327	15.3633	14.273	-0.1062
Ciclev10022543m.g	scaffold_3:41867168-41869851	8.23896	7.65427	-0.106197
Ciclev10015418m.g	scaffold_2:11084959-11095416	154.001	143.081	-0.106107
Ciclev10020999m.g,Ciclev10021004m.g	scaffold_3:39181844-39300715	5.60868	5.21114	-0.106061
-	scaffold_2:27360283-27360528	27.5837	25.6289	-0.10604
Ciclev10032390m.g	scaffold_4:25646916-25649041	8.77567	8.1543	-0.105948
Ciclev10028761m.g	scaffold_8:2130234-2138606	25.5052	23.7028	-0.105734
Ciclev10001331m.g	scaffold_5:20091692-20093357	14.4411	13.4241	-0.105351
Ciclev10025256m.g	scaffold_7:463993-470316	15.9833	14.8582	-0.105302
Ciclev10025097m.g	scaffold_7:1435628-1441314	11.5348	10.724	-0.105141
Ciclev10026596m.g	scaffold_7:2622968-2624754	43.1309	40.1035	-0.104996
Ciclev10020998m.g	scaffold_3:11479701-11483057	14.3788	13.3702	-0.104918

Ciclev10010953m.g	scaffold_6:352192-356318	18.9551	17.6257	-0.104904
Ciclev10021788m.g	scaffold_3:46263053-46265579	3.82531	3.55741	-0.104749
Ciclev10027770m.g	scaffold_8:116521-122122	45.9696	42.7522	-0.104682
Ciclev10005823m.g	scaffold_9:13532068-13535642	31.6874	29.4703	-0.104647
Ciclev10011142m.g	scaffold_6:22288440-22294247	6.37782	5.93198	-0.104551
-	scaffold_7:18617580-18635802	4.2177	3.92346	-0.104331
Ciclev10021008m.g	scaffold_3:39013555-39018244	38.4896	35.8052	-0.104299
Ciclev10007933m.g	scaffold_1:26026277-26032690	43.4906	40.4607	-0.104184
Ciclev10030626m.g	scaffold_4:19776761-19782840	328.3	305.45	-0.104078
Ciclev10023130m.g	scaffold_3:26577436-26586352	12.3992	11.5367	-0.104016
Ciclev10020967m.g	scaffold_3:7197711-7199779	15.9694	14.8589	-0.10398
Ciclev10015926m.g	scaffold_2:25755631-25757676	4.69491	4.36874	-0.103881
Ciclev10012656m.g	scaffold_6:12350242-12352049	16.8052	15.6392	-0.103746
Ciclev10008346m.g	scaffold_1:25919816-25923185	7.18746	6.68962	-0.103557
Ciclev10000054m.g	scaffold_5:42101237-42106547	11.149	10.3777	-0.10343
Ciclev10010556m.g	scaffold_1:924502-936397	3.50243	3.26022	-0.103387
Ciclev10032050m.g	scaffold_4:7301637-7308849	44.6467	41.5632	-0.103247
Ciclev10017504m.g	scaffold_2:28199752-28201931	6.09101	5.67136	-0.102987
Ciclev10016636m.g	scaffold_2:31534414-31538268	38.5403	35.8851	-0.102983
Ciclev10004829m.g	scaffold_9:22744497-22749685	3.84297	3.5785	-0.102868
Ciclev10004429m.g	scaffold_9:1654001-1660221	30.589	28.4842	-0.10285
Ciclev10021028m.g	scaffold_3:44565761-44570397	1.79841	1.67487	-0.102678
Ciclev10020429m.g	scaffold_3:49136199-49139802	11.4445	10.6605	-0.102378
Ciclev10016110m.g	scaffold_2:30865951-30871079	33.1284	30.8611	-0.102277
Ciclev10018447m.g	scaffold_3:47447468-47458060	11.4103	10.6305	-0.102134
Ciclev10008805m.g	scaffold_1:5276922-5280083	9.21049	8.58119	-0.102101
Ciclev10019914m.g	scaffold_3:39667818-39674194	16.889	15.7354	-0.102066
Ciclev10002064m.g	scaffold_5:9456036-9459483	17.8637	16.6453	-0.101917
Ciclev10005852m.g	scaffold_9:10376647-10380187	88.7996	82.747	-0.101847
Ciclev10022322m.g	scaffold_3:42889381-42891279	100.763	93.9069	-0.101657
Ciclev10020016m.g	scaffold_3:3241861-3247042	139.081	129.629	-0.101534
Ciclev10002868m.g	scaffold_5:37309558-37310597	17.4957	16.3082	-0.101404
Ciclev10009487m.g	scaffold_1:4538413-4541421	97.7666	91.1324	-0.101377
Ciclev10029274m.g	scaffold_8:2171927-2173777	0.87024	0.811212	-0.101334
Ciclev10000454m.g	scaffold_5:29808452-29811460	1.13502	1.05804	-0.10133
Ciclev10016668m.g	scaffold_2:23180084-23182810	58.434	54.4742	-0.101236
Ciclev10000672m.g	scaffold_5:5328722-5334353	31.6338	29.491	-0.101192
Ciclev10020911m.g	scaffold_3:37863406-37865715	51.1223	47.6643	-0.101044
Ciclev10004784m.g	scaffold_9:17513741-17515751	8.81549	8.21945	-0.101
Ciclev10001575m.g	scaffold_5:39105671-39108800	19.1652	17.8699	-0.100962
Ciclev10012376m.g	scaffold_6:17845656-17849940	26.9412	25.1266	-0.100603
Ciclev10020642m.g	scaffold_3:49195568-49198341	24.6201	22.9623	-0.100566
Ciclev10016804m.g	scaffold_2:28790924-28793650	50.7754	47.3573	-0.100545
Ciclev10011641m.g	scaffold_6:17965773-17969108	29.1394	27.1811	-0.100372
Ciclev10020896m.g	scaffold_3:41354693-41358021	9.94643	9.27817	-0.100338
Ciclev10031922m.g	scaffold_4:18324119-18329144	51.7702	48.2924	-0.100325
Ciclev10032791m.g	scaffold_4:14675665-14679314	16.6416	15.5253	-0.100178
Ciclev10025009m.g	scaffold_7:20060957-20065609	98.8409	92.2134	-0.100131
Ciclev10003645m.g,Ciclev10003790m.g	scaffold_5:329201-335201	62.9343	58.7181	-0.100041
Ciclev10027871m.g	scaffold_8:21262028-21271196	6.96287	6.49694	-0.0999215
Ciclev10009466m.g	scaffold_1:23557230-23561471	53.2954	49.7306	-0.0998763
Ciclev10000495m.g	scaffold_5:38081414-38083994	0.981523	0.915921	-0.0997978
Ciclev10007934m.g	scaffold_1:25175208-25179944	4.84618	4.52265	-0.0996802
Ciclev10013183m.g	scaffold_6:13243345-13243983	97.7967	91.2747	-0.0995714
Ciclev10016057m.g	scaffold_2:33561486-33563802	268.284	250.423	-0.099392

Ciclev10022757m.g	scaffold_3:32904022-32906262	293.458	274.075	-0.0985841
Ciclev10012127m.g	scaffold_6:9789369-9794598	16.94	15.8212	-0.09857
Ciclev10011216m.g	scaffold_6:2635537-2642250	4.84096	4.52154	-0.0984801
Ciclev10002418m.g	scaffold_5:33846261-33853353	115.187	107.59	-0.0984418
Ciclev10024708m.g	scaffold_7:1824291-1831357	4.89013	4.56816	-0.0982606
Ciclev10032389m.g	scaffold_4:593144-595986	72.6667	67.8856	-0.0981878
Ciclev10001211m.g	scaffold_5:37344548-37349705	26.5516	24.8047	-0.098187
Ciclev10004756m.g	scaffold_9:3324700-3329271	0.815567	0.762009	-0.0979947
Ciclev10009497m.g	scaffold_1:20003431-20007339	54.8866	51.2866	-0.0978703
Ciclev10000985m.g	scaffold_5:41028039-41032953	16.4095	15.3352	-0.0976787
Ciclev10015009m.g	scaffold_2:4486299-4489761	5.78593	5.40821	-0.0973978
Ciclev10025338m.g	scaffold_7:5898918-5904953	94.2545	88.1029	-0.0973723
Ciclev10029658m.g	scaffold_8:23917238-23919206	50.7809	47.475	-0.0971178
Ciclev10001437m.g	scaffold_5:38489256-38490776	20.4366	19.108	-0.0969748
Ciclev10022649m.g	scaffold_3:49508287-49511063	56.0623	52.4182	-0.0969634
Ciclev10033494m.g	scaffold_4:6152569-6156760	1699.97	1589.51	-0.0969239
Ciclev10020077m.g	scaffold_3:7892669-7894409	16.427	15.3621	-0.0966965
Ciclev10025602m.g	scaffold_7:128538-131642	25.4933	23.8424	-0.0965889
Ciclev10002477m.g	scaffold_5:17555131-17558910	21.047	19.6841	-0.0965817
Ciclev10007452m.g,Ciclev10007667m.g,Ciclev10007685m.g	scaffold_1:19496563-19588226	26.6607	24.9409	-0.0962015
Ciclev10003461m.g	scaffold_5:25932192-25937154	91.5864	85.6851	-0.096089
Ciclev10018476m.g	scaffold_3:50068318-50081188	7.94149	7.43045	-0.0959599
Ciclev10032366m.g	scaffold_4:23569923-23573605	106.825	99.9664	-0.0957285
Ciclev10004730m.g	scaffold_9:22728309-22730086	73.5719	68.8699	-0.0952824
Ciclev10014911m.g	scaffold_2:9383252-9392832	15.7383	14.7338	-0.095154
Ciclev10016445m.g	scaffold_2:133670-137229	31.6246	29.6064	-0.0951386
Ciclev10022373m.g	scaffold_3:8256397-8263550	154.479	144.638	-0.0949621
Ciclev10004983m.g	scaffold_9:21120310-21124458	54.5744	51.0982	-0.0949513
Ciclev10029217m.g	scaffold_8:22510546-22513077	23.8238	22.3064	-0.0949441
Ciclev10001050m.g	scaffold_5:35898988-35902917	14.8136	13.8702	-0.0949432
Ciclev10032289m.g	scaffold_4:1991726-1994096	75.3413	70.5439	-0.0949198
Ciclev10001148m.g	scaffold_5:38869619-38875564	57.8034	54.1306	-0.0947097
Ciclev10000126m.g	scaffold_5:29584087-29587183	0.428913	0.401669	-0.0946782
Ciclev10025626m.g	scaffold_7:9462802-9467067	54.5356	51.0729	-0.094638
-	scaffold_3:24926132-24927462	123.269	115.444	-0.0946099
Ciclev10009009m.g	scaffold_1:28777752-28780784	17.6681	16.547	-0.0945756
Ciclev10032245m.g	scaffold_4:24025104-24027334	15.2298	14.2663	-0.0942868
-	scaffold_5:5256426-5257045	7.41492	6.94828	-0.0937759
Ciclev10003408m.g	scaffold_5:38357215-38360227	0.812103	0.761031	-0.0937074
Ciclev10019380m.g	scaffold_3:49239725-49245837	67.586	63.3388	-0.0936343
Ciclev10014241m.g	scaffold_2:11310624-11315360	4.59676	4.30809	-0.0935698
Ciclev10022696m.g	scaffold_3:41709920-41714003	21.4076	20.0643	-0.0934965
Ciclev10018249m.g	scaffold_2:26499631-26508155	11.708	10.9734	-0.0934907
Ciclev10013436m.g	scaffold_6:18322427-18328900	55.8741	52.3733	-0.0933493
Ciclev10009135m.g	scaffold_1:24390380-24394113	31.8409	29.8473	-0.0932831
Ciclev10032274m.g	scaffold_4:23248422-23252556	48.5491	45.5098	-0.0932689
Ciclev10000611m.g	scaffold_5:42898493-42904653	28.2947	26.5342	-0.0926816
Ciclev10007583m.g	scaffold_1:25852011-25861455	9.68151	9.08041	-0.0924739
Ciclev10007648m.g	scaffold_1:28501439-28505393	10.8478	10.1763	-0.0921966
Ciclev10016037m.g	scaffold_2:3777396-3781539	30.6563	28.7589	-0.0921756
Ciclev10009036m.g	scaffold_1:512674-514181	24.3788	22.8712	-0.092091
Ciclev10009729m.g	scaffold_1:3647538-3651252	45.0082	42.2273	-0.0920138
Ciclev10022984m.g	scaffold_3:9862170-9866058	35.6276	33.4284	-0.0919191
Ciclev10017326m.g	scaffold_2:24737092-24836931	24.7538	23.2272	-0.0918335

Ciclev10025487m.g	scaffold_7:2713509-2717859	7.93905	7.45023	-0.0916811
Ciclev10008722m.g	scaffold_1:28002805-28008684	14.0926	13.2268	-0.0914729
Ciclev10025742m.g	scaffold_7:14599148-14601610	7.0178	6.58693	-0.0914123
Ciclev10001691m.g	scaffold_5:39469303-39473928	8.87123	8.32753	-0.0912451
Ciclev10018380m.g	scaffold_2:11393302-11396922	25.226	23.6803	-0.0912269
Ciclev10028681m.g	scaffold_8:24416581-24418982	3.29003	3.0885	-0.0911936
Ciclev10025928m.g	scaffold_7:9162737-9167341	8.88332	8.33984	-0.0910786
Ciclev10000183m.g	scaffold_5:41722611-41729501	6.57681	6.17457	-0.0910495
Ciclev10031678m.g	scaffold_4:7896108-7902453	18.8295	17.6809	-0.0908061
Ciclev10016959m.g	scaffold_2:30886468-30892663	48.4862	45.5294	-0.0907773
Ciclev10011306m.g	scaffold_6:24135780-24139460	11.9743	11.2441	-0.0907648
Ciclev10016676m.g	scaffold_2:2986242-2991381	55.2723	51.9036	-0.0907224
Ciclev10012555m.g	scaffold_6:19488228-19490353	3.26055	3.06219	-0.0905498
Ciclev10029477m.g	scaffold_8:4139952-4142124	17.759	16.6791	-0.0905152
Ciclev10019750m.g	scaffold_3:4254260-4259770	64.1629	60.2674	-0.0903612
Ciclev10000763m.g	scaffold_5:38477880-38481495	6.94233	6.52223	-0.0900531
Ciclev10005606m.g	scaffold_9:6889169-6891419	8.59204	8.07216	-0.0900454
Ciclev10002727m.g	scaffold_5:17899103-17900709	72.7723	68.369	-0.0900452
Ciclev10000514m.g	scaffold_5:26960451-26976490	18.273	17.1682	-0.0899739
Ciclev10022790m.g	scaffold_3:6322932-6325387	25.6751	24.1233	-0.089939
Ciclev10014980m.g	scaffold_2:34548502-34551175	5.33654	5.0141	-0.0899116
Ciclev10001333m.g	scaffold_5:26545479-26551827	26.8896	25.2676	-0.0897602
Ciclev10027741m.g,Ciclev10030442m.g	scaffold_8:1721092-1734306	32.5238	30.5625	-0.0897347
Ciclev10022961m.g	scaffold_3:49059460-49061723	82.2641	77.3051	-0.0897009
Ciclev10020255m.g	scaffold_3:36924550-37054826	9.18625	8.63409	-0.0894318
Ciclev10011975m.g	scaffold_6:8871294-8873669	3.95996	3.72206	-0.089386
Ciclev10008147m.g	scaffold_1:27636798-27648110	64.4653	60.5925	-0.0893824
Ciclev10029589m.g	scaffold_8:803823-805726	41.7741	39.2767	-0.0889324
Ciclev10005146m.g	scaffold_9:270955-272594	8.17855	7.6897	-0.0889191
Ciclev10021021m.g	scaffold_3:25645229-25649184	60.0282	56.444	-0.0888198
Ciclev10030921m.g	scaffold_4:14457765-14462726	7.37384	6.93378	-0.0887747
Ciclev10008515m.g	scaffold_1:233491-237597	24.8578	23.3821	-0.0882893
Ciclev10024303m.g	scaffold_3:45973845-45976552	5.06489	4.76461	-0.0881732
Ciclev10031484m.g	scaffold_4:6203851-6205425	10.3053	9.69453	-0.0881417
Ciclev10021697m.g	scaffold_3:743413-746343	69.9752	65.8299	-0.0881013
Ciclev10024071m.g	scaffold_3:24856113-24862275	9.28245	8.73444	-0.0877908
Ciclev10029177m.g	scaffold_8:24724842-24727091	152.776	143.765	-0.087707
Ciclev10015374m.g	scaffold_2:12286243-12288765	9.73474	9.16267	-0.0873745
Ciclev10028774m.g	scaffold_8:1491361-1556980	5.26303	4.95393	-0.0873221
Ciclev10009272m.g	scaffold_1:25492134-25496306	130.761	123.092	-0.0871912
Ciclev10022254m.g	scaffold_3:36924550-37054826	23.2109	21.8498	-0.0871796
Ciclev10032408m.g	scaffold_4:14351919-14356757	34.8606	32.8187	-0.0870815
Ciclev10012648m.g	scaffold_6:6201315-6203541	17.8215	16.7784	-0.0870143
Ciclev10020907m.g	scaffold_3:7058190-7060963	3.84183	3.61714	-0.0869422
Ciclev10020223m.g	scaffold_3:3730261-3733932	75.6661	71.2566	-0.0866226
Ciclev10015215m.g	scaffold_2:23163672-23168238	27.3152	25.7241	-0.0865869
Ciclev10016872m.g	scaffold_2:8750429-8751934	11.6006	10.9249	-0.0865818
Ciclev10023235m.g	scaffold_3:8254344-8256107	182.768	172.13	-0.0865139
Ciclev10006097m.g	scaffold_9:25802830-25803684	5.43346	5.11773	-0.0863672
Ciclev10015438m.g	scaffold_2:22791516-22794000	22.4844	21.1804	-0.0861928
Ciclev10019225m.g	scaffold_3:46398621-46401196	10.9098	10.2782	-0.0860386
Ciclev10032560m.g	scaffold_4:18835582-18837278	41.1424	38.7658	-0.0858417
Ciclev10014843m.g	scaffold_2:28968667-28973736	21.08	19.8636	-0.0857479
Ciclev10022181m.g	scaffold_3:42322391-42325025	6.47418	6.10087	-0.0856818
Ciclev10006919m.g	scaffold_9:14240153-14241982	14.5489	13.7103	-0.0856555

Ciclev10024068m.g	scaffold_3:31341369-31350323	16.0571	15.1316	-0.0856476
Ciclev10003998m.g	scaffold_5:41562454-41565167	6.45038	6.07865	-0.0856336
Ciclev10011716m.g	scaffold_6:23354840-23360917	9.48983	8.94335	-0.0855679
Ciclev10030117m.g	scaffold_8:11873007-11877160	1.89355	1.78466	-0.0854456
Ciclev10000130m.g	scaffold_5:36049676-36056860	3.23067	3.04504	-0.0853737
Ciclev10008289m.g	scaffold_1:2556147-2560603	28.9885	27.3239	-0.0853142
Ciclev10005931m.g	scaffold_9:4088439-4092614	325.848	307.166	-0.0851804
Ciclev10020830m.g	scaffold_3:43120047-43123491	25.692	24.2205	-0.085088
Ciclev10028687m.g	scaffold_8:18726775-18729035	12.6381	11.9155	-0.0849407
Ciclev10010576m.g	scaffold_1:23449429-23450071	213.777	201.568	-0.0848392
Ciclev10030857m.g	scaffold_4:9058741-9062625	0.925019	0.872229	-0.0847775
Ciclev10007968m.g	scaffold_1:3567763-3571574	42.5735	40.1581	-0.0842661
Ciclev10020581m.g	scaffold_3:13437029-13440178	281.568	265.698	-0.0836973
Ciclev10021956m.g	scaffold_3:9218595-9223630	45.9844	43.3929	-0.0836863
Ciclev10006070m.g	scaffold_9:29888205-29890214	47.7298	45.0431	-0.0835831
Ciclev10007868m.g	scaffold_1:26187065-26200106	29.9104	28.2306	-0.0833876
-	scaffold_7:19332457-19333946	8.04178	7.59018	-0.0833807
Ciclev10012536m.g	scaffold_6:18194883-18202571	20.4292	19.2849	-0.0831658
Ciclev10032759m.g	scaffold_4:23489032-23491138	8.14483	7.68935	-0.0830225
Ciclev10001644m.g	scaffold_5:42455993-42459424	13.7972	13.0257	-0.083009
Ciclev10017840m.g	scaffold_2:28403936-28488203	2.58905	2.44433	-0.0829842
Ciclev10011373m.g	scaffold_6:19498167-19507476	31.3713	29.623	-0.0827271
-	scaffold_8:19341975-19342972	3.39925	3.20982	-0.0827262
Ciclev10017018m.g	scaffold_2:22208364-22210333	126.362	119.332	-0.0825868
Ciclev10031118m.g	scaffold_4:17014581-17026325	55.495	52.4124	-0.0824512
Ciclev10014485m.g	scaffold_2:5185309-5189362	5.83679	5.51357	-0.0821891
Ciclev10012210m.g	scaffold_6:16397312-16411495	32.6704	30.8649	-0.0820185
-	scaffold_8:3399074-3434977	61.5317	58.1397	-0.0818063
Ciclev10031225m.g	scaffold_4:24231401-24235138	14.1056	13.328	-0.0818006
Ciclev10016068m.g	scaffold_2:11675928-11677404	13.4541	12.7151	-0.0815078
Ciclev10029072m.g	scaffold_8:3203192-3225772	0.281258	0.265835	-0.0813621
Ciclev10021116m.g	scaffold_3:49725478-49729775	24.0707	22.7514	-0.0813214
Ciclev10027865m.g	scaffold_8:24130781-24135421	28.8683	27.2867	-0.0812888
Ciclev10021262m.g	scaffold_3:28395654-28400713	17.5195	16.5602	-0.0812408
Ciclev10011558m.g	scaffold_6:23519245-23522691	16.7129	15.7983	-0.0811983
Ciclev10017002m.g	scaffold_2:36140662-36142765	44.4988	42.0667	-0.0810897
Ciclev10019420m.g	scaffold_3:706204-709582	32.9148	31.1197	-0.0809073
Ciclev10026473m.g	scaffold_7:14251173-14252914	4.67708	4.42201	-0.080904
Ciclev10020638m.g	scaffold_3:11048519-11053004	25.1598	23.789	-0.0808229
Ciclev10016204m.g	scaffold_2:21562074-21565269	66.1584	62.5569	-0.0807553
Ciclev10029284m.g	scaffold_8:6700440-6703748	75.798	71.6756	-0.0806773
Ciclev10027908m.g	scaffold_8:5489111-5494845	55.3638	52.3546	-0.0806272
Ciclev10021282m.g	scaffold_3:2568401-2573782	26.8165	25.3592	-0.0806113
Ciclev10013167m.g,Ciclev10013305m.g	scaffold_6:2375843-2381349	19.4998	18.4438	-0.0803243
Ciclev10008432m.g	scaffold_1:125554-130613	83.9639	79.4214	-0.0802412
Ciclev10032790m.g	scaffold_4:21310164-21312801	25.4578	24.0806	-0.0802388
Ciclev10020540m.g	scaffold_3:8228693-8232723	20.6642	19.5471	-0.080183
Ciclev10001278m.g	scaffold_5:39349613-39352321	19.8487	18.7757	-0.0801809
Ciclev10000727m.g	scaffold_5:15349807-15357863	46.7409	44.2155	-0.0801338
Ciclev10013106m.g	scaffold_6:10325664-10328016	8.31372	7.86466	-0.0801094
Ciclev10010114m.g	scaffold_1:3049949-3056785	12.4229	11.7519	-0.0801057
Ciclev10014413m.g	scaffold_2:34351104-34353959	3.64104	3.44467	-0.079985
Ciclev10015585m.g	scaffold_2:9091924-9095744	28.1109	26.5951	-0.0799697
Ciclev10000419m.g	scaffold_5:914285-943659	20.9675	19.837	-0.0799567
Ciclev10014526m.g	scaffold_2:156116-163601	19.3278	18.2863	-0.0799123

-	scaffold_8:3916201-3918592	61.2081	57.9108	-0.0798901
-	scaffold_5:28539341-28801354	61.3437	58.0435	-0.0797809
Ciclev10030684m.g	scaffold_4:1197439-1202880	4.13444	3.91214	-0.0797342
Ciclev10005060m.g	scaffold_9:2119183-2125696	30.254	28.6292	-0.0796361
Ciclev10001767m.g	scaffold_5:14808605-14812187	30.535	28.8972	-0.0795363
Ciclev10029548m.g,Ciclev10030175m.g,Ciclev10030367m.g	scaffold_8:15046335-15072887	32.0027	30.2864	-0.079527
Ciclev10029160m.g	scaffold_8:23158291-23162418	49.2703	46.6287	-0.0794997
Ciclev10025148m.g	scaffold_7:15505555-15510930	133.571	126.437	-0.0791861
Ciclev10021949m.g	scaffold_3:40219314-40224503	15.0375	14.2366	-0.0789575
Ciclev10032493m.g	scaffold_4:19089650-19093114	45.0558	42.6576	-0.0789093
Ciclev10032714m.g	scaffold_4:2861846-2865826	35.1151	33.2476	-0.0788421
Ciclev10019554m.g	scaffold_3:1223713-1226490	31.6686	29.9867	-0.0787336
Ciclev10023872m.g	scaffold_3:44620868-44623952	3.34854	3.17096	-0.0786149
Ciclev10012355m.g	scaffold_6:16795059-16797562	8.73252	8.26954	-0.0785912
Ciclev10021655m.g	scaffold_3:43284409-43286714	6.02	5.70108	-0.078529
Ciclev10020846m.g	scaffold_3:48389459-48395263	128.245	121.452	-0.0785175
Ciclev10012805m.g	scaffold_6:10349976-10351202	219.693	208.058	-0.0785077
Ciclev10014134m.g	scaffold_2:11503783-11518483	9.7027	9.18884	-0.0785036
Ciclev10024300m.g	scaffold_3:28021044-28027037	1.35003	1.27892	-0.0780567
Ciclev10028492m.g	scaffold_8:1434576-1439288	20.7837	19.6906	-0.0779465
Ciclev10012450m.g	scaffold_6:23053706-23056690	38.9407	36.8984	-0.0777222
Ciclev10022751m.g	scaffold_3:37434349-37435141	3.55422	3.36788	-0.0776902
Ciclev10026102m.g	scaffold_7:2364854-2367187	16.3739	15.516	-0.0776398
-	scaffold_9:20256125-20256414	84.6212	80.1877	-0.0776382
Ciclev10003466m.g	scaffold_5:35313169-35320014	17.3909	16.4807	-0.0775521
Ciclev10011353m.g	scaffold_6:18440759-18447914	84.9955	80.5578	-0.0773612
Ciclev10030694m.g	scaffold_4:3346410-3350676	24.5975	23.3145	-0.0772826
Ciclev10024611m.g	scaffold_3:24964762-24969178	4.79477	4.54534	-0.0770733
Ciclev10024916m.g	scaffold_7:11329166-11340059	12.4194	11.7744	-0.0769431
Ciclev10014251m.g	scaffold_2:26938142-26943385	33.038	31.3226	-0.0769231
Ciclev10014942m.g	scaffold_2:23824142-23825989	26.1262	24.7713	-0.0768272
Ciclev10023085m.g	scaffold_3:50946963-50949402	22.6516	21.4769	-0.0768251
Ciclev10029128m.g	scaffold_8:18289363-18294202	34.9217	33.1124	-0.076752
Ciclev10028780m.g	scaffold_8:5582934-5585920	74.6677	70.8126	-0.0764776
Ciclev10022536m.g	scaffold_3:17739030-17741083	111.799	106.03	-0.0764256
Ciclev10000018m.g	scaffold_5:18122994-18137457	2.46986	2.34282	-0.0761797
Ciclev10012009m.g	scaffold_6:20229288-20233481	37.5411	35.6204	-0.0757672
Ciclev10018157m.g	scaffold_2:27899209-27902966	36.9438	35.0549	-0.0757145
Ciclev10001463m.g	scaffold_5:17168718-17173839	32.134	30.4913	-0.0757008
Ciclev10025274m.g	scaffold_7:6192194-6207492	22.7389	21.5778	-0.0756139
Ciclev10032851m.g	scaffold_4:715596-718039	56.4208	53.5421	-0.075554
Ciclev10014147m.g	scaffold_2:19900319-19914295	10.4159	9.88449	-0.0755466
Ciclev10029529m.g	scaffold_8:864566-866877	56.0713	53.2111	-0.0755366
Ciclev10032093m.g	scaffold_4:23150166-23151971	27.0124	25.6354	-0.0754837
Ciclev10019276m.g	scaffold_3:47005592-47008998	24.547	23.2968	-0.0754163
Ciclev10006852m.g	scaffold_9:24201011-24203922	346.44	328.819	-0.0753129
-	scaffold_4:6533559-6535637	11.6096	11.0195	-0.0752569
Ciclev10006315m.g	scaffold_9:30040456-30046344	31.6667	30.0581	-0.0752126
Ciclev10008285m.g	scaffold_1:28686869-28690073	9.14505	8.68258	-0.0748673
Ciclev10014904m.g	scaffold_2:19028330-19031668	45.6916	43.381	-0.0748653
Ciclev10005495m.g	scaffold_9:12740845-12745213	18.175	17.2565	-0.0748145
Ciclev10008098m.g	scaffold_1:27984614-27989566	19.7352	18.7379	-0.0748115
Ciclev10004831m.g	scaffold_9:30991997-30999295	5.93143	5.63183	-0.0747758
Ciclev10005697m.g	scaffold_9:16055660-16060041	16.7946	15.9499	-0.0744524

Ciclev10028561m.g	scaffold_8:23774308-23780334	37.749	35.8581	-0.0741425
-	scaffold_1:12982516-12985117	6.2203	5.90917	-0.0740283
Ciclev10017290m.g	scaffold_2:15759168-15765725	0.522707	0.496563	-0.0740238
Ciclev10022151m.g	scaffold_3:41434063-41437122	68.2194	64.8102	-0.0739606
Ciclev10005607m.g	scaffold_9:22732689-22736939	30.5307	29.0081	-0.0738041
Ciclev10007567m.g	scaffold_1:28754554-28760192	13.4284	12.7613	-0.0735116
Ciclev10006353m.g	scaffold_9:28497953-28514398	235.082	223.418	-0.0734199
Ciclev10013269m.g	scaffold_6:7924188-7928192	0.239342	0.227471	-0.0733871
Ciclev10011366m.g	scaffold_6:16681649-16686551	10.5145	9.99571	-0.0730051
Ciclev10004883m.g	scaffold_9:2391518-2396505	18.7719	17.8473	-0.072864
Ciclev10001200m.g	scaffold_5:18169571-18173979	40.9099	38.8984	-0.072739
Ciclev10025803m.g	scaffold_7:7138272-7141719	30.136	28.6556	-0.0726703
Ciclev10016472m.g	scaffold_2:34075359-34081355	12.0188	11.4291	-0.0725777
Ciclev10031166m.g	scaffold_4:8356325-8362969	18.2731	17.3774	-0.0725052
Ciclev10031666m.g	scaffold_4:25623248-25628106	21.3433	20.2974	-0.0724922
Ciclev10018558m.g	scaffold_3:38617236-38627732	14.0269	13.3405	-0.0723832
Ciclev10021488m.g	scaffold_3:30873885-30877367	68.2748	64.934	-0.0723792
Ciclev10017566m.g	scaffold_2:10992197-10994372	0.691751	0.657942	-0.0722934
Ciclev10017711m.g	scaffold_2:5273993-5337902	3.06741	2.91758	-0.0722518
Ciclev10004375m.g	scaffold_9:3367717-3376233	20.0478	19.0691	-0.0722006
Ciclev10005719m.g	scaffold_9:1398262-1400187	46.2565	44.0018	-0.0720919
Ciclev10009016m.g	scaffold_1:26086436-26090277	31.9366	30.3825	-0.0719698
Ciclev10028070m.g	scaffold_8:793819-799239	120.446	114.589	-0.0719131
Ciclev10004705m.g	scaffold_9:2539406-2543899	8.51683	8.10489	-0.0715234
Ciclev10027674m.g	scaffold_8:25028327-25039422	18.9724	18.0576	-0.0713015
Ciclev10016900m.g	scaffold_2:35073072-35075210	181.622	172.904	-0.0709703
Ciclev10001335m.g	scaffold_5:20599440-20601673	18.5515	17.6638	-0.0707413
Ciclev10024128m.g	scaffold_3:43354399-43357281	25.4375	24.2204	-0.0707342
Ciclev10011087m.g	scaffold_6:14327887-14333437	26.4632	25.1971	-0.0707268
Ciclev10027837m.g	scaffold_8:23622130-23626601	47.831	45.5483	-0.0705482
Ciclev10001181m.g	scaffold_5:34574463-34591686	54.5976	51.9944	-0.0704793
Ciclev10001647m.g	scaffold_5:43288730-43293268	30.0957	28.6646	-0.0702852
Ciclev10012715m.g	scaffold_6:231680-234325	127.525	121.463	-0.0702605
Ciclev10011231m.g	scaffold_6:24666332-24671130	22.9137	21.8272	-0.0700856
Ciclev10016062m.g	scaffold_2:33604014-33606731	7.36305	7.01431	-0.070002
Ciclev10022880m.g	scaffold_3:7361611-7363184	193.224	184.075	-0.0699825
Ciclev10001794m.g	scaffold_5:6699439-6702636	17.6849	16.8484	-0.0699084
Ciclev10032621m.g	scaffold_4:14671075-14673988	54.7883	52.2018	-0.0697675
Ciclev10017333m.g	scaffold_2:405429-407503	8.63497	8.22747	-0.0697418
Ciclev10024917m.g	scaffold_7:11271704-11277014	115.26	109.85	-0.0693587
Ciclev10021373m.g	scaffold_3:40245541-40247885	7.62203	7.26451	-0.0693097
Ciclev10005772m.g	scaffold_9:27690957-27694712	21.086	20.098	-0.0692351
Ciclev10022527m.g	scaffold_3:9740615-9742355	243.625	232.251	-0.0689814
Ciclev10025695m.g	scaffold_7:15364010-15371713	41.1878	39.2663	-0.0689231
Ciclev10013034m.g	scaffold_6:24221841-24224671	485.034	462.437	-0.0688306
Ciclev10009429m.g	scaffold_1:20535940-20543308	43.3701	41.3501	-0.0688128
Ciclev10032022m.g	scaffold_4:24108947-24111030	3.52927	3.36554	-0.068533
Ciclev10026427m.g	scaffold_7:5575622-5577458	32.3878	30.8856	-0.0685162
Ciclev10029577m.g	scaffold_8:13121586-13122918	55.7221	53.1385	-0.0684922
Ciclev10002910m.g	scaffold_5:42452913-42455193	45.6998	43.5816	-0.0684714
Ciclev10007430m.g	scaffold_1:28172272-28175740	5.1817	4.94215	-0.0682844
Ciclev10030805m.g	scaffold_4:9293820-9298832	17.1829	16.3895	-0.0682054
Ciclev10021330m.g	scaffold_3:4068107-4070714	12.3312	11.762	-0.0681697
Ciclev10001891m.g	scaffold_5:38134661-38138860	18.3299	17.485	-0.0680864
Ciclev10018900m.g	scaffold_3:28065477-28341901	14.8878	14.2027	-0.0679633
Ciclev10006100m.g	scaffold_9:28534223-28535588	31.6567	30.2118	-0.0674001

Ciclev10001149m.g	scaffold_5:34773463-34775206	13.0643	12.4686	-0.0673313
Ciclev10012646m.g	scaffold_6:23416403-23418629	26.7044	25.4868	-0.0673221
Ciclev10031828m.g	scaffold_4:2058519-2060958	6.85504	6.54313	-0.0671841
Ciclev10011475m.g	scaffold_6:20380413-20384590	7.75228	7.39971	-0.0671522
Ciclev10032786m.g	scaffold_4:854970-858299	46.4641	44.3524	-0.0671047
Ciclev10026104m.g	scaffold_7:3692221-3699678	14.9584	14.2788	-0.0670764
Ciclev10029345m.g	scaffold_8:23607454-23610267	34.343	32.7868	-0.0669043
Ciclev10024803m.g	scaffold_7:8856632-8864522	13.5935	12.9811	-0.066509
Ciclev10011556m.g	scaffold_6:25001585-25004619	15.3105	14.6237	-0.0662151
Ciclev10008985m.g	scaffold_1:28330809-28332669	58.0875	55.4837	-0.0661635
-	scaffold_5:4076819-4284428	5.67908	5.42464	-0.0661298
Ciclev10032703m.g	scaffold_4:2422860-2424838	670.12	640.129	-0.0660556
Ciclev10022634m.g	scaffold_3:1828214-1831739	6.34401	6.06048	-0.0659628
Ciclev10031376m.g	scaffold_4:22737492-22740984	51.6924	49.3873	-0.0658119
Ciclev10016287m.g	scaffold_2:34911810-34912896	1641.26	1568.16	-0.065729
Ciclev10020730m.g	scaffold_3:3948056-3951755	11.2715	10.7721	-0.0653855
Ciclev10033287m.g	scaffold_4:19895236-19895854	2449.17	2340.76	-0.0653199
Ciclev10008462m.g	scaffold_1:9358333-9384788	25.1683	24.055	-0.0652684
Ciclev10000215m.g	scaffold_5:40200372-40208310	35.8686	34.2831	-0.0652228
Ciclev10026806m.g	scaffold_7:20056474-20058190	189.391	181.035	-0.065099
Ciclev10025309m.g	scaffold_7:4680000-4688815	66.576	63.6459	-0.0649346
Ciclev10011693m.g	scaffold_6:14244492-14249201	108.156	103.409	-0.0647426
Ciclev10010113m.g	scaffold_1:27293753-27296029	86.0216	82.2556	-0.0645843
Ciclev10020863m.g	scaffold_3:50693415-50695171	34.7144	33.1949	-0.0645731
Ciclev10005723m.g	scaffold_9:28437686-28440187	9.17113	8.77066	-0.0644146
Ciclev10001178m.g	scaffold_5:41443666-41446709	53.9977	51.6436	-0.06431
Ciclev10000845m.g	scaffold_5:26866035-26868420	1.70017	1.6261	-0.0642697
Ciclev10012967m.g	scaffold_6:16344481-16346981	17.379	16.6223	-0.0642261
Ciclev10008306m.g	scaffold_1:2997512-3001226	3.63255	3.47442	-0.0642087
Ciclev10005473m.g	scaffold_9:29180966-29185849	271.227	259.427	-0.0641745
-	scaffold_3:49976284-49978080	7.14577	6.83533	-0.0640775
Ciclev10019113m.g	scaffold_3:10096639-10102796	13.2381	12.6664	-0.063688
Ciclev10016034m.g	scaffold_2:31701376-31704115	28.8858	27.6396	-0.0636243
Ciclev10015507m.g	scaffold_2:9927539-9930839	0.696755	0.666715	-0.0635812
Ciclev10027954m.g	scaffold_8:12800121-12845493	121.338	116.107	-0.0635773
Ciclev10021047m.g	scaffold_3:36770524-36777412	147.876	141.502	-0.0635626
Ciclev10019703m.g	scaffold_3:5311686-5313601	4.39496	4.20572	-0.063496
Ciclev10008994m.g	scaffold_1:9300781-9304559	62.6974	60.0009	-0.0634217
Ciclev10010166m.g,Ciclev10010689m.g	scaffold_1:3877579-3881441	16.7157	15.9969	-0.0634116
Ciclev10011552m.g	scaffold_6:18315627-18321185	23.4031	22.3974	-0.0633657
Ciclev10004906m.g,Ciclev10004912m.g	scaffold_9:27015821-27051136	3.75128	3.5901	-0.0633602
Ciclev10016274m.g	scaffold_2:29140508-29143804	212.597	203.472	-0.0632913
Ciclev10001533m.g	scaffold_5:35420547-35446874	34.0186	32.5594	-0.0632479
Ciclev10028048m.g	scaffold_8:24253922-24258950	28.584	27.3584	-0.0632238
Ciclev10024541m.g	scaffold_3:1424548-1432166	16.8535	16.1377	-0.0626087
Ciclev10006141m.g	scaffold_9:3696391-3698741	66.2566	63.4453	-0.0625514
Ciclev10028624m.g	scaffold_8:18356458-18357631	3.19022	3.05488	-0.0625381
Ciclev10005183m.g	scaffold_9:14732402-14737039	87.8501	84.1313	-0.0624013
-	scaffold_5:9669156-9669808	4.3324	4.14928	-0.0623045
Ciclev10032688m.g	scaffold_4:21601247-21603994	25.9248	24.8291	-0.0622995
Ciclev10029713m.g	scaffold_8:18802723-18804492	65.7143	62.9383	-0.0622673
Ciclev10001274m.g	scaffold_5:39459444-39464982	14.3797	13.7726	-0.0622383
Ciclev10007734m.g	scaffold_1:26059372-26066070	18.7457	17.9544	-0.0622233
Ciclev10032247m.g	scaffold_4:10668473-10673182	13.5493	12.9774	-0.0622213

Ciclev10011033m.g	scaffold_6:20201379-20214287	180.781	173.164	-0.0621056
Ciclev10021365m.g	scaffold_3:4794700-4799518	10.3828	9.94743	-0.0618011
Ciclev10019755m.g,Ciclev10019899m.g	scaffold_3:7443493-7455910	11.6035	11.118	-0.0616603
Ciclev10006225m.g	scaffold_9:27761639-27763629	295.243	282.93	-0.0614586
Ciclev10002177m.g	scaffold_5:32970438-32972234	14.4287	13.8273	-0.0614245
Ciclev10014369m.g,Ciclev10018231m.g	scaffold_2:25165186-25173296	10.6507	10.2082	-0.0612185
Ciclev10019212m.g	scaffold_3:41490790-41497289	10.0391	9.62246	-0.0611475
Ciclev10028950m.g	scaffold_8:4028427-4030228	10.96	10.5063	-0.0609937
Ciclev10016061m.g	scaffold_2:13502267-13505597	233.464	223.803	-0.0609682
Ciclev10015043m.g	scaffold_2:10530810-10532986	3.63893	3.48898	-0.0607081
Ciclev10024811m.g	scaffold_7:2659389-2665704	15.5439	14.9043	-0.0606187
Ciclev10030283m.g	scaffold_8:18006578-18008605	34.4207	33.0048	-0.0606007
Ciclev10028244m.g	scaffold_8:20268210-20272901	11.735	11.2524	-0.0605784
Ciclev10026876m.g	scaffold_7:3930787-3932748	89.523	85.8436	-0.0605477
Ciclev10013136m.g	scaffold_6:25190230-25193223	32.461	31.1292	-0.0604391
Ciclev10031735m.g	scaffold_4:22259922-22264122	16.38	15.7087	-0.0603693
Ciclev10026249m.g	scaffold_7:13022420-13028396	39.7651	38.1356	-0.0603641
Ciclev10005645m.g	scaffold_9:785200-787443	17.631	16.9092	-0.0603098
Ciclev10009664m.g,Ciclev10009985m.g	scaffold_1:22775176-22783365	41.1485	39.4654	-0.06025
Ciclev10012262m.g	scaffold_6:6694889-6751336	6.03833	5.79143	-0.0602295
Ciclev10026744m.g	scaffold_7:6317443-6319715	1.60863	1.5429	-0.0601876
Ciclev10013078m.g	scaffold_6:14472656-14477261	98.1367	94.1297	-0.0601429
Ciclev10031802m.g	scaffold_4:21384127-21387825	15.6684	15.0294	-0.0600703
Ciclev10006777m.g	scaffold_9:2196657-2203788	8.22193	7.88672	-0.0600503
Ciclev10009343m.g	scaffold_1:27229292-27232045	10.2708	9.85293	-0.0599288
Ciclev10000277m.g	scaffold_5:31469286-31718393	0.570251	0.547083	-0.0598384
Ciclev10029753m.g	scaffold_8:20187445-20188888	128.857	123.631	-0.0597279
Ciclev10005597m.g	scaffold_9:12562561-12570023	26.0395	24.9866	-0.0595509
Ciclev10011337m.g	scaffold_6:6794723-6799925	15.4532	14.8286	-0.0595201
Ciclev10014540m.g	scaffold_2:25194944-25197852	42.1001	40.3992	-0.0594967
Ciclev10004596m.g	scaffold_9:5135149-5143656	15.2974	14.6803	-0.0594029
Ciclev10017874m.g	scaffold_2:25395950-25399299	22.9973	22.0704	-0.0593494
Ciclev10000028m.g	scaffold_5:39404242-39410935	9.49105	9.10859	-0.0593409
Ciclev10005159m.g	scaffold_9:17809951-17817998	222.59	213.656	-0.0591006
Ciclev10027241m.g	scaffold_7:17843932-17856374	10.6171	10.1913	-0.0590565
Ciclev10009994m.g	scaffold_1:27252346-27252852	84.5528	81.1628	-0.0590348
Ciclev10026865m.g	scaffold_7:14857550-14859352	18.8349	18.0823	-0.0588358
Ciclev10002760m.g	scaffold_5:26168593-26171673	19.4034	18.6353	-0.0582694
Ciclev10030149m.g	scaffold_8:19307457-19308075	1.57904	1.51678	-0.058043
Ciclev10007691m.g	scaffold_1:23428136-23434141	131.995	126.798	-0.0579419
Ciclev10007327m.g	scaffold_1:25337686-25347002	15.8923	15.2675	-0.0578601
Ciclev10026244m.g,Ciclev10027316m.g	scaffold_7:13916806-14069972	2.85087	2.73883	-0.0578431
Ciclev10009763m.g	scaffold_1:1559217-1561070	289.668	278.356	-0.0574651
Ciclev10029416m.g	scaffold_8:22043525-22044300	189.52	182.143	-0.0572785
Ciclev10000142m.g	scaffold_5:34716373-34732757	9.30552	8.94446	-0.0570935
Ciclev10008999m.g	scaffold_1:25255757-25259340	78.6978	75.6607	-0.0567779
Ciclev10024695m.g	scaffold_7:2499556-2509812	9.98779	9.60241	-0.0567691
Ciclev10002977m.g	scaffold_5:36984195-36985449	6.2214	5.98174	-0.0566738
Ciclev10026699m.g	scaffold_7:2583838-2585081	58.6934	56.4331	-0.0566559
Ciclev10024985m.g	scaffold_7:9833677-9840006	27.7338	26.6668	-0.0565979
Ciclev10022269m.g	scaffold_3:50812342-50815054	27.2979	26.2479	-0.0565858
Ciclev10009165m.g	scaffold_1:28254306-28257305	246.651	237.179	-0.0564967

Ciclev10027915m.g,Ciclev10028828m.g	scaffold_8:7666419-7675959	95.2737	91.6168	-0.056466
Ciclev10024563m.g	scaffold_3:18645464-18647805	4.45398	4.28331	-0.0563691
Ciclev10004737m.g	scaffold_9:26903996-26909650	8.6132	8.2834	-0.0563261
Ciclev10011153m.g	scaffold_6:24098001-24112094	16.09	15.4755	-0.0561787
Ciclev10019303m.g	scaffold_3:4679059-4684183	22.1453	21.2997	-0.0561734
Ciclev10028123m.g	scaffold_8:6293019-6297887	20.1521	19.3895	-0.0556568
Ciclev10012240m.g	scaffold_6:20490181-20493800	9.22447	8.87718	-0.0553632
Ciclev10026387m.g	scaffold_7:551892-553909	24.8224	23.8903	-0.05522
Ciclev10031193m.g	scaffold_4:17679209-17681073	2.45962	2.3673	-0.0551937
Ciclev10015951m.g	scaffold_2:9112388-9114946	12.6454	12.1713	-0.0551305
Ciclev10002981m.g	scaffold_5:19557276-19557816	850.349	818.469	-0.0551282
Ciclev10004361m.g	scaffold_9:5021287-5065070	10.6021	10.2062	-0.0549112
Ciclev10015500m.g	scaffold_2:23640210-23644601	21.5993	20.7948	-0.0547624
-	scaffold_3:6153213-6156534	3.81807	3.6759	-0.0547436
Ciclev10028028m.g	scaffold_8:20148632-20155238	25.5664	24.6183	-0.054519
Ciclev10017875m.g	scaffold_2:30325131-30365011	4.77888	4.60179	-0.0544774
Ciclev10031407m.g	scaffold_4:19900465-19906900	17.9094	17.2491	-0.0542005
Ciclev10026233m.g	scaffold_7:17212894-17215562	18.9372	18.2391	-0.0541858
Ciclev10028868m.g	scaffold_8:1431456-1434286	18.5193	17.8379	-0.0540829
Ciclev10031058m.g	scaffold_4:15058919-15066925	45.3798	43.7106	-0.0540676
Ciclev10019508m.g	scaffold_3:39405235-39409867	21.3899	20.6033	-0.0540493
Ciclev10014959m.g	scaffold_2:6636976-6643364	1.45757	1.40402	-0.0539978
Ciclev10012881m.g	scaffold_6:23661555-23664773	42.9062	41.3347	-0.0538305
Ciclev10005094m.g	scaffold_9:2727172-2731614	52.4343	50.5239	-0.053545
Ciclev10005714m.g	scaffold_9:4139971-4142301	36.5197	35.2015	-0.0530392
Ciclev10003403m.g	scaffold_5:34142654-34144954	6.03841	5.82089	-0.0529296
Ciclev10001861m.g	scaffold_5:42129356-42132690	6.10165	5.883	-0.0526468
Ciclev10015907m.g	scaffold_2:32087415-32090369	4.13777	3.98952	-0.0526362
Ciclev10030553m.g	scaffold_4:23774772-23783511	4.37199	4.21592	-0.0524414
Ciclev10024912m.g	scaffold_7:580473-583216	9.77164	9.42456	-0.0521751
Ciclev10020311m.g	scaffold_3:18938161-18963264	33.8015	32.6047	-0.0520067
Ciclev10000021m.g	scaffold_5:39593308-39602756	25.8983	24.9837	-0.0518662
Ciclev10025298m.g	scaffold_7:3323268-3331038	69.9278	67.4623	-0.0517838
Ciclev10012005m.g	scaffold_6:21121428-21126448	8.06692	7.78395	-0.0515167
-	scaffold_1:24538825-24540479	588.168	567.544	-0.0514953
Ciclev10014829m.g	scaffold_2:31235967-31237800	12.3081	11.8781	-0.0513053
Ciclev10012217m.g	scaffold_6:19372754-19376297	6.11094	5.89745	-0.0513036
Ciclev10018645m.g	scaffold_3:44050928-44054570	0.959811	0.926417	-0.0510888
Ciclev10015476m.g	scaffold_2:11598998-11601307	2708.97	2615.03	-0.0509167
Ciclev10001973m.g	scaffold_5:26990578-26998915	106.392	102.709	-0.0508217
Ciclev10006050m.g	scaffold_9:25594177-25597369	108.902	105.155	-0.0505225
Ciclev10032878m.g	scaffold_4:15511442-15513351	9.31459	8.99407	-0.0505193
Ciclev10017190m.g	scaffold_2:31447943-31450530	19.2743	18.6122	-0.0504301
Ciclev10028884m.g	scaffold_8:4938246-4941722	22.1545	21.3949	-0.0503351
Ciclev10018136m.g	scaffold_2:30650914-30658771	41.9495	40.5164	-0.0501466
Ciclev10024896m.g,Ciclev10024905m.g	scaffold_7:14123946-14154295	30.9768	29.9215	-0.0500084
Ciclev10028632m.g	scaffold_8:18652717-18655806	20.1045	19.4199	-0.0499848
Ciclev10001163m.g	scaffold_5:36869386-36872829	33.4711	32.3361	-0.0497716
Ciclev10003878m.g,Ciclev10003980m.g	scaffold_5:13978807-14002743	12.8813	12.4446	-0.0497575
Ciclev10011401m.g	scaffold_6:16986365-16997689	26.0529	25.1715	-0.0496531
Ciclev10015710m.g	scaffold_2:33593641-33595988	15.1195	14.6105	-0.0494053
Ciclev10030683m.g	scaffold_4:22868977-22880751	17.0706	16.4962	-0.0493752
Ciclev10023878m.g	scaffold_3:47729278-47730217	8.55573	8.26875	-0.0492207

-	scaffold_5:25203907-25204323	14.0911	13.6204	-0.0490167
Ciclev10011511m.g	scaffold_6:21186895-21189754	57.1828	55.2727	-0.0490154
Ciclev10008160m.g	scaffold_1:10678652-10684627	95.4129	92.256	-0.0485426
Ciclev10018605m.g	scaffold_3:44150039-44164086	11.2167	10.8467	-0.0483994
Ciclev10023972m.g	scaffold_3:40098482-40100273	36.5667	35.3635	-0.0482676
Ciclev10011104m.g	scaffold_6:21886850-21889998	10.2349	9.89865	-0.0481889
Ciclev10024114m.g	scaffold_3:26591204-26592308	2.68452	2.59642	-0.0481423
Ciclev10030708m.g	scaffold_4:3751929-3756856	23.6736	22.8968	-0.0481285
Ciclev10011562m.g	scaffold_6:20426503-20432090	13.9094	13.4543	-0.047991
Ciclev10016127m.g	scaffold_2:30923397-30926574	32.7691	31.7021	-0.0477558
Ciclev10015325m.g	scaffold_2:31887707-31891985	8.21351	7.94621	-0.0477314
Ciclev10020494m.g,Ciclev10020503m.g	scaffold_3:45271739-45292172	3.70191	3.58166	-0.0476388
Ciclev10031537m.g	scaffold_4:574631-578554	7.55221	7.30741	-0.0475387
Ciclev10000757m.g	scaffold_5:42824869-42827344	3.75551	3.63381	-0.0475264
Ciclev10004891m.g	scaffold_9:30842468-30845657	970.703	939.272	-0.047487
Ciclev10025419m.g	scaffold_7:8242178-8246823	13.3456	12.9137	-0.0474684
Ciclev10001597m.g	scaffold_5:37565046-37569231	47.5068	45.9709	-0.0474139
Ciclev10027807m.g	scaffold_8:6157356-6162759	0.333096	0.322342	-0.0473444
Ciclev10018525m.g	scaffold_3:46389250-46398564	3.73224	3.61219	-0.0471676
Ciclev10015712m.g	scaffold_2:9578497-9584531	18.0126	17.4336	-0.0471319
Ciclev10001184m.g	scaffold_5:17122443-17129244	38.1497	36.9254	-0.0470598
Ciclev10023707m.g	scaffold_3:45528991-45532080	86.6417	83.863	-0.0470259
Ciclev10026804m.g	scaffold_7:13405103-13408719	74.9309	72.5285	-0.0470132
Ciclev10019782m.g	scaffold_3:34014141-34022435	15.0381	14.5564	-0.0469653
Ciclev10009582m.g	scaffold_1:27141516-27145011	37.563	36.3612	-0.0469126
Ciclev10024895m.g	scaffold_7:5566974-5575433	15.7611	15.2579	-0.0468199
Ciclev10023064m.g	scaffold_3:50724060-50729319	137.358	132.98	-0.0467415
-	scaffold_1:16351442-16353996	23.1387	22.4019	-0.0466849
Ciclev10007976m.g	scaffold_1:16361639-16366855	30.0411	29.087	-0.046565
Ciclev10008890m.g	scaffold_1:381256-384321	76.0594	73.6467	-0.0465065
Ciclev10024014m.g	scaffold_3:49901775-49903805	8.21636	7.95587	-0.0464798
Ciclev10007855m.g	scaffold_1:25926884-25938245	21.0873	20.4216	-0.046284
Ciclev10018819m.g	scaffold_3:1966951-1971614	0.76212	0.738141	-0.0461215
Ciclev10021739m.g	scaffold_3:520015-521240	1.4331	1.38812	-0.0460102
Ciclev10007877m.g	scaffold_1:22021975-22031696	14.6795	14.2214	-0.045745
Ciclev10030498m.g	scaffold_4:1331391-1345587	2.34468	2.27154	-0.0457225
Ciclev10022131m.g	scaffold_3:46750835-46754323	173.224	167.822	-0.0457072
Ciclev10028184m.g	scaffold_8:1353306-1359319	10.2943	9.97461	-0.0455114
Ciclev10015233m.g	scaffold_2:36195301-36199265	32.136	31.14	-0.0454208
Ciclev10000797m.g	scaffold_5:39306388-39310225	28.1055	27.2351	-0.0453821
Ciclev10029087m.g	scaffold_8:12895696-12898172	51.8185	50.2295	-0.0449327
Ciclev10021271m.g	scaffold_3:11178538-11181135	26.4666	25.6587	-0.0447233
-	scaffold_4:11523554-11728349	24.9438	24.1828	-0.044703
Ciclev10000111m.g	scaffold_5:5274795-5282044	2.28738	2.21778	-0.0445769
Ciclev10019038m.g	scaffold_3:46724356-46729693	37.0509	35.9264	-0.0444623
Ciclev10014948m.g	scaffold_2:26852380-26857537	31.4429	30.4899	-0.044399
Ciclev10011021m.g	scaffold_6:20253159-20264672	22.1797	21.5087	-0.04432
Ciclev10008674m.g	scaffold_1:6213975-6218993	15.4763	15.0083	-0.0443047
Ciclev10011266m.g	scaffold_6:24606500-24612654	53.0529	51.4534	-0.0441652
Ciclev10000162m.g	scaffold_5:19865022-20070734	0.629714	0.610781	-0.0440419
Ciclev10019862m.g	scaffold_3:50311642-50315371	12.4445	12.0708	-0.0439819
Ciclev10004248m.g	scaffold_9:6358513-6368231	11.8686	11.5124	-0.0439603
Ciclev10012095m.g	scaffold_6:14188336-14190724	18.1171	17.5734	-0.0439581
Ciclev10031339m.g	scaffold_4:18947497-18952548	1.4108	1.36847	-0.0439398

Ciclev10015797m.g,Ciclev10018227m.g	scaffold_2:2361924-2371878	24.5934	23.8585	-0.0437721
Ciclev10015734m.g	scaffold_2:10982872-10988638	12.0346	11.6762	-0.043618
-	scaffold_3:50652980-50654252	530.379	514.654	-0.0434205
Ciclev10005442m.g	scaffold_9:26840222-26845637	38.2664	37.1338	-0.0433462
Ciclev10009123m.g	scaffold_1:8063529-8066056	37.8306	36.7135	-0.0432445
Ciclev10019585m.g	scaffold_3:42599724-42606052	58.3125	56.5931	-0.0431766
Ciclev10014774m.g	scaffold_2:27521106-27527377	42.1798	40.937	-0.0431457
Ciclev10031876m.g	scaffold_4:260907-262357	21.1539	20.5335	-0.0429478
Ciclev10030134m.g	scaffold_8:19553341-19555401	260.771	253.125	-0.0429366
Ciclev10013409m.g	scaffold_6:8193392-8195764	1552.96	1507.46	-0.042902
Ciclev10006749m.g	scaffold_9:12058366-12062169	16.4619	15.9845	-0.0424563
Ciclev10030983m.g	scaffold_4:11918617-11930009	9.47452	9.20096	-0.0422675
Ciclev10006162m.g	scaffold_9:3249709-3250487	209.976	203.922	-0.0422074
Ciclev10007320m.g	scaffold_1:27686387-27690703	17.259	16.7626	-0.0421032
Ciclev10023412m.g	scaffold_3:27206698-27208390	190.191	184.746	-0.041911
Ciclev10033060m.g	scaffold_4:2348400-2350039	357.785	347.592	-0.0416981
Ciclev10015513m.g	scaffold_2:34877897-34879535	6.69803	6.50868	-0.0413721
Ciclev10015933m.g	scaffold_2:33246220-33249159	53.0244	51.528	-0.0413005
Ciclev10008243m.g	scaffold_1:24157318-24162543	18.4296	17.9099	-0.0412634
-	scaffold_2:35022768-35023254	40.9369	39.7853	-0.0411662
Ciclev10002863m.g	scaffold_5:21229071-21230408	160.337	155.827	-0.0411641
Ciclev10002830m.g	scaffold_5:42664618-42666950	92.5366	89.9457	-0.0409695
Ciclev10024772m.g	scaffold_7:5010780-5016990	0.783699	0.761806	-0.0408745
Ciclev10001180m.g,Ciclev10001421m.g	scaffold_5:13886545-13915539	9.97421	9.69657	-0.0407283
Ciclev10021889m.g	scaffold_3:4077972-4120402	65.0587	63.2534	-0.0405999
Ciclev10001099m.g	scaffold_5:43036918-43044008	27.4469	26.6865	-0.0405315
Ciclev10008691m.g	scaffold_1:28574589-28576139	50.4496	49.0601	-0.0402922
Ciclev10009146m.g	scaffold_1:4606911-4610637	27.0428	26.2985	-0.0402627
Ciclev10011598m.g,Ciclev10011599m.g	scaffold_6:8958457-8986420	75.6075	73.5307	-0.0401836
Ciclev10026062m.g	scaffold_7:15572552-15580541	67.2645	65.4232	-0.0400421
Ciclev10009918m.g	scaffold_1:3750238-3752032	19.5626	19.0283	-0.0399506
Ciclev10016118m.g	scaffold_2:35795282-35797273	11.3651	11.0559	-0.0397886
Ciclev10028243m.g	scaffold_8:5069263-5071501	6.34755	6.1749	-0.0397842
Ciclev10002396m.g	scaffold_5:34161720-34162652	44.1719	42.9737	-0.0396742
Ciclev10032969m.g	scaffold_4:22532748-22533489	172.144	167.477	-0.0396539
Ciclev10009235m.g	scaffold_1:20056925-20059238	363.107	353.27	-0.0396229
Ciclev10022677m.g	scaffold_3:49810708-49812838	118.168	114.967	-0.039619
Ciclev10013054m.g	scaffold_6:25359771-25363336	216.463	210.645	-0.0393059
Ciclev10028013m.g	scaffold_8:20600991-20605052	5.86556	5.70799	-0.0392853
Ciclev10000834m.g	scaffold_5:18390056-18395489	2.22197	2.16237	-0.0392234
Ciclev10026416m.g	scaffold_7:7715827-7718957	31.528	30.6835	-0.0391711
Ciclev10018252m.g	scaffold_2:27774679-27778271	119.187	116.006	-0.0390343
Ciclev10008959m.g	scaffold_1:12399261-12405801	62.2313	60.5711	-0.0390111
Ciclev10007469m.g	scaffold_1:12798697-12801839	1.51061	1.47034	-0.0389835
Ciclev10028088m.g	scaffold_8:21981806-21988821	18.7507	18.2525	-0.0388568
Ciclev10000749m.g	scaffold_5:39221209-39227401	15.6839	15.2679	-0.0387852
Ciclev10026516m.g	scaffold_7:6135530-6137411	122.822	119.568	-0.0387368
Ciclev10005795m.g	scaffold_9:28903615-28907377	185.174	180.279	-0.0386471
Ciclev10003241m.g	scaffold_5:42626290-42633313	18.4074	17.9219	-0.038561
Ciclev10020183m.g	scaffold_3:49757721-49762041	17.9917	17.5193	-0.0383833
Ciclev10022255m.g	scaffold_3:46059306-46061610	264.019	257.098	-0.0383265
Ciclev10032303m.g	scaffold_4:25044516-25047286	10.6124	10.3365	-0.0380016
Ciclev10020756m.g	scaffold_3:70052-75415	41.8057	40.7206	-0.0379392

Ciclev10001907m.g	scaffold_5:38371610-38374396	14.4258	14.052	-0.0378828
Ciclev10032914m.g	scaffold_4:20616989-20618817	10.7462	10.4695	-0.0376374
Ciclev10015044m.g	scaffold_2:7360379-7367946	23.0641	22.4704	-0.0376212
Ciclev10029602m.g	scaffold_8:22292380-22293191	4.56206	4.445	-0.0375049
Ciclev10002455m.g	scaffold_5:24479555-24480973	90.081	87.7783	-0.037359
Ciclev10007057m.g	scaffold_9:30055313-30057157	133.808	130.401	-0.0372128
Ciclev10010817m.g	scaffold_1:23737305-23740299	5.48327	5.34413	-0.0370796
Ciclev10007362m.g	scaffold_1:14626717-14635291	5.02831	4.90074	-0.0370737
Ciclev10023848m.g	scaffold_3:44612672-44619838	20.2133	19.7008	-0.037054
Ciclev10022497m.g	scaffold_3:8267997-8269714	93.1938	90.8332	-0.0370135
Ciclev10025466m.g	scaffold_7:1809501-1813953	26.4635	25.7949	-0.0369162
Ciclev10014215m.g,Ciclev10017528m.g	scaffold_2:24626907-24636629	15.5321	15.1398	-0.0369015
Ciclev10006590m.g	scaffold_9:2078437-2078746	30.0282	29.2728	-0.0367598
Ciclev10013063m.g	scaffold_6:17748791-17752145	37.4934	36.5536	-0.0366256
Ciclev10002295m.g,Ciclev10003508m.g	scaffold_5:20932287-20940811	13.6307	13.2891	-0.036611
Ciclev10001436m.g	scaffold_5:19117554-19119789	21.216	20.6848	-0.0365775
Ciclev10025273m.g	scaffold_7:9341137-9343135	22.3212	21.7628	-0.036546
Ciclev10020215m.g	scaffold_3:41785800-41791121	33.6426	32.8012	-0.0365389
Ciclev10009204m.g	scaffold_1:8861088-8863904	20.9599	20.4371	-0.0364433
Ciclev10010071m.g	scaffold_1:7896109-7896650	216.16	210.794	-0.0362708
Ciclev10000735m.g	scaffold_5:25965962-25969199	1.61779	1.57776	-0.0361523
Ciclev10015402m.g	scaffold_2:28946266-28953481	33.5253	32.6963	-0.0361213
Ciclev10019111m.g	scaffold_3:1852219-1856865	3.87068	3.77539	-0.0359639
Ciclev10030687m.g	scaffold_4:3468082-3477274	12.3703	12.0673	-0.0357851
Ciclev10000424m.g	scaffold_5:39451877-39457800	4.5137	4.40354	-0.0356487
Ciclev10033093m.g	scaffold_4:23871592-23873478	222.978	217.551	-0.0355479
Ciclev10031881m.g	scaffold_4:25418409-25420648	5.20302	5.0764	-0.035544
Ciclev10009140m.g	scaffold_1:979693-982136	19.0038	18.5439	-0.0353422
Ciclev10015475m.g	scaffold_2:8071298-8076757	4.48328	4.37482	-0.0353297
Ciclev10028531m.g	scaffold_8:18155480-18157658	129.926	126.783	-0.0353289
Ciclev10028538m.g	scaffold_8:24950088-24953454	10.3321	10.0825	-0.0352831
Ciclev10021392m.g	scaffold_3:3006217-3008357	15.0908	14.7266	-0.0352408
Ciclev10030722m.g	scaffold_4:25481927-25485379	6.38337	6.22946	-0.0352117
Ciclev10012365m.g	scaffold_6:15146106-15150144	11.7725	11.4888	-0.0351952
Ciclev10032020m.g	scaffold_4:20367622-20370641	26.7504	26.1071	-0.0351202
Ciclev10028484m.g	scaffold_8:4436157-4440875	50.5161	49.3065	-0.0349648
Ciclev10004964m.g	scaffold_9:16701748-16703907	15.8334	15.4554	-0.034865
Ciclev10031799m.g	scaffold_4:1969589-1972842	106.312	103.799	-0.0345165
Ciclev10008120m.g	scaffold_1:18058467-18065853	11.2978	11.0312	-0.0344537
Ciclev10022130m.g	scaffold_3:1998920-2001278	415.901	406.117	-0.0343423
Ciclev10026167m.g	scaffold_7:18948566-18954524	9.64447	9.41803	-0.0342755
Ciclev10022693m.g	scaffold_3:5657540-5659830	23.0542	22.5135	-0.0342385
Ciclev10011979m.g	scaffold_6:18167217-18172180	42.2382	41.2563	-0.0339337
Ciclev10026398m.g	scaffold_7:1985642-1988236	20.1254	19.6581	-0.0338975
Ciclev10030714m.g	scaffold_4:3341907-3344935	1.27178	1.24246	-0.0336458
Ciclev10015080m.g	scaffold_2:34277348-34279235	31.027	30.3152	-0.0334815
Ciclev10004414m.g	scaffold_9:29114071-29122126	14.5035	14.1714	-0.0334157
Ciclev10020085m.g	scaffold_3:3441807-3446926	19.6677	19.218	-0.0333739
Ciclev10005110m.g	scaffold_9:13210206-13217706	7.12655	6.96393	-0.0333041
Ciclev10019086m.g	scaffold_3:7562027-7568949	6.07216	5.93422	-0.0331526
Ciclev10016661m.g	scaffold_2:29546438-29548318	39.8476	38.9424	-0.0331522
Ciclev10028542m.g,Ciclev10029502m.g	scaffold_8:2191754-2204285	15.6185	15.2694	-0.0326134
Ciclev10016016m.g	scaffold_2:34195932-34199187	22.3684	21.8687	-0.0325941

Ciclev10022154m.g	scaffold_3:29118903-29120612	18.9752	18.5515	-0.0325828
Ciclev10030635m.g	scaffold_4:25465894-25472365	19.146	18.7189	-0.0325452
Ciclev10020388m.g	scaffold_3:11660537-11664557	31.3995	30.7	-0.0325022
Ciclev10001639m.g	scaffold_5:38186494-38189379	62.2121	60.8298	-0.032418
Ciclev10011288m.g	scaffold_6:1035592-1038854	1.40427	1.37307	-0.0324122
Ciclev10025916m.g	scaffold_7:2862323-2866491	45.8996	44.8811	-0.0323743
Ciclev10023453m.g,Ciclev10024195m.g,Ciclev10024492m.g	scaffold_3:47009613-47016148	232.477	227.331	-0.0322926
Ciclev10011646m.g	scaffold_6:21667073-21673152	7.59486	7.42722	-0.0322014
Ciclev10030974m.g	scaffold_4:3540086-3543688	10.2914	10.0662	-0.0319182
Ciclev10031531m.g	scaffold_4:17962048-17968024	65.3419	63.9278	-0.0315636
Ciclev10003108m.g	scaffold_5:24277796-24282311	8.29087	8.11272	-0.0313382
Ciclev10014197m.g	scaffold_2:7048186-7051168	2.14624	2.10024	-0.0312605
Ciclev10025451m.g	scaffold_7:2084849-2088443	0.533979	0.52254	-0.0312429
Ciclev10007977m.g	scaffold_1:20176017-20179837	8.53934	8.3566	-0.0312077
-	scaffold_5:41565263-41567310	4.25235	4.1614	-0.0311917
Ciclev10006402m.g	scaffold_9:22671553-22687084	59.3478	58.0865	-0.0309895
Ciclev10002484m.g	scaffold_5:37866132-37869674	151.568	148.35	-0.030959
Ciclev10004606m.g	scaffold_9:2659243-2668343	23.8248	23.3191	-0.0309521
Ciclev10007418m.g	scaffold_1:24418892-24426157	7.60961	7.44931	-0.0307156
Ciclev10004645m.g	scaffold_9:1021127-1024583	44.5336	43.596	-0.0306997
Ciclev10019223m.g	scaffold_3:1858383-1861522	3.38377	3.31267	-0.0306363
Ciclev10019913m.g	scaffold_3:48747724-48751327	13.3864	13.1072	-0.0304092
Ciclev10018636m.g	scaffold_3:41566337-41574014	14.6424	14.3382	-0.0302821
Ciclev10002147m.g	scaffold_5:41925903-41928598	24.8888	24.3719	-0.0302745
Ciclev10021733m.g	scaffold_3:50175402-50177122	0.551477	0.540064	-0.0301722
Ciclev10000005m.g	scaffold_5:37817189-37832291	7.46528	7.31079	-0.0301684
Ciclev10012088m.g	scaffold_6:2067585-2073007	18.2032	17.8267	-0.0301504
Ciclev10025388m.g	scaffold_7:15526234-15537832	14.6991	14.3953	-0.0301321
Ciclev10016120m.g	scaffold_2:10978675-10982595	20.524	20.1041	-0.0298265
Ciclev10002374m.g	scaffold_5:40559956-40563236	22.2655	21.8105	-0.0297881
Ciclev10011749m.g	scaffold_6:19905963-19910394	12.3007	12.0496	-0.0297547
Ciclev10016293m.g	scaffold_2:30041291-30043021	51.2884	50.2438	-0.0296866
Ciclev10029027m.g	scaffold_8:2685268-2687978	50.1478	49.1375	-0.0293627
Ciclev10011185m.g	scaffold_6:8354953-8357167	2.51465	2.46403	-0.0293383
Ciclev10025788m.g	scaffold_7:3873187-3876358	14.8716	14.5738	-0.0291894
Ciclev10023083m.g	scaffold_3:45613682-45616014	117.555	115.203	-0.0291504
Ciclev10007357m.g	scaffold_1:924502-936397	21.9833	21.5442	-0.0291066
Ciclev10011748m.g	scaffold_6:18029680-18035774	23.8819	23.405	-0.0290986
-	scaffold_3:10595178-10595968	333.252	326.612	-0.0290363
Ciclev10015943m.g	scaffold_2:29335744-29338334	33.1141	32.4581	-0.0288644
Ciclev10011808m.g	scaffold_6:19252438-19260599	7.98006	7.82212	-0.0288399
Ciclev10025620m.g	scaffold_7:489315-492882	82.3352	80.7064	-0.0288267
Ciclev10017123m.g	scaffold_2:5391266-5393788	56.8946	55.7717	-0.0287606
Ciclev10030519m.g	scaffold_4:2987159-2996122	11.8492	11.6175	-0.0284894
Ciclev10015497m.g	scaffold_2:9849132-9852098	26.4041	25.8924	-0.0282339
Ciclev10014085m.g	scaffold_2:29432676-29444736	9.25283	9.07375	-0.0281962
Ciclev10014336m.g	scaffold_2:35122860-35130519	3.70065	3.62915	-0.0281468
Ciclev10001154m.g	scaffold_5:6748936-6753178	28.2919	27.746	-0.0281086
-	scaffold_3:4730086-4730338	45.4771	44.6139	-0.0276469
Ciclev10031384m.g	scaffold_4:766585-768993	7.35706	7.21751	-0.0276278
Ciclev10005577m.g	scaffold_9:7443086-7446177	23.3541	22.9135	-0.0274783
Ciclev10030523m.g	scaffold_4:7960462-7968303	12.7323	12.4924	-0.0274472
Ciclev10018757m.g	scaffold_3:48358057-48362592	18.2233	17.8802	-0.0274237
Ciclev10028702m.g	scaffold_8:18358288-18362569	16.095	15.7927	-0.0273523

Ciclev10008788m.g	scaffold_1:20553920-20558851	34.732	34.0809	-0.0273023
Ciclev10017466m.g	scaffold_2:27579626-27581411	17.2624	16.9393	-0.0272588
Ciclev10020618m.g	scaffold_3:46267038-46271127	44.8811	44.0483	-0.0270223
Ciclev10019089m.g	scaffold_3:8200430-8204846	22.7988	22.3758	-0.0270189
Ciclev10026642m.g	scaffold_7:656048-657475	109.985	107.949	-0.0269517
Ciclev10033053m.g	scaffold_4:6228379-6230405	17.094	16.7797	-0.0267719
Ciclev10018659m.g	scaffold_3:33388539-33592383	130.686	128.287	-0.026735
Ciclev10013189m.g	scaffold_6:20521698-20523527	177.265	174.012	-0.0267179
Ciclev10008941m.g	scaffold_1:10358964-10362816	29.378	28.8431	-0.0265087
Ciclev10012163m.g	scaffold_6:22719759-22722958	40.7736	40.0313	-0.0265062
Ciclev10003160m.g	scaffold_5:4076819-4284428	23.7153	23.2846	-0.026447
Ciclev10022502m.g	scaffold_3:6149885-6151789	13.1536	12.9152	-0.0263867
Ciclev10022385m.g	scaffold_3:42133240-42136087	17.7634	17.4421	-0.0263378
Ciclev10015844m.g	scaffold_2:11530118-11531262	5.28482	5.18929	-0.0263191
Ciclev10000408m.g	scaffold_5:2209329-2219786	11.0073	10.8084	-0.0263061
Ciclev10006264m.g	scaffold_9:814206-816324	47.0494	46.2017	-0.0262307
-	scaffold_7:19668426-19671013	190.543	187.121	-0.0261466
Ciclev10024112m.g	scaffold_3:46657920-46662192	13.4383	13.1982	-0.026003
Ciclev10027694m.g	scaffold_8:17590328-17600418	8.49442	8.34366	-0.0258366
Ciclev10031886m.g	scaffold_4:21243084-21244983	3680.92	3615.65	-0.0258097
Ciclev10005873m.g	scaffold_9:13572778-13575382	58.7977	57.758	-0.0257402
Ciclev10005299m.g	scaffold_9:1728859-1731673	12.1706	11.9559	-0.0256858
Ciclev10004190m.g	scaffold_9:7183563-7200161	16.0957	15.8122	-0.025633
Ciclev10032119m.g	scaffold_4:2743036-2744965	7.71239	7.57687	-0.0255755
-	scaffold_2:24362971-24363916	134.294	131.935	-0.0255662
Ciclev10021817m.g	scaffold_3:40225243-40228815	23.657	23.2427	-0.0254902
Ciclev10026635m.g	scaffold_7:3159263-3162290	28.5122	28.013	-0.0254805
Ciclev10000007m.g	scaffold_5:36660140-36674263	17.1723	16.8719	-0.025461
Ciclev10025224m.g	scaffold_7:5793094-5800482	52.1554	51.2442	-0.0254279
Ciclev10033653m.g	scaffold_4:11790187-11794396	28.5564	28.059	-0.0253495
Ciclev10031739m.g	scaffold_4:15048831-15052186	264.787	260.199	-0.0252152
Ciclev10030561m.g	scaffold_4:21822014-21827299	2.25987	2.22073	-0.0252108
Ciclev10021343m.g	scaffold_3:30005526-30010915	47.2727	46.457	-0.0251113
Ciclev10015333m.g	scaffold_2:12047114-12049476	16.2839	16.004	-0.0250092
Ciclev10020853m.g	scaffold_3:15663819-15673949	102.92	101.154	-0.0249702
Ciclev10012682m.g	scaffold_6:7561834-7666028	2.90649	2.85666	-0.0249491
Ciclev10004533m.g	scaffold_9:11993281-11999324	56.8395	55.8688	-0.0248515
Ciclev10022889m.g	scaffold_3:1244332-1245462	4.02705	3.95838	-0.0248147
Ciclev10028653m.g	scaffold_8:14617867-14620529	5.12194	5.03482	-0.0247496
Ciclev10032832m.g	scaffold_4:23653135-23655814	69.5173	68.3357	-0.0247332
Ciclev10020152m.g	scaffold_3:42374070-42379148	7.01289	6.89373	-0.0247235
Ciclev10008923m.g	scaffold_1:26338834-26346373	51.7181	50.8413	-0.0246687
Ciclev10007544m.g	scaffold_1:7284482-7293746	7.37251	7.24768	-0.0246368
Ciclev10012718m.g	scaffold_6:24205309-24207694	35.7023	35.0997	-0.0245612
Ciclev10008429m.g	scaffold_1:27650520-27657546	6.21835	6.11389	-0.0244399
Ciclev10000128m.g	scaffold_5:21203965-21212595	17.2524	16.9629	-0.0244093
Ciclev10006143m.g	scaffold_9:5287261-5290208	9.40137	9.24384	-0.0243788
Ciclev10009129m.g	scaffold_1:4635111-4638292	11.3071	11.118	-0.0243439
Ciclev10031492m.g	scaffold_4:24401840-24406470	19.5974	19.2697	-0.0243253
Ciclev10008417m.g	scaffold_1:444025-446732	11.2649	11.0776	-0.0241877
Ciclev10000858m.g	scaffold_5:18221784-18227885	7.04627	6.92989	-0.0240274
Ciclev10009149m.g	scaffold_1:18691170-18695249	84.2464	82.8593	-0.0239509
Ciclev10026076m.g	scaffold_7:8353000-8356326	18.5819	18.276	-0.0239504
Ciclev10031289m.g	scaffold_4:17713727-17718184	158.396	155.8	-0.0238375
Ciclev10033310m.g	scaffold_4:19001055-19040863	17.0135	16.7349	-0.023821
Ciclev10021673m.g	scaffold_3:47908877-47912289	75.2178	73.9906	-0.0237317

Ciclev10030836m.g	scaffold_4:16770530-16776259	25.1358	24.7273	-0.0236404
Ciclev10020553m.g,Ciclev10024062m.g,Ciclev10024495m.g	scaffold_3:39181844-39300715	11.2928	11.1093	-0.0236331
Ciclev10001021m.g	scaffold_5:32967396-32970116	12.3168	12.1199	-0.0232572
Ciclev10016286m.g	scaffold_2:34918216-34919258	3745.45	3685.6	-0.02324
Ciclev10023851m.g,Ciclev10024185m.g	scaffold_3:24026647-24169357	4.73307	4.65751	-0.0232186
Ciclev10001804m.g	scaffold_5:40290003-40294279	13.2809	13.0692	-0.0231866
Ciclev10016301m.g	scaffold_2:22742606-22744406	17.1123	16.8421	-0.022958
Ciclev10022387m.g	scaffold_3:49274178-49279167	4.09275	4.02816	-0.0229505
Ciclev10011108m.g	scaffold_6:11932383-11940795	80.4476	79.1823	-0.0228712
Ciclev10012334m.g	scaffold_6:22462533-22465498	56.5929	55.708	-0.0227364
Ciclev10016651m.g	scaffold_2:18791204-18794712	16.7485	16.4882	-0.0225955
Ciclev10001130m.g	scaffold_5:2455680-2457243	1.05515	1.03879	-0.0225436
Ciclev10008131m.g,Ciclev10008136m.g	scaffold_1:26110929-26124080	31.9456	31.4516	-0.0224832
Ciclev10016887m.g	scaffold_2:33187287-33189692	73.2575	72.128	-0.0224174
Ciclev10016720m.g	scaffold_2:13486891-13490006	63.5857	62.6101	-0.0223074
-	scaffold_2:428135-428529	9.29886	9.15687	-0.0221995
Ciclev10027715m.g	scaffold_8:2839325-2845836	30.6684	30.2021	-0.0221
Ciclev10000108m.g	scaffold_5:38626628-38638741	25.4591	25.0763	-0.0218612
Ciclev10025314m.g	scaffold_7:6785793-6793006	93.9065	92.5062	-0.0216748
Ciclev10008264m.g	scaffold_1:8411316-8412895	6.34407	6.24992	-0.0215703
Ciclev10005905m.g	scaffold_9:9766636-9771190	23.8837	23.5323	-0.021387
Ciclev10009238m.g	scaffold_1:3397422-3400719	1.66645	1.64195	-0.0213673
Ciclev10007871m.g	scaffold_1:21348357-21355352	10.172	10.0236	-0.0211987
Ciclev10027762m.g	scaffold_8:18024114-18039719	7.25262	7.14859	-0.0208436
Ciclev10012305m.g	scaffold_6:19093123-19096738	26.6456	26.2669	-0.0206495
Ciclev10017081m.g	scaffold_2:2992085-2993407	13.7358	13.5409	-0.0206238
Ciclev10000312m.g	scaffold_5:25969556-25975972	1.49821	1.47707	-0.0205066
Ciclev10005677m.g	scaffold_9:28396494-28399986	5.25336	5.17926	-0.0204949
Ciclev10007199m.g	scaffold_9:26496533-26497558	1.89321	1.86654	-0.0204683
Ciclev10018486m.g	scaffold_3:5585141-5593506	18.1141	17.8592	-0.0204477
Ciclev10001764m.g	scaffold_5:33178027-33182890	17.6229	17.375	-0.0204378
Ciclev10016681m.g	scaffold_2:3046637-3053472	19.7198	19.4439	-0.020332
Ciclev10007265m.g	scaffold_1:11099016-11111698	12.2606	12.0896	-0.0202662
Ciclev10032844m.g	scaffold_4:3663002-3667478	21.6934	21.3974	-0.0198236
Ciclev10026055m.g	scaffold_7:3525894-3529905	6.1191	6.03625	-0.0196657
Ciclev10014960m.g	scaffold_2:83182-92491	3.29935	3.25502	-0.0195156
Ciclev10002686m.g	scaffold_5:40185521-40188191	65.7433	64.8677	-0.0193451
Ciclev10004135m.g	scaffold_9:2493509-2503391	6.01298	5.93364	-0.0191625
Ciclev10018901m.g	scaffold_3:5038746-5181239	1.86622	1.84167	-0.019105
Ciclev10012396m.g	scaffold_6:15458264-15462034	19.087	18.8375	-0.018986
Ciclev10007535m.g	scaffold_1:27204997-27211059	46.427	45.828	-0.0187346
Ciclev10009205m.g	scaffold_1:24591597-24593689	79.6054	78.5792	-0.0187188
Ciclev10004189m.g	scaffold_9:4149888-4158331	12.2296	12.0735	-0.0185338
Ciclev10026016m.g,Ciclev10026797m.g	scaffold_7:8564201-8570178	32.4777	32.0666	-0.0183818
Ciclev10018545m.g	scaffold_3:33990063-33999397	2.91903	2.88224	-0.0182992
Ciclev10016094m.g	scaffold_2:29811672-29813258	10.2742	10.1448	-0.0182924
Ciclev10030962m.g	scaffold_4:6607574-6611460	25.658	25.3361	-0.0182116
Ciclev10008133m.g	scaffold_1:24263263-24271176	46.0386	45.4632	-0.0181457
Ciclev10032428m.g	scaffold_4:15087930-15266082	19.0541	18.8181	-0.0179793
Ciclev10032980m.g	scaffold_4:2976872-2978309	324.628	320.608	-0.0179772
Ciclev10005707m.g	scaffold_9:5310512-5314498	24.5091	24.207	-0.0178976

Ciclev10026547m.g	scaffold_7:240863-250983	14.4722	14.2951	-0.0177597
Ciclev10007846m.g	scaffold_1:24707531-24715399	76.5803	75.6461	-0.0177068
Ciclev10007912m.g	scaffold_1:26729765-26744426	12.5138	12.3614	-0.0176718
Ciclev10028552m.g,Ciclev10029974m.g	scaffold_8:9624097-9646817	23.6102	23.3231	-0.0176493
Ciclev10002605m.g	scaffold_5:35782418-35784765	89.4808	88.3947	-0.0176172
Ciclev10024841m.g	scaffold_7:14123946-14154295	4.32926	4.27697	-0.017531
Ciclev10000878m.g	scaffold_5:35840434-35842867	9.28447	9.17284	-0.0174512
Ciclev10019247m.g	scaffold_3:49712033-49714879	15.5791	15.3931	-0.0173288
Ciclev10021882m.g	scaffold_3:34642074-34646039	58.1419	57.4501	-0.0172687
Ciclev10004566m.g	scaffold_9:6343992-6348631	1.17436	1.16042	-0.0172344
Ciclev10009828m.g	scaffold_1:17550424-17551647	14.1224	13.9556	-0.017142
Ciclev10015964m.g	scaffold_2:27089070-27093047	2.91264	2.87839	-0.0170621
Ciclev10014087m.g	scaffold_2:34796752-34803516	2.24004	2.21389	-0.0169436
Ciclev10023714m.g	scaffold_3:44165477-44167561	5.68867	5.62227	-0.0169384
Ciclev10031552m.g,Ciclev10033615m.g	scaffold_4:11990662-12009743	48.0194	47.4602	-0.0168976
Ciclev10016206m.g	scaffold_2:22471207-22474774	236.679	233.955	-0.0167023
Ciclev10001613m.g	scaffold_5:24794458-24798226	5.41579	5.35349	-0.0166916
Ciclev10011043m.g	scaffold_6:11980340-11987654	21.5739	21.3259	-0.0166825
Ciclev10011229m.g	scaffold_6:23956142-23961518	2.70381	2.67276	-0.0166618
Ciclev10001298m.g	scaffold_5:33980142-33983575	144.964	143.302	-0.0166418
Ciclev10033133m.g,Ciclev10033274m.g	scaffold_4:20422139-20426654	21.8871	21.6385	-0.0164845
Ciclev10012803m.g	scaffold_6:21654951-21657001	153.827	152.08	-0.0164834
Ciclev10030282m.g	scaffold_8:8703759-8715211	11.6815	11.549	-0.0164624
Ciclev10008892m.g	scaffold_1:23365318-23368311	24.564	24.2853	-0.0164609
Ciclev10005632m.g,Ciclev10007114m.g	scaffold_9:17778241-17793258	142.312	140.701	-0.0164235
Ciclev10007292m.g	scaffold_1:26387637-26395168	12.9108	12.7685	-0.0159921
Ciclev10000482m.g	scaffold_5:32912953-32917197	0.390449	0.386162	-0.0159293
Ciclev10002196m.g	scaffold_5:32934959-32937201	50.2865	49.7407	-0.015746
Ciclev10004553m.g	scaffold_9:4510268-4520385	4.80345	4.75206	-0.0155176
Ciclev10000313m.g	scaffold_5:37043416-37051080	35.7728	35.3947	-0.0153306
Ciclev10032706m.g	scaffold_4:16330131-16331403	118.862	117.608	-0.0153007
Ciclev10017921m.g	scaffold_2:32038654-32040555	5.14175	5.08793	-0.0151806
Ciclev10028807m.g	scaffold_8:23448602-23450502	33.4701	33.121	-0.0151235
Ciclev10002808m.g	scaffold_5:37392780-37397462	8.42351	8.33586	-0.0150895
Ciclev10006301m.g	scaffold_9:6755090-6757926	9.53358	9.43524	-0.0149579
Ciclev10015345m.g	scaffold_2:14009702-14016024	55.1961	54.6289	-0.0149014
Ciclev10026781m.g	scaffold_7:1608633-1610659	566.884	561.09	-0.0148206
-	scaffold_3:33372179-33379535	4.54344	4.49713	-0.0147811
Ciclev10002369m.g	scaffold_5:41077761-41081130	41.5698	41.1508	-0.0146162
Ciclev10019513m.g	scaffold_3:3227470-3232235	17.1444	16.9744	-0.0143813
Ciclev10028554m.g	scaffold_8:22327965-22332095	4.00907	3.96937	-0.0143569
Ciclev10028453m.g	scaffold_8:20156680-20160637	18.4169	18.2354	-0.0142924
Ciclev10002565m.g	scaffold_5:39881995-39885588	86.6698	85.8232	-0.0141628
Ciclev10016236m.g	scaffold_2:32507259-32509012	3.41077	3.37753	-0.0141272
Ciclev10007610m.g	scaffold_1:3236336-3242326	9.11354	9.02607	-0.0139129
Ciclev10010046m.g	scaffold_1:27214098-27217053	217.659	215.591	-0.0137733
Ciclev10009512m.g	scaffold_1:25721038-25722484	25.5537	25.3121	-0.0137034
Ciclev10008857m.g	scaffold_1:6452791-6455084	87.5947	86.7669	-0.0137
Ciclev10000505m.g	scaffold_5:9223467-9232859	17.2716	17.1143	-0.0132037
Ciclev10014628m.g	scaffold_2:10040101-10043977	13.9619	13.8351	-0.013171
Ciclev10002394m.g	scaffold_5:15991107-15996585	27.7676	27.5196	-0.0129416
Ciclev10019098m.g	scaffold_3:12462320-12467645	4.96242	4.91811	-0.0129403

Ciclev10016497m.g	scaffold_2:25968067-25970211	11.6139	11.5103	-0.0129305
Ciclev10014025m.g	scaffold_2:8870126-8882246	6.42172	6.36455	-0.012903
Ciclev10027265m.g	scaffold_7:7234014-7235030	1.50859	1.4952	-0.0128573
Ciclev10015245m.g	scaffold_2:10058915-10097079	22.0975	21.9023	-0.0127986
Ciclev10019441m.g	scaffold_3:4149225-4153173	4.85132	4.80853	-0.0127813
Ciclev10020350m.g	scaffold_3:2705865-2710019	35.043	34.7357	-0.0127084
Ciclev10005393m.g	scaffold_9:3260202-3265806	11.779	11.6757	-0.012701
Ciclev10015322m.g	scaffold_2:36351031-36353884	18.7437	18.5798	-0.0126714
Ciclev10013467m.g	scaffold_6:23430033-23436552	48.96	48.5324	-0.0126566
Ciclev10020094m.g	scaffold_3:42935982-42941151	20.9691	20.786	-0.0126487
-	scaffold_3:1360950-1362206	20.5547	20.3763	-0.0125726
Ciclev10003355m.g	scaffold_5:21016539-21021020	2.98145	2.95586	-0.0124359
Ciclev10021861m.g	scaffold_3:7327403-7335639	1982.5	1965.53	-0.0124026
Ciclev10017543m.g	scaffold_2:36066568-36067618	4.67558	4.63572	-0.0123516
Ciclev10029927m.g	scaffold_8:3750665-3751492	26.9872	26.7586	-0.0122699
Ciclev10008541m.g	scaffold_1:332443-339146	27.1946	26.966	-0.012177
Ciclev10026749m.g	scaffold_7:1530712-1532413	63.6948	63.1598	-0.0121687
Ciclev10000168m.g	scaffold_5:30641621-30651133	16.1528	16.0182	-0.0120768
Ciclev10017283m.g	scaffold_2:23012305-23018997	355.044	352.087	-0.0120636
Ciclev10009450m.g	scaffold_1:1143011-1145502	73.2492	72.6429	-0.0119912
Ciclev10012597m.g	scaffold_6:25389382-25391779	22.9078	22.721	-0.0118151
Ciclev10017171m.g	scaffold_2:33660010-33663665	40.7772	40.4448	-0.0118073
Ciclev10000070m.g	scaffold_5:33241335-33246175	18.9414	18.7893	-0.0116325
Ciclev10001747m.g	scaffold_5:33861383-33864591	11.2134	11.1239	-0.0115698
-	scaffold_2:15086361-15087217	6.43687	6.38676	-0.0112746
Ciclev10028829m.g	scaffold_8:23329789-23331440	9.80815	9.73213	-0.0112252
Ciclev10025627m.g	scaffold_7:13734330-13738212	11.6585	11.5684	-0.0111904
Ciclev10009392m.g	scaffold_1:28567167-28570492	24.4608	24.2729	-0.011126
Ciclev10021320m.g	scaffold_3:7762092-7765661	18.704	18.5604	-0.0111198
Ciclev10014206m.g	scaffold_2:4499413-4511556	16.6217	16.4957	-0.0109763
Ciclev10008135m.g	scaffold_1:28433870-28437921	21.2925	21.1336	-0.0108067
Ciclev10016191m.g	scaffold_2:1818639-1824507	40.3209	40.0212	-0.010766
Ciclev10000632m.g	scaffold_5:34207131-34210961	7.16013	7.10759	-0.0106252
Ciclev10014183m.g	scaffold_2:31350792-31356125	8.75416	8.6905	-0.0105287
Ciclev10005444m.g	scaffold_9:4065406-4069591	14.1073	14.0051	-0.0104916
Ciclev10010775m.g	scaffold_1:1648643-1651133	0.368974	0.366307	-0.0104674
Ciclev10025851m.g	scaffold_7:10806594-10809165	19.1094	18.9725	-0.0103754
Ciclev10018705m.g	scaffold_3:33967765-33971051	5.12878	5.09238	-0.0102773
Ciclev10004667m.g	scaffold_9:21756015-21760483	14.1967	14.097	-0.0101645
Ciclev10016025m.g	scaffold_2:32998559-33003916	75.582	75.0688	-0.00982953
Ciclev10019625m.g	scaffold_3:2433229-2437314	39.3329	39.0685	-0.0097298
Ciclev10028454m.g	scaffold_8:3494003-3498365	81.1359	80.5999	-0.00956134
Ciclev10025731m.g	scaffold_7:4166461-4171233	58.1623	57.7827	-0.00944557
Ciclev10001596m.g	scaffold_5:27294152-27299210	28.827	28.6395	-0.00941362
Ciclev10016113m.g,Ciclev10018008m.g	scaffold_2:2938111-2944880	33.9322	33.7118	-0.00939901
Ciclev10009369m.g	scaffold_1:24837318-24840138	53.1093	52.7688	-0.00927844
Ciclev10028703m.g	scaffold_8:3665797-3669771	95.8338	95.2349	-0.00904427
Ciclev10032120m.g	scaffold_4:21606882-21611853	25.1412	24.9893	-0.00874339
Ciclev10018330m.g	scaffold_2:2014613-2020294	19.7415	19.6247	-0.00856052
Ciclev10009507m.g	scaffold_1:15749957-15751468	148.572	147.699	-0.00850716
Ciclev10029485m.g	scaffold_8:18020029-18023292	58.2024	57.8652	-0.00838229
Ciclev10005892m.g	scaffold_9:3448719-3449768	1128.18	1121.65	-0.00837821
Ciclev10025395m.g	scaffold_7:15170987-15174087	34.676	34.4788	-0.00822801
Ciclev10004769m.g	scaffold_9:26985083-26993527	7.61811	7.57537	-0.00811664
Ciclev10005753m.g	scaffold_9:28542274-28543641	142.297	141.505	-0.00805103

Ciclev10005062m.g,Ciclev10007203m.g	scaffold_9:16383850-16393557	31.6987	31.5238	-0.00797859
Ciclev10010942m.g	scaffold_6:23256040-23279093	6.81512	6.77826	-0.00782438
Ciclev10031045m.g	scaffold_4:6128951-6138477	29.5924	29.4336	-0.00776227
Ciclev10027736m.g	scaffold_8:20549938-20558940	6.49853	6.46496	-0.00747237
Ciclev10022604m.g	scaffold_3:42536785-42539161	72.3124	71.9419	-0.0074106
-	scaffold_8:63462-66679	14.7253	14.6505	-0.00734309
Ciclev10008657m.g	scaffold_1:12277892-12282510	31.1644	31.0099	-0.00716704
Ciclev10022328m.g	scaffold_3:4446203-4448711	113.492	112.948	-0.00693206
Ciclev10014889m.g	scaffold_2:34533345-34536438	14.7998	14.7293	-0.00689178
Ciclev10016469m.g	scaffold_2:33369933-33373279	28.6499	28.5194	-0.00658514
Ciclev10029287m.g	scaffold_8:2622956-2627138	22.7503	22.6475	-0.00653777
Ciclev10021015m.g	scaffold_3:11012406-11013967	8.49848	8.46047	-0.00646557
Ciclev10012871m.g	scaffold_6:23246512-23247603	23.1601	23.0583	-0.00635864
Ciclev10010972m.g	scaffold_6:19142306-19156510	18.4796	18.3991	-0.00629789
Ciclev10029379m.g	scaffold_8:21919214-21923184	34.9425	34.7944	-0.00612944
Ciclev10014352m.g	scaffold_2:5631548-5635227	0.276324	0.275169	-0.00603922
Ciclev10032794m.g	scaffold_4:20942461-20943383	25.6633	25.5581	-0.00592619
Ciclev10029823m.g	scaffold_8:20181157-20187216	25.9346	25.8296	-0.00584959
Ciclev10019060m.g	scaffold_3:3184717-3190341	17.1318	17.0634	-0.00577029
Ciclev10014876m.g	scaffold_2:28006349-28008046	2.82601	2.81474	-0.0057678
Ciclev10030700m.g	scaffold_4:25436276-25440925	14.5231	14.4659	-0.00569419
Ciclev10015524m.g	scaffold_2:11799005-11831046	3.77323	3.75862	-0.00559876
Ciclev10025790m.g	scaffold_7:7849714-7852581	30.0511	29.9352	-0.00557737
Ciclev10016002m.g	scaffold_2:31981937-31984803	21.3921	21.3101	-0.00554546
Ciclev10005973m.g	scaffold_9:16060458-16062639	31.3083	31.1893	-0.00549557
Ciclev10009877m.g	scaffold_1:8008508-8010469	225.561	224.712	-0.00544059
Ciclev10033810m.g	scaffold_4:6473970-6475080	5.01389	4.99546	-0.00531327
Ciclev10032098m.g	scaffold_4:914305-917467	58.0682	57.8602	-0.005177
Ciclev10009500m.g	scaffold_1:26105322-26108198	31.4303	31.3183	-0.00514877
Ciclev10003147m.g	scaffold_5:24926144-25131690	1.98533	1.9784	-0.00504578
Ciclev10002611m.g	scaffold_5:39204024-39206398	91.411	91.1044	-0.00484758
Ciclev10004424m.g	scaffold_9:19042248-19054031	32.1895	32.0872	-0.00459279
Ciclev10014794m.g	scaffold_2:23859015-23862998	66.2533	66.0434	-0.00457671
Ciclev10002732m.g	scaffold_5:27141621-27142692	3.12716	3.11809	-0.00418924
Ciclev10015025m.g	scaffold_2:21566356-21571128	5.91123	5.89446	-0.00409789
Ciclev10025334m.g	scaffold_7:7892486-7897544	54.7531	54.6004	-0.00402854
Ciclev10016071m.g	scaffold_2:3460369-3462174	8.92589	8.9025	-0.00378577
Ciclev10015976m.g	scaffold_2:33774600-33777726	8.24583	8.22434	-0.00376372
Ciclev10019321m.g	scaffold_3:41892790-41901735	12.5985	12.5673	-0.0035822
Ciclev10020317m.g	scaffold_3:7772091-7775118	6.26909	6.25382	-0.00351697
Ciclev10023760m.g	scaffold_3:3415760-3419914	7.50997	7.49187	-0.00348037
Ciclev10032982m.g	scaffold_4:8113418-8115629	20.0452	19.9971	-0.00346781
Ciclev10001297m.g	scaffold_5:41719098-41720940	20.356	20.3087	-0.00335468
Ciclev10000595m.g	scaffold_5:43060441-43066562	56.3551	56.2267	-0.0032891
Ciclev10009629m.g,Ciclev10010538m.g	scaffold_1:25098277-25105128	85.5915	85.3996	-0.00323898
Ciclev10027867m.g	scaffold_8:22877496-22880882	5.00071	4.99054	-0.00293589
Ciclev10007557m.g,Ciclev10007847m.g,Ciclev10010777m.g	scaffold_1:3472407-3502192	22.3765	22.3329	-0.00281601
Ciclev10013942m.g,Ciclev10013945m.g	scaffold_10:332735-345333	35.3253	35.2572	-0.00278455
Ciclev10020542m.g	scaffold_3:12254176-12259144	96.0938	95.9097	-0.00276664
Ciclev10014455m.g	scaffold_2:9739205-9752276	7.81524	7.80067	-0.00269194
Ciclev10007457m.g	scaffold_1:26952017-26957456	2.63062	2.62572	-0.00268867

Ciclev10000795m.g	scaffold_5:40683486-40688413	13.6302	13.6079	-0.00235892
Ciclev10001215m.g	scaffold_5:31084374-31089683	10.0276	10.0127	-0.00215032
Ciclev10021370m.g	scaffold_3:41703811-41708873	13.078	13.0587	-0.00213134
Ciclev10031378m.g	scaffold_4:816825-820866	48.2636	48.2007	-0.00188198
Ciclev10018566m.g	scaffold_3:9015706-9022288	36.6805	36.6342	-0.00181927
Ciclev10015609m.g	scaffold_2:31016607-31021566	15.4309	15.4118	-0.0017905
Ciclev10019771m.g	scaffold_3:44604897-44607136	11.6687	11.6547	-0.00173567
Ciclev10000875m.g	scaffold_5:35241679-35246264	12.4031	12.39	-0.00152431
Ciclev10007928m.g	scaffold_1:7141440-7146220	3.5166	3.51294	-0.00150545
Ciclev10000753m.g	scaffold_5:35913858-35917123	7.36306	7.35553	-0.00147618
Ciclev10019791m.g,Ciclev10022834m.g	scaffold_3:39845123-39854254	78.8956	78.8212	-0.00136254
Ciclev10016054m.g	scaffold_2:9902661-9906589	109.162	109.065	-0.0012827
Ciclev10002359m.g	scaffold_5:6440801-6444240	68.6765	68.6178	-0.00123555
Ciclev10012003m.g	scaffold_6:15293190-15296060	1.82405	1.82258	-0.00116211
Ciclev10001931m.g	scaffold_5:31091781-31094489	44.5581	44.5222	-0.00116112
Ciclev10012448m.g	scaffold_6:19654358-19658178	11.198	11.1892	-0.00113262
Ciclev10024687m.g	scaffold_7:7025546-7038216	5.42794	5.42395	-0.00106221
Ciclev10033055m.g	scaffold_4:24120686-24122263	36.7783	36.7544	-0.000936531
Ciclev10014338m.g	scaffold_2:11718517-11725069	35.8938	35.8706	-0.000934806
Ciclev10017240m.g	scaffold_2:31978482-31981260	2.17553	2.17461	-0.000609983
Ciclev10027772m.g	scaffold_8:3138528-3147253	0.882062	0.881841	-0.000360892
Ciclev10028924m.g	scaffold_8:7969909-7973993	184.88	184.85	-0.000235523
Ciclev10028957m.g	scaffold_8:23880258-23882545	55.0522	55.0448	-0.000192529
Ciclev10012944m.g	scaffold_6:7909208-7914618	84.1262	84.1183	-0.000134989
Ciclev10033765m.g	scaffold_4:22944705-22960987	6.43037	6.42996	-9.23E-05
Ciclev10005330m.g	scaffold_9:2545340-2550917	14.5149	14.5153	4.44E-05
Ciclev10018789m.g	scaffold_3:50317182-50327524	17.6297	17.6309	0.000100806
Ciclev10006008m.g	scaffold_9:4307053-4313440	18.1693	18.172	0.000214046
Ciclev10025436m.g	scaffold_7:3586916-3593143	7.8953	7.89663	0.000242733
Ciclev10021490m.g	scaffold_3:2244973-2248011	9.54754	9.54928	0.000262252
Ciclev10000006m.g	scaffold_5:13242991-13256147	7.58881	7.59048	0.000317901
Ciclev10004743m.g	scaffold_9:16416816-16418476	1.03185	1.03211	0.000363792
Ciclev10008784m.g	scaffold_1:3844451-3849882	13.1173	13.121	0.000400344
Ciclev10020251m.g	scaffold_3:29418916-29426392	47.9038	47.9189	0.000456522
Ciclev10013736m.g,Ciclev10013917m.g	scaffold_6:8887607-8891843	96.5786	96.6147	0.000539405
Ciclev10027793m.g	scaffold_8:20387728-20396163	25.491	25.5017	0.000604683
Ciclev10028127m.g	scaffold_8:23768051-23771639	75.5391	75.5737	0.000661359
Ciclev10019743m.g	scaffold_3:48625263-48629072	34.4503	34.4687	0.00076789
Ciclev10000949m.g	scaffold_5:37529107-37534944	116.097	116.159	0.000773062
Ciclev10018838m.g	scaffold_3:4400847-4407506	20.5233	20.5347	0.000800397
Ciclev10015871m.g	scaffold_2:10820097-10822432	65.9508	65.989	0.00083494
Ciclev10016930m.g	scaffold_2:4475828-4478168	114.927	115.005	0.000983077
Ciclev10012826m.g	scaffold_6:9323671-9330498	90.2168	90.2812	0.00102913
Ciclev10021909m.g	scaffold_3:43615422-43618371	60.1977	60.241	0.00103713
Ciclev10032201m.g	scaffold_4:3956723-3959799	24.442	24.4596	0.00103743
Ciclev10021838m.g	scaffold_3:4342979-4346001	22.1443	22.1603	0.00104202
Ciclev10020739m.g	scaffold_3:8167296-8171325	5.12221	5.12602	0.00107242
Ciclev10021624m.g	scaffold_3:46498626-46500232	13.6319	13.6422	0.00108699
Ciclev10011364m.g	scaffold_6:22203664-22208593	1.99103	1.99268	0.00119441
Ciclev10014106m.g	scaffold_2:31656509-31666206	33.7627	33.7959	0.0014148
Ciclev10003803m.g	scaffold_5:40575524-40578675	0.886768	0.8877	0.00151629
Ciclev10009669m.g	scaffold_1:4534160-4535260	3.96172	3.96605	0.00157359
Ciclev10026667m.g	scaffold_7:16559828-16562873	38.444	38.4893	0.00169946
Ciclev10025251m.g	scaffold_7:995484-1000767	33.2897	33.3291	0.00170694

Ciclev10014283m.g	scaffold_2:14649839-14656636	5.70071	5.70801	0.00184658
Ciclev10022485m.g	scaffold_3:40073873-40077841	20.9337	20.9641	0.00209283
Ciclev10010414m.g	scaffold_1:26364825-26370951	11.3774	11.3947	0.00218457
Ciclev10029736m.g	scaffold_8:6739650-6741385	11.5904	11.608	0.00218673
Ciclev10029559m.g	scaffold_8:2667021-2669684	9.5758	9.59191	0.0024261
Ciclev10008004m.g	scaffold_1:17472919-17478533	43.1752	43.2509	0.00252649
Ciclev10031573m.g	scaffold_4:25254909-25260255	15.502	15.5293	0.00253542
Ciclev10005207m.g	scaffold_9:29109608-29113056	30.821	30.8777	0.00265143
Ciclev10033184m.g	scaffold_4:20944981-20946486	8.4832	8.49926	0.00272762
Ciclev10001124m.g	scaffold_5:35664486-35669446	17.4108	17.4439	0.00273805
Ciclev10013411m.g	scaffold_6:7836924-7837242	125.676	125.917	0.0027714
Ciclev10006261m.g	scaffold_9:14353298-14357565	196.378	196.759	0.00279242
Ciclev10004186m.g	scaffold_9:2002593-2013219	9.66201	9.68151	0.00290834
Ciclev10033227m.g	scaffold_4:17384574-17386458	32.2884	32.3543	0.00294177
Ciclev10027661m.g	scaffold_8:1643862-1668932	34.3395	34.41	0.00295729
Ciclev10015959m.g	scaffold_2:24662157-24666956	24.9061	24.9574	0.00296648
Ciclev10020947m.g	scaffold_3:46313697-46317639	62.2935	62.4285	0.00312231
Ciclev10005160m.g	scaffold_9:3348522-3355090	47.5429	47.6463	0.00313379
Ciclev10008198m.g	scaffold_1:1949952-1952001	2.089	2.09361	0.00317748
Ciclev10002136m.g	scaffold_5:16081095-16094760	14.3896	14.4214	0.00318755
Ciclev10002916m.g	scaffold_5:37433507-37433971	3.89598	3.90528	0.0034406
Ciclev10021508m.g	scaffold_3:39631321-39637711	24.5388	24.5976	0.00345049
Ciclev10005483m.g	scaffold_9:29568968-29571900	20.7162	20.767	0.00352895
Ciclev10015336m.g	scaffold_2:31827818-31834730	22.7839	22.8406	0.00358953
Ciclev10014895m.g	scaffold_2:25355912-25361960	4.63401	4.64638	0.00384709
Ciclev10018883m.g	scaffold_3:2719885-2727822	3.25663	3.2656	0.00396811
Ciclev10015991m.g	scaffold_2:17555438-17559021	39.0085	39.1212	0.00416074
Ciclev10019626m.g	scaffold_3:5640368-5646423	19.7968	19.8548	0.00422326
Ciclev10033160m.g	scaffold_4:6093318-6096022	17.9868	18.0431	0.00451263
Ciclev10014310m.g	scaffold_2:35203212-35210387	46.2752	46.4203	0.00451709
Ciclev10013083m.g	scaffold_6:20329966-20331878	133.585	134.004	0.00451811
Ciclev10020318m.g	scaffold_3:30700356-30703880	17.5121	17.5688	0.00466194
Ciclev10022558m.g	scaffold_3:6409310-6411489	68.0699	68.2948	0.0047574
Ciclev10020286m.g	scaffold_3:24809537-24814289	64.1524	64.3689	0.00485999
Ciclev10031759m.g	scaffold_4:3145869-3150204	76.017	76.2752	0.00489249
Ciclev10028251m.g	scaffold_8:24689988-24692884	20.3669	20.4374	0.00498202
Ciclev10011929m.g	scaffold_6:7773984-7779814	7.77437	7.80126	0.00498278
Ciclev10009494m.g	scaffold_1:3738483-3742722	44.546	44.7006	0.00499893
Ciclev10000467m.g,Ciclev10000468m.g	scaffold_5:6756342-6768459	25.7907	25.8818	0.00508362
Ciclev10032440m.g	scaffold_4:20629462-20633732	23.753	23.8404	0.0053039
Ciclev10015404m.g	scaffold_2:30927775-30932047	28.9066	29.0143	0.00536872
Ciclev10005227m.g	scaffold_9:11215529-11221566	11.1808	11.2235	0.0055032
Ciclev10008437m.g	scaffold_1:24629646-24635508	147.661	148.234	0.00559427
Ciclev10022352m.g	scaffold_3:47099614-47109263	6.1795	6.20374	0.00564672
Ciclev10025657m.g,Ciclev10026661m.g	scaffold_7:5534602-5539527	11.403	11.4486	0.00575729
Ciclev10005941m.g	scaffold_9:2887551-2890750	7.67788	7.70875	0.00578846
Ciclev10002912m.g	scaffold_5:40603739-40604319	6.8078	6.83589	0.00593958
Ciclev10007782m.g	scaffold_1:14527800-14535285	18.7885	18.8663	0.00595471
Ciclev10032059m.g	scaffold_4:19331465-19333650	17.0211	17.093	0.0060816
Ciclev10008911m.g	scaffold_1:26810778-26816497	30.0968	30.2299	0.0063648
Ciclev10031073m.g,Ciclev10032680m.g	scaffold_4:23404931-23425248	57.6127	57.868	0.00637826
Ciclev10031375m.g,Ciclev10033371m.g	scaffold_4:467597-484070	12.9446	13.0057	0.00679553

Ciclev10001292m.g	scaffold_5:27065728-27070497	9.46313	9.50875	0.00693949
Ciclev10015385m.g	scaffold_2:4021166-4024377	815.044	819.048	0.00707006
Ciclev10018122m.g	scaffold_2:30901006-30904604	139.254	139.942	0.00711039
Ciclev10022455m.g	scaffold_3:42922693-42926274	44.5176	44.7385	0.00714388
Ciclev10026551m.g	scaffold_7:6444107-6448111	41.8421	42.0509	0.00718029
Ciclev10008686m.g	scaffold_1:10424094-10427597	66.6302	66.9654	0.0072404
Ciclev10001943m.g	scaffold_5:39677784-39679367	99.5542	100.055	0.00724542
Ciclev10012615m.g	scaffold_6:16418284-16421471	57.3847	57.6737	0.00724859
Ciclev10020575m.g	scaffold_3:7327403-7335639	18.3758	18.4692	0.00731566
Ciclev10023918m.g	scaffold_3:1084466-1086978	0.310601	0.312183	0.00733083
Ciclev10002344m.g	scaffold_5:39893322-39895874	119.769	120.382	0.00736066
Ciclev10015313m.g	scaffold_2:32836061-32841317	11.2095	11.2682	0.00753577
Ciclev10016374m.g	scaffold_2:27491551-27493045	14.633	14.7101	0.00758352
Ciclev10014107m.g	scaffold_2:34905088-34911624	7.66493	7.70541	0.00759968
-	scaffold_5:5805570-5807385	22.8429	22.9638	0.00761669
Ciclev10016776m.g	scaffold_2:32880455-32882923	17.8056	17.9005	0.0076686
Ciclev10009883m.g	scaffold_1:4622888-4624934	50.7897	51.0626	0.00772938
Ciclev10030616m.g	scaffold_4:17776018-17782386	119.631	120.28	0.00780667
Ciclev10032849m.g	scaffold_4:24111723-24114142	31.2216	31.3922	0.00786504
Ciclev10029319m.g	scaffold_8:24888285-24893511	8.16767	8.21232	0.00786512
Ciclev10033291m.g	scaffold_4:15087930-15266082	138.585	139.36	0.00804543
Ciclev10029401m.g	scaffold_8:305666-308738	26.9531	27.1044	0.00808018
Ciclev10024846m.g	scaffold_7:10932123-10935698	1.61285	1.62217	0.00831077
Ciclev10016241m.g	scaffold_2:10101189-10105053	79.2321	79.7005	0.00850414
-	scaffold_9:25890517-25893754	90.4121	90.9549	0.0086353
Ciclev10007904m.g	scaffold_1:24328900-24333161	10.5125	10.576	0.00868916
Ciclev10009464m.g	scaffold_1:1604941-1607921	49.5694	49.8701	0.0087243
Ciclev10004653m.g	scaffold_9:97226-100852	6.0751	6.11326	0.009033
Ciclev10028198m.g	scaffold_8:24419608-24425720	55.9661	56.322	0.00914678
Ciclev10020004m.g	scaffold_3:3050612-3056468	72.5967	73.0653	0.00928299
Ciclev10009825m.g	scaffold_1:3436725-3439978	19.4483	19.574	0.0092917
Ciclev10015910m.g	scaffold_2:34528354-34531488	7.32486	7.37254	0.00935916
Ciclev10013428m.g	scaffold_6:20243823-20247947	3.59313	3.6168	0.00947216
Ciclev10028505m.g	scaffold_8:19225736-19231654	5.88738	5.92662	0.00958249
Ciclev10020411m.g	scaffold_3:221807-224232	8.81735	8.87627	0.00960768
Ciclev10025461m.g	scaffold_7:16242047-16246857	28.5914	28.7915	0.0100631
Ciclev10000838m.g	scaffold_5:42371840-42373931	24.2094	24.3799	0.0101228
Ciclev10022372m.g	scaffold_3:24943609-24945394	31.6685	31.8951	0.0102885
Ciclev10012080m.g	scaffold_6:4005852-4015208	24.3628	24.5383	0.0103509
Ciclev10020928m.g	scaffold_3:40236707-40244926	9.83083	9.9039	0.0106842
Ciclev10005422m.g	scaffold_9:3700912-3705076	67.6168	68.1284	0.0108753
Ciclev10000793m.g	scaffold_5:15579156-15591668	50.4923	50.877	0.0109499
Ciclev10002221m.g	scaffold_5:41936718-41940030	8.08866	8.15034	0.0109583
Ciclev10012213m.g	scaffold_6:16589757-16592569	8.64152	8.70827	0.0111005
Ciclev10002478m.g	scaffold_5:42236417-42238575	10.0011	10.0792	0.011216
Ciclev10031988m.g	scaffold_4:22818800-22822277	95.9442	96.6957	0.0112561
Ciclev10004470m.g,Ciclev10004989m.g,Ciclev10005747m.g	scaffold_9:1148082-1172389	42.9436	43.2857	0.0114459
-	scaffold_7:5718909-5722082	8.90134	8.97256	0.0114976
Ciclev10001775m.g	scaffold_5:32315343-32320120	11.7368	11.8309	0.0115105
-	scaffold_2:9053922-9054287	18.8054	18.9576	0.0116313
Ciclev10026766m.g	scaffold_7:16875134-16877068	27.1253	27.3469	0.0117393
Ciclev10016103m.g	scaffold_2:3004548-3009540	10.3191	10.4035	0.0117592
Ciclev10022163m.g	scaffold_3:30012942-30016435	82.6434	83.3289	0.0119184
Ciclev10019746m.g	scaffold_3:48196021-48200802	31.0213	31.2801	0.0119853

Ciclev10028177m.g	scaffold_8:1294516-1297210	85.4387	86.1541	0.0120305
Ciclev10015921m.g	scaffold_2:21755323-21757468	12.1621	12.2644	0.0120955
Ciclev10004192m.g	scaffold_9:4983535-4993533	37.2766	37.5924	0.012172
Ciclev10015375m.g	scaffold_2:11344737-11350784	16.2444	16.3822	0.0121856
Ciclev10009426m.g	scaffold_1:4913878-4916578	196.438	198.118	0.0122888
Ciclev10013297m.g	scaffold_6:13750670-13756217	1950.18	1967.07	0.012441
Ciclev10008307m.g	scaffold_1:20022671-20027438	10.7463	10.8396	0.0124754
-	scaffold_5:9961309-9961600	28.9601	29.2119	0.0124908
Ciclev10010886m.g	scaffold_6:18624389-18644912	21.0445	21.2284	0.0125546
Ciclev10032527m.g	scaffold_4:7082498-7083644	1.3968	1.40912	0.012672
Ciclev10022740m.g	scaffold_3:7412377-7414923	103.963	104.893	0.0128547
Ciclev10011374m.g	scaffold_6:25443236-25450672	33.3704	33.6731	0.0130236
Ciclev10012044m.g	scaffold_6:6185825-6192865	21.0778	21.2697	0.013071
Ciclev10019028m.g	scaffold_3:25158171-25168081	130.252	131.449	0.0131898
Ciclev10031436m.g	scaffold_4:21153790-21158006	40.8974	41.2748	0.0132531
Ciclev10025154m.g	scaffold_7:7322971-7325525	11.0205	11.126	0.0137506
-	scaffold_8:3396301-3397384	6.50654	6.56919	0.0138251
Ciclev10023245m.g	scaffold_3:42784631-42787031	36.1085	36.4597	0.0139645
Ciclev10012013m.g	scaffold_6:21822312-21835198	3.90878	3.94699	0.0140333
Ciclev10012116m.g	scaffold_6:23462486-23466580	55.1778	55.7211	0.0141342
Ciclev10020973m.g	scaffold_3:17509019-17512265	5.17114	5.22241	0.0142337
-	scaffold_5:34202091-34206690	37.589	37.9629	0.0142799
Ciclev10014709m.g	scaffold_2:30054070-30060451	33.2147	33.5456	0.0143012
Ciclev10032487m.g	scaffold_4:3485907-3488046	11.0001	11.1106	0.014423
Ciclev10032816m.g	scaffold_4:20998707-21001535	6.99552	7.06584	0.0144302
Ciclev10012627m.g	scaffold_6:23109593-23113764	16.4787	16.6447	0.0144593
Ciclev10032806m.g	scaffold_4:893217-895239	8.11349	8.19549	0.0145074
Ciclev10027804m.g	scaffold_8:4856423-4864208	9.71671	9.81492	0.0145094
Ciclev10004862m.g	scaffold_9:17680493-17682839	4.50752	4.55316	0.014534
Ciclev10000484m.g	scaffold_5:11336688-11353774	7.09833	7.17048	0.0145891
Ciclev10015090m.g	scaffold_2:9323126-9327860	74.3452	75.1033	0.0146366
Ciclev10008747m.g	scaffold_1:21901402-21911501	3.47123	3.50717	0.0148607
Ciclev10005124m.g	scaffold_9:3843209-3846817	27.6841	27.9728	0.0149665
Ciclev10032324m.g	scaffold_4:2136442-2139662	181.375	183.287	0.0151352
Ciclev10004552m.g	scaffold_9:2533399-2538668	4.85927	4.91107	0.0153001
Ciclev10020610m.g,Ciclev10020801m.g,Ciclev10024150m.g	scaffold_3:38639877-38875210	3.41306	3.44971	0.0154082
Ciclev10031532m.g	scaffold_4:22045314-22050160	22.1417	22.3795	0.01541
Ciclev10015784m.g	scaffold_2:8381469-8387067	11.4601	11.5839	0.015503
Ciclev10019527m.g	scaffold_3:43914353-43920776	41.6489	42.1011	0.0155794
Ciclev10025383m.g	scaffold_7:8681725-8687812	63.5097	64.2031	0.0156667
Ciclev10032841m.g	scaffold_4:20149548-20150385	18.6651	18.8702	0.0157682
Ciclev10020558m.g,Ciclev10020561m.g	scaffold_3:35308423-35325009	14.6309	14.7938	0.0159772
Ciclev10008539m.g	scaffold_1:14999307-15006120	24.1317	24.4005	0.0159826
Ciclev10023032m.g	scaffold_3:18711385-18713655	62.8022	63.502	0.0159878
Ciclev10015049m.g	scaffold_2:23826322-23829613	88.5416	89.5384	0.0161512
Ciclev10025376m.g	scaffold_7:3122785-3127079	20.8909	21.1262	0.016157
Ciclev10021339m.g	scaffold_3:12826039-12830556	29.9166	30.2538	0.0161703
-	scaffold_5:34088243-34091898	7.31067	7.3933	0.0162144
Ciclev10001270m.g	scaffold_5:9214675-9219238	21.6964	21.9418	0.0162247
Ciclev10010819m.g	scaffold_1:27237493-27238333	5.43949	5.5011	0.0162495
Ciclev10028816m.g	scaffold_8:24843812-24846296	36.5433	36.9594	0.016334
Ciclev10032406m.g	scaffold_4:14565455-14568265	487.004	492.564	0.0163778
Ciclev10017311m.g	scaffold_2:33842526-33844587	199.667	201.951	0.0164097

Ciclev10007789m.g	scaffold_1:1434476-1438631	8.60155	8.70023	0.0164557
Ciclev10031578m.g	scaffold_4:25475965-25480702	24.3148	24.5963	0.0166045
Ciclev10000525m.g	scaffold_5:9713649-9935604	40.4412	40.912	0.0166987
Ciclev10008018m.g	scaffold_1:2521457-2523863	0.761177	0.770055	0.0167291
Ciclev10025327m.g	scaffold_7:255469-260075	14.8942	15.0695	0.0168792
Ciclev10025142m.g	scaffold_7:14303853-14308894	10.7453	10.8727	0.0170066
Ciclev10019745m.g	scaffold_3:2294215-2297791	10.4066	10.5303	0.0170542
Ciclev10019942m.g	scaffold_3:47687295-47692118	3.15324	3.19075	0.0170643
Ciclev10002120m.g	scaffold_5:26620636-26625312	15.4144	15.5981	0.0170879
Ciclev10011146m.g	scaffold_6:7091367-7100713	16.3147	16.5115	0.0173003
Ciclev10015028m.g	scaffold_2:486528-490767	2.22089	2.24774	0.0173422
Ciclev10026350m.g	scaffold_7:5289431-5293368	143.854	145.635	0.0177509
Ciclev10012260m.g	scaffold_6:23559844-23563738	16.5485	16.7537	0.0177816
Ciclev10011228m.g	scaffold_6:21634899-21642518	21.7479	22.018	0.017808
Ciclev10025599m.g	scaffold_7:5049158-5052734	2.95557	2.99237	0.0178515
Ciclev10019470m.g	scaffold_3:34619141-34622157	6.14092	6.2185	0.0181119
Ciclev10022164m.g	scaffold_3:29078195-29080185	1172.17	1186.99	0.0181344
Ciclev10029621m.g	scaffold_8:20137898-20139270	193.605	196.067	0.0182314
Ciclev10027713m.g	scaffold_8:21019346-21036698	12.4324	12.5908	0.0182656
Ciclev10032604m.g	scaffold_4:20126724-20132105	18.8718	19.1123	0.0182697
Ciclev10015324m.g	scaffold_2:33269484-33273841	9.90875	10.0357	0.0183685
Ciclev10032160m.g	scaffold_4:23887476-23889337	1.5316	1.55143	0.0185536
Ciclev10013771m.g	scaffold_6:18559952-18562820	140.61	142.449	0.0187465
Ciclev10003765m.g	scaffold_5:16345424-16346720	23.1391	23.4417	0.0187475
Ciclev10004791m.g	scaffold_9:29984483-29989049	17.2311	17.4567	0.0187602
Ciclev10033035m.g	scaffold_4:25619121-25619879	24.6659	24.9905	0.0188613
Ciclev10021511m.g	scaffold_3:50045013-50052161	20.8073	21.0813	0.0188756
Ciclev10017350m.g	scaffold_2:34984844-34985814	6.51491	6.60181	0.0191161
Ciclev10016281m.g	scaffold_2:8020119-8022107	38.9194	39.4422	0.0192485
Ciclev10023026m.g	scaffold_3:49451289-49452607	67.5366	68.4715	0.019834
Ciclev10032925m.g	scaffold_4:14351919-14356757	188.014	190.637	0.0199874
Ciclev10016139m.g	scaffold_2:9909042-9912785	7.65789	7.76544	0.0201208
Ciclev10009504m.g	scaffold_1:6903803-6918085	26.7449	27.1205	0.0201215
Ciclev10023159m.g	scaffold_3:1160991-1161777	4.42188	4.48477	0.0203748
Ciclev10027983m.g	scaffold_8:17088261-17094618	11.3522	11.5144	0.0204702
Ciclev10025076m.g	scaffold_7:1580231-1589493	22.1113	22.4288	0.0205665
Ciclev10012580m.g	scaffold_6:18165172-18167098	74.7922	75.8703	0.0206474
Ciclev10032457m.g	scaffold_4:19280283-19286048	12.6996	12.8839	0.0207915
Ciclev10021818m.g	scaffold_3:8908352-8913829	132.798	134.738	0.0209208
Ciclev10011110m.g	scaffold_6:19877107-19883332	37.819	38.3736	0.0210023
Ciclev10022990m.g	scaffold_3:37112247-37113743	363.066	368.399	0.0210392
Ciclev10022061m.g	scaffold_3:1203356-1206483	136.301	138.307	0.0210771
Ciclev10001847m.g	scaffold_5:35674415-35675935	4.98648	5.0602	0.0211713
Ciclev10002429m.g	scaffold_5:14956022-14957263	65.448	66.42	0.0212695
Ciclev10004434m.g	scaffold_9:6222186-6225652	0.277331	0.281469	0.0213681
Ciclev10018934m.g	scaffold_3:41621813-41627434	20.1225	20.4237	0.0214336
Ciclev10007804m.g	scaffold_1:24434598-24438564	5.54506	5.62824	0.0214803
Ciclev10027821m.g,Ciclev10030068m.g	scaffold_8:14355588-14380447	48.7668	49.4997	0.02152
Ciclev10004184m.g	scaffold_9:2318658-2324211	4.36858	4.43512	0.0218089
Ciclev10010026m.g	scaffold_1:17504347-17514220	1.07017	1.08669	0.0220991
Ciclev10011190m.g	scaffold_6:19473170-19479023	4.37886	4.44663	0.0221586
Ciclev10027780m.g	scaffold_8:24065122-24069971	8.23589	8.36448	0.0223503
Ciclev10022032m.g	scaffold_3:43850268-43852816	30.6604	31.1395	0.0223688
-	scaffold_5:18149824-18151138	46.9068	47.6458	0.0225512
Ciclev10011606m.g	scaffold_6:18112183-18134557	0.751256	0.763109	0.0225855

Ciclev10004611m.g	scaffold_9:4848641-4850807	1.16189	1.18023	0.0226008
Ciclev10004691m.g	scaffold_9:29575659-29579622	18.2047	18.4932	0.0226789
Ciclev10014778m.g	scaffold_2:12132151-12137292	31.6788	32.1822	0.0227475
Ciclev10002791m.g	scaffold_5:5098100-5099777	4.93931	5.01877	0.0230256
Ciclev10006948m.g	scaffold_9:20248357-20251630	53.0136	53.8685	0.0230802
Ciclev10029222m.g	scaffold_8:12899472-12901578	23.9064	24.2939	0.0231951
Ciclev10019847m.g	scaffold_3:2640117-2644300	4.14365	4.21084	0.0232053
Ciclev10018597m.g	scaffold_3:48792565-48802567	10.6026	10.7747	0.0232207
Ciclev10008137m.g	scaffold_1:4228014-4231637	12.9976	13.2092	0.0233031
-	scaffold_4:23753780-23753922	10801.6	10977.8	0.0233434
-	scaffold_2:23392854-23393281	12209.1	12408.9	0.0234184
Ciclev10021871m.g	scaffold_3:5437390-5439416	136.86	139.105	0.0234825
Ciclev10020189m.g	scaffold_3:33388539-33592383	37.5509	38.1689	0.0235508
Ciclev10007829m.g	scaffold_1:15883117-15891083	16.0314	16.2964	0.0236536
Ciclev10008604m.g	scaffold_1:27134772-27138192	20.6739	21.0159	0.0236729
Ciclev10009423m.g	scaffold_1:7328754-7330400	113.356	115.234	0.0237005
Ciclev10018625m.g	scaffold_3:15979996-15987727	50.5419	51.3794	0.0237101
Ciclev10018784m.g	scaffold_3:49636891-49644019	14.3297	14.5673	0.0237224
Ciclev10001627m.g	scaffold_5:15689221-15694276	23.6546	24.0475	0.0237712
Ciclev10020323m.g	scaffold_3:7707174-7710491	15.6194	15.8799	0.0238617
Ciclev10002666m.g	scaffold_5:35123212-35124730	74.9433	76.1989	0.0239694
Ciclev10008684m.g	scaffold_1:6772263-6775904	23.9234	24.3258	0.0240627
Ciclev10022847m.g	scaffold_3:17717681-17720651	30.9668	31.4909	0.0242118
Ciclev10012327m.g,Ciclev10013647m.g	scaffold_6:9429446-9443146	41.2081	41.907	0.0242603
Ciclev10012558m.g	scaffold_6:11112518-11114011	24.964	25.3899	0.0244059
Ciclev10022116m.g	scaffold_3:48470068-48472197	814.379	828.285	0.0244282
Ciclev10023391m.g	scaffold_3:22424734-22428364	0.985407	1.00226	0.0244586
Ciclev10028167m.g	scaffold_8:574203-577434	2.07693	2.11301	0.0248507
Ciclev10016955m.g	scaffold_2:24619304-24622426	13.2138	13.4453	0.0250521
Ciclev10018801m.g	scaffold_3:46034238-46042845	76.3918	77.7312	0.0250757
-	scaffold_3:50629161-50632106	42.7451	43.4995	0.0252403
Ciclev10008074m.g	scaffold_1:8875647-8878444	10.9884	11.1828	0.0253062
Ciclev10019525m.g	scaffold_3:49038081-49043531	8.69644	8.85099	0.0254136
Ciclev10025036m.g	scaffold_7:11670714-11674605	8.34702	8.49576	0.0254816
Ciclev10005332m.g	scaffold_9:27161750-27166301	30.6594	31.2112	0.0257343
Ciclev10001788m.g	scaffold_5:42608365-42611050	80.9763	82.4369	0.0257903
Ciclev10015541m.g	scaffold_2:26890334-26894409	31.3498	31.9162	0.0258295
Ciclev10012924m.g	scaffold_6:9001034-9007822	24.1985	24.6378	0.0259556
Ciclev10025180m.g	scaffold_7:20803910-20809080	4.43802	4.519	0.026087
Ciclev10030610m.g	scaffold_4:3367315-3376853	22.7266	23.1434	0.0262199
Ciclev10031726m.g	scaffold_4:25178432-25182794	14.8441	15.1165	0.0262321
Ciclev10016173m.g	scaffold_2:35045748-35049870	17.8988	18.2315	0.0265648
Ciclev10022855m.g	scaffold_3:13024245-13033708	163.028	166.06	0.0265852
Ciclev10007558m.g	scaffold_1:24324106-24328344	15.9213	16.2177	0.0266171
Ciclev10030361m.g	scaffold_8:6750380-6786902	0.648643	0.660733	0.0266434
Ciclev10031031m.g	scaffold_4:3308158-3312351	59.3065	60.4151	0.0267187
Ciclev10025839m.g	scaffold_7:2394470-2397293	19.3499	19.7123	0.0267656
Ciclev10028015m.g	scaffold_8:2105332-2111110	33.4773	34.1084	0.0269426
Ciclev10024345m.g	scaffold_3:21763501-21767140	2.32926	2.37317	0.026943
Ciclev10018889m.g	scaffold_3:46011237-46017249	67.8566	69.137	0.0269687
Ciclev10000739m.g	scaffold_5:35022339-35027549	17.6816	18.0159	0.0270217
Ciclev10026715m.g	scaffold_7:252319-254049	262.496	267.473	0.0270947
Ciclev10014131m.g	scaffold_2:25242457-25249484	14.2995	14.5719	0.0272244
Ciclev10030901m.g,Ciclev10033376m.g	scaffold_4:21620341-21627100	55.9417	57.0112	0.0273209

Ciclev10021870m.g	scaffold_3:1955304-1957656	9.93483	10.125	0.0273556
Ciclev10013033m.g,Ciclev10013868m.g	scaffold_6:17752818-17759941	410.969	418.866	0.0274598
Ciclev10020269m.g	scaffold_3:44362970-44366792	12.9093	13.1578	0.0275149
Ciclev10024201m.g	scaffold_3:25425989-25427403	55.8627	56.9494	0.0277958
Ciclev10015425m.g,Ciclev10018395m.g	scaffold_2:645022-651088	19.5885	19.9696	0.0277999
Ciclev10001186m.g	scaffold_5:13036882-13040255	27.9319	28.4782	0.0279449
Ciclev10001390m.g	scaffold_5:30572950-30577888	231.845	236.381	0.027952
Ciclev10029091m.g	scaffold_8:21777980-21780912	20.2469	20.6446	0.0280636
Ciclev10009817m.g	scaffold_1:23419298-23421924	42.3306	43.1623	0.0280685
Ciclev10010996m.g	scaffold_6:16836844-16842068	4.75521	4.8489	0.0281497
Ciclev10000917m.g	scaffold_5:17392923-17476446	0.432456	0.440984	0.0281719
Ciclev10022728m.g	scaffold_3:7851658-7854615	42.1864	43.0196	0.0282166
Ciclev10015615m.g	scaffold_2:26035371-26040724	12.5067	12.7538	0.0282246
Ciclev10025195m.g	scaffold_7:16123605-16128864	17.642	17.9933	0.0284453
Ciclev10026457m.g	scaffold_7:5173845-5179864	37.9908	38.748	0.028471
Ciclev10004702m.g	scaffold_9:26168832-26178068	12.803	13.0596	0.0286253
Ciclev10019968m.g	scaffold_3:45393856-45398298	6.92319	7.06203	0.028647
Ciclev10002193m.g	scaffold_5:32699552-32702689	92.117	93.9669	0.0286845
Ciclev10015833m.g	scaffold_2:7874155-7880463	90.4065	92.237	0.0289184
Ciclev10025882m.g	scaffold_7:1855642-1858827	113.754	116.063	0.0289865
Ciclev10032502m.g	scaffold_4:15056833-15058632	3219.93	3285.49	0.0290804
Ciclev10011124m.g	scaffold_6:24975343-24986759	49.0413	50.0432	0.0291762
Ciclev10015882m.g	scaffold_2:14736757-14739931	23.806	24.2962	0.0294057
Ciclev10012464m.g	scaffold_6:21575873-21577613	7.02134	7.16745	0.0297143
Ciclev10031217m.g	scaffold_4:2080912-2082940	2.74984	2.80757	0.0299746
Ciclev10028783m.g	scaffold_8:8008757-8012156	17.8217	18.1999	0.0302985
Ciclev10005787m.g	scaffold_9:13223033-13225922	5.81353	5.93703	0.0303258
Ciclev10000625m.g	scaffold_5:15344389-15349247	17.3892	17.759	0.0303585
Ciclev10020523m.g	scaffold_3:42439235-42441625	11.8007	12.0521	0.0304137
Ciclev10021628m.g	scaffold_3:47867871-47869626	103.361	105.571	0.0305197
Ciclev10010891m.g	scaffold_6:18818854-18834174	7.74989	7.9159	0.0305778
Ciclev10032648m.g	scaffold_4:21274489-21276044	200.285	204.577	0.03059
Ciclev10021811m.g	scaffold_3:43869582-43874373	41.5272	42.4256	0.0308783
Ciclev10011055m.g	scaffold_6:11871159-11876723	110.098	112.482	0.0309052
Ciclev10028779m.g	scaffold_8:5904925-5909700	6.3063	6.44347	0.0310449
Ciclev10018362m.g	scaffold_2:31567506-31569260	5.36553	5.48261	0.0311428
Ciclev10004047m.g	scaffold_5:22184567-22185917	19.6674	20.0974	0.0312042
Ciclev10022381m.g	scaffold_3:39759328-39764478	61.3076	62.6486	0.0312157
Ciclev10010411m.g	scaffold_1:17993530-17995718	26.3673	26.9479	0.0314208
Ciclev10013572m.g	scaffold_6:13444057-13444441	3.82934	3.91403	0.0315584
Ciclev10031798m.g	scaffold_4:3448975-3453191	11.7216	11.9814	0.0316241
Ciclev10012181m.g	scaffold_6:8936704-8942681	14.42	14.7399	0.0316621
-	scaffold_3:48676940-48679147	99.0809	101.298	0.0319257
Ciclev10032741m.g	scaffold_4:24147412-24149652	46.3974	47.4365	0.0319535
-	scaffold_5:32828334-32829161	21.7285	22.2166	0.0320492
Ciclev10004277m.g,Ciclev10006112m.g	scaffold_9:31123224-31144160	21.6186	22.1056	0.0321392
Ciclev10026419m.g	scaffold_7:8724648-8725833	29.3484	30.0147	0.0323905
Ciclev10018828m.g	scaffold_3:13369114-13379375	2.86412	2.93	0.0328093
Ciclev10008357m.g	scaffold_1:4264566-4266327	5.24355	5.36428	0.0328414
Ciclev10013767m.g	scaffold_6:20503216-20506773	14.2399	14.5681	0.0328735
Ciclev10025630m.g	scaffold_7:3532904-3535627	20.9175	21.4029	0.0330968
Ciclev10021522m.g	scaffold_3:40116212-40124281	19.1596	19.6092	0.0334659
Ciclev10020226m.g	scaffold_3:599754-602347	14.99	15.3428	0.0335624

Ciclev10008567m.g	scaffold_1:26892966-26898736	44.1341	45.1823	0.0338638
Ciclev10028563m.g	scaffold_8:15991330-15994901	20.8544	21.3504	0.0339072
Ciclev10001133m.g	scaffold_5:36894364-36899326	49.8009	50.99	0.0340432
Ciclev10008716m.g	scaffold_1:10409669-10413863	22.2945	22.8291	0.034186
Ciclev10010910m.g	scaffold_6:22795567-22805136	7.07668	7.2467	0.0342528
Ciclev10010030m.g	scaffold_1:2674514-2681844	4.14888	4.24864	0.034279
Ciclev10022415m.g	scaffold_3:21945866-21947624	27.07	27.7245	0.0344686
Ciclev10021976m.g	scaffold_3:49912778-49915964	19.4963	19.969	0.034559
Ciclev10004603m.g	scaffold_9:22671553-22687084	19.5089	19.9858	0.0348458
Ciclev10007388m.g	scaffold_1:22665982-22675655	5.77931	5.92116	0.0349811
Ciclev10022870m.g	scaffold_3:45827408-45829201	102.304	104.817	0.0350108
Ciclev10004200m.g	scaffold_9:29415524-29424081	31.8415	32.6259	0.0351081
Ciclev10031084m.g	scaffold_4:13567072-13573185	10.3916	10.6488	0.0352741
Ciclev10018892m.g	scaffold_3:5393850-5397793	3.32553	3.40799	0.0353367
Ciclev10033040m.g	scaffold_4:17149087-17151664	19.8164	20.309	0.0354258
Ciclev10030537m.g	scaffold_4:23303369-23319905	14.8803	15.2525	0.0356341
-	scaffold_5:4076819-4284428	52.5803	53.8975	0.0356963
Ciclev10019689m.g	scaffold_3:24796350-24800588	43.7082	44.8041	0.0357282
Ciclev10004188m.g	scaffold_9:7450000-7456419	1.66595	1.70788	0.0358544
Ciclev10001595m.g	scaffold_5:12849307-12853770	3.77746	3.8726	0.0358846
Ciclev10004114m.g	scaffold_9:421985-459245	19.3652	19.8541	0.0359666
Ciclev10032745m.g	scaffold_4:18894201-18895208	8.65336	8.87243	0.0360688
Ciclev10023704m.g	scaffold_3:2177776-2183574	10.2221	10.4809	0.0360718
Ciclev10008730m.g	scaffold_1:1485413-1488641	38.3935	39.3672	0.0361325
Ciclev10006180m.g	scaffold_9:2267071-2269627	147.373	151.11	0.0361329
Ciclev10029115m.g	scaffold_8:23480482-23484398	161.692	165.803	0.0362207
Ciclev10028695m.g	scaffold_8:157206-162831	30.694	31.4776	0.0363676
Ciclev10007562m.g	scaffold_1:27148936-27154125	50.7723	52.071	0.0364373
Ciclev10031009m.g	scaffold_4:12824060-12831489	21.1158	21.6564	0.036471
Ciclev10007601m.g	scaffold_1:22571740-22577273	11.0794	11.3643	0.0366301
Ciclev10022335m.g	scaffold_3:7498423-7499675	4.25134	4.36067	0.036632
Ciclev10030951m.g	scaffold_4:21192982-21199939	15.6888	16.0923	0.0366336
Ciclev10004547m.g	scaffold_9:987367-993071	13.9555	14.3154	0.0367308
Ciclev10007566m.g	scaffold_1:24362167-24371811	49.0105	50.2754	0.0367612
Ciclev10019296m.g	scaffold_3:19241016-19244670	10.2809	10.5493	0.0371781
Ciclev10003581m.g,Ciclev10004097m.g	scaffold_5:6455469-6459625	43.2587	44.389	0.0372111
Ciclev10000290m.g	scaffold_5:40126604-40138178	34.8344	35.7711	0.0382829
-	scaffold_6:11594026-11596313	65.8906	67.6717	0.0384804
Ciclev10019105m.g	scaffold_3:6748405-6756264	53.0389	54.4775	0.0386098
Ciclev10019829m.g	scaffold_3:501035-508040	19.5794	20.1108	0.0386342
Ciclev10001908m.g	scaffold_5:40317720-40321609	75.4646	77.5299	0.0389516
Ciclev10003247m.g	scaffold_5:26611274-26611664	100.677	103.433	0.0389606
Ciclev10003824m.g	scaffold_5:1552172-1984270	8.25281	8.48045	0.039254
Ciclev10025421m.g	scaffold_7:6448890-6453596	17.7824	18.2731	0.0392708
Ciclev10015010m.g	scaffold_2:8258113-8264958	11.846	12.1733	0.0393238
Ciclev10003996m.g	scaffold_5:27123825-27127430	1.78479	1.83412	0.039332
Ciclev10000146m.g	scaffold_5:37770211-37775957	65.3482	67.156	0.039367
Ciclev10014925m.g	scaffold_2:30561080-30566547	38.0719	39.1283	0.0394872
Ciclev10020924m.g	scaffold_3:7688340-7690805	11.1344	11.4436	0.0395194
Ciclev10002470m.g	scaffold_5:40689092-40691266	255.146	262.258	0.0396634
Ciclev10000386m.g	scaffold_5:34226353-34229316	2.34991	2.41547	0.0397009
Ciclev10002444m.g	scaffold_5:41450880-41454042	15.6328	16.0692	0.0397216
Ciclev10015598m.g	scaffold_2:21640161-21644769	11.9938	12.329	0.0397658
Ciclev10008648m.g	scaffold_1:2440597-2443742	12.8734	13.2334	0.039795
-	scaffold_5:10711182-10711834	2.28439	2.34838	0.0398583

Ciclev10008354m.g	scaffold_1:23415588-23419190	3.34939	3.44418	0.0402646
Ciclev10012668m.g	scaffold_6:23881967-23889247	19.8174	20.3786	0.0402854
Ciclev10011118m.g	scaffold_6:18256827-18261268	1.13051	1.16254	0.0402991
Ciclev10007305m.g	scaffold_1:26540978-26555789	27.6972	28.4848	0.0404527
Ciclev10031905m.g	scaffold_4:20755625-20759174	17.7206	18.2254	0.0405221
Ciclev10014090m.g	scaffold_2:30671354-30681515	13.5231	13.9088	0.0405718
Ciclev10000483m.g	scaffold_5:41138185-41140305	2.35093	2.41801	0.0405863
Ciclev10025536m.g	scaffold_7:4701795-4706997	27.6329	28.4217	0.040605
Ciclev10002227m.g	scaffold_5:42249317-42252520	101.058	103.948	0.0406706
Ciclev10002306m.g	scaffold_5:38738186-38739919	1.38482	1.42446	0.0407196
Ciclev10021009m.g	scaffold_3:3324860-3328761	44.4677	45.7413	0.0407382
Ciclev10011895m.g	scaffold_6:14174811-14178267	16.9101	17.3954	0.0408223
-	scaffold_6:18382260-18382764	955.865	983.34	0.0408839
Ciclev10018484m.g	scaffold_3:1436578-1444361	5.66789	5.83131	0.0410097
Ciclev10012168m.g	scaffold_6:22658269-22659772	23.4879	24.1682	0.0411932
Ciclev10021376m.g	scaffold_3:12706375-12710067	16.7125	17.1969	0.0412227
Ciclev10000461m.g	scaffold_5:32951374-32963622	4.31881	4.44441	0.0413579
Ciclev10015761m.g	scaffold_2:10253618-10264079	20.8621	21.4691	0.0413772
Ciclev10007536m.g	scaffold_1:22974132-22979718	61.4593	63.2478	0.0413832
Ciclev10014553m.g	scaffold_2:33631879-33636206	3.22938	3.32352	0.0414516
Ciclev10009865m.g	scaffold_1:20488461-20489759	520.257	535.493	0.0416423
Ciclev10024617m.g	scaffold_3:27203493-27205968	52.0847	53.6131	0.0417252
Ciclev10002360m.g	scaffold_5:17549352-17550928	46.7123	48.1003	0.0422424
Ciclev10027619m.g	scaffold_7:12324372-12327246	4.08815	4.21052	0.0425491
Ciclev10019002m.g	scaffold_3:16357226-16363309	52.2974	53.8704	0.042754
Ciclev10011385m.g	scaffold_6:18726196-18730460	14.8177	15.265	0.0429038
Ciclev10027859m.g	scaffold_8:1094365-1097096	18.7769	19.3437	0.0429041
Ciclev10021827m.g	scaffold_3:42433520-42436870	25.0432	25.8003	0.0429681
Ciclev10021031m.g	scaffold_3:45371716-45374708	53.7897	55.4204	0.0430867
Ciclev10017051m.g	scaffold_2:27856112-27857044	16.6531	17.1592	0.0431908
Ciclev10030699m.g	scaffold_4:15562924-15568021	5.62867	5.80094	0.0434942
Ciclev10028595m.g	scaffold_8:2349654-2354496	368.506	379.925	0.0440275
Ciclev10014731m.g	scaffold_2:29287736-29293648	10.5336	10.8604	0.0440811
Ciclev10004211m.g	scaffold_9:1799555-1809946	47.5406	49.0172	0.0441276
Ciclev10008864m.g	scaffold_1:9314856-9317133	13.2548	13.6666	0.0441395
Ciclev10000122m.g	scaffold_5:42042701-42048167	17.2956	17.8332	0.0441551
Ciclev10031404m.g	scaffold_4:302165-306822	5.42782	5.59663	0.0441856
Ciclev10001476m.g	scaffold_5:42853522-42856666	26.634	27.463	0.044222
Ciclev10005847m.g	scaffold_9:4479705-4481245	217.471	224.29	0.0445436
Ciclev10025544m.g	scaffold_7:14581361-14584518	104.318	107.589	0.044545
Ciclev10022571m.g	scaffold_3:47506791-47523094	820.555	846.446	0.0448193
Ciclev10021825m.g	scaffold_3:50534508-50538907	29.8154	30.7564	0.0448263
Ciclev10016375m.g	scaffold_2:35780437-35782811	32.523	33.5523	0.044948
Ciclev10031785m.g	scaffold_4:20051147-20058257	31.5376	32.5372	0.0450155
Ciclev10011020m.g,Ciclev10011630m.g	scaffold_6:19344904-19364132	15.2496	15.7332	0.0450357
Ciclev10032187m.g	scaffold_4:19762281-19765759	83.8779	86.5412	0.0450954
Ciclev10028631m.g	scaffold_8:24315719-24317671	1.31182	1.3535	0.0451158
Ciclev10031316m.g	scaffold_4:23949259-23950873	0.926826	0.956283	0.0451398
Ciclev10005842m.g	scaffold_9:22394206-22395728	100.031	103.211	0.0451459
Ciclev10022821m.g	scaffold_3:39523946-39529043	41.4277	42.7514	0.0453771
Ciclev10030727m.g	scaffold_4:17549043-17556268	3.88318	4.00728	0.0453835
Ciclev10000583m.g	scaffold_5:37295740-37302243	8.21871	8.4814	0.0453901
Ciclev10019070m.g	scaffold_3:549126-552792	19.4754	20.0985	0.0454356
Ciclev10001227m.g	scaffold_5:33385403-33390936	51.8805	53.5425	0.0454917
Ciclev10033357m.g	scaffold_4:3631069-3633691	4.7857	4.93908	0.0455119

Ciclev10017882m.g	scaffold_2:11620001-11621773	5.1002	5.26475	0.0458099
Ciclev10025073m.g	scaffold_7:16787405-16791422	6.43859	6.64695	0.0459483
Ciclev10009964m.g	scaffold_1:23411736-23412572	64.5014	66.5938	0.0460572
Ciclev10014456m.g	scaffold_2:8023716-8026756	6.09857	6.29649	0.0460776
Ciclev10030786m.g,Ciclev10033966m.g	scaffold_4:1591644-1639717	23.7427	24.5142	0.0461355
Ciclev10012366m.g	scaffold_6:24225829-24230019	44.6847	46.1508	0.0465753
Ciclev10004351m.g	scaffold_9:10326870-10334844	64.6907	66.8164	0.0466455
Ciclev10015636m.g	scaffold_2:24637052-24639946	12.8653	13.2889	0.0467358
Ciclev10005924m.g	scaffold_9:27727994-27730913	113.868	117.627	0.0468564
Ciclev10016252m.g	scaffold_2:30276677-30280577	73.4638	75.891	0.0468943
Ciclev10019481m.g	scaffold_3:49419039-49425912	4.5735	4.72505	0.0470292
Ciclev10009173m.g	scaffold_1:10890350-10895564	6.70486	6.92722	0.0470703
Ciclev10015447m.g	scaffold_2:28008518-28012694	5.08615	5.25484	0.0470744
Ciclev10011976m.g	scaffold_6:20433543-20436097	16.9454	17.5103	0.047309
Ciclev10000361m.g	scaffold_5:35014092-35021589	12.3138	12.7244	0.0473225
Ciclev10010579m.g	scaffold_1:8076057-8079627	2.24751	2.3228	0.0475354
Ciclev10021465m.g	scaffold_3:4298659-4301610	33.376	34.496	0.0476199
Ciclev10009642m.g	scaffold_1:22402592-22405792	5.33205	5.51144	0.0477374
Ciclev10028933m.g	scaffold_8:19400053-19404097	26.9267	27.8337	0.0477939
Ciclev10026407m.g,Ciclev10027256m.g	scaffold_7:11062213-11079547	4.4426	4.59239	0.0478419
Ciclev10024256m.g	scaffold_3:25474879-25477291	29.8962	30.9065	0.0479521
Ciclev10010911m.g,Ciclev10010912m.g	scaffold_6:9178837-9252105	3.99114	4.12622	0.0480221
-	scaffold_2:15477859-15615778	8.36348	8.6468	0.0480624
Ciclev10008953m.g	scaffold_1:18688872-18690138	0.827326	0.855418	0.0481739
Ciclev10013247m.g	scaffold_6:15399100-15400360	24.4341	25.2672	0.0483678
Ciclev10032771m.g	scaffold_4:23804128-23807087	4.07404	4.21295	0.0483717
Ciclev10023836m.g	scaffold_3:50083356-50087839	44.4839	46.0014	0.0483954
Ciclev10000415m.g	scaffold_5:33642730-33648714	34.4461	35.6214	0.0484009
Ciclev10028128m.g	scaffold_8:1607204-1610674	18.0358	18.6517	0.0484423
-	scaffold_9:3259645-3260042	106.962	110.618	0.0484819
Ciclev10020950m.g	scaffold_3:3391579-3395278	7.04452	7.2853	0.048487
Ciclev10021820m.g	scaffold_3:42034254-42039591	38.289	39.6	0.048569
Ciclev10026090m.g	scaffold_7:7351567-7354830	21.0656	21.7873	0.0486003
Ciclev10021988m.g	scaffold_3:49972519-49975925	22.4953	23.2689	0.0487844
Ciclev10029856m.g	scaffold_8:21037536-21042517	14.1729	14.6608	0.0488212
Ciclev10031350m.g	scaffold_4:3828231-3831707	10.3815	10.7402	0.0489989
Ciclev10011603m.g	scaffold_6:9847501-9854667	60.4677	62.557	0.0490075
Ciclev10023962m.g	scaffold_3:3461372-3466036	16.6533	17.2291	0.0490425
Ciclev10031034m.g	scaffold_4:12333307-12336127	21.4391	22.182	0.0491454
Ciclev10032646m.g	scaffold_4:12320438-12324228	113.555	117.499	0.0492634
Ciclev10025192m.g	scaffold_7:2949817-2954597	31.2278	32.3135	0.0493069
Ciclev10023123m.g	scaffold_3:45383456-45384566	16.0862	16.6467	0.0494177
Ciclev10002473m.g	scaffold_5:38139261-38142080	8.41861	8.71214	0.0494443
Ciclev10021491m.g	scaffold_3:50736749-50739959	46.8485	48.4863	0.0495742
Ciclev10014735m.g	scaffold_2:270631-276553	13.6621	14.1399	0.0495927
Ciclev10011464m.g	scaffold_6:12988555-12992279	17.0345	17.6309	0.0496482
Ciclev10001790m.g	scaffold_5:4289870-4293690	10.3165	10.6779	0.0496753
Ciclev10001220m.g	scaffold_5:2039384-2044991	37.3993	38.7126	0.0497903
Ciclev10011789m.g	scaffold_6:22955787-22957989	2.4445	2.53096	0.0501472
Ciclev10020615m.g	scaffold_3:50815812-50819403	14.4354	14.9465	0.0501993
Ciclev10015218m.g	scaffold_2:19525670-19529613	7.97421	8.25742	0.0503491
Ciclev10022247m.g	scaffold_3:38025538-38028617	10.7656	11.1486	0.0504263
Ciclev10022534m.g	scaffold_3:33204020-33205147	3.17931	3.29251	0.0504727

Ciclev10008697m.g	scaffold_1:20885101-20889290	23.5101	24.3512	0.0507126
Ciclev10005797m.g,Ciclev10006356m.g	scaffold_9:27790597-27828145	21.9633	22.7495	0.0507421
Ciclev10014224m.g	scaffold_2:34817074-34823291	41.3515	42.8339	0.050813
Ciclev10004800m.g	scaffold_9:30856526-30859889	9.62262	9.96806	0.050884
Ciclev10013207m.g	scaffold_6:23311866-23313814	26.7585	27.7203	0.0509435
Ciclev10023088m.g	scaffold_3:217086-218986	46.8504	48.537	0.0510223
Ciclev10015749m.g	scaffold_2:23323648-23326397	20.2492	20.9785	0.0510474
-	scaffold_6:13796771-13797091	7.76619	8.04613	0.0510871
Ciclev10000167m.g	scaffold_5:30744685-30751821	19.7417	20.4545	0.0511723
Ciclev10031139m.g	scaffold_4:3135017-3141002	8.95158	9.27504	0.0512101
Ciclev10032073m.g	scaffold_4:3290272-3293928	15.3963	15.9546	0.051386
Ciclev10021072m.g	scaffold_3:41985548-41991032	155.372	161.008	0.0514121
Ciclev10015448m.g	scaffold_2:29806648-29811506	50.712	52.5521	0.0514221
Ciclev10007250m.g	scaffold_1:3552295-3566235	26.056	27.0021	0.0514539
Ciclev10012700m.g	scaffold_6:25131255-25133522	49.7384	51.5474	0.051542
Ciclev10001941m.g,Ciclev10001942m.g	scaffold_5:30537417-30543922	98.3164	101.902	0.0516843
Ciclev10030867m.g	scaffold_4:22079445-22084318	7.96442	8.25535	0.0517602
Ciclev10016760m.g	scaffold_2:12125405-12128781	40.2698	41.7425	0.0518186
Ciclev10029611m.g	scaffold_8:6322766-6323840	21.5111	22.2998	0.0519452
Ciclev10019661m.g	scaffold_3:48957425-48965499	57.7787	59.8992	0.051998
Ciclev10013028m.g	scaffold_6:21845348-21846126	30.345	31.4604	0.0520771
Ciclev10027671m.g	scaffold_8:186494-196315	11.2738	11.6891	0.0521904
Ciclev10013051m.g	scaffold_6:20834426-20836151	214.849	222.798	0.0524128
Ciclev10033523m.g	scaffold_4:7532854-7706546	1.56864	1.62668	0.0524185
Ciclev10005538m.g	scaffold_9:3810721-3813210	24.0368	24.9276	0.0525036
Ciclev10022155m.g	scaffold_3:44374647-44377990	32.1819	33.3786	0.0526718
Ciclev10033083m.g	scaffold_4:3277374-3279208	212.456	220.385	0.0528604
Ciclev10007306m.g	scaffold_1:42874-54795	29.1712	30.2612	0.0529197
Ciclev10018223m.g	scaffold_2:16628954-16635471	11.4362	11.8636	0.0529408
Ciclev10025307m.g	scaffold_7:285298-292550	104.68	108.595	0.0529747
Ciclev10029481m.g	scaffold_8:989581-991156	96.4248	100.032	0.0529829
Ciclev10014634m.g	scaffold_2:9739205-9752276	8.19662	8.50386	0.0530897
Ciclev10019079m.g	scaffold_3:50281511-50287997	11.4757	11.9061	0.0531213
Ciclev10032053m.g	scaffold_4:2537672-2541483	12.6519	13.127	0.0531884
Ciclev10006210m.g	scaffold_9:31103264-31104961	33.5692	34.8301	0.0531937
Ciclev10029248m.g	scaffold_8:19272550-19276355	32.5141	33.7405	0.0534178
Ciclev10033081m.g	scaffold_4:21116125-21118815	171.546	178.018	0.0534297
Ciclev10000351m.g	scaffold_5:37134498-37138336	0.818009	0.848889	0.0534591
Ciclev10007450m.g	scaffold_1:25967214-25971892	18.2126	18.9012	0.0535397
Ciclev10011842m.g	scaffold_6:21598428-21600967	944.9	980.637	0.0535589
Ciclev10017754m.g	scaffold_2:22442840-22449517	89.2742	92.6546	0.0536192
Ciclev10031167m.g	scaffold_4:12376181-12382266	58.1797	60.3829	0.053624
Ciclev10004639m.g	scaffold_9:1787120-1791691	9.31307	9.66698	0.0538087
Ciclev10016548m.g	scaffold_2:21026742-21027937	11.7236	12.1692	0.0538114
Ciclev10007310m.g	scaffold_1:3940929-3948351	1486	1542.54	0.0538695
Ciclev10018936m.g	scaffold_3:46439053-46446904	17.9422	18.6265	0.0540012
Ciclev10026013m.g	scaffold_7:5466844-5469850	28.4948	29.5832	0.0540794
Ciclev10024583m.g	scaffold_3:23780859-23784857	1.25254	1.30039	0.0540902
Ciclev10008014m.g	scaffold_1:25646953-25655216	34.5729	35.8973	0.0542339
Ciclev10001469m.g	scaffold_5:13187677-13190472	7.93479	8.23936	0.0543396
Ciclev10023499m.g	scaffold_3:47055470-47056769	26.8768	27.9099	0.0544162
Ciclev10006879m.g	scaffold_9:20890036-21029395	0.963622	1.00075	0.054548
Ciclev10011123m.g	scaffold_6:18098919-18111094	17.3971	18.0683	0.0546101
Ciclev10020621m.g	scaffold_3:35360009-35365931	33.2597	34.5481	0.0548331

Ciclev10030925m.g	scaffold_4:9171771-9179197	36.65	38.074	0.0549927
Ciclev10005411m.g	scaffold_9:29534762-29538335	103.028	107.042	0.0551442
Ciclev10032140m.g	scaffold_4:21814596-21817535	16.2872	16.923	0.05524
Ciclev10006353m.g	scaffold_9:28497953-28514398	125.759	130.691	0.0554955
Ciclev10016090m.g	scaffold_2:77304-82511	31.8069	33.0552	0.0555401
Ciclev10005100m.g	scaffold_9:465443-468143	41.3485	42.9832	0.0559386
Ciclev10013059m.g	scaffold_6:23407232-23410378	124.893	129.833	0.0559697
Ciclev10017690m.g	scaffold_2:25420226-25420895	25.4038	26.4098	0.0560322
Ciclev10029042m.g	scaffold_8:7890298-7895996	57.4941	59.7724	0.0560651
Ciclev10021617m.g	scaffold_3:17462609-17468154	5.6988	5.92476	0.0560999
Ciclev10000372m.g	scaffold_5:41591990-41602791	27.2564	28.3384	0.0561633
Ciclev10005673m.g	scaffold_9:960840-962589	14.8991	15.4911	0.0562226
Ciclev10028457m.g	scaffold_8:789490-793183	105.18	109.367	0.0563232
Ciclev10008655m.g	scaffold_1:6617920-6622625	39.1683	40.7285	0.0563507
Ciclev10015050m.g	scaffold_2:28085183-28090646	15.7348	16.3629	0.0564646
Ciclev10001147m.g	scaffold_5:35232080-35237039	17.1446	17.8291	0.0564758
-	scaffold_3:35214149-35214696	257.332	267.612	0.0565112
Ciclev10011230m.g	scaffold_6:19578178-19583475	32.6819	33.9913	0.0566758
Ciclev10028005m.g	scaffold_8:671524-674851	8.36689	8.70227	0.056701
Ciclev10021690m.g	scaffold_3:48909434-48912096	70.5628	73.3921	0.0567162
Ciclev10026333m.g	scaffold_7:17574669-17579275	37.0003	38.4856	0.0567826
Ciclev10020798m.g	scaffold_3:34688016-34694549	54.4002	56.5868	0.0568549
Ciclev10009415m.g	scaffold_1:22999423-23001838	289.184	300.824	0.056932
Ciclev10007403m.g	scaffold_1:2566452-2570870	25.0351	26.05	0.0573325
Ciclev10007793m.g	scaffold_1:8791913-8799826	55.1479	57.3857	0.0573843
Ciclev10005476m.g	scaffold_9:3616195-3621117	23.2293	24.173	0.0574559
Ciclev10002111m.g	scaffold_5:21187557-21191341	26.1252	27.1871	0.0574779
Ciclev10031147m.g	scaffold_4:7200122-7204232	74.8952	77.9416	0.0575186
Ciclev10018919m.g	scaffold_3:11670819-11674422	10.8644	11.3102	0.0580219
Ciclev10014521m.g	scaffold_2:32395350-32398630	5.55571	5.78376	0.0580366
Ciclev10026570m.g	scaffold_7:6771772-6772812	23.1398	24.0952	0.0583683
Ciclev10003830m.g	scaffold_5:12898146-12904147	9.74385	10.147	0.058484
Ciclev10002965m.g	scaffold_5:36856825-36859536	21.1069	21.9827	0.0586591
Ciclev10001251m.g	scaffold_5:38695624-38699629	33.7685	35.1711	0.0587137
-	scaffold_8:24971655-24975242	8.35269	8.69972	0.0587279
Ciclev10031002m.g	scaffold_4:1507630-1511951	0.301322	0.313889	0.0589503
Ciclev10022404m.g	scaffold_3:34235709-34238511	65.2518	67.9739	0.0589618
Ciclev10029765m.g	scaffold_8:1241304-1253939	106.057	110.491	0.0590771
Ciclev10028378m.g	scaffold_8:23083365-23086667	23.9619	24.9646	0.0591443
-	scaffold_7:13033093-13033646	23.9982	25.0044	0.0592552
Ciclev10029317m.g	scaffold_8:12622754-12625953	62.488	65.1157	0.0594256
Ciclev10027705m.g,Ciclev10028758m.g	scaffold_8:3826174-3839610	27.2362	28.3821	0.0594567
Ciclev10026576m.g	scaffold_7:1672067-1674399	16.8423	17.5526	0.0595972
Ciclev10018965m.g	scaffold_3:41636615-41671116	65.0889	67.8354	0.0596281
Ciclev10003981m.g	scaffold_5:32201648-32202966	0.6811	0.709845	0.0596371
Ciclev10031204m.g	scaffold_4:1029899-1034510	97.041	101.149	0.0598184
Ciclev10026112m.g	scaffold_7:5865285-5867488	1.29774	1.35278	0.0599275
Ciclev10001232m.g	scaffold_5:40268947-40273949	9.62788	10.0364	0.0599583
Ciclev10013484m.g	scaffold_6:16233036-16238819	13.7585	14.3436	0.0600848
Ciclev10000979m.g	scaffold_5:35217312-35224175	15.9486	16.627	0.0600947
Ciclev10019752m.g	scaffold_3:36601874-36608003	14.7099	15.3371	0.0602365
Ciclev10026111m.g	scaffold_7:5678714-5681934	29.5802	30.8425	0.0602851
Ciclev10018927m.g	scaffold_3:41750448-41756882	39.2383	40.9165	0.0604211
Ciclev10032114m.g	scaffold_4:12587562-12592318	42.7606	44.5918	0.0604984
Ciclev10008851m.g	scaffold_1:4747929-4750674	3.96244	4.13226	0.0605447

Ciclev10024301m.g	scaffold_3:16286520-16294775	34.6569	36.1518	0.0609235
Ciclev10020481m.g	scaffold_3:28402146-28407834	63.891	66.6501	0.0609924
Ciclev10005966m.g	scaffold_9:23200353-23205342	9.84455	10.27	0.0610425
Ciclev10030993m.g	scaffold_4:1099413-1103825	6.19872	6.46664	0.0610476
Ciclev10019721m.g,Ciclev10020572m.g	scaffold_3:23193053-23204222	12.0849	12.6077	0.0611006
Ciclev10032308m.g	scaffold_4:947942-949892	227.228	237.06	0.061111
Ciclev10001095m.g	scaffold_5:25856737-25859047	16.3831	17.0955	0.0614046
Ciclev10000022m.g	scaffold_5:30707092-30725682	8.47892	8.84848	0.0615496
Ciclev10013772m.g	scaffold_6:3799763-3804403	143.297	149.547	0.0615978
Ciclev10011680m.g	scaffold_6:25039409-25045011	31.2337	32.597	0.0616364
Ciclev10011496m.g	scaffold_6:11133991-11144370	6.93564	7.23879	0.0617202
Ciclev10011900m.g	scaffold_6:18798767-18804051	13.567	14.1639	0.0621249
Ciclev10005293m.g	scaffold_9:2946300-2950297	27.3857	28.5911	0.0621452
Ciclev10025572m.g	scaffold_7:4557429-4559395	101.818	106.304	0.0621949
Ciclev10012815m.g	scaffold_6:15398059-15399018	56.8786	59.3891	0.062312
Ciclev10008577m.g	scaffold_1:4995947-5000076	128.352	134.023	0.0623666
Ciclev10002467m.g	scaffold_5:39446393-39448356	44.1214	46.0726	0.0624288
Ciclev10019092m.g	scaffold_3:7399932-7411047	12.8065	13.3732	0.0624717
Ciclev10011454m.g	scaffold_6:6811092-6823212	2.49088	2.60111	0.0624755
Ciclev10012115m.g	scaffold_6:22767248-22771845	14.8958	15.5568	0.0626404
Ciclev10007902m.g	scaffold_1:915402-918943	9.16329	9.57134	0.062855
Ciclev10002753m.g	scaffold_5:39289632-39299263	76.6429	80.0582	0.0628968
Ciclev10002010m.g	scaffold_5:35827903-35830008	43.6393	45.584	0.0629017
Ciclev10021391m.g	scaffold_3:47337005-47340450	34.1348	35.6585	0.0630021
Ciclev10000670m.g	scaffold_5:8440415-8447137	47.9823	50.1292	0.063148
Ciclev10012322m.g	scaffold_6:8621438-8630049	34.8802	36.4418	0.0631851
Ciclev10000383m.g	scaffold_5:43053903-43057074	7.4306	7.76341	0.0632121
-	scaffold_5:30165628-30170491	46.797	48.902	0.0634801
Ciclev10015496m.g	scaffold_2:5008442-5011807	37.857	39.5605	0.0634989
Ciclev10020918m.g	scaffold_3:50609652-50612344	12.2699	12.822	0.0635014
Ciclev10001457m.g	scaffold_5:42474627-42478305	68.0372	71.1139	0.0638085
Ciclev10012586m.g	scaffold_6:23079725-23083315	78.6533	82.2199	0.0639812
Ciclev10000790m.g,Ciclev10001057m.g	scaffold_5:35872808-35892772	17.5191	18.3137	0.0639916
Ciclev10029223m.g,Ciclev10030417m.g	scaffold_8:24591408-24594117	83.7092	87.5106	0.0640707
Ciclev10005918m.g	scaffold_9:5767144-5770193	36.907	38.587	0.0642225
Ciclev10001037m.g	scaffold_5:37117935-37123920	54.8896	57.3896	0.0642553
Ciclev10017644m.g	scaffold_2:8042357-8047411	123.295	128.92	0.0643618
Ciclev10001427m.g	scaffold_5:23710056-23715863	157.513	164.729	0.0646207
Ciclev10029998m.g	scaffold_8:7698015-7862701	29.5052	30.8576	0.0646555
Ciclev10025381m.g	scaffold_7:1228951-1232906	15.6804	16.3995	0.0646875
Ciclev10024593m.g	scaffold_3:49979609-49981565	2.87751	3.00963	0.0647656
Ciclev10020691m.g	scaffold_3:42149295-42153979	21.8788	22.8837	0.0647881
Ciclev10004460m.g	scaffold_9:3358260-3365948	78.0988	81.6899	0.0648568
Ciclev10012279m.g	scaffold_6:21657656-21660572	165.48	173.096	0.0649148
Ciclev10021129m.g	scaffold_3:41404066-41408588	84.3243	88.2069	0.0649418
Ciclev10015251m.g	scaffold_2:36237625-36240536	34.335	35.9202	0.0651128
Ciclev10015344m.g	scaffold_2:29798615-29804531	10.8071	11.3071	0.0652513
Ciclev10031723m.g	scaffold_4:115361-118025	0.947935	0.991915	0.0654275
Ciclev10018290m.g	scaffold_2:23751802-23755738	40.0914	41.9604	0.0657342
Ciclev10003364m.g	scaffold_5:42992090-42995727	16.5694	17.3424	0.0657758
Ciclev10031184m.g	scaffold_4:23054480-23060623	0.873022	0.913825	0.0659
Ciclev10000582m.g	scaffold_5:39774139-39791254	13.0808	13.6949	0.0661856
Ciclev10020155m.g	scaffold_3:45604805-45609651	28.7283	30.0828	0.0664633

Ciclev10032208m.g	scaffold_4:3521579-3524846	31.3158	32.7927	0.0664811
Ciclev10006582m.g	scaffold_9:29169120-29173419	8.37584	8.77092	0.0664937
Ciclev10010926m.g	scaffold_6:19105138-19113780	12.363	12.9465	0.066532
Ciclev10018782m.g	scaffold_3:28065477-28341901	19.8481	20.7852	0.0665511
Ciclev10032575m.g	scaffold_4:14113371-14114799	2.65398	2.77946	0.0666472
Ciclev10018833m.g	scaffold_3:7163696-7167661	9.69022	10.1504	0.0669389
Ciclev10012134m.g	scaffold_6:20319676-20323198	62.4956	65.4716	0.067114
Ciclev10015473m.g	scaffold_2:26600401-26606954	12.1926	12.7735	0.0671389
Ciclev10010958m.g	scaffold_6:25248952-25255062	48.6178	50.936	0.0671986
Ciclev10021281m.g,Ciclev10021283m.g	scaffold_3:8778585-8785599	13.7055	14.3596	0.067265
Ciclev10021737m.g	scaffold_3:2914294-2917040	9.79945	10.2674	0.0672933
Ciclev10027400m.g	scaffold_7:21024076-21029637	29.2174	30.6207	0.0676787
Ciclev10013870m.g	scaffold_6:1194238-1195432	5.23858	5.49147	0.0680155
Ciclev10028645m.g	scaffold_8:966123-968846	72.3637	75.8577	0.0680293
Ciclev10026680m.g	scaffold_7:8750949-8753447	0.796746	0.835326	0.0682194
Ciclev10006642m.g	scaffold_9:7561669-7565819	6.1686	6.4679	0.0683545
Ciclev10005064m.g	scaffold_9:13042914-13044996	8.58479	9.00156	0.0683923
Ciclev10015746m.g	scaffold_2:6905952-6909698	26.5812	27.878	0.0687205
Ciclev10028540m.g	scaffold_8:9731065-9737070	15.9061	16.6833	0.0688216
Ciclev10022660m.g	scaffold_3:38023393-38025262	17.6014	18.4616	0.0688345
Ciclev10033278m.g	scaffold_4:19899177-19899815	1795.61	1883.45	0.0689088
Ciclev10019433m.g	scaffold_3:38002204-38008464	3.09327	3.24471	0.0689566
Ciclev10022209m.g	scaffold_3:42404981-42407135	43.9198	46.0838	0.0693889
Ciclev10014587m.g	scaffold_2:32257052-32262117	7.91913	8.30933	0.0693896
Ciclev10027957m.g	scaffold_8:23719916-23722064	0.351592	0.368919	0.069405
Ciclev10015880m.g	scaffold_2:169712-173304	87.8354	92.1657	0.0694275
Ciclev10022867m.g	scaffold_3:5857184-5860112	116.223	121.954	0.069443
Ciclev10007683m.g	scaffold_1:4171157-4179810	43.9018	46.0671	0.0694565
Ciclev10004179m.g	scaffold_9:30504368-30511236	13.2922	13.9478	0.0694598
-	scaffold_6:24112828-24113651	14.1231	14.8198	0.0694737
Ciclev10014944m.g	scaffold_2:26011585-26016025	33.1112	34.7494	0.0696705
Ciclev10016148m.g	scaffold_2:9088938-9091599	63.1211	66.2549	0.0699046
Ciclev10007538m.g	scaffold_1:22046981-22052791	3.79688	3.98568	0.070011
Ciclev10012920m.g	scaffold_6:11607932-11614367	17.4621	18.3309	0.0700558
Ciclev10014614m.g	scaffold_2:32468277-32472724	12.1902	12.7983	0.0702229
Ciclev10018993m.g	scaffold_3:43557748-43564140	7.62337	8.00409	0.0703092
Ciclev10026191m.g,Ciclev10027411m.g	scaffold_7:7661635-7664868	15.1303	15.887	0.070406
Ciclev10031901m.g	scaffold_4:1828099-1836003	29.3197	30.7896	0.0705734
Ciclev10002550m.g	scaffold_5:27147111-27151050	19.2144	20.1788	0.0706551
Ciclev10013551m.g	scaffold_6:18758809-18764392	86.1526	90.4927	0.0709076
Ciclev10032728m.g	scaffold_4:24680366-24682650	35.5887	37.3834	0.0709749
Ciclev10014926m.g	scaffold_2:22101618-22105301	145.655	153.005	0.0710205
Ciclev10018943m.g	scaffold_3:6076948-6080875	297.819	312.905	0.0712924
Ciclev10013364m.g	scaffold_6:2101266-2105292	16.4669	17.3029	0.0714412
Ciclev10031129m.g	scaffold_4:24731582-24735863	39.9837	42.0135	0.0714422
-	scaffold_5:20149554-20149897	9.02533	9.48471	0.0716249
Ciclev10001478m.g	scaffold_5:32542379-32543973	59.1667	62.1791	0.0716444
Ciclev10014694m.g	scaffold_2:8295124-8306258	8.73882	9.18621	0.0720315
Ciclev10019329m.g	scaffold_3:4388732-4393496	34.4219	36.1846	0.07205
Ciclev10008870m.g	scaffold_1:14993045-14997677	7.5248	7.91032	0.0720829
Ciclev10008518m.g	scaffold_1:3419804-3425580	55.6921	58.5463	0.0721047
Ciclev10020938m.g	scaffold_3:20672102-20677318	86.157	90.5856	0.0723137
Ciclev10032099m.g	scaffold_4:16107198-16111155	30.0511	31.5973	0.0723868
Ciclev10024836m.g	scaffold_7:11686788-11771145	0.572979	0.602528	0.0725467

Ciclev10018497m.g	scaffold_3:3329527-3337905	14.2574	14.9932	0.0725986
-	scaffold_1:22577554-22577932	29.9433	31.4887	0.0726007
Ciclev10019467m.g	scaffold_3:8330156-8337179	8.10922	8.52777	0.072605
Ciclev10011668m.g	scaffold_6:17867901-17874268	26.478	27.8456	0.0726564
Ciclev10018464m.g	scaffold_3:11498991-11509198	4.05402	4.26347	0.0726735
Ciclev10027042m.g	scaffold_7:4226475-4231421	11.3076	11.8931	0.0728224
Ciclev10016848m.g	scaffold_2:4672768-4673615	2.26168	2.37885	0.0728742
Ciclev10008027m.g	scaffold_1:22642659-22648078	178.439	187.702	0.0730112
-	scaffold_5:10023590-10123855	60.533	63.6792	0.0730998
Ciclev10016786m.g	scaffold_2:35723131-35725455	4.21341	4.43268	0.0731905
Ciclev10016922m.g,Ciclev10017386m.g	scaffold_2:3035920-3045886	59.1266	62.2045	0.0732125
Ciclev10018196m.g	scaffold_2:26079556-26082625	4.48902	4.72272	0.073217
Ciclev10018352m.g	scaffold_2:13528033-13529425	1.58862	1.67142	0.0733016
Ciclev10008327m.g	scaffold_1:5358453-5364819	40.6965	42.8192	0.073356
Ciclev10012018m.g	scaffold_6:23805068-23807918	0.776544	0.81709	0.0734277
Ciclev10010680m.g	scaffold_1:199017-201722	0.543114	0.571507	0.0735167
Ciclev10000335m.g	scaffold_5:42113620-42120391	8.7376	9.1948	0.0735806
Ciclev10015864m.g	scaffold_2:15144187-15148000	4.80361	5.05513	0.0736292
Ciclev10028138m.g	scaffold_8:21415877-21420112	56.6376	59.6052	0.073677
Ciclev10033004m.g	scaffold_4:9453506-9456798	35.5348	37.3993	0.0737779
Ciclev10032681m.g	scaffold_4:20655150-20658347	23.8928	25.1466	0.0737913
Ciclev10019185m.g	scaffold_3:34303789-34309857	13.1002	13.7888	0.073909
Ciclev10004349m.g	scaffold_9:25386634-25390630	5.84466	6.1522	0.0739846
Ciclev10021440m.g	scaffold_3:4934654-4938469	18.6637	19.6469	0.074063
-	scaffold_8:18128912-18129223	33.715	35.4918	0.0740925
Ciclev10007885m.g	scaffold_1:16685842-16695676	13.5849	14.3022	0.0742348
Ciclev10023388m.g	scaffold_3:18881068-18884529	142.871	150.427	0.0743557
Ciclev10021169m.g	scaffold_3:8173863-8179112	87.6091	92.2687	0.0747605
Ciclev10030614m.g	scaffold_4:8589766-8593169	7.3404	7.73176	0.0749383
Ciclev10012663m.g	scaffold_6:17670231-17671170	2.14767	2.26229	0.0750082
Ciclev10013022m.g	scaffold_6:20327021-20329307	185.909	195.849	0.0751475
Ciclev10000781m.g	scaffold_5:31069236-31075092	48.5096	51.1099	0.0753334
Ciclev10019906m.g	scaffold_3:24893550-24898675	64.0064	67.4413	0.075416
Ciclev10019394m.g	scaffold_3:3551541-3555978	25.2674	26.6242	0.0754576
Ciclev10031478m.g	scaffold_4:12180728-12187148	13.2544	13.9663	0.075482
Ciclev10021901m.g	scaffold_3:4903591-4905871	133.881	141.083	0.0755924
Ciclev10001496m.g	scaffold_5:41948946-41952895	12.3222	12.986	0.075699
Ciclev10000673m.g	scaffold_5:15113861-15117721	30.619	32.2724	0.0758739
Ciclev10029089m.g	scaffold_8:6651050-6656927	33.27	35.074	0.0761818
Ciclev10000082m.g	scaffold_5:30615375-30629437	15.6515	16.5007	0.0762246
Ciclev10026172m.g	scaffold_7:17646726-17649424	10.1324	10.6828	0.0763117
Ciclev10015432m.g	scaffold_2:35880674-35883339	76.4795	80.6443	0.0765001
Ciclev10030490m.g	scaffold_4:17040658-17047269	2.93143	3.09124	0.0765832
Ciclev10011054m.g	scaffold_6:13536317-13546839	15.185	16.0131	0.0766109
-	scaffold_5:28089956-28090327	18.0987	19.0872	0.0767236
Ciclev10002385m.g	scaffold_5:36892920-36894344	0.587695	0.619829	0.0768028
Ciclev10004987m.g	scaffold_9:28071467-28075483	12.476	13.1619	0.077216
Ciclev10001685m.g	scaffold_5:36095843-36099890	158.066	166.783	0.0774463
Ciclev10013738m.g	scaffold_6:17719185-17720880	3.01019	3.17627	0.0774798
Ciclev10029640m.g	scaffold_8:23175892-23176688	138.911	146.58	0.0775266
Ciclev10003619m.g	scaffold_5:38535287-38544023	26.4918	27.9566	0.0776409
Ciclev10011376m.g	scaffold_6:9681476-9686509	55.6435	58.7213	0.0776711
Ciclev10019194m.g	scaffold_3:3925206-3929174	9.10925	9.61557	0.0780398
Ciclev10028872m.g	scaffold_8:23299885-23301690	7.86217	8.30079	0.078322
Ciclev10012047m.g	scaffold_6:21912437-21917736	262.52	277.168	0.0783348

Ciclev10002101m.g	scaffold_5:41439979-41441315	151.84	160.314	0.0783491
Ciclev10031036m.g	scaffold_4:5267484-5269955	1.0206	1.07757	0.0783663
Ciclev10019033m.g	scaffold_3:50559204-50563745	7.18609	7.58733	0.078386
Ciclev10028759m.g	scaffold_8:44040-45303	20.8132	21.977	0.0784929
Ciclev10002626m.g	scaffold_5:39143848-39144751	26.5826	28.0708	0.0785873
Ciclev10006134m.g	scaffold_9:29407887-29411173	16.2448	17.1548	0.0786351
Ciclev10014009m.g	scaffold_2:21223443-21235946	16.5529	17.4824	0.0788224
Ciclev10010909m.g	scaffold_6:18281864-18292648	0.199773	0.210994	0.0788388
Ciclev10008337m.g	scaffold_1:21300057-21310677	16.7845	17.7274	0.0788511
Ciclev10014519m.g	scaffold_2:29740181-29745654	2.02929	2.14347	0.0789752
Ciclev10000065m.g	scaffold_5:33016989-33027934	12.879	13.6046	0.0790642
Ciclev10021222m.g	scaffold_3:6334403-6339425	8.20607	8.66925	0.0792163
Ciclev10031853m.g	scaffold_4:7407833-7414015	68.9972	72.8929	0.0792401
Ciclev10021126m.g	scaffold_3:30926422-30928874	19.1216	20.2015	0.0792596
Ciclev10016413m.g	scaffold_2:33298784-33302612	32.1065	33.9208	0.0793039
Ciclev10022229m.g	scaffold_3:4004110-4008139	19.8478	20.9701	0.0793492
Ciclev10019821m.g	scaffold_3:3286553-3291773	21.058	22.2493	0.0793967
Ciclev10003006m.g	scaffold_5:33302588-33304334	365.24	385.918	0.0794503
Ciclev10012725m.g	scaffold_6:12998054-13001834	122.229	129.16	0.0795816
Ciclev10016628m.g	scaffold_2:7349634-7351606	69.7065	73.6612	0.0796114
Ciclev10009284m.g	scaffold_1:28075981-28077060	86.9294	91.8942	0.0801298
Ciclev10009050m.g	scaffold_1:18912039-18913817	0.640078	0.676649	0.0801609
Ciclev10011182m.g	scaffold_6:15519381-15529428	40.1347	42.4309	0.0802659
Ciclev10013042m.g	scaffold_6:21714715-21717652	20.6482	21.8297	0.0802765
Ciclev10020473m.g	scaffold_3:49880154-49886203	25.9033	27.3861	0.0803104
Ciclev10022594m.g	scaffold_3:16957426-16959735	417.663	441.58	0.0803379
Ciclev10029508m.g	scaffold_8:2870394-2871752	55.1297	58.2871	0.0803473
Ciclev10005346m.g	scaffold_9:7521997-7530730	25.8467	27.3282	0.0804088
Ciclev10012925m.g	scaffold_6:12279554-12281680	11.6464	12.3147	0.0805041
Ciclev10027667m.g,Ciclev10029869m.g	scaffold_8:8103136-8174407	6.35982	6.72496	0.0805413
Ciclev10028864m.g	scaffold_8:16590554-16594539	32.7908	34.6749	0.0806007
Ciclev10007431m.g	scaffold_1:26143661-26156067	8.84108	9.34909	0.0806035
Ciclev10017963m.g	scaffold_2:35633992-35637155	61.9632	65.5267	0.0806709
Ciclev10002171m.g	scaffold_5:14813703-14820676	12.3576	13.0686	0.0806959
Ciclev10019641m.g	scaffold_3:4925003-4928652	11.5544	12.2193	0.0807188
Ciclev10032810m.g	scaffold_4:24300925-24302703	107.953	114.171	0.0807899
Ciclev10011241m.g	scaffold_6:8228103-8232256	19.7831	20.9231	0.0808227
Ciclev10012084m.g	scaffold_6:24631826-24635337	95.1252	100.609	0.0808665
Ciclev10020315m.g	scaffold_3:44874251-44879208	196.341	207.663	0.0808812
Ciclev10018581m.g	scaffold_3:102152-115015	17.4275	18.4335	0.0809672
Ciclev10024582m.g	scaffold_3:20565833-20571082	14.0316	14.842	0.0810075
Ciclev10023768m.g	scaffold_3:296343-301419	59.2605	62.693	0.0812332
Ciclev10021545m.g	scaffold_3:6009155-6013920	108.262	114.535	0.0812654
Ciclev10007258m.g	scaffold_1:28555336-28565018	14.9468	15.8134	0.0813119
Ciclev10032170m.g	scaffold_4:3417931-3421514	90.9169	96.2042	0.0815515
Ciclev10030944m.g	scaffold_4:24464796-24471185	13.2543	14.0255	0.0815985
Ciclev10031991m.g	scaffold_4:15006423-15014271	12.2113	12.922	0.0816201
Ciclev10007508m.g	scaffold_1:1732229-1739391	9.87702	10.4535	0.0818346
Ciclev10021171m.g	scaffold_3:9080654-9087568	56.9498	60.2812	0.082017
Ciclev10019144m.g	scaffold_3:51010547-51016105	31.0451	32.8675	0.082295
Ciclev10018809m.g	scaffold_3:43529898-43537226	14.9963	15.8777	0.0823918
Ciclev10011051m.g	scaffold_6:19979951-19983345	1.74626	1.84907	0.0825308
Ciclev10004735m.g	scaffold_9:1769645-1774336	14.0233	14.8489	0.0825321
Ciclev10028181m.g	scaffold_8:6790356-6792194	0.553855	0.586481	0.0825761
Ciclev10000682m.g	scaffold_5:39700146-39702942	22.4467	23.7721	0.0827632

Ciclev10005716m.g	scaffold_9:26634597-26636952	8.20733	8.69203	0.0827807
Ciclev10012146m.g	scaffold_6:23130453-23133783	4.52389	4.79115	0.0828077
Ciclev10032327m.g,Ciclev10032713m.g	scaffold_4:22680905-22685514	173.347	183.639	0.0832132
Ciclev10020441m.g	scaffold_3:3350500-3352652	15.23	16.1366	0.0834273
Ciclev10022306m.g	scaffold_3:898448-901255	22.3665	23.7008	0.0835976
Ciclev10020003m.g	scaffold_3:44670368-44674788	3.12228	3.30861	0.0836229
Ciclev10016873m.g	scaffold_2:34346969-34350783	19.2614	20.4121	0.0837084
Ciclev10003868m.g	scaffold_5:19120977-19131912	9.30802	9.86434	0.0837474
Ciclev10008777m.g	scaffold_1:21325879-21329103	40.3963	42.8111	0.083763
Ciclev10030413m.g	scaffold_8:23711149-23713165	1.05036	1.11318	0.0838103
Ciclev10030502m.g	scaffold_4:24160707-24166121	6.77862	7.18412	0.0838198
Ciclev10007837m.g	scaffold_1:4109716-4112564	45.1486	47.8509	0.0838655
Ciclev10019287m.g	scaffold_3:563473-567226	50.1316	53.1381	0.0840266
Ciclev10023445m.g	scaffold_3:25248366-25288105	40.3253	42.7506	0.0842591
Ciclev10031752m.g	scaffold_4:23538778-23544461	25.4586	26.9922	0.0843848
Ciclev10032301m.g	scaffold_4:1455584-1458644	4.13237	4.38159	0.0844852
Ciclev10014018m.g	scaffold_2:8129595-8151433	12.3209	13.064	0.0844961
Ciclev10011855m.g	scaffold_6:15346134-15348332	331.656	351.676	0.08456
Ciclev10029743m.g	scaffold_8:17291958-17467945	6.39444	6.78074	0.0846255
Ciclev10021461m.g	scaffold_3:50360148-50363184	20.4804	21.7194	0.0847384
Ciclev10016883m.g	scaffold_2:27869738-27871549	72.8109	77.2195	0.0848094
Ciclev10017747m.g	scaffold_2:27792848-27804214	32.4341	34.3985	0.0848326
Ciclev10004238m.g	scaffold_9:18367739-18371241	6.08411	6.45309	0.0849425
Ciclev10033538m.g,Ciclev10033946m.g	scaffold_4:20724395-20736958	69.0272	73.2226	0.0851241
Ciclev10005448m.g	scaffold_9:28494275-28496277	33.9668	36.033	0.0851959
Ciclev10020185m.g	scaffold_3:21501940-21505994	69.4542	73.6796	0.0852049
Ciclev10019925m.g	scaffold_3:40937724-40942854	103.775	110.089	0.0852084
Ciclev10017136m.g	scaffold_2:23837167-23840360	113.398	120.311	0.0853762
Ciclev10021080m.g	scaffold_3:7016830-7019863	11.2362	11.9216	0.0854329
Ciclev10010938m.g	scaffold_6:18390179-18394551	2.30967	2.45085	0.0855938
Ciclev10021013m.g	scaffold_3:20571853-20576375	38.6521	41.0159	0.0856376
Ciclev10002167m.g	scaffold_5:34516142-34519012	13.8649	14.7131	0.0856643
Ciclev10020733m.g	scaffold_3:49445680-49449255	25.0282	26.5593	0.0856651
Ciclev10008732m.g	scaffold_1:7016412-7020773	95.1147	100.934	0.0856686
Ciclev10024969m.g	scaffold_7:6080409-6084393	24.8016	26.3194	0.0856935
Ciclev10010916m.g	scaffold_6:1938069-1945512	14.6247	15.5199	0.0857093
Ciclev10022134m.g	scaffold_3:224710-228632	48.9487	51.9461	0.0857445
Ciclev10002764m.g	scaffold_5:39012491-39014490	347.364	368.653	0.0858129
Ciclev10005598m.g	scaffold_9:29764963-29766145	98.5028	104.543	0.0858655
Ciclev10031419m.g	scaffold_4:24303094-24304894	50.0877	53.1659	0.0860452
Ciclev10029070m.g	scaffold_8:24143987-24146294	8.36663	8.88094	0.0860652
Ciclev10001784m.g	scaffold_5:34501166-34506609	54.7638	58.1306	0.0860762
Ciclev10020925m.g	scaffold_3:41067674-41229006	34.3831	36.5005	0.0862172
Ciclev10010961m.g	scaffold_6:19778324-19787052	22.637	24.032	0.0862761
Ciclev10018929m.g	scaffold_3:39708465-39714707	12.2553	13.0114	0.0863768
Ciclev10014137m.g	scaffold_2:26696361-26703280	7.74217	8.22111	0.0865944
Ciclev10001867m.g	scaffold_5:33801544-33802689	19.9162	21.149	0.0866456
Ciclev10026093m.g	scaffold_7:19678971-19684711	26.2481	27.8794	0.0869896
Ciclev10027686m.g	scaffold_8:22840532-22845718	11.8715	12.6097	0.0870397
Ciclev10020037m.g	scaffold_3:48191898-48194435	41.8736	44.4815	0.0871635
Ciclev10021552m.g	scaffold_3:47240811-47243207	16.6439	17.6818	0.0872731
Ciclev10021310m.g	scaffold_3:6507373-6510323	25.6352	27.2363	0.0874056
Ciclev10027829m.g	scaffold_8:22927774-22934887	9.26419	9.84411	0.0875969
Ciclev10027596m.g	scaffold_7:10756095-10758741	2.6717	2.83896	0.0876035

Ciclev10005464m.g	scaffold_9:25519002-25523556	21.5408	22.8898	0.0876341
Ciclev10005059m.g,Ciclev10006893m.g	scaffold_9:17075597-17085320	11.3973	12.1121	0.0877639
Ciclev10025530m.g	scaffold_7:1753132-1754952	2.0022	2.12795	0.0878788
Ciclev10027934m.g	scaffold_8:6929094-6933590	74.6637	79.365	0.0880947
Ciclev10015119m.g	scaffold_2:29370559-29376244	38.3394	40.76	0.0883273
Ciclev10020270m.g	scaffold_3:24831948-24837740	45.2944	48.1558	0.0883755
Ciclev10000255m.g	scaffold_5:24360294-24367588	16.6247	17.675	0.0883856
Ciclev10016738m.g	scaffold_2:35980340-35983262	77.4033	82.3017	0.0885264
Ciclev10004196m.g	scaffold_9:8659717-8664909	7.69542	8.18355	0.0887268
Ciclev10015063m.g	scaffold_2:33746687-33749902	4.96446	5.27952	0.0887712
Ciclev10000614m.g	scaffold_5:42888592-42893193	6.1597	6.55131	0.0889231
Ciclev10001868m.g,Ciclev10003684m.g	scaffold_5:32266175-32290730	14.8579	15.8037	0.0890312
Ciclev10020284m.g	scaffold_3:10940103-10948667	87.928	93.5329	0.0891508
Ciclev10028599m.g	scaffold_8:9656864-9660001	20.9124	22.2468	0.0892333
Ciclev10015005m.g	scaffold_2:10424778-10472483	79.1955	84.2536	0.0893187
Ciclev10010927m.g	scaffold_6:17678074-17686312	8.42903	8.96748	0.0893357
Ciclev10020468m.g	scaffold_3:45314015-45317065	20.0112	21.2907	0.0894182
Ciclev10009620m.g	scaffold_1:3332616-3333255	9.19241	9.78205	0.0896946
Ciclev10012495m.g	scaffold_6:23314956-23318185	175.701	186.977	0.0897326
Ciclev10016356m.g	scaffold_2:35018720-35021044	11.6235	12.3696	0.0897535
Ciclev10009295m.g	scaffold_1:22628770-22631628	23.0324	24.5118	0.0898117
Ciclev10005057m.g	scaffold_9:2243081-2249573	11.47	12.2075	0.0898939
Ciclev10015030m.g	scaffold_2:29492431-29498475	18.318	19.4958	0.0899031
Ciclev10008333m.g	scaffold_1:25028239-25030911	3.18883	3.39426	0.090072
Ciclev10012800m.g	scaffold_6:5264027-5266061	39.0475	41.5658	0.0901658
Ciclev10025760m.g	scaffold_7:1413738-1419214	41.9629	44.6714	0.0902382
Ciclev10020405m.g	scaffold_3:3201205-3204377	74.9346	79.7797	0.0903896
Ciclev10008478m.g	scaffold_1:24110289-24115326	30.8882	32.8885	0.0905289
Ciclev10016450m.g	scaffold_2:24690260-24692734	21.8292	23.2521	0.0911015
Ciclev10020647m.g	scaffold_3:18612962-18618580	10.0928	10.751	0.0911465
Ciclev10009012m.g	scaffold_1:22885750-22890756	11.4211	12.167	0.0912792
-	scaffold_2:29377636-29378097	13.349	14.221	0.0912894
Ciclev10022726m.g	scaffold_3:8926863-8933381	2.51372	2.67795	0.0913049
Ciclev10025460m.g	scaffold_7:4193146-4197960	5.00918	5.337	0.0914561
Ciclev10007512m.g	scaffold_1:24924137-24934718	13.4384	14.319	0.0915718
Ciclev10009459m.g	scaffold_1:11258634-11261886	14.4023	15.3486	0.091811
-	scaffold_7:19329779-19331382	4.43918	4.73123	0.0919215
Ciclev10001031m.g	scaffold_5:41356898-41361583	7.65038	8.15417	0.0920074
Ciclev10015532m.g	scaffold_2:13928276-13929910	5.90833	6.29795	0.092131
Ciclev10031746m.g	scaffold_4:18709283-18713551	4.0245	4.28995	0.0921497
Ciclev10025351m.g	scaffold_7:25041-32265	24.2208	25.8186	0.0921651
Ciclev10000928m.g,Ciclev10000944m.g,Ciclev10001071m.g	scaffold_5:25832875-25852629	40.2431	42.9029	0.0923364
Ciclev10025897m.g	scaffold_7:20841941-20846077	25.752	27.4548	0.0923731
Ciclev10002586m.g	scaffold_5:5078202-5082511	9.49839	10.1271	0.0924715
Ciclev10017115m.g	scaffold_2:3543305-3544895	292.501	311.866	0.0924848
-	scaffold_5:24324256-24327392	3.72468	3.97143	0.0925437
Ciclev10014122m.g	scaffold_2:30911110-30921615	13.917	14.8398	0.09262
Ciclev10030891m.g	scaffold_4:5344545-5351730	41.6096	44.3698	0.0926611
Ciclev10021065m.g	scaffold_3:42136500-42139444	14.419	15.3757	0.0926779
-	scaffold_5:1987153-1987691	6.18778	6.5985	0.0927146
Ciclev10011438m.g	scaffold_6:22145196-22149851	40.9154	43.6369	0.0929047
Ciclev10029551m.g	scaffold_8:17524834-17526697	634.123	676.314	0.0929311

Ciclev10014627m.g	scaffold_2:3539241-3541417	1.49103	1.59026	0.0929457
Ciclev10004486m.g	scaffold_9:13576938-13585685	31.4013	33.4938	0.0930715
Ciclev10029463m.g	scaffold_8:24218600-24220305	15.8401	16.8962	0.0931192
Ciclev10012766m.g	scaffold_6:14892938-14897320	74.0694	79.009	0.0931396
Ciclev10002495m.g	scaffold_5:25827406-25830697	14.2432	15.1966	0.0934798
Ciclev10014596m.g	scaffold_2:32716142-32719988	25.7484	27.4725	0.0935062
Ciclev10000370m.g	scaffold_5:35817026-35826170	15.4232	16.4568	0.0935835
Ciclev10031172m.g	scaffold_4:24357411-24361117	10.8492	11.5763	0.0935909
Ciclev10032825m.g	scaffold_4:17763595-17766370	88.097	94.0246	0.0939459
Ciclev10007291m.g	scaffold_1:5571474-5580690	2.09287	2.23395	0.094113
Ciclev10020573m.g	scaffold_3:29436972-29441324	1.05099	1.12203	0.0943589
Ciclev10018676m.g	scaffold_3:39472055-39480575	15.7137	16.7767	0.0944355
Ciclev10004328m.g	scaffold_9:22283731-22291092	7.77946	8.30584	0.094455
Ciclev10015123m.g	scaffold_2:13820781-13826499	24.2799	25.9236	0.0945078
Ciclev10019670m.g	scaffold_3:39639738-39649799	25.6742	27.4167	0.0947389
Ciclev10031248m.g	scaffold_4:23152321-23156143	58.2458	62.2021	0.0948081
Ciclev10009133m.g	scaffold_1:17185031-17189281	38.1188	40.7192	0.0952064
Ciclev10015755m.g	scaffold_2:14429308-14434365	42.447	45.3427	0.0952078
Ciclev10009013m.g	scaffold_1:24584864-24587207	31.8783	34.0534	0.0952255
Ciclev10030845m.g	scaffold_4:21507307-21516393	5.7415	6.13406	0.0954133
Ciclev10022933m.g	scaffold_3:21082203-21084675	27.1436	28.9999	0.095437
Ciclev10014675m.g	scaffold_2:35662163-35666978	13.3579	14.2721	0.0955024
Ciclev10031132m.g	scaffold_4:20675446-20680247	5.18121	5.53592	0.0955326
Ciclev10030475m.g	scaffold_4:17178738-17195078	9.27743	9.91293	0.0955868
Ciclev10000876m.g	scaffold_5:40154568-40160983	11.3344	12.1114	0.0956561
Ciclev10008561m.g	scaffold_1:22512435-22515454	4.37894	4.67929	0.0957059
Ciclev10026433m.g	scaffold_7:1507803-1510299	0.551839	0.589727	0.0957988
Ciclev10026672m.g	scaffold_7:16270393-16273383	35.7543	38.2105	0.0958518
Ciclev10004648m.g	scaffold_9:3238891-3241940	16.426	17.5576	0.0961077
Ciclev10021454m.g	scaffold_3:2627068-2629098	259.04	276.899	0.0961836
Ciclev10004378m.g	scaffold_9:15864623-15934825	17.1821	18.3693	0.0963871
Ciclev10011177m.g	scaffold_6:14911433-14917683	14.327	15.3173	0.0964279
Ciclev10011322m.g	scaffold_6:19252438-19260599	27.601	29.5092	0.0964435
Ciclev10001396m.g	scaffold_5:40850791-40856282	21.9314	23.4492	0.096536
Ciclev10004217m.g	scaffold_9:350493-358264	6.96578	7.44834	0.0966326
Ciclev10031866m.g	scaffold_4:10244322-10249406	311.09	332.653	0.0966885
Ciclev10015175m.g	scaffold_2:31333047-31339194	89.175	95.3565	0.0966914
Ciclev10030710m.g	scaffold_4:19578355-19591163	10.2646	10.9768	0.0967879
Ciclev10025372m.g	scaffold_7:4882519-4888243	1238.3	1324.34	0.0969138
Ciclev10014936m.g	scaffold_2:6846909-6850951	24.8805	26.6145	0.0971987
Ciclev10000412m.g	scaffold_5:24430820-24436636	9.55036	10.2162	0.097236
Ciclev10004506m.g	scaffold_9:10254197-10259673	10.2867	11.0048	0.0973558
Ciclev10023558m.g	scaffold_3:6532592-6534845	0.232172	0.248383	0.0973736
Ciclev10025538m.g	scaffold_7:1689327-1694589	9.26148	9.90868	0.0974507
Ciclev10009334m.g	scaffold_1:19049995-19054408	66.5488	71.2063	0.0975915
Ciclev10028209m.g	scaffold_8:17141881-17148641	44.1841	47.285	0.0978548
Ciclev10009053m.g	scaffold_1:25075621-25080986	20.0482	21.4584	0.098066
Ciclev10010753m.g	scaffold_1:4319754-4320591	16.324	17.4775	0.098509
Ciclev10033529m.g	scaffold_4:2457797-2458868	5.83207	6.24429	0.0985287
Ciclev10020913m.g	scaffold_3:2666074-2667855	41.3961	44.323	0.0985598
Ciclev10021986m.g	scaffold_3:3032786-3036641	18.3726	19.6722	0.098602
Ciclev10021841m.g	scaffold_3:38267540-38272242	14.8779	15.931	0.0986682
Ciclev10005558m.g	scaffold_9:24993974-24995774	11.3313	12.1343	0.0987779
Ciclev10000079m.g	scaffold_5:35687309-35694527	6.54636	7.01059	0.0988421
Ciclev10012670m.g	scaffold_6:2545014-2548583	33.7328	36.1257	0.0988743
Ciclev10025682m.g	scaffold_7:6954032-6955625	0.913926	0.978806	0.0989457

Ciclev10032082m.g	scaffold_4:23858829-23862441	37.7344	40.4167	0.099072
Ciclev10020422m.g	scaffold_3:29979422-29987188	6.18164	6.62119	0.0991008
Ciclev10020708m.g	scaffold_3:3320451-3324500	27.2096	29.1451	0.0991361
-	scaffold_3:2888603-2892868	6.04909	6.47978	0.0992259
Ciclev10029963m.g,Ciclev10030210m.g	scaffold_8:3520446-3525115	17.8773	19.1507	0.0992682
Ciclev10011406m.g	scaffold_6:19241699-19246874	19.8345	21.2486	0.0993593
Ciclev10007893m.g	scaffold_1:2674514-2681844	2.39129	2.56217	0.0995748
Ciclev10033624m.g,Ciclev10033884m.g	scaffold_4:10565450-10571345	37.5954	40.2828	0.0996081
Ciclev10007731m.g	scaffold_1:14089930-14104996	5.45698	5.84712	0.0996236
Ciclev10027878m.g	scaffold_8:23544758-23550076	7.82581	8.38547	0.0996519
Ciclev10004371m.g	scaffold_9:25285257-25288376	4.0297	4.31817	0.0997467
Ciclev10018910m.g	scaffold_3:42775429-42784038	31.3679	33.6205	0.100052
Ciclev10014086m.g	scaffold_2:14017066-14023845	13.0977	14.0426	0.10049
Ciclev10002345m.g	scaffold_5:12174213-12181845	16.0922	17.2543	0.100587
Ciclev10013909m.g	scaffold_6:13074822-13076033	63.1194	67.6839	0.100731
Ciclev10024729m.g	scaffold_7:6208349-6217283	6.42876	6.8939	0.10078
Ciclev10032620m.g	scaffold_4:25573595-25576886	126.46	135.626	0.100955
Ciclev10022115m.g	scaffold_3:42211507-42212945	4.19162	4.49554	0.100989
Ciclev10024626m.g,Ciclev10024671m.g	scaffold_3:1696061-1698775	109.826	117.797	0.101079
Ciclev10004535m.g	scaffold_9:28177909-28179955	4.38294	4.70111	0.101101
Ciclev10019381m.g	scaffold_3:43927967-43935770	9.35884	10.0418	0.101612
Ciclev10020974m.g	scaffold_3:3406566-3412591	17.5193	18.8023	0.101961
Ciclev10022841m.g	scaffold_3:46452892-46453717	1023.59	1098.55	0.101968
Ciclev10014556m.g	scaffold_2:30947327-30949906	6.58512	7.06781	0.102053
Ciclev10020182m.g	scaffold_3:12588721-12594337	9.95499	10.6852	0.102117
Ciclev10001860m.g	scaffold_5:37541298-37543916	15.5593	16.7015	0.102206
Ciclev10026352m.g	scaffold_7:7286297-7291367	77.6457	83.3611	0.102468
Ciclev10018087m.g	scaffold_2:3555024-3559126	0.81288	0.872747	0.102522
Ciclev10007298m.g	scaffold_1:23544822-23550437	3.02836	3.25149	0.102564
Ciclev10021899m.g	scaffold_3:18573256-18576843	158.614	170.312	0.102655
Ciclev10028502m.g	scaffold_8:205986-207578	1.12557	1.20861	0.102695
Ciclev10022767m.g	scaffold_3:50194846-50197125	35.2709	37.8732	0.102699
Ciclev10011952m.g	scaffold_6:12224055-12230012	41.5226	44.5918	0.102881
Ciclev10005228m.g	scaffold_9:29931102-29937516	11.1896	12.0186	0.103109
Ciclev10028257m.g,Ciclev10028268m.g	scaffold_8:9325618-9585469	45.3991	48.7644	0.103162
Ciclev10014466m.g	scaffold_2:9262666-9275068	29.639	31.8375	0.103232
Ciclev10001267m.g	scaffold_5:35225857-35227630	49.7106	53.4019	0.103339
Ciclev10003685m.g	scaffold_5:31469286-31718393	7.6003	8.16482	0.103364
Ciclev10013447m.g	scaffold_6:6694889-6751336	7.5735	8.13627	0.103407
Ciclev10013045m.g	scaffold_6:13547528-13550344	14.4263	15.4999	0.103558
Ciclev10026038m.g	scaffold_7:18568357-18573692	10.0251	10.7715	0.103598
Ciclev10013877m.g	scaffold_6:15246909-15248455	7.64306	8.21235	0.103643
Ciclev10029333m.g	scaffold_8:1703824-1706352	24.5868	26.4185	0.103668
Ciclev10019520m.g	scaffold_3:5601337-5604877	8.91746	9.5822	0.103724
Ciclev10029666m.g	scaffold_8:2403491-2406922	36.0558	38.7443	0.103753
Ciclev10019630m.g	scaffold_3:34312145-34319423	18.2415	19.6077	0.104199
Ciclev10032517m.g	scaffold_4:22672248-22680724	296.96	319.202	0.104202
Ciclev10007196m.g	scaffold_9:173440-176748	37.9697	40.816	0.104287
Ciclev10017364m.g	scaffold_2:28767811-28768582	10.8666	11.6819	0.104368
Ciclev10025176m.g	scaffold_7:1720103-1725002	0.988117	1.06228	0.104404
Ciclev10005002m.g	scaffold_9:28514720-28518575	25.2561	27.1523	0.104441
Ciclev10026303m.g	scaffold_7:2607669-2609433	190.945	205.3	0.104577

Ciclev10019332m.g	scaffold_3:44114718-44121535	11.0298	11.8595	0.104637
Ciclev10009919m.g	scaffold_1:8698642-8701746	76.5291	82.2933	0.104766
Ciclev10009377m.g	scaffold_1:7455491-7457898	174.36	187.546	0.105175
Ciclev10009652m.g	scaffold_1:180431-182328	155.629	167.399	0.105176
Ciclev10026243m.g	scaffold_7:6453881-6456945	32.1774	34.6108	0.105177
Ciclev10031569m.g	scaffold_4:10287065-10293014	175.809	189.139	0.105443
Ciclev10016186m.g	scaffold_2:8503008-8507698	22.8279	24.5591	0.105463
Ciclev10010888m.g	scaffold_6:24114251-24127929	10.0248	10.7858	0.105551
Ciclev10018854m.g	scaffold_3:39744782-39758090	53.8867	57.9831	0.105703
Ciclev10008909m.g	scaffold_1:14652519-14654663	234.579	252.475	0.106068
Ciclev10026089m.g	scaffold_7:4217935-4222514	10.0399	10.8061	0.106107
Ciclev10015579m.g	scaffold_2:8539409-8545538	94.7966	102.034	0.106138
Ciclev10017207m.g	scaffold_2:29258051-29260162	17.92	19.2886	0.106173
Ciclev10025502m.g	scaffold_7:2125830-2131056	99.1246	106.698	0.106224
Ciclev10000728m.g	scaffold_5:31895664-31900513	24.7877	26.6838	0.10634
Ciclev10008907m.g	scaffold_1:17471329-17472833	230.783	248.441	0.106364
Ciclev10002740m.g	scaffold_5:35260439-35261145	32.0626	34.5168	0.106409
Ciclev10018760m.g	scaffold_3:21914578-21927716	13.331	14.3516	0.106421
-	scaffold_8:24939651-24939943	49.0991	52.8676	0.106687
Ciclev10030660m.g	scaffold_4:23454165-23462968	19.5143	21.0124	0.106708
Ciclev10004573m.g	scaffold_9:30229137-30234704	23.0829	24.8565	0.1068
Ciclev10000707m.g	scaffold_5:42139641-42144610	73.8988	79.5826	0.106902
Ciclev10015576m.g	scaffold_2:6772920-6775813	32.1655	34.641	0.106967
Ciclev10014575m.g	scaffold_2:11225617-11229409	3.92505	4.22723	0.107001
Ciclev10010323m.g	scaffold_1:24532826-24536695	45.6738	49.1903	0.107009
Ciclev10011009m.g	scaffold_6:24734910-24739850	0.15676	0.168834	0.10705
Ciclev10000524m.g	scaffold_5:19682548-19686163	17.6193	18.9769	0.107092
Ciclev10012499m.g	scaffold_6:16909491-16912088	294.584	317.3	0.107166
Ciclev10029626m.g	scaffold_8:19410425-19412730	54.3491	58.5421	0.107216
Ciclev10018480m.g	scaffold_3:42716994-42733040	10.002	10.7769	0.107663
Ciclev10006489m.g,Ciclev10006630m.g	scaffold_9:29290414-29356041	7.95854	8.57569	0.107749
Ciclev10020054m.g	scaffold_3:48739712-48746026	44.9977	48.4911	0.107868
Ciclev10019827m.g	scaffold_3:44419110-44421778	16.644	17.9374	0.107966
Ciclev10019222m.g,Ciclev10024362m.g	scaffold_3:1411493-1417129	14.773	15.9229	0.108138
Ciclev10008440m.g,Ciclev10010830m.g	scaffold_1:21975140-22013742	67.0284	72.2667	0.108559
Ciclev10015350m.g	scaffold_2:36180160-36182248	19.0977	20.5914	0.10864
Ciclev10011312m.g	scaffold_6:6823299-6833292	20.4003	21.9969	0.108706
Ciclev10018592m.g	scaffold_3:16427553-16437603	6.46987	6.97663	0.108794
Ciclev10001076m.g	scaffold_5:11696248-11701383	13.3191	14.3625	0.108807
Ciclev10001807m.g	scaffold_5:29489011-29492069	17.2012	18.5511	0.108993
Ciclev10000800m.g	scaffold_5:41049807-41055695	15.5279	16.7489	0.109206
Ciclev10012354m.g	scaffold_6:22253435-22258616	94.7697	102.223	0.109215
Ciclev10031611m.g	scaffold_4:17171113-17174966	47.589	51.3355	0.109329
Ciclev10004788m.g	scaffold_9:2901313-2906603	16.6461	17.9575	0.109401
Ciclev10021305m.g	scaffold_3:32923706-32929662	16.3927	17.6852	0.109496
-	scaffold_9:22502478-22504841	2.39492	2.58376	0.109498
Ciclev10000013m.g	scaffold_5:40957570-40966096	22.9449	24.7542	0.109501
Ciclev10020854m.g	scaffold_3:41818451-41823411	19.939	21.5133	0.109637
Ciclev10031444m.g	scaffold_4:21799786-21804863	9.47993	10.229	0.109711
Ciclev10006127m.g	scaffold_9:4702401-4704790	157.635	170.101	0.109808
Ciclev10014364m.g	scaffold_2:32866282-32873723	4.41008	4.75887	0.109813
Ciclev10016162m.g	scaffold_2:11927401-11930902	9.04448	9.76055	0.109924
Ciclev10025254m.g	scaffold_7:6050556-6056779	10.1833	10.9897	0.109946

Ciclev10005943m.g	scaffold_9:6699174-6702019	36.2863	39.1608	0.109988
Ciclev10019843m.g	scaffold_3:50671059-50678689	18.4027	19.863	0.110162
Ciclev10027951m.g	scaffold_8:2939320-2944217	2.85057	3.07686	0.110206
Ciclev10022620m.g	scaffold_3:4899015-4901867	15.6215	16.8624	0.110281
Ciclev10019856m.g	scaffold_3:45468224-45473429	9.5923	10.3551	0.110392
Ciclev10002178m.g	scaffold_5:40200372-40208310	171.455	185.1	0.110474
Ciclev10003997m.g	scaffold_5:25221155-25545745	121.054	130.694	0.110546
Ciclev10030819m.g	scaffold_4:204632-209475	9.9726	10.7668	0.11055
Ciclev10012312m.g	scaffold_6:7924188-7928192	464.69	501.728	0.110635
Ciclev10020357m.g	scaffold_3:40229246-40235237	13.2815	14.3413	0.110754
Ciclev10021361m.g	scaffold_3:44754509-44755663	17.1143	18.4811	0.110845
Ciclev10024690m.g	scaffold_7:2968327-2981712	12.7993	13.8219	0.110889
Ciclev10031079m.g	scaffold_4:23484693-23488810	5.20658	5.62295	0.110992
Ciclev10007738m.g	scaffold_1:27839425-27844011	16.0625	17.3477	0.111054
Ciclev10025122m.g	scaffold_7:6583825-6587778	70.3243	75.9563	0.111147
Ciclev10028386m.g,Ciclev10029788m.g,Ciclev10029910m.g	scaffold_8:1443945-1489074	45.6723	49.3308	0.111169
Ciclev10007645m.g	scaffold_1:19595683-19598862	4.00585	4.3269	0.111227
Ciclev10009066m.g	scaffold_1:2925041-2932100	8.07051	8.71788	0.111318
Ciclev10004142m.g	scaffold_9:21061347-21073008	23.0637	24.9138	0.111326
Ciclev10022242m.g	scaffold_3:3659452-3662674	8.28493	8.95084	0.111533
Ciclev10001143m.g	scaffold_5:32780338-32783349	24.3036	26.2615	0.111775
Ciclev10030471m.g	scaffold_4:22775313-22788516	5.64462	6.09981	0.111888
Ciclev10013624m.g	scaffold_6:318069-325101	247.628	267.598	0.111889
Ciclev10028962m.g	scaffold_8:18112063-18117822	18.0275	19.4814	0.111895
Ciclev10016082m.g	scaffold_2:25524064-25526492	32.3493	34.9612	0.112018
Ciclev10018998m.g	scaffold_3:47579623-47583066	16.7246	18.0751	0.112033
Ciclev10021429m.g	scaffold_3:41427220-41432461	26.5005	28.6407	0.112045
Ciclev10012685m.g	scaffold_6:11126657-11131076	7.42838	8.02845	0.112075
Ciclev10007246m.g	scaffold_1:25046219-25065847	11.1992	12.1056	0.112287
Ciclev10018498m.g	scaffold_3:42618558-42630429	14.1994	15.3489	0.112303
Ciclev10032916m.g	scaffold_4:2766828-2770332	42.1496	45.5727	0.11265
Ciclev10032673m.g	scaffold_4:1175518-1180789	13.3879	14.4755	0.11268
Ciclev10004187m.g	scaffold_9:368943-376331	14.8827	16.0956	0.11303
Ciclev10005978m.g	scaffold_9:1906744-1908673	5.41407	5.85539	0.113053
Ciclev10008397m.g	scaffold_1:4585755-4588563	12.001	12.9835	0.113527
Ciclev10011770m.g	scaffold_6:9821167-9824796	5.52867	5.98179	0.113647
Ciclev10032090m.g	scaffold_4:23110215-23115421	28.7207	31.075	0.113666
Ciclev10005760m.g	scaffold_9:3705643-3707031	20.2182	21.8757	0.113677
Ciclev10015736m.g	scaffold_2:3944354-3948032	6.74517	7.29894	0.113832
Ciclev10009550m.g	scaffold_1:2214707-2222674	99.2663	107.42	0.113882
Ciclev10022162m.g	scaffold_3:42410481-42413511	59.9306	64.8539	0.113902
Ciclev10032202m.g	scaffold_4:22972716-22974735	23.488	25.4181	0.113933
Ciclev10025759m.g	scaffold_7:866844-870030	20.5458	22.2362	0.114066
Ciclev10021298m.g	scaffold_3:23175885-23177252	18.6851	20.2248	0.114238
Ciclev10008659m.g	scaffold_1:26872070-26875269	6.48416	7.01871	0.114286
Ciclev10011245m.g	scaffold_6:25178913-25180930	1.13729	1.23108	0.114328
Ciclev10028879m.g	scaffold_8:19397293-19398741	14.9151	16.1462	0.114427
Ciclev10021954m.g	scaffold_3:45076820-45080633	24.3315	26.3404	0.114454
Ciclev10001468m.g	scaffold_5:26175747-26181708	49.9163	54.0394	0.114501
Ciclev10010314m.g	scaffold_1:22175413-22384190	1.88829	2.04446	0.114641
Ciclev10012892m.g	scaffold_6:23916875-23941095	44.0635	47.7111	0.114739
Ciclev10018510m.g	scaffold_3:25558188-25563956	2.46554	2.66965	0.114746
Ciclev10025221m.g	scaffold_7:2220608-2225827	109.148	118.197	0.114909
Ciclev10027936m.g	scaffold_8:1005809-1009132	1.13328	1.22733	0.115018

Ciclev10011402m.g	scaffold_6:17974827-17979903	80.9038	87.6199	0.115051
Ciclev10020792m.g	scaffold_3:48724000-48728863	16.4623	17.8294	0.115097
Ciclev10000869m.g	scaffold_5:833390-839741	11.3708	12.3159	0.115184
Ciclev10000159m.g,Ciclev10003608m.g	scaffold_5:37780636-37790454	2.86458	3.10276	0.115228
-	scaffold_7:11686788-11771145	6.30181	6.82646	0.115371
Ciclev10032137m.g	scaffold_4:13723342-13724858	4.31169	4.67103	0.115488
Ciclev10015744m.g	scaffold_2:28186027-28194237	46.8666	50.7752	0.115564
Ciclev10019324m.g	scaffold_3:46429186-46435619	0.536474	0.581217	0.115569
Ciclev10022218m.g	scaffold_3:46334463-46336098	109.554	118.703	0.115714
Ciclev10033065m.g	scaffold_4:2856510-2858422	148.844	161.274	0.115716
Ciclev10014467m.g	scaffold_2:29260560-29269901	5.1431	5.57311	0.115843
Ciclev10008490m.g	scaffold_1:25111803-25117154	5.5658	6.0312	0.115854
Ciclev10028187m.g	scaffold_8:8209470-8214273	9.95168	10.7866	0.116227
Ciclev10031168m.g	scaffold_4:20982610-20988551	13.5425	14.6789	0.116246
Ciclev10013582m.g	scaffold_6:23446958-23455687	29.2609	31.7168	0.116273
Ciclev10031373m.g	scaffold_4:20138850-20141252	41.7462	45.2514	0.116319
Ciclev10002499m.g	scaffold_5:42835733-42838018	6.47407	7.01857	0.116504
Ciclev10028485m.g	scaffold_8:24987538-24993295	4.29319	4.65429	0.116512
Ciclev10023161m.g	scaffold_3:3491309-3493110	1.3743	1.48993	0.116545
Ciclev10022463m.g	scaffold_3:2878161-2881754	43.4269	47.0812	0.116564
Ciclev10015962m.g	scaffold_2:23070323-23071835	293.806	318.589	0.116835
Ciclev10007897m.g	scaffold_1:448784-452517	24.6847	26.768	0.116894
Ciclev10025443m.g	scaffold_7:4115966-4119050	18.2027	19.7403	0.116988
Ciclev10000185m.g	scaffold_5:38229418-38236940	40.2245	43.6229	0.117011
Ciclev10019538m.g	scaffold_3:44307102-44310571	1.08219	1.17372	0.117133
Ciclev10018855m.g	scaffold_3:49318105-49324231	33.208	36.0254	0.117484
Ciclev10020177m.g	scaffold_3:49336003-49339142	18.0484	19.58	0.117509
Ciclev10032842m.g	scaffold_4:19377410-19378601	99.4461	107.89	0.117576
Ciclev10000951m.g	scaffold_5:42704437-42709207	1257.79	1364.61	0.117592
Ciclev10022161m.g	scaffold_3:749175-751194	126.047	136.776	0.117851
Ciclev10016856m.g	scaffold_2:34832398-34834566	32.9376	35.7454	0.118024
Ciclev10029831m.g	scaffold_8:21348859-21352692	9.55033	10.3652	0.118122
Ciclev10031157m.g	scaffold_4:774030-778014	36.1259	39.2139	0.11833
Ciclev10007625m.g	scaffold_1:17150274-17158805	10.0295	10.887	0.118359
Ciclev10006136m.g	scaffold_9:6354795-6356630	226.587	245.965	0.118387
Ciclev10016366m.g	scaffold_2:29757722-29762309	19.7535	21.4437	0.118445
Ciclev10033231m.g	scaffold_4:6264316-6268506	17.1136	18.5781	0.118465
-	scaffold_3:50140118-50140491	14.72	15.9808	0.118555
Ciclev10015281m.g	scaffold_2:11519100-11522509	56.3258	61.1533	0.118635
Ciclev10029226m.g	scaffold_8:4950281-4953247	41.0571	44.5775	0.118687
Ciclev10028126m.g	scaffold_8:1111963-1115784	26.3509	28.611	0.118718
Ciclev10030550m.g	scaffold_4:23294042-23299797	2.02082	2.19425	0.118792
Ciclev10011841m.g	scaffold_6:21390476-21396087	77.5987	84.2788	0.119138
Ciclev10028499m.g	scaffold_8:5887970-5892945	23.6446	25.6839	0.119355
Ciclev10030693m.g	scaffold_4:23544778-23550502	12.4259	13.4979	0.119382
Ciclev10010350m.g	scaffold_1:6262793-6298426	84.809	92.1278	0.11942
Ciclev10028168m.g	scaffold_8:755039-759493	10.923	11.8665	0.11953
Ciclev10032978m.g	scaffold_4:16567711-16570325	45.4301	49.3617	0.119744
Ciclev10024482m.g	scaffold_3:21296577-21473295	5.76673	6.26621	0.119839
Ciclev10014023m.g	scaffold_2:33725771-33735828	22.1564	24.0757	0.119855
Ciclev10004656m.g	scaffold_9:3802714-3806843	11.9885	13.0276	0.119925
Ciclev10000038m.g	scaffold_5:41184179-41190033	10.9424	11.8911	0.119948
Ciclev10001309m.g	scaffold_5:34239480-34244107	8.92634	9.70083	0.12004
Ciclev10012509m.g	scaffold_6:19891325-19894889	63.5426	69.0614	0.120155
Ciclev10014710m.g	scaffold_2:25515915-25519821	154.548	167.98	0.120234

Ciclev10015047m.g	scaffold_2:35602949-35607440	14.359	15.6073	0.120268
Ciclev10019992m.g	scaffold_3:43399773-43405183	21.0374	22.8691	0.120445
-	scaffold_5:26608087-26611153	64.2481	69.8509	0.120626
Ciclev10011495m.g	scaffold_6:11410054-11414256	9.13633	9.9331	0.120629
Ciclev10005378m.g	scaffold_9:18348497-18357231	51.6596	56.1774	0.120955
Ciclev10025320m.g	scaffold_7:3204356-3210336	66.5782	72.401	0.120959
-	scaffold_8:9650172-9654215	13.3126	14.4778	0.121056
Ciclev10012666m.g	scaffold_6:12318603-12321335	18.6328	20.2659	0.121208
Ciclev10028992m.g	scaffold_8:14486037-14492800	18.5614	20.1887	0.121247
Ciclev10027973m.g	scaffold_8:24954435-24961347	37.3746	40.6521	0.12127
Ciclev10014760m.g	scaffold_2:35681537-35687125	55.7835	60.6781	0.121335
Ciclev10014066m.g,Ciclev10014530m.g	scaffold_2:5019440-5048791	22.7876	24.7873	0.121352
Ciclev10025236m.g	scaffold_7:19037369-19047271	13.8177	15.0304	0.121371
Ciclev10020740m.g	scaffold_3:2519413-2522243	18.9571	20.6217	0.121423
Ciclev10004461m.g	scaffold_9:12463483-12468099	77.0064	83.7712	0.121475
Ciclev10031408m.g	scaffold_4:302165-306822	50.1424	54.5483	0.121504
Ciclev10031719m.g	scaffold_4:2268335-2272720	4.30988	4.68895	0.121619
Ciclev10005925m.g	scaffold_9:27200749-27202181	18.4284	20.0513	0.121767
Ciclev10012466m.g	scaffold_6:19082592-19085506	21.6082	23.5125	0.121846
Ciclev10007803m.g,Ciclev10010169m.g	scaffold_1:3472407-3502192	32.1498	34.9835	0.121864
Ciclev10032940m.g	scaffold_4:10652376-10655082	21.8511	23.778	0.121923
Ciclev10005123m.g	scaffold_9:26813156-26816240	8.58244	9.3397	0.121989
Ciclev10011033m.g	scaffold_6:20201379-20214287	213.829	232.698	0.122003
Ciclev10011522m.g	scaffold_6:18491788-18493623	23.0598	25.0972	0.122146
Ciclev10002923m.g	scaffold_5:38603368-38606636	48.2468	52.5148	0.122289
Ciclev10020049m.g	scaffold_3:47374550-47377708	33.2328	36.1726	0.122293
Ciclev10019946m.g	scaffold_3:6256239-6258477	13.3343	14.5143	0.122333
Ciclev10017370m.g	scaffold_2:13350081-13351899	96.2347	104.758	0.122434
Ciclev10019299m.g	scaffold_3:42838147-42841432	14.3572	15.6314	0.122676
Ciclev10015089m.g	scaffold_2:30169032-30172263	9.54615	10.3934	0.122677
Ciclev10026140m.g	scaffold_7:9038417-9044438	3.23856	3.52619	0.122756
Ciclev10008517m.g	scaffold_1:28915797-28919965	8.03412	8.74836	0.122871
Ciclev10001727m.g	scaffold_5:39546369-39551019	9.43634	10.2762	0.123004
Ciclev10021945m.g	scaffold_3:6113048-6116055	176.27	191.96	0.12302
Ciclev10031487m.g	scaffold_4:23737486-23740513	27.35	29.7848	0.123033
Ciclev10015234m.g	scaffold_2:7753797-7755971	51.0428	55.5939	0.123218
Ciclev10009489m.g	scaffold_1:28353947-28356507	90.7775	98.8715	0.123219
Ciclev10019196m.g	scaffold_3:5368644-5374936	32.3569	35.2427	0.123254
Ciclev10012631m.g	scaffold_6:12220279-12223024	53.6129	58.3948	0.12326
Ciclev10008030m.g	scaffold_1:3774964-3781251	30.5352	33.2591	0.123274
Ciclev10014304m.g	scaffold_2:22607374-22611126	3.81229	4.15247	0.123312
Ciclev10031583m.g	scaffold_4:2734380-2741270	9.16082	9.97872	0.123378
Ciclev10008039m.g	scaffold_1:6412026-6416004	139.339	151.786	0.123445
Ciclev10030200m.g,Ciclev10030274m.g	scaffold_8:1312571-1315655	90.08	98.1322	0.12352
Ciclev10025986m.g	scaffold_7:3399492-3402834	1.62947	1.77528	0.12364
Ciclev10024961m.g	scaffold_7:6382228-6388196	3.95309	4.30707	0.123724
Ciclev10008980m.g	scaffold_1:28410232-28413508	4.036	4.39761	0.123793
Ciclev10011557m.g	scaffold_6:4691577-4697810	61.9421	67.4931	0.123821
Ciclev10028358m.g	scaffold_8:21972414-21975737	33.5533	36.5605	0.12383
Ciclev10032293m.g	scaffold_4:20407119-20411036	32.8044	35.7457	0.123878
Ciclev10009411m.g	scaffold_1:12274455-12277179	61.7951	67.3419	0.124014
Ciclev10004731m.g	scaffold_9:3158817-3162593	223.718	243.8	0.124022
Ciclev10006228m.g	scaffold_9:10223306-10227110	27.3134	29.7722	0.12436

Ciclev10021459m.g	scaffold_3:47223804-47226026	51.0479	55.6462	0.12443
Ciclev10000110m.g	scaffold_5:38426629-38430121	3.34286	3.64407	0.124467
Ciclev10003909m.g	scaffold_5:41136321-41137442	4.34371	4.73532	0.124532
Ciclev10012634m.g	scaffold_6:6180039-6183759	24.6975	26.9249	0.124577
Ciclev10005397m.g	scaffold_9:30826685-30828404	61.0393	66.5477	0.124651
Ciclev10030728m.g	scaffold_4:24788243-24793033	19.344	21.0939	0.124937
Ciclev10013679m.g	scaffold_6:12141279-12144299	2.30272	2.51113	0.125003
Ciclev10024909m.g	scaffold_7:5090145-5094501	40.6049	44.2818	0.125063
Ciclev10012008m.g	scaffold_6:20189754-20191150	0.324658	0.354059	0.125067
Ciclev10029793m.g	scaffold_8:18569342-18571053	1.426	1.55522	0.125144
Ciclev10030586m.g	scaffold_4:24777682-24783825	1.65142	1.80111	0.125175
Ciclev10019583m.g	scaffold_3:41336861-41344016	67.5642	73.6912	0.125235
Ciclev10019069m.g	scaffold_3:3919655-3924283	39.9673	43.5919	0.125242
-	scaffold_3:44252560-44254297	9.99947	10.9063	0.125243
Ciclev10019564m.g	scaffold_3:43241808-43248120	10.6197	11.5831	0.125277
Ciclev10026605m.g	scaffold_7:7050964-7054328	12.4857	13.6189	0.125331
Ciclev10005213m.g	scaffold_9:759432-762732	12.7677	13.9275	0.125434
Ciclev10028618m.g	scaffold_8:18919180-18923115	0.709767	0.774302	0.125549
Ciclev10027893m.g	scaffold_8:123934-128862	5.78665	6.31322	0.125649
Ciclev10007754m.g	scaffold_1:27475034-27482727	33.3757	36.4176	0.125838
Ciclev10031025m.g	scaffold_4:18307478-18313248	15.2045	16.5911	0.125912
Ciclev10026828m.g	scaffold_7:4565985-4569023	76.2088	83.1707	0.126118
Ciclev10004491m.g	scaffold_9:30490330-30500959	27.4529	29.9616	0.126157
Ciclev10000842m.g	scaffold_5:41201214-41208112	12.4086	13.5431	0.126218
Ciclev10004210m.g	scaffold_9:1363232-1371570	6.24964	6.8212	0.126253
Ciclev10016247m.g	scaffold_2:12016817-12021431	14.2796	15.5867	0.126361
Ciclev10032464m.g	scaffold_4:20124078-20126504	13.9881	15.2686	0.126372
Ciclev10020324m.g	scaffold_3:4383547-4387997	3.9787	4.34305	0.126411
Ciclev10004115m.g	scaffold_9:2284483-2302322	15.0748	16.4555	0.126426
Ciclev10033726m.g	scaffold_4:20372197-20377518	3.05925	3.33956	0.126481
Ciclev10001765m.g	scaffold_5:10004886-10009435	8.72397	9.5235	0.126506
-	scaffold_5:4076819-4284428	8.31091	9.07279	0.12654
Ciclev10024403m.g	scaffold_3:5439829-5441895	84.7978	92.572	0.126549
-	scaffold_3:35372249-35712253	19.3325	21.1074	0.126721
Ciclev10019842m.g	scaffold_3:1501464-1506491	40.6798	44.415	0.126734
Ciclev10034008m.g	scaffold_4:3364336-3365042	136.639	149.186	0.126734
Ciclev10001605m.g	scaffold_5:36021217-36023150	120.193	131.241	0.126861
Ciclev10015052m.g	scaffold_2:12239750-12244121	12.7619	13.9354	0.126907
Ciclev10024354m.g	scaffold_3:48979978-48981958	0.581688	0.635257	0.127094
Ciclev10007677m.g	scaffold_1:25830360-25839656	4.87933	5.32875	0.127116
Ciclev10014474m.g	scaffold_2:36048632-36052074	2.71007	2.9598	0.127169
Ciclev10015901m.g,Ciclev10015912m.g	scaffold_2:6964963-6980953	214.037	233.785	0.127318
Ciclev10005390m.g	scaffold_9:1902983-1906502	28.2693	30.8777	0.12733
Ciclev10024990m.g	scaffold_7:6595627-6602209	14.5075	15.8472	0.127426
Ciclev10018539m.g	scaffold_3:9369822-9381712	17.1438	18.7318	0.127801
Ciclev10021562m.g	scaffold_3:43260726-43272903	13.285	14.5164	0.127892
Ciclev10008924m.g	scaffold_1:17457108-17462147	16.4211	17.9433	0.127896
Ciclev10031659m.g	scaffold_4:14348149-14351500	7.9358	8.67331	0.128206
Ciclev10027802m.g	scaffold_8:20168558-20175359	13.8426	15.1327	0.128554
Ciclev10026735m.g	scaffold_7:18948566-18954524	160.442	175.427	0.128815
Ciclev10005126m.g	scaffold_9:2551275-2552750	5.36323	5.86418	0.128826
Ciclev10022962m.g	scaffold_3:47715714-47716698	17.1656	18.7695	0.128867
Ciclev10009250m.g	scaffold_1:1041085-1043376	0.493399	0.539513	0.128902
Ciclev10003846m.g	scaffold_5:36674808-36678923	8.87285	9.70224	0.128921
Ciclev10007493m.g	scaffold_1:23773533-23782088	10.7737	11.7811	0.128957

Ciclev10024713m.g	scaffold_7:7676288-7687190	8.49325	9.28784	0.129027
Ciclev10021036m.g	scaffold_3:13311420-13315166	35.7006	39.0424	0.129094
Ciclev10031908m.g	scaffold_4:21004218-21013226	39.9296	43.6677	0.129111
Ciclev10031194m.g	scaffold_4:6926074-6929005	4.85629	5.31179	0.129343
Ciclev10002322m.g	scaffold_5:16010186-16014546	30.6701	33.5476	0.129376
Ciclev10016775m.g	scaffold_2:9796351-9799528	14.3408	15.6878	0.129522
Ciclev10007886m.g	scaffold_1:8125278-8130885	61.0198	66.7552	0.129603
Ciclev10027499m.g	scaffold_7:6154282-6158701	12.1466	13.2889	0.129669
Ciclev10030781m.g	scaffold_4:24722956-24731239	13.5324	14.8065	0.129813
-	scaffold_2:29948187-29949275	5.13732	5.62129	0.129885
Ciclev10032364m.g	scaffold_4:24417601-24419019	66.8948	73.1982	0.129912
Ciclev10021696m.g	scaffold_3:48720836-48722931	144.54	158.168	0.129989
-	scaffold_5:16854446-16855340	3.18174	3.48219	0.130179
Ciclev10001161m.g	scaffold_5:39367032-39370754	24.8646	27.2137	0.130244
Ciclev10001990m.g	scaffold_5:34490116-34493454	10.082	11.0346	0.130258
Ciclev10014856m.g	scaffold_2:22752268-22755790	27.3009	29.881	0.130279
Ciclev10007314m.g	scaffold_1:17208075-17212038	2.25113	2.46394	0.130319
Ciclev10002443m.g,Ciclev10003050m.g	scaffold_5:13055388-13070403	16.8454	18.4379	0.130321
Ciclev10006760m.g	scaffold_9:25646847-25651910	13.168	14.4136	0.130398
Ciclev10026623m.g	scaffold_7:20922630-20924709	239.3	261.938	0.130408
Ciclev10018468m.g	scaffold_3:49574903-49587908	7.15619	7.83353	0.13047
Ciclev10011813m.g	scaffold_6:19032771-19035205	37.0885	40.6012	0.130551
Ciclev10009548m.g	scaffold_1:84901-86892	326.672	357.616	0.130567
Ciclev10025552m.g	scaffold_7:4245729-4250148	17.6214	19.293	0.130745
Ciclev10022792m.g	scaffold_3:13401556-13403611	147.861	161.89	0.130773
Ciclev10015528m.g	scaffold_2:32442421-32444029	53.6556	58.749	0.130838
Ciclev10015789m.g	scaffold_2:1581677-1585887	32.115	35.1639	0.130847
Ciclev10013005m.g	scaffold_6:21343420-21346199	75.8866	83.0984	0.130974
Ciclev10015286m.g	scaffold_2:9069333-9073496	60.4081	66.1508	0.131018
Ciclev10030053m.g	scaffold_8:7660705-7661377	3.16538	3.46648	0.131096
Ciclev10004654m.g	scaffold_9:2103210-2115677	9.51321	10.4182	0.131108
Ciclev10022656m.g	scaffold_3:41229261-41232505	17.4059	19.067	0.131503
Ciclev10023252m.g	scaffold_3:2645844-2647776	52.0986	57.0709	0.131509
Ciclev10020614m.g	scaffold_3:50961508-50967086	22.635	24.7975	0.13164
Ciclev10002332m.g	scaffold_5:42254118-42255871	203.025	222.436	0.131732
Ciclev10022760m.g	scaffold_3:1263749-1264641	84.7657	92.8772	0.131845
Ciclev10005180m.g	scaffold_9:143677-146962	10.4381	11.437	0.131853
Ciclev10001829m.g	scaffold_5:37437226-37439812	8.85613	9.70378	0.13187
Ciclev10008200m.g	scaffold_1:26710907-26715016	31.2466	34.2374	0.131875
Ciclev10026942m.g	scaffold_7:80191-85638	16.653	18.2501	0.13212
Ciclev10022582m.g	scaffold_3:12225044-12226547	114.776	125.786	0.132155
Ciclev10010574m.g	scaffold_1:6168507-6168822	47.2348	51.7667	0.132174
Ciclev10022283m.g	scaffold_3:23421698-23424030	153.741	168.493	0.132194
Ciclev10011436m.g	scaffold_6:25064461-25066126	3.89351	4.26725	0.132236
Ciclev10000205m.g	scaffold_5:36338559-36344138	15.9135	17.4412	0.13225
Ciclev10025758m.g	scaffold_7:9544920-9548282	9.7243	10.6607	0.132635
Ciclev10002978m.g	scaffold_5:12470098-12470442	5.566	6.10212	0.132669
Ciclev10009310m.g	scaffold_1:3118701-3121808	20.76	22.762	0.132817
Ciclev10003639m.g	scaffold_5:32997661-33008229	3.39674	3.72539	0.133244
Ciclev10023483m.g	scaffold_3:46720125-46721864	1.35307	1.48399	0.133246
Ciclev10032970m.g	scaffold_4:21090407-21092841	48.1605	52.8209	0.133257
Ciclev10005899m.g	scaffold_9:28889242-28892033	187.825	206.014	0.13335
Ciclev10026733m.g	scaffold_7:18561338-18565286	19.0137	20.8554	0.133387
Ciclev10027970m.g	scaffold_8:2006038-2014782	6.73381	7.3871	0.133584
Ciclev10007647m.g	scaffold_1:17895335-17903156	32.6213	35.7874	0.133636

Ciclev10027803m.g	scaffold_8:8428890-8438151	5.95412	6.53205	0.133647
Ciclev10014136m.g	scaffold_2:33545351-33550709	18.6444	20.4542	0.133653
Ciclev10014154m.g	scaffold_2:31581491-31592456	5.82411	6.39027	0.13384
Ciclev10025146m.g	scaffold_7:988690-993859	7.7199	8.47156	0.134045
Ciclev10004591m.g	scaffold_9:11274963-11277682	2.39298	2.62598	0.134051
Ciclev10009385m.g	scaffold_1:24224243-24227187	12.6992	13.9378	0.134274
Ciclev10021960m.g	scaffold_3:5606600-5609366	24.7546	27.1742	0.134542
Ciclev10012096m.g	scaffold_6:7519715-7525250	27.4789	30.1671	0.134651
Ciclev10008959m.g	scaffold_1:12399261-12405801	2.5423	2.7912	0.134753
Ciclev10033166m.g	scaffold_4:24921665-24923556	18.3468	20.1448	0.134883
Ciclev10001987m.g	scaffold_5:31889007-31893608	12.0916	13.2767	0.134899
Ciclev10015589m.g	scaffold_2:4461150-4463605	3.48081	3.82212	0.134951
Ciclev10031867m.g	scaffold_4:23042379-23046545	9.11558	10.0097	0.134994
-	scaffold_5:12360272-12360821	10.9694	12.0462	0.135092
Ciclev10007337m.g	scaffold_1:26628949-26632932	0.748689	0.822183	0.135094
Ciclev10029497m.g	scaffold_8:5163034-5164598	1.38327	1.51919	0.135219
Ciclev10021509m.g	scaffold_3:19310539-19608202	34.7661	38.1833	0.13526
Ciclev10011870m.g	scaffold_6:19420730-19427317	29.1196	31.9866	0.135479
Ciclev10020062m.g	scaffold_3:6879932-6884055	26.4745	29.0855	0.135699
Ciclev10005878m.g	scaffold_9:26426908-26430456	2.65104	2.91282	0.135855
Ciclev10005717m.g	scaffold_9:2603133-2605405	144.555	158.846	0.136012
Ciclev10029880m.g	scaffold_8:21056691-21057474	1.3017	1.43041	0.136027
Ciclev10011722m.g	scaffold_6:18572060-18576303	8.41296	9.24496	0.136054
Ciclev10022931m.g	scaffold_3:28631722-28633592	0.661831	0.727297	0.136083
Ciclev10012285m.g	scaffold_6:15774975-15777653	35.3762	38.8765	0.136117
Ciclev10025997m.g	scaffold_7:1242153-1245215	19.0616	20.9501	0.136287
Ciclev10018764m.g	scaffold_3:39771046-39787967	36.4293	40.039	0.136307
Ciclev10031717m.g	scaffold_4:22614912-22620498	29.476	32.3969	0.136315
Ciclev10002130m.g	scaffold_5:19593651-19596385	8.16653	8.97654	0.136437
Ciclev10005058m.g	scaffold_9:31225427-31229533	20.6935	22.7464	0.13646
Ciclev10030185m.g	scaffold_8:24976314-24979483	24.8022	27.2628	0.136467
Ciclev10031355m.g	scaffold_4:19173504-19177232	5.90408	6.4921	0.136973
Ciclev10000481m.g	scaffold_5:42782826-42787711	7.62616	8.3858	0.136991
Ciclev10012658m.g	scaffold_6:17623857-17628989	41.0612	45.1582	0.137212
Ciclev10030370m.g	scaffold_8:24698514-24701735	2.52462	2.77654	0.137221
Ciclev10032236m.g	scaffold_4:11310220-11312877	8.02498	8.82638	0.137324
Ciclev10026034m.g	scaffold_7:2586383-2588513	2821.52	3103.49	0.137417
Ciclev10004544m.g	scaffold_9:1303525-1309029	8.48555	9.33385	0.137465
Ciclev10008054m.g	scaffold_1:1533260-1537458	24.8822	27.3755	0.137774
Ciclev10016747m.g	scaffold_2:13999012-14002406	25.6464	28.2207	0.137999
-	scaffold_1:1667940-1670604	3.79812	4.17964	0.138095
Ciclev10031189m.g	scaffold_4:1159413-1164530	10.7314	11.8103	0.13821
Ciclev10026580m.g	scaffold_7:4233930-4238218	14.6972	16.1754	0.138256
Ciclev10012581m.g	scaffold_6:20088542-20090229	8.23179	9.06038	0.138364
Ciclev10012139m.g	scaffold_6:16412524-16416279	27.6067	30.3899	0.138574
Ciclev10015617m.g	scaffold_2:28833990-28838481	7.09649	7.81276	0.138728
Ciclev10011657m.g	scaffold_6:21464334-21477001	123.48	135.95	0.138797
Ciclev10015266m.g	scaffold_2:10399863-10403935	23.3706	25.7326	0.138902
Ciclev10022030m.g	scaffold_3:49751431-49755013	20.2493	22.2976	0.139017
Ciclev10032750m.g	scaffold_4:23273227-23276003	27.732	30.5373	0.139021
Ciclev10002098m.g	scaffold_5:36361964-36366774	37.6827	41.4949	0.139032
Ciclev10014664m.g	scaffold_2:29206817-29208977	0.263877	0.290586	0.139101
Ciclev10001382m.g,Ciclev10004052m.g	scaffold_5:38336208-38344772	77.2003	85.0218	0.139227
Ciclev10005920m.g	scaffold_9:185741-187387	42.9642	47.3215	0.139362
Ciclev10022597m.g	scaffold_3:18602702-18607570	52.2386	57.5397	0.139441

Ciclev10014908m.g,Ciclev10017357m.g	scaffold_2:22168520-22175294	38.0333	41.8966	0.139569
Ciclev10031412m.g	scaffold_4:25147682-25153345	47.3177	52.1259	0.139621
Ciclev10018634m.g	scaffold_3:39996924-40003176	11.8779	13.0859	0.139742
Ciclev10006954m.g	scaffold_9:5868242-5871900	0.607918	0.66976	0.139767
Ciclev10018985m.g	scaffold_3:3527347-3530487	6.0373	6.65218	0.139923
Ciclev10004369m.g	scaffold_9:1059387-1065318	10.5635	11.6394	0.139931
Ciclev10019197m.g	scaffold_3:9314953-9319905	13.0898	14.4234	0.139975
Ciclev10005352m.g	scaffold_9:12839344-12844087	42.3746	46.6971	0.140133
Ciclev10015632m.g	scaffold_2:16864117-16866936	40.6184	44.762	0.14014
Ciclev10005906m.g	scaffold_9:28796583-28801400	33.3047	36.7057	0.140279
Ciclev10015693m.g	scaffold_2:30811120-30815039	8.69628	9.58458	0.140318
Ciclev10020738m.g	scaffold_3:4036851-4040546	8.94512	9.85978	0.140454
Ciclev10014453m.g	scaffold_2:24524889-24530143	4.49309	4.95253	0.140458
Ciclev10014697m.g	scaffold_2:36057884-36060851	9.63585	10.6217	0.140534
Ciclev10007695m.g	scaffold_1:28750622-28753276	36.676	40.4292	0.140561
Ciclev10002515m.g	scaffold_5:37600816-37604471	65.2301	71.9172	0.1408
Ciclev10016911m.g	scaffold_2:34829299-34831997	56.3299	62.1059	0.14083
Ciclev10020996m.g	scaffold_3:49546711-49548191	12.102	13.3432	0.140861
Ciclev10030474m.g	scaffold_4:9547706-9559498	5.32036	5.86639	0.140949
Ciclev10014189m.g	scaffold_2:9951936-9960771	22.7142	25.0531	0.141398
Ciclev10007905m.g	scaffold_1:27930044-27937826	14.4973	15.9923	0.141594
Ciclev10008146m.g	scaffold_1:17313112-17316285	5.35325	5.90537	0.141613
Ciclev10005426m.g	scaffold_9:26886479-26890121	5.07787	5.60194	0.141704
Ciclev10002229m.g	scaffold_5:40166638-40169559	10.1613	11.2106	0.141774
Ciclev10018424m.g	scaffold_3:4595055-4624436	10.9391	12.0688	0.141786
Ciclev10020228m.g	scaffold_3:38254700-38259933	6.36489	7.02311	0.141974
Ciclev10011846m.g	scaffold_6:21541231-21543803	4.68098	5.1651	0.141987
Ciclev10010971m.g	scaffold_6:16665983-16677141	13.5442	14.9457	0.142063
Ciclev10031177m.g	scaffold_4:2290942-2317254	6.43828	7.10525	0.142209
Ciclev10031903m.g	scaffold_4:3561137-3564776	8.7689	9.67826	0.142351
Ciclev10017231m.g	scaffold_2:29309958-29313097	47.3166	52.2263	0.142431
Ciclev10022625m.g	scaffold_3:8715919-8718063	15.4649	17.0708	0.142528
-	scaffold_2:5650654-5706559	20.5712	22.7073	0.142532
Ciclev10029521m.g	scaffold_8:4551741-4553976	25.4261	28.0675	0.142591
Ciclev10014249m.g	scaffold_2:10224034-10231584	18.2467	20.1428	0.142633
Ciclev10030647m.g	scaffold_4:22767878-22774944	53.1639	58.6895	0.142656
Ciclev10029754m.g	scaffold_8:4205576-4222282	30.6102	33.7942	0.142761
-	scaffold_4:2942198-2942801	3.86089	4.26269	0.142831
Ciclev10018779m.g	scaffold_3:6394344-6403865	3.09498	3.41741	0.142975
Ciclev10009007m.g	scaffold_1:14089930-14104996	84.3787	93.1795	0.143135
Ciclev10028310m.g	scaffold_8:4953906-4958893	37.8695	41.8216	0.143212
Ciclev10022119m.g	scaffold_3:49740453-49745003	36.7318	40.5655	0.143224
Ciclev10011061m.g	scaffold_6:5411897-5422628	16.5482	18.2781	0.143442
Ciclev10024347m.g	scaffold_3:39789096-39789531	31.7289	35.0518	0.143691
Ciclev10018847m.g	scaffold_3:47259963-47263470	3.7637	4.15836	0.143864
Ciclev10020742m.g	scaffold_3:13318416-13324494	27.9606	30.8929	0.143877
Ciclev10000504m.g	scaffold_5:33233157-33240431	11.1959	12.3717	0.144079
Ciclev10026647m.g	scaffold_7:1354500-1356288	79.2532	87.5788	0.144112
Ciclev10020859m.g	scaffold_3:47291749-47295116	246.668	272.629	0.14437
Ciclev10017468m.g	scaffold_2:24517872-24523706	4.69956	5.19433	0.144413
Ciclev10022514m.g	scaffold_3:44431646-44433625	80.8324	89.3494	0.144525
Ciclev10030760m.g	scaffold_4:15444226-15457901	5.16021	5.70403	0.144553
Ciclev10028300m.g	scaffold_8:24007860-24043482	69.8975	77.2667	0.144605
Ciclev10029116m.g	scaffold_8:16045534-16047020	1701.35	1880.81	0.144677
Ciclev10018781m.g	scaffold_3:1759052-1765932	18.753	20.7324	0.144769

Ciclev10028883m.g	scaffold_8:130943-134457	5.955	6.58363	0.144781
Ciclev10011610m.g	scaffold_6:10598920-10603379	16.5522	18.3004	0.144852
Ciclev10011738m.g	scaffold_6:20161612-20163972	12.5984	13.9292	0.144868
Ciclev10030644m.g	scaffold_4:17719969-17729508	19.9281	22.0337	0.144911
Ciclev10001378m.g	scaffold_5:30639422-30641265	23.0496	25.4874	0.145045
Ciclev10020959m.g	scaffold_3:11191458-11193858	0.402503	0.445236	0.145571
Ciclev10031918m.g	scaffold_4:14897838-14901767	3.98124	4.40422	0.145668
Ciclev10014083m.g	scaffold_2:8664665-8670153	7.55578	8.35873	0.145703
Ciclev10031791m.g	scaffold_4:17053440-17056780	28.9567	32.0339	0.145703
Ciclev10000376m.g,Ciclev10000395m.g	scaffold_5:39644419-39664615	21.2812	23.5429	0.145711
Ciclev10008130m.g	scaffold_1:26127432-26131379	23.3885	25.8786	0.145964
Ciclev10024744m.g	scaffold_7:15962638-15973747	9.21248	10.1934	0.14597
Ciclev10008799m.g	scaffold_1:28474032-28489192	44.9981	49.7898	0.145985
Ciclev10011486m.g	scaffold_6:19542268-19544850	5.11055	5.65494	0.146032
Ciclev10014788m.g	scaffold_2:11799005-11831046	0.239628	0.26517	0.146117
Ciclev10010241m.g	scaffold_1:2631361-2632723	9.0766	10.0441	0.146126
Ciclev10013673m.g	scaffold_6:13071162-13073674	64.33	71.1884	0.146151
Ciclev10023124m.g	scaffold_3:18891079-18891585	35.2786	39.0399	0.146153
Ciclev10009371m.g	scaffold_1:23823652-23827297	10.1215	11.2014	0.146256
Ciclev10015567m.g	scaffold_2:10202276-10205534	3.82187	4.22993	0.146355
Ciclev10012184m.g	scaffold_6:22125811-22135730	35.0482	38.7924	0.146433
Ciclev10008114m.g	scaffold_1:14872334-14876238	20.9667	23.2069	0.146454
Ciclev10026475m.g	scaffold_7:615645-618369	40.4673	44.7964	0.146626
Ciclev10018763m.g	scaffold_3:4302072-4311270	31.7388	35.1384	0.146802
Ciclev10024813m.g	scaffold_7:164907-173806	36.8321	40.7809	0.146929
Ciclev10008214m.g	scaffold_1:23157246-23159095	2.72315	3.01518	0.146967
Ciclev10008557m.g	scaffold_1:25227977-25233899	5.27378	5.83955	0.147017
Ciclev10001268m.g	scaffold_5:36798371-36806685	77.1344	85.4115	0.147057
Ciclev10008544m.g	scaffold_1:5318850-5327042	4.91956	5.44756	0.147081
Ciclev10000281m.g	scaffold_5:40494581-40498159	43.5952	48.2744	0.147089
Ciclev10009049m.g	scaffold_1:17224875-17227961	31.6349	35.0312	0.14712
Ciclev10021263m.g	scaffold_3:44433921-44435536	16.5372	18.317	0.147471
Ciclev10005453m.g	scaffold_9:3996409-3999836	22.2776	24.6781	0.14764
Ciclev10007698m.g	scaffold_1:3125593-3128287	2.45317	2.71763	0.147703
Ciclev10026671m.g	scaffold_7:3478859-3481757	180.675	200.167	0.147806
Ciclev10011334m.g	scaffold_6:14451668-14454794	0.252802	0.28009	0.147886
Ciclev10000002m.g	scaffold_5:35462072-35488434	7.71995	8.55332	0.147893
Ciclev10032789m.g	scaffold_4:21831619-21837361	924.271	1024.12	0.147999
Ciclev10022706m.g	scaffold_3:1040100-1040986	1470.99	1630.06	0.148134
Ciclev10024274m.g	scaffold_3:24352153-24448329	1.68615	1.86867	0.148275
Ciclev10029051m.g	scaffold_8:23857321-23858484	20.2585	22.4532	0.14839
Ciclev10009595m.g	scaffold_1:28187318-28187926	4.08909	4.53268	0.148586
Ciclev10004234m.g	scaffold_9:320447-327846	18.1273	20.0947	0.148652
Ciclev10023068m.g	scaffold_3:4663210-4664667	222.801	247	0.148757
Ciclev10010920m.g	scaffold_6:17955316-17963554	20.2444	22.4446	0.148841
Ciclev10017507m.g	scaffold_2:10758866-10759898	1.50579	1.66955	0.14894
Ciclev10011898m.g	scaffold_6:22052370-22057463	11.3596	12.5951	0.148953
Ciclev10032349m.g	scaffold_4:16730149-16735677	37.793	41.9059	0.149037
Ciclev10019831m.g	scaffold_3:16308397-16313893	197.887	219.433	0.149106
Ciclev10007354m.g	scaffold_1:238855-247876	91.0462	100.961	0.149131
Ciclev10015679m.g	scaffold_2:10196716-10200643	19.6223	21.7594	0.149145
Ciclev10014019m.g	scaffold_2:7638847-7661169	21.0323	23.3233	0.149163
Ciclev10015967m.g	scaffold_2:13482004-13485923	12.7794	14.1719	0.149212
Ciclev10011781m.g	scaffold_6:24430778-24434240	18.6544	20.689	0.149343
Ciclev10031582m.g	scaffold_4:24670536-24673528	15.4534	17.1394	0.149392

Ciclev10030709m.g	scaffold_4:18238352-18241056	0.198986	0.220715	0.149515
Ciclev10019116m.g	scaffold_3:23932989-23936627	55.6199	61.7044	0.149773
Ciclev10025094m.g	scaffold_7:6984034-6989331	32.7188	36.2991	0.149814
Ciclev10000161m.g	scaffold_5:36181116-36190607	17.9117	19.8731	0.149911
Ciclev10005250m.g	scaffold_9:775883-777178	1.29118	1.43273	0.15007
Ciclev10016610m.g	scaffold_2:6530312-6531936	17.3667	19.2711	0.150116
Ciclev10007949m.g	scaffold_1:3414991-3419301	20.3443	22.5776	0.150271
Ciclev10004794m.g	scaffold_9:1090116-1095745	19.274	21.391	0.150345
Ciclev10015499m.g	scaffold_2:25982025-25985903	26.975	29.9402	0.150463
Ciclev10018551m.g	scaffold_3:17801102-17810498	14.7025	16.3189	0.150482
Ciclev10031568m.g	scaffold_4:10510017-10517939	28.0073	31.0871	0.150512
Ciclev10028298m.g	scaffold_8:17809427-17813065	30.9957	34.4067	0.15062
Ciclev10006888m.g	scaffold_9:15620369-15623726	17.9284	19.9015	0.150628
Ciclev10011038m.g	scaffold_6:19911967-19923790	12.3516	13.7115	0.150684
Ciclev10011411m.g	scaffold_6:18080701-18086773	11.1438	12.3707	0.15069
Ciclev10014143m.g	scaffold_2:30660123-30670171	17.0502	18.9284	0.150764
Ciclev10031363m.g	scaffold_4:22812345-22815857	51.9605	57.6894	0.150891
Ciclev10022360m.g	scaffold_3:43363460-43367561	20.284	22.5206	0.150905
Ciclev10008023m.g	scaffold_1:18676071-18683296	36.2001	40.1988	0.151156
Ciclev10022684m.g	scaffold_3:133777-135377	33.7602	37.494	0.151336
Ciclev10010918m.g	scaffold_6:17294105-17306487	20.3273	22.5756	0.151344
Ciclev10010894m.g	scaffold_6:25374985-25386569	12.5389	13.9259	0.151359
Ciclev10030855m.g	scaffold_4:2913252-2919063	11.3904	12.6514	0.151469
Ciclev10009524m.g	scaffold_1:13439122-13440998	8.16815	9.07305	0.151577
Ciclev10029911m.g	scaffold_8:23507393-23514304	9.1148	10.1249	0.151618
Ciclev10015368m.g	scaffold_2:2675774-2679971	18.3551	20.3893	0.151632
Ciclev10019024m.g	scaffold_3:673069-679076	3.86724	4.29589	0.151654
Ciclev10013138m.g	scaffold_6:23602788-23603142	13.9576	15.5056	0.151733
Ciclev10004629m.g	scaffold_9:170805-173284	10.4326	11.5918	0.152006
Ciclev10007961m.g	scaffold_1:6158270-6161582	20.9287	23.2551	0.152066
Ciclev10012864m.g	scaffold_6:21661969-21663972	168.059	186.752	0.152154
Ciclev10014505m.g	scaffold_2:14270260-14274038	5.61798	6.24314	0.152221
Ciclev10001174m.g	scaffold_5:34674627-34676788	10.3488	11.5017	0.15238
Ciclev10025641m.g	scaffold_7:14123946-14154295	27.9528	31.0686	0.152463
Ciclev10020126m.g	scaffold_3:42362870-42366230	8.21162	9.12782	0.152604
Ciclev10014243m.g	scaffold_2:34775231-34780330	20.5447	22.8369	0.152605
Ciclev10015236m.g	scaffold_2:899569-906091	13.3528	14.8429	0.152627
Ciclev10027059m.g	scaffold_7:2205171-2207008	0.491195	0.546067	0.152782
Ciclev10023868m.g	scaffold_3:44578529-44584573	32.1127	35.7005	0.152802
Ciclev10016224m.g	scaffold_2:11976868-11979833	99.4305	110.548	0.152912
-	scaffold_8:3257244-3394853	31.2756	34.7765	0.153076
Ciclev10019643m.g	scaffold_3:2752921-2755016	7.89806	8.78236	0.153109
Ciclev10032191m.g	scaffold_4:228996-232403	6.68474	7.43369	0.153207
Ciclev10000009m.g	scaffold_5:37855729-37864590	4.44644	4.94572	0.153531
Ciclev10011152m.g	scaffold_6:23748846-23755143	10.0277	11.154	0.15358
Ciclev10020879m.g	scaffold_3:25044599-25051607	0.379061	0.421646	0.153605
Ciclev10025482m.g	scaffold_7:6218451-6225133	26.5057	29.4842	0.153637
Ciclev10000742m.g	scaffold_5:42427317-42432272	11.7775	13.1019	0.153748
Ciclev10018489m.g	scaffold_3:6765568-6779230	3.13335	3.48587	0.153811
Ciclev10003046m.g	scaffold_5:427419-429934	81.4049	90.564	0.153822
Ciclev10011886m.g	scaffold_6:23233082-23238708	97.0786	108.002	0.153831
Ciclev10028130m.g	scaffold_8:20779068-20782853	14.5622	16.2008	0.153836
Ciclev10018730m.g	scaffold_3:45147481-45155340	29.376	32.6914	0.154274
Ciclev10022006m.g	scaffold_3:48730080-48732771	132.121	147.038	0.154332
Ciclev10016584m.g	scaffold_2:28653518-28656657	9.31954	10.372	0.154371
Ciclev10015158m.g	scaffold_2:7177135-7184525	6.08381	6.77164	0.15453

Ciclev10014844m.g	scaffold_2:30406774-30412389	68.9071	76.7069	0.154703
Ciclev10009427m.g	scaffold_1:2156678-2158871	193.428	215.331	0.154759
Ciclev10015550m.g	scaffold_2:412159-415182	104.016	115.796	0.154771
Ciclev10006038m.g	scaffold_9:3017547-3020758	6.81255	7.58405	0.154775
Ciclev10022626m.g	scaffold_3:44331059-44334243	17.7491	19.7594	0.154796
Ciclev10007308m.g	scaffold_1:25792926-25798214	18.1841	20.2481	0.155104
Ciclev10023263m.g	scaffold_3:45406214-45411786	41.3709	46.0689	0.155177
Ciclev10006222m.g	scaffold_9:29136066-29137568	332.755	370.563	0.155258
Ciclev10020039m.g	scaffold_3:45841250-45843998	230.686	256.917	0.155371
Ciclev10011131m.g,Ciclev10013912m.g	scaffold_6:20853955-20865611	9.33609	10.3992	0.155584
Ciclev10015783m.g,Ciclev10015790m.g,Ciclev10015793m.g	scaffold_2:4895612-4986690	14.2976	15.9258	0.155595
Ciclev10029476m.g	scaffold_8:21395526-21396128	211.523	235.621	0.155652
Ciclev10010813m.g	scaffold_1:1947258-1947945	80.8067	90.015	0.155689
Ciclev10007271m.g	scaffold_1:18719131-18732457	5.66586	6.31171	0.155736
Ciclev10032824m.g	scaffold_4:18983929-18986696	120.718	134.482	0.155776
Ciclev10013407m.g	scaffold_6:20783478-20784870	0.442568	0.493102	0.155988
Ciclev10001859m.g	scaffold_5:41855155-41858573	6.54187	7.28988	0.156192
Ciclev10023387m.g	scaffold_3:34001844-34002561	4.83649	5.38987	0.15629
Ciclev10008309m.g	scaffold_1:22862772-22866389	10.307	11.4872	0.156404
Ciclev10020598m.g	scaffold_3:37966578-37970062	29.8923	33.322	0.156703
Ciclev10013583m.g	scaffold_6:10573350-10576111	29.5582	32.9512	0.156772
Ciclev10033784m.g,Ciclev10033870m.g	scaffold_4:22356931-22361623	3.36354	3.75038	0.157055
Ciclev10001705m.g	scaffold_5:29646970-29649511	15.9077	17.7378	0.157109
Ciclev10002494m.g	scaffold_5:38988629-38991837	23.46	26.1631	0.15733
Ciclev10014721m.g	scaffold_2:27479906-27486732	66.4991	74.171	0.157519
Ciclev10003185m.g	scaffold_5:36401952-36405327	10.0043	11.1592	0.157609
Ciclev10014100m.g	scaffold_2:7847298-7855719	15.4554	17.2402	0.157669
Ciclev10024970m.g	scaffold_7:1762431-1766883	7.81021	8.71342	0.157879
Ciclev10001400m.g	scaffold_5:41657488-41660413	23.6308	26.3643	0.157918
Ciclev10004389m.g	scaffold_9:28544276-28551246	26.7188	29.8097	0.157926
Ciclev10005540m.g	scaffold_9:27184940-27187961	39.9304	44.5503	0.157949
Ciclev10005640m.g	scaffold_9:2037272-2040825	34.5089	38.5016	0.157951
Ciclev10018438m.g	scaffold_3:48871952-48884006	19.1134	21.3274	0.158117
Ciclev10009788m.g	scaffold_1:3547189-3549337	48.5115	54.132	0.158154
Ciclev10028242m.g	scaffold_8:2747670-2749378	5.77178	6.4409	0.158246
Ciclev10028839m.g	scaffold_8:24721936-24724569	88.425	98.6881	0.158421
Ciclev10018975m.g	scaffold_3:44624518-44630241	6.82665	7.61986	0.158587
Ciclev10008248m.g	scaffold_1:24450867-24452705	5.31329	5.93132	0.158748
Ciclev10014514m.g	scaffold_2:6800958-6813236	6.51745	7.27673	0.158982
Ciclev10025497m.g	scaffold_7:15749670-15751433	9.80872	10.9515	0.158988
Ciclev10018797m.g	scaffold_3:4449050-4458861	35.0263	39.1101	0.159102
Ciclev10011531m.g	scaffold_6:21074420-21078686	31.3184	34.9745	0.159294
Ciclev10017252m.g	scaffold_2:33551468-33556070	7.45846	8.32929	0.159316
Ciclev10000839m.g	scaffold_5:34815380-34819982	21.1852	23.6593	0.15935
Ciclev10027602m.g	scaffold_7:6610825-6614033	0.65674	0.733469	0.159413
Ciclev10028903m.g	scaffold_8:931346-934502	26.9795	30.1351	0.159577
Ciclev10018522m.g	scaffold_3:42470914-42478889	6.51396	7.27592	0.159595
Ciclev10005329m.g	scaffold_9:13039312-13041944	32.4567	36.2575	0.159761
Ciclev10020384m.g	scaffold_3:50038289-50042338	33.8845	37.8528	0.159777
Ciclev10033197m.g	scaffold_4:9055189-9056885	0.713836	0.797461	0.159821
Ciclev10032909m.g	scaffold_4:21351775-21355223	224.54	250.867	0.159949
Ciclev10020378m.g	scaffold_3:7265177-7267946	45.3211	50.6373	0.160017

Ciclev10001742m.g	scaffold_5:6473732-6478666	8.49012	9.48651	0.160092
Ciclev10004007m.g	scaffold_5:9134715-9136560	4.55435	5.08993	0.160402
Ciclev10014429m.g	scaffold_2:34115324-34119238	19.7384	22.0598	0.160417
Ciclev10007556m.g	scaffold_1:18463679-18466819	8.90557	9.95331	0.160469
Ciclev10028521m.g	scaffold_8:19603362-19605435	12.9716	14.4984	0.160532
Ciclev10029428m.g	scaffold_8:24044995-24049924	42.955	48.0219	0.160866
Ciclev10027777m.g,Ciclev10028148m.g	scaffold_8:3754499-3783084	4.37155	4.88858	0.16127
Ciclev10001017m.g	scaffold_5:40892737-40895876	7.17137	8.01967	0.161293
Ciclev10014071m.g	scaffold_2:2953668-2964530	8.81759	9.86128	0.16139
Ciclev10017571m.g	scaffold_2:7759601-7767192	10.678	11.9426	0.161473
Ciclev10032962m.g	scaffold_4:22165088-22165820	40.9375	45.7858	0.161478
Ciclev10007261m.g	scaffold_1:24125002-24131816	10.5782	11.832	0.1616
Ciclev10027922m.g	scaffold_8:3036000-3039225	4.90984	5.49208	0.161677
Ciclev10000100m.g	scaffold_5:43108471-43114247	17.024	19.0449	0.161834
Ciclev10000218m.g	scaffold_5:32690028-32694830	12.242	13.6972	0.162034
Ciclev10000604m.g	scaffold_5:41409272-41411910	29.0249	32.4755	0.162061
Ciclev10029393m.g	scaffold_8:2620011-2622409	26.8865	30.0835	0.162089
Ciclev10009380m.g,Ciclev10009383m.g	scaffold_1:17137677-17150200	15.9297	17.824	0.162103
Ciclev10008383m.g	scaffold_1:1876280-1881825	111.803	125.114	0.162283
Ciclev10020208m.g	scaffold_3:366258-370272	63.273	70.8068	0.162298
Ciclev10031884m.g	scaffold_4:2418387-2421858	14.741	16.4987	0.162521
Ciclev10033898m.g	scaffold_4:11950386-11953874	23.3985	26.1891	0.162554
Ciclev10004348m.g	scaffold_9:2374515-2381584	8.47731	9.48894	0.16264
Ciclev10004398m.g	scaffold_9:11969514-11979473	22.3981	25.0739	0.162806
Ciclev10031768m.g	scaffold_4:16741483-16745997	21.1895	23.7226	0.162913
Ciclev10008224m.g	scaffold_1:1401841-1406346	145.824	163.265	0.162983
Ciclev10032938m.g	scaffold_4:18986934-18989418	29.0534	32.5284	0.162995
Ciclev10027952m.g,Ciclev10029744m.g	scaffold_8:6024135-6031497	72.7723	81.4839	0.163127
-	scaffold_3:27951117-27952007	10.1633	11.3807	0.163213
Ciclev10026719m.g	scaffold_7:4280711-4281559	368.734	412.968	0.163448
Ciclev10004804m.g	scaffold_9:1395060-1397313	30.2364	33.8652	0.163518
Ciclev10009626m.g	scaffold_1:20995351-21034862	26.7198	29.927	0.163537
Ciclev10012686m.g	scaffold_6:25574146-25576968	46.7845	52.4017	0.163582
Ciclev10014110m.g	scaffold_2:29400984-29409394	6.07488	6.80444	0.163621
Ciclev10011430m.g	scaffold_6:23066383-23072168	36.3705	40.7417	0.16374
Ciclev10000469m.g	scaffold_5:35542503-35548256	27.7786	31.1205	0.16389
Ciclev10031072m.g	scaffold_4:6754145-6762440	13.1077	14.6884	0.164257
Ciclev10031995m.g	scaffold_4:24237867-24243284	9.72574	10.8996	0.164389
Ciclev10014444m.g	scaffold_2:4830566-4837258	58.8658	65.9916	0.164854
Ciclev10008416m.g	scaffold_1:5113848-5119533	63.423	71.1093	0.165031
Ciclev10005596m.g	scaffold_9:15107374-15113859	17.3721	19.4779	0.16507
Ciclev10002114m.g	scaffold_5:1474395-1479079	21.2866	23.8671	0.165082
Ciclev10004313m.g	scaffold_9:27191672-27197800	19.0079	21.3152	0.165286
Ciclev10000268m.g	scaffold_5:34147310-34157202	133	149.154	0.165375
Ciclev10030794m.g	scaffold_4:22340259-22348127	21.4176	24.0199	0.165432
Ciclev10011239m.g	scaffold_6:15703558-15706187	1.2883	1.4449	0.1655
Ciclev10009986m.g	scaffold_1:26177618-26179078	26.4614	29.6798	0.16559
Ciclev10027850m.g,Ciclev10027863m.g,Ciclev10030049m.g	scaffold_8:2946717-3001686	12.0308	13.4947	0.165653
Ciclev10028000m.g	scaffold_8:2462197-2466169	14.4754	16.2377	0.165746
Ciclev10015773m.g	scaffold_2:9922088-9926089	17.1919	19.2859	0.16582
Ciclev10018978m.g	scaffold_3:45848282-45854279	3.82976	4.29685	0.166025

Ciclev10018602m.g	scaffold_3:39678503-39689226	9.38278	10.5276	0.166095
Ciclev10019230m.g	scaffold_3:44021778-44027536	28.9287	32.4624	0.166266
Ciclev10021570m.g	scaffold_3:41610562-41616227	27.7294	31.1171	0.166294
Ciclev10022652m.g	scaffold_3:43753762-43756161	118.107	132.538	0.166317
Ciclev10025943m.g	scaffold_7:3236005-3240472	48.4177	54.3353	0.166355
Ciclev10018456m.g	scaffold_3:3970209-4002069	14.3495	16.1041	0.166433
Ciclev10004523m.g	scaffold_9:25396258-25402272	11.7352	13.1719	0.16662
Ciclev10028188m.g	scaffold_8:3002311-3006735	25.6484	28.7934	0.166868
Ciclev10025103m.g	scaffold_7:8247107-8250159	4.58793	5.1506	0.166899
Ciclev10003473m.g	scaffold_5:40912317-40927246	13.9297	15.6392	0.167008
Ciclev10003301m.g	scaffold_5:36751093-36761100	5.43756	6.10632	0.167343
Ciclev10014390m.g	scaffold_2:14217252-14224407	30.4472	34.2024	0.167788
Ciclev10018714m.g	scaffold_3:47099614-47109263	55.2152	62.0293	0.167885
Ciclev10015656m.g	scaffold_2:27303083-27306417	336.588	378.174	0.168066
Ciclev10004455m.g	scaffold_9:25156317-25159046	1.83207	2.05843	0.168068
Ciclev10025975m.g	scaffold_7:10701499-10706453	17.0224	19.1261	0.168113
Ciclev10030538m.g	scaffold_4:19816799-19825304	12.4681	14.009	0.168114
Ciclev10007870m.g	scaffold_1:25488563-25490475	0.654608	0.735528	0.16815
Ciclev10019182m.g	scaffold_3:30755858-30759190	14.8857	16.7262	0.168181
Ciclev10001571m.g	scaffold_5:41390245-41394472	15.0979	16.9654	0.168251
Ciclev10022661m.g	scaffold_3:42229223-42233850	7.05335	7.92604	0.168292
Ciclev10004613m.g	scaffold_9:3217261-3223074	9.73946	10.9453	0.168398
Ciclev10029767m.g	scaffold_8:4143726-4145846	41.1085	46.1987	0.168417
Ciclev10007551m.g	scaffold_1:27244319-27248139	18.9942	21.3484	0.168568
Ciclev10032148m.g	scaffold_4:14989163-14990893	3.11466	3.50078	0.168599
Ciclev10029538m.g	scaffold_8:23143377-23146210	284.035	319.31	0.168891
Ciclev10013675m.g	scaffold_6:7079175-7087001	22.4318	25.2186	0.168946
Ciclev10026634m.g	scaffold_7:4224387-4225072	20.0934	22.5933	0.169178
Ciclev10019576m.g	scaffold_3:36924550-37054826	5.07408	5.70607	0.16935
Ciclev10013871m.g	scaffold_6:17707405-17709937	78.4379	88.211	0.169407
Ciclev10002113m.g	scaffold_5:37901759-37903874	24.5095	27.5644	0.169464
Ciclev10004152m.g	scaffold_9:21163083-21173557	9.17549	10.32	0.169582
-	scaffold_7:5782316-5782870	10.2687	11.5522	0.169927
Ciclev10014625m.g	scaffold_2:27059110-27063827	29.3684	33.0404	0.169968
Ciclev10017950m.g	scaffold_2:35092369-35098568	128.601	144.688	0.170051
Ciclev10015498m.g	scaffold_2:24572810-24576260	106.431	119.748	0.17008
Ciclev10005413m.g	scaffold_9:3139914-3143078	14.5769	16.4009	0.170088
Ciclev10031037m.g	scaffold_4:22089994-22095521	12.0337	13.5402	0.170177
Ciclev10002140m.g,Ciclev10003172m.g	scaffold_5:16426017-16440179	22.9416	25.8142	0.170199
Ciclev10028392m.g	scaffold_8:23318602-23321320	36.8705	41.4882	0.170237
Ciclev10012287m.g	scaffold_6:8254731-8258915	31.9328	35.9361	0.170392
Ciclev10030994m.g	scaffold_4:22288054-22290485	8.10389	9.12018	0.170448
Ciclev10016181m.g	scaffold_2:15092750-15095115	83.225	93.6636	0.170472
Ciclev10004302m.g	scaffold_9:28840711-28847762	2.36541	2.66218	0.170519
Ciclev10031264m.g	scaffold_4:2008593-2013023	15.5709	17.5255	0.1706
Ciclev10007390m.g	scaffold_1:2481584-2489352	23.5476	26.5045	0.170659
Ciclev10023867m.g	scaffold_3:32847789-32868286	5.62911	6.33605	0.170677
Ciclev10007483m.g	scaffold_1:24814538-24820057	54.8911	61.7867	0.170723
Ciclev10002166m.g	scaffold_5:37834085-37836284	16.0421	18.058	0.170777
Ciclev10012475m.g	scaffold_6:13208437-13211113	28.6604	32.2649	0.170911
Ciclev10009792m.g	scaffold_1:28127730-28130521	65.163	73.3602	0.170945
Ciclev10002532m.g	scaffold_5:30631871-30632814	160.407	180.61	0.17114
Ciclev10031744m.g	scaffold_4:23192471-23198396	75.4796	84.9892	0.171193
Ciclev10025420m.g	scaffold_7:3732302-3735157	233.144	262.556	0.171398
Ciclev10010027m.g	scaffold_1:2821921-2826609	26.2686	29.5836	0.17146

Ciclev10022394m.g	scaffold_3:7973318-7975854	26.4594	29.7995	0.171507
Ciclev10014734m.g	scaffold_2:33198879-33205481	42.0669	47.3784	0.171545
Ciclev10001813m.g	scaffold_5:37212830-37217761	23.9044	26.9233	0.171582
Ciclev10013610m.g	scaffold_6:13801313-13814032	24.8331	27.97	0.171614
Ciclev10028680m.g	scaffold_8:6200167-6205693	35.2486	39.7058	0.171784
Ciclev10025931m.g	scaffold_7:1207140-1210820	39.3629	44.3404	0.171785
Ciclev10016130m.g	scaffold_2:32072446-32077800	40.4548	45.571	0.171804
Ciclev10028085m.g	scaffold_8:21578850-21582412	432.702	487.521	0.17209
Ciclev10000416m.g	scaffold_5:6447014-6453641	17.0455	19.2054	0.172126
Ciclev10019932m.g	scaffold_3:30066285-30161618	11.0268	12.4253	0.172267
Ciclev10021196m.g	scaffold_3:49253271-49256045	2.67467	3.01394	0.172292
Ciclev10027677m.g	scaffold_8:20570009-20579189	7.32245	8.25339	0.172661
Ciclev10010562m.g,Ciclev10010768m.g	scaffold_1:2033595-2037616	30.0637	33.8861	0.172674
Ciclev10017557m.g	scaffold_2:7132815-7134756	4.06427	4.5823	0.173076
Ciclev10012405m.g	scaffold_6:8893557-8898998	44.9489	50.6792	0.173107
Ciclev10032123m.g	scaffold_4:14043192-14044787	36.2645	40.8883	0.17313
Ciclev10008209m.g	scaffold_1:13148755-13158466	11.6469	13.132	0.173131
Ciclev10009515m.g	scaffold_1:3711748-3716370	50.9196	57.4223	0.173391
Ciclev10019145m.g	scaffold_3:36633448-36640384	17.797	20.0719	0.173542
Ciclev10032471m.g	scaffold_4:1537834-1540818	153.364	172.97	0.173564
Ciclev10016137m.g	scaffold_2:32971924-32975786	2.40037	2.70737	0.173637
Ciclev10004734m.g	scaffold_9:2063196-2068565	28.3537	31.9912	0.174138
Ciclev10031911m.g	scaffold_4:21235127-21237954	7.12925	8.04435	0.174226
Ciclev10012731m.g	scaffold_6:25114212-25116604	11.9594	13.4945	0.174228
Ciclev10026162m.g	scaffold_7:15097819-15101764	0.340027	0.383696	0.174314
Ciclev10016323m.g	scaffold_2:25237780-25240126	11.1665	12.6007	0.174331
Ciclev10025783m.g	scaffold_7:6522482-6526750	11.8734	13.3988	0.174362
Ciclev10026288m.g	scaffold_7:11097957-11100869	17.0741	19.2678	0.17438
Ciclev10023315m.g	scaffold_3:803190-806215	0.694857	0.784261	0.174618
Ciclev10025565m.g	scaffold_7:8704743-8708197	4.78007	5.39539	0.174695
Ciclev10005898m.g,Ciclev10007055m.g	scaffold_9:4969793-4980636	21.3761	24.1285	0.174741
Ciclev10023056m.g	scaffold_3:7025543-7039885	0.85037	0.959916	0.174817
Ciclev10030414m.g	scaffold_8:18009821-18010382	3.78968	4.27794	0.174842
Ciclev10032679m.g	scaffold_4:19462704-19467432	1.4411	1.62681	0.17488
Ciclev10019469m.g	scaffold_3:2691405-2695002	5.69124	6.4247	0.174887
Ciclev10028110m.g	scaffold_8:138021-146284	37.4124	42.237	0.17499
Ciclev10001305m.g	scaffold_5:42183489-42186862	14.4287	16.2894	0.174993
Ciclev10012601m.g	scaffold_6:16225489-16227461	24.2305	27.3567	0.175069
Ciclev10007792m.g	scaffold_1:23662147-23665854	13.0389	14.7215	0.175095
Ciclev10022547m.g	scaffold_3:45900655-45901592	26.2489	29.6487	0.175712
Ciclev10000836m.g	scaffold_5:42437642-42442753	36.3379	41.0447	0.17572
Ciclev10000341m.g	scaffold_5:38127346-38134197	14.8725	16.8013	0.17593
Ciclev10007581m.g	scaffold_1:28838025-28846371	31.2951	35.3601	0.176187
Ciclev10009375m.g	scaffold_1:27043123-27045483	89.3541	100.97	0.176324
Ciclev10008121m.g	scaffold_1:22991908-22995676	11.0211	12.4542	0.176365
Ciclev10011658m.g	scaffold_6:23326835-23331134	73.1426	82.6549	0.176389
Ciclev10011814m.g	scaffold_6:23547785-23552560	13.6378	15.4119	0.176432
Ciclev10019609m.g	scaffold_3:37906089-37910975	0.798727	0.902688	0.176524
Ciclev10027142m.g	scaffold_7:20045066-20048798	67.1801	75.9266	0.176572
Ciclev10012286m.g	scaffold_6:22572302-22575249	84.0418	94.9885	0.176646
Ciclev10030675m.g	scaffold_4:24065205-24072529	6.19155	6.99849	0.176743
Ciclev10000803m.g	scaffold_5:26514898-26519136	31.0667	35.1187	0.176869
Ciclev10008916m.g	scaffold_1:26957968-26961254	6.88368	7.78241	0.177037
Ciclev10030548m.g	scaffold_4:21593161-21599761	7.34998	8.30984	0.17708

Ciclev10002804m.g	scaffold_5:32313298-32314734	65.6342	74.2134	0.177232
Ciclev10002815m.g	scaffold_5:35894200-35898548	20.1936	22.8395	0.177628
Ciclev10025634m.g	scaffold_7:628942-635238	9.94725	11.252	0.177807
Ciclev10026339m.g	scaffold_7:782312-784526	58.4738	66.1532	0.17802
Ciclev10001411m.g	scaffold_5:35702667-35709316	6.13393	6.94016	0.178156
Ciclev10004380m.g	scaffold_9:4550507-4553584	5.95955	6.74321	0.178234
Ciclev10009632m.g	scaffold_1:22844240-22846485	24.6928	27.9417	0.178325
Ciclev10011212m.g	scaffold_6:20170180-20173803	4.05205	4.58519	0.178329
Ciclev10004590m.g	scaffold_9:17981882-17991830	21.3321	24.1409	0.17845
Ciclev10005579m.g	scaffold_9:28980608-28983750	11.5058	13.0252	0.178944
Ciclev10029395m.g	scaffold_8:24785531-24788306	15.6039	17.6645	0.178948
Ciclev10030774m.g	scaffold_4:21555574-21563562	54.2565	61.4234	0.178992
Ciclev10028293m.g	scaffold_8:2572438-2574295	229.142	259.414	0.179012
Ciclev10032370m.g	scaffold_4:17118915-17121135	141.852	160.618	0.179247
Ciclev10016816m.g	scaffold_2:36027354-36028643	50.4252	57.1009	0.179369
Ciclev10021795m.g	scaffold_3:42987963-42989360	1.44103	1.63181	0.179373
Ciclev10019465m.g	scaffold_3:50417889-50421625	40.4072	45.7697	0.17978
Ciclev10031187m.g	scaffold_4:2368537-2372365	87.5517	99.1923	0.180093
Ciclev10025108m.g	scaffold_7:1695501-1698071	1.74395	1.97602	0.180237
Ciclev10019993m.g	scaffold_3:9233301-9241034	17.7192	20.0775	0.180264
-	scaffold_6:18422991-18429125	18.8955	21.4116	0.180353
Ciclev10018880m.g	scaffold_3:42827086-42837797	8.93768	10.1294	0.180575
Ciclev10019229m.g	scaffold_3:34294666-34298815	19.8339	22.4786	0.180586
Ciclev10005507m.g	scaffold_9:17053579-17057386	7.24694	8.21336	0.180601
Ciclev10000230m.g	scaffold_5:40639760-40650497	34.0341	38.5759	0.180719
Ciclev10024806m.g	scaffold_7:14606082-14621902	25.5553	28.9661	0.180739
Ciclev10022297m.g	scaffold_3:3232477-3234759	43.4664	49.2726	0.180883
Ciclev10022575m.g	scaffold_3:44291485-44293163	18.588	21.0725	0.180992
Ciclev10033517m.g	scaffold_4:6866680-6869241	13.0473	14.7917	0.181043
Ciclev10014020m.g	scaffold_2:7441786-7452231	5.44807	6.17657	0.18106
Ciclev10024194m.g,Ciclev10024349m.g	scaffold_3:4992797-5000334	15.9966	18.1373	0.181193
Ciclev10018719m.g	scaffold_3:33145681-33153101	44.4628	50.4136	0.181213
Ciclev10012918m.g	scaffold_6:16359096-16369830	29.2117	33.1241	0.181336
Ciclev10028345m.g	scaffold_8:22543033-22545979	42.5532	48.2534	0.181362
Ciclev10025842m.g	scaffold_7:877884-881752	10.5063	11.9141	0.181419
Ciclev10025646m.g	scaffold_7:10480296-10482678	22.4532	25.4632	0.181497
Ciclev10004614m.g	scaffold_9:28835418-28840422	7.82567	8.87494	0.181524
Ciclev10031108m.g	scaffold_4:19785122-19789516	7.8741	8.93039	0.181609
Ciclev10002377m.g	scaffold_5:40148351-40150388	38.228	43.3567	0.181626
Ciclev10021261m.g	scaffold_3:4675157-4678109	8.38789	9.51335	0.181645
Ciclev10033741m.g	scaffold_4:20585520-20592849	0.145364	0.164877	0.181722
Ciclev10006256m.g	scaffold_9:955175-957425	8.21732	9.32049	0.181738
Ciclev10033075m.g	scaffold_4:17398843-17401989	7.00714	7.94832	0.181824
Ciclev10014965m.g	scaffold_2:34097796-34103044	7.00007	7.94034	0.18183
Ciclev10021253m.g	scaffold_3:14858208-14864855	84.4959	95.8515	0.181921
Ciclev10028326m.g	scaffold_8:1685503-1688837	4.65516	5.28097	0.181972
Ciclev10000233m.g	scaffold_5:5995714-6003233	21.5688	24.4688	0.181996
-	scaffold_3:40032668-40032899	25.7907	29.2594	0.182048
Ciclev10018754m.g	scaffold_3:22787984-22801272	25.9058	29.3919	0.18214
Ciclev10018708m.g	scaffold_3:35980222-35983153	0.164543	0.186701	0.182261
Ciclev10012015m.g	scaffold_6:22125811-22135730	19.4522	22.0727	0.182326
Ciclev10009622m.g	scaffold_1:28519496-28520746	299.859	340.283	0.18245
Ciclev10011039m.g	scaffold_6:17611200-17618263	13.4655	15.2831	0.182661
Ciclev10023597m.g	scaffold_3:5648369-5656690	5.13372	5.82668	0.182667
Ciclev10004704m.g	scaffold_9:27930132-27934521	12.5806	14.2792	0.182716

Ciclev10017484m.g	scaffold_2:27163799-27166928	1.69201	1.92079	0.182965
Ciclev10024784m.g	scaffold_7:14228840-14234852	11.609	13.1789	0.182991
Ciclev10015058m.g	scaffold_2:31294112-31299368	23.3006	26.4563	0.183243
Ciclev10020245m.g	scaffold_3:50267034-50276642	9.72034	11.0369	0.18326
Ciclev10000274m.g	scaffold_5:28852991-28857436	2.55187	2.89761	0.183305
Ciclev10008180m.g	scaffold_1:21901402-21911501	7.4835	8.49759	0.18334
Ciclev10029351m.g	scaffold_8:2397151-2398201	10.3668	11.7735	0.183574
Ciclev10000243m.g	scaffold_5:26183763-26192455	25.7465	29.2403	0.18358
Ciclev10009163m.g	scaffold_1:27300416-27304668	22.0681	25.0641	0.18366
Ciclev10008896m.g	scaffold_1:23523184-23525470	0.738615	0.838959	0.183777
Ciclev10004304m.g	scaffold_9:22377423-22382134	6.40107	7.27069	0.183779
Ciclev10012732m.g	scaffold_6:7123526-7128824	7.62658	8.66361	0.183933
Ciclev10024809m.g	scaffold_7:7158571-7166069	6.44145	7.31737	0.18394
Ciclev10022184m.g	scaffold_3:44455907-44460379	37.92	43.0791	0.184028
Ciclev10013571m.g	scaffold_6:17704054-17707152	16.8085	19.0966	0.184125
Ciclev10000562m.g	scaffold_5:30803767-30807677	12.0389	13.6779	0.184144
Ciclev10031706m.g	scaffold_4:18452652-18456449	60.8345	69.1201	0.184215
Ciclev10009748m.g	scaffold_1:16371167-16375666	83.412	94.7926	0.184519
Ciclev10004445m.g	scaffold_9:12523431-12535887	11.3153	12.8594	0.184548
Ciclev10016755m.g	scaffold_2:30956494-30957467	13.3411	15.1623	0.184611
Ciclev10004979m.g	scaffold_9:13375287-13379293	75.5299	85.846	0.184702
Ciclev10028065m.g	scaffold_8:24345532-24350242	91.8044	104.346	0.18474
Ciclev10004496m.g	scaffold_9:22204892-22216339	7.93915	9.0241	0.184799
Ciclev10018735m.g,Ciclev10024220m.g,Ciclev10024533m.g	scaffold_3:33810210-33895316	57.3709	65.2112	0.184801
Ciclev10014543m.g	scaffold_2:3663965-3674650	14.5834	16.5778	0.184919
Ciclev10008324m.g	scaffold_1:28011274-28013393	8.56534	9.73732	0.185015
Ciclev10032930m.g	scaffold_4:1124965-1127534	126.382	143.69	0.185174
Ciclev10016325m.g	scaffold_2:25528399-25533049	9.58277	10.8952	0.185175
Ciclev10028944m.g	scaffold_8:4100132-4102863	78.6592	89.4609	0.185642
Ciclev10006260m.g	scaffold_9:29245206-29245880	6.71647	7.63971	0.185816
Ciclev10002735m.g	scaffold_5:13759533-13761411	335.367	381.498	0.185935
Ciclev10024897m.g	scaffold_7:2527093-2533681	43.8089	49.8415	0.186124
Ciclev10005092m.g,Ciclev10006318m.g	scaffold_9:12904494-12916608	20.4914	23.3143	0.186199
Ciclev10016505m.g	scaffold_2:16708467-16713193	12.3226	14.0205	0.186234
Ciclev10020847m.g	scaffold_3:137599-141631	23.9889	27.2954	0.186289
Ciclev10019814m.g	scaffold_3:50640449-50643172	20.1353	22.9127	0.186419
Ciclev10010268m.g	scaffold_1:3882982-3885602	78.0855	88.8675	0.186602
Ciclev10009480m.g	scaffold_1:2574768-2577568	71.2051	81.0376	0.186611
Ciclev10004125m.g,Ciclev10004140m.g	scaffold_9:14393338-14461124	11.1373	12.6763	0.186733
Ciclev10000699m.g	scaffold_5:24818559-24855997	11.2913	12.8521	0.186804
Ciclev10016499m.g	scaffold_2:36322876-36326069	21.6028	24.5898	0.186838
-	scaffold_2:28539624-28539930	8.84358	10.0664	0.186845
Ciclev10028762m.g	scaffold_8:3486731-3491627	3.82654	4.35564	0.186847
Ciclev10014012m.g	scaffold_2:297542-314196	11.7657	13.3943	0.187024
Ciclev10028283m.g	scaffold_8:2593052-2597179	3.38409	3.85256	0.187048
Ciclev10009876m.g	scaffold_1:4535583-4538270	89.5651	101.973	0.18718
Ciclev10014898m.g	scaffold_2:14523710-14530229	20.9437	23.8459	0.187224
Ciclev10032342m.g	scaffold_4:125953-131376	102.548	116.76	0.187252
Ciclev10007050m.g	scaffold_9:5687882-5695888	20.3315	23.1497	0.187274
Ciclev10008832m.g	scaffold_1:23764336-23768786	12.6463	14.3997	0.18732
Ciclev10007739m.g	scaffold_1:4415727-4418878	3.55333	4.04611	0.187365
Ciclev10014800m.g	scaffold_2:822342-826856	9.6237	10.9594	0.187499

Ciclev10004383m.g	scaffold_9:7596484-7605014	4.34449	4.94772	0.187576
Ciclev10020620m.g	scaffold_3:42759651-42762628	108.671	123.766	0.187646
Ciclev10021113m.g	scaffold_3:49390883-49393176	43.8908	49.9875	0.187648
Ciclev10000127m.g,Ciclev10000346m.g	scaffold_5:39774139-39791254	3.18725	3.63024	0.187753
Ciclev10027927m.g	scaffold_8:23574079-23579813	12.585	14.3349	0.187829
Ciclev10018775m.g	scaffold_3:46741907-46749827	27.0881	30.8547	0.187833
Ciclev10017163m.g	scaffold_2:28813200-28814578	20.4626	23.3136	0.188184
Ciclev10008881m.g	scaffold_1:4615595-4619269	25.9752	29.5945	0.188194
Ciclev10026682m.g	scaffold_7:13916806-14069972	6.79923	7.7479	0.188433
Ciclev10000010m.g	scaffold_5:37569436-37579266	5.97679	6.81166	0.188636
Ciclev10001827m.g,Ciclev10004074m.g	scaffold_5:36952669-36956759	13.7424	15.6638	0.188798
Ciclev10030706m.g	scaffold_4:3124837-3130423	22.0727	25.159	0.188811
Ciclev10007427m.g	scaffold_1:25807711-25814164	17.7598	20.2439	0.188872
Ciclev10033025m.g	scaffold_4:24584063-24586056	65.7232	74.919	0.188927
Ciclev10031626m.g	scaffold_4:22789103-22792252	63.3717	72.2383	0.188927
Ciclev10014974m.g	scaffold_2:24604359-24608590	31.1302	35.4867	0.188964
Ciclev10029292m.g	scaffold_8:4864921-4867863	92.1985	105.103	0.188984
Ciclev10020716m.g	scaffold_3:4467424-4471381	10.9177	12.4463	0.189051
Ciclev10012804m.g	scaffold_6:6440925-6442977	24.5056	27.9391	0.189174
Ciclev10019310m.g	scaffold_3:25067424-25072316	92.2947	105.24	0.18936
Ciclev10026541m.g	scaffold_7:8743958-8748330	17.7573	20.2502	0.189523
Ciclev10030544m.g	scaffold_4:19947428-19954736	27.6399	31.54	0.190432
Ciclev10014510m.g	scaffold_2:34767188-34771631	40.932	46.709	0.190473
Ciclev10028040m.g	scaffold_8:2719653-2725331	22.1514	25.2788	0.190531
Ciclev10028991m.g	scaffold_8:24073627-24076678	4.77274	5.44688	0.190611
Ciclev10031346m.g	scaffold_4:2451344-2457577	44.4805	50.764	0.190634
Ciclev10030987m.g	scaffold_4:19142401-19148581	14.6603	16.7314	0.190642
Ciclev10018792m.g	scaffold_3:44168693-44174210	38.8354	44.3232	0.190689
Ciclev10002803m.g	scaffold_5:21399870-21400827	232.368	265.241	0.190893
Ciclev10014636m.g	scaffold_2:33847884-33852043	12.2551	13.9942	0.191444
Ciclev10033310m.g	scaffold_4:19001055-19040863	10.5251	12.0234	0.192007
Ciclev10018511m.g	scaffold_3:24819123-24831081	14.3192	16.362	0.192394
Ciclev10019663m.g	scaffold_3:5406446-5412349	3.36855	3.84928	0.192461
Ciclev10028431m.g	scaffold_8:3813869-3825919	16.3149	18.6466	0.192723
Ciclev10019655m.g	scaffold_3:27802934-27807050	8.15467	9.32062	0.192799
Ciclev10013192m.g,Ciclev10013845m.g	scaffold_6:9838374-9846042	20.2836	23.1838	0.192806
Ciclev10014139m.g	scaffold_2:33779001-33785794	15.2605	17.4447	0.192981
Ciclev10003328m.g	scaffold_5:38374950-38378462	12.8373	14.6747	0.192991
-	scaffold_3:47613852-47614797	357.162	408.313	0.193098
Ciclev10003766m.g	scaffold_5:43160577-43169797	20.4574	23.3894	0.193231
Ciclev10017261m.g	scaffold_2:26858715-26861439	135.653	155.103	0.193309
Ciclev10008917m.g	scaffold_1:28055353-28057726	52.4617	59.991	0.193481
Ciclev10028803m.g	scaffold_8:8022441-8026555	32.9197	37.6451	0.19351
Ciclev10001645m.g	scaffold_5:24386172-24389558	60.5389	69.2305	0.193547
Ciclev10016516m.g	scaffold_2:3841920-3845872	41.3375	47.2734	0.193576
Ciclev10014331m.g	scaffold_2:8360950-8371212	21.2642	24.3185	0.193629
Ciclev10023982m.g	scaffold_3:28679205-28682787	2.53252	2.89658	0.193776
Ciclev10003153m.g	scaffold_5:27391368-27393349	2.0361	2.32881	0.19378
Ciclev10006856m.g	scaffold_9:5671945-5676756	68.0776	77.8703	0.193893
Ciclev10009965m.g	scaffold_1:15473559-15474684	2.157	2.46757	0.194064
Ciclev10027594m.g	scaffold_7:12285085-12288663	5.11054	5.84702	0.194226
Ciclev10024198m.g,Ciclev10024650m.g	scaffold_3:30021997-30047514	185.007	211.686	0.194348

Ciclev10008343m.g	scaffold_1:26136546-26139615	222.54	254.665	0.194536
Ciclev10002819m.g	scaffold_5:30608390-30610556	243.289	278.429	0.194636
Ciclev10004289m.g	scaffold_9:7533788-7542235	3.51599	4.02456	0.194901
Ciclev10015263m.g	scaffold_2:36223845-36231874	5.8994	6.75278	0.194913
Ciclev10015660m.g	scaffold_2:182266-187365	16.0104	18.327	0.194964
Ciclev10025845m.g	scaffold_7:15484243-15485790	23.1076	26.4535	0.195091
Ciclev10007867m.g	scaffold_1:25685981-25691108	11.8889	13.6108	0.195143
Ciclev10022992m.g	scaffold_3:21537724-21543540	111.054	127.172	0.195519
Ciclev10022900m.g	scaffold_3:2049917-2051643	211.629	242.379	0.195727
Ciclev10020420m.g	scaffold_3:33754585-33759848	16.2512	18.6131	0.195767
Ciclev10011799m.g	scaffold_6:15208049-15210795	4.14334	4.74556	0.195784
Ciclev10025683m.g	scaffold_7:3933194-3939337	43.4646	49.7891	0.19599
Ciclev10022388m.g	scaffold_3:7042031-7042805	0.716442	0.820732	0.19606
Ciclev10022569m.g	scaffold_3:44796156-44798699	58.1677	66.6373	0.196111
Ciclev10018791m.g	scaffold_3:45137043-45145995	18.5918	21.2997	0.196167
Ciclev10012144m.g	scaffold_6:20465217-20471381	187.303	214.597	0.196253
Ciclev10011780m.g	scaffold_6:24082262-24084635	6.79331	7.78327	0.196263
Ciclev10028995m.g	scaffold_8:22463548-22466571	98.3722	112.713	0.196327
Ciclev10017919m.g	scaffold_2:28283067-28283796	2.9612	3.39305	0.196404
Ciclev10031038m.g,Ciclev10033565m.g	scaffold_4:1553214-1585121	14.2966	16.3821	0.196455
Ciclev10014035m.g	scaffold_2:36127937-36140106	33.0379	37.8576	0.19646
Ciclev10033027m.g	scaffold_4:17939218-17942908	44.6531	51.1706	0.196555
Ciclev10027815m.g	scaffold_8:24202383-24209439	82.3407	94.3601	0.196571
Ciclev10005778m.g	scaffold_9:21206590-21221111	14.8149	16.9825	0.196996
Ciclev10016587m.g	scaffold_2:29204679-29206501	0.357673	0.410032	0.197093
Ciclev10011472m.g	scaffold_6:21236902-21242261	67.2595	77.1088	0.197157
Ciclev10011125m.g	scaffold_6:6297302-6308052	35.4881	40.6867	0.197221
Ciclev10016079m.g	scaffold_2:8712235-8714558	30.5121	34.9833	0.197285
Ciclev10015670m.g	scaffold_2:30418999-30425113	38.7318	44.4089	0.197332
Ciclev10024090m.g	scaffold_3:40504072-40719738	29.692	34.0448	0.197358
Ciclev10025091m.g	scaffold_7:8371476-8376846	7.52007	8.62306	0.197455
Ciclev10010838m.g	scaffold_1:464202-470745	22.3695	25.6513	0.1975
Ciclev10014841m.g	scaffold_2:35818326-35822366	20.3012	23.2804	0.197552
Ciclev10031904m.g	scaffold_4:25485644-25491982	11.0996	12.73	0.197725
Ciclev10024728m.g	scaffold_7:19119061-19129862	16.3374	18.7394	0.197893
Ciclev10016117m.g	scaffold_2:29754007-29757232	5.60424	6.42851	0.197964
Ciclev10028809m.g	scaffold_8:16457124-16460388	39.0164	44.7641	0.198261
Ciclev10010939m.g	scaffold_6:16770266-16774127	0.413566	0.474555	0.198457
Ciclev10007978m.g	scaffold_1:22145494-22148716	0.818122	0.938819	0.198532
Ciclev10028634m.g	scaffold_8:8192741-8195885	43.1597	49.5317	0.198665
Ciclev10002428m.g	scaffold_5:40969733-40971025	33.3632	38.2916	0.19877
Ciclev10028023m.g	scaffold_8:5690694-5697304	66.2747	76.0689	0.19885
Ciclev10004777m.g	scaffold_9:30490330-30500959	21.1691	24.3014	0.199078
Ciclev10025481m.g	scaffold_7:1956586-1963890	14.1516	16.2508	0.199542
Ciclev10020022m.g	scaffold_3:44921431-44923178	16.4056	18.8404	0.199643
Ciclev10000204m.g	scaffold_5:39682995-39692504	14.153	16.2544	0.199718
Ciclev10031936m.g	scaffold_4:8078327-8081499	9.62079	11.0501	0.199826
Ciclev10011541m.g	scaffold_6:24626609-24630638	8.54271	9.81326	0.200039
Ciclev10006148m.g	scaffold_9:1844419-1846037	14.7619	16.958	0.200088
Ciclev10019201m.g	scaffold_3:8836496-8843394	28.5906	32.8445	0.200113
Ciclev10015261m.g,Ciclev10017431m.g	scaffold_2:5650654-5706559	13.7066	15.7467	0.200178
Ciclev10011319m.g	scaffold_6:21280302-21287360	26.9372	30.95	0.20034
Ciclev10008847m.g	scaffold_1:25427130-25431061	19.961	22.937	0.20049
Ciclev10005749m.g	scaffold_9:45494-48428	28.0491	32.2344	0.200648

Ciclev10000081m.g	scaffold_5:31411457-31441415	5.12488	5.8899	0.200725
Ciclev10002036m.g	scaffold_5:4432742-4458080	29.7507	34.1923	0.200745
Ciclev10015387m.g	scaffold_2:33418518-33420477	29.6038	34.0235	0.20075
Ciclev10004809m.g	scaffold_9:29562920-29568705	26.1392	30.0429	0.200806
Ciclev10027854m.g	scaffold_8:3649395-3652892	16.1744	18.5914	0.200922
Ciclev10017189m.g	scaffold_2:11903289-11903833	0.98599	1.13342	0.201032
Ciclev10010941m.g	scaffold_6:22598085-22612537	20.343	23.3852	0.201064
Ciclev10012508m.g	scaffold_6:1215581-1218314	130.341	149.837	0.201108
Ciclev10015481m.g	scaffold_2:27969463-27972032	1.71197	1.96828	0.201282
Ciclev10016069m.g	scaffold_2:32640742-32643349	33.1242	38.0839	0.201292
Ciclev10006443m.g,Ciclev10006507m.g	scaffold_9:24356661-24541601	11.5236	13.2492	0.201315
Ciclev10032912m.g	scaffold_4:16373248-16375305	186.888	214.898	0.201478
Ciclev10023911m.g	scaffold_3:36692324-36699752	2.21322	2.54497	0.201499
Ciclev10008203m.g	scaffold_1:8539646-8542949	12.0738	13.884	0.201542
Ciclev10007592m.g	scaffold_1:3616387-3622116	17.3314	19.9301	0.201564
Ciclev10012810m.g	scaffold_6:7824440-7826643	3.99995	4.59991	0.201621
Ciclev10000671m.g	scaffold_5:38177342-38180338	14.1466	16.2686	0.201628
Ciclev10002129m.g	scaffold_5:6680053-6681731	1777.69	2044.39	0.201671
Ciclev10021024m.g	scaffold_3:29277094-29281622	5.34888	6.15144	0.201688
Ciclev10000843m.g	scaffold_5:32695469-32699067	7.97419	9.17091	0.201727
Ciclev10025527m.g	scaffold_7:6739864-6744254	86.0696	98.9922	0.201811
Ciclev10008104m.g	scaffold_1:27798570-27800722	4.12133	4.7402	0.201838
Ciclev10030991m.g	scaffold_4:6341872-6358140	23.5992	27.1434	0.201862
Ciclev10002190m.g	scaffold_5:17895311-17898301	6.87784	7.9121	0.202106
Ciclev10008845m.g	scaffold_1:16406293-16411535	20.2099	23.253	0.20236
Ciclev10008406m.g	scaffold_1:8369163-8373525	48.6438	55.9705	0.202412
Ciclev10028642m.g	scaffold_8:3894265-3897671	26.1167	30.0529	0.20253
Ciclev10003187m.g	scaffold_5:42498451-42501027	0.728587	0.838436	0.202601
Ciclev10020474m.g	scaffold_3:7767514-7771356	7.43805	8.56013	0.202708
Ciclev10020533m.g	scaffold_3:10501002-10502526	8.48137	9.76094	0.202723
Ciclev10017903m.g	scaffold_2:8863229-8866953	3.76192	4.32953	0.202741
Ciclev10011222m.g	scaffold_6:11205924-11209239	0.44102	0.507605	0.202863
Ciclev10000871m.g	scaffold_5:35574051-35577732	12.5826	14.4831	0.202945
Ciclev10006144m.g	scaffold_9:5757685-5759195	9.44375	10.8704	0.202977
Ciclev10001269m.g	scaffold_5:33377661-33380733	23.7953	27.3905	0.202996
Ciclev10028210m.g	scaffold_8:21357002-21359868	3.24907	3.74008	0.203043
Ciclev10026280m.g	scaffold_7:678522-685008	1.98299	2.28268	0.203047
Ciclev10004861m.g	scaffold_9:26821278-26827367	3.29856	3.79726	0.203121
Ciclev10027778m.g	scaffold_8:812790-822376	14.2158	16.3669	0.203288
Ciclev10001939m.g	scaffold_5:42432895-42436163	34.0864	39.2451	0.203315
Ciclev10011194m.g	scaffold_6:10233537-10237433	7.87657	9.06962	0.203474
Ciclev10009771m.g	scaffold_1:2943952-2946933	22.2985	25.6764	0.203492
Ciclev10016569m.g	scaffold_2:22759340-22763218	34.8984	40.1877	0.203593
Ciclev10004551m.g	scaffold_9:4481898-4485272	12.0449	13.8717	0.203726
Ciclev10004860m.g	scaffold_9:5659650-5670438	24.2848	27.97	0.203825
Ciclev10001754m.g	scaffold_5:36175339-36178853	9.64484	11.1091	0.203918
Ciclev10005663m.g	scaffold_9:3961722-3964463	166.237	191.493	0.204053
Ciclev10018462m.g	scaffold_3:2011319-2026931	8.34321	9.61134	0.204134
Ciclev10020377m.g,Ciclev10020828m.g,Ciclev10024081m.g	scaffold_3:39891271-39925345	118.353	136.344	0.204149
Ciclev10032042m.g	scaffold_4:17048346-17052031	37.6546	43.3796	0.204192
Ciclev10015276m.g	scaffold_2:8528097-8536188	13.2145	15.2239	0.204217
Ciclev10022133m.g	scaffold_3:7256350-7258460	31.0341	35.7562	0.20434
Ciclev10021764m.g	scaffold_3:45758442-45764419	93.9246	108.219	0.204382

Ciclev10013795m.g	scaffold_6:21868596-21876620	14.581	16.8016	0.204505
Ciclev10004724m.g	scaffold_9:6837534-6845412	39.6365	45.6839	0.204854
Ciclev10027944m.g	scaffold_8:22261407-22266208	6.9305	7.98989	0.205217
Ciclev10020136m.g	scaffold_3:470593-473348	33.4293	38.54	0.205241
Ciclev10004236m.g	scaffold_9:17458637-17465570	11.1717	12.8802	0.205299
Ciclev10019354m.g	scaffold_3:2117636-2120558	1.71855	1.98137	0.205305
Ciclev10005012m.g	scaffold_9:23476660-23480861	7.80113	8.99537	0.2055
Ciclev10002450m.g	scaffold_5:40938164-40941920	78.6766	90.7252	0.205568
Ciclev10007391m.g	scaffold_1:1128688-1137063	59.5166	68.6338	0.205628
Ciclev10002215m.g	scaffold_5:28813012-28816638	65.7838	75.8653	0.205707
Ciclev10005826m.g	scaffold_9:13687750-13690455	7.18922	8.29147	0.205793
Ciclev10028050m.g	scaffold_8:1628722-1634372	23.9984	27.6794	0.205875
Ciclev10014319m.g	scaffold_2:35354148-35363026	46.0306	53.0989	0.206089
Ciclev10030522m.g	scaffold_4:24048817-24059799	14.8178	17.0953	0.206267
Ciclev10010331m.g	scaffold_1:1180096-1197884	20.9716	24.1991	0.206515
Ciclev10030712m.g	scaffold_4:12876959-12888609	6.03735	6.96663	0.206544
Ciclev10000750m.g	scaffold_5:34301652-34308746	10.3751	11.9739	0.206769
Ciclev10031028m.g	scaffold_4:15042762-15048013	22.0822	25.4891	0.206995
Ciclev10022215m.g	scaffold_3:13810496-13813004	55.7216	64.3231	0.207099
Ciclev10027732m.g	scaffold_8:1736190-1748494	10.7472	12.4083	0.207337
Ciclev10025720m.g	scaffold_7:15263395-15267892	170.877	197.31	0.207509
Ciclev10000109m.g	scaffold_5:592297-601081	0.925084	1.0682	0.207519
Ciclev10007397m.g	scaffold_1:25283806-25291472	2.44261	2.82061	0.207582
Ciclev10004232m.g	scaffold_9:11208947-11212521	7.17925	8.29105	0.207721
Ciclev10005587m.g	scaffold_9:13309143-13311747	14.2504	16.4578	0.207768
Ciclev10031092m.g	scaffold_4:18168861-18172610	42.4069	48.9838	0.208007
Ciclev10002513m.g	scaffold_5:19784724-19787578	39.3924	45.5033	0.208055
Ciclev10008476m.g	scaffold_1:385468-400033	110.147	127.262	0.208376
Ciclev10012061m.g	scaffold_6:23139261-23143611	7.15639	8.2686	0.20841
Ciclev10014791m.g	scaffold_2:19305432-19309405	58.4441	67.5278	0.208425
Ciclev10011965m.g	scaffold_6:20053810-20059518	12.2605	14.1671	0.208524
Ciclev10029374m.g	scaffold_8:24658685-24660267	28.0961	32.4659	0.208553
Ciclev10032636m.g	scaffold_4:25392819-25394708	164.09	189.624	0.208653
Ciclev10033638m.g	scaffold_4:11948587-11948791	178.538	206.323	0.208677
Ciclev10001793m.g	scaffold_5:30672426-30675502	60.2368	69.6133	0.208716
Ciclev10000092m.g	scaffold_5:38499023-38504883	4.86332	5.62057	0.208775
Ciclev10021526m.g	scaffold_3:49256305-49267922	4.48275	5.18105	0.20886
Ciclev10022655m.g	scaffold_3:40217787-40218744	15.3226	17.7096	0.208872
Ciclev10025040m.g	scaffold_7:1570331-1577684	21.0134	24.2883	0.20895
Ciclev10012294m.g	scaffold_6:15434941-15438427	17.3405	20.0443	0.209047
Ciclev10019524m.g,Ciclev10023521m.g	scaffold_3:49256305-49267922	53.0972	61.385	0.209251
Ciclev10018737m.g	scaffold_3:2355790-2361747	20.6701	23.8993	0.209428
Ciclev10032828m.g	scaffold_4:25415784-25418275	30.7382	35.5445	0.209596
Ciclev10002911m.g	scaffold_5:37128053-37130122	159.131	184.043	0.20982
Ciclev10024818m.g	scaffold_7:155385-164087	15.1918	17.5702	0.20984
Ciclev10028869m.g	scaffold_8:22280720-22284091	77.2989	89.4066	0.209933
Ciclev10017174m.g	scaffold_2:32425084-32426996	94.2441	109.022	0.210146
Ciclev10010805m.g	scaffold_1:8601165-8603225	5.0177	5.80496	0.210261
Ciclev10028628m.g	scaffold_8:545472-547304	27.7529	32.1123	0.210487
Ciclev10025230m.g	scaffold_7:16089142-16100419	21.5157	24.898	0.210638
Ciclev10018888m.g,Ciclev10020931m.g	scaffold_3:17741606-17758090	43.3793	50.2021	0.210742
Ciclev10033914m.g	scaffold_4:25071208-25077049	12.869	14.8959	0.21101
Ciclev10008607m.g	scaffold_1:1608394-1612445	7.37377	8.53531	0.211041
Ciclev10031613m.g	scaffold_4:7389825-7393924	4.40825	5.10347	0.211273

Ciclev10030476m.g	scaffold_4:25195793-25208811	20.7171	23.9844	0.211274
Ciclev10026858m.g	scaffold_7:9278684-9279195	272.539	315.531	0.211317
Ciclev10000087m.g	scaffold_5:41811141-41820743	43.5868	50.4667	0.21144
Ciclev10032286m.g	scaffold_4:20031655-20034465	10.8231	12.5333	0.211659
Ciclev10018979m.g	scaffold_3:4315373-4320978	12.3323	14.2814	0.2117
Ciclev10021170m.g	scaffold_3:14993578-14994805	587.192	680.014	0.211731
Ciclev10015087m.g	scaffold_2:29720005-29725465	44.2939	51.297	0.211766
Ciclev10015116m.g	scaffold_2:23318204-23323330	13.9716	16.1813	0.211821
Ciclev10028092m.g	scaffold_8:22217855-22223396	25.9751	30.0851	0.211921
Ciclev10007477m.g	scaffold_1:10340898-10345774	27.0888	31.376	0.211964
Ciclev10016087m.g	scaffold_2:13799455-13802309	16.0993	18.6505	0.21222
-	scaffold_1:19111708-19111854	753.299	872.878	0.212557
Ciclev10007191m.g	scaffold_9:28620532-28622110	4.18235	4.84692	0.212756
Ciclev10032463m.g	scaffold_4:22519740-22522957	21.1359	24.4962	0.212865
Ciclev10009039m.g	scaffold_1:4371292-4375983	25.4494	29.4978	0.212972
Ciclev10017175m.g	scaffold_2:30239710-30241487	63.5594	73.6757	0.213082
Ciclev10003566m.g	scaffold_5:3972007-3975173	150.501	174.474	0.213238
Ciclev10018461m.g	scaffold_3:7944701-7956243	11.2115	12.9981	0.213328
Ciclev10032291m.g	scaffold_4:1819353-1822508	69.7653	80.8878	0.213413
Ciclev10014558m.g	scaffold_2:9171633-9176184	12.5554	14.5572	0.213426
Ciclev10018441m.g	scaffold_3:49001401-49013646	17.1751	19.9141	0.213466
Ciclev10028443m.g	scaffold_8:22316998-22319482	85.3984	99.0293	0.213646
Ciclev10019514m.g	scaffold_3:42787844-42792527	14.1422	16.4002	0.213702
Ciclev10026654m.g	scaffold_7:10772696-10776886	57.3944	66.5661	0.213878
Ciclev10006964m.g	scaffold_9:4545006-4546975	1.30913	1.5186	0.214134
Ciclev10016393m.g	scaffold_2:27002919-27008945	54.3197	63.0119	0.214149
Ciclev10005326m.g	scaffold_9:8621402-8627840	38.4033	44.5489	0.214156
Ciclev10007884m.g	scaffold_1:25161783-25168552	6.36705	7.38621	0.21421
Ciclev10014563m.g	scaffold_2:27074281-27084009	7.31864	8.49027	0.214236
Ciclev10012921m.g	scaffold_6:19606302-19607695	38.0303	44.1227	0.21437
Ciclev10019496m.g	scaffold_3:33154203-33159959	10.4916	12.1736	0.214522
Ciclev10005512m.g	scaffold_9:24661017-24664935	14.9772	17.3788	0.21456
Ciclev10007726m.g	scaffold_1:1478171-1484977	5.93879	6.89171	0.214693
Ciclev10024172m.g	scaffold_3:49209813-49213513	56.964	66.1156	0.214942
Ciclev10027704m.g	scaffold_8:24777617-24783626	59.6667	69.2547	0.214985
Ciclev10005020m.g	scaffold_9:21629106-21634720	20.84	24.191	0.215117
Ciclev10032222m.g	scaffold_4:1586789-1589604	23.5055	27.2868	0.215202
Ciclev10002599m.g	scaffold_5:39153138-39156117	58.9355	68.4308	0.215511
Ciclev10012490m.g	scaffold_6:18738240-18741082	7.03028	8.16321	0.215554
Ciclev10031251m.g	scaffold_4:22059106-22062285	2.02658	2.35334	0.215663
Ciclev10030704m.g	scaffold_4:22827700-22833845	13.5455	15.7321	0.215901
Ciclev10018711m.g	scaffold_3:40504072-40719738	39.8041	46.2308	0.215936
Ciclev10007597m.g	scaffold_1:1524403-1527971	8.38671	9.7419	0.216099
Ciclev10024221m.g	scaffold_3:32543167-32579333	0.868976	1.0094	0.21611
Ciclev10019088m.g	scaffold_3:1649409-1655963	15.4838	17.9907	0.216493
Ciclev10019622m.g	scaffold_3:6372342-6373999	2.2001	2.55642	0.216556
Ciclev10003346m.g	scaffold_5:34020542-34022577	3.31686	3.85427	0.216638
Ciclev10007714m.g	scaffold_1:13325812-13336158	17.9839	20.9013	0.21688
Ciclev10027450m.g	scaffold_7:4056604-4060419	11.5705	13.4479	0.216938
Ciclev10023145m.g	scaffold_3:49085995-49088292	4.9222	5.72112	0.216993
Ciclev10033814m.g	scaffold_4:14327980-14329768	0.914173	1.06269	0.217181
Ciclev10019800m.g	scaffold_3:2710778-2715959	43.2724	50.3034	0.217207
Ciclev10020067m.g	scaffold_3:40994991-41001689	0.86672	1.00756	0.217234
Ciclev10030198m.g,Ciclev10030386m.g	scaffold_8:22575352-22578576	837.815	973.996	0.217284
Ciclev10004153m.g	scaffold_9:1641885-1648191	1.61629	1.87912	0.217371

Ciclev10015509m.g	scaffold_2:34293043-34297757	18.3047	21.2814	0.217381
Ciclev10021651m.g	scaffold_3:6602993-6605239	13.0808	15.2083	0.217403
Ciclev10033195m.g	scaffold_4:6891394-6897982	24.6775	28.6913	0.21742
Ciclev10027768m.g	scaffold_8:6047426-6054970	9.0406	10.5122	0.217568
-	scaffold_2:6124497-6129530	6.92415	8.05163	0.217644
Ciclev10007580m.g	scaffold_1:2214707-2222674	21.9027	25.4693	0.21765
Ciclev10014864m.g	scaffold_2:23645930-23655043	4.71713	5.48582	0.217796
Ciclev10007244m.g	scaffold_1:4545341-4564754	8.90105	10.3527	0.217961
Ciclev10000208m.g	scaffold_5:5978131-5984111	10.2715	11.9479	0.218119
Ciclev10002376m.g	scaffold_5:12183280-12186007	24.2505	28.2114	0.218264
Ciclev10016866m.g	scaffold_2:8494368-8499151	97.076	112.936	0.218312
Ciclev10033590m.g,Ciclev10033693m.g	scaffold_4:5321382-5331172	16.3707	19.0456	0.21834
Ciclev10015593m.g	scaffold_2:9961645-9964755	2.25462	2.62311	0.218393
Ciclev10000052m.g	scaffold_5:27564184-27578397	11.4729	13.3481	0.218403
Ciclev10026443m.g	scaffold_7:5635747-5639705	21.7201	25.2713	0.218469
Ciclev10012081m.g	scaffold_6:7489745-7495735	32.8481	38.2198	0.21851
Ciclev10023801m.g	scaffold_3:40452724-40470798	10.8964	12.6798	0.218686
Ciclev10028157m.g	scaffold_8:24326658-24329628	134.373	156.375	0.218766
Ciclev10015103m.g	scaffold_2:27137088-27140655	8.19272	9.53563	0.218985
Ciclev10025980m.g	scaffold_7:399184-411768	9.08076	10.5694	0.219014
Ciclev10000650m.g	scaffold_5:21243492-21247521	3.04418	3.54334	0.219056
Ciclev10013190m.g	scaffold_6:2549466-2552655	63.1485	73.5065	0.219123
Ciclev10004676m.g	scaffold_9:19607998-19615090	42.3785	49.3314	0.219174
-	scaffold_8:8099918-8100882	6.65495	7.74785	0.219369
Ciclev10015841m.g	scaffold_2:33557296-33561230	6.77558	7.88844	0.219395
Ciclev10002917m.g	scaffold_5:35357332-35359897	69.5433	80.9701	0.219477
Ciclev10031852m.g	scaffold_4:10500892-10508644	16.1926	18.8561	0.219699
Ciclev10025837m.g	scaffold_7:4733123-4737712	10.4042	12.1187	0.220079
-	scaffold_5:8159352-8216590	29.8438	34.7733	0.220548
Ciclev10018749m.g	scaffold_3:48173347-48182555	18.7706	21.8715	0.220577
Ciclev10007600m.g	scaffold_1:24317383-24322316	13.7781	16.0584	0.22095
Ciclev10019027m.g	scaffold_3:26517175-26525778	16.1958	18.8773	0.221039
Ciclev10028483m.g	scaffold_8:19779794-19784134	60.2868	70.2689	0.221044
Ciclev10011329m.g	scaffold_6:19223624-19230870	85.147	99.2574	0.221218
Ciclev10011323m.g	scaffold_6:17700680-17703452	40.2276	46.9012	0.221441
Ciclev10013091m.g	scaffold_6:22623524-22624276	9.695	11.3035	0.221461
Ciclev10007878m.g	scaffold_1:18035461-18040895	30.1401	35.1424	0.221525
Ciclev10009327m.g	scaffold_1:8015797-8020208	33.2877	38.8135	0.22157
Ciclev10031560m.g	scaffold_4:23038832-23041997	14.8488	17.3139	0.221581
Ciclev10031182m.g	scaffold_4:9457971-9466814	13.5029	15.7472	0.221831
Ciclev10032032m.g	scaffold_4:858604-862666	18.3702	21.4248	0.221913
Ciclev10014643m.g	scaffold_2:26197549-26202950	32.5625	37.978	0.221953
Ciclev10029428m.g	scaffold_8:24044995-24049924	248.568	289.934	0.222086
Ciclev10016096m.g	scaffold_2:10391378-10392539	3.56891	4.163	0.22214
Ciclev10022411m.g	scaffold_3:2184021-2186634	19.3762	22.6025	0.222193
Ciclev10032704m.g	scaffold_4:24248593-24250926	60.9571	71.109	0.222238
Ciclev10021060m.g	scaffold_3:793626-796219	213.632	249.216	0.222272
Ciclev10028175m.g	scaffold_8:16594751-16602473	7.65767	8.93346	0.222313
Ciclev10008309m.g	scaffold_1:22862772-22866389	15.0802	17.5945	0.222467
Ciclev10007513m.g	scaffold_1:7218501-7227996	29.6299	34.5712	0.22252
Ciclev10019328m.g	scaffold_3:39335623-39337978	11.0204	12.8591	0.22261
Ciclev10016308m.g	scaffold_2:21140781-21142910	76.8106	89.6342	0.222744
Ciclev10018692m.g	scaffold_3:9026019-9036386	12.8886	15.0416	0.222857
Ciclev10000821m.g	scaffold_5:37429289-37433447	55.8017	65.13	0.223014
Ciclev10033899m.g	scaffold_4:23439180-23445715	3.58962	4.18976	0.223034

Ciclev10000938m.g	scaffold_5:37261673-37266754	31.4773	36.7405	0.22306
Ciclev10005007m.g	scaffold_9:2670703-2676173	800.363	934.21	0.223091
Ciclev10011251m.g	scaffold_6:21958580-21963604	16.0062	18.6855	0.223288
Ciclev10029368m.g	scaffold_8:24288412-24289588	7.16249	8.36145	0.223292
Ciclev10029250m.g	scaffold_8:18835413-18837272	4.94905	5.77826	0.223484
Ciclev10020368m.g	scaffold_3:42972569-42974767	22.5331	26.3102	0.223576
-	scaffold_5:3980660-4069314	16.9659	19.8111	0.223671
Ciclev10000885m.g	scaffold_5:28802464-28809416	16.8036	19.6224	0.223732
Ciclev10019034m.g	scaffold_3:8914743-8921411	50.7574	59.2723	0.223741
Ciclev10007328m.g	scaffold_1:16565239-16568883	4.3783	5.11282	0.223748
Ciclev10001069m.g	scaffold_5:42985724-42990116	21.6902	25.3297	0.223787
Ciclev10020047m.g	scaffold_3:572549-576969	21.1616	24.7131	0.22383
Ciclev10015678m.g	scaffold_2:12301199-12304346	121	141.328	0.224032
Ciclev10009455m.g	scaffold_1:2888991-2892435	34.6442	40.4644	0.224041
Ciclev10030127m.g	scaffold_8:2854048-2856501	69.1956	80.8377	0.224349
Ciclev10031047m.g	scaffold_4:19476506-19485460	51.5115	60.1811	0.224417
Ciclev10025777m.g	scaffold_7:6977243-6983565	23.2992	27.2224	0.224515
Ciclev10016567m.g	scaffold_2:5958601-5961642	0.589121	0.688347	0.224572
Ciclev10003382m.g	scaffold_5:29849433-29857454	11.0706	12.9371	0.224781
Ciclev10014752m.g	scaffold_2:32780829-32782885	16.9666	19.8298	0.22497
Ciclev10012337m.g	scaffold_6:23502066-23506087	17.8926	20.9142	0.22512
Ciclev10028497m.g	scaffold_8:8033464-8038415	34.1015	39.8689	0.225432
Ciclev10017155m.g	scaffold_2:26105694-26158658	1.09296	1.27783	0.225462
Ciclev10027997m.g	scaffold_8:1087407-1089792	1.66331	1.94483	0.225594
Ciclev10029426m.g	scaffold_8:23010401-23012827	92.148	107.746	0.225613
Ciclev10030915m.g,Ciclev10030917m.g	scaffold_4:21870658-21895003	22.5287	26.3458	0.225808
Ciclev10011685m.g	scaffold_6:25174471-25176263	3.82682	4.47552	0.22591
Ciclev10025679m.g	scaffold_7:7973813-7977437	69.0628	80.7788	0.226068
Ciclev10028671m.g	scaffold_8:9883297-9891933	27.9859	32.7347	0.22612
Ciclev10000901m.g	scaffold_5:4461970-4470737	19.7606	23.1196	0.226487
Ciclev10028098m.g	scaffold_8:180850-185769	17.6502	20.6507	0.226502
Ciclev10004171m.g	scaffold_9:1794859-1799055	20.5632	24.0594	0.226542
Ciclev10013261m.g	scaffold_6:18210469-18212104	38.99	45.6218	0.226619
Ciclev10029627m.g	scaffold_8:5505610-5507405	423.88	496.035	0.226787
Ciclev10022600m.g	scaffold_3:26618335-26621274	89.0715	104.249	0.226991
Ciclev10000118m.g	scaffold_5:40253859-40262178	2.2329	2.61367	0.227155
Ciclev10030352m.g	scaffold_8:16629845-16632720	40.4983	47.4067	0.22723
Ciclev10018482m.g	scaffold_3:45704360-45713084	74.9245	87.7175	0.227426
Ciclev10031990m.g	scaffold_4:20914139-20917244	32.8912	38.5077	0.227443
Ciclev10027964m.g	scaffold_8:22912635-22918907	35.093	41.091	0.227636
Ciclev10006463m.g,Ciclev10006528m.g	scaffold_9:14339251-14343054	31.8492	37.2946	0.22771
Ciclev10014268m.g	scaffold_2:33325001-33329313	20.0814	23.52	0.228028
Ciclev10001708m.g	scaffold_5:36627643-36631386	3.4625	4.05557	0.22809
Ciclev10007460m.g	scaffold_1:27098792-27102412	31.0267	36.3426	0.228153
Ciclev10014785m.g	scaffold_2:32020958-32023334	145.079	169.941	0.228194
Ciclev10024924m.g	scaffold_7:7743507-7749913	13.21	15.4759	0.228395
Ciclev10001026m.g	scaffold_5:22571031-22573220	10.7135	12.5516	0.228439
Ciclev10002691m.g	scaffold_5:38770274-38772207	35.526	41.6217	0.228458
Ciclev10023812m.g	scaffold_3:39316767-39318533	0.394626	0.462378	0.228587
Ciclev10020370m.g	scaffold_3:50697007-50700171	14.1202	16.5477	0.228872
Ciclev10002308m.g	scaffold_5:12201829-12206033	38.9726	45.6738	0.228907
Ciclev10031113m.g	scaffold_4:3786289-3793916	13.6485	15.9983	0.229179
Ciclev10026251m.g	scaffold_7:4283861-4286784	85.0984	99.7791	0.229605
Ciclev10000072m.g	scaffold_5:30967584-30977437	5.16444	6.05616	0.229789

Ciclev10024933m.g	scaffold_7:15236918-15249870	10.5553	12.3795	0.229991
Ciclev10024166m.g,Ciclev10024328m.g	scaffold_3:6565320-6568570	23.9844	28.1314	0.230085
Ciclev10031219m.g	scaffold_4:13811072-13816892	23.7925	27.9108	0.230316
Ciclev10005792m.g	scaffold_9:13545136-13548134	23.6495	27.7465	0.230498
Ciclev10028324m.g	scaffold_8:309616-317158	32.2494	37.8381	0.230567
Ciclev10004842m.g	scaffold_9:4010425-4014599	16.1219	18.9162	0.230602
Ciclev10031722m.g	scaffold_4:2246671-2250342	21.8969	25.6929	0.230645
Ciclev10033010m.g	scaffold_4:22720916-22723663	20.3824	23.9183	0.230788
Ciclev10017037m.g	scaffold_2:34990218-34992028	17.9853	21.1066	0.230872
Ciclev10023466m.g	scaffold_3:42299597-42302284	107.204	125.812	0.230907
Ciclev10004900m.g	scaffold_9:28767081-28768852	4.84864	5.69048	0.23097
Ciclev10026700m.g	scaffold_7:46003-47512	21.2667	24.961	0.231085
Ciclev10018712m.g	scaffold_3:5344964-5350968	7.36198	8.64383	0.231578
Ciclev10002832m.g	scaffold_5:30555223-30556028	2.83993	3.33451	0.231621
Ciclev10020526m.g	scaffold_3:17555201-17556944	1.74687	2.05123	0.231719
Ciclev10009930m.g	scaffold_1:8392027-8398431	136.184	159.948	0.232043
Ciclev10025862m.g	scaffold_7:2211104-2215666	37.0114	43.4837	0.232507
Ciclev10011515m.g	scaffold_6:22744048-22746590	15.1021	17.745	0.232668
Ciclev10007230m.g	scaffold_1:106601-115752	10.7251	12.6023	0.232697
Ciclev10024375m.g	scaffold_3:263117-269684	15.875	18.6555	0.232838
Ciclev10030701m.g	scaffold_4:15528850-15538252	16.748	19.6824	0.232917
Ciclev10008680m.g	scaffold_1:7720491-7722440	13.576	15.9569	0.233128
Ciclev10000045m.g	scaffold_5:42809559-42821401	0.395341	0.464699	0.233197
Ciclev10019590m.g	scaffold_3:47139755-47144124	45.306	53.2667	0.233532
Ciclev10001629m.g	scaffold_5:23886542-23992055	30.3993	35.7424	0.233595
Ciclev10004129m.g	scaffold_9:12212754-12226099	7.02869	8.26518	0.233791
Ciclev10015637m.g	scaffold_2:19824011-19826726	30.0078	35.2897	0.233912
Ciclev10032427m.g	scaffold_4:23596433-23599906	59.0705	69.4738	0.234032
Ciclev10012038m.g	scaffold_6:25511427-25520662	7.51319	8.83673	0.234087
Ciclev10001043m.g	scaffold_5:40180739-40184016	27.5343	32.3872	0.234195
Ciclev10032129m.g	scaffold_4:23498186-23499805	7.91033	9.30472	0.234225
Ciclev10025049m.g	scaffold_7:294299-299476	3.79257	4.46185	0.234466
Ciclev10028673m.g	scaffold_8:2070691-2073676	9.9318	11.687	0.23478
Ciclev10014513m.g	scaffold_2:30292431-30296556	3.09613	3.64335	0.234802
Ciclev10033595m.g	scaffold_4:25591539-25593417	272.051	320.147	0.234855
-	scaffold_9:8921513-8921731	34.6446	40.7699	0.234874
-	scaffold_3:41539709-41543555	29.7379	34.9975	0.234952
Ciclev10004240m.g	scaffold_9:24858257-24873775	35.1806	41.4048	0.235016
Ciclev10012335m.g	scaffold_6:20486955-20489702	61.7501	72.6775	0.235067
Ciclev10028226m.g	scaffold_8:20309895-20313442	63.6072	74.8654	0.235109
Ciclev10020897m.g	scaffold_3:3937928-3945638	116.782	137.458	0.235177
Ciclev10029406m.g	scaffold_8:19566455-19567126	206.387	242.932	0.235199
Ciclev10007254m.g	scaffold_1:24301672-24308649	20.5203	24.157	0.235387
Ciclev10011553m.g	scaffold_6:6280767-6288163	6.01108	7.07651	0.235413
Ciclev10026609m.g	scaffold_7:18539346-18543298	488.451	575.027	0.235417
Ciclev10004799m.g	scaffold_9:1968170-1975282	21.6585	25.4974	0.235419
Ciclev10018728m.g	scaffold_3:2603073-2615687	11.7161	13.7944	0.235592
Ciclev10016555m.g	scaffold_2:8445988-8450370	174.921	205.954	0.235613
Ciclev10008632m.g	scaffold_1:4642935-4652060	19.8569	23.3797	0.235619
Ciclev10022769m.g	scaffold_3:41391592-41394798	8.72305	10.2725	0.235884
Ciclev10007623m.g	scaffold_1:28156954-28162994	15.1227	17.8104	0.236003
Ciclev10014539m.g	scaffold_2:35346197-35348518	4.357	5.13149	0.236042
Ciclev10011273m.g	scaffold_6:23735744-23738642	2.11661	2.49288	0.236059
Ciclev10009995m.g	scaffold_1:28211600-28214921	29.345	34.5623	0.236084
Ciclev10002702m.g	scaffold_5:29670606-29674529	18.5407	21.8384	0.236171

Ciclev10004358m.g	scaffold_9:14797053-14807343	17.552	20.6755	0.236287
Ciclev10015255m.g	scaffold_2:31282647-31286095	33.736	39.7419	0.236371
Ciclev10011267m.g	scaffold_6:11166388-11168314	0.286124	0.337062	0.236376
Ciclev10011536m.g	scaffold_6:15879060-15881796	29.5105	34.7704	0.236631
Ciclev10025611m.g	scaffold_7:17491173-17497237	61.323	72.2549	0.236668
Ciclev10009319m.g	scaffold_1:25659550-25663375	7.74035	9.12065	0.236738
Ciclev10024333m.g	scaffold_3:6320151-6320945	5.15567	6.07505	0.236738
Ciclev10011869m.g	scaffold_6:19271061-19274322	21.4465	25.2716	0.236773
Ciclev10022425m.g	scaffold_3:50850795-50851945	9.91008	11.678	0.23682
Ciclev10018788m.g	scaffold_3:41254855-41267108	29.6242	34.9156	0.237097
Ciclev10018925m.g	scaffold_3:46519382-46595230	28.552	33.6525	0.237121
Ciclev10026692m.g	scaffold_7:11850299-12160617	1.92136	2.2646	0.237127
Ciclev10033216m.g	scaffold_4:15927575-15929918	38.5094	45.3905	0.237178
Ciclev10029584m.g	scaffold_8:25006510-25009433	10.7685	12.6931	0.237233
Ciclev10000573m.g	scaffold_5:38609929-38615996	24.6839	29.0968	0.23729
Ciclev10002768m.g	scaffold_5:36722429-36724431	296.278	349.279	0.237427
Ciclev10007967m.g	scaffold_1:28807628-28814574	37.6467	44.3839	0.237514
Ciclev10024725m.g	scaffold_7:7393481-7401845	14.4627	17.051	0.237522
Ciclev10005612m.g	scaffold_9:20890036-21029395	3.7394	4.40918	0.237704
Ciclev10000765m.g	scaffold_5:35137903-35140955	112.865	133.097	0.237882
Ciclev10031314m.g	scaffold_4:15420335-15428071	10.8575	12.8044	0.237952
Ciclev10028398m.g	scaffold_8:16691160-16692948	0.577624	0.681202	0.237953
Ciclev10016151m.g	scaffold_2:36199642-36202168	10.6987	12.6179	0.238037
Ciclev10007689m.g	scaffold_1:4666520-4671623	10.026	11.8247	0.238045
Ciclev10007238m.g	scaffold_1:1653123-1661214	13.0077	15.3424	0.238156
Ciclev10023691m.g	scaffold_3:45920124-45922741	84.6782	99.8913	0.238368
Ciclev10008965m.g	scaffold_1:27018775-27024185	63.6501	75.0857	0.238376
Ciclev10000613m.g	scaffold_5:41957505-41962278	14.0126	16.5306	0.238407
Ciclev10030679m.g	scaffold_4:21342590-21351254	10.6794	12.5983	0.238408
Ciclev10020351m.g	scaffold_3:42451354-42455877	36.964	43.6081	0.238477
Ciclev10004230m.g	scaffold_9:13853760-13858173	19.8034	23.3653	0.238616
Ciclev10001649m.g	scaffold_5:26615176-26617732	6.84446	8.07569	0.238649
Ciclev10017346m.g	scaffold_2:3067336-3069249	233.858	275.955	0.238804
Ciclev10024797m.g	scaffold_7:2001164-2009327	7.54535	8.90425	0.238905
Ciclev10014024m.g	scaffold_2:13139552-13147518	4.51308	5.32638	0.239042
Ciclev10031464m.g	scaffold_4:889355-891165	63.1713	74.5713	0.239351
Ciclev10005107m.g	scaffold_9:25184761-25188839	40.815	48.1811	0.239367
Ciclev10016126m.g	scaffold_2:34540108-34540987	0.51475	0.607703	0.239493
Ciclev10000201m.g	scaffold_5:35610473-35618268	12.8352	15.1537	0.239559
Ciclev10007273m.g	scaffold_1:18370434-18381928	13.6556	16.124	0.23972
Ciclev10031221m.g	scaffold_4:23630189-23635034	6.95046	8.20769	0.239867
Ciclev10018561m.g	scaffold_3:46608333-46620141	9.76853	11.5355	0.239868
Ciclev10030717m.g	scaffold_4:22589150-22596272	28.2079	33.3122	0.239951
Ciclev10014270m.g	scaffold_2:30458977-30470621	13.6561	16.1289	0.2401
Ciclev10032372m.g	scaffold_4:2392026-2396558	2.38074	2.81238	0.240384
Ciclev10004408m.g	scaffold_9:579334-585547	19.4607	22.9901	0.240447
Ciclev10020399m.g	scaffold_3:32812595-32816536	12.1529	14.3575	0.2405
Ciclev10010930m.g	scaffold_6:24298135-24312605	66.4913	78.5622	0.240669
Ciclev10015913m.g	scaffold_2:29540811-29545332	6.00455	7.09465	0.240675
Ciclev10030886m.g	scaffold_4:22015762-22019247	8.78744	10.3832	0.24073
Ciclev10000638m.g	scaffold_5:26493800-26495822	6.74914	7.97474	0.240734
Ciclev10008521m.g	scaffold_1:2730459-2734088	28.9054	34.1676	0.241289
Ciclev10002266m.g	scaffold_5:36441612-36445735	51.1217	60.4329	0.2414
Ciclev10005637m.g	scaffold_9:25860664-25863110	129.369	152.94	0.241482
Ciclev10021077m.g	scaffold_3:50442358-50444191	2.49483	2.94945	0.241506
Ciclev10031757m.g	scaffold_4:23789924-23791600	12.5215	14.8044	0.241616

Ciclev10015634m.g	scaffold_2:10931514-10935724	23.3999	27.667	0.241666
Ciclev10032986m.g	scaffold_4:23618423-23625625	79.4389	93.9266	0.241687
Ciclev10020519m.g	scaffold_3:28004906-28011925	304.526	360.064	0.241689
Ciclev10008533m.g	scaffold_1:14862341-14871286	14.8551	17.5649	0.241734
Ciclev10012119m.g	scaffold_6:20691106-20706907	6.01559	7.11336	0.241826
Ciclev10012716m.g	scaffold_6:17992562-17993671	126.567	149.68	0.241982
Ciclev10014997m.g	scaffold_2:29933201-29941046	30.1813	35.6934	0.242005
Ciclev10016978m.g	scaffold_2:10686094-10688635	2.85403	3.37534	0.242034
Ciclev10031986m.g	scaffold_4:18332484-18333750	0.401307	0.474691	0.242284
Ciclev10028658m.g	scaffold_8:23704798-23707343	2.76466	3.27034	0.242339
Ciclev10022897m.g	scaffold_3:3248172-3249946	72.9472	86.2966	0.242451
Ciclev10014750m.g	scaffold_2:22918673-22922359	0.90203	1.06716	0.242535
-	scaffold_5:30358489-30358877	40.3927	47.7992	0.242892
Ciclev10004984m.g	scaffold_9:10984086-10989366	59.4775	70.3847	0.242918
Ciclev10015455m.g	scaffold_2:8104261-8107647	17.4561	20.658	0.24297
Ciclev10031978m.g	scaffold_4:25245188-25248157	7.3692	8.72219	0.243181
Ciclev10020868m.g	scaffold_3:3087934-3091281	9.0123	10.6676	0.243271
Ciclev10030729m.g	scaffold_4:21146020-21149851	9.93635	11.762	0.24334
Ciclev10032854m.g	scaffold_4:7532854-7706546	3.71547	4.39862	0.243506
Ciclev10014868m.g	scaffold_2:28867593-28870392	1.15912	1.37227	0.243524
Ciclev10025194m.g	scaffold_7:4207611-4214290	15.4636	18.3082	0.243614
Ciclev10005751m.g	scaffold_9:928701-932024	1.48813	1.76213	0.243824
Ciclev10009615m.g	scaffold_1:23341433-23343890	102.563	121.453	0.243882
Ciclev10010814m.g	scaffold_1:5094263-5097815	6.19182	7.33237	0.243916
Ciclev10031651m.g	scaffold_4:2793150-2796319	4.77911	5.66068	0.244234
Ciclev10032716m.g	scaffold_4:22561084-22564628	117.464	139.171	0.244645
Ciclev10009973m.g	scaffold_1:4927379-4929417	30.0004	35.5465	0.244727
-	scaffold_3:41379080-41380163	90.7203	107.497	0.244799
Ciclev10027707m.g	scaffold_8:20005139-20013439	8.39333	9.94625	0.244909
Ciclev10005755m.g,Ciclev10006119m.g	scaffold_9:23276220-23286770	6.78291	8.03923	0.245153
-	scaffold_6:18432067-18435472	15.1912	18.0056	0.245212
Ciclev10019076m.g	scaffold_3:6223081-6226734	2.06736	2.45062	0.245359
Ciclev10028305m.g,Ciclev10030328m.g	scaffold_8:2788251-2799301	97.3066	115.349	0.245395
Ciclev10011050m.g	scaffold_6:15530057-15536534	16.0662	19.0452	0.245401
Ciclev10022091m.g	scaffold_3:44295965-44298402	25.6268	30.3837	0.245644
Ciclev10032094m.g	scaffold_4:18953017-18957933	30.1432	35.7394	0.24568
Ciclev10014158m.g	scaffold_2:32165658-32173297	14.8832	17.6463	0.245686
Ciclev10019598m.g	scaffold_3:1778483-1784053	47.7547	56.6208	0.245688
Ciclev10015026m.g	scaffold_2:29870501-29873518	1.20449	1.42828	0.245858
Ciclev10012748m.g	scaffold_6:17947972-17948902	5.16224	6.12164	0.245923
Ciclev10028990m.g	scaffold_8:1054045-1059088	177.493	210.49	0.245994
Ciclev10004373m.g	scaffold_9:17538206-17550680	18.1329	21.5055	0.246094
Ciclev10020449m.g	scaffold_3:44565761-44570397	252.469	299.427	0.246098
Ciclev10032313m.g	scaffold_4:12926863-12928851	168.839	200.308	0.24657
-	scaffold_1:18386714-18387169	5.55565	6.59163	0.24668
Ciclev10000343m.g	scaffold_5:37790947-37797703	7.48329	8.87881	0.246694
Ciclev10030408m.g	scaffold_8:23975192-23976036	4.97082	5.89783	0.246702
Ciclev10010214m.g	scaffold_1:746711-751952	4.81511	5.7143	0.247006
Ciclev10011341m.g	scaffold_6:15116859-15121106	11.8929	14.1143	0.24706
Ciclev10006110m.g,Ciclev10006553m.g	scaffold_9:12019167-12030040	5.49267	6.51906	0.247157
Ciclev10015977m.g	scaffold_2:13238936-13243015	31.3134	37.1671	0.247248
Ciclev10007311m.g	scaffold_1:25880805-25887976	34.424	40.861	0.247308
Ciclev10031319m.g	scaffold_4:12483127-12490514	18.4566	21.908	0.247323

Ciclev10026383m.g	scaffold_7:7923448-7945523	39.1011	46.4195	0.247522
Ciclev10030495m.g	scaffold_4:15847300-15861868	1.0851	1.28827	0.247606
Ciclev10021017m.g	scaffold_3:45562190-45563752	8.468	10.0541	0.247697
Ciclev10031733m.g	scaffold_4:12907048-12913671	9.96008	11.8276	0.247925
Ciclev10015105m.g	scaffold_2:8954838-8978273	19.8002	23.5152	0.24808
Ciclev10018710m.g	scaffold_3:19266870-19273247	32.8561	39.0237	0.248191
Ciclev10011674m.g	scaffold_6:9915756-9917750	16.5128	19.6151	0.248377
Ciclev10003946m.g	scaffold_5:41018415-41027150	18.8738	22.422	0.248532
Ciclev10028792m.g	scaffold_8:5870693-5875012	16.3457	19.4204	0.24867
-	scaffold_9:28294136-28294594	5.48703	6.51949	0.248733
Ciclev10028612m.g	scaffold_8:520841-522929	11.4379	13.5902	0.24875
Ciclev10019645m.g	scaffold_3:43407109-43412174	39.1156	46.4775	0.248788
Ciclev10007383m.g	scaffold_1:7771870-7776941	3.19189	3.79295	0.248908
Ciclev10015925m.g	scaffold_2:32958088-32970806	51.5147	61.2287	0.249223
Ciclev10006524m.g	scaffold_9:24205626-24207060	27.0253	32.1247	0.24937
Ciclev10031516m.g	scaffold_4:6811553-6816394	23.1602	27.5333	0.24953
Ciclev10032565m.g	scaffold_4:21517371-21520194	6.25821	7.44009	0.24957
Ciclev10001203m.g	scaffold_5:16526796-16532892	10.3471	12.3019	0.249651
Ciclev10023441m.g	scaffold_3:40437276-40448184	14.2419	16.9333	0.249722
Ciclev10012505m.g	scaffold_6:5908591-5911371	300.368	357.134	0.249733
Ciclev10029380m.g	scaffold_8:23891479-23894163	23.8419	28.3479	0.249738
Ciclev10024812m.g	scaffold_7:20088918-20094162	2.59831	3.08941	0.249756
Ciclev10033600m.g	scaffold_4:21488110-21490190	54.5181	64.8292	0.24991
Ciclev10014886m.g	scaffold_2:7692306-7694086	9.45252	11.2411	0.250018
Ciclev10008061m.g	scaffold_1:21426249-21433920	15.451	18.3748	0.25003
Ciclev10011114m.g	scaffold_6:21855277-21858461	1.04289	1.24026	0.250058
Ciclev10027890m.g	scaffold_8:16704982-16710152	2.62818	3.1258	0.250163
Ciclev10008638m.g	scaffold_1:3356191-3360411	15.1182	17.9818	0.250247
Ciclev10010686m.g	scaffold_1:3426406-3433539	20.0748	23.8801	0.250421
Ciclev10006199m.g	scaffold_9:1691733-1693590	182.656	217.281	0.250432
Ciclev10025459m.g	scaffold_7:5699211-5702589	24.8036	29.5091	0.250608
Ciclev10008468m.g	scaffold_1:25716861-25720071	91.8086	109.23	0.250669
Ciclev10008108m.g	scaffold_1:25668702-25674585	10.7705	12.8167	0.250938
Ciclev10012141m.g	scaffold_6:15368186-15370563	61.1344	72.7524	0.251009
Ciclev10007522m.g	scaffold_1:23727903-23733335	13.5359	16.1085	0.251031
Ciclev10000200m.g	scaffold_5:15888736-15896759	10.719	12.7563	0.251046
Ciclev10007720m.g	scaffold_1:27457357-27462893	39.4552	46.9569	0.251119
Ciclev10020457m.g	scaffold_3:6546646-6552193	19.9812	23.7805	0.251135
Ciclev10009660m.g	scaffold_1:891332-892160	7.02024	8.35573	0.251246
Ciclev10030602m.g	scaffold_4:4825832-5025734	11.4276	13.6023	0.251319
Ciclev10015198m.g	scaffold_2:10548129-10551974	10.6806	12.7133	0.25134
Ciclev10000298m.g	scaffold_5:42418836-42424800	129.361	153.986	0.251391
Ciclev10004285m.g	scaffold_9:1777766-1782090	7.27054	8.65468	0.251417
Ciclev10007360m.g	scaffold_1:27000664-27011542	5.58969	6.65489	0.251646
Ciclev10026802m.g,Ciclev10026903m.g	scaffold_7:13779914-13786811	8.26875	9.84457	0.25166
Ciclev10006789m.g	scaffold_9:4810424-4811156	6.53053	7.77586	0.251802
Ciclev10026156m.g	scaffold_7:7951708-7953311	3.93203	4.68199	0.25185
Ciclev10009422m.g	scaffold_1:474706-476724	9.50472	11.3198	0.252135
Ciclev10023577m.g	scaffold_3:49221008-49223320	32.8843	39.1663	0.252213
Ciclev10025045m.g	scaffold_7:18963486-18968140	173.152	206.247	0.252332
Ciclev10011723m.g	scaffold_6:15380796-15382751	18.1503	21.6195	0.252341
Ciclev10031794m.g	scaffold_4:16780999-16786685	66.6074	79.3633	0.252791
Ciclev10015558m.g	scaffold_2:32280492-32283392	18.1654	21.6477	0.253016
Ciclev10008211m.g	scaffold_1:24171631-24175378	107.697	128.35	0.253104
Ciclev10011172m.g	scaffold_6:9344592-9349593	8.47594	10.1014	0.253118

Ciclev10018565m.g	scaffold_3:21878974-21892761	17.9277	21.3661	0.253131
Ciclev10011927m.g	scaffold_6:14237075-14240196	3.1655	3.77316	0.253337
Ciclev10009435m.g	scaffold_1:1709130-1710064	402.251	479.475	0.253359
Ciclev10025555m.g	scaffold_7:372727-375553	8.14685	9.71262	0.253618
Ciclev10007495m.g	scaffold_1:2112389-2119604	47.5012	56.6385	0.253818
Ciclev10030269m.g	scaffold_8:1764097-1774806	1.78507	2.12857	0.253903
Ciclev10031399m.g	scaffold_4:9470199-9473671	2.66257	3.17517	0.254013
Ciclev10015668m.g	scaffold_2:29534670-29539321	12.6924	15.1368	0.254097
Ciclev10014123m.g,Ciclev10017635m.g	scaffold_2:12050431-12085984	20.4549	24.3993	0.254393
Ciclev10001352m.g	scaffold_5:27585412-27592977	19.1322	22.8239	0.254547
Ciclev10000622m.g	scaffold_5:35548958-35554093	19.8375	23.6656	0.254566
Ciclev10025229m.g	scaffold_7:17580331-17588001	38.7052	46.178	0.254679
Ciclev10020554m.g	scaffold_3:7020441-7024801	33.6893	40.1942	0.254696
Ciclev10005461m.g	scaffold_9:17203150-17207632	34.8663	41.5989	0.25471
Ciclev10015484m.g	scaffold_2:3761357-3763055	1.41651	1.69025	0.254902
Ciclev10004317m.g	scaffold_9:15116834-15119883	1.51281	1.80519	0.254913
Ciclev10016602m.g	scaffold_2:8016079-8019134	10.8403	12.9359	0.254967
Ciclev10000178m.g	scaffold_5:33225099-33232451	22.0094	26.2677	0.255172
Ciclev10022300m.g	scaffold_3:23752351-23753350	15.3441	18.3135	0.255221
Ciclev10014507m.g	scaffold_2:36020215-36025046	17.4571	20.8355	0.255233
Ciclev10009098m.g	scaffold_1:2578556-2582000	44.4437	53.0451	0.255241
Ciclev10009993m.g	scaffold_1:18540963-18543354	138.881	165.795	0.255553
Ciclev10020365m.g	scaffold_3:43053574-43057823	29.5053	35.2256	0.255648
Ciclev10004462m.g	scaffold_9:27714382-27727799	37.4028	44.6581	0.255777
Ciclev10008090m.g	scaffold_1:9605197-9611169	56.0524	66.9305	0.255888
Ciclev10027682m.g,Ciclev10027684m.g,Ciclev10027685m.g	scaffold_8:17601835-17651635	27.1257	32.3901	0.255893
Ciclev10023883m.g	scaffold_3:12280363-12285163	19.9913	23.8726	0.255987
Ciclev10012720m.g	scaffold_6:16395497-16396640	9.2581	11.058	0.256303
Ciclev10000624m.g	scaffold_5:17900801-17904488	2.29426	2.74051	0.256417
Ciclev10002509m.g	scaffold_5:42648256-42649414	0.387483	0.462969	0.256783
Ciclev10014042m.g	scaffold_2:33074820-33084340	8.64407	10.3292	0.256939
Ciclev10001288m.g	scaffold_5:15023462-15026961	17.1496	20.4951	0.257108
Ciclev10014869m.g	scaffold_2:19572438-19577226	6.58642	7.87255	0.257336
Ciclev10029926m.g	scaffold_8:4292179-4375670	2.22043	2.65431	0.257498
Ciclev10020462m.g	scaffold_3:5945689-5952014	15.4592	18.4806	0.257545
Ciclev10019956m.g	scaffold_3:42990250-42995979	19.7885	23.6582	0.257678
Ciclev10014014m.g	scaffold_2:328653-345424	15.0887	18.0407	0.257793
Ciclev10020010m.g	scaffold_3:48343572-48345209	30.0904	35.9785	0.257832
Ciclev10018312m.g	scaffold_2:7686100-7688683	1.6493	1.97225	0.257992
Ciclev10028120m.g	scaffold_8:4966462-4969944	18.5864	22.2259	0.258
Ciclev10018923m.g	scaffold_3:8303878-8309177	4.35233	5.205	0.258112
Ciclev10021179m.g	scaffold_3:532881-536813	21.6078	25.8431	0.25823
Ciclev10009373m.g	scaffold_1:9321919-9325462	126.549	151.36	0.258283
Ciclev10016107m.g	scaffold_2:25335048-25337612	96.9564	115.972	0.258367
Ciclev10021378m.g	scaffold_3:45576862-45579197	86.1806	103.091	0.258484
Ciclev10019731m.g,Ciclev10019876m.g	scaffold_3:30066285-30161618	48.5858	58.1347	0.258864
Ciclev10011119m.g	scaffold_6:15477947-15483835	32.0127	38.3068	0.258956
Ciclev10021302m.g	scaffold_3:49782678-49786698	11.0511	13.2249	0.25907
Ciclev10021493m.g	scaffold_3:42915946-42921130	7.37486	8.82593	0.259133
Ciclev10005732m.g	scaffold_9:23075529-23122347	8.45664	10.1227	0.259436
Ciclev10031155m.g	scaffold_4:22802994-22807340	32.7569	39.2127	0.259523
Ciclev10000822m.g	scaffold_5:20137792-20140608	82.1433	98.3363	0.259581

Ciclev10002601m.g	scaffold_5:30613183-30615183	5.62741	6.73909	0.260084
Ciclev10011609m.g	scaffold_6:20541095-20546049	113.076	135.421	0.260163
Ciclev10033913m.g	scaffold_4:21178160-21184081	34.5026	41.3215	0.260186
Ciclev10004856m.g	scaffold_9:14812408-14818194	14.794	17.7181	0.260205
Ciclev10022701m.g	scaffold_3:10582057-10585006	15.2696	18.288	0.26023
Ciclev10006169m.g	scaffold_9:30799766-30803310	33.0386	39.5694	0.260236
Ciclev10016067m.g	scaffold_2:35656844-35659383	46.9598	56.2428	0.260242
Ciclev10015023m.g	scaffold_2:11601947-11603617	22.1611	26.542	0.260246
Ciclev10027747m.g	scaffold_8:6933871-6943843	15.5892	18.6731	0.260413
Ciclev10007432m.g	scaffold_1:3678599-3683434	14.3534	17.1938	0.260494
Ciclev10025761m.g	scaffold_7:20278362-20285118	23.7468	28.4512	0.260759
Ciclev10009941m.g	scaffold_1:28602401-28604161	32.1267	38.5028	0.261191
Ciclev10008206m.g	scaffold_1:23463842-23470914	7.82107	9.37407	0.261309
Ciclev10012086m.g	scaffold_6:22644643-22651260	39.1868	46.9689	0.261337
Ciclev10032797m.g	scaffold_4:23691916-23692734	115.301	138.199	0.26134
Ciclev10005088m.g	scaffold_9:22507920-22509455	12.6893	15.21	0.261408
Ciclev10003449m.g	scaffold_5:14888241-14890248	171.127	205.15	0.261617
Ciclev10018414m.g	scaffold_2:9831564-9834666	41.3019	49.5165	0.261699
Ciclev10021531m.g	scaffold_3:47576122-47578036	53.8066	64.5085	0.261706
Ciclev10018570m.g	scaffold_3:16491124-16502213	14.3339	17.1859	0.261794
Ciclev10020003m.g	scaffold_3:44670368-44674788	9.62664	11.542	0.261794
Ciclev10032178m.g	scaffold_4:17789804-17793631	17.093	20.4952	0.261885
Ciclev10032622m.g	scaffold_4:5272515-5276687	7.37551	8.84369	0.261906
Ciclev10030937m.g	scaffold_4:150077-155154	24.2766	29.1093	0.261914
Ciclev10027786m.g	scaffold_8:23146951-23149624	2.38323	2.85797	0.262074
Ciclev10031622m.g	scaffold_4:25027356-25030551	18.9724	22.7518	0.262082
Ciclev10031317m.g	scaffold_4:8082073-8090442	11.3141	13.5683	0.262112
Ciclev10029011m.g	scaffold_8:2678306-2680973	26.2103	31.4334	0.262164
Ciclev10015426m.g	scaffold_2:23701803-23704006	22.2591	26.696	0.262226
Ciclev10019010m.g	scaffold_3:45450484-45456856	35.4529	42.5212	0.262279
Ciclev10005441m.g	scaffold_9:28305830-28333082	26.0158	31.2067	0.262468
Ciclev10024839m.g	scaffold_7:9949384-9961255	14.1946	17.0347	0.263139
Ciclev10002323m.g	scaffold_5:36347766-36351111	21.0186	25.2267	0.263284
Ciclev10032579m.g	scaffold_4:338423-344420	12.0661	14.4823	0.263327
Ciclev10021755m.g	scaffold_3:50170156-50173660	12.7561	15.3105	0.263338
Ciclev10009603m.g	scaffold_1:909966-912526	46.8377	56.2202	0.263418
Ciclev10016401m.g	scaffold_2:36072084-36073563	14.3557	17.2325	0.263511
Ciclev10032613m.g	scaffold_4:24308364-24311539	10.2755	12.3357	0.263626
Ciclev10004081m.g	scaffold_5:7639004-7639810	4.09084	4.91141	0.263739
Ciclev10025891m.g	scaffold_7:20293514-20296161	14.5622	17.4834	0.263757
Ciclev10019541m.g,Ciclev10020899m.g	scaffold_3:2371343-2379103	6.14119	7.37322	0.263776
Ciclev10010785m.g	scaffold_1:17996315-17999191	5.80044	6.96474	0.263907
Ciclev10012185m.g	scaffold_6:7191339-7198163	11.009	13.2199	0.264027
Ciclev10024860m.g	scaffold_7:8603449-8610239	36.2522	43.541	0.264306
Ciclev10028908m.g	scaffold_8:1063710-1065289	21.4845	25.8097	0.264612
Ciclev10031551m.g	scaffold_4:1496781-1499746	3.488	4.19151	0.265071
Ciclev10028211m.g	scaffold_8:3583752-3587378	2.7757	3.3356	0.265097
Ciclev10026329m.g	scaffold_7:2432145-2435903	12.8516	15.4446	0.265151
Ciclev10014615m.g	scaffold_2:8247050-8249920	3.28873	3.95239	0.265197
Ciclev10019250m.g	scaffold_3:44597003-44604634	20.1802	24.2527	0.265199
Ciclev10002211m.g	scaffold_5:40846791-40849031	11.8136	14.1977	0.26521
Ciclev10015778m.g	scaffold_2:29275820-29278801	7.09306	8.52551	0.265377
Ciclev10002542m.g	scaffold_5:37964988-37967950	17.539	21.0816	0.26542
Ciclev10019083m.g	scaffold_3:50029139-50032046	11.5959	13.9389	0.265503
Ciclev10003762m.g	scaffold_5:25221155-25545745	23.4156	28.1472	0.265522

Ciclev10031005m.g	scaffold_4:14024854-14033394	12.2032	14.6695	0.265557
Ciclev10020529m.g	scaffold_3:47346846-47350348	30.0102	36.0824	0.265842
Ciclev10014186m.g	scaffold_2:8458193-8466681	48.3798	58.1757	0.26601
Ciclev10032923m.g	scaffold_4:3749808-3751724	153.106	184.111	0.266046
Ciclev10011843m.g	scaffold_6:18014433-18025689	19.3354	23.2516	0.266084
Ciclev10025889m.g	scaffold_7:321291-325988	9.26777	11.1462	0.266259
Ciclev10014617m.g	scaffold_2:8418788-8421982	86.9564	104.592	0.26641
Ciclev10000004m.g	scaffold_5:35420547-35446874	10.1559	12.216	0.26645
Ciclev10017539m.g	scaffold_2:9023996-9026131	53.5999	64.4772	0.266559
Ciclev10014027m.g	scaffold_2:27916298-27923913	20.6025	24.7851	0.266653
Ciclev10006886m.g	scaffold_9:29002920-29005831	15.6317	18.8052	0.266653
Ciclev10000719m.g	scaffold_5:39705412-39708788	39.0867	47.0252	0.266758
Ciclev10014200m.g	scaffold_2:11468589-11477257	26.162	31.4767	0.266807
Ciclev10025375m.g	scaffold_7:4495614-4502820	7.45214	8.9672	0.267003
-	scaffold_2:27827256-27829634	12.5621	15.1168	0.267073
Ciclev10030778m.g	scaffold_4:13621710-13628537	17.727	21.337	0.267409
Ciclev10001202m.g	scaffold_5:37189652-37192240	47.7113	57.4324	0.267535
Ciclev10001078m.g	scaffold_5:39665684-39672167	41.3819	49.8144	0.267564
Ciclev10005381m.g	scaffold_9:4503738-4508011	20.0882	24.184	0.267704
Ciclev10021207m.g	scaffold_3:16732451-16739154	22.4824	27.0665	0.267712
Ciclev10024544m.g	scaffold_3:23764295-23769362	1.93597	2.33164	0.268289
Ciclev10025261m.g	scaffold_7:7923448-7945523	1.75502	2.11409	0.268544
Ciclev10007866m.g	scaffold_1:24859987-24865086	29.8511	35.9626	0.268714
Ciclev10016175m.g	scaffold_2:14481357-14484929	60.8624	73.324	0.268735
Ciclev10020068m.g	scaffold_3:42460939-42467955	39.6882	47.8169	0.26881
Ciclev10017328m.g	scaffold_2:21986785-21988231	16.1893	19.5052	0.268814
Ciclev10028983m.g,Ciclev10029256m.g	scaffold_8:13334849-13339023	11.8241	14.2459	0.268821
Ciclev10007377m.g	scaffold_1:24829792-24836678	2.62145	3.15876	0.268996
Ciclev10025262m.g	scaffold_7:5788414-5792672	32.9049	39.6532	0.269133
Ciclev10030472m.g	scaffold_4:17057657-17074899	10.8655	13.0968	0.269461
Ciclev10025023m.g	scaffold_7:86682-93461	10.7604	12.9721	0.269677
Ciclev10015341m.g	scaffold_2:28209588-28216224	17.1954	20.7303	0.269717
Ciclev10013456m.g,Ciclev10013540m.g	scaffold_6:7046330-7049367	27.0962	32.6689	0.269829
Ciclev10016105m.g	scaffold_2:25342760-25346132	20.7085	24.9693	0.26993
Ciclev10000575m.g	scaffold_5:20140837-20144990	25.0502	30.2061	0.270017
Ciclev10025642m.g	scaffold_7:6753014-6756121	18.5857	22.4117	0.27006
Ciclev10018443m.g	scaffold_3:5518456-5529974	24.7296	29.8212	0.270098
Ciclev10028312m.g	scaffold_8:12967156-13120570	83.2815	100.43	0.270128
Ciclev10012939m.g	scaffold_6:18271726-18274742	0.414656	0.500043	0.270136
Ciclev10014823m.g	scaffold_2:35323614-35328102	15.5337	18.7328	0.270164
Ciclev10005325m.g	scaffold_9:2211861-2215512	32.2585	38.9047	0.270266
Ciclev10019124m.g	scaffold_3:43273847-43280370	32.6027	39.3209	0.270306
Ciclev10014221m.g	scaffold_2:29727558-29730207	1.79148	2.16068	0.270331
Ciclev10030612m.g	scaffold_4:18277365-18290523	8.60031	10.373	0.270367
Ciclev10011875m.g,Ciclev10011878m.g	scaffold_6:11782951-11825766	151.131	182.31	0.270596
Ciclev10000086m.g	scaffold_5:16510957-16526623	5.48949	6.62203	0.270602
Ciclev10020151m.g	scaffold_3:47019268-47024957	15.1112	18.2293	0.270642
Ciclev10015208m.g	scaffold_2:28039842-28042544	39.7645	47.9725	0.270728
Ciclev10007497m.g	scaffold_1:26102195-26105224	6.8685	8.28661	0.270788
Ciclev10020829m.g,Ciclev10020838m.g	scaffold_3:30545857-30576767	24.7939	29.9171	0.270983
Ciclev10032052m.g	scaffold_4:1791310-1793405	9.50889	11.4745	0.271079
Ciclev10005401m.g	scaffold_9:12079756-12083355	7.35419	8.87468	0.271128

Ciclev10027090m.g	scaffold_7:7379549-7392547	17.6367	21.2832	0.271137
Ciclev10025086m.g	scaffold_7:6889269-6896036	17.4112	21.0123	0.271217
Ciclev10013164m.g	scaffold_6:15421095-15422836	149.392	180.297	0.271271
Ciclev10000311m.g	scaffold_5:38036284-38041507	4.61266	5.56787	0.271527
Ciclev10019835m.g	scaffold_3:39466664-39471920	18.7243	22.6036	0.271637
Ciclev10032233m.g	scaffold_4:14580646-14583080	12.7128	15.3479	0.271765
Ciclev10018429m.g	scaffold_3:47996066-48011074	6.20928	7.49768	0.272019
Ciclev10000932m.g	scaffold_5:29189913-29191722	1.79516	2.16771	0.272061
-	scaffold_4:14104277-14110322	11.294	13.6402	0.272302
Ciclev10021006m.g	scaffold_3:49849756-49852941	26.6218	32.1573	0.272541
Ciclev10014230m.g,Ciclev10014240m.g,Ciclev10017637m.g	scaffold_2:6181064-6338878	25.9616	31.3605	0.272572
Ciclev10018538m.g	scaffold_3:45648417-45658606	55.263	66.7569	0.272603
Ciclev10002737m.g	scaffold_5:41386408-41389807	94.4322	114.095	0.272883
Ciclev10026354m.g	scaffold_7:118795-121913	48.9049	59.0887	0.272903
Ciclev10004056m.g	scaffold_5:31721154-31722262	0.446244	0.539227	0.27306
Ciclev10032800m.g,Ciclev10033755m.g	scaffold_4:23762836-23771567	161.271	194.878	0.273081
Ciclev10008202m.g	scaffold_1:24163229-24169344	13.1687	15.9175	0.273498
Ciclev10011982m.g	scaffold_6:17688685-17692940	27.1215	32.7837	0.273543
Ciclev10018804m.g	scaffold_3:40078102-40089151	11.1563	13.4864	0.273645
Ciclev10012934m.g	scaffold_6:16927887-16930377	46.2255	55.8806	0.273661
Ciclev10012150m.g	scaffold_6:5313543-5316394	46.5068	56.2222	0.273699
Ciclev10010681m.g	scaffold_1:17604570-17605299	9.23175	11.1613	0.273827
Ciclev10007228m.g	scaffold_1:2766538-2783333	0.763911	0.923626	0.273903
Ciclev10015457m.g	scaffold_2:21683268-21687604	24.6132	29.7604	0.273961
Ciclev10014885m.g	scaffold_2:12971882-12977820	26.8531	32.4729	0.27415
Ciclev10001081m.g	scaffold_5:21379383-21388939	48.9778	59.2297	0.274191
Ciclev10000179m.g	scaffold_5:31469286-31718393	1.21247	1.46643	0.274362
Ciclev10033399m.g	scaffold_4:24208608-24209694	3.8377	4.64168	0.274404
Ciclev10014969m.g	scaffold_2:28647503-28652278	0.836503	1.01177	0.274434
Ciclev10022774m.g	scaffold_3:42974946-42976794	77.3478	93.5564	0.274476
Ciclev10019858m.g	scaffold_3:49159890-49165911	64.3048	77.7941	0.274735
Ciclev10011862m.g	scaffold_6:11462009-11465215	40.3676	48.8395	0.274851
Ciclev10018002m.g	scaffold_2:34131738-34135657	11.4643	13.8703	0.27486
Ciclev10030995m.g	scaffold_4:2782277-2787033	16.624	20.113	0.27486
Ciclev10027720m.g	scaffold_8:24483405-24487972	10.1245	12.2515	0.275111
Ciclev10028767m.g	scaffold_8:23610900-23614086	0.298962	0.361771	0.275115
Ciclev10025998m.g	scaffold_7:6870469-6872299	2.49596	3.02041	0.27515
Ciclev10031598m.g	scaffold_4:19681883-19685929	48.2798	58.4309	0.275314
Ciclev10002108m.g	scaffold_5:34239480-34244107	24.7636	29.9731	0.275446
Ciclev10027789m.g	scaffold_8:4884015-4890575	30.9546	37.4669	0.27546
Ciclev10007274m.g	scaffold_1:14937919-14953966	14.2716	17.2742	0.275467
Ciclev10014173m.g	scaffold_2:9609887-9620427	17.162	20.7731	0.275501
Ciclev10025051m.g	scaffold_7:4689377-4697698	13.13	15.8937	0.275586
Ciclev10033678m.g	scaffold_4:7333256-7339171	2.21193	2.67781	0.27575
Ciclev10022444m.g	scaffold_3:23375588-23379970	13.1575	15.9355	0.276358
Ciclev10022430m.g	scaffold_3:4346328-4348464	84.9285	102.867	0.276457
Ciclev10028917m.g	scaffold_8:23900102-23903312	25.5178	30.9086	0.276507
-	scaffold_9:801700-810351	82.2341	99.6088	0.276536
Ciclev10019560m.g	scaffold_3:25458170-25463466	4.05171	4.9079	0.276576
Ciclev10029145m.g	scaffold_8:3738606-3741747	11.9956	14.5323	0.276757
Ciclev10033030m.g	scaffold_4:20797373-20799692	17.3477	21.0171	0.276819
Ciclev10011482m.g	scaffold_6:21338926-21342753	17.5232	21.2301	0.276838
Ciclev10033159m.g	scaffold_4:1793569-1795656	11.8377	14.3438	0.277031

Ciclev10015565m.g	scaffold_2:9940311-9943126	22.1274	26.8137	0.277135
Ciclev10006072m.g	scaffold_9:29605463-29607371	71.3556	86.4702	0.277178
Ciclev10024928m.g	scaffold_7:7915334-7922054	20.99	25.4364	0.277192
Ciclev10011964m.g	scaffold_6:24643526-24647275	6.3883	7.74277	0.277419
Ciclev10018908m.g,Ciclev10019246m.g,Ciclev10024393m.g	scaffold_3:30952562-31040200	12.813	15.5305	0.277501
Ciclev10030497m.g	scaffold_4:2820242-2830784	19.233	23.3144	0.277636
Ciclev10001005m.g	scaffold_5:30736530-30740889	5.56447	6.74566	0.277716
Ciclev10018812m.g	scaffold_3:47087018-47091796	14.0616	17.0497	0.277985
Ciclev10032739m.g	scaffold_4:24914067-24916333	43.3545	52.5706	0.278074
Ciclev10010304m.g	scaffold_1:22555903-22564833	0.377084	0.457272	0.278167
Ciclev10019503m.g	scaffold_3:10597926-10600347	14.4622	17.5399	0.278354
Ciclev10005711m.g	scaffold_9:21228790-21231253	95.3409	115.642	0.278493
Ciclev10011052m.g	scaffold_6:19086401-19090359	3.24257	3.93326	0.278589
Ciclev10025861m.g	scaffold_7:3038258-3041881	2.64294	3.20608	0.278668
Ciclev10025382m.g	scaffold_7:1450747-1452915	114.021	138.317	0.278689
Ciclev10008352m.g	scaffold_1:15763331-15768504	3.54134	4.29607	0.278722
Ciclev10032124m.g	scaffold_4:6111839-6116679	36.2805	44.0187	0.278922
Ciclev10008615m.g	scaffold_1:25830360-25839656	8.07222	9.79417	0.278958
Ciclev10022898m.g	scaffold_3:50744746-50746371	54.4445	66.0632	0.279059
Ciclev10011159m.g	scaffold_6:18413877-18419600	26.173	31.7605	0.279157
Ciclev10026068m.g	scaffold_7:4728926-4732378	11.4322	13.8741	0.279293
Ciclev10019392m.g	scaffold_3:42633340-42638729	17.0297	20.6673	0.279304
Ciclev10016769m.g	scaffold_2:30485160-30488443	109.552	132.976	0.27955
Ciclev10016633m.g	scaffold_2:29961647-29962922	14.0518	17.0604	0.279889
Ciclev10001408m.g	scaffold_5:36833742-36837131	0.584273	0.709393	0.279943
Ciclev10000809m.g	scaffold_5:26839045-26841490	1.81851	2.2083	0.280179
Ciclev10017597m.g	scaffold_2:7882351-7885966	6.33175	7.68923	0.280235
Ciclev10018446m.g	scaffold_3:42260805-42273658	10.8291	13.1512	0.280281
Ciclev10014930m.g	scaffold_2:9861898-9863490	12.6038	15.3067	0.280303
Ciclev10028041m.g	scaffold_8:19969432-19975072	5.20316	6.31916	0.280345
Ciclev10017269m.g	scaffold_2:35830654-35832364	4.01589	4.87724	0.280347
Ciclev10027523m.g	scaffold_7:11093532-11097571	4.38056	5.32028	0.280386
Ciclev10019344m.g	scaffold_3:2852621-2857777	14.9918	18.2084	0.280426
Ciclev10003031m.g	scaffold_5:36766088-36766771	2.7609	3.35334	0.280462
Ciclev10009346m.g	scaffold_1:12006765-12009517	387.568	470.761	0.280545
Ciclev10022685m.g	scaffold_3:45676506-45679283	35.6102	43.2554	0.28059
Ciclev10000310m.g	scaffold_5:19136105-19146196	7.49519	9.1046	0.280631
Ciclev10027692m.g	scaffold_8:19623099-19631587	13.0924	15.904	0.280655
Ciclev10019132m.g	scaffold_3:46061975-46066603	20.1567	24.4905	0.280962
Ciclev10011269m.g	scaffold_6:23693082-23698678	9.37008	11.3855	0.281064
Ciclev10009887m.g	scaffold_1:2184512-2186967	173.324	210.615	0.281136
Ciclev10022498m.g	scaffold_3:195777-197622	67.4761	81.9982	0.281216
Ciclev10021530m.g	scaffold_3:4337816-4340640	159.949	194.386	0.281313
Ciclev10018989m.g	scaffold_3:37868703-37880417	4.42334	5.37593	0.281379
Ciclev10019740m.g	scaffold_3:44771257-44776607	13.5522	16.4711	0.281407
Ciclev10022406m.g	scaffold_3:2803784-2805325	32.1245	39.0436	0.281413
Ciclev10014064m.g	scaffold_2:29478829-29490506	20.0384	24.3549	0.281447
Ciclev10017439m.g	scaffold_2:11703299-11711022	21.5837	26.2356	0.281582
Ciclev10014921m.g	scaffold_2:36015165-36019540	10.6815	12.9838	0.281603
Ciclev10020549m.g	scaffold_3:40984680-40986653	7.24214	8.80326	0.281622
Ciclev10001834m.g	scaffold_5:35414985-35417163	17.8853	21.7412	0.281655
Ciclev10031377m.g	scaffold_4:12941528-12945334	7.24358	8.80537	0.281681
Ciclev10008794m.g	scaffold_1:5020110-5021818	26.9425	32.7522	0.281709
Ciclev10007082m.g	scaffold_9:30382985-30428893	1.00261	1.21884	0.281758

Ciclev10028086m.g	scaffold_8:2649990-2655756	32.3035	39.2772	0.282002
Ciclev10022551m.g	scaffold_3:50919699-50922627	17.5766	21.3723	0.282087
Ciclev10029044m.g	scaffold_8:4558900-4567593	52.5319	63.8824	0.282224
Ciclev10027657m.g	scaffold_8:4039630-4057040	3.58983	4.36548	0.282226
Ciclev10030470m.g	scaffold_4:5801065-5817370	4.19604	5.10396	0.282591
Ciclev10002958m.g	scaffold_5:37155424-37158097	23.5513	28.6492	0.282691
Ciclev10028582m.g	scaffold_8:1321425-1323876	4.72428	5.74701	0.282719
Ciclev10026264m.g	scaffold_7:2960997-2963176	97.7359	118.898	0.282768
Ciclev10002311m.g	scaffold_5:40300305-40304768	23.1098	28.1152	0.282848
Ciclev10009162m.g	scaffold_1:26576058-26578193	126.75	154.215	0.282959
Ciclev10014975m.g	scaffold_2:5176433-5180701	35.2266	42.8613	0.283011
Ciclev10019794m.g	scaffold_3:40056975-40061574	4.01014	4.87944	0.283064
Ciclev10030902m.g	scaffold_4:1478188-1482912	15.3262	18.649	0.2831
Ciclev10011634m.g	scaffold_6:7873786-7876269	3.41635	4.15725	0.283176
Ciclev10018703m.g	scaffold_3:48852955-48859544	1.70594	2.07596	0.283214
Ciclev10025964m.g	scaffold_7:17315817-17319105	13.3601	16.2596	0.283356
Ciclev10014052m.g	scaffold_2:29996935-30005047	63.497	77.2801	0.283407
Ciclev10027515m.g	scaffold_7:15446268-15449214	487.219	593.143	0.283812
Ciclev10020174m.g	scaffold_3:7182920-7187456	67.2327	81.858	0.28396
Ciclev10020626m.g	scaffold_3:8280597-8282585	4.14529	5.04732	0.284045
Ciclev10008750m.g	scaffold_1:4612232-4614602	26.2983	32.0244	0.284203
Ciclev10025109m.g	scaffold_7:3446798-3451586	21.8804	26.6462	0.284291
Ciclev10021384m.g	scaffold_3:43866229-43868847	36.0618	43.9165	0.284292
Ciclev10000779m.g	scaffold_5:40500030-40505210	47.8757	58.3049	0.284326
Ciclev10023190m.g	scaffold_3:48160165-48163796	3.0914	3.76535	0.284526
Ciclev10031523m.g	scaffold_4:201555-204527	0.224515	0.273497	0.284717
Ciclev10025164m.g	scaffold_7:3946817-3949964	57.1652	69.6376	0.28473
Ciclev10024121m.g	scaffold_3:21296577-21473295	1.33947	1.63176	0.284771
Ciclev10014363m.g	scaffold_2:8395283-8404194	11.6196	14.1553	0.28478
Ciclev10005229m.g	scaffold_9:29290414-29356041	13.3131	16.2187	0.284802
Ciclev10023291m.g	scaffold_3:44668994-44669369	4.88585	5.95301	0.285011
Ciclev10019405m.g	scaffold_3:15167735-15179858	20.3107	24.7559	0.285534
-	scaffold_7:11685367-11686559	8.15938	9.94606	0.285665
Ciclev10016904m.g	scaffold_2:31927341-31929229	37.5921	45.8248	0.285697
Ciclev10007609m.g	scaffold_1:851899-857695	44.9644	54.8119	0.285706
Ciclev10015059m.g	scaffold_2:33347828-33350520	47.1781	57.5208	0.285967
Ciclev10000309m.g	scaffold_5:40126604-40138178	4.04303	4.931	0.286444
Ciclev10022928m.g	scaffold_3:49279536-49281019	2.45645	2.99596	0.286446
-	scaffold_7:11686788-11771145	4.25744	5.19267	0.286489
Ciclev10007750m.g	scaffold_1:8138750-8170757	44.3823	54.1364	0.286615
Ciclev10005756m.g	scaffold_9:28833096-28834876	125.511	153.106	0.286711
Ciclev10009554m.g	scaffold_1:26049785-26052249	31.2593	38.1434	0.287145
Ciclev10005261m.g	scaffold_9:5328356-5329868	1.42502	1.73887	0.287168
Ciclev10031055m.g	scaffold_4:19397704-19401269	84.1144	102.644	0.287227
Ciclev10019221m.g	scaffold_3:48097408-48100235	17.7154	21.6192	0.287313
Ciclev10013157m.g	scaffold_6:19399584-19401918	261.461	319.168	0.287723
Ciclev10014722m.g	scaffold_2:23668185-23671890	20.8242	25.4205	0.287731
Ciclev10002081m.g	scaffold_5:20349396-20384343	39.7923	48.5788	0.287835
Ciclev10027825m.g	scaffold_8:8786793-8797567	22.3508	27.2866	0.287867
Ciclev10029760m.g	scaffold_8:1779530-1779962	6.65273	8.12286	0.288043
Ciclev10016540m.g	scaffold_2:30647686-30649856	2192.27	2676.84	0.288111
Ciclev10015435m.g	scaffold_2:25871132-25874742	72.1851	88.1419	0.288128
Ciclev10018207m.g	scaffold_2:23913100-23917091	6.82221	8.33079	0.288215
Ciclev10007405m.g	scaffold_1:27123193-27131439	9.91602	12.1097	0.28833
Ciclev10029644m.g	scaffold_8:21110363-21111473	9.93089	12.128	0.28835
Ciclev10030571m.g	scaffold_4:18839545-18856226	8.34508	10.192	0.288433

Ciclev10012537m.g	scaffold_6:15741902-15746306	74.7381	91.282	0.288487
Ciclev10009015m.g	scaffold_1:8822833-8826308	48.0395	58.6826	0.288712
Ciclev10022149m.g	scaffold_3:25448857-25455844	23.3697	28.5476	0.288728
Ciclev10032694m.g	scaffold_4:16531224-16533770	62.3731	76.1956	0.288786
Ciclev10007235m.g	scaffold_1:2611573-2621129	8.55959	10.4566	0.288806
Ciclev10000915m.g	scaffold_5:36101457-36105593	12.7055	15.5229	0.288943
Ciclev10007762m.g	scaffold_1:27104322-27109731	41.0923	50.2049	0.288961
Ciclev10000776m.g	scaffold_5:15735613-15740485	46.3755	56.6609	0.288992
Ciclev10001487m.g	scaffold_5:36215841-36219128	21.944	26.8109	0.288997
Ciclev10005259m.g,Ciclev10005548m.g	scaffold_9:1886166-1894747	44.3816	54.2299	0.289129
Ciclev10000521m.g	scaffold_5:36698868-36703010	58.2915	71.2271	0.28914
Ciclev10031527m.g,Ciclev10033120m.g,Ciclev10033659m.g	scaffold_4:18515567-18675155	6.49213	7.93313	0.289198
Ciclev10021371m.g	scaffold_3:48545441-48550268	11.1626	13.6427	0.289461
Ciclev10016821m.g	scaffold_2:12406077-12408794	75.4655	92.2524	0.289769
Ciclev10000982m.g	scaffold_5:33217334-33221682	40.3406	49.3146	0.289782
Ciclev10025659m.g	scaffold_7:303828-305426	21.6442	26.4606	0.289864
Ciclev10012231m.g	scaffold_6:24370254-24373796	22.4443	27.4401	0.289936
Ciclev10008269m.g	scaffold_1:1461580-1465960	144.168	176.26	0.289957
Ciclev10028887m.g	scaffold_8:6901629-6904625	21.021	25.7006	0.28997
Ciclev10000603m.g	scaffold_5:35757161-35762784	58.2589	71.2305	0.290015
Ciclev10024331m.g	scaffold_3:43011640-43045787	0.458283	0.560342	0.29007
Ciclev10031257m.g	scaffold_4:21629501-21631584	3.07758	3.76298	0.290078
Ciclev10029213m.g	scaffold_8:531528-533825	39.102	47.8119	0.290126
Ciclev10031697m.g	scaffold_4:17702479-17706976	19.1732	23.4444	0.290154
Ciclev10000451m.g	scaffold_5:42626290-42633313	29.0096	35.4734	0.290209
Ciclev10021922m.g	scaffold_3:26526424-26531920	7.95683	9.73022	0.290278
Ciclev10008275m.g	scaffold_1:165671-167810	1.36732	1.67216	0.290363
Ciclev10029771m.g	scaffold_8:7259464-7260921	52.8342	64.6147	0.290391
Ciclev10014741m.g	scaffold_2:24938685-24943643	175.607	214.795	0.290607
Ciclev10012299m.g	scaffold_6:19115568-19117548	35.6056	43.5537	0.290691
Ciclev10031281m.g	scaffold_4:17111497-17118007	72.4538	88.6394	0.290886
Ciclev10028901m.g	scaffold_8:3919385-3921231	0.414395	0.507018	0.291028
Ciclev10012194m.g	scaffold_6:15824937-15828810	43.2474	52.9223	0.291264
Ciclev10024089m.g	scaffold_3:34255962-34259969	15.2055	18.6088	0.291397
Ciclev10013931m.g	scaffold_10:207950-323062	37.2156	45.5459	0.291413
Ciclev10002192m.g	scaffold_5:29339627-29387910	5.35605	6.55511	0.29145
Ciclev10018547m.g	scaffold_3:44799958-44811832	18.5912	22.7557	0.29161
Ciclev10010930m.g,Ciclev10010933m.g	scaffold_6:24298135-24312605	25.4914	31.2035	0.2917
Ciclev10011261m.g	scaffold_6:16641418-16644677	23.5751	28.8588	0.291748
Ciclev10004561m.g	scaffold_9:26672551-26679443	6.9305	8.48394	0.291776
Ciclev10008189m.g	scaffold_1:22593670-22599178	16.5435	20.2535	0.291908
Ciclev10007707m.g	scaffold_1:7903443-7910533	58.1582	71.2036	0.291969
Ciclev10001534m.g,Ciclev10003783m.g	scaffold_5:6599710-6620960	5.09248	6.23511	0.292047
Ciclev10001338m.g	scaffold_5:19604826-19609711	55.3842	67.8183	0.292199
Ciclev10022220m.g	scaffold_3:1683409-1686537	16.6642	20.4056	0.292208
Ciclev10009047m.g	scaffold_1:28928260-28932927	75.5729	92.5413	0.292228
Ciclev10031871m.g	scaffold_4:20868749-20872170	35.7027	43.7231	0.292363
Ciclev10030489m.g	scaffold_4:2351762-2364890	7.69262	9.42076	0.292369
Ciclev10030354m.g	scaffold_8:13961017-13965239	64.6359	79.1571	0.292382
Ciclev10020972m.g	scaffold_3:22877398-22878665	2.61023	3.19678	0.292441
Ciclev10013053m.g	scaffold_6:22345299-22346076	9.16945	11.2335	0.292897

Ciclev10016875m.g	scaffold_2:11369923-11372324	146.616	179.642	0.293083
Ciclev10012651m.g	scaffold_6:4289789-4292692	66.7165	81.7475	0.293132
Ciclev10003188m.g	scaffold_5:32643454-32646430	0.45303	0.555118	0.293187
Ciclev10002354m.g	scaffold_5:42809559-42821401	9.38127	11.4962	0.293303
Ciclev10004806m.g	scaffold_9:21610914-21615813	22.6742	27.7863	0.29332
Ciclev10026222m.g	scaffold_7:11505020-11507288	480.925	589.434	0.293519
-	scaffold_7:6995029-6996371	18.9755	23.2577	0.293571
Ciclev10014796m.g	scaffold_2:7163704-7168765	12.3623	15.1529	0.293638
Ciclev10010943m.g	scaffold_6:19455408-19468343	42.7671	52.429	0.293861
Ciclev10007659m.g	scaffold_1:13143875-13148629	23.2476	28.5037	0.294065
Ciclev10018699m.g	scaffold_3:36692324-36699752	1.30421	1.59928	0.294249
Ciclev10001464m.g	scaffold_5:27602513-27607801	7.72443	9.47251	0.294318
Ciclev10007725m.g,Ciclev10007821m.g	scaffold_1:17567551-17577842	22.041	27.033	0.294534
Ciclev10010720m.g,Ciclev10010856m.g	scaffold_1:24887261-24891021	13.2456	16.2456	0.294543
Ciclev10001576m.g	scaffold_5:38556513-38558181	10.9659	13.4502	0.294599
Ciclev10004869m.g	scaffold_9:25658749-25661504	4.17548	5.12158	0.294647
-	scaffold_2:29306914-29307182	24.7555	30.3655	0.294686
Ciclev10029013m.g	scaffold_8:23346733-23350423	3.98161	4.88405	0.294725
Ciclev10030567m.g	scaffold_4:18935474-18945259	1.55316	1.90526	0.294787
Ciclev10016593m.g	scaffold_2:3056083-3060230	17.5346	21.5117	0.294915
Ciclev10009709m.g	scaffold_1:23773533-23782088	30.0764	36.8983	0.294919
Ciclev10007247m.g	scaffold_1:8171298-8183906	6.20176	7.60846	0.294927
Ciclev10031468m.g	scaffold_4:2342670-2345393	9.74863	11.9606	0.295017
Ciclev10000564m.g	scaffold_5:35862624-35867133	16.5092	20.2552	0.29502
Ciclev10028158m.g	scaffold_8:717281-721441	9.11043	11.1782	0.295094
Ciclev10005118m.g,Ciclev10006333m.g	scaffold_9:11287911-11299368	16.5809	20.345	0.295153
Ciclev10026004m.g	scaffold_7:5546986-5550325	66.7868	81.9514	0.295205
Ciclev10004931m.g	scaffold_9:10215539-10221429	47.6168	58.4349	0.295359
Ciclev10019941m.g	scaffold_3:1544508-1547621	16.4095	20.1403	0.295551
Ciclev10005097m.g	scaffold_9:25896765-25897992	1.10937	1.3617	0.295676
Ciclev10016072m.g	scaffold_2:34791424-34795248	46.7089	57.3371	0.295772
Ciclev10031559m.g	scaffold_4:7204820-7209783	251.462	308.693	0.295831
Ciclev10002914m.g	scaffold_5:16049005-16051176	27.9654	34.3321	0.295915
Ciclev10015117m.g	scaffold_2:27654010-27658578	21.785	26.747	0.29604
Ciclev10001394m.g	scaffold_5:2465538-2469155	25.3687	31.1511	0.296233
Ciclev10000030m.g	scaffold_5:6346545-6435349	5.67715	6.97121	0.296242
-	scaffold_5:34100892-34102478	3.99784	4.90972	0.296422
Ciclev10014049m.g	scaffold_2:24674890-24684473	3.2322	3.96953	0.29645
Ciclev10007356m.g	scaffold_1:976041-979235	23.8356	29.2766	0.29663
Ciclev10009386m.g	scaffold_1:23753758-23756754	51.4897	63.2541	0.296876
Ciclev10015079m.g	scaffold_2:9736400-9738083	34.496	42.3802	0.296961
Ciclev10027985m.g	scaffold_8:746161-754353	59.6086	73.2383	0.297078
Ciclev10004757m.g	scaffold_9:25559400-25564464	214.174	263.15	0.297102
Ciclev10005655m.g	scaffold_9:29234483-29237736	31.6453	38.882	0.297111
Ciclev10021254m.g	scaffold_3:36363008-36553812	1.24223	1.52631	0.297116
Ciclev10016732m.g	scaffold_2:27237004-27240127	34.5181	42.4151	0.297226
Ciclev10008707m.g	scaffold_1:14695703-14701000	17.8849	21.9817	0.297567
Ciclev10013333m.g	scaffold_6:20497270-20502466	56.1598	69.0256	0.297594
Ciclev10031347m.g	scaffold_4:21360816-21364043	6.05467	7.44245	0.297728
Ciclev10020110m.g	scaffold_3:5952073-5953587	8.95501	11.0084	0.297832
Ciclev10018155m.g	scaffold_2:4895612-4986690	6.19243	7.61236	0.297838
Ciclev10018506m.g	scaffold_3:46963455-46975117	6.45414	7.93478	0.297966
Ciclev10016309m.g	scaffold_2:28046591-28048907	133.363	163.982	0.298175

Ciclev10001108m.g	scaffold_5:33334756-33337976	1.08515	1.33432	0.29821
Ciclev10007859m.g	scaffold_1:302652-306653	2.79649	3.43867	0.298232
Ciclev10002690m.g	scaffold_5:39717436-39718172	9.45798	11.63	0.298252
Ciclev10014168m.g	scaffold_2:33236102-33245595	14.8619	18.2752	0.298266
Ciclev10019030m.g	scaffold_3:2446819-2453782	12.2168	15.0245	0.298452
Ciclev10025222m.g	scaffold_7:20083135-20088779	12.5715	15.4609	0.298473
Ciclev10025593m.g	scaffold_7:20163-23417	32.2012	39.6032	0.298503
Ciclev10028821m.g	scaffold_8:18775605-18779638	11.6093	14.2786	0.298568
Ciclev10018017m.g	scaffold_2:22708489-22714803	13.0012	15.9915	0.298666
Ciclev10018589m.g	scaffold_3:14070858-14075179	4.9001	6.02738	0.298719
Ciclev10019562m.g	scaffold_3:2235278-2241566	27.7151	34.0962	0.298942
Ciclev10006462m.g	scaffold_9:27995439-27997785	15.0729	18.5447	0.299051
Ciclev10008169m.g	scaffold_1:3006499-3044316	58.2928	71.7258	0.299175
Ciclev10019877m.g,Ciclev10023773m.g	scaffold_3:21515673-21531402	24.4796	30.1222	0.299251
Ciclev10028546m.g	scaffold_8:22145662-22151625	23.4878	28.9189	0.300104
Ciclev10030583m.g	scaffold_4:18371227-18379222	5.07641	6.25037	0.300133
Ciclev10000931m.g	scaffold_5:39415803-39419710	5.59907	6.89417	0.300189
Ciclev10031042m.g	scaffold_4:22733204-22736675	26.6758	32.847	0.30023
Ciclev10008319m.g	scaffold_1:20664826-20823973	48.1785	59.3247	0.300241
Ciclev10025105m.g	scaffold_7:17064336-17069207	23.9874	29.5389	0.300341
Ciclev10005315m.g	scaffold_9:5301260-5303362	46.9476	57.818	0.300469
Ciclev10000432m.g	scaffold_5:43249555-43252914	4.43707	5.46528	0.300689
Ciclev10030436m.g	scaffold_8:1412846-1416494	23.4615	28.8989	0.300721
Ciclev10019251m.g	scaffold_3:8352176-8358299	30.2051	37.2063	0.300754
Ciclev10004699m.g,Ciclev10006487m.g	scaffold_9:30005927-30011317	8.52813	10.5054	0.30083
Ciclev10032138m.g	scaffold_4:453216-456681	21.1942	26.1133	0.301116
Ciclev10010282m.g,Ciclev10010285m.g	scaffold_1:19905420-19995040	8.25271	10.1683	0.301134
Ciclev10020371m.g	scaffold_3:18969849-18972432	2.88911	3.56059	0.30149
Ciclev10003913m.g	scaffold_5:31848912-31860704	42.9661	52.9561	0.301596
Ciclev10026267m.g	scaffold_7:1836617-1839960	9.32572	11.4943	0.30163
Ciclev10013147m.g	scaffold_6:20106156-20107288	1.98663	2.44874	0.301716
Ciclev10021498m.g	scaffold_3:39661026-39665269	6.2368	7.68768	0.301741
Ciclev10010093m.g	scaffold_1:21476759-21547573	6.52028	8.03728	0.301775
Ciclev10012055m.g	scaffold_6:17675266-17676898	136.408	168.156	0.301871
Ciclev10023239m.g	scaffold_3:4633465-4637285	11.5885	14.2857	0.301879
Ciclev10030551m.g	scaffold_4:23200935-23206254	21.0162	25.9091	0.30196
Ciclev10014141m.g	scaffold_2:29892183-29899865	11.734	14.4704	0.302405
Ciclev10009733m.g	scaffold_1:26767993-26770550	14.5879	17.99	0.302424
Ciclev10017967m.g	scaffold_2:5382643-5384275	1.44739	1.78506	0.302518
Ciclev10031335m.g	scaffold_4:24227702-24230754	0.234926	0.289744	0.302573
Ciclev10007929m.g	scaffold_1:23068075-23072144	11.4524	14.1256	0.302656
Ciclev10019518m.g	scaffold_3:4044054-4047260	17.6855	21.8178	0.302936
Ciclev10029202m.g	scaffold_8:20372405-20373832	3.0457	3.7574	0.302964
Ciclev10022481m.g	scaffold_3:1527483-1529254	84.2575	103.98	0.303425
Ciclev10007941m.g	scaffold_1:27698933-27703620	61.8346	76.3137	0.303528
Ciclev10008040m.g	scaffold_1:8105695-8111657	97.815	120.72	0.303537
-	scaffold_8:2897192-2899046	18.0831	22.3202	0.303713
Ciclev10021759m.g	scaffold_3:45630469-45633102	3.47635	4.29139	0.303874
Ciclev10002752m.g	scaffold_5:36974512-36975459	232.495	287.016	0.303929
Ciclev10006173m.g	scaffold_9:5528557-5531045	212.048	261.83	0.304237
Ciclev10012320m.g	scaffold_6:23790000-23794926	60.8096	75.0887	0.304297
Ciclev10014873m.g	scaffold_2:6915376-6920275	33.052	40.824	0.304681
Ciclev10027000m.g	scaffold_7:15110499-15114309	32.9029	40.64	0.304687

Ciclev10020605m.g	scaffold_3:44286095-44289701	1.01237	1.25053	0.304808
Ciclev10019476m.g	scaffold_3:41773650-41778957	66.879	82.6126	0.304809
Ciclev10024947m.g	scaffold_7:19278011-19285271	101.623	125.556	0.305104
Ciclev10001852m.g,Ciclev10003421m.g	scaffold_5:6013082-6023723	10.3719	12.8161	0.305276
Ciclev10000177m.g	scaffold_5:34507107-34513614	14.17	17.5093	0.305279
Ciclev10033724m.g	scaffold_4:4045291-4047833	1.20292	1.48646	0.305342
Ciclev10013268m.g	scaffold_6:18406192-18406840	7.07334	8.74123	0.305445
Ciclev10000945m.g	scaffold_5:6040244-6045626	29.2999	36.2156	0.305716
Ciclev10028467m.g	scaffold_8:11646122-11651777	79.2209	97.9212	0.30574
-	scaffold_6:16717586-16719431	23.8271	29.4538	0.305849
Ciclev10032162m.g	scaffold_4:14363612-14366438	9.27831	11.4698	0.305902
Ciclev10015542m.g	scaffold_2:28186027-28194237	7.84284	9.69554	0.305944
Ciclev10001190m.g	scaffold_5:39194419-39200052	5.4522	6.7405	0.306015
Ciclev10030284m.g	scaffold_8:21583147-21586298	6.13467	7.5843	0.306031
Ciclev10020840m.g,Ciclev10020843m.g,Ciclev10023680m.g	scaffold_3:30618246-30668406	38.0737	47.0749	0.306163
Ciclev10010329m.g	scaffold_1:21464647-21466099	4.25161	5.25693	0.306211
Ciclev10023477m.g	scaffold_3:45342527-45343515	1.79833	2.2237	0.306308
Ciclev10018519m.g	scaffold_3:660145-670778	10.9228	13.508	0.306466
Ciclev10031766m.g	scaffold_4:7729931-7734295	30.246	37.4055	0.306505
Ciclev10005196m.g	scaffold_9:30819802-30825913	36.5113	45.1565	0.306592
Ciclev10012792m.g,Ciclev10013286m.g,Ciclev10013450m.g	scaffold_6:18238195-18244037	12.3804	15.313	0.306697
Ciclev10021078m.g	scaffold_3:50053832-50055873	35.4438	43.8401	0.306721
Ciclev10025999m.g	scaffold_7:5957202-5961807	10.388	12.849	0.306731
Ciclev10015260m.g	scaffold_2:15833141-15836770	292.729	362.178	0.307136
Ciclev10008739m.g	scaffold_1:116680-118262	25.564	31.629	0.307137
Ciclev10021853m.g	scaffold_3:38342794-38345486	2.49775	3.0907	0.307307
Ciclev10024717m.g	scaffold_7:13390679-13402351	31.2987	38.7317	0.307412
Ciclev10031122m.g	scaffold_4:20268445-20272813	5.64673	6.98897	0.307665
Ciclev10005032m.g	scaffold_9:6885082-6888028	4.85243	6.00592	0.307677
Ciclev10031585m.g	scaffold_4:24150057-24154446	27.0089	33.43	0.307707
Ciclev10005396m.g	scaffold_9:10215539-10221429	3.53015	4.36945	0.307722
Ciclev10025412m.g	scaffold_7:18602272-18605349	14.6258	18.1054	0.307908
Ciclev10001373m.g	scaffold_5:19652838-19660511	113.282	140.255	0.308132
Ciclev10013155m.g	scaffold_6:17139987-17143172	17.5215	21.6953	0.308254
Ciclev10029239m.g	scaffold_8:24518327-24520487	77.7229	96.2472	0.308404
Ciclev10008401m.g	scaffold_1:3833514-3840394	6.65114	8.23682	0.308486
Ciclev10014411m.g	scaffold_2:28020357-28026621	76.7041	95.0082	0.308747
Ciclev10031175m.g	scaffold_4:964401-967053	10.2497	12.697	0.308904
Ciclev10011831m.g	scaffold_6:11782951-11825766	16.5486	20.5003	0.308934
Ciclev10026221m.g	scaffold_7:20667440-20671507	13.0384	16.1542	0.309138
Ciclev10026005m.g	scaffold_7:7696810-7698344	13.7137	16.9931	0.309331
Ciclev10004783m.g	scaffold_9:9772904-9777496	2.53061	3.13595	0.309417
Ciclev10026506m.g	scaffold_7:3573929-3576474	15.1987	18.8348	0.309451
Ciclev10012788m.g	scaffold_6:19212921-19215703	241.679	299.516	0.309542
Ciclev10011395m.g	scaffold_6:22387237-22395234	4.69277	5.81649	0.309708
Ciclev10001887m.g	scaffold_5:39490553-39494373	53.2192	65.9643	0.309739
Ciclev10027798m.g	scaffold_8:22106871-22114471	6.77131	8.39415	0.30995
Ciclev10028035m.g	scaffold_8:321062-323652	10.5257	13.0488	0.309999
Ciclev10020688m.g	scaffold_3:6202684-6205300	3.6747	4.55647	0.310289
Ciclev10019651m.g	scaffold_3:6499485-6501568	0.422102	0.523527	0.310671
Ciclev10028740m.g	scaffold_8:18960517-18961726	16.6011	20.591	0.310739

Ciclev10008123m.g	scaffold_1:24866280-24869688	28.8333	35.7679	0.310929
Ciclev10018930m.g	scaffold_3:50550333-50555053	16.3774	20.3197	0.311168
Ciclev10021677m.g	scaffold_3:9566585-9570590	12.6556	15.7068	0.311615
Ciclev10030998m.g	scaffold_4:17143349-17147918	52.6255	65.3136	0.311623
Ciclev10028252m.g,Ciclev10028519m.g,Ciclev10030113m.g	scaffold_8:20950309-21005489	9.59191	11.9046	0.311634
Ciclev10031701m.g	scaffold_4:216216-219257	4.36036	5.4121	0.311739
Ciclev10011504m.g	scaffold_6:5992347-5997768	3.9312	4.87949	0.31176
Ciclev10018632m.g	scaffold_3:13828974-13834857	23.2306	28.8348	0.311784
Ciclev10031841m.g	scaffold_4:1951539-1955276	41.5111	51.5397	0.312188
Ciclev10019186m.g	scaffold_3:6936968-6939298	0.137057	0.170194	0.3124
-	scaffold_2:31452764-31455679	9.57913	11.8994	0.312917
Ciclev10004243m.g	scaffold_9:2310559-2315045	5.65587	7.02601	0.312956
Ciclev10002413m.g	scaffold_5:2189583-2190474	172.052	213.743	0.313036
Ciclev10025089m.g	scaffold_7:3222482-3228894	391.466	486.391	0.31323
Ciclev10020577m.g	scaffold_3:48633780-48636593	7.09509	8.81559	0.313236
Ciclev10007189m.g	scaffold_9:30789728-30798141	10.7192	13.3198	0.313381
Ciclev10031769m.g	scaffold_4:11449236-11454816	9.94636	12.3596	0.313394
Ciclev10022934m.g	scaffold_3:44708641-44711422	84.0595	104.461	0.313476
Ciclev10025961m.g	scaffold_7:20577808-20579817	197.359	245.326	0.31388
Ciclev10005373m.g	scaffold_9:3173390-3176688	14.6278	18.1835	0.313916
Ciclev10017803m.g	scaffold_2:29941717-29943628	0.180914	0.224891	0.313924
Ciclev10004439m.g	scaffold_9:2103210-2115677	5.74938	7.14719	0.313969
Ciclev10020424m.g	scaffold_3:46115182-46119651	2.03429	2.52887	0.31397
Ciclev10003468m.g	scaffold_5:31918502-31919186	14.7977	18.3957	0.313999
Ciclev10015170m.g	scaffold_2:14437096-14440203	5.64252	7.01455	0.314012
Ciclev10018663m.g	scaffold_3:10238525-10247022	134.625	167.379	0.314171
Ciclev10010256m.g	scaffold_1:20924598-20929248	0.0884992	0.110034	0.314217
Ciclev10008112m.g	scaffold_1:28220636-28224080	10.8216	13.4552	0.314258
Ciclev10027585m.g	scaffold_7:5689893-5694050	0.694156	0.863191	0.314419
Ciclev10011246m.g	scaffold_6:12274207-12276268	0.961527	1.19567	0.314421
Ciclev10006289m.g,Ciclev10006924m.g	scaffold_9:685219-695673	46.3057	57.5917	0.314674
Ciclev10017265m.g	scaffold_2:9454516-9456360	182.281	226.714	0.314708
Ciclev10033854m.g	scaffold_4:1065180-1068268	0.193764	0.241032	0.314921
Ciclev10015698m.g	scaffold_2:32106219-32109319	8.61805	10.7206	0.314958
Ciclev10029616m.g	scaffold_8:24262616-24263799	2.53894	3.15847	0.315001
Ciclev10000635m.g	scaffold_5:36931439-36936819	13.2836	16.5251	0.315017
Ciclev10016311m.g	scaffold_2:15707894-15710570	22.3992	27.8659	0.315051
Ciclev10006319m.g	scaffold_9:2486938-2487564	11.8493	14.7427	0.315202
Ciclev10031638m.g	scaffold_4:20103395-20105539	7.27966	9.05769	0.315273
Ciclev10021713m.g	scaffold_3:50647752-50652010	12.1737	15.1475	0.315306
Ciclev10005586m.g	scaffold_9:30833298-30836109	4.37968	5.44971	0.315354
Ciclev10027574m.g	scaffold_7:1359859-1363252	0.117751	0.146525	0.315401
Ciclev10006227m.g	scaffold_9:28472038-28474628	81.4868	101.409	0.315552
Ciclev10023651m.g,Ciclev10024077m.g	scaffold_3:49861789-49864636	31.713	39.472	0.315756
Ciclev10028542m.g	scaffold_8:2191754-2204285	68.9155	85.7851	0.315898
Ciclev10025458m.g	scaffold_7:11632511-11637032	27.9605	34.806	0.315947
Ciclev10016391m.g	scaffold_2:4495595-4498439	119.185	148.367	0.315975
Ciclev10022882m.g	scaffold_3:43358805-43360374	22.7655	28.3428	0.316133
Ciclev10005237m.g	scaffold_9:29756269-29758077	7.27979	9.06361	0.316189
Ciclev10016157m.g	scaffold_2:29877985-29882527	14.8795	18.526	0.316231
Ciclev10016124m.g	scaffold_2:28141839-28144921	48.767	60.7351	0.316624
Ciclev10025275m.g	scaffold_7:3801149-3805118	8.88838	11.0707	0.316752

Ciclev10026058m.g	scaffold_7:3182425-3186292	25.0296	31.1767	0.316833
-	scaffold_2:15441406-15442024	3.30373	4.11527	0.316892
Ciclev10021280m.g	scaffold_3:50821998-50823393	4.2177	5.25381	0.316909
Ciclev10010959m.g	scaffold_6:21380951-21388541	28.8333	35.9187	0.316999
Ciclev10021088m.g	scaffold_3:21069935-21074261	154.397	192.373	0.317257
Ciclev10032300m.g	scaffold_4:1766647-1769955	9.2281	11.4998	0.3175
Ciclev10006024m.g	scaffold_9:29523785-29526379	277.336	345.613	0.31752
Ciclev10002678m.g	scaffold_5:24867957-24869641	106.134	132.274	0.317651
Ciclev10009349m.g	scaffold_1:177115-180417	89.4748	111.516	0.317701
Ciclev10008484m.g	scaffold_1:22021975-22031696	9.83218	12.2547	0.317749
Ciclev10024254m.g	scaffold_3:32907630-32909384	11.9951	14.9551	0.318192
Ciclev10022688m.g	scaffold_3:35784883-35785592	64.15	79.9803	0.318196
Ciclev10009913m.g	scaffold_1:7776969-7783395	0.980213	1.22219	0.318306
Ciclev10021533m.g	scaffold_3:7147477-7151091	23.2402	28.9787	0.318371
Ciclev10028135m.g	scaffold_8:24387591-24391895	5.76962	7.19475	0.318468
Ciclev10020011m.g	scaffold_3:28391011-28394696	5.51813	6.88306	0.318872
Ciclev10014419m.g	scaffold_2:33039313-33044618	0.456138	0.56897	0.318883
Ciclev10033123m.g	scaffold_4:22085156-22087099	28.5842	35.655	0.318883
Ciclev10000887m.g	scaffold_5:38059018-38064414	25.9635	32.3876	0.318955
Ciclev10008796m.g	scaffold_1:4984339-4987674	21.1371	26.3674	0.318976
Ciclev10028055m.g	scaffold_8:2646457-2649562	12.2997	15.3454	0.319177
Ciclev10018445m.g	scaffold_3:44970295-44986898	6.48642	8.0928	0.319216
Ciclev10006172m.g	scaffold_9:8435309-8438953	108.045	134.816	0.31937
Ciclev10026457m.g	scaffold_7:5173845-5179864	3.59722	4.48942	0.319646
Ciclev10014148m.g	scaffold_2:6650711-6656534	5.69902	7.11256	0.319655
Ciclev10030734m.g,Ciclev10030791m.g,Ciclev10031085m.g,Ciclev10033534m.g	scaffold_4:19001055-19040863	10.3516	12.9194	0.319686
Ciclev10012289m.g	scaffold_6:20917328-20919808	17.7825	22.199	0.320032
Ciclev10020922m.g	scaffold_3:4412462-4414352	6.68457	8.34519	0.320111
Ciclev10002183m.g	scaffold_5:15208665-15210042	12.9457	16.1625	0.32018
Ciclev10028067m.g	scaffold_8:3069765-3072920	63.798	79.6578	0.320306
-	scaffold_3:12619164-12622879	6.43816	8.03898	0.320365
Ciclev10014647m.g	scaffold_2:31572992-31576949	3.19961	3.99531	0.32041
Ciclev10014072m.g	scaffold_2:7336520-7346103	7.38206	9.2181	0.320445
Ciclev10031065m.g	scaffold_4:2519502-2522909	10.8919	13.6082	0.321226
Ciclev10020493m.g	scaffold_3:10019296-10022106	12.0974	15.1167	0.321445
Ciclev10018742m.g	scaffold_3:20908232-20916016	48.2309	60.2715	0.321518
Ciclev10028980m.g	scaffold_8:20946475-20949954	55.1922	68.9711	0.321527
Ciclev10008509m.g	scaffold_1:16319157-16321963	61.8268	77.2703	0.321681
-	scaffold_3:48869425-48871547	35.0393	43.7961	0.321829
Ciclev10012204m.g	scaffold_6:19745965-19748081	155.156	193.937	0.321873
Ciclev10029636m.g	scaffold_8:451251-453295	19.4411	24.3029	0.322014
Ciclev10028294m.g	scaffold_8:22191055-22193808	31.9934	39.997	0.322117
Ciclev10019285m.g	scaffold_3:48693337-48699105	11.3848	14.2351	0.322337
Ciclev10030936m.g	scaffold_4:11824153-11826442	1.41212	1.76579	0.322454
Ciclev10029227m.g	scaffold_8:7261107-7264467	12.624	15.7893	0.32278
Ciclev10011985m.g	scaffold_6:16614756-16619472	73.4043	91.8119	0.322816
Ciclev10032614m.g	scaffold_4:3836241-3838823	26.8243	33.5511	0.322818
Ciclev10021443m.g	scaffold_3:43874917-43880311	22.4519	28.0843	0.322926
Ciclev10023247m.g	scaffold_3:27989279-27990984	86.9398	108.76	0.323053
Ciclev10019462m.g	scaffold_3:6072459-6076187	56.142	70.2325	0.323058
Ciclev10021944m.g	scaffold_3:42003586-42008479	19.7155	24.6673	0.32327
Ciclev10027794m.g	scaffold_8:22730838-22737522	101.166	126.577	0.323299
Ciclev10007688m.g	scaffold_1:23184868-23193259	20.6697	25.8651	0.32349
Ciclev10010345m.g	scaffold_1:1496324-1502126	4.08059	5.10889	0.324233

Ciclev10007528m.g	scaffold_1:19905420-19995040	0.678425	0.849411	0.324274
Ciclev10004154m.g	scaffold_9:24666091-24671850	303.146	379.558	0.324306
Ciclev10022785m.g	scaffold_3:42456298-42460637	8.06436	10.0973	0.324342
Ciclev10014152m.g	scaffold_2:10782894-10791612	14.5627	18.2343	0.324379
Ciclev10020308m.g	scaffold_3:915817-919213	6.8418	8.56694	0.324404
Ciclev10000034m.g	scaffold_5:38595157-38602837	43.5897	54.588	0.324597
Ciclev10029076m.g	scaffold_8:20889875-20892918	6.14952	7.70145	0.324655
Ciclev10012522m.g	scaffold_6:23748846-23755143	60.5154	75.8007	0.324909
Ciclev10032343m.g	scaffold_4:17037503-17040349	11.9473	14.9671	0.325114
Ciclev10019614m.g	scaffold_3:4883123-4889593	27.1649	34.0313	0.325116
Ciclev10000704m.g	scaffold_5:33886877-33891304	10.324	12.9363	0.325427
Ciclev10033177m.g	scaffold_4:3167270-3169664	8.27644	10.3709	0.325463
Ciclev10013689m.g	scaffold_6:19432058-19432872	15.4712	19.3928	0.325934
Ciclev10024774m.g	scaffold_7:3154778-3159259	0.675267	0.846511	0.326071
Ciclev10012907m.g	scaffold_6:18509231-18510088	0.90252	1.1314	0.326076
Ciclev10025723m.g	scaffold_7:1805025-1808908	38.6605	48.4754	0.326394
Ciclev10009303m.g	scaffold_1:18482167-18486041	43.2062	54.1752	0.326395
Ciclev10004227m.g	scaffold_9:225970-232459	30.5205	38.2705	0.326454
Ciclev10031098m.g	scaffold_4:21994422-21997477	3.93673	4.93731	0.326727
Ciclev10024590m.g	scaffold_3:46338208-46345383	0.640584	0.803432	0.326788
Ciclev10025987m.g	scaffold_7:19156687-19162504	19.6831	24.6874	0.32682
Ciclev10015647m.g	scaffold_2:31964919-31969078	19.0315	23.8713	0.326888
Ciclev10029382m.g,Ciclev10029454m.g	scaffold_8:10593474-10661453	270.6	339.437	0.326986
Ciclev10026216m.g	scaffold_7:2425291-2426491	26.5304	33.2842	0.327192
Ciclev10017753m.g	scaffold_2:24034061-24286273	33.3182	41.803	0.327296
Ciclev10018546m.g	scaffold_3:4322397-4335550	17.9142	22.4765	0.327317
Ciclev10021205m.g	scaffold_3:8556240-8560871	10.517	13.1957	0.327352
Ciclev10031500m.g	scaffold_4:16272830-16282393	25.2393	31.6732	0.32759
-	scaffold_1:28675682-28676323	98.3022	123.373	0.327731
Ciclev10025214m.g	scaffold_7:4257374-4262465	14.6745	18.4181	0.327818
Ciclev10030390m.g	scaffold_8:23783947-23790202	52.9578	66.4781	0.328036
Ciclev10020727m.g	scaffold_3:1608779-1611774	25.4881	32.0006	0.328274
Ciclev10018588m.g	scaffold_3:3220003-3226144	3.2249	4.04899	0.328308
Ciclev10032568m.g	scaffold_4:3972722-3974668	1783.14	2238.85	0.328337
Ciclev10030873m.g	scaffold_4:24176880-24183219	5.73675	7.20288	0.32834
Ciclev10031774m.g	scaffold_4:16691368-16694266	99.9859	125.539	0.328345
Ciclev10001261m.g	scaffold_5:19027226-19032242	32.5225	40.8419	0.32861
Ciclev10005189m.g	scaffold_9:25543540-25545766	2.05922	2.58626	0.328769
Ciclev10018622m.g,Ciclev10023585m.g,Ciclev10023855m.g	scaffold_3:48241315-48261070	9.11677	11.4502	0.328781
Ciclev10028948m.g	scaffold_8:22948142-22952227	5.82436	7.31555	0.328867
Ciclev10005216m.g	scaffold_9:5483167-5487314	46.9093	58.9409	0.329396
Ciclev10019485m.g	scaffold_3:9726822-9731547	28.6295	35.973	0.329414
-	scaffold_5:3980660-4069314	1.39928	1.75821	0.329418
Ciclev10019068m.g	scaffold_3:650873-656557	18.4723	23.2119	0.329499
Ciclev10009689m.g	scaffold_1:21462012-21463528	153.263	192.59	0.329521
Ciclev10004688m.g,Ciclev10004901m.g,Ciclev10007168m.g	scaffold_9:30382985-30428893	9.62764	12.0985	0.329576
Ciclev10030960m.g	scaffold_4:23930631-23935744	7.21755	9.07038	0.329653
Ciclev10001334m.g	scaffold_5:41609583-41613450	26.6994	33.5537	0.329661
Ciclev10025010m.g	scaffold_7:9967693-9970991	20.9445	26.322	0.329698
Ciclev10004168m.g	scaffold_9:5739222-5749116	5.23332	6.57735	0.329779
-	scaffold_5:2189013-2189342	948.651	1192.38	0.329897

Ciclev10014865m.g	scaffold_2:22956757-22961853	8.3813	10.5359	0.330063
Ciclev10028261m.g	scaffold_8:15349-20600	56.6856	71.2716	0.330344
Ciclev10005313m.g	scaffold_9:140763-142940	6.87491	8.64398	0.330355
Ciclev10014451m.g	scaffold_2:24533043-24538470	89.8248	112.941	0.330381
Ciclev10008497m.g	scaffold_1:8812352-8816597	37.2187	46.7994	0.330462
Ciclev10009868m.g	scaffold_1:8377488-8379348	150.718	189.546	0.330697
Ciclev10004336m.g	scaffold_9:750422-755981	6.20937	7.80992	0.330861
Ciclev10013579m.g	scaffold_6:17582724-17583288	9.36432	11.7784	0.330892
Ciclev10018402m.g	scaffold_2:10029871-10032264	7.39445	9.30078	0.330907
Ciclev10004661m.g	scaffold_9:31284616-31288904	28.4625	35.8013	0.330951
Ciclev10022976m.g	scaffold_3:12716925-12717904	7.84966	9.87422	0.331037
Ciclev10006034m.g	scaffold_9:30984775-30985561	11.5787	14.5651	0.331042
Ciclev10020836m.g	scaffold_3:9431444-9441519	60.1529	75.6681	0.331049
Ciclev10015491m.g	scaffold_2:32692306-32695026	1.46951	1.84878	0.33124
Ciclev10010492m.g	scaffold_1:12045356-12047858	28.983	36.4643	0.331278
Ciclev10011201m.g	scaffold_6:19840880-19845215	162.11	203.968	0.331367
Ciclev10002844m.g	scaffold_5:34465299-34467958	16.0308	20.1701	0.33137
Ciclev10016572m.g	scaffold_2:34899671-34901698	13.9128	17.506	0.331437
Ciclev10033792m.g	scaffold_4:1433548-1437578	16.1216	20.2913	0.331865
Ciclev10011764m.g	scaffold_6:20836508-20840107	249.316	313.807	0.331899
Ciclev10023071m.g	scaffold_3:27798972-27801782	38.8181	48.8638	0.332036
Ciclev10021354m.g	scaffold_3:41319152-41323763	6.17291	7.77052	0.332061
Ciclev10017195m.g	scaffold_2:27785944-27788087	17.8658	22.4907	0.332126
Ciclev10033366m.g	scaffold_4:199616-201281	20.5202	25.8388	0.332489
Ciclev10000095m.g	scaffold_5:42827733-42834567	23.2657	29.2967	0.332534
Ciclev10021162m.g	scaffold_3:12984410-12986574	120.443	151.703	0.332904
Ciclev10027966m.g	scaffold_8:23335673-23341669	7.07034	8.90619	0.333029
Ciclev10011139m.g	scaffold_6:9568946-9576968	17.0794	21.5154	0.333111
Ciclev10014943m.g	scaffold_2:33206461-33209078	4.55479	5.73791	0.333141
Ciclev10020300m.g	scaffold_3:43123639-43131063	31.3847	39.5416	0.333307
Ciclev10025305m.g	scaffold_7:9212797-9232329	6.07101	7.65131	0.33377
Ciclev10013024m.g	scaffold_6:15224327-15225010	2.92412	3.68553	0.33387
Ciclev10016213m.g	scaffold_2:6611812-6619116	19.9961	25.2033	0.333895
Ciclev10016867m.g	scaffold_2:13627649-13630405	98.8648	124.614	0.333941
Ciclev10019360m.g	scaffold_3:693335-696610	25.9681	32.7316	0.333942
Ciclev10029691m.g	scaffold_8:22694105-22694963	2.47119	3.11511	0.334077
Ciclev10004888m.g	scaffold_9:25378274-25383336	23.201	29.2503	0.334263
Ciclev10018162m.g	scaffold_2:21023663-21025851	0.501161	0.632047	0.334755
Ciclev10020337m.g	scaffold_3:14878426-14882285	240.994	303.989	0.335026
Ciclev10004185m.g	scaffold_9:494940-503720	11.9776	15.11	0.335171
Ciclev10023329m.g	scaffold_3:4870168-4876564	6.17877	7.795	0.335227
Ciclev10024823m.g	scaffold_7:14470076-14480739	10.7059	13.5069	0.335281
Ciclev10005080m.g	scaffold_9:19413341-19421005	36.9431	46.6098	0.335327
Ciclev10006041m.g	scaffold_9:27616921-27620591	26.6755	33.6643	0.335704
Ciclev10008685m.g	scaffold_1:22840946-22844111	36.2347	45.7316	0.335819
Ciclev10031032m.g	scaffold_4:18359027-18365121	31.4099	39.6439	0.335878
Ciclev10000596m.g	scaffold_5:28837054-28840605	103.984	131.252	0.335977
Ciclev10005535m.g	scaffold_9:2435839-2439595	23.0223	29.0632	0.336164
Ciclev10023137m.g	scaffold_3:48407101-48407775	52.7833	66.6385	0.336273
Ciclev10016076m.g	scaffold_2:29247203-29252039	44.648	56.3716	0.336373
Ciclev10015832m.g	scaffold_2:557158-558667	12.9222	16.3161	0.33644
Ciclev10004265m.g	scaffold_9:25808766-25812748	0.203866	0.257426	0.33654
Ciclev10018471m.g	scaffold_3:2837632-2845138	25.162	31.7735	0.33658
Ciclev10032297m.g	scaffold_4:8198786-8204052	18.3392	23.1618	0.336814
Ciclev10022531m.g	scaffold_3:45244126-45245482	11.8547	14.9727	0.33688
Ciclev10004155m.g	scaffold_9:2272859-2279135	0.0805754	0.101771	0.336912

Ciclev10013159m.g	scaffold_6:23718658-23720066	9.11312	11.5104	0.336923
Ciclev10024320m.g	scaffold_3:3044715-3048272	22.4534	28.3606	0.336955
Ciclev10030681m.g	scaffold_4:16111311-16119023	43.3651	54.7756	0.336998
Ciclev10007935m.g	scaffold_1:8830276-8834089	5.21086	6.5827	0.337158
Ciclev10028235m.g	scaffold_8:393478-397894	2.86567	3.62021	0.337201
Ciclev10009170m.g	scaffold_1:26431494-26432570	40.3211	50.94	0.337263
Ciclev10005909m.g	scaffold_9:1372971-1374927	271.996	343.633	0.337282
Ciclev10031889m.g	scaffold_4:19626060-19629792	91.9095	116.125	0.337398
Ciclev10002163m.g	scaffold_5:36827104-36829909	44.1918	55.842	0.33757
Ciclev10007241m.g	scaffold_1:28696649-28708116	15.4316	19.5004	0.337615
Ciclev10021114m.g	scaffold_3:45754690-45756062	14.7962	18.6977	0.337634
Ciclev10027579m.g	scaffold_7:11062213-11079547	3.38634	4.28075	0.338137
Ciclev10008491m.g	scaffold_1:1005746-1010149	22.2933	28.1839	0.338264
Ciclev10027765m.g	scaffold_8:267244-272712	19.4019	24.5346	0.338614
Ciclev10018755m.g	scaffold_3:50423258-50430465	21.0142	26.5734	0.338623
Ciclev10027932m.g	scaffold_8:21791314-21794464	2.93243	3.70825	0.338639
Ciclev10014964m.g	scaffold_2:13941408-13949243	7.40097	9.35973	0.338752
Ciclev10008167m.g	scaffold_1:2880393-2885069	5.88726	7.44559	0.338789
Ciclev10000963m.g	scaffold_5:17163767-17168691	22.3546	28.2777	0.339094
Ciclev10027719m.g	scaffold_8:19809452-19818719	9.5235	12.0469	0.339104
Ciclev10026217m.g	scaffold_7:17393387-17399502	5.55188	7.02307	0.339124
Ciclev10015197m.g	scaffold_2:8275058-8283003	21.584	27.3079	0.339355
Ciclev10000490m.g	scaffold_5:29131688-29150431	25.5607	32.3437	0.339561
Ciclev10008220m.g	scaffold_1:18361230-18369626	3.46448	4.38414	0.339652
Ciclev10005493m.g	scaffold_9:11173861-11181012	10.7873	13.651	0.339669
Ciclev10014187m.g	scaffold_2:32926141-32932573	15.2519	19.3029	0.339827
Ciclev10002770m.g	scaffold_5:29463603-29466318	149.149	188.77	0.339875
Ciclev10030636m.g	scaffold_4:21103927-21113129	16.7226	21.1654	0.339906
Ciclev10030381m.g	scaffold_8:4023181-4024953	2.76615	3.5016	0.340137
Ciclev10009103m.g	scaffold_1:18476998-18480284	87.723	111.065	0.340383
Ciclev10014476m.g	scaffold_2:34288240-34291335	10.7987	13.6729	0.340465
Ciclev10031191m.g	scaffold_4:21861773-21865635	41.1973	52.1691	0.340645
Ciclev10032209m.g	scaffold_4:75212-77797	36.8474	46.6628	0.340712
Ciclev10031806m.g	scaffold_4:25472626-25475236	63.0868	79.8924	0.340721
Ciclev10018856m.g	scaffold_3:10029256-10039415	31.7147	40.1776	0.341241
Ciclev10013903m.g	scaffold_6:23975736-23977958	21.1425	26.7845	0.341255
Ciclev10030048m.g	scaffold_8:19218721-19223038	17.2849	21.9015	0.341516
Ciclev10000725m.g	scaffold_5:37904978-37910963	4.20265	5.3255	0.341617
Ciclev10025635m.g	scaffold_7:4015734-4022130	29.0259	36.7847	0.341765
Ciclev10001045m.g	scaffold_5:33343273-33347990	11.8475	15.017	0.342011
Ciclev10000422m.g	scaffold_5:42541636-42545268	494.462	626.882	0.342335
Ciclev10012456m.g	scaffold_6:17225616-17227093	690.395	875.408	0.342535
Ciclev10022877m.g	scaffold_3:50808693-50810356	28.996	36.7674	0.342572
Ciclev10022376m.g	scaffold_3:144464-146231	23.7979	30.177	0.342613
Ciclev10027327m.g	scaffold_7:3825287-3830771	25.7853	32.6993	0.342708
Ciclev10028967m.g	scaffold_8:1359783-1363806	87.5529	111.044	0.342905
Ciclev10026026m.g	scaffold_7:8726545-8730256	11.7006	14.8417	0.343074
Ciclev10000027m.g	scaffold_5:36903339-36915125	26.5651	33.6976	0.343115
Ciclev10011572m.g	scaffold_6:18368750-18372284	46.3688	58.828	0.343349
Ciclev10025809m.g	scaffold_7:1861022-1863868	16.4523	20.8763	0.343581
Ciclev10013172m.g	scaffold_6:16886073-16887558	4.36194	5.53503	0.343623
Ciclev10014213m.g	scaffold_2:35863287-35870345	21.6538	27.4859	0.344069
Ciclev10004876m.g	scaffold_9:28745697-28747208	2.72309	3.45678	0.344181
Ciclev10002432m.g	scaffold_5:7242375-7246665	8.26869	10.4973	0.344282
Ciclev10031893m.g	scaffold_4:25446697-25450233	19.1926	24.3663	0.344335
Ciclev10000910m.g	scaffold_5:40872792-40877775	30.5436	38.7798	0.344436

Ciclev10005565m.g	scaffold_9:12054497-12057375	21.1596	26.8664	0.344488
Ciclev10017125m.g	scaffold_2:32027842-32029978	32.189	40.8728	0.344573
Ciclev10028545m.g	scaffold_8:3238949-3242586	36.3633	46.1743	0.344607
Ciclev10017589m.g	scaffold_2:29110324-29112979	1.80025	2.28616	0.344726
Ciclev10004807m.g	scaffold_9:234274-236341	13.132	16.6784	0.344897
Ciclev10014529m.g	scaffold_2:7155713-7161520	0.182989	0.232411	0.344921
Ciclev10020027m.g	scaffold_3:4366801-4370111	25.2369	32.0553	0.345028
Ciclev10008052m.g	scaffold_1:27783418-27789937	19.3084	24.5264	0.345105
-	scaffold_3:10423835-10425663	8.66526	11.0078	0.345205
Ciclev10001001m.g	scaffold_5:36807244-36812356	25.5616	32.4723	0.345235
Ciclev10021852m.g	scaffold_3:37171844-37174369	8.50617	10.8075	0.345445
Ciclev10016161m.g	scaffold_2:29963275-29967760	4.91702	6.24747	0.345486
Ciclev10024751m.g	scaffold_7:4486952-4494209	17.6081	22.3727	0.345506
-	scaffold_1:5168820-5170757	9.13316	11.6053	0.345602
Ciclev10010526m.g	scaffold_1:3533435-3545037	6.22118	7.90576	0.345716
-	scaffold_1:5182853-5184073	19.6139	24.9264	0.345799
Ciclev10027670m.g	scaffold_8:1032128-1042803	33.3389	42.3702	0.345845
Ciclev10006177m.g	scaffold_9:6809424-6812481	251.221	319.28	0.345863
Ciclev10020348m.g	scaffold_3:44450601-44453436	28.481	36.2033	0.346123
Ciclev10025674m.g	scaffold_7:4479338-4484907	48.2294	61.3071	0.346142
Ciclev10030969m.g	scaffold_4:5053539-5061039	54.4124	69.1678	0.346166
Ciclev10019751m.g	scaffold_3:49959219-49961400	5.62403	7.14956	0.34625
Ciclev10011053m.g	scaffold_6:14918021-14920714	4.20808	5.3496	0.346267
Ciclev10016798m.g	scaffold_2:32627020-32628215	16.5486	21.0382	0.346302
Ciclev10002110m.g	scaffold_5:41177873-41181556	44.5378	56.6302	0.346542
Ciclev10028399m.g	scaffold_8:3232604-3236570	9.78827	12.4463	0.346597
Ciclev10012950m.g	scaffold_6:22467564-22470029	91.3741	116.196	0.346705
Ciclev10002993m.g	scaffold_5:37060930-37065943	0.950389	1.20878	0.346962
Ciclev10000551m.g	scaffold_5:26539930-26542156	4.14923	5.27762	0.347044
-	scaffold_2:35022458-35022649	123.06	156.531	0.347078
Ciclev10012647m.g	scaffold_6:15141548-15143362	33.2825	42.3361	0.347122
Ciclev10019292m.g	scaffold_3:43538600-43544145	0.297005	0.377819	0.347205
Ciclev10008073m.g,Ciclev10008411m.g	scaffold_1:23265680-23304211	24.9726	31.7683	0.34724
Ciclev10018786m.g	scaffold_3:1662829-1669591	29.4102	37.4165	0.347357
Ciclev10002076m.g	scaffold_5:34056983-34060701	4.18511	5.32461	0.347412
Ciclev10018722m.g	scaffold_3:21171042-21177148	0.350676	0.446173	0.347464
Ciclev10007288m.g	scaffold_1:25606486-25610642	4.19006	5.33111	0.347466
Ciclev10024130m.g	scaffold_3:30250018-30252266	1.43792	1.83013	0.347956
Ciclev10004338m.g	scaffold_9:26659422-26664691	27.2418	34.6721	0.347956
Ciclev10011926m.g	scaffold_6:23319091-23323666	22.1096	28.1409	0.347992
Ciclev10022280m.g	scaffold_3:38329426-38331574	7.40733	9.42831	0.348046
Ciclev10022173m.g	scaffold_3:6232482-6233579	7.83931	9.97887	0.348149
Ciclev10014343m.g	scaffold_2:4490383-4495119	4.65441	5.92528	0.348284
Ciclev10018616m.g	scaffold_3:4049052-4056197	7.14114	9.09181	0.348413
Ciclev10001601m.g	scaffold_5:12246601-12250494	25.9614	33.0554	0.348516
Ciclev10000286m.g	scaffold_5:33846261-33853353	16.8556	21.4619	0.34855
Ciclev10000662m.g	scaffold_5:24313313-24318602	7.02076	8.93971	0.348601
Ciclev10003044m.g	scaffold_5:441330-538185	13.1375	16.7293	0.348688
Ciclev10029196m.g	scaffold_8:21151653-21156106	176.784	225.117	0.34869
Ciclev10017015m.g	scaffold_2:34087042-34089212	154.843	197.202	0.348868
Ciclev10008555m.g	scaffold_1:4308474-4312291	9.36579	11.9287	0.348964
Ciclev10000336m.g	scaffold_5:2059128-2063462	8.81987	11.2334	0.348968
Ciclev10026913m.g	scaffold_7:6602351-6604084	11.8061	15.0388	0.349157
Ciclev10022114m.g	scaffold_3:30018348-30021173	49.682	63.2863	0.34917
Ciclev10006509m.g	scaffold_9:3131005-3134438	27.697	35.2834	0.349261

Ciclev10010074m.g	scaffold_1:27889525-27892022	117.296	149.436	0.349372
Ciclev10015443m.g	scaffold_2:22189784-22194196	35.2878	44.9585	0.349426
Ciclev10026375m.g	scaffold_7:494440-497911	81.4112	103.726	0.349484
Ciclev10031427m.g	scaffold_4:1512951-1514924	13.5075	17.211	0.349568
Ciclev10033116m.g	scaffold_4:226031-227831	119.273	151.981	0.349627
Ciclev10025951m.g	scaffold_7:18356171-18358623	18.2074	23.2019	0.349719
Ciclev10032692m.g	scaffold_4:12533646-12536489	43.4771	55.4051	0.349764
Ciclev10020330m.g	scaffold_3:50341244-50345193	66.8457	85.2107	0.350202
Ciclev10025202m.g	scaffold_7:5059982-5064123	5.41972	6.90952	0.350365
Ciclev10028313m.g	scaffold_8:861283-864282	60.5021	77.1378	0.350451
Ciclev10004833m.g	scaffold_9:29371201-29374592	10.8193	13.7968	0.35072
Ciclev10016299m.g	scaffold_2:11071586-11074479	155.968	198.896	0.350763
Ciclev10000105m.g,Ciclev10002279m.g	scaffold_5:35093131-35113815	21.2254	27.0738	0.351105
Ciclev10014588m.g	scaffold_2:26607128-26612420	10.171	12.9742	0.351187
Ciclev10033454m.g,Ciclev10033883m.g	scaffold_4:769153-772689	79.7972	101.791	0.351202
-	scaffold_9:8972312-8972454	1038.61	1324.9	0.351229
Ciclev10000547m.g	scaffold_5:39534245-39541218	17.0052	21.6945	0.351355
Ciclev10020910m.g	scaffold_3:5756056-5758185	2.95909	3.77526	0.35142
Ciclev10022624m.g	scaffold_3:7577407-7579357	351.332	448.258	0.351495
Ciclev10001538m.g	scaffold_5:38949629-38951212	20.8247	26.573	0.351665
Ciclev10020134m.g	scaffold_3:39347955-39352797	26.6262	33.9784	0.35177
Ciclev10005566m.g	scaffold_9:26654934-26658646	15.0289	19.1819	0.352007
Ciclev10008764m.g	scaffold_1:5060314-5064215	9.15125	11.6805	0.352066
Ciclev10013395m.g	scaffold_6:25493131-25494652	23.6298	30.1667	0.35235
Ciclev10000043m.g	scaffold_5:43222581-43234732	6.78701	8.66513	0.352445
Ciclev10029779m.g	scaffold_8:24112693-24116620	3.02278	3.85927	0.35245
-	scaffold_5:20905961-20907848	31.388	40.0752	0.352496
Ciclev10020499m.g	scaffold_3:50102854-50104168	0.766295	0.978491	0.352657
Ciclev10014029m.g	scaffold_2:31625515-31644135	31.5961	40.3537	0.352955
Ciclev10020582m.g	scaffold_3:28382217-28386683	107.152	136.867	0.35312
Ciclev10030877m.g	scaffold_4:838949-843998	60.8482	77.7257	0.353177
Ciclev10018358m.g	scaffold_2:33685356-33688185	1.55663	1.9885	0.353249
Ciclev10028351m.g	scaffold_8:24136063-24140554	11.0009	14.0555	0.353518
Ciclev10019281m.g	scaffold_3:1948153-1950966	12.512	15.9866	0.353553
Ciclev10009097m.g	scaffold_1:28547992-28551484	7.83487	10.012	0.353754
Ciclev10013683m.g	scaffold_6:11898826-11905679	101.998	130.346	0.353803
Ciclev10030542m.g	scaffold_4:2802040-2815866	4.25431	5.43727	0.353959
Ciclev10025331m.g	scaffold_7:2484766-2491081	57.1052	72.986	0.353999
Ciclev10019391m.g	scaffold_3:42281250-42284493	5.17881	6.61918	0.354031
Ciclev10000033m.g	scaffold_5:39311466-39320010	0.0777688	0.0994037	0.354107
Ciclev10021517m.g	scaffold_3:34723498-34728378	17.7645	22.7086	0.354236
Ciclev10001500m.g,Ciclev10001502m.g	scaffold_5:38314988-38322884	1.39049	1.77796	0.354627
Ciclev10011620m.g	scaffold_6:24457464-24461462	25.0908	32.0833	0.354668
Ciclev10027522m.g	scaffold_7:8873633-8875382	2.70789	3.46264	0.354701
-	scaffold_1:8280407-8280681	15.2278	19.4734	0.354792
Ciclev10018449m.g	scaffold_3:47587083-47598488	13.2742	16.9777	0.35501
Ciclev10011157m.g	scaffold_6:24756003-24759888	11.4781	14.6821	0.355173
Ciclev10011737m.g	scaffold_6:8281830-8285987	11.3257	14.4882	0.355274
Ciclev10019953m.g	scaffold_3:6949944-6959530	11.3986	14.5822	0.355358
Ciclev10026429m.g	scaffold_7:3135537-3139969	7.96012	10.1835	0.355377
Ciclev10017304m.g	scaffold_2:35788709-35790440	16.7396	21.4159	0.355417
Ciclev10014127m.g	scaffold_2:10500878-10504450	10.7527	13.7576	0.355535
Ciclev10029178m.g	scaffold_8:24379629-24382620	10.7622	13.7702	0.355579

Ciclev10015078m.g	scaffold_2:11952936-11961596	22.1054	28.2846	0.355621
Ciclev10031043m.g,Ciclev10032266m.g	scaffold_4:2290942-2317254	7.64069	9.77852	0.355913
Ciclev10009202m.g	scaffold_1:1794519-1800274	3.38946	4.33783	0.355918
Ciclev10029299m.g	scaffold_8:1331738-1332770	14.5762	18.6578	0.356168
Ciclev10029967m.g	scaffold_8:5913053-5914658	1.76497	2.25928	0.356214
Ciclev10022055m.g	scaffold_3:20776396-20779125	41.7284	53.4155	0.35623
Ciclev10014516m.g	scaffold_2:11111610-11115358	21.5652	27.6059	0.356271
Ciclev10031149m.g	scaffold_4:18081442-18086436	25.4039	32.5247	0.356484
Ciclev10025712m.g	scaffold_7:11319640-11321606	13.1426	16.8334	0.357075
Ciclev10014475m.g	scaffold_2:31772844-31778082	4.91983	6.30199	0.3572
Ciclev10016165m.g	scaffold_2:15738335-15741219	69.4261	88.9535	0.357573
Ciclev10028002m.g	scaffold_8:20175420-20179264	83.3575	106.806	0.357607
Ciclev10012107m.g	scaffold_6:16172272-16174821	65.9519	84.5198	0.357874
Ciclev10010385m.g	scaffold_1:26433433-26436619	10.687	13.6973	0.35804
Ciclev10029861m.g	scaffold_8:24264194-24267006	0.840739	1.07764	0.358147
Ciclev10028708m.g	scaffold_8:18184921-18191230	20.2359	25.9389	0.358202
Ciclev10029467m.g	scaffold_8:23355304-23356236	17.1316	21.9599	0.358213
Ciclev10020105m.g	scaffold_3:28065477-28341901	0.65889	0.844624	0.358271
Ciclev10026225m.g	scaffold_7:1400120-1403926	20.3432	26.0783	0.3583
Ciclev10027740m.g	scaffold_8:22756918-22766817	16.4414	21.0767	0.358319
Ciclev10026349m.g	scaffold_7:5118957-5121870	9.093	11.6566	0.358324
Ciclev10022224m.g	scaffold_3:47076402-47078991	4.6884	6.01051	0.358393
Ciclev10002954m.g	scaffold_5:39321822-39323796	180.846	231.893	0.3587
Ciclev10001388m.g,Ciclev10001393m.g	scaffold_5:30931924-30957967	107.544	137.906	0.358765
Ciclev10009547m.g	scaffold_1:6209570-6213398	5.0445	6.46909	0.358852
Ciclev10022407m.g	scaffold_3:43406102-43407095	10.8685	13.938	0.358867
Ciclev10030613m.g	scaffold_4:5303403-5314184	18.7705	24.0742	0.359022
Ciclev10009372m.g,Ciclev10010273m.g	scaffold_1:2854970-2876895	11.7422	15.0613	0.359142
-	scaffold_1:21816478-21820539	14.1718	18.1789	0.359247
Ciclev10007287m.g	scaffold_1:4216721-4225971	21.4229	27.4813	0.359298
Ciclev10006282m.g	scaffold_9:4307053-4313440	10.137	13.0065	0.359599
Ciclev10010647m.g	scaffold_1:1082798-1089103	50.1354	64.3278	0.359613
Ciclev10015048m.g	scaffold_2:6098264-6102942	13.9348	17.8814	0.359761
Ciclev10031632m.g	scaffold_4:25216578-25218712	8.00206	10.2691	0.359864
Ciclev10012370m.g	scaffold_6:16218092-16219922	1823.96	2340.95	0.360016
Ciclev10011909m.g	scaffold_6:16660739-16664765	79.1123	101.539	0.360057
Ciclev10028835m.g	scaffold_8:13652115-13655271	29.2635	37.5646	0.360273
Ciclev10025637m.g	scaffold_7:16657828-16659304	1.78831	2.29573	0.360354
Ciclev10009440m.g	scaffold_1:6444378-6447194	15.0233	19.2869	0.360422
Ciclev10007209m.g	scaffold_9:6321270-6325120	5.0651	6.50397	0.36073
Ciclev10019837m.g	scaffold_3:32700392-32702382	0.198513	0.254918	0.360801
Ciclev10010988m.g	scaffold_6:24251935-24259572	3.63801	4.67196	0.360878
Ciclev10002701m.g	scaffold_5:655534-811195	4.14194	5.31966	0.361026
Ciclev10007295m.g	scaffold_1:11936428-11954075	28.3582	36.4216	0.361031
Ciclev10033889m.g	scaffold_4:484228-485287	0.939721	1.20704	0.361167
Ciclev10011084m.g	scaffold_6:13195954-13201816	39.9455	51.3112	0.361242
Ciclev10019785m.g	scaffold_3:10951100-10959102	10.9625	14.0849	0.361577
Ciclev10025358m.g	scaffold_7:2665762-2669981	3.33086	4.28058	0.361913
Ciclev10031470m.g	scaffold_4:4065004-4070480	5.60882	7.20834	0.361969
Ciclev10019267m.g	scaffold_3:7959987-7970188	13.912	17.8798	0.362
Ciclev10017363m.g	scaffold_2:11532048-11575076	1.94427	2.49881	0.362013
Ciclev10000952m.g	scaffold_5:38941332-38946200	6.22707	8.00424	0.362211
Ciclev10027213m.g	scaffold_7:164907-173806	9.1058	11.7064	0.362443

Ciclev10010666m.g	scaffold_1:22175413-22384190	0.225801	0.29029	0.362445
Ciclev10015603m.g	scaffold_2:30453564-30458751	23.7475	30.5299	0.362448
Ciclev10019188m.g	scaffold_3:9080654-9087568	8.63807	11.1063	0.362597
Ciclev10001624m.g	scaffold_5:20805064-20810920	31.4139	40.3915	0.362649
Ciclev10019082m.g	scaffold_3:1123496-1128139	16.6119	21.3595	0.36266
Ciclev10014232m.g	scaffold_2:4817715-4826072	0.115749	0.148868	0.363034
Ciclev10018463m.g	scaffold_3:49675353-49684423	18.0095	23.1634	0.36309
Ciclev10024938m.g	scaffold_7:1502113-1506805	13.2608	17.056	0.363111
Ciclev10032524m.g	scaffold_4:2153717-2155194	16.9123	21.7544	0.363238
Ciclev10001875m.g	scaffold_5:15870168-15874904	56.2758	72.3884	0.363245
Ciclev10021916m.g	scaffold_3:40946284-40949763	13.7259	17.6583	0.363447
Ciclev10012962m.g	scaffold_6:20248739-20252251	68.7391	88.4349	0.363483
Ciclev10021773m.g	scaffold_3:14757372-14761583	51.9452	66.8345	0.363601
Ciclev10010887m.g	scaffold_6:19789300-19805057	10.8787	13.997	0.363614
Ciclev10029539m.g	scaffold_8:23152673-23153469	29.2379	37.6215	0.363717
Ciclev10009052m.g	scaffold_1:23062783-23065398	1.30489	1.67917	0.363818
Ciclev10006171m.g	scaffold_9:28676565-28678921	27.5447	35.4469	0.363886
Ciclev10002202m.g	scaffold_5:41492324-41495372	24.339	31.3257	0.364075
Ciclev10010954m.g	scaffold_6:12508550-12514405	2.25495	2.90247	0.364191
Ciclev10023087m.g	scaffold_3:2689327-2691196	171.089	220.225	0.364229
Ciclev10000906m.g	scaffold_5:42545538-42550588	40.086	51.6079	0.364494
Ciclev10009673m.g	scaffold_1:4448305-4450980	19.5956	25.2291	0.36456
Ciclev10000011m.g	scaffold_5:41621905-41632132	9.86727	12.7045	0.36462
Ciclev10002610m.g	scaffold_5:37650547-37651925	3.14447	4.04915	0.364806
Ciclev10010774m.g	scaffold_1:2728028-2730088	0.589248	0.758937	0.365106
Ciclev10002816m.g	scaffold_5:39823361-39824171	1.21427	1.56418	0.365312
Ciclev10028844m.g	scaffold_8:6750380-6786902	74.2317	95.6267	0.365377
Ciclev10019610m.g	scaffold_3:36324292-36327333	1.42377	1.83417	0.365413
Ciclev10013792m.g	scaffold_6:17824212-17831388	17.1443	22.0862	0.365414
Ciclev10010791m.g	scaffold_1:4971922-4972522	13.0185	16.7728	0.365566
Ciclev10016367m.g	scaffold_2:8485505-8492511	18.4689	23.7954	0.365589
Ciclev10030880m.g	scaffold_4:3323208-3331825	20.1899	26.0143	0.365671
Ciclev10029879m.g	scaffold_8:9702708-9708895	25.0146	32.2321	0.365729
Ciclev10000121m.g	scaffold_5:38288013-38296170	23.2987	30.0217	0.365754
Ciclev10018753m.g	scaffold_3:41726822-41735092	12.4142	15.9973	0.365841
Ciclev10031343m.g	scaffold_4:15087930-15266082	5.20594	6.70854	0.365841
Ciclev10032700m.g	scaffold_4:18812053-18815478	7.71031	9.93625	0.365913
Ciclev10024745m.g	scaffold_7:13193244-13202485	85.44	110.112	0.365992
Ciclev10008775m.g	scaffold_1:5686984-5689140	0.464176	0.598302	0.366203
Ciclev10028420m.g	scaffold_8:23494553-23497202	4.1829	5.39187	0.36628
Ciclev10030651m.g	scaffold_4:6487066-6496028	17.5324	22.6007	0.366346
Ciclev10023392m.g	scaffold_3:35372249-35712253	0.791363	1.0202	0.366437
Ciclev10012602m.g	scaffold_6:24796171-24799882	72.7511	93.8048	0.366692
Ciclev10023769m.g	scaffold_3:24026647-24169357	3.95445	5.1002	0.367076
Ciclev10016354m.g	scaffold_2:25346438-25350942	34.3528	44.311	0.367234
Ciclev10002597m.g	scaffold_5:33627471-33629218	95.9687	123.798	0.367352
Ciclev10022849m.g	scaffold_3:46823028-46824764	35.5496	45.8622	0.367475
Ciclev10028904m.g	scaffold_8:5324791-5326835	89.8697	115.941	0.367487
Ciclev10009572m.g	scaffold_1:28339863-28345269	7.76953	10.0236	0.367495
Ciclev10008576m.g	scaffold_1:2127890-2131214	9.77835	12.6174	0.36775
Ciclev10020243m.g	scaffold_3:41039361-41041171	0.296981	0.383222	0.367813
Ciclev10005119m.g	scaffold_9:3115970-3120271	16.7116	21.5728	0.36836
Ciclev10026378m.g	scaffold_7:14253707-14256760	13.1694	17.0028	0.368585
Ciclev10031699m.g	scaffold_4:21266555-21268715	33.3231	43.0353	0.368999
Ciclev10003372m.g	scaffold_5:34757530-34762152	1.40591	1.81578	0.369088
Ciclev10030834m.g	scaffold_4:20459400-20463799	9.74871	12.5914	0.36916

Ciclev10024227m.g	scaffold_3:46103410-46106578	24.1604	31.2096	0.369349
Ciclev10025155m.g	scaffold_7:5113511-5118403	6.03517	7.79644	0.36942
Ciclev10024899m.g	scaffold_7:3817184-3824624	14.9442	19.3072	0.369554
Ciclev10013486m.g	scaffold_6:21244192-21246155	3.86255	4.99061	0.369664
Ciclev10018697m.g	scaffold_3:46989629-46996051	51.7631	66.8815	0.369684
Ciclev10000716m.g	scaffold_5:31000791-31004619	3.1335	4.0487	0.369684
Ciclev10031830m.g	scaffold_4:19593445-19596908	27.2491	35.2081	0.369699
Ciclev10014361m.g	scaffold_2:7429309-7438670	20.5144	26.512	0.370009
Ciclev10001075m.g	scaffold_5:12779470-12783622	14.293	18.472	0.370026
Ciclev10020258m.g	scaffold_3:49080359-49082763	6.69511	8.65276	0.370052
Ciclev10026316m.g	scaffold_7:17588985-17593243	17.0909	22.0924	0.370318
Ciclev10011383m.g	scaffold_6:17180986-17183517	1.97712	2.55584	0.370394
Ciclev10025220m.g	scaffold_7:723345-728003	7.89869	10.211	0.370443
Ciclev10005025m.g	scaffold_9:681174-685000	43.8268	56.6677	0.370713
Ciclev10020559m.g	scaffold_3:4071704-4075209	55.4125	71.6483	0.370722
Ciclev10015278m.g	scaffold_2:15950080-15958056	10.7148	13.8542	0.370724
Ciclev10008002m.g	scaffold_1:1001712-1005232	8.36419	10.8149	0.370725
Ciclev10014759m.g	scaffold_2:4222506-4227899	22.1738	28.6761	0.37099
Ciclev10016329m.g	scaffold_2:13136723-13138380	10.4101	13.4634	0.37106
Ciclev10026446m.g	scaffold_7:17095850-17097091	4.78245	6.1854	0.371117
Ciclev10031430m.g	scaffold_4:20327325-20329019	4.27927	5.53464	0.371126
Ciclev10025249m.g	scaffold_7:13180566-13191638	39.6164	51.2431	0.371262
Ciclev10010506m.g	scaffold_1:9688212-9695111	6.51499	8.4275	0.371343
Ciclev10021575m.g	scaffold_3:41743624-41746276	4.11991	5.33008	0.371543
Ciclev10028417m.g	scaffold_8:23257042-23262019	188.61	244.03	0.371654
Ciclev10017092m.g	scaffold_2:23335440-23336977	131.875	170.625	0.37166
Ciclev10002148m.g	scaffold_5:41337992-41344179	43.2101	55.9133	0.371823
Ciclev10018702m.g	scaffold_3:47925932-47931243	6.56381	8.49361	0.371844
Ciclev10025157m.g	scaffold_7:12188739-12191264	1.86417	2.41308	0.372344
Ciclev10006976m.g	scaffold_9:29549721-29554186	0.576272	0.745964	0.372356
Ciclev10032566m.g	scaffold_4:2999061-3001093	1.06106	1.37365	0.372497
Ciclev10014609m.g	scaffold_2:33640675-33645349	29.6719	38.4249	0.372944
Ciclev10011936m.g	scaffold_6:9974673-9977940	27.8377	36.0514	0.373013
Ciclev10018472m.g	scaffold_3:2393805-2401659	9.00828	11.6668	0.373091
Ciclev10012632m.g	scaffold_6:18719564-18722021	68.0644	88.1557	0.373153
Ciclev10012743m.g	scaffold_6:12231036-12235473	41.3107	53.5058	0.373179
Ciclev10021221m.g	scaffold_3:20523827-20528809	8.00115	10.3633	0.373205
Ciclev10022729m.g	scaffold_3:48472884-48473641	77.1136	99.9013	0.373519
Ciclev10030804m.g	scaffold_4:23811510-23814763	9.85281	12.7646	0.373542
Ciclev10009837m.g	scaffold_1:1981542-1982426	3.57408	4.63044	0.373578
Ciclev10011348m.g	scaffold_6:18753400-18755463	3.0213	3.91435	0.373603
Ciclev10012856m.g	scaffold_6:23127019-23129649	169.685	219.858	0.37372
Ciclev10027836m.g	scaffold_8:21900357-21910275	26.7544	34.666	0.373747
Ciclev10002746m.g	scaffold_5:33875642-33877205	88.3526	114.483	0.373794
Ciclev10031855m.g	scaffold_4:17927956-17930419	434.228	562.658	0.373805
Ciclev10025744m.g	scaffold_7:13625503-13630800	28.1333	36.4548	0.373831
Ciclev10001418m.g	scaffold_5:32320172-32324171	5.75475	7.45764	0.373966
Ciclev10003220m.g,Ciclev10003734m.g	scaffold_5:39329809-39334627	16.427	21.2894	0.37407
Ciclev10030657m.g	scaffold_4:17996647-17999365	0.237379	0.307649	0.374093
Ciclev10004536m.g	scaffold_9:6814447-6818302	10.0709	13.0533	0.374216
Ciclev10004819m.g	scaffold_9:14712838-14725102	25.8827	33.55	0.374325
Ciclev10007223m.g	scaffold_1:541339-551976	3.82557	4.95894	0.374358
Ciclev10014551m.g	scaffold_2:12781840-12788122	5.22282	6.77088	0.374515
Ciclev10013891m.g	scaffold_6:20618961-20620281	2.13591	2.7692	0.374619
Ciclev10018467m.g	scaffold_3:45438419-45449823	6.56949	8.5175	0.374649

Ciclev10023735m.g	scaffold_3:34853978-34861631	72.1134	93.502	0.374729
Ciclev10002589m.g	scaffold_5:5074553-5076191	7.69989	9.9837	0.374736
Ciclev10014913m.g	scaffold_2:32763173-32766220	4.81255	6.24071	0.374909
Ciclev10000541m.g	scaffold_5:32889613-32896199	32.6226	42.3062	0.374998
Ciclev10023374m.g	scaffold_3:25612740-25615635	5.60898	7.27432	0.375074
Ciclev10002705m.g	scaffold_5:32680868-32684767	21.599	28.0127	0.375112
Ciclev10007498m.g	scaffold_1:11865833-11878182	8.38187	10.8711	0.375152
Ciclev10031858m.g	scaffold_4:18878250-18882217	46.4627	60.2658	0.375266
Ciclev10031587m.g	scaffold_4:15473803-15476351	78.1817	101.41	0.375294
Ciclev10022400m.g	scaffold_3:44948129-44951395	8.45304	10.9648	0.375339
Ciclev10000534m.g	scaffold_5:40929752-40932152	10.2916	13.3502	0.375396
Ciclev10016475m.g	scaffold_2:26888062-26889923	3.65372	4.73961	0.375402
Ciclev10007389m.g	scaffold_1:20857811-20863600	15.9426	20.6869	0.375829
Ciclev10027673m.g	scaffold_8:6632694-6643461	20.4595	26.5489	0.375881
Ciclev10014396m.g	scaffold_2:36089465-36092894	104.472	135.57	0.375925
Ciclev10010078m.g	scaffold_1:11698811-11706621	42.7386	55.4702	0.376173
Ciclev10013177m.g	scaffold_6:14495472-14496662	22.8023	29.5969	0.376268
Ciclev10016757m.g	scaffold_2:9573422-9575967	10.9357	14.1976	0.376603
Ciclev10001753m.g	scaffold_5:38149173-38151063	29.8611	38.7737	0.37681
Ciclev10012291m.g	scaffold_6:12023784-12025466	201.746	261.967	0.376844
Ciclev10008033m.g	scaffold_1:12905196-12915469	11.0146	14.3028	0.376876
Ciclev10018541m.g	scaffold_3:47275734-47289214	9.73767	12.6451	0.376927
Ciclev10018587m.g	scaffold_3:270725-278847	14.2416	18.4957	0.377081
Ciclev10032693m.g	scaffold_4:22822636-22826553	60.2654	78.274	0.377204
Ciclev10010045m.g	scaffold_1:27592803-27596919	133.042	172.807	0.377284
Ciclev10005031m.g	scaffold_9:15066041-15068663	6.43157	8.35526	0.377513
Ciclev10010442m.g	scaffold_1:2140345-2141955	3.15645	4.10092	0.377643
Ciclev10015444m.g	scaffold_2:27738396-27741912	20.6391	26.817	0.377768
Ciclev10008059m.g	scaffold_1:23589729-23591591	3.69327	4.79917	0.377885
Ciclev10028562m.g	scaffold_8:994686-998018	25.801	33.5275	0.377922
Ciclev10009623m.g	scaffold_1:23436709-23437686	33.9924	44.1737	0.377976
Ciclev10020232m.g	scaffold_3:9137248-9144663	7.01731	9.11912	0.377977
Ciclev10028526m.g	scaffold_8:5313876-5318536	5.89176	7.65897	0.378451
Ciclev10030527m.g	scaffold_4:19931957-19937465	12.2384	15.9121	0.378719
Ciclev10014483m.g	scaffold_2:23929162-23932468	15.0889	19.6321	0.379727
Ciclev10007650m.g	scaffold_1:8979708-8984340	2.47732	3.22335	0.379785
Ciclev10001194m.g	scaffold_5:37517042-37521275	155.516	202.394	0.380103
Ciclev10014977m.g	scaffold_2:148004-151853	76.1897	99.1631	0.380207
Ciclev10006087m.g	scaffold_9:4069837-4073991	8.85328	11.5231	0.380248
Ciclev10004820m.g	scaffold_9:208242-212051	9.42959	12.2741	0.380348
Ciclev10005439m.g	scaffold_9:16705916-16709812	13.1	17.0518	0.380353
Ciclev10000631m.g	scaffold_5:21144033-21157038	16.5823	21.5869	0.380514
Ciclev10028141m.g	scaffold_8:4292179-4375670	29.6826	38.6491	0.380821
Ciclev10031274m.g	scaffold_4:22314014-22318040	50.0006	65.1333	0.381451
Ciclev10030940m.g	scaffold_4:7933256-7944285	10.6437	13.8666	0.381616
Ciclev10007398m.g	scaffold_1:22915348-22923749	26.6143	34.6789	0.381857
Ciclev10022293m.g	scaffold_3:39151265-39154101	50.2371	65.4615	0.381891
Ciclev10025015m.g	scaffold_7:4440812-4442969	3.49242	4.55091	0.381928
Ciclev10027849m.g	scaffold_8:9731065-9737070	10.7054	13.9508	0.382001
Ciclev10007737m.g	scaffold_1:2955387-2961523	6.61127	8.61572	0.382043
Ciclev10024418m.g	scaffold_3:4757860-4766772	15.2479	19.8712	0.382062
Ciclev10007259m.g	scaffold_1:4243732-4257303	5.65127	7.36518	0.382145
Ciclev10018583m.g	scaffold_3:12162689-12171238	39.0244	50.8665	0.382339
Ciclev10022948m.g	scaffold_3:47785149-47786297	74.5484	97.1827	0.382523
Ciclev10030563m.g	scaffold_4:18291264-18295600	4.27675	5.57593	0.382698
Ciclev10002156m.g	scaffold_5:27831223-27833396	8.07577	10.5303	0.382875

Ciclev10008715m.g	scaffold_1:15605748-15609914	3.26019	4.2511	0.382879
Ciclev10018315m.g	scaffold_2:5940580-5943655	0.147043	0.191737	0.382893
Ciclev10020158m.g	scaffold_3:25053424-25057795	101.24	132.046	0.383264
Ciclev10025899m.g	scaffold_7:9961356-9965060	22.9504	29.9364	0.383381
Ciclev10023018m.g	scaffold_3:45365368-45366088	326.584	425.997	0.383385
Ciclev10013281m.g	scaffold_6:8893557-8898998	7.42011	9.6789	0.383403
Ciclev10014076m.g,Ciclev10017843m.g	scaffold_2:26788689-26811737	18.2037	23.7472	0.38353
Ciclev10005766m.g	scaffold_9:3112678-3115800	779.783	1017.37	0.383702
Ciclev10019482m.g	scaffold_3:6941467-6945830	12.0319	15.6983	0.383737
Ciclev10020296m.g	scaffold_3:49467548-49470697	19.0062	24.7977	0.383742
Ciclev10008976m.g	scaffold_1:4961853-4965772	21.211	27.6806	0.384067
Ciclev10020428m.g	scaffold_3:41380330-41386081	38.365	50.0752	0.384305
Ciclev10008710m.g	scaffold_1:25471896-25476789	19.1919	25.0501	0.384318
Ciclev10024910m.g	scaffold_7:3683496-3690871	20.7076	27.0291	0.384353
Ciclev10000325m.g	scaffold_5:42492097-42494989	4.13075	5.39261	0.38458
Ciclev10020644m.g	scaffold_3:1459980-1463905	17.4401	22.7677	0.384581
Ciclev10021242m.g	scaffold_3:6246288-6250662	5.07497	6.626	0.384739
Ciclev10033023m.g	scaffold_4:14047934-14052770	4.44676	5.8058	0.384742
Ciclev10031387m.g	scaffold_4:24364995-24369841	27.4429	35.8311	0.384781
Ciclev10028255m.g	scaffold_8:12589086-12593855	65.0681	84.9639	0.3849
Ciclev10004941m.g	scaffold_9:6761756-6765743	70.0763	91.5062	0.384943
Ciclev10017227m.g	scaffold_2:29703941-29705676	21.1761	27.6551	0.385113
Ciclev10014082m.g	scaffold_2:7507436-7569782	1.32897	1.73585	0.385335
Ciclev10009478m.g	scaffold_1:4056975-4060133	17.2987	22.597	0.385471
Ciclev10009248m.g	scaffold_1:28859824-28861482	74.0211	96.6943	0.385493
Ciclev10012842m.g	scaffold_6:20532020-20533092	10.0964	13.189	0.385493
Ciclev10000367m.g	scaffold_5:30816504-30825183	28.0222	36.6065	0.385533
Ciclev10007602m.g	scaffold_1:6368607-6374391	1.39792	1.82642	0.38574
Ciclev10019200m.g	scaffold_3:951690-958430	95.087	124.235	0.385757
Ciclev10003606m.g	scaffold_5:39570141-39572700	9.38729	12.2663	0.385923
Ciclev10016439m.g	scaffold_2:32069148-32072079	119.367	156.022	0.386344
Ciclev10025873m.g	scaffold_7:4760356-4765845	48.9924	64.0381	0.386373
Ciclev10026727m.g	scaffold_7:14467676-14469593	196.428	256.782	0.386548
Ciclev10029509m.g	scaffold_8:19348442-19349831	4.0615	5.30944	0.386548
Ciclev10004203m.g	scaffold_9:13602271-13608573	12.3813	16.186	0.386582
Ciclev10024597m.g	scaffold_3:35330009-35331719	0.714805	0.934499	0.386644
Ciclev10014711m.g	scaffold_2:8816260-8822464	8.19265	10.7111	0.386702
Ciclev10031575m.g	scaffold_4:13725385-13730929	4.04501	5.28899	0.386851
Ciclev10026736m.g	scaffold_7:6677313-6680214	36.2075	47.3598	0.387374
Ciclev10031111m.g	scaffold_4:1816357-1819039	19.5489	25.572	0.387481
Ciclev10024255m.g	scaffold_3:45025942-45042250	3.53658	4.62684	0.387674
Ciclev10016345m.g	scaffold_2:29565209-29567118	4.09047	5.35158	0.387697
Ciclev10017669m.g	scaffold_2:36262362-36266216	4.43207	5.79939	0.387921
Ciclev10007527m.g	scaffold_1:11005473-11012720	13.5335	17.7097	0.388005
Ciclev10002003m.g	scaffold_5:27136303-27139094	38.709	50.6591	0.388153
Ciclev10000306m.g	scaffold_5:42841158-42844776	20.5637	26.9162	0.388375
Ciclev10030842m.g	scaffold_4:21437814-21441565	0.934357	1.22304	0.388425
Ciclev10028403m.g	scaffold_8:21293714-21298947	3.01406	3.94599	0.388679
Ciclev10004166m.g	scaffold_9:17003692-17022778	5.36925	7.02963	0.388729
Ciclev10014383m.g	scaffold_2:32006141-32011337	3.21103	4.2041	0.388761
Ciclev10007414m.g	scaffold_1:25631988-25639111	38.8571	50.8785	0.388875
Ciclev10017161m.g	scaffold_2:27873386-27877069	4.40189	5.76381	0.3889
Ciclev10019860m.g	scaffold_3:4286793-4290307	27.4237	35.9094	0.388936
Ciclev10003034m.g	scaffold_5:36769277-36769885	3.36969	4.41249	0.388977
Ciclev10013712m.g	scaffold_6:19414622-19417125	8.46775	11.0889	0.389064

Ciclev10020072m.g	scaffold_3:37935541-37937312	0.709596	0.929338	0.389207
Ciclev10002820m.g	scaffold_5:41248084-41248874	0.413892	0.54208	0.38925
Ciclev10018729m.g	scaffold_3:45423443-45429922	9.22393	12.0808	0.389259
Ciclev10014151m.g	scaffold_2:7980149-7987103	33.3674	43.7077	0.389448
Ciclev10021118m.g	scaffold_3:41974016-41979701	22.4879	29.4572	0.389469
Ciclev10025644m.g	scaffold_7:6756681-6759366	3.82963	5.01778	0.389842
Ciclev10031877m.g	scaffold_4:24928232-24930971	152.898	200.349	0.389947
Ciclev10010654m.g	scaffold_1:7060621-7066678	19.076	24.9964	0.389965
Ciclev10018158m.g	scaffold_2:12003611-12005635	9.81512	12.8625	0.390091
Ciclev10030421m.g	scaffold_8:20615764-20616605	2.80891	3.68203	0.39049
Ciclev10016465m.g	scaffold_2:33532218-33534581	23.893	31.3214	0.390563
Ciclev10005679m.g	scaffold_9:1831426-1834117	31.2484	40.9668	0.390673
Ciclev10011367m.g	scaffold_6:22450441-22453093	10.701	14.0306	0.390822
Ciclev10020927m.g	scaffold_3:28061371-28064319	34.3455	45.0338	0.390886
Ciclev10009227m.g	scaffold_1:21887855-21892059	4.62173	6.06113	0.391155
Ciclev10032329m.g	scaffold_4:6128951-6138477	48.6785	63.8458	0.391305
Ciclev10006472m.g	scaffold_9:29162416-29167434	12.0804	15.8451	0.391365
Ciclev10007635m.g	scaffold_1:3389308-3396208	26.7443	35.0793	0.391384
-	scaffold_7:18463431-18464175	12.6575	16.6035	0.391498
Ciclev10014515m.g	scaffold_2:7232447-7237635	4.33199	5.68277	0.391566
Ciclev10009981m.g	scaffold_1:957780-963845	69.2626	90.8642	0.391635
Ciclev10009798m.g	scaffold_1:8417157-8419377	233.936	306.938	0.391836
Ciclev10007408m.g	scaffold_1:25313331-25322034	19.339	25.3747	0.391878
Ciclev10005360m.g	scaffold_9:13255658-13295174	109.174	143.258	0.391978
Ciclev10019957m.g	scaffold_3:50801312-50805251	14.5774	19.1297	0.392081
Ciclev10007718m.g	scaffold_1:28033284-28037413	0.699464	0.918015	0.392268
Ciclev10021537m.g	scaffold_3:45788774-45791990	21.7365	28.5395	0.392838
Ciclev10023024m.g	scaffold_3:880710-890544	12.2439	16.078	0.393032
Ciclev10008422m.g	scaffold_1:4974434-4979185	61.2032	80.371	0.393068
Ciclev10017819m.g	scaffold_2:9852587-9853712	0.605825	0.795599	0.39314
Ciclev10012360m.g	scaffold_6:18876865-18879586	23.7546	31.1966	0.393181
Ciclev10024810m.g	scaffold_7:2687600-2691651	9.91428	13.0206	0.393217
Ciclev10020565m.g	scaffold_3:17486020-17494538	9.5768	12.5782	0.39331
Ciclev10015626m.g	scaffold_2:31399377-31401993	35.2243	46.2689	0.393472
Ciclev10012519m.g	scaffold_6:10580777-10582472	13.3793	17.5785	0.393811
Ciclev10026664m.g	scaffold_7:1661267-1663931	30.7594	40.4172	0.393944
Ciclev10011740m.g	scaffold_6:19835875-19838692	7.7345	10.1642	0.39411
Ciclev10027725m.g	scaffold_8:2379775-2387443	6.48253	8.51929	0.394175
Ciclev10008682m.g	scaffold_1:24541001-24544916	18.2675	24.0089	0.394292
Ciclev10021582m.g,Ciclev10022324m.g	scaffold_3:41291408-41300335	70.0292	92.0517	0.394487
Ciclev10026115m.g	scaffold_7:15773090-15776638	80.4412	105.756	0.394727
Ciclev10005922m.g	scaffold_9:3663678-3669508	34.2356	45.0135	0.394861
Ciclev10014378m.g,Ciclev10014696m.g	scaffold_2:22672194-22695586	13.2757	17.457	0.39502
Ciclev10032949m.g	scaffold_4:24710901-24711846	36.4789	47.9732	0.395165
Ciclev10005929m.g	scaffold_9:28864144-28867403	48.669	64.0088	0.395266
Ciclev10020073m.g	scaffold_3:1726801-1730229	5.31178	6.98598	0.395268
Ciclev10014178m.g	scaffold_2:33223844-33232034	18.4137	24.2192	0.395366
Ciclev10011756m.g	scaffold_6:22748168-22755153	52.1326	68.5691	0.395373
Ciclev10013029m.g,Ciclev10013030m.g	scaffold_6:21559345-21565310	994.739	1308.53	0.395555
Ciclev10003991m.g	scaffold_5:33199319-33203370	0.29739	0.391228	0.395651
Ciclev10031608m.g	scaffold_4:6362396-6366438	7.25431	9.54479	0.395875
Ciclev10031203m.g	scaffold_4:10694813-10697194	11.5856	15.2439	0.395903
Ciclev10008928m.g	scaffold_1:27254202-27255476	1.43937	1.89395	0.395956

Ciclev10011868m.g	scaffold_6:21348969-21352847	73.2224	96.3546	0.396069
Ciclev10012347m.g	scaffold_6:19186488-19187721	20.3015	26.7249	0.396594
Ciclev10022836m.g	scaffold_3:45823465-45826290	12.3979	16.3217	0.396692
Ciclev10032336m.g	scaffold_4:16147441-16149813	51.2695	67.5125	0.397053
Ciclev10022555m.g	scaffold_3:16054037-16057669	19.6362	25.8598	0.3972
Ciclev10019532m.g	scaffold_3:39867761-39873495	6.52253	8.58992	0.397212
Ciclev10011325m.g	scaffold_6:23702260-23707255	36.8969	48.5961	0.397341
Ciclev10029344m.g	scaffold_8:20305916-20308628	15.601	20.5479	0.39735
Ciclev10013528m.g	scaffold_6:6591968-6593827	1.40825	1.8548	0.397355
Ciclev10011457m.g	scaffold_6:11158255-11165190	4.17448	5.49834	0.397399
Ciclev10027665m.g	scaffold_8:3160690-3172690	6.02291	7.93312	0.397427
Ciclev10006782m.g,Ciclev10006920m.g	scaffold_9:27790597-27828145	9.46857	12.4723	0.397512
Ciclev10025762m.g	scaffold_7:2216625-2218133	10.3313	13.6088	0.397519
Ciclev10020600m.g	scaffold_3:1876220-1878308	82.5861	108.79	0.397574
Ciclev10001136m.g	scaffold_5:15966065-15975618	26.9411	35.4939	0.397762
Ciclev10017898m.g	scaffold_2:11652561-11654706	0.92218	1.21504	0.397884
Ciclev10026218m.g	scaffold_7:1954665-1955933	3.08549	4.06537	0.397888
Ciclev10007037m.g	scaffold_9:1782429-1785771	3.38594	4.46123	0.397888
Ciclev10003235m.g	scaffold_5:31333200-31391489	5.27647	6.95218	0.397893
Ciclev10019075m.g	scaffold_3:6643301-6650516	10.2821	13.5476	0.397908
Ciclev10027663m.g	scaffold_8:21712356-21744083	3.98056	5.24483	0.397927
Ciclev10030768m.g	scaffold_4:23882176-23886646	3.85174	5.07546	0.398027
Ciclev10019142m.g	scaffold_3:24339985-24345431	1.45091	1.912	0.398124
Ciclev10000772m.g	scaffold_5:41364332-41369139	54.7599	72.1634	0.398148
Ciclev10028446m.g	scaffold_8:22300307-22303175	199.042	262.316	0.398232
Ciclev10028691m.g	scaffold_8:138021-146284	19.4424	25.6264	0.398421
Ciclev10017395m.g	scaffold_2:7832348-7846578	10.216	13.4675	0.398658
Ciclev10013529m.g	scaffold_6:24282684-24285185	21.6539	28.5478	0.398749
Ciclev10027881m.g	scaffold_8:22084231-22091205	264.167	348.283	0.398813
Ciclev10010976m.g	scaffold_6:11707952-11715646	7.00159	9.23145	0.398876
Ciclev10004301m.g	scaffold_9:26878479-26884581	14.2058	18.7365	0.399372
Ciclev10028522m.g	scaffold_8:2915507-2917414	24.4581	32.2594	0.399408
Ciclev10012200m.g	scaffold_6:25579196-25583801	4.3565	5.74745	0.399753
Ciclev10002842m.g	scaffold_5:8622433-8624033	7.85267	10.36	0.399768
Ciclev10020346m.g	scaffold_3:6424581-6428757	10.9238	14.4129	0.399891
Ciclev10007137m.g	scaffold_9:7795066-7795573	181.998	240.136	0.399931
-	scaffold_5:36944481-36950082	19.7987	26.1242	0.399983
Ciclev10011347m.g	scaffold_6:22160125-22162288	1.25206	1.65209	0.399985
Ciclev10030619m.g	scaffold_4:2373165-2378041	8.44641	11.1461	0.400134
-	scaffold_3:12339596-12342370	16.7101	22.055	0.400386
Ciclev10011705m.g,Ciclev10013161m.g	scaffold_6:9265328-9277718	36.0687	47.6062	0.400401
Ciclev10018851m.g	scaffold_3:48824168-48832822	5.17832	6.83613	0.400698
Ciclev10002305m.g	scaffold_5:41037880-41041106	21.9856	29.0276	0.400863
Ciclev10027846m.g	scaffold_8:2511377-2514836	0.144581	0.190892	0.400874
Ciclev10007637m.g	scaffold_1:17987192-17993013	3.33506	4.40439	0.40123
Ciclev10028256m.g	scaffold_8:799315-803110	5.03787	6.65349	0.401297
Ciclev10027868m.g	scaffold_8:4377469-4390284	11.491	15.1772	0.401402
Ciclev10012281m.g	scaffold_6:24742938-24746629	5.07582	6.70456	0.401503
Ciclev10000237m.g	scaffold_5:36415987-36422893	0.347681	0.459247	0.401506
Ciclev10019506m.g	scaffold_3:39052186-39061631	43.2028	57.0773	0.401793
Ciclev10033332m.g,Ciclev10033993m.g	scaffold_4:3396835-3402031	8.62956	11.4023	0.40196
Ciclev10006104m.g	scaffold_9:30486452-30489765	4.43338	5.85951	0.402372
Ciclev10002838m.g	scaffold_5:41709037-41711037	18.8236	24.8798	0.402432

Ciclev10011928m.g	scaffold_6:19427392-19430050	10.4672	13.8357	0.402528
Ciclev10029491m.g,Ciclev10029776m.g	scaffold_8:10846488-10858019	19.8431	26.2316	0.402665
Ciclev10017162m.g	scaffold_2:33624491-33626591	171.873	227.216	0.402725
Ciclev10018980m.g	scaffold_3:47039795-47043212	16.0022	21.1577	0.402913
Ciclev10019043m.g	scaffold_3:524088-530553	18.1833	24.0417	0.402925
Ciclev10018430m.g	scaffold_3:50144423-50165188	14.8442	19.6291	0.403096
Ciclev10009122m.g	scaffold_1:21439963-21442808	6.55378	8.66638	0.403101
Ciclev10000001m.g	scaffold_5:35379160-35394898	32.7581	43.3246	0.403337
Ciclev10024696m.g	scaffold_7:6820362-6832533	12.5956	16.6596	0.403439
Ciclev10004395m.g	scaffold_9:1112880-1116366	12.4675	16.4917	0.403567
Ciclev10005076m.g	scaffold_9:1187245-1191347	11.3374	14.9984	0.40372
Ciclev10024740m.g	scaffold_7:1406406-1412310	15.6053	20.6449	0.403749
Ciclev10005822m.g	scaffold_9:3144171-3146938	46.9916	62.1746	0.403921
Ciclev10015889m.g	scaffold_2:36117529-36119293	4.97559	6.58376	0.404044
Ciclev10002520m.g	scaffold_5:34692572-34695351	14.7197	19.4782	0.404106
Ciclev10026622m.g	scaffold_7:18305585-18306712	16.1428	21.3612	0.404106
Ciclev10014253m.g	scaffold_2:409270-411829	1.25985	1.66728	0.404243
Ciclev10018586m.g	scaffold_3:39181844-39300715	0.303988	0.402303	0.404268
Ciclev10014046m.g	scaffold_2:36267215-36275208	29.8527	39.5092	0.404326
Ciclev10019863m.g	scaffold_3:2702697-2705509	5.48821	7.26443	0.404515
Ciclev10011016m.g,Ciclev10013535m.g	scaffold_6:5951659-5977928	0.690156	0.913605	0.404647
Ciclev10025096m.g	scaffold_7:15307863-15310026	6.28659	8.322	0.404651
Ciclev10009486m.g	scaffold_1:22111807-22117031	67.2077	88.9729	0.40474
Ciclev10025609m.g	scaffold_7:5668378-5673913	51.029	67.5607	0.404866
Ciclev10030601m.g	scaffold_4:19095795-19099241	5.20221	6.88762	0.404882
Ciclev10031253m.g	scaffold_4:3226867-3232418	4.64551	6.15071	0.404917
Ciclev10004418m.g,Ciclev10006432m.g	scaffold_9:4946621-4955112	44.6757	59.157	0.405057
Ciclev10015917m.g	scaffold_2:26206634-26210648	59.411	78.6772	0.405216
Ciclev10031078m.g	scaffold_4:13892611-13901497	16.641	22.0382	0.405267
Ciclev10032989m.g	scaffold_4:24203177-24206181	2.70282	3.5797	0.405375
Ciclev10010952m.g	scaffold_6:21062900-21071745	31.6111	41.8687	0.405439
Ciclev10001165m.g	scaffold_5:41865104-41869493	1.05468	1.39692	0.40544
Ciclev10010418m.g	scaffold_1:6262793-6298426	0.88361	1.17055	0.405708
Ciclev10008662m.g	scaffold_1:25898213-25903698	48.0593	63.6673	0.405735
Ciclev10027573m.g	scaffold_7:18341497-18350627	8.7988	11.6564	0.40574
Ciclev10026175m.g	scaffold_7:2941831-2945263	14.3848	19.0569	0.405768
Ciclev10008667m.g	scaffold_1:14257823-14261262	42.1186	55.8036	0.4059
-	scaffold_5:40657252-40657866	15.4334	20.4484	0.405925
Ciclev10018988m.g	scaffold_3:44270205-44278189	30.0244	39.7843	0.406067
Ciclev10002333m.g,Ciclev10004087m.g	scaffold_5:39046740-39085639	6.76669	8.96664	0.406117
Ciclev10004206m.g,Ciclev10006393m.g	scaffold_9:30974809-30979522	19.8226	26.2726	0.406411
Ciclev10009261m.g	scaffold_1:18107908-18109347	65.4514	86.7697	0.406768
Ciclev10003904m.g	scaffold_5:30793518-30797026	2.09893	2.78301	0.406997
Ciclev10007657m.g	scaffold_1:369607-374728	37.4135	49.6097	0.407065
Ciclev10001383m.g	scaffold_5:32759552-32763764	6.02879	7.99459	0.407156
Ciclev10028993m.g	scaffold_8:2241690-2245635	5.72958	7.59791	0.407171
Ciclev10000250m.g	scaffold_5:40754816-40761430	10.4697	13.8861	0.407422
Ciclev10019168m.g	scaffold_3:1245722-1249605	10.4993	13.9299	0.407899
Ciclev10005452m.g	scaffold_9:469600-474975	36.8099	48.8407	0.407991
Ciclev10031728m.g	scaffold_4:9880494-10159290	6.22213	8.25649	0.40812
-	scaffold_1:21860132-21862352	2.89693	3.84438	0.40823

Ciclev10005568m.g	scaffold_9:4566887-4573847	9.1475	12.1409	0.40842
Ciclev10002608m.g	scaffold_5:39938189-39940217	6.82146	9.05398	0.408473
Ciclev10008790m.g	scaffold_1:1713891-1717819	9.97067	13.2355	0.408646
-	scaffold_4:23827923-23832666	22.1907	29.4592	0.408764
Ciclev10019422m.g	scaffold_3:47305898-47308863	2.87985	3.82316	0.408773
Ciclev10025549m.g	scaffold_7:4156914-4159721	14.6201	19.4091	0.40878
Ciclev10025832m.g	scaffold_7:16619581-16622613	8.37399	11.1172	0.408802
Ciclev10011292m.g	scaffold_6:21878310-21883782	22.7427	30.2184	0.410022
Ciclev10021096m.g	scaffold_3:50541845-50547524	10.2637	13.6376	0.410041
Ciclev10008197m.g	scaffold_1:22771093-22774928	11.3055	15.0251	0.410355
Ciclev10032237m.g	scaffold_4:22215106-22218673	8.21388	10.9167	0.410401
Ciclev10020390m.g	scaffold_3:4825128-4828999	8.6285	11.4681	0.410444
Ciclev10007136m.g	scaffold_9:23497007-23497580	174.611	232.091	0.410542
Ciclev10017429m.g	scaffold_2:28786443-28788097	70.6655	93.9276	0.410543
Ciclev10030574m.g	scaffold_4:24276199-24281574	12.5041	16.6227	0.410756
Ciclev10014983m.g	scaffold_2:32001592-32005788	23.6012	31.3814	0.411052
Ciclev10029241m.g	scaffold_8:23969949-23971183	19.7885	26.3131	0.411115
Ciclev10005525m.g	scaffold_9:13241445-13244261	306.222	407.274	0.411424
Ciclev10030762m.g	scaffold_4:21457539-21462191	17.901	23.8123	0.411668
Ciclev10031966m.g	scaffold_4:10757188-10759925	1.10309	1.46759	0.411899
Ciclev10023500m.g	scaffold_3:18546745-18550417	1.02872	1.36879	0.412059
Ciclev10010101m.g	scaffold_1:8791913-8799826	2.64216	3.51635	0.41236
Ciclev10030482m.g	scaffold_4:3892954-3907743	10.306	13.7178	0.412567
Ciclev10012551m.g,Ciclev10012767m.g	scaffold_6:18918019-18929260	2.47491	3.29459	0.412724
Ciclev10009277m.g	scaffold_1:289898-291872	14.4257	19.2037	0.412745
Ciclev10022057m.g	scaffold_3:34730822-34732211	0.911097	1.21299	0.412891
Ciclev10019113m.g	scaffold_3:10096639-10102796	2.51122	3.34444	0.413374
Ciclev10008100m.g	scaffold_1:1138081-1142093	21.522	28.6632	0.413392
Ciclev10002145m.g	scaffold_5:32731391-32733586	5.85219	7.79464	0.413505
Ciclev10012154m.g	scaffold_6:3003861-3006390	10.8065	14.3959	0.413752
Ciclev10021891m.g	scaffold_3:42124762-42131106	29.2992	39.0445	0.414257
Ciclev10013787m.g	scaffold_6:25473799-25476738	13.3695	17.8172	0.414322
Ciclev10021287m.g	scaffold_3:7047023-7049430	6.37951	8.50198	0.414353
Ciclev10023969m.g	scaffold_3:24806595-24809323	137.607	183.39	0.41436
Ciclev10015815m.g	scaffold_2:11585698-11587422	15.5086	20.6713	0.414568
Ciclev10028212m.g	scaffold_8:3062344-3066698	14.1269	18.8306	0.414639
Ciclev10017094m.g	scaffold_2:17955749-17956413	2.23468	2.97885	0.414684
Ciclev10020281m.g	scaffold_3:13967222-13970119	21.3033	28.3996	0.414793
Ciclev10029069m.g	scaffold_8:23805635-23807900	2.65721	3.5425	0.414856
Ciclev10004604m.g	scaffold_9:30669875-30675321	12.0908	16.1193	0.414878
Ciclev10010907m.g	scaffold_6:1358970-1368788	4.90465	6.54013	0.415168
Ciclev10032494m.g	scaffold_4:12814562-12816786	241.88	322.539	0.415182
Ciclev10000063m.g	scaffold_5:858466-871605	21.6866	28.9199	0.41526
Ciclev10018750m.g	scaffold_3:20972700-20987159	6.76478	9.02154	0.41533
Ciclev10027606m.g	scaffold_7:4881450-4882242	9.31537	12.4239	0.41543
Ciclev10008848m.g	scaffold_1:8985918-8989356	5.80583	7.74401	0.415577
Ciclev10011130m.g	scaffold_6:16712913-16716984	4.99658	6.66656	0.416002
Ciclev10006987m.g	scaffold_9:2953946-2955257	0.560561	0.747944	0.416059
Ciclev10011419m.g,Ciclev10012321m.g	scaffold_6:16565347-16576434	77.2592	103.088	0.416093
Ciclev10028102m.g	scaffold_8:24914290-24919111	28.9794	38.6725	0.416279
Ciclev10004325m.g	scaffold_9:4470829-4477670	31.0036	41.3772	0.416398
Ciclev10014772m.g	scaffold_2:8064309-8070501	22.3663	29.8518	0.416494
Ciclev10014124m.g	scaffold_2:9308799-9313080	12.6806	16.9248	0.416515
Ciclev10033822m.g	scaffold_4:23822614-23825148	2.26003	3.01668	0.416624

Ciclev10007263m.g	scaffold_1:17806632-17811579	5.868	7.83436	0.416948
Ciclev10007494m.g	scaffold_1:28262201-28267878	21.7976	29.1022	0.416959
Ciclev10019926m.g	scaffold_3:3363103-3366786	5.64006	7.53062	0.417059
-	scaffold_8:2899136-2904471	15.8166	21.1188	0.417088
Ciclev10009430m.g	scaffold_1:23717444-23719869	21.1868	28.2935	0.417305
Ciclev10031088m.g	scaffold_4:16561533-16565410	117.333	156.726	0.417634
Ciclev10026870m.g	scaffold_7:4311386-4312189	187.721	250.786	0.417866
Ciclev10010149m.g	scaffold_1:24913914-24923423	24.7959	33.1309	0.418075
Ciclev10003083m.g	scaffold_5:42941241-42942396	63.6736	85.078	0.41809
Ciclev10025925m.g	scaffold_7:3541923-3543863	35.3387	47.22	0.41815
Ciclev10007368m.g	scaffold_1:27052801-27058991	6.1052	8.15904	0.418362
Ciclev10026299m.g	scaffold_7:4855751-4858195	66.2782	88.5969	0.418722
Ciclev10009802m.g	scaffold_1:28733791-28736188	41.5992	55.6181	0.418999
Ciclev10018660m.g	scaffold_3:45778540-45786532	3.87392	5.17967	0.419068
Ciclev10020216m.g	scaffold_3:6226743-6229790	8.23241	11.0109	0.419541
Ciclev10028115m.g	scaffold_8:19124678-19132335	14.9115	19.9503	0.419985
Ciclev10000140m.g	scaffold_5:40363272-40372619	10.064	13.4653	0.420045
Ciclev10028600m.g	scaffold_8:771383-775656	23.6492	31.6439	0.420135
Ciclev10024908m.g	scaffold_7:6647524-6655916	15.0994	20.2071	0.420373
Ciclev10000608m.g	scaffold_5:24103195-24107361	39.7803	53.2392	0.420436
Ciclev10020061m.g	scaffold_3:7268418-7270557	245.924	329.144	0.420505
Ciclev10016195m.g	scaffold_2:31006299-31010627	13.0381	17.4509	0.420574
Ciclev10033161m.g	scaffold_4:22767878-22774944	5.39355	7.21908	0.42058
Ciclev10025364m.g	scaffold_7:3429961-3433239	11.7353	15.7096	0.420785
Ciclev10004108m.g	scaffold_271:11454-13254	1.07088	1.43384	0.421097
Ciclev10025041m.g	scaffold_7:4410541-4412778	0.699907	0.937336	0.421402
Ciclev10014497m.g	scaffold_2:13250966-13255951	30.2582	40.5247	0.421477
Ciclev10002092m.g	scaffold_5:39423966-39427964	14.8718	19.9189	0.421557
Ciclev10009021m.g	scaffold_1:27940262-27942612	29.0155	38.8806	0.422224
Ciclev10023383m.g	scaffold_3:48107869-48112955	4.36408	5.84857	0.422406
-	scaffold_1:20430559-20431652	14.2186	19.0576	0.422595
Ciclev10030816m.g	scaffold_4:25541395-25544724	3.83332	5.13817	0.422659
Ciclev10027788m.g	scaffold_8:23390336-23397139	12.3412	16.5422	0.42267
Ciclev10016536m.g	scaffold_2:10248431-10251685	59.1117	79.2408	0.422798
Ciclev10015353m.g	scaffold_2:9193531-9198207	1.56757	2.1015	0.422889
Ciclev10023945m.g	scaffold_3:12159667-12162084	5.05658	6.7806	0.423249
Ciclev10028714m.g	scaffold_8:324144-327011	17.4852	23.4467	0.423251
Ciclev10000570m.g	scaffold_5:32951374-32963622	29.7589	39.9058	0.423277
Ciclev10012374m.g	scaffold_6:11926640-11929148	23.5626	31.5971	0.423294
Ciclev10014277m.g	scaffold_2:4028302-4033006	19.054	25.5517	0.423326
Ciclev10023734m.g	scaffold_3:43435941-43439310	9.25449	12.4147	0.42382
Ciclev10020460m.g	scaffold_3:40137240-40144938	6.99992	9.39041	0.423851
Ciclev10005203m.g	scaffold_9:700794-702522	3.97707	5.3353	0.423865
Ciclev10019428m.g	scaffold_3:38488590-38497526	10.4665	14.0413	0.423892
Ciclev10020359m.g	scaffold_3:46079220-46084041	10.8441	14.5488	0.423991
Ciclev10028799m.g	scaffold_8:2125352-2129468	11.1171	14.9164	0.424124
Ciclev10005627m.g	scaffold_9:26809708-26811810	0.697013	0.935264	0.424188
Ciclev10019244m.g	scaffold_3:12706375-12710067	1.29763	1.74147	0.424427
Ciclev10008786m.g	scaffold_1:7767684-7769641	37.1555	49.8668	0.424506
Ciclev10025468m.g	scaffold_7:13226361-13228117	5.10855	6.85669	0.424597
Ciclev10023169m.g	scaffold_3:48389459-48395263	2.29723	3.08338	0.424618
Ciclev10001583m.g	scaffold_5:37060930-37065943	10.8137	14.5143	0.424624
Ciclev10004346m.g	scaffold_9:20791207-20796730	5.22358	7.01148	0.424679
Ciclev10004646m.g	scaffold_9:3903776-3911057	9.91034	13.303	0.424747
Ciclev10025787m.g	scaffold_7:5452931-5458072	22.6918	30.4621	0.424844
Ciclev10030416m.g	scaffold_8:24790199-24792342	92.3689	123.999	0.42485

Ciclev10017021m.g	scaffold_2:27223897-27228305	78.7648	105.751	0.425055
Ciclev10024677m.g	scaffold_7:1179798-1202412	20.7138	27.8146	0.425252
Ciclev10032698m.g	scaffold_4:21411587-21413510	0.969929	1.3025	0.425328
Ciclev10032039m.g	scaffold_4:2645163-2647756	4.42898	5.94767	0.425351
Ciclev10029806m.g	scaffold_8:24001336-24001739	1.03945	1.39636	0.425846
Ciclev10021618m.g	scaffold_3:50349038-50351296	48.9187	65.7239	0.426031
Ciclev10014373m.g	scaffold_2:21915245-21921807	10.1933	13.6971	0.426253
Ciclev10029139m.g	scaffold_8:1073116-1075556	21.4229	28.7891	0.426368
Ciclev10015408m.g	scaffold_2:15434076-15436594	0.352813	0.474177	0.426521
Ciclev10032814m.g	scaffold_4:12592780-12593507	10.6663	14.3356	0.426541
Ciclev10005652m.g	scaffold_9:30766469-30768259	3.85993	5.18948	0.427014
Ciclev10027696m.g	scaffold_8:1287727-1294050	9.49458	12.7684	0.4274
Ciclev10021897m.g	scaffold_3:7337404-7339222	44.4165	59.7392	0.42758
Ciclev10008927m.g	scaffold_1:4123448-4126399	4.09322	5.50528	0.427581
Ciclev10012473m.g	scaffold_6:12325774-12327887	48.2229	64.8657	0.427736
Ciclev10031947m.g	scaffold_4:24956269-24960557	56.3389	75.7875	0.427827
Ciclev10000427m.g	scaffold_5:2472804-2477375	25.4955	34.2983	0.427895
Ciclev10031642m.g	scaffold_4:25277185-25280491	15.9878	21.5107	0.428083
Ciclev10031724m.g	scaffold_4:23552855-23559896	51.6844	69.5424	0.428164
Ciclev10011133m.g	scaffold_6:11524599-11529218	1.63144	2.19524	0.428237
Ciclev10004291m.g,Ciclev10004342m.g	scaffold_9:1507151-1523443	2.79015	3.75511	0.428511
Ciclev10015259m.g	scaffold_2:5191701-5198751	55.1697	74.252	0.428554
Ciclev10012610m.g	scaffold_6:19417560-19419637	6.13325	8.25687	0.428944
Ciclev10028493m.g	scaffold_8:23252245-23256189	16.2948	21.939	0.429088
Ciclev10021970m.g	scaffold_3:6369397-6372018	36.1172	48.6279	0.429101
Ciclev10019037m.g	scaffold_3:3024294-3028262	21.2771	28.65	0.429236
Ciclev10019191m.g	scaffold_3:39356002-39363479	5.70495	7.68292	0.429441
Ciclev10015053m.g	scaffold_2:31036087-31038222	0.256652	0.345739	0.429872
Ciclev10001415m.g	scaffold_5:34289885-34293193	7.91907	10.668	0.429893
Ciclev10002008m.g	scaffold_5:31911886-31914892	22.8603	30.7967	0.429933
Ciclev10033326m.g	scaffold_4:136138-138482	0.340776	0.459096	0.429972
Ciclev10012469m.g	scaffold_6:21567679-21571164	12.1593	16.3838	0.430206
Ciclev10011708m.g	scaffold_6:24801751-24804719	0.976989	1.31643	0.430217
Ciclev10011670m.g	scaffold_6:22024177-22028646	13.3876	18.0419	0.430459
Ciclev10030955m.g	scaffold_4:21681608-21685350	14.0435	18.932	0.430921
Ciclev10030826m.g	scaffold_4:20667184-20674272	8.05977	10.8665	0.431071
Ciclev10004761m.g	scaffold_9:1607070-1611881	11.201	15.1056	0.431452
Ciclev10026250m.g	scaffold_7:7968655-7972006	6.58524	8.8811	0.431503
Ciclev10030787m.g	scaffold_4:194898-198678	9.36495	12.6316	0.431694
Ciclev10031842m.g	scaffold_4:24924267-24927599	40.7939	55.0276	0.431802
Ciclev10026168m.g	scaffold_7:8542857-8544906	26.5667	35.8386	0.431891
Ciclev10031463m.g	scaffold_4:282315-285292	6.22603	8.39962	0.43201
Ciclev10004711m.g	scaffold_9:2303353-2309219	49.5818	66.9	0.432197
Ciclev10033086m.g	scaffold_4:17681333-17682843	42.1648	56.8948	0.432257
Ciclev10012997m.g	scaffold_6:20090843-20095946	132.963	179.438	0.432463
Ciclev10029514m.g	scaffold_8:22454651-22456550	7.12304	9.61284	0.432469
Ciclev10000565m.g	scaffold_5:34395663-34402100	15.9783	21.5638	0.432495
Ciclev10014357m.g	scaffold_2:15121028-15123746	10.6772	14.4108	0.432622
Ciclev10011305m.g	scaffold_6:15214928-15222324	19.9814	26.9699	0.432698
Ciclev10032381m.g	scaffold_4:24154855-24157188	51.0756	68.9441	0.432793
Ciclev10013489m.g	scaffold_6:19296281-19304065	0.818124	1.10451	0.433012
Ciclev10002135m.g	scaffold_5:29706687-29710388	155.699	210.22	0.433136
Ciclev10007507m.g	scaffold_1:25943019-25946820	17.6364	23.812	0.433137
Ciclev10020919m.g	scaffold_3:867234-871452	46.1436	62.3084	0.433296
Ciclev10031158m.g	scaffold_4:17688928-17694225	19.4821	26.3075	0.433325

Ciclev10009534m.g	scaffold_1:8364058-8368382	87.2593	117.834	0.433369
Ciclev10032573m.g	scaffold_4:23712361-23714624	26.9173	36.3495	0.433399
Ciclev10032720m.g,Ciclev10032866m.g	scaffold_4:14358398-14361680	51.7114	69.8375	0.433519
Ciclev10016596m.g	scaffold_2:35778243-35779390	14.2551	19.2553	0.433771
Ciclev10009189m.g	scaffold_1:4382163-4383801	40.322	54.4722	0.433954
Ciclev10019418m.g	scaffold_3:11406395-11410163	27.5897	37.297	0.434928
Ciclev10030851m.g	scaffold_4:6970623-6975096	36.8876	49.8721	0.435097
Ciclev10013826m.g	scaffold_6:6548319-6548986	27.1381	36.692	0.435145
Ciclev10011653m.g	scaffold_6:13550995-13556946	24.1883	32.7081	0.435338
Ciclev10020720m.g	scaffold_3:13024245-13033708	26.2849	35.5519	0.435693
Ciclev10021025m.g	scaffold_3:3082159-3083571	32.3224	43.7201	0.435765
Ciclev10011689m.g	scaffold_6:21602444-21608654	1.2666	1.71336	0.43587
Ciclev10013782m.g	scaffold_6:21082466-21092656	8.69215	11.7597	0.436061
Ciclev10028700m.g	scaffold_8:122439-123867	6.76171	9.14797	0.436062
Ciclev10031207m.g	scaffold_4:14275847-14277827	2.62666	3.55408	0.436245
Ciclev10018483m.g,Ciclev10020262m.g	scaffold_3:8027149-8071028	13.7681	18.6297	0.436269
Ciclev10000721m.g	scaffold_5:38379424-38384975	5.22001	7.06358	0.436346
Ciclev10007810m.g	scaffold_1:7402729-7407601	47.8764	64.7915	0.43649
Ciclev10006145m.g	scaffold_9:27828773-27830893	146.87	198.768	0.436545
Ciclev10020784m.g	scaffold_3:49787296-49790393	7.73022	10.466	0.437133
Ciclev10024922m.g	scaffold_7:305924-314065	11.1024	15.0364	0.437585
Ciclev10021192m.g	scaffold_3:1419085-1422894	51.9644	70.3808	0.43766
Ciclev10014446m.g	scaffold_2:7719549-7723396	0.898228	1.21679	0.437924
Ciclev10031609m.g	scaffold_4:25038166-25043715	169.861	230.107	0.437953
Ciclev10005443m.g	scaffold_9:28284142-28285618	4.65506	6.30614	0.437958
Ciclev10000439m.g	scaffold_5:14967947-14980202	18.3877	24.9101	0.437986
Ciclev10017099m.g	scaffold_2:35138034-35138729	18.5472	25.1263	0.438001
-	scaffold_8:7207025-7207917	1.71832	2.32802	0.438108
-	scaffold_8:3257244-3394853	3.41192	4.62338	0.438363
Ciclev10033640m.g	scaffold_4:19770877-19773664	0.683472	0.926195	0.438435
Ciclev10033200m.g	scaffold_4:17925034-17925722	88.1825	119.557	0.439129
Ciclev10020497m.g	scaffold_3:40066214-40070707	39.6173	53.7183	0.439285
Ciclev10001002m.g	scaffold_5:42974881-42977561	7.77619	10.5444	0.439341
Ciclev10031397m.g	scaffold_4:19416843-19420948	8.01595	10.8695	0.439346
Ciclev10019515m.g,Ciclev10019543m.g	scaffold_3:2426855-2432054	48.8573	66.2524	0.4394
Ciclev10012256m.g	scaffold_6:20456125-20457507	79.3474	107.617	0.439646
Ciclev10031228m.g	scaffold_4:21697923-21702200	8.13303	11.0335	0.440031
Ciclev10029569m.g	scaffold_8:23097532-23099372	103.009	139.749	0.440067
Ciclev10025035m.g	scaffold_7:15284775-15295960	0.621629	0.843346	0.44007
Ciclev10008086m.g	scaffold_1:7776969-7783395	64.3106	87.2506	0.440107
Ciclev10005613m.g	scaffold_9:25047856-25049780	109.539	148.621	0.440198
Ciclev10022517m.g	scaffold_3:42316866-42320830	6.94609	9.42508	0.440303
Ciclev10010948m.g	scaffold_6:16512453-16520446	8.11595	11.0129	0.440365
Ciclev10011244m.g	scaffold_6:17119436-17123022	14.1381	19.1883	0.440636
Ciclev10033634m.g	scaffold_4:24036995-24037820	1.48674	2.0179	0.440696
Ciclev10025597m.g	scaffold_7:6785793-6793006	11.2718	15.2989	0.440708
Ciclev10014300m.g	scaffold_2:369700-374219	30.8734	41.9113	0.440975
Ciclev10022630m.g	scaffold_3:44942858-44944970	9.632	13.0761	0.441021
Ciclev10027779m.g	scaffold_8:24365468-24373136	4.17056	5.66199	0.44107
Ciclev10009906m.g	scaffold_1:21464647-21466099	1.24441	1.68944	0.441079
Ciclev10019140m.g	scaffold_3:1301191-1305787	49.1183	66.6873	0.44115
Ciclev10009240m.g	scaffold_1:28164513-28166098	68.8416	93.4737	0.44128
Ciclev10010896m.g	scaffold_6:25426907-25434850	13.6156	18.4876	0.441289

Ciclev10027844m.g	scaffold_8:5462574-5468717	9.1495	12.4238	0.441342
Ciclev10024946m.g	scaffold_7:12999371-13005156	204.466	277.655	0.441432
Ciclev10025940m.g	scaffold_7:19637028-19643271	34.5631	46.9392	0.441561
Ciclev10016806m.g	scaffold_2:8265616-8267440	36.4032	49.4399	0.441611
Ciclev10010193m.g	scaffold_1:3885965-3893799	11.4788	15.5907	0.441714
Ciclev10017652m.g	scaffold_2:7606429-7606780	0.99479	1.35118	0.441752
Ciclev10018995m.g	scaffold_3:48241315-48261070	16.4245	22.3089	0.441772
Ciclev10004659m.g	scaffold_9:29402156-29406903	29.9533	40.6849	0.441778
Ciclev10002024m.g	scaffold_5:34558361-34564638	56.9685	77.3803	0.441802
Ciclev10027689m.g	scaffold_8:24545358-24553253	4.91897	6.6832	0.442182
Ciclev10033410m.g	scaffold_4:24746663-24749131	5.75675	7.82203	0.442289
Ciclev10030028m.g	scaffold_8:22268752-22279122	35.6363	48.4221	0.442316
Ciclev10018746m.g	scaffold_3:9292304-9301943	5.08612	6.91394	0.442941
Ciclev10005137m.g	scaffold_9:3930180-3934147	41.1733	55.9779	0.443146
Ciclev10030419m.g	scaffold_8:668197-670400	36.8295	50.077	0.443287
Ciclev10030191m.g	scaffold_8:7360347-7361463	1.15625	1.57216	0.443298
Ciclev10026276m.g	scaffold_7:1246078-1248253	18.2236	24.7808	0.443417
Ciclev10015328m.g	scaffold_2:3630628-3632075	0.360561	0.490314	0.443464
Ciclev10019003m.g	scaffold_3:29379180-29385570	31.7591	43.19	0.443527
Ciclev10005625m.g	scaffold_9:2366165-2370407	31.8167	43.271	0.443615
Ciclev10019875m.g	scaffold_3:7240587-7244141	7.20699	9.80158	0.443617
Ciclev10000724m.g	scaffold_5:39438263-39443543	25.9822	35.3392	0.443745
Ciclev10021987m.g	scaffold_3:46386825-46389055	15.5859	21.1989	0.443748
Ciclev10004269m.g	scaffold_9:2480378-2484967	18.4535	25.1011	0.443855
Ciclev10006640m.g	scaffold_9:2029335-2030346	0.652602	0.887816	0.444058
Ciclev10003321m.g	scaffold_5:9502654-9503695	0.427536	0.581648	0.444102
Ciclev10010778m.g	scaffold_1:17837961-17838312	1.96664	2.67593	0.444305
Ciclev10004949m.g	scaffold_9:3125515-3130613	19.6013	26.6733	0.444445
Ciclev10009159m.g	scaffold_1:28571139-28573047	30.6489	41.7097	0.444547
Ciclev10019170m.g	scaffold_3:12865749-12869884	85.0838	115.81	0.444799
Ciclev10032593m.g	scaffold_4:1245264-1248682	4.70158	6.39957	0.444829
Ciclev10027823m.g	scaffold_8:22895733-22900045	9.46998	12.8902	0.444837
Ciclev10005117m.g	scaffold_9:29099671-29106227	9.88228	13.4527	0.444984
-	scaffold_6:1208282-1208999	2.65034	3.60823	0.445115
Ciclev10019459m.g	scaffold_3:48370681-48375261	69.4133	94.5123	0.445289
Ciclev10000955m.g	scaffold_5:33992597-33995513	9.75031	13.2787	0.445597
Ciclev10005190m.g	scaffold_9:29608461-29612474	61.1326	83.2555	0.445603
Ciclev10021771m.g	scaffold_3:2078675-2083111	68.3152	93.0406	0.445655
Ciclev10004669m.g	scaffold_9:30360747-30365013	31.4149	42.7875	0.445739
Ciclev10004136m.g	scaffold_9:3407759-3414934	20.8242	28.3707	0.446141
Ciclev10004482m.g	scaffold_9:2135796-2140355	11.9177	16.2371	0.446192
Ciclev10001785m.g	scaffold_5:21312880-21321683	15.1055	20.583	0.446373
Ciclev10015584m.g	scaffold_2:7400407-7405728	14.5256	19.7934	0.446422
Ciclev10019766m.g	scaffold_3:29541531-29546254	12.8131	17.4612	0.446529
Ciclev10020013m.g	scaffold_3:12216732-12222607	13.7073	18.6814	0.446662
Ciclev10018516m.g	scaffold_3:45636133-45640648	81.3174	110.833	0.446746
Ciclev10020636m.g	scaffold_3:538446-541520	9.63681	13.1357	0.446869
Ciclev10000685m.g,Ciclev10000711m.g	scaffold_5:31411457-31441415	26.1616	35.6607	0.446884
Ciclev10028315m.g	scaffold_8:12953963-12955665	167.796	228.732	0.446953
Ciclev10018853m.g	scaffold_3:48646634-48657995	17.8683	24.3575	0.446965
Ciclev10021784m.g	scaffold_3:1634919-1638281	24.778	33.7792	0.447076
Ciclev10007644m.g	scaffold_1:23346721-23353317	27.2759	37.1901	0.447291
Ciclev10027883m.g	scaffold_8:24493272-24496640	42.6269	58.1276	0.447458
Ciclev10007988m.g	scaffold_1:5381312-5388109	50.8595	69.357	0.447525
Ciclev10003116m.g	scaffold_5:25797130-25800818	10.5975	14.4523	0.44757

Ciclev10020632m.g	scaffold_3:29174601-29178929	8.89624	12.1324	0.447593
Ciclev10012818m.g	scaffold_6:19929535-19930469	11.909	16.2478	0.44819
Ciclev10007749m.g	scaffold_1:1703375-1707207	12.1188	16.537	0.448448
Ciclev10028610m.g	scaffold_8:22724914-22729049	17.9263	24.4637	0.44857
Ciclev10033457m.g	scaffold_4:2069192-2070651	11.8528	16.176	0.448619
Ciclev10008261m.g	scaffold_1:4869088-4870876	0.489533	0.668101	0.448659
Ciclev10007224m.g	scaffold_1:5226581-5243985	8.38552	11.4445	0.448679
Ciclev10033275m.g,Ciclev10033464m.g	scaffold_4:3455972-3458679	53.0638	72.4259	0.448776
Ciclev10011360m.g	scaffold_6:25211221-25215206	3.85723	5.26482	0.44882
-	scaffold_3:32911546-32912001	29.723	40.5709	0.448865
Ciclev10028213m.g	scaffold_8:22767811-22770804	11.0281	15.0533	0.448894
Ciclev10020442m.g	scaffold_3:42240186-42241689	7.39128	10.0893	0.44893
Ciclev10005734m.g	scaffold_9:12365118-12366329	30.8862	42.161	0.448947
Ciclev10005503m.g,Ciclev10005817m.g	scaffold_9:3675171-3684455	24.9556	34.0706	0.449162
-	scaffold_5:2530473-2531215	3.7864	5.16974	0.449264
Ciclev10018338m.g	scaffold_2:35339462-35345225	12.8346	17.5247	0.449349
Ciclev10017512m.g	scaffold_2:26410694-26412331	4.2852	5.85169	0.449491
Ciclev10026659m.g	scaffold_7:14872962-14875220	210.43	287.427	0.449856
-	scaffold_5:47221-49853	11.9033	16.2599	0.449949
Ciclev10024778m.g	scaffold_7:10459681-10463302	6.46718	8.83417	0.449957
Ciclev10008482m.g	scaffold_1:3061548-3065503	64.3542	87.9104	0.45
Ciclev10029102m.g	scaffold_8:15236481-15242013	24.9687	34.1107	0.4501
Ciclev10032226m.g	scaffold_4:25240112-25242812	4.9808	6.80455	0.450124
Ciclev10030931m.g	scaffold_4:20350674-20353893	48.7435	66.5936	0.450172
Ciclev10020133m.g	scaffold_3:43909413-43913344	29.2642	39.9834	0.450267
Ciclev10032446m.g	scaffold_4:21229372-21233185	48.9432	66.8709	0.450271
Ciclev10022589m.g	scaffold_3:9122336-9123264	5.52983	7.55608	0.450402
-	scaffold_5:39554602-39557107	9.50686	12.9914	0.450521
-	scaffold_8:2882615-2883985	4.14075	5.65927	0.450723
Ciclev10008727m.g	scaffold_1:26571815-26575812	15.5665	21.2779	0.450912
Ciclev10012502m.g	scaffold_6:13557051-13559924	167.585	229.072	0.450913
Ciclev10015680m.g	scaffold_2:35349062-35351201	702.712	960.863	0.451397
-	scaffold_4:14602104-14602872	8.96951	12.2654	0.45149
Ciclev10012415m.g	scaffold_6:336968-341181	12.4373	17.0107	0.45177
Ciclev10016592m.g	scaffold_2:8310445-8320631	103.212	141.175	0.45187
Ciclev10000023m.g	scaffold_5:21434576-21450040	11.156	15.2633	0.452241
Ciclev10006928m.g	scaffold_9:24291238-24345939	19.4422	26.6046	0.452489
Ciclev10015837m.g	scaffold_2:35913676-35918119	81.444	111.459	0.452635
Ciclev10011094m.g	scaffold_6:16688098-16692002	1.84854	2.53014	0.452833
Ciclev10012973m.g	scaffold_6:18723003-18725557	113.664	155.582	0.452905
Ciclev10031864m.g	scaffold_4:18315087-18319284	7.6731	10.5047	0.453152
Ciclev10017070m.g	scaffold_2:16603655-16604381	64.6423	88.5003	0.453205
Ciclev10033762m.g	scaffold_4:9262303-9263898	5.83876	7.99493	0.453423
Ciclev10000890m.g	scaffold_5:9999885-10003989	14.3753	19.686	0.453578
Ciclev10012575m.g	scaffold_6:23144465-23148874	7.60035	10.4084	0.453606
Ciclev10002522m.g	scaffold_5:39175823-39179944	0.88944	1.21805	0.453609
Ciclev10021153m.g	scaffold_3:26297948-26304739	23.9065	32.7435	0.453803
Ciclev10009690m.g	scaffold_1:26506044-26506884	25.8154	35.3583	0.453819
Ciclev10027979m.g	scaffold_8:10926217-10937057	5.95519	8.15724	0.453932
Ciclev10024721m.g	scaffold_7:2994408-3001770	9.11902	12.4919	0.454046
Ciclev10014723m.g	scaffold_2:32806564-32812384	3.80046	5.20624	0.45407
Ciclev10030507m.g	scaffold_4:23208705-23218988	20.2	27.6783	0.454401
Ciclev10015224m.g	scaffold_2:361540-366006	55.2067	75.6582	0.454653
Ciclev10024925m.g	scaffold_7:694091-701929	19.182	26.2891	0.454715

Ciclev10008900m.g	scaffold_1:4722888-4726339	15.675	21.487	0.454993
Ciclev10015051m.g	scaffold_2:33485254-33487811	61.1302	83.7959	0.454994
Ciclev10031993m.g	scaffold_4:1523235-1526522	25.0977	34.4045	0.45504
Ciclev10005736m.g	scaffold_9:5132750-5133592	13.7553	18.861	0.455417
Ciclev10007865m.g	scaffold_1:11213418-11215532	20.192	27.692	0.455685
Ciclev10015628m.g	scaffold_2:32499304-32500871	12.9646	17.7809	0.455747
Ciclev10030702m.g	scaffold_4:23873999-23877816	15.7	21.5327	0.455763
Ciclev10029240m.g,Ciclev10029451m.g	scaffold_8:4104240-4107705	27.7998	38.1403	0.456243
Ciclev10025664m.g	scaffold_7:3659310-3662741	21.0287	28.8534	0.456382
Ciclev10021340m.g	scaffold_3:6695170-6697814	47.9345	65.7801	0.456587
Ciclev10005774m.g	scaffold_9:30807744-30811237	121.116	166.225	0.456753
Ciclev10014434m.g	scaffold_2:16492221-16499995	10.0607	13.8083	0.456799
Ciclev10025514m.g	scaffold_7:19633608-19636833	5.84901	8.02795	0.456838
Ciclev10030964m.g	scaffold_4:22148940-22153109	12.3168	16.9102	0.45727
Ciclev10016954m.g	scaffold_2:25200097-25202857	45.8609	62.9683	0.457362
Ciclev10025161m.g	scaffold_7:5963469-5970157	15.4218	21.1753	0.457411
Ciclev10031285m.g	scaffold_4:21704534-21709168	10.9862	15.085	0.457418
Ciclev10021117m.g	scaffold_3:10457160-10461075	38.6422	53.0635	0.457542
Ciclev10022662m.g	scaffold_3:33162548-33166776	43.6664	59.9634	0.457559
Ciclev10023265m.g	scaffold_3:13565612-13567953	7.99146	10.9741	0.457573
Ciclev10015268m.g	scaffold_2:25415518-25418415	18.7368	25.7332	0.457755
Ciclev10031125m.g	scaffold_4:12807095-12810980	9.58565	13.1662	0.457893
Ciclev10030859m.g	scaffold_4:19055040-19057586	1.31052	1.80053	0.458283
Ciclev10001364m.g	scaffold_5:36878965-36882315	13.9447	19.16	0.45838
Ciclev10025201m.g	scaffold_7:4397038-4403337	13.3351	18.3235	0.45847
Ciclev10018428m.g	scaffold_3:40303864-40330808	10.006	13.7499	0.458558
Ciclev10014883m.g	scaffold_2:9973363-9979884	27.4535	37.7271	0.458614
Ciclev10003351m.g	scaffold_5:36400903-36401275	40.6023	55.7974	0.458636
Ciclev10019696m.g	scaffold_3:1881155-1887598	25.8073	35.4715	0.45888
Ciclev10033377m.g	scaffold_4:5378982-5678990	0.451818	0.621038	0.458939
Ciclev10014910m.g	scaffold_2:28910383-28922710	11.1749	15.3604	0.458953
Ciclev10029396m.g	scaffold_8:23103324-23105710	1.26856	1.74381	0.459056
Ciclev10021336m.g	scaffold_3:29395093-29402560	8.56166	11.7693	0.459071
Ciclev10018590m.g	scaffold_3:39725124-39735671	27.3982	37.6643	0.459116
Ciclev10028418m.g	scaffold_8:200402-205401	7.60508	10.4549	0.459139
Ciclev10033984m.g	scaffold_4:23047188-23050416	13.1241	18.0433	0.459241
Ciclev10008971m.g	scaffold_1:24194612-24198030	15.2876	21.0191	0.45934
Ciclev10005907m.g	scaffold_9:3864763-3867933	80.7528	111.036	0.459446
Ciclev10026608m.g	scaffold_7:7147318-7149017	32.9386	45.2921	0.45948
Ciclev10030726m.g	scaffold_4:21916591-21922770	10.4649	14.3898	0.459487
Ciclev10010908m.g	scaffold_6:24286015-24295002	15.3582	21.1234	0.459835
Ciclev10028171m.g	scaffold_8:8716825-8727008	16.7024	22.9759	0.460066
Ciclev10005479m.g	scaffold_9:23161293-23166939	15.1895	20.8984	0.460319
Ciclev10001991m.g	scaffold_5:29863748-29867354	18.4786	25.4253	0.460406
Ciclev10015354m.g	scaffold_2:26435539-26438838	38.4852	52.9819	0.461198
Ciclev10015083m.g	scaffold_2:15759168-15765725	5.78053	7.95933	0.461445
Ciclev10031450m.g	scaffold_4:1377912-1380944	6.73083	9.26947	0.461703
Ciclev10028741m.g	scaffold_8:24632490-24634666	39.7854	54.7913	0.461707
Ciclev10016559m.g	scaffold_2:35629384-35632985	20.8253	28.6805	0.461731
Ciclev10013312m.g	scaffold_6:18807098-18809986	26.0139	35.8301	0.461892
Ciclev10030755m.g	scaffold_4:22841107-22845784	4.98506	6.86625	0.46191
Ciclev10002155m.g	scaffold_5:41169401-41173804	38.5814	53.1481	0.46211
Ciclev10025894m.g	scaffold_7:18984800-18989877	17.0519	23.4929	0.462297
Ciclev10004417m.g	scaffold_9:11270177-11273939	30.5045	42.0288	0.462357
Ciclev10012319m.g	scaffold_6:18912059-18916468	26.7092	36.8027	0.462476

Ciclev10032869m.g	scaffold_4:3953536-3956410	40.5883	55.9348	0.462683
Ciclev10032173m.g	scaffold_4:19223472-19228902	39.8304	54.9014	0.462974
Ciclev10021857m.g	scaffold_3:44725849-44729241	6.29038	8.6709	0.463033
Ciclev10011129m.g	scaffold_6:1195616-1200620	12.4052	17.1022	0.463242
Ciclev10015290m.g	scaffold_2:9100424-9106143	12.0943	16.676	0.463448
Ciclev10019857m.g	scaffold_3:43157575-43161495	4.19624	5.78669	0.463641
Ciclev10026000m.g	scaffold_7:13281116-13311348	23.8476	32.8872	0.463681
Ciclev10021480m.g	scaffold_3:2736406-2739325	4.03473	5.56413	0.463686
Ciclev10005768m.g	scaffold_9:27154798-27158226	19.2348	26.5299	0.463902
Ciclev10002430m.g	scaffold_5:43134799-43135657	54.3724	75.0069	0.464148
Ciclev10011308m.g	scaffold_6:25282911-25285464	7.47983	10.3192	0.464255
Ciclev10031674m.g	scaffold_4:6966682-6970188	8.6791	11.9782	0.464798
Ciclev10007358m.g	scaffold_1:3297426-3301437	4.37618	6.04108	0.465136
Ciclev10030161m.g	scaffold_8:8439385-8443205	20.6127	28.4554	0.46517
Ciclev10031606m.g	scaffold_4:7193292-7197816	29.1283	40.2136	0.465261
Ciclev10018374m.g	scaffold_2:9129024-9130744	46.436	64.109	0.465284
Ciclev10031211m.g	scaffold_4:1468001-1471821	15.8224	21.8443	0.465285
Ciclev10019218m.g	scaffold_3:29267028-29274699	22.3486	30.8573	0.465429
Ciclev10022431m.g	scaffold_3:45689404-45692815	60.1085	82.9962	0.465476
Ciclev10025258m.g	scaffold_7:2887224-2891305	5.22609	7.21622	0.465511
Ciclev10032705m.g	scaffold_4:3545851-3547756	94.2815	130.185	0.465519
Ciclev10024678m.g	scaffold_7:2626580-2648246	4.80337	6.6333	0.465681
Ciclev10033462m.g	scaffold_4:17901445-17909767	15.928	21.9974	0.465762
Ciclev10015082m.g	scaffold_2:5167852-5171721	16.5762	22.8973	0.466067
Ciclev10011145m.g	scaffold_6:23478089-23480584	5.99112	8.27724	0.466324
Ciclev10025440m.g	scaffold_7:19454769-19457603	12.1254	16.757	0.466729
Ciclev10000691m.g	scaffold_5:39356005-39361396	7.54534	10.4275	0.466731
Ciclev10019366m.g	scaffold_3:45262576-45271104	32.9786	45.5782	0.466815
Ciclev10018027m.g	scaffold_2:22475628-22477014	0.7953	1.09916	0.466837
Ciclev10023125m.g	scaffold_3:33711467-33712000	3.86053	5.33645	0.467081
-	scaffold_5:17392923-17476446	5.32366	7.35905	0.467102
Ciclev10014269m.g	scaffold_2:29579700-29584494	0.353425	0.488619	0.467305
Ciclev10028074m.g	scaffold_8:18214033-18265229	15.2192	21.041	0.467314
Ciclev10019204m.g	scaffold_3:5897393-5901654	13.2964	18.3866	0.467618
Ciclev10002035m.g	scaffold_5:420506-424076	39.2624	54.299	0.467777
Ciclev10020301m.g	scaffold_3:15785091-15789066	27.6053	38.1789	0.467829
Ciclev10017793m.g	scaffold_2:8036158-8041911	3.18561	4.40585	0.46785
Ciclev10025508m.g	scaffold_7:5057775-5059577	1.30966	1.8114	0.467912
Ciclev10007416m.g	scaffold_1:6505836-6509558	3.24911	4.49528	0.468366
Ciclev10002634m.g	scaffold_5:33622687-33626661	25.449	35.2115	0.468436
Ciclev10020170m.g	scaffold_3:43776898-43781978	2.8661	3.96587	0.46855
Ciclev10008793m.g	scaffold_1:13398077-13403999	5.52113	7.64108	0.468814
Ciclev10027864m.g	scaffold_8:9821477-9829592	6.46173	8.94307	0.46885
Ciclev10004169m.g	scaffold_9:17516601-17522263	1.28504	1.77871	0.46902
-	scaffold_9:29446109-29446846	10.1609	14.0693	0.469527
Ciclev10016032m.g	scaffold_2:8327745-8334176	4.15649	5.75694	0.469935
Ciclev10015129m.g	scaffold_2:9567678-9570576	48.32	66.9278	0.469986
Ciclev10025250m.g	scaffold_7:1702265-1704963	1.93387	2.67868	0.470036
Ciclev10011642m.g	scaffold_6:21978547-21981020	0.407166	0.564159	0.470485
Ciclev10000620m.g	scaffold_5:41455031-41460155	16.0794	22.281	0.470601
Ciclev10024149m.g	scaffold_3:6065906-6066833	6.21452	8.61353	0.470962
Ciclev10032238m.g	scaffold_4:21443802-21447097	55.082	76.3479	0.471007
Ciclev10014165m.g	scaffold_2:31842326-31849429	27.8559	38.6191	0.471332
Ciclev10013566m.g	scaffold_6:21755851-21764086	22.0856	30.6212	0.471422
Ciclev10021557m.g	scaffold_3:13598139-13599344	17.6637	24.4938	0.471626
Ciclev10004844m.g	scaffold_9:25402634-25405664	25.411	35.2383	0.47169

Ciclev10030531m.g	scaffold_4:21422495-21433083	13.3539	18.5184	0.471698
Ciclev10007429m.g	scaffold_1:28450658-28453946	3.43792	4.76783	0.471799
Ciclev10027940m.g	scaffold_8:23599122-23602023	0.182156	0.252651	0.471972
Ciclev10005857m.g	scaffold_9:29470382-29473396	111.208	154.262	0.472118
Ciclev10029525m.g	scaffold_8:19499144-19501435	4.36511	6.05552	0.472235
Ciclev10021259m.g	scaffold_3:4061385-4063598	24.2262	33.61	0.472322
Ciclev10002848m.g	scaffold_5:33783474-33786446	9.05083	12.558	0.472481
Ciclev10014179m.g	scaffold_2:31309517-31316465	3.51004	4.87026	0.472513
Ciclev10022256m.g	scaffold_3:852922-854417	2.63176	3.65185	0.472599
Ciclev10031488m.g	scaffold_4:22211962-22214809	19.3029	26.7885	0.472796
Ciclev10022619m.g	scaffold_3:44300714-44301197	5.02166	6.96921	0.472829
Ciclev10014026m.g	scaffold_2:9008919-9021876	3.92254	5.44414	0.472914
Ciclev10004847m.g	scaffold_9:5627485-5632577	20.022	27.7893	0.472939
Ciclev10006918m.g	scaffold_9:10624185-10624583	21.9949	30.5328	0.473189
Ciclev10025757m.g	scaffold_7:9325318-9327565	22.8416	31.7092	0.473239
Ciclev10018984m.g	scaffold_3:5699454-5702088	7.09072	9.8441	0.473327
Ciclev10020725m.g	scaffold_3:47945751-47948264	8.35186	11.5955	0.473391
Ciclev10027683m.g,Ciclev10029721m.g	scaffold_8:24116862-24125700	9.74655	13.5333	0.473545
Ciclev10033607m.g	scaffold_4:20090276-20092935	72.3097	100.405	0.473565
Ciclev10026799m.g	scaffold_7:6745362-6746093	4.2175	5.85641	0.473628
Ciclev10029410m.g	scaffold_8:21997239-21998250	76.2776	105.922	0.473673
Ciclev10029166m.g	scaffold_8:9711923-9713705	63.6245	88.3599	0.473809
Ciclev10000093m.g	scaffold_5:37146864-37152552	10.0422	13.947	0.473879
Ciclev10000962m.g	scaffold_5:16114443-16118985	26.2618	36.4754	0.473956
Ciclev10028956m.g	scaffold_8:22792265-22793962	8.69684	12.0799	0.474048
Ciclev10030012m.g	scaffold_8:23672525-23673622	12.3224	17.116	0.47406
Ciclev10001121m.g	scaffold_5:41337992-41344179	19.9886	27.7666	0.474171
Ciclev10011712m.g	scaffold_6:22259168-22262662	2.69852	3.74871	0.474227
Ciclev10028144m.g	scaffold_8:24222171-24224311	37.5439	52.16	0.474367
Ciclev10009293m.g	scaffold_1:23722927-23726214	51.9281	72.1542	0.474567
Ciclev10001142m.g	scaffold_5:38644147-38648215	11.105	15.4312	0.47465
Ciclev10020657m.g	scaffold_3:44552564-44556556	14.1848	19.7114	0.474681
Ciclev10016833m.g	scaffold_2:34605745-34607702	15.9802	22.2085	0.474828
Ciclev10030222m.g	scaffold_8:9720487-9722281	0.891642	1.23922	0.474898
Ciclev10011871m.g	scaffold_6:18217725-18223869	17.0282	23.6682	0.47502
Ciclev10001689m.g	scaffold_5:29661411-29663964	20.0907	27.9298	0.47528
Ciclev10029104m.g	scaffold_8:23960672-23962726	4.82184	6.70326	0.47528
Ciclev10005375m.g	scaffold_9:2119183-2125696	12.9163	17.9598	0.475579
Ciclev10025246m.g	scaffold_7:2269736-2272048	12.021	16.7168	0.475735
Ciclev10005665m.g,Ciclev10006783m.g	scaffold_9:1567920-1599233	16.2372	22.5806	0.475782
Ciclev10031591m.g	scaffold_4:16167354-16171545	0.565671	0.786713	0.475875
Ciclev10005900m.g	scaffold_9:15076063-15079186	130.648	181.712	0.47597
Ciclev10012212m.g	scaffold_6:17651465-17654387	56.1077	78.0383	0.475983
Ciclev10028527m.g	scaffold_8:16606418-16612425	6.48753	9.02498	0.476253
Ciclev10019293m.g	scaffold_3:25663966-25668616	42.8696	59.6382	0.476281
Ciclev10009028m.g	scaffold_1:22987573-22990161	34.6752	48.2428	0.476407
Ciclev10001014m.g	scaffold_5:655534-811195	2.07296	2.88438	0.476567
Ciclev10019152m.g	scaffold_3:4157357-4163728	22.6186	31.4785	0.476857
Ciclev10005475m.g	scaffold_9:20360609-20494992	1.94827	2.71151	0.4769
Ciclev10016171m.g	scaffold_2:34545735-34547703	6.62475	9.22018	0.47693
Ciclev10032197m.g	scaffold_4:2719888-2722624	6.1977	8.62703	0.477132
Ciclev10013705m.g	scaffold_6:23193271-23194498	1.5965	2.22232	0.477149
Ciclev10020203m.g	scaffold_3:50846830-50850793	8.59844	11.971	0.477401
Ciclev10007632m.g	scaffold_1:7359981-7369322	8.1098	11.2936	0.477771

Ciclev10033637m.g	scaffold_4:24210763-24211777	18.7147	26.0641	0.477888
Ciclev10024840m.g	scaffold_7:539112-545474	84.8058	118.123	0.478051
Ciclev10019260m.g	scaffold_3:47328360-47334187	12.0883	16.8378	0.478094
Ciclev10024594m.g	scaffold_3:48806736-48807246	12.3894	17.2572	0.478096
Ciclev10033974m.g	scaffold_4:21827783-21829488	2.69984	3.76068	0.478118
Ciclev10026320m.g	scaffold_7:8483965-8488039	6.0942	8.49086	0.478475
Ciclev10018465m.g	scaffold_3:46415286-46428457	8.33478	11.6129	0.478516
Ciclev10000758m.g	scaffold_5:19019402-19023198	20.1611	28.0914	0.47855
Ciclev10004376m.g	scaffold_9:2608516-2611951	5.24825	7.31367	0.478758
Ciclev10005308m.g	scaffold_9:28392289-28395845	36.585	50.9929	0.479045
Ciclev10020661m.g	scaffold_3:39424396-39436836	15.1417	21.1067	0.479169
Ciclev10001659m.g	scaffold_5:33416340-33422684	18.3618	25.5964	0.479233
Ciclev10003086m.g	scaffold_5:10427531-10584700	0.423069	0.589788	0.479304
Ciclev10007701m.g	scaffold_1:23645332-23649664	8.80617	12.2773	0.479411
Ciclev10028022m.g	scaffold_8:106118-110743	15.6647	21.8411	0.479528
Ciclev10022742m.g	scaffold_3:2822440-2825889	21.9239	30.571	0.479663
Ciclev10020772m.g	scaffold_3:14062896-14066309	20.9611	29.2304	0.479753
Ciclev10007413m.g	scaffold_1:10488729-10494673	3.68394	5.13737	0.479778
Ciclev10026662m.g	scaffold_7:5232892-5233871	1.89312	2.64008	0.479818
Ciclev10007663m.g	scaffold_1:3757243-3763178	69.7753	97.3176	0.479985
Ciclev10020663m.g	scaffold_3:49286522-49291600	39.0734	54.4984	0.480028
Ciclev10000322m.g	scaffold_5:41588808-41591747	1.08605	1.51486	0.480096
Ciclev10028425m.g	scaffold_8:1273733-1276999	7.43539	10.3729	0.480347
Ciclev10021831m.g,Ciclev10021879m.g	scaffold_3:1974522-1986048	31.2249	43.5722	0.480712
Ciclev10030646m.g	scaffold_4:20500279-20506559	13.0123	18.1583	0.480755
Ciclev10028240m.g	scaffold_8:2210641-2213230	5.74191	8.01336	0.480875
Ciclev10001893m.g	scaffold_5:24044657-24050536	9.6746	13.5027	0.48097
Ciclev10028530m.g	scaffold_8:16543588-16549607	7.6454	10.6707	0.480987
Ciclev10028323m.g	scaffold_8:4933329-4937551	20.7732	28.9941	0.481038
Ciclev10023902m.g	scaffold_3:24985137-24987298	2.33414	3.25827	0.481218
Ciclev10027942m.g	scaffold_8:17798577-17807717	3.95163	5.51654	0.481317
Ciclev10014374m.g	scaffold_2:29469300-29477811	17.5113	24.4475	0.481395
Ciclev10001397m.g	scaffold_5:35089436-35093078	9.10349	12.7096	0.481425
Ciclev10014820m.g	scaffold_2:13599064-13602043	28.0255	39.1342	0.481689
Ciclev10004335m.g	scaffold_9:3229234-3237062	6.04244	8.43763	0.481707
Ciclev10023993m.g	scaffold_3:47556314-47559837	9.78468	13.6636	0.48174
Ciclev10004367m.g	scaffold_9:30178753-30183730	3.15466	4.40549	0.481821
Ciclev10006605m.g	scaffold_9:4346193-4349144	17.2232	24.055	0.481982
Ciclev10025312m.g	scaffold_7:18353181-18355609	7.43606	10.3857	0.481983
Ciclev10022477m.g	scaffold_3:30368010-30368924	9.60766	13.4233	0.482485
Ciclev10011304m.g	scaffold_6:17288987-17294039	29.4961	41.2141	0.482615
Ciclev10010990m.g	scaffold_6:22234109-22241211	4.01236	5.60773	0.482967
-	scaffold_7:4747454-4747926	6.48475	9.06538	0.483316
Ciclev10025070m.g,Ciclev10025078m.g	scaffold_7:6897906-6910505	29.615	41.4027	0.483398
Ciclev10014257m.g	scaffold_2:30960207-30962957	1.90689	2.66632	0.483629
Ciclev10033471m.g	scaffold_4:3832941-3835442	397.433	555.714	0.483633
Ciclev10029219m.g	scaffold_8:21133134-21136912	99.7124	139.436	0.483762
Ciclev10031331m.g	scaffold_4:24971611-24985270	3.67657	5.14174	0.483896
Ciclev10016365m.g	scaffold_2:15416347-15418933	25.2625	35.3337	0.484048
Ciclev10000229m.g	scaffold_5:41420210-41424077	1.42617	1.99476	0.484064
-	scaffold_6:7428472-7429280	2.40243	3.36091	0.484354
Ciclev10030830m.g	scaffold_4:21568160-21572693	2.61338	3.65648	0.484538
Ciclev10017805m.g	scaffold_2:6554459-6563699	8.55703	11.9733	0.484634
-	scaffold_5:3980660-4069314	12.6869	17.7522	0.484661

Ciclev10031015m.g	scaffold_4:23075692-23081009	21.7365	30.4178	0.484792
Ciclev10032896m.g	scaffold_4:23754484-23755585	124.213	173.836	0.484903
Ciclev10030776m.g	scaffold_4:9185628-9190318	19.5879	27.4161	0.485058
Ciclev10009281m.g	scaffold_1:24671979-24673930	383.098	536.24	0.485166
Ciclev10022846m.g	scaffold_3:11511552-11513118	50.5485	70.7572	0.485207
Ciclev10000612m.g	scaffold_5:39216417-39219746	14.717	20.6011	0.485233
Ciclev10010408m.g,Ciclev10010792m.g	scaffold_1:25041490-25045884	4.87639	6.82607	0.485243
Ciclev10001679m.g	scaffold_5:40569722-40573126	10.2287	14.3203	0.485428
Ciclev10030469m.g	scaffold_4:18758378-18781489	3.73875	5.23495	0.485619
Ciclev10003472m.g,Ciclev10003720m.g	scaffold_5:35811273-35813538	20.5851	28.8236	0.485651
Ciclev10004226m.g	scaffold_9:15377517-15383677	5.77341	8.08443	0.485722
Ciclev10022357m.g	scaffold_3:45333678-45335663	8.5763	12.0097	0.485776
Ciclev10029475m.g	scaffold_8:23997599-23999331	175.411	245.651	0.485878
Ciclev10021505m.g	scaffold_3:38532929-38534443	28.0219	39.2481	0.48607
Ciclev10014250m.g	scaffold_2:31344536-31347747	1.2539	1.75632	0.486135
Ciclev10002200m.g	scaffold_5:34023980-34026191	10.8235	15.1608	0.486179
Ciclev10018452m.g	scaffold_3:48136987-48153335	42.372	59.3542	0.486237
Ciclev10019100m.g	scaffold_3:9102993-9106855	2.78069	3.89527	0.486281
Ciclev10030764m.g	scaffold_4:14739986-14743864	10.0733	14.1146	0.486663
Ciclev10000133m.g	scaffold_5:14105880-14117664	7.83276	10.9755	0.486696
Ciclev10016322m.g	scaffold_2:6884273-6888069	19.21	26.9178	0.486705
Ciclev10007226m.g	scaffold_1:2583102-2599891	9.27704	12.9995	0.486715
-	scaffold_2:25976814-25978277	22.5203	31.5567	0.486722
Ciclev10004670m.g	scaffold_9:27134275-27139512	29.8667	41.8538	0.486821
Ciclev10012988m.g	scaffold_6:15233991-15234749	27.6113	38.6954	0.486906
Ciclev10004585m.g	scaffold_9:1342154-1346970	7.97693	11.1798	0.486983
Ciclev10018738m.g	scaffold_3:46456835-46462328	46.0906	64.6012	0.487089
Ciclev10023163m.g	scaffold_3:36779624-36780025	1.75616	2.46159	0.487168
Ciclev10028845m.g	scaffold_8:12203091-12205559	12.762	17.8898	0.487288
Ciclev10015546m.g	scaffold_2:35732485-35736448	59.4293	83.3094	0.487306
Ciclev10031093m.g	scaffold_4:11157752-11164902	45.2093	63.3761	0.48732
Ciclev10031859m.g	scaffold_4:17583022-17586426	11.4109	15.9965	0.487347
Ciclev10027701m.g	scaffold_8:7977805-7987270	15.9926	22.4229	0.487569
Ciclev10023685m.g	scaffold_3:48833331-48838933	3.12523	4.38193	0.487607
Ciclev10023955m.g	scaffold_3:47573435-47575182	3.48144	4.88268	0.487987
Ciclev10019400m.g	scaffold_3:42400707-42404790	19.8913	27.9059	0.48843
Ciclev10012426m.g	scaffold_6:16842977-16844008	1.31178	1.84033	0.48844
Ciclev10000263m.g	scaffold_5:33010919-33015057	6.11372	8.57715	0.488448
Ciclev10004406m.g	scaffold_9:1933926-1940744	49.1307	68.9367	0.488646
Ciclev10013058m.g	scaffold_6:20123786-20124852	44.2329	62.0651	0.488662
Ciclev10013587m.g	scaffold_6:25056111-25060813	2.0709	2.90609	0.488824
Ciclev10010684m.g	scaffold_1:20519518-20525115	8.64305	12.1324	0.489253
Ciclev10001599m.g	scaffold_5:34474998-34478522	14.1164	19.8163	0.489309
Ciclev10029043m.g	scaffold_8:20483473-20486898	33.1627	46.5538	0.489337
Ciclev10009655m.g	scaffold_1:570695-575294	70.5802	99.0812	0.489349
Ciclev10027737m.g	scaffold_8:20579277-20594708	18.0244	25.3058	0.489519
Ciclev10009216m.g	scaffold_1:6463485-6467814	12.0817	16.963	0.489564
Ciclev10011637m.g	scaffold_6:21499534-21504662	42.7954	60.0892	0.489651
Ciclev10029169m.g	scaffold_8:21374018-21379609	68.5496	96.2557	0.489724
Ciclev10008319m.g	scaffold_1:20664826-20823973	0.569412	0.799658	0.489911
Ciclev10005095m.g	scaffold_9:29596218-29597957	1.67457	2.3517	0.489914
Ciclev10020646m.g	scaffold_3:10232203-10235364	7.10093	9.97232	0.48992
Ciclev10014777m.g	scaffold_2:423767-426163	39.4352	55.3823	0.489941
Ciclev10012955m.g	scaffold_6:15192651-15194091	24.478	34.3774	0.489972

Ciclev10017148m.g	scaffold_2:34764293-34766525	14.6192	20.5323	0.490024
Ciclev10007980m.g	scaffold_1:22138396-22141621	2.14065	3.00654	0.490055
Ciclev10031644m.g	scaffold_4:20387505-20392078	12.7378	17.8907	0.490093
Ciclev10017238m.g	scaffold_2:15425226-15429206	9.24652	12.9887	0.490269
Ciclev10023277m.g	scaffold_3:11687448-12154880	1.69421	2.38002	0.490364
Ciclev10018215m.g	scaffold_2:36187959-36193470	34.1648	47.9974	0.490445
Ciclev10000698m.g	scaffold_5:30994811-30999834	17.3721	24.4069	0.49051
Ciclev10001453m.g	scaffold_5:5103989-5106917	115.684	162.533	0.490552
Ciclev10013049m.g	scaffold_6:15940241-15990100	247.541	347.806	0.490613
Ciclev10001559m.g	scaffold_5:41496791-41499173	1291.44	1814.61	0.490683
Ciclev10010292m.g	scaffold_1:23370938-23372640	43.572	61.2259	0.490739
Ciclev10007478m.g	scaffold_1:18958677-18968622	10.9214	15.347	0.490802
-	scaffold_1:20830459-20830824	22.124	31.0904	0.490862
Ciclev10026648m.g	scaffold_7:2012391-2014677	5.19529	7.30087	0.490864
Ciclev10001330m.g	scaffold_5:43282463-43287999	38.3555	53.9017	0.490896
Ciclev10029663m.g	scaffold_8:5029938-5030960	99.7918	140.27	0.491213
Ciclev10000877m.g	scaffold_5:4939532-4944344	7.91933	11.1344	0.491573
Ciclev10018548m.g	scaffold_3:43260726-43272903	6.23131	8.76188	0.491705
Ciclev10002276m.g	scaffold_5:38253834-38255589	43.9752	61.8439	0.491941
Ciclev10001219m.g	scaffold_5:371943-377068	8.15418	11.4689	0.492112
Ciclev10003653m.g	scaffold_5:27488060-27490869	3.87592	5.45204	0.492259
Ciclev10032134m.g	scaffold_4:15515959-15519963	14.9052	20.9696	0.492478
Ciclev10020143m.g	scaffold_3:42589824-42593481	93.165	131.077	0.492553
Ciclev10030407m.g	scaffold_8:23629245-23630961	18.8339	26.5043	0.492893
Ciclev10019071m.g	scaffold_3:4203406-4210711	21.8325	30.7338	0.493346
Ciclev10004473m.g,Ciclev10006554m.g	scaffold_9:3539542-3568051	9.62726	13.5527	0.493386
Ciclev10028354m.g	scaffold_8:3016413-3021094	15.9421	22.4455	0.49358
Ciclev10025197m.g	scaffold_7:19113404-19115749	14.1409	19.9111	0.493692
-	scaffold_2:34555183-34555943	8.78813	12.3753	0.493836
Ciclev10010507m.g	scaffold_1:18788221-18793080	35.9949	50.6878	0.493845
Ciclev10014683m.g	scaffold_2:35386137-35388993	2.2821	3.21393	0.493975
Ciclev10033803m.g	scaffold_4:17669389-17671500	80.5535	113.45	0.494033
Ciclev10031267m.g	scaffold_4:24027715-24031840	48.2594	67.9676	0.494036
Ciclev10012025m.g	scaffold_6:12912030-12915662	19.5568	27.5514	0.494455
Ciclev10028440m.g	scaffold_8:170344-173638	46.3837	65.3461	0.494483
Ciclev10007229m.g	scaffold_1:16576329-16586621	4.37505	6.16372	0.4945
Ciclev10031576m.g	scaffold_4:1138453-1143573	98.8486	139.301	0.494915
Ciclev10015627m.g	scaffold_2:31407924-31413611	59.05	83.2282	0.495135
Ciclev10029019m.g	scaffold_8:23043644-23045073	5.58967	7.87894	0.49524
Ciclev10014523m.g	scaffold_2:23307373-23313381	36.8424	51.9328	0.49528
Ciclev10021040m.g	scaffold_3:43944604-43945981	1.96861	2.77516	0.495395
Ciclev10017873m.g	scaffold_2:30512452-30515315	40.7726	57.48	0.495461
Ciclev10018677m.g	scaffold_3:10816569-10825312	5.24872	7.39954	0.49547
Ciclev10000538m.g	scaffold_5:34128462-34131986	1.51858	2.14093	0.495519
Ciclev10007206m.g	scaffold_9:1436101-1436628	10.2569	14.4606	0.495532
Ciclev10023615m.g	scaffold_3:49533317-49539442	49.4718	69.7505	0.495598
Ciclev10008172m.g	scaffold_1:8869060-8870835	55.3111	77.9869	0.495664
Ciclev10028344m.g	scaffold_8:1963035-1965358	0.692578	0.976518	0.495671
Ciclev10032837m.g	scaffold_4:23496019-23497466	20.1501	28.417	0.495968
Ciclev10011290m.g	scaffold_6:21464334-21477001	37.4314	52.7884	0.495973
Ciclev10025111m.g	scaffold_7:4377395-4381088	0.275996	0.389242	0.496021
Ciclev10010180m.g	scaffold_1:25594036-25598980	12.0247	16.9595	0.496086
Ciclev10013867m.g	scaffold_6:19322431-19344804	5.6674	7.99383	0.496201
Ciclev10013641m.g,Ciclev10013709m.g	scaffold_6:19668846-19675370	30.5238	43.0559	0.496279

Ciclev10013425m.g	scaffold_6:20788768-20790160	0.444089	0.626498	0.496462
Ciclev10009382m.g	scaffold_1:28553020-28554904	3.33315	4.70234	0.496493
Ciclev10014503m.g	scaffold_2:10268803-10282136	12.6987	17.9172	0.496663
Ciclev10024538m.g	scaffold_3:29723802-29727037	0.729573	1.02959	0.496947
Ciclev10000254m.g	scaffold_5:24381726-24384719	4.22279	5.95986	0.497083
Ciclev10015923m.g	scaffold_2:4351559-4358516	83.8328	118.351	0.497491
Ciclev10018861m.g	scaffold_3:46956022-46960642	12.673	17.8918	0.497544
Ciclev10009206m.g	scaffold_1:27177017-27180318	1.8043	2.54742	0.497596
Ciclev10020171m.g	scaffold_3:41238802-41241110	23.7648	33.5573	0.4978
Ciclev10017756m.g	scaffold_2:23051626-23057279	5.16538	7.29407	0.49785
Ciclev10029198m.g	scaffold_8:2817340-2822179	21.092	29.7846	0.497869
Ciclev10010213m.g	scaffold_1:149282-156258	13.7306	19.3899	0.497911
Ciclev10007794m.g	scaffold_1:28744870-28749453	32.5788	46.014	0.498141
Ciclev10032633m.g	scaffold_4:14769042-14770977	38.0723	53.7731	0.498145
Ciclev10019165m.g	scaffold_3:24744596-24750079	22.6991	32.0638	0.498313
Ciclev10012024m.g	scaffold_6:13594246-13595936	2.743	3.87532	0.498562
Ciclev10000934m.g	scaffold_5:34345967-34375442	1.39674	1.97344	0.498649
Ciclev10000428m.g	scaffold_5:37871001-37875272	7.48053	10.5696	0.498705
Ciclev10018859m.g	scaffold_3:10022296-10028450	45.9972	64.9919	0.498714
Ciclev10024950m.g	scaffold_7:8888124-8892704	4.01072	5.66718	0.498772
Ciclev10007447m.g	scaffold_1:2827326-2834096	11.0416	15.6042	0.498991
Ciclev10019446m.g	scaffold_3:34616339-34618467	5.7531	8.13156	0.499193
Ciclev10002739m.g	scaffold_5:21675612-21680155	1.01994	1.44164	0.499231
Ciclev10018716m.g	scaffold_3:48988754-48996232	6.69604	9.46491	0.499281
Ciclev10008037m.g	scaffold_1:28737316-28740295	25.0737	35.4426	0.499311
Ciclev10024681m.g	scaffold_7:3140603-3150592	8.863	12.5293	0.49944
Ciclev10016360m.g	scaffold_2:840270-841760	20.263	28.6452	0.499444
-	scaffold_8:16224047-16224332	42.9093	60.6637	0.499545
-	scaffold_3:20343765-20344578	2.7798	3.93081	0.499841
Ciclev10025107m.g	scaffold_7:19334465-19388718	5.06225	7.15968	0.500117
Ciclev10015417m.g	scaffold_2:573823-592599	9.46268	13.3845	0.500243
Ciclev10021754m.g	scaffold_3:49832547-49840403	6.94251	9.81999	0.500265
Ciclev10004909m.g	scaffold_9:17388035-17389599	31.9979	45.2619	0.500322
Ciclev10007423m.g	scaffold_1:27463642-27471312	12.9799	18.3614	0.500394
Ciclev10032109m.g,Ciclev10033769m.g	scaffold_4:14516982-14524650	6.9648	9.85488	0.500756
Ciclev10020747m.g	scaffold_3:4959825-4964659	170.889	241.803	0.500773
Ciclev10031389m.g	scaffold_4:24686693-24688788	1.59465	2.2568	0.501036
Ciclev10023133m.g	scaffold_3:49096567-49098225	30.3933	43.0156	0.501106
Ciclev10012249m.g	scaffold_6:8588269-8596361	22.6768	32.0965	0.501199
Ciclev10011370m.g	scaffold_6:20164450-20166395	5.07807	7.18843	0.501397
Ciclev10001167m.g	scaffold_5:33252069-33255967	3.42723	4.8517	0.501448
Ciclev10019588m.g	scaffold_3:41473798-41479421	37.1691	52.6226	0.501581
Ciclev10001602m.g	scaffold_5:34641430-34642548	1.59681	2.26088	0.501692
Ciclev10004294m.g	scaffold_9:23124106-23135069	14.4079	20.4026	0.501897
Ciclev10017291m.g	scaffold_2:7426102-7428721	3.33632	4.72471	0.501968
Ciclev10011356m.g	scaffold_6:23419104-23424251	51.2632	72.599	0.502024
Ciclev10016284m.g	scaffold_2:28817234-28818264	2.7857	3.94514	0.502037
Ciclev10017517m.g	scaffold_2:9652506-9655307	1.90219	2.69396	0.502071
Ciclev10029961m.g	scaffold_8:24004644-24005037	1.9213	2.72136	0.502247
Ciclev10020914m.g	scaffold_3:25448857-25455844	8.45598	11.9786	0.502413
Ciclev10011037m.g	scaffold_6:17407692-17413059	15.3909	21.8083	0.502801
Ciclev10008736m.g	scaffold_1:413414-417191	28.0871	39.7994	0.50284
Ciclev10022033m.g	scaffold_3:6495250-6496559	3.17744	4.50288	0.502986
Ciclev10019214m.g	scaffold_3:8731959-8740245	53.5982	75.961	0.503074
Ciclev10000334m.g	scaffold_5:37030668-37035492	12.6981	17.9999	0.503383

Ciclev10013784m.g	scaffold_6:3267839-3271244	2.27276	3.22246	0.503721
Ciclev10032430m.g	scaffold_4:24892837-24894612	9.78699	13.8771	0.503772
Ciclev10018808m.g	scaffold_3:1718192-1725123	96.6293	137.016	0.503809
Ciclev10011434m.g	scaffold_6:25101236-25104868	111.07	157.515	0.504013
Ciclev10028849m.g	scaffold_8:2697792-2701440	29.706	42.129	0.504059
Ciclev10004718m.g	scaffold_9:7521997-7530730	28.5612	40.5078	0.504144
Ciclev10019866m.g	scaffold_3:50741488-50744604	13.8006	19.5753	0.504301
Ciclev10013781m.g	scaffold_6:25306782-25315243	31.2972	44.3936	0.504318
Ciclev10027174m.g	scaffold_7:9452025-9462028	26.7529	37.9592	0.504757
Ciclev10019965m.g	scaffold_3:733105-736119	21.3078	30.2355	0.504861
Ciclev10028449m.g	scaffold_8:18683424-18684891	3.79644	5.38743	0.504949
Ciclev10026926m.g	scaffold_7:5829878-5830118	5.13699	7.28983	0.504961
Ciclev10007384m.g	scaffold_1:18119891-18126891	5.57911	7.91743	0.504997
-	scaffold_5:4716631-4717173	3.98126	5.65024	0.505085
-	scaffold_2:10133694-10134991	3.28247	4.65935	0.505347
Ciclev10001702m.g	scaffold_5:29664370-29670123	2.85765	4.0568	0.505511
Ciclev10001355m.g	scaffold_5:39754437-39762057	3.6453	5.17538	0.505626
Ciclev10030496m.g	scaffold_4:24662243-24670352	18.0037	25.5631	0.505769
Ciclev10032587m.g	scaffold_4:21748159-21749437	32.8471	46.6397	0.505792
Ciclev10029073m.g	scaffold_8:24471992-24474778	24.7538	35.1481	0.505794
Ciclev10013800m.g	scaffold_6:1994873-1997225	2.6037	3.69833	0.506315
Ciclev10009845m.g	scaffold_1:510072-510595	20.2049	28.7015	0.506421
Ciclev10007851m.g	scaffold_1:25196337-25202267	92.4985	131.406	0.506535
Ciclev10009188m.g	scaffold_1:24693905-24699886	10.2141	14.512	0.506682
Ciclev10020128m.g	scaffold_3:41443077-41447377	48.7008	69.1986	0.506796
Ciclev10014454m.g	scaffold_2:31489810-31497594	6.95969	9.89045	0.507014
Ciclev10023001m.g	scaffold_3:42384646-42385527	5.88514	8.36354	0.507036
Ciclev10008593m.g	scaffold_1:2925041-2932100	5.94827	8.45352	0.507081
Ciclev10026670m.g	scaffold_7:9292944-9294594	29.0834	41.333	0.507099
Ciclev10024399m.g	scaffold_3:7175715-7181525	35.0867	49.8704	0.507258
Ciclev10008372m.g	scaffold_1:6999844-7007241	32.9357	46.8168	0.507374
Ciclev10013233m.g	scaffold_6:25009565-25010598	21.8796	31.1051	0.507564
Ciclev10022008m.g	scaffold_3:12733128-12741687	11.8477	16.8443	0.50766
Ciclev10020107m.g	scaffold_3:47081716-47085017	22.1497	31.4935	0.507765
Ciclev10003767m.g	scaffold_5:15168046-15173092	58.8966	83.7482	0.507874
Ciclev10008222m.g	scaffold_1:15860187-15866603	13.6865	19.4626	0.507953
Ciclev10012994m.g	scaffold_6:20226863-20228959	5.4482	7.74771	0.507992
Ciclev10019759m.g	scaffold_3:45103011-45106320	12.4105	17.6506	0.508159
Ciclev10010841m.g	scaffold_1:25611760-25618071	20.691	29.4305	0.508307
Ciclev10001214m.g	scaffold_5:40383849-40388296	55.0667	78.3466	0.50869
Ciclev10012317m.g	scaffold_6:23481342-23483968	15.5685	22.1569	0.509123
Ciclev10028459m.g	scaffold_8:23365480-23369843	12.532	17.8366	0.50922
Ciclev10007586m.g	scaffold_1:23610858-23615349	4.2202	6.00746	0.509444
Ciclev10000134m.g	scaffold_5:34607063-34612877	22.7943	32.4517	0.509624
Ciclev10017847m.g	scaffold_2:12335864-12339694	23.676	33.7086	0.509695
Ciclev10012028m.g,Ciclev10013244m.g	scaffold_6:7909208-7914618	95.7653	136.354	0.509787
Ciclev10012216m.g	scaffold_6:21132426-21135018	64.0814	91.2416	0.509788
Ciclev10007364m.g	scaffold_1:503902-509879	26.4767	37.7	0.509841
Ciclev10004181m.g	scaffold_9:30962854-30970212	20.001	28.4827	0.510013
Ciclev10030076m.g	scaffold_8:17291958-17467945	16.902	24.0717	0.510146
Ciclev10014949m.g	scaffold_2:31483098-31487421	4.87987	6.95055	0.510284
Ciclev10001354m.g	scaffold_5:17881810-17890203	16.5432	23.5675	0.510558
Ciclev10027999m.g	scaffold_8:2831831-2837109	180.724	257.487	0.510708
Ciclev10000202m.g	scaffold_5:32773920-32779114	1.57004	2.23745	0.511057
Ciclev10027851m.g	scaffold_8:2301581-2307975	19.7378	28.1284	0.511068

Ciclev10004400m.g	scaffold_9:147883-152474	22.6532	32.2973	0.511696
Ciclev10013235m.g	scaffold_6:20376032-20377928	58.3495	83.1923	0.511731
Ciclev10016010m.g	scaffold_2:21664545-21665739	4.78913	6.82911	0.511933
Ciclev10032285m.g	scaffold_4:24341641-24346954	14.1479	20.1763	0.512073
Ciclev10000026m.g	scaffold_5:64758-82198	14.9369	21.3071	0.512453
Ciclev10001539m.g	scaffold_5:41093724-41097411	22.9354	32.7176	0.512494
Ciclev10010950m.g	scaffold_6:16871273-16876833	25.0327	35.7116	0.512582
Ciclev10005170m.g	scaffold_9:14725832-14730595	46.9018	66.9186	0.512764
Ciclev10030943m.g	scaffold_4:1109751-1113554	8.3178	11.8687	0.512894
Ciclev10019809m.g	scaffold_3:8378716-8380605	24.2876	34.6628	0.513167
Ciclev10031840m.g	scaffold_4:24553792-24557560	8.06519	11.5105	0.513173
Ciclev10030223m.g	scaffold_8:2660906-2662287	26.0251	37.1427	0.513177
Ciclev10017121m.g	scaffold_2:15135293-15135955	0.273857	0.390859	0.513224
Ciclev10027782m.g	scaffold_8:3923756-3931507	3.40571	4.86092	0.513274
Ciclev10024871m.g	scaffold_7:6941414-6947640	25.2574	36.0553	0.513505
Ciclev10022810m.g	scaffold_3:43192761-43194352	159.801	228.14	0.513647
Ciclev10011516m.g	scaffold_6:23722796-23725939	4.9139	7.01591	0.513761
Ciclev10007282m.g,Ciclev10008272m.g	scaffold_1:21476759-21547573	22.2209	31.7279	0.513834
Ciclev10001505m.g	scaffold_5:36540903-36547227	29.9574	42.7796	0.514011
Ciclev10000378m.g	scaffold_5:30828997-30836351	86.8348	124.045	0.514518
Ciclev10009863m.g	scaffold_1:20153437-20155846	65.2401	93.1985	0.514548
Ciclev10009269m.g	scaffold_1:16593874-16597598	6.25178	8.93146	0.514629
Ciclev10024656m.g	scaffold_3:46197339-46201959	33.3395	47.6304	0.514651
Ciclev10005105m.g	scaffold_9:5622857-5627193	14.7291	21.0433	0.514695
Ciclev10009061m.g	scaffold_1:17831332-17834889	24.9137	35.5998	0.514926
Ciclev10005247m.g	scaffold_9:15084106-15093683	3.48783	4.98412	0.515009
Ciclev10016835m.g	scaffold_2:30425174-30425976	0.392275	0.560609	0.515127
Ciclev10007753m.g	scaffold_1:28373758-28378683	51.6997	73.9059	0.515534
Ciclev10032003m.g	scaffold_4:22335856-22339919	137.595	196.72	0.51571
-	scaffold_5:2531968-2532515	9.53083	13.6266	0.515757
Ciclev10001621m.g	scaffold_5:34294852-34297359	34.4851	49.305	0.515761
Ciclev10028970m.g	scaffold_8:2392762-2395692	32.6911	46.7405	0.515777
Ciclev10002540m.g	scaffold_5:34672741-34674507	69.8202	99.8382	0.515947
Ciclev10011420m.g	scaffold_6:6329420-6335005	27.2682	38.9936	0.516015
Ciclev10002515m.g	scaffold_5:37600816-37604471	125.65	179.682	0.516039
Ciclev10028528m.g	scaffold_8:21626654-21631114	2.13389	3.05211	0.516318
Ciclev10007239m.g	scaffold_1:25180494-25191344	1.75795	2.51474	0.516513
Ciclev10005086m.g	scaffold_9:26365333-26367212	3.39151	4.85155	0.516515
Ciclev10018638m.g	scaffold_3:47898201-47903486	4.20804	6.02106	0.516867
Ciclev10033783m.g	scaffold_4:16817583-16984703	12.5301	17.9294	0.516926
Ciclev10020434m.g	scaffold_3:48584454-48588112	8.32218	11.9088	0.516999
Ciclev10019242m.g,Ciclev10024655m.g	scaffold_3:41827327-41842502	21.4976	30.7653	0.517131
Ciclev10011987m.g	scaffold_6:18953631-18957475	7.8841	11.283	0.517131
Ciclev10024794m.g	scaffold_7:12352172-12754529	1.10723	1.58478	0.517319
Ciclev10029763m.g	scaffold_8:9311237-9313454	1.72859	2.47432	0.517432
Ciclev10020229m.g	scaffold_3:41814893-41817767	15.8992	22.7621	0.517681
Ciclev10015070m.g	scaffold_2:254918-257378	12.4343	17.8028	0.517776
Ciclev10014618m.g	scaffold_2:28403936-28488203	2.11753	3.0322	0.517981
Ciclev10022144m.g	scaffold_3:35827349-35831778	7.54296	10.8017	0.518056
Ciclev10016133m.g	scaffold_2:25208922-25211881	52.5267	75.22	0.518065
Ciclev10011615m.g	scaffold_6:22546698-22548549	6.27278	8.98296	0.518087
Ciclev10022681m.g	scaffold_3:38551517-38552105	2.1635	3.09872	0.518304
Ciclev10018984m.g	scaffold_3:5699454-5702088	4.06859	5.82766	0.51839
Ciclev10001942m.g	scaffold_5:30537417-30543922	56.0765	80.3251	0.518453

Ciclev10019349m.g	scaffold_3:255780-258629	0.364651	0.522357	0.518519
Ciclev10027709m.g	scaffold_8:17819970-17836722	21.1641	30.3178	0.518544
Ciclev10028565m.g	scaffold_8:1107273-1111272	29.7442	42.6127	0.518677
Ciclev10000656m.g	scaffold_5:36848456-36853497	32.0612	45.9325	0.518687
-	scaffold_3:21910897-21911160	11.81	16.9197	0.518693
Ciclev10007604m.g	scaffold_1:26414606-26419009	3.18701	4.56609	0.518756
Ciclev10027501m.g	scaffold_7:10200471-10203603	0.839371	1.20292	0.519164
Ciclev10031878m.g	scaffold_4:18827761-18829983	13.1909	18.9044	0.519173
Ciclev10008035m.g	scaffold_1:25384528-25386604	5.56776	7.97999	0.519289
Ciclev10004581m.g	scaffold_9:11576287-11715575	20.7636	29.7617	0.519397
Ciclev10023157m.g	scaffold_3:45575453-45576142	24.0625	34.4969	0.519677
Ciclev10019534m.g	scaffold_3:7511644-7516994	22.5361	32.3144	0.519939
Ciclev10033015m.g	scaffold_4:1069256-1070891	42.276	60.6278	0.520143
Ciclev10011174m.g	scaffold_6:3894836-3901326	5.62287	8.06466	0.520308
Ciclev10018493m.g	scaffold_3:8309802-8317120	7.60785	10.9117	0.520311
Ciclev10008519m.g	scaffold_1:20896084-20910413	1.76387	2.53078	0.520834
Ciclev10009160m.g	scaffold_1:24883108-24885343	2.0455	2.93506	0.520936
Ciclev10010995m.g	scaffold_6:24146485-24154259	10.7431	15.4164	0.52105
Ciclev10004368m.g	scaffold_9:26339677-26348342	3.12966	4.49137	0.521149
Ciclev10028815m.g	scaffold_8:2204481-2208812	2.22809	3.19771	0.52123
Ciclev10015064m.g	scaffold_2:33837402-33842156	15.3763	22.0681	0.521254
Ciclev10004626m.g	scaffold_9:29874797-29879735	10.0817	14.4718	0.521516
Ciclev10030120m.g	scaffold_8:249390-263099	24.9142	35.7782	0.522114
Ciclev10024991m.g	scaffold_7:18940987-18947131	21.5995	31.0193	0.522168
Ciclev10005981m.g	scaffold_9:30087568-30090145	37.6678	54.0991	0.52227
Ciclev10008623m.g	scaffold_1:28830110-28833028	25.224	36.2301	0.522391
Ciclev10010824m.g	scaffold_1:16587639-16591052	2.57368	3.6967	0.522404
Ciclev10018709m.g	scaffold_3:48041555-48045776	8.99279	12.9174	0.522478
Ciclev10010897m.g	scaffold_6:11597186-11605542	16.374	23.5273	0.522931
Ciclev10006140m.g	scaffold_9:3671927-3674756	50.6727	72.8167	0.523061
Ciclev10015209m.g	scaffold_2:15409061-15415424	14.6503	21.0551	0.523237
Ciclev10010438m.g	scaffold_1:1967431-1970087	1.24525	1.78993	0.523463
Ciclev10027831m.g	scaffold_8:24271068-24276213	124.225	178.567	0.523505
Ciclev10019005m.g	scaffold_3:17722316-17728192	20.5955	29.6051	0.523522
Ciclev10014171m.g	scaffold_2:35556511-35562852	23.7084	34.0868	0.523814
Ciclev10023603m.g	scaffold_3:37066920-37069031	2.21018	3.17789	0.523907
Ciclev10006181m.g	scaffold_9:2488650-2490119	23.5506	33.8675	0.524137
Ciclev10015838m.g	scaffold_2:7612563-7617582	16.3009	23.4436	0.524241
Ciclev10028248m.g,Ciclev10028465m.g	scaffold_8:12967156-13120570	36.8944	53.0653	0.524366
Ciclev10015766m.g	scaffold_2:14294645-14298692	36.3515	52.2875	0.524451
Ciclev10023688m.g	scaffold_3:35372249-35712253	0.424998	0.611334	0.524504
Ciclev10014402m.g	scaffold_2:546097-552884	17.2126	24.7597	0.524531
Ciclev10016541m.g	scaffold_2:5191701-5198751	5.45167	7.84281	0.524673
Ciclev10002483m.g	scaffold_5:30797712-30800237	13.6217	19.5966	0.524693
Ciclev10031019m.g	scaffold_4:1183765-1187593	0.597437	0.859707	0.525057
Ciclev10030848m.g	scaffold_4:23398028-23402343	2.28391	3.28684	0.525192
Ciclev10028382m.g	scaffold_8:1424550-1429767	25.8339	37.1804	0.525281
Ciclev10032830m.g	scaffold_4:17944544-17945441	31.3732	45.1556	0.525376
Ciclev10025043m.g	scaffold_7:2610854-2615635	17.8212	25.6514	0.525444
Ciclev10008932m.g	scaffold_1:4541675-4543982	7.43377	10.701	0.525574
Ciclev10028160m.g	scaffold_8:20740038-20748864	11.3938	16.4021	0.525624
Ciclev10022366m.g	scaffold_3:3863699-3864880	69.0542	99.4433	0.526146
Ciclev10032299m.g	scaffold_4:23993127-23994257	20.6351	29.7164	0.526162
Ciclev10027775m.g	scaffold_8:4970589-4975356	2.21412	3.19027	0.526945
Ciclev10018203m.g	scaffold_2:8110613-8113890	19.6333	28.2896	0.526967

Ciclev10028109m.g	scaffold_8:22423340-22427299	8.26164	11.9053	0.5271
Ciclev10024112m.g	scaffold_3:46657920-46662192	1.57194	2.26532	0.527173
Ciclev10030909m.g	scaffold_4:22253188-22257152	11.5245	16.608	0.527179
Ciclev10001458m.g	scaffold_5:27012428-27017627	12.0971	17.4343	0.527266
Ciclev10014399m.g	scaffold_2:26297741-26303205	5.69258	8.20437	0.52731
Ciclev10025063m.g	scaffold_7:6531088-6535245	29.6639	42.7604	0.527567
Ciclev10029167m.g	scaffold_8:21106529-21107653	23.1469	33.3707	0.527761
Ciclev10017127m.g	scaffold_2:9697884-9698467	1.08377	1.56248	0.527778
-	scaffold_7:17480712-17481106	18.5977	26.8165	0.527998
-	scaffold_5:4071604-4071817	23.6024	34.0367	0.528159
Ciclev10005858m.g,Ciclev10006767m.g	scaffold_9:29506228-29514162	15.7973	22.7816	0.528191
Ciclev10019275m.g	scaffold_3:4372275-4374913	22.2139	32.038	0.528323
Ciclev10002154m.g	scaffold_5:40487889-40490483	3.67868	5.30562	0.528334
Ciclev10007576m.g	scaffold_1:28394517-28402379	29.6798	42.8067	0.528353
Ciclev10014973m.g	scaffold_2:35088624-35090531	1.49442	2.1559	0.528705
Ciclev10004643m.g	scaffold_9:28076062-28077772	0.945699	1.36448	0.528894
Ciclev10011258m.g	scaffold_6:25451951-25454726	4.53077	6.53774	0.529036
Ciclev10007887m.g	scaffold_1:26090515-26095622	10.5718	15.2577	0.529314
Ciclev10007603m.g	scaffold_1:4099684-4105561	29.4678	42.5334	0.529456
Ciclev10031499m.g	scaffold_4:9249664-9257760	6.46753	9.33599	0.529589
Ciclev10011989m.g	scaffold_6:24681355-24684487	12.4027	17.9099	0.530102
Ciclev10025092m.g	scaffold_7:929055-931550	1.91504	2.76628	0.530574
Ciclev10007453m.g	scaffold_1:19341935-19345148	1.21302	1.75224	0.530597
Ciclev10005070m.g	scaffold_9:2891879-2895934	25.4501	36.7667	0.530731
Ciclev10007579m.g	scaffold_1:18766405-18774720	11.9451	17.2569	0.530746
Ciclev10019101m.g,Ciclev10024265m.g	scaffold_3:7815776-7834934	17.9231	25.8991	0.531084
Ciclev10025205m.g	scaffold_7:4429588-4436363	3.5853	5.18093	0.531118
Ciclev10008029m.g	scaffold_1:11512414-11514707	10.657	15.4	0.531125
Ciclev10029009m.g	scaffold_8:4182383-4188979	10.1591	14.6811	0.531191
Ciclev10015479m.g	scaffold_2:32084546-32086152	11.4035	16.4833	0.531528
Ciclev10008034m.g	scaffold_1:14715306-14725997	63.6585	92.016	0.53153
Ciclev10013166m.g	scaffold_6:15537913-15538693	12.6523	18.2917	0.531786
Ciclev10016474m.g	scaffold_2:31456225-31458564	13.4537	19.453	0.531989
Ciclev10021576m.g	scaffold_3:17516413-17517777	2.01118	2.90823	0.532102
Ciclev10020397m.g	scaffold_3:2062739-2065492	13.1639	19.0356	0.532112
-	scaffold_1:20636018-20636271	17.2089	24.8877	0.532284
Ciclev10020488m.g	scaffold_3:12857546-12861331	21.7997	31.5274	0.532296
Ciclev10022917m.g	scaffold_3:50004213-50006768	20.3482	29.4317	0.53247
Ciclev10030488m.g	scaffold_4:1365732-1377467	45.1776	65.3456	0.532482
Ciclev10011233m.g	scaffold_6:24621238-24623632	10.0579	14.5481	0.532512
Ciclev10027591m.g	scaffold_7:20067040-20069864	11.9732	17.3192	0.532566
Ciclev10008944m.g	scaffold_1:13319160-13324341	19.97	28.8885	0.532661
Ciclev10024877m.g	scaffold_7:4843156-4848614	0.25523	0.369284	0.532933
Ciclev10020325m.g	scaffold_3:33705980-33709294	7.17773	10.3877	0.533276
Ciclev10004575m.g	scaffold_9:30676433-30683281	6.2624	9.06312	0.533291
Ciclev10000567m.g	scaffold_5:39146389-39150157	21.2435	30.7442	0.533292
Ciclev10022397m.g	scaffold_3:7311581-7314699	25.2112	36.4876	0.53334
Ciclev10010985m.g	scaffold_6:20848858-20853471	6.48086	9.38023	0.533438
Ciclev10011745m.g	scaffold_6:13446197-13450339	40.5248	58.6606	0.533589
Ciclev10023787m.g	scaffold_3:44213281-44214883	4.25041	6.15363	0.533836
-	scaffold_5:27484008-27484976	22.6081	32.7376	0.53411
Ciclev10005245m.g	scaffold_9:20227698-20230676	15.5845	22.5687	0.534209
Ciclev10018527m.g	scaffold_3:822590-829873	5.80847	8.4145	0.53472
Ciclev10032141m.g	scaffold_4:993891-996211	283.156	410.425	0.535522

Ciclev10022807m.g	scaffold_3:44413419-44415467	19.0348	27.5933	0.535678
Ciclev10030782m.g	scaffold_4:3356823-3359250	1.38259	2.00443	0.535813
Ciclev10032230m.g	scaffold_4:16295893-16298529	3.81335	5.52846	0.535819
Ciclev10022828m.g	scaffold_3:47844689-47846726	116.923	169.542	0.536079
Ciclev10005827m.g	scaffold_9:6370052-6372554	131.97	191.395	0.536336
Ciclev10026655m.g	scaffold_7:3197342-3198152	67.2115	97.4787	0.53638
Ciclev10026377m.g	scaffold_7:7888700-7892344	15.4418	22.3972	0.536476
Ciclev10001140m.g	scaffold_5:41945345-41948270	18.2576	26.4834	0.536589
Ciclev10020844m.g	scaffold_3:30687562-30690083	16.6267	24.1187	0.53665
Ciclev10022206m.g	scaffold_3:1010201-1012853	46.5333	67.5013	0.536652
Ciclev10019548m.g	scaffold_3:42516279-42519750	1.15081	1.66969	0.536929
Ciclev10000344m.g	scaffold_5:811792-817028	11.3317	16.4432	0.537131
Ciclev10026677m.g	scaffold_7:5812233-5814503	83.7215	121.488	0.537145
Ciclev10002655m.g	scaffold_5:32634453-32639205	8.78933	12.7551	0.537254
Ciclev10019130m.g	scaffold_3:1593961-1598283	79.5905	115.532	0.537628
Ciclev10007989m.g	scaffold_1:2918530-2922318	21.5409	31.2717	0.537781
Ciclev10014371m.g	scaffold_2:26159412-26162206	3.54192	5.14305	0.538092
Ciclev10029652m.g	scaffold_8:5039713-5041719	26.7515	38.8447	0.538095
Ciclev10007242m.g	scaffold_1:7882127-7893687	10.448	15.1732	0.538305
Ciclev10013046m.g	scaffold_6:15236223-15237145	21.667	31.467	0.53834
Ciclev10015419m.g	scaffold_2:8657806-8662884	15.2251	22.1123	0.538392
Ciclev10017366m.g	scaffold_2:32550546-32554289	13.8105	20.0591	0.538493
-	scaffold_5:2529749-2530017	11.0024	15.9818	0.538611
Ciclev10016031m.g	scaffold_2:31840456-31841624	31.9573	46.4233	0.538701
Ciclev10014667m.g	scaffold_2:31022883-31034411	11.504	16.7163	0.539123
Ciclev10020800m.g	scaffold_3:18680714-18685507	5.93264	8.6228	0.539482
Ciclev10033261m.g,Ciclev10033936m.g	scaffold_4:17215952-17218131	4.66561	6.78157	0.539553
Ciclev10004208m.g	scaffold_9:30640258-30649286	19.8874	28.9069	0.539557
Ciclev10024335m.g	scaffold_3:40740184-40742289	3.72194	5.41061	0.539735
Ciclev10031984m.g	scaffold_4:16184225-16186489	5.3543	7.78454	0.539914
Ciclev10014878m.g	scaffold_2:31761010-31769690	19.5545	28.4354	0.540186
Ciclev10005459m.g	scaffold_9:5896850-5899769	1.02634	1.49253	0.540254
Ciclev10033008m.g	scaffold_4:9127505-9132191	1.10949	1.6136	0.540395
Ciclev10008344m.g	scaffold_1:4240318-4243112	19.4805	28.3354	0.540577
Ciclev10027702m.g	scaffold_8:22377174-22400703	5.38669	7.83609	0.540736
Ciclev10000084m.g	scaffold_5:33791193-33800877	43.9965	64.0074	0.540852
-	scaffold_6:16102726-16102898	1822.26	2651.39	0.541022
Ciclev10012530m.g	scaffold_6:13244322-13247813	13.1293	19.1076	0.541352
Ciclev10033079m.g	scaffold_4:10499108-10499486	0.826392	1.20284	0.54155
Ciclev10009000m.g	scaffold_1:2821921-2826609	24.988	36.372	0.541593
Ciclev10026113m.g	scaffold_7:7739781-7742295	124.228	180.84	0.541721
Ciclev10020020m.g	scaffold_3:42105750-42109077	30.0964	43.8198	0.541988
Ciclev10011237m.g	scaffold_6:6694889-6751336	3.86919	5.63519	0.542433
Ciclev10018326m.g	scaffold_2:27721453-27722682	110.121	160.416	0.542726
Ciclev10007169m.g	scaffold_9:5227254-5233938	17.0933	24.9023	0.542845
Ciclev10032288m.g	scaffold_4:25079433-25080528	8.52568	12.4233	0.543158
Ciclev10012293m.g	scaffold_6:18836295-18838412	0.552345	0.804886	0.543215
Ciclev10007157m.g	scaffold_9:28305830-28333082	6.33725	9.23499	0.543253
Ciclev10014259m.g,Ciclev10015258m.g,Ciclev10018293m.g	scaffold_2:32555946-32577574	20.6213	30.0513	0.543291
Ciclev10031571m.g	scaffold_4:12804962-12807029	2.82582	4.11815	0.543327
Ciclev10009885m.g	scaffold_1:3719881-3722131	0.431179	0.628492	0.543607
Ciclev10021318m.g	scaffold_3:46501636-46505635	134.863	196.666	0.544254
Ciclev10001816m.g	scaffold_5:42353393-42356612	18.2855	26.6657	0.544287

Ciclev10026643m.g	scaffold_7:11107040-11109191	1.33333	1.94452	0.544379
-	scaffold_5:8745667-8989247	28.3926	41.4154	0.544652
Ciclev10028571m.g	scaffold_8:19776421-19778967	3.30141	4.81608	0.544775
Ciclev10004843m.g	scaffold_9:27896568-27900862	11.309	16.4978	0.544794
Ciclev10014606m.g	scaffold_2:27042648-27053881	5.0917	7.42785	0.544797
Ciclev10011854m.g	scaffold_6:25557796-25561675	24.2678	35.4078	0.54502
Ciclev10030654m.g	scaffold_4:16699044-16703735	13.0782	19.0838	0.545189
Ciclev10022175m.g,Ciclev10024016m.g	scaffold_3:33388539-33592383	27.0351	39.4503	0.545204
Ciclev10001035m.g	scaffold_5:37232472-37239661	8.70969	12.7111	0.5454
Ciclev10024686m.g	scaffold_7:517022-526314	14.2393	20.7815	0.545415
Ciclev10033939m.g	scaffold_4:2086884-2106219	17.4361	25.4542	0.545827
Ciclev10029007m.g	scaffold_8:478150-479719	31.9361	46.6233	0.545864
Ciclev10026747m.g	scaffold_7:9255649-9256153	39.7543	58.0381	0.545888
Ciclev10007192m.g	scaffold_9:13532068-13535642	3.06888	4.48041	0.545918
Ciclev10023947m.g	scaffold_3:3494283-3495948	0.772928	1.12845	0.545933
Ciclev10014624m.g	scaffold_2:33962414-33970658	13.2355	19.3242	0.545993
Ciclev10031689m.g	scaffold_4:6338297-6339713	3.16524	4.62141	0.546018
Ciclev10019373m.g	scaffold_3:8163217-8166534	10.6952	15.6158	0.546049
-	scaffold_6:16120036-16120250	298.944	436.511	0.546142
Ciclev10008763m.g	scaffold_1:23876790-23880665	12.2903	17.9464	0.546171
Ciclev10031618m.g	scaffold_4:19231922-19240440	3.59362	5.24829	0.546407
Ciclev10028640m.g	scaffold_8:1333548-1336613	8.69985	12.7057	0.546418
Ciclev10031100m.g	scaffold_4:17417354-17469137	1.18332	1.72824	0.546463
Ciclev10014469m.g	scaffold_2:36062171-36064699	61.2927	89.5181	0.546466
-	scaffold_8:3257244-3394853	30.9327	45.183	0.546647
Ciclev10028385m.g	scaffold_8:20535215-20539344	4.06068	5.93165	0.546712
Ciclev10001138m.g	scaffold_5:38906625-38908821	0.650524	0.95032	0.546811
Ciclev10018991m.g	scaffold_3:49827654-49832343	548.263	800.944	0.546834
Ciclev10011079m.g	scaffold_6:13220219-13227650	3.01776	4.40867	0.546863
Ciclev10010581m.g	scaffold_1:12327314-12332302	13.7505	20.0935	0.547244
Ciclev10020166m.g	scaffold_3:10913452-10924755	25.8898	37.8353	0.547348
Ciclev10012430m.g	scaffold_6:19232413-19236606	9.93858	14.5256	0.547485
Ciclev10024915m.g,Ciclev10026566m.g	scaffold_7:5724001-5737654	13.0142	19.0207	0.547491
Ciclev10015651m.g	scaffold_2:34835180-34838228	7.82794	11.4425	0.547703
Ciclev10004044m.g	scaffold_5:39000261-39010398	150.378	219.843	0.547884
Ciclev10018820m.g	scaffold_3:21491781-21499455	11.7546	17.1853	0.547948
Ciclev10020156m.g	scaffold_3:44858332-44862278	9.55951	13.9777	0.548116
Ciclev10008415m.g	scaffold_1:26690274-26692825	13.5385	19.797	0.548219
Ciclev10015441m.g	scaffold_2:35372728-35377822	4.58359	6.70295	0.548319
Ciclev10006435m.g	scaffold_9:30072480-30086933	30.0942	44.0154	0.54852
Ciclev10025979m.g	scaffold_7:5760451-5762350	17.1733	25.1185	0.54858
Ciclev10024519m.g	scaffold_3:22245049-22247068	3.41602	4.99679	0.548683
Ciclev10012552m.g	scaffold_6:23364302-23366061	37.9139	55.461	0.548745
Ciclev10009151m.g	scaffold_1:16467941-16471056	15.2194	22.265	0.548867
Ciclev10007857m.g,Ciclev10010188m.g,Ciclev10010641m.g	scaffold_1:3006499-3044316	11.4912	16.8156	0.54927
Ciclev10007643m.g	scaffold_1:521631-524099	3.89291	5.69683	0.54931
Ciclev10006161m.g	scaffold_9:1128045-1129318	29.1837	42.7138	0.549537
Ciclev10024796m.g	scaffold_7:4625708-4629899	5.43732	7.95843	0.549589
Ciclev10025944m.g	scaffold_7:15233160-15236047	223.898	327.723	0.549636
-	scaffold_1:3723301-3724050	2.49109	3.6469	0.549894
Ciclev10027965m.g	scaffold_8:1388215-1394789	19.727	28.8829	0.550041
Ciclev10013131m.g	scaffold_6:24860406-24861964	30.6145	44.8236	0.550044

Ciclev10028842m.g	scaffold_8:1376122-1378446	12.4414	18.2163	0.55008
-	scaffold_9:642978-644935	29.4037	43.0531	0.550117
-	scaffold_5:38071965-38073011	51.5753	75.5231	0.550238
Ciclev10022724m.g	scaffold_3:44932207-44933739	14.0125	20.5229	0.550518
Ciclev10033194m.g	scaffold_4:17981682-17982664	16.66	24.4041	0.550738
Ciclev10015229m.g	scaffold_2:22305296-22307562	46.5154	68.1387	0.550767
Ciclev10025231m.g	scaffold_7:13022420-13028396	20.1259	29.4847	0.550912
Ciclev10022233m.g	scaffold_3:47723098-47727226	43.3849	63.5616	0.550962
Ciclev10025766m.g	scaffold_7:9942705-9947272	11.9309	17.4806	0.551052
Ciclev10018977m.g	scaffold_3:2058310-2061637	2.74414	4.02094	0.551177
Ciclev10007595m.g	scaffold_1:2200404-2205620	11.7123	17.1633	0.551304
Ciclev10010780m.g	scaffold_1:25260252-25278175	9.64846	14.1402	0.551434
Ciclev10031173m.g	scaffold_4:23365406-23370600	19.5911	28.714	0.551557
Ciclev10002972m.g	scaffold_5:15039380-15040080	0.619198	0.907544	0.551566
Ciclev10027858m.g	scaffold_8:16466826-16472372	132.323	193.968	0.551754
Ciclev10018460m.g	scaffold_3:50751429-50760409	20.3246	29.7967	0.551926
Ciclev10016371m.g	scaffold_2:479436-484203	28.9904	42.5032	0.551999
Ciclev10000307m.g	scaffold_5:20081413-20086750	4.1994	6.1572	0.552092
Ciclev10016868m.g	scaffold_2:12549542-12550712	4.19586	6.15215	0.552123
Ciclev10030342m.g	scaffold_8:5000218-5001954	13.9693	20.4829	0.552163
Ciclev10011355m.g	scaffold_6:17528443-17532729	12.8251	18.8072	0.552318
Ciclev10029281m.g	scaffold_8:2227826-2230752	90.5332	132.782	0.552536
Ciclev10007807m.g,Ciclev10008679m.g	scaffold_1:18973977-19006253	4.44908	6.5258	0.552646
Ciclev10025775m.g	scaffold_7:10123449-10128449	14.2709	20.9351	0.552851
Ciclev10028470m.g	scaffold_8:16301152-16305410	19.4792	28.5794	0.55304
Ciclev10018459m.g	scaffold_3:44254777-44263274	12.9641	19.0254	0.553404
Ciclev10007312m.g	scaffold_1:1634244-1639600	4.54011	6.6637	0.553598
Ciclev10016994m.g	scaffold_2:32504790-32505737	4.80129	7.04708	0.553605
Ciclev10023445m.g	scaffold_3:25248366-25288105	15.871	23.3003	0.553953
Ciclev10014591m.g	scaffold_2:31594076-31599418	12.6721	18.6053	0.554062
Ciclev10016116m.g	scaffold_2:11973700-11976647	68.0361	99.9136	0.554379
Ciclev10024982m.g	scaffold_7:1533263-1536326	0.423355	0.621789	0.554559
Ciclev10011203m.g	scaffold_6:8200407-8209632	9.01193	13.2392	0.554905
Ciclev10011600m.g	scaffold_6:25160850-25166165	30.3166	44.5397	0.554984
Ciclev10007225m.g	scaffold_1:2682763-2707083	4.93427	7.24952	0.55505
Ciclev10030605m.g	scaffold_4:2874346-2879934	13.7732	20.2384	0.555232
Ciclev10033001m.g	scaffold_4:15291691-15292830	184.417	270.991	0.555271
Ciclev10012547m.g	scaffold_6:19926856-19929426	12.4039	18.2282	0.555383
Ciclev10011017m.g	scaffold_6:18338000-18345627	22.4727	33.0259	0.555427
Ciclev10031426m.g	scaffold_4:6198364-6202941	20.6121	30.3001	0.555829
Ciclev10029894m.g	scaffold_8:4479380-4481411	0.584923	0.859856	0.555848
Ciclev10024408m.g,Ciclev10024621m.g	scaffold_3:22043475-22177017	1.33361	1.96062	0.555973
Ciclev10029527m.g	scaffold_8:24353773-24354969	12.872	18.9266	0.556171
Ciclev10014816m.g	scaffold_2:9457191-9463218	15.4582	22.7338	0.556463
Ciclev10002254m.g	scaffold_5:35776945-35780088	54.8534	80.6709	0.556469
Ciclev10002603m.g	scaffold_5:34293367-34294163	0.332187	0.488608	0.55668
Ciclev10014237m.g	scaffold_2:7459183-7475509	11.0197	16.2091	0.556721
Ciclev10013112m.g	scaffold_6:5282482-5285250	21.6457	31.8427	0.556884
Ciclev10011357m.g	scaffold_6:23828643-23833709	5.64298	8.30155	0.556924
Ciclev10004423m.g	scaffold_9:25305958-25310181	43.1972	63.5491	0.556935
Ciclev10028284m.g	scaffold_8:5675865-5680482	23.2306	34.1757	0.556943
Ciclev10031861m.g	scaffold_4:19207459-19212886	7.1197	10.4772	0.557358
Ciclev10007834m.g	scaffold_1:28416014-28420995	54.6023	80.354	0.557408
Ciclev10016869m.g	scaffold_2:29260560-29269901	17.5866	25.8823	0.557487

Ciclev10007656m.g	scaffold_1:2934247-2940997	19.5888	28.8301	0.557545
Ciclev10026241m.g	scaffold_7:13006094-13011505	10.5067	15.4653	0.55772
Ciclev10007594m.g	scaffold_1:27704654-27709844	16.4481	24.2161	0.558044
Ciclev10024679m.g	scaffold_7:18310177-18334143	5.5691	8.20031	0.558234
Ciclev10027721m.g	scaffold_8:18642992-18650693	0.339339	0.499683	0.558286
Ciclev10021112m.g	scaffold_3:24741493-24743967	2.67227	3.93521	0.558378
Ciclev10014598m.g	scaffold_2:4864393-4884787	30.7273	45.2505	0.558411
Ciclev10006965m.g	scaffold_9:8453099-8457191	5.31947	7.83536	0.558717
Ciclev10011294m.g	scaffold_6:22683416-22687371	6.17645	9.10066	0.559193
Ciclev10018972m.g	scaffold_3:35354851-35358765	11.5743	17.0606	0.559742
Ciclev10017208m.g	scaffold_2:13044722-13048774	7.53045	11.103	0.560144
Ciclev10000409m.g	scaffold_5:42030907-42037428	19.3279	28.4992	0.560235
Ciclev10029290m.g	scaffold_8:3597904-3599596	0.81475	1.20141	0.560305
Ciclev10022651m.g	scaffold_3:43902919-43904813	38.7842	57.1946	0.560408
Ciclev10014170m.g	scaffold_2:30523899-30548559	7.65683	11.2979	0.561237
Ciclev10007326m.g	scaffold_1:6427074-6437114	4.30694	6.35716	0.561721
Ciclev10014957m.g	scaffold_2:13677327-13685473	17.9106	26.4401	0.561912
Ciclev10011344m.g	scaffold_6:25201576-25206677	14.0139	20.6879	0.561928
Ciclev10010922m.g	scaffold_6:23437986-23446056	1.92371	2.84054	0.562272
Ciclev10021228m.g	scaffold_3:45924472-45929249	6.27357	9.26353	0.562275
Ciclev10019647m.g	scaffold_3:1935326-1939594	17.5588	25.9355	0.562733
Ciclev10000209m.g	scaffold_5:12908400-12916815	31.4488	46.4539	0.562791
Ciclev10003020m.g	scaffold_5:13055388-13070403	0.386526	0.571009	0.562948
Ciclev10033209m.g	scaffold_4:10565450-10571345	0.662158	0.978215	0.562976
Ciclev10022440m.g	scaffold_3:48278577-48281322	73.5766	108.713	0.5632
Ciclev10001126m.g	scaffold_5:40081126-40086638	5.55799	8.21248	0.563255
Ciclev10033525m.g,Ciclev10033738m.g	scaffold_4:18515567-18675155	5.73688	8.48002	0.5638
Ciclev10032601m.g	scaffold_4:12996913-13001185	15.6565	23.1498	0.564239
Ciclev10012986m.g	scaffold_6:22458502-22460533	5.87705	8.6903	0.564314
Ciclev10016485m.g	scaffold_2:10552065-10556272	20.8694	30.8684	0.564742
Ciclev10026258m.g	scaffold_7:19950882-20044587	6.05518	8.95644	0.564756
Ciclev10030820m.g	scaffold_4:6860908-6866001	6.13391	9.07414	0.564954
Ciclev10025149m.g	scaffold_7:2069617-2075388	52.4519	77.5966	0.564998
Ciclev10009124m.g	scaffold_1:2723767-2725314	8.98334	13.292	0.565231
Ciclev10020048m.g	scaffold_3:3502908-3506768	13.5377	20.0351	0.565543
Ciclev10014182m.g	scaffold_2:23877363-23881540	3.16883	4.69029	0.565728
Ciclev10023386m.g	scaffold_3:49223553-49225128	152.378	225.546	0.565768
Ciclev10022060m.g	scaffold_3:48075723-48077996	7.05264	10.4421	0.566172
Ciclev10018495m.g	scaffold_3:8700207-8708402	2.7325	4.0459	0.566239
Ciclev10006411m.g	scaffold_9:3608942-3609912	16.7373	24.792	0.566805
Ciclev10017091m.g	scaffold_2:22916123-22917685	5.8457	8.65944	0.566897
Ciclev10005967m.g	scaffold_9:30374696-30381795	2.75173	4.07644	0.56697
Ciclev10033106m.g	scaffold_4:3761490-3766717	2.45967	3.64412	0.567103
Ciclev10011331m.g	scaffold_6:17832157-17836764	13.8079	20.4589	0.567236
Ciclev10026560m.g	scaffold_7:7172538-7174424	7.16411	10.6167	0.567471
Ciclev10005581m.g	scaffold_9:26280371-26283822	17.6646	26.1931	0.568331
Ciclev10026549m.g	scaffold_7:1309883-1311067	175.273	259.922	0.568475
Ciclev10031727m.g	scaffold_4:10887836-10892584	1.70085	2.52255	0.568629
Ciclev10011317m.g	scaffold_6:16709178-16712039	23.2556	34.4925	0.568703
Ciclev10003149m.g	scaffold_5:40900195-40909351	9.84287	14.5992	0.568737
Ciclev10024236m.g	scaffold_3:2258896-2263291	4.4353	6.57869	0.568768
Ciclev10025727m.g	scaffold_7:3232908-3235616	22.4801	33.3464	0.568883
Ciclev10027833m.g	scaffold_8:13615515-13622142	17.899	26.5513	0.568907
Ciclev10004706m.g	scaffold_9:10227751-10231262	10.4243	15.4665	0.569193
Ciclev10009024m.g	scaffold_1:23015966-23018242	24.6055	36.5102	0.569321

-	scaffold_8:8068239-8071033	1.24901	1.85371	0.569639
-	scaffold_7:2516197-2516390	43.0582	63.9207	0.569995
Ciclev10000639m.g	scaffold_5:885889-889099	1.82154	2.70483	0.570378
Ciclev10029545m.g	scaffold_8:2269769-2272113	69.1831	102.747	0.570603
Ciclev10030514m.g	scaffold_4:25395934-25407396	23.1743	34.4203	0.570731
Ciclev10024815m.g	scaffold_7:8656240-8661349	4.0815	6.06223	0.570748
Ciclev10028150m.g	scaffold_8:19266677-19272144	15.34	22.787	0.570913
Ciclev10003031m.g	scaffold_5:36766088-36766771	7.4048	11.0001	0.570985
Ciclev10002265m.g	scaffold_5:39150564-39152662	4.669	6.93637	0.571068
Ciclev10012066m.g	scaffold_6:17837515-17839197	36.6592	54.4626	0.571089
Ciclev10014133m.g	scaffold_2:30940302-30947067	9.21267	13.6882	0.571238
Ciclev10003687m.g	scaffold_5:27541640-27543165	7.67498	11.4036	0.571251
Ciclev10009399m.g	scaffold_1:7764323-7767138	7.91744	11.7653	0.571431
Ciclev10020409m.g	scaffold_3:27726234-27797748	14.2721	21.2083	0.571433
Ciclev10024507m.g	scaffold_3:22240999-22244465	0.762639	1.13367	0.571932
Ciclev10002223m.g	scaffold_5:38882563-38886957	17.9123	26.6369	0.572478
Ciclev10001491m.g	scaffold_5:5317018-5321025	0.755106	1.1234	0.573122
Ciclev10030617m.g	scaffold_4:6834480-6851598	10.035	14.9306	0.573223
Ciclev10000433m.g,Ciclev10003537m.g,Ciclev10003758m.g	scaffold_5:4944981-4979217	257.774	383.534	0.573245
Ciclev10030525m.g	scaffold_4:3656494-3661987	6.6535	9.89983	0.57329
Ciclev10027776m.g	scaffold_8:2443425-2451856	8.13311	12.1027	0.57345
Ciclev10014210m.g	scaffold_2:10381866-10390501	39.5702	58.902	0.573903
Ciclev10024688m.g	scaffold_7:5439777-5446884	5.82678	8.67345	0.573906
Ciclev10029263m.g	scaffold_8:23106539-23107913	321.241	478.249	0.574108
Ciclev10027900m.g	scaffold_8:5703232-5710003	14.7396	21.9445	0.574164
Ciclev10028083m.g	scaffold_8:1421392-1424145	7.13533	10.6246	0.574355
Ciclev10011924m.g	scaffold_6:16239057-16243537	17.8935	26.6452	0.574438
Ciclev10007569m.g	scaffold_1:22954824-22961222	12.835	19.1185	0.574879
Ciclev10031224m.g	scaffold_4:8117068-8120942	5.9734	8.89844	0.575
Ciclev10004332m.g	scaffold_9:24224374-24236554	13.7536	20.4904	0.57514
Ciclev10023806m.g	scaffold_3:47352606-47354979	3.55439	5.29581	0.575251
Ciclev10011761m.g	scaffold_6:11876945-11878540	3.10073	4.62	0.575281
Ciclev10000078m.g	scaffold_5:20865254-20886617	12.5867	18.7622	0.575923
Ciclev10029234m.g	scaffold_8:23371733-23373676	2.41543	3.601	0.576115
Ciclev10024782m.g	scaffold_7:11057544-11061889	3.52205	5.25168	0.576361
Ciclev10010111m.g	scaffold_1:238855-247876	0.757836	1.13013	0.576528
Ciclev10014571m.g	scaffold_2:5200296-5205642	139.525	208.125	0.576933
Ciclev10033721m.g	scaffold_4:19691547-19699605	7.04593	10.511	0.577038
Ciclev10034010m.g	scaffold_4:2045223-2050369	17.0327	25.4115	0.577176
Ciclev10000508m.g	scaffold_5:41372512-41377280	3.7659	5.61873	0.577248
Ciclev10018884m.g	scaffold_3:44778266-44783857	14.9841	22.358	0.57736
Ciclev10017499m.g	scaffold_2:9082569-9087523	4.98602	7.43985	0.577383
Ciclev10018504m.g	scaffold_3:24779629-24784345	1.77098	2.64313	0.577695
Ciclev10027662m.g	scaffold_8:20112108-20133769	0.268746	0.401152	0.577907
Ciclev10016858m.g	scaffold_2:4837587-4839968	27.989	41.7812	0.577994
Ciclev10001062m.g	scaffold_5:27004513-27012050	67.7451	101.173	0.578632
Ciclev10009141m.g	scaffold_1:27286386-27290085	8.24621	12.3164	0.578772
Ciclev10018084m.g	scaffold_2:8348177-8359719	14.2497	21.2839	0.57883
Ciclev10029792m.g	scaffold_8:13608878-13610532	6.64887	9.93108	0.578841
Ciclev10001682m.g	scaffold_5:36472136-36475001	31.2833	46.7286	0.578915
Ciclev10016768m.g	scaffold_2:29883680-29886063	27.2658	40.7302	0.579006
Ciclev10027599m.g	scaffold_7:5801207-5803835	0.763826	1.14104	0.579031
Ciclev10000014m.g	scaffold_5:29424164-29437482	10.3364	15.4422	0.579144
Ciclev10031898m.g	scaffold_4:5833445-5837560	7.99559	11.9454	0.57918

Ciclev10016039m.g	scaffold_2:21791664-21797802	13.4786	20.138	0.579248
Ciclev10004813m.g	scaffold_9:29224428-29227697	9.79948	14.6442	0.579555
Ciclev10016075m.g	scaffold_2:29732248-29735969	44.3251	66.2482	0.579759
Ciclev10020014m.g	scaffold_3:4041215-4043379	3.4466	5.15166	0.579863
Ciclev10024880m.g	scaffold_7:1300942-1308124	10.4758	15.6607	0.580089
Ciclev10028039m.g	scaffold_8:47361-52441	0.537092	0.802971	0.580179
Ciclev10030711m.g	scaffold_4:3960299-3971216	16.3984	24.5178	0.580276
Ciclev10026360m.g	scaffold_7:16070063-16072936	63.0746	94.3388	0.580791
Ciclev10030532m.g	scaffold_4:3249709-3254808	15.6772	23.4582	0.581417
Ciclev10013654m.g	scaffold_6:21925202-21932500	6.19933	9.27661	0.581485
Ciclev10009899m.g	scaffold_1:4601587-4603705	117.488	175.824	0.581624
Ciclev10030749m.g	scaffold_4:22297046-22302917	6.47094	9.68419	0.581655
Ciclev10014432m.g	scaffold_2:10901354-10906959	18.2479	27.3166	0.582045
Ciclev10027188m.g	scaffold_7:19148981-19150114	9.65448	14.4545	0.582245
Ciclev10005771m.g,Ciclev10005779m.g	scaffold_9:5579382-5584787	88.8473	133.061	0.582683
Ciclev10019348m.g	scaffold_3:41345628-41350936	47.501	71.1895	0.583706
Ciclev10020602m.g	scaffold_3:20870455-20876403	7.9391	11.9021	0.584169
Ciclev10020136m.g	scaffold_3:470593-473348	16.3377	24.496	0.584339
Ciclev10018210m.g	scaffold_2:10243837-10247274	9.19624	13.7884	0.584343
Ciclev10014017m.g	scaffold_2:121854-133496	6.59436	9.88738	0.584356
Ciclev10012559m.g	scaffold_6:21589936-21593918	72.6738	108.991	0.584706
Ciclev10001531m.g	scaffold_5:41316977-41320780	17.3232	25.9841	0.584924
Ciclev10024889m.g	scaffold_7:21048848-21055380	14.7118	22.0705	0.585148
Ciclev10020278m.g	scaffold_3:23769524-23773006	3.06696	4.60106	0.585157
Ciclev10026371m.g	scaffold_7:2902620-2905534	14.3686	21.5567	0.585211
Ciclev10010231m.g	scaffold_1:9292607-9294211	0.495513	0.743425	0.585264
Ciclev10000015m.g	scaffold_5:12783975-12798505	11.794	17.6951	0.585301
Ciclev10033172m.g	scaffold_4:17135851-17136472	0.483314	0.725162	0.585344
Ciclev10018770m.g	scaffold_3:28029335-28034214	2.09478	3.14326	0.585464
Ciclev10029031m.g	scaffold_8:179438-180802	5.23936	7.86281	0.585653
Ciclev10031342m.g	scaffold_4:22384964-22387523	9.26918	13.9107	0.585683
Ciclev10007740m.g	scaffold_1:23677552-23683204	15.1084	22.6752	0.585766
Ciclev10008274m.g	scaffold_1:2905891-2909734	12.5325	18.815	0.586209
Ciclev10027319m.g	scaffold_7:13738232-13742011	5.93582	8.91168	0.586251
Ciclev10025622m.g	scaffold_7:4830585-4833659	4.56343	6.85386	0.586799
Ciclev10031760m.g	scaffold_4:1528602-1531114	2.25015	3.37969	0.58687
Ciclev10029097m.g	scaffold_8:4079287-4081937	3.38905	5.09193	0.587333
Ciclev10006699m.g	scaffold_9:1663233-1663989	11.2473	16.8989	0.587344
Ciclev10018751m.g	scaffold_3:3150351-3159621	45.9907	69.1064	0.587478
Ciclev10010658m.g	scaffold_1:3045538-3049336	25.1595	37.8089	0.58762
Ciclev10020060m.g	scaffold_3:32543167-32579333	1.16031	1.74371	0.58765
Ciclev10007827m.g	scaffold_1:4119708-4122521	5.42678	8.15567	0.587707
Ciclev10007948m.g	scaffold_1:4148092-4154018	37.2984	56.0552	0.587735
Ciclev10005303m.g	scaffold_9:2516299-2517513	0.61587	0.925646	0.587835
Ciclev10030667m.g	scaffold_4:17983540-17986596	0.430685	0.647375	0.587969
Ciclev10013714m.g	scaffold_6:22678094-22682872	5.63641	8.47228	0.587974
Ciclev10015292m.g	scaffold_2:4456743-4460417	5.10496	7.67483	0.588235
Ciclev10031249m.g	scaffold_4:20781370-20785697	25.3783	38.154	0.588238
Ciclev10025711m.g	scaffold_7:718487-722647	31.4641	47.304	0.588255
Ciclev10005335m.g	scaffold_9:21619947-21622291	5.44269	8.18495	0.588654
Ciclev10010336m.g	scaffold_1:23373032-23377281	17.0979	25.7178	0.58895
Ciclev10025945m.g	scaffold_7:4612886-4617115	37.8103	56.8796	0.589131
Ciclev10010807m.g	scaffold_1:14639718-14641770	3.6178	5.44286	0.589251
Ciclev10013628m.g	scaffold_6:13645122-13648531	0.299021	0.450019	0.589739
Ciclev10006485m.g	scaffold_9:4591268-4592909	1.51395	2.2786	0.589833

Ciclev10004918m.g	scaffold_9:1695243-1699621	36.3685	54.7374	0.58984
Ciclev10031873m.g	scaffold_4:13751432-13754767	5.14975	7.756	0.59081
Ciclev10012657m.g	scaffold_6:21537951-21540938	21.0252	31.667	0.590862
Ciclev10006006m.g	scaffold_9:4116355-4117315	77.444	116.643	0.590877
Ciclev10027410m.g	scaffold_7:7923448-7945523	0.335102	0.504818	0.591163
-	scaffold_5:28539341-28801354	10.9108	16.4384	0.591316
Ciclev10027722m.g,Ciclev10029929m.g,Ciclev10030315m.g	scaffold_8:3257244-3394853	197.8	298.064	0.591578
Ciclev10028825m.g	scaffold_8:47361-52441	21.703	32.7077	0.591735
Ciclev10020375m.g	scaffold_3:45418663-45421172	4.00371	6.03639	0.59235
-	scaffold_7:2154775-2158292	56.802	85.6555	0.592604
Ciclev10026494m.g	scaffold_7:8302530-8310349	6.85475	10.3371	0.592663
Ciclev10008494m.g	scaffold_1:26664743-26668680	1.76529	2.66223	0.592731
Ciclev10027820m.g	scaffold_8:4960747-4966385	14.9492	22.5517	0.593169
Ciclev10033198m.g	scaffold_4:24439200-24442319	22.788	34.3784	0.593229
Ciclev10010260m.g	scaffold_1:23516499-23522116	11.7155	17.677	0.593456
Ciclev10003278m.g	scaffold_5:38762885-38763568	0.405175	0.611465	0.593725
Ciclev10004994m.g	scaffold_9:30811902-30816520	19.492	29.4191	0.593875
Ciclev10014719m.g	scaffold_2:22744617-22750220	41.1713	62.1428	0.593948
Ciclev10004441m.g	scaffold_9:3944485-3949429	0.732572	1.10606	0.594386
Ciclev10021278m.g	scaffold_3:3133682-3135227	1.67694	2.53201	0.594457
Ciclev10000537m.g	scaffold_5:39992233-39996269	87.376	131.972	0.594923
Ciclev10029458m.g	scaffold_8:876476-877346	6.71092	10.139	0.59533
Ciclev10030235m.g	scaffold_8:1241304-1253939	0.59892	0.905257	0.595965
Ciclev10007818m.g	scaffold_1:22604665-22610697	11.0053	16.6359	0.5961
-	scaffold_4:3315480-3315638	750.404	1135.22	0.597228
Ciclev10001008m.g	scaffold_5:35917383-35925361	6.30436	9.53757	0.597271
Ciclev10005501m.g	scaffold_9:28824995-28829025	16.0708	24.3195	0.597672
Ciclev10024683m.g	scaffold_7:11532703-11547475	29.1456	44.1075	0.597745
Ciclev10018718m.g,Ciclev10024632m.g	scaffold_3:812627-822104	25.3547	38.3738	0.59787
Ciclev10031994m.g,Ciclev10032128m.g	scaffold_4:24962416-24969318	104.275	157.822	0.597896
Ciclev10031016m.g	scaffold_4:15490682-15498619	4.06846	6.15849	0.598094
Ciclev10018690m.g	scaffold_3:45062124-45068788	16.2779	24.6435	0.598286
Ciclev10008084m.g	scaffold_1:920122-923236	2.50554	3.79387	0.598547
Ciclev10004396m.g	scaffold_9:22309969-22314448	1.97508	2.99094	0.598688
Ciclev10004122m.g	scaffold_9:26301777-26322747	7.57714	11.4753	0.598803
Ciclev10000722m.g	scaffold_5:40972046-40979507	22.1651	33.5761	0.599143
Ciclev10000511m.g	scaffold_5:40608944-40612620	4.89669	7.4199	0.599592
-	scaffold_3:30862136-30865641	15.9256	24.1326	0.599634
Ciclev10018093m.g	scaffold_2:12027300-12036334	4.27766	6.48314	0.599871
Ciclev10018807m.g	scaffold_3:46625278-46629564	98.5054	149.325	0.600182
Ciclev10011544m.g	scaffold_6:18484765-18486727	92.9812	140.956	0.600235
Ciclev10029381m.g	scaffold_8:22375152-22376849	15.2168	23.0723	0.60049
Ciclev10025300m.g	scaffold_7:8926401-8931884	17.1934	26.0738	0.600745
Ciclev10011183m.g	scaffold_6:25140517-25146487	6.49526	9.85133	0.60093
Ciclev10018740m.g	scaffold_3:39696781-39707257	9.32182	14.1418	0.601286
Ciclev10014552m.g	scaffold_2:17261319-17263476	4.72693	7.17255	0.601582
Ciclev10026120m.g	scaffold_7:8905795-8909126	47.9014	72.696	0.601808
Ciclev10001105m.g	scaffold_5:37066207-37070717	4.67925	7.10254	0.602058
Ciclev10014773m.g	scaffold_2:34469662-34473333	61.0769	92.7191	0.602238
Ciclev10012798m.g	scaffold_6:11739633-11741636	10.9037	16.5527	0.602252
Ciclev10019924m.g	scaffold_3:3650293-3652372	379.757	576.533	0.602326
Ciclev10024268m.g	scaffold_3:50830776-50839587	29.4823	44.7734	0.602796

Ciclev10011418m.g	scaffold_6:25048397-25053494	23.0585	35.0338	0.603448
Ciclev10026205m.g	scaffold_7:738497-741571	11.3138	17.19	0.603479
Ciclev10009059m.g	scaffold_1:21086306-21090724	12.5548	19.0759	0.603513
Ciclev10019982m.g	scaffold_3:48012510-48015634	13.4347	20.4151	0.603673
Ciclev10019952m.g	scaffold_3:21545317-21547709	2.01528	3.06282	0.603882
Ciclev10015758m.g	scaffold_2:8275058-8283003	21.2527	32.3	0.60389
Ciclev10030740m.g	scaffold_4:2317672-2326121	11.5651	17.582	0.60433
Ciclev10001195m.g	scaffold_5:37478178-37482337	88.0636	133.887	0.604397
Ciclev10012148m.g	scaffold_6:23782577-23786090	5.61582	8.5402	0.604773
Ciclev10032014m.g	scaffold_4:21639441-21642342	163.603	248.806	0.604819
Ciclev10005793m.g	scaffold_9:14837073-14840606	15.1246	23.0027	0.604903
Ciclev10022068m.g	scaffold_3:9868143-9869414	15.6702	23.8338	0.604981
Ciclev10025386m.g	scaffold_7:20901325-20907028	38.843	59.0922	0.605311
Ciclev10016825m.g	scaffold_2:13441812-13443080	130.534	198.636	0.605706
Ciclev10008564m.g	scaffold_1:21406618-21411215	14.8664	22.6255	0.605901
Ciclev10025418m.g	scaffold_7:5815048-5819452	13.4762	20.5121	0.606061
Ciclev10010402m.g	scaffold_1:4829562-4834618	0.29514	0.449329	0.606372
Ciclev10031473m.g	scaffold_4:2287613-2290410	5.75923	8.76877	0.606498
Ciclev10015531m.g	scaffold_2:34873434-34875323	2.79541	4.25636	0.60656
Ciclev10001989m.g	scaffold_5:43143531-43144749	0.209058	0.318353	0.606722
Ciclev10031652m.g	scaffold_4:17219853-17221317	16.6463	25.3512	0.606857
Ciclev10006195m.g	scaffold_9:5753012-5754613	131.018	199.551	0.607001
Ciclev10015808m.g	scaffold_2:6900991-6905827	40.5431	61.7656	0.607348
Ciclev10012138m.g	scaffold_6:17695125-17698186	8.00801	12.2002	0.60739
Ciclev10026982m.g	scaffold_7:16939640-16973171	4.87555	7.42953	0.607706
Ciclev10011942m.g	scaffold_6:19392565-19398731	5.15867	7.86109	0.607731
Ciclev10000550m.g	scaffold_5:35237122-35239909	12.2899	18.7336	0.608157
Ciclev10018335m.g	scaffold_2:34426124-34431846	121.135	184.697	0.608548
Ciclev10008092m.g	scaffold_1:26132645-26136128	6.14792	9.37625	0.608913
Ciclev10015133m.g	scaffold_2:32941542-32946565	4.43417	6.76398	0.609209
Ciclev10019042m.g,Ciclev10019193m.g	scaffold_3:36363008-36553812	2.93465	4.47758	0.60953
Ciclev10005019m.g	scaffold_9:30687093-30691159	14.4345	22.026	0.609682
Ciclev10015848m.g	scaffold_2:24610606-24613357	68.6674	104.799	0.609924
Ciclev10026081m.g	scaffold_7:5640018-5643125	70.7713	108.034	0.610246
Ciclev10031073m.g	scaffold_4:23404931-23425248	56.4105	86.147	0.610837
Ciclev10019177m.g	scaffold_3:13580885-13586189	65.3754	99.8606	0.611167
Ciclev10020467m.g	scaffold_3:41874927-41879717	11.3341	17.3149	0.611337
Ciclev10031629m.g	scaffold_4:11199918-11201484	41.4396	63.3072	0.61136
-	scaffold_7:5276005-5276785	25.3277	38.6936	0.611381
Ciclev10005752m.g,Ciclev10006994m.g	scaffold_9:2768407-2829028	6.02088	9.19906	0.611513
Ciclev10007702m.g,Ciclev10007965m.g	scaffold_1:863426-878303	61.6928	94.264	0.611605
Ciclev10003422m.g	scaffold_5:40296378-40296927	7.56204	11.555	0.61167
Ciclev10033747m.g	scaffold_4:24943994-24946215	53.2474	81.3778	0.611923
Ciclev10004825m.g	scaffold_9:28551250-28554733	2.58286	3.9475	0.611973
Ciclev10028147m.g	scaffold_8:21112803-21117580	115.709	176.953	0.612865
Ciclev10000659m.g	scaffold_5:9197773-9204364	15.283	23.3833	0.613552
-	scaffold_3:27954784-27955101	12.7617	19.5322	0.614035
Ciclev10002722m.g	scaffold_5:42284790-42286274	8.02502	12.2833	0.614118
-	scaffold_5:28539341-28801354	36.4547	55.8119	0.614469
Ciclev10030908m.g	scaffold_4:23514245-23516351	2.15642	3.3015	0.614487
Ciclev10009829m.g	scaffold_1:27111411-27114276	10.686	16.364	0.614803
Ciclev10001011m.g	scaffold_5:36883102-36887975	8.12778	12.4469	0.614852
Ciclev10020196m.g	scaffold_3:50765020-50767809	1.46001	2.23616	0.615045

Ciclev10030569m.g	scaffold_4:18130851-18141808	19.1822	29.3796	0.615045
Ciclev10004244m.g	scaffold_9:9493900-9499167	0.237352	0.363572	0.615216
Ciclev10030928m.g	scaffold_4:2717013-2719856	62.0353	95.0326	0.615334
Ciclev10000689m.g,Ciclev10002476m.g,Ciclev10002670m.g	scaffold_5:4471259-4568404	30.1095	46.1277	0.615414
Ciclev10019183m.g	scaffold_3:25441987-25444731	37.4431	57.3949	0.616222
Ciclev10010308m.g	scaffold_1:1578589-1580800	3.30573	5.06732	0.616254
Ciclev10013303m.g	scaffold_6:10032777-10034745	8.02892	12.3096	0.61651
Ciclev10000960m.g,Ciclev10000980m.g,Ciclev10001263m.g,Ciclev10003486m.g	scaffold_5:31925317-31954322	48.1114	73.7719	0.616691
Ciclev10007443m.g	scaffold_1:19326315-19329876	57.528	88.2187	0.616821
Ciclev10020407m.g	scaffold_3:479682-483210	23.5842	36.1678	0.616883
Ciclev10011087m.g	scaffold_6:14327887-14333437	14.1893	21.7653	0.617227
Ciclev10033394m.g	scaffold_4:21315342-21317721	30.3379	46.5386	0.617304
Ciclev10009594m.g	scaffold_1:26727577-26729609	7.11064	10.908	0.617335
Ciclev10016324m.g	scaffold_2:33988035-33989927	5.7248	8.78275	0.617449
Ciclev10029629m.g	scaffold_8:17291958-17467945	4.27949	6.56958	0.618361
Ciclev10021174m.g	scaffold_3:46685980-46687931	5.1112	7.84757	0.618583
Ciclev10009026m.g	scaffold_1:8543172-8547077	18.6937	28.7045	0.618725
Ciclev10025825m.g	scaffold_7:16201970-16207283	6.17414	9.48091	0.618787
Ciclev10009959m.g	scaffold_1:357266-358021	0.73675	1.13142	0.618888
Ciclev10012147m.g	scaffold_6:20027924-20028984	38.0535	58.4389	0.618901
Ciclev10026826m.g	scaffold_7:8651497-8653529	13.5843	20.8626	0.618972
Ciclev10009037m.g	scaffold_1:26946403-26948798	1.07606	1.65269	0.61906
Ciclev10011111m.g	scaffold_6:24165665-24169476	2.10545	3.23463	0.619473
Ciclev10016550m.g	scaffold_2:21269910-21273936	16.7271	25.6984	0.619493
-	scaffold_4:11971225-11973841	5.80523	8.921	0.619852
Ciclev10020878m.g	scaffold_3:541574-543121	6.16619	9.47587	0.61988
Ciclev10014640m.g	scaffold_2:224768-229598	311.81	479.191	0.619935
Ciclev10015213m.g	scaffold_2:14233233-14237268	30.5742	46.9887	0.619997
Ciclev10030914m.g	scaffold_4:20106584-20113259	0.761575	1.17069	0.620303
Ciclev10030825m.g	scaffold_4:22901625-22912461	21.0072	32.2946	0.620406
Ciclev10013651m.g	scaffold_6:16851371-16853293	3.54616	5.45274	0.620726
Ciclev10027380m.g	scaffold_7:8662040-8665543	19.1641	29.4686	0.620772
Ciclev10014346m.g	scaffold_2:15754315-15758930	0.058962	0.0906858	0.621091
Ciclev10001196m.g	scaffold_5:36409789-36413418	7.12799	10.9641	0.621225
Ciclev10019026m.g	scaffold_3:3682394-3687764	11.3005	17.3828	0.621277
Ciclev10005725m.g	scaffold_9:25850829-25854346	0.457961	0.704458	0.621288
-	scaffold_8:2189935-2191064	301.922	464.628	0.621901
Ciclev10018393m.g	scaffold_2:34789085-34791277	7.56142	11.6372	0.622011
-	scaffold_5:10979088-10979787	11.0849	17.0631	0.622286
Ciclev10023740m.g	scaffold_3:7025543-7039885	10.2823	15.8278	0.622302
Ciclev10001185m.g	scaffold_5:22686599-22688174	9.22165	14.1954	0.622331
Ciclev10005101m.g	scaffold_9:30836434-30841216	111.833	172.167	0.622467
Ciclev10007339m.g	scaffold_1:182933-193447	6.66907	10.2689	0.622718
Ciclev10017149m.g	scaffold_2:33394433-33397596	2.93953	4.5271	0.623002
Ciclev10032676m.g	scaffold_4:25022320-25025779	13.8977	21.4049	0.623099
-	scaffold_2:28503750-28504395	3.0956	4.76812	0.623202
Ciclev10007750m.g	scaffold_1:8138750-8170757	8.14216	12.5415	0.62323
Ciclev10030661m.g	scaffold_4:23575398-23582495	13.2826	20.4615	0.623372
Ciclev10008694m.g	scaffold_1:22719561-22721903	5.01377	7.72358	0.623373
Ciclev10025805m.g	scaffold_7:20863929-20867816	8.79837	13.5568	0.623711
-	scaffold_1:5167223-5167489	16.9705	26.1517	0.623879
Ciclev10003847m.g	scaffold_5:38412286-38413804	1.9306	2.97566	0.624157

Ciclev10001886m.g	scaffold_5:37797901-37801255	16.2104	24.9855	0.62417
Ciclev10006458m.g	scaffold_9:21624002-21626933	6.47867	9.98903	0.624646
Ciclev10002653m.g	scaffold_5:29699946-29702961	30.5433	47.1057	0.625047
Ciclev10010061m.g	scaffold_1:23476494-23477057	0.604391	0.932148	0.625076
Ciclev10017475m.g	scaffold_2:33506163-33509687	43.0922	66.4635	0.625136
Ciclev10013611m.g	scaffold_6:22928778-22931069	5.45081	8.41013	0.625658
Ciclev10000330m.g	scaffold_5:42618465-42624771	12.3618	19.0779	0.626017
Ciclev10028859m.g	scaffold_8:23069544-23072925	6.04903	9.34004	0.626724
Ciclev10008683m.g	scaffold_1:24530549-24532751	75.6075	116.75	0.626821
Ciclev10000998m.g	scaffold_5:38363304-38367051	5.30303	8.18912	0.626891
Ciclev10020872m.g	scaffold_3:31535236-31540844	1.87516	2.89596	0.627029
Ciclev10027347m.g	scaffold_7:13385055-13389872	3.22024	4.97451	0.627386
Ciclev10007293m.g	scaffold_1:1515319-1523526	52.7367	81.4693	0.62745
-	scaffold_5:18149187-18149742	142.441	220.059	0.627525
Ciclev10009982m.g	scaffold_1:2739961-2740568	1.24653	1.92595	0.627654
Ciclev10018286m.g	scaffold_2:32308878-32311400	5.16795	7.98638	0.627948
Ciclev10019708m.g	scaffold_3:3431093-3434532	24.0217	37.1283	0.628182
Ciclev10025160m.g	scaffold_7:8226020-8229352	17.5848	27.1866	0.628567
Ciclev10007232m.g	scaffold_1:28103918-28118736	42.1499	65.1722	0.628729
Ciclev10014608m.g	scaffold_2:31971270-31977611	10.0715	15.5827	0.629667
Ciclev10033211m.g	scaffold_4:17766907-17767454	0.529994	0.820187	0.629978
Ciclev10002968m.g	scaffold_5:35270039-35281019	14.9707	23.1682	0.630003
Ciclev10023480m.g	scaffold_3:38299628-38300495	0.202047	0.312694	0.630059
Ciclev10001692m.g	scaffold_5:42294269-42295612	1243.23	1924.06	0.630059
Ciclev10025884m.g	scaffold_7:9330973-9333708	20.055	31.046	0.630447
Ciclev10015622m.g	scaffold_2:28259600-28264707	22.7913	35.2842	0.63054
Ciclev10012172m.g	scaffold_6:22715562-22719006	71.1711	110.19	0.630632
Ciclev10008107m.g	scaffold_1:1592274-1598844	8.63911	13.377	0.630806
-	scaffold_9:13844210-13844401	61.5302	95.2794	0.630871
Ciclev10008444m.g	scaffold_1:22407411-22409529	1.5931	2.46735	0.631121
-	scaffold_5:29110006-29110688	2.13719	3.31025	0.631227
Ciclev10019254m.g	scaffold_3:8214732-8216864	0.507304	0.785839	0.631383
Ciclev10013200m.g	scaffold_6:22210588-22211749	3.50263	5.42583	0.631405
Ciclev10000967m.g	scaffold_5:39791663-39793900	2.33458	3.61709	0.631664
Ciclev10005278m.g	scaffold_9:17436968-17441987	15.273	23.6655	0.631805
Ciclev10026505m.g	scaffold_7:441499-443316	42.1773	65.3606	0.631955
Ciclev10014030m.g	scaffold_2:27310236-27325458	26.0218	40.3298	0.632126
-	scaffold_4:22936845-22938558	31.5244	48.8786	0.632735
Ciclev10027726m.g	scaffold_8:23689903-23696772	23.877	37.0277	0.632983
Ciclev10014932m.g	scaffold_2:9683239-9688853	14.5639	22.587	0.633103
Ciclev10017959m.g	scaffold_2:28397526-28398986	0.175271	0.272052	0.634295
Ciclev10010822m.g	scaffold_1:23708961-23710839	2.08512	3.23651	0.634312
Ciclev10001407m.g	scaffold_5:36777391-36780420	10.0565	15.6106	0.634398
Ciclev10004916m.g	scaffold_9:12733152-12739048	4.54816	7.06015	0.634416
Ciclev10028560m.g	scaffold_8:3733634-3738382	20.9005	32.4459	0.634499
-	scaffold_1115:2162-2334	99.3961	154.314	0.634608
Ciclev10001988m.g	scaffold_5:34048741-34052274	84.3881	131.051	0.635019
Ciclev10001046m.g	scaffold_5:39086782-39089864	14.756	22.9201	0.635317
Ciclev10029749m.g	scaffold_8:17291958-17467945	0.512694	0.796597	0.635753
Ciclev10022675m.g	scaffold_3:41757160-41761117	16.8216	26.1372	0.63579
Ciclev10019136m.g	scaffold_3:4026944-4034249	8.81463	13.7021	0.636423
Ciclev10027981m.g,Ciclev10028007m.g	scaffold_8:23272524-23285663	171.827	267.184	0.636871
Ciclev10027733m.g	scaffold_8:22549969-22554172	0.737582	1.14706	0.637064
Ciclev10031124m.g	scaffold_4:11775032-11780691	6.46537	10.0565	0.637321
Ciclev10003480m.g	scaffold_5:40278613-40285992	15.408	23.9671	0.637381

Ciclev10000572m.g	scaffold_5:22271542-22276598	20.7092	32.2137	0.637406
Ciclev10024476m.g	scaffold_3:46934726-46936108	39.9416	62.1414	0.637664
Ciclev10007234m.g	scaffold_1:24175844-24190285	44.2661	68.8711	0.637697
Ciclev10029245m.g	scaffold_8:2856890-2858693	40.9462	63.7191	0.637997
Ciclev10005014m.g	scaffold_9:574951-577149	11.0444	17.1878	0.638067
Ciclev10009264m.g	scaffold_1:25322077-25323384	91.3151	142.114	0.63812
Ciclev10011148m.g	scaffold_6:11590795-11593274	36.7762	57.2382	0.638207
Ciclev10003425m.g	scaffold_5:1437740-1439767	25.4387	39.5956	0.638315
Ciclev10027894m.g	scaffold_8:2888991-2895645	32.3988	50.4376	0.63856
Ciclev10018517m.g	scaffold_3:44849890-44857702	10.4463	16.2639	0.638685
Ciclev10007554m.g	scaffold_1:5468973-5476821	18.6651	29.0617	0.63878
Ciclev10007051m.g	scaffold_9:2606275-2607947	1.12592	1.75324	0.638922
Ciclev10019805m.g	scaffold_3:49592327-49594525	26.4328	41.164	0.639054
Ciclev10027649m.g	scaffold_1831:2634-3201	7.1034	11.0646	0.639371
Ciclev10012868m.g	scaffold_6:16221894-16225489	19.6582	30.6239	0.639528
Ciclev10009243m.g	scaffold_1:14107966-14109217	152.768	238.002	0.639632
Ciclev10027697m.g	scaffold_8:6193559-6199421	23.5575	36.7108	0.640017
Ciclev10001295m.g	scaffold_5:655534-811195	3.77846	5.88911	0.640252
Ciclev10015669m.g	scaffold_2:33250730-33255285	12.8071	19.9621	0.640313
Ciclev10004480m.g	scaffold_9:13861431-13863894	23.2369	36.2293	0.640738
Ciclev10027594m.g	scaffold_7:12285085-12288663	1.37064	2.1371	0.640803
Ciclev10010989m.g	scaffold_6:21767463-21775741	24.8947	38.8272	0.641226
Ciclev10028910m.g	scaffold_8:24248766-24251495	21.2078	33.0809	0.641405
Ciclev10020008m.g	scaffold_3:8868430-8872712	7.60161	11.8581	0.641494
-	scaffold_8:7696587-7697548	6.45903	10.0758	0.641498
Ciclev10020122m.g	scaffold_3:4270032-4272353	3.31488	5.17219	0.641818
Ciclev10005912m.g	scaffold_9:30704941-30705765	0.28805	0.449464	0.641886
Ciclev10008080m.g	scaffold_1:8004293-8008148	2.91872	4.55465	0.642003
Ciclev10004762m.g	scaffold_9:28735829-28740315	8.68161	13.5482	0.642068
Ciclev10030242m.g	scaffold_8:6355457-6359023	0.403251	0.629493	0.642511
Ciclev10022776m.g	scaffold_3:45137043-45145995	4.03265	6.29564	0.642625
Ciclev10032838m.g	scaffold_4:213481-215848	9.38464	14.6517	0.642696
Ciclev10005762m.g	scaffold_9:14202943-14204497	15.4225	24.0844	0.643062
Ciclev10016000m.g,Ciclev10016020m.g	scaffold_2:33216838-33223206	49.1789	76.8177	0.643399
Ciclev10015161m.g	scaffold_2:11668762-11672285	12.5735	19.6415	0.643516
Ciclev10010461m.g	scaffold_1:27025453-27027577	0.455976	0.712679	0.644295
Ciclev10007252m.g	scaffold_1:22943009-22953887	8.85479	13.8426	0.644583
Ciclev10003624m.g	scaffold_5:11355971-11379757	64.0589	100.151	0.644703
Ciclev10014145m.g	scaffold_2:29559639-29563784	2.33158	3.64539	0.644765
Ciclev10024919m.g	scaffold_7:4861024-4880959	103.642	162.044	0.644772
Ciclev10007869m.g	scaffold_1:18003471-18011122	1.77426	2.77428	0.644896
Ciclev10014801m.g	scaffold_2:33942477-33948382	22.6989	35.505	0.6454
Ciclev10010068m.g	scaffold_1:18886440-18886981	0.486154	0.760513	0.645561
Ciclev10010222m.g	scaffold_1:22527430-22529497	0.127899	0.200081	0.645577
Ciclev10014887m.g	scaffold_2:32137991-32143332	2.18527	3.41862	0.645601
Ciclev10004452m.g	scaffold_9:30163052-30167953	20.9295	32.7478	0.645857
Ciclev10014386m.g	scaffold_2:32605276-32609032	2.64615	4.14044	0.645887
Ciclev10007832m.g	scaffold_1:10530303-10534418	1.99895	3.12807	0.646032
Ciclev10024743m.g	scaffold_7:8388150-8399932	4.30361	6.73509	0.64615
Ciclev10010963m.g	scaffold_6:24463525-24479926	6.14771	9.6227	0.646391
Ciclev10009446m.g	scaffold_1:25730869-25733190	51.9082	81.2642	0.646657
Ciclev10020794m.g	scaffold_3:46413113-46415022	14.1227	22.1178	0.647192
Ciclev10000784m.g	scaffold_5:20105818-20108873	13.9775	21.8918	0.647282
Ciclev10026768m.g	scaffold_7:6039093-6040117	0.528657	0.828025	0.647344
Ciclev10018922m.g	scaffold_3:10896168-10902243	26.8241	42.0202	0.647554

Ciclev10019478m.g	scaffold_3:18798042-18805218	7.48701	11.729	0.647612
Ciclev10001495m.g	scaffold_5:37413098-37417182	2.6611	4.16932	0.647788
-	scaffold_5:40515045-40515395	121.286	190.038	0.647867
Ciclev10030218m.g	scaffold_8:22075532-22078940	30.4972	47.7888	0.647997
Ciclev10016611m.g	scaffold_2:26829274-26830778	0.431449	0.676193	0.648245
Ciclev10019579m.g,Ciclev10023270m.g	scaffold_3:38505310-38528624	3.70223	5.80328	0.648473
Ciclev10024040m.g	scaffold_3:27160749-27163347	9.68008	15.1737	0.648483
Ciclev10031144m.g	scaffold_4:565005-566771	0.103131	0.161705	0.648888
Ciclev10005970m.g	scaffold_9:24195179-24198282	88.537	138.827	0.648938
Ciclev10011066m.g	scaffold_6:18500224-18504827	2.65231	4.16034	0.649449
Ciclev10028858m.g	scaffold_8:9896063-9898975	5.19445	8.14799	0.649474
Ciclev10002419m.g	scaffold_5:15052797-15054938	70.4497	110.511	0.649519
Ciclev10000333m.g	scaffold_5:26136985-26141621	1.54621	2.42576	0.649699
Ciclev10011521m.g	scaffold_6:13620022-13622098	10.3968	16.3207	0.650565
Ciclev10021345m.g	scaffold_3:50351699-50353151	1.93691	3.04177	0.651156
Ciclev10008570m.g	scaffold_1:4873969-4878979	8.77314	13.7792	0.651324
Ciclev10030594m.g	scaffold_4:6464975-6472063	13.6206	21.3948	0.651475
Ciclev10031453m.g	scaffold_4:16763185-16765286	9.85721	15.486	0.651717
Ciclev10005521m.g,Ciclev10006184m.g	scaffold_9:27914456-27918109	20.457	32.1513	0.652283
Ciclev10014354m.g	scaffold_2:30447501-30452911	9.6268	15.1304	0.652318
Ciclev10010094m.g	scaffold_1:13902212-13905427	4.134	6.4987	0.652613
Ciclev10004128m.g	scaffold_9:23415521-23442978	10.7484	16.8976	0.652691
Ciclev10022295m.g	scaffold_3:43326235-43327717	0.169616	0.266658	0.652717
Ciclev10021362m.g	scaffold_3:41423374-41424845	50.7725	79.8346	0.652965
Ciclev10024953m.g	scaffold_7:15589419-15594676	15.4357	24.2711	0.652965
Ciclev10023613m.g	scaffold_3:27243915-27260256	0.284225	0.446974	0.653157
Ciclev10019256m.g	scaffold_3:42526929-42528973	2.94044	4.62417	0.653165
Ciclev10026151m.g	scaffold_7:14282400-14286582	22.151	34.8353	0.653177
Ciclev10019568m.g	scaffold_3:12945480-12949105	14.4665	22.7505	0.653179
Ciclev10008394m.g	scaffold_1:15806120-15811109	26.4866	41.6569	0.653293
Ciclev10004231m.g	scaffold_9:507375-518725	14.7176	23.151	0.653532
Ciclev10012001m.g	scaffold_6:12993286-12995307	2.50059	3.93482	0.654026
Ciclev10016477m.g	scaffold_2:27811961-27815404	0.936255	1.47352	0.654297
Ciclev10016303m.g	scaffold_2:20258323-20260070	0.218235	0.343499	0.654424
Ciclev10014031m.g	scaffold_2:31241079-31249842	3.63951	5.73046	0.654904
Ciclev10014062m.g	scaffold_2:33259396-33269388	14.7355	23.2041	0.655081
Ciclev10028838m.g	scaffold_8:6041183-6043615	27.4856	43.2897	0.655345
Ciclev10031772m.g	scaffold_4:8414981-8419415	32.5927	51.3561	0.655985
Ciclev10030581m.g	scaffold_4:7396188-7405205	4.31349	6.79704	0.656051
Ciclev10027681m.g	scaffold_8:22774397-22789780	4.2631	6.71773	0.656069
Ciclev10019447m.g	scaffold_3:25625681-25630835	2.11948	3.3417	0.656875
Ciclev10011151m.g	scaffold_6:10635891-10638105	2.15888	3.40419	0.657026
Ciclev10026606m.g	scaffold_7:13018358-13021613	43.3374	68.3394	0.657104
Ciclev10011465m.g	scaffold_6:25364316-25368608	47.4013	74.7849	0.657822
Ciclev10004157m.g	scaffold_9:22327182-22332447	1.3438	2.12027	0.657934
Ciclev10032361m.g	scaffold_4:1282349-1292520	15.4674	24.406	0.658006
Ciclev10010592m.g	scaffold_1:4582605-4584189	1.64439	2.59515	0.65827
Ciclev10022189m.g	scaffold_3:43215600-43219210	13.5586	21.3989	0.658333
Ciclev10019526m.g	scaffold_3:4264706-4268632	31.0378	48.9916	0.658507
Ciclev10021233m.g	scaffold_3:30867973-30870542	5.75968	9.09299	0.658767
Ciclev10002031m.g	scaffold_5:29120015-29124608	3.65286	5.7674	0.658894
Ciclev10010563m.g	scaffold_1:15994489-16002359	17.5333	27.6941	0.659479
Ciclev10000116m.g	scaffold_5:37735872-37749205	6.71884	10.6169	0.660085
Ciclev10011760m.g	scaffold_6:17369082-17372883	0.71456	1.12917	0.660134

Ciclev10019313m.g	scaffold_3:1690126-1693380	6.14329	9.70919	0.660339
Ciclev10030479m.g	scaffold_4:21786360-21793479	5.51784	8.72077	0.66035
Ciclev10022861m.g	scaffold_3:25074162-25076367	4.93255	7.79978	0.661101
Ciclev10023298m.g,Ciclev10024673m.g	scaffold_3:49853424-49859158	2.89558	4.57968	0.661394
Ciclev10003658m.g	scaffold_5:40340524-40342460	0.165832	0.262303	0.661508
Ciclev10004431m.g	scaffold_9:27464704-27467529	0.592866	0.937838	0.661633
Ciclev10000478m.g	scaffold_5:37082517-37084817	0.812989	1.28608	0.661678
Ciclev10026503m.g	scaffold_7:3951302-3954097	48.9512	77.4421	0.661772
Ciclev10005482m.g	scaffold_9:11301656-11303078	51.0004	80.7155	0.662338
Ciclev10002150m.g	scaffold_5:40226351-40230560	13.1849	20.8715	0.662648
Ciclev10033698m.g	scaffold_4:23784490-23786546	2.04944	3.24491	0.662947
Ciclev10007585m.g,Ciclev10010783m.g	scaffold_1:23561655-23577272	10.6301	16.8324	0.663089
Ciclev10028262m.g	scaffold_8:24521027-24524868	25.7153	40.7202	0.66312
Ciclev10030437m.g	scaffold_8:8192741-8195885	2.75138	4.35715	0.663229
Ciclev10033305m.g	scaffold_4:20738685-20743386	2.54372	4.02844	0.663284
Ciclev10003242m.g	scaffold_5:7225499-7225799	21.642	34.2756	0.663348
Ciclev10011548m.g	scaffold_6:24717947-24721579	2.2651	3.58816	0.663665
Ciclev10025548m.g	scaffold_7:13666318-13668698	9.77536	15.4876	0.663892
Ciclev10014879m.g	scaffold_2:26557493-26559148	1.22331	1.93885	0.664409
Ciclev10008640m.g	scaffold_1:16444730-16450516	8.81421	13.9722	0.664658
Ciclev10022428m.g	scaffold_3:45716859-45718557	27.0064	42.8106	0.664668
Ciclev10018281m.g	scaffold_2:25666295-25667505	0.712007	1.12891	0.66497
Ciclev10014348m.g	scaffold_2:35717169-35719814	20.5013	32.5061	0.664998
Ciclev10014821m.g	scaffold_2:26030785-26035127	30.3673	48.1569	0.665221
Ciclev10030570m.g	scaffold_4:24863453-24871794	25.0989	39.81	0.665506
Ciclev10024988m.g	scaffold_7:9170069-9175760	9.54854	15.147	0.665681
Ciclev10012628m.g	scaffold_6:21594496-21598242	4.81263	7.63501	0.665804
Ciclev10005485m.g,Ciclev10005831m.g	scaffold_9:21762291-21767104	18.9234	30.0222	0.665861
Ciclev10024720m.g	scaffold_7:20813210-20824784	22.6438	35.9298	0.666067
Ciclev10001787m.g	scaffold_5:26990578-26998915	6.98025	11.0765	0.666148
Ciclev10010701m.g	scaffold_1:3515774-3518431	0.264149	0.419166	0.666171
Ciclev10006149m.g	scaffold_9:1859698-1860593	0.400318	0.635389	0.666496
Ciclev10008583m.g	scaffold_1:15710756-15714366	35.5534	56.4382	0.666684
Ciclev10001212m.g	scaffold_5:21522432-21525218	4.78859	7.60231	0.666838
Ciclev10001035m.g	scaffold_5:37232472-37239661	13.2971	21.1125	0.666985
Ciclev10000217m.g	scaffold_5:41058941-41064267	0.399417	0.634297	0.667263
Ciclev10012964m.g	scaffold_6:15624796-15625794	332.444	528.001	0.66743
-	scaffold_8:10792426-10795911	6.07136	9.64338	0.667518
Ciclev10009443m.g	scaffold_1:22118202-22120791	42.8104	68.0165	0.667925
Ciclev10027796m.g	scaffold_8:24795575-24806623	30.6183	48.647	0.66796
Ciclev10023308m.g	scaffold_3:46519382-46595230	10.4932	16.6786	0.668538
Ciclev10018723m.g	scaffold_3:35750752-35754295	11.2703	17.9145	0.668606
Ciclev10033559m.g	scaffold_4:458503-463657	2.84598	4.52391	0.668644
Ciclev10030639m.g	scaffold_4:3430478-3436322	2.36149	3.75432	0.668853
Ciclev10014771m.g	scaffold_2:10234917-10241393	23.3587	37.1417	0.669078
Ciclev10024926m.g	scaffold_7:2680199-2686282	6.07765	9.6648	0.669227
Ciclev10028008m.g	scaffold_8:20514519-20516653	1.05824	1.68341	0.669722
Ciclev10028170m.g,Ciclev10028174m.g	scaffold_8:4506968-4526385	25.7752	41.0184	0.670288
Ciclev10012849m.g	scaffold_6:13498176-13501847	17.8797	28.4562	0.670421
Ciclev10032259m.g	scaffold_4:19183376-19189544	48.5047	77.2035	0.670542
Ciclev10008876m.g	scaffold_1:4009868-4027074	1.12133	1.78489	0.670621
Ciclev10020231m.g	scaffold_3:48290193-48293468	8.57189	13.6456	0.670749

Ciclev10025394m.g	scaffold_7:3797140-3800592	68.2965	108.729	0.670852
Ciclev10028169m.g	scaffold_8:5939928-5946922	16.1203	25.664	0.670862
Ciclev10004305m.g	scaffold_9:23481738-23487690	11.1295	17.7205	0.671038
-	scaffold_5:25548841-25549230	10.519	16.7489	0.671065
Ciclev10020524m.g	scaffold_3:35332190-35335063	7.73401	12.3154	0.67117
Ciclev10026432m.g	scaffold_7:10140827-10143902	8.04639	12.8128	0.67117
Ciclev10011284m.g	scaffold_6:9634290-9636185	1.89808	3.02332	0.671595
Ciclev10014098m.g	scaffold_2:14589553-14602226	4.38817	6.99019	0.67171
Ciclev10028638m.g	scaffold_8:2130234-2138606	171.974	273.965	0.671799
Ciclev10013692m.g	scaffold_6:18248776-18250446	27.6982	44.1276	0.67189
Ciclev10007563m.g	scaffold_1:24898768-24905716	16.7882	26.75	0.672095
Ciclev10027835m.g	scaffold_8:23736196-23740543	29.4689	46.9831	0.672947
Ciclev10013057m.g	scaffold_6:11521638-11523282	192.718	307.297	0.673146
Ciclev10014261m.g	scaffold_2:28737798-28743764	11.4383	18.2408	0.673291
Ciclev10026020m.g	scaffold_7:6878163-6882424	40.7381	64.9722	0.673444
Ciclev10019178m.g	scaffold_3:49626632-49630406	6.31905	10.0784	0.673484
Ciclev10009029m.g	scaffold_1:6455198-6457058	4.50802	7.19044	0.673585
Ciclev10029486m.g	scaffold_8:22319749-22322555	14.6115	23.3059	0.673591
Ciclev10028804m.g	scaffold_8:21369372-21370728	0.689213	1.09971	0.674103
Ciclev10030649m.g	scaffold_4:14476505-14481387	23.6377	37.7206	0.674261
Ciclev10021423m.g	scaffold_3:4840946-4864738	72.8486	116.28	0.674631
Ciclev10008149m.g	scaffold_1:4060374-4067203	48.2266	76.9852	0.674753
Ciclev10017877m.g	scaffold_2:28250063-28253234	18.7244	29.8913	0.674811
Ciclev10016011m.g	scaffold_2:9444644-9447429	3.73221	5.96131	0.675598
Ciclev10011628m.g	scaffold_6:20367278-20372481	7.21866	11.531	0.675716
Ciclev10022212m.g	scaffold_3:45551344-45552620	322.189	514.746	0.675954
Ciclev10000605m.g	scaffold_5:35262240-35264675	12.0301	19.2205	0.675989
Ciclev10016271m.g	scaffold_2:32819973-32821159	0.777434	1.24235	0.676281
Ciclev10014508m.g	scaffold_2:16752012-16759367	1.73095	2.76658	0.676538
Ciclev10014700m.g	scaffold_2:29365766-29369801	7.3552	11.7602	0.677079
Ciclev10010885m.g	scaffold_6:23916875-23941095	5.2062	8.32499	0.677218
Ciclev10031658m.g	scaffold_4:19524462-19527376	33.7163	53.9184	0.67733
Ciclev10029679m.g	scaffold_8:23446257-23448342	13.1092	20.9743	0.678043
Ciclev10005526m.g	scaffold_9:29739178-29741803	5.564	8.90322	0.678206
Ciclev10018621m.g	scaffold_3:48710999-48718124	21.4043	34.2579	0.678533
Ciclev10018600m.g	scaffold_3:41451332-41461154	9.28623	14.8635	0.678611
Ciclev10000867m.g	scaffold_5:35769297-35774649	1.5525	2.48495	0.678625
Ciclev10031362m.g	scaffold_4:23552855-23559896	8.66957	13.8776	0.678727
Ciclev10024883m.g	scaffold_7:9316580-9321526	51.2091	81.9731	0.67875
Ciclev10016703m.g	scaffold_2:29783147-29785224	93.4454	149.595	0.67887
Ciclev10030695m.g	scaffold_4:21389145-21402033	6.05894	9.70248	0.679288
Ciclev10010901m.g,Ciclev10010903m.g	scaffold_6:20891337-20913506	101.437	162.453	0.679448
Ciclev10024585m.g	scaffold_3:2056636-2057612	2.70052	4.32556	0.679649
Ciclev10002461m.g	scaffold_5:35510725-35512747	28.8909	46.2856	0.679947
-	scaffold_8:11065866-11066561	13.0164	20.8562	0.680142
Ciclev10031680m.g	scaffold_4:24020031-24023152	6.00584	9.62614	0.680593
Ciclev10015011m.g	scaffold_2:6537469-6541198	11.0961	17.7901	0.681019
Ciclev10005312m.g	scaffold_9:27918487-27923869	4.565	7.31949	0.681129
Ciclev10014254m.g	scaffold_2:8654457-8657680	3.03724	4.87011	0.681194
-	scaffold_1:1505298-1507335	70.9283	113.731	0.681197
Ciclev10008445m.g	scaffold_1:22902019-22904804	24.7576	39.7023	0.681348
Ciclev10013074m.g	scaffold_6:18556596-18557257	0.418311	0.670834	0.681379
Ciclev10001314m.g	scaffold_5:20894676-20899217	51.083	81.929	0.681533
Ciclev10023782m.g	scaffold_3:40078102-40089151	129.735	208.081	0.681579
Ciclev10011597m.g	scaffold_6:23624401-23627433	14.9093	23.913	0.681588

Ciclev10025141m.g	scaffold_7:1929223-1934021	6.36931	10.2173	0.681801
-	scaffold_2:5888550-5888804	54.1893	86.929	0.681828
Ciclev10031278m.g	scaffold_4:2051777-2055131	2.10306	3.37415	0.682035
Ciclev10015979m.g,Ciclev10017686m.g	scaffold_2:10058915-10097079	4.01027	6.43411	0.68204
Ciclev10005694m.g	scaffold_9:20360609-20494992	0.67293	1.0798	0.682235
Ciclev10005394m.g	scaffold_9:5549194-5553148	148.239	237.894	0.682391
Ciclev10031423m.g	scaffold_4:25183939-25190088	28.4776	45.7033	0.68247
Ciclev10007348m.g	scaffold_1:2990296-2996152	22.1124	35.4879	0.682473
Ciclev10007779m.g	scaffold_1:19354869-19358092	1.77491	2.84857	0.682492
Ciclev10025269m.g	scaffold_7:20846564-20853370	16.607	26.6544	0.682578
Ciclev10017765m.g	scaffold_2:25788936-25790076	0.442366	0.710005	0.68259
Ciclev10010396m.g	scaffold_1:28203081-28206498	2.87497	4.61484	0.682734
Ciclev10032023m.g	scaffold_4:7931032-7933167	8.4651	13.5908	0.68303
Ciclev10015139m.g	scaffold_2:34993441-34996954	15.7462	25.2812	0.683059
Ciclev10030360m.g	scaffold_8:781943-785484	0.0713292	0.114535	0.683223
Ciclev10025908m.g	scaffold_7:19005317-19007848	11.2893	18.1301	0.683429
-	scaffold_2:25878315-25878585	53.5282	85.9863	0.683808
Ciclev10033548m.g	scaffold_4:20251442-20253042	16.2578	26.1191	0.683975
Ciclev10005969m.g	scaffold_9:21029932-21033617	14.9096	23.9569	0.684202
Ciclev10025492m.g	scaffold_7:1518261-1522227	17.8811	28.7343	0.684343
Ciclev10017936m.g	scaffold_2:11316154-11320354	8.41798	13.5285	0.684457
Ciclev10029746m.g	scaffold_8:23143377-23146210	0.515797	0.829049	0.684654
Ciclev10011320m.g	scaffold_6:12628264-12630982	9.02499	14.5076	0.684814
Ciclev10027679m.g	scaffold_8:24392781-24402366	0.448819	0.721506	0.684878
Ciclev10004422m.g	scaffold_9:27924711-27929149	0.423772	0.681394	0.685202
Ciclev10027678m.g	scaffold_8:513651-520566	6.62306	10.6528	0.685657
Ciclev10010384m.g	scaffold_1:22016233-22020444	6.42233	10.3306	0.685758
Ciclev10000627m.g	scaffold_5:38669122-38672110	15.8765	25.5433	0.686054
Ciclev10007743m.g	scaffold_1:14395658-14402022	8.5272	13.7203	0.686166
Ciclev10020031m.g	scaffold_3:43175874-43177503	0.364087	0.585839	0.686221
Ciclev10009451m.g	scaffold_1:25417503-25419185	83.3434	134.106	0.686229
Ciclev10009201m.g	scaffold_1:4278094-4278940	0.397839	0.640413	0.686817
Ciclev10030624m.g	scaffold_4:18483872-18493963	54.94	88.4403	0.686845
Ciclev10018533m.g	scaffold_3:38552143-38556787	7.4037	11.9188	0.686918
Ciclev10008389m.g	scaffold_1:17370075-17377980	24.5901	39.5961	0.687282
Ciclev10025724m.g	scaffold_7:6747138-6750595	98.8663	159.215	0.687427
Ciclev10021100m.g	scaffold_3:8605286-8607326	25.1419	40.491	0.687505
Ciclev10008825m.g	scaffold_1:28124582-28127463	13.3079	21.4329	0.687549
Ciclev10011000m.g	scaffold_6:24483900-24490316	53.8521	86.7683	0.688166
Ciclev10027839m.g	scaffold_8:22248128-22254029	16.1991	26.1011	0.688199
Ciclev10017030m.g	scaffold_2:25761441-25762855	22.6697	36.5306	0.688341
Ciclev10019189m.g	scaffold_3:508373-511381	59.7016	96.2166	0.688516
Ciclev10002011m.g	scaffold_5:33416340-33422684	18.3887	29.6398	0.688717
Ciclev10021679m.g	scaffold_3:38139683-38141470	5.02382	8.10043	0.689214
Ciclev10030402m.g	scaffold_8:2266683-2269143	8.01077	12.9169	0.689248
Ciclev10016313m.g	scaffold_2:17813409-17818989	3.40533	5.49095	0.68926
Ciclev10031530m.g	scaffold_4:25378567-25383686	35.2995	56.919	0.689263
Ciclev10016941m.g	scaffold_2:29876139-29876970	4.50303	7.26212	0.689495
Ciclev10024676m.g	scaffold_7:6834675-6869445	4.86927	7.85299	0.689536
Ciclev10002417m.g	scaffold_5:39818783-39820264	77.7705	125.452	0.689837
Ciclev10014242m.g	scaffold_2:27533370-27540184	20.9135	33.736	0.689857
Ciclev10023412m.g	scaffold_3:27206698-27208390	29.6056	47.7635	0.69004
Ciclev10026234m.g	scaffold_7:14258623-14259635	1.53015	2.46924	0.690395
Ciclev10007989m.g	scaffold_1:2918530-2922318	13.2827	21.4355	0.690451
Ciclev10028480m.g	scaffold_8:14571201-14575430	42.9453	69.3117	0.690598

Ciclev10006249m.g	scaffold_9:5412307-5413638	12.1301	19.5775	0.690607
Ciclev10015592m.g	scaffold_2:32842061-32845829	7.39554	11.939	0.69095
Ciclev10019930m.g	scaffold_3:6999316-7003137	1.89726	3.06317	0.691108
Ciclev10028321m.g	scaffold_8:727650-733760	28.4529	45.9385	0.691129
-	scaffold_8:17291958-17467945	1.18682	1.91622	0.691167
Ciclev10015906m.g	scaffold_2:29118021-29123497	6.17649	9.97394	0.691377
Ciclev10014349m.g	scaffold_2:10844444-10847804	0.422693	0.682704	0.691651
Ciclev10018664m.g	scaffold_3:146901-154107	17.0129	27.4836	0.691944
Ciclev10014471m.g	scaffold_2:11418330-11422243	700.36	1131.47	0.692028
Ciclev10014676m.g	scaffold_2:9932490-9936598	3.75803	6.0744	0.692765
Ciclev10004642m.g,Ciclev10007040m.g	scaffold_9:15404452-15414798	18.3908	29.7275	0.692812
Ciclev10021146m.g	scaffold_3:1058011-1060668	32.9971	53.3589	0.69339
Ciclev10018563m.g	scaffold_3:49184284-49194926	37.1304	60.0587	0.693772
Ciclev10011611m.g	scaffold_6:24493971-24497469	8.60695	13.9282	0.694431
Ciclev10023302m.g	scaffold_3:48576216-48576693	3.82448	6.18906	0.694457
Ciclev10018814m.g	scaffold_3:688263-692703	20.2293	32.7466	0.694902
Ciclev10022648m.g	scaffold_3:6332714-6333228	0.632785	1.02434	0.694903
Ciclev10021149m.g	scaffold_3:1925788-1928534	2.44064	3.95099	0.694955
Ciclev10003671m.g	scaffold_5:41463512-41466022	1.19327	1.93174	0.694986
Ciclev10033885m.g	scaffold_4:15072503-15079709	4.55444	7.37384	0.695144
Ciclev10014097m.g	scaffold_2:379575-388226	17.2909	27.9977	0.695297
Ciclev10006451m.g	scaffold_9:26349056-26350658	7.29729	11.8178	0.695534
Ciclev10002080m.g	scaffold_5:41000993-41003992	39.1762	63.4457	0.695542
Ciclev10020194m.g	scaffold_3:942639-947359	23.4959	38.0515	0.695546
Ciclev10024595m.g	scaffold_3:7429295-7431389	0.447156	0.724282	0.695774
Ciclev10007786m.g	scaffold_1:24351536-24353944	4.70326	7.61998	0.696125
Ciclev10015430m.g	scaffold_2:29223571-29227229	1.22234	1.98052	0.696235
Ciclev10030083m.g	scaffold_8:5686873-5689988	0.198621	0.321856	0.696394
-	scaffold_2:35023363-35024196	10.4817	16.9874	0.696588
Ciclev10007375m.g	scaffold_1:28458305-28465466	31.4527	51.0002	0.697319
Ciclev10004790m.g	scaffold_9:31237088-31243866	11.4542	18.5735	0.697373
Ciclev10024755m.g	scaffold_7:5144667-5148251	0.0376577	0.0610665	0.697437
Ciclev10004303m.g	scaffold_9:19375410-19379547	0.0647923	0.105069	0.697445
Ciclev10014092m.g	scaffold_2:34780710-34787054	15.2841	24.7871	0.697562
Ciclev10028950m.g	scaffold_8:4028427-4030228	13.1831	21.3808	0.697625
Ciclev10020631m.g	scaffold_3:41955539-41957174	0.266414	0.43214	0.697829
Ciclev10018560m.g,Ciclev10024479m.g	scaffold_3:43011640-43045787	0.976602	1.58417	0.697885
Ciclev10004631m.g	scaffold_9:6880200-6882349	2.27989	3.69828	0.69789
Ciclev10010904m.g	scaffold_6:25133890-25139948	3.38233	5.48732	0.698087
Ciclev10024770m.g	scaffold_7:15893931-15916079	15.1214	24.5332	0.698141
Ciclev10030697m.g	scaffold_4:18965977-18968515	0.49682	0.806203	0.69842
Ciclev10000789m.g	scaffold_5:42286704-42288426	7.99877	12.9806	0.698511
Ciclev10007134m.g	scaffold_9:11576287-11715575	14.2935	23.2021	0.698897
Ciclev10019818m.g	scaffold_3:47116200-47119175	0.206727	0.335605	0.699033
Ciclev10024047m.g	scaffold_3:50112783-50114181	0.13861	0.225072	0.699354
Ciclev10000172m.g	scaffold_5:42460203-42468795	28.5178	46.3106	0.699481
Ciclev10016532m.g	scaffold_2:5220775-5225631	7.36688	11.9635	0.699518
Ciclev10031874m.g	scaffold_4:14610896-14614381	13.1802	21.4053	0.699597
Ciclev10023181m.g	scaffold_3:3919655-3924283	0.471569	0.766025	0.699922
-	scaffold_5:2297541-2300841	3.0343	4.92931	0.700023
Ciclev10004539m.g	scaffold_9:28361004-28369518	31.0258	50.4084	0.700197
Ciclev10001840m.g	scaffold_5:101796-108313	24.78	40.2766	0.700765
Ciclev10001518m.g	scaffold_5:22691011-22694289	25.4804	41.4258	0.701142

Ciclev10010899m.g,Ciclev10011209m.g	scaffold_6:17143868-17153388	15.2883	24.8565	0.701193
Ciclev10028832m.g	scaffold_8:17840220-17841556	7.94306	12.9182	0.701637
-	scaffold_6:20361868-20362191	6.05311	9.84447	0.701638
Ciclev10014743m.g	scaffold_2:9603289-9607349	10.7778	17.5367	0.702318
Ciclev10018691m.g	scaffold_3:34696660-34702240	11.109	18.0805	0.702702
Ciclev10030924m.g	scaffold_4:25366523-25370662	0.0871458	0.14186	0.702968
Ciclev10018474m.g	scaffold_3:2829629-2837378	14.1531	23.0397	0.703007
Ciclev10024095m.g	scaffold_3:42188413-42192854	1.80305	2.93559	0.703206
Ciclev10032213m.g	scaffold_4:23564250-23568956	243.101	395.983	0.703881
Ciclev10004865m.g	scaffold_9:20187043-20192121	0.10922	0.177936	0.704126
Ciclev10021235m.g	scaffold_3:45019199-45020690	6.53244	10.644	0.704342
Ciclev10017324m.g,Ciclev10017638m.g	scaffold_2:27587031-27587739	23.0434	37.5512	0.704503
Ciclev10028281m.g	scaffold_8:4137235-4139924	5.82688	9.4957	0.70455
Ciclev10009041m.g	scaffold_1:1867416-1870869	30.9812	50.5027	0.704965
Ciclev10030907m.g	scaffold_4:12263754-12266130	80.3617	131.007	0.705065
Ciclev10027875m.g	scaffold_8:17062550-17066112	8.41567	13.7209	0.705229
Ciclev10018904m.g	scaffold_3:2137776-2141308	5.23371	8.53348	0.705299
Ciclev10008806m.g	scaffold_1:17175698-17178249	0.569102	0.92805	0.705516
Ciclev10011739m.g	scaffold_6:25437607-25442326	12.5856	20.528	0.705816
Ciclev10006449m.g	scaffold_9:3771161-3771725	51.9551	84.7422	0.705816
Ciclev10023274m.g	scaffold_3:29315277-29322012	15.5092	25.2995	0.70598
Ciclev10018948m.g	scaffold_3:16208637-16228612	13.3635	21.8116	0.706794
Ciclev10002554m.g	scaffold_5:21581030-21583751	22.8767	37.349	0.707194
Ciclev10015714m.g	scaffold_2:7966422-7974085	7.19608	11.7528	0.707727
Ciclev10024974m.g	scaffold_7:20548587-20557694	1.59081	2.59865	0.708003
Ciclev10028010m.g,Ciclev10029238m.g	scaffold_8:3203192-3225772	19.6705	32.1337	0.708052
Ciclev10026382m.g,Ciclev10027183m.g	scaffold_7:21063926-21073964	12.8765	21.041	0.708461
Ciclev10029503m.g	scaffold_8:18125763-18128306	4.60389	7.52518	0.708873
Ciclev10023539m.g	scaffold_3:18795601-18797524	1.73922	2.84332	0.70914
Ciclev10015214m.g	scaffold_2:9198524-9203327	132.944	217.376	0.709372
Ciclev10011490m.g	scaffold_6:16075912-16078971	0.119145	0.194857	0.709697
Ciclev10012051m.g	scaffold_6:24521777-24523284	0.396516	0.648581	0.709906
Ciclev10002435m.g	scaffold_5:29398393-29399900	0.937109	1.533	0.710071
Ciclev10021257m.g	scaffold_3:43844423-43848613	8.02728	13.1319	0.710093
Ciclev10014585m.g,Ciclev10014598m.g	scaffold_2:4864393-4884787	16.036	26.2434	0.710638
Ciclev10020490m.g	scaffold_3:32716415-32805364	0.193265	0.316421	0.711268
Ciclev10002766m.g	scaffold_5:41154410-41155747	15.6509	25.6319	0.7117
Ciclev10014638m.g	scaffold_2:36165467-36169473	24.3614	39.8977	0.711707
Ciclev10006290m.g	scaffold_9:4835330-4836034	65.7562	107.711	0.711962
Ciclev10030999m.g	scaffold_4:23267087-23269311	21.8011	35.7114	0.711984
Ciclev10014180m.g	scaffold_2:8470087-8483205	14.028	22.9787	0.711991
Ciclev10025660m.g	scaffold_7:17208074-17211871	23.2326	38.058	0.71205
Ciclev10011792m.g	scaffold_6:22244923-22252231	95.7052	156.787	0.712139
Ciclev10022219m.g	scaffold_3:4736046-4740432	7.93806	13.0054	0.712249
Ciclev10016144m.g	scaffold_2:32447502-32450246	21.5572	35.3302	0.712733
Ciclev10015774m.g	scaffold_2:30321627-30324378	9.64149	15.8052	0.713072
-	scaffold_7:16519771-16523572	18.636	30.5509	0.71312
Ciclev10007395m.g	scaffold_1:26327609-26336293	5.86562	9.62134	0.713954
Ciclev10020547m.g	scaffold_3:36311966-36313263	0.136324	0.223626	0.714048
Ciclev10007304m.g	scaffold_1:21918725-21922966	6.58393	10.8029	0.714397
Ciclev10024232m.g	scaffold_3:24991888-24992787	0.547043	0.897592	0.714406

Ciclev10025341m.g	scaffold_7:3940273-3943841	66.3283	108.847	0.714604
Ciclev10027468m.g	scaffold_7:16878929-16882179	11.025	18.097	0.714971
Ciclev10004121m.g	scaffold_9:16068264-16086556	3.49989	5.74644	0.71536
Ciclev10012040m.g	scaffold_6:14754809-14757059	1.94737	3.19858	0.715902
Ciclev10023144m.g	scaffold_3:13318416-13324494	0.210156	0.345213	0.716029
Ciclev10002228m.g	scaffold_5:38275765-38277786	50.4576	82.8858	0.716053
Ciclev10025826m.g	scaffold_7:15347527-15349769	2.5841	4.24528	0.716199
Ciclev10004615m.g	scaffold_9:27242397-27245984	47.5061	78.0848	0.716929
Ciclev10024779m.g	scaffold_7:7729685-7737595	9.73428	16.001	0.717019
Ciclev10001637m.g	scaffold_5:33988528-33991585	1.11348	1.83038	0.717071
Ciclev10028433m.g	scaffold_8:17514422-17519410	6.06475	9.97037	0.7172
Ciclev10013878m.g	scaffold_6:16955859-16960624	5.61898	9.23766	0.717219
Ciclev10033734m.g	scaffold_4:19138974-19140711	7.95311	13.0776	0.717507
Ciclev10000861m.g	scaffold_5:36475617-36479093	4.02567	6.62124	0.71787
Ciclev10007953m.g	scaffold_1:25128983-25131787	7.08381	11.6514	0.717906
Ciclev10009756m.g	scaffold_1:25592493-25593617	58.8121	96.7398	0.717995
Ciclev10001055m.g	scaffold_5:34732987-34754900	16.498	27.1438	0.718329
Ciclev10009739m.g	scaffold_1:14668286-14669509	0.149212	0.245502	0.718372
Ciclev10001572m.g	scaffold_5:38685604-38688349	1.12906	1.85799	0.718613
Ciclev10011575m.g	scaffold_6:24505956-24509682	24.9353	41.0373	0.718748
Ciclev10017215m.g	scaffold_2:278578-279559	1.85563	3.05425	0.71891
Ciclev10029310m.g,Ciclev10029975m.g	scaffold_8:2191754-2204285	29.3502	48.3113	0.718991
Ciclev10008617m.g	scaffold_1:4143947-4146608	88.4901	145.661	0.71903
Ciclev10014260m.g	scaffold_2:25765570-25768934	0.776946	1.27904	0.719174
Ciclev10002974m.g	scaffold_5:29890725-29892233	1.48087	2.43827	0.719415
Ciclev10018902m.g	scaffold_3:5038746-5181239	0.625116	1.02936	0.719553
Ciclev10027700m.g	scaffold_8:17775097-17791072	17.6052	28.993	0.719703
Ciclev10001303m.g	scaffold_5:39967383-39969998	11.7451	19.3452	0.719926
Ciclev10011996m.g	scaffold_6:23825288-23827871	21.5735	35.5341	0.719941
Ciclev10030406m.g	scaffold_8:4032244-4034182	1.89653	3.12385	0.719961
Ciclev10012598m.g	scaffold_6:22807157-22810575	5.89313	9.70684	0.719967
-	scaffold_8:4995299-4996027	387.159	637.766	0.720102
Ciclev10031662m.g	scaffold_4:24457157-24462630	58.3777	96.1776	0.720284
Ciclev10002594m.g,Ciclev10003435m.g	scaffold_5:31411457-31441415	18.0095	29.6854	0.720991
Ciclev10010892m.g	scaffold_6:21099714-21111518	2.66228	4.38838	0.721028
Ciclev10020891m.g	scaffold_3:30469587-30471790	0.18502	0.304992	0.721094
Ciclev10031140m.g	scaffold_4:5315821-5319339	7.08993	11.689	0.721305
Ciclev10001357m.g	scaffold_5:6777321-6914637	5.47328	9.02408	0.721374
Ciclev10019962m.g	scaffold_3:49017414-49020095	0.203571	0.3358	0.72207
Ciclev10032221m.g	scaffold_4:18905503-18908874	82.1602	135.56	0.722423
Ciclev10005602m.g	scaffold_9:31220968-31224037	5.70657	9.41634	0.722541
Ciclev10014287m.g	scaffold_2:28131618-28137663	19.3858	31.9925	0.722736
Ciclev10027108m.g	scaffold_7:923460-926611	1260.5	2080.33	0.722818
Ciclev10005267m.g	scaffold_9:15072515-15074021	3.50444	5.78392	0.722865
Ciclev10029147m.g	scaffold_8:3046971-3051208	2.42802	4.00882	0.723401
Ciclev10009147m.g	scaffold_1:1993966-1994803	11.2866	18.6352	0.723425
Ciclev10018651m.g	scaffold_3:2270815-2274196	7.7913	12.8723	0.724331
Ciclev10010146m.g	scaffold_1:8588917-8599322	6.95197	11.4857	0.724343
Ciclev10015115m.g	scaffold_2:4809974-4813781	6.15484	10.1687	0.724348
Ciclev10019318m.g	scaffold_3:31421115-31429564	7.04134	11.6356	0.724625
Ciclev10002869m.g	scaffold_5:441330-538185	10.0652	16.6337	0.724734
Ciclev10015153m.g	scaffold_2:14442706-14449383	33.7158	55.7225	0.724834
Ciclev10001423m.g	scaffold_5:36058138-36063798	2.14922	3.55215	0.724874
Ciclev10000930m.g	scaffold_5:39613356-39615571	5.86329	9.69415	0.725404

Ciclev10004309m.g	scaffold_9:30513332-30517676	140.497	232.34	0.725703
Ciclev10014599m.g	scaffold_2:28745069-28749655	6.08811	10.0697	0.725954
Ciclev10014993m.g	scaffold_2:30380995-30382655	3.58326	5.93012	0.726789
Ciclev10027520m.g	scaffold_7:3898895-3902656	2.14689	3.5539	0.727151
Ciclev10011726m.g	scaffold_6:21137782-21142157	228.774	378.734	0.727263
Ciclev10008579m.g	scaffold_1:11230972-11238035	22.6058	37.4305	0.727519
Ciclev10002425m.g	scaffold_5:42093830-42095266	17.8465	29.5542	0.72772
Ciclev10010674m.g	scaffold_1:11543995-11547390	24.8787	41.2093	0.728057
Ciclev10031708m.g	scaffold_4:20749812-20753994	15.4087	25.5243	0.728122
Ciclev10019063m.g	scaffold_3:29908722-29911090	0.795089	1.31739	0.728498
Ciclev10010489m.g	scaffold_1:5085969-5088975	0.085653	0.141922	0.728523
Ciclev10023696m.g	scaffold_3:36363008-36553812	1.15734	1.9181	0.728868
Ciclev10025308m.g	scaffold_7:6764663-6770188	22.5384	37.3548	0.728908
Ciclev10007342m.g	scaffold_1:28821462-28827153	19.7781	32.7808	0.728943
Ciclev10031497m.g	scaffold_4:3916341-3921305	10.0957	16.7381	0.729397
Ciclev10015399m.g	scaffold_2:32742079-32746165	6.603	10.9487	0.729568
Ciclev10025185m.g	scaffold_7:8814827-8833655	14.2672	23.6612	0.729821
Ciclev10014699m.g	scaffold_2:9690543-9696681	25.8789	42.9324	0.730292
Ciclev10033985m.g	scaffold_4:17980243-17981153	0.662032	1.09842	0.730462
Ciclev10005134m.g,Ciclev10006837m.g,Ciclev10006973m.g	scaffold_9:2467026-2478719	53.7388	89.1687	0.730573
-	scaffold_6:19209254-19209800	2.51572	4.17521	0.730878
Ciclev10020181m.g	scaffold_3:10425914-10435293	13.9422	23.1419	0.731053
Ciclev10030580m.g	scaffold_4:19867285-19877521	65.7655	109.194	0.731491
Ciclev10004416m.g	scaffold_9:15158761-15163277	2.39386	3.97509	0.731649
Ciclev10007633m.g	scaffold_1:23814114-23817728	179.37	297.862	0.731706
Ciclev10032070m.g	scaffold_4:22584763-22588013	14.2181	23.6114	0.731757
Ciclev10022342m.g	scaffold_3:40267364-40273231	8.81892	14.6505	0.732274
Ciclev10005347m.g	scaffold_9:28556662-28560571	49.0247	81.4526	0.732453
Ciclev10028224m.g	scaffold_8:24965433-24970341	4.33928	7.21236	0.733016
Ciclev10005542m.g	scaffold_9:2115912-2118875	4.77093	7.93061	0.73316
Ciclev10031856m.g	scaffold_4:1215359-1218773	10.3921	17.2787	0.7335
Ciclev10025941m.g	scaffold_7:5388457-5389981	17.0118	28.2995	0.734235
Ciclev10028316m.g,Ciclev10028368m.g,Ciclev10029981m.g	scaffold_8:18973864-19114160	8.25944	13.7446	0.734746
Ciclev10011787m.g	scaffold_6:22360103-22364856	14.3265	23.841	0.734759
Ciclev10031528m.g	scaffold_4:5049339-5050811	0.132976	0.221363	0.735246
Ciclev10032639m.g	scaffold_4:23938841-23939939	38.6602	64.3589	0.73529
Ciclev10007963m.g	scaffold_1:14984379-14986585	32.4382	54.0019	0.735317
Ciclev10028981m.g	scaffold_8:24282482-24287657	27.7865	46.2665	0.735584
Ciclev10007268m.g	scaffold_1:24338301-24342997	2.66745	4.44222	0.735819
Ciclev10010012m.g	scaffold_1:22831846-22836046	10.2921	17.1399	0.73582
Ciclev10025463m.g	scaffold_7:5412236-5416674	0.368453	0.613871	0.736454
-	scaffold_6:334211-335003	6.91495	11.5221	0.736617
Ciclev10029878m.g	scaffold_8:4292179-4375670	0.123655	0.20605	0.736678
Ciclev10014339m.g	scaffold_2:36354214-36359772	4.15077	6.91741	0.736851
Ciclev10002835m.g	scaffold_5:41014863-41017027	1.59573	2.661	0.737746
Ciclev10010299m.g	scaffold_1:28637410-28642498	0.167825	0.279983	0.738382
Ciclev10024067m.g	scaffold_3:39452082-39453426	0.158873	0.265092	0.73862
Ciclev10028558m.g	scaffold_8:24851461-24855408	9.77744	16.3152	0.738686
Ciclev10028570m.g	scaffold_8:23489809-23493165	11.2721	18.8098	0.738726
Ciclev10000828m.g	scaffold_5:39525922-39529412	246.289	411.136	0.739264
Ciclev10012675m.g	scaffold_6:18354933-18359995	6.55793	10.9479	0.739343
Ciclev10019137m.g	scaffold_3:48864102-48867563	2.94052	4.91185	0.740193

-	scaffold_5:29107252-29107709	9.642	16.1067	0.740259
Ciclev10011980m.g	scaffold_6:20638048-20641557	9.8991	16.5401	0.740602
Ciclev10010923m.g	scaffold_6:10036059-10044395	24.6362	41.1789	0.741127
Ciclev10008387m.g	scaffold_1:17263260-17265543	0.30523	0.51033	0.741535
Ciclev10018128m.g	scaffold_2:20973562-20973913	3.03012	5.06678	0.741697
Ciclev10006599m.g	scaffold_9:3847284-3848066	1.8845	3.15247	0.742305
Ciclev10012311m.g	scaffold_6:7876588-7879960	0.268176	0.448633	0.742352
Ciclev10031829m.g	scaffold_4:18274170-18276675	1.21053	2.02513	0.742376
Ciclev10004258m.g	scaffold_9:1102132-1105709	0.0562133	0.0940452	0.742443
Ciclev10011340m.g	scaffold_6:22178322-22180545	0.201191	0.336632	0.742611
Ciclev10019676m.g,Ciclev10021101m.g	scaffold_3:1806576-1812029	13.0973	21.916	0.742717
Ciclev10004601m.g	scaffold_9:18837088-18844701	6.906	11.5578	0.742949
Ciclev10031604m.g	scaffold_4:16163636-16165371	7.8682	13.1686	0.742997
Ciclev10011900m.g	scaffold_6:18798767-18804051	32.4359	54.2884	0.743053
Ciclev10003112m.g	scaffold_5:16138325-16152068	0.134949	0.225875	0.74311
Ciclev10017376m.g	scaffold_2:22750224-22751793	0.304806	0.510184	0.743128
Ciclev10031607m.g	scaffold_4:24716951-24718896	0.112944	0.18915	0.74392
Ciclev10015535m.g	scaffold_2:11863905-11865549	47.1199	78.9281	0.744202
Ciclev10014196m.g,Ciclev10014274m.g	scaffold_2:30325131-30365011	21.3806	35.8161	0.744305
Ciclev10027659m.g	scaffold_8:3695023-3708840	0.0333164	0.0558155	0.744433
Ciclev10014738m.g	scaffold_2:31916439-31920491	71.1389	119.237	0.745117
Ciclev10004137m.g	scaffold_9:20009637-20033079	127.881	214.357	0.74522
Ciclev10026987m.g	scaffold_7:11115706-11117335	2.24189	3.75863	0.745493
Ciclev10029251m.g	scaffold_8:16581037-16582936	26.5727	44.5539	0.745609
Ciclev10030588m.g	scaffold_4:671841-679060	25.6021	42.9367	0.745948
Ciclev10032158m.g	scaffold_4:18413040-18422725	14.2166	23.8427	0.745968
Ciclev10024600m.g	scaffold_3:1629001-1629517	40.0817	67.2281	0.746121
Ciclev10014686m.g	scaffold_2:27543760-27548100	15.2117	25.5163	0.746237
Ciclev10012033m.g	scaffold_6:8413958-8415516	0.141252	0.236976	0.746471
Ciclev10018314m.g	scaffold_2:28489379-28492995	1.18302	1.98514	0.746758
Ciclev10014385m.g	scaffold_2:13959189-13963622	1.27219	2.13549	0.747255
Ciclev10018361m.g	scaffold_2:9624931-9626743	1.94015	3.257	0.747378
Ciclev10008096m.g	scaffold_1:5067103-5069631	6.08787	10.2202	0.747418
Ciclev10014313m.g	scaffold_2:32428284-32433624	0.11709	0.196583	0.747526
Ciclev10019346m.g	scaffold_3:47605941-47609861	315.183	529.259	0.747783
Ciclev10011696m.g,Ciclev10013565m.g	scaffold_6:1220295-1325561	8.77636	14.7396	0.748002
Ciclev10019093m.g	scaffold_3:4379279-4381458	1.54877	2.60141	0.748169
Ciclev10020731m.g	scaffold_3:3802386-3805494	156.526	263.001	0.748668
Ciclev10021717m.g	scaffold_3:48816184-48819252	10.7751	18.1081	0.748931
Ciclev10004588m.g	scaffold_9:22318671-22320880	25.7882	43.3396	0.748974
Ciclev10028626m.g	scaffold_8:22127138-22129495	24.3257	40.8908	0.749296
Ciclev10014704m.g	scaffold_2:30259485-30263334	3.09768	5.20867	0.749727
Ciclev10012768m.g	scaffold_6:19959037-19960378	94.737	159.331	0.750029
Ciclev10014223m.g	scaffold_2:6851264-6854086	0.0624305	0.105016	0.750292
Ciclev10001273m.g,Ciclev10001713m.g	scaffold_5:29602912-29636069	31.8549	53.5886	0.750412
Ciclev10022924m.g	scaffold_3:5628820-5629717	3.92151	6.59826	0.750678
Ciclev10007727m.g	scaffold_1:28403180-28407070	12.5921	21.1934	0.751095
Ciclev10029114m.g	scaffold_8:18168885-18171974	7.44115	12.5274	0.751489
Ciclev10025881m.g	scaffold_7:8378641-8381156	25.7432	43.3535	0.751956
Ciclev10016452m.g	scaffold_2:2982880-2985750	11.6534	19.6264	0.752047
Ciclev10014450m.g	scaffold_2:28732940-28736344	11.8246	19.9152	0.752084
Ciclev10028249m.g	scaffold_8:23285993-23288157	14.9003	25.0975	0.752206

Ciclev10007624m.g	scaffold_1:6439717-6444107	4.04604	6.81712	0.752654
Ciclev10025177m.g	scaffold_7:12297338-12301611	0.0907764	0.152958	0.752746
Ciclev10011473m.g	scaffold_6:15803863-15809998	31.401	52.9112	0.752763
Ciclev10005609m.g	scaffold_9:8509471-8512375	29.6541	49.9776	0.753048
Ciclev10010991m.g	scaffold_6:25193616-25201413	76.7911	129.426	0.753112
Ciclev10024323m.g	scaffold_3:45520965-45524742	0.152958	0.25785	0.7534
Ciclev10007465m.g	scaffold_1:25405167-25413254	4.91249	8.28255	0.75362
Ciclev10006903m.g	scaffold_9:25744567-25747429	0.123951	0.209011	0.753806
Ciclev10021550m.g	scaffold_3:2522582-2524737	19.4195	32.7504	0.754009
Ciclev10018487m.g	scaffold_3:16816356-16822991	0.196423	0.331268	0.754033
Ciclev10012867m.g	scaffold_6:24689085-24690862	12.7454	21.5066	0.75481
Ciclev10001672m.g	scaffold_5:8503780-8508594	16.2064	27.3537	0.755171
Ciclev10007188m.g	scaffold_9:29460865-29463385	2.98564	5.03945	0.755227
Ciclev10015886m.g	scaffold_2:29270207-29274050	12.412	20.9532	0.755436
Ciclev10010366m.g	scaffold_1:943321-945735	0.297825	0.50287	0.755718
Ciclev10014281m.g	scaffold_2:20370848-20373709	18.1437	30.6353	0.755727
Ciclev10023246m.g	scaffold_3:43260726-43272903	36.2841	61.2755	0.755971
Ciclev10030097m.g	scaffold_8:24302666-24304208	0.714822	1.20735	0.756186
Ciclev10006431m.g	scaffold_9:27352942-27354379	0.166319	0.280967	0.756452
Ciclev10007324m.g	scaffold_1:23147213-23156455	12.7347	21.5206	0.756948
Ciclev10000175m.g	scaffold_5:40348116-40353953	4.09358	6.91859	0.757115
Ciclev10000267m.g	scaffold_5:18229172-18239197	11.1056	18.7743	0.757471
Ciclev10019115m.g	scaffold_3:2442100-2445875	6.3534	10.7416	0.757605
Ciclev10007219m.g	scaffold_1:23527052-23542559	16.7471	28.3155	0.757676
Ciclev10011480m.g	scaffold_6:16903667-16906771	28.6715	48.479	0.757744
Ciclev10012779m.g	scaffold_6:19405284-19407278	155.206	262.476	0.758
Ciclev10029446m.g	scaffold_8:6325946-6328971	10.3282	17.4674	0.75807
-	scaffold_1:21476759-21547573	31.748	53.7324	0.759129
Ciclev10008489m.g	scaffold_1:6494972-6502196	12.6762	21.4549	0.759178
Ciclev10019613m.g	scaffold_3:3742475-3744508	0.099304	0.168116	0.759536
Ciclev10032576m.g	scaffold_4:6891394-6897982	16.3421	27.6704	0.759749
Ciclev10031939m.g	scaffold_4:18732269-18736444	45.0892	76.409	0.760963
Ciclev10015284m.g	scaffold_2:34107231-34111569	1.27182	2.15574	0.761286
Ciclev10019403m.g	scaffold_3:42193700-42198857	4.71661	7.99469	0.761292
Ciclev10001454m.g	scaffold_5:38829626-38855263	51.2244	86.8284	0.761335
Ciclev10027769m.g	scaffold_8:13408609-13413938	0.0572901	0.0971274	0.761592
Ciclev10019950m.g	scaffold_3:20877974-20880980	2.13335	3.61746	0.761858
Ciclev10016381m.g	scaffold_2:21340421-21343123	13.1787	22.348	0.761935
Ciclev10018208m.g	scaffold_2:11589345-11590297	1.10729	1.87771	0.761943
Ciclev10011167m.g	scaffold_6:12478047-12481063	7.54721	12.801	0.762244
Ciclev10004239m.g	scaffold_9:15055712-15063318	27.5686	46.7625	0.762326
Ciclev10010321m.g,Ciclev10010664m.g	scaffold_1:19721667-19778009	1.83017	3.10586	0.763015
Ciclev10006126m.g	scaffold_9:7229653-7232816	16.6158	28.2022	0.763251
Ciclev10000502m.g	scaffold_5:5323875-5327356	2.54702	4.32339	0.763351
Ciclev10005079m.g	scaffold_9:2033330-2036879	9.02024	15.3135	0.763568
Ciclev10020238m.g	scaffold_3:4226717-4231540	6.49116	11.0204	0.763633
Ciclev10005965m.g,Ciclev10006980m.g	scaffold_9:29198324-29207977	15.4815	26.296	0.764302
Ciclev10014393m.g	scaffold_2:5115870-5120839	7.19238	12.2195	0.76464
Ciclev10018469m.g	scaffold_3:7719436-7742996	2.64748	4.49828	0.764755
Ciclev10000448m.g	scaffold_5:17103752-17105870	1.35145	2.29644	0.764891
Ciclev10026886m.g	scaffold_7:5554618-5556993	0.317975	0.540439	0.765219
Ciclev10006542m.g	scaffold_9:31171508-31199360	7.41718	12.6147	0.766159
Ciclev10015958m.g	scaffold_2:30586459-30587943	4.52568	7.70014	0.766752
Ciclev10025039m.g	scaffold_7:8333497-8339140	33.658	57.2953	0.767466

Ciclev10012894m.g	scaffold_6:15553484-15554490	16.5958	28.255	0.767688
Ciclev10023973m.g	scaffold_3:4203406-4210711	0.228635	0.389319	0.767906
Ciclev10015820m.g,Ciclev10016621m.g	scaffold_2:10424778-10472483	8.48096	14.4428	0.768053
Ciclev10012772m.g	scaffold_6:19527908-19529725	11.6861	19.9021	0.76813
Ciclev10000581m.g,Ciclev10000653m.g,Ciclev10003350m.g,Ciclev10003850m.g	scaffold_5:32017860-32153975	21.5039	36.6232	0.768159
Ciclev10002602m.g	scaffold_5:29803195-29804310	3.94751	6.72435	0.768452
Ciclev10027257m.g	scaffold_7:14677798-14683109	0.0822648	0.14017	0.768826
Ciclev10000617m.g	scaffold_5:24110460-24114358	28.9039	49.2551	0.769008
Ciclev10020081m.g	scaffold_3:2788922-2790661	1.0513	1.79161	0.769085
Ciclev10028466m.g	scaffold_8:2456339-2458494	3.08954	5.2652	0.769098
Ciclev10009323m.g	scaffold_1:15047698-15049675	5.01702	8.55222	0.76947
Ciclev10030615m.g	scaffold_4:8779258-8782470	0.393145	0.670301	0.769747
Ciclev10008481m.g	scaffold_1:26540978-26555789	8.33454	14.2107	0.769799
Ciclev10025277m.g	scaffold_7:6114738-6120336	16.7386	28.5457	0.770093
Ciclev10016846m.g	scaffold_2:33499538-33502421	4.67916	7.98236	0.770565
Ciclev10007237m.g	scaffold_1:24794828-24807351	5.33224	9.09739	0.77071
Ciclev10030982m.g	scaffold_4:23469183-23472872	67.8423	115.754	0.770801
Ciclev10007671m.g	scaffold_1:5016545-5018537	0.889553	1.51818	0.77119
Ciclev10011048m.g	scaffold_6:19376615-19381536	45.515	77.6816	0.771231
Ciclev10019317m.g	scaffold_3:8225087-8228320	0.0841107	0.143563	0.771325
Ciclev10012049m.g	scaffold_6:21035909-21038368	121.714	207.766	0.771463
Ciclev10018656m.g	scaffold_3:21074501-21081431	37.1391	63.4301	0.772231
Ciclev10008308m.g	scaffold_1:26716952-26722169	74.5813	127.396	0.772435
Ciclev10010956m.g	scaffold_6:12916687-12923335	0.044845	0.0766296	0.772955
Ciclev10000440m.g	scaffold_5:29817690-29822386	6.27817	10.7281	0.772977
Ciclev10017547m.g	scaffold_2:16752012-16759367	1.85734	3.17399	0.773063
Ciclev10025550m.g	scaffold_7:16213380-16216825	3.16681	5.41446	0.773785
Ciclev10025641m.g	scaffold_7:14123946-14154295	26.729	45.7167	0.774314
Ciclev10015108m.g	scaffold_2:7420953-7424988	4.20453	7.19258	0.774565
Ciclev10007944m.g	scaffold_1:22847396-22852110	30.2235	51.7104	0.774781
Ciclev10009750m.g	scaffold_1:26836408-26837592	4.39922	7.52792	0.775003
Ciclev10030828m.g	scaffold_4:10715619-10721328	12.0871	20.6867	0.77523
Ciclev10005457m.g	scaffold_9:29474598-29476059	10.8146	18.5104	0.775349
Ciclev10014034m.g	scaffold_2:32041519-32053665	0.0370738	0.0634674	0.775615
Ciclev10033580m.g	scaffold_4:20045196-20046950	1.87328	3.20698	0.775645
Ciclev10017270m.g	scaffold_2:29813919-29815871	3.89424	6.66689	0.775672
Ciclev10016063m.g	scaffold_2:10424778-10472483	5.88354	10.0734	0.775792
Ciclev10022988m.g	scaffold_3:12722973-12725030	9.28883	15.9147	0.776787
Ciclev10018197m.g	scaffold_2:8244206-8246618	0.229331	0.393088	0.777424
-	scaffold_2:6181064-6338878	28.6161	49.0507	0.777449
Ciclev10010816m.g	scaffold_1:2134988-2137541	2.68582	4.60576	0.778075
Ciclev10005194m.g,Ciclev10006504m.g,Ciclev10006571m.g	scaffold_9:5227254-5233938	29.1765	50.0422	0.77834
Ciclev10031639m.g,Ciclev10031641m.g	scaffold_4:17329472-17351349	105.861	181.57	0.778358
Ciclev10020513m.g	scaffold_3:44005585-44008279	12.9136	22.1546	0.778711
Ciclev10005283m.g	scaffold_9:26890812-26894907	7.91886	13.5856	0.778715
Ciclev10011921m.g	scaffold_6:24708580-24709870	6.5032	11.1626	0.779453
Ciclev10014669m.g	scaffold_2:29955055-29960217	8.08872	13.8862	0.779672
Ciclev10021147m.g	scaffold_3:41391592-41394798	134.226	230.52	0.780223
Ciclev10014610m.g	scaffold_2:25506205-25509221	43.3627	74.4749	0.7803
Ciclev10000591m.g	scaffold_5:36371906-36378249	50.0129	85.9202	0.780695

Ciclev10020303m.g	scaffold_3:19792462-19795861	21.7401	37.3531	0.780866
Ciclev10014439m.g	scaffold_2:188503-193028	76.6496	131.702	0.780922
Ciclev10017075m.g	scaffold_2:33703021-33706271	17.0158	29.2395	0.781042
Ciclev10000301m.g	scaffold_5:34707456-34714949	12.9007	22.1756	0.78153
Ciclev10011283m.g	scaffold_6:11966811-11976771	6.0995	10.4856	0.78165
Ciclev10028550m.g	scaffold_8:858224-860429	53.9772	92.8518	0.782579
Ciclev10009193m.g	scaffold_1:9297359-9299641	21.4879	36.967	0.782711
Ciclev10027172m.g	scaffold_7:11686788-11771145	0.0683594	0.117638	0.783146
Ciclev10001254m.g	scaffold_5:40869559-40872161	10.9153	18.7841	0.783159
Ciclev10018971m.g	scaffold_3:555968-559888	20.3733	35.0645	0.78333
Ciclev10014078m.g	scaffold_2:866585-876264	3.94303	6.7878	0.783639
Ciclev10033972m.g	scaffold_4:5098356-5103879	2.20051	3.78828	0.783706
Ciclev10015364m.g	scaffold_2:24667101-24672331	6.42135	11.0554	0.7838
Ciclev10014916m.g	scaffold_2:32023667-32027248	1.01538	1.74956	0.78497
Ciclev10022815m.g	scaffold_3:23705179-23707053	27.8153	47.9336	0.785159
Ciclev10018370m.g	scaffold_2:18309091-18312175	14.0903	24.2844	0.785322
Ciclev10012690m.g	scaffold_6:22626806-22628852	9.23482	15.9221	0.785871
Ciclev10006075m.g	scaffold_9:29050999-29053612	2.9856	5.14793	0.785973
Ciclev10015335m.g	scaffold_2:30280786-30284224	20.7548	35.7891	0.786073
Ciclev10026841m.g	scaffold_7:9014487-9016262	72.3683	124.801	0.786202
Ciclev10008913m.g	scaffold_1:3217852-3221085	2.65851	4.58472	0.786217
Ciclev10003676m.g	scaffold_5:40431403-40438162	21.6618	37.3576	0.786248
Ciclev10009109m.g	scaffold_1:21789873-21790875	0.240992	0.415656	0.786406
Ciclev10025589m.g	scaffold_7:4139376-4141927	2.67046	4.60618	0.786483
Ciclev10005061m.g	scaffold_9:1836144-1840208	28.9084	49.8631	0.786483
Ciclev10030824m.g	scaffold_4:24742453-24746142	2.363	4.0786	0.787451
Ciclev10033579m.g	scaffold_4:6091433-6092444	22.2929	38.4886	0.787847
Ciclev10015149m.g	scaffold_2:25422296-25492273	9.9533	17.1895	0.788279
Ciclev10016560m.g	scaffold_2:4137731-4140288	18.9508	32.7308	0.788392
Ciclev10027724m.g	scaffold_8:22883593-22889315	13.5457	23.4001	0.788682
-	scaffold_5:3980660-4069314	27.0341	46.7088	0.788912
Ciclev10004584m.g	scaffold_9:31253072-31259207	5.47071	9.45239	0.788952
Ciclev10019636m.g	scaffold_3:5038746-5181239	24.4108	42.1885	0.789332
Ciclev10007700m.g	scaffold_1:27079480-27084934	21.5883	37.3132	0.789439
Ciclev10005611m.g	scaffold_9:26901509-26903617	7.5055	12.9747	0.789684
Ciclev10015212m.g	scaffold_2:20127451-20130458	0.146285	0.252892	0.789742
Ciclev10023176m.g	scaffold_3:649683-650483	15.76	27.25	0.789987
Ciclev10013989m.g	scaffold_13:175011-176530	10.6814	18.4717	0.790214
Ciclev10004308m.g	scaffold_9:30755051-30760514	10.245	17.7178	0.790275
Ciclev10025390m.g	scaffold_7:2246731-2249558	24.0068	41.5209	0.790393
Ciclev10005112m.g	scaffold_9:28577862-28581204	7.16159	12.3903	0.790863
Ciclev10007872m.g	scaffold_1:8387402-8391437	18.4115	31.8572	0.791014
Ciclev10004117m.g	scaffold_9:16711055-16728765	3.8877	6.72745	0.791145
Ciclev10008145m.g	scaffold_1:23650274-23654729	8.58412	14.8555	0.79125
Ciclev10020038m.g	scaffold_3:8256397-8263550	14.5614	25.2003	0.791296
Ciclev10001409m.g	scaffold_5:41173950-41177534	24.3614	42.1753	0.791803
Ciclev10022249m.g	scaffold_3:38323255-38328354	8.28094	14.3398	0.79216
Ciclev10031731m.g	scaffold_4:24887280-24890510	83.4709	144.552	0.792239
Ciclev10027907m.g	scaffold_8:17764711-17767812	1.438	2.49049	0.792368
Ciclev10025140m.g	scaffold_7:12352172-12754529	0.488832	0.846663	0.792448
Ciclev10014273m.g	scaffold_2:20453030-20458409	0.129593	0.224464	0.792502
Ciclev10019528m.g	scaffold_3:7053473-7056292	12.0866	20.9369	0.792636
Ciclev10030528m.g	scaffold_4:2832379-2840049	3.99481	6.92027	0.7927
Ciclev10004458m.g	scaffold_9:378360-382860	31.4292	54.4469	0.792743
Ciclev10031664m.g	scaffold_4:20718509-20723648	8.31274	14.404	0.793076
Ciclev10014164m.g	scaffold_2:11084959-11095416	13.6488	23.6539	0.793299

Ciclev10007553m.g	scaffold_1:6903803-6918085	59.4619	103.102	0.794039
Ciclev10025649m.g	scaffold_7:14540698-14542826	23.4656	40.7018	0.794549
Ciclev10023464m.g	scaffold_3:30618246-30668406	13.2082	22.9123	0.794688
Ciclev10000147m.g	scaffold_5:35395118-35401096	1.76274	3.05826	0.794896
Ciclev10029094m.g	scaffold_8:23533363-23535946	11.6921	20.2897	0.795207
Ciclev10000338m.g	scaffold_5:24926144-25131690	17.0748	29.6309	0.795236
Ciclev10029468m.g	scaffold_8:24225441-24226407	8.60325	14.9306	0.795319
Ciclev10017885m.g	scaffold_2:8867979-8870013	40.3473	70.0379	0.795664
Ciclev10026810m.g	scaffold_7:7007129-7008876	13.0984	22.7384	0.795737
Ciclev10004204m.g	scaffold_9:26962896-26977865	1.26293	2.19253	0.79582
Ciclev10024723m.g	scaffold_7:16686059-16697426	0.038794	0.0673537	0.795924
Ciclev10014320m.g	scaffold_2:29022428-29030812	39.1524	67.9859	0.796135
Ciclev10001304m.g	scaffold_5:35908180-35912917	9.75044	16.9318	0.796198
-	scaffold_5:25221155-25545745	10.1368	17.6041	0.796309
Ciclev10004887m.g	scaffold_9:6901275-6905348	55.8168	96.946	0.796481
-	scaffold_5:10399797-10402513	3.18456	5.53165	0.796615
-	scaffold_1:956150-957408	51.6665	89.757	0.796795
Ciclev10012331m.g	scaffold_6:10082733-10085131	654.317	1136.75	0.796857
Ciclev10014506m.g	scaffold_2:10726039-10735374	11.8403	20.5718	0.796961
Ciclev10028233m.g	scaffold_8:6402839-6404443	0.269873	0.468932	0.797101
Ciclev10022095m.g	scaffold_3:1034502-1037341	1.2478	2.16854	0.797339
Ciclev10025904m.g	scaffold_7:268528-270448	9.74639	16.9404	0.797532
Ciclev10012213m.g	scaffold_6:16589757-16592569	7.78816	13.5369	0.797541
Ciclev10032113m.g	scaffold_4:22523091-22528307	23.5988	41.0251	0.797792
Ciclev10011911m.g	scaffold_6:14444650-14447199	19.4921	33.8913	0.798025
Ciclev10011450m.g	scaffold_6:23306385-23311678	19.6699	34.2102	0.798439
Ciclev10030948m.g,Ciclev10033876m.g	scaffold_4:18714152-18730144	12.7965	22.2562	0.798459
Ciclev10014601m.g	scaffold_2:26947321-26952696	2.45752	4.27429	0.798481
-	scaffold_1:14645558-14645768	102.305	178.18	0.800462
Ciclev10005334m.g	scaffold_9:26117969-26159077	0.811914	1.41435	0.800739
Ciclev10000050m.g	scaffold_5:43198202-43209378	44.9833	78.3704	0.800918
Ciclev10030163m.g	scaffold_8:831694-832216	1.71335	2.98544	0.801122
-	scaffold_8:1236425-1239143	5.07489	8.84288	0.801139
-	scaffold_6:18804290-18806093	46.685	81.3475	0.80114
Ciclev10013195m.g	scaffold_6:1326098-1330553	8.18756	14.2674	0.80122
Ciclev10003743m.g	scaffold_5:37648147-37649503	0.74203	1.29337	0.801582
-	scaffold_9:29938322-29941957	2.90183	5.05944	0.802014
Ciclev10010172m.g	scaffold_1:25092758-25093973	1.06488	1.85667	0.802032
Ciclev10002733m.g	scaffold_5:39974112-39975525	3.55975	6.20875	0.802528
-	scaffold_4:20935892-20939411	16.4662	28.7308	0.803093
Ciclev10031003m.g	scaffold_4:18266261-18268327	291.511	508.867	0.80374
Ciclev10000047m.g	scaffold_5:414255-420341	0.703795	1.22864	0.803832
Ciclev10007998m.g	scaffold_1:14766998-14769846	46.9085	81.9256	0.804464
Ciclev10006644m.g	scaffold_9:31123224-31144160	7.7864	13.6023	0.804817
Ciclev10014534m.g	scaffold_2:32364366-32367574	37.5411	65.608	0.805402
Ciclev10031216m.g,Ciclev10031635m.g	scaffold_4:2086884-2106219	9.56729	16.7228	0.805638
Ciclev10014690m.g	scaffold_2:31460068-31465891	27.1636	47.4832	0.805744
Ciclev10024737m.g	scaffold_7:17901486-17905433	4.64301	8.12006	0.806429
Ciclev10003406m.g	scaffold_5:36277126-36278128	3.55046	6.21325	0.807343
Ciclev10017889m.g	scaffold_2:26847226-26851492	0.464901	0.813687	0.807549
Ciclev10030038m.g	scaffold_8:18973864-19114160	0.0806082	0.141103	0.807755
Ciclev10023469m.g	scaffold_3:32367986-32369348	0.127859	0.223818	0.807764
Ciclev10032870m.g	scaffold_4:23051199-23053949	29.7735	52.1278	0.808021
Ciclev10033304m.g	scaffold_4:20274608-20276840	0.625574	1.09545	0.808273

Ciclev10016996m.g	scaffold_2:25908004-25909392	44.8481	78.5584	0.808718
Ciclev10021946m.g	scaffold_3:12822910-12825620	165.459	289.853	0.808841
Ciclev10005562m.g	scaffold_9:6743599-6744829	1.63049	2.85649	0.808941
Ciclev10013702m.g	scaffold_6:368917-373454	22.2671	39.0115	0.808987
Ciclev10002438m.g	scaffold_5:39986259-39987337	1.70977	2.99625	0.809355
Ciclev10011260m.g	scaffold_6:13141260-13144183	87.5925	153.532	0.809661
Ciclev10013896m.g	scaffold_6:16854942-16857003	0.797545	1.39798	0.809706
Ciclev10002139m.g	scaffold_5:35852523-35856135	13.4696	23.6164	0.810082
-	scaffold_5:12360875-12368656	16.1878	28.3887	0.810409
Ciclev10030634m.g	scaffold_4:22348522-22356055	10.2629	18.0011	0.810643
Ciclev10014751m.g	scaffold_2:22698581-22702080	7.45316	13.0805	0.811492
Ciclev10019817m.g	scaffold_3:51017708-51019428	2.8561	5.01509	0.812232
Ciclev10012439m.g	scaffold_6:3344344-3349460	20.1427	35.3755	0.812495
Ciclev10007826m.g	scaffold_1:7760719-7763948	8.42855	14.8031	0.812543
-	scaffold_2:29308479-29309078	13.0042	22.8432	0.812795
Ciclev10007401m.g	scaffold_1:22702186-22710340	6.57257	11.5458	0.81284
Ciclev10011995m.g	scaffold_6:15040917-15043798	3.51594	6.17747	0.813108
Ciclev10025048m.g	scaffold_7:2227386-2234500	10.2495	18.0125	0.813443
Ciclev10032088m.g	scaffold_4:1351417-1352631	4.23504	7.44273	0.813455
Ciclev10007687m.g	scaffold_1:1740601-1745322	6.89336	12.1146	0.813464
Ciclev10001622m.g	scaffold_5:29339627-29387910	1.44476	2.53922	0.813555
Ciclev10025255m.g	scaffold_7:8230833-8235201	11.8511	20.8306	0.813681
Ciclev10024790m.g	scaffold_7:18358895-18371454	4.82469	8.48044	0.813703
Ciclev10000621m.g	scaffold_5:6028330-6034400	12.6155	22.1777	0.81391
Ciclev10010055m.g	scaffold_1:12972468-12977393	1.09766	1.92987	0.814075
Ciclev10015965m.g	scaffold_2:35713955-35715897	16.1097	28.3241	0.814106
Ciclev10018409m.g	scaffold_2:32318700-32319472	2.86495	5.03721	0.814113
Ciclev10006669m.g	scaffold_9:14231549-14234582	1.21984	2.14523	0.814442
Ciclev10018700m.g	scaffold_3:8718652-8721659	1.09649	1.92874	0.814764
Ciclev10031097m.g	scaffold_4:22628016-22631236	54.7457	96.383	0.816032
Ciclev10026602m.g	scaffold_7:13911683-13912447	1.27016	2.23629	0.816103
Ciclev10010951m.g	scaffold_6:346938-351349	5.74249	10.1115	0.816246
Ciclev10004134m.g	scaffold_9:299760-307726	4.77275	8.40431	0.816309
Ciclev10019161m.g	scaffold_3:42213216-42217391	1.9788	3.48513	0.816591
Ciclev10023283m.g	scaffold_3:24593001-24597811	0.0675656	0.119019	0.816828
Ciclev10032309m.g	scaffold_4:790280-792021	2.53747	4.47423	0.81825
Ciclev10010932m.g	scaffold_6:25270282-25275811	5.69142	10.036	0.818316
Ciclev10001146m.g,Ciclev10002748m.g	scaffold_5:22329881-22340033	21.7903	38.4317	0.818611
Ciclev10031940m.g	scaffold_4:6457129-6458200	0.564271	0.995551	0.819107
Ciclev10023451m.g,Ciclev10023552m.g,Ciclev10023830m.g	scaffold_3:18988839-19105549	10.0352	17.7081	0.819344
Ciclev10004147m.g	scaffold_9:27951358-27973537	3.09061	5.45493	0.819671
Ciclev10011525m.g	scaffold_6:1334470-1339417	148.231	261.655	0.819818
Ciclev10021275m.g	scaffold_3:43895524-43901046	24.4908	43.2348	0.819955
Ciclev10014054m.g	scaffold_2:23898669-23905586	11.0878	19.5765	0.820145
Ciclev10019054m.g	scaffold_3:3915454-3918620	15.9761	28.2109	0.820335
Ciclev10023311m.g	scaffold_3:31514228-31518751	0.251832	0.444696	0.820358
Ciclev10015216m.g	scaffold_2:30557670-30559566	0.32712	0.577794	0.820735
Ciclev10019209m.g	scaffold_3:2998774-3004236	43.996	77.7383	0.821252
Ciclev10032130m.g,Ciclev10033570m.g	scaffold_4:16995148-17013320	5.28073	9.33079	0.821261
-	scaffold_9:19365509-19369118	23.7222	41.934	0.821883
Ciclev10002795m.g	scaffold_5:33256353-33257201	0.842369	1.4894	0.822208
Ciclev10025570m.g	scaffold_7:5072310-5076026	2.73299	4.83272	0.822353

Ciclev10024664m.g	scaffold_3:4777418-4779553	4.51445	7.98405	0.822571
Ciclev10030087m.g	scaffold_8:10892950-10893742	54.1797	95.8352	0.822803
Ciclev10008729m.g	scaffold_1:966392-971529	41.0849	72.6792	0.822932
Ciclev10026129m.g	scaffold_7:10583645-10587830	0.9636	1.70465	0.822972
Ciclev10019658m.g	scaffold_3:50680639-50683144	36.028	63.7431	0.82315
Ciclev10020353m.g	scaffold_3:44768190-44770578	3.61294	6.39512	0.823797
Ciclev10017966m.g	scaffold_2:22371490-22383372	19.7998	35.0525	0.824034
Ciclev10005524m.g	scaffold_9:30991997-30999295	9.78447	17.3235	0.824169
Ciclev10007631m.g	scaffold_1:74485-82356	12.1683	21.5539	0.824816
Ciclev10029136m.g	scaffold_8:19503303-19505761	13.2126	23.4176	0.82568
Ciclev10028059m.g	scaffold_8:344009-350301	8.40618	14.9012	0.825903
Ciclev10025179m.g	scaffold_7:6664094-6668911	6.77391	12.0109	0.826285
Ciclev10015779m.g	scaffold_2:4895612-4986690	9.63012	17.0774	0.826464
Ciclev10005323m.g	scaffold_9:2866804-2871704	15.4397	27.3862	0.826803
Ciclev10030896m.g	scaffold_4:24323795-24326796	44.2379	78.4741	0.826934
Ciclev10028663m.g	scaffold_8:18350158-18353067	14.1524	25.1132	0.827401
-	scaffold_4:658703-662176	3.3054	5.86546	0.82742
Ciclev10004317m.g	scaffold_9:15116834-15119883	0.595749	1.05727	0.827568
Ciclev10013459m.g	scaffold_6:23699647-23702260	1.39321	2.47315	0.827935
Ciclev10032496m.g	scaffold_4:21778908-21781169	9.82	17.4335	0.828064
Ciclev10004721m.g	scaffold_9:29040440-29045769	8.31785	14.7703	0.828418
Ciclev10004456m.g	scaffold_9:29517562-29520928	15.9587	28.3385	0.828422
Ciclev10013015m.g	scaffold_6:18787839-18790676	4.48608	7.96663	0.828513
Ciclev10014963m.g	scaffold_2:29314175-29319032	57.8925	102.835	0.828878
Ciclev10019283m.g	scaffold_3:960587-963588	4.79857	8.52558	0.829194
Ciclev10022157m.g	scaffold_3:9087944-9091191	9.35319	16.6212	0.829496
-	scaffold_7:12328259-12331595	4.06453	7.22393	0.829694
Ciclev10022996m.g	scaffold_3:47033879-47035562	30.7011	54.5656	0.829705
Ciclev10001551m.g,Ciclev10001749m.g	scaffold_5:29653121-29660545	19.5001	34.6654	0.830018
Ciclev10008972m.g	scaffold_1:11350207-11354261	3.40865	6.05964	0.830032
Ciclev10014720m.g	scaffold_2:65556-69410	21.5992	38.4134	0.830634
Ciclev10004635m.g	scaffold_9:3571955-3575605	4.83769	8.60861	0.831461
Ciclev10003128m.g	scaffold_5:32333983-32338708	0.0629982	0.112109	0.83152
Ciclev10011506m.g	scaffold_6:21053175-21055123	14.9707	26.6443	0.831682
Ciclev10015461m.g	scaffold_2:32081839-32084232	2.90261	5.16594	0.831683
Ciclev10007376m.g	scaffold_1:20841651-20844762	1.3586	2.41802	0.831711
Ciclev10005921m.g	scaffold_9:2568033-2571241	203.715	362.719	0.8323
-	scaffold_4:5266368-5267034	3.38788	6.03306	0.832506
Ciclev10007823m.g	scaffold_1:22857598-22862279	9.73115	17.3337	0.832899
Ciclev10016927m.g	scaffold_2:17390388-17391229	184.316	328.603	0.834165
Ciclev10002769m.g	scaffold_5:27430779-27433174	6.17062	11.0036	0.834489
Ciclev10021875m.g	scaffold_3:2565082-2567770	5.85963	10.4512	0.834785
Ciclev10002782m.g	scaffold_5:42799537-42801821	375.432	669.702	0.834966
Ciclev10005989m.g	scaffold_9:2165865-2170588	205.277	366.324	0.835545
Ciclev10025360m.g	scaffold_7:12985953-12989228	17.0565	30.4412	0.835709
Ciclev10032276m.g	scaffold_4:24657014-24660141	9.65074	17.2276	0.836007
Ciclev10023658m.g	scaffold_3:18891625-18895238	13.4282	23.9726	0.836125
Ciclev10014191m.g	scaffold_2:29301902-29306348	4.32889	7.73064	0.83659
Ciclev10007960m.g	scaffold_1:16720227-16721894	2.41235	4.3086	0.836779
Ciclev10013690m.g	scaffold_6:19518148-19518937	11.0584	19.7547	0.837054
Ciclev10006456m.g	scaffold_9:24091944-24101776	4.89909	8.75584	0.837734
Ciclev10014263m.g	scaffold_2:31716485-31719535	1.72533	3.08378	0.837826
-	scaffold_1:3213352-3215134	11.6925	20.9031	0.838133
Ciclev10011534m.g	scaffold_6:23506490-23509585	3.17113	5.67008	0.838371
Ciclev10018515m.g	scaffold_3:26552199-26563660	0.503872	0.90105	0.838549

Ciclev10020807m.g	scaffold_3:47366615-47370779	16.8219	30.0886	0.838871
Ciclev10007521m.g	scaffold_1:2124312-2127315	7.11126	12.7202	0.838943
Ciclev10030306m.g	scaffold_8:1309648-1311169	71.9666	128.785	0.839559
Ciclev10002661m.g	scaffold_5:32715595-32718151	5.59282	10.0167	0.840758
Ciclev10032089m.g	scaffold_4:17918135-17920519	63.0901	112.997	0.840801
Ciclev10010889m.g	scaffold_6:24747384-24755124	3.23577	5.79622	0.841003
Ciclev10030499m.g	scaffold_4:22400757-22414668	25.3794	45.4722	0.841325
Ciclev10001093m.g,Ciclev10002998m.g	scaffold_5:15605851-15615844	48.1592	86.2996	0.841543
Ciclev10007313m.g	scaffold_1:25006889-25016472	1.72169	3.08527	0.841573
Ciclev10001461m.g	scaffold_5:27819067-27820508	13.6381	24.449	0.84213
Ciclev10022043m.g	scaffold_3:33388539-33592383	0.947413	1.69862	0.842297
Ciclev10005704m.g	scaffold_9:13311971-13317864	16.5569	29.6862	0.842355
Ciclev10008204m.g	scaffold_1:21412805-21415549	17.3261	31.0733	0.842731
Ciclev10020197m.g	scaffold_3:34215689-34218856	4.08053	7.32044	0.843174
Ciclev10019245m.g	scaffold_3:39987678-39993513	8.1635	14.6469	0.843336
Ciclev10015691m.g	scaffold_2:34342256-34345227	4.84246	8.68862	0.843387
Ciclev10000155m.g	scaffold_5:35039707-35043069	4.65047	8.34774	0.844008
Ciclev10000935m.g	scaffold_5:42792299-42794134	5.32912	9.57251	0.845
Ciclev10004180m.g	scaffold_9:29237784-29243574	15.5964	28.0197	0.84523
Ciclev10006185m.g	scaffold_9:4817124-4818758	1.56021	2.8033	0.845383
Ciclev10017806m.g	scaffold_2:33085024-33087353	2.5517	4.58565	0.845665
Ciclev10014605m.g,Ciclev10017460m.g	scaffold_2:1036400-1060029	20.6259	37.0745	0.84597
Ciclev10021736m.g	scaffold_3:36588158-36592116	3.96118	7.12204	0.846361
-	scaffold_1:25451638-25454206	6.5129	11.71	0.846373
Ciclev10031631m.g	scaffold_4:5031827-5038748	9.99326	17.968	0.846405
Ciclev10000152m.g,Ciclev10000404m.g	scaffold_5:27492965-27539324	5.58864	10.0583	0.847815
Ciclev10007675m.g	scaffold_1:8818108-8821490	1.65777	2.98656	0.849242
Ciclev10002928m.g	scaffold_5:41777476-41778540	2.11068	3.80383	0.849745
Ciclev10026915m.g	scaffold_7:14400675-14403763	1.88674	3.4011	0.850105
Ciclev10030103m.g	scaffold_8:644159-649675	21.9049	39.5093	0.850941
Ciclev10010344m.g	scaffold_1:2076755-2078027	0.760849	1.37245	0.851076
Ciclev10008977m.g	scaffold_1:18109647-18113127	17.3978	31.3887	0.85134
Ciclev10007456m.g	scaffold_1:26248370-26253868	0.550268	0.992787	0.851349
Ciclev10015002m.g	scaffold_2:33350601-33353154	3.01441	5.44092	0.851976
Ciclev10006632m.g	scaffold_9:26694563-26789030	1.69076	3.05354	0.852808
Ciclev10008058m.g	scaffold_1:18326556-18331589	0.580726	1.04906	0.85317
Ciclev10028133m.g	scaffold_8:6298165-6304194	1.68424	3.04293	0.853362
Ciclev10021401m.g	scaffold_3:43783860-43792802	19.8053	35.8093	0.854445
Ciclev10012224m.g	scaffold_6:24382559-24385222	17.151	31.0212	0.854958
Ciclev10021730m.g	scaffold_3:37070235-37072945	4.29737	7.77431	0.85526
Ciclev10016013m.g	scaffold_2:36163289-36165355	28.9355	52.3694	0.855885
Ciclev10024217m.g	scaffold_3:22823818-22824823	4.57516	8.28102	0.855987
Ciclev10032421m.g	scaffold_4:5278162-5282681	21.6442	39.1812	0.856182
Ciclev10015872m.g	scaffold_2:26630153-26632900	241.061	436.379	0.856184
Ciclev10026391m.g	scaffold_7:20872461-20874659	15.526	28.1068	0.856233
Ciclev10019423m.g,Ciclev10019432m.g,Ciclev10019435m.g,Ciclev10020241m.g	scaffold_3:36126169-36259240	7.75402	14.0401	0.856534
Ciclev10022127m.g	scaffold_3:39819129-39823025	11.4104	20.6646	0.856814
Ciclev10020578m.g	scaffold_3:37507243-37511616	13.0367	23.6102	0.856837
Ciclev10008689m.g	scaffold_1:20094574-20101958	9.30609	16.8605	0.857398
Ciclev10005440m.g	scaffold_9:25804058-25805380	2.4895	4.51059	0.85746
Ciclev10005306m.g	scaffold_9:30876327-30879006	3.76238	6.81825	0.857756

Ciclev10018070m.g	scaffold_2:8954838-8978273	1.99186	3.61333	0.859212
Ciclev10003573m.g	scaffold_5:38971129-38971396	19.284	34.9878	0.85945
Ciclev10004046m.g	scaffold_5:34308986-34310762	2.82958	5.13624	0.860124
Ciclev10030066m.g	scaffold_8:23460532-23462209	0.95318	1.73029	0.860191
Ciclev10024930m.g	scaffold_7:6808846-6815189	1.78849	3.24753	0.860597
Ciclev10023300m.g	scaffold_3:45793876-45798206	18.8154	34.1687	0.860758
Ciclev10000494m.g	scaffold_5:29424164-29437482	1.28259	2.33025	0.861427
Ciclev10003175m.g	scaffold_5:33321898-33325779	80.254	145.813	0.861471
Ciclev10032743m.g	scaffold_4:15299553-15301460	191.88	348.638	0.861527
Ciclev10025794m.g	scaffold_7:3998477-4004529	25.1454	45.6889	0.861549
Ciclev10029623m.g	scaffold_8:24532867-24534207	27.5965	50.1547	0.8619
Ciclev10020764m.g	scaffold_3:3017870-3022053	10.402	18.9083	0.862166
Ciclev10029400m.g	scaffold_8:20109360-20112043	4.56419	8.29667	0.862173
Ciclev10003719m.g	scaffold_5:12152922-12157751	3.62787	6.59473	0.862189
Ciclev10024487m.g	scaffold_3:40170767-40171724	0.695924	1.2654	0.862595
Ciclev10015269m.g	scaffold_2:1187217-1192976	10.1482	18.4548	0.862774
Ciclev10007619m.g	scaffold_1:14412070-14415252	1.43104	2.60299	0.8631
Ciclev10012485m.g	scaffold_6:15266062-15267878	17.4342	31.7119	0.863107
Ciclev10015556m.g	scaffold_2:8893934-8895732	5.94584	10.8189	0.8636
Ciclev10033591m.g	scaffold_4:22024173-22025980	49.4706	90.0199	0.863673
Ciclev10026544m.g	scaffold_7:8653583-8655366	34.6123	63.0144	0.864398
Ciclev10007831m.g	scaffold_1:6716128-6721429	0.887862	1.6165	0.86447
Ciclev10020079m.g	scaffold_3:25598721-25601734	2.72838	4.96809	0.864647
Ciclev10000755m.g	scaffold_5:42641055-42643541	7.63652	13.906	0.864716
Ciclev10015653m.g	scaffold_2:27873386-27877069	6.90312	12.5731	0.865022
Ciclev10019572m.g	scaffold_3:35760137-35764708	5.31882	9.69023	0.865425
Ciclev10010729m.g	scaffold_1:1219661-1222959	1.50385	2.74037	0.865707
Ciclev10028627m.g	scaffold_8:6289476-6292850	6.54565	11.929	0.865861
Ciclev10019744m.g	scaffold_3:42254410-42257087	6.83801	12.4639	0.866105
Ciclev10000894m.g	scaffold_5:39126563-39130916	11.5258	21.0091	0.866151
Ciclev10009878m.g	scaffold_1:27764735-27766035	6.81982	12.4327	0.866334
Ciclev10019724m.g	scaffold_3:1318118-1321956	13.3931	24.4215	0.866662
Ciclev10001600m.g	scaffold_5:40628243-40630241	19.6066	35.7702	0.86742
-	scaffold_6:20361413-20361804	4.72774	8.6256	0.867476
Ciclev10024478m.g	scaffold_3:38412633-38416083	0.361887	0.660357	0.867707
Ciclev10004132m.g	scaffold_9:25502069-25518959	3.97112	7.2473	0.867898
Ciclev10031547m.g	scaffold_4:4077556-4083819	26.8526	49.0258	0.86848
Ciclev10011342m.g	scaffold_6:23563966-23565958	1.50028	2.73975	0.868809
Ciclev10025411m.g	scaffold_7:7262186-7267580	61.2318	111.821	0.868833
Ciclev10008071m.g	scaffold_1:3243423-3246117	12.4258	22.7134	0.870203
Ciclev10009692m.g	scaffold_1:27745717-27747372	18.0334	32.9806	0.870941
Ciclev10014096m.g	scaffold_2:24302052-24307850	9.0174	16.4927	0.871042
Ciclev10015548m.g	scaffold_2:30607995-30610863	2.92296	5.34613	0.871066
Ciclev10008898m.g	scaffold_1:24052196-24054905	21.3715	39.1288	0.872538
Ciclev10002756m.g	scaffold_5:31121804-31181813	201.096	368.356	0.873212
Ciclev10004250m.g	scaffold_9:24807947-24823388	13.1411	24.0731	0.873331
Ciclev10031660m.g	scaffold_4:3421872-3425673	14.0616	25.7932	0.875228
Ciclev10000396m.g	scaffold_5:40479670-40485358	13.5521	24.863	0.875478
Ciclev10000762m.g	scaffold_5:20554135-20561666	18.962	34.8014	0.876035
Ciclev10004477m.g	scaffold_9:16418921-16422631	15.8583	29.1241	0.876973
Ciclev10019288m.g,Ciclev10023424m.g,Ciclev10024076m.g,Ciclev10024305m.g	scaffold_3:11687448-12154880	3.84572	7.06436	0.877306
Ciclev10000978m.g	scaffold_5:34211378-34217253	5.52115	10.1464	0.87792
-	scaffold_2:19827359-19828486	10.9447	20.1145	0.878003
Ciclev10001729m.g	scaffold_5:42978751-42983950	6.42852	11.8156	0.87814

Ciclev10027589m.g	scaffold_7:10555107-10583369	0.386931	0.711205	0.878189
Ciclev10014607m.g	scaffold_2:35122860-35130519	10.7597	19.7775	0.878225
Ciclev10024421m.g	scaffold_3:32396982-32398373	1.31808	2.42385	0.878859
-	scaffold_5:15797014-15799040	2.77939	5.11157	0.878996
Ciclev10007836m.g	scaffold_1:27264809-27270486	3.9544	7.2741	0.879309
Ciclev10020086m.g	scaffold_3:7500527-7502224	5.05681	9.30424	0.879661
Ciclev10012372m.g	scaffold_6:9323671-9330498	1.18774	2.18564	0.87983
Ciclev10024868m.g	scaffold_7:10555107-10583369	0.862842	1.58777	0.879831
Ciclev10025650m.g	scaffold_7:14224606-14226732	7.92727	14.592	0.880282
Ciclev10029689m.g	scaffold_8:1757873-1758718	14.2784	26.2837	0.880332
Ciclev10025301m.g	scaffold_7:21098070-21102921	16.9426	31.1911	0.880482
Ciclev10015458m.g	scaffold_2:23717880-23719637	0.88775	1.63442	0.880549
Ciclev10021925m.g	scaffold_3:4515194-4516982	57.1593	105.254	0.880814
-	scaffold_2:27825744-27827061	3.61633	6.66667	0.882439
Ciclev10004848m.g	scaffold_9:17242217-17245901	61.4104	113.316	0.883792
Ciclev10012796m.g	scaffold_6:8360224-8361151	17.8465	32.9338	0.883929
Ciclev10024065m.g	scaffold_3:43377782-43387734	13.57	25.0474	0.88424
Ciclev10011601m.g	scaffold_6:8958457-8986420	4.72909	8.73295	0.884906
Ciclev10015816m.g	scaffold_2:16365083-16368253	16.2261	29.9645	0.884938
Ciclev10004546m.g	scaffold_9:127872-132900	8.75386	16.1685	0.885192
Ciclev10015189m.g	scaffold_2:6658002-6660691	4.6044	8.50548	0.885378
Ciclev10000425m.g	scaffold_5:37669429-37671959	199.671	368.927	0.885712
Ciclev10018366m.g	scaffold_2:11413163-11414803	0.310986	0.574932	0.886541
Ciclev10008766m.g	scaffold_1:26346646-26350239	5.18808	9.59381	0.886902
Ciclev10031694m.g	scaffold_4:11210878-11216325	18.897	34.9618	0.887617
Ciclev10000324m.g	scaffold_5:41582117-41585127	8.8633	16.4115	0.888795
Ciclev10020327m.g	scaffold_3:18965761-18968313	22.7306	42.0957	0.889038
Ciclev10017563m.g	scaffold_2:5650654-5706559	7.5171	13.9232	0.889244
Ciclev10014036m.g	scaffold_2:27097749-27107605	3.72647	6.9033	0.889476
Ciclev10001562m.g	scaffold_5:41084108-41089200	11.8258	21.9112	0.889734
Ciclev10025479m.g	scaffold_7:1637567-1643002	1.91542	3.54897	0.889741
Ciclev10014481m.g	scaffold_2:24293274-24297396	1.39421	2.58351	0.88989
Ciclev10020718m.g	scaffold_3:44141304-44144453	6.56815	12.1711	0.8899
Ciclev10007639m.g	scaffold_1:1203924-1208231	6.66603	12.3526	0.889919
Ciclev10027396m.g,Ciclev10027506m.g	scaffold_7:16939640-16973171	17.3157	32.0935	0.890205
Ciclev10000732m.g	scaffold_5:32720670-32722787	0.534206	0.990452	0.890692
Ciclev10026618m.g	scaffold_7:16616365-16618215	62.7901	116.426	0.890804
Ciclev10015811m.g	scaffold_2:32238565-32241277	13.1107	24.3111	0.890869
Ciclev10027840m.g	scaffold_8:21485233-21489942	138.846	257.486	0.891007
Ciclev10028404m.g	scaffold_8:21314284-21316727	17.2962	32.0881	0.891587
Ciclev10025563m.g	scaffold_7:17862571-17866030	0.93259	1.73018	0.891603
Ciclev10021489m.g	scaffold_3:6916294-6919289	25.1445	46.6633	0.892045
Ciclev10014140m.g	scaffold_2:22131629-22139650	60.5075	112.315	0.892359
Ciclev10020125m.g	scaffold_3:15339160-15341593	2.95213	5.47999	0.892415
Ciclev10032511m.g	scaffold_4:2150475-2153029	12.5725	23.3412	0.892608
Ciclev10007387m.g	scaffold_1:26934197-26940906	2.12011	3.93696	0.892943
Ciclev10018576m.g	scaffold_3:39163076-39181525	11.426	21.2337	0.894042
Ciclev10003552m.g	scaffold_5:43178456-43181328	1.82426	3.39073	0.894286
Ciclev10030720m.g	scaffold_4:10237216-10239700	0.0805513	0.149735	0.894431
Ciclev10007518m.g	scaffold_1:26485086-26490576	6.18726	11.5028	0.894615
Ciclev10033799m.g	scaffold_4:15087930-15266082	2.17829	4.05051	0.894904
Ciclev10004974m.g	scaffold_9:31010082-31017820	10.8716	20.216	0.894935
Ciclev10007745m.g	scaffold_1:8138750-8170757	0.29403	0.546854	0.895193
Ciclev10025209m.g	scaffold_7:4345055-4348614	0.69539	1.29357	0.895464
Ciclev10019850m.g	scaffold_3:8207181-8212606	44.5332	82.8528	0.89567

Ciclev10027414m.g	scaffold_7:2477548-2479396	0.747625	1.39095	0.895678
Ciclev10004459m.g	scaffold_9:28065537-28070492	48.4472	90.1357	0.895686
Ciclev10002679m.g	scaffold_5:24789819-24793279	28.038	52.1749	0.89597
Ciclev10031062m.g	scaffold_4:23060905-23064861	6.01327	11.1925	0.89631
Ciclev10013541m.g	scaffold_6:20812858-20816305	0.674867	1.25662	0.896873
Ciclev10023415m.g,Ciclev10023802m.g	scaffold_3:10866466-10871599	4.21616	7.85157	0.897051
Ciclev10004524m.g,Ciclev10005363m.g	scaffold_9:13255658-13295174	33.411	62.2255	0.897184
Ciclev10019160m.g	scaffold_3:3567561-3569771	8.07174	15.0336	0.897238
Ciclev10018334m.g	scaffold_2:31002322-31004629	0.557193	1.03796	0.8975
Ciclev10015501m.g	scaffold_2:13933858-13935732	2.33063	4.34161	0.897507
Ciclev10020622m.g	scaffold_3:2598365-2601663	5.46922	10.1946	0.898394
Ciclev10019760m.g	scaffold_3:49125461-49133697	2.48661	4.6355	0.898546
Ciclev10006796m.g	scaffold_9:7458674-7466043	0.958905	1.78855	0.899329
Ciclev10003101m.g	scaffold_5:6099600-6100071	1.40414	2.61957	0.899648
Ciclev10016674m.g	scaffold_2:10755557-10758061	10.3223	19.259	0.899772
Ciclev10000296m.g	scaffold_5:42007123-42011753	96.4094	179.921	0.900116
Ciclev10011045m.g	scaffold_6:16846741-16850643	12.866	24.0378	0.901738
Ciclev10025152m.g	scaffold_7:5993827-5997780	1.97156	3.68383	0.901872
Ciclev10020997m.g	scaffold_3:41334609-41336413	18.8181	35.1616	0.90188
Ciclev10016802m.g	scaffold_2:21237107-21238109	1.4046	2.62492	0.902116
Ciclev10015629m.g	scaffold_2:12290288-12291745	0.712595	1.33207	0.902517
Ciclev10000241m.g	scaffold_5:41536513-41542715	1.52164	2.84598	0.903298
Ciclev10016201m.g	scaffold_2:2969160-2973743	3.15972	5.91217	0.903893
Ciclev10025661m.g	scaffold_7:10822407-10828538	4.2926	8.03483	0.904418
Ciclev10008568m.g	scaffold_1:19721667-19778009	1.27249	2.38198	0.904515
Ciclev10014674m.g	scaffold_2:11487590-11492415	35.2498	66	0.90485
Ciclev10004542m.g	scaffold_9:7365484-7367383	0.784454	1.46893	0.905008
-	scaffold_9:27948188-27948712	2.15636	4.04137	0.906245
-	scaffold_5:40314193-40314625	15.3598	28.8099	0.907408
Ciclev10011684m.g,Ciclev10012652m.g	scaffold_6:10816139-10936716	461.187	865.132	0.907569
Ciclev10000651m.g	scaffold_5:39858530-39862402	16.6577	31.2552	0.907902
Ciclev10024936m.g	scaffold_7:7249427-7252218	16.792	31.5432	0.909558
Ciclev10009018m.g,Ciclev10009977m.g	scaffold_1:26200785-26208427	32.0607	60.2346	0.909788
Ciclev10007808m.g	scaffold_1:17398025-17404386	46.7313	87.8007	0.909842
Ciclev10009299m.g	scaffold_1:23500656-23502285	17.0195	31.9808	0.910019
Ciclev10014290m.g	scaffold_2:35620831-35628855	2.45185	4.60728	0.910047
Ciclev10033239m.g	scaffold_4:22275312-22276162	16.4646	30.9435	0.91027
Ciclev10005517m.g	scaffold_9:2234065-2238313	65.7352	123.671	0.911765
Ciclev10025392m.g	scaffold_7:7194736-7198550	3.42511	6.44647	0.912362
Ciclev10032243m.g	scaffold_4:20847058-20849250	8.98121	16.9327	0.914828
Ciclev10031223m.g	scaffold_4:24353421-24355495	0.342057	0.644968	0.914989
Ciclev10019463m.g	scaffold_3:13145187-13150117	4.28897	8.08952	0.915422
-	scaffold_7:19327574-19328826	3.20276	6.04378	0.916134
Ciclev10022478m.g	scaffold_3:37140640-37141583	18.8619	35.5948	0.916192
Ciclev10011934m.g	scaffold_6:16553148-16557728	17.1612	32.3915	0.916467
-	scaffold_4:7421909-7422289	47.2796	89.2967	0.917389
Ciclev10008185m.g,Ciclev10010852m.g	scaffold_1:15740850-15743678	53.7135	101.453	0.917456
Ciclev10012325m.g	scaffold_6:21687997-21691710	8.84377	16.7046	0.917514
Ciclev10003517m.g	scaffold_5:37314659-37323758	1.02999	1.94562	0.917594
Ciclev10029371m.g	scaffold_8:5159427-5162015	4.51488	8.53525	0.918746
Ciclev10018817m.g	scaffold_3:2633411-2638404	11.2315	21.2397	0.919221

Ciclev10028300m.g	scaffold_8:24007860-24043482	27.4414	51.9104	0.919669
Ciclev10019923m.g,Ciclev10024572m.g	scaffold_3:45025942-45042250	5.22513	9.88939	0.920416
Ciclev10015633m.g	scaffold_2:26998939-27002554	9.67482	18.3185	0.920997
Ciclev10018669m.g	scaffold_3:37601492-37608736	16.875	31.9612	0.921431
Ciclev10031325m.g	scaffold_4:24138344-24141271	4.24311	8.03773	0.921666
Ciclev10014227m.g	scaffold_2:33957381-33961267	24.9259	47.2192	0.921727
Ciclev10028186m.g	scaffold_8:2427000-2430311	17.2714	32.7342	0.922414
Ciclev10025027m.g	scaffold_7:598930-603130	10.379	19.6811	0.923141
Ciclev10014726m.g	scaffold_2:31094174-31095934	1.17068	2.22011	0.923282
Ciclev10030493m.g	scaffold_4:20905383-20914107	9.36975	17.7723	0.923549
Ciclev10030137m.g	scaffold_8:17108463-17110622	5.80186	11.0078	0.923942
Ciclev10031010m.g	scaffold_4:1058824-1062698	6.93867	13.1696	0.924477
Ciclev10004484m.g,Ciclev10006614m.g	scaffold_9:3500323-3515705	32.9245	62.531	0.925411
Ciclev10033975m.g	scaffold_4:18272542-18273820	4.52842	8.60078	0.925461
Ciclev10028932m.g	scaffold_8:20479071-20482109	37.7939	71.7862	0.925555
Ciclev10015147m.g	scaffold_2:25422296-25492273	11.5603	21.9613	0.925788
Ciclev10010567m.g	scaffold_1:957780-963845	0.703397	1.33659	0.92615
Ciclev10011096m.g	scaffold_6:13412580-13417364	1.3982	2.65747	0.926483
Ciclev10008842m.g	scaffold_1:18002196-18003432	0.271283	0.515731	0.926819
Ciclev10019533m.g	scaffold_3:47390659-47394084	1.0056	1.91243	0.927357
Ciclev10016190m.g	scaffold_2:9570991-9573098	5.86398	11.1574	0.92805
-	scaffold_5:10124042-10124206	98.1409	186.761	0.928268
Ciclev10019007m.g	scaffold_3:6981494-6987498	0.71923	1.36885	0.928437
Ciclev10008024m.g,Ciclev10008854m.g	scaffold_1:17704705-17802531	8.74925	16.6534	0.928582
Ciclev10013715m.g	scaffold_6:24208288-24214431	7.40865	14.1034	0.928761
Ciclev10015110m.g	scaffold_2:13212996-13216067	33.8707	64.5167	0.929637
Ciclev10015091m.g	scaffold_2:31303295-31305804	0.606823	1.15595	0.929728
Ciclev10002541m.g	scaffold_5:42365305-42369429	31.9732	60.9142	0.929919
Ciclev10020920m.g	scaffold_3:8590482-8593922	5.87842	11.2057	0.930729
Ciclev10008983m.g	scaffold_1:22038516-22041113	10.63	20.2683	0.93109
Ciclev10004764m.g,Ciclev10004798m.g	scaffold_9:26790441-26802517	5.29985	10.1111	0.931918
Ciclev10032528m.g	scaffold_4:496918-498814	19.8335	37.8392	0.931947
Ciclev10018094m.g	scaffold_2:34157456-34157975	1.32486	2.52793	0.932114
Ciclev10006403m.g	scaffold_9:26492659-26494523	2.17177	4.14445	0.932311
Ciclev10018026m.g	scaffold_2:9805454-9816275	1.9478	3.71752	0.932495
Ciclev10018944m.g	scaffold_3:50703798-50710771	19.2321	36.7232	0.933176
Ciclev10025470m.g	scaffold_7:4252508-4254105	3.84459	7.34322	0.933584
Ciclev10023713m.g	scaffold_3:16697118-16701540	7.03125	13.4315	0.933763
Ciclev10029466m.g	scaffold_8:23131860-23132651	4.81665	9.20129	0.933807
Ciclev10025227m.g	scaffold_7:342296-346663	20.6243	39.4026	0.933944
Ciclev10019880m.g	scaffold_3:12209798-12213340	4.53859	8.67112	0.933974
Ciclev10019778m.g	scaffold_3:44999604-45002966	2.56841	4.90944	0.934681
Ciclev10021427m.g	scaffold_3:3748521-3752382	4.83205	9.23825	0.934984
-	scaffold_2:27899209-27902966	4.10687	7.85204	0.935028
Ciclev10015007m.g	scaffold_2:25769261-25772686	0.369038	0.705655	0.935192
Ciclev10019823m.g	scaffold_3:9583733-9585374	2.20723	4.22404	0.93639
Ciclev10015184m.g	scaffold_2:893773-899350	8.12635	15.554	0.936607
Ciclev10026248m.g	scaffold_7:3175616-3177722	108.45	207.79	0.938099
Ciclev10014380m.g	scaffold_2:29227933-29236921	20.8986	40.0484	0.938341
Ciclev10011785m.g	scaffold_6:22381647-22385288	103.943	199.235	0.938674
Ciclev10004282m.g,Ciclev10006498m.g	scaffold_9:23075529-23122347	28.2721	54.1972	0.93884

Ciclev10031414m.g	scaffold_4:12900776-12906838	65.3248	125.245	0.939045
Ciclev10027943m.g	scaffold_8:1278523-1282133	52.092	99.8841	0.939194
Ciclev10015792m.g	scaffold_2:35669891-35671475	14.6211	28.0357	0.93921
Ciclev10029017m.g	scaffold_8:4376348-4377405	5.649	10.8339	0.939491
Ciclev10007876m.g	scaffold_1:23135169-23139776	18.5902	35.6594	0.939743
Ciclev10024792m.g	scaffold_7:17322725-17327510	1.55245	2.97806	0.939826
Ciclev10019280m.g	scaffold_3:3829741-3831896	25.7154	49.3721	0.941066
Ciclev10028926m.g	scaffold_8:1993658-1996827	6.53396	12.5466	0.941264
Ciclev10000531m.g	scaffold_5:33273052-33276013	0.538226	1.03374	0.941585
Ciclev10008562m.g	scaffold_1:15883117-15891083	51.2057	98.4048	0.942423
Ciclev10028706m.g	scaffold_8:3587600-3591700	7.58601	14.5819	0.942769
Ciclev10030362m.g	scaffold_8:19647246-19652280	88.4061	169.951	0.942901
Ciclev10011485m.g	scaffold_6:22414764-22416814	0.627242	1.20647	0.943694
Ciclev10005197m.g	scaffold_9:26684726-26689715	27.2882	52.4882	0.943713
Ciclev10016159m.g	scaffold_2:19893157-19895112	2.10556	4.05034	0.943837
Ciclev10011424m.g	scaffold_6:2531349-2533383	10.0537	19.3471	0.944387
Ciclev10018382m.g	scaffold_2:13799455-13802309	1.25415	2.41386	0.94464
Ciclev10014987m.g	scaffold_2:34036935-34038622	2.68903	5.17878	0.945527
Ciclev10000256m.g	scaffold_5:42653993-42661287	20.1031	38.7194	0.945639
Ciclev10008781m.g	scaffold_1:25497068-25499014	4.30459	8.29119	0.945702
Ciclev10019091m.g	scaffold_3:17784071-17788611	25.9481	50.0145	0.946719
Ciclev10005043m.g	scaffold_9:2192718-2194311	15.1401	29.1916	0.947179
Ciclev10028148m.g	scaffold_8:3754499-3783084	24.324	46.9244	0.947956
Ciclev10018758m.g	scaffold_3:28686093-28689070	0.549361	1.05994	0.948152
Ciclev10013735m.g	scaffold_6:14504240-14507074	1639.09	3163.44	0.948598
Ciclev10011285m.g	scaffold_6:20404515-20411982	60.4719	116.712	0.948613
Ciclev10033013m.g	scaffold_4:1788017-1789949	15.8817	30.6528	0.948653
Ciclev10005165m.g	scaffold_9:30033674-30035553	0.324982	0.627417	0.949065
Ciclev10005570m.g	scaffold_9:26047508-26048622	8.37906	16.1821	0.949541
Ciclev10024875m.g	scaffold_7:4822339-4828769	8.69759	16.7996	0.94974
Ciclev10025380m.g	scaffold_7:9248408-9255253	4.45466	8.60686	0.950172
Ciclev10022644m.g	scaffold_3:8843643-8845811	38.6187	74.6204	0.950271
Ciclev10004312m.g	scaffold_9:181691-185323	3.74069	7.2279	0.950274
Ciclev10005938m.g	scaffold_9:23136975-23139812	21.1213	40.826	0.950788
Ciclev10026757m.g	scaffold_7:4779399-4780310	4.86763	9.40999	0.950972
Ciclev10014195m.g	scaffold_2:29856811-29860798	1.809	3.49721	0.951008
-	scaffold_8:7177993-7178285	14.3206	27.6925	0.951409
Ciclev10019516m.g	scaffold_3:43810827-43813244	6.65238	12.8703	0.952107
Ciclev10028445m.g	scaffold_8:22310748-22313373	41.7165	80.7176	0.952263
Ciclev10004503m.g	scaffold_9:2020990-2023201	0.51675	0.999958	0.952401
Ciclev10011684m.g	scaffold_6:10816139-10936716	164.754	318.819	0.952422
Ciclev10010984m.g	scaffold_6:5395328-5404024	5.59749	10.8338	0.952685
Ciclev10006852m.g	scaffold_9:24201011-24203922	75.4861	146.141	0.953076
Ciclev10029283m.g	scaffold_8:22437299-22439022	4.08236	7.90387	0.953157
Ciclev10004466m.g	scaffold_9:1532444-1536650	0.19049	0.368886	0.953465
Ciclev10024152m.g	scaffold_3:3611667-3615544	84.3501	163.37	0.953685
Ciclev10001417m.g	scaffold_5:36324041-36326387	2.68453	5.19946	0.953691
Ciclev10032474m.g	scaffold_4:20917508-20920961	113.059	219.064	0.954282
Ciclev10015403m.g	scaffold_2:36147488-36152899	18.6264	36.1111	0.955089
Ciclev10032152m.g	scaffold_4:20402471-20407007	1.66278	3.22463	0.955533
Ciclev10015367m.g	scaffold_2:35825239-35830297	52.1574	101.164	0.955758
Ciclev10031519m.g	scaffold_4:23449172-23453507	19.0207	36.8982	0.955985
Ciclev10004218m.g	scaffold_9:827117-833780	8.16365	15.8375	0.956055
Ciclev10009325m.g	scaffold_1:1234554-1235706	2.92791	5.6811	0.956297
Ciclev10016176m.g	scaffold_2:26623795-26627893	37.3273	72.4414	0.956582
Ciclev10012635m.g	scaffold_6:18451375-18452564	20.8672	40.499	0.956646

Ciclev10033677m.g	scaffold_4:21277783-21282582	1.82526	3.54248	0.956655
-	scaffold_2:35135128-35136788	1.03471	2.00859	0.956956
Ciclev10004343m.g	scaffold_9:16019969-16028847	11.7407	22.8043	0.957789
Ciclev10029190m.g	scaffold_8:2863477-2864361	34.9559	67.9053	0.957988
Ciclev10024764m.g	scaffold_7:20909143-20916408	20.5539	39.9418	0.958491
Ciclev10016976m.g	scaffold_2:33832515-33835764	2.48001	4.81969	0.958596
Ciclev10000294m.g	scaffold_5:37726071-37732015	6.60976	12.8456	0.958607
Ciclev10014235m.g	scaffold_2:24539105-24541966	2.77612	5.39678	0.959026
Ciclev10028614m.g,Ciclev10029419m.g	scaffold_8:21342049-21348646	11.5123	22.3857	0.959394
-	scaffold_8:5319270-5320406	4.34795	8.45586	0.959618
Ciclev10013933m.g	scaffold_10:185170-189831	5.81853	11.3171	0.95977
Ciclev10024503m.g	scaffold_3:45679580-45685821	0.816584	1.58827	0.95978
Ciclev10018862m.g	scaffold_3:1142954-1145685	2.36155	4.59496	0.960321
Ciclev10006159m.g	scaffold_9:16863322-16865884	101.725	197.935	0.96036
Ciclev10017173m.g	scaffold_2:220533-222401	0.643488	1.25227	0.960557
Ciclev10005701m.g	scaffold_9:1388154-1389685	33.2469	64.7065	0.960692
Ciclev10007338m.g	scaffold_1:22394279-22400807	3.93088	7.65411	0.961383
Ciclev10004525m.g	scaffold_9:5520874-5526394	39.2781	76.5045	0.961821
Ciclev10000154m.g	scaffold_5:33811595-33817196	24.7616	48.2618	0.962774
Ciclev10021037m.g	scaffold_3:4144938-4148359	356.279	694.53	0.963032
Ciclev10017789m.g	scaffold_2:10190564-10190975	26.2631	51.1986	0.963065
Ciclev10027712m.g	scaffold_8:16574010-16579819	1.76483	3.44064	0.963153
Ciclev10006626m.g	scaffold_9:30244180-30246642	0.346295	0.675195	0.963302
Ciclev10030276m.g	scaffold_8:22795443-22796355	1.46815	2.86458	0.964323
Ciclev10033163m.g	scaffold_4:22544604-22545846	28.3136	55.2718	0.965047
-	scaffold_5:28135815-28136390	3.23733	6.32513	0.966291
Ciclev10024458m.g	scaffold_3:35372249-35712253	0.216207	0.422502	0.966549
Ciclev10029161m.g	scaffold_8:24162046-24166715	0.672122	1.31386	0.967013
Ciclev10016520m.g	scaffold_2:8027601-8030614	3.68858	7.21238	0.967409
Ciclev10009286m.g	scaffold_1:25395687-25397278	0.493455	0.965275	0.968023
Ciclev10020681m.g	scaffold_3:39593063-39599737	10.3868	20.3205	0.968181
-	scaffold_6:22640156-22641392	18.2169	35.6394	0.968194
Ciclev10000918m.g	scaffold_5:34345967-34375442	5.17735	10.1304	0.968407
Ciclev10001892m.g	scaffold_5:40354838-40359170	5.20928	10.1937	0.968515
Ciclev10030757m.g	scaffold_4:14510770-14516327	10.5285	20.6097	0.969016
Ciclev10007590m.g	scaffold_1:6600969-6609338	37.9718	74.34	0.969208
Ciclev10025774m.g	scaffold_7:11532703-11547475	5.50649	10.7815	0.969353
Ciclev10004899m.g	scaffold_9:402203-407560	3.3044	6.47224	0.969875
Ciclev10031869m.g	scaffold_4:13821185-13824321	4.04293	7.93168	0.972226
Ciclev10000925m.g	scaffold_5:36251748-36253427	0.29728	0.583235	0.972253
Ciclev10011296m.g	scaffold_6:22152086-22156059	10.4367	20.4775	0.972372
Ciclev10007367m.g	scaffold_1:427144-433794	180.462	354.08	0.97238
-	scaffold_5:24327453-24329341	1.44005	2.82593	0.972604
Ciclev10016983m.g	scaffold_2:33859730-33862078	26.714	52.4266	0.972702
Ciclev10005284m.g	scaffold_9:214087-217850	9.76229	19.1675	0.97337
Ciclev10010717m.g	scaffold_1:16766047-17038798	1.13013	2.21926	0.97359
Ciclev10031823m.g	scaffold_4:20901475-20903241	19.9753	39.2281	0.973668
Ciclev10017289m.g	scaffold_2:8122076-8125478	35.4206	69.5641	0.973755
Ciclev10030393m.g	scaffold_8:7899188-7902959	3.63621	7.14152	0.973794
Ciclev10000733m.g	scaffold_5:33879329-33882234	0.761823	1.49641	0.973983
Ciclev10011679m.g	scaffold_6:25484656-25489755	1.54895	3.04268	0.974049
Ciclev10026083m.g	scaffold_7:8381923-8384520	4.63037	9.09987	0.974717
Ciclev10016196m.g	scaffold_2:30597997-30598935	0.594721	1.16896	0.974947
Ciclev10024906m.g,Ciclev10024916m.g	scaffold_7:11329166-11340059	4.38706	8.62514	0.975293

Ciclev10023309m.g	scaffold_3:42342716-42349162	10.0028	19.6677	0.975425
Ciclev10008637m.g	scaffold_1:27650520-27657546	1.64543	3.23586	0.975684
Ciclev10012004m.g	scaffold_6:24685225-24688395	8.53847	16.8013	0.976525
-	scaffold_1:3800435-3800805	7.49941	14.7609	0.976935
Ciclev10023948m.g	scaffold_3:49125461-49133697	8.30539	16.3502	0.977188
Ciclev10028942m.g	scaffold_8:21223661-21227017	4.68041	9.2167	0.977617
Ciclev10019266m.g	scaffold_3:3633886-3636246	6.02386	11.8624	0.977634
Ciclev10011315m.g	scaffold_6:17791806-17794647	0.286771	0.564817	0.977883
Ciclev10013887m.g	scaffold_6:12909847-12911223	1.32288	2.60675	0.978572
Ciclev10019846m.g	scaffold_3:2453911-2455600	1.26589	2.49489	0.978822
Ciclev10014786m.g	scaffold_2:27789142-27790943	17.5007	34.505	0.979391
Ciclev10000345m.g	scaffold_5:17707311-17710634	0.273032	0.538487	0.979843
Ciclev10019278m.g	scaffold_3:20773968-20775996	2.64359	5.21442	0.980007
Ciclev10021708m.g	scaffold_3:45936544-45937698	90.4861	178.545	0.980523
Ciclev10007347m.g	scaffold_1:1157850-1164891	10.5655	20.8479	0.98054
Ciclev10032167m.g	scaffold_4:23259051-23261023	40.0789	79.101	0.980853
Ciclev10006239m.g	scaffold_9:20360609-20494992	58.525	115.523	0.981062
Ciclev10010039m.g	scaffold_1:3258468-3261672	15.262	30.139	0.981686
Ciclev10028606m.g	scaffold_8:13938257-13953640	5.9792	11.8131	0.982363
Ciclev10017705m.g	scaffold_2:5975366-6022331	74.3531	146.906	0.982425
Ciclev10027888m.g	scaffold_8:56852-60066	8.08741	15.9792	0.982442
Ciclev10031112m.g	scaffold_4:13640935-13643024	3.25401	6.43038	0.982685
Ciclev10004776m.g	scaffold_9:7080248-7086989	85.4751	168.934	0.982886
Ciclev10006755m.g	scaffold_9:7309756-7312287	0.378028	0.74736	0.983313
Ciclev10004260m.g	scaffold_9:30803951-30807200	1.29522	2.56137	0.983716
Ciclev10019167m.g	scaffold_3:23737103-23744297	10.408	20.5827	0.983742
Ciclev10016229m.g	scaffold_2:34412151-34415059	12.6585	25.0362	0.983914
Ciclev10025241m.g	scaffold_7:9469560-9473686	2.3539	4.65802	0.984661
Ciclev10014715m.g	scaffold_2:21269910-21273936	0.840187	1.66263	0.984682
Ciclev10022271m.g	scaffold_3:42200298-42203934	19.2838	38.1614	0.984721
Ciclev10028341m.g	scaffold_8:5650287-5652512	3.19779	6.3306	0.985268
Ciclev10012292m.g	scaffold_6:10816139-10936716	292.548	579.222	0.985441
Ciclev10029496m.g	scaffold_8:23988855-23989764	13.7877	27.2994	0.985487
Ciclev10004570m.g	scaffold_9:11137652-11142760	2.56975	5.08935	0.985852
Ciclev10005369m.g	scaffold_9:23600427-23601504	0.760024	1.50621	0.986806
Ciclev10021133m.g	scaffold_3:44945314-44948012	7.3836	14.6366	0.987182
Ciclev10023587m.g	scaffold_3:39723028-39724187	0.407835	0.808822	0.987836
Ciclev10011570m.g	scaffold_6:20494595-20496114	1.84406	3.65733	0.987907
Ciclev10016995m.g	scaffold_2:33900204-33901184	31.6915	62.873	0.988345
Ciclev10001580m.g	scaffold_5:29180179-29184765	0.380581	0.755535	0.989297
Ciclev10023719m.g	scaffold_3:21179235-21180612	1.18051	2.34457	0.989915
Ciclev10019356m.g	scaffold_3:8669956-8673841	8.79564	17.4727	0.990242
Ciclev10004928m.g	scaffold_9:27982557-27984091	0.269848	0.536062	0.990255
Ciclev10029659m.g	scaffold_8:17214537-17218258	1.45177	2.88423	0.990374
-	scaffold_3:4666869-4667147	14.4697	28.7514	0.990598
Ciclev10008031m.g	scaffold_1:6301968-6304016	1.21587	2.41617	0.99073
Ciclev10005328m.g	scaffold_9:3922377-3926095	42.879	85.2572	0.991551
Ciclev10006517m.g	scaffold_9:10635378-10638957	1.02794	2.04401	0.991649
Ciclev10026393m.g	scaffold_7:2721714-2817730	6.90453	13.7312	0.991846
Ciclev10011254m.g	scaffold_6:23543720-23546710	14.0257	27.906	0.992502
Ciclev10027061m.g,Ciclev10027168m.g	scaffold_7:2036653-2039100	25.9857	51.7158	0.992886
Ciclev10006285m.g	scaffold_9:4663881-4697961	0.42438	0.844711	0.9931
-	scaffold_2:28401984-28402422	2.98946	5.95059	0.993145
Ciclev10024634m.g	scaffold_3:42379628-42381792	2.43807	4.85321	0.993202
Ciclev10006304m.g	scaffold_9:14647455-14649273	3.65165	7.27186	0.993776

Ciclev10000120m.g	scaffold_5:34008553-34016913	8.12407	16.1842	0.99431
Ciclev10007175m.g	scaffold_9:30817398-30818556	1.15746	2.30663	0.994828
Ciclev10031313m.g	scaffold_4:18874265-18878127	4.38316	8.73723	0.995206
Ciclev10028437m.g	scaffold_8:24053047-24054881	0.416316	0.83008	0.995571
Ciclev10020693m.g	scaffold_3:34837005-34838471	4.17951	8.33724	0.996235
Ciclev10004144m.g	scaffold_9:7653928-7671163	0.106967	0.213411	0.996478
Ciclev10013854m.g	scaffold_6:6168704-6176448	3.5062	6.99784	0.996999
Ciclev10006081m.g	scaffold_9:900722-902354	92.1987	184.018	0.99703
Ciclev10016145m.g	scaffold_2:29060835-29062676	0.460889	0.920234	0.99758
Ciclev10007697m.g	scaffold_1:23750663-23753403	19.4279	38.7924	0.997646
Ciclev10024663m.g	scaffold_3:27092412-27094486	206.096	411.598	0.99792
Ciclev10024213m.g	scaffold_3:49566472-49573241	1.16057	2.31806	0.998079
Ciclev10031688m.g	scaffold_4:23491316-23494241	1.3468	2.69089	0.998546
Ciclev10009067m.g	scaffold_1:18391461-18393694	14.0404	28.0566	0.998756
Ciclev10012191m.g	scaffold_6:20323639-20326161	3.71172	7.4172	0.998787
Ciclev10010914m.g	scaffold_6:18395419-18403183	4.2486	8.4993	1.00036
Ciclev10015173m.g	scaffold_2:29192387-29194491	4.42447	8.85205	1.00051
Ciclev10002180m.g	scaffold_5:34619621-34621368	9.00786	18.0226	1.00055
Ciclev10001438m.g	scaffold_5:41479362-41481770	9.73569	19.4797	1.00062
Ciclev10034003m.g	scaffold_4:14961548-14963518	0.942018	1.88551	1.00113
Ciclev10030653m.g	scaffold_4:21537707-21545270	77.6591	155.443	1.00116
Ciclev10031999m.g	scaffold_4:118380-122560	21.4426	42.9217	1.00123
Ciclev10020545m.g	scaffold_3:45343900-45347433	18.3847	36.8039	1.00135
Ciclev10011893m.g	scaffold_6:23969911-23973324	5.48335	10.9782	1.00151
Ciclev10018795m.g	scaffold_3:48969346-48975465	42.3871	84.8723	1.00167
Ciclev10023333m.g	scaffold_3:35372249-35712253	1.26975	2.54292	1.00194
Ciclev10033235m.g	scaffold_4:24651072-24655710	12.6273	25.3038	1.00281
Ciclev10025170m.g	scaffold_7:10483853-10489121	43.5492	87.2747	1.00292
Ciclev10011437m.g	scaffold_6:12316729-12318356	3.22975	6.47335	1.00309
Ciclev10030686m.g	scaffold_4:1448835-1455316	32.0802	64.3112	1.00339
Ciclev10000247m.g	scaffold_5:27463515-27469472	28.0725	56.2812	1.0035
Ciclev10025982m.g	scaffold_7:20568866-20570682	0.445584	0.893421	1.00364
Ciclev10032900m.g	scaffold_4:20007863-20008619	3.17732	6.37253	1.00406
Ciclev10015162m.g	scaffold_2:35422597-35425980	108.967	218.665	1.00483
Ciclev10006422m.g	scaffold_9:3835567-3836224	1.28514	2.57946	1.00514
-	scaffold_5:1552172-1984270	1.59676	3.20577	1.00553
Ciclev10025151m.g	scaffold_7:13128486-13174591	0.625466	1.25706	1.00705
Ciclev10032699m.g	scaffold_4:19155211-19156289	15.5251	31.2077	1.0073
Ciclev10000934m.g	scaffold_5:34345967-34375442	2.04671	4.11521	1.00766
Ciclev10025838m.g	scaffold_7:11502531-11504662	9.74827	19.6096	1.00834
Ciclev10013558m.g	scaffold_6:18948192-18951132	11.4375	23.0152	1.00882
Ciclev10030682m.g	scaffold_4:17709554-17712859	5.86512	11.8045	1.0091
Ciclev10007333m.g	scaffold_1:28176131-28185088	22.9318	46.1554	1.00915
Ciclev10018562m.g	scaffold_3:37103737-37111403	4.0513	8.1544	1.00919
Ciclev10026170m.g	scaffold_7:4121598-4124180	22.5979	45.4854	1.00921
Ciclev10010987m.g	scaffold_6:23085686-23089615	0.732077	1.47386	1.00954
Ciclev10004347m.g	scaffold_9:23454444-23457114	1.53193	3.08551	1.01016
Ciclev10016599m.g	scaffold_2:35497039-35504104	2.82146	5.68343	1.01032
Ciclev10004283m.g	scaffold_9:3858969-3863789	0.332083	0.668993	1.01045
Ciclev10001414m.g	scaffold_5:23484138-23485588	8.13211	16.3884	1.01097
Ciclev10004522m.g	scaffold_9:30219241-30225811	2.99299	6.03387	1.01149
Ciclev10024760m.g	scaffold_7:3850620-3863167	2.48622	5.01249	1.01157
Ciclev10010033m.g	scaffold_1:24405245-24407298	2.05034	4.13406	1.0117
Ciclev10015492m.g	scaffold_2:23762751-23765126	6.65603	13.4227	1.01194
Ciclev10022535m.g	scaffold_3:35327505-35329126	14.8466	29.9588	1.01285
Ciclev10018916m.g	scaffold_3:5716259-5719416	4.57389	9.23	1.01291

Ciclev10017848m.g	scaffold_2:20340312-20340724	0.74753	1.50906	1.01344
Ciclev10017987m.g	scaffold_2:9214894-9220152	3.06842	6.1945	1.01349
-	scaffold_5:28135337-28135622	26.5843	53.6977	1.01428
Ciclev10017356m.g	scaffold_2:645022-651088	0.587785	1.18729	1.01431
Ciclev10020820m.g	scaffold_3:39927104-39929040	1.75764	3.55034	1.01432
Ciclev10018655m.g	scaffold_3:23001025-23009675	33.12	66.9243	1.01483
Ciclev10000088m.g	scaffold_5:2485638-2492388	28.883	58.3743	1.01512
Ciclev10001449m.g	scaffold_5:36510764-36512399	5.07746	10.2625	1.0152
Ciclev10024861m.g	scaffold_7:10787932-10805299	5.69279	11.5063	1.01522
Ciclev10009914m.g	scaffold_1:12048207-12049095	58.4469	118.172	1.01569
Ciclev10011381m.g	scaffold_6:15747187-15751231	2.21236	4.47489	1.01626
Ciclev10010129m.g	scaffold_1:8617771-8620480	0.725878	1.46832	1.01637
Ciclev10017573m.g	scaffold_2:29009422-29010205	1.1223	2.27057	1.01659
Ciclev10018970m.g	scaffold_3:5776941-5786422	4.24075	8.58169	1.01694
Ciclev10018974m.g	scaffold_3:50267034-50276642	12.8131	25.9372	1.0174
Ciclev10007571m.g	scaffold_1:23581312-23584733	6.52615	13.2183	1.01823
Ciclev10001740m.g	scaffold_5:40265387-40267186	15.1363	30.6795	1.01927
Ciclev10031498m.g	scaffold_4:18901362-18903987	74.9142	151.895	1.01976
Ciclev10032310m.g	scaffold_4:23833095-23836044	0.276308	0.560582	1.02065
Ciclev10033400m.g	scaffold_4:18515567-18675155	1.41425	2.86971	1.02087
Ciclev10013660m.g	scaffold_6:20416758-20421738	2.76608	5.6131	1.02095
Ciclev10030633m.g	scaffold_4:20294239-20301426	3.16517	6.42624	1.02169
-	scaffold_1:26830809-26831290	15.0359	30.536	1.0221
Ciclev10007317m.g	scaffold_1:14109498-14121586	7.85358	15.9504	1.02217
Ciclev10028791m.g	scaffold_8:23390336-23397139	20.0053	40.6309	1.02219
Ciclev10011729m.g	scaffold_6:18294990-18296658	0.804406	1.63387	1.0223
Ciclev10028698m.g	scaffold_8:17201721-17206594	18.1187	36.8018	1.0223
Ciclev10001835m.g	scaffold_5:30655730-30659789	38.4055	78.0129	1.0224
Ciclev10011195m.g	scaffold_6:68521-70633	0.20786	0.42228	1.02259
Ciclev10032611m.g	scaffold_4:3063806-3064887	1.11439	2.26493	1.02321
Ciclev10015311m.g	scaffold_2:29214221-29216105	20.1627	40.98	1.02323
Ciclev10027252m.g	scaffold_7:17545631-17550302	0.361425	0.734836	1.02373
Ciclev10013727m.g	scaffold_6:1134443-1137227	0.168319	0.342231	1.02377
Ciclev10029923m.g	scaffold_8:13485857-13486664	5.03905	10.2492	1.02428
Ciclev10009761m.g	scaffold_1:25604508-25605857	9.0666	18.4455	1.02464
Ciclev10005808m.g	scaffold_9:5565051-5566296	0.410107	0.83484	1.0255
Ciclev10027612m.g	scaffold_7:8282443-8283871	0.356264	0.725478	1.02599
Ciclev10029797m.g	scaffold_8:18402680-18408394	13.0117	26.5113	1.0268
Ciclev10018412m.g	scaffold_2:573823-592599	3.43428	6.99738	1.02681
Ciclev10008984m.g	scaffold_1:27897953-27901766	4.25569	8.67375	1.02726
Ciclev10014682m.g	scaffold_2:30508202-30510699	4.44501	9.05986	1.0273
Ciclev10019184m.g	scaffold_3:680834-684433	110.777	225.796	1.02737
-	scaffold_9:2283946-2284437	2.41498	4.92354	1.02768
Ciclev10018720m.g	scaffold_3:2202467-2207986	8.56537	17.4764	1.02882
Ciclev10022495m.g	scaffold_3:9153392-9154297	4.06525	8.29739	1.02931
Ciclev10025542m.g	scaffold_7:10464299-10466430	7.35648	15.0191	1.02971
Ciclev10011723m.g	scaffold_6:15380796-15382751	6.02768	12.3078	1.0299
Ciclev10014051m.g	scaffold_2:6747733-6759030	5.70782	11.6631	1.03094
Ciclev10025166m.g	scaffold_7:1869042-1870911	4.49207	9.18197	1.03142
Ciclev10006235m.g	scaffold_9:3135965-3136838	41.3741	84.5896	1.03175
Ciclev10020495m.g	scaffold_3:35938548-35940183	6.90563	14.1187	1.03176
Ciclev10024705m.g	scaffold_7:6345751-6352245	53.1901	108.872	1.0334
Ciclev10021342m.g	scaffold_3:9330367-9331940	5.20748	10.6594	1.03347
Ciclev10021134m.g	scaffold_3:6510695-6513519	51.6092	105.686	1.03408
Ciclev10003867m.g	scaffold_5:23818533-23819802	5.2017	10.657	1.03475
-	scaffold_5:10404740-10405290	3.4798	7.13286	1.03547

-	scaffold_4:15291388-15291659	15.8435	32.477	1.03552
Ciclev10028746m.g	scaffold_8:18970031-18971180	0.496681	1.01828	1.03575
Ciclev10030204m.g	scaffold_8:13177010-13178480	0.258366	0.52972	1.03582
Ciclev10023433m.g,Ciclev10024146m.g	scaffold_3:45910746-45914762	6.83726	14.022	1.0362
Ciclev10015219m.g	scaffold_2:7607950-7610032	2.96924	6.08993	1.03633
Ciclev10013613m.g,Ciclev10013901m.g	scaffold_6:17219987-17222666	1.58424	3.25092	1.03706
Ciclev10009768m.g	scaffold_1:27321745-27324279	5.3592	10.9984	1.03721
Ciclev10025746m.g	scaffold_7:2137372-2140497	1.62439	3.33368	1.03722
Ciclev10015796m.g	scaffold_2:23819705-23822719	5.47039	11.2302	1.03767
-	scaffold_8:1620413-1622704	11.3458	23.2924	1.03771
Ciclev10018596m.g	scaffold_3:48518563-48523574	34.9688	71.82	1.03832
Ciclev10000257m.g	scaffold_5:37071269-37075490	0.13708	0.281549	1.03837
Ciclev10014496m.g,Ciclev10014504m.g	scaffold_2:32352768-32363488	38.8321	79.7693	1.03858
Ciclev10017105m.g	scaffold_2:22750224-22751793	43.3922	89.1398	1.03863
Ciclev10029919m.g	scaffold_8:4151702-4152956	4.93911	10.1464	1.03864
Ciclev10004042m.g	scaffold_5:10427531-10584700	0.173185	0.355818	1.03883
Ciclev10024776m.g	scaffold_7:1976183-1984531	7.49198	15.394	1.03895
Ciclev10010788m.g	scaffold_1:26915371-26917713	1.40915	2.8961	1.03929
Ciclev10019795m.g	scaffold_3:46811987-46814341	1.78387	3.66676	1.03949
Ciclev10011509m.g	scaffold_6:18406855-18412391	42.226	86.8285	1.04004
Ciclev10011887m.g	scaffold_6:25504762-25507198	22.7434	46.768	1.04007
Ciclev10025110m.g	scaffold_7:2718093-2720097	1.09418	2.25023	1.04022
Ciclev10031810m.g	scaffold_4:11959424-11960988	1.42628	2.93403	1.04063
Ciclev10006593m.g	scaffold_9:3751951-3754713	1.05731	2.17536	1.04086
Ciclev10020980m.g	scaffold_3:4747156-4751029	6.49896	13.3751	1.04127
Ciclev10004394m.g	scaffold_9:4963066-4967349	37.7629	77.7299	1.0415
Ciclev10030863m.g	scaffold_4:13910898-13916883	294.326	606.068	1.04207
Ciclev10017684m.g	scaffold_2:14701585-14702197	1.79888	3.70424	1.04208
Ciclev10008285m.g	scaffold_1:28686869-28690073	7.99014	16.4685	1.04342
Ciclev10008330m.g	scaffold_1:4575674-4580798	5.65917	11.6659	1.04364
Ciclev10001428m.g	scaffold_5:5993469-5995467	0.990414	2.04222	1.04404
Ciclev10031280m.g	scaffold_4:2236038-2238244	1.32287	2.72793	1.04414
Ciclev10019944m.g	scaffold_3:47328360-47334187	2.4699	5.0943	1.04443
Ciclev10024277m.g	scaffold_3:50376207-50377890	1.0983	2.26597	1.04486
Ciclev10011660m.g	scaffold_6:11954096-11958888	10.3085	21.2698	1.04498
Ciclev10018485m.g	scaffold_3:35765200-35775783	1.43269	2.95635	1.04509
Ciclev10021244m.g	scaffold_3:48917308-48920333	21.1977	43.7687	1.04599
Ciclev10027600m.g	scaffold_7:9108907-9112268	0.156174	0.32247	1.04602
Ciclev10001419m.g	scaffold_5:2069219-2072819	3.55118	7.33355	1.04621
Ciclev10013430m.g	scaffold_6:18307254-18308912	2.96971	6.13301	1.04627
Ciclev10027647m.g	scaffold_1822:2778-3447	126.129	260.52	1.04649
Ciclev10006073m.g	scaffold_9:2907530-2909473	3.39611	7.01694	1.04696
Ciclev10002888m.g	scaffold_5:40971195-40971829	1.2086	2.49772	1.04728
Ciclev10012219m.g	scaffold_6:19748310-19752120	6.06999	12.5478	1.04767
Ciclev10027962m.g	scaffold_8:8671548-8673629	0.355564	0.735635	1.04888
Ciclev10012152m.g	scaffold_6:24874839-24876388	8.38908	17.359	1.0491
Ciclev10014397m.g	scaffold_2:33289743-33294803	6.60217	13.6658	1.04955
Ciclev10008262m.g	scaffold_1:24150425-24154226	6.83649	14.1545	1.04993
Ciclev10005863m.g	scaffold_9:26164943-26165955	56.6543	117.329	1.0503
Ciclev10031053m.g	scaffold_4:20155379-20159762	7.69184	15.937	1.05098
Ciclev10019301m.g	scaffold_3:972288-976751	2.31812	4.80418	1.05133
Ciclev10010902m.g	scaffold_6:20876276-20883375	29.1442	60.4005	1.05135
Ciclev10019838m.g	scaffold_3:46069753-46076045	11.1828	23.1769	1.05141

Ciclev10028118m.g	scaffold_8:3530536-3532841	2.46566	5.11191	1.05189
Ciclev10014727m.g	scaffold_2:15106721-15111727	34.6417	71.8391	1.05226
Ciclev10029506m.g	scaffold_8:23995447-23996072	6.1153	12.6848	1.0526
Ciclev10000988m.g	scaffold_5:29467470-29469049	0.837815	1.7381	1.05281
Ciclev10025235m.g	scaffold_7:20271437-20274020	1.6779	3.48487	1.05445
Ciclev10032212m.g	scaffold_4:5264934-5265855	4.14744	8.61435	1.05452
Ciclev10010607m.g	scaffold_1:27770698-27773026	13.7915	28.6635	1.05544
Ciclev10011398m.g	scaffold_6:18610805-18612989	5.70985	11.869	1.05568
Ciclev10022193m.g	scaffold_3:29111936-29113841	99.9878	207.87	1.05586
Ciclev10004495m.g	scaffold_9:16274122-16276257	0.924454	1.92194	1.05589
Ciclev10027787m.g	scaffold_8:263631-266502	1.27559	2.65266	1.05628
Ciclev10012130m.g	scaffold_6:20731407-20732679	101.02	210.15	1.05678
Ciclev10012014m.g	scaffold_6:18771349-18773504	3.11789	6.49017	1.05768
Ciclev10019009m.g	scaffold_3:40730256-40736911	5.32835	11.0949	1.05814
-	scaffold_5:31333200-31391489	1.16877	2.43447	1.05862
Ciclev10015824m.g	scaffold_2:31881150-31884001	8.11081	16.8962	1.05878
Ciclev10016686m.g	scaffold_2:23239276-23240481	0.729177	1.52043	1.06014
Ciclev10016864m.g	scaffold_2:27366724-27369317	8.82053	18.3939	1.06029
Ciclev10004580m.g	scaffold_9:30368968-30372269	1.12767	2.35164	1.06032
Ciclev10019480m.g	scaffold_3:11488634-11497113	39.9753	83.3837	1.06066
Ciclev10008188m.g	scaffold_1:25502041-25504073	7.59486	15.8513	1.0615
Ciclev10020719m.g	scaffold_3:11000796-11001900	0.853265	1.78099	1.06161
Ciclev10015879m.g	scaffold_2:33578013-33579268	0.763339	1.59332	1.06164
Ciclev10026357m.g	scaffold_7:3456104-3460607	30.2928	63.2519	1.06214
Ciclev10023621m.g	scaffold_3:7504855-7506322	2.35102	4.91285	1.06327
Ciclev10028580m.g	scaffold_8:20596435-20600624	36.0895	75.4167	1.06331
Ciclev10031268m.g,Ciclev10031272m.g	scaffold_4:17655893-17668391	164.849	344.51	1.0634
Ciclev10026821m.g	scaffold_7:2098374-2102116	3.33058	6.96511	1.06437
-	scaffold_2:26983531-26990109	10.2995	21.5496	1.06508
Ciclev10000426m.g	scaffold_5:34653390-34657430	1.9718	4.12706	1.0656
Ciclev10024853m.g	scaffold_7:8186885-8191388	7.64742	16.02	1.06683
Ciclev10015030m.g	scaffold_2:29492431-29498475	6.93406	14.5301	1.06727
Ciclev10014749m.g	scaffold_2:12370900-12374131	56.6042	118.619	1.06735
Ciclev10032547m.g	scaffold_4:24434004-24435526	122.234	256.383	1.06865
Ciclev10032374m.g	scaffold_4:20931219-20932775	4.70533	9.87628	1.06967
-	scaffold_3:40504072-40719738	5.81883	12.2152	1.06988
Ciclev10018385m.g	scaffold_2:28585075-28586386	2.50296	5.25463	1.06996
Ciclev10011848m.g	scaffold_6:8878181-8884354	79.089	166.074	1.07028
Ciclev10013510m.g,Ciclev10013700m.g	scaffold_6:18089900-18094713	25.3925	53.344	1.07093
Ciclev10025337m.g	scaffold_7:5305999-5311944	4.88476	10.262	1.07095
Ciclev10000226m.g	scaffold_5:36789179-36794175	7.35375	15.4575	1.07176
Ciclev10018015m.g	scaffold_2:35934588-35935920	6.54533	13.7616	1.07212
Ciclev10006846m.g	scaffold_9:30947714-30951799	0.733082	1.54166	1.07244
Ciclev10010522m.g	scaffold_1:22149810-22152338	1.52136	3.2011	1.07321
Ciclev10006058m.g	scaffold_9:16742484-16743851	16.4892	34.7028	1.07353
Ciclev10018683m.g	scaffold_3:43594637-43605172	2.62552	5.52597	1.07362
Ciclev10010514m.g	scaffold_1:5649979-5655677	31.0421	65.339	1.07372
Ciclev10024855m.g	scaffold_7:10965344-10968310	1.61087	3.39152	1.07409
Ciclev10028026m.g	scaffold_8:3135301-3137607	9.98275	21.0267	1.07472
Ciclev10023781m.g	scaffold_3:41277046-41278770	0.470691	0.99178	1.07524
Ciclev10011063m.g	scaffold_6:20927562-20940324	20.8117	43.8559	1.07537
Ciclev10020776m.g	scaffold_3:46998744-47002683	14.3044	30.1681	1.07656
Ciclev10018219m.g	scaffold_2:35590452-35594163	0.446658	0.942023	1.07659
Ciclev10002425m.g	scaffold_5:42093830-42095266	5.76442	12.1651	1.0775

Ciclev10030449m.g	scaffold_978:1386-4908	29.6242	62.528	1.07773
Ciclev10033840m.g	scaffold_4:23479258-23479825	1.33823	2.8248	1.07782
Ciclev10011224m.g	scaffold_6:24437228-24439391	6.19672	13.0833	1.07815
Ciclev10024180m.g	scaffold_3:3909933-3914931	2.67164	5.64137	1.07832
Ciclev10028230m.g	scaffold_8:1080301-1085905	0.777715	1.64244	1.07853
Ciclev10031208m.g	scaffold_4:25164475-25166250	0.893085	1.88654	1.07887
Ciclev10008350m.g	scaffold_1:13194347-13198000	11.6652	24.6453	1.0791
Ciclev10025528m.g	scaffold_7:1736345-1738113	5.7307	12.111	1.07953
Ciclev10030533m.g	scaffold_4:16253274-16260236	0.688659	1.45543	1.07958
Ciclev10028929m.g,Ciclev10030040m.g	scaffold_8:20950309-21005489	9.14693	19.3337	1.07976
Ciclev10011545m.g	scaffold_6:19410354-19412887	1.16597	2.46489	1.07999
Ciclev10008705m.g	scaffold_1:24998677-25004726	0.135722	0.287043	1.08061
Ciclev10010761m.g	scaffold_1:3468880-3472058	2.52761	5.34854	1.08137
Ciclev10028766m.g	scaffold_8:10574672-10578797	25.8661	54.7477	1.08174
Ciclev10002536m.g	scaffold_5:30514545-30517126	9.56566	20.2468	1.08176
Ciclev10015230m.g	scaffold_2:25879361-25882448	4.10668	8.69437	1.08211
Ciclev10008260m.g	scaffold_1:2893290-2896777	11.3369	24.0098	1.08259
Ciclev10010974m.g	scaffold_6:20354019-20360573	0.299691	0.634762	1.08274
Ciclev10023003m.g	scaffold_3:5560632-5562922	11.9977	25.4159	1.08297
Ciclev10000576m.g	scaffold_5:15998433-16007240	9.95497	21.0961	1.08349
-	scaffold_2:8844235-8856366	44.2811	93.9133	1.08464
Ciclev10026574m.g	scaffold_7:8571966-8574224	0.540594	1.14653	1.08466
Ciclev10028195m.g	scaffold_8:5496423-5502056	24.5359	52.0484	1.08496
Ciclev10030516m.g	scaffold_4:632094-638772	8.62624	18.3042	1.08537
Ciclev10017905m.g	scaffold_2:11922956-11925717	3.04543	6.46623	1.08628
Ciclev10013576m.g	scaffold_6:9146212-9146431	4.77347	10.1383	1.08671
Ciclev10001741m.g	scaffold_5:33038071-33039438	4.09002	8.69293	1.08774
Ciclev10000445m.g	scaffold_5:38078578-38080699	1.16431	2.47481	1.08784
Ciclev10023076m.g	scaffold_3:18652913-18653886	4.01453	8.5496	1.09062
Ciclev10003712m.g	scaffold_5:36794935-36796792	1.41686	3.01809	1.09094
Ciclev10018509m.g	scaffold_3:27365801-27371376	0.296409	0.631539	1.09128
Ciclev10030245m.g	scaffold_8:23310114-23311691	10.1883	21.7088	1.09136
Ciclev10026732m.g	scaffold_7:7879715-7880713	8.25771	17.5978	1.09158
Ciclev10010966m.g	scaffold_6:24098001-24112094	4.6277	9.86251	1.09166
Ciclev10025131m.g	scaffold_7:10810162-10816510	2.76663	5.90303	1.09333
Ciclev10030248m.g	scaffold_8:2541369-2542050	1.69905	3.62586	1.09359
Ciclev10030179m.g	scaffold_8:10593474-10661453	14.3623	30.6539	1.09379
Ciclev10018364m.g	scaffold_2:29699277-29700642	1.97703	4.2198	1.09384
Ciclev10011236m.g	scaffold_6:23569320-23571882	8.33008	17.7818	1.094
-	scaffold_7:20186368-20187350	1.40446	2.99812	1.09404
Ciclev10001773m.g	scaffold_5:9486034-9487216	13.1727	28.1203	1.09406
-	scaffold_4:7122100-7123270	2.08691	4.45723	1.09478
Ciclev10022741m.g	scaffold_3:36573009-36577672	13.3108	28.4345	1.09504
Ciclev10032030m.g	scaffold_4:3437576-3441367	5.05337	10.7964	1.09523
Ciclev10002449m.g	scaffold_5:33818577-33821459	5.97719	12.7758	1.09588
Ciclev10004565m.g	scaffold_9:10236647-10243890	4.96712	10.62	1.0963
Ciclev10014623m.g	scaffold_2:27610483-27613965	94.1674	201.41	1.09684
-	scaffold_9:637254-641446	11.7214	25.0731	1.09699
Ciclev10015289m.g	scaffold_2:19897882-19899288	0.37351	0.799339	1.09766
Ciclev10001016m.g	scaffold_5:40841687-40843369	20.0902	43.0057	1.09804
Ciclev10008844m.g	scaffold_1:25739601-25743738	0.490186	1.04979	1.0987
Ciclev10000434m.g	scaffold_5:41132581-41135791	6.00908	12.8731	1.09914
Ciclev10025533m.g	scaffold_7:17412019-17414421	7.60354	16.289	1.09915
Ciclev10010445m.g	scaffold_1:1617669-1620134	0.957763	2.05249	1.09963
Ciclev10010083m.g	scaffold_1:18574114-18576781	4.9941	10.7044	1.09991

Ciclev10014999m.g	scaffold_2:25772797-25775870	3.68838	7.90905	1.10052
Ciclev10031020m.g,Ciclev10033569m.g	scaffold_4:15087930-15266082	18.5357	39.7488	1.1006
Ciclev10030606m.g	scaffold_4:14433269-14441599	14.882	31.9134	1.1006
Ciclev10000035m.g	scaffold_5:35361068-35371122	0.0855602	0.183543	1.1011
Ciclev10023505m.g	scaffold_3:22180064-22184610	0.72666	1.55924	1.10149
Ciclev10030158m.g	scaffold_8:23991834-23992880	0.503099	1.07995	1.10205
Ciclev10003362m.g	scaffold_5:41424594-41428360	0.597568	1.28292	1.10226
Ciclev10019048m.g	scaffold_3:48209381-48212809	4.51948	9.70447	1.10249
Ciclev10028069m.g	scaffold_8:19574428-19578315	1.85925	3.99235	1.10252
Ciclev10011036m.g	scaffold_6:21300611-21303565	3.70155	7.94855	1.10256
Ciclev10019550m.g	scaffold_3:7432133-7433804	1.57962	3.39298	1.10297
Ciclev10018932m.g	scaffold_3:44322171-44325084	1.80991	3.88912	1.10353
Ciclev10020129m.g	scaffold_3:8346284-8348461	1.72637	3.71104	1.10408
Ciclev10026265m.g	scaffold_7:6290648-6292417	3.95804	8.51015	1.1044
Ciclev10014470m.g	scaffold_2:16713444-16717134	3.02068	6.49861	1.10526
Ciclev10004713m.g	scaffold_9:31018835-31027361	16.7025	35.9392	1.1055
Ciclev10004262m.g	scaffold_9:4663881-4697961	22.1732	47.7212	1.10581
Ciclev10030145m.g	scaffold_8:6805620-6806699	0.412043	0.886881	1.10594
Ciclev10000271m.g	scaffold_5:2073605-2076567	10.2748	22.118	1.10611
Ciclev10028637m.g	scaffold_8:19538137-19541255	16.2774	35.0468	1.10641
Ciclev10018905m.g	scaffold_3:1573985-1576505	2.07575	4.47039	1.10677
Ciclev10000176m.g	scaffold_5:254183-279075	21.4059	46.1131	1.10716
Ciclev10027320m.g	scaffold_7:5047523-5048544	3.98889	8.59593	1.10767
Ciclev10024055m.g	scaffold_3:18980102-18981479	1.54454	3.32853	1.1077
Ciclev10007708m.g	scaffold_1:1806178-1809814	27.8332	60.0601	1.1096
Ciclev10019473m.g	scaffold_3:9713108-9718085	5.65056	12.1934	1.10963
Ciclev10025107m.g	scaffold_7:19334465-19388718	1.35553	2.92526	1.10971
Ciclev10011623m.g	scaffold_6:22819211-22821782	2.48469	5.36468	1.11043
-	scaffold_8:11061519-11062022	5.78525	12.4918	1.11054
Ciclev10031171m.g	scaffold_4:21831619-21837361	50.8326	109.916	1.11258
Ciclev10024145m.g	scaffold_3:35372249-35712253	0.488144	1.05714	1.11478
Ciclev10020309m.g	scaffold_3:48164031-48167425	2.74634	5.9499	1.11536
-	scaffold_5:32546088-32620214	3.80498	8.24418	1.11549
Ciclev10018567m.g	scaffold_3:37381091-37420501	4.37388	9.48437	1.11664
Ciclev10013618m.g	scaffold_6:1039888-1047952	0.0806384	0.174939	1.11732
Ciclev10009607m.g	scaffold_1:24574963-24582425	9.66887	20.9784	1.11748
-	scaffold_5:10427531-10584700	14.729	31.9627	1.11773
Ciclev10019883m.g	scaffold_3:21940550-21944032	1.40121	3.04338	1.11901
Ciclev10026374m.g	scaffold_7:3988365-3989679	63.4306	137.778	1.1191
Ciclev10019764m.g	scaffold_3:626899-630720	4.44541	9.65871	1.11951
Ciclev10029459m.g	scaffold_8:1491361-1556980	0.613756	1.33376	1.11976
Ciclev10034011m.g	scaffold_4:22944705-22960987	12.6794	27.5665	1.12043
Ciclev10014583m.g	scaffold_2:29414141-29418691	0.637252	1.38635	1.12136
Ciclev10028076m.g,Ciclev10030299m.g	scaffold_8:6944685-7159404	33.11	72.049	1.12171
Ciclev10004390m.g	scaffold_9:6272841-6279562	0.707626	1.54024	1.1221
Ciclev10021546m.g	scaffold_3:3576674-3577847	24.6245	53.6511	1.12351
Ciclev10030098m.g	scaffold_8:843952-844828	10.31	22.4705	1.12398
Ciclev10020078m.g	scaffold_3:3629890-3633557	0.428233	0.933471	1.12421
Ciclev10001032m.g	scaffold_5:31919716-31923369	3.33695	7.2779	1.12499
Ciclev10023150m.g	scaffold_3:38300970-38301733	0.355392	0.775128	1.12502
Ciclev10024968m.g	scaffold_7:891045-894710	1.83401	4.00198	1.12571
Ciclev10017225m.g	scaffold_2:4682812-4683331	1.30519	2.84934	1.12636
Ciclev10009004m.g	scaffold_1:9401460-9403208	66.5171	145.242	1.12666
-	scaffold_3:1717149-1717448	15.1852	33.1688	1.12716

Ciclev10008511m.g	scaffold_1:23111732-23116072	12.5026	27.3131	1.12737
Ciclev10004298m.g	scaffold_9:4494462-4503620	8.76879	19.1567	1.1274
Ciclev10020886m.g	scaffold_3:9810084-9812211	7.30801	15.9759	1.12835
Ciclev10004726m.g	scaffold_9:27688895-27690633	2.36354	5.16708	1.1284
Ciclev10001842m.g	scaffold_5:39096918-39102158	3.29104	7.19976	1.1294
Ciclev10028770m.g	scaffold_8:10684115-10718089	119.357	261.155	1.12962
Ciclev10001250m.g	scaffold_5:36490751-36492636	1.03509	2.26538	1.13
Ciclev10032702m.g	scaffold_4:17361709-17362822	128.525	281.352	1.13032
Ciclev10031011m.g	scaffold_4:23919073-23921396	2.01967	4.42184	1.13052
Ciclev10011168m.g	scaffold_6:1128137-1130789	1.54603	3.38501	1.13059
Ciclev10029362m.g	scaffold_8:3129971-3134221	10.7803	23.612	1.13112
Ciclev10004242m.g	scaffold_9:30999298-31006503	9.80418	21.4945	1.1325
Ciclev10011867m.g	scaffold_6:17461338-17462663	2.32216	5.09823	1.13453
Ciclev10023429m.g	scaffold_3:8682990-8683860	0.179392	0.393862	1.13457
Ciclev10030965m.g	scaffold_4:22441484-22444695	3.11934	6.85177	1.13523
Ciclev10032165m.g	scaffold_4:23772801-23774659	11.117	24.4281	1.13577
Ciclev10016066m.g	scaffold_2:29736310-29739662	1.09834	2.41367	1.13591
Ciclev10026956m.g	scaffold_7:6458770-6463886	0.30332	0.66713	1.13713
Ciclev10020041m.g	scaffold_3:42980878-42986842	1.98674	4.3708	1.13749
Ciclev10008215m.g	scaffold_1:7281197-7282662	0.738048	1.62459	1.13829
Ciclev10032075m.g	scaffold_4:18912921-18917225	0.739194	1.62722	1.13838
Ciclev10003121m.g	scaffold_5:25216207-25218151	0.942185	2.07425	1.13851
Ciclev10000024m.g	scaffold_5:8636099-8657848	9.74515	21.4577	1.13874
Ciclev10018233m.g	scaffold_2:29751763-29752216	0.587491	1.29392	1.13911
Ciclev10014316m.g	scaffold_2:23865787-23871757	45.7361	100.741	1.13924
Ciclev10000587m.g	scaffold_5:16129552-16133405	17.704	39.0071	1.13966
Ciclev10011492m.g	scaffold_6:21490919-21492479	1.93956	4.27652	1.14071
Ciclev10026550m.g	scaffold_7:122764-125623	0.635634	1.40206	1.14128
Ciclev10004678m.g	scaffold_9:23210512-23213164	12.6728	27.9731	1.1423
Ciclev10013666m.g	scaffold_6:24196883-24201974	7.65061	16.9034	1.14367
Ciclev10024069m.g	scaffold_3:37585248-37588412	0.604899	1.33682	1.14404
Ciclev10007502m.g	scaffold_1:25903934-25909511	12.8126	28.328	1.14467
Ciclev10025737m.g	scaffold_7:12339218-12345195	6.55662	14.4972	1.14475
Ciclev10007345m.g	scaffold_1:136601-149015	6.79246	15.0257	1.14542
Ciclev10013605m.g	scaffold_6:13451896-13492783	5.2463	11.6082	1.14577
Ciclev10001399m.g,Ciclev10001446m.g	scaffold_5:36112257-36158452	45.6267	100.979	1.1461
Ciclev10020218m.g	scaffold_3:39373367-39379398	11.8279	26.1859	1.1466
Ciclev10017893m.g	scaffold_2:24297920-24301094	0.105352	0.233272	1.14679
Ciclev10012483m.g,Ciclev10013117m.g,Ciclev10013415m.g	scaffold_6:18579811-18601057	6.98753	15.4771	1.14728
Ciclev10011943m.g	scaffold_6:23290587-23296030	1.06942	2.37099	1.14866
Ciclev10022425m.g	scaffold_3:50850795-50851945	1.9268	4.27347	1.1492
Ciclev10011720m.g,Ciclev10013238m.g	scaffold_6:16246772-16266831	168.916	374.726	1.14953
Ciclev10000889m.g	scaffold_5:41081415-41083138	8.29387	18.4002	1.14961
Ciclev10024579m.g	scaffold_3:50089580-50089973	6.45449	14.3198	1.14963
-	scaffold_9:4715823-4717699	4.96402	11.0158	1.14999
Ciclev10030082m.g	scaffold_8:6411572-6413114	0.261352	0.580077	1.15025
Ciclev10005915m.g	scaffold_9:4037319-4039184	4.93351	10.9552	1.15093
Ciclev10005204m.g	scaffold_9:27148019-27150148	13.396	29.7501	1.15109
Ciclev10033965m.g	scaffold_4:24526777-24529276	1.11642	2.4801	1.15152
Ciclev10004281m.g	scaffold_9:3443237-3447919	3.13722	6.9764	1.153
Ciclev10025313m.g	scaffold_7:6977243-6983565	1.91713	4.26794	1.15459
-	scaffold_9:9764154-9764347	28.7055	63.9207	1.15496

Ciclev10015143m.g	scaffold_2:11532048-11575076	2.93499	6.5364	1.15514
Ciclev10033256m.g	scaffold_4:13582991-13583996	4.85587	10.8147	1.1552
Ciclev10000852m.g	scaffold_5:40582687-40584770	0.627774	1.39849	1.15555
Ciclev10023584m.g	scaffold_3:24347492-24351466	0.147927	0.32954	1.15556
Ciclev10021874m.g	scaffold_3:10951100-10959102	12.8319	28.5861	1.15558
Ciclev10012468m.g	scaffold_6:20276330-20278559	0.22544	0.502269	1.15572
Ciclev10031521m.g	scaffold_4:20922679-20926115	6.31058	14.0643	1.1562
-	scaffold_5:17029791-17030312	4.08852	9.11237	1.15625
Ciclev10033996m.g	scaffold_4:354241-358913	0.881532	1.9655	1.15681
Ciclev10006211m.g	scaffold_9:26578135-26578846	0.441645	0.984959	1.15718
Ciclev10029360m.g	scaffold_8:3134317-3135122	9.48164	21.1534	1.15768
Ciclev10026723m.g	scaffold_7:5810179-5810922	3.01314	6.72634	1.15856
Ciclev10000693m.g	scaffold_5:36863308-36868015	4.58833	10.2466	1.1591
Ciclev10006074m.g	scaffold_9:1295418-1296297	1.77695	3.96873	1.15927
Ciclev10014514m.g	scaffold_2:6800958-6813236	4.89291	10.9285	1.15933
Ciclev10023178m.g	scaffold_3:18648425-18650443	0.624575	1.39567	1.16001
Ciclev10011046m.g	scaffold_6:2846626-2851983	33.3821	74.6456	1.16098
Ciclev10020543m.g	scaffold_3:24598361-24600642	0.724635	1.62041	1.16103
Ciclev10024315m.g	scaffold_3:35372249-35712253	0.808641	1.80826	1.16103
Ciclev10010993m.g	scaffold_6:21898329-21902563	2.19003	4.8994	1.16166
Ciclev10000527m.g	scaffold_5:32937344-32939324	0.903291	2.02438	1.16422
Ciclev10030422m.g	scaffold_8:3999903-4000312	5.08795	11.4041	1.16439
Ciclev10024461m.g	scaffold_3:50406001-50412944	6.6525	14.9119	1.1645
Ciclev10018707m.g	scaffold_3:24026647-24169357	0.121033	0.271375	1.16489
-	scaffold_8:19465708-19466223	1.11063	2.49079	1.16522
Ciclev10026051m.g	scaffold_7:21043065-21044289	5.31859	11.9347	1.16605
Ciclev10011107m.g	scaffold_6:10077278-10081077	4.23773	9.51271	1.16656
Ciclev10014836m.g	scaffold_2:434536-439663	43.8373	98.4164	1.16674
Ciclev10008150m.g	scaffold_1:1623342-1627229	1.5407	3.4605	1.16739
Ciclev10023278m.g	scaffold_3:16688556-16695010	5.48031	12.3141	1.16798
Ciclev10000981m.g	scaffold_5:34994036-35002166	45.2648	101.722	1.16817
Ciclev10003997m.g	scaffold_5:25221155-25545745	14.8045	33.2721	1.16827
Ciclev10016164m.g	scaffold_2:22705496-22707933	1.07056	2.40714	1.16896
Ciclev10007201m.g	scaffold_9:29432438-29439510	1.94351	4.37123	1.16937
Ciclev10023672m.g	scaffold_3:11687448-12154880	7.0057	15.7587	1.16954
Ciclev10002302m.g,Ciclev10003933m.g	scaffold_5:33432127-33472849	10.1038	22.7319	1.16982
Ciclev10030743m.g	scaffold_4:20302569-20308990	106.667	240.068	1.17033
Ciclev10000029m.g	scaffold_5:20095172-20099940	0.441578	0.993957	1.17051
Ciclev10004216m.g	scaffold_9:12404008-12414330	2.51338	5.66094	1.17141
Ciclev10021453m.g	scaffold_3:43746030-43747389	66.3952	149.571	1.17168
Ciclev10022581m.g	scaffold_3:6040117-6041010	1.91273	4.30972	1.17196
-	scaffold_4:7121650-7122002	3.62361	8.16505	1.17203
Ciclev10027580m.g	scaffold_7:2480699-2482607	0.664716	1.49811	1.17233
Ciclev10018890m.g	scaffold_3:49303692-49306122	0.497026	1.12073	1.17305
Ciclev10010008m.g	scaffold_1:25626609-25628366	7.66603	17.297	1.17397
Ciclev10012726m.g	scaffold_6:16312839-16314254	94.1799	212.711	1.1754
Ciclev10013882m.g	scaffold_6:13451896-13492783	3.99844	9.0309	1.17543
Ciclev10033834m.g	scaffold_4:3663002-3667478	0.707339	1.59779	1.17561
Ciclev10025167m.g	scaffold_7:3751892-3753821	4.85685	10.977	1.1764
Ciclev10031067m.g	scaffold_4:23528236-23533150	6.53499	14.7703	1.17644
Ciclev10003612m.g	scaffold_5:27192349-27195683	0.868778	1.96625	1.17839
-	scaffold_1:27927779-27927922	382.233	865.256	1.17867
Ciclev10026365m.g	scaffold_7:4262660-4264675	2.35374	5.32986	1.17915
Ciclev10024735m.g	scaffold_7:9132940-9138231	0.784363	1.77741	1.18019
Ciclev10020366m.g	scaffold_3:14122347-14127114	10.362	23.5177	1.18244

Ciclev10001374m.g	scaffold_5:30879493-30881250	4.45018	10.1005	1.1825
Ciclev10014863m.g	scaffold_2:6140139-6141999	9.31665	21.1632	1.18367
Ciclev10009433m.g	scaffold_1:20889859-20890704	43.1901	98.1759	1.18467
Ciclev10006512m.g	scaffold_9:26668974-26670807	2.34406	5.33002	1.18513
Ciclev10009836m.g	scaffold_1:4129009-4129985	17.07	38.8281	1.18564
-	scaffold_5:40659586-40660092	2.22367	5.05959	1.18608
Ciclev10030875m.g	scaffold_4:18751931-18757951	37.3623	85.0322	1.18643
Ciclev10011018m.g	scaffold_6:11630380-11638362	7.13932	16.2516	1.18672
Ciclev10028716m.g	scaffold_8:1156026-1158611	0.129638	0.295214	1.18728
Ciclev10023881m.g	scaffold_3:32923706-32929662	4.91054	11.1905	1.18833
Ciclev10004453m.g	scaffold_9:28716254-28723013	4.03151	9.19685	1.18982
-	scaffold_5:25221155-25545745	7.17016	16.3741	1.19134
Ciclev10009903m.g	scaffold_1:4465152-4466043	2.26053	5.16237	1.19137
Ciclev10031004m.g	scaffold_4:20336494-20339198	0.555288	1.26832	1.19161
Ciclev10018887m.g	scaffold_3:3367082-3371823	6.8053	15.5452	1.19173
Ciclev10018229m.g	scaffold_2:7018896-7024367	0.145539	0.332536	1.1921
Ciclev10031476m.g	scaffold_4:19043101-19049169	4.74325	10.855	1.19441
Ciclev10014028m.g	scaffold_2:31603940-31623860	1.31614	3.01279	1.19479
Ciclev10019859m.g	scaffold_3:1218780-1220857	23.1684	53.0616	1.19551
Ciclev10004927m.g	scaffold_9:4198706-4201805	50.0288	114.607	1.19586
Ciclev10031987m.g	scaffold_4:17608077-17611774	1.57254	3.60303	1.19611
Ciclev10001616m.g	scaffold_5:33249884-33251932	2.37617	5.4455	1.19643
Ciclev10012680m.g	scaffold_6:16292018-16293376	32.8366	75.2607	1.19659
Ciclev10002776m.g	scaffold_5:35403322-35406075	11.0682	25.3694	1.19668
Ciclev10011372m.g	scaffold_6:24247852-24251028	4.61458	10.5836	1.19756
Ciclev10004235m.g	scaffold_9:4436333-4441963	1.45158	3.32972	1.19778
Ciclev10019688m.g	scaffold_3:23748300-23749986	1.2335	2.83045	1.19827
Ciclev10027955m.g	scaffold_8:18363675-18368222	112.423	258.05	1.19871
Ciclev10020320m.g	scaffold_3:33962653-33966484	0.190836	0.438041	1.19873
Ciclev10006151m.g	scaffold_9:4266407-4267837	2.28478	5.25512	1.20167
Ciclev10024665m.g	scaffold_3:44783924-44784587	4.47354	10.2922	1.20207
Ciclev10024526m.g	scaffold_3:11687448-12154880	9.35084	21.5498	1.20451
-	scaffold_4:14858358-14859767	1.85634	4.27822	1.20455
Ciclev10025584m.g,Ciclev10027557m.g	scaffold_7:13128486-13174591	98.4577	226.941	1.20474
Ciclev10005552m.g	scaffold_9:28305830-28333082	0.576599	1.32924	1.20496
Ciclev10018766m.g,Ciclev10024136m.g	scaffold_3:7976669-7989624	11.7215	27.0381	1.20583
Ciclev10031308m.g	scaffold_4:4049850-4052952	21.803	50.2946	1.20588
Ciclev10023514m.g	scaffold_3:42338787-42342162	4.31965	9.96697	1.20624
Ciclev10011940m.g,Ciclev10012062m.g	scaffold_6:18471090-18478424	52.3375	120.834	1.20711
Ciclev10030800m.g	scaffold_4:17259275-17264391	0.601489	1.39067	1.20917
Ciclev10014405m.g	scaffold_2:35564237-35570619	2.49288	5.76592	1.20974
Ciclev10006545m.g	scaffold_9:28002672-28004859	0.366481	0.847766	1.20993
-	scaffold_5:28539341-28801354	6.08207	14.0832	1.21135
-	scaffold_5:20349396-20384343	2.22812	5.16245	1.21223
Ciclev10004253m.g	scaffold_9:20890036-21029395	5.21297	12.0782	1.21223
Ciclev10001054m.g	scaffold_5:39909445-39913557	449.336	1041.81	1.21323
-	scaffold_5:9385658-9389294	2.83617	6.57739	1.21357
Ciclev10011651m.g	scaffold_6:15870650-15872961	6.09347	14.1346	1.2139
-	scaffold_5:27880621-27881514	3.92177	9.10521	1.21519
Ciclev10004197m.g	scaffold_9:1329562-1337140	3.91229	9.08781	1.21592
Ciclev10002292m.g	scaffold_5:43127571-43129737	14.6486	34.0331	1.21617
Ciclev10027550m.g	scaffold_7:20737605-20740430	3.12963	7.27576	1.21711
Ciclev10028553m.g	scaffold_8:2551462-2555278	11.7564	27.3504	1.21811

Ciclev10026815m.g	scaffold_7:7248028-7249201	0.401686	0.934671	1.21839
Ciclev10013768m.g	scaffold_6:22577286-22578586	0.320908	0.746938	1.21883
Ciclev10007618m.g	scaffold_1:5268561-5271164	0.0796706	0.185453	1.21893
Ciclev10023767m.g	scaffold_3:48281627-48288259	8.39365	19.5406	1.2191
Ciclev10003038m.g	scaffold_5:42405270-42408220	0.165798	0.386114	1.2196
Ciclev10004388m.g	scaffold_9:26276748-26279190	0.237377	0.552889	1.21981
Ciclev10014312m.g	scaffold_2:5368058-5378200	17.0675	39.7654	1.22026
Ciclev10018739m.g	scaffold_3:2193102-2199392	9.12926	21.2775	1.22076
Ciclev10016255m.g	scaffold_2:28588646-28590596	2.13866	4.98506	1.2209
Ciclev10005761m.g	scaffold_9:2764316-2767059	0.456583	1.06453	1.22127
-	scaffold_1:1852088-1857394	2.63979	6.16232	1.22305
Ciclev10020147m.g	scaffold_3:24899222-24901003	7.39101	17.2557	1.22323
Ciclev10001737m.g	scaffold_5:35217312-35224175	0.368692	0.861033	1.22365
Ciclev10019990m.g	scaffold_3:33956348-33961039	3.52914	8.24639	1.22445
Ciclev10005891m.g	scaffold_9:26016153-26017103	38.9315	91.0255	1.22533
Ciclev10024051m.g	scaffold_3:14110911-14111952	0.502001	1.17454	1.22634
Ciclev10003518m.g	scaffold_5:21294213-21310948	4.42872	10.3621	1.22636
Ciclev10001119m.g	scaffold_5:41871238-41875409	4.22911	9.89509	1.22636
Ciclev10006813m.g	scaffold_9:14665553-14666987	3.04588	7.12675	1.22639
Ciclev10031215m.g	scaffold_4:2131157-2133384	65.7907	153.979	1.22678
Ciclev10027404m.g	scaffold_7:16592404-16594393	1.78779	4.18434	1.22682
Ciclev10031337m.g	scaffold_4:7060366-7066219	3.13827	7.34543	1.22688
Ciclev10001796m.g	scaffold_5:41266494-41270668	77.4684	181.381	1.22734
Ciclev10005336m.g	scaffold_9:14219066-14223386	24.0369	56.3021	1.22794
Ciclev10001546m.g	scaffold_5:35932958-35935079	44.6993	104.79	1.22918
Ciclev10027300m.g	scaffold_7:7669162-7675085	0.393958	0.924927	1.2313
Ciclev10030935m.g	scaffold_4:18215095-18217092	1.02383	2.40383	1.23136
Ciclev10002729m.g	scaffold_5:1311648-1355130	1.54618	3.63073	1.23156
Ciclev10020480m.g	scaffold_3:7379394-7381175	3.81076	8.95038	1.23187
Ciclev10024832m.g	scaffold_7:12265873-12268813	0.173909	0.408933	1.23353
Ciclev10018383m.g	scaffold_2:32653103-32653709	0.545542	1.28348	1.2343
Ciclev10003373m.g	scaffold_5:32546088-32620214	30.6168	72.0324	1.23432
Ciclev10005133m.g	scaffold_9:10246238-10248409	0.118743	0.27941	1.23454
Ciclev10013197m.g	scaffold_6:21062900-21071745	55.599	131.004	1.23648
Ciclev10018726m.g	scaffold_3:6678996-6684558	7.97617	18.7947	1.23656
Ciclev10014159m.g	scaffold_2:9471060-9480543	1.78676	4.21491	1.23816
Ciclev10008319m.g	scaffold_1:20664826-20823973	10.0701	23.7586	1.23837
Ciclev10015109m.g	scaffold_2:573823-592599	4.39837	10.3843	1.23936
-	scaffold_4:20366580-20367089	3.4001	8.03316	1.24039
Ciclev10023430m.g	scaffold_3:905377-906457	6.02574	14.2593	1.24269
Ciclev10013882m.g	scaffold_6:13451896-13492783	0.620002	1.46763	1.24314
Ciclev10024856m.g	scaffold_7:13230970-13233708	1.03103	2.44059	1.24315
Ciclev10025134m.g	scaffold_7:4420564-4422853	2.06929	4.90196	1.24422
Ciclev10019671m.g	scaffold_3:45157245-45159167	6.41959	15.218	1.24522
Ciclev10013612m.g	scaffold_6:11001661-11002524	11.9204	28.2604	1.24535
Ciclev10021341m.g	scaffold_3:44427594-44429073	4.09225	9.70816	1.2463
Ciclev10005970m.g	scaffold_9:24195179-24198282	102.029	242.081	1.24652
Ciclev10028197m.g	scaffold_8:24213702-24217580	9.0401	21.4508	1.24662
Ciclev10021787m.g	scaffold_3:1362482-1364964	5.20028	12.3429	1.24702
Ciclev10000884m.g,Ciclev10000902m.g,Ciclev10001179m.g	scaffold_5:4471259-4568404	9.88794	23.4719	1.24719
Ciclev10023394m.g	scaffold_3:24497773-24502810	0.747763	1.7751	1.24724
-	scaffold_6:16916158-16916959	14.7636	35.1057	1.24966
Ciclev10023632m.g,Ciclev10024548m.g	scaffold_3:41303785-41309278	19.5106	46.4369	1.25101

Ciclev10029331m.g	scaffold_8:1443945-1489074	0.405668	0.965851	1.2515
Ciclev10015533m.g	scaffold_2:35273687-35277921	11.3781	27.093	1.25166
-	scaffold_9:801700-810351	7.75319	18.4624	1.25173
Ciclev10005807m.g	scaffold_9:28023166-28026244	7.34964	17.5028	1.25184
Ciclev10017927m.g	scaffold_2:9557694-9560141	0.326187	0.777148	1.25249
Ciclev10004576m.g	scaffold_9:1950343-1952161	0.624785	1.48891	1.25283
Ciclev10008523m.g	scaffold_1:23334505-23336098	22.6511	53.9858	1.253
Ciclev10017867m.g	scaffold_2:34240827-34242711	0.317994	0.758096	1.25338
Ciclev10010156m.g	scaffold_1:19035252-19035680	17.3001	41.2757	1.25451
Ciclev10008164m.g	scaffold_1:25518710-25521121	24.5809	58.6472	1.25453
Ciclev10024023m.g	scaffold_3:50747607-50749440	1.21766	2.90525	1.25455
Ciclev10019067m.g	scaffold_3:48636710-48639277	2.3363	5.57473	1.25467
Ciclev10015332m.g	scaffold_2:28145056-28149178	76.2347	182.044	1.25577
-	scaffold_5:4076819-4284428	4.76349	11.3856	1.25712
Ciclev10003281m.g	scaffold_5:32707347-32708294	0.260677	0.62314	1.25729
Ciclev10022248m.g	scaffold_3:44442840-44444278	444.487	1063.69	1.25886
Ciclev10020118m.g	scaffold_3:44879955-44884323	10.4187	24.934	1.25894
-	scaffold_1:16412990-16414916	16.1941	38.7592	1.25907
-	scaffold_2:10475677-10475957	11.7626	28.182	1.26057
Ciclev10018626m.g	scaffold_3:36643514-36649262	0.460795	1.1047	1.26146
Ciclev10004564m.g,Ciclev10004598m.g	scaffold_9:30135921-30156463	6.36643	15.266	1.26177
Ciclev10033648m.g	scaffold_4:11523554-11728349	1.69224	4.05951	1.26237
Ciclev10013365m.g	scaffold_6:6577984-6580690	1.94442	4.67005	1.26409
Ciclev10015489m.g	scaffold_2:25921164-25925496	7.91531	19.0109	1.26411
Ciclev10007184m.g	scaffold_9:28286086-28287064	1.05234	2.52793	1.26435
Ciclev10023732m.g	scaffold_3:45045346-45046987	1.39592	3.35495	1.26507
Ciclev10018369m.g	scaffold_2:34760816-34762840	0.362286	0.870743	1.26512
Ciclev10031051m.g	scaffold_4:1869993-1873860	30.0702	72.3097	1.26585
Ciclev10007330m.g	scaffold_1:26963008-26970238	8.69801	20.9177	1.26596
Ciclev10009728m.g	scaffold_1:27512649-27513436	0.255374	0.61451	1.26682
Ciclev10012232m.g	scaffold_6:19738991-19743867	20.6327	49.6496	1.26685
Ciclev10021706m.g	scaffold_3:24617311-24619000	0.616019	1.48239	1.26687
Ciclev10001065m.g	scaffold_5:29825418-29827587	3.61374	8.69749	1.26711
Ciclev10032769m.g	scaffold_4:22219867-22224013	26.7739	64.4681	1.26776
Ciclev10027718m.g	scaffold_8:17075576-17080706	2.05335	4.94555	1.26815
Ciclev10026181m.g	scaffold_7:914352-916536	0.784301	1.88908	1.26821
Ciclev10015762m.g	scaffold_2:10667178-10669367	0.18975	0.457195	1.26871
Ciclev10030460m.g	scaffold_511:2652-7086	2.66215	6.41565	1.269
Ciclev10028119m.g	scaffold_8:2334262-2336645	0.10148	0.244854	1.27073
Ciclev10031344m.g	scaffold_4:687052-688954	0.117462	0.283498	1.27115
Ciclev10020440m.g	scaffold_3:9852176-9854040	24.9024	60.1082	1.27127
Ciclev10005102m.g	scaffold_9:30778802-30783116	0.35327	0.853418	1.27248
Ciclev10023562m.g	scaffold_3:49799714-49801790	4.44024	10.7277	1.27263
Ciclev10020539m.g	scaffold_3:41326034-41334146	31.1618	75.3079	1.27302
Ciclev10006645m.g	scaffold_9:29562920-29568705	11.3126	27.3424	1.27321
Ciclev10031354m.g	scaffold_4:24874577-24876231	5.03628	12.1788	1.27393
Ciclev10029453m.g	scaffold_8:24007860-24043482	34.6027	83.7451	1.27512
Ciclev10008748m.g	scaffold_1:24438795-24442687	928.522	2247.31	1.27519
Ciclev10019203m.g	scaffold_3:41805344-41811790	41.6807	100.905	1.27555
Ciclev10014984m.g	scaffold_2:30971741-30974734	0.114672	0.277963	1.27738
Ciclev10009102m.g	scaffold_1:27031372-27034308	10.4182	25.2561	1.27753
Ciclev10023104m.g,Ciclev10023107m.g	scaffold_3:31396282-31412958	93.1201	225.855	1.27823
Ciclev10004567m.g	scaffold_9:17248553-17250729	1.2579	3.05115	1.27834
Ciclev10026492m.g	scaffold_7:13412504-13413562	0.336414	0.816032	1.27839

Ciclev10001896m.g	scaffold_5:36358701-36360083	0.182307	0.442373	1.2789
-	scaffold_3:11687448-12154880	2.59947	6.30966	1.27934
Ciclev10010861m.g	scaffold_79:15679-18715	9.29485	22.5835	1.28076
Ciclev10001289m.g	scaffold_5:38607425-38609135	2.30874	5.61377	1.28187
Ciclev10015348m.g	scaffold_2:33994409-33996561	84.7124	205.995	1.28196
Ciclev10008359m.g	scaffold_1:21893605-21895163	1.02886	2.50219	1.28214
Ciclev10004167m.g	scaffold_9:29082889-29097015	12.9265	31.5073	1.28536
Ciclev10031135m.g	scaffold_4:16519045-16521918	1.51535	3.69502	1.28593
Ciclev10007525m.g,Ciclev10007741m.g	scaffold_1:19496563-19588226	0.453971	1.10732	1.2864
Ciclev10015422m.g	scaffold_2:4840138-4844193	6.43573	15.702	1.28677
Ciclev10014573m.g	scaffold_2:29713049-29718691	24.1688	58.9876	1.28727
Ciclev10028684m.g	scaffold_8:22715771-22719805	12.3437	30.1274	1.2873
Ciclev10001420m.g,Ciclev10001629m.g	scaffold_5:23886542-23992055	30.4528	74.3579	1.28791
Ciclev10020496m.g	scaffold_3:2733120-2735358	0.398002	0.971865	1.28798
Ciclev10013291m.g	scaffold_6:22902996-22904047	3.05994	7.47284	1.28815
Ciclev10022930m.g	scaffold_3:35367354-35370474	0.527975	1.29055	1.28944
Ciclev10010020m.g	scaffold_1:18890741-18891473	2.26075	5.52638	1.28953
Ciclev10002058m.g	scaffold_5:5260411-5262567	8.18108	20.0059	1.29006
Ciclev10030946m.g	scaffold_4:19647959-19656482	5.76626	14.1036	1.29035
Ciclev10030505m.g	scaffold_4:25427891-25434437	0.124363	0.304466	1.29172
Ciclev10014094m.g	scaffold_2:25228847-25237434	10.4099	25.4939	1.2922
-	scaffold_9:5924305-5925034	43.191	105.804	1.29259
Ciclev10010327m.g	scaffold_1:385468-400033	0.855281	2.09641	1.29345
Ciclev10025257m.g	scaffold_7:6617947-6624998	6.5444	16.0473	1.29399
Ciclev10032371m.g	scaffold_4:1774505-1776022	0.290682	0.713075	1.29461
Ciclev10029840m.g	scaffold_8:24512739-24517606	0.057618	0.141431	1.29551
Ciclev10011551m.g	scaffold_6:15400555-15402884	6.93658	17.0317	1.29592
Ciclev10023368m.g	scaffold_3:4022307-4023918	0.114012	0.279986	1.29617
Ciclev10013390m.g	scaffold_6:13626637-13628971	0.152815	0.37533	1.29637
Ciclev10004031m.g	scaffold_5:36289977-36292068	1.02695	2.52327	1.29694
Ciclev10015301m.g	scaffold_2:32681451-32684775	7.56723	18.5981	1.29732
-	scaffold_8:19132836-19211350	21.1826	52.0955	1.29828
Ciclev10025112m.g	scaffold_7:10555107-10583369	38.2433	94.0879	1.2988
Ciclev10028935m.g	scaffold_8:5116750-5120818	0.491051	1.20913	1.30002
Ciclev10004796m.g	scaffold_9:4923462-4925049	0.569138	1.40165	1.30028
Ciclev10028518m.g	scaffold_8:2545493-2549731	41.9883	103.465	1.30109
Ciclev10018083m.g	scaffold_2:31877297-31879481	0.413589	1.01929	1.3013
Ciclev10018999m.g	scaffold_3:7261863-7264301	0.754337	1.85912	1.30134
Ciclev10025296m.g	scaffold_7:14850156-14852780	1.49564	3.6887	1.30235
Ciclev10018479m.g	scaffold_3:42863957-42872639	7.40864	18.2923	1.30396
Ciclev10017298m.g,Ciclev10017300m.g	scaffold_2:19944532-19959075	11.3764	28.0963	1.30434
Ciclev10000558m.g	scaffold_5:33698384-33702241	4.24742	10.4927	1.30473
Ciclev10031639m.g	scaffold_4:17329472-17351349	164.823	407.18	1.30475
Ciclev10019350m.g	scaffold_3:5563150-5564986	0.940387	2.324	1.30528
Ciclev10022365m.g	scaffold_3:13034653-13036082	2.39871	5.92867	1.30545
Ciclev10013477m.g	scaffold_6:12650790-12658440	0.0926784	0.229092	1.30562
Ciclev10010482m.g	scaffold_1:405054-406125	0.172225	0.425959	1.30642
Ciclev10032086m.g	scaffold_4:14895727-14896991	0.329864	0.815941	1.30659
Ciclev10005884m.g	scaffold_9:29705765-29706553	6.90092	17.0717	1.30674
Ciclev10011474m.g	scaffold_6:24405133-24407191	4.55945	11.2888	1.30796
Ciclev10031826m.g	scaffold_4:25358198-25361196	5.36099	13.2783	1.30849
Ciclev10003343m.g	scaffold_5:40406627-40408591	0.173926	0.430864	1.30876
Ciclev10014703m.g	scaffold_2:34043130-34046810	7.32719	18.1603	1.30946

Ciclev10007711m.g,Ciclev10007833m.g	scaffold_1:21975140-22013742	2.28252	5.65833	1.30975
Ciclev10011090m.g	scaffold_6:24730399-24734346	3.65999	9.08325	1.31137
Ciclev10026963m.g	scaffold_7:1645821-1649037	16.6396	41.3103	1.31188
Ciclev10028400m.g	scaffold_8:22039940-22042383	0.700418	1.73918	1.31212
Ciclev10024609m.g	scaffold_3:17618990-17621610	3.16288	7.8538	1.31215
Ciclev10017398m.g	scaffold_2:13384976-13387611	13.8936	34.5004	1.31219
Ciclev10028269m.g,Ciclev10030057m.g	scaffold_8:9325618-9585469	12.5199	31.1004	1.3127
Ciclev10004133m.g	scaffold_9:1954167-1964488	0.908801	2.26047	1.31459
Ciclev10014335m.g	scaffold_2:23811560-23815068	4.46727	11.1243	1.31625
Ciclev10010341m.g	scaffold_1:19905420-19995040	1.6855	4.19793	1.3165
Ciclev10028100m.g	scaffold_8:21300864-21303025	0.946064	2.35663	1.31671
Ciclev10024913m.g	scaffold_7:11850299-12160617	0.563876	1.40486	1.31698
Ciclev10019784m.g	scaffold_3:24949254-24954319	0.247479	0.616606	1.31704
Ciclev10033602m.g	scaffold_4:25017111-25019556	0.264721	0.659876	1.31772
Ciclev10020979m.g	scaffold_3:2697354-2700326	0.149514	0.37279	1.31808
Ciclev10032043m.g	scaffold_4:22605812-22607702	3.85612	9.62171	1.31914
Ciclev10021676m.g	scaffold_3:24837937-24840296	10.5928	26.4341	1.31932
Ciclev10032901m.g	scaffold_4:24373547-24374755	7.03109	17.5487	1.31954
Ciclev10018920m.g	scaffold_3:5442699-5452619	0.162765	0.406261	1.31962
Ciclev10018146m.g	scaffold_2:31075994-31077746	1.82141	4.55207	1.32146
Ciclev10001720m.g	scaffold_5:32300020-32302876	18.8521	47.1272	1.32184
Ciclev10024396m.g	scaffold_3:27120137-27122618	0.150357	0.376225	1.3232
Ciclev10008409m.g	scaffold_1:26705758-26710444	19.9305	49.8747	1.32333
Ciclev10018554m.g	scaffold_3:43006063-43010078	0.50427	1.2623	1.32379
Ciclev10022631m.g	scaffold_3:28362010-28363192	0.214788	0.537774	1.32409
Ciclev10026385m.g	scaffold_7:17356834-17360150	0.516665	1.29367	1.32416
Ciclev10014848m.g	scaffold_2:6994733-6996507	75.7645	189.766	1.32463
Ciclev10021526m.g	scaffold_3:49256305-49267922	1.96262	4.91693	1.32498
Ciclev10030177m.g	scaffold_8:22994986-22998185	0.435161	1.09044	1.32529
Ciclev10027167m.g	scaffold_7:1758739-1762044	0.245218	0.614869	1.32621
Ciclev10030815m.g	scaffold_4:3807730-3813265	3.40466	8.53744	1.32629
Ciclev10025811m.g	scaffold_7:342296-346663	1.82344	4.57637	1.32755
Ciclev10019231m.g	scaffold_3:49853424-49859158	13.3654	33.5551	1.32803
Ciclev10000986m.g	scaffold_5:15915501-15917455	0.293908	0.738587	1.3294
Ciclev10015543m.g	scaffold_2:25339915-25342426	0.712133	1.78982	1.32959
Ciclev10004729m.g	scaffold_9:3387858-3392926	86.1361	216.494	1.32963
Ciclev10025426m.g	scaffold_7:9055027-9085218	0.356936	0.897161	1.3297
Ciclev10016906m.g	scaffold_2:28271854-28275282	50.4883	126.915	1.32984
Ciclev10033739m.g	scaffold_4:10885192-10887328	17.4063	43.7796	1.33065
Ciclev10033667m.g	scaffold_4:4102993-4106226	13.6708	34.3987	1.33126
Ciclev10007255m.g	scaffold_1:6724910-6731810	3.06056	7.70185	1.33141
Ciclev10021842m.g	scaffold_3:23170539-23174896	5.06753	12.7925	1.33595
Ciclev10015021m.g,Ciclev10015056m.g	scaffold_2:5834136-5839731	6.27033	15.8316	1.3362
Ciclev10014208m.g	scaffold_2:20410112-20414242	2.85919	7.22557	1.33751
Ciclev10026916m.g	scaffold_7:4305755-4307778	0.241733	0.611081	1.33795
Ciclev10028153m.g	scaffold_8:22234237-22238783	1.62653	4.11485	1.33904
Ciclev10020548m.g	scaffold_3:4489092-4491310	16.618	42.0572	1.3396
Ciclev10012577m.g	scaffold_6:18653736-18656792	0.418805	1.05993	1.33962
Ciclev10008500m.g	scaffold_1:905111-909081	1.36596	3.458	1.34003
Ciclev10020606m.g	scaffold_3:11687448-12154880	0.400284	1.01406	1.34104
Ciclev10019686m.g	scaffold_3:6060977-6065155	11.3501	28.7565	1.34118
Ciclev10018175m.g	scaffold_2:33409221-33411202	0.492829	1.24934	1.34201
Ciclev10012290m.g	scaffold_6:10816139-10936716	70.2713	178.177	1.3423

Ciclev10002077m.g	scaffold_5:39227920-39237745	1.14525	2.90481	1.34278
Ciclev10024857m.g	scaffold_7:15435206-15437898	0.180507	0.457919	1.34304
Ciclev10004901m.g	scaffold_9:30382985-30428893	14.3533	36.4124	1.34305
Ciclev10006800m.g	scaffold_9:22015107-22021712	0.112473	0.285501	1.34392
Ciclev10025430m.g	scaffold_7:2375819-2379589	3.06764	7.78733	1.344
-	scaffold_1:5516484-5516711	16.8605	42.8167	1.34453
Ciclev10021316m.g	scaffold_3:42413612-42416194	3.54163	8.99537	1.34477
Ciclev10024056m.g	scaffold_3:4584397-4588993	0.307388	0.781729	1.34661
Ciclev10000264m.g	scaffold_5:39500186-39503920	0.750873	1.91084	1.34757
Ciclev10018785m.g,Ciclev10024057m.g	scaffold_3:11248388-11275176	18.299	46.6104	1.34888
Ciclev10017133m.g	scaffold_2:6094581-6095295	34.8481	88.8189	1.34979
Ciclev10002531m.g	scaffold_5:40163477-40166214	1.66642	4.24782	1.34997
Ciclev10001812m.g	scaffold_5:39862761-39864726	76.7245	195.659	1.35058
-	scaffold_2:22385359-22385660	50.3105	128.326	1.35088
Ciclev10002574m.g	scaffold_5:5083070-5085647	3.75242	9.57233	1.35105
Ciclev10002629m.g	scaffold_5:21475234-21479008	10.09	25.7479	1.35152
-	scaffold_3:36700035-36701868	1.06907	2.7308	1.35297
Ciclev10019916m.g	scaffold_3:20779569-20782178	2.71872	6.94504	1.35306
Ciclev10013171m.g	scaffold_6:20423036-20424121	1.79012	4.57521	1.35378
Ciclev10030645m.g	scaffold_4:24676756-24680171	4.93238	12.622	1.35558
Ciclev10010770m.g	scaffold_1:9385704-9389138	3.84364	9.83664	1.35569
Ciclev10017743m.g	scaffold_2:5379661-5382121	0.742343	1.90261	1.35782
Ciclev10030004m.g	scaffold_8:9309825-9310869	24.7088	63.3562	1.35846
Ciclev10026902m.g	scaffold_7:5277964-5281312	0.164552	0.422109	1.35907
Ciclev10033740m.g	scaffold_4:14974793-14988390	0.0521444	0.133909	1.36067
Ciclev10011116m.g	scaffold_6:10438272-10440667	0.251711	0.646453	1.36078
Ciclev10014549m.g	scaffold_2:28403936-28488203	0.981208	2.52167	1.36175
Ciclev10023272m.g	scaffold_3:24352153-24448329	0.31573	0.811612	1.3621
Ciclev10018115m.g	scaffold_2:33329909-33331332	2.10793	5.41931	1.36228
Ciclev10006241m.g	scaffold_9:27198507-27199642	178.117	458.23	1.36325
Ciclev10032943m.g	scaffold_4:12596112-12597155	1.85121	4.76467	1.3639
Ciclev10013345m.g	scaffold_6:482249-483701	0.43621	1.12308	1.36437
Ciclev10001802m.g	scaffold_5:284909-289108	1.85202	4.77023	1.36496
Ciclev10003397m.g,Ciclev10003554m.g	scaffold_5:7518692-7521669	1.68638	4.34384	1.36504
Ciclev10026924m.g	scaffold_7:13622757-13624577	1.84008	4.74461	1.36652
-	scaffold_1:18387332-18387726	2.78966	7.19468	1.36684
Ciclev10005013m.g	scaffold_9:25320788-25322187	1.10334	2.84734	1.36774
Ciclev10009734m.g	scaffold_1:27895994-27896644	2.07113	5.34998	1.36912
Ciclev10017138m.g	scaffold_2:32041519-32053665	36.8462	95.287	1.37077
Ciclev10000536m.g	scaffold_5:24115896-24122184	0.101721	0.263183	1.37145
Ciclev10029187m.g	scaffold_8:4131965-4132968	7.63553	19.7584	1.37167
Ciclev10015505m.g	scaffold_2:28893662-28896553	2.7403	7.0919	1.37184
Ciclev10016791m.g	scaffold_2:34239978-34240691	0.928373	2.4049	1.3732
Ciclev10007134m.g	scaffold_9:11576287-11715575	0.0598239	0.155063	1.37406
Ciclev10027511m.g	scaffold_7:11850299-12160617	0.183353	0.475521	1.37488
Ciclev10003483m.g	scaffold_5:19865022-20070734	0.100738	0.26135	1.37538
Ciclev10027427m.g	scaffold_7:14199038-14201793	0.172533	0.447775	1.3759
Ciclev10032998m.g	scaffold_4:1175518-1180789	3.35122	8.69875	1.37612
Ciclev10011537m.g	scaffold_6:21398291-21401373	1.14783	2.98307	1.37789
Ciclev10015169m.g	scaffold_2:35140489-35143580	2.36335	6.14656	1.37895
-	scaffold_5:34100235-34100803	3.30175	8.58734	1.37898
Ciclev10023099m.g	scaffold_3:47255283-47259374	0.229953	0.598233	1.37937
Ciclev10014465m.g	scaffold_2:35461899-35466268	4.76229	12.3935	1.37986
Ciclev10021079m.g	scaffold_3:49772951-49774607	3.60335	9.38276	1.38068

Ciclev10009252m.g	scaffold_1:25513691-25515549	23.8525	62.1259	1.38105
Ciclev10024768m.g	scaffold_7:20070207-20082892	3.35063	8.73787	1.38285
Ciclev10000602m.g	scaffold_5:32296452-32298508	2.18852	5.70733	1.38286
Ciclev10026860m.g	scaffold_7:9370617-9373482	20.8663	54.4193	1.38294
Ciclev10020527m.g	scaffold_3:2242607-2244545	4.62294	12.057	1.38299
Ciclev10024615m.g	scaffold_3:30922823-30924113	0.757591	1.97679	1.38367
Ciclev10005233m.g	scaffold_9:25722747-25725484	8.83394	23.057	1.38408
Ciclev10001480m.g	scaffold_5:33293771-33295895	3.21257	8.38541	1.38415
Ciclev10033689m.g	scaffold_4:1784157-1786292	0.286407	0.747712	1.38442
Ciclev10017056m.g	scaffold_2:27616965-27617813	12.2209	31.9331	1.38571
Ciclev10013234m.g	scaffold_6:15151155-15151909	18.7105	48.894	1.38581
Ciclev10025608m.g	scaffold_7:19675118-19678145	6.73749	17.6161	1.38661
Ciclev10010056m.g	scaffold_1:19049995-19054408	0.252089	0.659449	1.38733
Ciclev10013410m.g,Ciclev10013724m.g	scaffold_6:21646666-21650760	8.79159	23.0073	1.3879
Ciclev10028402m.g	scaffold_8:17498897-17505378	2.32653	6.09012	1.38829
Ciclev10006939m.g	scaffold_9:1073655-1075956	0.625429	1.63744	1.38852
Ciclev10005668m.g	scaffold_9:30227047-30229005	1.18028	3.09353	1.39013
Ciclev10001512m.g	scaffold_5:39487474-39489870	4.46691	11.7129	1.39075
Ciclev10004812m.g	scaffold_9:7515903-7517749	26.0545	68.3239	1.39086
Ciclev10028134m.g	scaffold_8:23439131-23442638	0.908753	2.38331	1.39101
-	scaffold_8:15117014-15119555	9.32187	24.4812	1.39298
Ciclev10011903m.g	scaffold_6:18615190-18618530	24.3639	64.1437	1.39657
Ciclev10011991m.g	scaffold_6:16175185-16177940	0.965477	2.54191	1.3966
Ciclev10005047m.g	scaffold_9:2504122-2508310	5.02565	13.2345	1.39692
Ciclev10014382m.g	scaffold_2:6927080-6932080	27.6915	72.9289	1.39704
Ciclev10031836m.g	scaffold_4:21672606-21675316	9.32361	24.5628	1.39752
Ciclev10027866m.g	scaffold_8:20620043-20723794	1095.48	2888.67	1.39885
Ciclev10033606m.g	scaffold_4:24284147-24286972	0.757489	1.99884	1.39987
Ciclev10018864m.g	scaffold_3:1259959-1263364	2.86734	7.5751	1.40155
-	scaffold_6:8362033-8362588	2.93867	7.77737	1.40412
Ciclev10028277m.g	scaffold_8:10039552-10041219	7.88522	20.8707	1.40426
-	scaffold_1:26098796-26099948	2.30431	6.1008	1.40466
Ciclev10020864m.g	scaffold_3:35789561-35793592	11.1417	29.5284	1.40614
Ciclev10019035m.g	scaffold_3:2670294-2673111	3.25128	8.62487	1.4075
Ciclev10012447m.g	scaffold_6:14804827-14808139	12.1966	32.3707	1.40821
Ciclev10000948m.g	scaffold_5:42695342-42700919	277.95	737.908	1.40862
Ciclev10008231m.g	scaffold_1:25135404-25136905	1.08722	2.88932	1.41008
Ciclev10004079m.g	scaffold_5:5066410-5068283	1.08756	2.8919	1.41092
Ciclev10005901m.g	scaffold_9:5639022-5640306	0.566821	1.50989	1.41348
Ciclev10013516m.g	scaffold_6:5754834-5780076	1.77687	4.73445	1.41386
Ciclev10019657m.g	scaffold_3:462332-464454	2.63818	7.03152	1.41429
Ciclev10010250m.g	scaffold_1:24214004-24218228	0.664224	1.77181	1.41549
Ciclev10027512m.g	scaffold_7:19624697-19627055	0.520994	1.3902	1.41595
Ciclev10015840m.g	scaffold_2:13520397-13525214	110.12	293.94	1.41645
Ciclev10014117m.g	scaffold_2:30701895-30712151	1.27496	3.40557	1.41744
Ciclev10002231m.g	scaffold_5:34665210-34666402	3.0166	8.05831	1.41756
Ciclev10008520m.g	scaffold_1:18117263-18119451	222.03	593.483	1.41845
Ciclev10025476m.g	scaffold_7:5654375-5658039	36.0484	96.4055	1.41918
Ciclev10018100m.g	scaffold_2:31797602-31799123	0.941904	2.52065	1.42014
Ciclev10018903m.g	scaffold_3:1253711-1256833	0.634911	1.71024	1.42957
Ciclev10004694m.g	scaffold_9:5493707-5510148	48.8849	131.685	1.42963
-	scaffold_3:20345057-20345526	1.96844	5.30362	1.42993
Ciclev10030981m.g	scaffold_4:2241672-2245747	0.40591	1.09417	1.4306
Ciclev10016966m.g	scaffold_2:13103890-13104876	4.97414	13.4083	1.43061
Ciclev10016008m.g	scaffold_2:4516987-4518242	4.892	13.1916	1.43112

Ciclev10030536m.g	scaffold_4:4808143-4816895	0.0573888	0.15476	1.43119
Ciclev10033749m.g	scaffold_4:8681670-8684630	0.456179	1.23123	1.43243
Ciclev10024995m.g	scaffold_7:10093155-10095915	11.1392	30.113	1.43474
Ciclev10029816m.g	scaffold_8:1260974-1263712	1.22231	3.30441	1.43478
Ciclev10011041m.g	scaffold_6:24141801-24144468	0.692773	1.87313	1.43499
Ciclev10011889m.g	scaffold_6:5754834-5780076	12.0282	32.5772	1.43744
Ciclev10025699m.g	scaffold_7:265244-268499	4.17588	11.3184	1.43852
-	scaffold_5:29642408-29642605	25.1194	68.0858	1.43855
Ciclev10004694m.g	scaffold_9:5493707-5510148	217.301	589.034	1.43866
Ciclev10019999m.g	scaffold_3:45125079-45126713	3.51662	9.53909	1.43966
Ciclev10000645m.g	scaffold_5:13531936-13535930	30.5692	82.9543	1.44024
Ciclev10027497m.g	scaffold_7:15490272-15493088	7.55208	20.514	1.44167
Ciclev10022531m.g	scaffold_3:45244126-45245482	3.02395	8.22165	1.44299
Ciclev10024942m.g	scaffold_7:11123794-11126263	0.0796851	0.216654	1.44301
Ciclev10024866m.g	scaffold_7:10471135-10474241	2.43923	6.63216	1.44305
Ciclev10002186m.g	scaffold_5:36352562-36356244	57.7778	157.185	1.44387
Ciclev10033347m.g,Ciclev10033406m.g	scaffold_4:3761490-3766717	0.751788	2.0458	1.44426
Ciclev10013205m.g	scaffold_6:19596977-19597721	0.87665	2.38563	1.4443
Ciclev10030972m.g	scaffold_4:17403142-17408809	3.38511	9.21275	1.44443
Ciclev10021079m.g	scaffold_3:49772951-49774607	2.46317	6.7092	1.44562
Ciclev10011584m.g	scaffold_6:23534437-23537172	2.19414	6.00018	1.45135
Ciclev10014586m.g	scaffold_2:22488688-22492808	7.06337	19.3203	1.45169
Ciclev10032979m.g	scaffold_4:132245-134933	3.61596	9.90157	1.45328
Ciclev10015846m.g	scaffold_2:27344586-27346090	3.65349	10.0087	1.45391
Ciclev10018983m.g	scaffold_3:27385465-27391796	7.74668	21.2337	1.45471
Ciclev10024447m.g	scaffold_3:44766784-44767267	1.50253	4.11915	1.45495
Ciclev10021742m.g	scaffold_3:33593683-33595447	6.57745	18.0407	1.45565
Ciclev10012729m.g	scaffold_6:17230957-17232230	19.3511	53.1224	1.4569
Ciclev10004036m.g	scaffold_5:36821525-36826251	13.3672	36.7069	1.45735
-	scaffold_9:8460944-8465503	36.5455	100.594	1.46078
Ciclev10029407m.g	scaffold_8:23963892-23969651	2.9129	8.02542	1.46212
Ciclev10004307m.g	scaffold_9:3917762-3920327	0.63773	1.75824	1.46312
Ciclev10013604m.g	scaffold_6:22828055-22898575	19.2621	53.1565	1.46448
Ciclev10007937m.g	scaffold_1:10398794-10401278	0.256587	0.709378	1.46711
Ciclev10029246m.g	scaffold_8:23037767-23040948	13.7305	38.0047	1.4688
-	scaffold_9:28815847-28816222	4.1542	11.5013	1.46916
Ciclev10033973m.g	scaffold_4:14782768-14785138	2.37567	6.5823	1.47026
Ciclev10007621m.g	scaffold_1:7876115-7880443	2.67737	7.42211	1.47101
Ciclev10006697m.g	scaffold_9:31053074-31059875	0.660777	1.83227	1.4714
Ciclev10031586m.g	scaffold_4:1825112-1827197	1.87738	5.20589	1.47143
Ciclev10000895m.g	scaffold_5:34312616-34314987	4.46379	12.3936	1.47326
Ciclev10017662m.g	scaffold_2:11651212-11652208	6.29119	17.4747	1.47387
Ciclev10005617m.g	scaffold_9:14615727-14619152	2.78162	7.72959	1.47446
-	scaffold_5:40313832-40314134	12.8845	35.8161	1.47497
Ciclev10023744m.g	scaffold_3:46519382-46595230	1.00584	2.79613	1.47504
Ciclev10006194m.g	scaffold_9:3646480-3648592	30.272	84.4179	1.47957
Ciclev10001145m.g	scaffold_5:36785809-36787450	0.270605	0.754736	1.47978
Ciclev10028755m.g	scaffold_8:18926024-18929765	28.9631	80.7919	1.47999
Ciclev10032686m.g	scaffold_4:17365401-17366484	822.011	2293.3	1.4802
Ciclev10015267m.g	scaffold_2:25198038-25200016	2.04215	5.69747	1.48023
Ciclev10003732m.g	scaffold_5:40655648-40656368	2.17807	6.07756	1.48044
Ciclev10003637m.g	scaffold_5:16331006-16331354	4.12088	11.4987	1.48045
Ciclev10001401m.g	scaffold_5:354415-357611	4.34429	12.1229	1.48054
Ciclev10024955m.g	scaffold_7:2852146-2854426	0.390302	1.09038	1.48216
Ciclev10002132m.g	scaffold_5:43051578-43053394	0.697882	1.95221	1.48406

Ciclev10029308m.g	scaffold_8:17508327-17509747	1.44055	4.03711	1.4867
Ciclev10033817m.g	scaffold_4:24949304-24952363	0.210722	0.590748	1.4872
Ciclev10031892m.g	scaffold_4:3319449-3320632	0.397358	1.11466	1.48809
Ciclev10011156m.g	scaffold_6:20412427-20414813	1.01029	2.83476	1.48846
-	scaffold_9:26183811-26184172	20.4424	57.3735	1.48882
Ciclev10028190m.g	scaffold_8:21228884-21231946	12.4674	35.0428	1.49096
Ciclev10028812m.g	scaffold_8:12531186-12533619	48.2473	135.742	1.49235
Ciclev10019927m.g	scaffold_3:48337104-48339028	41.1087	115.781	1.49388
Ciclev10022490m.g	scaffold_3:7574382-7576856	2.28929	6.44835	1.49403
Ciclev10005221m.g	scaffold_9:3149357-3152560	7.16987	20.2252	1.49614
Ciclev10010058m.g	scaffold_1:7007441-7008006	0.526353	1.48692	1.49822
Ciclev10029037m.g	scaffold_8:20620043-20723794	5.43511	15.371	1.49983
Ciclev10013904m.g	scaffold_6:24163654-24165092	0.390926	1.10633	1.50082
Ciclev10017318m.g	scaffold_2:30378876-30379401	2.32755	6.59123	1.50174
Ciclev10008789m.g	scaffold_1:22931442-22932626	8.12621	23.0345	1.50314
Ciclev10020568m.g,Ciclev10020613m.g	scaffold_3:38558307-38564955	40.6848	115.337	1.50329
Ciclev10014661m.g	scaffold_2:4850204-4853824	2.58482	7.3281	1.50337
Ciclev10032484m.g	scaffold_4:164207-166483	3.12952	8.87408	1.50366
Ciclev10014162m.g	scaffold_2:7193916-7200026	5.0079	14.2054	1.50416
-	scaffold_3:27152647-27152907	6.17255	17.5341	1.50623
Ciclev10011291m.g	scaffold_6:12440025-12443884	1.37008	3.90012	1.50926
Ciclev10011150m.g	scaffold_6:18335280-18337503	0.863383	2.45931	1.51018
Ciclev10004384m.g	scaffold_9:27873724-27878354	1.95194	5.56543	1.51159
Ciclev10008378m.g	scaffold_1:23039478-23042402	41.9397	119.587	1.51167
Ciclev10026594m.g	scaffold_7:3721374-3722616	10.1082	28.8341	1.51225
-	scaffold_1:6460702-6460948	15.4875	44.2164	1.51348
Ciclev10011059m.g	scaffold_6:22028926-22032079	1.40846	4.02374	1.51442
Ciclev10020743m.g	scaffold_3:42753918-42757004	2.99044	8.55142	1.5158
Ciclev10006288m.g	scaffold_9:23075529-23122347	1.34823	3.85552	1.51586
Ciclev10019732m.g	scaffold_3:44959565-44964478	12.0102	34.45	1.52024
Ciclev10028524m.g	scaffold_8:22303407-22305179	1.89855	5.44672	1.52049
Ciclev10028247m.g	scaffold_8:2561844-2563605	48.1179	138.11	1.52117
Ciclev10014595m.g	scaffold_2:25663219-25665115	1.06933	3.07269	1.52279
Ciclev10014547m.g,Ciclev10017353m.g	scaffold_2:13148139-13151298	0.777248	2.23425	1.52334
Ciclev10005226m.g	scaffold_9:8153323-8154836	3.45504	9.93585	1.52394
Ciclev10016377m.g	scaffold_2:3627335-3628146	2.11524	6.09665	1.52719
Ciclev10014857m.g	scaffold_2:31148848-31150616	1.67985	4.84606	1.52848
Ciclev10012060m.g	scaffold_6:12883560-12885088	10.4141	30.0555	1.52909
Ciclev10022447m.g	scaffold_3:23810558-23814249	2.99288	8.64182	1.5298
-	scaffold_8:11259049-11271539	2.90724	8.39821	1.53043
Ciclev10014464m.g,Ciclev10017482m.g,Ciclev10018149m.g	scaffold_2:26105694-26158658	9.29315	26.858	1.53111
Ciclev10002794m.g,Ciclev10002900m.g	scaffold_5:441330-538185	16.3438	47.2926	1.53287
Ciclev10019612m.g	scaffold_3:11647118-11653316	32.8674	95.1852	1.53408
Ciclev10030949m.g	scaffold_4:538718-543204	1.23584	3.57974	1.53436
Ciclev10029756m.g	scaffold_8:4205576-4222282	7.73751	22.466	1.5378
Ciclev10016993m.g	scaffold_2:30640566-30642762	10.1523	29.5031	1.53905
Ciclev10015817m.g	scaffold_2:35163539-35165344	1.93953	5.63754	1.53936
-	scaffold_4:8708296-8709237	0.686619	1.99583	1.53941
Ciclev10022542m.g	scaffold_3:46381608-46383163	229.08	666.319	1.54036
Ciclev10032689m.g	scaffold_4:16306112-16307984	2.80276	8.16166	1.54202
Ciclev10029636m.g	scaffold_8:451251-453295	15.6276	45.5185	1.54236

Ciclev10019150m.g	scaffold_3:23560517-23571254	25.933	75.5497	1.54264
-	scaffold_8:3676427-3676792	4.42479	12.8912	1.5427
Ciclev10018337m.g	scaffold_2:29786637-29788170	1.07773	3.14069	1.54309
Ciclev10004483m.g	scaffold_9:25272615-25275949	0.462671	1.34874	1.54355
Ciclev10027797m.g	scaffold_8:23418714-23422996	1.24671	3.63471	1.54372
Ciclev10003562m.g	scaffold_5:40720953-40723821	0.861541	2.5128	1.5443
Ciclev10023721m.g	scaffold_3:40782160-40821316	8.67326	25.3207	1.54567
Ciclev10033340m.g	scaffold_4:14396168-14402910	1.6694	4.88393	1.54872
-	scaffold_9:28942008-28942580	98.7237	288.836	1.54878
Ciclev10020045m.g	scaffold_3:41485984-41488852	0.861245	2.52198	1.55006
Ciclev10026127m.g	scaffold_7:6541780-6543278	0.860314	2.52449	1.55306
Ciclev10004587m.g	scaffold_9:28491241-28493112	0.292549	0.858696	1.55347
Ciclev10011507m.g	scaffold_6:24957159-24958992	0.271511	0.797081	1.55371
Ciclev10022863m.g	scaffold_3:46841461-46841986	2.74766	8.06882	1.55416
Ciclev10010435m.g	scaffold_1:17597537-17599607	2.27655	6.69133	1.55544
Ciclev10003522m.g	scaffold_5:31469286-31718393	0.344827	1.01397	1.55607
Ciclev10015069m.g	scaffold_2:33796710-33800442	0.239335	0.70457	1.55771
Ciclev10030374m.g	scaffold_8:2080286-2082461	0.348878	1.02742	1.55823
Ciclev10008095m.g,Ciclev10008099m.g	scaffold_1:9491339-9521614	53.337	157.126	1.55871
Ciclev10003730m.g	scaffold_5:29339627-29387910	1.29539	3.81609	1.55871
-	scaffold_5:25221155-25545745	8.566	25.2366	1.55882
Ciclev10004980m.g	scaffold_9:6823895-6826870	0.466168	1.37363	1.55907
Ciclev10003138m.g	scaffold_5:39999082-40000451	1.47411	4.34394	1.55916
-	scaffold_1:13563115-13572331	26.273	77.4954	1.56053
Ciclev10025279m.g	scaffold_7:20298359-20304509	12.0799	35.6534	1.56143
Ciclev10013812m.g	scaffold_6:20061355-20063705	0.283954	0.838275	1.56177
Ciclev10033622m.g	scaffold_4:2758837-2761074	7.26618	21.4601	1.56239
Ciclev10023382m.g	scaffold_3:21296577-21473295	19.2436	56.847	1.5627
Ciclev10011220m.g	scaffold_6:17016021-17021844	0.571471	1.68957	1.5639
Ciclev10026728m.g	scaffold_7:10817044-10818074	0.187013	0.553302	1.56493
Ciclev10005414m.g	scaffold_9:2927434-2929229	6.57199	19.4488	1.56528
Ciclev10004509m.g	scaffold_9:27573431-27578714	4.16552	12.329	1.56549
Ciclev10021210m.g	scaffold_3:33388539-33592383	6.10368	18.0831	1.56689
Ciclev10030338m.g	scaffold_8:22720417-22724438	0.378497	1.12169	1.56732
Ciclev10023882m.g	scaffold_3:24324288-24325928	0.341734	1.01484	1.5703
Ciclev10024210m.g	scaffold_3:5548624-5549620	0.226316	0.672135	1.57041
Ciclev10008044m.g	scaffold_1:7060621-7066678	10.4546	31.0605	1.57094
Ciclev10027589m.g	scaffold_7:10555107-10583369	7.96658	23.6906	1.57228
Ciclev10014858m.g,Ciclev10014862m.g	scaffold_2:5975366-6022331	43.5466	129.515	1.57249
Ciclev10001855m.g	scaffold_5:32292685-32294775	1.5087	4.49333	1.57448
Ciclev10010448m.g	scaffold_1:1229336-1230149	2.88258	8.58703	1.5748
Ciclev10032899m.g	scaffold_4:2921615-2922311	0.267725	0.798706	1.57691
Ciclev10021090m.g	scaffold_3:29481041-29482628	1.60935	4.81153	1.58002
Ciclev10032674m.g	scaffold_4:23926316-23927742	0.227484	0.680465	1.58076
Ciclev10001177m.g	scaffold_5:42755250-42758065	1.23797	3.70446	1.58129
Ciclev10016570m.g	scaffold_2:36045360-36046453	0.810171	2.42786	1.58338
Ciclev10031409m.g	scaffold_4:2947329-2950463	2.57091	7.70963	1.58438
Ciclev10027774m.g	scaffold_8:14879290-14883733	0.406928	1.22065	1.5848
Ciclev10016158m.g	scaffold_2:23984516-23987081	3.695	11.0928	1.58598
Ciclev10010823m.g	scaffold_1:4762671-4764882	2.65223	7.9641	1.58631
Ciclev10027014m.g	scaffold_7:9212797-9232329	1.77168	5.32276	1.58706
Ciclev10020867m.g	scaffold_3:43758914-43761623	11.2324	33.7598	1.58764
Ciclev10006418m.g	scaffold_9:3832886-3833402	2.85052	8.57042	1.58814
Ciclev10021010m.g	scaffold_3:46932120-46933456	0.193869	0.58292	1.58822

Ciclev10033731m.g	scaffold_4:12011560-12012217	0.991967	2.98699	1.59033
Ciclev10014216m.g	scaffold_2:7507436-7569782	2.23169	6.72325	1.59102
Ciclev10001872m.g	scaffold_5:34032195-34033465	12.1314	36.5482	1.59106
Ciclev10024863m.g	scaffold_7:9644559-9649024	0.393726	1.1867	1.59169
Ciclev10019365m.g	scaffold_3:11458953-11461162	11.0908	33.4358	1.59203
-	scaffold_3:3338897-3339101	20.2218	60.9698	1.59218
Ciclev10027231m.g,Ciclev10027485m.g	scaffold_7:19264758-19277183	4.84784	14.6283	1.59335
Ciclev10019806m.g	scaffold_3:47615596-47617973	0.960878	2.90125	1.59425
Ciclev10024987m.g	scaffold_7:1905248-1910215	10.868	32.8661	1.59652
Ciclev10008413m.g	scaffold_1:23668676-23673239	3.62449	10.9616	1.59661
Ciclev10026798m.g	scaffold_7:7002455-7004704	23.2036	70.177	1.59665
Ciclev10020447m.g	scaffold_3:21088026-21090566	0.782676	2.36926	1.59795
-	scaffold_7:17481226-17482416	5.78163	17.5181	1.5993
Ciclev10009803m.g	scaffold_1:9396867-9398773	2.16012	6.55619	1.60174
Ciclev10030109m.g	scaffold_8:19305845-19307173	1.78327	5.42644	1.60548
Ciclev10028353m.g	scaffold_8:4198086-4199601	10.1001	30.7397	1.60573
Ciclev10005496m.g	scaffold_9:28305830-28333082	1.76525	5.37523	1.60645
Ciclev10032346m.g	scaffold_4:3935676-3937432	2.43803	7.42529	1.60673
Ciclev10027806m.g	scaffold_8:17184361-17191255	0.301373	0.919556	1.60939
Ciclev10006438m.g	scaffold_9:6891592-6893622	0.541409	1.65224	1.60963
Ciclev10012763m.g	scaffold_6:19968259-19969180	7.33251	22.3774	1.60966
Ciclev10010341m.g	scaffold_1:19905420-19995040	0.222637	0.680429	1.61176
Ciclev10012518m.g	scaffold_6:14194453-14196761	8.0449	24.5888	1.61186
Ciclev10006122m.g	scaffold_9:15770091-15772553	3.04563	9.31223	1.61239
Ciclev10016943m.g	scaffold_2:27530639-27533140	11.7052	35.8569	1.61511
Ciclev10011213m.g	scaffold_6:20098727-20105049	1.61211	4.93854	1.61513
Ciclev10004292m.g	scaffold_9:4463236-4465833	0.655758	2.00959	1.61567
Ciclev10014698m.g	scaffold_2:29746360-29748343	17.197	52.7032	1.61574
Ciclev10027703m.g	scaffold_8:22445572-22451342	6.86207	21.0348	1.61606
Ciclev10027988m.g	scaffold_8:24277190-24282294	17.609	53.9919	1.61643
Ciclev10003568m.g	scaffold_5:39552843-39554535	1.89029	5.80112	1.61772
Ciclev10008731m.g	scaffold_1:4289573-4291259	5.16898	15.8765	1.61894
-	scaffold_3:41419553-41421648	10.7247	32.9699	1.62021
Ciclev10022024m.g	scaffold_3:13309397-13310636	9.21435	28.3457	1.62118
Ciclev10004229m.g,Ciclev10004266m.g,Ciclev10007058m.g	scaffold_9:24682631-24756976	8.92327	27.5083	1.62422
Ciclev10005812m.g	scaffold_9:5569630-5570998	17.5901	54.2272	1.62425
Ciclev10028237m.g	scaffold_8:2443425-2451856	2.51583	7.76236	1.62546
Ciclev10021692m.g	scaffold_3:44570652-44573213	0.494357	1.52607	1.62619
Ciclev10000220m.g	scaffold_5:36963546-36968045	56.3879	174.21	1.62737
Ciclev10016655m.g	scaffold_2:25973420-25975797	4.59715	14.253	1.63246
Ciclev10012067m.g	scaffold_6:22422263-22423561	4.30557	13.3525	1.63283
Ciclev10004062m.g	scaffold_5:38703230-38703922	15.1612	47.0447	1.63365
Ciclev10001818m.g	scaffold_5:43031750-43032859	0.159495	0.495532	1.63546
-	scaffold_5:17002680-17002977	3.87664	12.0491	1.63604
Ciclev10001981m.g	scaffold_5:43115215-43116448	1.00564	3.13173	1.63885
Ciclev10029710m.g	scaffold_8:2433769-2434541	6.32082	19.6948	1.63963
Ciclev10019032m.g	scaffold_3:6180648-6183653	112.874	351.927	1.64057
Ciclev10033987m.g	scaffold_4:1880495-1882331	4.83017	15.0747	1.64199
Ciclev10002416m.g	scaffold_5:36427010-36428163	2.2775	7.11592	1.6436
Ciclev10002725m.g	scaffold_5:29032157-29033401	3.6947	11.547	1.64399
Ciclev10006218m.g	scaffold_9:14612551-14614247	0.407246	1.27436	1.64579
Ciclev10019294m.g	scaffold_3:43499108-43502911	3.59571	11.2542	1.64611
Ciclev10021666m.g	scaffold_3:37912397-37914894	2.34102	7.33008	1.64669

Ciclev10014452m.g	scaffold_2:12575329-12583762	11.6484	36.4734	1.64671
Ciclev10004048m.g	scaffold_5:33929649-33931104	0.592987	1.85803	1.6477
Ciclev10031247m.g	scaffold_4:22035401-22037541	5.03575	15.8007	1.64971
Ciclev10010964m.g	scaffold_6:19650956-19654079	0.748065	2.34732	1.64978
-	scaffold_5:18031027-18031222	13.4159	42.1143	1.65036
-	scaffold_9:1290697-1291184	2.51919	7.90927	1.65059
Ciclev10009260m.g	scaffold_1:17418248-17419200	6.28474	19.7406	1.65124
Ciclev10003223m.g	scaffold_5:30703989-30705227	0.360092	1.13282	1.65348
Ciclev10030032m.g	scaffold_8:4292179-4375670	0.0783402	0.246507	1.65381
Ciclev10024709m.g	scaffold_7:10281834-10421429	0.47485	1.49504	1.65464
Ciclev10011449m.g	scaffold_6:24409993-24412120	0.898817	2.83329	1.65638
Ciclev10022177m.g	scaffold_3:42043036-42044066	1.51038	4.7638	1.6572
-	scaffold_9:28489337-28490199	1.54129	4.86214	1.65746
Ciclev10014445m.g	scaffold_2:30317146-30319476	0.545332	1.72093	1.65798
Ciclev10030449m.g	scaffold_978:1386-4908	5.54849	17.5501	1.66131
Ciclev10024707m.g,Ciclev10024710m.g,Ciclev10024711m.g,Ciclev10024715m.g,Ciclev10027401m.g	scaffold_7:10281834-10421429	24.6168	77.873	1.66148
Ciclev10028503m.g	scaffold_8:1398234-1404988	28.7288	90.9549	1.66266
-	scaffold_5:19865022-20070734	2.04085	6.47228	1.66511
Ciclev10001620m.g,Ciclev10001623m.g,Ciclev10001648m.g	scaffold_5:29233648-29276842	23.1352	73.3786	1.66527
Ciclev10013652m.g	scaffold_6:16023581-16026609	0.396621	1.25888	1.66631
Ciclev10011274m.g	scaffold_6:20400291-20402202	0.284343	0.903711	1.66823
Ciclev10022566m.g	scaffold_3:2128332-2130417	2.58573	8.22055	1.66866
Ciclev10014579m.g	scaffold_2:8756347-8761120	0.0780693	0.248228	1.66884
Ciclev10025208m.g	scaffold_7:5932627-5935568	4.35325	13.8427	1.66896
Ciclev10020257m.g	scaffold_3:6200206-6201965	0.588334	1.87149	1.66948
Ciclev10031737m.g	scaffold_4:19318980-19320538	0.169864	0.540669	1.67037
-	scaffold_7:6995029-6996371	8.50882	27.0961	1.67105
Ciclev10025387m.g	scaffold_7:2696563-2699816	0.0856514	0.272848	1.67155
Ciclev10006682m.g	scaffold_9:4142538-4146072	0.157815	0.503214	1.67294
Ciclev10019312m.g	scaffold_3:45857630-45864043	0.643935	2.05481	1.67401
-	scaffold_5:25221155-25545745	2.48493	7.93531	1.67508
Ciclev10032203m.g	scaffold_4:15410724-15413831	1.72646	5.5168	1.67602
Ciclev10008411m.g	scaffold_1:23265680-23304211	3.6678	11.7317	1.67742
Ciclev10005971m.g	scaffold_9:3833804-3834728	5.26302	16.8464	1.67848
Ciclev10026978m.g	scaffold_7:9930764-9934489	0.19004	0.610308	1.68324
Ciclev10015830m.g	scaffold_2:27768962-27771217	0.751762	2.42044	1.68692
-	scaffold_8:24630025-24630549	3.17267	10.2411	1.69059
-	scaffold_5:24926144-25131690	4.24482	13.7122	1.69169
Ciclev10024830m.g	scaffold_7:17159730-17163074	3.12302	10.1094	1.69468
Ciclev10016256m.g	scaffold_2:26083155-26086391	21.3726	69.2405	1.69585
Ciclev10021076m.g	scaffold_3:46474776-46476646	0.25815	0.837346	1.69761
-	scaffold_8:16495049-16495823	21.8886	71.1198	1.70007
Ciclev10011192m.g	scaffold_6:24866184-24869560	2.42785	7.90464	1.70302
Ciclev10027508m.g	scaffold_7:19234195-19235072	6.66554	21.7128	1.70375
Ciclev10000236m.g	scaffold_5:32990159-32995860	13.1705	42.928	1.70461
Ciclev10012261m.g	scaffold_6:20146652-20148628	7.2504	23.6329	1.70466
Ciclev10031064m.g	scaffold_4:1985412-1987612	27.058	88.2509	1.70556
Ciclev10019620m.g	scaffold_3:22358093-22360230	0.834024	2.73052	1.71101
Ciclev10004264m.g	scaffold_9:6232149-6235801	0.317123	1.03936	1.71259
Ciclev10026001m.g	scaffold_7:18468709-18470598	0.315629	1.03629	1.71513
Ciclev10019788m.g	scaffold_3:45738968-45744379	5.05844	16.6181	1.716

Ciclev10007972m.g	scaffold_1:20995351-21034862	72.8518	239.377	1.71624
Ciclev10003037m.g	scaffold_5:19692511-19713573	0.409856	1.34702	1.71659
Ciclev10030784m.g	scaffold_4:11798333-11800710	0.667964	2.19758	1.71807
Ciclev10029500m.g	scaffold_8:20294505-20295288	0.324953	1.06991	1.71918
Ciclev10029758m.g	scaffold_8:23143377-23146210	0.629506	2.07365	1.71988
-	scaffold_5:3968429-3969882	1.59581	5.27116	1.72383
Ciclev10002343m.g	scaffold_5:42222977-42226094	1.11718	3.69243	1.7247
-	scaffold_8:1195538-1195866	29.0153	96.0327	1.72671
Ciclev10019753m.g	scaffold_3:29485032-29487092	0.990184	3.27729	1.72673
Ciclev10028646m.g	scaffold_8:1914528-1917827	2.23899	7.42633	1.7298
Ciclev10011057m.g	scaffold_6:16735134-16738033	0.153701	0.50981	1.72984
Ciclev10013519m.g	scaffold_6:6593965-6594899	0.967436	3.2119	1.73119
Ciclev10000117m.g	scaffold_5:43243673-43249114	0.0509994	0.169465	1.73244
Ciclev10001096m.g	scaffold_5:37199634-37201893	11.0075	36.6033	1.73349
Ciclev10023120m.g	scaffold_3:13991542-13993628	0.249521	0.82988	1.73374
Ciclev10007369m.g	scaffold_1:2089779-2096750	0.0504565	0.167817	1.73378
Ciclev10031900m.g	scaffold_4:19706562-19709138	0.451667	1.50251	1.73404
-	scaffold_2:15477859-15615778	4.03373	13.4241	1.73464
Ciclev10033448m.g,Ciclev10033550m.g	scaffold_4:22940199-22942611	4.12906	13.7425	1.73476
-	scaffold_3:42105181-42105373	29.71	98.9069	1.73512
Ciclev10000702m.g	scaffold_5:6482365-6484923	2.57775	8.5981	1.73791
Ciclev10010400m.g	scaffold_1:10556695-10559221	0.228579	0.76273	1.73848
Ciclev10030608m.g	scaffold_4:20287175-20291630	0.833694	2.78611	1.74066
-	scaffold_4:16291015-16291511	1.18595	3.96378	1.74083
Ciclev10014570m.g	scaffold_2:27030187-27034752	10.1388	33.9086	1.74176
Ciclev10004518m.g	scaffold_9:4663881-4697961	0.0934692	0.312657	1.74202
Ciclev10018771m.g	scaffold_3:31140607-31147183	0.250558	0.83834	1.74239
Ciclev10006521m.g	scaffold_9:20201293-20206747	0.112728	0.377187	1.74243
Ciclev10003587m.g	scaffold_5:17085588-17088502	2.7867	9.32554	1.74263
Ciclev10024523m.g	scaffold_3:32872983-32877956	0.45284	1.51669	1.74385
Ciclev10029970m.g	scaffold_8:23709067-23710855	0.102002	0.342051	1.74561
Ciclev10025158m.g	scaffold_7:16832246-16834201	0.184949	0.620369	1.746
Ciclev10028395m.g	scaffold_8:416712-422050	2.82639	9.4914	1.74766
Ciclev10023452m.g	scaffold_3:36332197-36333378	0.421227	1.41478	1.74791
-	scaffold_5:32831731-32863079	2.34712	7.88353	1.74795
Ciclev10003599m.g	scaffold_5:17678632-17679904	1.5653	5.26417	1.74977
Ciclev10012092m.g	scaffold_6:23149132-23152767	2.04097	6.87422	1.75194
Ciclev10019648m.g	scaffold_3:34094574-34096585	0.095348	0.321314	1.75271
Ciclev10015488m.g	scaffold_2:5821400-5825173	2.9733	10.0327	1.75457
Ciclev10015288m.g	scaffold_2:13602967-13604560	0.53491	1.80619	1.75558
-	scaffold_8:20950309-21005489	19.7977	66.8555	1.75571
Ciclev10029307m.g	scaffold_8:24159189-24160849	11.2556	38.0147	1.75591
Ciclev10024591m.g	scaffold_3:27952801-27953704	8.83492	29.8402	1.75597
Ciclev10030091m.g	scaffold_8:18577222-18579132	0.246687	0.833445	1.7564
Ciclev10000734m.g	scaffold_5:31307195-31309895	2.0631	6.98293	1.75902
Ciclev10014861m.g	scaffold_2:5975366-6022331	14.5243	49.1794	1.75958
Ciclev10018858m.g	scaffold_3:1344191-1349935	0.367607	1.24663	1.76179
Ciclev10031954m.g	scaffold_4:21898103-21903480	2.36935	8.04104	1.76289
Ciclev10000193m.g	scaffold_5:1411431-1414337	0.841441	2.85581	1.76297
Ciclev10015035m.g	scaffold_2:28168729-28171678	2.368	8.05012	1.76534
Ciclev10033648m.g	scaffold_4:11523554-11728349	1.90038	6.46476	1.76631
Ciclev10009810m.g	scaffold_1:24132094-24133290	21.6956	73.814	1.76649
Ciclev10004353m.g	scaffold_9:25430450-25437071	0.391426	1.33237	1.76718
Ciclev10005986m.g	scaffold_9:2177336-2179856	0.556609	1.89689	1.7689
-	scaffold_9:3269874-3270003	1672.31	5699.68	1.76904

Ciclev10000819m.g	scaffold_5:42957413-42961730	94.6291	323.09	1.77158
Ciclev10033434m.g	scaffold_4:24128866-24130158	4.93627	16.8587	1.772
Ciclev10000141m.g,Ciclev10003386m.g,Ciclev10003540m.g	scaffold_5:19865022-20070734	3.53904	12.0868	1.772
Ciclev10024266m.g	scaffold_3:21905999-21908234	0.264117	0.902556	1.77284
Ciclev10020717m.g	scaffold_3:40963773-40965744	9.59808	32.8032	1.77302
Ciclev10013610m.g	scaffold_6:13801313-13814032	1.12417	3.84354	1.77357
Ciclev10017345m.g	scaffold_2:19940400-19941012	106.323	363.614	1.77396
Ciclev10014334m.g	scaffold_2:5249719-5252152	0.0774508	0.265064	1.77499
Ciclev10014994m.g	scaffold_2:27428714-27432877	0.546805	1.87202	1.7755
Ciclev10012554m.g	scaffold_6:11704198-11706676	45.4045	155.658	1.77747
Ciclev10032730m.g	scaffold_4:15690355-15782388	0.155089	0.531869	1.77798
-	scaffold_4:2942866-2945994	0.974362	3.34384	1.77898
Ciclev10025008m.g	scaffold_7:11686788-11771145	0.0704765	0.242118	1.7805
Ciclev10024920m.g	scaffold_7:9119792-9125872	4.25184	14.6364	1.7834
Ciclev10023464m.g	scaffold_3:30618246-30668406	1.56307	5.38429	1.78438
Ciclev10028964m.g	scaffold_8:461188-468899	1.65734	5.71739	1.78649
Ciclev10033964m.g	scaffold_4:20642616-20644140	3.58471	12.367	1.78657
Ciclev10033888m.g	scaffold_4:18033199-18034351	0.194605	0.671479	1.78679
Ciclev10012846m.g	scaffold_6:18783833-18784757	0.268789	0.927887	1.78747
Ciclev10033623m.g	scaffold_4:17211717-17215345	0.141603	0.489897	1.79063
Ciclev10004922m.g	scaffold_9:29440187-29442935	17.7954	61.6477	1.79254
Ciclev10007182m.g	scaffold_9:2884733-2886821	0.746179	2.58583	1.79303
Ciclev10006774m.g	scaffold_9:20890036-21029395	0.158669	0.550172	1.79386
Ciclev10003894m.g	scaffold_5:32404311-32420675	0.499544	1.73229	1.794
Ciclev10027517m.g	scaffold_7:12207912-12210810	0.0619566	0.215034	1.79524
Ciclev10007386m.g	scaffold_1:21300057-21310677	0.0823409	0.285869	1.79567
Ciclev10028028m.g	scaffold_8:20148632-20155238	5.47532	19.0098	1.79573
Ciclev10020076m.g	scaffold_3:7897950-7899836	5.00749	17.4026	1.79715
Ciclev10006300m.g,Ciclev10006380m.g	scaffold_9:1440855-1478113	27.4911	95.6865	1.79935
Ciclev10012151m.g	scaffold_6:4232130-4234093	0.65365	2.27849	1.80149
Ciclev10016956m.g	scaffold_2:6911305-6912781	0.916657	3.20164	1.80436
Ciclev10004103m.g	scaffold_27:62969-65013	0.106647	0.372535	1.80453
Ciclev10007257m.g	scaffold_1:1029921-1040732	0.183642	0.642737	1.80733
Ciclev10005368m.g	scaffold_9:23487944-23490847	0.27835	0.97425	1.80739
Ciclev10009575m.g	scaffold_1:4306288-4307367	0.782549	2.74411	1.81008
Ciclev10028481m.g	scaffold_8:6373588-6374942	0.161767	0.568116	1.81227
Ciclev10008407m.g	scaffold_1:2835449-2841581	0.98254	3.455	1.8141
Ciclev10024083m.g	scaffold_3:31770209-32016548	0.212402	0.747587	1.81544
Ciclev10015138m.g	scaffold_2:15477859-15615778	0.623028	2.19553	1.8172
Ciclev10012710m.g	scaffold_6:15177285-15178154	2.99187	10.5887	1.82341
-	scaffold_5:8216834-8217182	7.41907	26.2691	1.82406
Ciclev10002204m.g,Ciclev10003407m.g	scaffold_5:8159352-8216590	6.15909	21.814	1.82447
Ciclev10022942m.g	scaffold_3:1155549-1156499	18.8715	66.8413	1.82453
Ciclev10000528m.g	scaffold_5:36680107-36686830	16.3473	57.9555	1.8259
Ciclev10014524m.g	scaffold_2:460773-463498	0.259888	0.92301	1.82846
Ciclev10004104m.g	scaffold_27:33569-36958	6.99294	24.8595	1.82983
Ciclev10002406m.g	scaffold_5:35913858-35917123	44.5348	158.341	1.83003
Ciclev10018914m.g	scaffold_3:24629369-24734809	0.0804688	0.286321	1.83113
Ciclev10017011m.g	scaffold_2:7273711-7336406	2.30431	8.20148	1.83155
Ciclev10016425m.g	scaffold_2:33937786-33940051	3.02835	10.7878	1.8328
Ciclev10024485m.g	scaffold_3:21982709-21986624	0.121456	0.432833	1.83338
Ciclev10019095m.g	scaffold_3:1531642-1535015	13.8822	49.4796	1.83359

Ciclev10012886m.g	scaffold_6:24161532-24163143	2.0047	7.15009	1.83458
Ciclev10015106m.g	scaffold_2:32315938-32318574	0.849873	3.03821	1.8379
Ciclev10012763m.g	scaffold_6:19968259-19969180	3.14238	11.2397	1.83868
Ciclev10025863m.g	scaffold_7:2703579-2705194	0.362496	1.29795	1.8402
Ciclev10017953m.g	scaffold_2:29764354-29764735	1.00636	3.6069	1.84162
Ciclev10030777m.g,Ciclev10031026m.g	scaffold_4:1553214-1585121	7.40977	26.5729	1.84246
Ciclev10004247m.g	scaffold_9:24682631-24756976	1.06024	3.80282	1.84268
Ciclev10005850m.g,Ciclev10005851m.g	scaffold_9:7804322-8050719	6.69729	24.0344	1.84345
Ciclev10021734m.g	scaffold_3:26976582-26978658	0.419752	1.50636	1.84346
Ciclev10001939m.g	scaffold_5:42432895-42436163	2.16502	7.77142	1.8438
Ciclev10033947m.g	scaffold_4:2493519-2495321	0.865215	3.10756	1.84465
Ciclev10031742m.g	scaffold_4:21728370-21730627	1.70735	6.13541	1.84541
Ciclev10025490m.g	scaffold_7:2356951-2358892	0.469015	1.68725	1.84696
Ciclev10007294m.g	scaffold_1:21964188-21969517	0.115063	0.414101	1.84756
Ciclev10034005m.g	scaffold_4:9834092-9835443	2.42488	8.73557	1.84899
Ciclev10011595m.g	scaffold_6:16798475-16801193	1.64091	5.93166	1.85394
Ciclev10024088m.g	scaffold_3:32598704-32599862	0.378975	1.37298	1.85714
Ciclev10012901m.g	scaffold_6:17223123-17224258	3.70451	13.4602	1.86134
Ciclev10013248m.g	scaffold_6:22902372-22902940	2.64717	9.63889	1.86441
Ciclev10025139m.g	scaffold_7:12352172-12754529	0.5168	1.88236	1.86487
Ciclev10011082m.g	scaffold_6:17184522-17199957	3.62817	13.2711	1.87098
Ciclev10031017m.g	scaffold_4:11152390-11156903	2.79297	10.2374	1.87398
Ciclev10014222m.g	scaffold_2:7084062-7088036	0.71891	2.6513	1.88282
Ciclev10028688m.g,Ciclev10028699m.g,Ciclev10029921m.g	scaffold_8:3843789-3891595	31.5982	116.604	1.8837
Ciclev10012561m.g	scaffold_6:11694661-11696169	11.0253	40.8168	1.88834
Ciclev10015577m.g	scaffold_2:27167288-27171203	66.4875	246.578	1.89089
-	scaffold_6:24671372-24672476	3.07849	11.4174	1.89094
Ciclev10022186m.g	scaffold_3:47302694-47304614	9.07857	33.6813	1.89141
-	scaffold_7:6993765-6994947	1.88922	7.01826	1.89332
-	scaffold_2:24401745-24433873	137.918	512.579	1.89396
Ciclev10004323m.g	scaffold_9:30892247-30894820	1.63428	6.10155	1.90052
Ciclev10010615m.g	scaffold_1:8961640-8964866	0.383713	1.43315	1.90109
Ciclev10025114m.g	scaffold_7:14913725-14915981	0.955459	3.56934	1.90139
Ciclev10001721m.g	scaffold_5:29643474-29645970	0.23964	0.895352	1.90159
Ciclev10023580m.g	scaffold_3:6931627-6932155	0.47595	1.77933	1.90245
Ciclev10004069m.g	scaffold_5:28289811-28536377	1.79679	6.74156	1.90766
Ciclev10010119m.g	scaffold_1:13123279-13126225	0.912017	3.43318	1.91241
Ciclev10015126m.g	scaffold_2:16207262-16210651	7.96517	30.0076	1.91355
Ciclev10008336m.g,Ciclev10009274m.g	scaffold_1:1352943-1372522	2.72486	10.2817	1.91582
-	scaffold_9:1437943-1438287	14.0775	53.1288	1.9161
Ciclev10027582m.g	scaffold_7:11850299-12160617	0.198643	0.750082	1.91687
Ciclev10016752m.g	scaffold_2:32529419-32532287	4.12208	15.5845	1.91867
Ciclev10018323m.g	scaffold_2:6156047-6158225	0.10062	0.380991	1.92084
Ciclev10007236m.g	scaffold_1:21975140-22013742	0.838601	3.17575	1.92104
Ciclev10011753m.g	scaffold_6:23019784-23022324	0.244724	0.927154	1.92165
Ciclev10007520m.g	scaffold_1:28658749-28661954	1.75537	6.673	1.92656
Ciclev10000769m.g	scaffold_5:20113856-20116341	3.77754	14.3758	1.92813
Ciclev10009087m.g	scaffold_1:9724204-9725697	4.3977	16.7644	1.93058
Ciclev10001346m.g	scaffold_5:347068-349282	15.3265	58.4946	1.93227
Ciclev10016267m.g	scaffold_2:13963798-13966850	3.38874	12.9671	1.93603
-	scaffold_4:21724566-21725178	0.838457	3.20999	1.93676

Ciclev10027439m.g	scaffold_7:4861024-4880959	0.364481	1.39597	1.93735
Ciclev10018528m.g	scaffold_3:27861794-27874202	4.39959	16.8631	1.93843
Ciclev10020819m.g	scaffold_3:31463778-31467068	1.16172	4.45311	1.93855
Ciclev10020963m.g	scaffold_3:45110214-45112873	22.5632	86.4928	1.93861
Ciclev10001429m.g	scaffold_5:34891099-34894176	0.420724	1.61343	1.93919
Ciclev10012398m.g	scaffold_6:24516036-24517772	4.61699	17.7464	1.9425
Ciclev10028278m.g	scaffold_8:9325618-9585469	4.78661	18.4242	1.94452
-	scaffold_8:22047276-22047742	5.31199	20.4656	1.94588
Ciclev10033839m.g	scaffold_4:22324780-22329618	8.47132	32.6905	1.94821
Ciclev10007069m.g	scaffold_9:15270818-15272030	0.656109	2.53547	1.95024
Ciclev10008319m.g	scaffold_1:20664826-20823973	4.96096	19.2049	1.95278
Ciclev10019509m.g	scaffold_3:28037825-28040140	0.545932	2.11419	1.95331
-	scaffold_9:1440855-1478113	4.21949	16.4071	1.95918
Ciclev10031077m.g	scaffold_4:20622923-20626646	3.08713	12.0084	1.9597
Ciclev10026392m.g	scaffold_7:19458241-19460738	1.80829	7.03586	1.9601
Ciclev10016254m.g	scaffold_2:28285183-28286267	0.174166	0.679051	1.96306
Ciclev10005646m.g	scaffold_9:14552135-14553570	0.298221	1.1638	1.96439
Ciclev10024835m.g	scaffold_7:11850299-12160617	0.113484	0.442876	1.96441
Ciclev10007451m.g,Ciclev10007464m.g,Ciclev10007466m.g,Ciclev10010587m.g	scaffold_1:19612175-19653994	10.4772	40.9029	1.96495
Ciclev10010036m.g	scaffold_1:17554648-17556053	1933.65	7550.14	1.96518
Ciclev10029021m.g	scaffold_8:23982040-23983893	7.86302	30.7107	1.96559
Ciclev10027253m.g	scaffold_7:5236061-5236430	1.52866	5.97089	1.96568
Ciclev10001082m.g	scaffold_5:17984020-17985454	0.594689	2.32554	1.96736
Ciclev10012617m.g	scaffold_6:18190872-18193790	0.219107	0.858241	1.96975
Ciclev10018397m.g	scaffold_2:34961912-34964011	1.31326	5.14527	1.97009
Ciclev10011614m.g	scaffold_6:24416604-24418297	10.7456	42.1407	1.97147
Ciclev10021349m.g	scaffold_3:27993864-27998641	0.725693	2.84834	1.97269
Ciclev10016707m.g	scaffold_2:33354367-33355106	0.363937	1.42893	1.97318
Ciclev10023398m.g	scaffold_3:41035476-41037184	0.639005	2.5155	1.97695
-	scaffold_1:14852159-14852579	1.62558	6.40386	1.97799
Ciclev10016221m.g	scaffold_2:14702407-14706158	46.1032	181.843	1.97975
Ciclev10001266m.g	scaffold_5:40192463-40194246	2.49745	9.8646	1.98181
Ciclev10026893m.g	scaffold_7:16939640-16973171	1.83987	7.28161	1.98465
Ciclev10001726m.g	scaffold_5:38960062-38963401	4.92796	19.5132	1.98539
Ciclev10029277m.g,Ciclev10029278m.g	scaffold_8:3754499-3783084	5.29391	21.0063	1.98842
Ciclev10007994m.g	scaffold_1:8479610-8481816	8.52023	33.8706	1.99107
Ciclev10006927m.g	scaffold_9:30447133-30449719	0.843557	3.3552	1.99184
Ciclev10002988m.g	scaffold_5:36767716-36768536	106.391	423.187	1.99192
Ciclev10010626m.g	scaffold_1:8422904-8424670	13.602	54.2427	1.99561
Ciclev10017773m.g	scaffold_2:6047974-6048544	0.462782	1.84607	1.99605
Ciclev10028300m.g	scaffold_8:24007860-24043482	2.8689	11.4517	1.997
Ciclev10027402m.g	scaffold_7:9347349-9351521	2.3846	9.52739	1.99833
Ciclev10026022m.g	scaffold_7:9442634-9445710	0.397861	1.58994	1.99863
Ciclev10009699m.g	scaffold_1:24349192-24351132	10.7565	42.9936	1.99892
Ciclev10014457m.g	scaffold_2:30682399-30686499	0.070518	0.282197	2.00064
Ciclev10009783m.g	scaffold_1:26863714-26864182	7.39842	29.6196	2.00126
Ciclev10017971m.g	scaffold_2:4124020-4126939	0.0782717	0.313454	2.00169
Ciclev10031400m.g	scaffold_4:16817583-16984703	0.333939	1.33747	2.00185
Ciclev10015857m.g	scaffold_2:30296874-30298160	0.304683	1.22124	2.00297
Ciclev10028712m.g	scaffold_8:2751584-2753575	0.234604	0.941367	2.00453
Ciclev10029985m.g	scaffold_8:7698015-7862701	9.7674	39.1971	2.0047
Ciclev10005201m.g	scaffold_9:15141679-15146508	13.7394	55.1518	2.00509
Ciclev10022368m.g	scaffold_3:40744172-40747967	0.241637	0.970572	2.00599

Ciclev10031846m.g	scaffold_4:19635414-19637061	18.0006	72.4231	2.00841
Ciclev10018950m.g	scaffold_3:3535833-3540825	5.69532	22.9374	2.00985
Ciclev10010150m.g	scaffold_1:22635900-22637346	0.134128	0.540553	2.01082
Ciclev10004310m.g	scaffold_9:29731923-29738569	3.02757	12.2427	2.01569
Ciclev10008993m.g	scaffold_1:9290164-9291548	0.167852	0.679085	2.0164
Ciclev10015986m.g	scaffold_2:29036245-29037753	0.422636	1.71479	2.02054
Ciclev10006998m.g	scaffold_9:5407960-5409728	0.135844	0.552688	2.02451
Ciclev10014659m.g	scaffold_2:7507436-7569782	0.475148	1.93585	2.02652
Ciclev10016643m.g	scaffold_2:32283518-32286736	4.85508	19.8214	2.02949
Ciclev10010584m.g	scaffold_1:18763579-18765093	0.142028	0.579916	2.02967
-	scaffold_3:27139030-27141471	1.84556	7.54835	2.0321
Ciclev10006633m.g	scaffold_9:29711413-29713699	0.117436	0.480437	2.03247
Ciclev10033976m.g	scaffold_4:13917008-13918467	56.393	230.832	2.03325
Ciclev10019820m.g	scaffold_3:47121092-47124518	4.64161	19.027	2.03535
Ciclev10021462m.g	scaffold_3:23472673-23473723	0.237989	0.975958	2.03592
Ciclev10033306m.g	scaffold_4:11940407-11943644	0.217113	0.891102	2.03715
Ciclev10003877m.g	scaffold_5:19865022-20070734	0.794061	3.26489	2.03971
Ciclev10015145m.g	scaffold_2:25422296-25492273	0.124145	0.510629	2.04025
Ciclev10022512m.g,Ciclev10022725m.g	scaffold_3:13698282-13704788	42.2849	173.942	2.04039
-	scaffold_3:35372249-35712253	4.21438	17.3395	2.04067
Ciclev10008595m.g	scaffold_1:16739007-16742148	0.36276	1.49259	2.04073
Ciclev10019217m.g	scaffold_3:27827994-27830405	0.805891	3.32162	2.04323
Ciclev10007523m.g	scaffold_1:4780911-4783394	0.37582	1.54902	2.04325
Ciclev10017349m.g	scaffold_2:19962334-19963097	0.341809	1.41404	2.04856
Ciclev10014915m.g	scaffold_2:28673581-28677741	2.58847	10.7231	2.05055
Ciclev10006992m.g	scaffold_9:10360492-10368126	0.04456	0.184722	2.05154
Ciclev10022187m.g	scaffold_3:8946824-8947475	0.374118	1.55124	2.05186
Ciclev10013517m.g	scaffold_6:2381893-2382298	0.970775	4.02722	2.05258
Ciclev10026404m.g	scaffold_7:2721714-2817730	4.78604	19.8987	2.05577
Ciclev10025353m.g	scaffold_7:6817260-6819191	0.583243	2.42522	2.05595
Ciclev10030037m.g,Ciclev10030156m.g,Ciclev10030238m.g	scaffold_8:20620043-20723794	4.37753	18.2082	2.0564
Ciclev10003224m.g	scaffold_5:32017860-32153975	15.1178	63.0613	2.0605
Ciclev10011832m.g	scaffold_6:2292106-2295403	6.42509	26.8144	2.06122
Ciclev10011134m.g	scaffold_6:10000923-10003899	0.484019	2.02186	2.06255
Ciclev10016765m.g	scaffold_2:30884478-30885712	0.972542	4.0652	2.0635
Ciclev10017611m.g	scaffold_2:32701245-32703799	0.366985	1.53579	2.06519
Ciclev10011255m.g	scaffold_6:13052096-13054052	0.0858246	0.359268	2.0656
Ciclev10023640m.g	scaffold_3:19171727-19172666	0.287387	1.20391	2.06666
Ciclev10025660m.g	scaffold_7:17208074-17211871	3.96654	16.6501	2.06958
Ciclev10004433m.g	scaffold_9:3779319-3781672	1.03155	4.34225	2.07363
Ciclev10032298m.g	scaffold_4:954715-956225	3.26892	13.761	2.0737
Ciclev10014161m.g	scaffold_2:175428-179259	0.066464	0.280429	2.07699
Ciclev10014255m.g	scaffold_2:32593506-32599641	3.75233	15.8505	2.07867
Ciclev10022123m.g	scaffold_3:36817012-36818205	43.3257	183.057	2.07899
Ciclev10021418m.g	scaffold_3:37921994-37929048	0.505303	2.14017	2.0825
Ciclev10013547m.g,Ciclev10013584m.g	scaffold_6:14932984-15010648	3.37579	14.3221	2.08495
Ciclev10000275m.g	scaffold_5:30209767-30213485	0.115043	0.488879	2.0873
Ciclev10003027m.g	scaffold_5:24456299-24464558	0.300789	1.27908	2.08828
Ciclev10017226m.g	scaffold_2:7480081-7480758	3.35233	14.2647	2.08921
Ciclev10014845m.g	scaffold_2:23829861-23832918	11.1864	47.8551	2.09692
Ciclev10032651m.g	scaffold_4:23923756-23925178	0.423179	1.81105	2.09749
Ciclev10030487m.g	scaffold_4:13448427-13455670	0.128675	0.551361	2.09926

Ciclev10007545m.g	scaffold_1:24145774-24149646	2.93425	12.5812	2.1002
Ciclev10018573m.g	scaffold_3:27855181-27861301	0.328613	1.4106	2.10185
Ciclev10002747m.g	scaffold_5:38789127-38789955	0.301014	1.29239	2.10214
Ciclev10025465m.g	scaffold_7:20690093-20692017	2.32453	9.98547	2.10289
Ciclev10023885m.g	scaffold_3:23867821-23871328	0.123757	0.533947	2.10919
Ciclev10033566m.g	scaffold_4:17970260-17972867	0.124454	0.538485	2.11329
Ciclev10004252m.g	scaffold_9:20890036-21029395	0.0793314	0.343629	2.11489
Ciclev10011084m.g	scaffold_6:13195954-13201816	2.08238	9.05083	2.11982
Ciclev10007531m.g	scaffold_1:9428840-9434694	19.7203	85.8983	2.12295
Ciclev10031197m.g	scaffold_4:16079268-16089881	0.19873	0.866055	2.12365
-	scaffold_3:45042528-45042715	17.7831	77.6887	2.1272
Ciclev10017659m.g	scaffold_2:6664288-6666265	0.0898076	0.392846	2.12905
-	scaffold_5:9447952-9448976	0.8214	3.59713	2.13069
Ciclev10022951m.g	scaffold_3:14776219-14776871	8.39002	36.7714	2.13184
Ciclev10001326m.g	scaffold_5:29339627-29387910	0.266324	1.17037	2.13571
Ciclev10025175m.g	scaffold_7:9810928-9813429	0.598519	2.64806	2.14546
Ciclev10027194m.g	scaffold_7:747118-747856	4.93956	21.873	2.1467
Ciclev10032157m.g	scaffold_4:3994697-3998355	0.566016	2.50656	2.1468
Ciclev10016958m.g	scaffold_2:35600894-35602157	1.44904	6.41987	2.14745
Ciclev10007177m.g	scaffold_9:5493707-5510148	35.1657	155.941	2.14875
Ciclev10004791m.g	scaffold_9:29984483-29989049	1.64561	7.30835	2.15092
Ciclev10013860m.g	scaffold_6:25322359-25325348	4.05342	18.008	2.15143
Ciclev10016471m.g	scaffold_2:23800913-23802482	4.13779	18.3882	2.15185
Ciclev10018657m.g	scaffold_3:3582058-3586955	0.731222	3.25084	2.15243
Ciclev10017664m.g	scaffold_2:16492221-16499995	1.13196	5.03848	2.15416
Ciclev10023938m.g	scaffold_3:22023775-22029457	0.660598	2.94131	2.15462
Ciclev10033191m.g	scaffold_4:19836359-19837449	0.280592	1.25282	2.15864
Ciclev10019629m.g	scaffold_3:49720727-49724239	0.197605	0.882301	2.15865
Ciclev10005338m.g	scaffold_9:28267304-28268543	1.14705	5.12514	2.15966
Ciclev10008765m.g	scaffold_1:21082901-21086082	2.43602	10.9071	2.16267
Ciclev10025169m.g	scaffold_7:2049067-2051708	3.72103	16.7607	2.17131
Ciclev10026754m.g	scaffold_7:7700119-7702124	8.2615	37.2172	2.17149
Ciclev10026344m.g	scaffold_7:3362859-3364363	2.87341	12.999	2.17756
Ciclev10026283m.g	scaffold_7:8506090-8508795	7.61618	34.4572	2.17767
Ciclev10014814m.g	scaffold_2:35752167-35756328	0.95623	4.33125	2.17936
Ciclev10027103m.g	scaffold_7:6887389-6888718	1.24008	5.61911	2.17991
Ciclev10029208m.g	scaffold_8:2534277-2535315	2.07155	9.3978	2.18162
Ciclev10023149m.g	scaffold_3:21981822-21982343	0.600541	2.72574	2.18231
Ciclev10020145m.g	scaffold_3:41869947-41871954	1.5362	6.98334	2.18455
Ciclev10009092m.g	scaffold_1:3526496-3530029	0.302079	1.3754	2.18685
Ciclev10012180m.g	scaffold_6:16924570-16927200	1.92277	8.75458	2.18685
-	scaffold_3:22031856-22032428	1.39913	6.37406	2.18769
Ciclev10005785m.g	scaffold_9:13161608-13163121	1.13077	5.15588	2.18891
Ciclev10025754m.g	scaffold_7:4041509-4043117	0.1672	0.763289	2.19066
Ciclev10006505m.g,Ciclev10006802m.g	scaffold_9:15639029-15642935	3.078	14.091	2.19471
Ciclev10012859m.g	scaffold_6:19072173-19073636	2.95965	13.5713	2.19706
Ciclev10019466m.g	scaffold_3:45012130-45014455	0.301591	1.38693	2.20123
Ciclev10024388m.g	scaffold_3:44080659-44083536	0.563188	2.59049	2.20154
Ciclev10000338m.g	scaffold_5:24926144-25131690	1.44832	6.68079	2.20564
-	scaffold_8:8274096-8274737	0.781191	3.60741	2.20722
Ciclev10033020m.g	scaffold_4:19860573-19862744	8.03916	37.186	2.20964
Ciclev10030898m.g	scaffold_4:11319739-11326020	4.68077	21.6865	2.21198
-	scaffold_5:1552172-1984270	1.36023	6.31028	2.21386
Ciclev10011390m.g	scaffold_6:16192356-16196105	1.82271	8.46885	2.21608

Ciclev10005195m.g,Ciclev10005316m.g,Ciclev10006696m.g	scaffold_9:21248441-21518846	57.3351	266.623	2.21731
Ciclev10028095m.g	scaffold_8:24226754-24229739	6.08447	28.3617	2.22074
Ciclev10028217m.g	scaffold_8:24007860-24043482	1.47576	6.8869	2.2224
Ciclev10012593m.g	scaffold_6:23850969-23856058	0.147143	0.688411	2.22605
-	scaffold_5:42365094-42365253	64.4047	301.684	2.2278
Ciclev10015140m.g,Ciclev10015155m.g	scaffold_2:25493711-25501915	5.27196	24.7476	2.23088
Ciclev10006663m.g	scaffold_9:10189845-10195548	0.0953	0.448953	2.23602
Ciclev10030424m.g	scaffold_8:23634205-23639792	1.00355	4.72831	2.23621
Ciclev10032534m.g	scaffold_4:2964200-2965543	29.2947	138.763	2.24391
Ciclev10032380m.g	scaffold_4:21990483-21994382	1.80644	8.6099	2.25285
Ciclev10024285m.g	scaffold_3:28065477-28341901	0.126043	0.601549	2.25477
Ciclev10026057m.g	scaffold_7:370951-372493	1.34546	6.46741	2.26509
Ciclev10016980m.g	scaffold_2:11362237-11363252	125.264	602.615	2.26626
Ciclev10030988m.g	scaffold_4:7026873-7029119	8.97062	43.1907	2.26744
Ciclev10031315m.g	scaffold_4:23840999-23842995	1.25722	6.08724	2.27555
Ciclev10022104m.g	scaffold_3:7073186-7074114	4.73224	22.9263	2.27641
Ciclev10005833m.g	scaffold_9:7770683-7771919	1.10728	5.37663	2.27968
Ciclev10028304m.g	scaffold_8:5661830-5663654	2.45398	11.9267	2.281
Ciclev10027208m.g	scaffold_7:359258-362043	0.336347	1.64072	2.28631
Ciclev10014204m.g	scaffold_2:5469049-5475142	0.123864	0.604907	2.28795
Ciclev10024212m.g	scaffold_3:23886297-23890863	0.176398	0.865301	2.29436
Ciclev10000227m.g	scaffold_5:33286115-33291455	0.116719	0.573514	2.29679
Ciclev10016829m.g	scaffold_2:23695880-23697100	39.834	196.274	2.30079
Ciclev10017695m.g	scaffold_2:26105694-26158658	0.074344	0.366638	2.30207
Ciclev10018179m.g	scaffold_2:13627649-13630405	0.269998	1.33212	2.30271
Ciclev10029768m.g	scaffold_8:20756546-20759427	1.2489	6.16563	2.3036
Ciclev10022596m.g	scaffold_3:37452120-37452833	0.390227	1.92854	2.30512
-	scaffold_8:21762040-21767256	11.0429	54.627	2.3065
Ciclev10022922m.g	scaffold_3:25591666-25592171	0.491233	2.43395	2.30882
Ciclev10006836m.g	scaffold_9:4636689-4638899	0.208367	1.03552	2.31315
Ciclev10023027m.g	scaffold_3:36573009-36577672	0.478102	2.3784	2.3146
Ciclev10029888m.g	scaffold_8:4482515-4485560	0.169433	0.845406	2.31893
Ciclev10027040m.g	scaffold_7:16843104-16845450	0.298863	1.49129	2.319
Ciclev10012386m.g	scaffold_6:16788757-16790403	2.16978	10.8375	2.32041
Ciclev10011948m.g	scaffold_6:25086390-25088722	1.54238	7.71142	2.32184
Ciclev10014458m.g	scaffold_2:6586730-6589503	0.0998293	0.500098	2.32467
Ciclev10029622m.g	scaffold_8:7327125-7328004	1.28157	6.42974	2.32685
Ciclev10028742m.g	scaffold_8:3514030-3515256	0.979563	4.91781	2.32781
Ciclev10014130m.g	scaffold_2:8693073-8696367	0.119661	0.601991	2.33079
Ciclev10008628m.g	scaffold_1:18298598-18300295	11.7958	59.3895	2.33193
Ciclev10029294m.g	scaffold_8:6253005-6254421	0.223667	1.13102	2.3382
Ciclev10017437m.g	scaffold_2:12586850-12593702	0.869702	4.3984	2.33839
Ciclev10031246m.g	scaffold_4:12812216-12813776	0.11903	0.602349	2.33928
Ciclev10017113m.g	scaffold_2:12112789-12113903	234.808	1188.44	2.33952
Ciclev10019305m.g	scaffold_3:41513566-41516390	0.493024	2.49635	2.34009
Ciclev10010036m.g	scaffold_1:17554648-17556053	11.0584	56.1445	2.344
Ciclev10014264m.g	scaffold_2:30287229-30291578	0.106623	0.541809	2.34526
Ciclev10020596m.g	scaffold_3:36561445-36563068	0.149029	0.758408	2.34739
Ciclev10023560m.g	scaffold_3:44644976-44648303	1.95204	9.94393	2.34883
Ciclev10002439m.g	scaffold_5:29706687-29710388	5.43974	27.7108	2.34884
Ciclev10018844m.g	scaffold_3:11065876-11069713	2.19188	11.1786	2.3505
Ciclev10019402m.g	scaffold_3:42184837-42186790	0.0886251	0.452157	2.35103
Ciclev10017693m.g	scaffold_2:9968925-9971998	0.0645396	0.329404	2.3516

Ciclev10008188m.g	scaffold_1:25502041-25504073	3.18611	16.2639	2.35181
Ciclev10020732m.g	scaffold_3:45554378-45555692	0.426053	2.18742	2.36013
-	scaffold_1:20664826-20823973	9.53869	49.0507	2.36241
Ciclev10024838m.g	scaffold_7:11850299-12160617	0.0610356	0.314116	2.36357
Ciclev10029791m.g	scaffold_8:6944685-7159404	0.156361	0.805566	2.36512
Ciclev10009784m.g	scaffold_1:27582593-27583241	3.38086	17.429	2.36603
Ciclev10016618m.g	scaffold_2:29572733-29574402	4.89429	25.2971	2.3698
Ciclev10027748m.g	scaffold_8:1409153-1412353	0.431268	2.23149	2.37135
Ciclev10024820m.g	scaffold_7:14328879-14331672	0.11501	0.596507	2.37478
Ciclev10008735m.g	scaffold_1:24559622-24563143	1.83436	9.52791	2.37688
Ciclev10007088m.g	scaffold_9:30058933-30061075	0.26083	1.35489	2.377
Ciclev10024606m.g	scaffold_3:28065477-28341901	0.0620191	0.322769	2.37972
Ciclev10027708m.g,Ciclev10027711m.g	scaffold_8:4205576-4222282	24.3883	127.111	2.38183
Ciclev10031687m.g	scaffold_4:14695805-14698040	0.299173	1.55935	2.38189
Ciclev10024831m.g	scaffold_7:11850299-12160617	0.0564142	0.295494	2.389
Ciclev10014472m.g	scaffold_2:11194588-11210308	3.87882	20.345	2.39099
Ciclev10020040m.g	scaffold_3:7367886-7369329	0.249093	1.3104	2.39525
Ciclev10009953m.g	scaffold_1:4716069-4716821	1.58342	8.37372	2.40283
Ciclev10008382m.g	scaffold_1:4642935-4652060	8.62249	45.6513	2.40448
Ciclev10019728m.g	scaffold_3:36113342-36118653	0.184492	0.979385	2.40832
Ciclev10006986m.g	scaffold_9:5156626-5157913	0.628602	3.34139	2.41023
Ciclev10029798m.g	scaffold_8:5756170-5759050	0.0937058	0.498422	2.41116
Ciclev10009598m.g	scaffold_1:26849194-26849981	0.284334	1.51951	2.41795
Ciclev10026825m.g	scaffold_7:944360-945110	28.8597	154.311	2.41872
Ciclev10005365m.g	scaffold_9:10357083-10359834	2.25938	12.1129	2.42254
-	scaffold_8:7698015-7862701	10.7629	57.8375	2.42594
Ciclev10023794m.g	scaffold_3:41974016-41979701	2.00296	10.7779	2.42787
Ciclev10011571m.g	scaffold_6:2753292-2755765	3.19593	17.2111	2.42903
Ciclev10017716m.g	scaffold_2:6629795-6632053	0.246381	1.33303	2.43575
Ciclev10010114m.g	scaffold_1:3049949-3056785	0.296677	1.60647	2.43693
Ciclev10016627m.g	scaffold_2:27132896-27133794	1.14766	6.22797	2.44007
-	scaffold_2:24401745-24433873	17.7184	96.3283	2.44271
Ciclev10014330m.g	scaffold_2:30951168-30955052	0.197895	1.07857	2.44631
Ciclev10019000m.g	scaffold_3:759536-763184	4.20141	22.9931	2.45226
Ciclev10001395m.g	scaffold_5:37913156-37914626	0.126071	0.690772	2.45397
Ciclev10000940m.g	scaffold_5:37180883-37182577	0.218367	1.19717	2.4548
Ciclev10003464m.g	scaffold_5:28289811-28536377	0.857384	4.70642	2.45662
Ciclev10018277m.g	scaffold_2:28519150-28520215	0.180858	0.994394	2.45896
Ciclev10000983m.g	scaffold_5:24066569-24070257	0.41947	2.31728	2.46579
Ciclev10014965m.g	scaffold_2:34097796-34103044	72.3019	399.83	2.46728
Ciclev10001849m.g	scaffold_5:39325975-39327488	2.34927	13.0174	2.47015
Ciclev10003814m.g	scaffold_5:40001655-40005938	1.69802	9.41389	2.47094
Ciclev10025956m.g	scaffold_7:9884001-9885389	2.68663	14.9262	2.47397
Ciclev10003176m.g	scaffold_5:31798469-31803028	0.114058	0.638372	2.48463
-	scaffold_5:9440503-9446061	1.20501	6.7606	2.48811
Ciclev10017805m.g,Ciclev10018319m.g	scaffold_2:6554459-6563699	0.299223	1.6819	2.4908
Ciclev10025278m.g	scaffold_7:3100604-3104483	0.275341	1.55419	2.49687
Ciclev10014322m.g	scaffold_2:3643632-3646749	0.715283	4.04592	2.49988
Ciclev10010498m.g	scaffold_1:2624782-2630038	0.070351	0.398792	2.50299
Ciclev10023313m.g	scaffold_3:33935883-33939540	0.297555	1.69698	2.51174
-	scaffold_8:22049748-22050018	13.382	76.6059	2.51716
Ciclev10019997m.g	scaffold_3:41067674-41229006	0.748499	4.29363	2.52013
Ciclev10013665m.g	scaffold_6:11288007-11289897	0.168011	0.970924	2.53081
Ciclev10013480m.g	scaffold_6:20158646-20161144	0.24254	1.40196	2.53115

Ciclev10029021m.g	scaffold_8:23982040-23983893	9.3741	54.401	2.53688
Ciclev10018512m.g	scaffold_3:49457050-49465862	1.65316	9.62533	2.54161
Ciclev10010106m.g	scaffold_1:28686869-28690073	0.707236	4.11865	2.54191
Ciclev10032548m.g	scaffold_4:24005220-24007211	10.8614	63.6319	2.55054
Ciclev10004779m.g,Ciclev10006533m.g,Ciclev10007139m.g	scaffold_9:26694563-26789030	1.00605	5.90089	2.55223
Ciclev10002205m.g	scaffold_5:35659719-35661331	0.631629	3.70826	2.55359
Ciclev10008017m.g	scaffold_1:27276795-27278532	0.677318	3.98509	2.55671
-	scaffold_9:21858292-21859376	2.15451	12.6931	2.55861
Ciclev10018556m.g	scaffold_3:38467971-38472111	0.0400384	0.236662	2.56337
Ciclev10008412m.g	scaffold_1:16431589-16436163	0.181185	1.07292	2.56601
Ciclev10019426m.g	scaffold_3:2348068-2350389	4.90986	29.0766	2.5661
Ciclev10004775m.g	scaffold_9:26790441-26802517	0.968166	5.74557	2.56912
Ciclev10013385m.g	scaffold_6:20629133-20630882	0.108324	0.645407	2.57486
Ciclev10027866m.g,Ciclev10027910m.g,Ciclev10030182m.g	scaffold_8:20620043-20723794	112.084	668.73	2.57684
Ciclev10015764m.g	scaffold_2:9149551-9151269	0.739131	4.43389	2.58467
Ciclev10004274m.g	scaffold_9:27060890-27063744	0.0682635	0.409691	2.58535
Ciclev10017705m.g	scaffold_2:5975366-6022331	9.39567	56.6441	2.59186
Ciclev10021499m.g	scaffold_3:44346025-44349632	0.248414	1.51666	2.61008
Ciclev10001768m.g	scaffold_5:29438710-29443724	0.961409	5.88778	2.6145
-	scaffold_6:23279219-23283029	0.649014	3.99617	2.6223
Ciclev10031309m.g	scaffold_4:2184380-2186045	0.119859	0.742299	2.63067
Ciclev10024092m.g	scaffold_3:24611866-24615456	0.153495	0.957209	2.64065
Ciclev10027711m.g	scaffold_8:4205576-4222282	2.63618	16.4549	2.642
-	scaffold_9:22498657-22499357	4.33958	27.2797	2.6522
-	scaffold_8:6944685-7159404	1.82217	11.4592	2.65278
Ciclev10009527m.g	scaffold_1:20529779-20531009	0.473347	2.98133	2.65499
Ciclev10000513m.g	scaffold_5:15186861-15191417	0.169186	1.07003	2.66097
Ciclev10032012m.g	scaffold_4:16046321-16048971	0.290099	1.83537	2.66145
Ciclev10016749m.g	scaffold_2:24290272-24291409	1.02284	6.49439	2.66661
Ciclev10018065m.g	scaffold_2:11532048-11575076	0.746135	4.76251	2.67421
Ciclev10027396m.g	scaffold_7:16939640-16973171	0.576845	3.68612	2.67585
Ciclev10000924m.g	scaffold_5:41680810-41682843	3.25434	20.834	2.6785
Ciclev10010401m.g	scaffold_1:1592274-1598844	3.00733	19.2801	2.68056
-	scaffold_3:29414134-29414449	1.62437	10.4338	2.68331
Ciclev10017001m.g,Ciclev10017259m.g	scaffold_2:24881666-24911470	1.9498	12.5537	2.68671
-	scaffold_5:6743977-6744326	16.1275	103.909	2.68772
-	scaffold_7:14622506-14624452	1.30317	8.44063	2.69532
Ciclev10011766m.g	scaffold_6:18517610-18519337	5.34814	34.6705	2.6966
Ciclev10018645m.g	scaffold_3:44050928-44054570	0.516032	3.34554	2.69671
Ciclev10014355m.g,Ciclev10018131m.g,Ciclev10018333m.g	scaffold_2:24737092-24836931	30.7383	199.613	2.69909
Ciclev10005248m.g	scaffold_9:30880571-30882582	0.346483	2.25656	2.70327
Ciclev10002409m.g	scaffold_5:27212522-27215391	0.632005	4.11706	2.70361
Ciclev10031126m.g	scaffold_4:22422506-22424799	0.421528	2.74603	2.70365
Ciclev10014324m.g	scaffold_2:5384913-5387562	0.188904	1.2315	2.7047
Ciclev10015727m.g	scaffold_2:32244183-32245928	188.386	1229.23	2.70599
Ciclev10016167m.g	scaffold_2:19844962-19847663	14.0328	91.6347	2.70709
Ciclev10015413m.g	scaffold_2:26097342-26099320	3.12436	20.4359	2.70948
Ciclev10004592m.g	scaffold_9:6418986-6422197	0.104215	0.682571	2.71141
Ciclev10015454m.g	scaffold_2:5172293-5175519	0.502564	3.29408	2.71249

Ciclev10028058m.g	scaffold_8:2358346-2360716	0.167102	1.09598	2.71342
Ciclev10014317m.g	scaffold_2:26817592-26820324	1.18782	7.79687	2.71458
Ciclev10030410m.g	scaffold_8:834671-837104	0.43104	2.83284	2.71636
Ciclev10021474m.g	scaffold_3:8111520-8113053	0.216195	1.42341	2.71894
Ciclev10030929m.g	scaffold_4:15087930-15266082	0.085187	0.561028	2.71937
Ciclev10008097m.g	scaffold_1:3683536-3688814	327.009	2157.93	2.72225
Ciclev10006745m.g	scaffold_9:7804322-8050719	15.6674	103.573	2.72481
Ciclev10018357m.g	scaffold_2:31183440-31185105	0.844587	5.59433	2.72765
Ciclev10000905m.g	scaffold_5:40878422-40882623	0.179887	1.19631	2.73343
Ciclev10011458m.g	scaffold_6:16868998-16870928	0.174688	1.16193	2.73367
Ciclev10010618m.g	scaffold_1:1574099-1575910	0.085305	0.569897	2.74
Ciclev10024882m.g	scaffold_7:9855687-9858922	0.310137	2.07379	2.74129
Ciclev10028084m.g	scaffold_8:16622616-16625543	7.35479	49.2215	2.74253
Ciclev10024080m.g	scaffold_3:35372249-35712253	0.0915834	0.613423	2.74372
Ciclev10018532m.g	scaffold_3:47067938-47074076	1.7508	11.7475	2.74626
Ciclev10032508m.g	scaffold_4:11827036-11828869	0.639199	4.29857	2.74952
Ciclev10005528m.g	scaffold_9:1787120-1791691	0.139796	0.943053	2.75402
Ciclev10019001m.g	scaffold_3:3795320-3799204	0.0715643	0.483002	2.75472
Ciclev10006843m.g	scaffold_9:24980605-24991190	0.0614723	0.415693	2.75751
-	scaffold_5:40658088-40658646	2.4274	16.532	2.76778
-	scaffold_3:28624578-28625799	0.994036	6.79765	2.77367
Ciclev10006907m.g	scaffold_9:3518380-3520907	0.38725	2.66032	2.78026
Ciclev10017839m.g	scaffold_2:24737092-24836931	12.4693	85.8328	2.78315
Ciclev10029033m.g	scaffold_8:23018434-23019836	0.701128	4.83174	2.78479
Ciclev10003974m.g	scaffold_5:31469286-31718393	0.10127	0.698121	2.78527
Ciclev10017839m.g	scaffold_2:24737092-24836931	1.11884	7.71369	2.78541
Ciclev10013175m.g	scaffold_6:23618757-23619373	4.66743	32.4449	2.79729
Ciclev10011647m.g	scaffold_6:15800586-15802381	9.08526	63.4538	2.80411
Ciclev10019547m.g	scaffold_3:4212100-4216950	0.757284	5.3037	2.80809
Ciclev10003224m.g	scaffold_5:32017860-32153975	1.25153	8.77358	2.80947
Ciclev10013731m.g	scaffold_6:355-1144	2.33099	16.3458	2.80991
Ciclev10015109m.g	scaffold_2:573823-592599	19.3721	135.996	2.81152
Ciclev10010884m.g	scaffold_497:5490-8108	0.716899	5.08215	2.8256
Ciclev10006671m.g	scaffold_9:5575905-5577948	8.23255	58.6955	2.83384
Ciclev10012535m.g	scaffold_6:20133102-20134805	19.7846	141.156	2.83484
Ciclev10019558m.g	scaffold_3:4012632-4015392	0.0973575	0.696551	2.83886
Ciclev10033582m.g	scaffold_4:4825832-5025734	0.12969	0.934806	2.8496
Ciclev10009513m.g	scaffold_1:1540620-1542895	10.4892	76.0533	2.85811
-	scaffold_9:6142910-6143360	0.709233	5.16575	2.86465
Ciclev10017830m.g	scaffold_2:28403936-28488203	0.31299	2.30674	2.88167
Ciclev10023081m.g	scaffold_3:7154153-7154697	0.601948	4.4394	2.88265
Ciclev10028146m.g	scaffold_8:19802919-19804545	0.229319	1.69574	2.88649
Ciclev10012941m.g	scaffold_6:20482587-20483257	3.98762	29.5463	2.88938
Ciclev10005244m.g	scaffold_9:14211430-14212939	0.581808	4.31298	2.89007
Ciclev10003762m.g	scaffold_5:25221155-25545745	0.120865	0.89819	2.89362
Ciclev10033549m.g	scaffold_4:24287300-24289538	0.238587	1.7851	2.90342
-	scaffold_5:10976025-10978963	1.03586	7.77813	2.90859
Ciclev10021085m.g	scaffold_3:3395546-3398414	2.85214	21.4512	2.91094
Ciclev10028001m.g	scaffold_8:167935-170159	0.0794473	0.598979	2.91443
Ciclev10011227m.g	scaffold_6:24959462-24962278	0.94432	7.22037	2.93473
Ciclev10005999m.g	scaffold_9:3836727-3837285	0.456956	3.49427	2.93487
Ciclev10001766m.g	scaffold_5:27492965-27539324	1.28332	9.83391	2.93789
Ciclev10027166m.g	scaffold_7:16709732-16712947	0.293293	2.26385	2.94837
Ciclev10018070m.g	scaffold_2:8954838-8978273	20.2615	156.625	2.9505
Ciclev10019573m.g	scaffold_3:1306957-1310158	0.221887	1.71548	2.95072
Ciclev10004089m.g	scaffold_5:24789819-24793279	0.418347	3.24378	2.95491

Ciclev10033971m.g	scaffold_4:18055306-18056467	0.196957	1.53064	2.95818
Ciclev10003094m.g	scaffold_5:35270039-35281019	0.656956	5.11307	2.96032
Ciclev10000115m.g	scaffold_5:31319624-31325108	0.130632	1.01694	2.96066
Ciclev10012852m.g	scaffold_6:24225829-24230019	1.45338	11.3943	2.97083
Ciclev10015111m.g	scaffold_2:9356314-9358708	0.280164	2.20417	2.97589
Ciclev10004321m.g	scaffold_9:10441486-10445440	0.0811243	0.638996	2.9776
Ciclev10030224m.g	scaffold_8:17291958-17467945	0.204221	1.61079	2.97957
Ciclev10027887m.g	scaffold_8:1263886-1268743	3.76947	29.7872	2.98226
-	scaffold_3:43181283-43182285	0.211101	1.67905	2.99164
Ciclev10017179m.g	scaffold_2:31864222-31865040	2.30326	18.4457	3.00153
Ciclev10029934m.g	scaffold_8:22377174-22400703	1.412	11.3224	3.00337
Ciclev10010811m.g	scaffold_1:21111204-21136102	3.33613	26.7557	3.0036
Ciclev10021602m.g	scaffold_3:36791206-36793775	0.639248	5.12953	3.00438
Ciclev10031572m.g	scaffold_4:21963362-21964776	1.41926	11.5182	3.0207
Ciclev10032742m.g	scaffold_4:22400757-22414668	0.477881	3.88241	3.02223
Ciclev10022412m.g	scaffold_3:44341478-44342385	0.476816	3.88585	3.02672
Ciclev10033599m.g	scaffold_4:9835605-9843707	2.31427	18.8771	3.02801
Ciclev10007550m.g	scaffold_1:23701398-23704524	4.50029	36.7851	3.03103
Ciclev10015005m.g	scaffold_2:10424778-10472483	4.40138	36.0996	3.03595
-	scaffold_2:25553292-25553676	0.984166	8.23965	3.06561
-	scaffold_2:32887396-32887790	0.929886	7.84874	3.07734
Ciclev10030197m.g	scaffold_8:7659484-7659841	0.937537	7.93125	3.0806
Ciclev10004407m.g	scaffold_9:30374696-30381795	0.0731126	0.619546	3.08302
Ciclev10018328m.g	scaffold_2:7273711-7336406	0.0707281	0.601246	3.0876
Ciclev10005491m.g	scaffold_9:27174469-27175800	10.8579	92.3294	3.08804
Ciclev10028245m.g	scaffold_8:19259622-19261508	0.337159	2.88386	3.0965
Ciclev10012167m.g	scaffold_6:19521790-19525103	87.579	756.392	3.11048
Ciclev10014927m.g	scaffold_2:31792652-31794402	0.100314	0.869381	3.11546
Ciclev10025445m.g,Ciclev10027567m.g	scaffold_7:19334465-19388718	6.30837	54.7748	3.11818
-	scaffold_3:39493254-39493694	0.740732	6.44088	3.12023
Ciclev10008047m.g	scaffold_1:23265680-23304211	4.31956	37.9775	3.13619
Ciclev10005754m.g	scaffold_9:20283397-20284497	0.330899	2.92593	3.14443
Ciclev10031895m.g	scaffold_4:13825957-13829506	0.132846	1.17845	3.14906
Ciclev10017142m.g	scaffold_2:30105993-30107427	0.152549	1.35984	3.15609
Ciclev10013506m.g	scaffold_6:8742663-8744139	0.465472	4.21784	3.17974
Ciclev10025756m.g	scaffold_7:12956764-12962208	0.138227	1.26033	3.18869
Ciclev10014309m.g	scaffold_2:24034061-24286273	0.588393	5.36879	3.18975
Ciclev10024879m.g	scaffold_7:7999675-8005144	0.0635186	0.583405	3.19925
Ciclev10003681m.g	scaffold_5:39090511-39094403	2.7032	24.8545	3.20076
Ciclev10020341m.g	scaffold_3:40274195-40275794	0.342397	3.15867	3.20558
Ciclev10006871m.g	scaffold_9:26694563-26789030	0.128233	1.18306	3.20569
Ciclev10019799m.g	scaffold_3:376011-378538	1.11546	10.2954	3.20629
Ciclev10015972m.g	scaffold_2:32376116-32377371	0.639355	5.92154	3.21128
Ciclev10031639m.g	scaffold_4:17329472-17351349	241.021	2244.87	3.2194
Ciclev10022806m.g	scaffold_3:1485615-1487011	6.98073	65.0325	3.21971
Ciclev10027855m.g	scaffold_8:24935282-24939307	2.80416	26.1931	3.22355
Ciclev10020624m.g	scaffold_3:33687571-33691590	0.716856	6.75008	3.23515
Ciclev10003745m.g	scaffold_5:15998433-16007240	11.3224	106.812	3.23782
Ciclev10025318m.g	scaffold_7:14519635-14527139	1.86566	17.6119	3.23879
-	scaffold_1:21232579-21232929	1.22512	11.5675	3.23909
Ciclev10001690m.g	scaffold_5:5208801-5210344	0.235629	2.23249	3.24407
Ciclev10024701m.g	scaffold_7:8465191-8474194	1.06807	10.1319	3.24583
Ciclev10017736m.g	scaffold_2:13379667-13380066	2.63319	25.0803	3.25167
Ciclev10012769m.g	scaffold_6:2988031-2988830	0.299132	2.85535	3.25481
Ciclev10025485m.g	scaffold_7:7653183-7656303	0.129213	1.2345	3.2561

Ciclev10025686m.g	scaffold_7:17272551-17304761	3.39278	32.7833	3.27242
Ciclev10003442m.g	scaffold_5:25221155-25545745	1.0943	10.5769	3.27284
Ciclev10000379m.g	scaffold_5:38887374-38894064	0.40633	3.94251	3.27839
-	scaffold_5:2498660-2498830	72.7284	707.515	3.28217
Ciclev10018257m.g	scaffold_2:10799358-10800072	0.355038	3.4564	3.28323
Ciclev10023549m.g	scaffold_3:3745746-3747441	1.51389	15.046	3.31305
Ciclev10011529m.g	scaffold_6:20175618-20180643	0.111628	1.10946	3.31309
-	scaffold_7:11818803-11819074	2.64059	26.2909	3.31563
Ciclev10032400m.g	scaffold_4:2252412-2254829	1.18441	11.7972	3.31621
Ciclev10014185m.g	scaffold_2:23948692-23951464	2.97032	29.6519	3.31943
Ciclev10005466m.g	scaffold_9:12119032-12122259	0.781908	7.80719	3.31973
Ciclev10010284m.g	scaffold_1:14828560-14829793	0.163414	1.63353	3.32139
-	scaffold_9:26497761-26498104	1.28933	12.9337	3.32644
Ciclev10009046m.g	scaffold_1:215151-217266	3.53562	35.6333	3.33319
Ciclev10012583m.g	scaffold_6:16936931-16938169	2.12442	21.5306	3.34125
Ciclev10033178m.g	scaffold_4:23892713-23896882	0.322949	3.28404	3.3461
Ciclev10004511m.g	scaffold_9:3534735-3538902	0.232808	2.37023	3.34781
Ciclev10032523m.g	scaffold_4:16324347-16328584	1.25776	12.8638	3.35438
Ciclev10003620m.g	scaffold_5:41115973-41117293	7.49863	76.8632	3.35759
Ciclev10031130m.g	scaffold_4:3949137-3952715	2.95228	30.6541	3.37618
Ciclev10011766m.g	scaffold_6:18517610-18519337	1.37712	14.62	3.40822
-	scaffold_3:16380349-16380963	1.44645	15.3867	3.4111
Ciclev10003140m.g	scaffold_5:9452591-9454877	0.433178	4.64804	3.42359
Ciclev10005843m.g	scaffold_9:7804322-8050719	0.796016	8.57889	3.42992
Ciclev10002398m.g	scaffold_5:38579085-38579793	0.965985	10.4291	3.43247
-	scaffold_8:7224264-7224412	259.996	2810.8	3.43442
Ciclev10025675m.g	scaffold_7:17264385-17266778	0.634672	6.88142	3.43863
Ciclev10021176m.g	scaffold_3:42427840-42430486	0.194146	2.13037	3.45589
-	scaffold_1:20664826-20823973	1.15796	12.7409	3.45981
Ciclev10021398m.g	scaffold_3:12814730-12817148	0.835234	9.23195	3.46638
-	scaffold_9:22497646-22498529	0.746364	8.25302	3.46697
Ciclev10017667m.g	scaffold_2:32451103-32454677	0.205712	2.27955	3.47005
-	scaffold_2:4895612-4986690	1.48542	16.6346	3.48524
Ciclev10007424m.g	scaffold_1:678920-682015	0.0672261	0.754397	3.48823
Ciclev10001630m.g	scaffold_5:36280827-36282328	0.144934	1.63194	3.49312
Ciclev10028239m.g	scaffold_8:9911761-9913703	2.80621	31.8012	3.50238
Ciclev10006610m.g	scaffold_9:26694563-26789030	0.153155	1.74883	3.51333
Ciclev10001106m.g	scaffold_5:42634963-42636368	0.598218	6.83725	3.51467
Ciclev10017757m.g	scaffold_2:24034061-24286273	0.114595	1.32498	3.53135
Ciclev10001622m.g,Ciclev10001626m.g	scaffold_5:29339627-29387910	0.174215	2.01692	3.53322
Ciclev10027207m.g	scaffold_7:3847851-3849850	0.0939375	1.08944	3.53574
Ciclev10030322m.g	scaffold_8:24888285-24893511	0.236378	2.74541	3.53785
Ciclev10018241m.g	scaffold_2:22799087-22800053	0.265534	3.13616	3.56203
Ciclev10012177m.g	scaffold_6:19512162-19517094	4.59277	54.3178	3.56399
Ciclev10027224m.g	scaffold_7:10270961-10279203	0.0397101	0.471881	3.57084
Ciclev10018531m.g	scaffold_3:25248366-25288105	0.0431096	0.515377	3.57955
Ciclev10017249m.g	scaffold_2:7507436-7569782	0.788133	9.43911	3.58214
Ciclev10006096m.g	scaffold_9:1285174-1286468	65.547	789.011	3.58945
Ciclev10032785m.g	scaffold_4:24169844-24170997	1.03809	12.5139	3.59153
Ciclev10014011m.g	scaffold_2:31518238-31526707	0.0275657	0.333541	3.59692
Ciclev10018230m.g	scaffold_2:34859929-34862413	0.236794	2.87574	3.60223
Ciclev10028769m.g	scaffold_8:10767029-10768985	2.51887	30.7725	3.61079
Ciclev10027269m.g	scaffold_7:19527523-19532809	0.614393	7.52538	3.61453
Ciclev10024858m.g	scaffold_7:11027711-11030551	0.167439	2.06293	3.62299
Ciclev10018938m.g	scaffold_3:45506313-45510965	0.144798	1.80097	3.63666

Ciclev10005988m.g	scaffold_9:29017293-29019361	1.14614	14.261	3.63722
-	scaffold_5:10980607-10981274	1.10417	13.9369	3.65787
Ciclev10013433m.g	scaffold_6:20476534-20477032	0.48583	6.17888	3.66882
-	scaffold_2:12648680-12651246	1.16566	14.8265	3.66897
Ciclev10000389m.g	scaffold_5:41126162-41131196	1.19499	15.7879	3.72375
Ciclev10028125m.g	scaffold_8:1145386-1148464	6.46975	85.5684	3.7253
Ciclev10016767m.g	scaffold_2:30266211-30266814	0.413417	5.50723	3.73566
Ciclev10032696m.g	scaffold_4:17356449-17357705	0.693018	9.24791	3.73816
Ciclev10005202m.g	scaffold_9:5167162-5168993	0.506763	6.85034	3.75679
Ciclev10020821m.g	scaffold_3:39972301-39974329	0.160767	2.18834	3.76679
Ciclev10007245m.g	scaffold_1:23236311-23242984	1.14577	15.7284	3.77899
Ciclev10013232m.g	scaffold_6:21667073-21673152	0.0463164	0.646912	3.80398
Ciclev10020637m.g	scaffold_3:5456335-5459361	0.112426	1.57462	3.80796
Ciclev10022126m.g	scaffold_3:49794489-49795281	0.27274	3.87215	3.82754
Ciclev10023988m.g	scaffold_3:10629682-10637896	0.561101	7.97218	3.82864
Ciclev10012511m.g,Ciclev10012621m.g	scaffold_6:12204778-12219666	0.78743	11.2252	3.83344
Ciclev10033760m.g	scaffold_4:24379347-24383934	0.0829752	1.18295	3.83356
Ciclev10005496m.g	scaffold_9:28305830-28333082	0.166352	2.37353	3.83472
Ciclev10015474m.g	scaffold_2:15302833-15304872	0.574093	8.23811	3.84296
Ciclev10013188m.g	scaffold_6:22970177-22976234	14.448	208.164	3.84878
Ciclev10004107m.g	scaffold_27:31122-32537	0.200449	2.93252	3.87084
Ciclev10012048m.g	scaffold_6:19313878-19315276	1.15075	17.1744	3.8996
Ciclev10011407m.g	scaffold_6:21306579-21310209	6.39858	95.8347	3.90473
Ciclev10007266m.g	scaffold_1:23223352-23230532	2.6139	39.3057	3.91046
Ciclev10002716m.g	scaffold_5:34768271-34769014	0.231501	3.59434	3.95664
Ciclev10017651m.g	scaffold_2:6491656-6492880	0.205956	3.26246	3.98556
Ciclev10026154m.g	scaffold_7:8161015-8168598	16.5581	264.236	3.99621
-	scaffold_9:3255152-3256251	0.1879	3.00199	3.99789
Ciclev10024057m.g	scaffold_3:11248388-11275176	0.951642	15.212	3.99864
Ciclev10011865m.g	scaffold_6:18194883-18202571	0.485354	7.84182	4.01408
Ciclev10033551m.g	scaffold_4:24509367-24511768	0.575168	9.30185	4.01546
Ciclev10015631m.g,Ciclev10017958m.g	scaffold_2:24034061-24286273	0.88584	14.35	4.01786
-	scaffold_3:25382388-25382891	0.578525	9.47656	4.03391
Ciclev10025012m.g,Ciclev10025047m.g	scaffold_7:10035359-10069948	0.816949	13.457	4.04197
Ciclev10027247m.g	scaffold_7:2179391-2181208	0.249642	4.14551	4.05362
Ciclev10007062m.g	scaffold_9:24682631-24756976	0.228936	3.84396	4.06958
Ciclev10019989m.g	scaffold_3:38041462-38044515	0.364941	6.22504	4.09235
Ciclev10025396m.g	scaffold_7:7189177-7192059	0.0813742	1.40042	4.10514
Ciclev10010467m.g	scaffold_1:21169449-21172416	0.376284	6.51571	4.11403
Ciclev10025042m.g	scaffold_7:13125146-13127602	0.214299	3.72385	4.1191
Ciclev10003759m.g	scaffold_5:24926144-25131690	0.481392	8.43009	4.13026
Ciclev10026359m.g	scaffold_7:18617580-18635802	12.4555	219.874	4.14182
Ciclev10001070m.g	scaffold_5:40109326-40112318	0.124748	2.27503	4.1888
Ciclev10004600m.g	scaffold_9:6434054-6439050	0.0954221	1.75859	4.20395
Ciclev10020376m.g	scaffold_3:47266707-47270591	7.59895	141.575	4.21962
Ciclev10026831m.g	scaffold_7:8915395-8916282	0.294233	5.53869	4.23451
Ciclev10033882m.g	scaffold_4:14508272-14510330	0.0848397	1.60032	4.23748
-	scaffold_7:18743277-18800003	0.222427	4.23697	4.25163
Ciclev10026452m.g	scaffold_7:17090620-17091988	0.161015	3.09618	4.26523
Ciclev10004022m.g	scaffold_5:25221155-25545745	0.0609366	1.17333	4.26715
Ciclev10029146m.g	scaffold_8:24820205-24822089	0.202348	4.01549	4.31067
Ciclev10025159m.g	scaffold_7:10714821-10718598	0.147257	2.99017	4.34381
Ciclev10014016m.g	scaffold_2:24018371-24032229	0.0362772	0.74665	4.36329

Ciclev10032889m.g	scaffold_4:2927385-2928219	0.232431	4.94685	4.41164
Ciclev10014113m.g	scaffold_2:7273711-7336406	0.143565	3.05586	4.4118
Ciclev10000444m.g	scaffold_5:27554715-27559349	1.58168	33.93	4.42303
Ciclev10012053m.g	scaffold_6:23296731-23297981	0.349401	7.49863	4.42367
Ciclev10010811m.g	scaffold_1:21111204-21136102	2.4546	54.9036	4.48334
Ciclev10025271m.g	scaffold_7:16549515-16552404	0.0703574	1.59831	4.5057
Ciclev10015798m.g,Ciclev10015799m.g	scaffold_2:573823-592599	25.1399	572.42	4.50902
Ciclev10025102m.g	scaffold_7:14999480-15001757	1.05009	24.0831	4.51944
Ciclev10027296m.g	scaffold_7:16857633-16859201	0.162439	3.82266	4.55661
Ciclev10007341m.g	scaffold_1:21279833-21282818	0.215908	5.2591	4.60633
Ciclev10025462m.g	scaffold_7:2341528-2343403	0.097815	2.39108	4.61146
Ciclev10014209m.g	scaffold_2:5582926-5587253	0.128299	3.20124	4.64105
Ciclev10004174m.g	scaffold_9:21936963-21944427	0.0435089	1.11267	4.67657
Ciclev10021130m.g	scaffold_3:21672632-21673939	0.438901	11.2263	4.67685
Ciclev10019767m.g	scaffold_3:1745121-1746932	0.125829	3.3191	4.72125
Ciclev10004649m.g,Ciclev10006792m.g	scaffold_9:1031276-1045594	0.754147	20.0783	4.73465
Ciclev10006106m.g	scaffold_9:1271893-1273092	0.638706	17.427	4.77003
Ciclev10005497m.g	scaffold_9:6297330-6299023	0.875093	24.1671	4.78747
-	scaffold_6:8740699-8741849	0.243572	6.80938	4.8051
-	scaffold_3:10992603-10994041	0.135755	3.94249	4.86003
Ciclev10025893m.g	scaffold_7:7783570-7785474	0.166584	4.90432	4.87973
Ciclev10018112m.g	scaffold_2:7273711-7336406	0.0784147	2.35613	4.90915
Ciclev10024511m.g	scaffold_3:22043475-22177017	0.0561316	1.69775	4.91866
Ciclev10014006m.g	scaffold_2:9700437-9713691	0.0872053	2.67798	4.94058
Ciclev10011377m.g	scaffold_6:15301641-15304785	0.307161	9.70934	4.98231
Ciclev10020761m.g,Ciclev10024037m.g	scaffold_3:31211928-31302670	0.873347	28.6902	5.03786
Ciclev10029528m.g	scaffold_8:17586990-17587899	2.52329	83.1697	5.04268
Ciclev10029889m.g	scaffold_8:5792721-5794233	0.302885	10.0595	5.05364
Ciclev10005649m.g	scaffold_9:23409096-23411093	0.263302	8.9613	5.08892
-	scaffold_5:38520688-38522154	2.97201	103.04	5.11563
Ciclev10017645m.g	scaffold_2:24034061-24286273	0.687726	23.8458	5.11576
Ciclev10011232m.g	scaffold_6:23027636-23031603	0.0863196	3.05368	5.14472
Ciclev10014810m.g	scaffold_2:31153656-31155460	0.101602	3.60658	5.14963
Ciclev10026359m.g,Ciclev10026361m.g	scaffold_7:18617580-18635802	1.46888	52.6602	5.16393
Ciclev10023337m.g	scaffold_3:49595413-49597863	0.224245	8.05805	5.16728
Ciclev10008874m.g	scaffold_1:20995351-21034862	0.587547	22.1624	5.23727
Ciclev10026415m.g	scaffold_7:4975418-4981695	0.466622	17.8398	5.2567
Ciclev10020782m.g,Ciclev10020818m.g	scaffold_3:47506791-47523094	0.420452	16.2667	5.27383
Ciclev10028401m.g	scaffold_8:20910212-20913273	0.140642	5.64619	5.32717
Ciclev10023421m.g	scaffold_3:22223358-22228209	0.0472111	1.91573	5.34262
Ciclev10006108m.g	scaffold_9:2458792-2459573	0.365922	15.6846	5.42166
Ciclev10014838m.g	scaffold_2:31051732-31053468	0.114295	5.06558	5.4699
Ciclev10016595m.g	scaffold_2:29886355-29887239	0.252776	11.4672	5.50351
Ciclev10028122m.g	scaffold_8:6535911-6540574	1.32505	62.9597	5.57031
Ciclev10006884m.g	scaffold_9:26084767-26085938	0.196769	9.48302	5.59077
Ciclev10015109m.g	scaffold_2:573823-592599	7.83436	424.628	5.76024
Ciclev10000810m.g,Ciclev10004013m.g	scaffold_5:5137961-5163853	0.475151	25.9133	5.76916
Ciclev10025130m.g	scaffold_7:10082660-10086068	0.0978137	5.36477	5.77734
Ciclev10022111m.g,Ciclev10022113m.g	scaffold_3:40504072-40719738	0.788651	45.8217	5.8605

Ciclev10024887m.g,Ciclev10024927m.g	scaffold_7:9055027-9085218	0.0754697	4.65886	5.94794
Ciclev10025259m.g,Ciclev10027399m.g	scaffold_7:6548429-6564074	0.45937	29.0927	5.98486
Ciclev10030225m.g	scaffold_8:9025006-9167585	0.127576	8.38565	6.0385
Ciclev10024363m.g	scaffold_3:43538600-43544145	0.00482319	0.32575	6.07764
Ciclev10025453m.g	scaffold_7:5947441-5950189	0.112171	7.73849	6.10828
Ciclev10001398m.g	scaffold_5:9330187-9333235	0.524827	39.8002	6.24479
Ciclev10015814m.g	scaffold_2:9404427-9406140	0.438398	33.3727	6.25028
Ciclev10014972m.g	scaffold_2:33319563-33323869	2.00971	161.604	6.32933
Ciclev10012789m.g	scaffold_6:18551898-18553017	0.318695	25.8934	6.34426
Ciclev10006713m.g	scaffold_9:1198746-1199583	7.8045	658.551	6.39885
Ciclev10018343m.g	scaffold_2:503874-505085	0.543646	58.2208	6.74272
Ciclev10006656m.g	scaffold_9:26117969-26159077	0.543473	58.6833	6.7546
Ciclev10005342m.g	scaffold_9:26117969-26159077	0.127091	16.0364	6.97934
Ciclev10029431m.g	scaffold_8:1491361-1556980	0.29931	80.12	8.06438
Ciclev10012590m.g	scaffold_6:5538880-5540740	0.396303	143.64	8.50163
Ciclev10007167m.g	scaffold_9:8509471-8512375	0.000849474	2.0644	11.2469
Ciclev10008843m.g	scaffold_1:26404749-26409802	0.000602871	6.69755	13.4395
Ciclev10008503m.g	scaffold_1:1311999-1316002	0	29.3755	inf
Ciclev10010610m.g	scaffold_1:2046474-2046988	0	4.08333	inf
Ciclev10010291m.g	scaffold_1:2207951-2208442	0	1.55624	inf
Ciclev10010426m.g	scaffold_1:2492451-2494280	0	0.507824	inf
Ciclev10010127m.g	scaffold_1:3598738-3598973	0	8.81295	inf
Ciclev10010069m.g	scaffold_1:4662424-4666008	0	0.557485	inf
Ciclev10010829m.g	scaffold_1:7953496-7954235	0	5.94662	inf
Ciclev10010057m.g	scaffold_1:12622639-12634394	0	0.304341	inf
Ciclev10010529m.g	scaffold_1:14207112-14208238	0	0.304375	inf
Ciclev10010041m.g	scaffold_1:14857472-14858266	0	0.274602	inf
Ciclev10010717m.g	scaffold_1:16766047-17038798	0	20.4725	inf
Ciclev10009308m.g	scaffold_1:17412138-17416007	0	0.122604	inf
Ciclev10010317m.g	scaffold_1:18168396-18169352	0	1.18361	inf
Ciclev10010205m.g	scaffold_1:18515122-18518456	0	0.0661022	inf
Ciclev10009473m.g	scaffold_1:18972163-18973223	0	0.529743	inf
Ciclev10007471m.g	scaffold_1:19189251-19192844	0	0.178488	inf
Ciclev10010688m.g	scaffold_1:19481290-19483482	0	0.269721	inf
Ciclev10007509m.g	scaffold_1:19496563-19588226	0	0.859397	inf
Ciclev10010187m.g	scaffold_1:19656356-19659025	0	0.220502	inf
Ciclev10010375m.g	scaffold_1:19721667-19778009	0	1.32094	inf
Ciclev10007674m.g	scaffold_1:19721667-19778009	0	0.201966	inf
Ciclev10007547m.g	scaffold_1:19905420-19995040	0	0.371585	inf
-	scaffold_1:20664826-20823973	0	24.4095	inf
Ciclev10008319m.g	scaffold_1:20664826-20823973	0	47.9486	inf
Ciclev10010122m.g	scaffold_1:20664826-20823973	0	1.96086	inf
Ciclev10010225m.g	scaffold_1:21146780-21147788	0	0.449078	inf
Ciclev10010405m.g	scaffold_1:21239885-21242798	0	0.0749175	inf
Ciclev10009212m.g	scaffold_1:21975140-22013742	0	0.240881	inf
Ciclev10008338m.g	scaffold_1:22175413-22384190	0	0.339449	inf
Ciclev10008872m.g	scaffold_1:22517142-22522527	0	1.77578	inf
Ciclev10008745m.g	scaffold_1:23930594-23932397	0	0.696878	inf
Ciclev10010142m.g	scaffold_1:25708274-25710232	0	0.345649	inf
Ciclev10008809m.g	scaffold_1:26018427-26020452	0	1.48717	inf
Ciclev10010229m.g	scaffold_1:26173957-26174442	0	1.12038	inf
Ciclev10010849m.g	scaffold_1:26729765-26744426	0	0.487925	inf
Ciclev10010480m.g	scaffold_1:26853733-26854240	0	0.399094	inf
Ciclev10010130m.g	scaffold_1:27286386-27290085	0	0.292626	inf

Ciclev10009891m.g	scaffold_1:27291801-27292599	0	0.263813	inf
Ciclev10010200m.g	scaffold_1:28383277-28384146	0	0.390795	inf
Ciclev10010726m.g	scaffold_1:28646428-28649662	0	0.958407	inf
Ciclev10010769m.g	scaffold_1:28651776-28654413	0	0.3917	inf
Ciclev10007957m.g	scaffold_1:28683840-28686491	0	0.092524	inf
Ciclev10010164m.g	scaffold_1:33638-33911	0	1.53312	inf
Ciclev10007370m.g	scaffold_1:628390-633381	0	0.270283	inf
Ciclev10010696m.g	scaffold_1:642699-647057	0	0.16753	inf
Ciclev10010797m.g	scaffold_1:2074308-2076320	0	0.0882404	inf
Ciclev10010709m.g	scaffold_1:2237003-2346942	0	0.237799	inf
Ciclev10010301m.g	scaffold_1:2237003-2346942	0	2.86653	inf
Ciclev10010537m.g	scaffold_1:2378354-2380519	0	5.14747	inf
Ciclev10010745m.g	scaffold_1:3921707-3938807	0	0.158007	inf
Ciclev10010334m.g	scaffold_1:3979841-3983332	0	0.424235	inf
Ciclev10009984m.g	scaffold_1:4764990-4766132	0	1.23208	inf
Ciclev10010763m.g	scaffold_1:6587351-6587637	0	79.4493	inf
Ciclev10007269m.g	scaffold_1:7679273-7685541	0	0.320405	inf
Ciclev10010846m.g	scaffold_1:14993045-14997677	0	0.275215	inf
Ciclev10010123m.g	scaffold_1:15207670-15209252	0	0.302134	inf
Ciclev10010683m.g	scaffold_1:18564666-18565092	0	3.64408	inf
Ciclev10010246m.g	scaffold_1:19305268-19305619	0	1.33053	inf
Ciclev10008012m.g	scaffold_1:20102611-20107488	0	0.0829417	inf
Ciclev10010032m.g	scaffold_1:20462859-20465557	0	1.07942	inf
Ciclev10010362m.g	scaffold_1:20935699-20938217	0	0.167928	inf
Ciclev10009940m.g	scaffold_1:21078113-21080074	0	1.44813	inf
Ciclev10010191m.g	scaffold_1:21726151-21727553	0	0.213905	inf
Ciclev10010171m.g	scaffold_1:21749467-21751617	0	0.759334	inf
Ciclev10010803m.g	scaffold_1:21770959-21773617	0	0.228101	inf
Ciclev10007917m.g	scaffold_1:22651298-22653980	0	0.0980159	inf
Ciclev10008798m.g	scaffold_1:22831846-22836046	0	1.13563	inf
Ciclev10008001m.g	scaffold_1:22963841-22967722	0	0.247178	inf
Ciclev10007280m.g	scaffold_1:23026293-23032032	0	0.137861	inf
Ciclev10010218m.g	scaffold_1:23055163-23057117	0	0.080592	inf
Ciclev10010386m.g	scaffold_1:23246042-23251931	0	0.199197	inf
Ciclev10010103m.g	scaffold_1:24349192-24351132	0	1.18007	inf
Ciclev10010564m.g	scaffold_1:24485304-24486952	0	0.369866	inf
Ciclev10010773m.g	scaffold_1:25119112-25122528	0	0.278799	inf
Ciclev10008785m.g	scaffold_1:26531856-26535022	0	0.372637	inf
Ciclev10010067m.g	scaffold_1:26752333-26756663	0	0.446435	inf
Ciclev10010002m.g	scaffold_1:27141516-27145011	0	0.732177	inf
Ciclev10008669m.g	scaffold_1:28474032-28489192	0	7.69335	inf
Ciclev10007588m.g	scaffold_1:28690241-28695756	0	0.111454	inf
-	scaffold_1:5172903-5173125	0	50.1683	inf
-	scaffold_1:9007492-9081493	0	9.92353	inf
-	scaffold_1:9099082-9099381	0	35.538	inf
-	scaffold_1:16766047-17038798	0	6.97787	inf
Ciclev10013937m.g	scaffold_10:181133-182805	0	0.896214	inf
-	scaffold_1726:1110-1368	0	52.1059	inf
Ciclev10017525m.g	scaffold_2:110586-111324	0	0.26172	inf
Ciclev10016753m.g	scaffold_2:417980-419662	0	2.5217	inf
Ciclev10015715m.g	scaffold_2:493064-494510	0	6.60756	inf
Ciclev10018098m.g	scaffold_2:4600839-4602648	0	0.111673	inf
Ciclev10014967m.g	scaffold_2:4641049-4643067	0	0.788502	inf
Ciclev10017810m.g	scaffold_2:5273993-5337902	0	2.14552	inf
Ciclev10017210m.g	scaffold_2:5808061-5809175	0	0.581015	inf
Ciclev10018279m.g	scaffold_2:5963579-5967232	0	0.982405	inf

Ciclev10015295m.g	scaffold_2:6579636-6581607	0	0.232468	inf
Ciclev10017600m.g	scaffold_2:6662287-6663655	0	0.875295	inf
Ciclev10014854m.g	scaffold_2:8049815-8052326	0	0.0841906	inf
Ciclev10018107m.g	scaffold_2:8063338-8064162	0	1.37044	inf
Ciclev10018041m.g	scaffold_2:8716340-8719451	0	0.262045	inf
Ciclev10017531m.g	scaffold_2:13341556-13343067	0	1.14488	inf
Ciclev10017246m.g	scaffold_2:13984363-13985994	0	0.328384	inf
Ciclev10014441m.g	scaffold_2:14104993-14108307	0	0.145044	inf
Ciclev10017486m.g	scaffold_2:19888951-19892367	0	0.165696	inf
Ciclev10017668m.g	scaffold_2:20109705-20110967	0	0.164193	inf
Ciclev10014992m.g	scaffold_2:20149859-20151451	0	1.00582	inf
Ciclev10018206m.g	scaffold_2:21634858-21635854	0	0.208196	inf
Ciclev10017428m.g	scaffold_2:23030623-23032740	0	0.127589	inf
Ciclev10017753m.g	scaffold_2:24034061-24286273	0	1.21191	inf
Ciclev10014792m.g	scaffold_2:24034061-24286273	0	0.110236	inf
Ciclev10017904m.g	scaffold_2:25422296-25492273	0	0.54743	inf
Ciclev10017682m.g	scaffold_2:25422296-25492273	0	0.105001	inf
Ciclev10017279m.g	scaffold_2:25553966-25555256	0	1.07545	inf
Ciclev10017951m.g	scaffold_2:26105694-26158658	0	0.364284	inf
Ciclev10015346m.g	scaffold_2:26838905-26845798	0	0.130124	inf
Ciclev10018132m.g	scaffold_2:27393144-27417687	0	0.196087	inf
Ciclev10018299m.g	scaffold_2:27447573-27450204	0	0.442327	inf
Ciclev10017870m.g	scaffold_2:28540755-28541247	0	0.595727	inf
Ciclev10016426m.g	scaffold_2:28960362-28961103	0	1.26509	inf
Ciclev10017514m.g	scaffold_2:30180423-30183207	0	0.0672072	inf
Ciclev10018217m.g	scaffold_2:30201437-30205192	0	0.0774213	inf
Ciclev10018073m.g	scaffold_2:31111140-31112748	0	1.60358	inf
Ciclev10017251m.g	scaffold_2:31181989-31183274	0	1.36427	inf
-	scaffold_2:32348947-32350092	0	4.57674	inf
Ciclev10014789m.g	scaffold_2:32401637-32405896	0	0.229691	inf
Ciclev10017679m.g	scaffold_2:32651990-32653016	0	0.538546	inf
Ciclev10018064m.g	scaffold_2:32662539-32664330	0	0.191517	inf
Ciclev10017813m.g	scaffold_2:33629173-33629692	0	1.09857	inf
Ciclev10018148m.g	scaffold_2:34440678-34441543	0	0.794508	inf
Ciclev10017416m.g	scaffold_2:34513877-34514103	0	3.01674	inf
Ciclev10017750m.g	scaffold_2:35000957-35005649	0	0.0626295	inf
Ciclev10017385m.g	scaffold_2:35075675-35078521	0	0.325133	inf
Ciclev10016605m.g	scaffold_2:35301652-35302927	0	0.240286	inf
Ciclev10017316m.g	scaffold_2:101800-103654	0	0.327237	inf
Ciclev10015700m.g	scaffold_2:528287-529556	0	0.40772	inf
Ciclev10017451m.g	scaffold_2:2993717-2995287	0	0.567274	inf
Ciclev10018300m.g	scaffold_2:4683615-4685255	0	0.402528	inf
Ciclev10018076m.g	scaffold_2:4694828-4695857	0	0.302654	inf
Ciclev10018265m.g	scaffold_2:4727638-4728025	0	0.818185	inf
Ciclev10014212m.g	scaffold_2:5454555-5460438	0	0.052385	inf
Ciclev10017577m.g	scaffold_2:5738229-5742051	0	0.140445	inf
Ciclev10014410m.g	scaffold_2:5763117-5765380	0	0.88324	inf
Ciclev10015029m.g	scaffold_2:5827671-5829186	0	4.06526	inf
Ciclev10015045m.g	scaffold_2:5831894-5833396	0	0.229849	inf
Ciclev10017612m.g	scaffold_2:5845644-5878657	0	1.45014	inf
Ciclev10017546m.g	scaffold_2:5845644-5878657	0	0.129702	inf
Ciclev10018270m.g	scaffold_2:5886837-5887530	0	0.845332	inf
Ciclev10014228m.g	scaffold_2:7507436-7569782	0	0.396243	inf
Ciclev10018289m.g	scaffold_2:8055065-8056876	0	0.0960987	inf
Ciclev10018360m.g	scaffold_2:8553963-8554914	0	0.17908	inf
Ciclev10015024m.g	scaffold_2:10328906-10331724	0	4.84812	inf

Ciclev10015630m.g	scaffold_2:10646178-10649019	0	59.0317	inf
Ciclev10015835m.g	scaffold_2:10761349-10762893	0	0.142767	inf
Ciclev10017601m.g	scaffold_2:11174745-11177034	0	0.16966	inf
Ciclev10018247m.g	scaffold_2:11617675-11618766	0	0.562317	inf
Ciclev10017437m.g	scaffold_2:12586850-12593702	0	14.9392	inf
Ciclev10015302m.g	scaffold_2:13105781-13108297	0	1.3113	inf
Ciclev10016043m.g	scaffold_2:13432224-13433850	0	1.21296	inf
Ciclev10017561m.g	scaffold_2:13692700-13695522	0	0.143201	inf
Ciclev10018005m.g	scaffold_2:16407830-16408493	0	0.505871	inf
Ciclev10017327m.g	scaffold_2:20001711-20002253	0	0.96678	inf
Ciclev10014278m.g	scaffold_2:20362298-20365054	0	0.526469	inf
Ciclev10017568m.g	scaffold_2:21376835-21377632	0	10.2588	inf
Ciclev10018322m.g	scaffold_2:23708181-23711287	0	0.216663	inf
Ciclev10017897m.g	scaffold_2:23947459-23948367	0	1.80264	inf
Ciclev10017625m.g	scaffold_2:24004820-24006291	0	0.744792	inf
Ciclev10017274m.g	scaffold_2:25940789-25941473	0	3.05571	inf
Ciclev10017055m.g	scaffold_2:27638162-27639131	0	0.219142	inf
Ciclev10016683m.g	scaffold_2:27929983-27932386	0	6.42873	inf
Ciclev10015022m.g	scaffold_2:28158700-28161820	0	1.12612	inf
Ciclev10017418m.g	scaffold_2:29078014-29078281	0	2.11589	inf
Ciclev10015747m.g	scaffold_2:29089914-29091171	0	0.13709	inf
Ciclev10018222m.g	scaffold_2:29238939-29244737	0	0.157532	inf
Ciclev10018399m.g	scaffold_2:30561080-30566547	0	2.36977	inf
Ciclev10018201m.g	scaffold_2:30736179-30773700	0	0.651195	inf
Ciclev10017768m.g	scaffold_2:31039163-31040678	0	0.0963061	inf
Ciclev10018353m.g	scaffold_2:31055616-31057248	0	0.281732	inf
Ciclev10017782m.g	scaffold_2:31115015-31116647	0	0.0937296	inf
Ciclev10014802m.g	scaffold_2:31163236-31165409	0	7.23538	inf
Ciclev10014803m.g	scaffold_2:31190072-31191728	0	0.0996446	inf
Ciclev10018259m.g	scaffold_2:31511401-31515740	0	0.0553904	inf
Ciclev10017572m.g	scaffold_2:31987477-31988278	0	0.264367	inf
Ciclev10017801m.g	scaffold_2:32147047-32152345	0	0.0938807	inf
Ciclev10017560m.g	scaffold_2:32223938-32228274	0	0.0776508	inf
Ciclev10017282m.g	scaffold_2:32987483-32989092	0	0.906574	inf
Ciclev10014693m.g	scaffold_2:33167653-33169593	0	8.48128	inf
Ciclev10015562m.g	scaffold_2:33871400-33873599	0	0.119102	inf
Ciclev10018204m.g	scaffold_2:33952242-33956586	0	0.0586604	inf
Ciclev10018236m.g	scaffold_2:34095437-34096507	0	0.143755	inf
Ciclev10016384m.g	scaffold_2:34346969-34350783	0	0.426994	inf
Ciclev10016680m.g	scaffold_2:34378906-34380424	0	0.794147	inf
Ciclev10017841m.g	scaffold_2:34449294-34452089	0	0.0891154	inf
Ciclev10014561m.g	scaffold_2:34678885-34684185	0	0.0721546	inf
Ciclev10015767m.g	scaffold_2:34702791-34704898	0	0.129202	inf
Ciclev10024010m.g	scaffold_3:309488-310142	0	0.300641	inf
Ciclev10019909m.g	scaffold_3:402942-405487	0	0.120203	inf
Ciclev10023100m.g	scaffold_3:572549-576969	0	1.30716	inf
Ciclev10024616m.g	scaffold_3:1456045-1459905	0	0.135665	inf
Ciclev10022789m.g	scaffold_3:2623913-2624424	0	0.84534	inf
Ciclev10024108m.g	scaffold_3:3935596-3936364	0	0.46785	inf
Ciclev10024278m.g	scaffold_3:4154232-4156752	0	0.12764	inf
Ciclev10022894m.g	scaffold_3:4751692-4752419	0	4.256	inf
Ciclev10022946m.g	scaffold_3:4756514-4757253	0	0.346222	inf
Ciclev10019315m.g	scaffold_3:5508278-5513535	0	0.38722	inf
Ciclev10024284m.g	scaffold_3:6310629-6312250	0	0.124906	inf
Ciclev10019758m.g	scaffold_3:6863325-6864905	0	0.357442	inf
Ciclev10019047m.g	scaffold_3:6911058-6914961	0	0.0652395	inf

Ciclev10022100m.g	scaffold_3:7080221-7081373	0	45.4084	inf
Ciclev10024049m.g	scaffold_3:7123159-7123759	0	1.21587	inf
Ciclev10023301m.g	scaffold_3:7272722-7274213	0	0.29415	inf
Ciclev10023186m.g	scaffold_3:7311581-7314699	0	0.254787	inf
Ciclev10024165m.g	scaffold_3:7658092-7661444	0	0.88456	inf
Ciclev10023416m.g	scaffold_3:8222443-8223685	0	0.177534	inf
Ciclev10024207m.g	scaffold_3:8492624-8493753	0	0.228463	inf
Ciclev10022419m.g	scaffold_3:9746535-9747499	0	0.29869	inf
Ciclev10023578m.g	scaffold_3:10015825-10018162	0	0.189497	inf
Ciclev10023709m.g	scaffold_3:10931878-10932265	0	0.606046	inf
Ciclev10019768m.g	scaffold_3:11687448-12154880	0	0.129578	inf
Ciclev10019295m.g	scaffold_3:11687448-12154880	0	0.0990817	inf
Ciclev10024171m.g	scaffold_3:16611167-16614029	0	0.0694581	inf
Ciclev10024468m.g	scaffold_3:16789903-16796624	0	0.149003	inf
Ciclev10023892m.g	scaffold_3:17498056-17501627	0	0.238299	inf
Ciclev10023148m.g	scaffold_3:17722316-17728192	0	0.250945	inf
Ciclev10024537m.g	scaffold_3:19310539-19608202	0	0.193352	inf
Ciclev10018973m.g	scaffold_3:21296577-21473295	0	0.183373	inf
Ciclev10024161m.g	scaffold_3:21296577-21473295	0	0.0810318	inf
Ciclev10024358m.g	scaffold_3:21814051-21815034	0	0.212753	inf
Ciclev10020805m.g	scaffold_3:22987881-22989816	0	0.145803	inf
Ciclev10023823m.g	scaffold_3:23292161-23293113	0	0.898671	inf
Ciclev10024380m.g	scaffold_3:23497988-23501483	0	0.0973841	inf
Ciclev10023600m.g	scaffold_3:23539224-23539470	0	4.27434	inf
Ciclev10023827m.g	scaffold_3:23793865-23798309	0	0.134136	inf
Ciclev10024424m.g	scaffold_3:23838887-23843652	0	0.0620762	inf
Ciclev10022364m.g	scaffold_3:23844421-23845662	0	0.254182	inf
Ciclev10024483m.g	scaffold_3:24026647-24169357	0	0.229793	inf
Ciclev10023502m.g	scaffold_3:24200821-24205153	0	0.124621	inf
Ciclev10024522m.g	scaffold_3:24352153-24448329	0	0.23761	inf
Ciclev10024540m.g	scaffold_3:24531215-24534832	0	0.0687581	inf
Ciclev10018689m.g	scaffold_3:24546483-24550815	0	0.0550494	inf
Ciclev10024119m.g	scaffold_3:25216525-25220629	0	0.0349432	inf
Ciclev10018594m.g	scaffold_3:25318177-25322601	0	0.137501	inf
Ciclev10018499m.g	scaffold_3:25361088-25365339	0	0.175928	inf
Ciclev10023662m.g	scaffold_3:27453995-27456865	0	0.124547	inf
Ciclev10024240m.g	scaffold_3:27512874-27514002	0	1.8437	inf
Ciclev10024208m.g	scaffold_3:27579559-27591874	0	0.730628	inf
Ciclev10023474m.g	scaffold_3:27610924-27611502	0	2.34341	inf
Ciclev10021536m.g	scaffold_3:27624873-27626398	0	1.79525	inf
Ciclev10023899m.g	scaffold_3:27717304-27722483	0	1.01294	inf
Ciclev10018816m.g	scaffold_3:27726234-27797748	0	0.836862	inf
Ciclev10018798m.g	scaffold_3:27726234-27797748	0	1.02939	inf
Ciclev10023350m.g	scaffold_3:28065477-28341901	0	0.486404	inf
Ciclev10024480m.g	scaffold_3:28585537-28590076	0	0.0900622	inf
-	scaffold_3:28622728-28624051	0	3.61266	inf
Ciclev10019874m.g	scaffold_3:29017623-29019269	0	0.18602	inf
Ciclev10023790m.g	scaffold_3:29250144-29251716	0	1.7587	inf
Ciclev10023422m.g	scaffold_3:30508198-30509068	0	0.191264	inf
Ciclev10024500m.g	scaffold_3:30930869-30932508	0	0.231131	inf
Ciclev10022009m.g	scaffold_3:31119096-31120465	0	0.926528	inf
Ciclev10024032m.g	scaffold_3:32208346-32211347	0	2.22014	inf
Ciclev10019454m.g	scaffold_3:32711814-32715376	0	0.0993707	inf
Ciclev10020204m.g	scaffold_3:33017443-33018751	0	0.120445	inf
Ciclev10021050m.g	scaffold_3:33252462-33253730	0	0.141303	inf
Ciclev10024230m.g	scaffold_3:33267937-33268945	0	0.183268	inf

Ciclev10021981m.g	scaffold_3:33388539-33592383	0	0.269785	inf
Ciclev10024360m.g	scaffold_3:33388539-33592383	0	0.359264	inf
Ciclev10023094m.g	scaffold_3:33687571-33691590	0	0.262201	inf
Ciclev10021711m.g	scaffold_3:33779593-33781481	0	0.224795	inf
Ciclev10024395m.g	scaffold_3:33810210-33895316	0	0.101249	inf
Ciclev10023285m.g	scaffold_3:35139148-35139886	0	0.401422	inf
Ciclev10023175m.g	scaffold_3:36286796-36287881	0	0.414735	inf
Ciclev10024205m.g	scaffold_3:36750919-36754775	0	0.133373	inf
Ciclev10019871m.g	scaffold_3:37424654-37426942	0	0.0952498	inf
Ciclev10019845m.g	scaffold_3:37453219-37455335	0	0.779629	inf
Ciclev10024652m.g	scaffold_3:37556380-37557620	0	0.792313	inf
Ciclev10024187m.g	scaffold_3:37759070-37759334	0	1.22738	inf
Ciclev10023804m.g	scaffold_3:38587757-38589577	0	0.142843	inf
Ciclev10023061m.g	scaffold_3:39631321-39637711	0	0.666203	inf
Ciclev10021888m.g	scaffold_3:39884338-39886161	0	0.499897	inf
Ciclev10022118m.g	scaffold_3:40728336-40729280	0	2.94559	inf
Ciclev10023683m.g	scaffold_3:40824773-40826388	0	0.118874	inf
Ciclev10023966m.g	scaffold_3:40844360-40846159	0	0.467851	inf
Ciclev10021457m.g	scaffold_3:42073315-42076287	0	0.167547	inf
Ciclev10023074m.g	scaffold_3:43350018-43353840	0	1.5717	inf
Ciclev10023546m.g	scaffold_3:43489731-43491123	0	0.360294	inf
Ciclev10019274m.g	scaffold_3:43837475-43842610	0	0.148429	inf
Ciclev10021400m.g	scaffold_3:45233398-45241100	0	7.17955	inf
Ciclev10021291m.g	scaffold_3:45584832-45586407	0	0.658264	inf
Ciclev10023798m.g	scaffold_3:45939190-45939709	0	0.379905	inf
Ciclev10019602m.g	scaffold_3:46004399-46007704	0	0.0701556	inf
Ciclev10023809m.g	scaffold_3:47361319-47363515	0	0.166651	inf
Ciclev10019970m.g	scaffold_3:48669636-48672194	0	0.317173	inf
Ciclev10019773m.g	scaffold_3:49898590-49901084	0	0.840971	inf
Ciclev10022525m.g	scaffold_3:50635794-50636621	0	0.341732	inf
Ciclev10023199m.g	scaffold_3:296343-301419	0	1.71977	inf
Ciclev10022654m.g	scaffold_3:488829-489773	0	0.42361	inf
Ciclev10023484m.g	scaffold_3:1377074-1378343	0	0.252305	inf
Ciclev10024061m.g	scaffold_3:1452875-1454058	0	0.1503	inf
Ciclev10019383m.g	scaffold_3:2226804-2230144	0	2.45474	inf
Ciclev10023958m.g	scaffold_3:2555697-2556529	0	2.46536	inf
Ciclev10019363m.g	scaffold_3:2986786-2988745	0	0.0804794	inf
Ciclev10019797m.g	scaffold_3:3701452-3703185	0	0.542211	inf
Ciclev10019477m.g	scaffold_3:4782614-4784946	0	0.913501	inf
Ciclev10019159m.g	scaffold_3:5230511-5232888	0	0.0576936	inf
Ciclev10024157m.g	scaffold_3:5637095-5639060	0	0.249801	inf
Ciclev10022839m.g	scaffold_3:5823246-5823833	0	0.369959	inf
Ciclev10019148m.g	scaffold_3:6968895-6978361	0	0.133295	inf
Ciclev10022533m.g	scaffold_3:7050992-7053101	0	0.372718	inf
Ciclev10019991m.g	scaffold_3:7901179-7902867	0	1.62562	inf
Ciclev10024657m.g	scaffold_3:9026019-9036386	0	0.555099	inf
Ciclev10024246m.g	scaffold_3:9281218-9282526	0	0.134172	inf
Ciclev10019854m.g	scaffold_3:11185438-11190180	0	0.371196	inf
-	scaffold_3:11687448-12154880	0	5.05869	inf
Ciclev10020975m.g	scaffold_3:12583893-12587229	0	0.085088	inf
Ciclev10023517m.g	scaffold_3:12834748-12836191	0	2.12486	inf
Ciclev10023080m.g	scaffold_3:13024245-13033708	0	0.353576	inf
Ciclev10018613m.g	scaffold_3:13258123-13262266	0	0.0527287	inf
Ciclev10023932m.g	scaffold_3:14068209-14068589	0	4.38229	inf
Ciclev10024344m.g	scaffold_3:14354102-14355585	0	0.121021	inf
Ciclev10024190m.g	scaffold_3:16871835-16874343	0	0.190468	inf

Ciclev10022284m.g	scaffold_3:18283646-18284477	0	0.198928	inf
Ciclev10022124m.g	scaffold_3:18472966-18487087	0	0.129404	inf
Ciclev10021204m.g	scaffold_3:18988839-19105549	0	0.982688	inf
Ciclev10023166m.g	scaffold_3:19305598-19309195	0	0.348396	inf
Ciclev10020873m.g	scaffold_3:20047473-20050335	0	0.345436	inf
Ciclev10020249m.g	scaffold_3:20577773-20598741	0	0.61097	inf
Ciclev10023345m.g	scaffold_3:20746896-20747388	0	0.342897	inf
Ciclev10018644m.g	scaffold_3:20782316-20786064	0	0.105597	inf
Ciclev10024428m.g	scaffold_3:21678085-21719797	0	0.141682	inf
Ciclev10024640m.g	scaffold_3:21878974-21892761	0	1.52483	inf
Ciclev10023438m.g	scaffold_3:21993009-21993945	0	1.21297	inf
Ciclev10024660m.g	scaffold_3:22043475-22177017	0	1.29719	inf
Ciclev10023905m.g	scaffold_3:22205299-22208758	0	0.0590422	inf
Ciclev10019367m.g	scaffold_3:22355290-22357723	0	2.00885	inf
Ciclev10023258m.g	scaffold_3:22782283-22786467	0	0.373799	inf
Ciclev10020564m.g	scaffold_3:23031239-23033606	0	0.176157	inf
Ciclev10020625m.g	scaffold_3:23232280-23233432	0	0.680801	inf
Ciclev10022647m.g	scaffold_3:23726935-23729328	0	0.484	inf
Ciclev10019166m.g	scaffold_3:25596183-25598211	0	0.0777315	inf
Ciclev10018637m.g	scaffold_3:25756519-25759773	0	0.178256	inf
Ciclev10021728m.g	scaffold_3:26847876-26850055	0	0.613485	inf
Ciclev10024456m.g	scaffold_3:28353792-28357632	0	0.229521	inf
Ciclev10021099m.g	scaffold_3:30697833-30699210	0	0.207342	inf
Ciclev10019789m.g	scaffold_3:32036503-32038936	0	18.0683	inf
Ciclev10020178m.g	scaffold_3:32478992-32480318	0	0.449534	inf
Ciclev10022462m.g	scaffold_3:32716415-32805364	0	0.61249	inf
Ciclev10023223m.g	scaffold_3:33154203-33159959	0	0.333511	inf
Ciclev10024125m.g	scaffold_3:33388539-33592383	0	0.15387	inf
Ciclev10018659m.g	scaffold_3:33388539-33592383	0	0.166405	inf
Ciclev10024426m.g	scaffold_3:33388539-33592383	0	0.0796883	inf
Ciclev10021200m.g	scaffold_3:33900596-33905302	0	1.37944	inf
Ciclev10024465m.g	scaffold_3:34585413-34589410	0	0.11965	inf
Ciclev10023519m.g	scaffold_3:34802791-34803289	0	1.01737	inf
Ciclev10022991m.g	scaffold_3:34803441-34804342	0	0.290998	inf
Ciclev10024659m.g	scaffold_3:35827349-35831778	0	0.333184	inf
Ciclev10018678m.g	scaffold_3:35906755-35910117	0	0.281101	inf
Ciclev10021705m.g	scaffold_3:36616813-36617700	0	1.04538	inf
Ciclev10018762m.g	scaffold_3:36685659-36688401	0	0.317919	inf
Ciclev10022063m.g	scaffold_3:36825381-36828004	0	2.08448	inf
Ciclev10022050m.g,Ciclev1<br>0023297m.g	scaffold_3:36833800-36848785	0	171.446	inf
Ciclev10024204m.g	scaffold_3:37062710-37063599	0	0.192596	inf
Ciclev10023811m.g	scaffold_3:39318711-39319464	0	1.09284	inf
Ciclev10019775m.g	scaffold_3:40023515-40025316	0	0.10141	inf
Ciclev10020765m.g	scaffold_3:40157408-40158807	0	0.660363	inf
Ciclev10024109m.g	scaffold_3:40504072-40719738	0	9.20745	inf
Ciclev10022117m.g	scaffold_3:40504072-40719738	0	5.50602	inf
Ciclev10023846m.g	scaffold_3:40504072-40719738	0	7.54229	inf
Ciclev10023210m.g	scaffold_3:41067674-41229006	0	1.41315	inf
Ciclev10021611m.g	scaffold_3:41448470-41449519	0	0.157873	inf
Ciclev10018560m.g	scaffold_3:43011640-43045787	0	0.212711	inf
Ciclev10024118m.g	scaffold_3:43503874-43504885	0	0.179897	inf
Ciclev10023996m.g	scaffold_3:43564318-43565586	0	0.334616	inf
Ciclev10018875m.g	scaffold_3:44463216-44469327	0	0.0590525	inf
Ciclev10019918m.g	scaffold_3:45021474-45024988	0	0.103848	inf
Ciclev10018752m.g	scaffold_3:45225060-45228493	0	0.0479207	inf

Ciclev10023774m.g	scaffold_3:45337919-45339263	0	0.278622	inf
Ciclev10023999m.g	scaffold_3:45367643-45367952	0	1.92264	inf
Ciclev10023180m.g	scaffold_3:45552688-45554358	0	3.31987	inf
Ciclev10024028m.g	scaffold_3:46175348-46175786	0	0.41218	inf
Ciclev10023756m.g	scaffold_3:46275682-46277314	0	0.18543	inf
Ciclev10024151m.g	scaffold_3:46519382-46595230	0	1.16749	inf
Ciclev10021203m.g,Ciclev10024034m.g	scaffold_3:46941296-46944629	0	3.65013	inf
Ciclev10023548m.g	scaffold_3:47247393-47248103	0	2.06337	inf
Ciclev10023282m.g	scaffold_3:47975844-47979072	0	0.0696857	inf
Ciclev10023512m.g	scaffold_3:48465732-48466641	0	0.445448	inf
Ciclev10024257m.g	scaffold_3:48618879-48620746	0	1.10355	inf
Ciclev10024569m.g	scaffold_3:49598243-49599935	0	0.925004	inf
Ciclev10021623m.g	scaffold_3:50119463-50120716	0	15.4847	inf
Ciclev10023644m.g	scaffold_3:50923229-50926895	0	0.384716	inf
-	scaffold_3:11687448-12154880	0	14.84	inf
-	scaffold_3:18788092-18788548	0	6.56741	inf
-	scaffold_3:35918733-35919993	0	2.68033	inf
-	scaffold_3:44640552-44640997	0	13.1616	inf
Ciclev10033472m.g	scaffold_4:58162-60065	0	0.112011	inf
Ciclev10030832m.g	scaffold_4:467597-484070	0	0.0826481	inf
Ciclev10032034m.g	scaffold_4:621326-623180	0	0.147275	inf
-	scaffold_4:941075-942315	0	2.86148	inf
Ciclev10033293m.g	scaffold_4:1089584-1093128	0	0.0531304	inf
Ciclev10033088m.g	scaffold_4:1856877-1857951	0	1.33583	inf
Ciclev10031099m.g	scaffold_4:2198030-2200212	0	0.591001	inf
Ciclev10031307m.g	scaffold_4:2212800-2214458	0	0.382401	inf
Ciclev10033384m.g	scaffold_4:2328695-2330790	0	0.12593	inf
Ciclev10033907m.g	scaffold_4:2631074-2631485	0	1.70572	inf
Ciclev10031673m.g	scaffold_4:2656758-2659632	0	0.240483	inf
Ciclev10033956m.g	scaffold_4:3727217-3728587	0	4.55028	inf
Ciclev10032782m.g	scaffold_4:3740850-3741757	0	0.536711	inf
Ciclev10033473m.g	scaffold_4:4825832-5025734	0	0.4857	inf
Ciclev10032473m.g	scaffold_4:5184143-5185255	0	9.19988	inf
Ciclev10033353m.g	scaffold_4:5188584-5191247	0	1.26865	inf
Ciclev10034001m.g	scaffold_4:5872491-5873343	0	0.554772	inf
Ciclev10033231m.g	scaffold_4:6264316-6268506	0	1.58163	inf
Ciclev10031865m.g	scaffold_4:6959320-6961933	0	0.0924807	inf
Ciclev10033495m.g	scaffold_4:8435878-8436794	0	0.444075	inf
Ciclev10033613m.g	scaffold_4:12254306-12255435	0	0.767244	inf
Ciclev10032216m.g	scaffold_4:14325994-14327398	0	0.174915	inf
Ciclev10031361m.g	scaffold_4:14758847-14763882	0	0.387257	inf
Ciclev10031115m.g	scaffold_4:15087930-15266082	0	0.083958	inf
Ciclev10033908m.g	scaffold_4:15087930-15266082	0	0.134389	inf
Ciclev10031749m.g	scaffold_4:15087930-15266082	0	0.103966	inf
Ciclev10033385m.g	scaffold_4:15616972-15619422	0	0.344925	inf
Ciclev10033786m.g	scaffold_4:15820848-15822766	0	0.203686	inf
Ciclev10033574m.g	scaffold_4:17487524-17491633	0	0.743215	inf
Ciclev10031213m.g	scaffold_4:17504552-17507814	0	1.06879	inf
Ciclev10033575m.g	scaffold_4:18189783-18190920	0	0.204295	inf
Ciclev10030817m.g	scaffold_4:18515567-18675155	0	0.516697	inf
Ciclev10032139m.g	scaffold_4:19320923-19322586	0	0.140471	inf
Ciclev10033073m.g	scaffold_4:19359941-19360737	0	1.74532	inf
Ciclev10033382m.g	scaffold_4:19744617-19746027	0	0.101048	inf
Ciclev10031511m.g	scaffold_4:21014798-21021050	0	0.301994	inf
Ciclev10031269m.g	scaffold_4:21374686-21376599	0	0.878943	inf

Ciclev10032558m.g	scaffold_4:21612918-21619733	0	0.503848	inf
Ciclev10033428m.g	scaffold_4:22638528-22641168	0	0.114297	inf
Ciclev10033917m.g	scaffold_4:22706532-22708389	0	0.166644	inf
Ciclev10033765m.g	scaffold_4:22944705-22960987	0	0.358275	inf
Ciclev10033827m.g	scaffold_4:23656910-23658886	0	0.159058	inf
Ciclev10033837m.g	scaffold_4:25160454-25162215	0	2.40027	inf
Ciclev10033074m.g	scaffold_4:1016949-1017384	0	0.630779	inf
Ciclev10032781m.g	scaffold_4:1591644-1639717	0	4.76603	inf
Ciclev10030707m.g	scaffold_4:1750456-1753272	0	0.119276	inf
Ciclev10033390m.g	scaffold_4:1933784-1934480	0	0.723041	inf
Ciclev10032922m.g	scaffold_4:2934167-2934674	0	2.0105	inf
Ciclev10033605m.g	scaffold_4:3028951-3031749	0	0.103054	inf
Ciclev10033820m.g	scaffold_4:3223637-3224743	0	0.275234	inf
Ciclev10031952m.g	scaffold_4:4448713-4454106	0	0.549561	inf
Ciclev10031951m.g	scaffold_4:4471371-4475002	0	0.110921	inf
Ciclev10030526m.g	scaffold_4:4825832-5025734	0	0.0599058	inf
Ciclev10033432m.g	scaffold_4:5026252-5027791	0	1.15983	inf
Ciclev10031953m.g	scaffold_4:6077108-6080729	0	2.21839	inf
Ciclev10032588m.g	scaffold_4:7532854-7706546	0	0.237919	inf
Ciclev10033301m.g	scaffold_4:9292549-9293110	0	0.348278	inf
Ciclev10033228m.g	scaffold_4:10715619-10721328	0	0.29293	inf
Ciclev10033687m.g	scaffold_4:12710835-12715174	0	0.364023	inf
Ciclev10033363m.g	scaffold_4:12864647-12865247	0	0.330324	inf
Ciclev10033379m.g	scaffold_4:12890964-12891661	0	0.623693	inf
Ciclev10030813m.g	scaffold_4:14162131-14167688	0	0.0490693	inf
Ciclev10033669m.g	scaffold_4:14920606-14922229	0	10.0185	inf
Ciclev10033811m.g	scaffold_4:14934142-14935765	0	0.0963738	inf
Ciclev10033825m.g	scaffold_4:15268401-15270837	0	0.752545	inf
Ciclev10031231m.g	scaffold_4:16364267-16367228	0	0.177353	inf
Ciclev10033957m.g	scaffold_4:16995148-17013320	0	0.15418	inf
Ciclev10033646m.g	scaffold_4:17358020-17359301	0	0.144615	inf
Ciclev10033851m.g	scaffold_4:17650705-17652253	0	0.0958314	inf
Ciclev10033588m.g	scaffold_4:17831479-17835258	0	0.152285	inf
Ciclev10033474m.g	scaffold_4:18062797-18063943	0	0.159864	inf
Ciclev10033220m.g	scaffold_4:18324119-18329144	0	0.680902	inf
Ciclev10033594m.g	scaffold_4:18515567-18675155	0	0.0549575	inf
Ciclev10033311m.g	scaffold_4:18515567-18675155	0	0.228656	inf
Ciclev10030620m.g	scaffold_4:18679709-18682834	0	0.0544882	inf
Ciclev10032404m.g	scaffold_4:19845900-19847050	0	0.343865	inf
Ciclev10033730m.g	scaffold_4:20384078-20385402	0	0.63101	inf
Ciclev10033770m.g	scaffold_4:20573682-20575539	0	0.449679	inf
Ciclev10033488m.g	scaffold_4:20664150-20666836	0	0.450894	inf
Ciclev10033875m.g	scaffold_4:22246775-22249816	0	0.730357	inf
Ciclev10032466m.g	scaffold_4:23101410-23102275	0	0.205768	inf
Ciclev10033641m.g	scaffold_4:24316997-24319189	0	0.103523	inf
Ciclev10033671m.g	scaffold_4:24321609-24321960	0	1.04638	inf
Ciclev10033510m.g	scaffold_4:24690193-24692350	0	0.270408	inf
Ciclev10033247m.g	scaffold_4:25044516-25047286	0	0.545939	inf
Ciclev10033269m.g	scaffold_4:25363527-25363898	0	6.09141	inf
Ciclev10033911m.g	scaffold_4:25485644-25491982	0	0.372607	inf
Ciclev10033386m.g	scaffold_4:25493770-25496117	0	0.229519	inf
-	scaffold_4:9717654-9718171	0	3.71373	inf
-	scaffold_4:10335034-10335274	0	29.8949	inf
-	scaffold_4:24511855-24512128	0	18.1658	inf
Ciclev10002464m.g	scaffold_5:254183-279075	0	2.30569	inf
-	scaffold_5:898790-899291	0	185.637	inf

Ciclev10000326m.g	scaffold_5:953144-1184934	0	1.66921	inf
Ciclev10001029m.g	scaffold_5:953144-1184934	0	1.25444	inf
Ciclev10000629m.g	scaffold_5:953144-1184934	0	1.57395	inf
Ciclev10000813m.g	scaffold_5:5198673-5200885	0	1.36395	inf
Ciclev10000817m.g	scaffold_5:5213078-5216672	0	11.2295	inf
Ciclev10003600m.g	scaffold_5:5837850-5838555	0	1.19346	inf
Ciclev10003892m.g	scaffold_5:6636139-6639173	0	0.323694	inf
Ciclev10003578m.g	scaffold_5:7053038-7056763	0	0.723476	inf
Ciclev10001661m.g	scaffold_5:7157124-7159270	0	0.632931	inf
Ciclev10003956m.g	scaffold_5:8033099-8034302	0	0.295227	inf
Ciclev10000814m.g	scaffold_5:8159352-8216590	0	2.75271	inf
Ciclev10000811m.g	scaffold_5:8241246-8243511	0	3.9697	inf
Ciclev10002141m.g	scaffold_5:8300864-8301824	0	6.21617	inf
-	scaffold_5:10985200-10985910	0	2.93446	inf
Ciclev10003470m.g	scaffold_5:11710509-11711116	0	0.381363	inf
Ciclev10003383m.g	scaffold_5:15038807-15039241	0	5.52434	inf
Ciclev10004039m.g	scaffold_5:17645976-17649124	0	0.0690351	inf
Ciclev10002267m.g	scaffold_5:19690722-19691804	0	0.246926	inf
Ciclev10000680m.g	scaffold_5:21729484-21732150	0	0.0748607	inf
Ciclev10003971m.g	scaffold_5:24035767-24039477	0	0.0679697	inf
Ciclev10003750m.g	scaffold_5:24757343-24759689	0	3.61941	inf
Ciclev10001723m.g	scaffold_5:25625107-25626537	0	0.104998	inf
Ciclev10000846m.g	scaffold_5:26691139-26693785	0	0.168141	inf
Ciclev10001118m.g	scaffold_5:26715484-26717938	0	0.100048	inf
Ciclev10003834m.g	scaffold_5:26881356-26882250	0	0.282404	inf
Ciclev10004096m.g	scaffold_5:28132337-28133194	0	1.7068	inf
Ciclev10001426m.g	scaffold_5:29072428-29075604	0	0.384411	inf
Ciclev10004033m.g	scaffold_5:29571389-29572233	0	3.64126	inf
Ciclev10003695m.g	scaffold_5:29574923-29576567	0	3.41552	inf
Ciclev10002774m.g	scaffold_5:29580437-29581464	0	0.717448	inf
Ciclev10003261m.g	scaffold_5:29602912-29636069	0	0.145511	inf
Ciclev10003276m.g	scaffold_5:29786844-29788591	0	0.111746	inf
Ciclev10003890m.g	scaffold_5:29847245-29848902	0	0.228445	inf
Ciclev10003029m.g	scaffold_5:31925317-31954322	0	4.46216	inf
Ciclev10000623m.g	scaffold_5:32186366-32189739	0	0.324321	inf
Ciclev10000055m.g	scaffold_5:32339882-32346597	0	0.0369125	inf
Ciclev10003865m.g	scaffold_5:32404311-32420675	0	0.0466387	inf
Ciclev10000586m.g	scaffold_5:33902777-33906708	0	0.373571	inf
Ciclev10002805m.g	scaffold_5:34796710-34797694	0	0.198521	inf
Ciclev10003248m.g	scaffold_5:34862069-34863869	0	0.120336	inf
Ciclev10003230m.g	scaffold_5:35250275-35255004	0	22.5829	inf
Ciclev10000958m.g	scaffold_5:36267512-36270615	0	1.39286	inf
Ciclev10003030m.g	scaffold_5:36814201-36816256	0	0.355894	inf
Ciclev10002054m.g	scaffold_5:36978026-36980013	0	0.125069	inf
Ciclev10002933m.g	scaffold_5:37413098-37417182	0	0.274881	inf
Ciclev10004034m.g	scaffold_5:37609413-37614258	0	0.123334	inf
Ciclev10003071m.g	scaffold_5:37989803-37990104	0	11.5286	inf
Ciclev10003122m.g	scaffold_5:38409325-38411705	0	0.188052	inf
-	scaffold_5:38527066-38528134	0	125.6	inf
Ciclev10003797m.g	scaffold_5:39584829-39589357	0	5.87223	inf
Ciclev10003536m.g	scaffold_5:40231255-40231474	0	7.98618	inf
Ciclev10003465m.g	scaffold_5:40710052-40712570	0	0.170699	inf
Ciclev10003726m.g	scaffold_5:41074572-41074842	0	4.134	inf
Ciclev10004091m.g	scaffold_5:42257731-42258386	0	1.74288	inf
Ciclev10003773m.g	scaffold_5:953144-1184934	0	0.0586285	inf
Ciclev10004078m.g	scaffold_5:953144-1184934	0	2.12184	inf

Ciclev10003963m.g	scaffold_5:4383936-4385684	0	1.76424	inf
Ciclev10003938m.g	scaffold_5:4572471-4574898	0	0.0759423	inf
Ciclev10003390m.g	scaffold_5:5041215-5044644	0	0.0782344	inf
Ciclev10002584m.g	scaffold_5:5984332-5986046	0	0.961266	inf
Ciclev10000976m.g	scaffold_5:6070992-6076799	0	0.0874506	inf
Ciclev10002462m.g	scaffold_5:7350488-7351678	0	3.65091	inf
Ciclev10002084m.g	scaffold_5:7366267-7368258	0	0.147801	inf
Ciclev10002201m.g	scaffold_5:8359633-8360521	0	0.528636	inf
Ciclev10002259m.g	scaffold_5:8409346-8410432	0	0.457217	inf
Ciclev10001863m.g	scaffold_5:8562147-8563362	0	0.895343	inf
Ciclev10003300m.g	scaffold_5:9053339-9055168	0	0.324372	inf
Ciclev10002503m.g	scaffold_5:11129408-11130029	0	0.333298	inf
Ciclev10003910m.g	scaffold_5:11878818-11880132	0	0.61608	inf
Ciclev10001089m.g	scaffold_5:11931811-11933520	0	0.568888	inf
Ciclev10001158m.g	scaffold_5:12075746-12077161	0	0.217358	inf
Ciclev10002112m.g	scaffold_5:13886545-13915539	0	0.192313	inf
Ciclev10002423m.g	scaffold_5:15468104-15469451	0	0.181518	inf
Ciclev10003076m.g	scaffold_5:17021241-17021864	0	0.509341	inf
Ciclev10003200m.g	scaffold_5:19865022-20070734	0	0.149443	inf
Ciclev10003339m.g	scaffold_5:21093707-21094469	0	0.208633	inf
Ciclev10003270m.g	scaffold_5:21776469-21776889	0	1.14669	inf
Ciclev10003533m.g	scaffold_5:23602337-23605476	0	0.0519635	inf
Ciclev10003899m.g	scaffold_5:24926144-25131690	0	0.0514283	inf
Ciclev10003155m.g	scaffold_5:25221155-25545745	0	0.119359	inf
Ciclev10003416m.g	scaffold_5:25587441-25590126	0	0.455193	inf
Ciclev10004054m.g	scaffold_5:25670067-25673881	0	0.0923518	inf
Ciclev10000844m.g	scaffold_5:26217705-26220288	0	0.0995571	inf
Ciclev10003799m.g	scaffold_5:27191609-27192009	0	2.07732	inf
Ciclev10001923m.g	scaffold_5:27349476-27355235	0	0.532855	inf
Ciclev10002921m.g	scaffold_5:28138392-28140825	0	0.374817	inf
Ciclev10003563m.g	scaffold_5:30180306-30184569	0	0.393569	inf
Ciclev10003541m.g	scaffold_5:30235957-30239503	0	0.117744	inf
Ciclev10002963m.g	scaffold_5:30579601-30580104	0	0.504598	inf
Ciclev10003433m.g	scaffold_5:30766135-30766510	0	2.02404	inf
Ciclev10001299m.g	scaffold_5:30901446-30903844	0	0.226551	inf
Ciclev10002052m.g	scaffold_5:31469286-31718393	0	0.452423	inf
Ciclev10000297m.g	scaffold_5:32733964-32736806	0	0.34499	inf
Ciclev10003498m.g	scaffold_5:33070453-33071464	0	0.568354	inf
Ciclev10003793m.g	scaffold_5:33079707-33163140	0	0.523854	inf
Ciclev10003948m.g	scaffold_5:33168125-33169160	0	0.576874	inf
Ciclev10001013m.g	scaffold_5:33480565-33482350	0	0.818951	inf
Ciclev10003596m.g	scaffold_5:33630055-33631345	0	0.483863	inf
Ciclev10003721m.g	scaffold_5:36424591-36425059	0	5.07597	inf
Ciclev10003964m.g	scaffold_5:36715465-36718694	0	0.188328	inf
Ciclev10001085m.g	scaffold_5:36770515-36774625	0	1.35319	inf
Ciclev10003741m.g	scaffold_5:37125075-37126689	0	0.153672	inf
Ciclev10003127m.g	scaffold_5:37161363-37162615	0	0.183714	inf
Ciclev10000954m.g	scaffold_5:37749886-37752104	0	0.201562	inf
Ciclev10001443m.g	scaffold_5:38345360-38347483	0	0.101795	inf
Ciclev10003213m.g	scaffold_5:39512542-39513982	0	0.244661	inf
Ciclev10003236m.g	scaffold_5:39581934-39582345	0	1.64014	inf
Ciclev10003691m.g	scaffold_5:39610055-39610534	0	2.3325	inf
Ciclev10003019m.g	scaffold_5:40081126-40086638	0	0.539602	inf
Ciclev10003565m.g	scaffold_5:40469988-40471086	0	0.767487	inf
-	scaffold_5:4076819-4284428	0	1.50264	inf
-	scaffold_5:7558178-7558349	0	146.825	inf

-	scaffold_5:8252984-8253440	0	5.55704	inf
-	scaffold_5:8350832-8351507	0	6.15536	inf
-	scaffold_5:10986496-10986897	0	9.4954	inf
-	scaffold_5:12833334-12833523	0	87.1529	inf
-	scaffold_5:24449525-24449723	0	55.1387	inf
-	scaffold_5:26484065-26484670	0	3.91608	inf
-	scaffold_5:28539341-28801354	0	2.33548	inf
-	scaffold_5:28539341-28801354	0	3.63199	inf
-	scaffold_5:28539341-28801354	0	21.2821	inf
-	scaffold_5:32546088-32620214	0	3.79699	inf
-	scaffold_5:32546088-32620214	0	14.9796	inf
-	scaffold_5:33079707-33163140	0	11.6078	inf
-	scaffold_5:33079707-33163140	0	7.09152	inf
-	scaffold_5:33676229-33676847	0	238.369	inf
Ciclev10030461m.g	scaffold_511:553-1126	0	0.381169	inf
Ciclev10030465m.g	scaffold_56:26603-31803	0	0.166457	inf
Ciclev10012629m.g	scaffold_6:10164-14058	0	1.2673	inf
Ciclev10010949m.g	scaffold_6:1148650-1152274	0	0.0420135	inf
Ciclev10013355m.g	scaffold_6:1186850-1191699	0	0.0448281	inf
Ciclev10013684m.g	scaffold_6:1409754-1410018	0	1.50811	inf
Ciclev10013801m.g	scaffold_6:1422775-1423039	0	5.7153	inf
Ciclev10013874m.g	scaffold_6:3376740-3377613	0	0.76407	inf
Ciclev10013848m.g	scaffold_6:5257344-5257725	0	1.17381	inf
Ciclev10013744m.g	scaffold_6:5525101-5529479	0	0.235074	inf
Ciclev10013446m.g	scaffold_6:10154859-10157031	0	0.202832	inf
Ciclev10013278m.g	scaffold_6:12883198-12883555	0	2.03825	inf
Ciclev10013858m.g	scaffold_6:14809566-14811313	0	0.15378	inf
Ciclev10011384m.g	scaffold_6:15311564-15314620	0	1.07073	inf
Ciclev10013588m.g	scaffold_6:15678836-15684730	0	0.0534949	inf
Ciclev10013688m.g	scaffold_6:16004466-16005081	0	0.313468	inf
Ciclev10010962m.g	scaffold_6:16760473-16763602	0	0.164182	inf
Ciclev10012298m.g	scaffold_6:17166406-17167643	0	0.134776	inf
Ciclev10012959m.g	scaffold_6:17179866-17180480	0	0.227845	inf
Ciclev10011350m.g	scaffold_6:17421408-17423458	0	0.154347	inf
Ciclev10013414m.g	scaffold_6:19013419-19014408	0	0.638283	inf
Ciclev10013531m.g	scaffold_6:20266784-20267804	0	0.145391	inf
Ciclev10013431m.g	scaffold_6:20535343-20539657	0	0.070429	inf
Ciclev10013512m.g	scaffold_6:21314187-21317092	0	0.0552613	inf
Ciclev10012099m.g	scaffold_6:21892604-21893891	0	8.25107	inf
Ciclev10013082m.g	scaffold_6:22176948-22177972	0	0.822078	inf
Ciclev10013386m.g	scaffold_6:22670045-22672321	0	0.0935987	inf
Ciclev10013623m.g	scaffold_6:22762831-22763343	0	0.713142	inf
Ciclev10013443m.g	scaffold_6:22994964-22995723	0	0.255441	inf
Ciclev10013755m.g	scaffold_6:23040221-23050285	0	0.578888	inf
Ciclev10013240m.g	scaffold_6:23115094-23119251	0	1.08645	inf
Ciclev10013823m.g	scaffold_6:23796190-23796731	0	1.29285	inf
Ciclev10013180m.g	scaffold_6:24219056-24219753	0	0.396869	inf
Ciclev10013747m.g	scaffold_6:24336575-24337137	0	1.38296	inf
Ciclev10013839m.g	scaffold_6:24649126-24651539	0	0.121898	inf
Ciclev10011501m.g	scaffold_6:24662453-24665352	0	0.166555	inf
Ciclev10013448m.g	scaffold_6:24673772-24677434	0	18.3668	inf
Ciclev10011828m.g	scaffold_6:24954704-24956405	0	0.920474	inf
Ciclev10013783m.g	scaffold_6:24972596-24974590	0	0.437312	inf
Ciclev10013457m.g	scaffold_6:25167732-25170028	0	0.251329	inf
Ciclev10011769m.g	scaffold_6:25244682-25248276	0	0.109411	inf
Ciclev10013630m.g	scaffold_6:1220295-1325561	0	0.112795	inf

Ciclev10011635m.g	scaffold_6:3807370-3811072	0	0.254474	inf
Ciclev10011923m.g	scaffold_6:7049852-7053668	0	0.197629	inf
Ciclev10013419m.g	scaffold_6:10652105-10652330	0	3.61468	inf
Ciclev10013515m.g	scaffold_6:11274929-11277919	0	0.136334	inf
Ciclev10013509m.g	scaffold_6:11734006-11735086	0	0.302784	inf
Ciclev10011144m.g	scaffold_6:12347021-12349259	0	0.0661834	inf
Ciclev10013495m.g	scaffold_6:12381601-12383003	0	3.52482	inf
Ciclev10011147m.g	scaffold_6:12497441-12499682	0	0.370019	inf
Ciclev10012389m.g	scaffold_6:13633286-13635478	0	0.310302	inf
Ciclev10011359m.g	scaffold_6:14932984-15010648	0	0.0783966	inf
Ciclev10012026m.g	scaffold_6:16429542-16431668	0	0.87852	inf
Ciclev10013617m.g	scaffold_6:16536501-16539631	0	1.80679	inf
Ciclev10013559m.g	scaffold_6:16888495-16892179	0	0.122139	inf
Ciclev10013403m.g	scaffold_6:16963849-16970364	0	0.0440988	inf
Ciclev10013230m.g	scaffold_6:17623857-17628989	0	0.644227	inf
Ciclev10013314m.g	scaffold_6:17623857-17628989	0	0.541815	inf
Ciclev10013711m.g	scaffold_6:17693653-17694284	0	0.287529	inf
Ciclev10013392m.g	scaffold_6:17730417-17731431	0	0.64592	inf
-	scaffold_6:17808048-17808630	0	8.49857	inf
Ciclev10013321m.g	scaffold_6:18647507-18649731	0	0.763524	inf
Ciclev10013344m.g	scaffold_6:18730873-18735717	0	0.0768444	inf
Ciclev10013347m.g	scaffold_6:19716193-19716727	0	0.375302	inf
Ciclev10011847m.g	scaffold_6:19822789-19824194	0	0.388058	inf
Ciclev10013282m.g	scaffold_6:19885485-19888972	0	0.350652	inf
Ciclev10013825m.g	scaffold_6:20352004-20353765	0	0.652568	inf
Ciclev10011166m.g	scaffold_6:20390386-20395751	0	0.171683	inf
Ciclev10011743m.g	scaffold_6:20566274-20568076	0	0.105083	inf
Ciclev10012087m.g	scaffold_6:20997251-20998995	0	0.117049	inf
Ciclev10013432m.g	scaffold_6:21040743-21044405	0	0.486077	inf
Ciclev10013374m.g	scaffold_6:21612984-21615876	0	0.168539	inf
Ciclev10012421m.g	scaffold_6:22070752-22071850	0	0.152385	inf
Ciclev10011549m.g	scaffold_6:22281545-22285426	0	0.0912969	inf
Ciclev10012974m.g	scaffold_6:22543667-22544711	0	0.275373	inf
Ciclev10013322m.g	scaffold_6:22638981-22639317	0	3.23634	inf
Ciclev10013530m.g	scaffold_6:23006704-23008187	0	0.253468	inf
Ciclev10011421m.g	scaffold_6:23498042-23501435	0	0.0890286	inf
Ciclev10012543m.g	scaffold_6:24189855-24190876	0	0.170505	inf
-	scaffold_6:19200151-19200762	0	13.8353	inf
-	scaffold_6:25539794-25540135	0	5.23863	inf
Ciclev10025564m.g	scaffold_7:300246-303269	0	0.116125	inf
Ciclev10027274m.g	scaffold_7:687578-688450	0	0.27317	inf
Ciclev10026231m.g	scaffold_7:1631545-1632652	0	1.09722	inf
Ciclev10025211m.g	scaffold_7:3775848-3778438	0	0.0685422	inf
Ciclev10027584m.g	scaffold_7:4329615-4330173	0	0.695687	inf
Ciclev10026033m.g	scaffold_7:4785947-4788267	0	0.775429	inf
Ciclev10025074m.g	scaffold_7:4965340-4971291	0	1.80429	inf
Ciclev10026878m.g	scaffold_7:5173845-5179864	0	0.367509	inf
Ciclev10027273m.g	scaffold_7:5406805-5411994	0	0.0554846	inf
Ciclev10026934m.g	scaffold_7:6321056-6321383	0	3.68715	inf
Ciclev10027128m.g	scaffold_7:6722212-6722940	0	3.52476	inf
Ciclev10026856m.g	scaffold_7:6990817-6991432	0	0.426204	inf
Ciclev10024773m.g	scaffold_7:7403229-7406773	0	0.436861	inf
Ciclev10026830m.g	scaffold_7:8171703-8172405	0	6.04612	inf
Ciclev10026890m.g	scaffold_7:8176336-8177031	0	3.07511	inf
Ciclev10026882m.g	scaffold_7:8184600-8185225	0	5.22375	inf
Ciclev10027308m.g	scaffold_7:9258105-9258516	0	0.61165	inf

Ciclev10027424m.g	scaffold_7:9677385-9679215	0	0.355063	inf
Ciclev10026928m.g,Ciclev10027187m.g	scaffold_7:9733633-9808728	0	3.90764	inf
Ciclev10024873m.g	scaffold_7:9866628-9869292	0	0.598708	inf
Ciclev10027169m.g	scaffold_7:10555107-10583369	0	0.125091	inf
Ciclev10027144m.g	scaffold_7:11375465-11376707	0	2.2264	inf
Ciclev10025000m.g	scaffold_7:11675047-11677298	0	0.915565	inf
Ciclev10027362m.g	scaffold_7:11850299-12160617	0	0.147651	inf
Ciclev10027593m.g	scaffold_7:11850299-12160617	0	0.054686	inf
Ciclev10024833m.g	scaffold_7:11850299-12160617	0	0.0476522	inf
Ciclev10025019m.g	scaffold_7:12783212-12790432	0	0.177726	inf
Ciclev10025726m.g	scaffold_7:13916806-14069972	0	3.54854	inf
Ciclev10026430m.g	scaffold_7:15088571-15091916	0	1.03135	inf
Ciclev10024852m.g	scaffold_7:15425968-15428800	0	0.281631	inf
Ciclev10026207m.g	scaffold_7:16575375-16577192	0	0.585753	inf
Ciclev10026901m.g	scaffold_7:16616365-16618215	0	1.52111	inf
Ciclev10027045m.g	scaffold_7:17073722-17074316	0	0.406214	inf
Ciclev10027382m.g	scaffold_7:18056820-18058484	0	3.43326	inf
Ciclev10025385m.g	scaffold_7:18579205-18580747	0	0.0983607	inf
Ciclev10025807m.g	scaffold_7:18672488-18674078	0	11.4033	inf
Ciclev10027326m.g	scaffold_7:19093472-19094081	0	2.43672	inf
Ciclev10027547m.g	scaffold_7:19596007-19596694	0	0.538409	inf
Ciclev10025240m.g	scaffold_7:19950882-20044587	0	0.23159	inf
Ciclev10026060m.g	scaffold_7:20049935-20052008	0	10.5124	inf
Ciclev10027051m.g	scaffold_7:20531863-20534975	0	0.223631	inf
Ciclev10027060m.g	scaffold_7:20536313-20540020	0	0.0880725	inf
Ciclev10026099m.g	scaffold_7:1956586-1963890	0	0.323165	inf
Ciclev10027249m.g	scaffold_7:2347626-2349072	0	0.205361	inf
Ciclev10027622m.g	scaffold_7:2610854-2615635	0	0.318907	inf
Ciclev10027017m.g	scaffold_7:3702756-3703353	0	0.834754	inf
Ciclev10025567m.g	scaffold_7:3972875-3974255	0	0.127526	inf
Ciclev10027204m.g	scaffold_7:5223852-5224188	0	4.42877	inf
Ciclev10027092m.g	scaffold_7:5224953-5225875	0	0.417015	inf
Ciclev10024976m.g	scaffold_7:6020268-6022786	0	0.175321	inf
Ciclev10025243m.g	scaffold_7:6548429-6564074	0	0.337836	inf
Ciclev10025100m.g	scaffold_7:7127012-7130647	0	0.0744615	inf
Ciclev10025729m.g,Ciclev10025922m.g	scaffold_7:7517438-7641496	0	18.8928	inf
Ciclev10026201m.g	scaffold_7:7788844-7791875	0	0.141867	inf
Ciclev10027156m.g	scaffold_7:7946417-7947875	0	0.114937	inf
Ciclev10026969m.g	scaffold_7:8098846-8102988	0	0.120456	inf
Ciclev10026930m.g	scaffold_7:8145467-8149699	0	1.14707	inf
Ciclev10025841m.g	scaffold_7:9050210-9052723	0	0.223489	inf
Ciclev10026993m.g	scaffold_7:9055027-9085218	0	0.344256	inf
Ciclev10024931m.g	scaffold_7:9104989-9108131	0	0.125694	inf
Ciclev10024714m.g	scaffold_7:10281834-10421429	0	0.10471	inf
Ciclev10025732m.g	scaffold_7:10595204-10596667	0	0.858672	inf
Ciclev10027242m.g	scaffold_7:10787932-10805299	0	0.060539	inf
Ciclev10027078m.g	scaffold_7:10961025-10961724	0	4.24634	inf
Ciclev10027336m.g	scaffold_7:10970809-10974414	0	0.473501	inf
Ciclev10027340m.g	scaffold_7:11365519-11365819	0	0.995981	inf
Ciclev10027096m.g	scaffold_7:11577923-11579702	0	0.194646	inf
Ciclev10025958m.g	scaffold_7:11591724-11593393	0	0.142053	inf
-	scaffold_7:11838567-11838935	0	27.8176	inf
Ciclev10027407m.g	scaffold_7:11850299-12160617	0	0.196553	inf
Ciclev10027180m.g	scaffold_7:11850299-12160617	0	3.96639	inf

Ciclev10025198m.g	scaffold_7:12352172-12754529	0	2.52547	inf
Ciclev10027264m.g	scaffold_7:12352172-12754529	0	6.21871	inf
Ciclev10026665m.g	scaffold_7:13375804-13379772	0	0.262237	inf
Ciclev10027595m.g	scaffold_7:13510252-13511938	0	0.0989401	inf
Ciclev10027021m.g	scaffold_7:13833459-13838345	0	0.0722479	inf
Ciclev10025077m.g	scaffold_7:13870276-13872819	0	0.151877	inf
Ciclev10024814m.g	scaffold_7:14385041-14388195	0	0.277801	inf
Ciclev10026861m.g	scaffold_7:15096780-15097784	0	0.20257	inf
Ciclev10027391m.g	scaffold_7:17272551-17304761	0	1.9073	inf
Ciclev10027376m.g	scaffold_7:18398719-18401565	0	0.871801	inf
Ciclev10025090m.g	scaffold_7:18820878-18823325	0	0.0789042	inf
Ciclev10026966m.g	scaffold_7:19428360-19430437	0	1.26956	inf
Ciclev10026027m.g	scaffold_7:19688702-19689980	0	0.553336	inf
Ciclev10027089m.g	scaffold_7:19737000-19738215	0	0.751395	inf
Ciclev10027283m.g	scaffold_7:19950882-20044587	0	0.108126	inf
-	scaffold_7:3711580-3712105	0	5.23837	inf
-	scaffold_7:9119411-9119658	0	39.4462	inf
-	scaffold_7:14296698-14296913	0	50.6752	inf
Ciclev10028925m.g	scaffold_8:1382419-1384318	0	0.243522	inf
Ciclev10029950m.g	scaffold_8:1576053-1576533	0	30.8189	inf
Ciclev10027822m.g	scaffold_8:1637544-1641072	0	0.0512724	inf
Ciclev10029838m.g	scaffold_8:2250303-2251238	0	0.508365	inf
Ciclev10028922m.g	scaffold_8:2437677-2439587	0	0.265366	inf
Ciclev10030275m.g	scaffold_8:2526107-2526803	0	0.470918	inf
Ciclev10028156m.g	scaffold_8:4096293-4099510	0	0.0790731	inf
Ciclev10029642m.g	scaffold_8:4417256-4417859	0	0.354184	inf
Ciclev10030072m.g	scaffold_8:5378293-5380297	0	0.506559	inf
Ciclev10029948m.g	scaffold_8:6608854-6610058	0	3.76998	inf
Ciclev10029745m.g	scaffold_8:6651050-6656927	0	0.28816	inf
Ciclev10028488m.g	scaffold_8:9325618-9585469	0	0.900454	inf
Ciclev10029866m.g	scaffold_8:9789014-9789753	0	0.211571	inf
Ciclev10029819m.g	scaffold_8:10593474-10661453	0	2.55255	inf
Ciclev10030195m.g	scaffold_8:11043183-11043909	0	5.64522	inf
Ciclev10028972m.g	scaffold_8:11052484-11059274	0	4.63245	inf
Ciclev10029949m.g	scaffold_8:16565605-16565893	0	1.0319	inf
Ciclev10029734m.g	scaffold_8:17291958-17467945	0	1.01534	inf
Ciclev10029725m.g	scaffold_8:17510925-17513265	0	0.649891	inf
Ciclev10030088m.g	scaffold_8:18818111-18818648	0	1.81857	inf
Ciclev10030271m.g	scaffold_8:20231586-20232948	0	0.130032	inf
Ciclev10030045m.g	scaffold_8:20244584-20245784	0	0.85237	inf
Ciclev10029678m.g	scaffold_8:20361174-20363775	0	0.848188	inf
Ciclev10029752m.g	scaffold_8:21374018-21379609	0	0.568054	inf
Ciclev10030290m.g	scaffold_8:22893978-22894488	0	0.374804	inf
Ciclev10030050m.g	scaffold_8:22906465-22909241	0	0.174548	inf
Ciclev10029134m.g	scaffold_8:23036290-23037524	0	0.152161	inf
Ciclev10030201m.g	scaffold_8:23758162-23760211	0	0.0767965	inf
Ciclev10030147m.g	scaffold_8:23773416-23773788	0	0.715401	inf
Ciclev10029047m.g	scaffold_8:23855427-23856852	0	2.31634	inf
Ciclev10029817m.g	scaffold_8:23896445-23896997	0	0.634485	inf
Ciclev10030173m.g	scaffold_8:24433990-24437012	0	0.134519	inf
Ciclev10029962m.g	scaffold_8:24816478-24817308	0	0.391538	inf
Ciclev10028356m.g	scaffold_8:24835725-24837870	0	0.0921384	inf
Ciclev10029761m.g	scaffold_8:393478-397894	0	0.527259	inf
Ciclev10030346m.g	scaffold_8:608576-608987	0	0.559757	inf
Ciclev10029720m.g	scaffold_8:803823-805726	0	0.624157	inf
Ciclev10028943m.g	scaffold_8:1169450-1171206	0	0.46389	inf

Ciclev10030029m.g	scaffold_8:1443945-1489074	0	12.3211	inf
Ciclev10029403m.g	scaffold_8:1957965-1958623	0	0.307518	inf
Ciclev10028183m.g	scaffold_8:1975977-1980834	0	0.0860443	inf
Ciclev10029599m.g	scaffold_8:3399074-3434977	0	1.31086	inf
Ciclev10029958m.g	scaffold_8:4058124-4059089	0	0.194839	inf
Ciclev10028333m.g	scaffold_8:4165948-4167414	0	1.41035	inf
Ciclev10029953m.g	scaffold_8:4430605-4431541	0	0.157125	inf
Ciclev10029805m.g	scaffold_8:6178780-6179651	0	0.303108	inf
Ciclev10029650m.g	scaffold_8:7378617-7379170	0	2.693	inf
Ciclev10028275m.g	scaffold_8:8247229-8248860	0	0.847503	inf
Ciclev10028273m.g	scaffold_8:9025006-9167585	0	0.153781	inf
Ciclev10028276m.g	scaffold_8:10154368-10156192	0	0.623924	inf
Ciclev10030244m.g	scaffold_8:10887281-10889137	0	0.201551	inf
Ciclev10030243m.g	scaffold_8:10985579-10985960	0	0.679067	inf
Ciclev10030195m.g	scaffold_8:11043183-11043909	0	2.26459	inf
Ciclev10029839m.g	scaffold_8:11052484-11059274	0	4.14484	inf
Ciclev10028972m.g	scaffold_8:11052484-11059274	0	9.40691	inf
Ciclev10029811m.g	scaffold_8:13417527-13420177	0	0.0852586	inf
Ciclev10030104m.g	scaffold_8:17173235-17173659	0	6.99403	inf
Ciclev10030085m.g	scaffold_8:17532317-17532840	0	1.59813	inf
Ciclev10029327m.g	scaffold_8:17536171-17537653	0	1.42656	inf
Ciclev10029328m.g	scaffold_8:17543099-17544465	0	1.06487	inf
Ciclev10030285m.g	scaffold_8:17700391-17703618	0	0.440549	inf
Ciclev10030150m.g	scaffold_8:17970888-17971293	0	0.495712	inf
Ciclev10029978m.g	scaffold_8:18427607-18430214	0	0.702187	inf
Ciclev10027723m.g	scaffold_8:18538163-18541888	0	0.0549684	inf
Ciclev10028749m.g	scaffold_8:18973864-19114160	0	0.9767	inf
Ciclev10029080m.g	scaffold_8:18973864-19114160	0	0.122522	inf
Ciclev10029984m.g	scaffold_8:21126528-21127708	0	0.317788	inf
Ciclev10030227m.g	scaffold_8:21643318-21644836	0	0.411301	inf
Ciclev10030317m.g	scaffold_8:22176801-22177680	0	0.189488	inf
Ciclev10030433m.g	scaffold_8:25006510-25009433	0	0.363753	inf
-	scaffold_8:3399074-3434977	0	12.5205	inf
-	scaffold_8:5801973-5802305	0	10.1805	inf
-	scaffold_8:19132836-19211350	0	45.5021	inf
Ciclev10006996m.g	scaffold_9:863889-865750	0	0.205607	inf
Ciclev10006963m.g	scaffold_9:1077086-1077668	0	0.594934	inf
-	scaffold_9:1202701-1203016	0	498.01	inf
Ciclev10007081m.g	scaffold_9:1267486-1268491	0	4.30976	inf
Ciclev10005259m.g	scaffold_9:1886166-1894747	0	6.83808	inf
Ciclev10006650m.g	scaffold_9:2746708-2747212	0	1.29376	inf
Ciclev10006350m.g	scaffold_9:2768407-2829028	0	0.211804	inf
Ciclev10007187m.g	scaffold_9:3027196-3028465	0	2.36085	inf
Ciclev10007197m.g	scaffold_9:3675171-3684455	0	0.667764	inf
Ciclev10006770m.g	scaffold_9:3854320-3855298	0	0.778329	inf
Ciclev10005603m.g	scaffold_9:4865058-4866116	0	0.121502	inf
Ciclev10004874m.g	scaffold_9:4935788-4938423	0	1.10462	inf
Ciclev10006313m.g	scaffold_9:4999269-5000020	0	0.317674	inf
Ciclev10007125m.g	scaffold_9:5021287-5065070	0	0.145489	inf
Ciclev10005809m.g	scaffold_9:5573317-5575024	0	5.39196	inf
Ciclev10007034m.g	scaffold_9:6539810-6546914	0	0.129294	inf
Ciclev10005281m.g	scaffold_9:6852155-6854052	0	3.26456	inf
Ciclev10004866m.g	scaffold_9:7436354-7440439	0	0.0870767	inf
Ciclev10005841m.g	scaffold_9:7804322-8050719	0	2.58616	inf
Ciclev10006624m.g	scaffold_9:8621402-8627840	0	0.227291	inf
Ciclev10006296m.g	scaffold_9:9897529-9900153	0	0.75465	inf

Ciclev10006428m.g	scaffold_9:10403517-10409781	0	0.0983084	inf
Ciclev10006466m.g	scaffold_9:10631890-10634603	0	0.122637	inf
Ciclev10006779m.g	scaffold_9:11576287-11715575	0	1.51768	inf
Ciclev10006460m.g	scaffold_9:13821818-13822721	0	0.208364	inf
Ciclev10005659m.g	scaffold_9:14578431-14579950	0	0.82236	inf
Ciclev10005791m.g	scaffold_9:14676236-14679090	0	0.163076	inf
Ciclev10006693m.g	scaffold_9:14772006-14772668	0	0.526479	inf
Ciclev10005230m.g	scaffold_9:15422640-15491415	0	0.28984	inf
Ciclev10006576m.g	scaffold_9:16167889-16169537	0	0.222515	inf
Ciclev10006137m.g	scaffold_9:20360609-20494992	0	1.07017	inf
Ciclev10005181m.g	scaffold_9:21248441-21518846	0	37.0056	inf
Ciclev10005653m.g	scaffold_9:22953693-22955969	0	0.792051	inf
Ciclev10006797m.g	scaffold_9:24682631-24756976	0	0.0883644	inf
Ciclev10004787m.g	scaffold_9:25684459-25689922	0	0.261906	inf
Ciclev10005700m.g	scaffold_9:25733960-25737217	0	0.230017	inf
Ciclev10007144m.g	scaffold_9:26325271-26327639	0	0.108683	inf
Ciclev10006327m.g,Ciclev10006895m.g	scaffold_9:26389069-26391138	0	6.12741	inf
Ciclev10007060m.g	scaffold_9:27391209-27395869	0	0.0812757	inf
Ciclev10005149m.g	scaffold_9:27470740-27472322	0	0.978104	inf
Ciclev10005888m.g	scaffold_9:27633409-27634188	0	0.454423	inf
Ciclev10006518m.g	scaffold_9:27660095-27662540	0	0.40201	inf
Ciclev10004935m.g	scaffold_9:28484459-28487210	0	0.120788	inf
Ciclev10007208m.g	scaffold_9:28693001-28697846	0	1.30086	inf
Ciclev10007021m.g	scaffold_9:29663656-29665195	0	0.752426	inf
Ciclev10006768m.g	scaffold_9:30213003-30215415	0	0.135064	inf
Ciclev10004563m.g	scaffold_9:30329357-30336981	0	0.0798915	inf
Ciclev10006208m.g	scaffold_9:30628790-30632184	0	0.313702	inf
Ciclev10005553m.g	scaffold_9:344504-345613	0	0.209113	inf
Ciclev10006030m.g	scaffold_9:964810-967550	0	0.226956	inf
Ciclev10007186m.g	scaffold_9:1600935-1605603	0	1.18054	inf
Ciclev10006926m.g	scaffold_9:1895518-1900796	0	0.117472	inf
Ciclev10006579m.g	scaffold_9:2768407-2829028	0	0.685965	inf
Ciclev10004995m.g	scaffold_9:2962884-2964424	0	0.368762	inf
Ciclev10005241m.g	scaffold_9:3782133-3783405	0	0.142965	inf
Ciclev10006620m.g	scaffold_9:4223892-4225907	0	3.0556	inf
Ciclev10007070m.g	scaffold_9:5013969-5014284	0	5.37339	inf
Ciclev10007123m.g	scaffold_9:5014844-5015236	0	37.9753	inf
Ciclev10005835m.g	scaffold_9:7785534-7786957	0	5.64344	inf
Ciclev10006901m.g	scaffold_9:7804322-8050719	0	2.62053	inf
Ciclev10006616m.g	scaffold_9:7804322-8050719	0	1.55093	inf
Ciclev10007011m.g	scaffold_9:8577405-8581930	0	0.0672044	inf
Ciclev10007039m.g	scaffold_9:9817144-9818401	0	0.143637	inf
Ciclev10006659m.g	scaffold_9:9835684-9837080	0	0.274126	inf
Ciclev10005120m.g	scaffold_9:9859952-9862224	0	0.233441	inf
Ciclev10005919m.g	scaffold_9:9943904-9944829	0	0.157524	inf
Ciclev10005017m.g	scaffold_9:9983011-9984385	0	1.1506	inf
Ciclev10005543m.g	scaffold_9:11232320-11235048	0	0.441099	inf
Ciclev10007117m.g	scaffold_9:11576287-11715575	0	0.321667	inf
Ciclev10007217m.g	scaffold_9:19843325-19844670	0	7.08613	inf
Ciclev10006889m.g	scaffold_9:20890036-21029395	0	0.548476	inf
Ciclev10006496m.g	scaffold_9:20890036-21029395	0	0.40323	inf
Ciclev10004886m.g	scaffold_9:23144625-23146202	0	0.384206	inf
Ciclev10005368m.g	scaffold_9:23487944-23490847	0	0.860214	inf
Ciclev10005983m.g	scaffold_9:25868407-25869181	0	1.01524	inf
Ciclev10006867m.g	scaffold_9:26271132-26272356	0	0.71804	inf

Ciclev10005090m.g	scaffold_9:26274556-26275771	0	0.178772	inf
Ciclev10004971m.g	scaffold_9:26385232-26387268	0	0.589147	inf
Ciclev10006785m.g	scaffold_9:26411664-26413063	0	0.249026	inf
Ciclev10007127m.g	scaffold_9:26466915-26487635	0	2.50295	inf
Ciclev10006376m.g	scaffold_9:26466915-26487635	0	0.407554	inf
Ciclev10006672m.g	scaffold_9:26691051-26694187	0	0.542555	inf
Ciclev10004936m.g	scaffold_9:26694563-26789030	0	0.509097	inf
Ciclev10007190m.g	scaffold_9:27076627-27080193	0	0.882052	inf
Ciclev10005151m.g	scaffold_9:27270413-27276140	0	17.3258	inf
Ciclev10006737m.g	scaffold_9:27580479-27582318	0	0.767925	inf
Ciclev10006594m.g	scaffold_9:28673139-28675442	0	0.610718	inf
Ciclev10006265m.g	scaffold_9:28781519-28783407	0	1.2124	inf
Ciclev10006820m.g	scaffold_9:30119093-30122978	0	0.186346	inf
Ciclev10006936m.g	scaffold_9:30157877-30161153	0	0.0915471	inf
Ciclev10005465m.g	scaffold_9:30533004-30536569	0	0.224517	inf
Ciclev10006695m.g	scaffold_9:30550297-30551528	0	0.378168	inf
Ciclev10007156m.g	scaffold_9:30581462-30583125	0	1.87955	inf
Ciclev10006882m.g	scaffold_9:30591032-30592658	0	0.125295	inf
Ciclev10007054m.g	scaffold_9:30707233-30713317	0	5.25719	inf
Ciclev10006591m.g	scaffold_9:31147928-31149521	0	0.157611	inf
-	scaffold_9:1233793-1233997	0	51.5898	inf
-	scaffold_9:1264737-1265015	0	24.4387	inf
-	scaffold_9:6041076-6041354	0	14.3757	inf
-	scaffold_9:30744441-30745008	0	4.66363	inf
Ciclev10030448m.g	scaffold_935:1553-3173	0	0.511007	inf
Ciclev10010134m.g	scaffold_1:738190-743742	1.92312	0	#NAME?
-	scaffold_1:779311-812949	234.949	0	#NAME?
-	scaffold_1:822300-823342	4.83786	0	#NAME?
Ciclev10010622m.g	scaffold_1:903506-904220	3.22534	0	#NAME?
Ciclev10010440m.g	scaffold_1:1886649-1889832	0.141101	0	#NAME?
Ciclev10010173m.g	scaffold_1:2143941-2144169	17.519	0	#NAME?
Ciclev10008396m.g	scaffold_1:2237003-2346942	0.71573	0	#NAME?
Ciclev10010018m.g	scaffold_1:2429757-2430704	0.236492	0	#NAME?
Ciclev10010431m.g	scaffold_1:2433379-2439823	0.0378692	0	#NAME?
Ciclev10008128m.g	scaffold_1:2544153-2549879	0.0938487	0	#NAME?
Ciclev10010565m.g	scaffold_1:3001865-3002249	1.67101	0	#NAME?
Ciclev10008050m.g	scaffold_1:3187977-3192424	0.0972615	0	#NAME?
Ciclev10010609m.g	scaffold_1:3208798-3212434	0.182059	0	#NAME?
Ciclev10009158m.g	scaffold_1:3442375-3444018	0.513868	0	#NAME?
Ciclev10009991m.g	scaffold_1:4343811-4351112	1.58109	0	#NAME?
Ciclev10009574m.g	scaffold_1:4682972-4684163	0.244549	0	#NAME?
Ciclev10007850m.g	scaffold_1:4803573-4806836	0.577414	0	#NAME?
Ciclev10010748m.g	scaffold_1:5090444-5093541	0.0804669	0	#NAME?
Ciclev10010307m.g	scaffold_1:5413232-5416822	0.713154	0	#NAME?
Ciclev10010204m.g	scaffold_1:5533736-5538432	0.221917	0	#NAME?
Ciclev10010642m.g	scaffold_1:5862184-5863328	1.08189	0	#NAME?
Ciclev10010340m.g	scaffold_1:6245181-6245493	30.0031	0	#NAME?
Ciclev10008914m.g	scaffold_1:6309705-6310686	0.246662	0	#NAME?
Ciclev10009432m.g	scaffold_1:6317159-6317866	1.88159	0	#NAME?
Ciclev10007894m.g	scaffold_1:6491367-6493720	0.394651	0	#NAME?
Ciclev10009070m.g	scaffold_1:6847017-6850082	0.142208	0	#NAME?
Ciclev10009138m.g	scaffold_1:7151774-7153282	5.85074	0	#NAME?
Ciclev10010258m.g	scaffold_1:9437872-9442295	0.086942	0	#NAME?
Ciclev10010657m.g	scaffold_1:11489361-11489649	3.64683	0	#NAME?
-	scaffold_1:14847404-14848726	9.01688	0	#NAME?
Ciclev10009812m.g	scaffold_1:17682050-17682636	0.417683	0	#NAME?

Ciclev10010335m.g	scaffold_1:18323300-18326219	0.115713	0	#NAME?
Ciclev10009758m.g	scaffold_1:18493265-18493944	0.532726	0	#NAME?
Ciclev10007596m.g	scaffold_1:19808532-19811747	0.076314	0	#NAME?
Ciclev10007572m.g	scaffold_1:19905420-19995040	0.143068	0	#NAME?
Ciclev10010750m.g	scaffold_1:20482906-20483685	0.484263	0	#NAME?
Ciclev10010312m.g	scaffold_1:22104256-22107361	0.075455	0	#NAME?
Ciclev10010555m.g	scaffold_1:22175413-22384190	0.109868	0	#NAME?
Ciclev10010571m.g	scaffold_1:22498750-22504815	1.33954	0	#NAME?
Ciclev10009874m.g	scaffold_1:22632248-22634743	1.5474	0	#NAME?
Ciclev10007329m.g	scaffold_1:23833802-23837179	0.176435	0	#NAME?
Ciclev10010746m.g	scaffold_1:23902989-23904990	0.0836701	0	#NAME?
Ciclev10010352m.g	scaffold_1:24154498-24154849	1.20899	0	#NAME?
Ciclev10007805m.g	scaffold_1:24281879-24283664	0.261895	0	#NAME?
Ciclev10007343m.g	scaffold_1:24294488-24297455	0.114329	0	#NAME?
Ciclev10010343m.g	scaffold_1:24769194-24772566	0.275453	0	#NAME?
Ciclev10010235m.g	scaffold_1:25170832-25172421	0.313661	0	#NAME?
Ciclev10009014m.g	scaffold_1:25222374-25226019	1.77299	0	#NAME?
Ciclev10010595m.g	scaffold_1:25390738-25392395	0.369856	0	#NAME?
Ciclev10010543m.g	scaffold_1:25675939-25678679	0.230533	0	#NAME?
Ciclev10010281m.g	scaffold_1:26364825-26370951	0.203826	0	#NAME?
Ciclev10010702m.g	scaffold_1:26523083-26523395	1.53733	0	#NAME?
Ciclev10010065m.g	scaffold_1:26578934-26579388	1.65208	0	#NAME?
Ciclev10008951m.g	scaffold_1:27419631-27421046	1.98667	0	#NAME?
Ciclev10008778m.g	scaffold_1:27446277-27447735	0.391441	0	#NAME?
Ciclev10009118m.g	scaffold_1:27449397-27451036	0.327113	0	#NAME?
Ciclev10010558m.g	scaffold_1:27894315-27895500	0.441403	0	#NAME?
Ciclev10007374m.g	scaffold_1:28678971-28683086	0.283794	0	#NAME?
Ciclev10010302m.g	scaffold_1:204819-205338	0.526808	0	#NAME?
Ciclev10007641m.g	scaffold_1:584264-587895	1.37236	0	#NAME?
Ciclev10009970m.g	scaffold_1:691605-692511	1.51929	0	#NAME?
Ciclev10010446m.g	scaffold_1:1102613-1102853	3.45105	0	#NAME?
Ciclev10010719m.g	scaffold_1:1753963-1755103	0.189144	0	#NAME?
Ciclev10010712m.g	scaffold_1:1826664-1830235	0.490277	0	#NAME?
Ciclev10010300m.g	scaffold_1:2030627-2030933	1.36498	0	#NAME?
Ciclev10010324m.g	scaffold_1:2109918-2110839	4.36102	0	#NAME?
Ciclev10008711m.g	scaffold_1:2173895-2176927	11.4728	0	#NAME?
Ciclev10010490m.g	scaffold_1:2916045-2918030	0.471896	0	#NAME?
Ciclev10007892m.g	scaffold_1:2981715-2984571	1.24143	0	#NAME?
Ciclev10010551m.g	scaffold_1:3113082-3117053	0.303472	0	#NAME?
Ciclev10010157m.g	scaffold_1:3704737-3706120	0.578895	0	#NAME?
Ciclev10009387m.g	scaffold_1:3975910-3976753	0.509133	0	#NAME?
Ciclev10009700m.g	scaffold_1:4179882-4180823	0.86707	0	#NAME?
Ciclev10010410m.g	scaffold_1:5189846-5192178	0.336276	0	#NAME?
Ciclev10010697m.g	scaffold_1:5225008-5225868	0.384541	0	#NAME?
Ciclev10009470m.g	scaffold_1:6647157-6649599	1.46865	0	#NAME?
Ciclev10008296m.g	scaffold_1:7793458-7795200	0.286138	0	#NAME?
Ciclev10008962m.g	scaffold_1:8211437-8213973	0.148013	0	#NAME?
Ciclev10009867m.g	scaffold_1:8680638-8681043	3.93095	0	#NAME?
Ciclev10010673m.g	scaffold_1:11211640-11212081	3.28244	0	#NAME?
Ciclev10010272m.g	scaffold_1:12956852-12957134	3.87249	0	#NAME?
Ciclev10010561m.g	scaffold_1:15231882-15232893	0.196068	0	#NAME?
Ciclev10009079m.g	scaffold_1:16102481-16104368	10.7541	0	#NAME?
Ciclev10008720m.g	scaffold_1:16289228-16291434	0.497063	0	#NAME?
Ciclev10010201m.g	scaffold_1:16471756-16473040	0.15396	0	#NAME?
Ciclev10010694m.g	scaffold_1:18461483-18462594	0.204584	0	#NAME?
Ciclev10007816m.g	scaffold_1:19612175-19653994	0.213353	0	#NAME?

Ciclev10010640m.g	scaffold_1:21879943-21881649	0.142787	0	#NAME?
Ciclev10007334m.g	scaffold_1:22064722-22067780	0.143648	0	#NAME?
Ciclev10010510m.g	scaffold_1:22722026-22723655	0.653537	0	#NAME?
Ciclev10010148m.g	scaffold_1:22724566-22725936	0.188979	0	#NAME?
Ciclev10007620m.g	scaffold_1:22736221-22741607	0.0788429	0	#NAME?
Ciclev10007984m.g	scaffold_1:22784903-22786989	0.691328	0	#NAME?
Ciclev10010586m.g	scaffold_1:23020421-23020933	0.715897	0	#NAME?
Ciclev10010194m.g	scaffold_1:23356218-23357706	0.25998	0	#NAME?
Ciclev10008571m.g	scaffold_1:23446758-23448254	0.314097	0	#NAME?
Ciclev10007682m.g	scaffold_1:23483863-23485840	0.276882	0	#NAME?
Ciclev10008392m.g	scaffold_1:24456729-24458491	0.14593	0	#NAME?
Ciclev10008257m.g	scaffold_1:25462099-25463684	0.344402	0	#NAME?
Ciclev10008460m.g	scaffold_1:25713468-25716134	0.265252	0	#NAME?
Ciclev10010109m.g	scaffold_1:25782999-25783920	0.783811	0	#NAME?
Ciclev10009567m.g	scaffold_1:25816948-25818472	3.82254	0	#NAME?
Ciclev10010653m.g	scaffold_1:26758507-26763534	0.0979875	0	#NAME?
Ciclev10010690m.g	scaffold_1:27122125-27122904	0.614716	0	#NAME?
Ciclev10010853m.g	scaffold_1:27454075-27454751	0.453464	0	#NAME?
Ciclev10010751m.g	scaffold_1:27537821-27538499	0.364156	0	#NAME?
Ciclev10008554m.g	scaffold_1:27809481-27811561	0.151402	0	#NAME?
Ciclev10010602m.g	scaffold_1:27877088-27878260	2.87705	0	#NAME?
Ciclev10008144m.g	scaffold_1:28087897-28093193	0.0929832	0	#NAME?
Ciclev10010237m.g	scaffold_1:28096308-28098687	0.185869	0	#NAME?
Ciclev10008182m.g	scaffold_1:28356620-28361117	0.45705	0	#NAME?
Ciclev10010403m.g	scaffold_1:28587251-28588220	0.382941	0	#NAME?
Ciclev10008821m.g	scaffold_1:28782168-28786731	43.5327	0	#NAME?
-	scaffold_1:16728356-16728612	32.8133	0	#NAME?
-	scaffold_1:25090683-25090863	303.879	0	#NAME?
Ciclev10013976m.g	scaffold_1197:653-2074	0.355952	0	#NAME?
Ciclev10013996m.g	scaffold_13:170037-171305	0.267261	0	#NAME?
Ciclev10027642m.g	scaffold_1761:321-1194	0.28039	0	#NAME?
Ciclev10027644m.g	scaffold_1791:1056-1674	3.39131	0	#NAME?
Ciclev10027656m.g	scaffold_1969:2278-2881	2.29585	0	#NAME?
Ciclev10017738m.g	scaffold_2:115379-116326	0.320641	0	#NAME?
Ciclev10018053m.g	scaffold_2:258527-260759	0.931843	0	#NAME?
Ciclev10017914m.g	scaffold_2:519940-521234	10.9288	0	#NAME?
Ciclev10017584m.g	scaffold_2:1061472-1064126	0.219505	0	#NAME?
Ciclev10015735m.g	scaffold_2:3106112-3108042	0.163742	0	#NAME?
Ciclev10017988m.g	scaffold_2:3615420-3615723	1.42857	0	#NAME?
Ciclev10018248m.g	scaffold_2:4675203-4675785	0.567443	0	#NAME?
Ciclev10015398m.g	scaffold_2:4749153-4750530	0.316964	0	#NAME?
Ciclev10015342m.g	scaffold_2:4790587-4792123	0.163702	0	#NAME?
Ciclev10017204m.g	scaffold_2:4860613-4861568	0.492859	0	#NAME?
Ciclev10018144m.g	scaffold_2:5078754-5083103	0.0494798	0	#NAME?
Ciclev10018194m.g	scaffold_2:5968187-5973023	2.17238	0	#NAME?
Ciclev10015508m.g	scaffold_2:6135150-6136571	0.415078	0	#NAME?
Ciclev10017530m.g	scaffold_2:6378117-6378959	0.322531	0	#NAME?
Ciclev10015312m.g	scaffold_2:6402410-6404196	0.952216	0	#NAME?
Ciclev10015684m.g	scaffold_2:6444507-6445673	0.414918	0	#NAME?
Ciclev10015137m.g	scaffold_2:6461655-6463229	2.11103	0	#NAME?
Ciclev10017658m.g	scaffold_2:6821901-6823565	0.460614	0	#NAME?
Ciclev10014939m.g	scaffold_2:6939112-6941487	0.0860282	0	#NAME?
Ciclev10017244m.g	scaffold_2:7477429-7479641	5.56069	0	#NAME?
Ciclev10015199m.g	scaffold_2:7789813-7792125	1.04908	0	#NAME?
Ciclev10016163m.g	scaffold_2:7958390-7959604	0.215166	0	#NAME?
Ciclev10017108m.g	scaffold_2:7964983-7965603	0.374448	0	#NAME?

Ciclev10017751m.g	scaffold_2:8095206-8097625	0.80778	0	#NAME?
Ciclev10017369m.g	scaffold_2:8129595-8151433	0.333904	0	#NAME?
Ciclev10016805m.g	scaffold_2:9048500-9051025	0.136914	0	#NAME?
Ciclev10016451m.g	scaffold_2:9060253-9065081	0.256302	0	#NAME?
Ciclev10017556m.g	scaffold_2:9205108-9212700	0.869663	0	#NAME?
Ciclev10016934m.g	scaffold_2:9302418-9303123	0.255158	0	#NAME?
Ciclev10017380m.g	scaffold_2:10421404-10421775	2.7103	0	#NAME?
Ciclev10017822m.g	scaffold_2:10508676-10511706	6.57838	0	#NAME?
Ciclev10017956m.g	scaffold_2:10600286-10602868	0.160605	0	#NAME?
Ciclev10016745m.g	scaffold_2:11647999-11648924	8.67521	0	#NAME?
Ciclev10015370m.g	scaffold_2:12546038-12549074	0.299019	0	#NAME?
Ciclev10015254m.g	scaffold_2:12609674-12614222	0.0888508	0	#NAME?
Ciclev10017534m.g	scaffold_2:13009831-13010215	0.871482	0	#NAME?
Ciclev10017602m.g	scaffold_2:13384976-13387611	3.06333	0	#NAME?
Ciclev10018317m.g	scaffold_2:13804883-13807889	0.127972	0	#NAME?
Ciclev10017394m.g	scaffold_2:13909410-13917679	3.1802	0	#NAME?
Ciclev10015280m.g	scaffold_2:15477859-15615778	0.341993	0	#NAME?
Ciclev10017473m.g	scaffold_2:16369571-16371813	0.149559	0	#NAME?
Ciclev10016270m.g	scaffold_2:18181399-18183878	0.800649	0	#NAME?
Ciclev10017701m.g	scaffold_2:19183607-19184030	2.37348	0	#NAME?
Ciclev10016159m.g	scaffold_2:19893157-19895112	3.12497	0	#NAME?
Ciclev10018089m.g	scaffold_2:20317333-20317849	1.31216	0	#NAME?
Ciclev10017992m.g	scaffold_2:20522768-20524073	0.203038	0	#NAME?
Ciclev10017968m.g	scaffold_2:21018428-21020505	0.438474	0	#NAME?
Ciclev10017097m.g	scaffold_2:21326708-21327604	2.60789	0	#NAME?
Ciclev10017196m.g	scaffold_2:22165223-22165777	0.569237	0	#NAME?
Ciclev10017308m.g	scaffold_2:23169240-23170061	0.257716	0	#NAME?
Ciclev10017791m.g	scaffold_2:23772178-23772739	1.62216	0	#NAME?
Ciclev10017825m.g	scaffold_2:23779968-23783164	2.34019	0	#NAME?
Ciclev10018021m.g	scaffold_2:24356826-24357552	3.17301	0	#NAME?
Ciclev10017890m.g	scaffold_2:24687331-24688417	3.34087	0	#NAME?
Ciclev10017948m.g	scaffold_2:24724190-24726432	0.281311	0	#NAME?
Ciclev10015681m.g	scaffold_2:25256236-25258995	0.373144	0	#NAME?
Ciclev10017820m.g	scaffold_2:25266840-25270230	0.0456154	0	#NAME?
Ciclev10014088m.g	scaffold_2:25323392-25327614	0.0432418	0	#NAME?
Ciclev10018050m.g	scaffold_2:25364550-25367417	0.318011	0	#NAME?
Ciclev10017139m.g	scaffold_2:25949443-25950694	0.512047	0	#NAME?
Ciclev10018355m.g	scaffold_2:25994465-25995956	0.347758	0	#NAME?
Ciclev10016207m.g	scaffold_2:26186879-26187859	0.188276	0	#NAME?
Ciclev10016789m.g	scaffold_2:26496914-26498229	1.22956	0	#NAME?
Ciclev10017511m.g	scaffold_2:26652370-26653431	0.449126	0	#NAME?
Ciclev10017657m.g	scaffold_2:27684970-27686533	0.21135	0	#NAME?
Ciclev10015241m.g	scaffold_2:28173643-28175932	0.164806	0	#NAME?
Ciclev10016091m.g	scaffold_2:28299798-28301388	0.131886	0	#NAME?
Ciclev10016885m.g	scaffold_2:28750960-28752040	0.928465	0	#NAME?
Ciclev10018331m.g	scaffold_2:28845910-28867192	53.9351	0	#NAME?
Ciclev10016461m.g	scaffold_2:29047757-29049298	0.200296	0	#NAME?
Ciclev10017355m.g	scaffold_2:29178268-29179947	4.51967	0	#NAME?
Ciclev10015034m.g	scaffold_2:29864677-29866537	0.358565	0	#NAME?
Ciclev10017650m.g	scaffold_2:30173800-30176421	0.0932887	0	#NAME?
Ciclev10017448m.g	scaffold_2:30233543-30236390	0.0771002	0	#NAME?
Ciclev10017493m.g	scaffold_2:30874329-30882715	2.01506	0	#NAME?
Ciclev10017778m.g	scaffold_2:31451288-31452190	1.28486	0	#NAME?
Ciclev10017926m.g	scaffold_2:31673119-31674064	0.256594	0	#NAME?
Ciclev10017152m.g	scaffold_2:31801438-31804455	0.167156	0	#NAME?
Ciclev10016725m.g	scaffold_2:32292657-32294714	2.69513	0	#NAME?

Ciclev10018168m.g	scaffold_2:32379265-32380015	0.331526	0	#NAME?
Ciclev10017836m.g	scaffold_2:32473592-32476324	2.57833	0	#NAME?
Ciclev10017674m.g	scaffold_2:32502347-32503086	0.409905	0	#NAME?
Ciclev10016060m.g	scaffold_2:32917197-32918336	0.324638	0	#NAME?
Ciclev10017808m.g	scaffold_2:32948414-32957830	0.352838	0	#NAME?
Ciclev10015663m.g	scaffold_2:32958088-32970806	0.152045	0	#NAME?
Ciclev10015894m.g	scaffold_2:33051429-33052705	0.767557	0	#NAME?
Ciclev10015093m.g	scaffold_2:33095852-33100051	0.129501	0	#NAME?
Ciclev10017784m.g	scaffold_2:33852534-33853911	1.05642	0	#NAME?
Ciclev10017321m.g	scaffold_2:34151656-34152301	1.00132	0	#NAME?
Ciclev10017787m.g	scaffold_2:34649711-34658144	0.0651013	0	#NAME?
Ciclev10017854m.g	scaffold_2:35330528-35334033	0.739747	0	#NAME?
Ciclev10018138m.g	scaffold_2:35440154-35440739	0.963198	0	#NAME?
Ciclev10014825m.g	scaffold_2:35442261-35446108	0.0980754	0	#NAME?
Ciclev10018040m.g	scaffold_2:35690666-35691155	0.549848	0	#NAME?
Ciclev10017384m.g	scaffold_2:35806348-35811690	0.920026	0	#NAME?
Ciclev10014746m.g	scaffold_2:36154259-36156265	0.22394	0	#NAME?
Ciclev10014163m.g	scaffold_2:83182-92491	1.77651	0	#NAME?
Ciclev10018348m.g	scaffold_2:699750-701347	0.175696	0	#NAME?
Ciclev10016030m.g	scaffold_2:737044-738121	1.5374	0	#NAME?
Ciclev10014713m.g	scaffold_2:3526273-3529653	0.640987	0	#NAME?
Ciclev10018091m.g	scaffold_2:4469350-4470252	0.790096	0	#NAME?
Ciclev10017776m.g	scaffold_2:5501695-5504211	2.29995	0	#NAME?
Ciclev10014207m.g	scaffold_2:5533590-5538729	0.34582	0	#NAME?
Ciclev10014934m.g	scaffold_2:6106686-6109539	0.111019	0	#NAME?
Ciclev10017960m.g	scaffold_2:6181064-6338878	0.142855	0	#NAME?
Ciclev10015310m.g	scaffold_2:6433215-6434534	3.17172	0	#NAME?
Ciclev10015539m.g	scaffold_2:6767817-6770987	0.476636	0	#NAME?
Ciclev10017500m.g	scaffold_2:7012362-7013949	0.340844	0	#NAME?
Ciclev10017529m.g	scaffold_2:7024875-7027557	0.576222	0	#NAME?
Ciclev10016919m.g	scaffold_2:8467023-8467784	0.692438	0	#NAME?
Ciclev10017414m.g	scaffold_2:9383252-9392832	0.180786	0	#NAME?
Ciclev10016997m.g	scaffold_2:9659616-9660567	0.204913	0	#NAME?
Ciclev10017006m.g	scaffold_2:9671428-9672843	2.02632	0	#NAME?
Ciclev10017779m.g	scaffold_2:9906804-9908265	0.180497	0	#NAME?
Ciclev10015113m.g	scaffold_2:10034772-10039656	0.121621	0	#NAME?
Ciclev10018240m.g	scaffold_2:10424778-10472483	2.47835	0	#NAME?
Ciclev10016797m.g	scaffold_2:10737295-10738854	4.53069	0	#NAME?
Ciclev10017452m.g	scaffold_2:11061826-11062240	5.21918	0	#NAME?
Ciclev10018346m.g	scaffold_2:11063249-11063663	1.21608	0	#NAME?
Ciclev10017576m.g	scaffold_2:11407537-11410870	0.102756	0	#NAME?
Ciclev10017239m.g	scaffold_2:11410968-11411710	0.345464	0	#NAME?
Ciclev10017691m.g	scaffold_2:11575182-11576163	0.232887	0	#NAME?
Ciclev10018390m.g	scaffold_2:11754293-11785844	0.488717	0	#NAME?
Ciclev10016468m.g	scaffold_2:12874615-12875806	0.469014	0	#NAME?
Ciclev10014509m.g	scaffold_2:13589254-13595952	0.16516	0	#NAME?
Ciclev10017288m.g	scaffold_2:13818909-13820177	0.544672	0	#NAME?
Ciclev10016947m.g	scaffold_2:13835287-13836116	9.08225	0	#NAME?
Ciclev10015317m.g	scaffold_2:13873572-13874934	0.311518	0	#NAME?
Ciclev10014358m.g	scaffold_2:13919661-13923501	0.382304	0	#NAME?
Ciclev10018388m.g	scaffold_2:14217252-14224407	2.11007	0	#NAME?
Ciclev10017340m.g	scaffold_2:16326053-16328552	0.269773	0	#NAME?
Ciclev10017406m.g	scaffold_2:16740246-16740758	0.522323	0	#NAME?
Ciclev10017555m.g	scaffold_2:16815930-16819481	0.30258	0	#NAME?
Ciclev10017068m.g	scaffold_2:16972863-16975595	0.939577	0	#NAME?
Ciclev10017315m.g	scaffold_2:19998730-19999449	0.332654	0	#NAME?

Ciclev10015305m.g	scaffold_2:21497411-21500211	0.155409	0	#NAME?
Ciclev10015544m.g	scaffold_2:22112814-22116763	0.212792	0	#NAME?
Ciclev10017550m.g	scaffold_2:22238685-22239075	0.950213	0	#NAME?
Ciclev10017119m.g	scaffold_2:23346121-23346773	1.13711	0	#NAME?
Ciclev10014495m.g	scaffold_2:23672336-23677032	0.159956	0	#NAME?
Ciclev10017883m.g	scaffold_2:23749777-23750980	0.182756	0	#NAME?
Ciclev10016961m.g	scaffold_2:23937651-23938164	3.58931	0	#NAME?
Ciclev10018016m.g	scaffold_2:23994300-23998585	0.268911	0	#NAME?
Ciclev10017742m.g	scaffold_2:24504361-24507645	0.377886	0	#NAME?
Ciclev10017488m.g	scaffold_2:24695460-24696387	0.207746	0	#NAME?
Ciclev10015033m.g	scaffold_2:24926665-24929114	0.149852	0	#NAME?
Ciclev10015171m.g	scaffold_2:27019344-27022514	2.4368	0	#NAME?
Ciclev10017061m.g	scaffold_2:27651567-27652231	4.88277	0	#NAME?
Ciclev10017342m.g	scaffold_2:27895402-27895826	0.82735	0	#NAME?
Ciclev10017676m.g	scaffold_2:28065770-28066808	0.869186	0	#NAME?
Ciclev10017575m.g	scaffold_2:28683489-28684109	3.88791	0	#NAME?
Ciclev10017934m.g	scaffold_2:28704879-28705287	0.71536	0	#NAME?
Ciclev10018307m.g	scaffold_2:29133267-29135449	0.251564	0	#NAME?
Ciclev10014061m.g	scaffold_2:29647105-29650955	0.251909	0	#NAME?
Ciclev10017714m.g	scaffold_2:30494005-30499118	0.849533	0	#NAME?
Ciclev10017495m.g	scaffold_2:31010943-31012807	1.24745	0	#NAME?
Ciclev10017587m.g	scaffold_2:31271384-31272035	1.81002	0	#NAME?
Ciclev10017741m.g	scaffold_2:31785535-31786453	2.80014	0	#NAME?
Ciclev10014970m.g	scaffold_2:32121968-32123805	2.32388	0	#NAME?
Ciclev10018014m.g	scaffold_2:32207429-32208005	0.85244	0	#NAME?
Ciclev10018056m.g	scaffold_2:32255284-32256094	1.42751	0	#NAME?
Ciclev10017033m.g	scaffold_2:33054090-33058053	1.03804	0	#NAME?
Ciclev10015724m.g	scaffold_2:33825839-33827631	0.377855	0	#NAME?
Ciclev10016801m.g	scaffold_2:33882291-33883520	1.04638	0	#NAME?
Ciclev10016441m.g	scaffold_2:34028656-34032642	0.357595	0	#NAME?
Ciclev10015361m.g	scaffold_2:34058699-34060913	0.891996	0	#NAME?
Ciclev10017947m.g	scaffold_2:34089641-34090860	0.416496	0	#NAME?
Ciclev10017869m.g	scaffold_2:34263775-34264495	0.530205	0	#NAME?
Ciclev10014718m.g	scaffold_2:34305190-34308435	1.44931	0	#NAME?
Ciclev10014155m.g	scaffold_2:34363430-34369047	17.0005	0	#NAME?
Ciclev10014740m.g	scaffold_2:34506213-34509604	0.831111	0	#NAME?
Ciclev10015686m.g	scaffold_2:34638803-34640718	0.845775	0	#NAME?
Ciclev10017977m.g	scaffold_2:34942462-34945203	0.0749635	0	#NAME?
Ciclev10017551m.g,Ciclev10018176m.g	scaffold_2:35054706-35069540	3.84062	0	#NAME?
Ciclev10017838m.g	scaffold_2:35148737-35150984	0.348399	0	#NAME?
Ciclev10018276m.g	scaffold_2:35183201-35184629	0.300975	0	#NAME?
Ciclev10015646m.g	scaffold_2:35188858-35190323	0.167781	0	#NAME?
Ciclev10018055m.g	scaffold_2:35382238-35384696	4.64991	0	#NAME?
Ciclev10017592m.g	scaffold_2:35429102-35438566	4.68762	0	#NAME?
Ciclev10015307m.g	scaffold_2:35478125-35479448	0.149396	0	#NAME?
Ciclev10017678m.g	scaffold_2:35594718-35599768	0.318267	0	#NAME?
Ciclev10015581m.g	scaffold_2:35642411-35646764	0.167992	0	#NAME?
Ciclev10017464m.g	scaffold_2:35922515-35923934	0.364979	0	#NAME?
Ciclev10017866m.g	scaffold_2:35964310-35966370	0.620716	0	#NAME?
Ciclev10017876m.g	scaffold_2:36005532-36007204	0.350717	0	#NAME?
Ciclev10017604m.g	scaffold_2:36235554-36237203	0.456497	0	#NAME?
-	scaffold_2:4895612-4986690	11.5881	0	#NAME?
-	scaffold_2:14294263-14294484	51.5534	0	#NAME?
-	scaffold_2:26287509-26287809	20.6649	0	#NAME?
Ciclev10013921m.g	scaffold_2193:541-2227	0.1161	0	#NAME?

Ciclev10004102m.g	scaffold_26:7739-8517	0.513705	0	#NAME?
Ciclev10004105m.g	scaffold_27:46053-47886	0.118574	0	#NAME?
Ciclev10023360m.g	scaffold_3:165206-166379	0.567635	0	#NAME?
Ciclev10021141m.g	scaffold_3:328702-330551	1.62838	0	#NAME?
Ciclev10019978m.g	scaffold_3:345990-348332	0.235554	0	#NAME?
Ciclev10020066m.g	scaffold_3:544248-547628	0.340076	0	#NAME?
Ciclev10023976m.g	scaffold_3:928478-929596	0.359673	0	#NAME?
Ciclev10021073m.g	scaffold_3:1172384-1173975	5.73809	0	#NAME?
Ciclev10022439m.g	scaffold_3:1281118-1281817	0.39659	0	#NAME?
Ciclev10021161m.g	scaffold_3:1520525-1522232	9.21593	0	#NAME?
Ciclev10022121m.g	scaffold_3:1563443-1564461	0.610186	0	#NAME?
Ciclev10024238m.g	scaffold_3:1824061-1825837	0.48785	0	#NAME?
Ciclev10020164m.g	scaffold_3:1888556-1892031	10.4072	0	#NAME?
Ciclev10024427m.g	scaffold_3:3197508-3198989	0.304051	0	#NAME?
Ciclev10022371m.g	scaffold_3:3483333-3485637	0.766617	0	#NAME?
Ciclev10024338m.g	scaffold_3:3520726-3522138	0.511021	0	#NAME?
Ciclev10019903m.g	scaffold_3:3757805-3760648	0.129694	0	#NAME?
Ciclev10023980m.g	scaffold_3:3772470-3775151	1.1217	0	#NAME?
Ciclev10018826m.g	scaffold_3:3900687-3905306	0.0663375	0	#NAME?
Ciclev10018967m.g	scaffold_3:4280664-4284103	0.602886	0	#NAME?
Ciclev10023697m.g	scaffold_3:4655628-4659852	0.112132	0	#NAME?
Ciclev10021884m.g	scaffold_3:4867450-4869045	0.913353	0	#NAME?
Ciclev10018727m.g	scaffold_3:5262190-5267863	0.0606651	0	#NAME?
Ciclev10021224m.g	scaffold_3:5572631-5573700	1.23495	0	#NAME?
Ciclev10023495m.g	scaffold_3:5674874-5678132	1.52044	0	#NAME?
Ciclev10021695m.g	scaffold_3:6140331-6142252	3.53956	0	#NAME?
Ciclev10019712m.g	scaffold_3:6163496-6165504	0.467004	0	#NAME?
Ciclev10021289m.g	scaffold_3:6594400-6595511	0.561259	0	#NAME?
Ciclev10023012m.g	scaffold_3:7235574-7236465	1.5721	0	#NAME?
Ciclev10022426m.g	scaffold_3:7318471-7319287	0.235751	0	#NAME?
Ciclev10018803m.g	scaffold_3:7494073-7496768	0.214565	0	#NAME?
Ciclev10022875m.g	scaffold_3:7665179-7665801	0.809943	0	#NAME?
Ciclev10022680m.g	scaffold_3:8528291-8529236	21.6502	0	#NAME?
Ciclev10024471m.g	scaffold_3:9004871-9005261	1.02005	0	#NAME?
Ciclev10023347m.g	scaffold_3:9012255-9012738	0.659907	0	#NAME?
Ciclev10024348m.g	scaffold_3:9411820-9414172	2.55945	0	#NAME?
Ciclev10023156m.g	scaffold_3:9414396-9419290	0.238435	0	#NAME?
Ciclev10023693m.g	scaffold_3:10065273-10065846	0.416838	0	#NAME?
Ciclev10018876m.g	scaffold_3:10121821-10124752	0.126779	0	#NAME?
Ciclev10024647m.g	scaffold_3:10126534-10126972	5.02396	0	#NAME?
Ciclev10018793m.g	scaffold_3:10250670-10255338	0.0607069	0	#NAME?
Ciclev10023995m.g	scaffold_3:10367410-10370786	0.0941359	0	#NAME?
Ciclev10024231m.g	scaffold_3:11061326-11063283	0.413368	0	#NAME?
Ciclev10019325m.g	scaffold_3:12698337-12703025	8.34368	0	#NAME?
Ciclev10023044m.g	scaffold_3:12913263-12914342	0.213118	0	#NAME?
Ciclev10024463m.g	scaffold_3:18609525-18610227	0.263336	0	#NAME?
Ciclev10023543m.g	scaffold_3:18659988-18663009	0.140503	0	#NAME?
Ciclev10023378m.g	scaffold_3:18728079-18730435	0.39371	0	#NAME?
Ciclev10023321m.g	scaffold_3:19310539-19608202	0.169252	0	#NAME?
Ciclev10020869m.g	scaffold_3:19310539-19608202	0.304352	0	#NAME?
Ciclev10021070m.g	scaffold_3:19310539-19608202	0.277232	0	#NAME?
Ciclev10024063m.g	scaffold_3:20816533-20817052	0.398579	0	#NAME?
Ciclev10023614m.g	scaffold_3:23094689-23097446	0.204655	0	#NAME?
Ciclev10023189m.g	scaffold_3:23717694-23718002	3.66184	0	#NAME?
Ciclev10021154m.g	scaffold_3:23734620-23736313	0.172846	0	#NAME?
Ciclev10024515m.g	scaffold_3:23750817-23752263	0.224585	0	#NAME?

Ciclev10023334m.g	scaffold_3:23987122-23991416	0.0648005	0	#NAME?
Ciclev10023819m.g	scaffold_3:24584261-24588809	0.123119	0	#NAME?
Ciclev10023525m.g	scaffold_3:25398285-25402615	0.0446429	0	#NAME?
Ciclev10023481m.g	scaffold_3:25577397-25580479	0.0565584	0	#NAME?
Ciclev10024280m.g	scaffold_3:32366452-32367235	0.406396	0	#NAME?
Ciclev10022152m.g	scaffold_3:32471567-32472547	0.280618	0	#NAME?
Ciclev10023725m.g	scaffold_3:32716415-32805364	0.926044	0	#NAME?
Ciclev10023243m.g	scaffold_3:32716415-32805364	1.25405	0	#NAME?
Ciclev10022056m.g	scaffold_3:32835188-32836309	3.02764	0	#NAME?
Ciclev10020934m.g	scaffold_3:32935330-32936414	1.23478	0	#NAME?
Ciclev10023127m.g	scaffold_3:34563399-34565913	0.3329	0	#NAME?
Ciclev10019504m.g	scaffold_3:34937386-34940238	0.125741	0	#NAME?
Ciclev10022222m.g	scaffold_3:35325266-35326970	0.40947	0	#NAME?
Ciclev10021753m.g	scaffold_3:36283014-36285414	0.0841055	0	#NAME?
Ciclev10024273m.g	scaffold_3:36334574-36335701	0.852183	0	#NAME?
Ciclev10023861m.g	scaffold_3:36782082-36782313	5.5757	0	#NAME?
Ciclev10023757m.g	scaffold_3:37116787-37117330	1.1042	0	#NAME?
Ciclev10018839m.g	scaffold_3:37156877-37163307	0.558324	0	#NAME?
Ciclev10022657m.g	scaffold_3:37169470-37170698	1.76123	0	#NAME?
Ciclev10023140m.g	scaffold_3:38153397-38153973	0.492256	0	#NAME?
Ciclev10024501m.g	scaffold_3:40009839-40010505	0.311523	0	#NAME?
Ciclev10023108m.g	scaffold_3:40176470-40209547	5.00116	0	#NAME?
Ciclev10020515m.g	scaffold_3:40426485-40429683	1.6652	0	#NAME?
Ciclev10021887m.g	scaffold_3:41067674-41229006	1.7027	0	#NAME?
Ciclev10024612m.g	scaffold_3:41374237-41376700	0.572065	0	#NAME?
Ciclev10022629m.g	scaffold_3:41598253-41598952	0.396566	0	#NAME?
Ciclev10022272m.g	scaffold_3:41601980-41602845	0.601919	0	#NAME?
Ciclev10023784m.g	scaffold_3:42307194-42307967	0.311279	0	#NAME?
Ciclev10023582m.g	scaffold_3:42542121-42545462	0.0723458	0	#NAME?
Ciclev10024004m.g	scaffold_3:42879643-42879874	4.33485	0	#NAME?
Ciclev10021908m.g	scaffold_3:42978992-42979928	0.318124	0	#NAME?
Ciclev10018639m.g	scaffold_3:42996885-43001710	30.7497	0	#NAME?
Ciclev10023447m.g	scaffold_3:43058605-43062372	0.855669	0	#NAME?
Ciclev10024170m.g	scaffold_3:43210423-43211500	0.241054	0	#NAME?
Ciclev10021312m.g	scaffold_3:43301683-43305338	4.65953	0	#NAME?
Ciclev10019720m.g	scaffold_3:43736679-43740546	0.107072	0	#NAME?
Ciclev10023105m.g	scaffold_3:43743925-43744777	0.357074	0	#NAME?
Ciclev10019087m.g	scaffold_3:44544846-44550637	0.0815256	0	#NAME?
Ciclev10023426m.g	scaffold_3:44665199-44667475	0.159018	0	#NAME?
Ciclev10024532m.g	scaffold_3:44826056-44832928	0.0875734	0	#NAME?
Ciclev10023684m.g	scaffold_3:44886488-44897661	0.395789	0	#NAME?
Ciclev10022778m.g	scaffold_3:45131583-45132737	4.99219	0	#NAME?
Ciclev10022084m.g	scaffold_3:45633804-45634659	0.992596	0	#NAME?
Ciclev10022952m.g	scaffold_3:46231514-46232388	0.288931	0	#NAME?
Ciclev10022416m.g	scaffold_3:46240095-46240980	5.13715	0	#NAME?
Ciclev10023268m.g	scaffold_3:46406510-46410608	0.0506861	0	#NAME?
Ciclev10020746m.g	scaffold_3:47364686-47366375	0.165388	0	#NAME?
Ciclev10022564m.g	scaffold_3:47387247-47388927	0.391565	0	#NAME?
Ciclev10024035m.g	scaffold_3:47654464-47655859	0.69694	0	#NAME?
Ciclev10019421m.g	scaffold_3:47676026-47679559	1.4182	0	#NAME?
Ciclev10023470m.g	scaffold_3:48069399-48074465	0.747434	0	#NAME?
Ciclev10022783m.g	scaffold_3:48201265-48202164	0.713764	0	#NAME?
Ciclev10019998m.g	scaffold_3:48769861-48771283	0.141095	0	#NAME?
Ciclev10024524m.g	scaffold_3:48982894-48983903	18.6744	0	#NAME?
Ciclev10023491m.g	scaffold_3:49115558-49116119	5.74162	0	#NAME?
Ciclev10020246m.g	scaffold_3:49410685-49412893	0.842976	0	#NAME?

Ciclev10024592m.g	scaffold_3:49845551-49849464	0.102336	0	#NAME?
Ciclev10024438m.g	scaffold_3:49865953-49866583	1.29193	0	#NAME?
Ciclev10021990m.g	scaffold_3:49966448-49968000	0.657913	0	#NAME?
Ciclev10020616m.g	scaffold_3:50790358-50791746	0.551928	0	#NAME?
Ciclev10023652m.g	scaffold_3:50806433-50806997	7.29205	0	#NAME?
Ciclev10020482m.g	scaffold_3:50878856-50881433	0.309816	0	#NAME?
Ciclev10019302m.g	scaffold_3:91759-94717	0.171474	0	#NAME?
Ciclev10023091m.g	scaffold_3:647381-648251	0.512217	0	#NAME?
Ciclev10021975m.g	scaffold_3:740048-742193	0.416094	0	#NAME?
Ciclev10023228m.g	scaffold_3:1220972-1221201	6.8498	0	#NAME?
Ciclev10019505m.g	scaffold_3:1786654-1788423	0.415895	0	#NAME?
Ciclev10024046m.g	scaffold_3:1914239-1914836	0.416906	0	#NAME?
Ciclev10023304m.g	scaffold_3:1952048-1952545	0.687176	0	#NAME?
Ciclev10023625m.g	scaffold_3:2145906-2147220	0.128449	0	#NAME?
Ciclev10020193m.g	scaffold_3:2263800-2265408	0.619877	0	#NAME?
Ciclev10020412m.g	scaffold_3:2764071-2765392	0.177028	0	#NAME?
Ciclev10018842m.g	scaffold_3:2900626-2904422	0.23716	0	#NAME?
Ciclev10019699m.g	scaffold_3:3726531-3728329	1.18002	0	#NAME?
Ciclev10020150m.g	scaffold_3:3784901-3787878	0.146126	0	#NAME?
Ciclev10024237m.g	scaffold_3:3840822-3841937	0.211447	0	#NAME?
Ciclev10023504m.g	scaffold_3:4015686-4016716	1.29851	0	#NAME?
Ciclev10023532m.g	scaffold_3:4767634-4768210	0.946234	0	#NAME?
Ciclev10018937m.g	scaffold_3:4907648-4913054	0.150495	0	#NAME?
Ciclev10022832m.g	scaffold_3:5837607-5838327	1.3679	0	#NAME?
Ciclev10024218m.g	scaffold_3:6020292-6023283	1.37382	0	#NAME?
Ciclev10023344m.g	scaffold_3:6084408-6085020	0.494796	0	#NAME?
Ciclev10020815m.g	scaffold_3:6569550-6570874	0.433689	0	#NAME?
Ciclev10023840m.g	scaffold_3:6837305-6837995	5.05764	0	#NAME?
Ciclev10024020m.g	scaffold_3:7056593-7057466	0.5523	0	#NAME?
Ciclev10020101m.g	scaffold_3:7680773-7684296	0.560071	0	#NAME?
Ciclev10024412m.g	scaffold_3:7928075-7929769	0.865697	0	#NAME?
Ciclev10024251m.g	scaffold_3:8179430-8185060	0.15125	0	#NAME?
Ciclev10023912m.g	scaffold_3:8414565-8421117	4.04347	0	#NAME?
Ciclev10018952m.g	scaffold_3:9052208-9056378	0.0663569	0	#NAME?
Ciclev10023659m.g	scaffold_3:12373976-12375919	0.19208	0	#NAME?
Ciclev10019529m.g	scaffold_3:13277453-13279946	0.0853668	0	#NAME?
Ciclev10024520m.g	scaffold_3:15950728-15954114	0.53572	0	#NAME?
Ciclev10018661m.g	scaffold_3:16295416-16299477	1.18688	0	#NAME?
Ciclev10024138m.g	scaffold_3:16685254-16687071	0.236124	0	#NAME?
Ciclev10024002m.g	scaffold_3:18522066-18524096	0.367612	0	#NAME?
Ciclev10023307m.g	scaffold_3:18612962-18618580	0.701181	0	#NAME?
Ciclev10023124m.g	scaffold_3:18891079-18891585	3.71414	0	#NAME?
Ciclev10024432m.g	scaffold_3:19310539-19608202	0.525526	0	#NAME?
Ciclev10018772m.g	scaffold_3:22476201-22480686	0.0715977	0	#NAME?
Ciclev10023637m.g	scaffold_3:22505970-22512141	0.0899759	0	#NAME?
Ciclev10024368m.g	scaffold_3:23205106-23207318	3.80273	0	#NAME?
Ciclev10019334m.g	scaffold_3:23346017-23349680	0.4544	0	#NAME?
Ciclev10021643m.g	scaffold_3:24629369-24734809	0.832576	0	#NAME?
Ciclev10023093m.g	scaffold_3:27210507-27242516	4.41666	0	#NAME?
Ciclev10022950m.g	scaffold_3:27726234-27797748	0.268166	0	#NAME?
Ciclev10022628m.g	scaffold_3:29948665-29949259	1.35097	0	#NAME?
Ciclev10024135m.g	scaffold_3:31127230-31130491	1.71048	0	#NAME?
Ciclev10023940m.g	scaffold_3:33153213-33153651	0.471805	0	#NAME?
Ciclev10018783m.g	scaffold_3:33388539-33592383	4.0726	0	#NAME?
Ciclev10023356m.g	scaffold_3:35890300-35892350	0.22669	0	#NAME?
Ciclev10024589m.g	scaffold_3:37868703-37880417	0.213899	0	#NAME?

Ciclev10024610m.g	scaffold_3:38008670-38012902	0.0515719	0	#NAME?
Ciclev10023306m.g	scaffold_3:40236707-40244926	1.90508	0	#NAME?
Ciclev10021209m.g	scaffold_3:42205628-42210257	1.91469	0	#NAME?
Ciclev10023425m.g	scaffold_3:42220155-42222744	4.21174	0	#NAME?
Ciclev10018776m.g	scaffold_3:42291471-42296255	1.43463	0	#NAME?
Ciclev10021356m.g	scaffold_3:42444808-42447119	6.7511	0	#NAME?
Ciclev10021437m.g	scaffold_3:42958144-42960351	0.685907	0	#NAME?
Ciclev10024066m.g	scaffold_3:43630475-43641127	0.0965957	0	#NAME?
Ciclev10023785m.g	scaffold_3:43982207-43982516	2.19364	0	#NAME?
Ciclev10023275m.g	scaffold_3:44012822-44014650	0.118743	0	#NAME?
Ciclev10024247m.g	scaffold_3:44113524-44114514	0.408994	0	#NAME?
-	scaffold_3:44416690-44417023	145.201	0	#NAME?
Ciclev10019118m.g	scaffold_3:44630857-44636267	0.520547	0	#NAME?
Ciclev10020857m.g	scaffold_3:44927934-44929235	0.359284	0	#NAME?
Ciclev10022737m.g	scaffold_3:45198152-45199507	0.590009	0	#NAME?
Ciclev10022826m.g	scaffold_3:45271739-45292172	0.270246	0	#NAME?
Ciclev10023420m.g	scaffold_3:45476720-45479172	0.387369	0	#NAME?
Ciclev10023880m.g	scaffold_3:45719347-45719656	3.25965	0	#NAME?
Ciclev10024241m.g	scaffold_3:46203504-46205788	0.0921352	0	#NAME?
Ciclev10023679m.g	scaffold_3:46490976-46492797	0.0986247	0	#NAME?
Ciclev10021487m.g	scaffold_3:46494951-46495855	0.752201	0	#NAME?
Ciclev10023884m.g	scaffold_3:46600039-46600512	1.62962	0	#NAME?
Ciclev10024556m.g	scaffold_3:46855732-46859065	0.622041	0	#NAME?
Ciclev10022784m.g	scaffold_3:46984553-46985820	0.269658	0	#NAME?
Ciclev10023961m.g	scaffold_3:47136226-47137387	1.93509	0	#NAME?
Ciclev10023395m.g	scaffold_3:47270666-47274045	0.113954	0	#NAME?
Ciclev10024641m.g	scaffold_3:47458436-47460269	0.191101	0	#NAME?
Ciclev10021586m.g	scaffold_3:47620773-47623158	0.130234	0	#NAME?
Ciclev10024292m.g	scaffold_3:47849782-47853144	1.87683	0	#NAME?
Ciclev10023643m.g	scaffold_3:47966199-47968775	0.12015	0	#NAME?
Ciclev10021837m.g	scaffold_3:48442655-48443670	4.1757	0	#NAME?
Ciclev10021269m.g	scaffold_3:48446076-48449604	0.77095	0	#NAME?
Ciclev10024550m.g	scaffold_3:48607677-48609120	0.401077	0	#NAME?
Ciclev10019219m.g	scaffold_3:49031440-49035556	0.430333	0	#NAME?
Ciclev10023281m.g	scaffold_3:49103622-49109291	3.87891	0	#NAME?
Ciclev10023951m.g	scaffold_3:49237730-49239023	0.489525	0	#NAME?
Ciclev10021992m.g	scaffold_3:49691192-49693851	0.473924	0	#NAME?
Ciclev10021687m.g	scaffold_3:49985665-49989341	0.1225	0	#NAME?
Ciclev10023530m.g	scaffold_3:50717743-50721841	6.79479	0	#NAME?
-	scaffold_3:28381335-28381949	5.42256	0	#NAME?
Ciclev10010866m.g	scaffold_32:27841-29782	0.0909944	0	#NAME?
Ciclev10010871m.g	scaffold_33:3630-6726	0.0722707	0	#NAME?
Ciclev10010867m.g	scaffold_33:55014-58169	0.102469	0	#NAME?
Ciclev10033544m.g	scaffold_4:418562-424467	0.292686	0	#NAME?
Ciclev10030839m.g	scaffold_4:490138-496519	0.0767008	0	#NAME?
Ciclev10031656m.g	scaffold_4:1591644-1639717	9.35764	0	#NAME?
Ciclev10032961m.g	scaffold_4:1845890-1847367	0.82149	0	#NAME?
Ciclev10031875m.g	scaffold_4:2086884-2106219	0.811074	0	#NAME?
Ciclev10033265m.g	scaffold_4:2443561-2444626	0.920112	0	#NAME?
Ciclev10032416m.g	scaffold_4:2542398-2543774	0.327863	0	#NAME?
Ciclev10033700m.g	scaffold_4:2688164-2688824	0.770476	0	#NAME?
Ciclev10033950m.g	scaffold_4:2748228-2748786	0.439338	0	#NAME?
Ciclev10033425m.g	scaffold_4:3338867-3340076	3.57806	0	#NAME?
Ciclev10032535m.g	scaffold_4:3650225-3653414	0.139648	0	#NAME?
Ciclev10031437m.g	scaffold_4:3930462-3934057	0.138584	0	#NAME?
Ciclev10033307m.g	scaffold_4:5043516-5044810	0.528799	0	#NAME?

Ciclev10032671m.g	scaffold_4:5287990-5290366	0.697261	0	#NAME?
Ciclev10033954m.g	scaffold_4:6555783-6557473	0.873586	0	#NAME?
Ciclev10033959m.g	scaffold_4:6816588-6817092	0.94861	0	#NAME?
Ciclev10033380m.g	scaffold_4:7114783-7115935	0.508042	0	#NAME?
Ciclev10033676m.g	scaffold_4:7273821-7276261	0.300813	0	#NAME?
Ciclev10033407m.g	scaffold_4:7903527-7904663	0.516921	0	#NAME?
Ciclev10032253m.g	scaffold_4:10443187-10444366	0.184162	0	#NAME?
Ciclev10033277m.g	scaffold_4:11201523-11202378	0.286355	0	#NAME?
Ciclev10033478m.g	scaffold_4:11303299-11304215	0.407136	0	#NAME?
Ciclev10033360m.g	scaffold_4:12318896-12320357	0.568878	0	#NAME?
Ciclev10033415m.g	scaffold_4:13082570-13083117	1.29906	0	#NAME?
Ciclev10031141m.g	scaffold_4:14017437-14019821	0.115446	0	#NAME?
Ciclev10032668m.g	scaffold_4:14466704-14467625	5.4295	0	#NAME?
Ciclev10033226m.g	scaffold_4:14516982-14524650	0.611959	0	#NAME?
Ciclev10033865m.g	scaffold_4:14692996-14694933	1.26427	0	#NAME?
Ciclev10032737m.g	scaffold_4:15314968-15316460	0.618743	0	#NAME?
Ciclev10033186m.g	scaffold_4:15528850-15538252	0.394817	0	#NAME?
Ciclev10032788m.g	scaffold_4:15544373-15546322	0.247837	0	#NAME?
Ciclev10033775m.g	scaffold_4:15882670-15884818	0.298329	0	#NAME?
Ciclev10032169m.g	scaffold_4:16176835-16178421	0.441541	0	#NAME?
Ciclev10030887m.g	scaffold_4:16193833-16197222	0.145492	0	#NAME?
Ciclev10032401m.g	scaffold_4:16500882-16502028	1.40392	0	#NAME?
Ciclev10030861m.g	scaffold_4:16526280-16530377	0.239845	0	#NAME?
Ciclev10033835m.g	scaffold_4:16767378-16768904	0.964381	0	#NAME?
Ciclev10033453m.g	scaffold_4:16817583-16984703	0.678005	0	#NAME?
Ciclev10033674m.g	scaffold_4:17546570-17547137	0.977167	0	#NAME?
Ciclev10033833m.g	scaffold_4:17739985-17740654	0.609848	0	#NAME?
Ciclev10032936m.g	scaffold_4:17993453-17995081	0.975514	0	#NAME?
Ciclev10033639m.g	scaffold_4:19775249-19776433	1.50758	0	#NAME?
Ciclev10031958m.g	scaffold_4:20324052-20325765	0.581658	0	#NAME?
Ciclev10032151m.g	scaffold_4:20344096-20346156	10.3265	0	#NAME?
Ciclev10031848m.g	scaffold_4:20414924-20418140	0.118794	0	#NAME?
Ciclev10033250m.g	scaffold_4:20464536-20464815	15.4002	0	#NAME?
Ciclev10033196m.g	scaffold_4:20856340-20862687	0.457919	0	#NAME?
Ciclev10033248m.g	scaffold_4:20921221-20922004	0.894753	0	#NAME?
Ciclev10033355m.g	scaffold_4:21416137-21416917	1.35489	0	#NAME?
Ciclev10033501m.g	scaffold_4:21644110-21650532	2.80339	0	#NAME?
Ciclev10033961m.g	scaffold_4:21709849-21710401	0.539566	0	#NAME?
Ciclev10033895m.g	scaffold_4:21806090-21808858	1.50298	0	#NAME?
Ciclev10031429m.g	scaffold_4:21857649-21859855	0.537178	0	#NAME?
Ciclev10033489m.g	scaffold_4:22103242-22106168	0.140322	0	#NAME?
Ciclev10032733m.g	scaffold_4:22108968-22109650	0.93043	0	#NAME?
Ciclev10031753m.g	scaffold_4:22133205-22135726	0.205466	0	#NAME?
Ciclev10032827m.g	scaffold_4:22648404-22649254	1.5246	0	#NAME?
Ciclev10032164m.g	scaffold_4:22801202-22802540	0.395471	0	#NAME?
Ciclev10033710m.g	scaffold_4:22984729-22985230	1.62634	0	#NAME?
Ciclev10033943m.g	scaffold_4:23183896-23184565	2.46998	0	#NAME?
Ciclev10031712m.g	scaffold_4:23916366-23918260	0.562969	0	#NAME?
Ciclev10033125m.g	scaffold_4:24171283-24172468	0.177572	0	#NAME?
Ciclev10033877m.g	scaffold_4:24445740-24445977	10.894	0	#NAME?
Ciclev10031481m.g	scaffold_4:24750936-24753262	1.30789	0	#NAME?
Ciclev10033208m.g	scaffold_4:25012967-25014256	0.189652	0	#NAME?
Ciclev10031896m.g	scaffold_4:25050646-25052168	1.08116	0	#NAME?
Ciclev10032944m.g	scaffold_4:25146798-25147305	6.56927	0	#NAME?
Ciclev10033424m.g	scaffold_4:25458348-25461128	0.0813299	0	#NAME?
Ciclev10033658m.g	scaffold_4:25535482-25537665	3.8747	0	#NAME?

Ciclev10031018m.g	scaffold_4:821566-824794	0.0929793	0	#NAME?
Ciclev10033597m.g	scaffold_4:1475714-1476242	1.13366	0	#NAME?
Ciclev10033691m.g	scaffold_4:1828099-1836003	0.13977	0	#NAME?
Ciclev10033224m.g	scaffold_4:1853214-1853769	1.32957	0	#NAME?
Ciclev10032684m.g	scaffold_4:1906152-1907054	4.36384	0	#NAME?
Ciclev10033210m.g	scaffold_4:2032720-2032975	3.90962	0	#NAME?
Ciclev10033685m.g	scaffold_4:2345493-2347587	0.135124	0	#NAME?
Ciclev10033927m.g	scaffold_4:3255082-3256594	0.395524	0	#NAME?
Ciclev10033022m.g	scaffold_4:3976739-3978807	1.43733	0	#NAME?
Ciclev10033944m.g	scaffold_4:4662803-4666508	0.0922045	0	#NAME?
Ciclev10033466m.g	scaffold_4:4825832-5025734	0.0879361	0	#NAME?
Ciclev10033735m.g	scaffold_4:4825832-5025734	0.168019	0	#NAME?
Ciclev10033773m.g	scaffold_4:6085518-6086965	0.196843	0	#NAME?
Ciclev10031240m.g	scaffold_4:6231491-6235447	0.0813568	0	#NAME?
Ciclev10031131m.g	scaffold_4:6505292-6511538	0.452887	0	#NAME?
Ciclev10033801m.g	scaffold_4:9168919-9170852	2.85326	0	#NAME?
Ciclev10031295m.g	scaffold_4:9442385-9444633	0.208431	0	#NAME?
Ciclev10031284m.g	scaffold_4:9614195-9617653	2.42661	0	#NAME?
Ciclev10033909m.g	scaffold_4:9880494-10159290	0.205379	0	#NAME?
Ciclev10033447m.g	scaffold_4:10341305-10341980	0.4532	0	#NAME?
Ciclev10031514m.g	scaffold_4:11273911-11277114	0.238911	0	#NAME?
Ciclev10033933m.g	scaffold_4:12228052-12228997	5.28453	0	#NAME?
Ciclev10033645m.g	scaffold_4:12921757-12923221	0.376958	0	#NAME?
Ciclev10033502m.g	scaffold_4:14405611-14406364	1.00391	0	#NAME?
Ciclev10033441m.g	scaffold_4:14953252-14954910	0.380471	0	#NAME?
Ciclev10033349m.g	scaffold_4:16150824-16151826	0.206538	0	#NAME?
Ciclev10033970m.g	scaffold_4:16476947-16477838	10.5137	0	#NAME?
Ciclev10033968m.g	scaffold_4:16589131-16590637	0.35158	0	#NAME?
Ciclev10033536m.g	scaffold_4:16817583-16984703	0.387525	0	#NAME?
Ciclev10033436m.g	scaffold_4:16817583-16984703	0.391497	0	#NAME?
Ciclev10033585m.g	scaffold_4:17390112-17396921	0.24119	0	#NAME?
Ciclev10031557m.g	scaffold_4:18230005-18231524	0.626959	0	#NAME?
Ciclev10033587m.g	scaffold_4:18675830-18676572	0.305697	0	#NAME?
Ciclev10033465m.g	scaffold_4:18816458-18817547	0.330343	0	#NAME?
Ciclev10032081m.g	scaffold_4:19102843-19104818	0.378092	0	#NAME?
Ciclev10031441m.g	scaffold_4:19530493-19535452	0.865833	0	#NAME?
Ciclev10032777m.g	scaffold_4:20167983-20168637	1.50988	0	#NAME?
Ciclev10033852m.g	scaffold_4:20249580-20249922	1.24822	0	#NAME?
Ciclev10031594m.g	scaffold_4:20309964-20312362	0.115646	0	#NAME?
Ciclev10033308m.g	scaffold_4:20509200-20510118	0.522711	0	#NAME?
Ciclev10033402m.g	scaffold_4:20799965-20802735	0.187916	0	#NAME?
Ciclev10032817m.g	scaffold_4:20867377-20868383	1.5492	0	#NAME?
Ciclev10030662m.g	scaffold_4:21577251-21583731	0.0688128	0	#NAME?
Ciclev10032277m.g	scaffold_4:21797819-21799196	0.276878	0	#NAME?
Ciclev10032926m.g	scaffold_4:22416477-22417324	1.00967	0	#NAME?
Ciclev10031899m.g	scaffold_4:22912614-22914628	0.284488	0	#NAME?
Ciclev10032973m.g	scaffold_4:22966590-22967646	3.44915	0	#NAME?
Ciclev10033115m.g	scaffold_4:23072591-23073840	0.640849	0	#NAME?
Ciclev10033182m.g	scaffold_4:23719284-23719932	1.85573	0	#NAME?
Ciclev10033317m.g	scaffold_4:24258914-24260129	0.403665	0	#NAME?
Ciclev10033712m.g	scaffold_4:24327472-24328664	0.86303	0	#NAME?
Ciclev10032019m.g	scaffold_4:24384807-24386261	0.213446	0	#NAME?
Ciclev10033772m.g	scaffold_4:25114864-25116550	0.11646	0	#NAME?
Ciclev10031320m.g	scaffold_4:25354556-25358068	63.5994	0	#NAME?
Ciclev10033633m.g	scaffold_4:25546206-25549020	0.170138	0	#NAME?
Ciclev10032915m.g	scaffold_4:25558417-25559382	1.42413	0	#NAME?

Ciclev10033941m.g	scaffold_4:25568040-25570063	0.481637	0	#NAME?
Ciclev10033998m.g	scaffold_4:25629279-25629850	51.0083	0	#NAME?
-	scaffold_5:306162-307366	10.8165	0	#NAME?
Ciclev10003026m.g	scaffold_5:307473-309522	26.347	0	#NAME?
Ciclev10003136m.g	scaffold_5:1208259-1209032	0.813025	0	#NAME?
Ciclev10002026m.g	scaffold_5:2485638-2492388	4.97509	0	#NAME?
Ciclev10003659m.g	scaffold_5:3491441-3493889	0.407304	0	#NAME?
Ciclev10003972m.g	scaffold_5:3513596-3515399	0.586299	0	#NAME?
Ciclev10003820m.g	scaffold_5:3595115-3597836	0.187939	0	#NAME?
Ciclev10002887m.g	scaffold_5:5011560-5011914	1.28861	0	#NAME?
Ciclev10003660m.g	scaffold_5:5849697-5853066	0.05294	0	#NAME?
Ciclev10003995m.g	scaffold_5:6437462-6438239	1.227	0	#NAME?
Ciclev10003742m.g	scaffold_5:6503935-6505075	0.527982	0	#NAME?
Ciclev10003075m.g	scaffold_5:6915929-6967656	9.26725	0	#NAME?
Ciclev10003263m.g	scaffold_5:7103212-7103545	32.7138	0	#NAME?
Ciclev10002632m.g	scaffold_5:7104382-7105206	12.5039	0	#NAME?
Ciclev10004059m.g	scaffold_5:10427531-10584700	1.40332	0	#NAME?
Ciclev10003400m.g	scaffold_5:10930095-10930422	4.70295	0	#NAME?
Ciclev10000420m.g	scaffold_5:15801848-15810954	1.77117	0	#NAME?
Ciclev10003379m.g	scaffold_5:17138599-17139583	3.63864	0	#NAME?
Ciclev10002726m.g	scaffold_5:17602752-17605413	0.15583	0	#NAME?
Ciclev10002969m.g	scaffold_5:17651458-17652769	0.742961	0	#NAME?
Ciclev10002949m.g	scaffold_5:18167624-18169065	20.2013	0	#NAME?
Ciclev10003954m.g	scaffold_5:18206356-18208321	0.115848	0	#NAME?
Ciclev10004018m.g	scaffold_5:19072974-19076466	0.160977	0	#NAME?
Ciclev10003475m.g	scaffold_5:21638465-21643506	1.08593	0	#NAME?
Ciclev10003922m.g	scaffold_5:25663254-25664623	11.0932	0	#NAME?
Ciclev10003041m.g	scaffold_5:26960451-26976490	0.349995	0	#NAME?
Ciclev10003490m.g	scaffold_5:28834667-28836149	0.28199	0	#NAME?
Ciclev10003886m.g	scaffold_5:29200844-29203401	0.668368	0	#NAME?
Ciclev10002569m.g	scaffold_5:29413142-29414562	2.09655	0	#NAME?
Ciclev10003728m.g	scaffold_5:30591414-30592370	1.01285	0	#NAME?
Ciclev10002663m.g	scaffold_5:31121804-31181813	154.839	0	#NAME?
-	scaffold_5:31233657-31263788	171.751	0	#NAME?
-	scaffold_5:31233657-31263788	1617.43	0	#NAME?
Ciclev10003572m.g	scaffold_5:31310526-31313935	0.400143	0	#NAME?
Ciclev10004032m.g	scaffold_5:31333200-31391489	6.40235	0	#NAME?
Ciclev10003974m.g	scaffold_5:31469286-31718393	2.68963	0	#NAME?
Ciclev10003944m.g	scaffold_5:32676117-32677436	0.608804	0	#NAME?
Ciclev10002075m.g	scaffold_5:32973613-32974814	0.383207	0	#NAME?
Ciclev10002897m.g	scaffold_5:33314344-33315010	0.354722	0	#NAME?
Ciclev10003399m.g	scaffold_5:33348110-33349960	0.12677	0	#NAME?
Ciclev10003628m.g	scaffold_5:33901361-33901730	1.0019	0	#NAME?
Ciclev10003448m.g	scaffold_5:34391102-34395047	0.157389	0	#NAME?
Ciclev10003860m.g	scaffold_5:34424623-34427092	0.0918899	0	#NAME?
Ciclev10002812m.g	scaffold_5:34470366-34471009	7.17586	0	#NAME?
Ciclev10002755m.g	scaffold_5:34488984-34490090	0.699474	0	#NAME?
Ciclev10002758m.g	scaffold_5:34682778-34684454	0.583886	0	#NAME?
Ciclev10002882m.g	scaffold_5:34791962-34793333	1.40243	0	#NAME?
Ciclev10001103m.g	scaffold_5:34826870-34830732	0.126135	0	#NAME?
Ciclev10002591m.g	scaffold_5:36026830-36027744	6.75692	0	#NAME?
Ciclev10003256m.g	scaffold_5:36371906-36378249	0.34161	0	#NAME?
Ciclev10003580m.g	scaffold_5:36901331-36902036	2.65143	0	#NAME?
Ciclev10001717m.g	scaffold_5:36927763-36929032	0.362922	0	#NAME?
Ciclev10003221m.g	scaffold_5:37211287-37212788	1.04136	0	#NAME?
Ciclev10002342m.g	scaffold_5:37293095-37294625	0.53521	0	#NAME?

Ciclev10003984m.g	scaffold_5:37659007-37663063	0.128769	0	#NAME?
Ciclev10000927m.g	scaffold_5:37664655-37668864	0.101411	0	#NAME?
Ciclev10003376m.g	scaffold_5:38011132-38012582	0.562148	0	#NAME?
Ciclev10003429m.g	scaffold_5:38095623-38100811	0.10426	0	#NAME?
Ciclev10003011m.g	scaffold_5:38496409-38497402	0.776212	0	#NAME?
Ciclev10002644m.g	scaffold_5:38780108-38781399	0.190752	0	#NAME?
Ciclev10003908m.g	scaffold_5:39145643-39146057	1.89389	0	#NAME?
Ciclev10003863m.g	scaffold_5:39400548-39401835	0.233911	0	#NAME?
Ciclev10000723m.g	scaffold_5:39740866-39742793	0.296276	0	#NAME?
Ciclev10000411m.g	scaffold_5:40046649-40048839	0.602615	0	#NAME?
Ciclev10000554m.g	scaffold_5:40196231-40199730	0.488985	0	#NAME?
Ciclev10003727m.g	scaffold_5:40306193-40306532	2.53242	0	#NAME?
Ciclev10002239m.g	scaffold_5:40449646-40450911	5.71798	0	#NAME?
Ciclev10000579m.g	scaffold_5:41100158-41102051	0.213105	0	#NAME?
Ciclev10003104m.g	scaffold_5:41252803-41257611	0.196386	0	#NAME?
Ciclev10002280m.g	scaffold_5:41461922-41463141	0.15188	0	#NAME?
Ciclev10003275m.g	scaffold_5:41643985-41645593	3.59322	0	#NAME?
Ciclev10001255m.g	scaffold_5:41881296-41883386	1.24108	0	#NAME?
Ciclev10003489m.g	scaffold_5:42135435-42136408	0.239948	0	#NAME?
Ciclev10003990m.g	scaffold_5:42234741-42235981	0.186704	0	#NAME?
Ciclev10004092m.g	scaffold_5:42928371-42928783	0.947099	0	#NAME?
Ciclev10003316m.g	scaffold_5:279257-280613	0.284104	0	#NAME?
Ciclev10002337m.g	scaffold_5:282609-284150	1.16061	0	#NAME?
-	scaffold_5:914285-943659	1523.76	0	#NAME?
Ciclev10002953m.g	scaffold_5:1364434-1365129	1.59541	0	#NAME?
Ciclev10002695m.g	scaffold_5:1481991-1482664	0.700945	0	#NAME?
Ciclev10003166m.g	scaffold_5:1552172-1984270	0.255363	0	#NAME?
Ciclev10002849m.g	scaffold_5:2800167-2804104	0.286107	0	#NAME?
Ciclev10000354m.g	scaffold_5:4471259-4568404	0.0734177	0	#NAME?
Ciclev10003545m.g	scaffold_5:4757742-4759034	0.295029	0	#NAME?
Ciclev10002941m.g	scaffold_5:4913989-4914545	0.816179	0	#NAME?
Ciclev10003819m.g	scaffold_5:5260411-5262567	3.44723	0	#NAME?
Ciclev10003110m.g	scaffold_5:6103258-6105114	0.928733	0	#NAME?
Ciclev10004077m.g	scaffold_5:6777321-6914637	3.09763	0	#NAME?
Ciclev10003544m.g	scaffold_5:6777321-6914637	7.43593	0	#NAME?
Ciclev10000860m.g	scaffold_5:7063960-7066280	0.842216	0	#NAME?
-	scaffold_5:9713649-9935604	5.59803	0	#NAME?
Ciclev10003941m.g	scaffold_5:10011300-10012215	0.255733	0	#NAME?
Ciclev10003052m.g	scaffold_5:13299944-13300363	0.593269	0	#NAME?
Ciclev10003558m.g	scaffold_5:16632479-16633124	0.309537	0	#NAME?
Ciclev10003205m.g	scaffold_5:16870168-16871366	1.01928	0	#NAME?
Ciclev10003686m.g	scaffold_5:17629251-17630388	0.581666	0	#NAME?
Ciclev10003331m.g	scaffold_5:17654268-17654919	2.06691	0	#NAME?
Ciclev10002604m.g	scaffold_5:18364742-18365569	0.310114	0	#NAME?
Ciclev10003780m.g	scaffold_5:18396019-18396994	0.419543	0	#NAME?
Ciclev10003438m.g	scaffold_5:19746687-19749407	0.0981785	0	#NAME?
Ciclev10002946m.g	scaffold_5:20865254-20886617	0.675874	0	#NAME?
Ciclev10003586m.g	scaffold_5:21554209-21554959	0.772522	0	#NAME?
Ciclev10003932m.g	scaffold_5:22227782-22263681	1.91123	0	#NAME?
Ciclev10002596m.g	scaffold_5:22718835-22719826	0.63588	0	#NAME?
Ciclev10004049m.g	scaffold_5:22919338-23141855	0.174769	0	#NAME?
Ciclev10003283m.g	scaffold_5:22919338-23141855	1.23692	0	#NAME?
Ciclev10000136m.g	scaffold_5:23199924-23446011	183.654	0	#NAME?
Ciclev10000151m.g	scaffold_5:23199924-23446011	0.113554	0	#NAME?
Ciclev10001992m.g	scaffold_5:24483477-24484660	1.05795	0	#NAME?
Ciclev10003759m.g	scaffold_5:24926144-25131690	0.0598403	0	#NAME?

Ciclev10003135m.g	scaffold_5:25707436-25711484	0.0689485	0	#NAME?
Ciclev10003404m.g	scaffold_5:25832875-25852629	0.16336	0	#NAME?
-	scaffold_5:26380563-26382328	5.08536	0	#NAME?
Ciclev10001934m.g	scaffold_5:27153993-27156141	0.160233	0	#NAME?
Ciclev10001916m.g	scaffold_5:27161365-27163409	0.179111	0	#NAME?
Ciclev10003126m.g	scaffold_5:28289811-28536377	2.07543	0	#NAME?
Ciclev10003736m.g	scaffold_5:28822355-28823684	0.160366	0	#NAME?
Ciclev10003706m.g	scaffold_5:29816033-29816666	2.56089	0	#NAME?
Ciclev10003228m.g	scaffold_5:29898786-29900442	0.195628	0	#NAME?
Ciclev10000327m.g	scaffold_5:29917519-29920233	0.0593195	0	#NAME?
Ciclev10000323m.g	scaffold_5:30043537-30045934	0.0666937	0	#NAME?
Ciclev10000315m.g	scaffold_5:30111178-30113590	0.0629818	0	#NAME?
Ciclev10003453m.g	scaffold_5:30445359-30448160	0.0595116	0	#NAME?
Ciclev10003811m.g	scaffold_5:32266175-32290730	0.0646605	0	#NAME?
Ciclev10000664m.g	scaffold_5:32783456-32786240	0.106048	0	#NAME?
Ciclev10001510m.g	scaffold_5:33079707-33163140	0.537289	0	#NAME?
Ciclev10000203m.g	scaffold_5:33276866-33279877	0.0690434	0	#NAME?
Ciclev10001944m.g	scaffold_5:33617068-33620365	1.31224	0	#NAME?
Ciclev10003641m.g	scaffold_5:33692662-33694653	0.606223	0	#NAME?
Ciclev10003837m.g	scaffold_5:34133489-34136917	0.168955	0	#NAME?
Ciclev10001544m.g	scaffold_5:34321003-34323724	0.0848472	0	#NAME?
Ciclev10001560m.g	scaffold_5:34684729-34686032	0.299929	0	#NAME?
Ciclev10003617m.g	scaffold_5:35554388-35557671	0.294094	0	#NAME?
Ciclev10001098m.g	scaffold_5:36013317-36015037	5.34142	0	#NAME?
Ciclev10002069m.g	scaffold_5:37100833-37102074	0.362578	0	#NAME?
Ciclev10000466m.g	scaffold_5:37349892-37356610	5.55936	0	#NAME?
Ciclev10002384m.g	scaffold_5:38153461-38154253	0.328475	0	#NAME?
Ciclev10001803m.g	scaffold_5:38165020-38166215	0.55394	0	#NAME?
Ciclev10003445m.g	scaffold_5:38224900-38225522	0.47706	0	#NAME?
Ciclev10000794m.g	scaffold_5:38323685-38328788	0.988773	0	#NAME?
Ciclev10002282m.g	scaffold_5:38388921-38389958	0.296909	0	#NAME?
Ciclev10003821m.g	scaffold_5:38563225-38565511	1.03465	0	#NAME?
Ciclev10003945m.g	scaffold_5:38735535-38737052	0.205392	0	#NAME?
Ciclev10003530m.g	scaffold_5:39158906-39161971	0.331251	0	#NAME?
Ciclev10003091m.g	scaffold_5:39466847-39468398	1.56127	0	#NAME?
Ciclev10002926m.g	scaffold_5:39809408-39809996	0.440384	0	#NAME?
Ciclev10000658m.g	scaffold_5:40214034-40216955	4.37689	0	#NAME?
Ciclev10002762m.g	scaffold_5:41161957-41162677	0.964648	0	#NAME?
Ciclev10002504m.g	scaffold_5:41890162-41891434	0.939492	0	#NAME?
Ciclev10001877m.g	scaffold_5:42240496-42242058	1.68053	0	#NAME?
Ciclev10003183m.g	scaffold_5:42484752-42488408	0.137423	0	#NAME?
Ciclev10003575m.g	scaffold_5:42511699-42512119	0.731034	0	#NAME?
Ciclev10003381m.g	scaffold_5:42565118-42566009	6.75178	0	#NAME?
Ciclev10003823m.g	scaffold_5:42638005-42640532	0.128655	0	#NAME?
-	scaffold_5:323149-323337	274.172	0	#NAME?
-	scaffold_5:4076819-4284428	74.7068	0	#NAME?
-	scaffold_5:11517889-11518315	11.8465	0	#NAME?
-	scaffold_5:22919338-23141855	56.3587	0	#NAME?
-	scaffold_5:23199924-23446011	170.469	0	#NAME?
-	scaffold_5:23446200-23447029	7.03871	0	#NAME?
-	scaffold_5:23629757-23630395	3.147	0	#NAME?
-	scaffold_5:27993382-27993665	43.1012	0	#NAME?
-	scaffold_5:28289811-28536377	7.72749	0	#NAME?
-	scaffold_5:30106549-30106849	37.5725	0	#NAME?
Ciclev10013648m.g	scaffold_6:36987-39398	0.829485	0	#NAME?
Ciclev10013349m.g	scaffold_6:704501-707618	0.0698315	0	#NAME?

Ciclev10013444m.g	scaffold_6:6311396-6313802	0.958364	0	#NAME?
Ciclev10013740m.g	scaffold_6:6438430-6440459	0.143903	0	#NAME?
Ciclev10012906m.g	scaffold_6:7809151-7810755	16.356	0	#NAME?
Ciclev10013463m.g	scaffold_6:7886947-7888689	0.601753	0	#NAME?
Ciclev10011671m.g	scaffold_6:8543518-8546262	0.147307	0	#NAME?
Ciclev10012995m.g	scaffold_6:8696693-8697639	154.265	0	#NAME?
Ciclev10013498m.g	scaffold_6:9114995-9121942	0.123673	0	#NAME?
Ciclev10013753m.g	scaffold_6:9552627-9554799	0.289349	0	#NAME?
Ciclev10012588m.g	scaffold_6:10597544-10598514	42.2642	0	#NAME?
Ciclev10012036m.g	scaffold_6:11374941-11377354	15.8514	0	#NAME?
Ciclev10013284m.g	scaffold_6:11530918-11531156	20.1229	0	#NAME?
Ciclev10013573m.g	scaffold_6:11554083-11561079	0.0816639	0	#NAME?
-	scaffold_6:12303696-12304227	13.6699	0	#NAME?
Ciclev10012982m.g	scaffold_6:12558710-12559233	2.74943	0	#NAME?
Ciclev10011363m.g	scaffold_6:14351024-14353403	1.57958	0	#NAME?
Ciclev10013212m.g	scaffold_6:14721969-14722645	12.1595	0	#NAME?
Ciclev10013699m.g	scaffold_6:15049007-15049757	0.7188	0	#NAME?
Ciclev10013493m.g	scaffold_6:15143660-15145518	0.416108	0	#NAME?
Ciclev10013757m.g	scaffold_6:16065631-16068788	0.145383	0	#NAME?
Ciclev10013453m.g	scaffold_6:16421923-16423174	1.98649	0	#NAME?
Ciclev10011779m.g	scaffold_6:16579537-16581740	8.13103	0	#NAME?
Ciclev10012687m.g	scaffold_6:16780133-16781815	1.30618	0	#NAME?
Ciclev10013527m.g	scaffold_6:16946899-16949118	0.46157	0	#NAME?
Ciclev10012677m.g	scaffold_6:17239221-17285298	1.0087	0	#NAME?
Ciclev10011631m.g	scaffold_6:17335733-17338606	0.665927	0	#NAME?
Ciclev10012953m.g	scaffold_6:17547279-17548067	5.32142	0	#NAME?
Ciclev10011664m.g	scaffold_6:17593686-17595531	2.00609	0	#NAME?
Ciclev10013471m.g	scaffold_6:17790299-17791542	0.568693	0	#NAME?
Ciclev10013885m.g	scaffold_6:18176839-18178519	0.44055	0	#NAME?
Ciclev10013369m.g	scaffold_6:18194883-18202571	2.02025	0	#NAME?
Ciclev10011953m.g	scaffold_6:18457554-18460527	1.69658	0	#NAME?
Ciclev10013846m.g	scaffold_6:18512663-18513350	0.479666	0	#NAME?
Ciclev10013331m.g	scaffold_6:19216449-19217181	28.3488	0	#NAME?
Ciclev10013719m.g	scaffold_6:19231406-19231961	0.777643	0	#NAME?
Ciclev10011699m.g	scaffold_6:19280478-19282066	0.252956	0	#NAME?
Ciclev10011369m.g	scaffold_6:19558581-19562546	0.592625	0	#NAME?
Ciclev10011594m.g	scaffold_6:20677175-20680157	2.41226	0	#NAME?
Ciclev10012029m.g	scaffold_6:20749660-20751257	1.19808	0	#NAME?
Ciclev10012205m.g	scaffold_6:21508917-21511131	0.247082	0	#NAME?
Ciclev10012209m.g	scaffold_6:21549744-21550875	3.94235	0	#NAME?
Ciclev10011391m.g	scaffold_6:21777513-21779251	0.101098	0	#NAME?
Ciclev10012428m.g	scaffold_6:21803726-21804560	0.324625	0	#NAME?
Ciclev10013196m.g	scaffold_6:22048077-22048926	0.374584	0	#NAME?
Ciclev10013649m.g	scaffold_6:22516127-22518028	0.32694	0	#NAME?
Ciclev10013229m.g	scaffold_6:22529561-22529879	4.67327	0	#NAME?
Ciclev10013745m.g	scaffold_6:22788794-22790878	0.330908	0	#NAME?
Ciclev10013644m.g	scaffold_6:23035940-23039269	0.376935	0	#NAME?
Ciclev10013788m.g	scaffold_6:23566889-23567233	8.88189	0	#NAME?
Ciclev10013793m.g	scaffold_6:23910053-23910693	1.30594	0	#NAME?
Ciclev10012489m.g	scaffold_6:24398375-24399529	3.51794	0	#NAME?
Ciclev10013370m.g	scaffold_6:24413006-24414461	0.118368	0	#NAME?
Ciclev10013898m.g	scaffold_6:24691579-24696010	0.074601	0	#NAME?
Ciclev10013552m.g	scaffold_6:24702104-24704504	0.129261	0	#NAME?
Ciclev10013209m.g	scaffold_6:24791116-24792456	0.410673	0	#NAME?
Ciclev10013598m.g	scaffold_6:24863293-24865581	0.0891338	0	#NAME?
Ciclev10013454m.g	scaffold_6:24913374-24914177	1.43416	0	#NAME?

Ciclev10013670m.g	scaffold_6:25265420-25266769	0.810741	0	#NAME?
Ciclev10012903m.g	scaffold_6:25303946-25305475	0.283377	0	#NAME?
Ciclev10013417m.g	scaffold_6:25317493-25320442	0.0794664	0	#NAME?
Ciclev10013137m.g	scaffold_6:25466925-25469211	2.73779	0	#NAME?
Ciclev10012969m.g	scaffold_6:25562966-25563826	5.91939	0	#NAME?
Ciclev10013545m.g	scaffold_6:25577568-25578881	0.495224	0	#NAME?
Ciclev10013594m.g	scaffold_6:1459170-1462546	0.0876358	0	#NAME?
Ciclev10012353m.g	scaffold_6:4823522-4825584	0.20619	0	#NAME?
Ciclev10011981m.g	scaffold_6:5407449-5408659	0.154748	0	#NAME?
Ciclev10013671m.g	scaffold_6:5951659-5977928	0.0973959	0	#NAME?
Ciclev10013193m.g	scaffold_6:9855742-9856320	5.79434	0	#NAME?
Ciclev10013881m.g	scaffold_6:11499112-11501221	0.0992725	0	#NAME?
Ciclev10011234m.g	scaffold_6:12362571-12364596	0.531285	0	#NAME?
Ciclev10013809m.g	scaffold_6:12694281-12697607	0.20094	0	#NAME?
Ciclev10013343m.g	scaffold_6:12809435-12811692	0.224192	0	#NAME?
Ciclev10013802m.g	scaffold_6:13451896-13492783	1.03242	0	#NAME?
Ciclev10011362m.g	scaffold_6:14379908-14382609	1.4316	0	#NAME?
Ciclev10013132m.g	scaffold_6:15164057-15164782	8.82501	0	#NAME?
Ciclev10013135m.g	scaffold_6:15167311-15168695	31.1423	0	#NAME?
Ciclev10013520m.g	scaffold_6:15222408-15223143	0.596134	0	#NAME?
Ciclev10013537m.g	scaffold_6:16084537-16087479	0.170167	0	#NAME?
Ciclev10013451m.g	scaffold_6:16893382-16896109	0.560788	0	#NAME?
Ciclev10013329m.g	scaffold_6:17969526-17970856	1.50464	0	#NAME?
Ciclev10013178m.g	scaffold_6:18795762-18796153	1.71567	0	#NAME?
Ciclev10012978m.g	scaffold_6:18881311-18882154	6.54359	0	#NAME?
Ciclev10012576m.g	scaffold_6:19469489-19471685	0.323842	0	#NAME?
Ciclev10013876m.g	scaffold_6:19722524-19723865	0.130915	0	#NAME?
Ciclev10013351m.g	scaffold_6:20167713-20169179	0.269037	0	#NAME?
Ciclev10013701m.g	scaffold_6:20333618-20338112	0.0959764	0	#NAME?
Ciclev10013717m.g	scaffold_6:20649510-20649897	2.87104	0	#NAME?
Ciclev10011455m.g	scaffold_6:21150415-21155246	1.12301	0	#NAME?
Ciclev10011287m.g	scaffold_6:21330016-21334043	0.155171	0	#NAME?
Ciclev10011293m.g	scaffold_6:21421140-21424496	0.687597	0	#NAME?
Ciclev10012137m.g	scaffold_6:22226252-22227966	0.159132	0	#NAME?
Ciclev10012358m.g	scaffold_6:22349583-22351196	1.47201	0	#NAME?
Ciclev10013581m.g	scaffold_6:22636610-22637327	1.66472	0	#NAME?
Ciclev10013897m.g	scaffold_6:22912745-22914438	1.1198	0	#NAME?
Ciclev10013668m.g	scaffold_6:22918339-22918773	0.64008	0	#NAME?
Ciclev10012528m.g	scaffold_6:23366802-23368271	0.805644	0	#NAME?
Ciclev10012300m.g	scaffold_6:23376591-23378582	0.0982922	0	#NAME?
Ciclev10012869m.g	scaffold_6:24261073-24262645	0.683484	0	#NAME?
Ciclev10012771m.g	scaffold_6:24350158-24350821	0.318273	0	#NAME?
Ciclev10013642m.g	scaffold_6:24366000-24367444	1.16895	0	#NAME?
Ciclev10013340m.g	scaffold_6:24380153-24381442	0.741564	0	#NAME?
Ciclev10013418m.g	scaffold_6:24673772-24677434	0.11307	0	#NAME?
Ciclev10012730m.g	scaffold_6:24915881-24918093	0.190787	0	#NAME?
Ciclev10011967m.g	scaffold_6:25020995-25022851	0.16583	0	#NAME?
-	scaffold_6:8931920-8932124	181.996	0	#NAME?
-	scaffold_6:14714266-14714448	560.622	0	#NAME?
-	scaffold_6:14885892-14886036	2290.26	0	#NAME?
Ciclev10027531m.g	scaffold_7:261366-263946	0.462869	0	#NAME?
Ciclev10027360m.g	scaffold_7:376784-377693	0.6938	0	#NAME?
Ciclev10026923m.g	scaffold_7:483920-485556	0.324013	0	#NAME?
Ciclev10025531m.g	scaffold_7:558533-561202	0.248222	0	#NAME?
Ciclev10026929m.g	scaffold_7:713305-717895	0.0730514	0	#NAME?
Ciclev10027345m.g	scaffold_7:887175-889016	0.395821	0	#NAME?

Ciclev10027011m.g	scaffold_7:1665396-1665948	1.34718	0	#NAME?
Ciclev10027293m.g	scaffold_7:1867572-1868472	0.870322	0	#NAME?
Ciclev10026557m.g	scaffold_7:2881143-2882746	0.592321	0	#NAME?
Ciclev10025796m.g	scaffold_7:3514481-3515863	26.7479	0	#NAME?
Ciclev10026765m.g	scaffold_7:4545731-4546422	0.992471	0	#NAME?
Ciclev10026941m.g	scaffold_7:4837060-4839737	0.0825761	0	#NAME?
Ciclev10026783m.g	scaffold_7:4892605-4893281	0.686303	0	#NAME?
-	scaffold_7:5283909-5285275	16.4809	0	#NAME?
Ciclev10026995m.g	scaffold_7:5370300-5374200	0.394702	0	#NAME?
Ciclev10026131m.g	scaffold_7:5430764-5432498	0.328213	0	#NAME?
Ciclev10025064m.g	scaffold_7:5579839-5582692	0.08716	0	#NAME?
Ciclev10027116m.g	scaffold_7:5744590-5745610	0.294754	0	#NAME?
Ciclev10027519m.g	scaffold_7:5784646-5786383	0.198525	0	#NAME?
Ciclev10027284m.g	scaffold_7:6084603-6087800	2.08384	0	#NAME?
Ciclev10026773m.g	scaffold_7:6724442-6725666	1.05486	0	#NAME?
Ciclev10027624m.g	scaffold_7:7038918-7044450	0.359487	0	#NAME?
Ciclev10025181m.g	scaffold_7:7063425-7067137	0.086044	0	#NAME?
Ciclev10025595m.g	scaffold_7:7169014-7170817	0.829721	0	#NAME?
Ciclev10027206m.g	scaffold_7:7452136-7454161	0.677531	0	#NAME?
Ciclev10027015m.g	scaffold_7:7950587-7951187	0.408681	0	#NAME?
Ciclev10026959m.g	scaffold_7:8530315-8532413	0.104212	0	#NAME?
Ciclev10026617m.g	scaffold_7:8756797-8757937	1.15103	0	#NAME?
Ciclev10026471m.g	scaffold_7:9023226-9024094	0.672486	0	#NAME?
Ciclev10026796m.g	scaffold_7:9237720-9238164	1.9504	0	#NAME?
Ciclev10024865m.g	scaffold_7:10515554-10518467	0.177403	0	#NAME?
Ciclev10027198m.g	scaffold_7:10532977-10535623	0.247767	0	#NAME?
Ciclev10027091m.g	scaffold_7:11552867-11555818	0.355685	0	#NAME?
Ciclev10026439m.g	scaffold_7:12890747-12891580	0.252149	0	#NAME?
Ciclev10025126m.g	scaffold_7:13678024-13680322	4.5996	0	#NAME?
Ciclev10024817m.g	scaffold_7:13824420-13827222	0.0601291	0	#NAME?
Ciclev10027287m.g	scaffold_7:14569807-14570444	0.423448	0	#NAME?
Ciclev10025431m.g	scaffold_7:14975308-14977096	0.121202	0	#NAME?
Ciclev10024854m.g	scaffold_7:15405817-15408687	0.0562778	0	#NAME?
Ciclev10027033m.g	scaffold_7:15876223-15877623	1.26519	0	#NAME?
Ciclev10027477m.g	scaffold_7:15892914-15893382	1.48187	0	#NAME?
Ciclev10027291m.g	scaffold_7:15994987-15999622	0.410075	0	#NAME?
Ciclev10027067m.g	scaffold_7:16741930-16744020	4.06945	0	#NAME?
Ciclev10027387m.g	scaffold_7:16871439-16873712	0.112288	0	#NAME?
Ciclev10024978m.g	scaffold_7:16930006-16932528	0.375418	0	#NAME?
Ciclev10027263m.g	scaffold_7:18431876-18434849	0.259287	0	#NAME?
Ciclev10027199m.g	scaffold_7:19476881-19477856	0.242771	0	#NAME?
Ciclev10026247m.g	scaffold_7:20305386-20307351	0.314703	0	#NAME?
Ciclev10027432m.g	scaffold_7:20987963-20988302	9.75562	0	#NAME?
Ciclev10027145m.g	scaffold_7:646321-646750	7.32796	0	#NAME?
Ciclev10025182m.g	scaffold_7:730872-734557	0.452744	0	#NAME?
Ciclev10026054m.g	scaffold_7:1674666-1676101	2.66457	0	#NAME?
Ciclev10027136m.g	scaffold_7:1772383-1772894	1.58648	0	#NAME?
Ciclev10026228m.g	scaffold_7:2009430-2010279	1.71952	0	#NAME?
Ciclev10025821m.g	scaffold_7:2281201-2285659	0.468891	0	#NAME?
Ciclev10026624m.g	scaffold_7:2564081-2565016	0.263661	0	#NAME?
Ciclev10026968m.g	scaffold_7:2577347-2578182	0.354766	0	#NAME?
Ciclev10027038m.g	scaffold_7:2601466-2601700	2.77725	0	#NAME?
Ciclev10026965m.g	scaffold_7:3242848-3244847	0.133256	0	#NAME?
Ciclev10025537m.g	scaffold_7:3245923-3249254	0.936882	0	#NAME?
Ciclev10026646m.g	scaffold_7:3825287-3830771	0.566762	0	#NAME?
Ciclev10025339m.g	scaffold_7:5078111-5082133	0.108849	0	#NAME?

Ciclev10025623m.g	scaffold_7:6047990-6049508	8.3488	0	#NAME?
Ciclev10026976m.g	scaffold_7:6066056-6067392	0.174725	0	#NAME?
Ciclev10026136m.g	scaffold_7:6084603-6087800	1.62702	0	#NAME?
Ciclev10027521m.g	scaffold_7:6151858-6153347	1.07679	0	#NAME?
Ciclev10026135m.g	scaffold_7:7142294-7143997	0.234918	0	#NAME?
Ciclev10027460m.g	scaffold_7:7495821-7498037	0.313837	0	#NAME?
Ciclev10025568m.g	scaffold_7:7517438-7641496	0.100692	0	#NAME?
Ciclev10025604m.g	scaffold_7:7641939-7644738	0.12933	0	#NAME?
Ciclev10027341m.g	scaffold_7:8900385-8902865	0.0331105	0	#NAME?
Ciclev10025449m.g	scaffold_7:9024844-9028598	0.147724	0	#NAME?
Ciclev10024859m.g	scaffold_7:9924266-9927481	1.52746	0	#NAME?
Ciclev10027545m.g	scaffold_7:10246752-10248102	0.217251	0	#NAME?
Ciclev10026043m.g	scaffold_7:10927499-10930373	0.322732	0	#NAME?
Ciclev10027437m.g	scaffold_7:11163358-11167847	1.56653	0	#NAME?
Ciclev10027305m.g	scaffold_7:11244642-11245338	0.75158	0	#NAME?
Ciclev10027428m.g	scaffold_7:11604463-11607028	0.0672231	0	#NAME?
Ciclev10027054m.g	scaffold_7:11850299-12160617	2.88125	0	#NAME?
Ciclev10027064m.g	scaffold_7:11850299-12160617	1.0481	0	#NAME?
Ciclev10027426m.g	scaffold_7:13381507-13384450	1.38046	0	#NAME?
Ciclev10026952m.g	scaffold_7:13775244-13775643	0.819592	0	#NAME?
Ciclev10025378m.g	scaffold_7:14802049-14804801	0.102853	0	#NAME?
Ciclev10027532m.g	scaffold_7:15135435-15138904	0.118316	0	#NAME?
Ciclev10027461m.g	scaffold_7:15313218-15313473	8.19115	0	#NAME?
Ciclev10027371m.g	scaffold_7:15689334-15694745	7.00338	0	#NAME?
Ciclev10026487m.g	scaffold_7:17166934-17167692	0.353751	0	#NAME?
Ciclev10027458m.g	scaffold_7:17245763-17246760	0.435248	0	#NAME?
Ciclev10027490m.g	scaffold_7:17253388-17254223	1.63321	0	#NAME?
Ciclev10026534m.g	scaffold_7:17409665-17410321	0.481397	0	#NAME?
Ciclev10026032m.g	scaffold_7:17533682-17535311	0.285866	0	#NAME?
Ciclev10027175m.g	scaffold_7:17556614-17559862	0.132752	0	#NAME?
Ciclev10026869m.g	scaffold_7:17809780-17810550	0.759542	0	#NAME?
Ciclev10027036m.g	scaffold_7:18127912-18129340	0.152374	0	#NAME?
Ciclev10027050m.g	scaffold_7:18532754-18535461	0.129298	0	#NAME?
Ciclev10026884m.g	scaffold_7:18566244-18566513	2.65126	0	#NAME?
Ciclev10027583m.g	scaffold_7:18808601-18810707	0.18448	0	#NAME?
Ciclev10025017m.g	scaffold_7:18815388-18817728	0.132436	0	#NAME?
Ciclev10027546m.g	scaffold_7:19542763-19544935	0.0941202	0	#NAME?
Ciclev10027611m.g	scaffold_7:20278362-20285118	2.52811	0	#NAME?
Ciclev10025101m.g	scaffold_7:20320963-20325082	0.180688	0	#NAME?
Ciclev10027186m.g	scaffold_7:20788851-20789178	4.35103	0	#NAME?
-	scaffold_7:2925502-2925990	10.3767	0	#NAME?
-	scaffold_7:5835362-5835606	40.1122	0	#NAME?
-	scaffold_7:10648994-10649290	19.5899	0	#NAME?
-	scaffold_7:11686788-11771145	3.62679	0	#NAME?
-	scaffold_7:14297091-14297289	145.919	0	#NAME?
Ciclev10010859m.g	scaffold_73:24580-25569	3.88872	0	#NAME?
Ciclev10028834m.g	scaffold_8:21218-23609	1.09495	0	#NAME?
Ciclev10028616m.g	scaffold_8:103631-105490	0.649413	0	#NAME?
Ciclev10028739m.g	scaffold_8:157206-162831	3.40757	0	#NAME?
Ciclev10029829m.g	scaffold_8:175209-176152	0.455871	0	#NAME?
Ciclev10029905m.g	scaffold_8:651325-652763	0.287481	0	#NAME?
Ciclev10030124m.g	scaffold_8:1026859-1031230	0.0692595	0	#NAME?
Ciclev10028271m.g	scaffold_8:1176549-1180044	1.50801	0	#NAME?
Ciclev10028923m.g	scaffold_8:1347901-1348816	0.217301	0	#NAME?
Ciclev10030063m.g	scaffold_8:1823963-1885505	0.62664	0	#NAME?
Ciclev10028360m.g	scaffold_8:1823963-1885505	11.085	0	#NAME?

Ciclev10030001m.g	scaffold_8:2019296-2019887	2.68047	0	#NAME?
Ciclev10028641m.g	scaffold_8:2388372-2391976	0.756764	0	#NAME?
Ciclev10028330m.g	scaffold_8:3499169-3501467	0.237423	0	#NAME?
Ciclev10029171m.g	scaffold_8:3748963-3750082	0.226207	0	#NAME?
Ciclev10028501m.g	scaffold_8:3898408-3900744	0.143384	0	#NAME?
Ciclev10029698m.g	scaffold_8:3996834-3998789	0.630779	0	#NAME?
Ciclev10030388m.g	scaffold_8:4109901-4110953	0.247171	0	#NAME?
Ciclev10027975m.g	scaffold_8:4227577-4230238	0.152029	0	#NAME?
Ciclev10030404m.g	scaffold_8:5303725-5305282	0.133459	0	#NAME?
Ciclev10028896m.g	scaffold_8:5382891-5385164	0.18204	0	#NAME?
Ciclev10030257m.g	scaffold_8:6057617-6062060	0.16522	0	#NAME?
Ciclev10030263m.g	scaffold_8:6905015-6905627	4.69319	0	#NAME?
Ciclev10029985m.g	scaffold_8:7698015-7862701	115.298	0	#NAME?
Ciclev10029985m.g	scaffold_8:7698015-7862701	0.714138	0	#NAME?
Ciclev10030264m.g	scaffold_8:9619731-9620740	0.278788	0	#NAME?
Ciclev10030445m.g	scaffold_8:9624097-9646817	0.740556	0	#NAME?
Ciclev10029632m.g	scaffold_8:11235253-11235953	2.46168	0	#NAME?
Ciclev10029738m.g	scaffold_8:14280173-14282846	0.166412	0	#NAME?
Ciclev10028113m.g	scaffold_8:17933011-17934818	0.103117	0	#NAME?
Ciclev10028411m.g	scaffold_8:18619680-18621955	0.115673	0	#NAME?
Ciclev10029271m.g	scaffold_8:18768589-18769277	1.63286	0	#NAME?
Ciclev10028507m.g	scaffold_8:19830629-19836353	0.13106	0	#NAME?
Ciclev10030019m.g	scaffold_8:20740038-20748864	2.73741	0	#NAME?
Ciclev10030059m.g	scaffold_8:21450265-21450682	1.69396	0	#NAME?
Ciclev10029902m.g	scaffold_8:21468487-21470031	0.468502	0	#NAME?
Ciclev10029836m.g	scaffold_8:21515129-21516722	0.384332	0	#NAME?
Ciclev10028060m.g	scaffold_8:21632900-21637287	3.32655	0	#NAME?
Ciclev10029471m.g	scaffold_8:22080777-22081499	3.87589	0	#NAME?
Ciclev10030205m.g	scaffold_8:22178877-22179717	1.28694	0	#NAME?
Ciclev10030366m.g	scaffold_8:22403188-22403650	0.8431	0	#NAME?
Ciclev10030166m.g	scaffold_8:22563059-22565235	0.132177	0	#NAME?
Ciclev10028090m.g	scaffold_8:22832243-22834513	1.25303	0	#NAME?
Ciclev10029192m.g	scaffold_8:22988629-22989880	2.63028	0	#NAME?
Ciclev10029904m.g	scaffold_8:22993068-22993932	0.977799	0	#NAME?
Ciclev10029908m.g	scaffold_8:23054037-23057430	0.427226	0	#NAME?
Ciclev10030058m.g	scaffold_8:23155492-23155813	1.5573	0	#NAME?
Ciclev10029999m.g	scaffold_8:23595327-23597157	0.312071	0	#NAME?
Ciclev10029728m.g	scaffold_8:23648206-23648728	0.463667	0	#NAME?
Ciclev10029718m.g	scaffold_8:23707401-23708233	0.579843	0	#NAME?
Ciclev10030165m.g	scaffold_8:23742085-23743630	0.163697	0	#NAME?
Ciclev10029901m.g	scaffold_8:24141787-24143544	0.549547	0	#NAME?
Ciclev10028117m.g	scaffold_8:24234355-24237562	0.993552	0	#NAME?
Ciclev10029990m.g	scaffold_8:24624910-24625702	2.60796	0	#NAME?
Ciclev10029610m.g	scaffold_8:24665724-24666513	0.301947	0	#NAME?
Ciclev10030073m.g	scaffold_8:24883332-24885857	0.163096	0	#NAME?
Ciclev10029924m.g	scaffold_8:24963937-24965130	1.82556	0	#NAME?
Ciclev10028897m.g	scaffold_8:81820-83163	0.138631	0	#NAME?
Ciclev10029408m.g	scaffold_8:149913-151236	2.98207	0	#NAME?
Ciclev10030233m.g	scaffold_8:279496-280435	0.434323	0	#NAME?
Ciclev10030136m.g	scaffold_8:588109-588553	1.30221	0	#NAME?
Ciclev10030357m.g	scaffold_8:606188-607767	0.379753	0	#NAME?
Ciclev10030409m.g	scaffold_8:621322-621706	1.77154	0	#NAME?
Ciclev10028644m.g	scaffold_8:1022628-1025738	0.133768	0	#NAME?
Ciclev10030341m.g	scaffold_8:1417678-1420340	0.0741599	0	#NAME?
Ciclev10030194m.g	scaffold_8:1795916-1796300	2.58408	0	#NAME?
Ciclev10030382m.g	scaffold_8:2044887-2049247	0.429731	0	#NAME?

Ciclev10029597m.g	scaffold_8:2187471-2188993	1.80443	0	#NAME?
Ciclev10027845m.g	scaffold_8:2237490-2240363	0.172634	0	#NAME?
Ciclev10030186m.g	scaffold_8:2479552-2480791	0.16736	0	#NAME?
Ciclev10028578m.g	scaffold_8:2929767-2932689	0.794409	0	#NAME?
Ciclev10028506m.g	scaffold_8:3617543-3620157	0.145903	0	#NAME?
Ciclev10029862m.g	scaffold_8:3933666-3935389	0.433652	0	#NAME?
Ciclev10029726m.g	scaffold_8:4025025-4025709	0.439423	0	#NAME?
Ciclev10027711m.g	scaffold_8:4205576-4222282	0.665963	0	#NAME?
Ciclev10029006m.g	scaffold_8:4787381-4788802	0.380186	0	#NAME?
-	scaffold_8:6822371-6834202	29.5549	0	#NAME?
Ciclev10029995m.g	scaffold_8:6905873-6909096	0.068671	0	#NAME?
Ciclev10028097m.g	scaffold_8:7552931-7556106	0.103121	0	#NAME?
Ciclev10029653m.g	scaffold_8:7612713-7613313	1.62814	0	#NAME?
Ciclev10029714m.g	scaffold_8:7909414-7965005	5.03038	0	#NAME?
Ciclev10029543m.g	scaffold_8:7909414-7965005	0.418799	0	#NAME?
Ciclev10029993m.g	scaffold_8:8891248-8893540	0.10468	0	#NAME?
Ciclev10029804m.g	scaffold_8:9325618-9585469	15.3371	0	#NAME?
-	scaffold_8:9325618-9585469	10.0874	0	#NAME?
Ciclev10029594m.g	scaffold_8:11869284-11869899	2.81229	0	#NAME?
Ciclev10030152m.g	scaffold_8:13207690-13210120	2.7257	0	#NAME?
Ciclev10030281m.g	scaffold_8:18557946-18562222	0.131785	0	#NAME?
Ciclev10028374m.g	scaffold_8:18771986-18773823	2.1521	0	#NAME?
Ciclev10030340m.g	scaffold_8:18863193-18917480	4.66488	0	#NAME?
Ciclev10029837m.g	scaffold_8:18973864-19114160	0.31362	0	#NAME?
Ciclev10027808m.g	scaffold_8:18973864-19114160	1.20969	0	#NAME?
Ciclev10030176m.g	scaffold_8:18973864-19114160	0.076447	0	#NAME?
Ciclev10027879m.g	scaffold_8:19114676-19117756	0.201228	0	#NAME?
Ciclev10028751m.g	scaffold_8:19132836-19211350	0.29888	0	#NAME?
Ciclev10029960m.g	scaffold_8:19132836-19211350	3.1514	0	#NAME?
Ciclev10028361m.g	scaffold_8:19568817-19573518	0.47961	0	#NAME?
Ciclev10030335m.g	scaffold_8:20016894-20019693	0.158955	0	#NAME?
-	scaffold_8:20620043-20723794	20.0125	0	#NAME?
Ciclev10030021m.g	scaffold_8:20894390-20896536	1.36647	0	#NAME?
Ciclev10029305m.g	scaffold_8:21070331-21071095	4.14487	0	#NAME?
Ciclev10030358m.g	scaffold_8:21527343-21528936	0.127872	0	#NAME?
Ciclev10028790m.g	scaffold_8:21605600-21610218	0.932082	0	#NAME?
Ciclev10030292m.g	scaffold_8:21940754-21942371	0.361971	0	#NAME?
Ciclev10030333m.g	scaffold_8:21944342-21947090	0.129676	0	#NAME?
Ciclev10029639m.g	scaffold_8:22284160-22284844	0.877612	0	#NAME?
-	scaffold_8:22583142-22585257	3.39524	0	#NAME?
Ciclev10029356m.g	scaffold_8:22821613-22822718	3.06883	0	#NAME?
Ciclev10028377m.g	scaffold_8:22959627-22961317	0.335957	0	#NAME?
Ciclev10030119m.g	scaffold_8:23201057-23204187	0.0799634	0	#NAME?
Ciclev10029478m.g	scaffold_8:23377508-23378382	2.93462	0	#NAME?
Ciclev10029907m.g	scaffold_8:23619358-23620279	1.04675	0	#NAME?
Ciclev10028036m.g	scaffold_8:23668041-23670573	3.33061	0	#NAME?
Ciclev10029648m.g	scaffold_8:23883894-23884448	0.710597	0	#NAME?
Ciclev10029951m.g	scaffold_8:24461658-24464097	0.429678	0	#NAME?
Ciclev10030260m.g	scaffold_8:24920509-24925230	0.777728	0	#NAME?
Ciclev10030148m.g	scaffold_8:24929812-24932205	0.130241	0	#NAME?
-	scaffold_8:7698015-7862701	76.2181	0	#NAME?
-	scaffold_8:9325618-9585469	7.16114	0	#NAME?
Ciclev10018419m.g	scaffold_82:25257-30461	0.236698	0	#NAME?
Ciclev10005815m.g	scaffold_9:153985-155186	0.950121	0	#NAME?
Ciclev10006437m.g	scaffold_9:771289-775197	0.348194	0	#NAME?
Ciclev10004497m.g	scaffold_9:887055-891912	0.0937735	0	#NAME?

Ciclev10004586m.g	scaffold_9:913000-914983	0.0853595	0	#NAME?
Ciclev10005421m.g	scaffold_9:1014655-1016846	113.222	0	#NAME?
Ciclev10006099m.g	scaffold_9:1211134-1212022	0.336103	0	#NAME?
Ciclev10004341m.g	scaffold_9:1499633-1505333	0.0628132	0	#NAME?
Ciclev10006769m.g	scaffold_9:1921660-1922194	0.363208	0	#NAME?
Ciclev10005161m.g	scaffold_9:2131513-2134136	1.74107	0	#NAME?
Ciclev10006861m.g	scaffold_9:2147112-2148141	0.231274	0	#NAME?
Ciclev10004839m.g	scaffold_9:2386887-2390568	0.0915991	0	#NAME?
Ciclev10005996m.g	scaffold_9:2461477-2462311	51.3043	0	#NAME?
Ciclev10005035m.g	scaffold_9:3039746-3041225	0.868105	0	#NAME?
Ciclev10006962m.g	scaffold_9:3085560-3086868	0.413567	0	#NAME?
Ciclev10006359m.g	scaffold_9:4345601-4346076	1.30681	0	#NAME?
Ciclev10005789m.g	scaffold_9:4540248-4542147	16.3487	0	#NAME?
Ciclev10005027m.g	scaffold_9:4566887-4573847	0.426826	0	#NAME?
Ciclev10005654m.g	scaffold_9:4584734-4586034	0.378543	0	#NAME?
Ciclev10004701m.g	scaffold_9:4656634-4660371	0.529126	0	#NAME?
Ciclev10004875m.g	scaffold_9:5086952-5092404	1.0196	0	#NAME?
Ciclev10007056m.g	scaffold_9:5802891-5803743	0.236095	0	#NAME?
-	scaffold_9:6075452-6076812	5.13455	0	#NAME?
Ciclev10005297m.g	scaffold_9:7266232-7268592	0.118352	0	#NAME?
Ciclev10006335m.g	scaffold_9:8088694-8091407	4.35667	0	#NAME?
Ciclev10004698m.g	scaffold_9:8376736-8414244	0.11497	0	#NAME?
Ciclev10006747m.g	scaffold_9:10565044-10567426	0.316484	0	#NAME?
Ciclev10005530m.g	scaffold_9:11144610-11148313	0.192774	0	#NAME?
Ciclev10006543m.g	scaffold_9:12493821-12494580	0.297491	0	#NAME?
Ciclev10005821m.g	scaffold_9:12770329-12773458	1.2801	0	#NAME?
Ciclev10006808m.g	scaffold_9:13858804-13859350	4.83059	0	#NAME?
Ciclev10005647m.g	scaffold_9:14650183-14651602	1.4488	0	#NAME?
Ciclev10006377m.g	scaffold_9:16838229-16846426	0.700944	0	#NAME?
Ciclev10006416m.g	scaffold_9:23234580-23235414	0.778696	0	#NAME?
Ciclev10004287m.g	scaffold_9:24356661-24541601	0.20389	0	#NAME?
Ciclev10006443m.g	scaffold_9:24356661-24541601	0.283612	0	#NAME?
Ciclev10006818m.g	scaffold_9:24591498-24592806	0.149341	0	#NAME?
Ciclev10005549m.g	scaffold_9:25393462-25394344	0.652341	0	#NAME?
Ciclev10006519m.g	scaffold_9:25461262-25461976	0.306413	0	#NAME?
Ciclev10004476m.g	scaffold_9:27288794-27291710	0.836745	0	#NAME?
Ciclev10007178m.g	scaffold_9:27629620-27630250	0.773589	0	#NAME?
Ciclev10004821m.g	scaffold_9:27753859-27755705	0.634667	0	#NAME?
Ciclev10006506m.g	scaffold_9:27790597-27828145	0.295735	0	#NAME?
Ciclev10004550m.g	scaffold_9:28686868-28689850	0.52782	0	#NAME?
Ciclev10004612m.g	scaffold_9:29153014-29158197	0.105381	0	#NAME?
Ciclev10004673m.g	scaffold_9:29290414-29356041	0.100183	0	#NAME?
Ciclev10004997m.g	scaffold_9:29647272-29648944	0.116955	0	#NAME?
Ciclev10006338m.g	scaffold_9:30698694-30699348	1.35154	0	#NAME?
Ciclev10004556m.g	scaffold_9:30906256-30912952	0.171466	0	#NAME?
Ciclev10005040m.g	scaffold_9:31159646-31162362	0.205461	0	#NAME?
Ciclev10006511m.g	scaffold_9:654465-654996	0.561495	0	#NAME?
Ciclev10006832m.g	scaffold_9:876348-879293	0.155695	0	#NAME?
Ciclev10006674m.g	scaffold_9:1297964-1298553	0.505854	0	#NAME?
Ciclev10006857m.g	scaffold_9:1347062-1350653	0.731747	0	#NAME?
Ciclev10007176m.g	scaffold_9:1682281-1683762	1.78619	0	#NAME?
Ciclev10004993m.g	scaffold_9:2957538-2959079	0.623711	0	#NAME?
Ciclev10005615m.g	scaffold_9:3331077-3332205	0.368658	0	#NAME?
Ciclev10005024m.g	scaffold_9:3580467-3584207	0.222166	0	#NAME?
Ciclev10006578m.g	scaffold_9:3723456-3725817	0.866047	0	#NAME?
Ciclev10007103m.g	scaffold_9:4035240-4036365	0.63539	0	#NAME?

Ciclev10006336m.g	scaffold_9:5021287-5065070	1.90629	0	#NAME?
Ciclev10005356m.g	scaffold_9:6758405-6759856	0.270263	0	#NAME?
Ciclev10006446m.g	scaffold_9:7578018-7579637	0.258983	0	#NAME?
Ciclev10006937m.g	scaffold_9:8109166-8109454	3.17351	0	#NAME?
Ciclev10007065m.g	scaffold_9:8665160-8665598	0.859763	0	#NAME?
Ciclev10004202m.g	scaffold_9:9744445-9748104	0.103671	0	#NAME?
Ciclev10006343m.g	scaffold_9:9853410-9854105	2.33001	0	#NAME?
Ciclev10005049m.g	scaffold_9:9890949-9892950	0.14867	0	#NAME?
Ciclev10006384m.g	scaffold_9:10075988-10076573	3.75165	0	#NAME?
Ciclev10006476m.g	scaffold_9:10420186-10424438	0.116866	0	#NAME?
Ciclev10006754m.g	scaffold_9:11368154-11373973	0.048077	0	#NAME?
Ciclev10004436m.g	scaffold_9:15163738-15166496	0.171487	0	#NAME?
Ciclev10006459m.g	scaffold_9:16135001-16135463	1.29211	0	#NAME?
Ciclev10004582m.g	scaffold_9:16821131-16823929	0.191738	0	#NAME?
Ciclev10006617m.g	scaffold_9:18738370-18740295	1.3044	0	#NAME?
Ciclev10007027m.g	scaffold_9:20890036-21029395	0.080646	0	#NAME?
Ciclev10007026m.g	scaffold_9:21604068-21606001	0.179783	0	#NAME?
Ciclev10006607m.g	scaffold_9:23460140-23461613	0.226244	0	#NAME?
Ciclev10006299m.g	scaffold_9:24125764-24129301	2.2344	0	#NAME?
Ciclev10005317m.g	scaffold_9:25064864-25067379	0.445896	0	#NAME?
Ciclev10007195m.g	scaffold_9:25575627-25576261	102.843	0	#NAME?
Ciclev10004697m.g	scaffold_9:26263460-26265080	4.62091	0	#NAME?
Ciclev10005633m.g	scaffold_9:26509544-26511289	0.445044	0	#NAME?
Ciclev10006088m.g	scaffold_9:27151953-27152892	0.552147	0	#NAME?
Ciclev10006988m.g	scaffold_9:27513802-27515432	2.00992	0	#NAME?
Ciclev10004719m.g	scaffold_9:27540635-27542779	0.620151	0	#NAME?
Ciclev10006448m.g	scaffold_9:28440341-28441712	0.458947	0	#NAME?
Ciclev10006541m.g	scaffold_9:28462025-28466344	0.312949	0	#NAME?
Ciclev10005834m.g	scaffold_9:28881122-28882519	0.732927	0	#NAME?
Ciclev10006187m.g	scaffold_9:28944546-28945624	1.16497	0	#NAME?
Ciclev10006720m.g	scaffold_9:29676414-29680037	0.068705	0	#NAME?
Ciclev10004921m.g	scaffold_9:30524879-30526299	0.315252	0	#NAME?
Ciclev10005428m.g	scaffold_9:30861628-30863510	0.715806	0	#NAME?
Ciclev10005103m.g	scaffold_9:30923986-30925850	0.282919	0	#NAME?
Ciclev10006555m.g	scaffold_9:30988226-30988751	0.900943	0	#NAME?
Ciclev10005341m.g	scaffold_9:31093423-31096400	0.21259	0	#NAME?
Ciclev10004908m.g	scaffold_9:31207178-31209810	0.203843	0	#NAME?
-	scaffold_9:2399887-2400213	13.2766	0	#NAME?
-	scaffold_9:8195073-8195266	229.644	0	#NAME?
-	scaffold_9:9520908-9521376	5.92868	0	#NAME?
-	scaffold_9:23318530-23318746	203.934	0	#NAME?

transcripts identified in Flying Dragon RNA-Seq

Gene_id	Locus	FPKM Value (greenhouse-grown)	FPKM Value (field-collected)	log2(fold_change)
Ciclev10027014m.g	scaffold_7:9212797-9232329	5.20506	0.000970568	-12.3888
Ciclev10000466m.g	scaffold_5:37349892-37356610	7.52372	0.00218675	-11.7484
Ciclev10007321m.g	scaffold_1:28248422-28254232	38.3995	0.0423919	-9.82308
Ciclev10018639m.g	scaffold_3:42996885-43001548	37.6338	0.0448098	-9.714
-	scaffold_8:17878427-17881811	127.74	0.155329	-9.68367
Ciclev10014707m.g	scaffold_2:32481548-32484643	366.824	0.449732	-9.6718
Ciclev10007167m.g	scaffold_9:8509471-8558807	2.8779	0.00500445	-9.16759
Ciclev10009194m.g	scaffold_1:3986692-3988340	155.281	0.290616	-9.06155
Ciclev10005136m.g	scaffold_9:8566657-8568825	11530.1	25.5528	-8.8177
Ciclev10013766m.g	scaffold_6:20981041-20981593	239.136	0.559575	-8.73928
Ciclev10020292m.g	scaffold_3:8410167-8412277	37.9603	0.108348	-8.45267
-	scaffold_1:790598-824170	70.6285	0.227168	-8.28034
Ciclev10017249m.g	scaffold_2:7543355-7569782	175.542	0.63503	-8.11078
Ciclev10009935m.g	scaffold_1:1472059-1472860	74.4941	0.285283	-8.02859
Ciclev10015194m.g,Ciclev10015199m.g	scaffold_2:7789813-7814952	378.033	1.5223	-7.95612
Ciclev10005777m.g	scaffold_9:4699069-4700431	58.6176	0.255151	-7.84384
Ciclev10028930m.g	scaffold_8:3113094-3114407	234.353	1.0783	-7.76378
Ciclev10001968m.g	scaffold_5:36226059-36227269	225.473	1.08117	-7.70421
Ciclev10011400m.g	scaffold_6:15761840-15764472	17.0121	0.0832348	-7.67516
Ciclev10031021m.g	scaffold_4:21321026-21325773	17.1461	0.0859182	-7.6407
Ciclev10017776m.g	scaffold_2:5501695-5504211	19.4185	0.101279	-7.58296
Ciclev10029032m.g	scaffold_8:19288176-19290093	109.603	0.661562	-7.37219
Ciclev10032858m.g	scaffold_4:24521144-24523696	544.507	3.4919	-7.28479
Ciclev10002142m.g	scaffold_5:1443599-1445440	44.9842	0.300928	-7.22386
Ciclev10017057m.g	scaffold_2:10566017-10566892	305.635	2.04477	-7.22373
Ciclev10022120m.g	scaffold_3:45622522-45629218	50.526	0.346354	-7.18863
Ciclev10005687m.g	scaffold_9:3453528-3455361	413.087	2.86733	-7.1706
Ciclev10015599m.g	scaffold_2:26745506-26746983	182.105	1.27784	-7.15492
-	scaffold_1:790598-824170	17.5295	0.13374	-7.03421
Ciclev10031320m.g	scaffold_4:25354556-25358068	36.4358	0.279945	-7.02407
Ciclev10017435m.g,Ciclev10018311m.g	scaffold_2:9758751-9769135	92.3669	0.733901	-6.97565
Ciclev10015163m.g	scaffold_2:7789813-7814952	1590.7	13.2656	-6.90584
Ciclev10002026m.g	scaffold_5:2485691-2492388	12.6354	0.107697	-6.87434
Ciclev10028988m.g	scaffold_8:22184809-22187211	60.7758	0.526099	-6.85202
Ciclev10033998m.g	scaffold_4:25629279-25629850	62.2463	0.55668	-6.80499
Ciclev10001665m.g	scaffold_5:42957413-42961730	69.5069	0.644067	-6.7538
Ciclev10002675m.g	scaffold_5:36080141-36081013	31.0049	0.312502	-6.63249
Ciclev10014216m.g	scaffold_2:7543355-7569782	23.2245	0.245379	-6.56449
Ciclev10016590m.g	scaffold_2:26650149-26651148	31.5688	0.334216	-6.56157
Ciclev10022337m.g	scaffold_3:49295580-49296607	18.5875	0.197087	-6.55936
Ciclev10014155m.g	scaffold_2:34363430-34369047	22.9633	0.244773	-6.55174
Ciclev10025272m.g	scaffold_7:16553737-16556732	6.76336	0.0764183	-6.46768
Ciclev10005996m.g	scaffold_9:2461477-2462311	25.7429	0.304158	-6.40321
Ciclev10001970m.g	scaffold_5:36219782-36221019	48.0857	0.568273	-6.40288
Ciclev10011217m.g	scaffold_6:17453268-17455623	4.76971	0.0573937	-6.37686
Ciclev10009285m.g	scaffold_1:26683179-26687729	265.748	3.27156	-6.34393
Ciclev10027295m.g	scaffold_7:7194736-7222786	79.5169	1.00204	-6.31025
Ciclev10027998m.g	scaffold_8:22969361-22971701	17.5945	0.221806	-6.30969
-	scaffold_7:1774752-1776831	2339.21	29.6206	-6.30328

-	scaffold_5:33676225-33676837	219.164	2.84166	-6.26913
Ciclev10015449m.g	scaffold_2:28206320-28209328	32.7439	0.440596	-6.21563
Ciclev10007068m.g	scaffold_9:28562375-28563020	94.4187	1.28641	-6.19766
Ciclev10020247m.g	scaffold_3:33005684-33007151	47.0511	0.649847	-6.17798
Ciclev10031625m.g	scaffold_4:16379986-16381763	94.4748	1.32573	-6.15507
-	scaffold_1:790598-824170	541.757	7.78787	-6.12027
Ciclev10015460m.g	scaffold_2:22436684-22438478	130.22	1.87528	-6.1177
Ciclev10010491m.g	scaffold_1:28782168-28786731	308.026	4.50561	-6.09518
Ciclev10020164m.g	scaffold_3:1888556-1892035	18.1982	0.277436	-6.0355
Ciclev10023723m.g	scaffold_3:45988657-45993348	12.8775	0.199886	-6.00953
Ciclev10014082m.g,Ciclev10014554m.g	scaffold_2:7543355-7569782	40.8197	0.648771	-5.97541
Ciclev10011765m.g	scaffold_6:24942145-24947983	274.664	4.55195	-5.91504
Ciclev10019940m.g	scaffold_3:44205871-44208997	10.2538	0.170494	-5.9103
Ciclev10001451m.g	scaffold_5:39905513-39907734	12.7019	0.212482	-5.90156
-	scaffold_5:914285-943664	541.312	9.34542	-5.85606
Ciclev10025958m.g,Ciclev10027096m.g	scaffold_7:11577923-11593393	12.1071	0.210339	-5.847
Ciclev10032008m.g	scaffold_4:20060786-20063674	38.8665	0.682506	-5.83154
Ciclev10029464m.g	scaffold_8:23078211-23079067	298.036	5.23703	-5.83059
Ciclev10015743m.g	scaffold_2:26076215-26078590	65.4052	1.15524	-5.82314
-	scaffold_9:1447307-1447834	50.4935	0.897972	-5.81328
Ciclev10005152m.g	scaffold_9:24624293-24625478	8.93162	0.159529	-5.80703
Ciclev10020341m.g	scaffold_3:40274195-40275794	28.1874	0.504618	-5.80371
Ciclev10014970m.g	scaffold_2:32121968-32123805	4.00439	0.0717794	-5.80187
Ciclev10032829m.g	scaffold_4:17127920-17129243	90.7145	1.65041	-5.78043
Ciclev10024825m.g	scaffold_7:176885-180673	116.225	2.12139	-5.77577
Ciclev10013311m.g	scaffold_6:12933746-12934265	19.9784	0.366275	-5.76937
Ciclev10016770m.g	scaffold_2:36099095-36101018	45.0428	0.827938	-5.76563
Ciclev10005869m.g	scaffold_9:28569930-28570930	17.3205	0.333976	-5.69659
Ciclev10022001m.g	scaffold_3:18341165-18342395	22287.5	438.672	-5.66695
Ciclev10000437m.g	scaffold_5:34837869-34841284	61.7126	1.22789	-5.65131
Ciclev10000658m.g	scaffold_5:40214034-40216955	4.11127	0.081942	-5.64884
Ciclev10001881m.g,Ciclev10003596m.g	scaffold_5:33629909-33634531	25.7468	0.516244	-5.64019
-	scaffold_9:1202683-1203016	434.567	8.80652	-5.62486
Ciclev10021277m.g	scaffold_3:47366615-47370788	9.61425	0.196494	-5.61262
Ciclev10004697m.g	scaffold_9:26263460-26265080	4.89151	0.0999787	-5.61252
Ciclev10015630m.g	scaffold_2:10646172-10649019	35.5468	0.731996	-5.60174
Ciclev10014209m.g	scaffold_2:5582926-5587253	336.088	7.10596	-5.56367
Ciclev10015351m.g	scaffold_2:6400796-6402311	6.64611	0.141035	-5.55839
Ciclev10011844m.g	scaffold_6:19813517-19815060	66.4347	1.42593	-5.54196
-	scaffold_4:16358963-16359491	20.4898	0.447596	-5.51657
Ciclev10027912m.g,Ciclev10027913m.g	scaffold_8:21712356-21744083	5.79131	0.127731	-5.50271
Ciclev10008951m.g	scaffold_1:27419631-27421046	8.50686	0.189605	-5.48756
Ciclev10016351m.g	scaffold_2:8406644-8407971	401.344	8.96187	-5.4849
-	scaffold_1:11018842-11020797	1829.28	40.8886	-5.48343
Ciclev10007843m.g	scaffold_1:20440642-20444068	677.332	15.4524	-5.45396
Ciclev10009484m.g	scaffold_1:16724986-16726058	1538.55	35.1789	-5.45072
Ciclev10017299m.g	scaffold_2:28030133-28030667	32.3172	0.747188	-5.43469
Ciclev10003017m.g	scaffold_5:26171952-26172322	46.9399	1.10128	-5.41356
Ciclev10014163m.g	scaffold_2:83182-92491	1.86338	0.0438024	-5.41077
Ciclev10014659m.g	scaffold_2:7524254-7527181	11.8939	0.282837	-5.39411
-	scaffold_7:20327996-20328419	27.8262	0.663044	-5.39119
Ciclev10022737m.g	scaffold_3:45198152-45199507	10.5741	0.255444	-5.37139

Ciclev10011455m.g	scaffold_6:21150415-21155246	2.96537	0.0721698	-5.36067
Ciclev10020222m.g	scaffold_3:43135377-43138351	30.1522	0.74382	-5.34117
Ciclev10031286m.g	scaffold_4:9423809-9425662	9.81855	0.244902	-5.32523
-	scaffold_2:26287888-26288149	96.3852	2.42188	-5.31461
Ciclev10023060m.g	scaffold_3:13416060-13417219	1060.61	27.3863	-5.2753
Ciclev10027663m.g	scaffold_8:21712356-21744083	24.6121	0.653007	-5.23612
-	scaffold_6:14886115-14886335	597.429	16.2293	-5.2021
Ciclev10009184m.g	scaffold_1:3921881-3923362	19495.7	530.278	-5.20026
Ciclev10005789m.g	scaffold_9:4540248-4542147	32.8118	0.896062	-5.19447
Ciclev10016691m.g	scaffold_2:11634676-11635703	6.09291	0.168689	-5.17469
Ciclev10000991m.g	scaffold_5:37687806-37690745	93.2583	2.61229	-5.15784
Ciclev10017020m.g	scaffold_2:34758767-34759899	10.5887	0.297039	-5.15573
Ciclev10007402m.g	scaffold_1:27636798-27640535	6.08965	0.17107	-5.1537
Ciclev10009540m.g	scaffold_1:24667692-24668658	1347.82	38.3142	-5.13661
Ciclev10016917m.g	scaffold_2:32720608-32721917	43.7849	1.25238	-5.12768
Ciclev10015955m.g	scaffold_2:33473684-33475325	130.732	3.74152	-5.12684
Ciclev10013026m.g	scaffold_6:22728225-22728909	10.4967	0.301956	-5.11945
Ciclev10029149m.g	scaffold_8:23240222-23241308	11.2606	0.327725	-5.10266
Ciclev10025012m.g,Ciclev10025047m.g	scaffold_7:10035354-10116411	24.125	0.709922	-5.08673
Ciclev10016694m.g	scaffold_2:28111468-28112452	133.774	3.93735	-5.08643
Ciclev10009118m.g,Ciclev10009119m.g	scaffold_1:27422722-27451036	30.1824	0.895157	-5.07542
Ciclev10022753m.g	scaffold_3:4311922-4312682	173.087	5.16147	-5.06757
Ciclev10007591m.g	scaffold_1:1762846-1768718	5.64493	0.168679	-5.0646
Ciclev10012857m.g	scaffold_6:15341356-15343333	128.493	3.84268	-5.06344
Ciclev10030540m.g	scaffold_4:17121877-17126289	75.8464	2.27133	-5.06147
Ciclev10017155m.g	scaffold_2:26105694-26162104	15.5099	0.466273	-5.05587
Ciclev10002169m.g	scaffold_5:42790656-42792286	28.2838	0.851463	-5.05389
Ciclev10011754m.g	scaffold_6:21271369-21273131	26.835	0.811919	-5.04664
Ciclev10009680m.g	scaffold_1:22093835-22094718	47.2788	1.43944	-5.03762
Ciclev10033110m.g	scaffold_4:25171279-25172219	40.8109	1.24721	-5.03218
Ciclev10028254m.g	scaffold_8:20047847-20049651	300.199	9.21573	-5.02568
Ciclev10028090m.g	scaffold_8:22832243-22834513	3.38942	0.106288	-4.99498
Ciclev10000431m.g	scaffold_5:20550262-20553313	7.85434	0.246617	-4.99315
Ciclev10021398m.g	scaffold_3:12814730-12817148	4.07985	0.128326	-4.99063
Ciclev10001822m.g	scaffold_5:35721730-35723544	128.611	4.05353	-4.98769
Ciclev10029854m.g	scaffold_8:7925785-7963167	43.9299	1.38978	-4.98227
Ciclev10010728m.g	scaffold_1:8090395-8092825	2.11866	0.0674544	-4.97309
Ciclev10029088m.g	scaffold_8:502051-503766	196.504	6.25754	-4.97282
Ciclev10002399m.g	scaffold_5:35872808-35892772	108.492	3.47572	-4.96413
Ciclev10017438m.g	scaffold_2:32980874-32985696	19.5658	0.632803	-4.95044
Ciclev10024208m.g	scaffold_3:27579559-27626412	1.14841	0.0372304	-4.94701
Ciclev10019837m.g,Ciclev10023439m.g,Ciclev10023835m.g	scaffold_3:32543189-32702382	7.45084	0.24237	-4.94212
Ciclev10019427m.g,Ciclev10019611m.g	scaffold_3:6736405-6745205	75.6741	2.46903	-4.93778
-	scaffold_3:9784868-9785222	88.8554	2.90514	-4.93478
Ciclev10013212m.g	scaffold_6:14721969-14722645	11.1072	0.367035	-4.91943
Ciclev10001325m.g	scaffold_5:43048046-43051036	26.5319	0.88602	-4.90424
Ciclev10031284m.g	scaffold_4:9614195-9617653	3.24036	0.108591	-4.89917
Ciclev10005075m.g	scaffold_9:13365217-13368617	37.9563	1.27463	-4.89618
Ciclev10029198m.g	scaffold_8:2817297-2822179	12.9141	0.4352	-4.89112
Ciclev10016436m.g	scaffold_2:34381654-34382452	29.1595	0.995909	-4.87181
Ciclev10029316m.g	scaffold_8:2006038-2014782	22.4338	0.770985	-4.86283

Ciclev10014632m.g	scaffold_2:32488694-32493305	591.581	20.3706	-4.86002
Ciclev10017620m.g	scaffold_2:23858359-23858920	12.1931	0.420011	-4.8595
Ciclev10017106m.g	scaffold_2:33791774-33792471	744.144	25.7893	-4.85074
Ciclev10031540m.g	scaffold_4:23082915-23085609	346.925	12.0727	-4.84481
Ciclev10022438m.g	scaffold_3:23315528-23316461	15.958	0.558893	-4.83556
Ciclev10028834m.g	scaffold_8:21218-23609	4.51772	0.158996	-4.82853
Ciclev10004884m.g	scaffold_9:22730945-22732373	3.95438	0.140077	-4.81916
Ciclev10004099m.g	scaffold_5:17583731-17584966	28.6163	1.01592	-4.81597
Ciclev10011655m.g	scaffold_6:23669284-23671821	10.2188	0.364745	-4.80819
Ciclev10025893m.g	scaffold_7:7783570-7785474	4.23085	0.151578	-4.80281
-	scaffold_3:9796583-9797058	311.537	11.2444	-4.79213
Ciclev10015253m.g	scaffold_2:26750606-26752196	28.0737	1.0164	-4.78768
-	scaffold_3:44741195-44741701	26.2716	0.960622	-4.77339
Ciclev10015478m.g	scaffold_2:10156609-10177311	22.6357	0.831234	-4.7672
Ciclev10028253m.g	scaffold_8:20027849-20029377	37.2323	1.3693	-4.76505
Ciclev10005113m.g	scaffold_9:4742708-4746966	5.22825	0.192548	-4.76304
Ciclev10032960m.g	scaffold_4:16250556-16251633	9.63182	0.35872	-4.74688
Ciclev10029408m.g	scaffold_8:149913-151236	7.77834	0.28973	-4.74668
Ciclev10001160m.g	scaffold_5:8745603-8989235	3.10876	0.116146	-4.74232
Ciclev10004574m.g	scaffold_9:28928844-28932535	38.3839	1.45093	-4.72545
Ciclev10013568m.g	scaffold_6:4405921-4407715	47.8599	1.82631	-4.71181
-	scaffold_9:27418916-27419740	18.2304	0.70063	-4.70155
Ciclev10028203m.g	scaffold_8:8221246-8224426	40.8099	1.57477	-4.69571
Ciclev10024088m.g	scaffold_3:32543189-32702382	16.7965	0.650593	-4.69026
Ciclev10011626m.g	scaffold_6:18361274-18364004	2.66295	0.104422	-4.67254
-	scaffold_9:23318537-23318747	179.701	7.11983	-4.65761
Ciclev10002632m.g	scaffold_5:7104382-7105206	22.8396	0.910368	-4.64894
Ciclev10022370m.g	scaffold_3:43862439-43863744	152.82	6.09843	-4.64725
Ciclev10002179m.g	scaffold_5:38447705-38448556	136.553	5.49367	-4.63555
Ciclev10020935m.g	scaffold_3:40289940-40295349	3.17229	0.128568	-4.62493
Ciclev10000849m.g	scaffold_5:41833069-41836812	25.2437	1.02847	-4.61736
Ciclev10029632m.g	scaffold_8:11235253-11235953	10.4786	0.42788	-4.6141
Ciclev10002900m.g	scaffold_5:432292-517176	1148.99	47.6367	-4.59215
Ciclev10015595m.g	scaffold_2:12531372-12534397	141.336	5.86873	-4.58993
Ciclev10028106m.g	scaffold_8:7862728-7866997	34.4469	1.43063	-4.58965
Ciclev10031429m.g	scaffold_4:21857649-21859855	2.12345	0.0882204	-4.58915
Ciclev10000420m.g	scaffold_5:15801848-15810954	1.77294	0.0737496	-4.58736
Ciclev10022129m.g	scaffold_3:18472966-18473951	2513.1	105.059	-4.5802
Ciclev10009639m.g	scaffold_1:18140595-18142122	157.537	6.58829	-4.57964
-	scaffold_4:11522840-11523150	65.26	2.72954	-4.57947
Ciclev10003275m.g	scaffold_5:41643985-41645593	5.22446	0.218728	-4.57807
Ciclev10003989m.g	scaffold_5:5815714-5818239	28.1697	1.1821	-4.57472
Ciclev10001854m.g	scaffold_5:36229309-36230518	83.9954	3.5424	-4.56751
Ciclev10003688m.g	scaffold_5:21041859-21042911	127.213	5.36937	-4.56635
Ciclev10016982m.g	scaffold_2:33654375-33655865	55.4355	2.34198	-4.56501
Ciclev10026661m.g	scaffold_7:5538772-5539370	7.14403	0.303004	-4.55933
Ciclev10017209m.g	scaffold_2:35905503-35906345	332.453	14.1233	-4.557
Ciclev10011673m.g	scaffold_6:1078459-1084125	23.9266	1.01747	-4.55556
-	scaffold_5:28289811-28801386	11.6577	0.495942	-4.55497
Ciclev10022124m.g,Ciclev10023287m.g	scaffold_3:18478836-18487058	3471.14	147.938	-4.55235
Ciclev10006056m.g	scaffold_9:2676492-2677559	24.0153	1.03094	-4.54192
Ciclev10025237m.g,Ciclev10025240m.g	scaffold_7:19858337-20044570	7.69306	0.332082	-4.53395
Ciclev10000816m.g	scaffold_5:33695911-33697986	3.86561	0.16888	-4.51662
Ciclev10022074m.g	scaffold_3:33714569-33716049	9.76963	0.426831	-4.51657

Ciclev10012533m.g	scaffold_6:18228712-18229800	24.1196	1.05564	-4.51402
Ciclev10019134m.g	scaffold_3:5981065-5985524	17.4747	0.765417	-4.51288
Ciclev10021356m.g	scaffold_3:42444808-42447119	5.15428	0.226094	-4.51077
Ciclev10025332m.g	scaffold_7:7666254-7668285	4.24994	0.187532	-4.50224
Ciclev10012912m.g	scaffold_6:10341293-10342204	186.961	8.2815	-4.4967
Ciclev10017975m.g	scaffold_2:7517663-7520570	34.2239	1.52783	-4.48545
Ciclev10030843m.g	scaffold_4:21976522-21979652	6.30996	0.282266	-4.4825
Ciclev10017302m.g	scaffold_2:5916118-5916575	37.8561	1.70496	-4.47271
Ciclev10024949m.g	scaffold_7:5042507-5046278	307.484	13.9971	-4.45731
Ciclev10007801m.g	scaffold_1:22295399-22298532	40.741	1.85506	-4.45694
Ciclev10021141m.g	scaffold_3:328702-330551	2.53459	0.115587	-4.45471
Ciclev10028824m.g	scaffold_8:21432040-21433970	183.373	8.37397	-4.45272
Ciclev10000083m.g	scaffold_5:41604995-41608538	1.05765	0.0483888	-4.45005
Ciclev10018906m.g	scaffold_3:5182211-5185070	8.5102	0.39073	-4.44495
Ciclev10011795m.g	scaffold_6:22985432-22987317	26.3358	1.21286	-4.44054
Ciclev10020289m.g	scaffold_3:32543189-32702382	13.3675	0.616237	-4.43911
Ciclev10006989m.g	scaffold_9:29415524-29424081	2.00682	0.0925721	-4.43819
Ciclev10009800m.g	scaffold_1:25204978-25205573	6.90803	0.320014	-4.43207
-	scaffold_5:9780154-9780566	15.0269	0.698212	-4.42773
Ciclev10033694m.g	scaffold_4:25155260-25156786	14.4559	0.679416	-4.41122
Ciclev10029305m.g	scaffold_8:21070331-21071095	5.98553	0.282829	-4.40348
Ciclev10029174m.g	scaffold_8:13205352-13206868	135.763	6.44233	-4.39736
Ciclev10001552m.g	scaffold_5:35027721-35030201	121.217	5.75228	-4.39731
Ciclev10009138m.g	scaffold_1:7151774-7153282	7.05617	0.336635	-4.38963
Ciclev10030216m.g	scaffold_8:7699848-7862562	8.79925	0.422756	-4.37948
Ciclev10011115m.g	scaffold_6:12707185-12711139	14.7521	0.709175	-4.37864
Ciclev10001946m.g	scaffold_5:41158107-41160518	214.392	10.3338	-4.37481
Ciclev10025834m.g	scaffold_7:70854-73880	546.087	26.3751	-4.37189
Ciclev10026545m.g	scaffold_7:4127517-4128342	38.4929	1.86386	-4.36822
Ciclev10031481m.g	scaffold_4:24750936-24753262	1.55651	0.0757461	-4.361
Ciclev10000761m.g	scaffold_5:43016597-43020200	172.171	8.38251	-4.36032
Ciclev10005327m.g,Ciclev10005484m.g	scaffold_9:280914-294262	24.6102	1.20001	-4.35814
Ciclev10012953m.g	scaffold_6:17547279-17548067	6.23229	0.304279	-4.3563
Ciclev10017395m.g	scaffold_2:7832348-7846578	43.9061	2.14545	-4.35507
Ciclev10033022m.g	scaffold_4:3975803-3978807	1.94097	0.0962732	-4.3335
Ciclev10033519m.g	scaffold_4:1804770-1805424	3815.69	189.648	-4.33055
Ciclev10033215m.g	scaffold_4:23142594-23143167	62.1818	3.1218	-4.31604
Ciclev10033111m.g	scaffold_4:22932585-22935026	8.53841	0.429359	-4.31371
Ciclev10012276m.g	scaffold_6:21048051-21049344	3.01086	0.152366	-4.30456
-	scaffold_8:2273239-2274799	127.435	6.44934	-4.30447
Ciclev10019666m.g	scaffold_3:4975978-4977714	44.9947	2.2868	-4.29835
Ciclev10024819m.g,Ciclev10027295m.g	scaffold_7:7194736-7222786	299.099	15.2939	-4.2896
Ciclev10018959m.g	scaffold_3:46357699-46364094	9.3446	0.47801	-4.28902
Ciclev10028564m.g	scaffold_8:18342017-18343462	6.96187	0.35625	-4.28851
Ciclev10012140m.g	scaffold_6:23349635-23354158	17.1189	0.876022	-4.28848
Ciclev10002761m.g	scaffold_5:32261505-32263373	3.24453	0.166192	-4.28709
Ciclev10018798m.g	scaffold_3:27755539-27797748	1.34324	0.0689239	-4.28457
Ciclev10027093m.g	scaffold_7:5393033-5397048	3.60617	0.186955	-4.2697
Ciclev10014212m.g	scaffold_2:5454555-5460438	1.07529	0.055814	-4.26796
-	scaffold_8:653922-655977	69.8516	3.65109	-4.25789
Ciclev10011293m.g	scaffold_6:21421140-21424496	1.68074	0.0879036	-4.25703
Ciclev10000364m.g	scaffold_5:40040263-40042949	23.3374	1.22779	-4.24851
Ciclev10011088m.g	scaffold_6:21745027-21747494	1.27445	0.0670524	-4.24844
Ciclev10030732m.g	scaffold_4:1206775-1209356	43.645	2.31844	-4.23459

Ciclev10030893m.g	scaffold_4:2709803-2712515	10.5493	0.561083	-4.23278
Ciclev10001440m.g	scaffold_5:38719566-38721176	411.533	21.9359	-4.22964
Ciclev10021859m.g	scaffold_3:16348511-16350656	3.12212	0.166619	-4.22791
Ciclev10001964m.g	scaffold_5:39852395-39853836	2.53046	0.137366	-4.20331
Ciclev10026376m.g	scaffold_7:7340883-7342206	431.498	23.4508	-4.20165
Ciclev10003381m.g	scaffold_5:42565118-42566009	9.58253	0.522284	-4.1975
-	scaffold_8:2274851-2275080	399.245	21.7995	-4.19491
Ciclev10031870m.g	scaffold_4:23135569-23137302	72.7852	4.00926	-4.18224
Ciclev10019364m.g	scaffold_3:29351853-29354190	245.793	13.6018	-4.17557
Ciclev10004484m.g,Ciclev10006614m.g	scaffold_9:3500323-3515616	265.678	14.7164	-4.17418
Ciclev10025537m.g	scaffold_7:3245923-3249254	3.4186	0.189509	-4.17307
-	scaffold_6:10361043-10361232	264.288	14.7389	-4.16441
Ciclev10003593m.g	scaffold_5:3753102-3965022	52.5289	2.93189	-4.16321
Ciclev10020744m.g	scaffold_3:48321395-48324697	447.962	25.064	-4.15969
Ciclev10005992m.g	scaffold_9:5417912-5418877	19.3935	1.08818	-4.15558
Ciclev10028082m.g	scaffold_8:89020-91588	68.4596	3.87454	-4.14316
Ciclev10013304m.g	scaffold_6:1516209-1517464	460.823	26.1123	-4.14141
-	scaffold_5:28289811-28801386	100.221	5.69127	-4.13829
Ciclev10022487m.g	scaffold_3:46371639-46372839	252.449	14.3511	-4.13675
Ciclev10022041m.g	scaffold_3:18363071-18364170	23474.5	1346.18	-4.12415
Ciclev10011238m.g	scaffold_6:21226626-21229106	9.41545	0.540689	-4.12216
Ciclev10004503m.g	scaffold_9:2020990-2023201	1.09226	0.0631011	-4.11351
Ciclev10025400m.g	scaffold_7:20360085-20395372	239.746	13.8649	-4.112
Ciclev10030245m.g	scaffold_8:23310114-23311691	43.2589	2.50913	-4.10774
Ciclev10009180m.g,Ciclev10010303m.g	scaffold_1:27880115-27883611	590.104	34.2654	-4.10615
-	scaffold_5:1002641-1003231	3070.64	178.519	-4.10439
Ciclev10004677m.g	scaffold_9:24171226-24173238	24.2624	1.41075	-4.10419
Ciclev10008531m.g	scaffold_1:7564442-7569154	28.2768	1.64595	-4.10263
Ciclev10028881m.g	scaffold_8:22859339-22860462	15.1606	0.894485	-4.08312
Ciclev10015182m.g	scaffold_2:31277543-31279358	97.9874	5.88449	-4.05761
Ciclev10013132m.g,Ciclev10013135m.g,Ciclev10013663m.g	scaffold_6:15162387-15168695	71.139	4.27294	-4.05734
Ciclev10019844m.g	scaffold_3:5363038-5365631	101.531	6.10372	-4.05608
Ciclev10016041m.g	scaffold_2:8592978-8594800	9.17091	0.552552	-4.05288
Ciclev10017198m.g	scaffold_2:35270273-35271375	461.824	27.9846	-4.04464
Ciclev10026224m.g	scaffold_7:11513495-11515546	69.4175	4.21873	-4.04042
Ciclev10011211m.g	scaffold_6:23100708-23105430	9.21917	0.560547	-4.03973
Ciclev10012707m.g	scaffold_6:10149054-10152999	2.37496	0.144415	-4.03961
Ciclev10013158m.g	scaffold_6:17009448-17011100	31.0129	1.88582	-4.0396
Ciclev10019961m.g	scaffold_3:42080453-42083713	13.6616	0.832329	-4.03683
Ciclev10009229m.g	scaffold_1:17879965-17882892	85.3902	5.21284	-4.03393
Ciclev10005780m.g	scaffold_9:1539789-1541532	42.9306	2.64172	-4.02246
-	scaffold_5:28289811-28801386	48.8157	3.00501	-4.0219
Ciclev10018030m.g	scaffold_2:14229496-14231959	2.32057	0.143222	-4.01815
Ciclev10020075m.g	scaffold_3:12397236-12399414	3.17604	0.196302	-4.01608
Ciclev10032018m.g	scaffold_4:6175118-6178220	106.965	6.6308	-4.01181
Ciclev10028965m.g	scaffold_8:18824261-18825514	2091.39	130.796	-3.99907
Ciclev10013217m.g	scaffold_6:10367818-10368201	948.858	59.6423	-3.99178
Ciclev10013262m.g,Ciclev10013503m.g	scaffold_6:18374365-18379901	963.393	60.6288	-3.99005
Ciclev10002949m.g	scaffold_5:18167624-18169065	4.71702	0.297913	-3.98491
Ciclev10033507m.g	scaffold_4:7493700-7706546	3.06927	0.195213	-3.97477
Ciclev10011279m.g	scaffold_6:15403653-15407830	7.98324	0.509544	-3.9697

Ciclev10017613m.g	scaffold_2:32301724-32302396	4.93682	0.316112	-3.96507
-	scaffold_1:20351486-20354327	244.162	15.648	-3.96379
Ciclev10028590m.g	scaffold_8:24576172-24578442	804.59	51.6441	-3.96158
Ciclev10023899m.g	scaffold_3:27717304-27722483	0.881215	0.056931	-3.95221
Ciclev10005998m.g	scaffold_9:3839532-3840449	5.92979	0.383483	-3.95075
Ciclev10029124m.g	scaffold_8:2612943-2614424	13.5907	0.881491	-3.94653
Ciclev10011703m.g	scaffold_6:19628726-19632850	46.5862	3.03096	-3.94206
Ciclev10032350m.g	scaffold_4:22656180-22657410	57.2618	3.73694	-3.93764
Ciclev10007916m.g	scaffold_1:4991983-4994456	66.7621	4.36495	-3.93499
Ciclev10011494m.g	scaffold_6:17100555-17102810	408.224	26.783	-3.92997
Ciclev10028165m.g	scaffold_8:22936691-22940430	170.308	11.1861	-3.92837
Ciclev10009740m.g	scaffold_1:14026556-14027565	2712.3	178.558	-3.92505
Ciclev10006445m.g	scaffold_9:2440181-2442253	1.75133	0.117151	-3.90201
Ciclev10032182m.g	scaffold_4:24623710-24627543	240.237	16.1211	-3.89743
Ciclev10011242m.g	scaffold_6:25225200-25228665	1.12644	0.0756223	-3.89681
Ciclev10015210m.g	scaffold_2:32155294-32158290	522.462	35.1111	-3.89533
Ciclev10014832m.g	scaffold_2:21177784-21180989	2.40324	0.161633	-3.89418
Ciclev10008085m.g	scaffold_1:10575666-10577716	1.23546	0.0834929	-3.88725
Ciclev10009152m.g	scaffold_1:18808739-18811363	18.5051	1.25231	-3.88526
Ciclev10012971m.g	scaffold_6:22731981-22732700	41.2447	2.79317	-3.88423
-	scaffold_4:11711487-11728366	80.5877	5.47612	-3.87934
Ciclev10027856m.g	scaffold_8:6659339-6662205	2.65422	0.180598	-3.87744
Ciclev10031831m.g	scaffold_4:23185702-23187916	46.7632	3.18297	-3.87693
Ciclev10025810m.g	scaffold_7:181765-184804	2.0273	0.138162	-3.87513
Ciclev10020060m.g	scaffold_3:32543189-32702382	8.67657	0.592443	-3.87238
Ciclev10011255m.g	scaffold_6:13052096-13054052	2.25044	0.153732	-3.87171
Ciclev10009874m.g	scaffold_1:22632248-22634743	5.32482	0.365825	-3.86351
Ciclev10019920m.g	scaffold_3:1478431-1481737	35.0629	2.41281	-3.86116
Ciclev10002297m.g	scaffold_5:34552215-34553382	2746.73	189.749	-3.85555
Ciclev10022546m.g	scaffold_3:172151-173201	14.5578	1.01011	-3.8492
Ciclev10015566m.g	scaffold_2:26170760-26172226	6.05043	0.421479	-3.8435
Ciclev10016355m.g	scaffold_2:31808374-31811065	21.9942	1.53718	-3.83877
Ciclev10005494m.g	scaffold_9:23071000-23074750	16.4496	1.15409	-3.83323
-	scaffold_5:35146951-35147323	42.6373	2.9945	-3.83173
Ciclev10026538m.g	scaffold_7:20656098-20658251	156.831	11.0217	-3.8308
Ciclev10009975m.g	scaffold_1:22384421-22385632	1893.64	133.466	-3.82661
Ciclev10031447m.g	scaffold_4:982499-984170	609.745	43.0027	-3.82571
Ciclev10025771m.g	scaffold_7:11369605-11373020	3.23669	0.228278	-3.82566
Ciclev10002066m.g	scaffold_5:11336688-11353674	5.11574	0.361649	-3.82228
Ciclev10013139m.g	scaffold_6:17572791-17573553	264.492	18.7381	-3.81918
-	scaffold_5:9849756-9935604	20.4643	1.4513	-3.81769
Ciclev10028738m.g	scaffold_8:23681244-23682676	283.591	20.1305	-3.81635
-	scaffold_1101:964-1396	8.93838	0.636792	-3.81112
Ciclev10027333m.g	scaffold_7:20837583-20839251	965.733	69.175	-3.8033
Ciclev10024795m.g	scaffold_7:4288204-4292989	86.62	6.21966	-3.79979
Ciclev10019664m.g,Ciclev10022787m.g	scaffold_3:19834362-19911599	721.489	51.8696	-3.79802
Ciclev10017714m.g	scaffold_2:30494005-30499118	0.906298	0.0657446	-3.78504
Ciclev10030719m.g	scaffold_4:7885342-7891645	571.065	41.7037	-3.77541
Ciclev10002947m.g	scaffold_5:33399338-33401428	203.224	14.9215	-3.76761
-	scaffold_2:9184409-9185981	63.0942	4.64176	-3.76476
Ciclev10025933m.g	scaffold_7:4997088-4999784	596.361	43.9673	-3.76168
Ciclev10029863m.g	scaffold_8:1597559-1598760	2.9375	0.216646	-3.76118
Ciclev10007378m.g	scaffold_1:5539850-5542980	66.2248	4.89342	-3.75846
Ciclev10001677m.g	scaffold_5:35974953-35979998	759.486	56.7106	-3.74333
Ciclev10004832m.g	scaffold_9:28081759-28084212	1.13206	0.0850047	-3.73527

Ciclev10032181m.g	scaffold_4:24820268-24824732	260.343	19.5804	-3.73293
Ciclev10029439m.g	scaffold_8:19760076-19761961	232.249	17.504	-3.72992
Ciclev10006470m.g	scaffold_9:29368680-29370684	1.83945	0.138968	-3.72645
-	scaffold_7:5283913-5285274	22.302	1.69292	-3.71959
Ciclev10029402m.g	scaffold_8:20200487-20201896	13.6735	1.03879	-3.7184
Ciclev10016434m.g	scaffold_2:12818316-12819910	584.312	44.4749	-3.71568
Ciclev10028495m.g	scaffold_8:23871220-23873313	46.7075	3.56133	-3.71317
Ciclev10016463m.g	scaffold_2:33673906-33675071	438.043	33.5374	-3.70723
Ciclev10020962m.g	scaffold_3:44822522-44823980	19.8686	1.52582	-3.70283
Ciclev10012678m.g	scaffold_6:19964328-19965315	234.771	18.0896	-3.69803
Ciclev10021095m.g	scaffold_3:18466261-18467774	940.768	72.7206	-3.6934
Ciclev10001658m.g	scaffold_5:40728916-40730477	62.4743	4.83359	-3.6921
Ciclev10023029m.g	scaffold_3:45895270-45897033	3054.54	237.225	-3.68663
Ciclev10008127m.g	scaffold_1:25022793-25024741	2.95512	0.22963	-3.68584
Ciclev10005147m.g	scaffold_9:31044658-31047363	20.6153	1.60235	-3.68546
Ciclev10006431m.g	scaffold_9:27352942-27354379	1.97544	0.154275	-3.6786
Ciclev10009761m.g	scaffold_1:25604508-25605857	7.43794	0.582423	-3.67476
Ciclev10010348m.g	scaffold_1:25143730-25144390	15.8614	1.24571	-3.67048
Ciclev10005818m.g	scaffold_9:20182668-20185445	1.8842	0.149244	-3.6582
Ciclev10016725m.g	scaffold_2:32292657-32294714	4.56369	0.361703	-3.65732
Ciclev10012776m.g	scaffold_6:17943467-17944691	105.694	8.37866	-3.65703
Ciclev10026991m.g	scaffold_7:5129293-5133799	0.443942	0.0354021	-3.64846
Ciclev10031201m.g	scaffold_4:23066609-23069480	39.0043	3.1148	-3.64642
Ciclev10024111m.g	scaffold_3:48311653-48313181	29.163	2.33063	-3.64535
Ciclev10013091m.g,Ciclev10013249m.g	scaffold_6:22622445-22624276	88.7209	7.09601	-3.64419
Ciclev10032510m.g	scaffold_4:19622199-19623356	210.639	16.8474	-3.64418
Ciclev10023251m.g	scaffold_3:31553969-31555056	16.4695	1.3201	-3.64108
Ciclev10001701m.g	scaffold_5:35972716-35974167	8.46222	0.682253	-3.63266
Ciclev10022416m.g	scaffold_3:46240095-46240980	7.22893	0.588022	-3.61984
Ciclev10003474m.g	scaffold_5:34941739-34942330	5.45332	0.444967	-3.61537
Ciclev10012421m.g	scaffold_6:22070752-22071850	3.95519	0.323578	-3.61156
Ciclev10001803m.g	scaffold_5:38165020-38166215	2.0886	0.171274	-3.60816
Ciclev10029455m.g	scaffold_8:1103305-1104886	11.7178	0.961946	-3.60661
Ciclev10010254m.g	scaffold_1:7040305-7041553	1.88145	0.154681	-3.60448
Ciclev10013188m.g,Ciclev10013191m.g	scaffold_6:22970177-22976234	870.868	71.8609	-3.59918
Ciclev10024602m.g	scaffold_3:5382489-5385009	7.35617	0.607363	-3.59832
Ciclev10006109m.g	scaffold_9:19673321-19676059	6.53011	0.542922	-3.58829
Ciclev10018699m.g	scaffold_3:36692319-36700292	3.06266	0.25592	-3.58102
Ciclev10016486m.g	scaffold_2:24720358-24721501	16.8189	1.40578	-3.58064
Ciclev10001722m.g	scaffold_5:20746474-20747919	114.636	9.58772	-3.57972
Ciclev10000518m.g	scaffold_5:39934354-39937311	0.908139	0.0759764	-3.57929
Ciclev10013710m.g	scaffold_6:1507854-1509072	122.91	10.302	-3.57661
Ciclev10005144m.g	scaffold_9:25192480-25197226	1.72077	0.144454	-3.57437
Ciclev10032774m.g	scaffold_4:1797536-1798886	3.37986	0.284588	-3.57002
Ciclev10008652m.g	scaffold_1:24583051-24584498	1.5276	0.128944	-3.56645
Ciclev10011382m.g	scaffold_6:21365188-21367260	120.82	10.2238	-3.56285
Ciclev10001048m.g	scaffold_5:39963242-39965562	24.091	2.03931	-3.56234
Ciclev10031811m.g	scaffold_4:1258145-1262635	103.256	8.75123	-3.5606
Ciclev10004558m.g	scaffold_9:15483679-15487436	2.15047	0.182958	-3.55507
Ciclev10032304m.g	scaffold_4:24041592-24043549	254.823	21.7807	-3.54837
Ciclev10026128m.g	scaffold_7:5258044-5259521	268.196	22.9332	-3.54778
Ciclev10013628m.g	scaffold_6:13645122-13648531	1.24672	0.106974	-3.5428
Ciclev10030920m.g	scaffold_4:1277010-1280156	1694.36	145.989	-3.53681
Ciclev10005992m.g	scaffold_9:5417912-5418877	202.085	17.4799	-3.53119

Ciclev10022349m.g	scaffold_3:30290830-30303977	6.4079	0.554431	-3.53077
Ciclev10019012m.g	scaffold_3:12742965-12746586	5.70091	0.493362	-3.53047
Ciclev10031957m.g	scaffold_4:19498805-19500222	2.96655	0.257378	-3.52683
Ciclev10021067m.g	scaffold_3:5360112-5362918	784.059	68.1383	-3.52442
Ciclev10000171m.g	scaffold_5:34929761-34934474	5.0286	0.437747	-3.52199
Ciclev10013244m.g	scaffold_6:7909208-7914618	1.761	0.15589	-3.4978
Ciclev10005926m.g	scaffold_9:27621066-27622194	4.10875	0.364514	-3.49466
Ciclev10003186m.g	scaffold_5:29751-30872	4.67571	0.415139	-3.49352
Ciclev10007249m.g	scaffold_1:5137336-5146463	11.4333	1.01603	-3.49223
Ciclev10019448m.g	scaffold_3:41861778-41864121	22.0694	1.96306	-3.49087
Ciclev10017569m.g	scaffold_2:28282823-28590596	8.53575	0.760748	-3.48803
Ciclev10028011m.g	scaffold_8:3117336-3120914	98.08	8.76269	-3.48451
Ciclev10001676m.g	scaffold_5:34627882-34630017	145.519	13.0066	-3.48389
-	scaffold_5:202124-202497	9.5953	0.859637	-3.48053
-	scaffold_5:40670967-40671733	180.793	16.2345	-3.4772
Ciclev10027587m.g	scaffold_7:8639641-8640373	77.3534	6.95975	-3.47436
-	scaffold_2:28282823-28590596	21.1271	1.91038	-3.46716
Ciclev10013107m.g	scaffold_6:24764241-24764625	5.05435	0.457952	-3.46426
Ciclev10013653m.g	scaffold_6:18506890-18508941	3.73616	0.339703	-3.45921
Ciclev10031048m.g	scaffold_4:24060144-24061905	0.897465	0.0822515	-3.44774
Ciclev10030876m.g	scaffold_4:22796096-22798154	19.2797	1.76705	-3.44767
Ciclev10032029m.g	scaffold_4:1641629-1643936	76.1635	7.00021	-3.44363
Ciclev10021936m.g	scaffold_3:44202967-44205663	424.532	39.0315	-3.44316
Ciclev10019107m.g	scaffold_3:9414403-9419290	76.023	7.01497	-3.43793
Ciclev10015600m.g	scaffold_2:29069901-29071600	138.051	12.7766	-3.43363
Ciclev10026072m.g	scaffold_7:5769059-5771090	3.93368	0.366395	-3.42441
Ciclev10017890m.g	scaffold_2:24687331-24688417	6.98184	0.650689	-3.42357
Ciclev10019279m.g	scaffold_3:50868095-50873164	20.6016	1.92697	-3.41835
Ciclev10009420m.g	scaffold_1:21317417-21318453	19.7453	1.85134	-3.41487
Ciclev10026733m.g	scaffold_7:18561106-18565286	108.395	10.1698	-3.41393
Ciclev10018340m.g	scaffold_2:11478184-11480297	1.2682	0.119214	-3.41115
Ciclev10014502m.g	scaffold_2:10695423-10700095	128.785	12.1249	-3.40892
Ciclev10029512m.g	scaffold_8:4117462-4118362	39.0716	3.69207	-3.40362
Ciclev10022299m.g	scaffold_3:38345734-38347292	20.7599	1.96404	-3.4019
Ciclev10028633m.g	scaffold_8:7890298-7895996	6.3895	0.604627	-3.40159
Ciclev10020552m.g	scaffold_3:31620762-31715908	2.05174	0.194338	-3.4002
Ciclev10016832m.g	scaffold_2:32829645-32831351	143.318	13.6044	-3.39707
Ciclev10011291m.g	scaffold_6:12440025-12443884	5.58869	0.53204	-3.3929
Ciclev10012926m.g	scaffold_6:22794370-22795208	25.2688	2.41175	-3.3892
Ciclev10007294m.g	scaffold_1:21964188-21969517	2.13478	0.204663	-3.38276
Ciclev10021911m.g	scaffold_3:49496617-49501009	1.60237	0.154077	-3.37848
Ciclev10028941m.g	scaffold_8:24502069-24504031	5.82269	0.562145	-3.37267
Ciclev10009191m.g	scaffold_1:1016170-1019117	140.731	13.6031	-3.37094
Ciclev10028185m.g	scaffold_8:24583238-24585924	47.8158	4.62201	-3.3709
Ciclev10016276m.g	scaffold_2:15671428-15672816	705.026	68.1523	-3.37084
Ciclev10031442m.g	scaffold_4:22375081-22378853	70.5788	6.83918	-3.36734
Ciclev10015207m.g	scaffold_2:32184972-32187593	953.737	92.9216	-3.3595
-	scaffold_5:35145671-35146756	23.4341	2.29981	-3.34902
Ciclev10021486m.g	scaffold_3:4182370-4183602	3.25554	0.319891	-3.34724
Ciclev10031707m.g	scaffold_4:20978332-20980853	4.06215	0.399875	-3.34462
Ciclev10004838m.g	scaffold_9:28042023-28044097	34.6335	3.41218	-3.3434
Ciclev10005954m.g	scaffold_9:1134875-1136007	138.131	13.6201	-3.34223
Ciclev10007673m.g	scaffold_1:12900391-12905055	11.9147	1.17608	-3.34069
-	scaffold_5:35145186-35145548	46.4028	4.59725	-3.33537
Ciclev10015870m.g	scaffold_2:35334244-35336841	932.103	92.5147	-3.33274
Ciclev10025952m.g	scaffold_7:2441451-2443391	13.357	1.33034	-3.32773

Ciclev10005534m.g	scaffold_9:4252386-4253603	4.27545	0.426064	-3.32693
Ciclev10009957m.g	scaffold_1:19682378-19682942	445.315	44.3777	-3.32692
Ciclev10010212m.g	scaffold_1:3802591-3805681	18.0001	1.80045	-3.32158
Ciclev10030947m.g	scaffold_4:22714723-22717388	10.021	1.0024	-3.32149
-	scaffold_7:20186480-20187346	7.52973	0.755608	-3.31689
Ciclev10014403m.g	scaffold_2:9161512-9166682	12.2135	1.22578	-3.31671
Ciclev10020141m.g	scaffold_3:48560244-48562027	453.526	45.5259	-3.31643
Ciclev10004905m.g	scaffold_9:30520041-30521726	6.0111	0.605176	-3.3122
Ciclev10007519m.g	scaffold_1:25798675-25801889	210.118	21.1622	-3.31164
Ciclev10010508m.g	scaffold_1:22014946-22015402	927.412	93.47	-3.31064
Ciclev10031929m.g	scaffold_4:6955111-6956891	3.52978	0.356221	-3.30873
Ciclev10005368m.g	scaffold_9:23487944-23490847	1.32797	0.134028	-3.30862
Ciclev10026407m.g,Ciclev10027579m.g	scaffold_7:11062213-11079258	4.73142	0.477624	-3.30833
Ciclev10015331m.g	scaffold_2:6388612-6390719	1.05004	0.106303	-3.30419
Ciclev10027941m.g	scaffold_8:24702274-24706772	8.03689	0.816767	-3.29864
-	scaffold_5:17035068-17035865	18.1467	1.84592	-3.29729
Ciclev10003043m.g	scaffold_5:36023351-36024604	1.64279	0.167735	-3.29189
Ciclev10011062m.g	scaffold_6:21404401-21408079	4.61352	0.471929	-3.28923
Ciclev10014790m.g	scaffold_2:14461691-14464311	72.3695	7.41435	-3.28699
Ciclev10014941m.g	scaffold_2:35505668-35509846	25.9086	2.65557	-3.28634
-	scaffold_7:6147879-6148224	29.0486	2.98995	-3.28028
Ciclev10004474m.g	scaffold_9:543443-546746	390.582	40.2665	-3.27798
Ciclev10023125m.g	scaffold_3:33711467-33712000	13.4845	1.39128	-3.27681
Ciclev10022709m.g	scaffold_3:46088036-46089232	9.76094	1.00861	-3.27465
Ciclev10017881m.g	scaffold_2:29014466-29017706	1.14388	0.118486	-3.27114
Ciclev10019393m.g	scaffold_3:45594729-45597938	0.709122	0.0734634	-3.27094
Ciclev10004664m.g	scaffold_9:4261825-4263993	0.660911	0.0684767	-3.27077
Ciclev10004995m.g	scaffold_9:2962884-2964424	1.24066	0.128736	-3.26863
-	scaffold_3:31734567-31734958	500.428	52.0464	-3.26529
Ciclev10009470m.g	scaffold_1:6647157-6649599	1.25915	0.130975	-3.26509
Ciclev10031467m.g	scaffold_4:3297088-3298858	32.9694	3.43303	-3.26357
Ciclev10010155m.g	scaffold_1:25325244-25331925	1.34608	0.140449	-3.26064
Ciclev10006253m.g	scaffold_9:3789152-3789631	36.9505	3.85679	-3.26012
Ciclev10028947m.g	scaffold_8:21289995-21292877	1.44218	0.150696	-3.25854
Ciclev10006602m.g	scaffold_9:4253668-4255801	1.41894	0.148419	-3.25706
Ciclev10007797m.g	scaffold_1:22175575-22263277	3.40289	0.356513	-3.25474
Ciclev10013296m.g	scaffold_6:18180430-18181409	19.2422	2.01803	-3.25326
Ciclev10014770m.g	scaffold_2:35895661-35898841	135.6	14.222	-3.25316
Ciclev10028046m.g	scaffold_8:22645848-22651938	3.30106	0.34695	-3.25013
Ciclev10025521m.g	scaffold_7:4296122-4300353	4.54742	0.479344	-3.24592
Ciclev10002433m.g	scaffold_5:39379288-39380137	10.7872	1.13808	-3.24465
Ciclev10020447m.g	scaffold_3:21088026-21090566	2.20543	0.233399	-3.24019
Ciclev10017840m.g	scaffold_2:28282823-28590596	16.7239	1.77458	-3.23636
Ciclev10031557m.g	scaffold_4:18230005-18231524	0.865997	0.091923	-3.23586
Ciclev10018481m.g	scaffold_3:50910496-50917526	1.81868	0.193149	-3.2351
Ciclev10029531m.g	scaffold_8:11297753-11299510	15.2057	1.61675	-3.23344
Ciclev10022835m.g	scaffold_3:49993997-49994799	54.6042	5.82812	-3.22791
Ciclev10019202m.g	scaffold_3:42509538-42515993	1.2428	0.133383	-3.21995
Ciclev10001513m.g	scaffold_5:36526057-36528589	13.824	1.48719	-3.21651
Ciclev10028271m.g	scaffold_8:1176549-1180044	1.88448	0.203074	-3.21409
Ciclev10026527m.g	scaffold_7:7337322-7338669	629.423	67.8366	-3.21389
Ciclev10024516m.g	scaffold_3:32543189-32702382	25.5432	2.75518	-3.21272
Ciclev10005761m.g	scaffold_9:2764316-2767059	1.76978	0.191276	-3.20985
Ciclev10013035m.g	scaffold_6:17629347-17629878	3.65954	0.395597	-3.20956
Ciclev10000260m.g	scaffold_5:41432161-41439014	36.6994	3.97745	-3.20584

Ciclev10028286m.g	scaffold_8:8226378-8229284	68.5892	7.4344	-3.20569
Ciclev10021602m.g	scaffold_3:36791206-36793775	6.64309	0.720261	-3.20526
Ciclev10032900m.g	scaffold_4:20007863-20008619	23.8772	2.58987	-3.20468
Ciclev10020530m.g	scaffold_3:50307771-50309441	0.958748	0.104182	-3.20205
Ciclev10025330m.g	scaffold_7:13581499-13583486	1033.6	112.494	-3.19976
Ciclev10002988m.g	scaffold_5:36767716-36768536	278.926	30.3629	-3.1995
Ciclev10000500m.g	scaffold_5:34232950-34237425	134.257	14.6378	-3.19722
Ciclev10030600m.g	scaffold_4:25389336-25392417	1.30822	0.142981	-3.19371
Ciclev10006027m.g	scaffold_9:14114993-14116304	10.7847	1.18203	-3.18964
Ciclev10022349m.g	scaffold_3:30290830-30303977	16.2464	1.78111	-3.18927
-	scaffold_7:2922899-2923416	140.775	15.4388	-3.18876
Ciclev10013109m.g	scaffold_6:15817496-15818342	2.56179	0.282509	-3.18078
Ciclev10016932m.g	scaffold_2:33707330-33709334	5.05407	0.559313	-3.17572
Ciclev10026775m.g	scaffold_7:11294376-11295280	1009.63	111.96	-3.17277
Ciclev10019694m.g	scaffold_3:1582674-1586208	15.3239	1.71276	-3.16139
Ciclev10014240m.g	scaffold_2:6295897-6302897	3.33145	0.373723	-3.15611
Ciclev10020489m.g	scaffold_3:45087591-45089434	7.62334	0.856715	-3.15354
Ciclev10023118m.g	scaffold_3:9744902-9745821	15.9588	1.80406	-3.14503
Ciclev10021450m.g	scaffold_3:42357954-42361557	255.559	28.9467	-3.14219
Ciclev10028014m.g	scaffold_8:8656194-8658247	1.33297	0.152589	-3.12692
Ciclev10004929m.g	scaffold_9:21034568-21037653	3.93094	0.453182	-3.11671
Ciclev10011932m.g	scaffold_6:15054580-15056184	44.1456	5.09375	-3.11547
Ciclev10008653m.g	scaffold_1:24619125-24621953	764.094	88.4941	-3.1101
Ciclev10015040m.g	scaffold_2:29449881-29459747	36.834	4.26614	-3.11004
Ciclev10017232m.g	scaffold_2:21031587-21032341	34.8438	4.04152	-3.10793
Ciclev10021076m.g	scaffold_3:46474776-46476646	1.9033	0.221277	-3.10458
Ciclev10009900m.g	scaffold_1:4930631-4931370	121.373	14.1109	-3.10456
Ciclev10008808m.g	scaffold_1:3793875-3798310	62.8161	7.31382	-3.10244
Ciclev10026322m.g	scaffold_7:3842848-3844735	28.046	3.2733	-3.09898
Ciclev10002523m.g	scaffold_5:43186617-43190241	132.041	15.4268	-3.09748
Ciclev10009681m.g	scaffold_1:173462-175858	341.094	39.8938	-3.09594
Ciclev10020532m.g	scaffold_3:50242162-50246469	1.44	0.168548	-3.09484
Ciclev10031199m.g	scaffold_4:23940022-23941837	4.1596	0.486941	-3.09462
Ciclev10019502m.g	scaffold_3:42928892-42932215	30.3116	3.55871	-3.09044
Ciclev10015869m.g	scaffold_2:10298470-10299566	1.72407	0.202425	-3.09036
Ciclev10005561m.g	scaffold_9:17424109-17425707	26.2106	3.07801	-3.09008
Ciclev10021357m.g	scaffold_3:8457314-8464161	1.44242	0.169473	-3.08936
Ciclev10031789m.g	scaffold_4:1297726-1299898	29.6504	3.48485	-3.08888
Ciclev10005374m.g	scaffold_9:3464275-3467426	10.4126	1.22548	-3.08691
Ciclev10006512m.g	scaffold_9:26668974-26670807	13.5337	1.5929	-3.08683
Ciclev10032218m.g	scaffold_4:24412331-24414624	13.9202	1.64208	-3.08358
Ciclev10004556m.g	scaffold_9:30906256-30912952	0.655997	0.0774523	-3.08231
Ciclev10005833m.g	scaffold_9:7770683-7771919	8.58585	1.01457	-3.0811
Ciclev10026136m.g	scaffold_7:6084603-6087800	1.54159	0.183171	-3.07316
Ciclev10026015m.g	scaffold_7:4103298-4105423	0.853669	0.101574	-3.07114
Ciclev10017684m.g	scaffold_2:14701585-14702197	4.92608	0.587046	-3.0689
Ciclev10008801m.g	scaffold_1:8854441-8858300	16.2474	1.93644	-3.06873
Ciclev10031332m.g	scaffold_4:19249944-19251830	5.09402	0.607168	-3.06864
Ciclev10028764m.g	scaffold_8:4404811-4408202	1.02463	0.122841	-3.06024
Ciclev10026269m.g	scaffold_7:20888715-20891804	1.43442	0.172111	-3.05905
-	scaffold_2:6282870-6283521	36.155	4.3437	-3.0572
Ciclev10020006m.g	scaffold_3:8684447-8686118	14.0323	1.68656	-3.05659
Ciclev10023729m.g	scaffold_3:47635843-47636167	8.22285	0.990872	-3.05287
Ciclev10019147m.g	scaffold_3:2939619-2941934	39.8221	4.79898	-3.05277
Ciclev10016805m.g	scaffold_2:9048500-9051025	1.03146	0.124315	-3.05261
Ciclev10024134m.g	scaffold_3:1221448-1222315	2.04966	0.248194	-3.04584

Ciclev10012401m.g	scaffold_6:12257536-12259103	1.65183	0.200028	-3.04579
Ciclev10018054m.g	scaffold_2:28282823-28590596	7.76022	0.943088	-3.04063
Ciclev10022068m.g	scaffold_3:9868143-9869414	41.6453	5.06525	-3.03945
Ciclev10015507m.g	scaffold_2:9927539-9930839	0.981495	0.120063	-3.03119
Ciclev10013713m.g	scaffold_6:14918021-15036696	1.12787	0.138167	-3.02911
Ciclev10007117m.g	scaffold_9:11576287-11715575	2.91802	0.358218	-3.02608
Ciclev10003620m.g	scaffold_5:41115967-41117289	58.5161	7.18861	-3.02505
Ciclev10025408m.g	scaffold_7:15707974-15709919	37.9904	4.66996	-3.02415
Ciclev10018228m.g	scaffold_2:26268587-26269460	364.702	44.8858	-3.02239
Ciclev10028943m.g	scaffold_8:1169450-1171206	1.35868	0.167287	-3.02182
Ciclev10001956m.g	scaffold_5:15762363-15766976	894.754	110.175	-3.0217
Ciclev10003865m.g	scaffold_5:32404311-32420681	0.410005	0.0505304	-3.02042
Ciclev10009011m.g	scaffold_1:3822952-3824251	6.80769	0.839352	-3.01982
Ciclev10011453m.g	scaffold_6:17915203-17919250	2.23795	0.27614	-3.01871
Ciclev10012818m.g	scaffold_6:19929535-19930469	28.058	3.46364	-3.01805
Ciclev10028401m.g	scaffold_8:20910212-20913273	3.07897	0.380159	-3.01777
Ciclev10033393m.g	scaffold_4:6445702-6447613	5.18505	0.640699	-3.01664
Ciclev10026710m.g	scaffold_7:7839320-7840234	303.843	37.5469	-3.01656
Ciclev10028058m.g	scaffold_8:2358346-2360716	6.78627	0.83896	-3.01594
Ciclev10029376m.g	scaffold_8:18110127-18117822	2.10698	0.261077	-3.01263
Ciclev10001308m.g	scaffold_5:34257704-34260311	47.7769	5.98442	-2.99703
Ciclev10012444m.g	scaffold_6:20135561-20136831	3.20755	0.403492	-2.99086
Ciclev10021994m.g	scaffold_3:47882117-47883277	31.2148	3.93242	-2.98874
Ciclev10033273m.g	scaffold_4:7885342-7891645	16.117	2.03792	-2.98341
Ciclev10030930m.g	scaffold_4:22697055-22699336	2.67279	0.338458	-2.9813
Ciclev10006049m.g	scaffold_9:1724964-1727375	59.9841	7.61648	-2.97738
Ciclev10016828m.g	scaffold_2:4660423-4661352	92.0408	11.7147	-2.97395
Ciclev10029932m.g	scaffold_8:8756363-8760308	61.4947	7.84412	-2.97078
Ciclev10005200m.g	scaffold_9:30442074-30445262	22.1309	2.8284	-2.968
Ciclev10021285m.g	scaffold_3:5253280-5254755	546.843	70.0467	-2.96474
Ciclev10008063m.g	scaffold_1:2660827-2662640	3.10231	0.397407	-2.96465
-	scaffold_2:28282823-28590596	18.1521	2.33981	-2.95567
Ciclev10018045m.g	scaffold_2:32460490-32463102	213.89	27.5825	-2.95505
Ciclev10015826m.g	scaffold_2:28714734-28717185	17.6192	2.27279	-2.95461
Ciclev10010110m.g	scaffold_1:24121895-24122708	23.0195	2.97396	-2.9524
Ciclev10024638m.g	scaffold_3:15994162-16000998	5.48569	0.71159	-2.94655
Ciclev10000599m.g	scaffold_5:29840773-29846737	9.06489	1.17727	-2.94484
Ciclev10023800m.g	scaffold_3:42757707-42759180	0.924319	0.120105	-2.94409
Ciclev10030403m.g	scaffold_8:4408513-4409614	1.0876	0.141627	-2.94098
Ciclev10006198m.g	scaffold_9:29545502-29547180	411.278	53.5756	-2.94046
-	scaffold_5:2882144-2882572	9.91102	1.2964	-2.93452
Ciclev10009650m.g	scaffold_1:7296550-7297509	273.204	35.9449	-2.92612
Ciclev10021069m.g	scaffold_3:21579108-21583976	16.8341	2.21598	-2.92538
Ciclev10013224m.g	scaffold_6:11107416-11108496	10.8922	1.43439	-2.92478
Ciclev10028101m.g	scaffold_8:8041480-8043984	158.311	20.9028	-2.921
Ciclev10013527m.g	scaffold_6:16946899-16949118	2.01094	0.265795	-2.91948
Ciclev10009014m.g	scaffold_1:25222374-25226019	3.67825	0.486572	-2.9183
Ciclev10031496m.g	scaffold_4:16426858-16429926	107.106	14.1761	-2.9175
-	scaffold_9:24267838-24268841	4.43606	0.58731	-2.91709
Ciclev10004499m.g	scaffold_9:11150757-11155385	1.69862	0.224923	-2.91686
Ciclev10029356m.g	scaffold_8:22821613-22822718	5.53201	0.733669	-2.9146
Ciclev10009942m.g	scaffold_1:27908366-27909139	22.3486	2.97222	-2.91057
Ciclev10027164m.g	scaffold_7:18387836-18390858	2.62222	0.349144	-2.90889
Ciclev10023841m.g	scaffold_3:7306771-7311180	0.765114	0.102142	-2.9051

Ciclev10003207m.g,Ciclev10003500m.g,Ciclev10003621m.g	scaffold_5:26144194-26153441	103.032	13.7789	-2.90257
Ciclev10006357m.g	scaffold_9:647899-648331	104.794	14.0284	-2.90113
Ciclev10020033m.g	scaffold_3:1731127-1732943	267.995	35.9948	-2.89635
Ciclev10001122m.g	scaffold_5:15762363-15766976	23.0182	3.09951	-2.89266
Ciclev10014882m.g	scaffold_2:29616639-29620120	15.0318	2.02875	-2.88935
Ciclev10033969m.g	scaffold_4:24314605-24316379	2.69686	0.364074	-2.88898
Ciclev10022077m.g	scaffold_3:18239858-18242696	12.0719	1.63126	-2.88759
Ciclev10025730m.g	scaffold_7:20111321-20115683	14.3567	1.94319	-2.88522
-	scaffold_3:7848597-7849318	25.825	3.49626	-2.88489
Ciclev10029965m.g	scaffold_8:2427036-2430311	1.9735	0.267472	-2.8833
Ciclev10015770m.g	scaffold_2:23243925-23246266	62.0072	8.40954	-2.88234
Ciclev10011864m.g	scaffold_6:8798479-8801299	40.2564	5.46006	-2.88223
Ciclev10012618m.g	scaffold_6:21681865-21683202	301.866	41.1794	-2.87391
-	scaffold_5:9849756-9935604	6.9034	0.942049	-2.87343
Ciclev10019990m.g	scaffold_3:33956348-33961039	16.4847	2.2501	-2.87307
Ciclev10006520m.g	scaffold_9:25829140-25830361	53.5808	7.31517	-2.87275
Ciclev10020894m.g	scaffold_3:8545461-8547035	7.09457	0.970474	-2.86995
Ciclev10017094m.g	scaffold_2:17955749-17956413	7.10088	0.975171	-2.86427
Ciclev10012698m.g	scaffold_6:20636355-20637195	19.7397	2.71187	-2.86374
Ciclev10019672m.g	scaffold_3:47697603-47699546	88.2072	12.1356	-2.86165
Ciclev10000098m.g	scaffold_5:26641956-26646157	2.2979	0.317077	-2.85741
Ciclev10019376m.g	scaffold_3:11399184-11403123	127.528	17.5998	-2.85718
Ciclev10012000m.g	scaffold_6:14402663-14405661	84.7356	11.7	-2.85646
Ciclev10004870m.g	scaffold_9:28110540-28112377	2.6462	0.365461	-2.85613
Ciclev10016432m.g	scaffold_2:28282823-28590596	8.93739	1.24013	-2.84936
Ciclev10015825m.g	scaffold_2:35525984-35527550	2.16276	0.300353	-2.84814
Ciclev10021038m.g	scaffold_3:1054256-1056078	131.439	18.2645	-2.84728
Ciclev10013952m.g	scaffold_10:49950-57109	20.0793	2.79278	-2.84593
Ciclev10005134m.g	scaffold_9:2467102-2468727	3.02654	0.421623	-2.84364
Ciclev10018818m.g	scaffold_3:5428605-5435876	162.743	22.6886	-2.84255
Ciclev10002708m.g	scaffold_5:42259212-42259753	3.66436	0.510877	-2.84251
Ciclev10026272m.g	scaffold_7:6629035-6630243	198.878	27.7402	-2.84184
Ciclev10027191m.g	scaffold_7:175856-176522	69.7769	9.74129	-2.84056
Ciclev10032760m.g	scaffold_4:1149635-1152481	143.335	20.0767	-2.8358
Ciclev10000629m.g	scaffold_5:1084199-1143725	2.05959	0.289557	-2.83044
Ciclev10025451m.g	scaffold_7:2084849-2088443	0.662099	0.0935239	-2.82364
Ciclev10031574m.g	scaffold_4:19662367-19664575	78.627	11.1143	-2.82261
Ciclev10014228m.g	scaffold_2:7530826-7533742	1.9728	0.279061	-2.82159
Ciclev10001578m.g	scaffold_5:42170317-42173218	2.92805	0.415292	-2.81774
Ciclev10007179m.g	scaffold_9:11355736-11362565	0.251992	0.03578	-2.81615
Ciclev10001697m.g	scaffold_5:33068049-33069093	1.47996	0.210231	-2.81551
Ciclev10021652m.g	scaffold_3:5240107-5241300	91.9552	13.0912	-2.81233
Ciclev10019330m.g	scaffold_3:42308560-42311669	1.61425	0.230062	-2.81077
Ciclev10006491m.g	scaffold_9:21186802-21188959	1.55268	0.221488	-2.80946
Ciclev10025377m.g	scaffold_7:14727275-14729453	1.36023	0.1942	-2.80824
-	scaffold_2:6282240-6282664	55.4202	7.92025	-2.80679
Ciclev10014529m.g	scaffold_2:7155713-7161520	0.571899	0.0817521	-2.80643
Ciclev10032987m.g	scaffold_4:22161570-22162681	11.6339	1.66319	-2.80631
-	scaffold_5:29738549-29740164	3.83466	0.548553	-2.8054
Ciclev10030798m.g	scaffold_4:21982830-21986241	0.992513	0.142184	-2.80332
Ciclev10017055m.g	scaffold_2:27638162-27639131	1.62936	0.233419	-2.80331
Ciclev10024060m.g	scaffold_3:30728988-30729637	3.04748	0.436613	-2.80319
-	scaffold_4:11529684-11529991	19.5544	2.80693	-2.80042
Ciclev10012752m.g	scaffold_6:25013158-25014095	470.334	67.5151	-2.8004

Ciclev10020453m.g	scaffold_3:46920513-46923179	77.1701	11.0941	-2.79825
Ciclev10021322m.g	scaffold_3:3205519-3208111	2.80534	0.403348	-2.79808
-	scaffold_5:17033889-17034483	10.3109	1.48658	-2.7941
Ciclev10012033m.g	scaffold_6:8413958-8415516	0.87156	0.125692	-2.79371
Ciclev10030698m.g	scaffold_4:24651072-24654533	14.7863	2.1348	-2.79209
Ciclev10019452m.g	scaffold_3:46349126-46351795	2.1692	0.314248	-2.78719
Ciclev10027907m.g	scaffold_8:17764711-17767812	4.5938	0.6659	-2.78631
Ciclev10011710m.g	scaffold_6:19531984-19534809	0.574208	0.083293	-2.78531
Ciclev10031202m.g	scaffold_4:15371023-15373637	0.652238	0.0946329	-2.78499
Ciclev10009291m.g	scaffold_1:14007945-14009687	3.03242	0.440306	-2.78389
Ciclev10010118m.g	scaffold_1:28063113-28066620	2.89845	0.421448	-2.78186
Ciclev10014435m.g	scaffold_2:34258298-34262758	1.55482	0.226104	-2.78169
Ciclev10007394m.g	scaffold_1:24741259-24748937	1.35733	0.197454	-2.78118
Ciclev10000411m.g	scaffold_5:40046649-40048839	0.50098	0.0730389	-2.77802
Ciclev10014951m.g	scaffold_2:36008353-36010461	47.8454	6.98158	-2.77675
Ciclev10001191m.g	scaffold_5:13273930-13275652	139.058	20.3033	-2.7759
Ciclev10023752m.g	scaffold_3:43388179-43391091	0.833078	0.121819	-2.77372
Ciclev10017492m.g	scaffold_2:4340637-4345933	0.821095	0.12026	-2.77139
Ciclev10032842m.g	scaffold_4:19377410-19378601	403.947	59.1875	-2.7708
Ciclev10019616m.g	scaffold_3:1751879-1754219	13.419	1.9673	-2.76999
Ciclev10024293m.g	scaffold_3:25657663-25663490	0.272272	0.0400115	-2.76656
Ciclev10004908m.g	scaffold_9:31207178-31209810	0.608081	0.0894726	-2.76475
Ciclev10020714m.g	scaffold_3:16767-18635	21.786	3.20956	-2.76296
-	scaffold_5:40671871-40672573	215.209	31.7057	-2.76292
Ciclev10028315m.g	scaffold_8:12953963-12968792	83.8874	12.3639	-2.76232
Ciclev10026388m.g	scaffold_7:7794727-7795624	2.66146	0.392966	-2.75974
Ciclev10002288m.g	scaffold_5:40752810-40753702	4.598	0.67947	-2.75852
Ciclev10021209m.g	scaffold_3:42205628-42210257	1.98315	0.293298	-2.75736
Ciclev10012209m.g	scaffold_6:21549744-21550875	3.61727	0.535405	-2.7562
Ciclev10026501m.g	scaffold_7:529835-530825	343.425	50.8334	-2.75614
Ciclev10002505m.g	scaffold_5:25188306-25547508	127.557	18.894	-2.75515
Ciclev10030714m.g	scaffold_4:3341907-3344935	5.99138	0.889583	-2.75169
Ciclev10014549m.g,Ciclev10017919m.g,Ciclev10018302m.g,Ciclev10018314m.g	scaffold_2:28282823-28590596	35.1935	5.22716	-2.75121
Ciclev10018672m.g	scaffold_3:49116717-49121222	135.909	20.2469	-2.74688
Ciclev10012045m.g	scaffold_6:15377565-15379102	4.94402	0.738513	-2.74299
Ciclev10008351m.g	scaffold_1:20457914-20462359	7.94547	1.18707	-2.74273
Ciclev10008930m.g	scaffold_1:1051154-1052947	695.777	104.083	-2.74089
-	scaffold_8:19146289-19210612	9.47345	1.41731	-2.74073
Ciclev10016018m.g	scaffold_2:35798995-35801167	0.656037	0.0984144	-2.73684
Ciclev10033712m.g	scaffold_4:24327472-24328664	1.21165	0.182221	-2.73321
Ciclev10026400m.g	scaffold_7:9976620-9980176	6.31972	0.950456	-2.73317
Ciclev10002231m.g	scaffold_5:34665210-34666402	19.9953	3.009	-2.73231
-	scaffold_2:8823026-8823925	18.2497	2.75334	-2.72862
Ciclev10024240m.g	scaffold_3:27512874-27514002	1.11276	0.16818	-2.72607
Ciclev10003687m.g	scaffold_5:27541639-27543184	42.7434	6.46259	-2.72552
Ciclev10005633m.g	scaffold_9:26509544-26511289	1.32498	0.200397	-2.72504
Ciclev10032355m.g	scaffold_4:1849347-1851693	4.29728	0.650581	-2.72362
Ciclev10015005m.g	scaffold_2:10424778-10472483	45.9988	6.9659	-2.72321
Ciclev10028798m.g	scaffold_8:2763024-2764293	4.9939	0.756565	-2.72263
Ciclev10005387m.g	scaffold_9:532816-535001	9.10956	1.38008	-2.72263
Ciclev10012468m.g	scaffold_6:20276330-20278559	1.20215	0.182162	-2.72233
Ciclev10023970m.g	scaffold_3:19328523-19332329	1.03419	0.156743	-2.72203
Ciclev10004465m.g	scaffold_9:27282486-27287560	43.2481	6.56588	-2.71958
Ciclev10005533m.g	scaffold_9:6747773-6753151	11.7791	1.79209	-2.71651

Ciclev10007376m.g	scaffold_1:20841651-20844762	3.86864	0.589757	-2.71363
Ciclev10008935m.g	scaffold_1:4192564-4194432	4.44734	0.681476	-2.70621
Ciclev10030744m.g	scaffold_4:2020042-2025300	2.00761	0.30783	-2.70527
Ciclev10008812m.g	scaffold_1:18270817-18273015	369.681	56.6918	-2.70507
-	scaffold_2:21693952-21694463	6.15928	0.944926	-2.70449
Ciclev10021699m.g	scaffold_3:43420186-43422432	17.0086	2.61193	-2.70308
Ciclev10029135m.g	scaffold_8:177830-178744	1.48595	0.228423	-2.70161
Ciclev10004431m.g	scaffold_9:27464704-27467529	7.59031	1.16802	-2.70009
-	scaffold_569:3035-3661	14.1865	2.18418	-2.69935
Ciclev10024509m.g	scaffold_3:19401305-19403096	2.94296	0.453503	-2.69809
Ciclev10030797m.g	scaffold_4:23618423-23625625	4.99825	0.771475	-2.69573
Ciclev10016684m.g	scaffold_2:27460832-27461914	68.5679	10.5855	-2.69544
Ciclev10028214m.g	scaffold_8:1080301-1085905	102.646	15.8521	-2.69493
Ciclev10000393m.g	scaffold_5:15822059-15834446	5.3377	0.824416	-2.69477
Ciclev10025414m.g	scaffold_7:3302375-3305817	2.84224	0.439757	-2.69225
-	scaffold_5:29742802-29743276	6.94697	1.07487	-2.69222
Ciclev10029663m.g	scaffold_8:5029938-5030960	39.0175	6.03927	-2.69168
Ciclev10003899m.g	scaffold_5:24926169-25131690	126.335	19.558	-2.69142
Ciclev10011300m.g	scaffold_6:11514709-11519693	2.13564	0.330707	-2.69105
Ciclev10025235m.g	scaffold_7:20271437-20274020	9.55492	1.47972	-2.69092
Ciclev10023485m.g	scaffold_3:33388539-33592383	3.3159	0.515526	-2.68528
Ciclev10010000m.g	scaffold_1:27397739-27398620	52.2927	8.14607	-2.68243
Ciclev10029154m.g	scaffold_8:4466846-4468794	3.4884	0.545167	-2.6778
Ciclev10028264m.g	scaffold_8:4292179-4370290	2.74506	0.42927	-2.67688
Ciclev10031919m.g	scaffold_4:24631410-24633675	46.0128	7.19572	-2.67682
Ciclev10021778m.g	scaffold_3:47931341-47933843	11.782	1.8433	-2.67622
Ciclev10006656m.g	scaffold_9:26117969-26159098	87.7177	13.7809	-2.6702
Ciclev10028419m.g	scaffold_8:23919567-23921211	3.62849	0.570328	-2.66951
Ciclev10025875m.g	scaffold_7:4575502-4577125	7.30732	1.15029	-2.66734
Ciclev10012712m.g	scaffold_6:3654838-3656896	221.594	34.8878	-2.66712
Ciclev10019664m.g	scaffold_3:19834362-19911599	30.9178	4.87514	-2.66492
Ciclev10018839m.g	scaffold_3:37156877-37163307	0.786091	0.124252	-2.66143
Ciclev10007548m.g	scaffold_1:20535940-20543308	10.3976	1.6438	-2.66115
Ciclev10026703m.g	scaffold_7:10853486-10855215	3.37875	0.535159	-2.65845
Ciclev10020212m.g	scaffold_3:32982611-32984120	14.1938	2.24859	-2.65816
Ciclev10009825m.g	scaffold_1:3436725-3439978	41.4943	6.59406	-2.65367
Ciclev10010881m.g	scaffold_483:9442-11246	74.1351	11.7908	-2.6525
Ciclev10026702m.g	scaffold_7:5344867-5345946	78.1641	12.4361	-2.65197
-	scaffold_5:2578645-2579654	21.4511	3.41623	-2.65057
-	scaffold_3:11687448-12154789	24.3351	3.87965	-2.64904
Ciclev10022730m.g	scaffold_3:46450125-46451301	639.858	102.503	-2.64208
Ciclev10012557m.g	scaffold_6:23093342-23094697	1.84044	0.29649	-2.634
Ciclev10017244m.g	scaffold_2:7477429-7479641	12.4937	2.01413	-2.63298
Ciclev10004981m.g	scaffold_9:28166148-28170644	1.06813	0.1725	-2.63042
Ciclev10022388m.g	scaffold_3:7042031-7042805	3.6218	0.585316	-2.62942
Ciclev10000964m.g	scaffold_5:37473063-37478070	21.9462	3.55103	-2.62766
Ciclev10019056m.g	scaffold_3:49802635-49805960	17.3591	2.81521	-2.62438
Ciclev10020144m.g	scaffold_3:47189458-47191383	2.3493	0.381601	-2.62209
-	scaffold_5:2664261-2664623	11.3178	1.8389	-2.62167
Ciclev10003662m.g	scaffold_5:35059782-35061627	16.8133	2.73229	-2.62142
Ciclev10004276m.g	scaffold_9:25603643-25606763	0.823794	0.134039	-2.61962
Ciclev10018346m.g	scaffold_2:11063249-11063663	3.19603	0.520105	-2.6194
Ciclev10020627m.g	scaffold_3:238937-243369	2.9265	0.476294	-2.61925
-	scaffold_5:38002039-38002460	69.7279	11.3759	-2.61575
Ciclev10012100m.g	scaffold_6:23907307-23908869	339.331	55.495	-2.61226
Ciclev10023468m.g	scaffold_3:32543189-32702382	4.43015	0.725283	-2.61074

Ciclev10027873m.g	scaffold_8:6834486-6837762	937.386	153.823	-2.60737
Ciclev10001072m.g	scaffold_5:37105295-37107915	13.7177	2.25423	-2.60533
Ciclev10019457m.g	scaffold_3:49437523-49445438	64.3214	10.5783	-2.60419
Ciclev10031451m.g	scaffold_4:24609446-24612338	8.05481	1.32741	-2.60124
Ciclev10002672m.g	scaffold_5:27365221-27366726	16.1114	2.65578	-2.60088
Ciclev10019272m.g	scaffold_3:49346936-49350957	45.6811	7.53081	-2.60072
-	scaffold_5:655534-811195	88.9473	14.6694	-2.60014
Ciclev10022355m.g	scaffold_3:45800027-45801135	2.34768	0.387924	-2.59739
Ciclev10005146m.g	scaffold_9:270955-272594	34.5968	5.71786	-2.59709
Ciclev10019173m.g	scaffold_3:19253239-19255491	20.9713	3.47137	-2.59484
-	scaffold_5:1503259-1988798	5.88003	0.973926	-2.59394
Ciclev10016648m.g	scaffold_2:22386726-22388706	133.686	22.197	-2.59041
-	scaffold_1:6460693-6460932	84.9936	14.1305	-2.58854
Ciclev10005745m.g	scaffold_9:660308-663410	21.4478	3.57556	-2.58459
Ciclev10028078m.g	scaffold_8:24554321-24561746	79.2318	13.2192	-2.58344
Ciclev10032937m.g	scaffold_4:19956091-19956932	15.9989	2.67937	-2.57801
Ciclev10009275m.g	scaffold_1:3253345-3254158	1.3616	0.228146	-2.57727
Ciclev10015468m.g	scaffold_2:29113360-29116904	120.699	20.2312	-2.57676
Ciclev10030625m.g	scaffold_4:2261149-2266209	2.89111	0.484746	-2.57632
Ciclev10028539m.g	scaffold_8:232122-235806	30.7338	5.1535	-2.5762
Ciclev10003661m.g	scaffold_5:39592381-39593026	4.18397	0.701911	-2.57551
Ciclev10009229m.g	scaffold_1:17879965-17882892	38.8695	6.52767	-2.574
Ciclev10011857m.g	scaffold_6:19119417-19123027	13.0754	2.20025	-2.57112
Ciclev10028674m.g	scaffold_8:16674700-16678943	22.0477	3.71284	-2.57004
Ciclev10027954m.g	scaffold_8:12800121-12845493	717.67	120.886	-2.56967
Ciclev10022410m.g	scaffold_3:48051030-48051986	43.0421	7.25258	-2.56918
Ciclev10007505m.g	scaffold_1:25193363-25195811	7.47188	1.25942	-2.56871
Ciclev10002743m.g	scaffold_5:36634134-36636064	507.98	85.641	-2.5684
Ciclev10031654m.g	scaffold_4:19460014-19462157	8.7256	1.47147	-2.568
Ciclev10017454m.g	scaffold_2:34672458-34672887	11.5697	1.95313	-2.56649
Ciclev10033863m.g	scaffold_4:23612265-23614044	3.88359	0.656548	-2.56442
Ciclev10030742m.g	scaffold_4:25267552-25269940	1.52581	0.258141	-2.56334
Ciclev10025530m.g	scaffold_7:1753132-1754952	9.82066	1.6652	-2.56012
Ciclev10015945m.g	scaffold_2:9224783-9226076	44.0883	7.48236	-2.55883
Ciclev10012382m.g	scaffold_6:24923533-24935909	91.4012	15.5316	-2.55701
Ciclev10019718m.g	scaffold_3:31555090-31557262	10.8374	1.84655	-2.55312
Ciclev10009338m.g	scaffold_1:25369130-25371445	13.2372	2.25849	-2.55116
-	scaffold_6:10361361-10361701	37.4148	6.40547	-2.54623
Ciclev10012611m.g	scaffold_6:25033675-25035365	5.1447	0.881119	-2.54568
Ciclev10005459m.g	scaffold_9:5896850-5899769	5.70067	0.976862	-2.5449
Ciclev10023696m.g	scaffold_3:36363009-36553824	2.9687	0.508965	-2.54419
Ciclev10022171m.g	scaffold_3:47460751-47463827	8.5224	1.46179	-2.54352
Ciclev10002522m.g	scaffold_5:39175823-39179944	2.88472	0.495179	-2.54241
Ciclev10028959m.g	scaffold_8:445468-446936	257.824	44.262	-2.54224
Ciclev10000363m.g	scaffold_5:34474998-34482201	46.7262	8.02407	-2.54182
Ciclev10025637m.g	scaffold_7:16657828-16659304	3.18495	0.547807	-2.53953
Ciclev10029158m.g	scaffold_8:2874894-2876912	122.881	21.1591	-2.53792
Ciclev10030803m.g	scaffold_4:312145-317398	42.8771	7.38968	-2.53662
Ciclev10012544m.g	scaffold_6:17786943-17788933	2.3773	0.41063	-2.53341
Ciclev10005288m.g	scaffold_9:28641385-28643731	70.0255	12.1309	-2.52919
Ciclev10002306m.g	scaffold_5:38738186-38739919	3.33953	0.579448	-2.5269
Ciclev10027751m.g	scaffold_8:18671466-18676601	6.61103	1.1486	-2.52499
Ciclev10028711m.g	scaffold_8:8206052-8208375	6.4395	1.11909	-2.52463
Ciclev10017229m.g	scaffold_2:11497514-11498250	1.74191	0.303001	-2.52328
Ciclev10011650m.g	scaffold_6:17402876-17405928	276.058	48.0517	-2.52231
Ciclev10017728m.g	scaffold_2:28282823-28590596	14.2943	2.49945	-2.51576

Ciclev10006927m.g	scaffold_9:30447133-30449719	15.1595	2.65675	-2.51248
Ciclev10017805m.g,Ciclev10018319m.g	scaffold_2:6554459-6563743	3.81949	0.669717	-2.51176
Ciclev10011179m.g	scaffold_6:22998183-23002700	17.009	2.98772	-2.50918
-	scaffold_5:2607070-2607287	299.548	52.6517	-2.50823
Ciclev10015613m.g	scaffold_2:11920048-11922578	6.74001	1.18534	-2.50745
Ciclev10032995m.g	scaffold_4:16694754-16695286	9.51029	1.67278	-2.50724
Ciclev10015896m.g	scaffold_2:33303510-33305516	5.22069	0.918401	-2.50704
Ciclev10030577m.g,Ciclev10031220m.g	scaffold_4:22564847-22582276	9.44376	1.66172	-2.50668
Ciclev10012164m.g	scaffold_6:19753977-19756116	88.6005	15.593	-2.50641
Ciclev10015707m.g	scaffold_2:34052179-34054960	19.4172	3.42749	-2.50211
Ciclev10033218m.g	scaffold_4:24994951-24995502	9.28164	1.64208	-2.49885
Ciclev10013618m.g	scaffold_6:1039888-1047952	0.209242	0.0370952	-2.49587
Ciclev10005615m.g	scaffold_9:3331077-3332205	0.896294	0.15931	-2.49214
Ciclev10015033m.g	scaffold_2:24926665-24929114	0.778704	0.138528	-2.4909
Ciclev10015783m.g	scaffold_2:4895612-4898595	16.0492	2.85587	-2.4905
Ciclev10014038m.g	scaffold_2:31811546-31820596	0.880505	0.156903	-2.48846
Ciclev10002654m.g	scaffold_5:40652360-40653042	3.97453	0.709683	-2.48554
Ciclev10025158m.g	scaffold_7:16832246-16834201	2.78854	0.49808	-2.48506
Ciclev10028348m.g	scaffold_8:4918555-4921989	2.18859	0.391062	-2.48453
Ciclev10024783m.g	scaffold_7:240863-250983	4.66573	0.833717	-2.48447
Ciclev10020360m.g	scaffold_3:47549852-47553563	5.93278	1.06049	-2.48398
Ciclev10028017m.g	scaffold_8:21771140-21777001	1.38476	0.247735	-2.48277
Ciclev10026847m.g	scaffold_7:13348931-13360472	14.4292	2.58887	-2.47859
Ciclev10025017m.g	scaffold_7:18815388-18817728	0.327669	0.0588455	-2.47723
-	scaffold_4:11523555-11524317	15.0503	2.70361	-2.47683
Ciclev10019368m.g	scaffold_3:13977376-13980070	175.911	31.6085	-2.47647
Ciclev10025168m.g	scaffold_7:18763895-18800038	140.955	25.3304	-2.47629
Ciclev10021413m.g	scaffold_3:24617311-24734809	1.00027	0.180022	-2.47415
Ciclev10000835m.g	scaffold_5:36196012-36199839	183.036	32.9452	-2.47399
Ciclev10023662m.g	scaffold_3:27453995-27456865	0.370338	0.0666694	-2.47375
Ciclev10013012m.g	scaffold_6:22520369-22521865	82.076	14.7835	-2.47298
Ciclev10004519m.g	scaffold_9:1144640-1147524	32.913	5.93902	-2.47036
Ciclev10015578m.g	scaffold_2:13525591-13527457	3.53379	0.637918	-2.46977
Ciclev10003563m.g	scaffold_5:30180306-30184569	0.290316	0.0524092	-2.46973
Ciclev10010003m.g	scaffold_1:26490582-26491432	2.07856	0.375236	-2.46971
Ciclev10020145m.g	scaffold_3:41869947-41871954	6.18053	1.11685	-2.4683
Ciclev10027052m.g	scaffold_7:17225399-17226338	1.20219	0.217527	-2.4664
Ciclev10023999m.g	scaffold_3:45367643-45367952	13.4351	2.43144	-2.46613
Ciclev10001979m.g	scaffold_5:39839983-39841860	105.394	19.0755	-2.466
Ciclev10010774m.g	scaffold_1:2728028-2730088	0.494761	0.0897152	-2.46331
Ciclev10031030m.g	scaffold_4:349655-353012	0.39171	0.0710596	-2.46269
Ciclev10016995m.g	scaffold_2:33900204-33901184	130.267	23.6739	-2.4601
Ciclev10029065m.g	scaffold_8:2145881-2147154	488.308	88.7699	-2.45965
Ciclev10016841m.g	scaffold_2:15774221-15776937	14.5143	2.6388	-2.45952
Ciclev10015739m.g	scaffold_2:2801928-2804205	47.4659	8.64219	-2.45742
Ciclev10021070m.g	scaffold_3:19501097-19504893	0.679487	0.123836	-2.45601
Ciclev10001291m.g	scaffold_5:41275200-41278829	0.564194	0.10318	-2.45103
Ciclev10019712m.g	scaffold_3:6163496-6165504	0.915669	0.167474	-2.45089
Ciclev10025982m.g	scaffold_7:20568866-20570682	1.4778	0.270405	-2.45026
Ciclev10013266m.g	scaffold_6:3804845-3806162	4.28957	0.785	-2.45007
Ciclev10011268m.g	scaffold_6:15070589-15076199	1.50168	0.274847	-2.44987
Ciclev10014223m.g	scaffold_2:6851264-6854086	0.308189	0.0564385	-2.44906
Ciclev10011707m.g	scaffold_6:22197698-22200838	1.98141	0.362892	-2.44892
Ciclev10030914m.g	scaffold_4:20106584-20113259	2.70831	0.496811	-2.44662

-	scaffold_9:1843567-1844163	17.7157	3.25082	-2.44615
Ciclev10017541m.g	scaffold_2:13863970-13866058	0.446618	0.082006	-2.44524
Ciclev10013453m.g	scaffold_6:16421923-16423174	1.327	0.243804	-2.44438
Ciclev10004051m.g	scaffold_5:29545386-29545962	2.00195	0.368138	-2.44309
Ciclev10032642m.g	scaffold_4:2229954-2231280	140.63	25.8622	-2.44298
Ciclev10001467m.g	scaffold_5:29056983-29060046	0.574831	0.10573	-2.44275
-	scaffold_5:28289811-28801386	53.908	9.92091	-2.44196
Ciclev10013727m.g	scaffold_6:1134443-1137227	0.284005	0.052303	-2.44095
Ciclev10010503m.g	scaffold_1:15714853-15716692	1.01895	0.187808	-2.43975
Ciclev10018129m.g	scaffold_2:17956818-17957625	1.49413	0.275621	-2.43855
Ciclev10021409m.g	scaffold_3:49354444-49357035	6.97035	1.28681	-2.43743
Ciclev10027045m.g	scaffold_7:17073722-17074316	2.47396	0.457787	-2.43408
Ciclev10027025m.g	scaffold_7:16191640-16194242	2.30263	0.426691	-2.43202
Ciclev10009761m.g	scaffold_1:25604508-25605857	32.0621	5.95163	-2.42951
Ciclev10026105m.g	scaffold_7:14560603-14562711	135.478	25.1666	-2.42848
Ciclev10011277m.g	scaffold_6:19937889-19942725	0.426856	0.0793133	-2.42812
Ciclev10028033m.g	scaffold_8:2161904-2167291	2.65294	0.493014	-2.42789
Ciclev10010231m.g	scaffold_1:9292607-9294211	0.611135	0.113689	-2.4264
Ciclev10030665m.g	scaffold_4:7493700-7706546	17.1628	3.19405	-2.42583
Ciclev10032412m.g	scaffold_4:23333288-23337136	3.53515	0.658236	-2.4251
Ciclev10019211m.g	scaffold_3:1943641-1947676	7.22019	1.34444	-2.42503
Ciclev10003034m.g	scaffold_5:36769277-36769885	6.17279	1.14975	-2.42461
Ciclev10011555m.g,Ciclev10011889m.g	scaffold_6:5754834-5780076	84.0227	15.6505	-2.42457
Ciclev10032084m.g	scaffold_4:12308934-12311438	169.465	31.5987	-2.42304
Ciclev10033846m.g	scaffold_4:23651506-23652719	1.16197	0.216771	-2.42232
Ciclev10012256m.g	scaffold_6:20456125-20457507	243.258	45.3813	-2.42232
Ciclev10011672m.g	scaffold_6:10520679-10522783	45.7735	8.54419	-2.4215
Ciclev10018941m.g	scaffold_3:3423785-3427132	1.608	0.300374	-2.42043
Ciclev10000764m.g	scaffold_5:42516005-42519605	131.852	24.63	-2.42043
Ciclev10022679m.g	scaffold_3:46168530-46169395	9.74887	1.82656	-2.4161
Ciclev10009878m.g	scaffold_1:27764735-27766035	69.8107	13.0812	-2.41596
Ciclev10023959m.g	scaffold_3:42678067-42680841	12.7955	2.3994	-2.4149
Ciclev10003491m.g	scaffold_5:35256589-35260160	0.811549	0.152329	-2.41349
Ciclev10016265m.g	scaffold_2:27247683-27249818	4.96846	0.933051	-2.41277
Ciclev10005737m.g	scaffold_9:1667150-1670619	64.626	12.142	-2.41211
Ciclev10017210m.g	scaffold_2:5808061-5809175	1.10705	0.20805	-2.41172
Ciclev10026716m.g	scaffold_7:1529253-1529846	81.7248	15.3737	-2.41031
Ciclev10008171m.g	scaffold_1:3746177-3749903	59.0101	11.1153	-2.40841
Ciclev10018878m.g	scaffold_3:1801449-1805257	5.21626	0.982806	-2.40804
Ciclev10004520m.g	scaffold_9:4058003-4062738	5.8999	1.11163	-2.40802
Ciclev10029927m.g	scaffold_8:3750665-3751478	1.34803	0.254377	-2.40581
Ciclev10022241m.g	scaffold_3:48908163-48909219	1.24848	0.235597	-2.40578
Ciclev10008218m.g	scaffold_1:23456082-23459416	30.5155	5.76789	-2.40343
Ciclev10028641m.g	scaffold_8:2388372-2391976	0.713554	0.134975	-2.40233
-	scaffold_7:2154405-2154669	36.7442	6.95171	-2.40208
Ciclev10001753m.g	scaffold_5:38149173-38151063	92.2505	17.4898	-2.39904
Ciclev10003612m.g	scaffold_5:27192349-27195683	1.87156	0.354944	-2.39858
Ciclev10029524m.g	scaffold_8:16214251-16215800	1.35849	0.257765	-2.39788
Ciclev10033331m.g	scaffold_4:2408399-2410046	0.528014	0.100218	-2.39743
Ciclev10026052m.g	scaffold_7:17497719-17499325	1.31576	0.249968	-2.39608
Ciclev10001187m.g	scaffold_5:42265152-42269693	115.975	22.0378	-2.39576
Ciclev10028578m.g	scaffold_8:2929767-2932689	0.744552	0.141606	-2.39449
Ciclev10018237m.g	scaffold_2:7223552-7226407	2.42435	0.461666	-2.39268
Ciclev10012938m.g	scaffold_6:22083544-22084946	1.01754	0.194195	-2.3895
Ciclev10028548m.g	scaffold_8:24610683-24612555	4.8806	0.932847	-2.38735

Ciclev10010068m.g	scaffold_1:18886440-18886981	2.23922	0.428035	-2.3872
Ciclev10033003m.g	scaffold_4:7368910-7369710	22.1929	4.24241	-2.38714
Ciclev10028756m.g	scaffold_8:210020-211568	12.6276	2.41866	-2.3843
Ciclev10007939m.g	scaffold_1:6388569-6391123	3.36856	0.645245	-2.38422
Ciclev10033500m.g	scaffold_4:17131152-17132002	1.99886	0.383113	-2.38333
Ciclev10029037m.g	scaffold_8:20620078-20723794	52.2505	10.0185	-2.38278
Ciclev10013191m.g	scaffold_6:22970177-22976234	3891.5	746.888	-2.38136
Ciclev10013473m.g	scaffold_6:16907253-16907900	1.61601	0.310698	-2.37885
Ciclev10012837m.g	scaffold_6:22365432-22367412	403.01	77.5107	-2.37835
-	scaffold_7:13676420-13677826	23.3918	4.50078	-2.37776
Ciclev10017183m.g	scaffold_2:21493977-21494506	7.77243	1.49593	-2.37732
Ciclev10005082m.g	scaffold_9:26566368-26570006	47.506	9.15804	-2.375
Ciclev10012055m.g	scaffold_6:17675266-17676898	463.056	89.3947	-2.37293
Ciclev10007975m.g	scaffold_1:20553874-20558851	16.9801	3.28386	-2.37039
Ciclev10033700m.g	scaffold_4:2688164-2688824	1.773	0.342926	-2.37022
Ciclev10033634m.g	scaffold_4:24036995-24037820	2.3175	0.449015	-2.36773
Ciclev10027617m.g	scaffold_7:1319136-1320544	33.8334	6.561	-2.36646
Ciclev10028242m.g	scaffold_8:2747670-2749378	34.1375	6.64349	-2.36135
Ciclev10011691m.g	scaffold_6:16503593-16506386	503.768	98.1468	-2.35975
Ciclev10015972m.g	scaffold_2:32376116-32377371	12.0896	2.35597	-2.35937
Ciclev10033991m.g	scaffold_4:19413411-19416331	5.85388	1.14122	-2.35882
Ciclev10020389m.g	scaffold_3:936910-938678	1.45358	0.283488	-2.35825
Ciclev10016328m.g	scaffold_2:8900284-8901430	8.65875	1.68996	-2.35717
Ciclev10030014m.g	scaffold_8:7380432-7386783	36.6941	7.1783	-2.35383
Ciclev10017805m.g	scaffold_2:6554459-6563743	17.1635	3.36108	-2.35235
Ciclev10015097m.g	scaffold_2:4367067-4371625	16.0874	3.15071	-2.35218
Ciclev10014458m.g	scaffold_2:6586730-6589503	1.36544	0.26779	-2.3502
Ciclev10002294m.g	scaffold_5:37219889-37221066	2.98972	0.587959	-2.34623
-	scaffold_5:2761698-2762321	21.0911	4.15031	-2.34534
Ciclev10026925m.g	scaffold_7:4819411-4820934	0.417992	0.0824114	-2.34256
Ciclev10015458m.g	scaffold_2:23717880-23719637	4.16633	0.822163	-2.34128
Ciclev10031682m.g	scaffold_4:583973-586423	80.0201	15.7932	-2.34106
Ciclev10033602m.g	scaffold_4:25017111-25019556	1.77572	0.350981	-2.33894
Ciclev10000538m.g	scaffold_5:34128462-34131986	1.47345	0.291897	-2.33567
Ciclev10019596m.g	scaffold_3:47666935-47669808	2.65597	0.526261	-2.33539
Ciclev10022362m.g	scaffold_3:49740453-49745002	39.8013	7.89666	-2.3335
Ciclev10028359m.g	scaffold_8:20352231-20354410	34.302	6.80583	-2.33345
Ciclev10025741m.g	scaffold_7:149298-153895	99.7251	19.8205	-2.33097
Ciclev10012130m.g	scaffold_6:20731407-20732679	352.623	70.1195	-2.33024
Ciclev10020068m.g	scaffold_3:42460939-42467955	111.242	22.1721	-2.32689
Ciclev10022453m.g	scaffold_3:39092284-39094629	288.143	57.4595	-2.32617
Ciclev10019706m.g,Ciclev10019727m.g	scaffold_3:42061468-42069114	231.595	46.2305	-2.32469
Ciclev10000891m.g	scaffold_5:12168883-12172755	21.7141	4.34037	-2.32274
Ciclev10033175m.g	scaffold_4:23283690-23285802	12.427	2.48694	-2.32104
Ciclev10024731m.g	scaffold_7:2472536-2477250	1.30822	0.261914	-2.32044
Ciclev10017452m.g	scaffold_2:11061826-11062240	5.41011	1.0843	-2.3189
Ciclev10011214m.g	scaffold_6:3129654-3132355	346.849	69.5788	-2.31759
Ciclev10020606m.g	scaffold_3:11687448-12154789	2.43673	0.489608	-2.31524
Ciclev10033648m.g	scaffold_4:11711487-11728366	15.597	3.13396	-2.31521
Ciclev10012617m.g	scaffold_6:18190872-18193790	0.9108	0.183043	-2.31496
Ciclev10016312m.g	scaffold_2:23679907-23681698	5.91639	1.19262	-2.31058
Ciclev10027084m.g	scaffold_7:611716-612950	4.73831	0.955874	-2.30948
Ciclev10004736m.g	scaffold_9:4429888-4432137	12.2768	2.48191	-2.30641
Ciclev10006713m.g	scaffold_9:1198744-1199675	442.199	89.4386	-2.30573
Ciclev10031239m.g	scaffold_4:14996039-15002581	141.518	28.654	-2.30418

Ciclev10031007m.g	scaffold_4:18202742-18205466	0.707118	0.143187	-2.30405
Ciclev10028623m.g	scaffold_8:24554321-24561746	7.0484	1.42765	-2.30365
Ciclev10010666m.g	scaffold_1:22175575-22263277	1.00486	0.20384	-2.30149
Ciclev10003452m.g	scaffold_5:16198486-16201340	0.638259	0.129598	-2.3001
Ciclev10010970m.g	scaffold_6:21429204-21435111	0.402041	0.0816551	-2.29973
-	scaffold_1:20148919-20152462	33.5851	6.8331	-2.29721
Ciclev10000125m.g	scaffold_5:39941852-39952725	4.62241	0.941967	-2.2949
Ciclev10004881m.g	scaffold_9:17070430-17072209	88.8087	18.1178	-2.29329
Ciclev10011622m.g	scaffold_6:25349416-25351949	100.898	20.6244	-2.29047
Ciclev10020109m.g	scaffold_3:3358969-3361865	8.75944	1.79499	-2.28686
Ciclev10027138m.g	scaffold_7:8550403-8551979	0.85282	0.174824	-2.28634
Ciclev10000874m.g	scaffold_5:31403726-31408001	7.00039	1.43609	-2.28529
Ciclev10021422m.g	scaffold_3:48433759-48435013	2.67351	0.549031	-2.28378
Ciclev10014642m.g	scaffold_2:35513795-35516719	286.184	58.7723	-2.28373
Ciclev10004732m.g	scaffold_9:28990093-28993179	64.1151	13.1672	-2.28372
Ciclev10001510m.g	scaffold_5:33100312-33101760	3.30631	0.679017	-2.2837
Ciclev10030884m.g	scaffold_4:23234711-23239227	4.83731	0.993711	-2.28331
Ciclev10008142m.g	scaffold_1:6402442-6406121	27.9734	5.75132	-2.28209
Ciclev10009178m.g	scaffold_1:4036230-4038959	9.14217	1.88078	-2.28121
Ciclev10012846m.g	scaffold_6:18783833-18784757	3.58604	0.739196	-2.27836
Ciclev10011580m.g	scaffold_6:21983043-21991500	7.69493	1.58719	-2.27743
Ciclev10005384m.g	scaffold_9:28704398-28707148	37.8005	7.79918	-2.27701
Ciclev10013499m.g	scaffold_6:17507773-17510719	1.85577	0.382954	-2.27677
Ciclev10030623m.g	scaffold_4:20142444-20145612	0.631067	0.130227	-2.27676
Ciclev10019922m.g	scaffold_3:41270984-41274884	18.5441	3.82851	-2.27611
Ciclev10005097m.g	scaffold_9:25896765-25897992	2.05115	0.424481	-2.27266
Ciclev10031104m.g	scaffold_4:15505583-15508159	4.7271	0.978607	-2.27215
Ciclev10019265m.g	scaffold_3:50861812-50866146	49.912	10.3353	-2.2718
Ciclev10025988m.g	scaffold_7:8712252-8715371	104.151	21.5743	-2.27129
Ciclev10032525m.g	scaffold_4:17822401-17824104	71.3255	14.7895	-2.26985
Ciclev10007206m.g	scaffold_9:1436101-1436628	20.085	4.16584	-2.26944
Ciclev10018942m.g	scaffold_3:229426-233034	17.4204	3.61388	-2.26916
Ciclev10028375m.g	scaffold_8:24832652-24835113	3.66183	0.760203	-2.26811
Ciclev10005281m.g	scaffold_9:6852155-6854052	7.55378	1.56899	-2.26737
Ciclev10019217m.g	scaffold_3:27827994-27830405	11.6103	2.41324	-2.26636
Ciclev10000129m.g	scaffold_5:25999616-26032106	0.865713	0.180005	-2.26585
Ciclev10005894m.g	scaffold_9:2452852-2455181	177.733	36.9608	-2.26564
Ciclev10019887m.g	scaffold_3:27378258-27383787	34.3095	7.13545	-2.26553
Ciclev10010470m.g	scaffold_1:27224300-27225158	2.19579	0.458016	-2.26127
-	scaffold_7:18191269-18191793	6.51559	1.35963	-2.26068
Ciclev10001478m.g	scaffold_5:32542379-32543973	107.614	22.49	-2.25851
-	scaffold_3:17890506-17890836	16.5295	3.45463	-2.25844
Ciclev10028790m.g	scaffold_8:21605600-21610218	1.5861	0.33168	-2.25762
Ciclev10000450m.g	scaffold_5:42121458-42125439	14.1911	2.97159	-2.25568
Ciclev10010820m.g	scaffold_1:24498030-24498483	60.4245	12.6652	-2.25426
Ciclev10030031m.g	scaffold_8:16550333-16550840	18.4389	3.86506	-2.25419
Ciclev10021187m.g	scaffold_3:39657612-39660222	175.202	36.7339	-2.25383
Ciclev10028241m.g	scaffold_8:23456629-23459073	23.7155	4.97296	-2.25365
Ciclev10019339m.g	scaffold_3:48063482-48066433	196.72	41.2701	-2.25298
Ciclev10019657m.g	scaffold_3:462332-464454	10.9225	2.29456	-2.25101
Ciclev10003203m.g	scaffold_5:20777927-20778458	2.13609	0.448952	-2.25034
Ciclev10025050m.g	scaffold_7:12956764-12962208	1.34918	0.284313	-2.24652
Ciclev10019374m.g	scaffold_3:2648279-2652347	14.0526	2.96146	-2.24645
Ciclev10027010m.g	scaffold_7:17948447-18051030	54.2382	11.4431	-2.24484
Ciclev10016038m.g	scaffold_2:32934831-32936263	22.1816	4.6814	-2.24435
Ciclev10010665m.g	scaffold_1:24964825-24981789	13.9388	2.94196	-2.24426

Ciclev10023669m.g	scaffold_3:27210507-27212925	7.93099	1.67483	-2.24349
Ciclev10020482m.g	scaffold_3:50878856-50881433	0.886189	0.187178	-2.2432
Ciclev10022257m.g	scaffold_3:8825969-8828784	1.58344	0.334489	-2.24303
Ciclev10014602m.g	scaffold_2:28282823-28590596	6.34038	1.34075	-2.24152
Ciclev10025703m.g	scaffold_7:662559-667430	15.0808	3.18984	-2.24116
Ciclev10007796m.g	scaffold_1:3082252-3085455	25.0244	5.30972	-2.23663
Ciclev10029563m.g	scaffold_8:2264875-2265853	5.7224	1.21449	-2.23627
Ciclev10013655m.g,Ciclev10013676m.g	scaffold_6:6314327-6318607	2167.15	460.052	-2.23593
Ciclev10014144m.g,Ciclev10014153m.g	scaffold_2:30523899-30548559	9.694	2.05845	-2.23554
Ciclev10028780m.g	scaffold_8:5582845-5585920	2.51361	0.533855	-2.23524
Ciclev10032081m.g	scaffold_4:19102843-19104818	1.51834	0.323024	-2.23278
Ciclev10027855m.g	scaffold_8:24935282-24939307	22.0088	4.68598	-2.23166
-	scaffold_5:2787300-2787760	95.6312	20.4094	-2.22824
-	scaffold_3:21296577-21473212	7.73875	1.65218	-2.22773
Ciclev10032192m.g	scaffold_4:2333546-2335111	330.669	70.6699	-2.22622
Ciclev10031553m.g	scaffold_4:19133591-19137895	32.4197	6.93439	-2.22503
Ciclev10020715m.g	scaffold_3:46149264-46153166	17.0194	3.64252	-2.22417
Ciclev10004518m.g	scaffold_9:4663884-4697961	1.54566	0.330936	-2.2236
Ciclev10002405m.g	scaffold_5:42530863-42532444	23.1379	4.95484	-2.22335
Ciclev10019790m.g	scaffold_3:8640419-8644420	25.0221	5.36211	-2.22233
Ciclev10014333m.g	scaffold_2:9592457-9595926	20.2954	4.35658	-2.21989
Ciclev10027716m.g	scaffold_8:6255915-6268429	7.69134	1.6532	-2.21797
-	scaffold_9:17385183-17385418	5808.17	1248.51	-2.21788
Ciclev10017830m.g	scaffold_2:28282823-28590596	12.9955	2.80024	-2.21438
Ciclev10033106m.g	scaffold_4:3762484-3766722	5.94128	1.28142	-2.21303
Ciclev10020415m.g	scaffold_3:11687448-12154789	54.3521	11.7284	-2.21233
Ciclev10006144m.g	scaffold_9:5757685-5759195	26.0689	5.63047	-2.211
Ciclev10000733m.g	scaffold_5:33879329-33882234	5.54374	1.19789	-2.21036
Ciclev10008711m.g	scaffold_1:2173895-2176927	10.0176	2.16474	-2.21027
Ciclev10031813m.g	scaffold_4:20702894-20707125	6.17561	1.33871	-2.20573
Ciclev10002961m.g	scaffold_5:35354185-35354887	18.7513	4.06922	-2.20417
Ciclev10022688m.g	scaffold_3:35784883-35785592	194.279	42.2441	-2.20131
Ciclev10001255m.g	scaffold_5:41881296-41883386	1.53304	0.333396	-2.20109
Ciclev10004951m.g	scaffold_9:4460615-4462312	216.319	47.098	-2.19942
-	scaffold_8:7161096-7161396	27.5987	6.00975	-2.19922
Ciclev10022509m.g	scaffold_3:44816497-44817321	3.79405	0.827188	-2.19745
Ciclev10025820m.g	scaffold_7:2450661-2454470	3.81014	0.830822	-2.19723
Ciclev10003010m.g	scaffold_5:226515-230191	1.23276	0.270085	-2.1904
Ciclev10015634m.g	scaffold_2:10931514-10935724	48.3777	10.6091	-2.18903
Ciclev10010561m.g	scaffold_1:15231882-15232893	1.55451	0.342006	-2.18437
Ciclev10009238m.g	scaffold_1:3397422-3400719	2.70096	0.594601	-2.18348
Ciclev10025354m.g	scaffold_7:1739442-1743149	3.7992	0.836803	-2.18274
Ciclev10010778m.g	scaffold_1:17837961-17838312	3.55489	0.783464	-2.18186
Ciclev10021081m.g	scaffold_3:22851985-22853202	30.1166	6.63916	-2.18148
Ciclev10004944m.g	scaffold_9:8338761-8342569	1.65421	0.364684	-2.18143
Ciclev10027800m.g	scaffold_8:7966629-7969209	4.28366	0.945487	-2.17971
Ciclev10005395m.g	scaffold_9:6864745-6869616	2.99218	0.661093	-2.17827
Ciclev10000103m.g	scaffold_5:38805240-38812427	41.6232	9.20178	-2.1774
Ciclev10008862m.g	scaffold_1:23759729-23762184	18.6593	4.12863	-2.17616
Ciclev10025915m.g	scaffold_7:2416583-2419376	235.67	52.2273	-2.17389
Ciclev10001460m.g	scaffold_5:37254881-37257398	23.9634	5.31406	-2.17294
Ciclev10002005m.g	scaffold_5:43068092-43070554	94.3781	20.9363	-2.17245
Ciclev10014091m.g	scaffold_2:23893669-23897580	19.1451	4.24835	-2.172
Ciclev10018577m.g	scaffold_3:1906737-1912326	3.29984	0.732438	-2.17162

Ciclev10021551m.g	scaffold_3:47200054-47201893	180.273	40.0908	-2.16885
Ciclev10021733m.g	scaffold_3:50175402-50177122	1.04461	0.232409	-2.16823
Ciclev10015968m.g	scaffold_2:32670180-32672307	5.56691	1.23963	-2.16697
Ciclev10030977m.g	scaffold_4:16129998-16136902	54.335	12.1038	-2.16642
Ciclev10021039m.g	scaffold_3:18826019-18831201	167.927	37.4204	-2.16594
Ciclev10009510m.g	scaffold_1:20930297-20931158	56.5962	12.6191	-2.16509
-	scaffold_3:19834362-19911599	9.44351	2.10691	-2.16419
Ciclev10029391m.g	scaffold_8:19146289-19210612	188.94	42.1662	-2.16377
Ciclev10011035m.g	scaffold_6:19440365-19445030	3.24489	0.725991	-2.16015
Ciclev10003935m.g	scaffold_5:42084911-42087039	1.40835	0.315166	-2.15982
Ciclev10009529m.g	scaffold_1:22414121-22415456	4.68405	1.04932	-2.15831
Ciclev10025729m.g	scaffold_7:7485765-7641496	8.49689	1.9036	-2.1582
Ciclev10015924m.g	scaffold_2:21717831-21719535	5.38584	1.20821	-2.15629
Ciclev10013077m.g	scaffold_6:23639246-23639974	36.0863	8.09798	-2.15582
Ciclev10005738m.g	scaffold_9:2854991-2857646	9.44075	2.11967	-2.15506
Ciclev10014884m.g	scaffold_2:22867079-22870230	1.84984	0.415461	-2.15462
-	scaffold_3:40504072-40684964	49.072	11.0287	-2.15364
Ciclev10000326m.g	scaffold_5:1084199-1143725	1.83527	0.412494	-2.15355
Ciclev10020881m.g	scaffold_3:855006-857471	8.45343	1.90067	-2.15303
Ciclev10016386m.g	scaffold_2:11388043-11389521	2.88307	0.648535	-2.15235
Ciclev10028315m.g	scaffold_8:12953963-12968792	230.351	51.8191	-2.15228
Ciclev10024475m.g	scaffold_3:41318013-41318352	267.069	60.1275	-2.15112
Ciclev10031681m.g	scaffold_4:14681256-14684932	29.912	6.74069	-2.14976
Ciclev10003821m.g	scaffold_5:38563225-38565511	2.01679	0.455169	-2.14759
Ciclev10004709m.g	scaffold_9:2994223-2997285	1.44426	0.326762	-2.14402
Ciclev10011613m.g	scaffold_6:20005404-20007246	39.205	8.87535	-2.14316
Ciclev10014341m.g	scaffold_2:13677327-13685473	67.9779	15.3902	-2.14306
Ciclev10027275m.g	scaffold_7:2895802-2896693	1.86392	0.422209	-2.14231
Ciclev10004982m.g	scaffold_9:9827281-9829466	3.55353	0.806581	-2.13936
Ciclev10023053m.g	scaffold_3:33746265-33746994	31.0265	7.04468	-2.13889
Ciclev10023860m.g	scaffold_3:20604772-20606940	0.463346	0.105505	-2.13478
Ciclev10025304m.g	scaffold_7:9157872-9161992	4.10782	0.936236	-2.13343
Ciclev10026215m.g	scaffold_7:9040593-9041542	9.385	2.13973	-2.13293
Ciclev10022153m.g	scaffold_3:5280718-5282122	6.53917	1.49125	-2.13259
Ciclev10029394m.g	scaffold_8:3007025-3009612	69.0435	15.7512	-2.13204
Ciclev10014498m.g	scaffold_2:33680381-33684343	67.9555	15.51	-2.13139
Ciclev10014811m.g	scaffold_2:35970576-35973973	156.057	35.6642	-2.12952
Ciclev10022211m.g,Ciclev10022911m.g,Ciclev10023557m.g	scaffold_3:31770209-32016675	1956.21	447.075	-2.12947
Ciclev10016303m.g	scaffold_2:20258323-20260070	4.09133	0.936641	-2.127
Ciclev10008496m.g	scaffold_1:25940744-25942304	77.9457	17.8684	-2.12506
Ciclev10011957m.g	scaffold_6:20691107-20706907	31.0412	7.12847	-2.12252
Ciclev10014113m.g	scaffold_2:7292922-7336431	4.77781	1.09727	-2.12243
Ciclev10010134m.g	scaffold_1:738190-743742	1.7581	0.404221	-2.12081
Ciclev10015392m.g	scaffold_2:6472570-6474273	7.58004	1.74364	-2.12011
Ciclev10011169m.g	scaffold_6:21905556-21908832	123.108	28.335	-2.11927
Ciclev10011796m.g	scaffold_6:22186299-22187922	8.62463	1.98596	-2.11863
Ciclev10006489m.g,Ciclev10006630m.g	scaffold_9:29290439-29356041	11.2752	2.60733	-2.1125
Ciclev10033100m.g	scaffold_4:2038851-2039526	7.71949	1.78534	-2.11231
Ciclev10028716m.g	scaffold_8:1156026-1158611	0.897675	0.207623	-2.11223
Ciclev10007028m.g	scaffold_9:3500323-3515616	18.9645	4.39038	-2.11089
Ciclev10026522m.g	scaffold_7:9996369-9997710	43.3589	10.0429	-2.11015
Ciclev10006337m.g	scaffold_9:9986212-9986984	5.00396	1.16016	-2.10875
Ciclev10019533m.g	scaffold_3:47390659-47410320	42.8149	9.93341	-2.10775

Ciclev10010050m.g	scaffold_1:17879965-17882892	12.7952	2.9706	-2.10678
Ciclev10014787m.g	scaffold_2:32218212-32220325	16.7311	3.89276	-2.10367
Ciclev10018778m.g	scaffold_3:41991277-41994183	5.33116	1.24121	-2.10271
Ciclev10025910m.g	scaffold_7:3282640-3284143	210.049	48.9889	-2.1002
Ciclev10026873m.g	scaffold_7:16675946-16676725	1.94334	0.454288	-2.09686
Ciclev10001341m.g	scaffold_5:35989375-35991063	47.1702	11.0328	-2.09608
Ciclev10006930m.g	scaffold_9:24356650-24541643	0.289563	0.0677696	-2.09517
Ciclev10001800m.g	scaffold_5:17564728-17565994	76.2039	17.888	-2.09087
Ciclev10026916m.g	scaffold_7:4305755-4307778	1.86522	0.437922	-2.0906
Ciclev10008140m.g	scaffold_1:17308565-17311235	4.42346	1.04124	-2.08688
Ciclev10029615m.g	scaffold_8:2073735-2074304	81.8474	19.3062	-2.08387
Ciclev10028372m.g	scaffold_8:4903915-4909930	76.9431	18.1562	-2.08333
Ciclev10018328m.g	scaffold_2:7292922-7336431	1.10401	0.260613	-2.08277
Ciclev10027853m.g	scaffold_8:2634214-2638475	1.66782	0.393924	-2.08198
Ciclev10029205m.g	scaffold_8:19276634-19278102	28.8203	6.81837	-2.07959
Ciclev10019494m.g	scaffold_3:2210484-2215202	10.1164	2.39372	-2.07938
Ciclev10005594m.g	scaffold_9:25161131-25163412	296.619	70.1856	-2.07936
Ciclev10025746m.g	scaffold_7:2137425-2140497	6.92367	1.63831	-2.07933
Ciclev10008603m.g	scaffold_1:28505845-28510309	13.9099	3.3006	-2.07531
Ciclev10020557m.g	scaffold_3:40883953-40888631	0.480948	0.114166	-2.07474
Ciclev10020687m.g	scaffold_3:42933883-42935909	510.945	121.586	-2.07119
Ciclev10032164m.g	scaffold_4:22801202-22802540	0.702573	0.167347	-2.0698
Ciclev10008731m.g	scaffold_1:4289573-4291259	39.3405	9.38107	-2.06819
Ciclev10024550m.g	scaffold_3:48607677-48609120	1.03158	0.246367	-2.06597
Ciclev10015295m.g	scaffold_2:6579636-6581607	0.527005	0.12593	-2.06519
-	scaffold_5:655534-811195	7.86165	1.87864	-2.06514
Ciclev10018046m.g	scaffold_2:34897744-34899006	1.30129	0.311589	-2.06222
Ciclev10027040m.g	scaffold_7:16843104-16845450	1.94195	0.465826	-2.05964
Ciclev10024529m.g	scaffold_3:40848610-40883215	0.447804	0.107441	-2.05932
Ciclev10014459m.g	scaffold_2:11493088-11497181	1.87073	0.449221	-2.05811
Ciclev10022661m.g	scaffold_3:42229223-42233850	17.5023	4.20477	-2.05745
Ciclev10021119m.g	scaffold_3:7004357-7007866	58.5782	14.0818	-2.05653
Ciclev10033480m.g	scaffold_4:7493700-7706546	61.5632	14.803	-2.05618
Ciclev10004322m.g	scaffold_9:18502292-18505531	1.18517	0.284981	-2.05616
Ciclev10008858m.g	scaffold_1:2571060-2573956	2.27042	0.546176	-2.05552
Ciclev10030945m.g	scaffold_4:16324347-16328585	2.9788	0.716775	-2.05514
Ciclev10024850m.g	scaffold_7:13348931-13360472	0.199453	0.0480101	-2.05464
Ciclev10002349m.g	scaffold_5:42489683-42491501	151.752	36.5742	-2.05281
Ciclev10027010m.g	scaffold_7:17948447-18051030	0.550943	0.132814	-2.0525
Ciclev10006527m.g	scaffold_9:8376736-8414192	0.382049	0.0921279	-2.05205
Ciclev10004069m.g	scaffold_5:28289811-28801386	16.023	3.86718	-2.05079
Ciclev10008045m.g	scaffold_1:2211677-2213570	1.29109	0.311615	-2.05076
Ciclev10009050m.g	scaffold_1:18912039-18913817	1.54594	0.373997	-2.04739
Ciclev10005102m.g	scaffold_9:30778802-30783116	0.42057	0.101773	-2.047
Ciclev10020705m.g	scaffold_3:48188237-48190851	4.97245	1.20334	-2.04692
Ciclev10017802m.g	scaffold_2:233379-237733	0.312759	0.0757048	-2.0466
Ciclev10000297m.g	scaffold_5:32546088-32865063	0.302787	0.0733155	-2.04611
Ciclev10005040m.g	scaffold_9:31159646-31162362	0.378654	0.0917739	-2.04472
Ciclev10025613m.g	scaffold_7:3920917-3924142	4.4617	1.08162	-2.0444
Ciclev10017001m.g,Ciclev10017259m.g	scaffold_2:24737092-24926440	20.2969	4.92422	-2.04329
Ciclev10021728m.g	scaffold_3:26847876-26850055	0.67961	0.164884	-2.04326
Ciclev10019893m.g	scaffold_3:39133969-39135992	32.5747	7.90979	-2.04204
Ciclev10000358m.g	scaffold_5:37849212-37853413	39.5667	9.61076	-2.04157
Ciclev10001976m.g	scaffold_5:37586833-37589255	586.67	142.664	-2.03993
Ciclev10027341m.g	scaffold_7:8900338-8902756	3.49473	0.849902	-2.03981

Ciclev10002979m.g	scaffold_5:34404146-34405161	1.03421	0.251558	-2.03956
Ciclev10030887m.g	scaffold_4:16193833-16197222	0.262768	0.0639256	-2.03933
Ciclev10030285m.g	scaffold_8:17700391-17703618	0.659294	0.160413	-2.03913
Ciclev10021437m.g	scaffold_3:42958144-42960351	0.804585	0.195939	-2.03784
Ciclev10005968m.g	scaffold_9:27640073-27641178	75.5543	18.4004	-2.03777
Ciclev10000479m.g	scaffold_5:2479777-2485129	0.591778	0.144143	-2.03755
Ciclev10001712m.g	scaffold_5:37381117-37384357	38.7154	9.44134	-2.03585
Ciclev10015249m.g	scaffold_2:7725023-7755971	30.8655	7.5273	-2.03579
Ciclev10006543m.g	scaffold_9:12493821-12494580	1.09369	0.26688	-2.03495
Ciclev10023307m.g	scaffold_3:18617854-18618580	1.04874	0.256911	-2.02932
Ciclev10028699m.g	scaffold_8:3860725-3891595	0.49627	0.121656	-2.02832
Ciclev10002430m.g	scaffold_5:43134799-43135657	62.2539	15.2634	-2.02809
Ciclev10009542m.g	scaffold_1:1421601-1422908	402.393	98.7318	-2.02702
Ciclev10006335m.g	scaffold_9:8088694-8091407	1.24093	0.30456	-2.02663
Ciclev10005557m.g	scaffold_9:28665022-28667783	19.1155	4.69546	-2.0254
Ciclev10011134m.g	scaffold_6:10000923-10003899	5.82971	1.43215	-2.02525
Ciclev10022623m.g	scaffold_3:50207879-50208921	75.637	18.6031	-2.02355
Ciclev10019207m.g	scaffold_3:44244253-44249010	3.39388	0.835227	-2.02269
Ciclev10024191m.g	scaffold_3:2594993-2596969	0.348535	0.0857907	-2.02241
Ciclev10016953m.g	scaffold_2:35861294-35862848	12.1313	2.98632	-2.02229
-	scaffold_5:2700618-2700870	22.6791	5.58379	-2.02205
Ciclev10012736m.g	scaffold_6:17949430-17950757	40.2178	9.90409	-2.02174
Ciclev10022593m.g	scaffold_3:13807410-13808572	2.54733	0.627583	-2.02111
-	scaffold_9:7493202-7493512	1416.62	349.381	-2.01958
Ciclev10021132m.g	scaffold_3:50463538-50464855	2.74467	0.677329	-2.0187
Ciclev10031102m.g	scaffold_4:21735793-21738770	3.06428	0.756508	-2.01812
Ciclev10018087m.g	scaffold_2:3555024-3559126	1.90404	0.470162	-2.01783
-	scaffold_2:28282823-28590596	8.72007	2.15443	-2.01703
Ciclev10012786m.g	scaffold_6:24264176-24265636	0.82609	0.204129	-2.01682
Ciclev10029840m.g	scaffold_8:24512739-24517606	0.617805	0.152889	-2.01467
Ciclev10031489m.g	scaffold_4:17753989-17757513	47.4842	11.7525	-2.01448
Ciclev10026336m.g	scaffold_7:6261296-6263128	21.0199	5.20518	-2.01373
Ciclev10028361m.g	scaffold_8:19568817-19573518	0.837884	0.207589	-2.01302
Ciclev10028426m.g	scaffold_8:1698324-1700656	79.8762	19.8039	-2.01198
Ciclev10031986m.g	scaffold_4:18332484-18333750	2.06761	0.512741	-2.01166
Ciclev10025688m.g	scaffold_7:1498560-1500104	4.39587	1.09188	-2.00933
Ciclev10016129m.g	scaffold_2:21057079-21059316	15.4551	3.84351	-2.00758
Ciclev10025302m.g	scaffold_7:15496820-15499377	1.21655	0.302552	-2.00754
Ciclev10018595m.g	scaffold_3:9970535-9975305	3.62789	0.903176	-2.00605
Ciclev10001638m.g	scaffold_5:24467954-24473569	1.45764	0.362955	-2.00577
Ciclev10010157m.g	scaffold_1:3704737-3706120	0.685038	0.170868	-2.0033
Ciclev10000262m.g	scaffold_5:27087957-27094914	10.0437	2.50556	-2.00309
Ciclev10017895m.g	scaffold_2:28282823-28590596	22.7544	5.67886	-2.00247
Ciclev10032958m.g	scaffold_4:22636710-22637581	4.67202	1.1661	-2.00235
Ciclev10005017m.g	scaffold_9:9983011-9984385	1.67248	0.417772	-2.00121
Ciclev10007289m.g	scaffold_1:23842972-23850135	2.51991	0.629467	-2.00117
Ciclev10000355m.g	scaffold_5:22304299-22309822	6.26611	1.56571	-2.00075
Ciclev10008000m.g	scaffold_1:4156422-4160412	73.3231	18.3274	-2.00027
Ciclev10012472m.g	scaffold_6:21446970-21449098	7.9377	1.98524	-1.9994
Ciclev10026997m.g	scaffold_7:399184-411768	1.00726	0.252419	-1.99654
Ciclev10002050m.g	scaffold_5:19780297-19783698	0.670149	0.167968	-1.99629
Ciclev10028602m.g	scaffold_8:23908852-23911480	8.06474	2.02251	-1.99548
-	scaffold_5:1503259-1988798	12.5992	3.16325	-1.99386
Ciclev10016466m.g	scaffold_2:16392850-16394263	212.864	53.4629	-1.99332
Ciclev10005598m.g	scaffold_9:29764963-29766145	324.602	81.5484	-1.99295
Ciclev10028778m.g	scaffold_8:2220868-2224576	29.3301	7.37606	-1.99146

Ciclev10018388m.g	scaffold_2:14217252-14224335	3.94401	0.992155	-1.99103
-	scaffold_3:40703351-40719738	19.7675	4.97317	-1.99089
Ciclev10032279m.g	scaffold_4:23695431-23697703	16.4917	4.14988	-1.9906
Ciclev10004715m.g	scaffold_9:30970842-30972432	0.855675	0.21532	-1.99058
Ciclev10025903m.g	scaffold_7:1295838-1297378	50.1609	12.6236	-1.99044
Ciclev10033047m.g	scaffold_4:24490926-24493186	247.77	62.3844	-1.98974
Ciclev10011449m.g	scaffold_6:24409993-24412120	6.43112	1.62226	-1.98707
Ciclev10019325m.g	scaffold_3:12698337-12703025	14.2349	3.59643	-1.98479
-	scaffold_9:22499151-22499367	71.3348	18.0281	-1.98436
Ciclev10010710m.g	scaffold_1:158901-161375	0.604404	0.152755	-1.98429
Ciclev10019749m.g	scaffold_3:47737983-47739878	71.6179	18.107	-1.98377
Ciclev10029826m.g	scaffold_8:4555221-4556238	0.768628	0.194375	-1.98344
Ciclev10006590m.g	scaffold_9:2078437-2078746	87.9499	22.2769	-1.98113
Ciclev10020735m.g	scaffold_3:5271978-5274369	107.922	27.3825	-1.97867
Ciclev10001500m.g	scaffold_5:38320640-38322884	0.436706	0.110884	-1.97762
Ciclev10023527m.g	scaffold_3:4954020-4959276	286.008	72.6849	-1.97633
Ciclev10010252m.g	scaffold_1:8696318-8697796	11.0845	2.81922	-1.97518
Ciclev10016846m.g	scaffold_2:33499538-33502421	55.1939	14.0395	-1.97502
Ciclev10024857m.g	scaffold_7:15435206-15437898	1.72913	0.44026	-1.97362
Ciclev10033435m.g	scaffold_4:1171785-1173887	1.70055	0.433305	-1.97255
-	scaffold_8:7159549-7160493	7.67437	1.95618	-1.97201
Ciclev10018037m.g	scaffold_2:5155107-5159194	5.40734	1.37944	-1.97083
-	scaffold_6:23249918-23250044	16332.4	4170.5	-1.96945
Ciclev10033543m.g	scaffold_4:6025714-6026668	184.536	47.144	-1.96876
Ciclev10017097m.g	scaffold_2:21326708-21327604	0.973791	0.249391	-1.9652
Ciclev10032927m.g	scaffold_4:12434969-12439835	3.34573	0.857	-1.96495
Ciclev10019810m.g	scaffold_3:23665051-23668159	19.334	4.95529	-1.9641
-	scaffold_3:18781221-18784599	9.94741	2.55194	-1.96273
-	scaffold_5:655534-811195	192.47	49.4025	-1.96198
-	scaffold_2:33520837-33522137	39.8382	10.2293	-1.96145
Ciclev10025280m.g	scaffold_7:15456947-15460803	69.3691	17.8278	-1.96017
Ciclev10014973m.g	scaffold_2:35088624-35090531	2.98338	0.767297	-1.95909
Ciclev10024083m.g	scaffold_3:31770209-32016675	1.1684	0.300741	-1.95794
Ciclev10029880m.g	scaffold_8:21056691-21057474	1.37576	0.354299	-1.95719
Ciclev10001636m.g	scaffold_5:39928704-39930585	99.372	25.6003	-1.95668
Ciclev10032323m.g	scaffold_4:23897860-23901686	25.9279	6.68997	-1.95443
Ciclev10026434m.g	scaffold_7:16614910-16616240	4.83429	1.24801	-1.95367
Ciclev10031403m.g	scaffold_4:1303839-1306777	3.02743	0.781614	-1.95357
Ciclev10017189m.g	scaffold_2:11903289-11903833	1.63503	0.422617	-1.95189
Ciclev10006076m.g	scaffold_9:744345-745408	422.118	109.161	-1.9512
Ciclev10003738m.g	scaffold_5:40017023-40019409	10.6195	2.75062	-1.94888
Ciclev10016441m.g	scaffold_2:34028656-34032642	0.593333	0.153706	-1.94867
Ciclev10024767m.g	scaffold_7:3403053-3409092	7.4306	1.92542	-1.94831
Ciclev10006541m.g	scaffold_9:28462025-28466344	0.324717	0.0841695	-1.94781
Ciclev10029921m.g	scaffold_8:3843789-3844502	1.40869	0.365408	-1.94677
Ciclev10011117m.g	scaffold_6:15424850-15429724	11.2506	2.91989	-1.94601
Ciclev10002301m.g	scaffold_5:37372295-37374350	20.8002	5.39907	-1.94581
Ciclev10006286m.g	scaffold_9:2084224-2084935	479.044	124.348	-1.94578
Ciclev10005967m.g	scaffold_9:30374696-30381795	10.5165	2.73054	-1.9454
Ciclev10006074m.g	scaffold_9:1295418-1296297	7.19285	1.86887	-1.9444
Ciclev10007602m.g	scaffold_1:6368607-6374391	5.29779	1.37678	-1.9441
Ciclev10020723m.g	scaffold_3:8427637-8430390	80.1	20.8203	-1.94381
Ciclev10020116m.g	scaffold_3:39072115-39079586	180.097	46.8696	-1.94205
Ciclev10001380m.g	scaffold_5:38491114-38492945	25.1503	6.55192	-1.94058
Ciclev10012328m.g	scaffold_6:21780333-21781641	2.46449	0.642375	-1.9398
Ciclev10019309m.g	scaffold_3:45385156-45387558	2.82153	0.735652	-1.93938

Ciclev10026559m.g	scaffold_7:13022420-13023593	3.4282	0.894318	-1.93859
Ciclev10025715m.g	scaffold_7:1880857-1882892	32.0908	8.38124	-1.93692
Ciclev10009893m.g	scaffold_1:4752414-4753820	127.211	33.2347	-1.93646
Ciclev10020342m.g	scaffold_3:45233398-45241100	11.8106	3.08814	-1.93527
Ciclev10016106m.g	scaffold_2:26016188-26017223	3.45521	0.904841	-1.93304
Ciclev10031507m.g	scaffold_4:23130215-23131775	9.43833	2.47443	-1.93143
Ciclev10002620m.g	scaffold_5:11470191-11471157	17.3596	4.55367	-1.93063
Ciclev10009186m.g	scaffold_1:26473623-26476630	21.0442	5.52263	-1.92999
Ciclev10011493m.g	scaffold_6:23946605-23949478	6.00552	1.5787	-1.92755
Ciclev10015567m.g	scaffold_2:10202276-10205534	12.027	3.16665	-1.92525
Ciclev10014114m.g	scaffold_2:11943426-11947775	0.826404	0.217779	-1.92398
Ciclev10014991m.g	scaffold_2:22635763-22640239	1232.22	324.789	-1.92369
Ciclev10028034m.g	scaffold_8:2175780-2183619	1.07003	0.28246	-1.92153
Ciclev10002978m.g	scaffold_5:12470098-12470442	17.5435	4.63114	-1.9215
Ciclev10031845m.g,Ciclev10033583m.g	scaffold_4:7493700-7706546	23.3414	6.16191	-1.92144
Ciclev10020017m.g	scaffold_3:2073077-2074731	1.48233	0.391809	-1.91965
Ciclev10032615m.g	scaffold_4:16355234-16356284	85.2579	22.5664	-1.91766
Ciclev10012432m.g	scaffold_6:22067348-22069877	5.89214	1.56135	-1.916
Ciclev10014618m.g	scaffold_2:28282823-28590596	11.5924	3.0732	-1.91537
Ciclev10010618m.g	scaffold_1:1574099-1575910	0.258775	0.0686472	-1.91442
Ciclev10015469m.g	scaffold_2:24379680-24381884	12.8395	3.40622	-1.91435
Ciclev10026796m.g	scaffold_7:9237720-9238164	2.07918	0.551982	-1.91332
Ciclev10016454m.g	scaffold_2:8151969-8154993	48.1262	12.7779	-1.91317
Ciclev10012858m.g	scaffold_6:17376854-17377530	5.42332	1.44031	-1.9128
Ciclev10002567m.g	scaffold_5:34971111-34972764	6.80792	1.80863	-1.91232
Ciclev10029315m.g	scaffold_8:20424286-20425903	617.75	164.168	-1.91185
Ciclev10019280m.g	scaffold_3:3829741-3831896	131.911	35.0701	-1.91126
Ciclev10025153m.g	scaffold_7:12754890-12834217	6.6105	1.75781	-1.91098
Ciclev10017111m.g	scaffold_2:30862924-30863577	1.56738	0.417173	-1.90963
Ciclev10021329m.g	scaffold_3:3235233-3236815	3.03297	0.80881	-1.90686
Ciclev10019936m.g	scaffold_3:47876447-47878633	64.9863	17.3451	-1.90561
-	scaffold_5:3330444-3331668	68.4871	18.3017	-1.90386
Ciclev10011663m.g	scaffold_6:22739630-22741028	2.18013	0.582829	-1.90327
Ciclev10017118m.g	scaffold_2:10056013-10056639	17.5408	4.69085	-1.90279
Ciclev10014492m.g	scaffold_2:28826133-28829533	53.5434	14.3268	-1.90199
Ciclev10004689m.g	scaffold_9:2230933-2233792	392.517	105.211	-1.89947
Ciclev10031390m.g	scaffold_4:24362007-24364244	28.2174	7.56896	-1.89842
Ciclev10031705m.g	scaffold_4:19720290-19721661	1.4403	0.386374	-1.8983
Ciclev10020940m.g	scaffold_3:41848676-41854429	2.34891	0.630857	-1.89661
Ciclev10015976m.g	scaffold_2:33774600-33777882	10.7754	2.89406	-1.89658
-	scaffold_5:8945-9188	60.9011	16.3603	-1.89627
Ciclev10006217m.g	scaffold_9:28421143-28423180	40.8104	10.9686	-1.89556
Ciclev10001618m.g	scaffold_5:42228571-42230813	29.7224	7.9906	-1.89517
Ciclev10023136m.g	scaffold_3:42882908-42885640	25.0451	6.73594	-1.89458
-	scaffold_2:27865392-27865939	56.5809	15.2182	-1.89452
Ciclev10022909m.g	scaffold_3:31620762-31715908	980.601	263.771	-1.89438
Ciclev10031341m.g	scaffold_4:10959748-10962466	14.47	3.89674	-1.89272
Ciclev10024477m.g	scaffold_3:13964885-13966737	2.12045	0.571225	-1.89224
Ciclev10015068m.g	scaffold_2:27279579-27284866	44.0333	11.863	-1.89213
-	scaffold_9:19365492-19369118	33.9893	9.16044	-1.89159
Ciclev10021607m.g	scaffold_3:7083372-7084271	5.41933	1.46097	-1.89119
Ciclev10020385m.g	scaffold_3:47202108-47206941	30.2105	8.14602	-1.89088
Ciclev10003218m.g	scaffold_5:31873014-31876484	1.91117	0.515804	-1.88956
Ciclev10019609m.g	scaffold_3:37906089-37910975	1.64615	0.444907	-1.88752
Ciclev10011917m.g	scaffold_6:18332185-18334838	1.66477	0.450924	-1.88437

Ciclev10006916m.g	scaffold_9:27992868-27993378	1.91025	0.517586	-1.88389
Ciclev10030868m.g	scaffold_4:19891311-19894519	3.31788	0.899391	-1.88324
Ciclev10002023m.g	scaffold_5:40740373-40741597	5.49842	1.49096	-1.88278
Ciclev10023327m.g	scaffold_3:8857722-8858955	2.63156	0.714577	-1.88075
Ciclev10008957m.g	scaffold_1:27733135-27734134	2.62146	0.711947	-1.88053
Ciclev10017085m.g	scaffold_2:31396676-31397374	1.93729	0.526438	-1.87971
Ciclev10003845m.g	scaffold_5:42209335-42210351	26.4328	7.18567	-1.87914
-	scaffold_1:13563118-13572331	49.6401	13.4947	-1.87912
Ciclev10018112m.g	scaffold_2:7273711-7276393	2.46227	0.669736	-1.87832
Ciclev10009892m.g	scaffold_1:1676367-1677018	44.2086	12.03	-1.87769
Ciclev10016513m.g	scaffold_2:34807975-34810333	7.12567	1.93935	-1.87745
Ciclev10031440m.g	scaffold_4:16652627-16655000	2.77165	0.755003	-1.87619
Ciclev10012292m.g	scaffold_6:10816139-10936716	611.276	166.824	-1.8735
Ciclev10017421m.g	scaffold_2:32980874-32985696	72.8293	19.8887	-1.87257
Ciclev10011717m.g	scaffold_6:22011951-22016247	46.595	12.7247	-1.87254
Ciclev10008075m.g	scaffold_1:23326836-23328814	33.2188	9.07814	-1.87153
Ciclev10028107m.g	scaffold_8:13190040-13194829	69.4536	18.9859	-1.87112
Ciclev10005878m.g	scaffold_9:26426908-26430456	6.53461	1.78811	-1.86966
Ciclev10006211m.g	scaffold_9:26578135-26578846	5.30702	1.4563	-1.86559
Ciclev10016863m.g	scaffold_2:10795981-10797667	0.734723	0.201692	-1.86505
Ciclev10030236m.g	scaffold_8:19224319-19225160	1.29869	0.356561	-1.86484
Ciclev10031793m.g	scaffold_4:19605472-19608059	114.442	31.428	-1.8645
Ciclev10031996m.g	scaffold_4:23089670-23090833	5.84884	1.60653	-1.8642
Ciclev10017894m.g	scaffold_2:4598253-4598946	3.11487	0.856198	-1.86315
Ciclev10017778m.g	scaffold_2:31451288-31452190	0.918266	0.252496	-1.86265
Ciclev10003031m.g	scaffold_5:36766238-36766767	4.83842	1.33102	-1.862
Ciclev10031331m.g	scaffold_4:24971611-24985270	20.456	5.6291	-1.86155
Ciclev10002907m.g	scaffold_5:42014992-42015616	62.3227	17.169	-1.85996
Ciclev10023664m.g	scaffold_3:1888556-1892035	12.8145	3.53344	-1.85864
Ciclev10019962m.g	scaffold_3:49017414-49020095	0.982892	0.271026	-1.8586
Ciclev10028331m.g	scaffold_8:5612396-5663654	1.77816	0.491158	-1.85613
Ciclev10015406m.g	scaffold_2:30146184-30148379	8.04038	2.22185	-1.8555
Ciclev10001563m.g	scaffold_5:36550506-36552800	527.768	145.878	-1.85514
Ciclev10017794m.g	scaffold_2:7091005-7093729	1.31783	0.364994	-1.85222
Ciclev10033867m.g	scaffold_4:7493700-7706546	145.527	40.3264	-1.85149
Ciclev10019651m.g	scaffold_3:6499485-6501568	1.01947	0.282515	-1.85142
Ciclev10021106m.g	scaffold_3:50253005-50255392	2.74277	0.760727	-1.85018
Ciclev10000182m.g	scaffold_5:39477781-39484781	45.9355	12.755	-1.84855
Ciclev10000850m.g	scaffold_5:39495950-39498826	326.296	90.6735	-1.84743
Ciclev10021254m.g	scaffold_3:36363009-36553824	2.5754	0.715749	-1.84727
Ciclev10009250m.g	scaffold_1:1041085-1043376	4.16535	1.15927	-1.84522
Ciclev10005425m.g,Ciclev10005451m.g,Ciclev10006037m.g,Ciclev10006950m.g	scaffold_9:1567923-1599233	16.4861	4.58911	-1.84497
Ciclev10019828m.g	scaffold_3:348559-351141	0.938816	0.26144	-1.84436
Ciclev10017731m.g	scaffold_2:35303011-35304493	2.76434	0.769934	-1.84413
Ciclev10026904m.g	scaffold_7:16076145-16076620	14.5214	4.04755	-1.84306
Ciclev10009246m.g	scaffold_1:4043015-4044281	36.2613	10.1137	-1.84213
Ciclev10016664m.g	scaffold_2:25672324-25673455	4.08867	1.14146	-1.84075
Ciclev10020517m.g	scaffold_3:10404108-10407777	36.83	10.2833	-1.84058
Ciclev10022800m.g	scaffold_3:45299524-45303593	11.1966	3.12873	-1.83941
Ciclev10005617m.g,Ciclev10005670m.g	scaffold_9:14540536-14619383	24.9138	6.96572	-1.8386
Ciclev10031824m.g	scaffold_4:9505297-9506517	2.48103	0.693699	-1.83856
-	scaffold_9:27223271-27223540	46.3336	12.9588	-1.83813
Ciclev10000998m.g	scaffold_5:38363304-38367051	15.3773	4.31222	-1.8343

Ciclev10017848m.g	scaffold_2:20340312-20340724	2.12438	0.595761	-1.83424
Ciclev10030194m.g	scaffold_8:1795916-1796300	2.6682	0.748323	-1.83414
Ciclev10014952m.g	scaffold_2:14657848-14665608	1.5992	0.448599	-1.83385
Ciclev10025391m.g,Ciclev10025392m.g	scaffold_7:7194736-7222786	39.8644	11.1981	-1.83185
Ciclev10022851m.g	scaffold_3:47912700-47913512	61.4947	17.2757	-1.83172
Ciclev10015853m.g	scaffold_2:34718353-34720151	2.78161	0.782405	-1.82993
Ciclev10017104m.g	scaffold_2:33601731-33602996	30.9333	8.70424	-1.82937
Ciclev10004755m.g	scaffold_9:28984971-28989373	32.3672	9.10885	-1.82919
Ciclev10032441m.g	scaffold_4:18997584-18999613	89.3409	25.1884	-1.82656
Ciclev10008645m.g	scaffold_1:26820626-26825048	4.53194	1.27802	-1.82622
Ciclev10010836m.g	scaffold_1:28928260-28932927	3.73842	1.05481	-1.82544
Ciclev10011315m.g	scaffold_6:17791806-17794647	0.931176	0.262876	-1.82467
Ciclev10029998m.g	scaffold_8:7699848-7862562	8.30359	2.34438	-1.82453
Ciclev10027280m.g,Ciclev10027539m.g	scaffold_7:352102-357146	13.6124	3.84622	-1.82341
Ciclev10021862m.g	scaffold_3:12637866-12639182	8.43586	2.38801	-1.82073
Ciclev10031168m.g	scaffold_4:20982608-20988551	11.0055	3.11556	-1.82065
Ciclev10012982m.g	scaffold_6:12558710-12559233	1.30701	0.370574	-1.81843
Ciclev10020945m.g	scaffold_3:7526226-7527689	2.73136	0.774481	-1.81832
-	scaffold_1:21973198-21974850	14.6488	4.15415	-1.81816
Ciclev10033091m.g	scaffold_4:7366608-7367250	21.3989	6.06844	-1.81814
Ciclev10001371m.g	scaffold_5:29050806-29055968	159.537	45.2552	-1.81774
Ciclev10027054m.g	scaffold_7:11948804-11997291	3.45588	0.980526	-1.81743
Ciclev10007630m.g	scaffold_1:26000428-26003254	15.6388	4.44021	-1.81644
Ciclev10004326m.g	scaffold_9:2655671-2658618	2.62568	0.746264	-1.81493
Ciclev10022512m.g,Ciclev10022725m.g	scaffold_3:13698282-13704788	397.476	113.072	-1.81363
Ciclev10025133m.g	scaffold_7:12352172-12754529	1.77428	0.504762	-1.81356
Ciclev10002882m.g	scaffold_5:34791962-34793333	0.993114	0.282578	-1.81331
Ciclev10020882m.g	scaffold_3:39023498-39027970	76.7793	21.8565	-1.81266
Ciclev10025462m.g	scaffold_7:2341528-2343403	2.95399	0.841314	-1.81195
Ciclev10003671m.g	scaffold_5:41463512-41466022	1.20178	0.342497	-1.81101
Ciclev10016285m.g	scaffold_2:4424381-4427034	185.402	52.8615	-1.81037
Ciclev10009037m.g	scaffold_1:26946403-26948798	7.4206	2.11598	-1.81021
Ciclev10028667m.g	scaffold_8:22568165-22571151	160.489	45.7893	-1.80939
Ciclev10024534m.g	scaffold_3:16912550-16917880	7.6684	2.18939	-1.80839
-	scaffold_2:28282823-28590596	45.8081	13.08	-1.80824
Ciclev10020875m.g	scaffold_3:20132361-20135883	2.22336	0.63523	-1.80739
Ciclev10032280m.g	scaffold_4:24497388-24499313	17.7463	5.07149	-1.80703
Ciclev10008009m.g	scaffold_1:1149149-1151996	1.31331	0.375632	-1.80582
Ciclev10019963m.g	scaffold_3:40950523-40954728	133.31	38.138	-1.80548
Ciclev10005220m.g	scaffold_9:29969028-29970745	1.25009	0.357694	-1.80523
Ciclev10030019m.g	scaffold_8:20740038-20748864	2.60767	0.746469	-1.80461
Ciclev10006201m.g	scaffold_9:1828507-1829243	346.495	99.2276	-1.80402
Ciclev10026392m.g	scaffold_7:19458241-19460738	7.77655	2.22806	-1.80334
Ciclev10020883m.g	scaffold_3:17470554-17474419	14.0075	4.01406	-1.80307
Ciclev10032402m.g	scaffold_4:3875677-3879087	3.13587	0.898907	-1.80262
Ciclev10004620m.g	scaffold_9:7579899-7583093	1269.11	364.135	-1.80128
Ciclev10021349m.g	scaffold_3:27993864-27998641	4.20198	1.20781	-1.79867
Ciclev10014748m.g,Ciclev10014826m.g	scaffold_2:31211055-31225439	312.695	89.9599	-1.7974
Ciclev10019351m.g	scaffold_3:44215332-44220917	5.62859	1.62444	-1.79283
Ciclev10025685m.g	scaffold_7:20624956-20628592	1.00086	0.289868	-1.78778
Ciclev10011386m.g	scaffold_6:17113587-17116563	48.2128	13.9669	-1.78741
Ciclev10030691m.g	scaffold_4:22855851-22860862	45.1144	13.0728	-1.78702

Ciclev10014358m.g	scaffold_2:13919661-13923501	0.480493	0.139416	-1.78512
Ciclev10003463m.g	scaffold_5:17572998-17573997	31.306	9.0962	-1.7831
Ciclev10013413m.g	scaffold_6:21415823-21418494	5.15647	1.49967	-1.78173
Ciclev10026210m.g	scaffold_7:4531275-4532990	1279.37	372.213	-1.78123
Ciclev10017395m.g	scaffold_2:7832348-7846578	10.9124	3.17616	-1.78061
Ciclev10026801m.g	scaffold_7:14281893-14282247	9.6512	2.81499	-1.77758
Ciclev10020418m.g	scaffold_3:48506025-48509461	61.4791	17.9404	-1.77688
Ciclev10031098m.g	scaffold_4:21994422-21997477	6.44739	1.88222	-1.77628
Ciclev10005666m.g	scaffold_9:27698502-27699483	6.14281	1.79381	-1.77588
Ciclev10001869m.g	scaffold_5:32900140-32903124	32.8966	9.61059	-1.77524
Ciclev10025452m.g	scaffold_7:8340087-8344492	2.79592	0.816826	-1.77522
Ciclev10007746m.g	scaffold_1:20039958-20042536	3.41338	0.997951	-1.77416
Ciclev10016831m.g	scaffold_2:8546087-8548227	36.6345	10.7175	-1.77324
Ciclev10002859m.g	scaffold_5:41432161-41439014	71.9012	21.0555	-1.77182
Ciclev10031607m.g	scaffold_4:24716951-24718896	0.683347	0.200125	-1.77172
Ciclev10015185m.g	scaffold_2:5968187-5973023	1.47675	0.432963	-1.77011
Ciclev10010023m.g	scaffold_1:6479157-6480196	1.8403	0.539571	-1.77005
Ciclev10018774m.g	scaffold_3:27807171-27813248	3.38173	0.995736	-1.76393
Ciclev10023108m.g	scaffold_3:40176478-40209546	539.777	159.028	-1.76308
Ciclev10010038m.g	scaffold_1:6240403-6241344	15.0589	4.43673	-1.76304
Ciclev10009432m.g	scaffold_1:6317159-6317866	2.25205	0.663975	-1.76204
Ciclev10024881m.g	scaffold_7:1613689-1620021	12.7563	3.76536	-1.76035
Ciclev10027176m.g	scaffold_7:4670800-4676448	1.97553	0.583499	-1.75943
Ciclev10014947m.g	scaffold_2:23959295-23961957	15.0526	4.44787	-1.75883
Ciclev10015247m.g	scaffold_2:10050551-10055534	122.509	36.201	-1.75879
Ciclev10014060m.g	scaffold_2:29155997-29160335	0.245066	0.0724416	-1.75828
Ciclev10004948m.g	scaffold_9:1817993-1819854	31.0645	9.18491	-1.75793
Ciclev10022323m.g	scaffold_3:4376923-4378771	1.27365	0.377041	-1.75617
Ciclev10005345m.g	scaffold_9:24072148-24073547	6.35211	1.88234	-1.75471
Ciclev10014788m.g	scaffold_2:11824122-11827334	0.477331	0.141459	-1.7546
Ciclev10011852m.g	scaffold_6:20441370-20442942	0.612884	0.181817	-1.75313
Ciclev10032177m.g	scaffold_4:1166288-1169372	4.79131	1.42524	-1.74921
Ciclev10005597m.g	scaffold_9:12562561-12570023	10.6675	3.17319	-1.74921
Ciclev10010159m.g	scaffold_1:577734-581006	6.27256	1.86882	-1.74692
Ciclev10012523m.g	scaffold_6:24182173-24183883	16.0371	4.78487	-1.74486
Ciclev10022919m.g	scaffold_3:9743613-9744406	10.4026	3.1078	-1.74297
Ciclev10022928m.g	scaffold_3:49279536-49281019	5.27773	1.578	-1.74182
Ciclev10000102m.g	scaffold_5:41765024-41772007	31.9048	9.55263	-1.73981
Ciclev10028621m.g	scaffold_8:20607773-20611605	156.005	46.7982	-1.73707
Ciclev10022912m.g	scaffold_3:13122009-13122890	82.3621	24.7171	-1.73647
Ciclev10011009m.g	scaffold_6:24734910-24739850	0.602644	0.181169	-1.73397
Ciclev10012747m.g	scaffold_6:21782043-21783120	8.88131	2.67003	-1.73392
Ciclev10004630m.g	scaffold_9:25255735-25259023	0.627835	0.188902	-1.73275
Ciclev10019472m.g	scaffold_3:848754-852261	1.57718	0.474736	-1.73215
Ciclev10031033m.g	scaffold_4:22480681-22484154	8.81384	2.65523	-1.73093
Ciclev10014842m.g	scaffold_2:22362547-22366644	9.85365	2.96987	-1.73026
Ciclev10004284m.g	scaffold_9:24356650-24541643	0.623506	0.187964	-1.72995
Ciclev10013354m.g	scaffold_6:14353652-14356639	30.8447	9.30422	-1.72906
Ciclev10014629m.g	scaffold_2:34070830-34075209	0.785188	0.237268	-1.72652
Ciclev10027953m.g,Ciclev10027954m.g	scaffold_8:12800121-12845493	203.746	61.589	-1.72603
Ciclev10020837m.g	scaffold_3:6811815-6815073	28.2948	8.56847	-1.72343
Ciclev10032888m.g	scaffold_4:24255612-24257964	12.2041	3.70001	-1.72176
Ciclev10027808m.g	scaffold_8:18973473-19114160	5.51653	1.67271	-1.72158
-	scaffold_9:27106040-27106223	4822.34	1463.74	-1.72008

Ciclev10019877m.g,Ciclev10023773m.g	scaffold_3:21515578-21531402	66.3547	20.1567	-1.71894
Ciclev10000917m.g	scaffold_5:17461566-17463320	0.923767	0.280632	-1.71885
Ciclev10012108m.g	scaffold_6:19634013-19638215	12.8228	3.89712	-1.71823
Ciclev10019107m.g	scaffold_3:9414403-9419290	35.5096	10.7931	-1.71809
Ciclev10024846m.g	scaffold_7:10932123-10935698	3.98932	1.21325	-1.71727
Ciclev10022906m.g	scaffold_3:31620762-31715908	4345.54	1321.69	-1.71716
Ciclev10015965m.g	scaffold_2:35713955-35715897	42.6145	12.9618	-1.71708
Ciclev10009458m.g	scaffold_1:4267475-4268413	1.91513	0.583322	-1.71507
Ciclev10025912m.g	scaffold_7:2832674-2834027	4.12808	1.25788	-1.71447
Ciclev10025417m.g	scaffold_7:14844174-14846782	12.8725	3.9231	-1.71422
Ciclev10028003m.g	scaffold_8:286659-289583	5.74578	1.7518	-1.71367
Ciclev10017273m.g	scaffold_2:22722919-22723789	9.39823	2.86834	-1.71218
Ciclev10000827m.g	scaffold_5:41258613-41263689	6.59089	2.01267	-1.71136
-	scaffold_8:16495048-16495823	53.5384	16.3738	-1.70919
Ciclev10022668m.g	scaffold_3:46847633-46848378	4.66244	1.42628	-1.70883
Ciclev10024858m.g	scaffold_7:11027711-11030551	0.821229	0.251391	-1.70785
Ciclev10015452m.g	scaffold_2:24557241-24561827	111.671	34.1846	-1.70784
Ciclev10016341m.g	scaffold_2:33274227-33276034	13.108	4.01684	-1.70632
Ciclev10032209m.g	scaffold_4:75212-77797	75.7985	23.2299	-1.70619
Ciclev10001435m.g	scaffold_5:37388292-37391250	35.2258	10.8027	-1.70524
Ciclev10005038m.g	scaffold_9:3088508-3089928	722.657	222.062	-1.70235
Ciclev10014430m.g	scaffold_2:34501500-34504295	65.9692	20.2814	-1.70163
Ciclev10023101m.g	scaffold_3:2718185-2719011	44.4517	13.6664	-1.70161
Ciclev10024911m.g	scaffold_7:4805544-4809377	2.05565	0.632108	-1.70135
Ciclev10011715m.g,Ciclev10013108m.g	scaffold_6:12546929-12553718	214.042	65.8379	-1.7009
Ciclev10029391m.g	scaffold_8:19146289-19210612	26.2186	8.0899	-1.6964
Ciclev10006626m.g	scaffold_9:30244180-30246642	1.67624	0.517238	-1.69633
Ciclev10004977m.g	scaffold_9:29246789-29250126	8.67063	2.67622	-1.69594
-	scaffold_5:2580115-2580362	39.3945	12.1698	-1.69468
Ciclev10016998m.g	scaffold_2:25930307-25931908	182.468	56.4484	-1.69264
Ciclev10011832m.g	scaffold_6:2292117-2295362	21.7199	6.71937	-1.69262
-	scaffold_2:14746000-14746162	18911.2	5857.09	-1.69099
Ciclev10007809m.g	scaffold_1:16203906-16208234	173.555	53.7624	-1.69072
Ciclev10029566m.g	scaffold_8:17304294-17459957	1.72162	0.534238	-1.68821
Ciclev10020814m.g	scaffold_3:13749082-13753019	445.537	138.478	-1.68588
Ciclev10011848m.g	scaffold_6:8878181-8884354	303.486	94.4096	-1.68462
Ciclev10025938m.g,Ciclev10025957m.g	scaffold_7:8210740-8221720	137.838	42.894	-1.68413
Ciclev10012290m.g	scaffold_6:10816139-10936716	181.746	56.6017	-1.683
-	scaffold_3:29952473-29952712	67.9949	21.1958	-1.68165
Ciclev10032357m.g	scaffold_4:3083089-3084520	1.81759	0.566811	-1.68109
Ciclev10012121m.g	scaffold_6:13586707-13589828	5.64469	1.76043	-1.68097
-	scaffold_5:318528-319410	5.47518	1.70827	-1.68037
Ciclev10026364m.g	scaffold_7:16185817-16187223	62.1627	19.4266	-1.67802
Ciclev10002601m.g	scaffold_5:30613183-30615183	8.48918	2.65359	-1.67768
Ciclev10026803m.g	scaffold_7:9468244-9468907	2.65768	0.831881	-1.67572
Ciclev10016696m.g	scaffold_2:33746698-33749841	6.02982	1.88848	-1.67489
Ciclev10009294m.g	scaffold_1:3070376-3071739	11.4639	3.59146	-1.67446
Ciclev10001660m.g	scaffold_5:30531742-30535211	6.67335	2.09442	-1.67186
Ciclev10031493m.g	scaffold_4:17895342-17898031	3.35813	1.05446	-1.67116
Ciclev10030544m.g	scaffold_4:19947264-19954736	99.1074	31.1455	-1.66997
Ciclev10010049m.g	scaffold_1:27086758-27087231	15.1562	4.77227	-1.66716
Ciclev10029994m.g	scaffold_8:4178523-4179087	18.6727	5.88103	-1.66679
Ciclev10012250m.g	scaffold_6:15275664-15280221	230.843	72.7139	-1.66661

Ciclev10011029m.g	scaffold_6:20309804-20313653	4.88906	1.54291	-1.6639
Ciclev10009722m.g	scaffold_1:24764471-24765333	3.816	1.20465	-1.66344
Ciclev10003867m.g	scaffold_5:23818533-23819802	16.0688	5.08282	-1.66056
Ciclev10001340m.g	scaffold_5:34936798-34940733	7.21411	2.28248	-1.66022
Ciclev10022568m.g	scaffold_3:49483584-49489946	93.7254	29.6732	-1.65928
Ciclev10011627m.g	scaffold_6:20821525-20825227	31.8974	10.1062	-1.6582
Ciclev10000566m.g	scaffold_5:42555316-42559854	6.40903	2.03088	-1.658
Ciclev10024637m.g	scaffold_3:41697051-41697860	1.0426	0.330452	-1.65768
Ciclev10028242m.g	scaffold_8:2747670-2749378	17.4032	5.51841	-1.65702
Ciclev10001332m.g	scaffold_5:40427855-40429578	63.4687	20.1663	-1.6541
Ciclev10017407m.g	scaffold_2:6552251-6553118	3.063	0.974823	-1.65173
Ciclev10031241m.g	scaffold_4:17518784-17523468	170.05	54.1776	-1.65019
Ciclev10025238m.g	scaffold_7:7904864-7908319	210.104	66.9485	-1.64998
Ciclev10028907m.g	scaffold_8:1710548-1711561	1.92473	0.613758	-1.64891
-	scaffold_8:19211060-19211350	47.0345	15.0217	-1.64667
Ciclev10027967m.g	scaffold_8:4156439-4161350	12.627	4.03364	-1.64636
Ciclev10026890m.g	scaffold_7:8176336-8177031	9.32276	2.98214	-1.64441
Ciclev10012908m.g	scaffold_6:23161518-23163869	173.534	55.5826	-1.64251
Ciclev10014480m.g	scaffold_2:11054066-11057781	0.496485	0.159158	-1.64129
Ciclev10010422m.g	scaffold_1:19100681-19101041	9.27019	2.97183	-1.64125
Ciclev10016616m.g	scaffold_2:25723305-25725690	23.481	7.52938	-1.64089
Ciclev10005342m.g	scaffold_9:26117969-26159098	25.0208	8.04357	-1.63722
Ciclev10016920m.g	scaffold_2:27377594-27378867	10.1258	3.25566	-1.63701
Ciclev10021501m.g	scaffold_3:50399570-50401808	2.15559	0.694014	-1.63505
Ciclev10026022m.g	scaffold_7:9442634-9445710	2.37094	0.76348	-1.6348
Ciclev10006327m.g,Ciclev10006895m.g	scaffold_9:26389069-26391138	9.37157	3.01913	-1.63416
Ciclev10024934m.g	scaffold_7:7826602-7829404	6.23228	2.01087	-1.63194
Ciclev10024760m.g	scaffold_7:3850610-3863166	6.77726	2.18688	-1.63183
Ciclev10001851m.g	scaffold_5:40053323-40055053	18.1967	5.87253	-1.63162
Ciclev10022253m.g	scaffold_3:446846-448641	17.7679	5.73853	-1.63052
Ciclev10013731m.g	scaffold_6:355-1144	11.4902	3.71373	-1.62947
Ciclev10022528m.g	scaffold_3:7759856-7761731	19.6851	6.36524	-1.62882
Ciclev10031737m.g	scaffold_4:19318980-19320538	1.36888	0.442643	-1.62878
Ciclev10031340m.g	scaffold_4:16737522-16740944	2.89573	0.936626	-1.62838
Ciclev10026552m.g	scaffold_7:1381760-1382875	156.986	50.8895	-1.6252
Ciclev10026415m.g	scaffold_7:4975418-4981695	17.6455	5.72363	-1.6243
Ciclev10009966m.g	scaffold_1:18803915-18804969	157.75	51.1771	-1.62407
Ciclev10007412m.g	scaffold_1:4952748-4958215	12.0212	3.90009	-1.62401
Ciclev10016376m.g	scaffold_2:32646916-32649898	42.9971	13.9563	-1.62332
Ciclev10013661m.g	scaffold_6:15603247-15618784	1135.96	368.94	-1.62246
Ciclev10033427m.g	scaffold_4:20708796-20712687	4.37399	1.42165	-1.62139
Ciclev10001575m.g	scaffold_5:39105671-39108800	30.8649	10.0374	-1.62058
-	scaffold_5:29172891-29173169	29.524	9.60328	-1.62029
Ciclev10012937m.g	scaffold_6:20683218-20684669	95.4106	31.0354	-1.62024
Ciclev10013365m.g	scaffold_6:6577978-6580701	9.94418	3.23505	-1.62006
Ciclev10028774m.g	scaffold_8:1491362-1556953	11.4353	3.72201	-1.61934
Ciclev10021984m.g	scaffold_3:2265849-2267575	5.86743	1.91089	-1.61848
Ciclev10030143m.g	scaffold_8:5612396-5663654	17.882	5.82809	-1.61742
Ciclev10031282m.g	scaffold_4:15277369-15280873	2.74593	0.895473	-1.61657
Ciclev10006302m.g	scaffold_9:1067608-1069647	9.58844	3.12786	-1.61612
Ciclev10020762m.g	scaffold_3:3880733-3883449	401.607	131.053	-1.61563
Ciclev10025174m.g	scaffold_7:20874760-20878316	84.4989	27.6257	-1.61292
Ciclev10000630m.g	scaffold_5:34230287-34232445	12.9337	4.23061	-1.6122
Ciclev10031232m.g	scaffold_4:12359537-12362422	13.2064	4.32512	-1.61043
Ciclev10020328m.g	scaffold_3:49621664-49623875	3.74516	1.22661	-1.61036

Ciclev10024944m.g	scaffold_7:1851369-1855115	1.11449	0.365543	-1.60828
Ciclev10008459m.g	scaffold_1:552219-553956	6.09139	1.99917	-1.60737
Ciclev10031761m.g	scaffold_4:22166699-22169904	55.4693	18.2083	-1.60709
Ciclev10007906m.g	scaffold_1:28132429-28135867	51.3048	16.8491	-1.60642
Ciclev10025325m.g	scaffold_7:870924-875490	2.93627	0.965897	-1.60404
Ciclev10028631m.g	scaffold_8:24315719-24317671	3.65296	1.20198	-1.60365
Ciclev10018331m.g	scaffold_2:28845910-28867192	41.1914	13.5541	-1.60361
Ciclev10033970m.g	scaffold_4:16476947-16477838	18.7535	6.17159	-1.60345
Ciclev10000466m.g	scaffold_5:37349892-37356610	12.2115	4.02213	-1.60221
Ciclev10019025m.g	scaffold_3:7840951-7847272	14.2228	4.68597	-1.60178
Ciclev10008822m.g	scaffold_1:4163201-4166973	9.71497	3.20633	-1.59928
Ciclev10026351m.g	scaffold_7:8678321-8680075	92.2276	30.4686	-1.59787
Ciclev10033212m.g	scaffold_4:19435922-19437101	7.47712	2.47081	-1.5975
Ciclev10007426m.g,Ciclev10007475m.g	scaffold_1:19443215-19466175	32.155	10.6281	-1.59716
Ciclev10005971m.g	scaffold_9:3833804-3834728	9.35717	3.0929	-1.59711
Ciclev10009581m.g	scaffold_1:28022653-28025416	2.37736	0.786266	-1.59627
Ciclev10031937m.g	scaffold_4:23957033-23959284	21.8698	7.24356	-1.59417
Ciclev10011632m.g	scaffold_6:20032678-20037205	6.03435	2.00084	-1.59259
Ciclev10029720m.g	scaffold_8:803857-805726	1.62539	0.53943	-1.59128
Ciclev10021075m.g	scaffold_3:50263606-50266309	5.15261	1.71019	-1.59115
Ciclev10004756m.g	scaffold_9:3324700-3329271	1.76055	0.58465	-1.59038
Ciclev10028710m.g	scaffold_8:23163228-23167352	29.3967	9.76547	-1.58989
Ciclev10008649m.g	scaffold_1:17363822-17365612	86.5716	28.7701	-1.58932
Ciclev10032886m.g	scaffold_4:17162156-17164814	93.1754	30.9744	-1.58887
Ciclev10033422m.g	scaffold_4:19340420-19345467	28.965	9.63241	-1.58834
Ciclev10001805m.g	scaffold_5:36845846-36847355	3.9977	1.33028	-1.58744
Ciclev10018219m.g	scaffold_2:35590452-35594163	2.19532	0.730938	-1.58661
-	scaffold_4:11530289-11530697	34.1895	11.391	-1.58566
Ciclev10031694m.g	scaffold_4:11210878-11216325	58.6177	19.5381	-1.58504
Ciclev10014670m.g	scaffold_2:17174394-17177247	45.5307	15.1774	-1.58492
Ciclev10026901m.g	scaffold_7:16616365-16618215	1.66027	0.553904	-1.58371
Ciclev10012342m.g	scaffold_6:16485915-16487373	16.8942	5.63753	-1.5834
Ciclev10019304m.g	scaffold_3:7744610-7750724	88.5179	29.619	-1.57945
Ciclev10000770m.g	scaffold_5:43153229-43157156	2.98915	1.00062	-1.57884
Ciclev10030777m.g,Ciclev10031026m.g	scaffold_4:1553214-1585115	35.1985	11.794	-1.57746
Ciclev10001139m.g	scaffold_5:39518032-39523027	14.8384	4.97836	-1.5756
Ciclev10018793m.g	scaffold_3:10250670-10255338	0.93307	0.313235	-1.57474
Ciclev10008832m.g	scaffold_1:23764336-23768786	29.4235	9.89835	-1.57171
Ciclev10010059m.g	scaffold_1:18812069-18812720	6.23565	2.1008	-1.5696
Ciclev10021157m.g	scaffold_3:10140903-10143672	17.7343	5.97745	-1.56895
Ciclev10020874m.g	scaffold_3:18517468-18520307	1.50523	0.507593	-1.56824
Ciclev10010056m.g	scaffold_1:19049995-19054408	1.40319	0.47345	-1.56742
Ciclev10017401m.g	scaffold_2:10097307-10100347	4.76069	1.60788	-1.56601
Ciclev10022032m.g	scaffold_3:43850268-43852816	30.4429	10.2872	-1.56525
Ciclev10005522m.g	scaffold_9:29880404-29881583	42.5306	14.3854	-1.5639
Ciclev10000825m.g	scaffold_5:40617690-40619818	74.4508	25.2342	-1.56091
Ciclev10009569m.g	scaffold_1:27514736-27515607	2.14209	0.726083	-1.56081
Ciclev10004660m.g	scaffold_9:2732899-2738896	71.0439	24.1226	-1.55833
Ciclev10020224m.g	scaffold_3:246167-250029	188.555	64.0354	-1.55804
Ciclev10011974m.g	scaffold_6:18937352-18940413	6.06934	2.0626	-1.55708
Ciclev10014428m.g	scaffold_2:30161875-30166336	35.6804	12.1344	-1.55603
Ciclev10018035m.g	scaffold_2:3069920-3070541	3.53962	1.20389	-1.55589
Ciclev10022307m.g	scaffold_3:4931883-4932873	84.3493	28.7445	-1.55309
Ciclev10007747m.g	scaffold_1:4234078-4236501	30.0203	10.2368	-1.55217

Ciclev10021151m.g	scaffold_3:31352097-31356324	32.5755	11.1084	-1.55214
Ciclev10033723m.g	scaffold_4:16817583-17013320	69.0499	23.6283	-1.54712
Ciclev10004864m.g	scaffold_9:29500558-29503424	2.98737	1.0231	-1.54592
Ciclev10025232m.g	scaffold_7:7898244-7901186	89.5261	30.6649	-1.54572
Ciclev10024794m.g	scaffold_7:12352172-12754529	2.82905	0.969949	-1.54434
Ciclev10025985m.g	scaffold_7:15182949-15186486	7.02138	2.40735	-1.54431
Ciclev10011191m.g	scaffold_6:15170699-15173761	1.35171	0.463677	-1.54359
Ciclev10012636m.g	scaffold_6:17945664-17947610	20.7312	7.11973	-1.54191
Ciclev10030485m.g	scaffold_4:13525690-13533032	3.37406	1.15881	-1.54185
Ciclev10025042m.g	scaffold_7:13125146-13127602	1.16809	0.401261	-1.54153
Ciclev10003772m.g	scaffold_5:33319302-33321501	222.505	76.4759	-1.54076
Ciclev10032657m.g	scaffold_4:12191110-12193951	4.59394	1.57913	-1.54061
Ciclev10028062m.g	scaffold_8:6922347-6926626	48.9657	16.838	-1.54005
Ciclev10004714m.g	scaffold_9:11247817-11252767	3.34115	1.14898	-1.53999
Ciclev10011719m.g	scaffold_6:22002890-22006522	227.431	78.3448	-1.53752
Ciclev10001867m.g	scaffold_5:33801544-33802689	51.9676	17.9146	-1.53648
Ciclev10013182m.g	scaffold_6:16349273-16352688	0.18571	0.0640194	-1.53647
Ciclev10001077m.g	scaffold_5:35578104-35582361	1.77059	0.611115	-1.53471
Ciclev10029730m.g	scaffold_8:4461644-4464561	1.92615	0.66542	-1.53338
Ciclev10024373m.g	scaffold_3:42114765-42116061	1.89608	0.655205	-1.533
Ciclev10000600m.g	scaffold_5:113390-116883	95.4422	32.9987	-1.53222
Ciclev10028876m.g,Ciclev10030431m.g	scaffold_8:22743891-22747501	25.3987	8.79411	-1.53015
Ciclev10007354m.g	scaffold_1:238855-247876	57.2307	19.8318	-1.52897
Ciclev10025125m.g	scaffold_7:8447413-8453714	100.252	34.7488	-1.52859
-	scaffold_9:1474217-1474760	6.17	2.13865	-1.52857
Ciclev10014192m.g	scaffold_2:6777394-6790546	4.31379	1.49619	-1.52767
Ciclev10031270m.g	scaffold_4:20564205-20569389	6.52037	2.26196	-1.52738
Ciclev10009482m.g	scaffold_1:24398799-24399607	5.09014	1.76733	-1.52614
Ciclev10027598m.g	scaffold_7:20780152-20781475	51.7391	17.973	-1.52542
Ciclev10008049m.g	scaffold_1:7045212-7048799	60.0472	20.877	-1.52419
Ciclev10024190m.g	scaffold_3:16871835-16874343	0.29406	0.102248	-1.52405
Ciclev10005589m.g	scaffold_9:729076-730936	61.9254	21.5358	-1.5238
Ciclev10022769m.g	scaffold_3:41391592-41394789	17.2178	5.99791	-1.52137
Ciclev10026978m.g	scaffold_7:9930764-9934489	0.171256	0.0597384	-1.51942
Ciclev10023166m.g	scaffold_3:19305598-19309195	1.09577	0.382504	-1.5184
Ciclev10002462m.g	scaffold_5:7350269-7556732	15.1521	5.29062	-1.51801
Ciclev10020990m.g	scaffold_3:23182642-23185114	17.0923	5.98316	-1.51437
Ciclev10031336m.g	scaffold_4:608211-615318	36.4587	12.7676	-1.51378
Ciclev10016939m.g	scaffold_2:4471155-4473536	46.4421	16.2649	-1.51367
Ciclev10001029m.g	scaffold_5:1084199-1143725	2.44116	0.854965	-1.51363
Ciclev10020663m.g	scaffold_3:49286521-49291600	20.7385	7.26485	-1.51331
Ciclev10030541m.g	scaffold_4:18415077-18422725	0.28882	0.101177	-1.51329
Ciclev10026471m.g	scaffold_7:9023226-9024094	0.559139	0.195913	-1.51299
Ciclev10024551m.g	scaffold_3:24991888-25030809	2.07248	0.726552	-1.51222
Ciclev10023789m.g	scaffold_3:25445567-25448031	0.361609	0.126783	-1.51207
Ciclev10021633m.g	scaffold_3:5865765-5866801	6.71154	2.35385	-1.51162
Ciclev10028164m.g	scaffold_8:20499093-20504304	20.8757	7.32339	-1.51124
Ciclev10024485m.g	scaffold_3:21982709-21986624	0.330103	0.115813	-1.51111
Ciclev10028248m.g,Ciclev10028465m.g	scaffold_8:13101221-13120570	78.533	27.5566	-1.5109
Ciclev10016629m.g	scaffold_2:2175205-2176049	199.94	70.231	-1.50939
Ciclev10017793m.g	scaffold_2:8036158-8041911	6.11519	2.1486	-1.509
Ciclev10021877m.g	scaffold_3:47803711-47805628	199.291	70.0594	-1.50822
Ciclev10027816m.g	scaffold_8:2412964-2419157	15.2926	5.37906	-1.5074
Ciclev10000756m.g	scaffold_5:19740132-19745850	28.4655	10.0144	-1.50714

Ciclev10008087m.g	scaffold_1:28827438-28830021	9.22723	3.24662	-1.50696
-	scaffold_2:23392855-23393288	34985.8	12322.3	-1.5055
Ciclev10028812m.g	scaffold_8:12531219-12533619	139.074	48.9891	-1.50532
Ciclev10020607m.g	scaffold_3:42534420-42536728	161.326	56.8484	-1.50479
Ciclev10022908m.g	scaffold_3:47351428-47352153	15.1962	5.35891	-1.5037
Ciclev10004240m.g	scaffold_9:24807735-24873775	49.081	17.321	-1.50265
Ciclev10022547m.g	scaffold_3:45900655-45901592	47.8399	16.889	-1.50213
Ciclev10001335m.g	scaffold_5:20599440-20601673	31.7934	11.2248	-1.50204
Ciclev10024869m.g,Ciclev10025053m.g	scaffold_7:8034110-8051948	5.83945	2.06437	-1.50013
Ciclev10008775m.g	scaffold_1:5686984-5689140	0.893586	0.315947	-1.49992
Ciclev10006817m.g	scaffold_9:29360866-29361294	29.2528	10.3512	-1.49877
Ciclev10027481m.g	scaffold_7:9138390-9140782	12.759	4.51824	-1.49769
Ciclev10025847m.g	scaffold_7:7779859-7782141	1.55803	0.55222	-1.49641
Ciclev10017141m.g	scaffold_2:35391011-35392163	358.471	127.069	-1.49624
Ciclev10033523m.g	scaffold_4:7493700-7706546	1.23044	0.436315	-1.49573
Ciclev10011789m.g	scaffold_6:22955787-22957989	3.46829	1.23111	-1.49427
Ciclev10009617m.g	scaffold_1:3383959-3384623	11.0611	3.92662	-1.49414
Ciclev10011127m.g	scaffold_6:25107388-25111099	0.372015	0.132124	-1.49347
-	scaffold_5:2578183-2578350	239.916	85.2497	-1.49276
Ciclev10006925m.g	scaffold_9:24554550-24557154	0.183989	0.0653826	-1.49264
Ciclev10013222m.g	scaffold_6:1378067-1378751	1.02859	0.365817	-1.49147
Ciclev10018678m.g	scaffold_3:35906755-35910117	0.840956	0.2991	-1.4914
Ciclev10013783m.g	scaffold_6:24972596-24974590	0.44762	0.15922	-1.49125
Ciclev10013905m.g	scaffold_6:64999-67276	0.683228	0.243087	-1.4909
Ciclev10014219m.g	scaffold_2:32903447-32914855	38.8209	13.8124	-1.49087
Ciclev10011474m.g	scaffold_6:24405133-24407191	21.4409	7.62955	-1.4907
Ciclev10010968m.g	scaffold_6:16069001-16072484	0.162367	0.0577794	-1.49064
Ciclev10008884m.g	scaffold_1:8865235-8868711	33.5987	11.9621	-1.48994
Ciclev10006124m.g	scaffold_9:22114942-22116474	592.85	211.13	-1.48954
Ciclev10019888m.g	scaffold_3:47046134-47049135	5.15918	1.83757	-1.48934
Ciclev10030808m.g	scaffold_4:24123582-24127783	0.211738	0.0754544	-1.4886
Ciclev10004275m.g	scaffold_9:2424914-2429331	0.163826	0.0583986	-1.48815
Ciclev10018816m.g	scaffold_3:27737585-27740735	0.193312	0.0689284	-1.48776
Ciclev10006315m.g	scaffold_9:30040456-30046344	59.7628	21.322	-1.4869
Ciclev10019941m.g	scaffold_3:1544508-1547621	44.9338	16.0379	-1.48631
Ciclev10017360m.g	scaffold_2:10640192-10646119	11.8593	4.23444	-1.48577
Ciclev10025950m.g	scaffold_7:8438701-8442381	273.169	97.5802	-1.48513
Ciclev10007424m.g	scaffold_1:678920-682015	0.172809	0.0618187	-1.48307
Ciclev10025217m.g	scaffold_7:5884672-5887407	4.33755	1.55171	-1.48302
Ciclev10000688m.g	scaffold_5:18358820-18361410	0.274865	0.0983754	-1.48236
Ciclev10000934m.g	scaffold_5:34345967-34375527	5.54091	1.98328	-1.48223
Ciclev10010366m.g	scaffold_1:943321-945735	0.374751	0.134331	-1.48014
Ciclev10008695m.g	scaffold_1:26163937-26169507	5.86706	2.10469	-1.47903
Ciclev10018771m.g	scaffold_3:31140607-31147183	0.311575	0.111813	-1.4785
Ciclev10023520m.g	scaffold_3:30066338-30252280	0.439491	0.157792	-1.47781
Ciclev10020206m.g	scaffold_3:45205979-45211054	8.50722	3.05618	-1.47696
Ciclev10015726m.g	scaffold_2:36178013-36179176	3.2567	1.16996	-1.47695
Ciclev10014971m.g	scaffold_2:8906363-8909753	1.53056	0.550011	-1.47652
Ciclev10018670m.g	scaffold_3:46090114-46095977	3.64591	1.31124	-1.47535
-	scaffold_3:46141979-46142326	19.764	7.10923	-1.47511
Ciclev10007444m.g	scaffold_1:6481259-6485491	0.814415	0.292955	-1.47508
Ciclev10015287m.g	scaffold_2:24333955-24336070	25.7512	9.26326	-1.47505
Ciclev10007422m.g	scaffold_1:17704705-17802486	0.164172	0.0590637	-1.47486
Ciclev10000018m.g	scaffold_5:18100083-18137609	7.04134	2.53369	-1.47461
Ciclev10030257m.g	scaffold_8:6057617-6062060	0.201776	0.07261	-1.47451

Ciclev10021269m.g	scaffold_3:48446076-48449604	0.483212	0.173925	-1.47419
Ciclev10013901m.g	scaffold_6:17219987-17220953	0.618176	0.222531	-1.47401
Ciclev10010358m.g	scaffold_1:1627613-1630842	0.512308	0.184422	-1.474
Ciclev10025498m.g	scaffold_7:10007336-10013431	0.523045	0.188291	-1.47397
Ciclev10033918m.g	scaffold_4:25375725-25378032	0.187801	0.0676203	-1.47368
Ciclev10030779m.g	scaffold_4:3209099-3212422	1.92211	0.692152	-1.47353
-	scaffold_5:17540360-17540546	7970.74	2870.59	-1.47337
Ciclev10024929m.g	scaffold_7:3292992-3295657	0.171543	0.0618028	-1.47283
Ciclev10004698m.g	scaffold_9:8376736-8414192	0.284872	0.102647	-1.47262
Ciclev10008983m.g	scaffold_1:22038516-22041113	30.3942	10.9554	-1.47215
Ciclev10011302m.g	scaffold_6:23579127-23582757	9.03091	3.2557	-1.4719
Ciclev10002442m.g	scaffold_5:40300305-40304768	2.97416	1.07228	-1.4718
Ciclev10013588m.g	scaffold_6:15678836-15684730	0.157458	0.0567816	-1.47147
Ciclev10031906m.g	scaffold_4:25580547-25582930	0.893774	0.32245	-1.47083
Ciclev10010825m.g	scaffold_1:5054550-5056146	12.4833	4.50463	-1.47052
Ciclev10009242m.g	scaffold_1:1430454-1434069	32.3107	11.6599	-1.47046
Ciclev10017000m.g	scaffold_2:261439-264627	18.5837	6.7108	-1.46948
Ciclev10026433m.g	scaffold_7:1507803-1510299	0.874552	0.315822	-1.46943
Ciclev10000586m.g	scaffold_5:33902777-33906708	0.221902	0.0801545	-1.46906
Ciclev10022222m.g	scaffold_3:35325266-35326970	0.530072	0.191521	-1.46868
Ciclev10013062m.g	scaffold_6:18750204-18752251	33.115	11.9661	-1.46853
Ciclev10006903m.g	scaffold_9:25744567-25747429	0.618414	0.223493	-1.46834
Ciclev10026950m.g	scaffold_7:17934378-17935818	1.14108	0.412478	-1.46802
Ciclev10022989m.g	scaffold_3:45381554-45381989	72.4037	26.1902	-1.46704
Ciclev10004912m.g	scaffold_9:27015821-27051136	68.9761	24.952	-1.46694
Ciclev10013925m.g	scaffold_2343:15-1400	13.244	4.79122	-1.46687
Ciclev10011960m.g	scaffold_6:12664435-12665581	1.02895	0.372336	-1.4665
Ciclev10025329m.g	scaffold_7:5159994-5165972	73.8446	26.7263	-1.46623
Ciclev10006910m.g	scaffold_9:29779396-29781905	1.8713	0.677294	-1.46619
Ciclev10023364m.g	scaffold_3:48420367-48420695	57.7922	20.9323	-1.46514
-	scaffold_2:28282823-28590596	11.996	4.34568	-1.4649
-	scaffold_5:28289811-28801386	72.7705	26.3734	-1.46427
Ciclev10023108m.g	scaffold_3:40176478-40209546	1.44887	0.525288	-1.46375
Ciclev10029007m.g	scaffold_8:478150-479719	167.908	60.8887	-1.46342
Ciclev10029015m.g	scaffold_8:21875916-21880999	7.82426	2.8382	-1.46298
Ciclev10020065m.g	scaffold_3:25470794-25473216	0.371745	0.134874	-1.4627
Ciclev10027132m.g	scaffold_7:7457152-7458577	0.35224	0.127877	-1.4618
Ciclev10013859m.g	scaffold_6:18569400-18571341	2.95919	1.07436	-1.46173
Ciclev10009660m.g	scaffold_1:891332-892160	8.87925	3.22378	-1.46169
Ciclev10007296m.g	scaffold_1:1981538-2047130	57.228	20.7931	-1.46062
Ciclev10000806m.g	scaffold_5:42079047-42083003	0.515406	0.187354	-1.45994
-	scaffold_9:27106338-27106517	11741	4269.8	-1.45932
Ciclev10030928m.g	scaffold_4:2717013-2719856	174.216	63.3762	-1.45887
Ciclev10033117m.g	scaffold_4:19355160-19355952	3.97666	1.44918	-1.45632
Ciclev10000884m.g,Ciclev10000902m.g,Ciclev10001179m.g	scaffold_5:4471259-4568404	31.1415	11.3516	-1.45594
Ciclev10010681m.g	scaffold_1:17604570-17605299	43.3264	15.7951	-1.45576
Ciclev10014868m.g	scaffold_2:28867593-28870392	2.62704	0.958017	-1.45531
Ciclev10011338m.g	scaffold_6:21028836-21031557	1.35992	0.495929	-1.45531
Ciclev10029878m.g	scaffold_8:4292179-4370290	0.306563	0.111804	-1.45521
Ciclev10029187m.g	scaffold_8:4131965-4132968	18.6884	6.81741	-1.45485
Ciclev10029790m.g	scaffold_8:21682089-21684075	0.566174	0.206664	-1.45396
Ciclev10005805m.g	scaffold_9:7646164-7647453	0.308739	0.112714	-1.45371
Ciclev10017002m.g	scaffold_2:36140662-36142765	68.9015	25.1649	-1.45312
-	scaffold_2:23400957-23401121	417.253	152.451	-1.45258

Ciclev10024980m.g	scaffold_7:7295516-7300054	49.4778	18.0812	-1.45229
Ciclev10022082m.g	scaffold_3:46124041-46130078	77.0694	28.172	-1.4519
Ciclev10027595m.g	scaffold_7:13510252-13511938	0.294358	0.10761	-1.45176
Ciclev10015686m.g	scaffold_2:34638803-34640718	0.34062	0.124532	-1.45165
Ciclev10017589m.g	scaffold_2:29110316-29112979	2.77488	1.01503	-1.45091
Ciclev10022279m.g	scaffold_3:50687205-50688531	1.7071	0.624478	-1.45083
Ciclev10007940m.g,Ciclev10007942m.g	scaffold_1:24964825-24981789	13.5117	4.94457	-1.45029
Ciclev10025101m.g	scaffold_7:20320963-20325082	0.224818	0.0822845	-1.45006
Ciclev10027827m.g	scaffold_8:242707-246711	2.93167	1.07314	-1.44989
Ciclev10012575m.g	scaffold_6:23145894-23148874	13.6791	5.00801	-1.44966
Ciclev10030157m.g	scaffold_8:23765296-23767237	0.223537	0.0818664	-1.44917
Ciclev10033419m.g	scaffold_4:22988925-22990379	8.48256	3.10792	-1.44855
Ciclev10004707m.g	scaffold_9:25094081-25099067	13.0598	4.78935	-1.44723
Ciclev10020083m.g	scaffold_3:12791629-12795091	62.3785	22.8812	-1.44688
Ciclev10017943m.g	scaffold_2:28908048-28909959	0.408261	0.149765	-1.4468
Ciclev10022657m.g	scaffold_3:37169470-37170698	1.05297	0.386672	-1.44528
Ciclev10026240m.g	scaffold_7:17340784-17343713	0.766654	0.281667	-1.44459
Ciclev10002208m.g	scaffold_5:34896600-34897733	0.992532	0.364661	-1.44456
Ciclev10010150m.g	scaffold_1:22635900-22637346	0.631654	0.232165	-1.44399
Ciclev10015492m.g	scaffold_2:23762751-23765126	28.7497	10.5675	-1.44391
Ciclev10011369m.g	scaffold_6:19558581-19562546	0.8065	0.296446	-1.4439
Ciclev10018050m.g	scaffold_2:25364550-25367417	0.781299	0.287193	-1.44385
Ciclev10021650m.g	scaffold_3:48018377-48020545	11.9149	4.37993	-1.44379
Ciclev10004956m.g	scaffold_9:28693001-28697846	114.349	42.0394	-1.44364
Ciclev10021802m.g	scaffold_3:123864-125349	0.435943	0.160272	-1.44362
Ciclev10001515m.g	scaffold_5:43079827-43083323	0.744989	0.27389	-1.44362
Ciclev10011175m.g	scaffold_6:10016563-10019369	63.1361	23.2211	-1.44303
Ciclev10004781m.g	scaffold_9:26796473-26798774	1.35322	0.497709	-1.44303
Ciclev10011022m.g	scaffold_6:12863237-12867758	1.69403	0.62314	-1.44283
Ciclev10011234m.g	scaffold_6:12362571-12364596	1.28793	0.473895	-1.44241
Ciclev10009663m.g	scaffold_1:25072653-25073652	100.753	37.0825	-1.44201
Ciclev10003108m.g	scaffold_5:24277909-24280261	6.65535	2.45	-1.44173
Ciclev10011847m.g	scaffold_6:19822789-19824194	0.380424	0.140047	-1.4417
Ciclev10017895m.g	scaffold_2:28282823-28590596	23.3941	8.61652	-1.44097
Ciclev10030731m.g	scaffold_4:12328244-12330656	1.17311	0.432692	-1.43892
Ciclev10012428m.g	scaffold_6:21803726-21804560	0.78019	0.287814	-1.43869
Ciclev10003971m.g	scaffold_5:24035767-24039477	0.194836	0.0719177	-1.43784
-	scaffold_8:7178004-7178286	44.6039	16.4644	-1.43782
Ciclev10001155m.g	scaffold_5:40597271-40598741	3.00127	1.10829	-1.43723
Ciclev10032585m.g	scaffold_4:3102074-3103327	5.63246	2.0804	-1.4369
Ciclev10033827m.g	scaffold_4:23656910-23658886	0.469098	0.173305	-1.43658
Ciclev10017543m.g	scaffold_2:36066568-36067618	2.2983	0.849681	-1.43557
Ciclev10028154m.g	scaffold_8:13414399-13417017	0.224657	0.0831154	-1.43454
Ciclev10005436m.g	scaffold_9:22368666-22372918	66.846	24.7351	-1.43428
Ciclev10030927m.g	scaffold_4:23426700-23429072	8.02026	2.96989	-1.43324
Ciclev10020958m.g	scaffold_3:363597-364689	0.827174	0.306399	-1.43278
Ciclev10033865m.g	scaffold_4:14692996-14694933	0.328919	0.121899	-1.43205
Ciclev10017627m.g	scaffold_2:17347948-17351501	13.5128	5.00984	-1.4315
Ciclev10026223m.g	scaffold_7:8255045-8256442	19.3044	7.15826	-1.43125
Ciclev10017131m.g	scaffold_2:5957911-5958497	12.6692	4.70124	-1.43021
Ciclev10027028m.g	scaffold_7:17486660-17489826	1.62514	0.603144	-1.42999
Ciclev10033073m.g	scaffold_4:19359941-19360737	0.962639	0.357415	-1.42939
Ciclev10029847m.g	scaffold_8:22022370-22024705	9.908	3.67978	-1.42897
Ciclev10003091m.g	scaffold_5:39466847-39468398	0.712841	0.26477	-1.42884
Ciclev10032154m.g	scaffold_4:24623710-24627543	5.81353	2.16045	-1.42808

Ciclev10011883m.g	scaffold_6:25477064-25480914	9.45094	3.51223	-1.42807
Ciclev10033695m.g	scaffold_4:21782302-21783402	0.527133	0.196113	-1.42648
Ciclev10002796m.g	scaffold_5:39444680-39445917	8.4429	3.14466	-1.42483
Ciclev10006936m.g	scaffold_9:30157877-30161153	0.266446	0.0992608	-1.42455
Ciclev10025216m.g	scaffold_7:2984807-2989107	2.53001	0.942813	-1.4241
Ciclev10024589m.g	scaffold_3:37868703-37880417	0.466574	0.173905	-1.42381
Ciclev10033777m.g	scaffold_4:21719885-21721178	0.805535	0.300518	-1.4225
Ciclev10028274m.g	scaffold_8:13035173-13037307	86.7503	32.3859	-1.4215
Ciclev10012635m.g	scaffold_6:18451375-18452564	53.5744	20.0012	-1.42146
Ciclev10022834m.g	scaffold_3:39852204-39854254	13.997	5.22773	-1.42086
Ciclev10015035m.g	scaffold_2:28168729-28171678	8.62612	3.22181	-1.42084
Ciclev10000195m.g	scaffold_5:42861981-42867071	8.6937	3.24842	-1.42023
Ciclev10006884m.g	scaffold_9:26084767-26085938	18.9687	7.08854	-1.42006
Ciclev10002596m.g	scaffold_5:22718835-22719826	0.744174	0.278189	-1.41957
Ciclev10011109m.g	scaffold_6:19699519-19703543	28.3182	10.5936	-1.41853
Ciclev10031169m.g	scaffold_4:10728378-10731133	9.39899	3.51632	-1.41844
Ciclev10018603m.g	scaffold_3:40475280-40479296	2.32141	0.868812	-1.41789
Ciclev10031834m.g	scaffold_4:12571525-12575185	2.66181	0.996881	-1.41691
Ciclev10014531m.g	scaffold_2:8727345-8730038	2.63055	0.985749	-1.41607
Ciclev10029254m.g	scaffold_8:13288298-13289998	31.5355	11.8183	-1.41596
Ciclev10018385m.g	scaffold_2:28282823-28590596	11.6574	4.36913	-1.41583
Ciclev10018044m.g	scaffold_2:367178-368057	0.546976	0.205131	-1.41493
Ciclev10015764m.g	scaffold_2:9149551-9151269	3.56159	1.33589	-1.41472
Ciclev10009025m.g	scaffold_1:25084405-25087091	536.715	201.371	-1.4143
Ciclev10004530m.g	scaffold_9:25713468-25717244	0.813158	0.305177	-1.41389
Ciclev10001756m.g	scaffold_5:34978110-34983399	149.96	56.2912	-1.41359
Ciclev10000897m.g	scaffold_5:42272202-42276387	9.79905	3.67927	-1.41322
Ciclev10002041m.g	scaffold_5:40637572-40639211	1.40677	0.528469	-1.41249
Ciclev10031570m.g	scaffold_4:19572950-19576064	10.5599	3.96704	-1.41246
Ciclev10028119m.g	scaffold_8:2334262-2336645	0.708885	0.266407	-1.41192
Ciclev10028549m.g	scaffold_8:851241-853420	1.66114	0.624767	-1.41078
Ciclev10022177m.g	scaffold_3:42043036-42044066	7.65287	2.87905	-1.41041
Ciclev10002644m.g	scaffold_5:38780108-38781399	0.416034	0.156553	-1.41005
Ciclev10001723m.g	scaffold_5:25625107-25626537	0.291509	0.109794	-1.40874
Ciclev10029331m.g	scaffold_8:1443946-1489074	0.909756	0.34266	-1.4087
Ciclev10018350m.g	scaffold_2:11400151-11401889	0.688989	0.259566	-1.40838
Ciclev10011965m.g	scaffold_6:20053810-20059518	21.4646	8.08718	-1.40825
Ciclev10004385m.g	scaffold_9:4888530-4892972	1.57538	0.59376	-1.40775
Ciclev10003023m.g	scaffold_5:34657438-34659553	47.6444	17.9581	-1.40768
Ciclev10032400m.g	scaffold_4:2252412-2254829	12.3697	4.66393	-1.40719
Ciclev10016732m.g	scaffold_2:27237004-27240127	86.1008	32.4704	-1.4069
Ciclev10016611m.g	scaffold_2:26829274-26830778	1.47285	0.555514	-1.40671
Ciclev10020415m.g	scaffold_3:11687448-12154789	22.1575	8.35741	-1.40667
Ciclev10021747m.g	scaffold_3:4644459-4648658	3.67116	1.38486	-1.4065
Ciclev10001511m.g	scaffold_5:33916070-33917739	2.3369	0.881771	-1.40612
Ciclev10011736m.g	scaffold_6:22166951-22168833	43.9084	16.57	-1.40592
Ciclev10017867m.g	scaffold_2:34240827-34242711	1.46005	0.550992	-1.40591
Ciclev10006682m.g	scaffold_9:4142538-4146072	0.362357	0.136797	-1.40538
Ciclev10025686m.g	scaffold_7:17272836-17304761	9.77539	3.69111	-1.4051
Ciclev10013693m.g	scaffold_6:14918021-15036696	3.43405	1.29687	-1.40487
-	scaffold_5:24533398-24534008	4.7252	1.78481	-1.40461
Ciclev10005024m.g	scaffold_9:3580467-3584207	0.748458	0.283043	-1.4029
Ciclev10024520m.g	scaffold_3:15950728-15954114	1.2451	0.470871	-1.40286
Ciclev10000654m.g	scaffold_5:12677413-12680761	170.524	64.5055	-1.40248
Ciclev10025342m.g	scaffold_7:5611185-5614200	3.48859	1.31974	-1.40239
Ciclev10029367m.g	scaffold_8:14277630-14278898	30.846	11.6712	-1.40213

Ciclev10032404m.g	scaffold_4:19845900-19847050	0.328708	0.124422	-1.40157
Ciclev10022407m.g	scaffold_3:43406102-43407095	20.9094	7.91524	-1.40144
Ciclev10007805m.g	scaffold_1:24281879-24283664	0.40728	0.154179	-1.40141
Ciclev10000909m.g	scaffold_5:101796-108313	5.2368	1.98309	-1.40093
Ciclev10025286m.g	scaffold_7:13323582-13328683	41.6865	15.7869	-1.40085
Ciclev10022272m.g	scaffold_3:41601980-41602845	0.712238	0.269809	-1.40042
Ciclev10025449m.g	scaffold_7:9024844-9028598	0.325097	0.123162	-1.40032
Ciclev10026270m.g	scaffold_7:4392029-4392845	3.74723	1.42029	-1.39964
Ciclev10026247m.g	scaffold_7:20305386-20307351	0.368174	0.139569	-1.39941
Ciclev10031212m.g	scaffold_4:25285785-25288711	21.9185	8.312	-1.39888
Ciclev10030291m.g	scaffold_8:19146289-19210612	13.4255	5.0943	-1.39802
Ciclev10025717m.g	scaffold_7:15872235-15875783	62.8407	23.8525	-1.39756
Ciclev10009710m.g	scaffold_1:20063995-20065196	22.2763	8.45572	-1.39751
Ciclev10019205m.g	scaffold_3:46730992-46737126	75.3847	28.6294	-1.39677
Ciclev10008442m.g	scaffold_1:27088283-27095331	83.6138	31.7847	-1.39541
Ciclev10007919m.g	scaffold_1:26032708-26035828	11.3272	4.3111	-1.39366
Ciclev10033990m.g	scaffold_4:354241-355274	12.4385	4.73447	-1.39354
Ciclev10010158m.g	scaffold_1:17627428-17628097	0.676462	0.257618	-1.39277
-	scaffold_5:23199930-23456438	55.3727	21.0973	-1.39212
Ciclev10000783m.g	scaffold_5:42445213-42449994	5.07909	1.93543	-1.39192
Ciclev10019872m.g	scaffold_3:41921729-41924701	6.21507	2.37022	-1.39075
Ciclev10033308m.g	scaffold_4:20509200-20510118	0.558349	0.213278	-1.38843
Ciclev10002029m.g	scaffold_5:19149108-19154358	46.6315	17.8227	-1.38759
-	scaffold_8:23426608-23426791	248.574	95.0479	-1.38695
Ciclev10012384m.g	scaffold_6:15896415-15898044	226.392	86.5806	-1.38671
Ciclev10019438m.g	scaffold_3:47990361-47993786	10.1587	3.88517	-1.38667
Ciclev10022449m.g	scaffold_3:49298532-49299308	0.591842	0.226575	-1.38522
Ciclev10012756m.g	scaffold_6:2445029-2447489	4.02201	1.54001	-1.38498
Ciclev10026756m.g	scaffold_7:8310614-8313304	70.2266	26.8921	-1.38484
Ciclev10014129m.g	scaffold_2:26050435-26057582	3.98992	1.5287	-1.38405
Ciclev10031206m.g	scaffold_4:23431991-23438332	40.9326	15.6882	-1.38357
Ciclev10016102m.g	scaffold_2:14178448-14179345	0.748111	0.286864	-1.38288
Ciclev10026574m.g	scaffold_7:8571966-8574224	0.623999	0.239412	-1.38205
Ciclev10021291m.g	scaffold_3:45584832-45586407	0.365896	0.140503	-1.38083
Ciclev10007685m.g	scaffold_1:19496171-19588291	3.46862	1.33277	-1.37994
Ciclev10015974m.g	scaffold_2:34208421-34209850	65.8905	25.3668	-1.37713
Ciclev10011060m.g	scaffold_6:13305252-13310801	4.9091	1.89108	-1.37625
Ciclev10022648m.g	scaffold_3:6332714-6333228	2.97604	1.14693	-1.37561
Ciclev10033380m.g	scaffold_4:7114783-7115935	0.377281	0.1454	-1.37561
Ciclev10021371m.g	scaffold_3:48545441-48550268	26.0016	10.0326	-1.37391
Ciclev10005680m.g	scaffold_9:8416603-8419944	24.372	9.40496	-1.37373
Ciclev10028649m.g	scaffold_8:2261181-2264145	29.5957	11.4222	-1.37355
Ciclev10018267m.g	scaffold_2:13869716-13870142	20.7517	8.009	-1.37353
Ciclev10031070m.g	scaffold_4:1742588-1745145	0.462581	0.178656	-1.37252
Ciclev10031955m.g	scaffold_4:3882717-3885303	104.505	40.3894	-1.37152
Ciclev10033622m.g	scaffold_4:2758837-2761074	44.1914	17.0834	-1.37117
Ciclev10024183m.g	scaffold_3:15662562-15663341	2.01566	0.779271	-1.37105
Ciclev10025189m.g	scaffold_7:8763666-8766821	10.1433	3.92222	-1.37079
Ciclev10001432m.g	scaffold_5:9941408-9943881	5.17795	2.00344	-1.3699
Ciclev10003889m.g	scaffold_5:8429091-8434975	2.20575	0.853648	-1.36956
Ciclev10032368m.g	scaffold_4:18423062-18426424	30.2197	11.6968	-1.36938
Ciclev10013118m.g	scaffold_6:2541593-2542087	0.944443	0.365561	-1.36935
Ciclev10017282m.g	scaffold_2:32987483-32989092	1.27118	0.49234	-1.36844
Ciclev10014296m.g	scaffold_2:26658861-26663240	47.6935	18.4745	-1.36826
Ciclev10022327m.g	scaffold_3:907954-909544	17.5637	6.80954	-1.36697
Ciclev10013705m.g	scaffold_6:23193271-23194498	2.95485	1.14651	-1.36583

Ciclev10022025m.g	scaffold_3:50492189-50493037	1.58499	0.615195	-1.36535
Ciclev10020570m.g	scaffold_3:4009157-4010937	13.966	5.4209	-1.36531
Ciclev10013572m.g	scaffold_6:13444057-13444441	7.34744	2.85219	-1.36517
Ciclev10025355m.g	scaffold_7:3594985-3597230	35.9236	13.9468	-1.365
Ciclev10011470m.g	scaffold_6:17775710-17778462	9.26584	3.59786	-1.36478
Ciclev10001649m.g	scaffold_5:26615176-26617732	18.9793	7.37237	-1.36423
Ciclev10009052m.g	scaffold_1:23062783-23065398	2.4998	0.971279	-1.36386
Ciclev10005815m.g	scaffold_9:153985-155186	0.328575	0.127669	-1.36381
Ciclev10029697m.g	scaffold_8:14795152-14795671	19.0506	7.40444	-1.36338
Ciclev10031301m.g	scaffold_4:14385102-14389073	30.602	11.9001	-1.36266
Ciclev10012667m.g	scaffold_6:24990725-24991982	31.1174	12.1041	-1.36222
-	scaffold_5:25188306-25547508	10.4477	4.06407	-1.36219
Ciclev10010058m.g	scaffold_1:7007441-7008006	1.11871	0.435333	-1.36164
Ciclev10032149m.g	scaffold_4:3258854-3262212	32.4141	12.6195	-1.36096
Ciclev10004874m.g	scaffold_9:4935788-4938423	1.94529	0.757404	-1.36085
Ciclev10032372m.g	scaffold_4:2392026-2396558	2.04116	0.795159	-1.36007
Ciclev10014266m.g	scaffold_2:31431080-31437122	9.22208	3.59536	-1.35895
Ciclev10014231m.g	scaffold_2:28845910-28867192	2.34487	0.914578	-1.35833
Ciclev10029923m.g	scaffold_8:13485857-13486664	16.4945	6.43558	-1.35785
Ciclev10012053m.g	scaffold_6:23296731-23297981	1.52447	0.594809	-1.35781
Ciclev10012093m.g	scaffold_6:25072091-25074076	1.36268	0.531725	-1.3577
Ciclev10031983m.g	scaffold_4:2776988-2781436	54.2784	21.1829	-1.35748
Ciclev10031082m.g	scaffold_4:1940592-1944128	6.97456	2.72205	-1.35741
Ciclev10002100m.g	scaffold_5:32903812-32907034	50.2249	19.6535	-1.35362
Ciclev10030486m.g	scaffold_4:13477039-13482717	1.05514	0.413137	-1.35274
Ciclev10023951m.g	scaffold_3:49237730-49239023	1.15568	0.452577	-1.35251
Ciclev10019509m.g	scaffold_3:28037825-28040140	3.88847	1.52402	-1.35132
Ciclev10026982m.g	scaffold_7:16939639-16973173	7.77623	3.04809	-1.35116
Ciclev10020504m.g	scaffold_3:46981627-46984276	454.292	178.29	-1.34939
Ciclev10006674m.g	scaffold_9:1297964-1298553	1.12517	0.441823	-1.3486
Ciclev10014607m.g	scaffold_2:35126755-35130519	29.5666	11.6125	-1.34828
Ciclev10026004m.g	scaffold_7:5546986-5550325	112.56	44.2853	-1.3458
Ciclev10026064m.g	scaffold_7:3967395-3969310	13.4443	5.29079	-1.34543
Ciclev10022936m.g,Ciclev10023160m.g	scaffold_3:6631931-6639335	82.4113	32.4353	-1.34528
Ciclev10019989m.g	scaffold_3:38041462-38044515	4.69243	1.84716	-1.34503
Ciclev10015162m.g	scaffold_2:35422597-35425980	223.682	88.1041	-1.34417
Ciclev10007516m.g	scaffold_1:25441527-25445434	17.5767	6.92571	-1.34363
Ciclev10017392m.g	scaffold_2:13388729-13389316	16.9569	6.68738	-1.34236
Ciclev10001389m.g	scaffold_5:40818912-40822881	144.545	57.0257	-1.34183
Ciclev10008287m.g	scaffold_1:7848670-7851354	4.46343	1.76144	-1.34139
Ciclev10002755m.g	scaffold_5:34488984-34490090	0.355574	0.140345	-1.34117
-	scaffold_9:29446114-29446847	32.2483	12.7309	-1.34089
Ciclev10011034m.g	scaffold_6:17744578-17747968	87.7953	34.6805	-1.34002
Ciclev10023872m.g	scaffold_3:44620868-44623952	2.99674	1.18524	-1.33822
-	scaffold_3:18429131-18429278	22709.8	8982.95	-1.33806
Ciclev10032822m.g	scaffold_4:2772549-2773801	14.9203	5.90505	-1.33726
Ciclev10023990m.g,Ciclev10024314m.g	scaffold_3:30345272-30357916	48.8507	19.3342	-1.33722
Ciclev10000876m.g	scaffold_5:40154568-40160983	22.479	8.91434	-1.33438
Ciclev10013873m.g	scaffold_6:21665844-21666600	0.568757	0.225622	-1.3339
Ciclev10012687m.g	scaffold_6:16780133-16781815	0.389798	0.154653	-1.33369
Ciclev10003097m.g	scaffold_5:32546088-32865063	14.629	5.80748	-1.33285
Ciclev10014620m.g	scaffold_2:33446561-33451465	14.361	5.70293	-1.33238
-	scaffold_4:14602416-14602882	52.3607	20.8246	-1.3302
Ciclev10015454m.g	scaffold_2:5172293-5175519	3.27426	1.30237	-1.33003

Ciclev10013342m.g	scaffold_6:24180385-24180971	1.01208	0.402575	-1.33
Ciclev10017742m.g	scaffold_2:24504361-24507645	0.414095	0.164834	-1.32895
Ciclev10014197m.g	scaffold_2:7048186-7051168	5.80159	2.30939	-1.32894
Ciclev10027044m.g	scaffold_7:14243032-14244426	1.4165	0.563858	-1.32893
Ciclev10004353m.g	scaffold_9:25430450-25437071	1.62005	0.644936	-1.32881
Ciclev10029813m.g	scaffold_8:22457312-22460427	24.8505	9.91526	-1.32556
Ciclev10033036m.g	scaffold_4:11749785-11752531	39.9305	15.9468	-1.32422
Ciclev10021502m.g	scaffold_3:1771667-1774647	181.201	72.3663	-1.3242
Ciclev10021138m.g	scaffold_3:7751030-7755076	22.264	8.89174	-1.32418
Ciclev10030627m.g	scaffold_4:14034228-14040846	9.60722	3.83935	-1.32326
Ciclev10022194m.g	scaffold_3:49560909-49561959	1.63951	0.655873	-1.32178
Ciclev10004867m.g	scaffold_9:29829158-29830992	29.3222	11.7367	-1.32097
Ciclev10019894m.g	scaffold_3:2035202-2041798	32.7881	13.1309	-1.32021
Ciclev10033713m.g	scaffold_4:3104604-3105354	1.85423	0.742594	-1.32017
Ciclev10014733m.g	scaffold_2:8431292-8440408	456.579	183.089	-1.31832
Ciclev10023304m.g	scaffold_3:1952048-1952545	1.4454	0.579906	-1.31758
Ciclev10014149m.g	scaffold_2:8410447-8415480	3.06548	1.23062	-1.31673
Ciclev10030950m.g	scaffold_4:24212229-24217804	8.6388	3.46924	-1.31621
Ciclev10026107m.g	scaffold_7:3674155-3676384	193.07	77.5863	-1.31525
Ciclev10003775m.g	scaffold_5:42095626-42097285	9.39383	3.77768	-1.31421
Ciclev10020219m.g	scaffold_3:36286796-36289645	14.6831	5.90825	-1.31336
Ciclev10026820m.g	scaffold_7:18975821-18981398	61.806	24.8725	-1.31319
Ciclev10018971m.g	scaffold_3:555968-559888	41.6042	16.7436	-1.31312
Ciclev10011415m.g	scaffold_6:24233895-24235536	4.28992	1.72661	-1.31301
Ciclev10027936m.g	scaffold_8:1005809-1009132	1.79077	0.720762	-1.31298
Ciclev10032856m.g,Ciclev10033560m.g	scaffold_4:23345084-23349566	7.99496	3.21881	-1.31256
Ciclev10025367m.g	scaffold_7:4406271-4410156	5.36213	2.15894	-1.31248
Ciclev10005441m.g	scaffold_9:28284142-28312014	28.5768	11.5126	-1.31163
Ciclev10023965m.g	scaffold_3:39127971-39130179	5.89551	2.37513	-1.31161
Ciclev10002103m.g	scaffold_5:1332522-1335656	5.23346	2.10875	-1.31138
Ciclev10003235m.g,Ciclev10003673m.g,Ciclev10003722m.g,Ciclev10003903m.g	scaffold_5:31333200-31391484	15.1612	6.11015	-1.3111
Ciclev10010749m.g	scaffold_1:13865678-13873172	40.5463	16.3509	-1.3102
Ciclev10023495m.g	scaffold_3:5674874-5678132	1.92616	0.776849	-1.31002
Ciclev10030621m.g	scaffold_4:7425521-7429658	34.882	14.072	-1.30966
Ciclev10016752m.g	scaffold_2:32529419-32532287	15.7181	6.34416	-1.30892
Ciclev10019786m.g	scaffold_3:47157493-47161633	17.762	7.17308	-1.30813
Ciclev10017873m.g	scaffold_2:30512452-30515315	59.4223	24.0259	-1.30641
Ciclev10023799m.g	scaffold_3:44702969-44705592	23.2268	9.41348	-1.30299
Ciclev10001207m.g	scaffold_5:16649434-16653004	6.83664	2.7731	-1.30179
Ciclev10011051m.g	scaffold_6:19979951-19983345	3.21834	1.3056	-1.30161
Ciclev10017927m.g	scaffold_2:9557694-9560141	0.741992	0.301066	-1.30132
Ciclev10025088m.g	scaffold_7:4710776-4716941	172.649	70.07	-1.30098
-	scaffold_2:32202526-32202888	18.1084	7.35559	-1.29975
Ciclev10031368m.g	scaffold_4:308083-310275	86.1475	35.0014	-1.2994
Ciclev10012553m.g	scaffold_6:5109625-5111097	582.847	237.934	-1.29256
Ciclev10030163m.g	scaffold_8:831694-832216	3.73506	1.52483	-1.29248
-	scaffold_5:2607796-2610283	11.1701	4.56088	-1.29226
Ciclev10001231m.g	scaffold_5:42595067-42600153	8.88937	3.63205	-1.2913
Ciclev10028400m.g	scaffold_8:22039940-22042383	3.06982	1.2547	-1.29081
Ciclev10024940m.g	scaffold_7:6398676-6401404	0.62937	0.25725	-1.29074
Ciclev10020371m.g	scaffold_3:18969849-18972432	6.43784	2.63182	-1.29052
Ciclev10018612m.g	scaffold_3:21260283-21264863	11.3262	4.63174	-1.29003
Ciclev10032478m.g	scaffold_4:10986501-10988823	34.9678	14.3044	-1.28957

Ciclev10026860m.g	scaffold_7:9370617-9373563	38.4215	15.7172	-1.28957
Ciclev10012202m.g	scaffold_6:17971824-17973787	7.42438	3.03731	-1.28948
Ciclev10016236m.g	scaffold_2:32507259-32509012	7.82264	3.20085	-1.2892
Ciclev10010435m.g	scaffold_1:17597537-17599607	20.2723	8.29726	-1.28881
Ciclev10000808m.g	scaffold_5:29385813-29387910	13.3265	5.45458	-1.28876
Ciclev10012705m.g,Ciclev10013687m.g	scaffold_6:17239196-17285298	134.585	55.0899	-1.28866
Ciclev10021702m.g	scaffold_3:9046601-9047756	2.00267	0.819879	-1.28844
-	scaffold_5:3968178-3969881	4.05831	1.66187	-1.28807
Ciclev10028397m.g	scaffold_8:21639623-21642175	3.22789	1.32218	-1.28767
Ciclev10028891m.g	scaffold_8:18152117-18155413	15.3658	6.29486	-1.28748
Ciclev10029285m.g	scaffold_8:5894756-5896346	0.326112	0.133681	-1.28658
-	scaffold_5:28099256-28136390	123.404	50.6298	-1.28534
Ciclev10033127m.g	scaffold_4:23117904-23118901	3.64951	1.49882	-1.28387
-	scaffold_6:128097-130159	9.84619	4.04786	-1.28241
Ciclev10017797m.g	scaffold_2:4538989-4539919	5.84648	2.40511	-1.28146
Ciclev10027341m.g	scaffold_7:8900338-8902756	0.658342	0.270842	-1.28138
Ciclev10030810m.g	scaffold_4:1219998-1223875	9.54897	3.92929	-1.28108
Ciclev10005151m.g	scaffold_9:27270413-27276140	34.4128	14.1613	-1.28099
Ciclev10010750m.g	scaffold_1:20482906-20483685	1.01132	0.416434	-1.28008
Ciclev10004145m.g,Ciclev10004149m.g	scaffold_9:29891230-29928223	12.0465	4.96233	-1.27952
Ciclev10031523m.g	scaffold_4:201555-204527	1.18087	0.487111	-1.27752
Ciclev10008448m.g	scaffold_1:16657240-16660189	58.7801	24.2536	-1.27713
Ciclev10023758m.g	scaffold_3:50780245-50780941	13.0424	5.38167	-1.27708
Ciclev10017022m.g	scaffold_2:33700448-33702556	17.9444	7.40655	-1.27666
Ciclev10012620m.g	scaffold_6:16563164-16563910	4.42815	1.8283	-1.2762
-	scaffold_5:1503259-1988798	10.6411	4.39754	-1.27487
Ciclev10000854m.g	scaffold_5:38085441-38087688	3.07656	1.27217	-1.27403
Ciclev10003001m.g	scaffold_5:33268943-33270204	2.01107	0.8322	-1.27296
Ciclev10010994m.g	scaffold_6:17638531-17643264	3.80588	1.57554	-1.27238
Ciclev10023722m.g	scaffold_3:36363009-36553824	7.38808	3.0594	-1.27195
Ciclev10015376m.g	scaffold_2:28195184-28198839	4.0788	1.68941	-1.27163
Ciclev10001646m.g	scaffold_5:36504700-36506386	0.885607	0.366833	-1.27154
Ciclev10026882m.g	scaffold_7:8184600-8185225	9.36505	3.88031	-1.27111
Ciclev10005661m.g	scaffold_9:10966420-10967873	2.65396	1.10001	-1.27062
Ciclev10007511m.g,Ciclev10007765m.g	scaffold_1:28138396-28149541	9.56936	3.9666	-1.27052
Ciclev10020653m.g	scaffold_3:13560845-13564434	11.3696	4.71563	-1.26966
Ciclev10026467m.g	scaffold_7:5438638-5439587	8.32929	3.45907	-1.26781
Ciclev10019410m.g	scaffold_3:5991280-5995312	11.7158	4.86666	-1.26745
Ciclev10012748m.g	scaffold_6:17947972-17948902	9.97759	4.14481	-1.26739
Ciclev10016552m.g	scaffold_2:11739858-11742644	20.7091	8.60406	-1.26717
-	scaffold_2:25403702-25404213	14.7823	6.14202	-1.26708
Ciclev10011915m.g	scaffold_6:23372891-23374463	4.25059	1.76637	-1.26688
Ciclev10017436m.g	scaffold_2:31211055-31225439	80.6162	33.5166	-1.26619
Ciclev10014058m.g	scaffold_2:34736069-34742644	4.47765	1.86168	-1.26613
Ciclev10013896m.g	scaffold_6:16854942-16857003	3.39858	1.41329	-1.26587
Ciclev10028993m.g	scaffold_8:2241690-2245635	14.8109	6.16805	-1.26377
Ciclev10014658m.g	scaffold_2:29506073-29516708	18.0683	7.53872	-1.26107
Ciclev10028665m.g	scaffold_8:23383309-23386750	13.6205	5.68312	-1.26103
Ciclev10008355m.g	scaffold_1:27914229-27916509	312.589	130.478	-1.26046
Ciclev10016071m.g	scaffold_2:3460369-3462174	11.2693	4.70532	-1.26003
Ciclev10008742m.g	scaffold_1:2784789-2787063	978.093	408.618	-1.25922
Ciclev10011912m.g	scaffold_6:497151-499116	124.461	51.9986	-1.25914
Ciclev10026244m.g	scaffold_7:13916806-14069972	3.03979	1.27022	-1.2589

Ciclev10013661m.g	scaffold_6:15603247-15618784	204.961	85.6846	-1.25824
Ciclev10009879m.g	scaffold_1:17188024-17189271	22.1771	9.27298	-1.25797
Ciclev10017736m.g	scaffold_2:13379667-13380066	11.4592	4.79144	-1.25797
Ciclev10029655m.g	scaffold_8:1987727-1990725	280.161	117.154	-1.25785
Ciclev10005819m.g	scaffold_9:176954-178079	16.1045	6.73494	-1.25772
Ciclev10032875m.g	scaffold_4:25168530-25170468	5.38351	2.25337	-1.25646
Ciclev10015234m.g	scaffold_2:7725023-7755971	73.0062	30.564	-1.25619
Ciclev10010352m.g	scaffold_1:24154498-24154849	2.2133	0.926861	-1.25577
Ciclev10019935m.g	scaffold_3:7835501-7838656	2.24814	0.941658	-1.25546
Ciclev10012845m.g	scaffold_6:22805831-22806783	5.72489	2.39895	-1.25485
Ciclev10009830m.g	scaffold_1:25211314-25212784	77.0533	32.2924	-1.25466
Ciclev10021651m.g	scaffold_3:6602993-6605239	13.516	5.67496	-1.25198
Ciclev10004572m.g	scaffold_9:836229-842228	47.5344	19.9594	-1.2519
Ciclev10010307m.g	scaffold_1:5413232-5416822	16.6166	6.97967	-1.25139
Ciclev10002693m.g	scaffold_5:37891704-37892899	124.64	52.3689	-1.25098
-	scaffold_5:653503-653828	17.1068	7.19124	-1.25026
Ciclev10022123m.g	scaffold_3:36817012-36818205	191.207	80.4589	-1.24881
Ciclev10001416m.g	scaffold_5:39875927-39877965	4.03581	1.69845	-1.24864
Ciclev10007575m.g	scaffold_1:5388449-5396900	17.4746	7.35505	-1.24846
Ciclev10016924m.g	scaffold_2:34204472-34205506	86.8158	36.5777	-1.24699
Ciclev10020276m.g	scaffold_3:42109588-42112025	32.3786	13.6474	-1.24642
Ciclev10026035m.g	scaffold_7:6230493-6232544	13.7642	5.80417	-1.24575
Ciclev10022517m.g	scaffold_3:42316866-42320830	12.2507	5.16714	-1.24542
Ciclev10006155m.g	scaffold_9:29952004-29956306	201.332	84.9566	-1.24478
Ciclev10025939m.g	scaffold_7:17255495-17259980	15.9222	6.72117	-1.24425
Ciclev10014726m.g	scaffold_2:31094174-31095934	4.69669	1.98358	-1.24354
Ciclev10020629m.g	scaffold_3:18619210-18623445	6.96025	2.94014	-1.24325
Ciclev10003202m.g	scaffold_5:22625617-22628186	33.7202	14.2566	-1.24199
Ciclev10030204m.g	scaffold_8:13177010-13178480	1.09844	0.464441	-1.24188
Ciclev10002876m.g,Ciclev10002900m.g,Ciclev10002906m.g	scaffold_5:432292-517176	76.228	32.2385	-1.24154
Ciclev10026646m.g	scaffold_7:3826028-3830771	4.47672	1.89451	-1.24061
Ciclev10017916m.g	scaffold_2:35210722-35210959	33.3938	14.1322	-1.2406
Ciclev10010884m.g	scaffold_497:5490-8108	5.43197	2.29902	-1.24046
Ciclev10021941m.g	scaffold_3:2106809-2109266	35.2978	14.9435	-1.24006
Ciclev10000544m.g	scaffold_5:20952024-20954797	0.593907	0.251585	-1.23919
Ciclev10008225m.g	scaffold_1:1070372-1072218	2.91181	1.23363	-1.23901
Ciclev10017711m.g	scaffold_2:5335434-5337882	2.95166	1.25135	-1.23804
Ciclev10000913m.g	scaffold_5:36638720-36641298	7.58209	3.21717	-1.2368
Ciclev10004707m.g	scaffold_9:25094081-25099067	45.0451	19.1217	-1.23616
Ciclev10014532m.g	scaffold_2:34232452-34235596	11.3795	4.83135	-1.23593
Ciclev10004108m.g	scaffold_271:11454-13254	2.33387	0.991029	-1.23572
Ciclev10025420m.g	scaffold_7:3732302-3735157	294.936	125.332	-1.23464
Ciclev10012291m.g	scaffold_6:12023784-12025466	377.396	160.462	-1.23385
Ciclev10018607m.g	scaffold_3:10050622-10055433	40.2333	17.1068	-1.23382
Ciclev10017241m.g	scaffold_2:9720272-9721084	2.83863	1.2071	-1.23365
Ciclev10031152m.g	scaffold_4:6273404-6278204	0.548542	0.233269	-1.23361
Ciclev10013434m.g	scaffold_6:9032386-9034306	1.86302	0.79258	-1.23302
Ciclev10013559m.g	scaffold_6:16888386-16892179	13.9029	5.92207	-1.23121
Ciclev10030037m.g,Ciclev10030156m.g,Ciclev10030238m.g	scaffold_8:20620078-20723794	26.1626	11.1495	-1.23052
Ciclev10019040m.g	scaffold_3:48775889-48781106	71.3459	30.4128	-1.23015
Ciclev10026151m.g	scaffold_7:14282417-14286582	32.3419	13.787	-1.2301
Ciclev10023412m.g	scaffold_3:27206698-27210418	73.0312	31.1375	-1.22986

Ciclev10025539m.g	scaffold_7:3578162-3583653	0.605577	0.258422	-1.22858
Ciclev10028475m.g	scaffold_8:18160844-18166421	16.5301	7.05531	-1.22832
-	scaffold_5:641276-642007	10.2994	4.39743	-1.22783
Ciclev10027564m.g	scaffold_7:5743041-5743449	31.1606	13.3091	-1.22732
Ciclev10032710m.g	scaffold_4:24472219-24474178	34.6059	14.7807	-1.2273
Ciclev10011584m.g	scaffold_6:23534437-23537172	6.64951	2.84021	-1.22725
Ciclev10032628m.g	scaffold_4:19790478-19792588	8.65424	3.69879	-1.22635
Ciclev10006399m.g	scaffold_9:556724-557601	47.2059	20.1767	-1.22628
-	scaffold_1095:2518-4078	20.4529	8.74809	-1.22527
Ciclev10012019m.g	scaffold_6:21443412-21444863	9.78094	4.18479	-1.22482
Ciclev10029506m.g	scaffold_8:23995447-23996072	30.2404	12.9444	-1.22414
-	scaffold_2:29445574-29448189	27.8014	11.9028	-1.22385
Ciclev10020221m.g	scaffold_3:4625770-4630292	26.5556	11.3699	-1.2238
Ciclev10015330m.g,Ciclev10018105m.g,Ciclev10018331m.g	scaffold_2:28845910-28867192	7.82868	3.35296	-1.22334
Ciclev10030821m.g	scaffold_4:6290058-6294163	40.5679	17.3787	-1.22302
Ciclev10031023m.g	scaffold_4:22074798-22078747	12.6575	5.43439	-1.2198
Ciclev10021094m.g	scaffold_3:37993755-37999062	80.2687	34.495	-1.21845
Ciclev10009544m.g	scaffold_1:28521510-28524566	105.615	45.4137	-1.21761
Ciclev10003759m.g	scaffold_5:24926169-25131690	10.7095	4.60844	-1.21654
Ciclev10031415m.g	scaffold_4:18800789-18805604	75.958	32.6917	-1.21628
Ciclev10023404m.g	scaffold_3:48463394-48465325	5.64506	2.42971	-1.2162
Ciclev10024601m.g	scaffold_3:28055808-28059361	114.122	49.1452	-1.21545
Ciclev10015129m.g	scaffold_2:9567678-9570576	59.5308	25.6432	-1.21506
Ciclev10029888m.g	scaffold_8:4482515-4485560	1.42922	0.615719	-1.21489
Ciclev10011973m.g	scaffold_6:13666332-13669690	86.626	37.3453	-1.21387
Ciclev10020979m.g	scaffold_3:2697354-2700326	0.929077	0.400715	-1.21322
Ciclev10006654m.g	scaffold_9:28427766-28431251	278.019	119.918	-1.21313
Ciclev10011218m.g	scaffold_6:14486904-14489292	112.779	48.6491	-1.21301
Ciclev10019801m.g	scaffold_3:39100221-39102696	34.8122	15.0197	-1.21274
Ciclev10026444m.g	scaffold_7:9233534-9235297	9.26253	3.99687	-1.21254
Ciclev10028037m.g	scaffold_8:24920509-24922833	56.8053	24.5334	-1.21128
Ciclev10023483m.g	scaffold_3:46720125-46721864	1.69461	0.731923	-1.21119
Ciclev10017826m.g	scaffold_2:21754131-21754443	2.76259	1.19366	-1.21063
Ciclev10025349m.g	scaffold_7:16030857-16033528	8.4523	3.65297	-1.21027
-	scaffold_3:27951136-27951973	18.2397	7.884	-1.21009
Ciclev10030611m.g	scaffold_4:2484696-2491144	18.8862	8.1636	-1.21006
Ciclev10010857m.g	scaffold_1:28303018-28323314	9.52753	4.1195	-1.20963
Ciclev10017624m.g	scaffold_2:4025762-4026947	0.807186	0.349483	-1.20768
Ciclev10031592m.g	scaffold_4:12834102-12835666	1.06851	0.46268	-1.20751
Ciclev10021190m.g	scaffold_3:41286225-41287833	2.02476	0.877199	-1.20677
Ciclev10025168m.g	scaffold_7:18763895-18800038	28.9713	12.5534	-1.20654
Ciclev10020783m.g	scaffold_3:1199308-1203079	2.52306	1.09342	-1.20633
Ciclev10030736m.g	scaffold_4:21909660-21916503	13.4075	5.81064	-1.20628
Ciclev10023916m.g	scaffold_3:838028-839032	2.82678	1.22517	-1.20618
Ciclev10007453m.g	scaffold_1:19341935-19345148	1.64935	0.714998	-1.20589
Ciclev10019743m.g	scaffold_3:48625263-48629072	57.991	25.1588	-1.20477
Ciclev10026688m.g	scaffold_7:185146-185751	35.2688	15.331	-1.20194
Ciclev10026017m.g	scaffold_7:8647365-8650863	30.9088	13.447	-1.20074
Ciclev10012375m.g,Ciclev10012379m.g,Ciclev10012633m.g	scaffold_6:10374765-10420975	37.0878	16.1404	-1.20027
-	scaffold_9:22498654-22499062	35.989	15.6627	-1.20023
Ciclev10011479m.g	scaffold_6:24177353-24178998	0.723696	0.315186	-1.19918

Ciclev10000277m.g,Ciclev10003685m.g	scaffold_5:31469286-31635063	2.81215	1.22555	-1.19824
Ciclev10023449m.g	scaffold_3:3217382-3219499	3.5001	1.52663	-1.19705
Ciclev10016504m.g	scaffold_2:31256159-31258762	2.61423	1.14049	-1.19673
Ciclev10003627m.g	scaffold_5:2683792-2684772	117.125	51.1403	-1.19552
Ciclev10030938m.g	scaffold_4:22097089-22102186	17.391	7.59408	-1.1954
Ciclev10015378m.g	scaffold_2:31954701-31957411	91.8857	40.1427	-1.1947
Ciclev10011237m.g	scaffold_6:6694889-6751336	10.3106	4.50761	-1.1937
Ciclev10016785m.g	scaffold_2:16553370-16554188	2.90097	1.26841	-1.19351
Ciclev10032490m.g	scaffold_4:20514641-20520202	25.7459	11.2596	-1.19319
Ciclev10004238m.g	scaffold_9:18367739-18371241	10.2477	4.48235	-1.19297
Ciclev10000760m.g	scaffold_5:42409836-42412693	1.43299	0.626937	-1.19263
Ciclev10024179m.g	scaffold_3:21296577-21473212	0.711637	0.311437	-1.1922
Ciclev10031051m.g	scaffold_4:1869993-1873860	42.1904	18.4642	-1.19218
Ciclev10024165m.g	scaffold_3:7658092-7661444	0.760302	0.33285	-1.1917
Ciclev10015623m.g	scaffold_2:2945400-2947403	18.9793	8.31037	-1.19144
Ciclev10007668m.g	scaffold_1:3407664-3413915	178.56	78.2196	-1.19081
-	scaffold_9:9764147-9766447	41.6877	18.2623	-1.19075
Ciclev10012740m.g	scaffold_6:13630037-13632023	1.89566	0.830841	-1.19005
Ciclev10027024m.g	scaffold_7:5319072-5319801	1.32859	0.582738	-1.18897
Ciclev10018358m.g	scaffold_2:33685356-33688185	1.69292	0.74282	-1.18843
Ciclev10028804m.g	scaffold_8:21369372-21370728	1.35547	0.595075	-1.18765
Ciclev10010955m.g	scaffold_6:25511406-25520662	1.97497	0.86724	-1.18732
Ciclev10019679m.g	scaffold_3:45304615-45309598	0.806675	0.354259	-1.18718
Ciclev10006122m.g	scaffold_9:15770091-15772562	15.6909	6.89745	-1.1858
Ciclev10018997m.g	scaffold_3:46325341-46329222	2.43016	1.0686	-1.18533
Ciclev10020552m.g	scaffold_3:31620762-31715908	45606.1	20056.5	-1.18516
Ciclev10004993m.g	scaffold_9:2957538-2959079	0.875838	0.385188	-1.1851
Ciclev10005800m.g	scaffold_9:29948086-29950884	34.0059	14.9557	-1.18509
-	scaffold_3:9362365-9362932	9.98803	4.39306	-1.18497
Ciclev10025455m.g,Ciclev10026995m.g	scaffold_7:5359516-5374200	36.4421	16.0307	-1.18477
Ciclev10017032m.g	scaffold_2:24652426-24654825	121.921	53.6643	-1.18391
Ciclev10008248m.g	scaffold_1:24450867-24452705	10.3698	4.56823	-1.18268
Ciclev10032686m.g	scaffold_4:17365401-17366484	3101.24	1366.36	-1.1825
-	scaffold_5:2703902-2704504	5.77898	2.54909	-1.18083
Ciclev10014994m.g	scaffold_2:27428714-27432877	3.65217	1.6117	-1.18017
Ciclev10007201m.g	scaffold_9:29432438-29439510	14.8462	6.55276	-1.17992
Ciclev10021739m.g	scaffold_3:520015-521240	2.27572	1.00501	-1.17911
Ciclev10014584m.g	scaffold_2:27935054-27938445	1.36806	0.604463	-1.17841
-	scaffold_3:27206698-27210418	476.595	210.687	-1.17766
Ciclev10016111m.g	scaffold_2:32948414-32957830	10.2735	4.54378	-1.17696
Ciclev10020503m.g	scaffold_3:45271739-45275296	0.733822	0.324634	-1.17661
Ciclev10028398m.g	scaffold_8:16691160-16692948	1.1736	0.519311	-1.17627
Ciclev10006002m.g	scaffold_9:4524400-4525971	19.9587	8.83725	-1.17534
Ciclev10030800m.g	scaffold_4:17259275-17264391	2.85931	1.26669	-1.1746
Ciclev10013229m.g	scaffold_6:22529561-22529879	2.14655	0.951449	-1.17382
Ciclev10004041m.g	scaffold_5:42911507-42912104	160.1	71.0121	-1.17283
Ciclev10007651m.g	scaffold_1:7114557-7118282	2.14849	0.953072	-1.17266
Ciclev10026750m.g	scaffold_7:10736369-10737126	2.41164	1.07001	-1.1724
Ciclev10001885m.g	scaffold_5:41987451-41990041	61.876	27.4558	-1.17227
Ciclev10029614m.g	scaffold_8:20292559-20294016	6.40724	2.84407	-1.17175
Ciclev10032373m.g	scaffold_4:21329512-21333529	3.55926	1.58001	-1.17164
Ciclev10007969m.g	scaffold_1:18896163-18901623	33.6863	14.9573	-1.17131
Ciclev10018031m.g	scaffold_2:3313126-3315167	3.30437	1.46721	-1.1713
Ciclev10018599m.g	scaffold_3:3372882-3377278	8.1631	3.62527	-1.17103

Ciclev10033310m.g	scaffold_4:19001055-19040863	12.7809	5.68515	-1.16872
Ciclev10015231m.g	scaffold_2:34270986-34272863	59.1084	26.3019	-1.1682
Ciclev10008624m.g	scaffold_1:1245556-1247064	14.6235	6.51703	-1.166
Ciclev10032882m.g	scaffold_4:25065382-25066587	268.645	119.749	-1.16569
Ciclev10006505m.g,Ciclev10006802m.g	scaffold_9:15639000-15642935	15.8468	7.06741	-1.16493
Ciclev10022804m.g	scaffold_3:47974459-47975237	35.7093	15.93	-1.16456
Ciclev10021149m.g	scaffold_3:1925788-1928534	6.60385	2.95022	-1.16249
Ciclev10018627m.g	scaffold_3:22582145-22586403	4.13884	1.84978	-1.16187
Ciclev10018373m.g	scaffold_2:27274971-27275698	165.567	74.0422	-1.16099
Ciclev10010766m.g	scaffold_1:403275-404286	4.87501	2.18097	-1.16044
Ciclev10029949m.g	scaffold_8:16565605-16565893	2.90273	1.29902	-1.15999
Ciclev10031089m.g	scaffold_4:19471732-19475606	5.35586	2.39687	-1.15997
Ciclev10026582m.g	scaffold_7:14773830-14775350	17.8581	7.99334	-1.15971
Ciclev10017876m.g	scaffold_2:36005532-36007204	1.02281	0.457817	-1.1597
Ciclev10021726m.g	scaffold_3:42166129-42169227	35.237	15.7788	-1.1591
Ciclev10019538m.g	scaffold_3:44307102-44311569	1.87837	0.841211	-1.15894
Ciclev10025116m.g	scaffold_7:7258155-7260647	31.6227	14.1658	-1.15855
Ciclev10008768m.g	scaffold_1:6358029-6359568	6.04004	2.70836	-1.15714
Ciclev10003762m.g	scaffold_5:25188306-25547508	0.634127	0.284402	-1.15684
Ciclev10005026m.g	scaffold_9:1313424-1319164	63.148	28.3248	-1.15667
Ciclev10008435m.g	scaffold_1:5194084-5198860	60.8262	27.2949	-1.15606
Ciclev10024989m.g	scaffold_7:7149220-7153371	82.3083	36.9367	-1.15598
Ciclev10014810m.g	scaffold_2:31153656-31155460	1.64055	0.736365	-1.15569
Ciclev10028367m.g	scaffold_8:20273416-20274830	1.59419	0.715579	-1.15564
Ciclev10011730m.g	scaffold_6:17901617-17906439	62.2336	27.9482	-1.15494
Ciclev10024766m.g	scaffold_7:17630699-17638750	25.6186	11.509	-1.15443
Ciclev10017914m.g,Ciclev10018343m.g	scaffold_2:503858-521421	59.2418	26.624	-1.15389
Ciclev10005566m.g	scaffold_9:26654934-26658646	29.6532	13.3315	-1.15335
Ciclev10028140m.g	scaffold_8:539940-542765	3.95556	1.77838	-1.15331
Ciclev10017343m.g	scaffold_2:29110316-29112979	9.8615	4.43631	-1.15245
Ciclev10004249m.g	scaffold_9:29130638-29136024	53.3176	23.9901	-1.15217
Ciclev10004022m.g	scaffold_5:25188306-25547508	0.634263	0.285468	-1.15175
Ciclev10001705m.g	scaffold_5:29646970-29649511	26.1188	11.7632	-1.1508
-	scaffold_5:18197252-18202010	9.44507	4.25436	-1.15062
Ciclev10026265m.g	scaffold_7:6290648-6292417	8.11063	3.65349	-1.15054
Ciclev10031968m.g	scaffold_4:15477116-15478843	120.503	54.2931	-1.15023
Ciclev10009724m.g	scaffold_1:24248880-24251393	37.2328	16.7773	-1.15007
Ciclev10009236m.g	scaffold_1:3862912-3866849	11.835	5.33335	-1.14994
Ciclev10017875m.g	scaffold_2:30325131-30365011	4.45351	2.00745	-1.14958
Ciclev10016567m.g	scaffold_2:5958601-5961642	1.62651	0.733222	-1.14946
Ciclev10001864m.g	scaffold_5:40835332-40838299	11.2875	5.08947	-1.14914
Ciclev10025475m.g	scaffold_7:8845919-8853010	33.8451	15.2613	-1.14907
Ciclev10017555m.g	scaffold_2:16815559-16819481	4.79232	2.16165	-1.14859
Ciclev10002729m.g	scaffold_5:1341617-1342638	11.4037	5.14386	-1.14858
Ciclev10003039m.g	scaffold_5:19275096-19276356	34.8202	15.7131	-1.14795
Ciclev10025415m.g	scaffold_7:20472101-20474601	40.6974	18.3693	-1.14764
Ciclev10006604m.g	scaffold_9:26557905-26560040	1.49574	0.675217	-1.14744
Ciclev10031073m.g	scaffold_4:23404931-23425248	67.5018	30.4779	-1.14716
Ciclev10021090m.g	scaffold_3:29481041-29482628	4.42564	1.99865	-1.14686
Ciclev10019326m.g	scaffold_3:47830376-47832891	5.26669	2.37935	-1.14633
Ciclev10024972m.g	scaffold_7:5094560-5100082	8.32569	3.76154	-1.14625
Ciclev10025401m.g	scaffold_7:3560793-3565200	26.1132	11.7995	-1.14605
Ciclev10031049m.g	scaffold_4:1515155-1517052	1.28635	0.581967	-1.14428
Ciclev10023610m.g	scaffold_3:42906090-42915510	0.738992	0.334371	-1.14411

Ciclev10006137m.g	scaffold_9:20360608-20494992	1.0704	0.484466	-1.14368
Ciclev10031244m.g	scaffold_4:14688023-14692200	29.3883	13.3039	-1.14339
Ciclev10027962m.g	scaffold_8:8671548-8673629	2.25907	1.0227	-1.14334
Ciclev10010040m.g	scaffold_1:4861411-4866144	1.22852	0.556272	-1.14306
Ciclev10011143m.g	scaffold_6:19569459-19577780	12.0895	5.47538	-1.14273
Ciclev10032920m.g	scaffold_4:14404153-14405075	57.3023	25.963	-1.14214
Ciclev10031645m.g	scaffold_4:23659237-23662888	43.6064	19.7633	-1.14171
Ciclev10007694m.g	scaffold_1:5100429-5104636	10.2661	4.65489	-1.14107
Ciclev10011774m.g	scaffold_6:24375408-24378405	11.8597	5.37905	-1.14065
Ciclev10029281m.g	scaffold_8:2227826-2230752	253.615	115.093	-1.13984
Ciclev10028762m.g	scaffold_8:3486731-3491627	8.01146	3.63921	-1.13844
Ciclev10014437m.g	scaffold_2:6861955-6865446	13.4662	6.11792	-1.13824
Ciclev10032214m.g	scaffold_4:216216-222771	48.3208	21.9534	-1.1382
Ciclev10026671m.g	scaffold_7:3478859-3481757	285.587	129.834	-1.13726
Ciclev10027309m.g	scaffold_7:13343315-13343936	4.97499	2.26249	-1.13678
Ciclev10017588m.g	scaffold_2:30523899-30548559	6.11916	2.7836	-1.13638
Ciclev10001975m.g	scaffold_5:31903631-31906548	16.5846	7.55703	-1.13396
Ciclev10032807m.g	scaffold_4:22597164-22601079	265.059	120.891	-1.1326
Ciclev10018741m.g	scaffold_3:1387596-1394177	14.5235	6.6256	-1.13226
Ciclev10005529m.g	scaffold_9:21107893-21109983	77.2424	35.2465	-1.13191
Ciclev10016428m.g	scaffold_2:33991109-33993524	5.25117	2.39677	-1.13155
-	scaffold_4:509246-509579	1009.74	460.907	-1.13144
Ciclev10028301m.g	scaffold_8:6681475-6683886	0.745984	0.341196	-1.12854
Ciclev10012991m.g	scaffold_6:17347523-17349224	35.8776	16.4099	-1.12851
Ciclev10027574m.g	scaffold_7:1359859-1363252	0.343317	0.157041	-1.1284
Ciclev10011771m.g	scaffold_6:17438269-17440122	25.9218	11.8574	-1.12838
Ciclev10016165m.g	scaffold_2:15738335-15741219	146.878	67.187	-1.12836
Ciclev10016218m.g	scaffold_2:19918573-19923588	20.1676	9.2273	-1.12806
Ciclev10021060m.g	scaffold_3:793626-796219	676.79	309.656	-1.12804
Ciclev10029272m.g	scaffold_8:1689940-1692902	191.298	87.5354	-1.12788
Ciclev10020826m.g,Ciclev10024045m.g	scaffold_3:35372249-35712237	6.90442	3.16111	-1.12709
Ciclev10014764m.g	scaffold_2:24641958-24644161	34.9447	16.0086	-1.12623
Ciclev10012397m.g	scaffold_6:7799873-7806415	87.3342	40.0579	-1.12446
Ciclev10023703m.g	scaffold_3:27274446-27274841	55.6837	25.5519	-1.12382
Ciclev10029259m.g	scaffold_8:23087004-23091069	29.8788	13.717	-1.12316
Ciclev10000532m.g	scaffold_5:34533360-34540306	32.6193	14.9924	-1.12149
Ciclev10029428m.g	scaffold_8:24044995-24049924	68.902	31.6794	-1.121
Ciclev10014943m.g	scaffold_2:33206461-33209078	7.32153	3.3676	-1.12042
Ciclev10004507m.g	scaffold_9:566505-571956	33.9108	15.6192	-1.11842
Ciclev10031790m.g	scaffold_4:16068388-16075095	3.72589	1.71686	-1.11781
Ciclev10007993m.g	scaffold_1:24986847-24990060	23.187	10.686	-1.11759
Ciclev10007469m.g	scaffold_1:12798697-12801839	1.35703	0.625834	-1.1166
Ciclev10033572m.g	scaffold_4:2013663-2017905	51.967	23.9804	-1.11574
Ciclev10029499m.g	scaffold_8:19546540-19549348	25.4818	11.7636	-1.11513
Ciclev10009871m.g	scaffold_1:8013468-8015328	35.3439	16.3239	-1.11447
Ciclev10023246m.g	scaffold_3:43267424-43272903	55.4048	25.6053	-1.11357
Ciclev10000464m.g	scaffold_5:5001604-5005111	17.0555	7.88308	-1.11341
Ciclev10030953m.g	scaffold_4:25053029-25057026	1.22263	0.565133	-1.11332
Ciclev10020214m.g	scaffold_3:41627949-41634431	21.2405	9.81816	-1.11329
Ciclev10009512m.g	scaffold_1:25721038-25722484	34.6231	16.0052	-1.1132
Ciclev10005807m.g	scaffold_9:28023166-28026244	22.8562	10.5717	-1.11239
Ciclev10029262m.g	scaffold_8:24742619-24743741	10.8661	5.03111	-1.11088
Ciclev10016840m.g	scaffold_2:28156068-28158670	21.2643	9.84924	-1.11035
Ciclev10007670m.g	scaffold_1:6420803-6425294	23.433	10.8544	-1.11027
Ciclev10008112m.g	scaffold_1:28220636-28224080	23.7375	10.9962	-1.11016

Ciclev10004257m.g	scaffold_9:28912262-28917003	6.17423	2.86149	-1.1095
Ciclev10031856m.g	scaffold_4:1215359-1218773	30.3574	14.07	-1.10942
Ciclev10022863m.g	scaffold_3:46841461-46841986	10.3277	4.78714	-1.10929
Ciclev10025374m.g	scaffold_7:593833-598812	31.5666	14.6372	-1.10876
Ciclev10032302m.g	scaffold_4:6100707-6103243	275.906	127.945	-1.10866
Ciclev10003909m.g	scaffold_5:41136321-41137442	4.71447	2.18632	-1.1086
-	scaffold_8:3655720-3656777	12.4387	5.76883	-1.10849
Ciclev10018693m.g	scaffold_3:9893235-9898231	6.52762	3.02762	-1.10837
Ciclev10005130m.g	scaffold_9:11951187-11955254	6.10064	2.8298	-1.10826
Ciclev10022127m.g	scaffold_3:39819129-39823025	37.1793	17.2507	-1.10784
Ciclev10021364m.g	scaffold_3:6299686-6303749	34.1399	15.8659	-1.10553
Ciclev10025611m.g	scaffold_7:17491173-17497183	90.7988	42.2093	-1.10511
Ciclev10029295m.g	scaffold_8:1589388-1592716	6.01508	2.79642	-1.105
-	scaffold_2:20451196-20451722	30.0316	13.9619	-1.10498
Ciclev10029306m.g	scaffold_8:3245556-3247054	9.19897	4.27825	-1.10445
Ciclev10014440m.g	scaffold_2:35429102-35435228	14.8327	6.89875	-1.10437
Ciclev10009068m.g	scaffold_1:28492007-28494909	4.01855	1.8699	-1.10371
Ciclev10031290m.g	scaffold_4:15917251-15921592	9.36048	4.35565	-1.10369
Ciclev10015215m.g	scaffold_2:23163672-23168238	44.1279	20.5395	-1.10329
Ciclev10003191m.g	scaffold_5:33705152-33709918	1.50097	0.698694	-1.10316
Ciclev10021815m.g	scaffold_3:48517015-48518527	5.41363	2.52056	-1.10285
Ciclev10001024m.g	scaffold_5:17606041-17618431	21.9102	10.2052	-1.10229
Ciclev10008109m.g	scaffold_1:940513-942972	11.8094	5.50138	-1.10207
Ciclev10004356m.g	scaffold_9:25619086-25625135	6.4022	2.98384	-1.1014
Ciclev10018391m.g	scaffold_2:843361-853044	2.68294	1.25081	-1.10095
Ciclev10015828m.g	scaffold_2:31872091-31877015	14.7551	6.88031	-1.10067
Ciclev10015131m.g	scaffold_2:35401066-35405149	2.96362	1.38226	-1.10033
Ciclev10007227m.g	scaffold_1:23487199-23497316	0.18215	0.0849572	-1.10032
Ciclev10029378m.g	scaffold_8:19298370-19299813	94.0524	43.8844	-1.09976
Ciclev10026390m.g	scaffold_7:11307058-11308330	2.83099	1.32122	-1.09943
Ciclev10017208m.g	scaffold_2:13044722-13048774	16.1959	7.5598	-1.09921
Ciclev10007964m.g	scaffold_1:24942841-24945912	92.188	43.0722	-1.09782
Ciclev10012548m.g	scaffold_6:11100646-11103340	288.845	135.028	-1.09704
Ciclev10005752m.g	scaffold_9:2768141-2829028	2.47741	1.15851	-1.09656
Ciclev10020497m.g	scaffold_3:40066214-40070707	89.3388	41.8	-1.09579
Ciclev10028516m.g	scaffold_8:23168353-23172621	54.0099	25.2987	-1.09416
Ciclev10000508m.g	scaffold_5:41372512-41377106	4.82906	2.26283	-1.09362
Ciclev10032065m.g	scaffold_4:17842211-17846211	0.818493	0.383547	-1.09357
Ciclev10007470m.g	scaffold_1:26802786-26810465	14.4082	6.75373	-1.09313
Ciclev10024055m.g	scaffold_3:18980102-18981479	5.15226	2.41529	-1.09301
Ciclev10006948m.g	scaffold_9:20248357-20251630	66.2817	31.0773	-1.09275
Ciclev10012482m.g,Ciclev10012795m.g	scaffold_6:25413891-25421461	21.3215	9.99913	-1.09243
Ciclev10014177m.g	scaffold_2:33156191-33161018	4.30709	2.02097	-1.09167
Ciclev10001591m.g	scaffold_5:40526247-40529184	150.934	70.8246	-1.0916
Ciclev10004478m.g	scaffold_9:27848858-27851831	1.0685	0.501728	-1.09061
Ciclev10028906m.g	scaffold_8:975146-976388	2.10715	0.989614	-1.09036
Ciclev10019408m.g	scaffold_3:41857383-41859305	8.53969	4.01085	-1.09028
Ciclev10020410m.g	scaffold_3:5967420-5971786	2.44876	1.15026	-1.09009
Ciclev10000515m.g	scaffold_5:43170340-43172802	55.4142	26.0309	-1.09003
Ciclev10029025m.g,Ciclev10029347m.g,Ciclev10030340m.g	scaffold_8:18863193-18917480	41.5539	19.525	-1.08966
Ciclev10018707m.g	scaffold_3:24026649-24169357	0.492536	0.231488	-1.08929
Ciclev10024786m.g	scaffold_7:3488264-3498732	2.35952	1.10896	-1.08929
Ciclev10025446m.g	scaffold_7:2117362-2121600	24.4453	11.4954	-1.08849

Ciclev10031522m.g	scaffold_4:1976174-1977527	1.04831	0.493255	-1.08765
Ciclev10007269m.g	scaffold_1:7679273-7685541	0.560995	0.264085	-1.08699
Ciclev10028097m.g	scaffold_8:7552931-7556106	0.397402	0.187107	-1.08673
Ciclev10029184m.g,Ciclev10029366m.g	scaffold_8:2782317-2789467	27.491	12.9498	-1.08603
Ciclev10008275m.g	scaffold_1:165671-167810	2.51476	1.18466	-1.08595
Ciclev10018829m.g	scaffold_3:2387800-2392607	34.0506	16.0493	-1.08517
Ciclev10001766m.g	scaffold_5:27492965-27539324	1.02479	0.483276	-1.08441
Ciclev10026307m.g,Ciclev10027442m.g	scaffold_7:2721693-2817739	55.3769	26.1185	-1.08421
Ciclev10018341m.g	scaffold_2:25351240-25353303	18.9491	8.93744	-1.08419
Ciclev10018939m.g	scaffold_3:4165513-4171242	49.9666	23.5758	-1.08365
Ciclev10016298m.g	scaffold_2:32754954-32757337	87.4723	41.2795	-1.0834
Ciclev10015243m.g	scaffold_2:11654897-11658922	13.943	6.58231	-1.08287
Ciclev10011141m.g	scaffold_6:14266025-14269088	52.8771	24.9632	-1.08284
Ciclev10026114m.g	scaffold_7:1333010-1335044	1.56635	0.739478	-1.08283
Ciclev10017082m.g	scaffold_2:31801438-31804455	1.53293	0.723772	-1.08268
Ciclev10030874m.g	scaffold_4:14003070-14010665	0.531528	0.250995	-1.08249
Ciclev10024049m.g	scaffold_3:7123159-7123759	2.10082	0.992548	-1.08175
Ciclev10019142m.g	scaffold_3:24339985-24345431	1.51441	0.71557	-1.0816
Ciclev10001235m.g	scaffold_5:42675741-42682366	1.20462	0.569582	-1.08061
-	scaffold_9:26183811-26184153	166.893	78.9153	-1.08055
Ciclev10003083m.g	scaffold_5:42941241-42942396	124.785	59.0066	-1.0805
Ciclev10018671m.g	scaffold_3:47892245-47897508	13.9501	6.59743	-1.0803
Ciclev10005629m.g	scaffold_9:5544553-5546110	19.3878	9.17268	-1.07973
Ciclev10010708m.g	scaffold_1:5492915-5494405	18.1164	8.57199	-1.0796
Ciclev10025081m.g	scaffold_7:7280314-7285822	32.689	15.4673	-1.07959
Ciclev10002801m.g	scaffold_5:42963149-42967105	19.3284	9.1459	-1.07953
Ciclev10019943m.g	scaffold_3:49398912-49401345	0.491686	0.232724	-1.07912
Ciclev10006202m.g	scaffold_9:14748935-14749813	17.5575	8.31069	-1.07905
-	scaffold_1095:4129-4693	64.6411	30.6058	-1.07865
Ciclev10029033m.g	scaffold_8:23018434-23019836	12.1801	5.76714	-1.0786
Ciclev10004491m.g	scaffold_9:30490330-30500959	36.2631	17.1741	-1.07827
Ciclev10022286m.g	scaffold_3:49145486-49149088	120.095	56.9115	-1.07739
Ciclev10027474m.g	scaffold_7:921137-921932	22.4218	10.635	-1.07609
Ciclev10004298m.g	scaffold_9:4494462-4503620	28.474	13.5108	-1.07553
Ciclev10029669m.g	scaffold_8:5123744-5125235	2.17854	1.03423	-1.07481
Ciclev10000463m.g	scaffold_5:36317431-36322995	43.3934	20.6036	-1.07458
Ciclev10014233m.g	scaffold_2:8583245-8588715	63.0833	29.9754	-1.07348
Ciclev10024188m.g	scaffold_3:7135108-7136388	3.19196	1.51737	-1.07287
Ciclev10033347m.g	scaffold_4:3762484-3766722	2.14913	1.02181	-1.07263
Ciclev10002560m.g	scaffold_5:37676182-37679875	3.69299	1.75622	-1.07231
Ciclev10025618m.g	scaffold_7:13810685-13814703	9.53865	4.53848	-1.07158
Ciclev10013506m.g	scaffold_6:8742663-8744139	0.876592	0.417389	-1.07051
Ciclev10002438m.g	scaffold_5:39986259-39987337	4.41192	2.1009	-1.0704
Ciclev10011684m.g	scaffold_6:10816139-10936716	245.794	117.145	-1.06916
Ciclev10006396m.g	scaffold_9:30590131-30590466	13.834	6.59336	-1.06913
Ciclev10031223m.g	scaffold_4:24353421-24355495	0.644663	0.307374	-1.06855
Ciclev10006096m.g	scaffold_9:1285174-1286468	905.266	431.797	-1.06799
-	scaffold_3:35372249-35712237	33.1985	15.8428	-1.06729
Ciclev10010582m.g	scaffold_1:23184868-23193259	10.0226	4.78839	-1.06565
Ciclev10020608m.g	scaffold_3:31620762-31715908	18.3815	8.78246	-1.06556
Ciclev10029937m.g	scaffold_8:19315361-19315979	2.67288	1.27711	-1.06552
Ciclev10000533m.g	scaffold_5:39289639-39299263	3.83356	1.83178	-1.06544
Ciclev10022899m.g	scaffold_3:7277472-7278283	12.9075	6.16902	-1.0651
Ciclev10033959m.g	scaffold_4:6816588-6817092	1.56883	0.749943	-1.06484

Ciclev10033593m.g	scaffold_4:16066332-16068145	0.66457	0.317734	-1.0646
Ciclev10032524m.g	scaffold_4:2153717-2155194	101.281	48.4457	-1.06392
Ciclev10021950m.g	scaffold_3:43093260-43096086	3.42642	1.63914	-1.06376
Ciclev10029482m.g	scaffold_8:17992652-17993246	7.85502	3.75795	-1.06367
Ciclev10020528m.g	scaffold_3:44525265-44527119	1.21301	0.580398	-1.06347
Ciclev10019184m.g	scaffold_3:680834-684433	254.085	121.656	-1.06251
Ciclev10025900m.g	scaffold_7:681058-685008	7.17391	3.43501	-1.06245
Ciclev10025130m.g	scaffold_7:10035354-10116411	4.13821	1.9822	-1.06191
Ciclev10004053m.g	scaffold_5:18100083-18137609	3.51162	1.68253	-1.0615
Ciclev10018948m.g	scaffold_3:16208637-16228612	29.7254	14.2472	-1.06102
Ciclev10021613m.g	scaffold_3:49207143-49209171	7.91239	3.7928	-1.06085
Ciclev10021492m.g	scaffold_3:7248626-7253007	44.6691	21.4208	-1.06026
Ciclev10014386m.g	scaffold_2:32605276-32609032	6.0265	2.89092	-1.05979
Ciclev10016137m.g	scaffold_2:32971924-32975786	4.61506	2.21462	-1.05929
Ciclev10007736m.g	scaffold_1:14601222-14605899	1.30393	0.625895	-1.05888
Ciclev10016277m.g	scaffold_2:13310843-13314055	14.8164	7.11304	-1.05866
Ciclev10011445m.g	scaffold_6:12670836-12673970	50.1257	24.0642	-1.05866
Ciclev10024288m.g,Ciclev10024291m.g	scaffold_3:50855159-50859573	181.309	87.0661	-1.05827
Ciclev10011910m.g	scaffold_6:22136738-22139832	32.4819	15.6016	-1.05794
Ciclev10010570m.g	scaffold_1:8681248-8686058	28.1902	13.5413	-1.05782
Ciclev10017839m.g	scaffold_2:24737092-24926440	8.108	3.89529	-1.05762
Ciclev10021788m.g	scaffold_3:46263053-46265579	3.57955	1.72098	-1.05655
Ciclev10025935m.g	scaffold_7:2390009-2391658	187.334	90.0971	-1.05606
Ciclev10020159m.g	scaffold_3:6353032-6356406	11.7686	5.66166	-1.05565
Ciclev10016918m.g,Ciclev10017041m.g	scaffold_2:27393144-27417691	452.936	217.909	-1.05558
Ciclev10033438m.g	scaffold_4:19171922-19173038	0.69231	0.333072	-1.05558
Ciclev10001047m.g	scaffold_5:26935348-26947618	71.7365	34.5186	-1.05533
Ciclev10000554m.g	scaffold_5:40196231-40199730	0.255968	0.123177	-1.05523
Ciclev10006640m.g	scaffold_9:2029335-2030346	0.802015	0.386002	-1.05502
Ciclev10013514m.g	scaffold_6:20142308-20143274	1.74053	0.837941	-1.0546
Ciclev10019450m.g	scaffold_3:6471378-6473755	5.55469	2.67439	-1.05449
Ciclev10014108m.g	scaffold_2:9232125-9242195	22.0825	10.6331	-1.05434
Ciclev10027640m.g	scaffold_1723:182-3359	1.91904	0.924101	-1.05426
Ciclev10026849m.g	scaffold_7:15353294-15355555	59.8617	28.8283	-1.05415
Ciclev10021198m.g	scaffold_3:3675198-3677594	31.6639	15.2604	-1.05304
Ciclev10021059m.g	scaffold_3:46054709-46056408	18.5653	8.95102	-1.05249
Ciclev10020327m.g	scaffold_3:18965761-18968313	61.4549	29.6307	-1.05243
Ciclev10011654m.g	scaffold_6:17735050-17737623	5.80918	2.80391	-1.05089
Ciclev10027468m.g	scaffold_7:16878927-16882179	18.3578	8.86098	-1.05085
Ciclev10002010m.g	scaffold_5:35827903-35830008	91.7453	44.2892	-1.05068
Ciclev10025480m.g	scaffold_7:5377222-5381234	44.1203	21.2987	-1.05068
Ciclev10025448m.g	scaffold_7:6436784-6440864	10.8194	5.22707	-1.04954
Ciclev10008257m.g	scaffold_1:25462099-25463684	1.03956	0.50226	-1.04947
Ciclev10022616m.g	scaffold_3:9984084-9985782	161.647	78.1207	-1.04907
Ciclev10029235m.g	scaffold_8:21817948-21821252	64.9814	31.4104	-1.04879
Ciclev10020872m.g	scaffold_3:31535236-31540844	4.22636	2.04301	-1.04872
Ciclev10021569m.g	scaffold_3:5420052-5424166	122.699	59.337	-1.04811
Ciclev10018850m.g,Ciclev10024514m.g	scaffold_3:41311463-41317333	6.35379	3.07285	-1.04804
Ciclev10001694m.g	scaffold_5:6464855-6470466	36.436	17.6225	-1.04795
-	scaffold_5:38071964-38073010	83.5176	40.3943	-1.04793
-	scaffold_5:2902744-2905294	28.052	13.568	-1.04789
Ciclev10011683m.g	scaffold_6:5895318-5897382	0.377458	0.182636	-1.04735
Ciclev10017402m.g	scaffold_2:13390080-13390603	7.69576	3.72378	-1.0473

-	scaffold_2:28637240-28637663	10.9618	5.30435	-1.04724
Ciclev10015086m.g	scaffold_2:23021192-23023457	6.5084	3.14968	-1.0471
Ciclev10013140m.g	scaffold_6:7782778-7784020	6.93548	3.35648	-1.04705
Ciclev10020024m.g	scaffold_3:43227841-43234655	21.584	10.4472	-1.04684
Ciclev10008892m.g	scaffold_1:23365318-23368311	29.8928	14.4704	-1.0467
Ciclev10018566m.g	scaffold_3:9015706-9022288	57.1995	27.6918	-1.04654
Ciclev10008567m.g	scaffold_1:26892991-26898736	57.8355	28.0085	-1.04609
Ciclev10031956m.g	scaffold_4:17499129-17500420	8.49675	4.11491	-1.04605
Ciclev10004922m.g	scaffold_9:29440187-29442935	83.3723	40.3781	-1.04599
Ciclev10012512m.g	scaffold_6:20039906-20042958	3.52022	1.7058	-1.04522
Ciclev10007524m.g	scaffold_1:25987802-25992258	33.4264	16.2093	-1.04417
Ciclev10009804m.g	scaffold_1:5413232-5416822	12.9642	6.28843	-1.04376
Ciclev10026517m.g	scaffold_7:3093668-3095978	42.7044	20.7183	-1.04348
Ciclev10032565m.g	scaffold_4:21517371-21520194	8.37956	4.06762	-1.04269
Ciclev10005186m.g	scaffold_9:1556090-1558575	27.7872	13.4915	-1.04238
Ciclev10031679m.g	scaffold_4:702796-704974	37.3225	18.1242	-1.04213
Ciclev10001383m.g	scaffold_5:32546088-32865063	12.7401	6.18685	-1.0421
Ciclev10019975m.g	scaffold_3:33683416-33687458	1.80284	0.875638	-1.04187
Ciclev10017361m.g	scaffold_2:19185435-19186538	78.9026	38.3302	-1.04159
Ciclev10005111m.g	scaffold_9:27259812-27263097	23.6477	11.4903	-1.04128
Ciclev10004816m.g	scaffold_9:25313692-25319614	0.770316	0.374519	-1.04041
Ciclev10012456m.g	scaffold_6:17225616-17227093	1507.46	732.922	-1.04039
Ciclev10012685m.g	scaffold_6:11126657-11131076	15.5058	7.54394	-1.03942
Ciclev10022119m.g	scaffold_3:49740453-49745002	62.6254	30.4707	-1.03932
Ciclev10027439m.g	scaffold_7:4873675-4875066	2.50208	1.21748	-1.03923
Ciclev10008412m.g	scaffold_1:16431589-16436163	0.668699	0.325412	-1.03909
Ciclev10031364m.g	scaffold_4:19553769-19555593	2.44491	1.19046	-1.03826
Ciclev10015037m.g	scaffold_2:20031904-20042011	17.7632	8.65125	-1.03791
Ciclev10020878m.g	scaffold_3:541574-543121	17.8962	8.72178	-1.03696
Ciclev10011631m.g	scaffold_6:17335733-17338606	0.422107	0.205787	-1.03646
Ciclev10027612m.g	scaffold_7:8282443-8283871	0.635207	0.309946	-1.03521
Ciclev10026481m.g	scaffold_7:7118758-7119761	658.074	321.164	-1.03494
Ciclev10007366m.g	scaffold_1:28710366-28713767	1.72529	0.842102	-1.03478
Ciclev10020959m.g	scaffold_3:11191458-11193858	0.477313	0.233018	-1.03449
Ciclev10000302m.g	scaffold_5:34276104-34281941	1.3345	0.651551	-1.03435
Ciclev10031760m.g	scaffold_4:1528602-1531114	4.33221	2.11596	-1.03379
Ciclev10026510m.g	scaffold_7:2461705-2462587	17.6344	8.61471	-1.03352
Ciclev10017219m.g	scaffold_2:17347948-17351501	1.61541	0.789529	-1.03283
Ciclev10015026m.g	scaffold_2:29870501-29873518	2.09013	1.0216	-1.03276
-	scaffold_2:22351561-22352958	22.1832	10.8465	-1.03223
Ciclev10000060m.g	scaffold_5:27441741-27448756	8.46266	4.13941	-1.03169
Ciclev10021041m.g	scaffold_3:632881-635612	3.79776	1.85792	-1.03146
Ciclev10024885m.g	scaffold_7:1729021-1732466	42.8756	20.9755	-1.03145
Ciclev10019109m.g	scaffold_3:3823695-3826499	0.880154	0.430628	-1.03132
Ciclev10014823m.g	scaffold_2:35323614-35328102	25.8034	12.6253	-1.03124
Ciclev10000542m.g	scaffold_5:35350023-35354136	0.2832	0.138581	-1.03109
Ciclev10019174m.g	scaffold_3:48026617-48031503	1.86206	0.911565	-1.03049
Ciclev10011601m.g	scaffold_6:8958448-8986420	11.8229	5.78789	-1.03048
Ciclev10017693m.g	scaffold_2:9968925-9971998	0.241823	0.118404	-1.03024
Ciclev10000311m.g	scaffold_5:38036284-38041507	7.04684	3.45157	-1.02972
Ciclev10019131m.g	scaffold_3:767883-772407	36.4522	17.8906	-1.0268
Ciclev10024511m.g	scaffold_3:22043158-22176987	0.7607	0.373401	-1.0266
Ciclev10014768m.g	scaffold_2:16833386-16839435	10.326	5.06961	-1.02633
Ciclev10005215m.g	scaffold_9:187478-190288	106.245	52.1825	-1.02576
Ciclev10011768m.g	scaffold_6:17051740-17053964	4.12952	2.02825	-1.02574
Ciclev10015930m.g	scaffold_2:8991164-8994650	10.7202	5.26778	-1.02506

Ciclev10021315m.g	scaffold_3:41497498-41503110	40.3671	19.8417	-1.02465
Ciclev10005127m.g	scaffold_9:3024636-3026071	190.207	93.5329	-1.02403
Ciclev10021425m.g	scaffold_3:37597623-37600961	189.954	93.4147	-1.02393
Ciclev10005208m.g,Ciclev10005211m.g	scaffold_9:5227254-5269794	88.5329	43.5688	-1.02292
Ciclev10022857m.g,Ciclev10024365m.g	scaffold_3:31467767-31474442	28.2681	13.9134	-1.0227
Ciclev10000155m.g	scaffold_5:35039707-35043069	18.3596	9.0388	-1.02233
Ciclev10014824m.g	scaffold_2:34866212-34872606	11.2809	5.55396	-1.02229
Ciclev10010591m.g	scaffold_1:24935568-24937990	0.719528	0.354387	-1.02172
Ciclev10021982m.g	scaffold_3:44556802-44560092	11.8036	5.81912	-1.02035
Ciclev10032860m.g	scaffold_4:3098508-3099415	3.12507	1.54134	-1.0197
Ciclev10021060m.g	scaffold_3:793626-796219	350.235	172.787	-1.01933
Ciclev10015754m.g	scaffold_2:31316929-31322323	13.5502	6.69204	-1.01779
Ciclev10008918m.g	scaffold_1:21664720-21665886	0.758177	0.374637	-1.01704
Ciclev10010409m.g	scaffold_1:22175575-22263277	8.31326	4.10852	-1.01679
Ciclev10001166m.g	scaffold_5:42314433-42319785	18.9891	9.38645	-1.01652
Ciclev10015582m.g	scaffold_2:4441284-4443386	4.05597	2.0054	-1.01615
Ciclev10012951m.g	scaffold_6:14178289-14181908	19.722	9.75145	-1.01612
Ciclev10020864m.g	scaffold_3:35789565-35793592	31.2452	15.4493	-1.01609
Ciclev10011596m.g	scaffold_6:15932015-15936176	44.2332	21.8725	-1.01601
Ciclev10028305m.g,Ciclev10030328m.g	scaffold_8:2790429-2799293	146.946	72.6747	-1.01576
Ciclev10012426m.g	scaffold_6:16842977-16844008	1.34842	0.666947	-1.01562
Ciclev10021441m.g	scaffold_3:6437053-6441117	30.9437	15.3138	-1.01481
Ciclev10018911m.g	scaffold_3:43987024-43990983	4.66314	2.30802	-1.01465
Ciclev10033070m.g	scaffold_4:1114644-1117928	84.9266	42.0456	-1.01426
Ciclev10000352m.g	scaffold_5:36588686-36592786	15.5535	7.70236	-1.01387
Ciclev10033553m.g	scaffold_4:14957124-14958803	3.71348	1.84033	-1.01281
Ciclev10015676m.g	scaffold_2:8720763-8725932	76.885	38.105	-1.01272
Ciclev10031472m.g	scaffold_4:20856340-20862677	11.8185	5.85811	-1.01254
Ciclev10033063m.g	scaffold_4:25192542-25194288	23.3984	11.6005	-1.01223
Ciclev10000940m.g	scaffold_5:37180883-37182577	0.358096	0.177644	-1.01135
Ciclev10030478m.g	scaffold_4:15783866-15805068	4.33496	2.1507	-1.01121
Ciclev10012313m.g	scaffold_6:21720468-21723489	25.6442	12.7254	-1.01092
Ciclev10014175m.g	scaffold_2:21993084-21998728	15.6978	7.79283	-1.01034
Ciclev10022085m.g	scaffold_3:4914762-4919442	9.79532	4.86387	-1.00999
Ciclev10033908m.g	scaffold_4:15121961-15266095	0.578862	0.287481	-1.00976
Ciclev10003220m.g,Ciclev10003734m.g	scaffold_5:39329804-39334594	39.46	19.598	-1.00969
Ciclev10018927m.g	scaffold_3:41750448-41756882	47.9696	23.8251	-1.00964
Ciclev10013886m.g	scaffold_6:14515616-14517074	2.75322	1.36756	-1.00952
Ciclev10029234m.g	scaffold_8:23371733-23373676	1.84614	0.917298	-1.00905
Ciclev10011987m.g	scaffold_6:18953631-18957474	13.5325	6.7247	-1.00888
Ciclev10030978m.g	scaffold_4:1517400-1521860	5.99846	2.98239	-1.00812
Ciclev10010063m.g	scaffold_1:5003828-5004439	9.32946	4.6405	-1.00751
Ciclev10029126m.g	scaffold_8:2639801-2641261	13.1112	6.52157	-1.00751
-	scaffold_3:11687448-12154789	16.5333	8.22456	-1.00737
Ciclev10005826m.g	scaffold_9:13687699-13690439	5.6351	2.8045	-1.0067
-	scaffold_7:11685370-11685936	15.2849	7.60904	-1.00632
-	scaffold_4:7421905-7422285	109.637	54.6072	-1.00557
Ciclev10000610m.g,Ciclev10000618m.g	scaffold_5:37701505-37709444	24.213	12.0621	-1.0053
Ciclev10004502m.g	scaffold_9:29392884-29395387	46.0924	22.9658	-1.00504
Ciclev10031200m.g	scaffold_4:16210719-16218022	66.1643	32.9675	-1.00501
Ciclev10005609m.g	scaffold_9:8509471-8558807	71.1995	35.5114	-1.00358

Ciclev10022920m.g	scaffold_3:39483386-39486219	7.49877	3.74135	-1.0031
Ciclev10016599m.g	scaffold_2:35497039-35504104	6.06171	3.02717	-1.00175
Ciclev10015132m.g	scaffold_2:17353079-17356820	8.05872	4.0287	-1.00024
Ciclev10003912m.g	scaffold_5:22562652-22570573	4.75228	2.37597	-1.00011
Ciclev10012880m.g	scaffold_6:22229222-22231270	17.0215	8.51917	-0.998571
Ciclev10028412m.g	scaffold_8:24406838-24409538	14.8428	7.43024	-0.998281
Ciclev10012751m.g	scaffold_6:5286319-5290832	10.9248	5.47328	-0.997132
Ciclev10022564m.g	scaffold_3:47387247-47388927	1.11249	0.557521	-0.996699
Ciclev10009645m.g	scaffold_1:27540014-27543499	27.7315	13.8995	-0.996491
Ciclev10002263m.g	scaffold_5:9979514-9982669	27.4565	13.7633	-0.99632
Ciclev10029486m.g	scaffold_8:22319749-22322555	32.7648	16.432	-0.995639
Ciclev10020314m.g	scaffold_3:48450782-48455853	29.1064	14.6048	-0.994894
Ciclev10005087m.g	scaffold_9:25662048-25666791	28.4438	14.2733	-0.994797
Ciclev10001888m.g	scaffold_5:36449345-36452178	85.1794	42.7696	-0.993919
-	scaffold_9:4715824-4717692	8.5625	4.3023	-0.992924
Ciclev10009045m.g	scaffold_1:3665140-3672863	50.0102	25.1314	-0.992731
Ciclev10032656m.g	scaffold_4:6937471-6940171	23.3468	11.7355	-0.992348
Ciclev10022553m.g	scaffold_3:2110307-2113166	36.4473	18.334	-0.991292
Ciclev10032093m.g	scaffold_4:23150166-23151971	35.5433	17.8874	-0.990638
Ciclev10005866m.g	scaffold_9:22996539-22997969	6.44296	3.24466	-0.989657
Ciclev10016994m.g	scaffold_2:32504790-32505737	9.80007	4.93532	-0.989649
Ciclev10010826m.g	scaffold_1:28510741-28513573	34.686	17.4697	-0.989498
Ciclev10032341m.g	scaffold_4:16418137-16422763	26.6799	13.4418	-0.989024
Ciclev10032222m.g	scaffold_4:1586789-1589604	29.0573	14.6479	-0.988205
Ciclev10030313m.g	scaffold_8:2770499-2771191	1.13879	0.574417	-0.987336
Ciclev10008315m.g	scaffold_1:28623623-28626008	144.994	73.1596	-0.986876
Ciclev10015887m.g,Ciclev10016063m.g	scaffold_2:10424778-10472483	28.8323	14.5577	-0.985905
Ciclev10021570m.g	scaffold_3:41610562-41616227	39.1084	19.7467	-0.985869
Ciclev10008263m.g	scaffold_1:1406639-1410403	20.545	10.3759	-0.985544
Ciclev10015483m.g	scaffold_2:35212690-35214865	23.6354	11.9407	-0.985062
Ciclev10026457m.g	scaffold_7:5173845-5179864	6.11056	3.09119	-0.983144
Ciclev10002683m.g	scaffold_5:41972942-41973902	561.785	284.383	-0.982182
Ciclev10003992m.g	scaffold_5:20211283-20217483	0.509942	0.258165	-0.982036
Ciclev10030023m.g	scaffold_8:2673134-2674232	23.2515	11.7737	-0.981761
Ciclev10018842m.g	scaffold_3:2900626-2904422	0.533596	0.270274	-0.981326
Ciclev10000353m.g	scaffold_5:30767754-30771272	0.849133	0.430145	-0.981169
Ciclev10031490m.g	scaffold_4:21716795-21718205	13.6616	6.92581	-0.98007
Ciclev10025326m.g	scaffold_7:2839617-2843824	13.1124	6.64782	-0.979978
Ciclev10008902m.g	scaffold_1:2987979-2990176	13.6561	6.9236	-0.979951
Ciclev10018038m.g	scaffold_2:30719028-30725506	0.713606	0.361831	-0.979812
Ciclev10020951m.g	scaffold_3:44353874-44358046	37.225	18.8789	-0.979499
Ciclev10020676m.g	scaffold_3:3852140-3855898	16.4733	8.3559	-0.979265
Ciclev10027064m.g	scaffold_7:12028509-12029412	0.910372	0.461997	-0.978571
Ciclev10004541m.g	scaffold_9:13517151-13519780	31.0301	15.748	-0.978501
Ciclev10015513m.g	scaffold_2:34877897-34879535	7.05807	3.58278	-0.978194
Ciclev10020261m.g	scaffold_3:49428800-49433203	4.24712	2.15606	-0.978086
Ciclev10019081m.g	scaffold_3:3299618-3311799	7.95417	4.0401	-0.977323
Ciclev10020910m.g	scaffold_3:5756043-5758185	5.80542	2.94957	-0.976895
Ciclev10024663m.g	scaffold_3:27092406-27094282	362.602	184.275	-0.976527
Ciclev10022415m.g	scaffold_3:21945866-21947624	39.4832	20.0686	-0.9763
Ciclev10025011m.g	scaffold_7:7977526-7984425	23.158	11.772	-0.976152
Ciclev10014723m.g	scaffold_2:32806564-32812384	6.03837	3.0704	-0.975734
Ciclev10021430m.g	scaffold_3:8186688-8189902	30.1755	15.3667	-0.973567
Ciclev10011124m.g	scaffold_6:24975343-24986759	61.3302	31.2327	-0.973541
Ciclev10013889m.g	scaffold_6:23800860-23803332	0.262153	0.13354	-0.973135

Ciclev10032611m.g	scaffold_4:3063806-3064887	1.93208	0.984197	-0.973132
Ciclev10028136m.g	scaffold_8:22403868-22408174	67.7317	34.5144	-0.972631
Ciclev10032021m.g	scaffold_4:6297563-6301762	84.9735	43.3076	-0.972392
Ciclev10001264m.g	scaffold_5:34784367-34786120	24.098	12.282	-0.97237
Ciclev10020205m.g	scaffold_3:3767474-3771747	78.0355	39.7728	-0.972346
Ciclev10020563m.g	scaffold_3:46248144-46249558	1.89771	0.967405	-0.972065
Ciclev10024513m.g	scaffold_3:45486980-45489883	3.13339	1.59814	-0.971329
Ciclev10029695m.g	scaffold_8:2308971-2309555	2.3482	1.19767	-0.971319
Ciclev10031809m.g	scaffold_4:22390118-22394239	6.59159	3.36312	-0.970826
Ciclev10014589m.g	scaffold_2:35579402-35586357	103.692	52.9074	-0.97076
-	scaffold_7:97880-100646	3.60045	1.83927	-0.969044
Ciclev10031448m.g	scaffold_4:22862807-22865758	4.11631	2.10314	-0.968808
Ciclev10009685m.g	scaffold_1:25955462-25957753	17.5326	8.96218	-0.968122
Ciclev10026666m.g	scaffold_7:7270100-7271553	106.184	54.3088	-0.967316
Ciclev10008771m.g	scaffold_1:7916944-7921026	2.94822	1.50802	-0.967183
Ciclev10027140m.g	scaffold_7:3583793-3584499	1.30912	0.669927	-0.966522
Ciclev10033156m.g	scaffold_4:12415429-12415833	2.54015	1.30064	-0.965695
Ciclev10024525m.g	scaffold_3:22335164-22336541	9.11853	4.67177	-0.96483
Ciclev10001775m.g	scaffold_5:32316721-32320120	52.0285	26.6613	-0.964557
-	scaffold_4:23753780-23753922	15016.8	7695.51	-0.964488
Ciclev10016407m.g	scaffold_2:10569900-10571074	25.0138	12.8271	-0.963529
Ciclev10016529m.g	scaffold_2:33694162-33699245	132.063	67.7407	-0.963126
Ciclev10005604m.g	scaffold_9:29990608-29993190	14.3374	7.35765	-0.962461
Ciclev10004263m.g	scaffold_9:17340139-17344170	5.51828	2.83311	-0.961831
Ciclev10014761m.g	scaffold_2:12897886-12901322	8.94474	4.59309	-0.961576
Ciclev10029478m.g	scaffold_8:23377508-23378382	3.3043	1.69684	-0.961492
Ciclev10019816m.g	scaffold_3:43654586-43656842	49.4869	25.4155	-0.961335
Ciclev10009218m.g	scaffold_1:2155051-2156056	6.07207	3.11936	-0.96094
Ciclev10002167m.g	scaffold_5:34516142-34519012	18.9784	9.75296	-0.960443
Ciclev10001739m.g	scaffold_5:37302526-37305633	25.4694	13.0904	-0.960258
Ciclev10026292m.g	scaffold_7:5747603-5748976	9.22304	4.74425	-0.959062
Ciclev10026875m.g	scaffold_7:4203875-4205830	26.0174	13.388	-0.958538
Ciclev10028766m.g	scaffold_8:10574672-10578797	52.521	27.0445	-0.957558
Ciclev10008761m.g	scaffold_1:25618962-25621811	30.9671	15.9581	-0.956447
Ciclev10018601m.g	scaffold_3:8291226-8297326	2.01221	1.03697	-0.956405
Ciclev10002793m.g	scaffold_5:8435122-8435971	4560.35	2350.23	-0.956343
Ciclev10020574m.g	scaffold_3:20762973-20770394	15.8275	8.16	-0.95579
Ciclev10016702m.g	scaffold_2:32919901-32922181	69.1662	35.6622	-0.955671
Ciclev10012871m.g	scaffold_6:23246512-23247603	33.3591	17.2085	-0.954961
Ciclev10023135m.g	scaffold_3:12271368-12272973	44.8642	23.1455	-0.954832
Ciclev10017852m.g	scaffold_2:9430328-9435446	36.7734	18.9739	-0.954645
Ciclev10023277m.g	scaffold_3:11687448-12154789	7.37323	3.80518	-0.954332
Ciclev10020275m.g	scaffold_3:2096571-2098539	34.7492	17.9432	-0.953543
Ciclev10017362m.g	scaffold_2:10912561-10913785	10.4456	5.39464	-0.953294
Ciclev10032201m.g	scaffold_4:3956723-3959799	39.0476	20.1727	-0.952826
-	scaffold_2:14028885-14029238	22.6424	11.6983	-0.952723
Ciclev10002714m.g	scaffold_5:34219816-34223850	187.797	97.0317	-0.952644
Ciclev10014453m.g	scaffold_2:24524889-24530143	8.40579	4.34487	-0.95207
Ciclev10005709m.g	scaffold_9:29399277-29401620	3.54175	1.83115	-0.951709
Ciclev10002009m.g	scaffold_5:42125534-42127625	55.4345	28.6701	-0.951237
Ciclev10027109m.g,Ciclev10027260m.g	scaffold_7:5823228-5827517	113.748	58.8535	-0.950635
Ciclev10026302m.g	scaffold_7:13717850-13722266	20.2769	10.4974	-0.949806
Ciclev10020438m.g	scaffold_3:41880758-41884653	4.94028	2.55797	-0.949595
Ciclev10002396m.g	scaffold_5:34161720-34162652	41.9642	21.7297	-0.949488
Ciclev10008821m.g	scaffold_1:28782168-28786731	435.158	225.465	-0.948633

Ciclev10014673m.g	scaffold_2:34695247-34699412	81.6923	42.3377	-0.948257
Ciclev10008439m.g	scaffold_1:22443441-22448835	20.3398	10.5481	-0.947327
Ciclev10014630m.g	scaffold_2:11715434-11718336	0.876313	0.454499	-0.947168
Ciclev10022476m.g	scaffold_3:5874123-5878839	1.79169	0.929405	-0.946941
Ciclev10020321m.g	scaffold_3:40900742-40904111	2.18625	1.13412	-0.946886
Ciclev10030903m.g	scaffold_4:18393185-18399566	45.1311	23.4183	-0.946489
Ciclev10021043m.g	scaffold_3:43316045-43317909	8.25505	4.28364	-0.94644
Ciclev10006705m.g	scaffold_9:30429041-30434565	42.2832	21.9451	-0.946183
Ciclev10014351m.g,Ciclev10014355m.g,Ciclev10018131m.g,Ciclev10018333m.g	scaffold_2:24737092-24926440	120.577	62.6035	-0.945638
Ciclev10011599m.g	scaffold_6:8958448-8986420	24.0049	12.4635	-0.945617
Ciclev10021601m.g	scaffold_3:39352984-39354543	5.61544	2.9161	-0.945357
Ciclev10005830m.g	scaffold_9:29931102-29937516	54.0018	28.0558	-0.944709
Ciclev10003645m.g,Ciclev10003790m.g	scaffold_5:329359-335208	79.1608	41.1371	-0.944347
Ciclev10026158m.g	scaffold_7:8921794-8925991	30.1485	15.6673	-0.944325
Ciclev10013913m.g	scaffold_6:21253669-21258519	7.27871	3.78354	-0.943944
Ciclev10003329m.g,Ciclev10003424m.g	scaffold_5:22621836-22625401	34.5826	17.9785	-0.943773
Ciclev10010284m.g	scaffold_1:14828560-14829793	1.4232	0.739923	-0.943688
Ciclev10026009m.g	scaffold_7:8733486-8736636	10.4013	5.40832	-0.943506
-	scaffold_8:14549860-14551486	46.3978	24.1278	-0.943359
Ciclev10001912m.g	scaffold_5:41975363-41977621	11.6026	6.03909	-0.942044
Ciclev10007003m.g	scaffold_9:30980432-30984188	59.8902	31.1758	-0.941892
Ciclev10012388m.g	scaffold_6:25370287-25373154	12.9409	6.73672	-0.941822
Ciclev10016561m.g	scaffold_2:24692918-24694730	0.966621	0.503798	-0.940107
Ciclev10008839m.g	scaffold_1:4851969-4855156	75.0238	39.1046	-0.940008
Ciclev10020506m.g	scaffold_3:1510186-1511798	0.350594	0.182852	-0.93912
Ciclev10009640m.g	scaffold_1:27818757-27819890	35.5467	18.5459	-0.938618
Ciclev10019635m.g	scaffold_3:1131355-1134462	5.32526	2.77838	-0.938605
Ciclev10013128m.g	scaffold_6:22758026-22760875	134.748	70.35	-0.937641
Ciclev10023412m.g	scaffold_3:27206698-27210418	484.539	252.999	-0.937479
Ciclev10015510m.g	scaffold_2:30142886-30145311	1.03052	0.53819	-0.937184
Ciclev10027326m.g	scaffold_7:19093472-19094081	11.4335	5.97135	-0.937141
Ciclev10004254m.g	scaffold_9:26920857-26925316	2.25718	1.17893	-0.937046
Ciclev10028909m.g	scaffold_8:12563101-12564623	81.0326	42.3339	-0.93669
Ciclev10007513m.g	scaffold_1:7218501-7227996	56.391	29.4765	-0.935901
Ciclev10007998m.g	scaffold_1:14766998-14769846	136.766	71.4909	-0.93588
Ciclev10013833m.g	scaffold_6:25170423-25172480	42.8358	22.3916	-0.93586
Ciclev10012944m.g	scaffold_6:7909208-7914618	95.2607	49.807	-0.935532
Ciclev10008166m.g	scaffold_1:22053023-22055471	1.68141	0.87988	-0.934296
Ciclev10012964m.g	scaffold_6:15624796-15625794	1231.21	644.824	-0.933103
Ciclev10031823m.g	scaffold_4:20901475-20903241	21.6693	11.3632	-0.931287
Ciclev10024879m.g	scaffold_7:7999675-8005144	0.536807	0.281556	-0.930982
Ciclev10014537m.g	scaffold_2:691426-698055	5.25292	2.75564	-0.930733
Ciclev10005611m.g	scaffold_9:26901509-26903559	8.61269	4.51893	-0.930482
Ciclev10002525m.g	scaffold_5:35307568-35311024	38.31	20.1064	-0.930069
Ciclev10015466m.g	scaffold_2:9074063-9077904	14.0362	7.36685	-0.930032
Ciclev10015334m.g	scaffold_2:12893460-12896650	8.52879	4.47838	-0.929364
Ciclev10025336m.g	scaffold_7:3571637-3573361	2.50434	1.31517	-0.929181
Ciclev10029659m.g	scaffold_8:17214537-17218258	2.51407	1.32036	-0.929094
Ciclev10019288m.g,Ciclev10024076m.g,Ciclev10024305m.g	scaffold_3:11687448-12154789	25.5615	13.4268	-0.928852
Ciclev10001565m.g	scaffold_5:33766086-33769182	13.5045	7.09752	-0.928059

Ciclev10032916m.g	scaffold_4:2766828-2770332	56.8967	29.9084	-0.927796
Ciclev10014434m.g	scaffold_2:16494775-16499968	19.3668	10.1807	-0.927755
Ciclev10033850m.g	scaffold_4:3086846-3088752	2.14774	1.12936	-0.92732
Ciclev10002923m.g	scaffold_5:38603368-38606770	100.859	53.1473	-0.924274
Ciclev10007139m.g	scaffold_9:26778538-26796341	1.27736	0.67342	-0.923581
Ciclev10015206m.g	scaffold_2:10231920-10233354	2.23497	1.17873	-0.923018
Ciclev10003404m.g	scaffold_5:25832875-25852772	5.40246	2.85037	-0.922464
Ciclev10027951m.g	scaffold_8:2939320-2944264	4.90042	2.58552	-0.922449
Ciclev10009394m.g	scaffold_1:25765316-25766713	89.6806	47.317	-0.922438
Ciclev10030565m.g	scaffold_4:6349486-6358140	6.95342	3.66893	-0.922364
Ciclev10005099m.g	scaffold_9:28446117-28453825	8.07302	4.26008	-0.922227
Ciclev10029657m.g	scaffold_8:24294600-24295449	1010.1	533.213	-0.921711
Ciclev10017396m.g	scaffold_2:13396980-13397490	23.7717	12.5495	-0.921612
Ciclev10008798m.g	scaffold_1:22831846-22836046	0.69315	0.365927	-0.92161
Ciclev10019121m.g	scaffold_3:7621981-7626079	284.012	150.067	-0.920346
Ciclev10008167m.g	scaffold_1:2880393-2885069	8.38718	4.43345	-0.919756
Ciclev10024684m.g	scaffold_7:20100386-20110034	12.5627	6.6471	-0.918351
Ciclev10011949m.g	scaffold_6:18896260-18902614	159.057	84.1629	-0.918286
Ciclev10018914m.g	scaffold_3:24617311-24734809	32.5442	17.2222	-0.91813
Ciclev10029562m.g	scaffold_8:23561256-23563244	20.6827	10.946	-0.918021
Ciclev10025093m.g	scaffold_7:2132854-2136209	1.41329	0.748135	-0.917688
Ciclev10010727m.g	scaffold_1:496309-496510	225.033	119.125	-0.917658
Ciclev10011732m.g	scaffold_6:18385421-18387448	23.7401	12.5708	-0.91725
Ciclev10013313m.g	scaffold_6:13228028-13229656	183.082	96.9563	-0.917081
Ciclev10014329m.g	scaffold_2:5366119-5378200	21.8025	11.5492	-0.916698
Ciclev10012654m.g	scaffold_6:10316998-10318328	771.856	408.986	-0.916282
Ciclev10014250m.g	scaffold_2:31344536-31347747	1.67307	0.886612	-0.916121
Ciclev10019544m.g	scaffold_3:42144665-42148483	2.45126	1.2994	-0.915675
Ciclev10018994m.g	scaffold_3:37979247-37984468	11.5544	6.12541	-0.915558
Ciclev10022162m.g	scaffold_3:42410481-42413511	90.7324	48.104	-0.91546
Ciclev10033431m.g	scaffold_4:24481755-24485276	26.3135	13.9539	-0.915136
Ciclev10016103m.g	scaffold_2:3003265-3009540	3.75915	1.99378	-0.914897
Ciclev10002622m.g	scaffold_5:34245417-34248236	58.4225	30.9941	-0.914532
Ciclev10028312m.g	scaffold_8:12953963-12968792	67.0055	35.5643	-0.913849
Ciclev10027579m.g	scaffold_7:11062213-11079258	4.57273	2.4274	-0.913643
Ciclev10022381m.g	scaffold_3:39759328-39764478	86.7372	46.0568	-0.913236
Ciclev10007723m.g	scaffold_1:4050230-4052482	5.57691	2.96156	-0.913108
Ciclev10024633m.g	scaffold_3:27179650-27180616	28.9286	15.3625	-0.913082
Ciclev10001617m.g	scaffold_5:42589391-42594049	22.0679	11.7207	-0.912897
Ciclev10019700m.g	scaffold_3:43815646-43818118	0.352448	0.187278	-0.912227
Ciclev10019256m.g	scaffold_3:42526929-42528973	6.30264	3.3491	-0.912182
Ciclev10014352m.g	scaffold_2:5631548-5635227	0.731117	0.388519	-0.912116
Ciclev10014518m.g	scaffold_2:25914012-25918879	2.25038	1.19587	-0.912108
Ciclev10021520m.g	scaffold_3:23388133-23393485	24.6653	13.1076	-0.912078
Ciclev10006032m.g	scaffold_9:658783-659509	8.55407	4.54921	-0.910996
Ciclev10018693m.g	scaffold_3:9893235-9898231	7.65748	4.07267	-0.910894
Ciclev10024021m.g	scaffold_3:44397510-44398185	1.57835	0.839904	-0.910125
Ciclev10008890m.g	scaffold_1:381256-384321	112.737	60.0944	-0.907655
Ciclev10002666m.g	scaffold_5:35123212-35124730	100.18	53.4175	-0.907215
Ciclev10003999m.g	scaffold_5:35938507-35945435	2.66942	1.42405	-0.90653
Ciclev10025373m.g	scaffold_7:7995868-7999139	1.18509	0.632341	-0.90622
Ciclev10012898m.g	scaffold_6:40380-43868	302.433	161.379	-0.906162
Ciclev10029139m.g	scaffold_8:1073116-1075556	35.7371	19.0978	-0.90402
Ciclev10022669m.g	scaffold_3:2221594-2224612	160.637	85.8626	-0.903702
Ciclev10033140m.g	scaffold_4:20658464-20660778	376.649	201.401	-0.903152
Ciclev10030650m.g	scaffold_4:13903685-13910633	4.78373	2.55827	-0.902967

Ciclev10022892m.g	scaffold_3:43610650-43613471	45.7548	24.4734	-0.90271
Ciclev10025289m.g	scaffold_7:7964710-7967426	19.9338	10.6625	-0.902678
Ciclev10002716m.g	scaffold_5:34768271-34769014	2.06005	1.10267	-0.901678
Ciclev10032346m.g	scaffold_4:3935676-3937432	6.3577	3.40579	-0.900516
Ciclev10026112m.g	scaffold_7:5865285-5867488	2.33023	1.24833	-0.900475
Ciclev10032069m.g	scaffold_4:23666783-23669594	55.1499	29.5473	-0.900334
Ciclev10000148m.g	scaffold_5:5126652-5131092	2.66059	1.42559	-0.900182
Ciclev10008836m.g	scaffold_1:526812-529150	140.48	75.2774	-0.900076
Ciclev10014273m.g	scaffold_2:20453030-20458409	0.335375	0.179722	-0.900007
Ciclev10032775m.g	scaffold_4:6602433-6606585	27.488	14.7321	-0.899835
Ciclev10014161m.g	scaffold_2:175428-179259	0.336363	0.180293	-0.899674
Ciclev10021714m.g	scaffold_3:30729865-30732384	137.053	73.545	-0.898033
Ciclev10026842m.g	scaffold_7:15252031-15255159	63.395	34.0304	-0.897547
Ciclev10020087m.g	scaffold_3:46479396-46481959	4.66028	2.50277	-0.896893
Ciclev10015916m.g	scaffold_2:31721816-31726756	4.7809	2.56781	-0.896742
Ciclev10002911m.g	scaffold_5:37128053-37130122	248.618	133.547	-0.896581
Ciclev10015042m.g	scaffold_2:12377694-12379170	31.0505	16.684	-0.896154
Ciclev10001480m.g	scaffold_5:33293771-33295895	6.88697	3.70086	-0.896008
Ciclev10032697m.g	scaffold_4:3335944-3338632	72.6172	39.0296	-0.895743
Ciclev10030878m.g	scaffold_4:177578-182508	54.8896	29.5036	-0.895643
Ciclev10012408m.g	scaffold_6:22060987-22063187	16.6842	8.96852	-0.895542
Ciclev10026407m.g,Ciclev10027256m.g	scaffold_7:11062213-11079258	8.68952	4.67259	-0.895053
Ciclev10033194m.g	scaffold_4:17962048-17995081	21.2066	11.4125	-0.893897
Ciclev10005631m.g	scaffold_9:192171-196670	54.5868	29.3785	-0.893791
Ciclev10008261m.g	scaffold_1:4869088-4870876	0.491502	0.264563	-0.893586
Ciclev10028986m.g	scaffold_8:3102685-3107816	28.4707	15.3254	-0.893557
Ciclev10016496m.g	scaffold_2:1099014-1099731	1.87379	1.00911	-0.892877
Ciclev10017277m.g	scaffold_2:26182548-26186470	108.755	58.5723	-0.892791
Ciclev10003131m.g	scaffold_5:35337109-35341788	2.04622	1.10211	-0.892701
Ciclev10008015m.g	scaffold_1:2551919-2555129	9.09469	4.89999	-0.892248
Ciclev10009213m.g	scaffold_1:19056290-19058412	28.9679	15.6079	-0.892177
Ciclev10024215m.g	scaffold_3:21873837-21877495	10.8537	5.85171	-0.89126
Ciclev10001247m.g	scaffold_5:34778775-34781315	7.9158	4.26811	-0.891136
Ciclev10014683m.g	scaffold_2:35386137-35388993	7.19964	3.88202	-0.891117
Ciclev10023167m.g	scaffold_3:44707536-44708255	2.45248	1.32305	-0.890376
Ciclev10009934m.g	scaffold_1:21883405-21885741	58.745	31.7115	-0.88946
Ciclev10021272m.g	scaffold_3:2459619-2463757	2.18302	1.17845	-0.889427
Ciclev10029427m.g	scaffold_8:23911932-23914475	44.9587	24.2723	-0.88929
Ciclev10021341m.g	scaffold_3:44427594-44429073	6.17942	3.3375	-0.888703
Ciclev10031891m.g	scaffold_4:1358791-1360710	5.01233	2.70742	-0.888564
-	scaffold_5:319524-319807	10.0401	5.42339	-0.888508
Ciclev10025501m.g	scaffold_7:16249522-16255343	13.6689	7.38526	-0.888182
Ciclev10000671m.g	scaffold_5:38177342-38180338	28.2319	15.255	-0.888048
Ciclev10004902m.g	scaffold_9:16997186-17000071	116.414	62.9236	-0.887595
Ciclev10015234m.g	scaffold_2:7725023-7755971	63.9906	34.5904	-0.887491
Ciclev10008387m.g	scaffold_1:17263260-17265543	1.27409	0.688867	-0.887168
Ciclev10001901m.g	scaffold_5:21098160-21102608	185.752	100.457	-0.886807
Ciclev10000217m.g	scaffold_5:41058941-41064267	20.0929	10.8774	-0.885351
Ciclev10028343m.g	scaffold_8:2287525-2290355	19.0378	10.309	-0.884963
Ciclev10005369m.g	scaffold_9:23600427-23601504	1.00002	0.541657	-0.884583
Ciclev10019825m.g	scaffold_3:48944260-48947832	33.1437	17.9548	-0.884364
Ciclev10003065m.g	scaffold_5:29590361-29592399	3.27277	1.77314	-0.88421
Ciclev10029941m.g	scaffold_8:8065794-8112310	17.9671	9.73653	-0.883882
Ciclev10005764m.g	scaffold_9:314840-317599	12.8262	6.95512	-0.882944
Ciclev10032679m.g	scaffold_4:19462704-19467432	0.979369	0.5312	-0.882597

Ciclev10018779m.g	scaffold_3:6394344-6403865	5.64246	3.06059	-0.882512
Ciclev10023834m.g	scaffold_3:48016068-48017656	163.053	88.4468	-0.882462
Ciclev10011817m.g	scaffold_6:25117897-25121077	268.698	145.803	-0.881962
Ciclev10005528m.g	scaffold_9:1787120-1791691	0.697807	0.378776	-0.881483
Ciclev10012153m.g	scaffold_6:21554199-21556921	3.48626	1.89292	-0.881067
Ciclev10013001m.g	scaffold_6:9949966-9952126	150.972	82.0109	-0.880391
Ciclev10026869m.g	scaffold_7:17809780-17810550	1.93862	1.05315	-0.880321
Ciclev10031193m.g	scaffold_4:17679209-17681073	6.19644	3.36638	-0.880243
Ciclev10014776m.g	scaffold_2:27701171-27707522	7.88672	4.28523	-0.880053
Ciclev10015048m.g	scaffold_2:6098264-6102942	24.3794	13.2465	-0.88005
Ciclev10008143m.g	scaffold_1:842000-849183	69.7131	37.8801	-0.879989
Ciclev10008250m.g	scaffold_1:4151518-4154018	5.87053	3.19086	-0.879545
Ciclev10032331m.g	scaffold_4:21222709-21223927	5.75514	3.12853	-0.879365
Ciclev10023210m.g	scaffold_3:41067677-41229006	2.85911	1.55449	-0.879125
Ciclev10017551m.g	scaffold_2:35054706-35055341	3.17715	1.72894	-0.877848
Ciclev10020426m.g	scaffold_3:39339397-39346894	16.7143	9.099	-0.877305
Ciclev10012644m.g	scaffold_6:20285950-20289908	39.885	21.7154	-0.877132
Ciclev10004403m.g,Ciclev10004405m.g	scaffold_9:6243723-6272016	9.47303	5.15872	-0.876811
Ciclev10004257m.g	scaffold_9:28912262-28917003	7.23615	3.94177	-0.876381
Ciclev10007428m.g	scaffold_1:27713331-27716492	46.0025	25.0678	-0.875877
Ciclev10025588m.g	scaffold_7:4587901-4590419	38.2623	20.8545	-0.875563
Ciclev10012376m.g	scaffold_6:17845656-17849940	37.6804	20.5437	-0.875117
Ciclev10007682m.g	scaffold_1:23483863-23485840	0.461161	0.251439	-0.875059
Ciclev10033853m.g	scaffold_4:25231939-25233757	20.7507	11.3143	-0.875018
Ciclev10005750m.g	scaffold_9:26053929-26055086	81.9242	44.6771	-0.874754
Ciclev10002760m.g	scaffold_5:26168593-26171673	16.5727	9.03899	-0.874577
Ciclev10030989m.g	scaffold_4:23845536-23850285	54.3601	29.6501	-0.874508
Ciclev10033029m.g	scaffold_4:2844907-2846923	220.313	120.169	-0.874491
Ciclev10021562m.g	scaffold_3:43267424-43272903	28.1194	15.3393	-0.874339
Ciclev10015614m.g	scaffold_2:5108673-5112244	13.2012	7.20236	-0.87413
Ciclev10006321m.g	scaffold_9:24788901-24789488	8.95701	4.88721	-0.874006
Ciclev10002865m.g	scaffold_5:37721099-37724344	88.6375	48.4328	-0.871932
Ciclev10003615m.g	scaffold_5:36171021-36173637	92.5449	50.5706	-0.871855
Ciclev10001159m.g	scaffold_5:40139284-40142825	104.719	57.2298	-0.871689
Ciclev10009449m.g	scaffold_1:4857159-4860226	6.74994	3.68907	-0.871618
Ciclev10030902m.g	scaffold_4:1478188-1482912	28.663	15.6661	-0.871546
Ciclev10012768m.g	scaffold_6:19959037-19960378	247.391	135.255	-0.871107
Ciclev10028096m.g	scaffold_8:523462-526795	25.9323	14.1852	-0.870367
Ciclev10005935m.g	scaffold_9:19571264-19577393	5.92228	3.24	-0.87016
Ciclev10004178m.g	scaffold_9:27704793-27713963	0.237901	0.130199	-0.869645
Ciclev10014837m.g	scaffold_2:36183108-36186773	0.717181	0.392625	-0.869185
Ciclev10010931m.g	scaffold_6:24774103-24780997	6.13379	3.35808	-0.869141
Ciclev10000800m.g	scaffold_5:41049807-41055702	21.5385	11.7934	-0.868938
Ciclev10004299m.g	scaffold_9:24960880-24963735	0.375349	0.205539	-0.868819
Ciclev10005065m.g	scaffold_9:21593472-21597037	1.18617	0.649565	-0.868767
Ciclev10014204m.g	scaffold_2:5469049-5475142	0.891246	0.488375	-0.867833
Ciclev10012473m.g	scaffold_6:12325774-12327887	74.2948	40.7153	-0.86769
Ciclev10025839m.g	scaffold_7:2394470-2397293	31.5599	17.2984	-0.867456
Ciclev10020812m.g	scaffold_3:17578350-17580588	3.38244	1.85408	-0.86736
Ciclev10024917m.g	scaffold_7:11271704-11277014	228.372	125.209	-0.867045
Ciclev10028488m.g	scaffold_8:9325640-9566326	0.767168	0.420705	-0.866734
Ciclev10029703m.g	scaffold_8:2020262-2030440	26.8711	14.7366	-0.866652
Ciclev10017695m.g	scaffold_2:26105694-26162104	0.358169	0.196466	-0.866365
Ciclev10021759m.g	scaffold_3:45630469-45633102	4.30644	2.36262	-0.866107
Ciclev10022881m.g	scaffold_3:8327048-8328901	148.883	81.6915	-0.865921

Ciclev10020322m.g	scaffold_3:48753306-48756857	258.801	142.007	-0.865878
Ciclev10032479m.g	scaffold_4:24503837-24505365	17.2122	9.44552	-0.86573
Ciclev10025641m.g	scaffold_7:14123947-14154289	55.3603	30.3826	-0.865607
-	scaffold_5:8745603-8989235	20.0078	10.9819	-0.865439
Ciclev10002937m.g	scaffold_5:30526755-30528544	43.0862	23.6544	-0.86512
Ciclev10020034m.g	scaffold_3:208865-211492	6.25984	3.43817	-0.864484
Ciclev10027523m.g	scaffold_7:11093532-11097571	8.47476	4.65698	-0.863777
Ciclev10027728m.g	scaffold_8:22846836-22853502	11.4482	6.29151	-0.863648
Ciclev10004535m.g	scaffold_9:28177909-28179955	10.6244	5.8421	-0.862821
Ciclev10016025m.g	scaffold_2:32998559-33003916	119.844	65.9111	-0.862561
Ciclev10028651m.g	scaffold_8:2599716-2602090	91.4225	50.3176	-0.861486
-	scaffold_5:8745603-8989235	72.1356	39.7155	-0.861011
Ciclev10010368m.g	scaffold_1:19688665-19692235	2.66386	1.46739	-0.860263
Ciclev10007564m.g	scaffold_1:2742372-2746341	2.70851	1.49285	-0.859432
Ciclev10005995m.g	scaffold_9:75347-78181	33.2405	18.3307	-0.858678
Ciclev10024852m.g	scaffold_7:15425968-15428800	0.275784	0.152111	-0.858413
Ciclev10003996m.g	scaffold_5:27123825-27127430	1.49931	0.827102	-0.858166
Ciclev10011799m.g	scaffold_6:15208049-15210795	7.33821	4.04843	-0.858066
Ciclev10023815m.g	scaffold_3:21647622-21652368	13.8174	7.6243	-0.857811
Ciclev10015642m.g	scaffold_2:145508-147469	50.4783	27.8544	-0.857759
Ciclev10015593m.g	scaffold_2:9961645-9964755	3.15298	1.74041	-0.857292
Ciclev10000482m.g	scaffold_5:32912953-32917197	0.649129	0.35834	-0.857175
Ciclev10014336m.g	scaffold_2:35122860-35126714	0.846675	0.467394	-0.857171
Ciclev10000648m.g	scaffold_5:27842975-27853846	43.6595	24.103	-0.857081
Ciclev10015549m.g	scaffold_2:213637-217240	16.0441	8.8582	-0.856959
Ciclev10024791m.g	scaffold_7:1369083-1375811	3.97466	2.19462	-0.856861
Ciclev10000316m.g	scaffold_5:33969748-33974347	13.2102	7.29596	-0.856486
Ciclev10031019m.g	scaffold_4:1183765-1187593	1.23663	0.68306	-0.856326
Ciclev10010993m.g	scaffold_6:21898329-21902563	6.89676	3.81105	-0.85573
Ciclev10014628m.g	scaffold_2:10040101-10043977	25.3124	13.9922	-0.855227
Ciclev10030158m.g	scaffold_8:23991834-23992880	1.49501	0.827271	-0.853727
-	scaffold_5:21372927-21374137	25.149	13.9211	-0.853226
Ciclev10029471m.g	scaffold_8:22080777-22081499	3.12002	1.72709	-0.853209
Ciclev10002888m.g	scaffold_5:40971195-40971829	1.84337	1.02069	-0.852794
Ciclev10005717m.g	scaffold_9:2603133-2605405	219.097	121.414	-0.851634
Ciclev10033987m.g	scaffold_4:1880495-1882329	14.2507	7.89833	-0.851416
Ciclev10026339m.g	scaffold_7:782312-784526	99.5929	55.2038	-0.851275
Ciclev10009500m.g	scaffold_1:26105322-26108198	39.8231	22.0755	-0.851162
Ciclev10004372m.g	scaffold_9:25073692-25080376	72.4565	40.1663	-0.85113
Ciclev10011554m.g	scaffold_6:21450810-21454164	46.018	25.5131	-0.850957
Ciclev10020998m.g	scaffold_3:11479701-11483057	22.5559	12.5069	-0.850779
Ciclev10016641m.g	scaffold_2:34415188-34418709	45.6565	25.3183	-0.850636
Ciclev10029198m.g	scaffold_8:2817297-2822179	45.7615	25.3803	-0.850426
Ciclev10003746m.g	scaffold_5:34542518-34543297	40.4859	22.4556	-0.850345
Ciclev10021720m.g	scaffold_3:26660973-26662413	12.3195	6.83431	-0.850071
Ciclev10021283m.g	scaffold_3:8781653-8785599	6.19776	3.43834	-0.850037
Ciclev10005031m.g	scaffold_9:15066217-15068663	3.79001	2.10358	-0.849355
Ciclev10004560m.g	scaffold_9:5648638-5654374	54.0846	30.0295	-0.848836
Ciclev10032798m.g	scaffold_4:21113893-21115780	9.38879	5.21374	-0.848619
Ciclev10026866m.g	scaffold_7:17841900-17842342	17.2047	9.55453	-0.848543
Ciclev10022988m.g	scaffold_3:12722973-12725030	21.7872	12.1003	-0.848433
-	scaffold_5:1503259-1988798	16.7314	9.29393	-0.848196
Ciclev10012026m.g	scaffold_6:16429542-16431668	1.70262	0.945988	-0.84786
Ciclev10006992m.g	scaffold_9:10360492-10368126	0.49097	0.272841	-0.847577
Ciclev10007404m.g	scaffold_1:21471414-21476410	5.65959	3.14546	-0.847426
Ciclev10027901m.g	scaffold_8:21887371-21893128	11.6782	6.49357	-0.846734

Ciclev10012332m.g	scaffold_6:2969526-2973147	130.947	72.8251	-0.846476
Ciclev10028873m.g	scaffold_8:281618-285300	24.1353	13.4233	-0.846411
Ciclev10033733m.g	scaffold_4:532097-535696	39.1179	21.7563	-0.846399
Ciclev10000893m.g	scaffold_5:29125854-29130238	6.05436	3.36787	-0.846137
Ciclev10028013m.g	scaffold_8:20600991-20605052	8.48976	4.72301	-0.846016
Ciclev10003471m.g	scaffold_5:6255728-6257807	0.682085	0.37955	-0.845662
Ciclev10018802m.g	scaffold_3:6449634-6452593	2.91179	1.62064	-0.845347
Ciclev10019483m.g	scaffold_3:5530363-5535891	2.60048	1.44808	-0.844635
Ciclev10017568m.g	scaffold_2:21376835-21377632	8.41853	4.69027	-0.843897
Ciclev10006138m.g	scaffold_9:25677727-25682495	2.41638	1.3465	-0.843631
Ciclev10016142m.g	scaffold_2:12387212-12388669	49.1672	27.4005	-0.843493
Ciclev10025975m.g	scaffold_7:10701499-10706453	25.1048	13.9919	-0.843371
Ciclev10019237m.g	scaffold_3:48493863-48497265	3.7627	2.09722	-0.843294
Ciclev10023211m.g	scaffold_3:41559329-41560866	1.47061	0.819695	-0.84326
Ciclev10022056m.g	scaffold_3:32835188-32836309	0.624082	0.347901	-0.843058
-	scaffold_2:26983531-26990071	21.0679	11.7447	-0.843034
Ciclev10008024m.g	scaffold_1:17704705-17802486	28.0337	15.6308	-0.842767
Ciclev10020042m.g	scaffold_3:6711278-6716416	70.6708	39.4133	-0.842431
Ciclev10024935m.g	scaffold_7:3832206-3838543	15.3134	8.54329	-0.841934
Ciclev10007483m.g	scaffold_1:24814538-24820057	103.12	57.5387	-0.841721
Ciclev10031803m.g	scaffold_4:16350388-16352768	101.721	56.807	-0.840484
Ciclev10002738m.g	scaffold_5:41285150-41288221	1.40293	0.783936	-0.839632
Ciclev10022949m.g	scaffold_3:20887902-20889521	174.849	97.7764	-0.838554
Ciclev10019738m.g	scaffold_3:6465376-6470870	8.82154	4.93357	-0.838397
Ciclev10002869m.g	scaffold_5:432292-517176	22.5861	12.6342	-0.838096
Ciclev10011623m.g	scaffold_6:22819211-22821782	7.08521	3.9646	-0.837635
Ciclev10008114m.g	scaffold_1:14872334-14876217	35.2013	19.6997	-0.837459
Ciclev10024042m.g	scaffold_3:24352383-24354307	14.0852	7.8854	-0.836922
Ciclev10025175m.g	scaffold_7:9810928-9813429	1.19304	0.668481	-0.83569
Ciclev10032970m.g	scaffold_4:21090407-21092841	82.2717	46.0984	-0.835679
Ciclev10012173m.g	scaffold_6:14318184-14321646	92.2224	51.6852	-0.835365
Ciclev10011298m.g	scaffold_6:17655685-17661763	151.295	84.7962	-0.835289
Ciclev10026503m.g	scaffold_7:3951302-3954097	51.3047	28.759	-0.835078
Ciclev10016688m.g	scaffold_2:10097307-10100347	89.3679	50.1395	-0.833809
-	scaffold_5:18149823-18151138	61.989	34.7952	-0.833122
Ciclev10009076m.g	scaffold_1:738190-743742	138.933	77.9901	-0.833031
Ciclev10016399m.g	scaffold_2:32629907-32634194	48.8665	27.4372	-0.832711
Ciclev10021320m.g	scaffold_3:7762092-7765661	30.9168	17.3658	-0.832144
Ciclev10027750m.g	scaffold_8:20856006-20863026	1.25718	0.706956	-0.830499
Ciclev10015814m.g	scaffold_2:9404427-9406140	55.9168	31.4449	-0.830456
-	scaffold_5:432292-517176	29.6356	16.6816	-0.829074
Ciclev10001807m.g	scaffold_5:29489011-29492069	43.3787	24.4207	-0.82888
Ciclev10013834m.g,Ciclev10013866m.g	scaffold_6:23555036-23557737	888.141	500.126	-0.828496
Ciclev10007910m.g	scaffold_1:1550194-1553253	1.8372	1.03502	-0.827848
Ciclev10024737m.g	scaffold_7:17901397-17905433	10.7772	6.0719	-0.827761
Ciclev10017191m.g	scaffold_2:4679375-4680117	14.9763	8.43911	-0.827516
Ciclev10001031m.g	scaffold_5:41356898-41361583	11.2798	6.35651	-0.827428
Ciclev10008908m.g	scaffold_1:2599956-2603377	97.0068	54.6688	-0.827368
Ciclev10022662m.g	scaffold_3:33162548-33166776	80.4685	45.3491	-0.827351
Ciclev10017201m.g	scaffold_2:8125926-8128247	24.8356	13.9968	-0.827308
Ciclev10027924m.g	scaffold_8:23400114-23403555	44.2307	24.9324	-0.827025
Ciclev10000033m.g	scaffold_5:39311466-39320010	0.500255	0.282063	-0.826643
Ciclev10025139m.g	scaffold_7:12352172-12754529	1.20459	0.679623	-0.825739
Ciclev10028618m.g	scaffold_8:18919180-18923115	1.27321	0.718363	-0.825688
Ciclev10000421m.g	scaffold_5:40589993-40595575	0.709717	0.40056	-0.825227

Ciclev10020713m.g	scaffold_3:6708215-6710187	23.3352	13.1723	-0.824998
Ciclev10012021m.g	scaffold_6:9455950-9460264	322.618	182.126	-0.824888
-	scaffold_8:7207295-7207938	7.01579	3.96077	-0.824823
Ciclev10005535m.g	scaffold_9:2435833-2439595	51.9855	29.3522	-0.824637
Ciclev10028683m.g	scaffold_8:24442335-24444606	73.973	41.7803	-0.824177
Ciclev10019143m.g	scaffold_3:2504394-2508834	12.6175	7.12665	-0.824129
Ciclev10008522m.g	scaffold_1:2664059-2666819	10.5617	5.96573	-0.824073
Ciclev10006358m.g,Ciclev10007074m.g	scaffold_9:15422630-15466157	44.4425	25.1125	-0.823537
Ciclev10024310m.g	scaffold_3:2310797-2312390	235.036	132.818	-0.823429
Ciclev10007936m.g	scaffold_1:898373-902695	51.3979	29.0452	-0.823413
Ciclev10025677m.g	scaffold_7:190663-192426	98.6611	55.768	-0.823045
Ciclev10000235m.g	scaffold_5:119880-126181	254.136	143.732	-0.822225
Ciclev10007490m.g	scaffold_1:19589031-19593011	4.55007	2.5734	-0.822215
Ciclev10023317m.g	scaffold_3:47687295-47692118	44.9813	25.4482	-0.821763
Ciclev10007982m.g	scaffold_1:24547302-24552107	17.2881	9.78143	-0.821662
Ciclev10030207m.g	scaffold_8:5957669-5958563	1.66197	0.940585	-0.821261
Ciclev10026628m.g	scaffold_7:5135979-5139197	94.9657	53.7462	-0.821243
Ciclev10025698m.g	scaffold_7:5434910-5437325	20.8184	11.785	-0.820907
Ciclev10016686m.g	scaffold_2:23239276-23240481	1.73015	0.979427	-0.820886
Ciclev10026012m.g	scaffold_7:14498049-14501384	4.92568	2.78868	-0.82074
Ciclev10019406m.g	scaffold_3:24840385-24845254	40.4152	22.895	-0.819864
Ciclev10016392m.g	scaffold_2:27679704-27683861	20.3015	11.5018	-0.819728
Ciclev10007645m.g	scaffold_1:19595683-19598862	4.90067	2.77647	-0.819724
Ciclev10014603m.g	scaffold_2:34337212-34341162	2.02462	1.14713	-0.819619
Ciclev10020822m.g	scaffold_3:17627459-17631734	22.4173	12.7028	-0.819465
-	scaffold_3:28858148-28858765	14.0007	7.93546	-0.819108
Ciclev10006216m.g	scaffold_9:6697571-6699157	626.939	355.611	-0.818025
Ciclev10009066m.g	scaffold_1:2925041-2932100	12.3758	7.02026	-0.817921
Ciclev10013528m.g	scaffold_6:6591968-6593571	1.50807	0.855486	-0.817886
Ciclev10008969m.g	scaffold_1:25095004-25097485	41.247	23.4023	-0.817639
Ciclev10004743m.g	scaffold_9:16416816-16418476	0.851953	0.4834	-0.817557
Ciclev10015440m.g	scaffold_2:35254805-35258303	40.8309	23.1684	-0.817506
-	scaffold_3:21536334-21537421	64.3203	36.5029	-0.817263
Ciclev10004863m.g	scaffold_9:29774047-29776010	4.52121	2.56591	-0.817241
Ciclev10027892m.g	scaffold_8:21558538-21563671	8.22739	4.66976	-0.817088
Ciclev10008811m.g	scaffold_1:14458466-14461177	3.02079	1.71535	-0.816424
Ciclev10025720m.g	scaffold_7:15263395-15267892	297.495	168.938	-0.816373
Ciclev10023271m.g	scaffold_3:642196-645175	4.65634	2.64717	-0.814744
Ciclev10020015m.g	scaffold_3:7486704-7493083	31.8784	18.1251	-0.814587
Ciclev10027788m.g	scaffold_8:23390336-23397139	17.7567	10.0963	-0.81454
Ciclev10001035m.g	scaffold_5:37232472-37239661	30.4568	17.3179	-0.814498
Ciclev10029409m.g	scaffold_8:23220436-23224438	92.2516	52.4832	-0.813719
Ciclev10029559m.g	scaffold_8:2667021-2669684	12.0049	6.83062	-0.81354
Ciclev10015992m.g	scaffold_2:35259734-35263295	12.5536	7.14503	-0.813092
Ciclev10027506m.g	scaffold_7:16939639-16973173	14.8184	8.43799	-0.812421
Ciclev10018996m.g	scaffold_3:29979422-29987188	8.61163	4.90388	-0.812364
Ciclev10002756m.g	scaffold_5:31121804-31154170	232.235	132.256	-0.812256
Ciclev10028611m.g	scaffold_8:19744964-19746459	2.55046	1.45304	-0.81168
Ciclev10028415m.g	scaffold_8:19885917-19891514	0.538914	0.307088	-0.8114
Ciclev10002211m.g	scaffold_5:40846791-40847806	23.549	13.4232	-0.810935
Ciclev10026597m.g	scaffold_7:11163358-11167847	217.111	123.928	-0.808927
Ciclev10011693m.g	scaffold_6:14244492-14249201	171.752	98.0403	-0.808879
Ciclev10016972m.g	scaffold_2:30445448-30446994	6.73083	3.84258	-0.80871
Ciclev10012022m.g	scaffold_6:23593515-23595492	189.966	108.459	-0.80859
Ciclev10002618m.g	scaffold_5:34191763-34193830	1.08471	0.619333	-0.808523

Ciclev10032726m.g	scaffold_4:1755430-1757505	46.1169	26.3399	-0.808046
Ciclev10031373m.g	scaffold_4:20138850-20141252	68.6083	39.1884	-0.807955
Ciclev10015683m.g	scaffold_2:9291189-9294021	25.3643	14.4883	-0.807911
Ciclev10032455m.g	scaffold_4:22658548-22662302	10.7425	6.13855	-0.807359
Ciclev10009999m.g	scaffold_1:936818-939301	31.0049	17.7179	-0.807286
Ciclev10028229m.g	scaffold_8:19389328-19394447	5.74721	3.2848	-0.807058
Ciclev10006350m.g	scaffold_9:2768141-2829028	22.2145	12.7055	-0.806054
Ciclev10018573m.g	scaffold_3:27857214-27861301	1.50015	0.85804	-0.805988
Ciclev10004749m.g	scaffold_9:4708309-4714548	9.53707	5.45524	-0.805904
Ciclev10028099m.g	scaffold_8:21461942-21467922	14.8648	8.50588	-0.805373
Ciclev10023204m.g	scaffold_3:11687448-12154789	34.7305	19.8738	-0.805334
Ciclev10010916m.g	scaffold_6:1938069-1945512	32.3275	18.5015	-0.805119
Ciclev10028453m.g	scaffold_8:20156680-20160637	31.1889	17.8564	-0.804595
Ciclev10025569m.g	scaffold_7:10000260-10003641	152.737	87.4529	-0.804476
Ciclev10025654m.g	scaffold_7:15473584-15481602	1.02517	0.587065	-0.804266
Ciclev10022488m.g	scaffold_3:46814908-46817550	272.543	156.089	-0.804118
Ciclev10019469m.g	scaffold_3:2691405-2695002	7.70336	4.41197	-0.804064
Ciclev10010250m.g	scaffold_1:24214004-24218228	1.79119	1.02601	-0.803867
Ciclev10025755m.g,Ciclev10026185m.g	scaffold_7:4984937-4993217	62.4998	35.8011	-0.803849
Ciclev10016122m.g	scaffold_2:22428192-22431792	47.3297	27.1114	-0.803848
Ciclev10014190m.g	scaffold_2:22005782-22011411	81.5003	46.688	-0.803753
Ciclev10000216m.g	scaffold_5:2045916-2057989	0.89322	0.511758	-0.803553
Ciclev10006063m.g	scaffold_9:16155704-16161524	36.6469	21.0038	-0.803042
Ciclev10026163m.g	scaffold_7:5676655-5677970	10.6207	6.0946	-0.801279
Ciclev10002702m.g	scaffold_5:29670690-29674529	30.9586	17.7736	-0.800605
Ciclev10026608m.g	scaffold_7:7147318-7149017	40.6155	23.3254	-0.800132
Ciclev10026741m.g	scaffold_7:1324163-1325758	66.2425	38.0686	-0.799156
Ciclev10033677m.g	scaffold_4:21277958-21282582	0.724679	0.416515	-0.798973
Ciclev10012142m.g	scaffold_6:20221207-20222224	2.0249	1.16402	-0.798734
Ciclev10009361m.g	scaffold_1:22810928-22812161	65.5395	37.678	-0.798642
Ciclev10031404m.g	scaffold_4:302165-306794	8.59444	4.94292	-0.79804
Ciclev10027703m.g	scaffold_8:22445585-22451342	19.0634	10.9647	-0.79794
Ciclev10014860m.g	scaffold_2:7966422-7974085	6.47099	3.72393	-0.79716
Ciclev10011265m.g	scaffold_6:18052473-18058151	11.5069	6.6253	-0.796437
Ciclev10023137m.g	scaffold_3:48407101-48407775	66.9934	38.5791	-0.796199
Ciclev10001973m.g	scaffold_5:26990578-26998915	139.488	80.327	-0.796187
Ciclev10010094m.g	scaffold_1:13902170-13905404	15.7927	9.09559	-0.796015
Ciclev10023704m.g	scaffold_3:2177735-2183514	9.92674	5.72261	-0.794648
Ciclev10021249m.g	scaffold_3:20556192-20557621	12.7193	7.33336	-0.794474
Ciclev10016191m.g	scaffold_2:1818384-1824507	56.8578	32.8192	-0.792819
Ciclev10011970m.g	scaffold_6:17523139-17526888	55.772	32.1927	-0.792809
Ciclev10019912m.g	scaffold_3:25286482-25288105	48.61	28.061	-0.792685
Ciclev10008752m.g	scaffold_1:22390817-22394165	12.9568	7.48097	-0.792412
Ciclev10016334m.g	scaffold_2:34146750-34151105	39.9146	23.0479	-0.792283
Ciclev10002668m.g	scaffold_5:38863413-38865093	7.74488	4.47544	-0.791214
Ciclev10032511m.g	scaffold_4:2150475-2153029	39.8589	23.0568	-0.789712
Ciclev10019647m.g	scaffold_3:1935326-1939593	21.4112	12.386	-0.789654
Ciclev10031602m.g	scaffold_4:2434487-2438383	26.7164	15.4588	-0.789296
Ciclev10021785m.g	scaffold_3:2278674-2280175	25.2156	14.5994	-0.78841
Ciclev10012338m.g	scaffold_6:21253669-21258519	79.255	45.8929	-0.788232
Ciclev10006246m.g	scaffold_9:29549721-29554186	2.10156	1.21694	-0.788201
Ciclev10000692m.g	scaffold_5:40112334-40116721	2.30993	1.33799	-0.787778
Ciclev10022057m.g	scaffold_3:34730822-34732211	1.83064	1.06058	-0.787498
Ciclev10030723m.g	scaffold_4:25527915-25534725	50.357	29.175	-0.787457
Ciclev10028768m.g	scaffold_8:23182128-23187363	17.856	10.3469	-0.787215

Ciclev10006560m.g	scaffold_9:28035354-28036664	210.323	121.927	-0.786588
Ciclev10032126m.g	scaffold_4:9593405-9595710	52.8396	30.6338	-0.786497
Ciclev10021370m.g	scaffold_3:41703811-41708879	15.8887	9.21502	-0.785944
Ciclev10001853m.g	scaffold_5:36210238-36213202	115.647	67.0776	-0.785825
Ciclev10011514m.g	scaffold_6:15539584-15544811	8.88414	5.15365	-0.785638
Ciclev10032623m.g	scaffold_4:12339807-12341113	57.0315	33.085	-0.785581
Ciclev10029280m.g	scaffold_8:15963291-15967798	97.1624	56.3889	-0.784987
-	scaffold_8:1620398-1622580	29.682	17.2308	-0.784592
Ciclev10001283m.g	scaffold_5:38352720-38355754	10.9519	6.36014	-0.784057
Ciclev10000597m.g	scaffold_5:39630110-39635609	14.0513	8.16185	-0.783737
Ciclev10027966m.g	scaffold_8:23335673-23341669	16.2942	9.46503	-0.783681
Ciclev10019349m.g	scaffold_3:255780-258629	0.544478	0.316398	-0.783134
Ciclev10012143m.g	scaffold_6:20715589-20719704	8.16418	4.74523	-0.782829
Ciclev10002982m.g	scaffold_5:13193182-13193944	51.3946	29.8779	-0.782537
Ciclev10016080m.g	scaffold_2:33978527-33982559	17.9634	10.4472	-0.781941
Ciclev10032979m.g	scaffold_4:132245-134933	9.15968	5.32828	-0.781626
Ciclev10008549m.g	scaffold_1:4379880-4381807	6.43019	3.74294	-0.780688
Ciclev10008155m.g	scaffold_1:24251484-24257969	46.7428	27.2108	-0.780568
Ciclev10009497m.g	scaffold_1:20003431-20007339	79.9568	46.5482	-0.780494
Ciclev10009804m.g	scaffold_1:5413232-5416822	335.583	195.459	-0.779807
Ciclev10014736m.g	scaffold_2:23045427-23048317	5.13334	2.98999	-0.779759
Ciclev10012623m.g	scaffold_6:19948155-19948958	9.74404	5.67771	-0.77921
Ciclev10028243m.g	scaffold_8:5069263-5071501	9.30733	5.42372	-0.779086
Ciclev10003046m.g	scaffold_5:427419-429934	94.8112	55.2508	-0.779061
Ciclev10020086m.g	scaffold_3:7500527-7502224	13.087	7.62699	-0.778948
Ciclev10000481m.g	scaffold_5:42782826-42787711	9.32766	5.43761	-0.778543
Ciclev10009133m.g	scaffold_1:17185031-17187893	59.4389	34.6675	-0.777826
Ciclev10015597m.g	scaffold_2:34609195-34614203	40.6723	23.7318	-0.777221
Ciclev10026305m.g	scaffold_7:4436885-4440321	70.9467	41.4048	-0.776939
Ciclev10015036m.g	scaffold_2:31706112-31709106	2.97223	1.73482	-0.776764
Ciclev10012928m.g	scaffold_6:20956325-20958294	161.925	94.5388	-0.776347
Ciclev10022976m.g	scaffold_3:12716925-12717904	14.349	8.37803	-0.776271
Ciclev10022088m.g	scaffold_3:50793519-50795382	4.08817	2.38745	-0.775988
Ciclev10014977m.g	scaffold_2:148004-151853	110.256	64.3962	-0.775803
Ciclev10001790m.g,Ciclev10003963m.g	scaffold_5:4289872-4385684	20.9835	12.2566	-0.775705
-	scaffold_2:29308637-29309184	16.6414	9.72273	-0.775345
Ciclev10005291m.g	scaffold_9:31010082-31017820	65.4431	38.238	-0.775234
Ciclev10014117m.g	scaffold_2:30701895-30712151	4.94313	2.88852	-0.775092
Ciclev10033159m.g	scaffold_4:1793569-1795656	15.8016	9.23424	-0.775008
Ciclev10006304m.g	scaffold_9:14647455-14649273	9.98178	5.83322	-0.775004
Ciclev10013137m.g	scaffold_6:25467116-25469211	4.54356	2.65532	-0.774941
Ciclev10019972m.g	scaffold_3:31304518-31310511	71.3376	41.6906	-0.77494
Ciclev10007892m.g	scaffold_1:2981715-2984571	1.23542	0.722562	-0.773804
Ciclev10012261m.g	scaffold_6:20146652-20148628	27.1572	15.8875	-0.773444
Ciclev10031458m.g,Ciclev10031461m.g	scaffold_4:14370384-14384897	176.652	103.359	-0.773249
Ciclev10028297m.g	scaffold_8:12947987-12949765	69.7462	40.8421	-0.772056
Ciclev10025844m.g	scaffold_7:1474246-1475927	19.3364	11.3267	-0.771593
Ciclev10026499m.g	scaffold_7:20885778-20887861	10.1423	5.94122	-0.771559
-	scaffold_8:2882617-2883966	4.31427	2.52724	-0.771557
Ciclev10012002m.g	scaffold_6:2571940-2573984	21.8505	12.7997	-0.771552
Ciclev10000723m.g	scaffold_5:39740866-39742793	0.576911	0.338175	-0.770581
Ciclev10005915m.g	scaffold_9:4037319-4039184	10.4357	6.11807	-0.770377
Ciclev10010761m.g	scaffold_1:3468880-3472058	6.89706	4.04365	-0.770324
Ciclev10013930m.g	scaffold_2451:33-975	1.98813	1.16591	-0.769961

Ciclev10021475m.g	scaffold_3:3211557-3213928	115.63	67.8229	-0.769673
Ciclev10017903m.g	scaffold_2:8863229-8866953	5.75022	3.37343	-0.769398
Ciclev10005714m.g	scaffold_9:4139971-4142301	61.503	36.0838	-0.769305
Ciclev10000483m.g	scaffold_5:41138185-41140305	3.20118	1.87825	-0.76921
Ciclev10001768m.g	scaffold_5:29438710-29443724	3.34331	1.96179	-0.769104
Ciclev10012654m.g	scaffold_6:10316998-10318328	995.442	584.45	-0.768259
Ciclev10028542m.g	scaffold_8:2191753-2197281	8.35841	4.90789	-0.768126
Ciclev10004527m.g	scaffold_9:25578970-25584480	15.7305	9.23674	-0.768111
Ciclev10030962m.g	scaffold_4:6607574-6611460	33.3271	19.5826	-0.767122
Ciclev10001782m.g	scaffold_5:4979770-4981645	4.08917	2.40335	-0.766759
Ciclev10023464m.g	scaffold_3:30618266-30668406	13.3999	7.87585	-0.766709
Ciclev10016196m.g	scaffold_2:30597997-30598935	0.842139	0.495244	-0.765918
Ciclev10021170m.g	scaffold_3:14993578-14994805	455.53	267.901	-0.765848
Ciclev10013044m.g	scaffold_6:20039906-20042958	44.9568	26.4402	-0.765807
Ciclev10019258m.g	scaffold_3:50570867-50578436	11.7094	6.88722	-0.765676
Ciclev10012095m.g	scaffold_6:14188336-14190724	17.348	10.2047	-0.765539
Ciclev10007570m.g	scaffold_1:16463444-16466075	18.5013	10.8835	-0.765483
Ciclev10019904m.g	scaffold_3:5665575-5667630	1.9761	1.16338	-0.764336
Ciclev10005732m.g	scaffold_9:23075529-23122347	17.8544	10.5153	-0.763789
Ciclev10009299m.g	scaffold_1:23500656-23502285	38.6111	22.7401	-0.763777
Ciclev10015508m.g	scaffold_2:6135150-6136571	0.627486	0.369587	-0.763672
Ciclev10001268m.g	scaffold_5:36798413-36806682	0.973762	0.573633	-0.763442
Ciclev10024848m.g	scaffold_7:12181226-12185029	0.338091	0.199231	-0.762967
Ciclev10004151m.g	scaffold_9:253574-260425	17.6084	10.3765	-0.762944
Ciclev10030925m.g	scaffold_4:9171771-9179222	38.0816	22.4415	-0.762926
Ciclev10008821m.g	scaffold_1:28782168-28786731	18.293	10.7824	-0.762611
Ciclev10028353m.g	scaffold_8:4198086-4199601	26.6995	15.743	-0.762096
Ciclev10001706m.g,Ciclev10003426m.g	scaffold_5:35187716-35211685	28.9809	17.0905	-0.761907
Ciclev10006973m.g	scaffold_9:2469386-2472256	30.3427	17.8972	-0.761616
Ciclev10016983m.g	scaffold_2:33859730-33862078	40.1945	23.7133	-0.761301
Ciclev10029538m.g	scaffold_8:23143377-23146210	428.304	252.717	-0.761114
Ciclev10017399m.g	scaffold_2:13440052-13441745	150.64	88.9011	-0.760833
Ciclev10002684m.g	scaffold_5:35558555-35562044	19.1617	11.3086	-0.760803
Ciclev10029611m.g	scaffold_8:6322766-6323840	32.8956	19.415	-0.760726
Ciclev10012358m.g	scaffold_6:22349583-22351196	0.869552	0.513303	-0.76046
Ciclev10015447m.g	scaffold_2:28008518-28012694	8.84847	5.22399	-0.760275
Ciclev10021025m.g	scaffold_3:3082159-3083571	71.8397	42.4189	-0.760074
Ciclev10026208m.g	scaffold_7:535263-538670	29.8152	17.6058	-0.759993
Ciclev10005865m.g	scaffold_9:4029725-4032006	5.22873	3.08782	-0.759873
Ciclev10000054m.g	scaffold_5:42101279-42106547	11.6062	6.85561	-0.75954
Ciclev10017350m.g	scaffold_2:34984844-34985814	7.04857	4.1644	-0.759223
Ciclev10009141m.g	scaffold_1:27286386-27290085	6.7147	3.96883	-0.758607
Ciclev10023233m.g	scaffold_3:46380263-46381057	6.95279	4.11147	-0.757939
Ciclev10017034m.g	scaffold_2:35667231-35668414	8.75447	5.17688	-0.757936
-	scaffold_7:6229367-6229688	14.6648	8.67232	-0.757867
Ciclev10001298m.g	scaffold_5:33980142-33983575	225.138	133.193	-0.757294
Ciclev10002895m.g	scaffold_5:2461405-2462911	65.5332	38.7744	-0.757122
Ciclev10028304m.g	scaffold_8:5612396-5663654	13.2402	7.83656	-0.756629
Ciclev10022658m.g	scaffold_3:602712-605468	31.8584	18.8568	-0.756588
Ciclev10002389m.g	scaffold_5:16056455-16060841	30.0017	17.7585	-0.756533
Ciclev10022697m.g	scaffold_3:23731183-23733880	20.8768	12.3598	-0.756244
Ciclev10030739m.g	scaffold_4:19921297-19926352	3.36647	1.9934	-0.756001
Ciclev10027246m.g	scaffold_7:6692687-6700236	0.357482	0.211729	-0.755655
Ciclev10008958m.g	scaffold_1:5262744-5266604	69.4748	41.1673	-0.754991
Ciclev10022576m.g	scaffold_3:45906878-45907641	24.2188	14.3563	-0.754437

Ciclev10031288m.g	scaffold_4:2755399-2757980	51.7829	30.7049	-0.754006
-	scaffold_8:7696577-7697551	15.529	9.2113	-0.753487
Ciclev10020521m.g	scaffold_3:5475151-5477416	14.7027	8.72164	-0.753406
Ciclev10031377m.g	scaffold_4:12941528-12945334	11.1632	6.62258	-0.753282
Ciclev10012988m.g	scaffold_6:15233991-15234749	27.1591	16.1166	-0.752892
Ciclev10031413m.g	scaffold_4:1012478-1015971	18.8027	11.1589	-0.752749
Ciclev10029961m.g	scaffold_8:24004644-24005037	13.2334	7.85422	-0.752644
Ciclev10026059m.g	scaffold_7:486047-489142	11.0732	6.57363	-0.752306
Ciclev10025353m.g	scaffold_7:6817260-6819191	3.47417	2.0627	-0.752131
Ciclev10021032m.g	scaffold_3:44018159-44021078	164.965	97.9468	-0.752087
Ciclev10026629m.g	scaffold_7:10787932-10805299	23.3601	13.871	-0.751981
Ciclev10021508m.g	scaffold_3:39631321-39637711	29.3877	17.4637	-0.750853
Ciclev10005304m.g	scaffold_9:30527059-30531535	8.7375	5.19365	-0.75047
Ciclev10005496m.g	scaffold_9:28312220-28313482	1.86231	1.10699	-0.750458
Ciclev10028983m.g,Ciclev10029256m.g	scaffold_8:13334846-13338940	20.1523	11.9864	-0.749543
Ciclev10004396m.g	scaffold_9:22309969-22314448	2.35328	1.39972	-0.749534
Ciclev10032300m.g	scaffold_4:1766647-1769955	14.0985	8.38576	-0.749531
Ciclev10005408m.g	scaffold_9:9805898-9809222	25.2219	15.0035	-0.749383
Ciclev10011951m.g	scaffold_6:12700075-12705209	23.8752	14.2113	-0.748471
Ciclev10030143m.g	scaffold_8:5612396-5663654	3.52588	2.09884	-0.748389
Ciclev10032815m.g	scaffold_4:13584975-13588353	55.6522	33.129	-0.748344
Ciclev10012619m.g	scaffold_6:23881967-23889247	80.1427	47.7118	-0.748225
Ciclev10018086m.g	scaffold_2:9374794-9377121	0.896536	0.533821	-0.748005
Ciclev10017243m.g	scaffold_2:21759010-21761073	811.83	483.416	-0.747913
Ciclev10026159m.g	scaffold_7:19000089-19004615	17.6957	10.5389	-0.747665
Ciclev10007718m.g	scaffold_1:28033284-28037413	1.53388	0.91362	-0.747519
Ciclev10019980m.g	scaffold_3:5615539-5620092	9.30097	5.54651	-0.745802
Ciclev10025675m.g	scaffold_7:17264385-17266778	3.38241	2.01722	-0.745678
Ciclev10009122m.g	scaffold_1:21439965-21442808	11.0477	6.58934	-0.745534
Ciclev10032786m.g	scaffold_4:854970-858299	76.4017	45.571	-0.74549
Ciclev10018877m.g,Ciclev10024009m.g	scaffold_3:7528922-7546947	7.68239	4.58375	-0.745027
Ciclev10002707m.g	scaffold_5:35624924-35626992	40.1828	23.9772	-0.744912
Ciclev10023685m.g	scaffold_3:48833027-48838933	5.86606	3.50032	-0.744907
Ciclev10028898m.g	scaffold_8:438274-441426	18.9748	11.3253	-0.744537
Ciclev10029481m.g	scaffold_8:989581-991156	129.589	77.3501	-0.744471
Ciclev10019426m.g	scaffold_3:2348068-2350389	28.9328	17.283	-0.743355
Ciclev10015394m.g	scaffold_2:16548473-16553062	13.0572	7.8003	-0.743246
Ciclev10014255m.g	scaffold_2:32593347-32599764	15.8207	9.45399	-0.74282
Ciclev10009652m.g	scaffold_1:180431-182328	167.717	100.231	-0.7427
Ciclev10032190m.g	scaffold_4:17152925-17155643	3.88432	2.32156	-0.74257
Ciclev10014130m.g	scaffold_2:8693073-8696367	0.814309	0.486733	-0.742447
Ciclev10000851m.g	scaffold_5:26790423-26792763	7.00362	4.18628	-0.74243
Ciclev10009973m.g	scaffold_1:4927379-4929410	33.5251	20.04	-0.742359
Ciclev10011085m.g	scaffold_6:18048848-18051957	1.20763	0.721979	-0.742156
Ciclev10022494m.g	scaffold_3:41437317-41440547	57.3029	34.2674	-0.741773
Ciclev10032771m.g	scaffold_4:23804128-23807087	8.78937	5.25702	-0.741515
Ciclev10007959m.g	scaffold_1:28888520-28892194	23.9587	14.3373	-0.74077
Ciclev10025379m.g	scaffold_7:7317933-7321602	7.61648	4.55849	-0.740569
Ciclev10022028m.g	scaffold_3:2130469-2131672	11.8514	7.09421	-0.740343
Ciclev10032575m.g	scaffold_4:14113371-14114799	5.02111	3.00592	-0.740199
Ciclev10019024m.g	scaffold_3:673069-679076	7.46038	4.46635	-0.740154
Ciclev10026771m.g,Ciclev10026951m.g	scaffold_7:21074579-21091634	35.5517	21.2856	-0.740041
Ciclev10002616m.g	scaffold_5:42850228-42852413	13.3117	7.97035	-0.739979

Ciclev10015142m.g	scaffold_2:35174732-35180227	3.59053	2.15042	-0.739576
Ciclev10012571m.g	scaffold_6:15283058-15290088	12.9948	7.78473	-0.739219
Ciclev10009188m.g	scaffold_1:24693905-24698632	17.8439	10.6954	-0.738434
Ciclev10013553m.g	scaffold_6:20294201-20299455	58.0052	34.7764	-0.738072
Ciclev10024095m.g	scaffold_3:42188413-42192854	4.03651	2.42065	-0.737715
Ciclev10014701m.g	scaffold_2:24698852-24702216	2.13862	1.2828	-0.737389
Ciclev10015574m.g,Ciclev10015586m.g	scaffold_2:10011214-10026423	21.0415	12.6221	-0.737284
Ciclev10031938m.g	scaffold_4:21947337-21948907	0.878879	0.527278	-0.7371
Ciclev10009532m.g	scaffold_1:27145165-27146787	0.995247	0.597227	-0.736777
Ciclev10008850m.g	scaffold_1:2605752-2609177	18.0867	10.855	-0.736564
Ciclev10004795m.g	scaffold_9:22620067-22622404	1.38024	0.828674	-0.736047
Ciclev10022776m.g	scaffold_3:45137043-45145995	4.48311	2.69178	-0.735942
Ciclev10020907m.g	scaffold_3:7058190-7060963	3.79471	2.28067	-0.734532
Ciclev10016681m.g	scaffold_2:3046637-3053472	24.914	14.9736	-0.734529
Ciclev10029052m.g	scaffold_8:23875186-23878850	48.6637	29.2489	-0.73446
Ciclev10032379m.g	scaffold_4:712740-715475	51.2472	30.8059	-0.734264
Ciclev10025781m.g	scaffold_7:569164-574397	49.7579	29.9196	-0.733837
Ciclev10026239m.g	scaffold_7:4267405-4272218	44.9572	27.0347	-0.733741
Ciclev10032010m.g	scaffold_4:22284473-22287046	30.0979	18.1023	-0.733491
Ciclev10029488m.g	scaffold_8:18410915-18411414	9.94775	5.9849	-0.733042
Ciclev10009046m.g	scaffold_1:215151-217266	6.47294	3.89549	-0.732617
Ciclev10023989m.g	scaffold_3:45317349-45317935	6.59718	3.97074	-0.732441
Ciclev10011456m.g	scaffold_6:20973975-20977952	1.49566	0.900337	-0.732245
Ciclev10013206m.g	scaffold_6:12896099-12897242	2.35685	1.41952	-0.731461
Ciclev10028252m.g,Ciclev10028519m.g,Ciclev10028929m.g	scaffold_8:20958969-21005511	42.3852	25.5385	-0.730889
Ciclev10028053m.g	scaffold_8:2800370-2802203	3.39895	2.04855	-0.730487
Ciclev10013540m.g	scaffold_6:7046382-7049367	39.9245	24.0632	-0.730445
Ciclev10016976m.g	scaffold_2:33832515-33835764	4.98502	3.00543	-0.730029
Ciclev10010314m.g	scaffold_1:22175575-22263277	4.10362	2.47507	-0.72943
Ciclev10023264m.g	scaffold_3:45128946-45129672	3.75621	2.26603	-0.729115
Ciclev10025085m.g	scaffold_7:3501753-3506479	13.4383	8.10723	-0.729073
Ciclev10001524m.g	scaffold_5:29481816-29485113	186.481	112.508	-0.729005
Ciclev10019997m.g	scaffold_3:41067677-41229006	1.46771	0.88566	-0.728746
Ciclev10001002m.g	scaffold_5:42974881-42977561	12.4049	7.48613	-0.728614
Ciclev10029483m.g	scaffold_8:3040541-3041447	45.7766	27.6259	-0.728588
Ciclev10031688m.g	scaffold_4:23491316-23494241	2.24203	1.35327	-0.728353
Ciclev10028295m.g	scaffold_8:24090577-24094232	61.9574	37.3978	-0.728324
Ciclev10020097m.g,Ciclev10021527m.g	scaffold_3:8383306-8410026	26.4088	15.943	-0.728099
Ciclev10010372m.g	scaffold_1:15739884-15740196	14.101	8.52185	-0.726563
Ciclev10002153m.g	scaffold_5:42291779-42292947	19.109	11.549	-0.726477
Ciclev10005403m.g	scaffold_9:28757273-28759075	15.1262	9.1428	-0.726337
Ciclev10004297m.g	scaffold_9:1909018-1913742	2.83472	1.71355	-0.726216
Ciclev10013081m.g	scaffold_6:24491130-24492254	197.056	119.119	-0.726194
Ciclev10027792m.g	scaffold_8:386835-392585	8.28276	5.00917	-0.725539
Ciclev10015962m.g	scaffold_2:23070323-23071835	503.192	304.347	-0.725391
Ciclev10005768m.g	scaffold_9:27154798-27158226	38.6283	23.3645	-0.725342
Ciclev10028726m.g	scaffold_8:22998842-23000849	11.6639	7.05655	-0.725011
Ciclev10006232m.g	scaffold_9:275896-277516	20.6657	12.5033	-0.724934
Ciclev10022502m.g	scaffold_3:6149885-6151789	17.8864	10.8317	-0.723595
Ciclev10022922m.g	scaffold_3:25591666-25592171	1.91298	1.1585	-0.723562
Ciclev10022760m.g	scaffold_3:1263749-1264641	101.41	61.4166	-0.723493
Ciclev10021279m.g	scaffold_3:45659221-45663090	46.9019	28.4061	-0.723449

Ciclev10014611m.g	scaffold_2:8911566-8917638	88.4387	53.5651	-0.723386
Ciclev10000775m.g	scaffold_5:38559098-38562819	14.9087	9.03179	-0.723067
Ciclev10005243m.g,Ciclev10005580m.g	scaffold_9:26211987-26236468	20.3118	12.3062	-0.722925
Ciclev10020030m.g	scaffold_3:42025356-42028727	23.6519	14.3319	-0.72273
Ciclev10025980m.g	scaffold_7:399184-411768	13.4844	8.17194	-0.722539
Ciclev10008638m.g	scaffold_1:3356191-3359284	95.6926	58.0145	-0.721995
Ciclev10032919m.g	scaffold_4:24884255-24887178	4.57675	2.77486	-0.721907
Ciclev10002284m.g	scaffold_5:40856377-40860211	6.38121	3.86893	-0.721895
Ciclev10026007m.g	scaffold_7:14481483-14487002	285.876	173.359	-0.721627
Ciclev10011396m.g	scaffold_6:23628745-23631556	22.9592	13.9276	-0.721125
Ciclev10010079m.g	scaffold_1:23554124-23557055	236.057	143.212	-0.720983
Ciclev10027734m.g	scaffold_8:2848883-2853210	1.12063	0.67993	-0.720856
Ciclev10019841m.g	scaffold_3:7792187-7795727	25.3564	15.3922	-0.720153
Ciclev10031989m.g	scaffold_4:12678670-12681681	53.4974	32.4858	-0.719661
Ciclev10016992m.g	scaffold_2:23699663-23700842	12.2173	7.421	-0.71924
Ciclev10007784m.g	scaffold_1:27562750-27565535	0.643834	0.391157	-0.718942
Ciclev10032828m.g	scaffold_4:25415784-25418275	34.0874	20.7102	-0.718897
Ciclev10022285m.g	scaffold_3:6107860-6109237	0.911853	0.554026	-0.718847
Ciclev10019600m.g	scaffold_3:45833455-45838349	59.9331	36.4259	-0.718386
Ciclev10015017m.g	scaffold_2:26262942-26266468	219.488	133.403	-0.718354
Ciclev10021136m.g	scaffold_3:39606430-39608387	24.8237	15.0901	-0.718117
Ciclev10011760m.g	scaffold_6:17369082-17372883	0.791369	0.481086	-0.718056
Ciclev10020049m.g	scaffold_3:47374550-47377688	51.4688	31.2944	-0.717793
Ciclev10025402m.g,Ciclev10027139m.g	scaffold_7:2160504-2167974	27.5547	16.7572	-0.717521
Ciclev10020512m.g	scaffold_3:43950064-43952660	67.3523	40.9745	-0.717
Ciclev10001700m.g	scaffold_5:38404014-38407692	4.29557	2.61329	-0.716981
Ciclev10022158m.g	scaffold_3:49734900-49738269	55.7705	33.9315	-0.716879
Ciclev10007213m.g	scaffold_9:3172304-3172987	6.79112	4.13401	-0.71611
Ciclev10022625m.g	scaffold_3:8715919-8718063	30.265	18.4237	-0.716087
Ciclev10021791m.g	scaffold_3:3556858-3558332	5.22186	3.17911	-0.715944
Ciclev10003326m.g	scaffold_5:26086885-26089960	0.380963	0.231952	-0.715826
Ciclev10009498m.g	scaffold_1:23445787-23446632	1.84836	1.12613	-0.714865
Ciclev10000287m.g	scaffold_5:36445868-36449020	2.4554	1.49629	-0.714571
Ciclev10007365m.g	scaffold_1:18169934-18176457	4.84339	2.95308	-0.713799
Ciclev10024813m.g	scaffold_7:164827-173806	50.4944	30.7882	-0.713746
Ciclev10026011m.g	scaffold_7:5006488-5007808	1.93325	1.17884	-0.713668
Ciclev10028235m.g	scaffold_8:393478-398184	4.80117	2.92823	-0.71336
Ciclev10020507m.g	scaffold_3:4637681-4641334	4.83232	2.94732	-0.713314
Ciclev10009729m.g	scaffold_1:3647538-3651252	55.7986	34.0462	-0.712737
Ciclev10002367m.g	scaffold_5:40735855-40737496	86.4651	52.787	-0.711936
Ciclev10012277m.g,Ciclev10013094m.g	scaffold_6:13420635-13426552	57.9371	35.3758	-0.711727
Ciclev10021719m.g	scaffold_3:31550421-31552673	6.44883	3.93869	-0.711322
Ciclev10022953m.g	scaffold_3:45792121-45793164	41.4037	25.2912	-0.711123
Ciclev10010292m.g	scaffold_1:23370938-23372640	92.4256	56.4598	-0.711068
Ciclev10026581m.g	scaffold_7:221279-224106	10.7364	6.56011	-0.710713
Ciclev10002499m.g	scaffold_5:42835733-42838018	6.86109	4.19343	-0.710306
Ciclev10021692m.g	scaffold_3:44570652-44573213	2.25311	1.37726	-0.710119
Ciclev10001866m.g	scaffold_5:13779700-13783960	53.9917	33.005	-0.710052
Ciclev10031917m.g	scaffold_4:22882713-22885472	5.61844	3.43478	-0.709952
Ciclev10011861m.g	scaffold_6:7976197-7982000	216.171	132.17	-0.709778
Ciclev10020691m.g	scaffold_3:42149295-42153979	29.5388	18.0606	-0.709766
Ciclev10000895m.g	scaffold_5:34312616-34314987	15.8003	9.66361	-0.709315
Ciclev10027848m.g	scaffold_8:23589701-23593390	16.3098	9.97776	-0.708952

Ciclev10018990m.g	scaffold_3:3145372-3149274	14.8568	9.09043	-0.708706
Ciclev10022793m.g	scaffold_3:10425703-10435293	5575.4	3412.58	-0.70821
Ciclev10007706m.g	scaffold_1:1747313-1750072	20.4689	12.5296	-0.708091
Ciclev10018210m.g	scaffold_2:10244022-10247216	14.5912	8.93215	-0.708017
Ciclev10028533m.g	scaffold_8:350998-354814	8.3645	5.12077	-0.707917
Ciclev10006986m.g	scaffold_9:5156626-5157913	1.13201	0.693032	-0.707893
Ciclev10002751m.g	scaffold_5:42187713-42189727	359.636	220.176	-0.707879
Ciclev10016090m.g	scaffold_2:77304-82511	47.854	29.2978	-0.707844
Ciclev10028860m.g	scaffold_8:6474194-6475882	43.3746	26.5671	-0.707208
Ciclev10016695m.g	scaffold_2:35317326-35318478	2.16278	1.32514	-0.706738
Ciclev10024033m.g	scaffold_3:31770209-32016675	479.764	293.998	-0.70652
Ciclev10029601m.g,Ciclev10030428m.g	scaffold_8:6735307-6738952	122.71	75.2193	-0.706076
Ciclev10030460m.g	scaffold_511:2642-7497	8.70162	5.3342	-0.706013
Ciclev10009996m.g	scaffold_1:26483958-26484701	26.7996	16.432	-0.705702
Ciclev10013877m.g	scaffold_6:15246909-15248455	12.2299	7.50034	-0.705384
Ciclev10020981m.g	scaffold_3:49529027-49531042	18.6203	11.4201	-0.705299
Ciclev10005686m.g	scaffold_9:29465748-29470033	23.9524	14.6925	-0.705093
Ciclev10003724m.g	scaffold_5:4985573-4987190	5.47927	3.36173	-0.70478
-	scaffold_1:23382079-23382663	10.5693	6.48471	-0.704761
Ciclev10002220m.g	scaffold_5:39207052-39212539	14.6348	8.98372	-0.704013
Ciclev10012679m.g	scaffold_6:17002903-17003946	265.352	162.901	-0.70391
-	scaffold_5:2607379-2607589	57.9681	35.5991	-0.703417
Ciclev10010071m.g	scaffold_1:7896109-7896650	326.443	200.486	-0.70333
Ciclev10011313m.g	scaffold_6:24361565-24363601	3.73457	2.29381	-0.703198
Ciclev10018269m.g	scaffold_2:25520323-25523768	0.332184	0.204031	-0.703193
Ciclev10002074m.g	scaffold_5:33745670-33747052	2.34499	1.44034	-0.70317
Ciclev10012484m.g	scaffold_6:10785023-10788727	19.2199	11.8073	-0.702917
Ciclev10013028m.g	scaffold_6:21845348-21846126	51.6536	31.741	-0.702519
Ciclev10023445m.g	scaffold_3:25248366-25251368	65.9205	40.5156	-0.702247
Ciclev10011188m.g	scaffold_6:10089074-10092385	2.19871	1.35142	-0.702183
Ciclev10017819m.g	scaffold_2:9852587-9853712	1.14876	0.70615	-0.702032
Ciclev10009334m.g	scaffold_1:19049995-19054408	95.8026	58.8964	-0.701886
-	scaffold_5:6470597-6472518	9.50937	5.84728	-0.701584
Ciclev10014313m.g	scaffold_2:32428284-32433624	0.510887	0.314195	-0.701344
Ciclev10020248m.g	scaffold_3:8647528-8652282	5.61033	3.45043	-0.701311
Ciclev10012181m.g	scaffold_6:8936704-8942681	17.381	10.6929	-0.700864
Ciclev10009897m.g	scaffold_1:24574963-24578460	21.4227	13.1815	-0.700621
Ciclev10032956m.g	scaffold_4:24237867-24243284	448.659	276.212	-0.699844
Ciclev10009595m.g	scaffold_1:28187318-28187926	8.1363	5.01072	-0.699356
Ciclev10003485m.g	scaffold_5:36398845-36399845	26.5606	16.3574	-0.699347
Ciclev10003464m.g	scaffold_5:28289811-28801386	15.671	9.65654	-0.698518
Ciclev10029324m.g	scaffold_8:5471135-5474488	88.6982	54.6695	-0.698168
Ciclev10000997m.g	scaffold_5:39589972-39592011	12.8831	7.94069	-0.698142
Ciclev10002355m.g	scaffold_5:35847755-35849138	52.419	32.3234	-0.697512
-	scaffold_2:4983885-4984540	16.6645	10.2804	-0.696887
Ciclev10026120m.g	scaffold_7:8905795-8909126	73.7185	45.4773	-0.696879
Ciclev10005507m.g	scaffold_9:17053579-17057386	5.57471	3.43957	-0.69667
Ciclev10023374m.g	scaffold_3:25612740-25615635	7.44935	4.59773	-0.696192
Ciclev10025732m.g	scaffold_7:10595204-10596667	1.27017	0.78403	-0.696045
Ciclev10024920m.g	scaffold_7:9119761-9125872	14.3096	8.833	-0.696008
Ciclev10012993m.g	scaffold_6:309808-311028	8.72046	5.38326	-0.695925
Ciclev10002804m.g	scaffold_5:32313298-32314734	102.215	63.1429	-0.694916
Ciclev10007097m.g	scaffold_9:2412444-2414535	35.5908	21.9871	-0.694848
Ciclev10022618m.g	scaffold_3:49633249-49635677	28.9715	17.8992	-0.694733
Ciclev10014998m.g	scaffold_2:16597777-16602257	66.9999	41.3972	-0.694626

Ciclev10003932m.g	scaffold_5:22238856-22240725	0.732084	0.452635	-0.693661
Ciclev10032042m.g	scaffold_4:17048346-17052031	39.7977	24.6077	-0.693576
Ciclev10001784m.g	scaffold_5:34501140-34506609	66.9412	41.3924	-0.693527
Ciclev10009626m.g	scaffold_1:20995351-21034862	37.0485	22.9097	-0.693454
Ciclev10022492m.g	scaffold_3:1069368-1070035	51.5956	31.9069	-0.693382
Ciclev10002478m.g	scaffold_5:42236417-42238575	10.5897	6.55312	-0.692408
Ciclev10031847m.g	scaffold_4:2543855-2546646	78.7127	48.7115	-0.692334
Ciclev10011865m.g	scaffold_6:18194883-18202657	19.5329	12.0898	-0.69211
Ciclev10027866m.g,Ciclev10027910m.g	scaffold_8:20620078-20723794	24.3859	15.0939	-0.692076
Ciclev10022885m.g	scaffold_3:38639877-38643065	7.7174	4.7793	-0.691317
Ciclev10012396m.g	scaffold_6:15458257-15462034	24.6561	15.2703	-0.69121
Ciclev10003084m.g	scaffold_5:11913693-11914695	67.5019	41.8254	-0.690551
Ciclev10031833m.g	scaffold_4:19127245-19130791	17.5054	10.8467	-0.690545
Ciclev10006079m.g	scaffold_9:361253-363893	146.411	90.7216	-0.690507
Ciclev10008581m.g	scaffold_1:26509535-26513207	16.3135	10.1097	-0.690317
Ciclev10017178m.g	scaffold_2:23905996-23909204	98.6835	61.166	-0.690079
Ciclev10018495m.g	scaffold_3:8700207-8708402	5.83348	3.6161	-0.689922
Ciclev10029096m.g	scaffold_8:1935173-1936163	9.27886	5.75217	-0.689841
Ciclev10019146m.g	scaffold_3:49512337-49517307	9.71491	6.02435	-0.689396
Ciclev10026785m.g	scaffold_7:1272258-1274372	130.791	81.1141	-0.689236
Ciclev10031633m.g	scaffold_4:22511997-22515961	26.9629	16.724	-0.689058
Ciclev10004522m.g	scaffold_9:30219241-30225790	8.39819	5.21016	-0.68875
Ciclev10009355m.g	scaffold_1:24807941-24811326	97.3697	60.4315	-0.688172
Ciclev10022288m.g	scaffold_3:34673450-34674900	3.56452	2.21262	-0.687956
Ciclev10012997m.g	scaffold_6:20090843-20095946	231.997	144.129	-0.686744
Ciclev10022105m.g	scaffold_3:5275890-5280289	36.1492	22.4593	-0.686653
Ciclev10005426m.g	scaffold_9:26886479-26890121	6.39818	3.97518	-0.68664
Ciclev10022601m.g	scaffold_3:7012273-7013949	145.364	90.3157	-0.686617
Ciclev10021340m.g	scaffold_3:6695170-6697430	54.0749	33.6153	-0.685841
Ciclev10031452m.g	scaffold_4:7379155-7388483	6.64943	4.1342	-0.685622
Ciclev10013129m.g	scaffold_6:25568568-25569321	76.5837	47.6339	-0.685048
Ciclev10007716m.g	scaffold_1:985260-989352	63.3322	39.3927	-0.68501
Ciclev10021514m.g	scaffold_3:2289044-2290757	4.34939	2.70549	-0.684923
Ciclev10015811m.g,Ciclev10016912m.g	scaffold_2:32236437-32241277	38.751	24.1094	-0.684636
Ciclev10021411m.g	scaffold_3:23891294-23893475	10.8017	6.72043	-0.684632
Ciclev10021199m.g	scaffold_3:24617311-24734809	4.49632	2.79797	-0.684366
Ciclev10007983m.g	scaffold_1:22378858-22384122	28.5435	17.7624	-0.684341
Ciclev10033080m.g	scaffold_4:355917-356561	194.857	121.261	-0.684302
Ciclev10008350m.g	scaffold_1:13194347-13198000	16.8042	10.4709	-0.682439
Ciclev10010078m.g	scaffold_1:11698839-11706621	77.276	48.1553	-0.682328
Ciclev10019431m.g	scaffold_3:22301511-22304667	3.40969	2.12547	-0.68186
Ciclev10009887m.g	scaffold_1:2184512-2186967	223.685	139.452	-0.681702
Ciclev10027721m.g	scaffold_8:18642992-18650693	0.424966	0.265024	-0.681228
Ciclev10029296m.g	scaffold_8:10520203-10524265	59.8578	37.3381	-0.680891
Ciclev10014962m.g	scaffold_2:7388981-7393977	11.6761	7.28338	-0.680873
Ciclev10026528m.g	scaffold_7:13888033-13889844	6.00565	3.74633	-0.68084
Ciclev10020170m.g	scaffold_3:43776898-43781978	7.11407	4.43791	-0.680796
Ciclev10016769m.g	scaffold_2:30485160-30488443	158.135	98.6603	-0.680616
Ciclev10007849m.g	scaffold_1:27167270-27171073	3.37485	2.10675	-0.679806
Ciclev10026362m.g	scaffold_7:5243897-5245847	20.5175	12.8095	-0.679647
Ciclev10033947m.g	scaffold_4:2493519-2495321	1.7769	1.10955	-0.67939
Ciclev10016668m.g	scaffold_2:23180084-23182810	60.6533	37.8842	-0.678988
-	scaffold_3:21296577-21473212	8.04315	5.02392	-0.678946
Ciclev10013040m.g	scaffold_6:23325023-23326443	0.688141	0.42984	-0.678905

Ciclev10013100m.g	scaffold_6:18217725-18223869	34.6341	21.6383	-0.678605
Ciclev10023143m.g	scaffold_3:12330210-12331677	181.014	113.135	-0.678054
Ciclev10008869m.g	scaffold_1:23654784-23658857	27.2388	17.0367	-0.677017
-	scaffold_5:10404740-10539701	50.8896	31.8297	-0.676997
Ciclev10027142m.g	scaffold_7:20045067-20048699	93.2903	58.3631	-0.67667
Ciclev10000130m.g	scaffold_5:36049676-36056860	4.62589	2.89475	-0.676293
Ciclev10002435m.g	scaffold_5:29398393-29399900	2.63333	1.64789	-0.676265
Ciclev10000612m.g	scaffold_5:39216417-39219746	23.9539	14.991	-0.67617
Ciclev10028964m.g	scaffold_8:461188-468899	201.956	126.4	-0.676048
Ciclev10020808m.g,Ciclev10024646m.g	scaffold_3:48542252-48545077	8.96078	5.60883	-0.675924
Ciclev10029688m.g	scaffold_8:19551715-19552158	10.9996	6.88624	-0.675659
Ciclev10014104m.g	scaffold_2:13909410-13917679	16.405	10.2738	-0.675174
Ciclev10000980m.g,Ciclev10001263m.g	scaffold_5:31925317-31943860	32.0897	20.0975	-0.675095
Ciclev10025668m.g	scaffold_7:16217360-16226054	9.64792	6.04296	-0.674963
Ciclev10017099m.g	scaffold_2:35138034-35138729	25.9289	16.2406	-0.674955
Ciclev10015901m.g,Ciclev10015912m.g	scaffold_2:6964963-6980953	241.535	151.314	-0.674684
Ciclev10017164m.g	scaffold_2:35112849-35114799	83.3798	52.2429	-0.674462
Ciclev10016262m.g	scaffold_2:35546691-35549835	78.0169	48.8887	-0.674286
Ciclev10026570m.g	scaffold_7:6771772-6772812	27.5931	17.2927	-0.674149
Ciclev10014208m.g	scaffold_2:20410112-20451140	6.83206	4.28257	-0.673843
Ciclev10024221m.g	scaffold_3:32543189-32702382	4.1601	2.60842	-0.673439
Ciclev10017248m.g	scaffold_2:27141147-27141869	94.149	59.0438	-0.673159
Ciclev10001412m.g	scaffold_5:23972062-23973828	113.704	71.31	-0.673112
Ciclev10011724m.g	scaffold_6:20987327-20988808	1.16405	0.730039	-0.673107
Ciclev10000454m.g	scaffold_5:29808452-29811460	1.58664	0.995493	-0.672493
Ciclev10011444m.g	scaffold_6:25183899-25188972	15.4228	9.68085	-0.671862
Ciclev10019080m.g	scaffold_3:18902661-18909006	33.3281	20.9225	-0.671683
Ciclev10014850m.g	scaffold_2:27551455-27556561	9.01575	5.66085	-0.671428
Ciclev10025991m.g	scaffold_7:4008632-4012745	13.5227	8.49259	-0.671104
Ciclev10021135m.g	scaffold_3:47341663-47343185	7.18879	4.51557	-0.670841
Ciclev10011959m.g	scaffold_6:20767620-20771052	15.9055	9.99107	-0.670815
Ciclev10002419m.g	scaffold_5:15052797-15054938	105.648	66.3667	-0.67073
Ciclev10033040m.g	scaffold_4:17149087-17151664	29.3308	18.4264	-0.670648
Ciclev10006140m.g	scaffold_9:3671927-3674756	73.0481	45.891	-0.670637
Ciclev10033818m.g	scaffold_4:24544918-24546401	1.16656	0.733063	-0.670256
Ciclev10013679m.g	scaffold_6:12141279-12144299	3.90599	2.45484	-0.670061
Ciclev10004456m.g	scaffold_9:29517562-29520928	22.4251	14.094	-0.670031
Ciclev10023978m.g	scaffold_3:18212175-18214200	375.876	236.257	-0.669899
Ciclev10000970m.g,Ciclev10004068m.g	scaffold_5:41703786-41708779	39.3203	24.7158	-0.669843
Ciclev10007175m.g	scaffold_9:30817398-30818556	3.48436	2.19099	-0.669309
Ciclev10001334m.g	scaffold_5:41609583-41613450	34.4966	21.6943	-0.669138
Ciclev10028831m.g	scaffold_8:461188-468899	203.819	128.226	-0.668601
Ciclev10005597m.g	scaffold_9:12562561-12570023	51.0736	32.1351	-0.668428
Ciclev10015947m.g	scaffold_2:3029121-3032017	1.88332	1.18535	-0.667967
Ciclev10032055m.g	scaffold_4:22554705-22557986	23.529	14.8102	-0.667849
Ciclev10001640m.g	scaffold_5:34472166-34474847	12.5456	7.89881	-0.667472
Ciclev10022591m.g	scaffold_3:4360528-4364138	41.8732	26.3659	-0.667356
Ciclev10017176m.g	scaffold_2:35382238-35384647	63.7218	40.1269	-0.667217
Ciclev10004391m.g	scaffold_9:24672267-24677081	12.9765	8.17175	-0.667184
Ciclev10019660m.g	scaffold_3:48927020-48930561	4.45881	2.80837	-0.666927
Ciclev10013246m.g	scaffold_6:16811267-16812118	7.47716	4.70979	-0.666826
-	scaffold_5:1503259-1988798	2.93235	1.8474	-0.66656

Ciclev10002352m.g	scaffold_5:41735342-41738372	11.7031	7.3735	-0.666464
Ciclev10013891m.g	scaffold_6:20618961-20620281	1.79839	1.13309	-0.666444
Ciclev10012265m.g	scaffold_6:22305921-22308021	10.5479	6.64736	-0.666101
Ciclev10005137m.g	scaffold_9:3930050-3934147	60.5232	38.1472	-0.66591
Ciclev10019102m.g	scaffold_3:46951319-46953737	119.152	75.1158	-0.665611
Ciclev10021379m.g	scaffold_3:48689811-48692761	23.4637	14.7923	-0.665579
Ciclev10026818m.g,Ciclev10027192m.g	scaffold_7:17883681-17891230	16.845	10.621	-0.665409
Ciclev10021137m.g	scaffold_3:44180162-44185000	15.7981	9.96227	-0.665209
-	scaffold_3:41539716-41543598	35.7363	22.5448	-0.664598
Ciclev10002106m.g	scaffold_5:38469257-38470252	4.6708	2.94668	-0.664578
Ciclev10004050m.g	scaffold_5:39282607-39283237	1.32029	0.832954	-0.66455
Ciclev10002549m.g	scaffold_5:42838210-42840133	5.09269	3.21306	-0.664479
Ciclev10012227m.g	scaffold_6:2198314-2200549	208.393	131.481	-0.664453
Ciclev10005161m.g	scaffold_9:2131513-2134136	0.85412	0.538919	-0.66437
Ciclev10005544m.g	scaffold_9:6977143-6980019	3.0241	1.90891	-0.663755
Ciclev10026261m.g	scaffold_7:1794791-1797256	50.896	32.1276	-0.663741
Ciclev10003605m.g	scaffold_5:40676228-40679319	5.70456	3.60271	-0.663033
Ciclev10002883m.g	scaffold_5:26949950-26952841	90.0238	56.8641	-0.662788
Ciclev10011209m.g	scaffold_6:17143868-17145969	3.46187	2.18704	-0.662569
Ciclev10015100m.g	scaffold_2:35997708-36001681	13.8732	8.76583	-0.662339
Ciclev10015659m.g	scaffold_2:35772673-35774362	1.15778	0.731626	-0.662177
Ciclev10033270m.g	scaffold_4:21151909-21153516	394.1	249.049	-0.662134
Ciclev10001978m.g	scaffold_5:40832602-40834625	38.5128	24.3379	-0.662133
Ciclev10006005m.g,Ciclev10007209m.g	scaffold_9:6318925-6325120	18.286	11.5557	-0.66213
Ciclev10013829m.g	scaffold_6:21288355-21290142	1.7272	1.09212	-0.6613
Ciclev10012703m.g	scaffold_6:17983936-17985312	41.0981	25.992	-0.661007
Ciclev10015391m.g	scaffold_2:10963885-10968435	41.0748	25.9795	-0.660882
Ciclev10015505m.g	scaffold_2:28893662-28896553	7.221	4.56956	-0.660143
Ciclev10029495m.g	scaffold_8:24051886-24052549	2.31785	1.46707	-0.659851
Ciclev10012852m.g	scaffold_6:24225829-24230019	8.16785	5.1707	-0.659598
Ciclev10033989m.g	scaffold_4:62661-63517	15.5255	9.82909	-0.659506
Ciclev10012794m.g	scaffold_6:23084454-23085332	3.17233	2.00886	-0.65917
Ciclev10012950m.g	scaffold_6:22467564-22470029	115.769	73.3164	-0.659035
Ciclev10014610m.g	scaffold_2:25506205-25509221	80.0401	50.6897	-0.65903
Ciclev10011594m.g	scaffold_6:20677175-20680157	1.06	0.671397	-0.658827
Ciclev10032655m.g	scaffold_4:22558446-22560710	6.3003	3.99061	-0.658811
Ciclev10031208m.g	scaffold_4:25164475-25166250	1.31771	0.834865	-0.658423
Ciclev10005148m.g	scaffold_9:25493377-25500370	80.0204	50.6994	-0.658398
Ciclev10012732m.g	scaffold_6:7123526-7128824	13.1804	8.35158	-0.658274
Ciclev10033122m.g	scaffold_4:17175040-17177917	14.4976	9.18632	-0.658256
Ciclev10032989m.g	scaffold_4:24203177-24206181	3.00274	1.90291	-0.658073
Ciclev10031367m.g	scaffold_4:3795951-3799940	22.3866	14.1888	-0.657883
Ciclev10028542m.g	scaffold_8:2191753-2197281	65.2948	41.405	-0.657165
Ciclev10006199m.g	scaffold_9:1691733-1693684	230.344	146.124	-0.656603
Ciclev10023925m.g	scaffold_3:50700488-50702648	9.66079	6.13211	-0.655758
Ciclev10030170m.g	scaffold_8:14782878-14784817	13.0352	8.27547	-0.6555
Ciclev10018352m.g	scaffold_2:13528033-13529425	1.21935	0.774164	-0.655397
Ciclev10022242m.g	scaffold_3:3659452-3662674	11.1831	7.10277	-0.654863
Ciclev10014400m.g	scaffold_2:35497039-35504104	0.829012	0.526675	-0.654479
Ciclev10001493m.g	scaffold_5:40745800-40750063	24.2687	15.4196	-0.654328
Ciclev10010628m.g	scaffold_1:25871864-25876939	10.0616	6.39359	-0.654157
-	scaffold_5:2735523-2736062	3.40346	2.16427	-0.653122
Ciclev10020446m.g	scaffold_3:46191686-46193918	4.11395	2.61654	-0.652864
Ciclev10005315m.g	scaffold_9:5301260-5303362	40.7305	25.9071	-0.652761

Ciclev10004880m.g	scaffold_9:166431-170727	52.6307	33.4842	-0.652424
Ciclev10023071m.g	scaffold_3:27798972-27801782	55.0867	35.0484	-0.652357
Ciclev10002744m.g	scaffold_5:26510552-26513762	40.6383	25.8577	-0.652248
Ciclev10008537m.g	scaffold_1:18068296-18070262	10.9083	6.94128	-0.652152
Ciclev10029290m.g	scaffold_8:3597904-3599596	1.53109	0.974525	-0.651787
Ciclev10025247m.g	scaffold_7:8976000-8980710	1.78079	1.13363	-0.651565
Ciclev10023298m.g,Ciclev10024673m.g	scaffold_3:49856927-49859158	4.38271	2.79069	-0.651203
Ciclev10000611m.g	scaffold_5:42898493-42904653	39.16	24.9389	-0.65098
Ciclev10019396m.g	scaffold_3:43795212-43800518	19.7319	12.5699	-0.650555
Ciclev10022435m.g	scaffold_3:45916862-45919225	210.863	134.398	-0.649793
Ciclev10028224m.g	scaffold_8:24965433-24970341	11.2433	7.16634	-0.649759
Ciclev10001068m.g	scaffold_5:35538653-35541459	53.2834	33.9783	-0.64907
Ciclev10024071m.g	scaffold_3:24856115-24862275	10.644	6.78791	-0.648996
Ciclev10005284m.g	scaffold_9:214087-217850	18.6048	11.865	-0.648962
Ciclev10001058m.g	scaffold_5:41662679-41665662	36.7675	23.4498	-0.648853
Ciclev10000539m.g	scaffold_5:12689295-12694012	5.80375	3.70236	-0.648538
Ciclev10008900m.g	scaffold_1:4722888-4726339	30.2491	19.2968	-0.648532
Ciclev10016738m.g	scaffold_2:35980340-35984186	117.348	74.8651	-0.648432
Ciclev10020312m.g	scaffold_3:30393564-30398972	61.4359	39.2008	-0.648199
Ciclev10001438m.g	scaffold_5:41479362-41481770	22.6985	14.4896	-0.647574
Ciclev10012115m.g	scaffold_6:22767248-22771845	23.2761	14.8745	-0.646005
Ciclev10009395m.g	scaffold_1:25761569-25764926	68.937	44.0572	-0.645901
Ciclev10013228m.g	scaffold_6:1950120-1950908	91.0646	58.199	-0.645895
Ciclev10033263m.g	scaffold_4:21417274-21419616	32.5745	20.8249	-0.645433
Ciclev10015570m.g	scaffold_2:7250563-7255818	4.5225	2.8916	-0.645251
Ciclev10029517m.g	scaffold_8:23443331-23445027	93.7268	59.9426	-0.64488
Ciclev10033061m.g	scaffold_4:25058290-25061910	114.316	73.1152	-0.64478
Ciclev10026431m.g	scaffold_7:14268410-14275412	19.5116	12.4867	-0.643934
Ciclev10031358m.g	scaffold_4:16817583-17013320	2.54994	1.632	-0.643816
Ciclev10009534m.g	scaffold_1:8364058-8368382	157.611	100.878	-0.643747
Ciclev10004130m.g	scaffold_9:3954506-3961401	1.9852	1.27073	-0.64362
Ciclev10016689m.g	scaffold_2:35790870-35793694	27.0487	17.3155	-0.643496
Ciclev10019314m.g	scaffold_3:1188545-1191618	8.36788	5.35839	-0.643061
Ciclev10029587m.g	scaffold_8:18844078-18844621	13.3474	8.54707	-0.643054
Ciclev10016687m.g	scaffold_2:36277702-36279976	90.4626	57.9424	-0.642702
Ciclev10016161m.g	scaffold_2:29963275-29967760	5.47013	3.5049	-0.642204
Ciclev10006008m.g	scaffold_9:4307053-4313440	24.1321	15.4631	-0.642125
Ciclev10017578m.g	scaffold_2:35968350-35970144	7.84296	5.02658	-0.641821
Ciclev10026260m.g	scaffold_7:14309893-14311942	95.1515	61.0022	-0.641364
Ciclev10031151m.g	scaffold_4:18742364-18749147	14.6563	9.40263	-0.640389
Ciclev10020950m.g	scaffold_3:3391579-3395278	7.43986	4.77476	-0.639846
Ciclev10033729m.g	scaffold_4:23609396-23612148	77.8968	50.0041	-0.639517
Ciclev10022847m.g	scaffold_3:17717681-17720651	40.1273	25.7623	-0.639324
Ciclev10016008m.g	scaffold_2:4516987-4518242	8.21279	5.274	-0.638977
Ciclev10001615m.g	scaffold_5:33079707-33082768	15.215	9.77151	-0.638844
Ciclev10011553m.g	scaffold_6:6280767-6288163	8.86716	5.69588	-0.638552
Ciclev10013282m.g	scaffold_6:19885485-19888972	0.598916	0.385053	-0.637296
Ciclev10024995m.g	scaffold_7:10035354-10116411	11.769	7.56724	-0.637158
Ciclev10021469m.g	scaffold_3:41043662-41046480	237.391	152.744	-0.636155
Ciclev10027745m.g	scaffold_8:24537-40607	128.63	82.7757	-0.635948
Ciclev10009810m.g	scaffold_1:24132094-24133290	61.4262	39.5403	-0.635531
-	scaffold_9:22497579-22498553	9.92836	6.39151	-0.635398
Ciclev10031676m.g	scaffold_4:19462704-19467432	13.0803	8.42141	-0.635263
Ciclev10026399m.g	scaffold_7:11136740-11138535	18.6983	12.0393	-0.635158
Ciclev10000926m.g	scaffold_5:34382123-34384067	1.98769	1.28007	-0.634865

Ciclev10002917m.g	scaffold_5:35357332-35359897	77.9028	50.1699	-0.634853
Ciclev10005664m.g	scaffold_9:4126568-4131986	57.3285	36.9317	-0.634396
Ciclev10033682m.g	scaffold_4:2941167-2941449	16.2565	10.4744	-0.634142
Ciclev10013702m.g	scaffold_6:368900-373454	46.3913	29.8972	-0.633843
Ciclev10008199m.g,Ciclev10010827m.g	scaffold_1:1970230-1976964	11.6649	7.52485	-0.632442
Ciclev10028781m.g	scaffold_8:10587797-10592154	52.0013	33.5471	-0.632361
Ciclev10008093m.g	scaffold_1:24602748-24608633	15.4349	9.95794	-0.63228
Ciclev10006724m.g	scaffold_9:2330431-2331657	16.6187	10.7292	-0.631262
Ciclev10001464m.g	scaffold_5:27602513-27607801	8.94711	5.77698	-0.631107
Ciclev10015122m.g	scaffold_2:33018147-33022811	19.7478	12.7517	-0.631007
Ciclev10001023m.g	scaffold_5:36743352-36746199	32.2709	20.8397	-0.630897
Ciclev10009047m.g	scaffold_1:28928260-28932927	122.087	78.8542	-0.630648
Ciclev10008847m.g	scaffold_1:25427130-25431022	39.0369	25.226	-0.629923
Ciclev10009952m.g	scaffold_1:24468087-24470417	238.325	154.017	-0.629838
Ciclev10002627m.g	scaffold_5:36646218-36647375	21.9631	14.1964	-0.629554
Ciclev10009147m.g	scaffold_1:1981538-2047130	17.5356	11.3382	-0.62909
Ciclev10000614m.g	scaffold_5:42888592-42893193	6.33958	4.10046	-0.628604
Ciclev10031177m.g	scaffold_4:2290942-2317254	9.75187	6.30799	-0.628498
Ciclev10032230m.g	scaffold_4:16295893-16298529	4.96594	3.21254	-0.628355
Ciclev10024916m.g	scaffold_7:11329174-11340037	7.93751	5.13507	-0.628304
Ciclev10014293m.g	scaffold_2:11194588-11210308	19.6329	12.7013	-0.628301
Ciclev10001422m.g	scaffold_5:23986984-23992055	427.636	276.714	-0.627989
Ciclev10008455m.g	scaffold_1:17487217-17491004	17.5836	11.3809	-0.627608
Ciclev10018688m.g	scaffold_3:25100777-25104843	4.91557	3.18175	-0.627539
Ciclev10026281m.g	scaffold_7:16273806-16281313	15.5024	10.0393	-0.626828
Ciclev10014855m.g	scaffold_2:27125174-27129923	38.0712	24.6571	-0.626699
Ciclev10000853m.g	scaffold_5:39626614-39629746	17.8163	11.5395	-0.626622
Ciclev10002837m.g	scaffold_5:29677633-29680577	77.3388	50.0966	-0.626477
Ciclev10018724m.g	scaffold_3:33748482-33753225	0.441524	0.286079	-0.626078
Ciclev10015656m.g	scaffold_2:27303083-27306417	490.413	317.767	-0.626028
Ciclev10026828m.g	scaffold_7:4565985-4569023	123.535	80.0598	-0.625775
Ciclev10033624m.g,Ciclev10033884m.g	scaffold_4:10565457-10571296	34.5287	22.3771	-0.625774
Ciclev10005842m.g	scaffold_9:22394206-22395728	191.296	123.991	-0.625574
Ciclev10015386m.g	scaffold_2:11306353-11307978	31.1678	20.2036	-0.625441
Ciclev10011643m.g	scaffold_6:11401277-11405083	6.36462	4.12703	-0.62497
Ciclev10021913m.g	scaffold_3:8859135-8863221	21.3634	13.855	-0.624734
Ciclev10021722m.g	scaffold_3:5234417-5238376	23.5033	15.2437	-0.624646
Ciclev10029371m.g	scaffold_8:5159427-5162015	13.1724	8.54388	-0.624551
Ciclev10001506m.g	scaffold_5:35521571-35526568	11.7769	7.64022	-0.62428
Ciclev10012455m.g	scaffold_6:20131173-20132496	36.6191	23.7577	-0.624202
Ciclev10029395m.g	scaffold_8:24785531-24788306	12.5386	8.13818	-0.623597
Ciclev10025725m.g	scaffold_7:1677727-1684978	5.64209	3.66271	-0.623317
Ciclev10020899m.g	scaffold_3:2371343-2375148	4.20959	2.73382	-0.622762
Ciclev10018822m.g	scaffold_3:46648299-46651560	0.587779	0.381802	-0.622451
Ciclev10005578m.g	scaffold_9:25585517-25588460	11.0864	7.20161	-0.622396
Ciclev10011869m.g	scaffold_6:19271061-19274322	33.9989	22.0866	-0.622321
Ciclev10028617m.g	scaffold_8:18043132-18047093	17.3112	11.2543	-0.621231
Ciclev10005921m.g	scaffold_9:2568033-2571241	102.531	66.659	-0.621195
Ciclev10005824m.g	scaffold_9:25727826-25731198	5.89956	3.83553	-0.621181
Ciclev10032833m.g	scaffold_4:12732810-12733635	3.01095	1.95812	-0.62075
Ciclev10026488m.g	scaffold_7:5277964-5281312	164.592	107.044	-0.62069
-	scaffold_8:21397777-21398138	17.0721	11.1036	-0.620618
Ciclev10025242m.g	scaffold_7:133318-138971	14.0458	9.13599	-0.620509
Ciclev10026073m.g	scaffold_7:5557829-5560118	4.69843	3.0565	-0.6203

Ciclev10032288m.g	scaffold_4:25079433-25080528	13.8768	9.02774	-0.620238
Ciclev10009520m.g	scaffold_1:24812068-24813774	69.1604	44.9984	-0.620072
Ciclev10006197m.g	scaffold_9:2362828-2365557	89.5043	58.2377	-0.620003
Ciclev10014689m.g	scaffold_2:26821202-26826986	14.6724	9.5491	-0.619665
Ciclev10001485m.g	scaffold_5:37652360-37656378	20.431	13.3044	-0.618852
Ciclev10003744m.g	scaffold_5:21183146-21187249	1.02191	0.665569	-0.618609
Ciclev10031841m.g	scaffold_4:1951539-1955276	50.7866	33.0828	-0.618368
Ciclev10002305m.g	scaffold_5:41035559-41041106	50.9492	33.2024	-0.617773
Ciclev10014848m.g	scaffold_2:6994733-6996507	137.354	89.5228	-0.61757
Ciclev10031509m.g	scaffold_4:22007450-22012267	12.616	8.22365	-0.617407
Ciclev10020225m.g	scaffold_3:47162657-47165654	3.26332	2.12781	-0.616971
Ciclev10032050m.g	scaffold_4:7301637-7308849	39.795	25.9511	-0.616787
Ciclev10008332m.g	scaffold_1:26450529-26458334	26.3718	17.1989	-0.616682
Ciclev10030941m.g	scaffold_4:2414210-2417518	8.74727	5.70503	-0.616599
Ciclev10020600m.g	scaffold_3:1876220-1878308	104.12	67.9364	-0.615985
Ciclev10009976m.g	scaffold_1:22837499-22839817	157.251	102.607	-0.615938
Ciclev10001908m.g	scaffold_5:40317720-40321609	97.4429	63.6092	-0.615322
Ciclev10030352m.g	scaffold_8:16622597-16633140	22.9487	14.9806	-0.615314
Ciclev10001181m.g	scaffold_5:34574495-34591686	60.7028	39.628	-0.615243
Ciclev10008084m.g	scaffold_1:920122-923236	7.10162	4.63741	-0.614828
Ciclev10031583m.g	scaffold_4:2734380-2741269	14.5833	9.52434	-0.614626
Ciclev10012146m.g	scaffold_6:23130453-23133783	10.6363	6.94673	-0.614585
Ciclev10026511m.g	scaffold_7:2590902-2593669	49.3979	32.2749	-0.614036
Ciclev10026914m.g	scaffold_7:9089279-9091276	317.281	207.409	-0.61328
Ciclev10026263m.g	scaffold_7:17424213-17426197	41.675	27.2443	-0.613225
Ciclev10022189m.g	scaffold_3:43215600-43219210	20.8458	13.6294	-0.613034
Ciclev10016228m.g	scaffold_2:30499704-30504591	51.0514	33.3904	-0.612518
Ciclev10012559m.g	scaffold_6:21589936-21593918	117.489	76.8564	-0.612294
Ciclev10018950m.g	scaffold_3:3535833-3540825	6.90747	4.51884	-0.612205
Ciclev10026213m.g	scaffold_7:6388670-6392102	18.5205	12.117	-0.612088
Ciclev10029385m.g	scaffold_8:24102432-24104345	7.10163	4.64766	-0.611647
Ciclev10000812m.g	scaffold_5:37693881-37699178	21.4498	14.0402	-0.6114
Ciclev10028931m.g	scaffold_8:998266-1001763	38.9557	25.5047	-0.61107
Ciclev10026473m.g	scaffold_7:14251173-14252914	2.92114	1.91312	-0.610606
Ciclev10005072m.g	scaffold_9:27749863-27751612	4.48656	2.93841	-0.610573
Ciclev10002412m.g	scaffold_5:41850269-41852717	48.5156	31.7796	-0.610348
Ciclev10002741m.g	scaffold_5:39957970-39960666	42.069	27.5619	-0.610081
Ciclev10002059m.g	scaffold_5:38743042-38746428	11.33	7.42431	-0.609817
Ciclev10032537m.g	scaffold_4:102163-107421	161.955	106.129	-0.609766
Ciclev10013652m.g	scaffold_6:16023581-16026609	1.12376	0.736548	-0.609478
Ciclev10009711m.g	scaffold_1:28209010-28211161	7.54212	4.94642	-0.608585
Ciclev10011798m.g	scaffold_6:23484090-23485669	10.6532	6.98694	-0.608554
Ciclev10009944m.g	scaffold_1:7737644-7741202	21.9009	14.3657	-0.608358
Ciclev10016259m.g	scaffold_2:9987433-9992419	27.2675	17.8884	-0.608157
-	scaffold_1:25681597-25682217	10.6182	6.96719	-0.607895
Ciclev10011137m.g	scaffold_6:23469416-23474907	2.12813	1.39646	-0.60781
Ciclev10001844m.g	scaffold_5:165096-169348	329.961	216.567	-0.60748
Ciclev10008258m.g	scaffold_1:27503995-27506104	1.70806	1.12139	-0.607068
Ciclev10025879m.g	scaffold_7:16587337-16590811	2.23732	1.46905	-0.606885
Ciclev10007443m.g	scaffold_1:19326315-19329876	82.5022	54.1748	-0.606809
Ciclev10011587m.g	scaffold_6:22213543-22219300	195.319	128.268	-0.60667
Ciclev10011319m.g	scaffold_6:21280302-21287360	40.7769	26.7789	-0.606655
Ciclev10011666m.g	scaffold_6:22117549-22123151	6.97516	4.58274	-0.606017
Ciclev10003522m.g	scaffold_5:31469286-31635063	1.05367	0.692781	-0.604951
Ciclev10023382m.g	scaffold_3:21296577-21473212	18.9973	12.491	-0.604904
Ciclev10028748m.g	scaffold_8:152384-156478	8.11667	5.33781	-0.604641

Ciclev10028687m.g	scaffold_8:18726775-18729035	17.5975	11.5752	-0.604335
Ciclev10016149m.g	scaffold_2:3632402-3633350	26.084	17.1583	-0.604254
Ciclev10007383m.g	scaffold_1:7771870-7776941	4.58212	3.01449	-0.604101
-	scaffold_8:18129037-18129357	26.5872	17.4917	-0.604059
Ciclev10031300m.g,Ciclev10032410m.g	scaffold_4:16460022-16475213	25.0159	16.459	-0.603967
Ciclev10022321m.g	scaffold_3:29153424-29156189	138.387	91.0582	-0.603849
Ciclev10025279m.g	scaffold_7:20298359-20304509	26.1958	17.2442	-0.603223
Ciclev10011665m.g	scaffold_6:12779845-12784130	19.3194	12.7195	-0.60301
Ciclev10006114m.g	scaffold_9:5144382-5146965	49.5278	32.6108	-0.602886
Ciclev10004463m.g	scaffold_9:29432438-29439510	26.0496	17.1527	-0.602825
Ciclev10005563m.g	scaffold_9:20219861-20223557	314.869	207.341	-0.602748
Ciclev10030535m.g	scaffold_4:24588405-24594706	11.1451	7.33948	-0.602665
-	scaffold_6:18382262-18382764	1239.56	816.329	-0.60261
Ciclev10007997m.g	scaffold_1:24383593-24387969	12.1416	8.00232	-0.601469
Ciclev10006192m.g	scaffold_9:15045799-15049915	2.72921	1.79902	-0.601276
-	scaffold_6:14408775-14408968	152.869	100.784	-0.601026
Ciclev10007702m.g	scaffold_1:861278-878303	54.8085	36.1387	-0.600856
Ciclev10022955m.g	scaffold_3:6218544-6219405	74.3711	49.0377	-0.600851
Ciclev10015639m.g	scaffold_2:29629667-29632222	3.48395	2.29727	-0.600803
Ciclev10022455m.g	scaffold_3:42922693-42926274	49.3525	32.5506	-0.60044
Ciclev10011567m.g	scaffold_6:21352984-21358482	36.905	24.3413	-0.600407
Ciclev10021834m.g	scaffold_3:6000265-6002833	6.94408	4.58113	-0.600079
Ciclev10002302m.g,Ciclev10003933m.g	scaffold_5:33432090-33472845	23.8807	15.7588	-0.599686
Ciclev10002797m.g	scaffold_5:41899221-41901896	48.3462	31.9067	-0.599543
Ciclev10017011m.g	scaffold_2:7292922-7336431	10.1359	6.69104	-0.599174
Ciclev10005330m.g	scaffold_9:2545340-2550917	20.494	13.5288	-0.599167
Ciclev10011574m.g	scaffold_6:15283058-15290088	15.0849	9.95851	-0.599108
Ciclev10023306m.g	scaffold_3:40236721-40244926	0.426666	0.281714	-0.598874
Ciclev10013115m.g	scaffold_6:5988162-5990923	125.149	82.6685	-0.59824
Ciclev10022884m.g	scaffold_3:18902661-18909006	3.17672	2.09914	-0.59774
Ciclev10020624m.g	scaffold_3:33687571-33691590	29.6018	19.5631	-0.597553
Ciclev10001961m.g	scaffold_5:36463101-36467207	11.1001	7.33642	-0.597424
Ciclev10023690m.g,Ciclev10024516m.g	scaffold_3:32543189-32702382	18.4872	12.2189	-0.597407
Ciclev10026612m.g	scaffold_7:11302181-11306350	87.7597	58.006	-0.597357
Ciclev10012646m.g	scaffold_6:23416403-23418629	40.5661	26.8135	-0.597316
Ciclev10025246m.g	scaffold_7:2269736-2272048	16.0078	10.5853	-0.596713
Ciclev10016078m.g	scaffold_2:23002424-23005703	10.024	6.62908	-0.596577
Ciclev10029369m.g,Ciclev10029534m.g	scaffold_8:2655906-2660636	899.773	595.077	-0.596486
Ciclev10033019m.g	scaffold_4:25166545-25167334	134.71	89.0951	-0.596439
Ciclev10011895m.g	scaffold_6:14174811-14178267	23.829	15.7617	-0.5963
Ciclev10026450m.g	scaffold_7:7656669-7660212	44.0943	29.1741	-0.595904
Ciclev10026350m.g	scaffold_7:5289431-5293368	209.098	138.402	-0.595309
Ciclev10001277m.g	scaffold_5:4607325-4613461	51.3062	33.9625	-0.595189
Ciclev10008333m.g	scaffold_1:25028239-25030911	4.10435	2.71724	-0.595013
Ciclev10015463m.g	scaffold_2:27497100-27501650	10.3242	6.83538	-0.594941
Ciclev10022424m.g	scaffold_3:5759570-5761045	38.4394	25.4521	-0.5948
Ciclev10021916m.g	scaffold_3:40946284-40949763	26.1252	17.3008	-0.594603
Ciclev10029767m.g	scaffold_8:4143726-4145846	53.0199	35.1166	-0.594382
Ciclev10016576m.g	scaffold_2:13857321-13860557	16.4609	10.9044	-0.594127
Ciclev10017385m.g	scaffold_2:35075675-35078521	0.530764	0.35168	-0.593809
Ciclev10026767m.g	scaffold_7:7313005-7315653	25.9753	17.2123	-0.593702
Ciclev10008802m.g	scaffold_1:20468397-20472963	3.17158	2.10166	-0.593675

Ciclev10024374m.g	scaffold_3:7716702-7717651	2.49003	1.65035	-0.593394
Ciclev10022479m.g	scaffold_3:8477602-8488997	39.2077	25.9867	-0.593365
Ciclev10018935m.g	scaffold_3:42800194-42807761	2.90562	1.92599	-0.593247
Ciclev10030996m.g	scaffold_4:23705325-23711365	17.9279	11.8838	-0.593212
Ciclev10006836m.g	scaffold_9:4636689-4638899	3.02391	2.00461	-0.593097
Ciclev10029872m.g	scaffold_8:6750492-6786903	0.205425	0.13619	-0.592995
Ciclev10016749m.g	scaffold_2:24290272-24291409	9.35956	6.20623	-0.592725
Ciclev10022969m.g	scaffold_3:4232103-4234557	104.564	69.3369	-0.592696
Ciclev10007063m.g	scaffold_9:1638769-1641073	77.6451	51.4934	-0.592508
Ciclev10028299m.g	scaffold_8:20879068-20883824	9.37706	6.22024	-0.592164
Ciclev10011684m.g,Ciclev10012652m.g	scaffold_6:10816139-10936716	523.155	347.115	-0.591825
Ciclev10031090m.g	scaffold_4:24916682-24919674	17.1957	11.4101	-0.591734
Ciclev10032964m.g	scaffold_4:24505807-24507682	7.56871	5.02488	-0.590957
Ciclev10029854m.g	scaffold_8:7925785-7963167	213.001	141.421	-0.590862
Ciclev10006522m.g	scaffold_9:2968846-2971662	2.06507	1.3712	-0.590759
Ciclev10031798m.g	scaffold_4:3448975-3453191	13.977	9.28078	-0.590737
Ciclev10029354m.g	scaffold_8:18064760-18071003	71.0467	47.193	-0.590195
Ciclev10012355m.g	scaffold_6:16795059-16797562	13.4177	8.9145	-0.589912
Ciclev10031445m.g	scaffold_4:24770753-24773948	11.8297	7.86	-0.589807
Ciclev10031861m.g	scaffold_4:19207459-19212937	11.6987	7.77304	-0.589793
Ciclev10004925m.g	scaffold_9:5680840-5687460	41.012	27.2515	-0.58971
Ciclev10028466m.g	scaffold_8:2456339-2458494	6.86473	4.56187	-0.589577
Ciclev10005476m.g	scaffold_9:3616195-3621117	41.3651	27.4887	-0.589577
Ciclev10008916m.g	scaffold_1:26957968-26961254	9.91597	6.59079	-0.589302
Ciclev10019413m.g	scaffold_3:9998252-10001532	1.1398	0.757593	-0.589291
Ciclev10015857m.g	scaffold_2:30296874-30298160	1.55641	1.03456	-0.589202
Ciclev10004589m.g	scaffold_9:22593942-22600743	6.94541	4.61758	-0.588924
Ciclev10003138m.g	scaffold_5:39999082-40000451	4.57822	3.04394	-0.588846
Ciclev10024345m.g	scaffold_3:21763501-21767140	3.13575	2.08511	-0.588688
Ciclev10004641m.g	scaffold_9:1027069-1029381	7.86289	5.23033	-0.588159
Ciclev10015355m.g	scaffold_2:7952692-7954772	7.64206	5.08641	-0.587314
Ciclev10014600m.g	scaffold_2:32873929-32878999	22.1331	14.7342	-0.587043
Ciclev10002660m.g	scaffold_5:31895664-31902875	6.90798	4.59986	-0.586676
Ciclev10033094m.g	scaffold_4:12900776-12906838	37.0611	24.6906	-0.585943
Ciclev10016372m.g	scaffold_2:35836829-35839763	14.3528	9.5639	-0.585657
Ciclev10000047m.g	scaffold_5:414255-420341	1.46525	0.976364	-0.585652
Ciclev10025057m.g,Ciclev10027466m.g	scaffold_7:7357041-7362305	5.94372	3.96099	-0.585504
Ciclev10019338m.g	scaffold_3:1813569-1816531	1.24625	0.830716	-0.585171
Ciclev10013275m.g	scaffold_6:1735295-1740289	235.557	157.049	-0.584862
Ciclev10000062m.g	scaffold_5:43093043-43098705	7.60719	5.07214	-0.584769
Ciclev10008079m.g	scaffold_1:93834-96613	4.04525	2.69803	-0.584325
Ciclev10009527m.g	scaffold_1:20529779-20531009	1.75198	1.16852	-0.584299
Ciclev10012450m.g	scaffold_6:23053706-23056690	44.8752	29.9316	-0.584251
Ciclev10013611m.g	scaffold_6:22928778-22931069	10.652	7.10602	-0.584014
Ciclev10009515m.g	scaffold_1:3711748-3716370	63.085	42.0924	-0.583736
Ciclev10025511m.g	scaffold_7:8962354-8965569	71.4867	47.7084	-0.583432
Ciclev10031787m.g	scaffold_4:16570651-16572491	4.81727	3.21504	-0.58338
Ciclev10021053m.g	scaffold_3:1149032-1152392	15.9513	10.6481	-0.583078
Ciclev10015551m.g	scaffold_2:6764624-6767330	2.82557	1.88633	-0.582961
Ciclev10033290m.g	scaffold_4:2774149-2775819	43.9507	29.3602	-0.582022
Ciclev10006262m.g	scaffold_9:17213186-17213665	67.6354	45.1987	-0.581498
Ciclev10033978m.g	scaffold_4:17111497-17118007	1.04847	0.700861	-0.581084
Ciclev10016324m.g	scaffold_2:33988035-33989927	9.45328	6.32073	-0.580725
Ciclev10020789m.g	scaffold_3:31133565-31137650	67.0361	44.8393	-0.580176

Ciclev10012495m.g	scaffold_6:23314956-23318185	248.784	166.418	-0.580086
Ciclev10008750m.g	scaffold_1:4612232-4614602	36.2917	24.2819	-0.579756
Ciclev10004633m.g	scaffold_9:23562-25565	1.3593	0.909495	-0.579727
Ciclev10013279m.g	scaffold_6:9261148-9263251	145.621	97.4663	-0.579244
Ciclev10008790m.g	scaffold_1:1713891-1717819	18.9234	12.6659	-0.579219
Ciclev10019007m.g	scaffold_3:6981494-6987498	0.701353	0.469461	-0.579135
Ciclev10002993m.g	scaffold_5:37060930-37065943	0.616666	0.412784	-0.579103
Ciclev10012697m.g	scaffold_6:17491240-17494671	51.9924	34.8055	-0.578985
Ciclev10025791m.g	scaffold_7:2938058-2940407	1.20681	0.807918	-0.578917
Ciclev10012715m.g	scaffold_6:231680-234280	153.038	102.458	-0.578848
Ciclev10024269m.g	scaffold_3:49202498-49205157	53.353	35.7208	-0.578804
Ciclev10007452m.g,Ciclev10007525m.g,Ciclev10007667m.g,Ciclev10007741m.g	scaffold_1:19496171-19588291	33.2938	22.2993	-0.578255
Ciclev10018323m.g	scaffold_2:6156047-6158225	0.150196	0.100632	-0.577766
Ciclev10016134m.g,Ciclev10016136m.g	scaffold_2:35407310-35413897	352.621	236.259	-0.577752
Ciclev10015803m.g	scaffold_2:2671734-2674368	10.2786	6.88724	-0.577645
Ciclev10015076m.g	scaffold_2:10691117-10694680	18.3163	12.2748	-0.577433
Ciclev10009428m.g	scaffold_1:1671834-1674259	41.5285	27.8324	-0.577335
Ciclev10002639m.g	scaffold_5:31300142-31302469	37.3978	25.0662	-0.577211
-	scaffold_7:11686956-11687523	8.93666	5.99054	-0.577049
Ciclev10021543m.g	scaffold_3:44483698-44485554	23.8604	15.9949	-0.577007
Ciclev10005618m.g	scaffold_9:383810-387133	2.5054	1.67952	-0.576992
Ciclev10033044m.g	scaffold_4:5040356-5042760	63.8954	42.8343	-0.576946
Ciclev10022463m.g	scaffold_3:2878161-2881754	56.5049	37.8861	-0.576708
Ciclev10009427m.g	scaffold_1:2156678-2158871	239.179	160.372	-0.576666
Ciclev10018823m.g	scaffold_3:30878294-30882778	7.23743	4.85323	-0.576532
Ciclev10005704m.g	scaffold_9:13311971-13317864	33.3665	22.3779	-0.576321
Ciclev10016179m.g	scaffold_2:26271555-26273261	8.27741	5.55204	-0.57616
Ciclev10001248m.g	scaffold_5:33825678-33829182	13.8796	9.31025	-0.576072
Ciclev10028655m.g	scaffold_8:4892021-4896871	58.0524	38.9597	-0.575375
Ciclev10014979m.g	scaffold_2:23326559-23330494	9.59299	6.43922	-0.575094
Ciclev10021784m.g	scaffold_3:1634919-1638281	49.6079	33.3032	-0.57491
Ciclev10004793m.g	scaffold_9:469600-472684	149.3	100.267	-0.574368
Ciclev10010043m.g	scaffold_1:26752315-26756663	185.87	124.831	-0.574319
Ciclev10015725m.g	scaffold_2:34965938-34971995	13.2468	8.89759	-0.574158
Ciclev10011809m.g	scaffold_6:6265277-6272266	27.7755	18.6607	-0.573812
Ciclev10012836m.g	scaffold_6:12352194-12353054	17.3005	11.6235	-0.573765
Ciclev10024591m.g	scaffold_3:27952801-27953704	24.8807	16.7245	-0.573069
Ciclev10000140m.g	scaffold_5:40363272-40372586	15.2102	10.2241	-0.573063
Ciclev10019763m.g	scaffold_3:49554049-49560198	77.7528	52.277	-0.572717
Ciclev10031558m.g	scaffold_4:17682884-17686035	18.0417	12.1321	-0.5725
Ciclev10021860m.g	scaffold_3:48603076-48605404	43.7864	29.4509	-0.572169
Ciclev10016113m.g	scaffold_2:2938111-2943331	52.4775	35.3046	-0.571842
Ciclev10001246m.g	scaffold_5:38766602-38769204	11.7639	7.91438	-0.571813
Ciclev10008681m.g	scaffold_1:487183-489198	20.3896	13.7215	-0.571394
Ciclev10029774m.g	scaffold_8:16726668-16727617	9.85484	6.63269	-0.571239
Ciclev10021207m.g	scaffold_3:16732451-16739154	24.3852	16.4127	-0.571192
Ciclev10016475m.g	scaffold_2:26888062-26889923	5.4935	3.69752	-0.571168
Ciclev10019675m.g	scaffold_3:42816820-42820382	16.8206	11.3215	-0.571163
Ciclev10009141m.g	scaffold_1:27286386-27290085	9.62569	6.47977	-0.570947
Ciclev10004330m.g	scaffold_9:1763487-1769433	24.4571	16.4659	-0.570778
Ciclev10029525m.g	scaffold_8:19499144-19501435	7.78173	5.24067	-0.57034
Ciclev10006699m.g	scaffold_9:1663233-1663989	10.5362	7.09703	-0.570067
Ciclev10025225m.g	scaffold_7:19613448-19617030	2.18626	1.47276	-0.569941

Ciclev10019223m.g	scaffold_3:1858388-1861721	2.95115	1.98828	-0.569755
Ciclev10029536m.g	scaffold_8:17529062-17529928	67.8968	45.7442	-0.569754
Ciclev10031598m.g	scaffold_4:19681883-19685929	68.3534	46.0598	-0.569506
Ciclev10008851m.g	scaffold_1:4747929-4750674	5.01444	3.37956	-0.569251
Ciclev10001293m.g	scaffold_5:9983418-9985631	1.73327	1.16834	-0.569027
Ciclev10015472m.g	scaffold_2:34516498-34518924	49.4604	33.3495	-0.568609
Ciclev10021561m.g	scaffold_3:2755858-2757382	3.22158	2.17361	-0.567674
Ciclev10011520m.g	scaffold_6:21816067-21819488	43.0309	29.0391	-0.567378
Ciclev10005316m.g	scaffold_9:21248395-21518846	9.68791	6.53791	-0.567356
Ciclev10019938m.g	scaffold_3:48089472-48091232	17.4523	11.7842	-0.566563
Ciclev10002551m.g,Ciclev10004073m.g	scaffold_5:42109315-42112542	42.7022	28.8397	-0.566253
Ciclev10016517m.g	scaffold_2:3658542-3663160	70.9258	47.9079	-0.566046
Ciclev10021493m.g	scaffold_3:42915946-42921130	12.7516	8.61407	-0.565911
Ciclev10006052m.g	scaffold_9:9489994-9490507	68.7557	46.4468	-0.565899
Ciclev10008816m.g	scaffold_1:1599577-1603604	9.48433	6.40865	-0.565526
Ciclev10031911m.g	scaffold_4:21235127-21237954	9.70799	6.56008	-0.565459
Ciclev10017234m.g	scaffold_2:35785115-35786782	17.3611	11.7348	-0.565062
Ciclev10021292m.g	scaffold_3:5351245-5355794	15.138	10.2339	-0.564811
Ciclev10015153m.g	scaffold_2:14442706-14449384	94.7348	64.0461	-0.564785
Ciclev10014720m.g	scaffold_2:65556-69410	30.1668	20.3971	-0.564595
Ciclev10010087m.g	scaffold_1:9666063-9668524	135.635	91.723	-0.564378
Ciclev10022810m.g	scaffold_3:43192761-43194352	187.334	126.702	-0.564173
Ciclev10031317m.g	scaffold_4:8082073-8090442	18.5508	12.5467	-0.564173
Ciclev10011695m.g	scaffold_6:22039490-22042363	8.47775	5.7355	-0.563762
Ciclev10005143m.g	scaffold_9:2983707-2988080	14.6967	9.94477	-0.563482
Ciclev10002710m.g	scaffold_5:41618252-41620257	164.368	111.271	-0.562848
Ciclev10030474m.g	scaffold_4:9547398-9559504	8.78557	5.94879	-0.562539
Ciclev10009111m.g	scaffold_1:697202-700509	61.4629	41.6334	-0.561973
Ciclev10025591m.g	scaffold_7:6127463-6131417	76.3124	51.6991	-0.561778
Ciclev10031777m.g	scaffold_4:19383823-19390838	105.533	71.497	-0.561736
-	scaffold_5:28029347-28029893	5.00625	3.39174	-0.561705
Ciclev10026615m.g	scaffold_7:2152754-2153670	25.66	17.385	-0.561673
Ciclev10032423m.g	scaffold_4:19970059-19974163	11.8083	8.00288	-0.561217
Ciclev10004815m.g	scaffold_9:664986-669744	21.6642	14.6834	-0.561132
Ciclev10003193m.g	scaffold_5:27263742-27265461	3.43486	2.3288	-0.560667
Ciclev10002653m.g	scaffold_5:29699946-29702961	51.4428	34.8841	-0.5604
Ciclev10026066m.g	scaffold_7:15381031-15382603	10.4423	7.0814	-0.560336
Ciclev10018754m.g	scaffold_3:22787984-22801272	16.1299	10.9391	-0.560252
Ciclev10032646m.g	scaffold_4:12320438-12324228	135.159	91.6694	-0.56014
Ciclev10004685m.g	scaffold_9:629682-634705	14.3712	9.74884	-0.559875
Ciclev10012731m.g	scaffold_6:25114212-25116604	17.724	12.0233	-0.559865
Ciclev10009207m.g	scaffold_1:14838663-14840710	88.8706	60.2896	-0.559798
-	scaffold_7:19858337-20044570	10.8361	7.35119	-0.559797
Ciclev10003019m.g	scaffold_5:40081126-40086638	0.43174	0.29292	-0.559653
Ciclev10028262m.g	scaffold_8:24521115-24524868	33.9729	23.0501	-0.559614
Ciclev10025395m.g	scaffold_7:15170987-15174087	44.9756	30.5182	-0.55947
Ciclev10016506m.g	scaffold_2:36319743-36321247	13.3721	9.07454	-0.559328
Ciclev10032757m.g	scaffold_4:11964849-11971089	34.5415	23.4492	-0.55879
Ciclev10031391m.g	scaffold_4:21172015-21177408	6.1277	4.16019	-0.558697
Ciclev10014272m.g	scaffold_2:12125405-12128792	0.968888	0.657834	-0.558606
Ciclev10025728m.g	scaffold_7:5035428-5038431	15.1106	10.2629	-0.558125
-	scaffold_3:2744117-2744770	13.3027	9.03549	-0.55805
Ciclev10012656m.g	scaffold_6:12350242-12352049	25.1964	17.1166	-0.557818
Ciclev10018739m.g	scaffold_3:2193102-2199392	19.5362	13.2716	-0.557809
Ciclev10012096m.g	scaffold_6:7519715-7525186	30.6096	20.7966	-0.557638

Ciclev10029365m.g	scaffold_8:4461644-4464561	220.399	149.748	-0.557578
Ciclev10012797m.g	scaffold_6:20270430-20274129	47.5138	32.2929	-0.55713
Ciclev10032268m.g	scaffold_4:2430023-2433146	65.051	44.226	-0.556677
Ciclev10003884m.g	scaffold_5:108928-112283	2.00923	1.36632	-0.556345
Ciclev10028092m.g	scaffold_8:22217855-22223396	36.5678	24.8677	-0.556302
Ciclev10014505m.g	scaffold_2:14270260-14274038	8.33158	5.66618	-0.556215
Ciclev10015084m.g	scaffold_2:23790786-23796225	11.3616	7.72746	-0.556094
Ciclev10022339m.g	scaffold_3:36096008-36099799	64.715	44.0159	-0.556076
Ciclev10027981m.g,Ciclev10028007m.g	scaffold_8:23272500-23285645	226.234	153.898	-0.555835
Ciclev10020064m.g	scaffold_3:39628209-39629734	71.4648	48.6184	-0.555731
-	scaffold_5:12360290-12360660	20.5824	14.0028	-0.555704
Ciclev10028340m.g	scaffold_8:4911564-4916480	69.8109	47.515	-0.55507
Ciclev10000392m.g	scaffold_5:34866518-34875003	6.72024	4.57507	-0.554717
Ciclev10017152m.g	scaffold_2:31801438-31804455	0.232444	0.158273	-0.554473
Ciclev10021716m.g	scaffold_3:41576812-41581730	9.42215	6.41715	-0.554123
Ciclev10009883m.g	scaffold_1:4622888-4624934	60.7055	41.3616	-0.553534
Ciclev10007967m.g	scaffold_1:28807628-28814574	54.5344	37.1614	-0.553365
Ciclev10017296m.g	scaffold_2:32252308-32254004	57.9736	39.5192	-0.552843
Ciclev10003286m.g	scaffold_5:17805800-17809117	1.40618	0.958832	-0.552431
Ciclev10003097m.g	scaffold_5:32546088-32865063	5.99903	4.092	-0.551924
Ciclev10032931m.g	scaffold_4:3193713-3196564	1159.95	791.295	-0.551772
Ciclev10008156m.g	scaffold_1:6644076-6646249	7.72692	5.2721	-0.551515
Ciclev10009015m.g	scaffold_1:8822833-8826308	70.7136	48.2485	-0.551505
Ciclev10020094m.g	scaffold_3:42935982-42941151	22.9564	15.6655	-0.551307
Ciclev10005844m.g	scaffold_9:1850387-1853743	43.4489	29.6513	-0.551226
Ciclev10018308m.g	scaffold_2:23330798-23334408	11.0962	7.57258	-0.551209
Ciclev10030703m.g	scaffold_4:21524992-21532610	1.17191	0.799831	-0.551091
Ciclev10029956m.g	scaffold_8:22461635-22462367	3.22496	2.2013	-0.550923
Ciclev10008932m.g	scaffold_1:4541675-4543982	10.3976	7.09895	-0.550567
Ciclev10001823m.g	scaffold_5:37449700-37453928	37.1004	25.3331	-0.550411
Ciclev10031034m.g	scaffold_4:12333307-12336127	20.4222	13.9497	-0.549904
Ciclev10028403m.g	scaffold_8:21293714-21298947	2.99192	2.04416	-0.549565
Ciclev10023782m.g	scaffold_3:40078083-40089151	226.22	154.597	-0.549212
Ciclev10000706m.g	scaffold_5:36090414-36095771	27.388	18.7182	-0.549101
Ciclev10023183m.g	scaffold_3:25085744-25086451	191.025	130.564	-0.549004
Ciclev10012570m.g	scaffold_6:20651753-20654352	28.2761	19.3275	-0.548927
Ciclev10032367m.g	scaffold_4:8400377-8404853	47.4867	32.4596	-0.548878
Ciclev10015661m.g	scaffold_2:32541377-32543726	135.396	92.5615	-0.548703
Ciclev10026754m.g	scaffold_7:7700119-7702124	21.4653	14.6771	-0.548445
Ciclev10030967m.g	scaffold_4:22486236-22491982	12.8643	8.79667	-0.548342
Ciclev10012552m.g	scaffold_6:23364302-23366061	77.1901	52.7956	-0.547996
Ciclev10028468m.g	scaffold_8:20765981-20774157	39.1644	26.7912	-0.547783
-	scaffold_7:13769733-13772358	141.637	96.892	-0.547749
Ciclev10026609m.g	scaffold_7:18539346-18543298	569.573	389.637	-0.547749
Ciclev10019453m.g	scaffold_3:43966964-43972591	13.0562	8.93159	-0.547746
Ciclev10024227m.g	scaffold_3:46103661-46106597	25.8031	17.6532	-0.54761
Ciclev10022425m.g	scaffold_3:50850795-50851962	11.4764	7.85192	-0.547547
Ciclev10011576m.g	scaffold_6:8029487-8035109	41.5146	28.4058	-0.547432
Ciclev10018554m.g	scaffold_3:43006063-43010078	1.97754	1.35349	-0.547024
Ciclev10032676m.g	scaffold_4:25022320-25025779	26.1167	17.8776	-0.546818
Ciclev10022935m.g	scaffold_3:32071460-32076377	42.9813	29.4231	-0.54676
Ciclev10016636m.g	scaffold_2:31534414-31538268	46.6964	31.9667	-0.546741
Ciclev10021083m.g	scaffold_3:40130520-40133133	48.4987	33.209	-0.546371
Ciclev10002925m.g	scaffold_5:34520419-34522509	740.054	506.817	-0.546164
Ciclev10022229m.g	scaffold_3:4004110-4008140	24.5528	16.8148	-0.546155

Ciclev10006377m.g	scaffold_9:16838237-16846412	2.1464	1.47041	-0.545706
Ciclev10005352m.g	scaffold_9:12839344-12844087	52.1875	35.7651	-0.54515
Ciclev10012243m.g	scaffold_6:14794014-14796956	32.8413	22.5075	-0.545106
Ciclev10020406m.g	scaffold_3:44866567-44869888	16.9133	11.5945	-0.544718
Ciclev10026686m.g	scaffold_7:4214422-4217517	62.4551	42.8159	-0.544673
Ciclev10004300m.g	scaffold_9:12845640-12852274	5.11176	3.50501	-0.544401
Ciclev10022137m.g	scaffold_3:39601127-39604586	66.7874	45.7947	-0.544396
Ciclev10022710m.g	scaffold_3:1184671-1187488	115.307	79.1096	-0.543549
Ciclev10022413m.g	scaffold_3:2798608-2801856	37.7606	25.9096	-0.543396
-	scaffold_3:28065477-28197319	31.9356	21.9214	-0.542826
Ciclev10026591m.g	scaffold_7:11131540-11135740	41.5969	28.5567	-0.542646
-	scaffold_7:11707510-11707894	7.05341	4.84241	-0.542597
Ciclev10001479m.g	scaffold_5:35159689-35164384	54.9888	37.7526	-0.54256
Ciclev10021191m.g	scaffold_3:2102635-2106125	23.4921	16.1315	-0.542297
Ciclev10027611m.g	scaffold_7:20278338-20285118	0.902876	0.620115	-0.541993
Ciclev10016520m.g	scaffold_2:8027601-8030614	6.67922	4.58785	-0.541862
Ciclev10019379m.g	scaffold_3:2330116-2334317	3.14668	2.16171	-0.541658
Ciclev10012281m.g	scaffold_6:24742938-24746629	8.58567	5.8987	-0.541534
Ciclev10011289m.g	scaffold_6:22334900-22338038	9.93933	6.82934	-0.541404
Ciclev10020458m.g	scaffold_3:39365661-39369964	54.1586	37.216	-0.541266
Ciclev10011053m.g	scaffold_6:14918021-15036696	4.74034	3.25826	-0.540889
Ciclev10011926m.g	scaffold_6:23319091-23323666	27.1321	18.6527	-0.540615
Ciclev10029225m.g	scaffold_8:7886182-7889942	85.4713	58.7608	-0.540587
Ciclev10028945m.g	scaffold_8:21325199-21327234	2.59585	1.78473	-0.540505
Ciclev10014260m.g	scaffold_2:25765570-25768934	1.43823	0.988892	-0.540413
Ciclev10023549m.g	scaffold_3:3745688-3747485	4.17719	2.87226	-0.540343
Ciclev10009926m.g	scaffold_1:22451735-22454183	128.18	88.1597	-0.539981
Ciclev10017429m.g	scaffold_2:28786443-28788097	96.6496	66.4938	-0.539545
Ciclev10015347m.g	scaffold_2:11353148-11358883	35.4503	24.39	-0.539509
Ciclev10031313m.g	scaffold_4:18874265-18878127	4.20463	2.89503	-0.5384
Ciclev10017489m.g	scaffold_2:7935487-7939189	10.5739	7.28098	-0.538305
Ciclev10029337m.g	scaffold_8:10525313-10527112	73.7262	50.7835	-0.537818
Ciclev10026644m.g	scaffold_7:2469747-2471188	18.7273	12.8996	-0.537816
Ciclev10005906m.g	scaffold_9:28796583-28801400	47.0289	32.4093	-0.53714
Ciclev10016443m.g	scaffold_2:347633-354348	23.0777	15.9082	-0.536726
Ciclev10024941m.g	scaffold_7:41476-45638	2.98948	2.0609	-0.536618
Ciclev10033051m.g	scaffold_4:11203437-11207093	26.7563	18.4528	-0.536039
Ciclev10032244m.g	scaffold_4:24612462-24616531	51.4642	35.4959	-0.535916
Ciclev10029115m.g	scaffold_8:23480482-23484398	230.344	158.894	-0.53572
Ciclev10029501m.g	scaffold_8:18120878-18123765	197.201	136.064	-0.535382
Ciclev10012128m.g	scaffold_6:9922495-9927448	38.0427	26.2506	-0.535269
Ciclev10031396m.g	scaffold_4:2404352-2407969	28.8316	19.8957	-0.535194
Ciclev10019778m.g	scaffold_3:44999604-45002966	5.18601	3.57886	-0.535127
Ciclev10000306m.g	scaffold_5:42841158-42844776	32.2402	22.2564	-0.534645
Ciclev10029291m.g	scaffold_8:24431063-24433341	376.783	260.116	-0.534579
Ciclev10023400m.g	scaffold_3:40042527-40046590	11.0502	7.62913	-0.534477
Ciclev10004985m.g	scaffold_9:1676852-1678628	11.4444	7.90249	-0.534266
Ciclev10006072m.g	scaffold_9:29605463-29607371	107.378	74.1632	-0.533926
Ciclev10030216m.g	scaffold_8:7699848-7862562	2.07381	1.43282	-0.53343
Ciclev10015580m.g	scaffold_2:22344915-22350916	12.397	8.56602	-0.533293
Ciclev10030490m.g	scaffold_4:17040648-17047269	8.77565	6.06452	-0.533113
-	scaffold_2:27825723-27829604	12.8678	8.89519	-0.532668
Ciclev10015128m.g,Ciclev10016515m.g	scaffold_2:21506878-21515192	59.9592	41.4514	-0.53256
Ciclev10000841m.g	scaffold_5:40534832-40539098	13.6048	9.4066	-0.532366
Ciclev10032238m.g	scaffold_4:21443802-21447097	85.7819	59.3145	-0.532289

Ciclev10004090m.g	scaffold_5:43147647-43148472	0.473844	0.32771	-0.531992
Ciclev10016586m.g	scaffold_2:34699890-34702062	98.5393	68.151	-0.531964
Ciclev10024649m.g	scaffold_3:2793364-2795811	137.473	95.1123	-0.531448
Ciclev10017054m.g	scaffold_2:29054437-29056627	103.707	71.7599	-0.53126
Ciclev10033200m.g	scaffold_4:17925034-17925722	93.9554	65.0169	-0.531162
Ciclev10000459m.g	scaffold_5:40007932-40012974	28.3691	19.6354	-0.530865
Ciclev10014880m.g	scaffold_2:36223845-36231874	2.59275	1.79486	-0.530611
Ciclev10003068m.g	scaffold_5:19692394-19713573	0.715885	0.495622	-0.530487
Ciclev10000488m.g	scaffold_5:27589631-27592701	4.10507	2.84247	-0.53026
Ciclev10020804m.g	scaffold_3:6484725-6486543	12.7075	8.79987	-0.530128
Ciclev10006127m.g	scaffold_9:4702401-4704790	213.826	148.085	-0.530012
Ciclev10029509m.g	scaffold_8:19348442-19349831	3.73114	2.58473	-0.529605
Ciclev10009295m.g	scaffold_1:22628770-22631628	26.2009	18.1531	-0.5294
Ciclev10025151m.g	scaffold_7:13128486-13174611	0.843852	0.584682	-0.529336
Ciclev10022809m.g	scaffold_3:3623693-3625333	324.331	224.816	-0.52872
-	scaffold_9:13697905-13698416	32.0283	22.2058	-0.528412
Ciclev10008867m.g	scaffold_1:3609305-3615258	65.6954	45.5502	-0.528335
Ciclev10020054m.g	scaffold_3:48739712-48746026	61.0029	42.3222	-0.527464
Ciclev10019196m.g	scaffold_3:5368644-5374936	50.8527	35.293	-0.526941
Ciclev10001527m.g	scaffold_5:38120562-38123758	33.5441	23.2878	-0.526487
Ciclev10010130m.g	scaffold_1:27286386-27290085	0.459757	0.319192	-0.526448
Ciclev10003038m.g	scaffold_5:42405270-42408220	0.828707	0.575381	-0.526344
Ciclev10008092m.g	scaffold_1:26132645-26136128	7.45203	5.17446	-0.526225
Ciclev10013102m.g	scaffold_6:16199887-16202624	15.9761	11.0934	-0.526214
Ciclev10020605m.g	scaffold_3:44286095-44289701	1.44692	1.00471	-0.526211
Ciclev10011742m.g	scaffold_6:12594909-12596760	37.3816	25.957	-0.526203
Ciclev10018720m.g	scaffold_3:2202467-2207986	19.3464	13.4362	-0.525937
Ciclev10006214m.g	scaffold_9:16867068-16867712	52.6287	36.5617	-0.525517
Ciclev10011941m.g,Ciclev10013819m.g	scaffold_6:4067938-4074052	43.9125	30.5101	-0.525346
Ciclev10028110m.g	scaffold_8:138021-146284	45.4622	31.5907	-0.525167
Ciclev10030198m.g,Ciclev10030386m.g	scaffold_8:22575352-22578572	1109.35	771.038	-0.524838
Ciclev10015541m.g	scaffold_2:26890334-26894409	44.0758	30.6382	-0.524654
Ciclev10003927m.g	scaffold_5:39112746-39118165	64.6099	44.9124	-0.524643
Ciclev10001634m.g	scaffold_5:424432-427199	12.9563	9.00766	-0.524429
Ciclev10001758m.g	scaffold_5:36616631-36620799	21.5888	15.0141	-0.523965
Ciclev10019340m.g	scaffold_3:3654153-3657882	2.18784	1.52177	-0.52376
Ciclev10026097m.g	scaffold_7:4667364-4670010	82.2195	57.1973	-0.523533
Ciclev10032896m.g	scaffold_4:23754484-23755585	236.131	164.276	-0.523469
Ciclev10030016m.g	scaffold_8:4006362-4025709	3.37878	2.35094	-0.523267
Ciclev10020553m.g,Ciclev10024062m.g,Ciclev10024495m.g	scaffold_3:39181844-39318533	16.2697	11.3214	-0.523141
Ciclev10028346m.g	scaffold_8:23634092-23639792	39.9146	27.7771	-0.523019
Ciclev10008911m.g	scaffold_1:26810794-26816497	43.6396	30.3708	-0.522956
Ciclev10016959m.g	scaffold_2:30886468-30892663	52.5741	36.5892	-0.522935
Ciclev10021697m.g	scaffold_3:743413-746343	79.2112	55.1347	-0.522743
Ciclev10002915m.g	scaffold_5:42495261-42497381	40.6992	28.3314	-0.522602
Ciclev10029230m.g	scaffold_8:6554901-6560226	21.1266	14.709	-0.522361
Ciclev10022818m.g	scaffold_3:42303544-42306061	51.5076	35.8694	-0.522034
Ciclev10015859m.g	scaffold_2:31402026-31406669	13.7166	9.55378	-0.521776
-	scaffold_5:2530471-2531216	5.37966	3.74798	-0.521401
Ciclev10004235m.g	scaffold_9:4436333-4441963	6.01462	4.19042	-0.52138
Ciclev10016330m.g	scaffold_2:14209265-14216265	16.3089	11.3652	-0.521035
Ciclev10009612m.g	scaffold_1:4662424-4666008	3.62642	2.52724	-0.520986

Ciclev10031934m.g	scaffold_4:25260540-25262610	2.3095	1.60988	-0.520628
Ciclev10018726m.g	scaffold_3:6678996-6684558	15.7877	11.0073	-0.520339
Ciclev10005677m.g	scaffold_9:28396494-28399986	7.33644	5.11588	-0.520097
Ciclev10028459m.g	scaffold_8:23365480-23369843	20.4095	14.2333	-0.519973
Ciclev10025268m.g	scaffold_7:112220-116430	13.0898	9.12988	-0.51978
Ciclev10012189m.g	scaffold_6:25295298-25299298	23.8967	16.6678	-0.519752
Ciclev10022575m.g	scaffold_3:44291485-44293163	21.371	14.9064	-0.519722
Ciclev10013048m.g	scaffold_6:11835445-11837145	17.1159	11.9385	-0.519719
Ciclev10022993m.g	scaffold_3:42229223-42233850	352.839	246.225	-0.519036
Ciclev10026516m.g	scaffold_7:6135530-6137411	155.007	108.183	-0.518858
Ciclev10021922m.g	scaffold_3:26526424-26531920	11.186	7.80725	-0.518811
Ciclev10011595m.g	scaffold_6:16798475-16801193	4.00055	2.79233	-0.518731
Ciclev10031031m.g	scaffold_4:3308158-3312351	83.957	58.6122	-0.51845
Ciclev10000484m.g	scaffold_5:11336688-11353674	9.69241	6.76711	-0.518315
Ciclev10014533m.g	scaffold_2:31603940-31623860	1.16808	0.815551	-0.518296
Ciclev10032673m.g	scaffold_4:1175518-1180789	15.8536	11.0728	-0.517793
Ciclev10024856m.g	scaffold_7:13230970-13233708	2.07963	1.45274	-0.517551
Ciclev10005675m.g	scaffold_9:8376736-8414192	41.9827	29.3327	-0.517288
Ciclev10031153m.g	scaffold_4:24596903-24602264	9.10028	6.35838	-0.517253
Ciclev10025251m.g	scaffold_7:995484-1000767	38.6314	27.0039	-0.516606
Ciclev10033072m.g	scaffold_4:2096561-2133384	75.1067	52.5063	-0.516453
Ciclev10002817m.g	scaffold_5:41651170-41652391	393.753	275.352	-0.516013
Ciclev10032851m.g	scaffold_4:715596-718039	56.7108	39.6605	-0.515923
Ciclev10001582m.g,Ciclev10003487m.g	scaffold_5:24058278-24064704	19.1933	13.4231	-0.515883
Ciclev10033481m.g	scaffold_4:7055580-7057811	4.56615	3.19416	-0.515544
Ciclev10026699m.g	scaffold_7:2583838-2584921	42.2548	29.5589	-0.515521
Ciclev10019792m.g	scaffold_3:41716542-41719311	1.59925	1.11907	-0.515088
Ciclev10032790m.g	scaffold_4:21310164-21312801	29.8774	20.912	-0.514723
[truncated: 1,247,293 more chars]
