# Supplementary material for: Genome resequencing and transcriptome profiling reveal structural diversity and expression patterns of constitutive disease resistance genes in Huanglongbing-tolerant Poncirus trifoliata and its hybrids
Source: Hortic Res. 2017 Nov 15;4:17064–. doi: 10.1038/hortres.2017.64 (PMC5686287; doi:10.1038/hortres.2017.64)
Supplement: Supplementary Table 2 [file hortres201764-s2.pdf]

Supplementry Table 2: List of the total number of identified citrus CDR genes along with predicted peptide lengths, molecular masses and pI values.

CDR gene	Predicted number of amino acid	Molecular mass (Kilo Dalton(kDa)	pI
CcCDR1	480	51.7	9.7
CcCDR2	184	20.4	5.9
CcCDR3	422	45.2	7.5
CcCDR4	422	45.2	7.5
CcCDR5	428	45.9	6.6
CcCDR6	428	45.9	6.2
CcCDR7	399	43.1	5.6
CcCDR8	304	32.6	4.8
CcCDR9	343	36.8	4.9
CsCDR1	480	51.7	9.7
CsCDR2	290	31.3	4.8
CsCDR3	345	37.1	5.3
CsCDR4	423	45.3	6.2
CsCDR5	394	42.2	6.6
CsCDR6	374	40.2	5
CsCDR7	427	45.8	5.4
CsCDR8	374	40.2	4.9
