# Supplementary material for: Genome resequencing and transcriptome profiling reveal structural diversity and expression patterns of constitutive disease resistance genes in Huanglongbing-tolerant Poncirus trifoliata and its hybrids
Source: Hortic Res. 2017 Nov 15;4:17064–. doi: 10.1038/hortres.2017.64 (PMC5686287; doi:10.1038/hortres.2017.64)
Supplement: Supplementary Table 3 [file hortres201764-s3.pdf]

Supplementry Table 3: Subcellular localization of predicted CDR proteins are presented based on CELLO online tool.

#SeqNO.	Comp.Result	Di-pep.Result	part-Comp.Result	chemo-type.Result	Neighbor	extra	outer	peri	inner	cyto	#Most-likely-Location	#SeqName
#1	OuterMembrane	OuterMembrane	OuterMembrane	Periplasmic	OuterMembrane	0.603	3.431	0.848	0.049	0.069	OuterMembrane	CcCDR1_(Ciclev10019938m)
#2	OuterMembrane	Cytoplasmic	OuterMembrane	Extracellular	OuterMembrane	0.928	2.268	0.452	0.407	0.945	OuterMembrane	CcCDR2_(Ciclev10033351m)
#3	OuterMembrane	OuterMembrane	OuterMembrane	Extracellular	OuterMembrane	1.608	3.161	0.176	0.036	0.019	OuterMembrane	CcCDR3_(Ciclev10031641m)
#4	OuterMembrane	OuterMembrane	OuterMembrane	Extracellular	OuterMembrane	1.7	3.075	0.161	0.041	0.023	OuterMembrane	CcCDR4_(Ciclev10031639m)
#5	OuterMembrane	OuterMembrane	OuterMembrane	Extracellular	OuterMembrane	1.6	3.172	0.193	0.02	0.015	OuterMembrane	CcCDR5_(Ciclev10020249m)
#6	OuterMembrane	OuterMembrane	OuterMembrane	Extracellular	OuterMembrane	1.639	3.132	0.194	0.021	0.015	OuterMembrane	CcCDR6_(Ciclev10020250m)
#7	OuterMembrane	Extracellular	OuterMembrane	Extracellular	OuterMembrane	1.736	2.746	0.371	0.072	0.075	OuterMembrane	CcCDR7_(Ciclev10008524m)
#8	Extracellular	Extracellular	Extracellular	Extracellular	Extracellular	3.022	0.855	0.411	0.209	0.503	Extracellular	CcCDR8_(Ciclev10010208m)
#9	Extracellular	Extracellular	Extracellular	Extracellular	Extracellular	3.046	1.347	0.298	0.101	0.209	Extracellular	CcCDR9_(Ciclev10008826m)
#10	OuterMembrane	OuterMembrane	OuterMembrane	Periplasmic	OuterMembrane	0.657	3.35	0.86	0.055	0.078	OuterMembrane	CsCDR1_(orange1.1g040810m)
#11	OuterMembrane	Cytoplasmic	OuterMembrane	Periplasmic	OuterMembrane	0.373	2.527	1.057	0.517	0.526	OuterMembrane	CsCDR2_(orange1.1g046014m)
#12	OuterMembrane	Extracellular	OuterMembrane	Extracellular	OuterMembrane	1.55	3.168	0.211	0.044	0.027	OuterMembrane	CsCDR3_(orange1.1g019179m)
#13	OuterMembrane	OuterMembrane	OuterMembrane	Extracellular	OuterMembrane	1.475	3.325	0.149	0.032	0.019	OuterMembrane	CsCDR4_(orange1.1g014537m)
#14	OuterMembrane	Extracellular	OuterMembrane	Extracellular	OuterMembrane	1.554	3.223	0.167	0.034	0.022	OuterMembrane	CsCDR5_(orange1.1g016180m)
#15	OuterMembrane	OuterMembrane	OuterMembrane	Extracellular	OuterMembrane	1.467	3.269	0.197	0.043	0.023	OuterMembrane	CsCDR6_(orange1.1g017265m)
#16	OuterMembrane	OuterMembrane	OuterMembrane	Extracellular	OuterMembrane	1.542	3.243	0.186	0.015	0.014	OuterMembrane	CsCDR7_(orange1.1g040562m)
#17	OuterMembrane	Extracellular	OuterMembrane	Extracellular	OuterMembrane	1.839	2.553	0.385	0.082	0.141	OuterMembrane	CsCDR8_(orange1.1g048503m)
