# Supplementary material for: Genome resequencing and transcriptome profiling reveal structural diversity and expression patterns of constitutive disease resistance genes in Huanglongbing-tolerant Poncirus trifoliata and its hybrids
Source: Hortic Res. 2017 Nov 15;4:17064–. doi: 10.1038/hortres.2017.64 (PMC5686287; doi:10.1038/hortres.2017.64)
Supplement: Supplementary Table 4 [file hortres201764-s4.pdf]

Supplementry Table 4: Description of total number of variants (including SNPs and indel) and their respective position (5’UTR, exon, intron, and 3’UTR) in Poncirus and Poncirus hybrids with respect to *Citrus CDR* genes.

S.No.	CDR gene in Poncirus	Citrus CDR orthologs	CDR gene name	Genotype	Total No. of SNPs	Type of SNPs					Position of SNPs			
					variations	Transition	Transversion	Substitution	Deletion	Insersion	5'UTR	Exon	Intron	3'UTR
1	*PtCDR1*	orange1.1g040810m	*CsCDR1*	DPI	20	15	4	1				20		
				FD	20	15	4	1				20		
				US-897	_									
				US-812	_									
2	*PtCDR2*	orange1.1g019179m	*CsCDR3*	DPI	71	33	25	7	2 (one each in 5'and 3'UTR)	4 (two each in 5' and 3'UTR)	10	34	9	18
				FD	56	27	21	3	1 (5'UTR)	4 (two each in exon and 3'UTR)	10	25	5	16
				US-897	7	3	4				4	3		
				US-812	12	4	5		2 (3'UTR)	1 (5'UTR)	4	1		7
3	*PtCDR3*	orange1.1g040562m	*CsCDR7*	DPI	30	14	16					22		8
				FD	22	10	12					16		6
				US-897	23	12	11					18		5
				US-812	_									
4	*PtCDR4*	Ciclev10020250m	*CcCDR6*	DPI	27	13	13		1 (exon)			27		
				FD	26	12	14					26		
				US-897	_							_		
				US-812	4	1	3					27		
5	*PtCDR5*	Ciclev10020249m	*CcCDR5*	DPI	24	10	14					24		
				FD	21	8	13					21		
				US-897	_							_		
				US-812	28	10	14	4				28		
6	*PtCDR6*	Ciclev10019938m	*CcCDR1*	DPI	33	17	9	2	3 (3'UTR)	2 (one each in exon and 3'UTR)	_	17	_	16
				FD	31	18	6	3	3 (3'UTR)	1 (3'UTR)	_	15	_	16
				US-897	11	8	3				_	11	_	
				US-812	5	4	1				_	5	_	

S.No.	CDR gene in Poncirus	Citrus CDR orthologs	CDR gene name	Genotype	Total No. of SNPs	Type of SNPs					Position of SNPs			
					variations	Transition	Transversion	Substitution	Deletion	Insersion	5'UTR	Exon	Intron	3'UTR
7	*PtCDR7*	Ciclev10008826m	*CcCDR9*	DPI	3	2	1					3		
				FD	3	2	1					3		
				US-897	_									
				US-812	7	3	4					7		
8	*PtCDR8*	Ciclev10031641m	*CcCDR3*	DPI	24	10	11			3 (exon)		21		3
				FD	22	8	11			3 (exon)		20		2
				US-897	7	3	1			3 (exon)		7		
				US-812	9	4	1			4 (exon)		9		
