# Supplementary material for: Genome resequencing and transcriptome profiling reveal structural diversity and expression patterns of constitutive disease resistance genes in Huanglongbing-tolerant Poncirus trifoliata and its hybrids
Source: Hortic Res. 2017 Nov 15;4:17064–. doi: 10.1038/hortres.2017.64 (PMC5686287; doi:10.1038/hortres.2017.64)
Supplement: Supplementary Table 5 [file hortres201764-s5.pdf]

Supplementry Table 5: Consensus sequence for each *PtCDR* gene was retrieved after mapping the paired-

CDR orthologs in Poncirus-hybrids and Poncirus genotypes	Gene sequence
PtCDR1_812	CGCACCCGCGCGGAGCAAACCCGATCCGTGAAGCGGCCAAATCATAAACAACCCGGAAGCCCTGTTGCTGAATATTTC<br>CAATTATCGACAACCCGCTCATGGTTCCTGCAAATGCAAAGCAAAATGTTCCGCTACTATCCACGGGTATTAGATAATTA<br>GTCGCCGGCAACGACACATCWGCACCTCTGAAATGCAACACCACCGTGGGCACCTTCACTTCCGTCTTTCCCGACAAAT<br>CGAAGCACGTATCAAACAGCGAAAAGTCTGGCGCACGCTTCAAGGAGGATGCTCCAGCTCGGAAAGCATCACGCAAT<br>GCGATGTAAGCGGGTCGGGTCAAACGGGTCACAGACGTACCCGAGTCTATGATGACCCCACCGTTACCGGCCGGATCG<br>AGTTTGAACAAAGAAGCAGTGATGCCGCGGACATGAGCACCGCCGACGCTGATTCCAACTAGTTCAACATAGTAGAAT<br>GTGTCGAGCTTAGGGTTTGCGAGTAACGGAGTGAACCGGGCGGTTCGTGAAACGGCCGAGTCACCGAATACCATCGA<br>TGAAGGTTTAGCAGAAGTGGATCGGTCCACTAAACAGTAAGAAAACTTACGGTTGAACCGGCGACCGGTTTGWRTTG<br>GGAATGACAACCTCCCACGACCGAGCCCCAATAAGCCCGCAGCAGCAACGAAGAGGCCCTCATTGTCGTGTCCACAAC<br>CGAGGGCCACGCGCGCAACTCTGGTGCCMCGAAACGTCAGYGTTTCAGTGGAAAAATCACCGACGGTGATAGAACCG<br>TCTCCGTARGAGACTTGGTAAAGACACGTGTTCCTGCGGTTACAGCCAGAAGAGTCGAGCTTGCGGCACAAAGGGGAS<br>CGACAGGGTACGGTAGCAAACGAGCGGGACTTGGCAGGGTCGAAGACAGGGTCGGTTTGAGAGTAGCATTTCTTGCA<br>YGGAGCGCACTGTATCCAAACGACGTCGCTACCGGTGTCTAAGACCATGTAGACATACCTAGGGGGTGTGCCAACTCC<br>GAGGCGCGTGAAGTACTCGCCGCTGCCTTGTGCGAGYCCTGATATGACGGAGCTACTAAAGCCTCCGTTGGCACGCCC<br>GCGRGAGCGGTTGCGCGGGGGARCACGAACGGCAGATTCAGCGAAGGCGKTTAAGGATTTGACACGTAAGACGTCGC<br>GTTGGATGCGGAGGTTGAAGAGGTGCTCTGGAGTTCTGTTGAAGGACAGAGAGTCTACGTGATGTAAACGTAGGGAR<br>AGCGAGGATTCTGCGTCRGGTGCTGGTAGAGGGAGYGAAGATTCGGATTCGGAWACGGATACGGATTCAGGCCAAG<br>AGAGAGTGGATGGGGTAGGGAGAGAGTTGAGGACGAAGGTTTGATACTGGAGAGAGGCAGCGGCGGTGAAGAAGA<br>AGGAGAAGAGAAGGAGAAGATGGTTTCTTGCTTTCCCTTCCAT
PtCDR1_897	CCGCGCGGAGCAAACCCGATCCGTGAAGCGGCCAAATCATAAACAACCCGGAAGCCCTGTTGCTGAATATTTCCAATT<br>ATCGACAACCCGCTCATGGTTCCTGCAAATGCAAAGCAAAATGTTCCGCTACTATCCACGGGTATTAGATAATTAGTCG<br>CCGGCAACGACACATCTGCACCTCTGAAATGCAACACCACCGTGGGCACCTTCACTTCCGTCTTTCCCGACAAATCGAA<br>GCACGTATCAAACAGCGAAAAGTCTGGCGCACGCTTCAAGGAGGATGCTCCAGCTCGGAAAGCATCACGCAATGCGAT<br>GTAAGCGGGTCGGGTCAAACGGGTCACAGACGTACCCGAGTCTATGATGACCCCACCGTTACCGGCCGGATCGAGTTT<br>GAACAAAGAAGCAGTGATGCCGCGGACATGAGCACCGCCGACGCTGATTCCAACTAGTTCAACATAGTAGAATGTGTC<br>GAGCTTAGGGTTTGCGAGTAACGGAGTGAACCGGGCGGTTCGTGAAACGGCCGAGTCACCGAATACCATCGATGAAG<br>GTTTAGCAGAAGTGGATCGGTCCACTAAACAGTAAGAAAACTTACGGTTGAACCGGCGACCGGTTTGWRTTGGRAAT<br>GACAACCTCCCACGACCGAGCCCCAATAAGCCCGCAGCAGCAACGAAGAGGCCCTCATTGTCGTGTCCACAACCGAGG<br>GCCACGCGCGCAACTCTGGTGCCACGAAACGTCAGCGTTTCAGTGGAAAAATCACCGACGGTGATAGAACCGTCTCCG<br>TAGGAGACTTGGTAAAGACACGTGTTCCTGCGGTTACAGCCAGAAGAGTCGAGCTTGCGGCACAAAGGGGACCGACA<br>GGGTACGGTAGCAAACGAGCGGGACTTGGCAGGGTCGAAGACAGGGTCGGTTTGAGAGTAGCATTTCTTGCACGGAG<br>CGCACTGTATCCAAACGACGTCGCTACCGGTGTCTAAGACCATGTAGACATACCTAGGGGGTGTGCCAACTCCGAGGC<br>GCGTGAAGTACTCGCCGCTGCCTTGTGCGAGCCCTGATATGACGGAGCTACTAAAGCCTCCGTTGGCACGCCCGCGGG<br>AGCGGTTGCGCGGGGGAACACGAACGGCAGATTCAGCGAAGGCGGTTAAGGATTTGACACGTAAGACGTCGCGTTG<br>GATGCGGAGGTTGAAGAGGTGCTCTGGAGTTCTGTTGAAGGACAGAGAGTCTACGTGATGTAAACGTAGGGAGAGCG<br>AGGATTCTGCGTCAGGTGCTGGTAGAGGGAGTGAAGATTCGGATTCGGAAACGGATACGGATTCAGGCCAAGAGAGA<br>GTGGATGGGGTAGGGAGAGAGTTAAGGACGAAGGTTTGATACTGGAGAGAGGCAGCGGCGGTGAAGAAGAAGGAG<br>AAGAGAAGGAGAAGATGGTTTCTTGCTTTCCCT

PtCDR1_DPI-50-7	GCACCCGCGCGGAGCAAACCCGATCCGTGAAGCGGCCAAATCATAAACAACCCGGAAGCCCTGTTGCTGAATATTTCC<br>AATTATCGACAACCCGCTCATGGTTCCTGCAAATGCAAAGCAAAATGTTCCGCTACTATCCACGGGTATTAGATAATTA<br>GTCGCCGGCAACGACACATCAGCACCTCTGAAATGCAACACCACCGTGGGCACCTTCACTTCCGTCTTTCCCGACAAAT<br>CGAAGCACGTATCAAACAGCGAAAAGTCTGGCGCACGCTTCAAGGAGGATGCTCCAGCTCGGAAAGCATCACGCAGT<br>GCGATGTAAGCGGGTCGGGTCAAACGAGTCACACGTACCCGAGTCTATGATGACCCCACCGTTACCGGCCGGATCGAG<br>TTTGAACAAAGAAGCAGTGATGCCACGGACATGAGCACCACCGACGCTGATTCCAACTAGTTCAACATAGTAGAATGT<br>GTCGAGCTTAGGGTTTGTGAGTAACGGAGTGAACCGGGCGGTTCGTGAAACGGCCGAGTCRCCGAATACCATCGATG<br>AAGGTTTAGCAGAAGTGGATCGGTCCACTAAACAGTAAGAAAACTTACGGTTGAACCGGCGACCGGTTTGTATTGGGA<br>ATGACAACCTCCCACGACCGAGCCCCAATAAGCCCGCAGCAGCAACGAAGAGGCCCTCATTGTCGTGTCCACAACCGA<br>GGGCCACGCGCGCAACTCTGGTGCCCCGAAACGTCAGTGTTTCAGTGGAAAAATCACCGACGGTGATAGAACCGTCTC<br>CGTAAGAGACTTGGTAAAGACACGTGTTCCTGCGGTTACAGCCAGAAGAGTCGAGCTTGCGGCACAAAGGGGAGCGA<br>CAGGGTACGGTAGCAAACGAGCGGGACTTGGCAGGGTCGAAGACAGGGTCGGTTTGAGAGTAGCATTTCTTGCATGG<br>AGCGCACTGTATCCAAACGACGTCGCTACCGGTGTCTAAGACCATGTAGACATACCTAGGGGGTGTGCCAACTCCGAG<br>GCGCGTGAAGTACTCGCCGCTGCCTTGTGCGAGTCCTGATATGACGGAGCTACTAAAGCCTCCGTTGGCACGCCCGCG<br>AGAGCGGTTGCGCGGGGGAGCACGAACGGCAGATTCAGCGAAGGCGTTTAAGGATTTGACACGTAAGACGTCGCGTT<br>GGATGCGGAGGTTGAAGAGGTGCTCTGGAGTTCTGTTGAAGGACAGAGAGTCTACGTGATGTAAACGTAGGGAAAGC<br>GAGGATTCTGCGTCGGGTGCTGGTAGAGGGAGCGAAGATTCGGATTCGGATACGGATACGGATTCAGGCCAAGAGA<br>GAGTGGATGGGGTAGGGAGAGAGTTGAGGACGAAGGTTTGATACTGGAGAGAGGCAGCGGCGGTGAAGAAGAAGG<br>AGAAGAGAAGGAGAAGATGGTTTCTTGCTTTCCCTTCCAT
PtCDR1_FD	GCGCACCCGCGCGGAGCAAACCCGATCCGTGAAGCGGCCAAATCATAAACAACCCGGAAGCCCTGTTGCTGAATATTT<br>CCAATTATCGACAACCCGCTCATGGTTCCTGCAAATGCAAAGCAAAATGTTCCGCTACTATCCACGGGTATTAGATAATT<br>AGTCGCCGGCAACGACACATCAGCACCTCTGAAATGCAACACCACCGTGGGCACCTTCACTTCCGTCTTTCCCGACAAA<br>TCGAAGCACGTATCAAACAGCGAAAAGTCTGGCGCACGCTTCAAGGAGGATGCTCCAGCTCGGAAAGCATCACGCAGT<br>GCGATGTAAGCGGGTCGGGTCAAACGAGTCGGATCGAGTTTGAACAAAGAAGCAGTGATGCCACGGACATGAGCACC<br>ACCGACGCTGATTCCAACTAGTTCAACATAGTAGAATGTGTCGAGCTTAGGGTTTGTGAGTAACGGAGTGAACCGGGC<br>GGTTCGTGAAACGGCCGAGTCACCGAATACCATCGATGAAGGTTTAGCAGAAGTGGATCGGTCCACTAAACAGTAAGA<br>AAACTTACGGTTGAACCGGCGACCGGTTTGTATTGGGAATGACAACCTCCCACGACCGAGCCCCAATAAGCCCGCAGC<br>AGCAACGAAGAGGCCCTCATTGTCGTGTCCACAACCGAGGGCCACGCGCGCAACTCTGGTGCCCCGAAACGTCAGTGT<br>TTCAGTGGAAAAATCACCGACGGTGATAGAACCGTCTCCGTAAGAGACTTGGTAAAGACACGTGTTCCTGCGGTTACA<br>GCCAGAAGAGTCGAGCTTGCGGCACAAAGGGGAGCGACAGGGTACGGTAGCAAACGAGCGGGACTTGGCAGGGTCG<br>AAGACAGGGTCGGTTTGAGAGTAGCATTTCTTGCATGGAGCGCACTGTATCCAAACGACGTCGCTACCGGTGTCTAAG<br>ACCATGTAGACATACCTAGGGGGTGTGCCAACTCCGAGGCGCGTGAAGTACTCRCCGCTRCCTTGTGCGAGTCCTGAT<br>ATGACGGAGCTACTAAAGCCTCCGTTGGCACGCCCGCGAGAGCGGTTGCGCGGGGGAGCACGAACGGCAGATTCAGC<br>GAAGGCGGTTAAGGATTTGACACGTAAGACGTCGCGTTGGATGCGGAGGTTGAAGAGGTGCTCTGGAGTTTTGTTGA<br>AGGACAGAGAGTCTACGTGATGTAAACGTAGGGAGAGCGAGGATTCTGCGTCGGGTGCTGGTAGAGGGAGCGAAGA<br>TTCGGATTCGGNNNNNNATNNNNNNACGGATACGGATTCAGGCCAAGAGAGAGTGGATGGGGTAGGGAGAGAGTT<br>GAGGACGAAGGTTTGATACTGGAGAGAGGCAGCGGCGGTGAAGAAGAAGGAGAAGAGAAGGAGAAGATGGTTTCT<br>TGCTTTCCCTTCCAT

PtCDR2_812	ATTGTCACCCAAGTTGCAACAATAAGATAAGGCTGGCGCTAACTTATATTTAACATAACTTGCGCCTCTCACAGCAATAG<br>TACCCGGTCCAATCAGTAGTACTGTTGCTGTGAGAGGGCGTAAGTTACGYTAAACGTAACTTAGGCCCTCCCATAAGAT<br>AATTGTCACTCTAACAAAAGCTGACTACATRAGAAAGTTTCATTTTCTTGGAAGCTCATYTTCTTTMCTTTTTTTTTTTTT<br>GGTCTTCTATTCAAAATTAATTAAATTTTKCAACTTATCTCTCTGTGTCCTCCTCTATATAAGCATAGACTCGTTGCCACTT<br>TCTTATTATCCTCTCAATCGTCTCAATCACTTTAATCAGAACTCATCAATATCTTCATCAAAAGCTTCCCAAGCTGATATAA<br>TACCCAATAACGCGAACTATCTCATACGTATATCAATTGGTACTCCCCCTACTGAAAGACTAGCTGTTGCTGATACAGGA<br>AGTGACCTCATATGGACGCAGTGCGAGCCTTGCCCACCATCACAATGTTACATGCAGGACTCTCCACTTTTTGATCCTAA<br>AATGTCATCTACTTACAAATCTCTTCCATGCTCSTCAAGCCAATGTGCATCTCTTARCCAAAAATCTTGCTCTGGGGTAAA<br>TTGTCAATACTCGGTCTCTTATGGAGATGGATCCTTCTCAAATGGCAATCTTGCTACCGAAACTGTTACTTTGGGTTCAA<br>CAACAGGACAGGCCGTGGCTCTGCCTGGAATAACCTTTGGTTGCGGAACAAACAATGGTGGCTTGTTTAATTCAAAAA<br>CAACCGGAATTGTTGGCCTTGGAGGCGGCGATATTTCACTTATTTCTCAAATGCGAACTACAATTGCTGGTAAATTCTCC<br>TATTGTTTGGTTCCAGYAASCTCAACCAAAATCAATTTTGGCACCAATGGAATTGTTTCAGGTCCAGGAGTGGTTTCTAC<br>TCCCTTGACTAAAGCCAAAACCTTTTATGTTCTCACAATTGATGCAATCAGTGTAGGAAACCAAAAACTAGGAGTATCG<br>ACTCCAGACATTGTAATCGATTCTGGTACAACACTTACCTTCTTACCACAAGGTTATAACTCAAACTTGTTGTCAGTAATG<br>TCTAGTATGATTGAGGCACAACCTGTTGCAGACCCTACAGGATCACTTGAACTTTGCTACAGTTTTAATTCACTATCTCA<br>AGTTCCTGAAGTTACAATACATTTCAGAGGTGCAGATAGATTGCACCAAGCAGTAATTAATTGGGAAGCTTAAGTGATA<br>GTCTCCACTCTACTGGAGTTTTGTCGAGTGACTTTGAAGCCACTTTCTTCGGCCGACCGACCGATTTCAAATTCTCATGC<br>AACTGGGGKSCCCCCCCCGGGGGGGGGGGGGTGGGCGAGTCTGTGAAGCTTGGTTTTAATTTTGTAGTTCGCAAGATT<br>GTATCATCCTACGAACGATTAGCTCGTTTTACTGTGTTTGGTTTTGAATCAATATGGCATAAATTAAGATATTTGATGTTA<br>TTTGTGTCACTGTGTCACTCAAAAGATCGTCAATCACTAAATCAACACAAAACACAATATCTGATCCATTATTAATTAAC<br>AATCATCTATCAGTCGCCAACAGCTACGATATATATTCCACTCAACACTTTCTAACAGTTTGACATAATTAACTGCTTCAC<br>TGTATTTTCTTTTGTATTAATTACWAAAGTATAAGCTAAC
PtCDR2_897	GTTAGCTTATACTTTAGTAATTAATACAAAAGAAAATACAGTGAAGCAGTTAATTATGTCAAACTGTTAGAAAGTGTTG<br>AGTGGAATATATATCGTAGCTGTTGGCGACTRATAGATGATTGTTAATTAATAATGRAYCAGATATTGTGTTTTGTGTTA<br>GATTTAGTGATTGAYGATCTTTTSAGTGACACAGTGACACAAATAACATCAAATATCTTAATTTATGCCATATTGATTCAA<br>AACCAAACACAGTAAAACGAGCTAATCGTTCGTAGGATGATACAATCTTGCGAACTACAAAATTAAAACCAAGCTTCAC<br>AGACTCGCCCACCCCCCCCCCCCCAATTTCACATCTGCACCTCTGAAATGTATTGTAACTTCAGGAACTTGAGATAGTG<br>AATTAAAACTGTAGCAAAGTTCAAGTGATCCTGTAGGGTCTGCAACAGGTTGTGCCTCAATCATACTAGACATTACTGA<br>CAACAAGTTTGAGTTATAACCTTGTGGTAAGAAGGTAAGTGTTGTACCAGAATCGATTACAATGTCTGGAGTCGATACT<br>CCTAGTCTTTGGTTTCCTACACTGATTGCATCAATTGTGAGAACATAAAAGGTTTTGGCTTTAGTCAAGGGAGTAGAAA<br>CCACTCCTGGACCTGAAACAATTCCATTGGTGCCAAAATTGATTTTGGTTGAGCTTACTGGAACCAAACAATAGGAGAA<br>TTTACCAGCAATTGTAGTTCGCATTTGAGAAATAAGTGAAATATCGCCGCCTCCAAGGCCAACAATTCCGGTTGTTTTTG<br>AATTAAACAAGCCACCATTGTTTGTTCCGCAACCAAAGGTTATTCCAGGCAGAGCCACGGCCTGTCCTGTTGTTGAACC<br>CAAAGTAACAGTTTCGGTAGCAAGATTGCCATTTGAGAAGGATCCATCTCCATAAGAGACCGAGTATTGACAATTTACC<br>CCAGAGCAAGATTTTTGGTTAAGAGATGCACATTGGCTTGACGAGCATGGAAGAGATTTGTAAGTAGATGACATTTTA<br>GGATCAAAAAGTGGAGAGTCCTGCATGTAACATTGTGATGGTGGGCAAGGCTCGCACTGCGTCCATATGAGGTCACTT<br>CCTGTATCAGCAACAGCTAGTCTTTCAGTAGGGGGAGTACCAATTGATATACGTATGAGATAGTTCGCGTTATTGGGTA<br>TTATATCAGCTTGGGAAGCTTTTGATGAAGATATTGATGAGTTCTGATTAAAGTGATTGAGACGATTGAGAGAACGAGT<br>CAAAGCATCTCTCAATCGTTGGTAGGGAGTTTCACTGGAGTTGTAAAAGGGAGACTTGGGAGAGTCACGGTGGATTAG<br>CTCAACACTGAAACCTCCAGTTTGAGCTTCTATAGGAGAGACGACATAAAAACAAAGAAAGAAGAGAATGAATACACA<br>ACTCAAGAAGGTCGCCATAATCAAAGCTGTGAGTGAGATAATAAGAAAGTGGCAACGAGTCTATGCTTATATAGAGGA<br>GGACACGGAGAGATAATTTGAAAAATTTAATTAATTTTCAATAGAAGACCAAAAATTAAAAAAAAAGGAAAGAAAATG<br>AGCTTCCAAGTTCCAAGAAAATGTAACTTTCTCGCATAGTAAGCTTTTGTTAGAGTGACAACCATCTTATGGGAGGGCC<br>TAAGTTACGTTGAACGTAACTTACGCCCTCTCACAACAACAATAAAGTTATGTTAAATATAAGTTAGCGCCAGCCTTATC<br>TTATTGTTGCAACTTGGGTGACAAT

PtCDR2_DPI-50-7	TTGAGAGGAATATATATTGTAGCTGTTGGCGACTAATAGATGATTGTTAATTATTAATGAATCAGATATTGTGTTTTGTG<br>TTAGATTTAGTGATTGATGATCTTTTCAGTGACACAGCGACACAAATAACATCAAATATGTTAATTTATAGTTTTATACCA<br>TATCGATTCAAAACCAAATACAGTAAAACGAACTAATCGTTCGTAGGATGATACAATCTTGCGAAATACAAAATTTAATT<br>ACTGCTTGGTGCAGTCAGTCGGTCTAAATGACACTGTTTGTTGTTCTATGTCATAGCCGACTAAAAAGTTAGTCTGCATT<br>ATATTACCATAAAGTTGCAGACTATTGGTGGTACCTTTAAAAACAGAACATACAATATCCTTCGAAACCTTCACAAAAAA<br>GTTGGAACGACTCAATTTCACATCTGCACCTCTGAAATGTATTGTAACTTCAGGAACTTGAGATAGTGAATTAAAACTGT<br>AGCAAAGTTCAAATGATCCTTTGGGGTCTGCAACAGGTTGTGCCTCAATCATACTGGACATTACTGACAACAATTTTGA<br>GTTATAACCTTGTGGCAATAAGGTAAATGTTGTACCAGAATCGATTACAATGTCTCTTCCTGGAGTCGATCCAGACATA<br>ACTTCTAGTCTTTGGTTTCCTACACTGATTGCATCAATTGTGAGAACATAAGAGGTTTTGGCTTTAGTCAAGGGAGTAGA<br>AACCACTCCTGGGCCTGAAACAATTCCATTGGTTCCAAAATTGATTTTGGTTGAGGTTGCTGGAACCAAACAATAGGAG<br>AATTTACCAGCAATTGTAGTTCGCATTTGAGAAATAAGTGAAATATCGCCGCCTCCAAGGCCAACAATTCCGGTTGTTTT<br>TGAATTAAACAAGCCACCATTGTTTGTTCCGCAACCAAAGGTTATTCCAGGCAGAGCCACGGCCTGTCCTGTTGTTGAA<br>CCCAAAGTAACAGTTTCGGTAGCAAGATTGCCATTTGAGAAGGATCCATCTCCATAAGAGACCGAGTATTGACAATTTA<br>CCCCAGAGCAAGATTTTTGGCTAAGAGATGCACATTGGCTTGAGGAGCATGGAAGAGATTTGTAAGTAGATGACTTTT<br>TAGGATCAAAAAGTGGAGAGTCCTGCATGTAACATTTTGATGGTGGGCAAGGCTCGCACTGCGTCCATATGAGGTCAC<br>TTCCTGTATCAGCAACAGCTAGTATTTCAGTAGCGGGAGTACCAATTGATATACGTATGAGATATTCTCCCCTATTGGGT<br>ATTATATCAGCTTGGGAAGCGGCTGATGAAGATATTGATGAGTTCTGATTAAAGTGATTAAGACGATTGAGAGAACGA<br>GTCAAAGCATTTCTCAATCGTTGGTAGGGAGTTTCACTGGAGTTGTAAAAGGGAGACTTGGGAGAGTCACGGTGGATT<br>AGCTCAACACTGAAACCTCCAGTTTGAGCTTCTATTGGAGAGACAACATAAAAACAAAGAAAGAAGAGAATGAATGCA<br>CAACTCAAGAAGGTTGCCATAATCAAAGCTGTGATTGAGGTAATAAGAAAGTGGCAACGAGTCTATGCTTATATAGAG<br>GAGGACACAGAGAGATAAGTTGCAAAATTTAATTAATTTTGAATAGAAGACCAAAAAAAAAAAAAAAAAGGAAAGAAA<br>ATGAGCTTCCAAGAAAATGAAACTTTCTTGTGTAGTAAGCTTTTGTTATAGTGACAATTATCTTATGGGAGGGCCTAAGT<br>TACGTTYAACGTAAYTTAYGCCCTCTCACA
PtCDR2_FD	TGAGAGGAATATATATTGTAGCTGTTGGCGACTAATAGATGATTGTTAATTATTAATGAATCAGATATTGTGTTTTGTGT<br>TAGATTTAGTGATTGATGATCTTTTCAGTGACACAGCGACACAAATAAYATCAAATATGTTAATTTATAGTTTTATACCAT<br>ATCGATTCAAAACCAAATACAGTAAAACGAACTAATCGTTCGTAGGATGATACAATCTTGCGAAATACAAAATGAACAT<br>ACAATATCCTTCGAAACCTTCACAAAAAAGTTGGAACGACTCAATTTCACATCTGCACCTCTGAAATGTATTGTAACTTC<br>AGGAACTTGAGATAGTGAATTAAAACTGTAGCAAAGTTCAAATGATCCTTTGGGGTCTGCAACAGGTTGTGCCTCAATC<br>ATACTGGACATTACTGACAACAATTTTGAGTTATAACCTTGTGGCAATAAGGTAAATGTTGTACCAGAATCGATTACAAT<br>GTCTCTTCCTGGAGTCGATCCAGACATAACTTCTAGTCTTTGGTTTCCTACACTGATTGCATCAATTGTGAGAACATAAG<br>AGGTTTTGGCTTTAGTCAAGGGAGTAGAAACCACTCCTGGGCCTGAAACAATTCCATTGGTTCCAAAATTGATTTTGGT<br>TGAGGTTGCTGGAACCAAACAATAGGAGAATTTACCAGCAATTGTAGTTCGCATTTGAGAAATAAGTGAAATATCGCC<br>GCCTCCAAGGCCAACAATTCCGGTTGTTTTTGAATTAAACAAGCCACCATTGTTTGTTCCGCAACCAAAGGTTATTCCAG<br>GCAGAGCCACGGCCTGTCCTGTTGTTGAACCCAAAGTAACAGTTTCGGTAGCAAGATTGCCATTTGAGAAGGATCCATC<br>TCCATAAGAGACCGAGTATTGACAATTTACCCCAGAGCAAGRTTTTTGGCTAAGAGATGCACATTGGCTTGAGGAGCAT<br>GGAAGAGATTTGTAAGTAGATGACTTTTTAGGATCAAAAAGTGGAGAGTCCTGCATGTAACATTTTGATGGTGGGCAA<br>GGCTCGCACTGCGTCCATATGAGGTCACTTCCTGTATCAGCAACAGCTAGTATTTCAGTAGCGGGAGTACCAATTGATA<br>TACGTATGAGATATTCTCCGCTATTGGGTATTATATCAGCTTGGGAAGCGGCTGATGAAGATATTGATGAGTTCTGATT<br>AAAGTGATTAAGACGATTGAGAGAACGAGTCAAAGCAGTTGTAAAAGGGAGACTTGGGAGAGTCACGGTGGATTAGC<br>TCAACACTGAAACCTCCAGTTTGAGCTTCTATTGGAGAGACAACATAAAAACAAAGAAAGAAGAGAATGAATGCACAA<br>CTCAAGAAGGTTGCCATAATCAAAGCTGTGATTGAGATAATAAGAAAGTGGCAACGAGTCTATGCTTATATAGAGGAG<br>GACACAGAGAGATAAGTTGCAAAATTTAATTAATTTTGAATAGAAGACCAAAAAAAAAAAAAAAGGAAAGAAAATGAG<br>CTTCCAAGAAAATGAAACTTTCTTGTGTAGTAAGCTTTTGTTATAGTGACAATTATCTTATGGGAGGGCCTAAGTTACGT<br>TCAACGTAAYTTAYGCCCTCTCACA

PtCDR3_812	ATGGAAACCTTCTTGAGTTGTGCATTCCTTCTCTTCTTCCTTTGTCTCTCAGTTCTCTCTCCAGCAGAGGCTCAAACTGTA<br>GGGTTCAGTGTTGAGCTGATCCACCGTGACTCTCCAAAGTCTCCCTTCTATAACCCTAATGAAACTCCCTACCAACGGTT<br>GAGAAATGCTTTGAATCGTTCTGCCAATCGTCTCCGTCACTTCAATAAAAACTCATCAGTCTCTTCATCAAAAGTTTCCCA<br>AGCTGATATAATACCTAATGTTGGTGASTATCTCATTCGTATATCAATCGGTACTCCCCCTGTTGAAATACTAGCTGTGG<br>CTGACACAGGAAGTGATCTCATATGGACACAGTGTCAACCCTGCCCACCATCACAATGTTACAAGCAAGACAATCCACT<br>TTTTGATCCCCAAAGGTCTTCCACTTACAAATATCTTTCTTGCTCATCGRGCCAATGCGCACCACCGATCAAAGATTCTTG<br>CTCTGCGGAGGGGAATTGCAAATATTCAGTCTCCTACGGAGACGATTCTTTCTCAAATGGCGATCTAGCTACCGAAACT<br>GTTACTGTGGGATCAACATCCGGACAGGCCGTGGCTCTGCCAGAAATAGTCTTCGGCTGTGGAACAAAAAATGGTGGG<br>AAATTTAATTCAAAAACTGMTGGCATCGTTGGSCTTGGGGGAGGTGATGSTTCCCTTATWTCTCAAATGAAAACTACA<br>ATTGCAGGTAAATTCTCCTACTGCTTGGTGCAACAAAGCTCAACCAAAATCAATTTTGGCACCAATGGAATTGTTTCAGG<br>TTCTGGCGTAGTTTCTACTCCCTTGCTTGCTAAGAATCCCAAAACCTTCTATTCCCTCACACTCGATGCAATCAGTGTAGG<br>AGATCAAAGACTAGGTGTTATATCAGGATCAAATCCGGGAGGAGACATTGTAATTGATTCCGGTACTACACTTACCTAT<br>TTACCACCAGCTTATGCCTCGAAATTGCTTTCAGTAATGTCCAGTATGATAGCGGCACAACCCGTTGAAGGGCCATATG<br>ATCTCTGCTACAGTATTAGTTCACGACCTCGATTTCCTGAAGTCACAATACATTTCAGRGATGCAGATGTGAAGTTGAGC<br>MCTTCCAACGTTTTTATGAATATTTCAGAAGACCTTGTGTGCTCAGTTTTTAATGCTCGCGATGACATTCCACTTTATGG<br>TAACATTATGCAGACCAACTTTTTGATCGGCTATGACATTGAGGGACGCACAGTTTCGTTTAAACCAACTGACTGCTCCA<br>AGCAGTAG
PtCDR3_897	ATGGCAACCTTCTTGAGTTGTGCATTCATTCTCTTCTTCCTTTGTCTCTCAGTTCTCTCTCCAGCAGAGGCTCAAACTGGA<br>GGATTCAGTGTTGAGCTGATCCACCGTGACTCTCCAAAGTCTCCCTTCTATAACCCTAATGAAACTCCCTACCAACGGTT<br>GAGAAATGCTTTGAATCGTTCTGCCAATCGTCTCAGTCASTTCAATAAAAACTCATCAGTCTCTTCATCAAAAGTTTCCCA<br>AGCTGATATAATACCTAATGTTGGTGACTATCTCATTCGTATATCAATTGGTACTCCCCCTGTTGAAATACTAGCTGTGG<br>CTGACACAGGAAGTGATCTCATATGGACACAGTGTCAACCCTGCCCACCATCACAATGTTACAAGCAAGACAATCCACT<br>TTTTGATCCTCAAAAGTCTTCCACTTACAAATATCTTTCTTGCTCATCGGGCCAATGCGCACCACCGATCAAAGATTCTTG<br>CTCTGCGGAGGGGAATTGCAAATATTCAGTCTCCTACGGAGACGATTCTTTCTCAAATGGCGATCTAGCTACCGAAACT<br>GTTACTGTGGGATCAACATCCGGACAGGCCGTGGCTCTGCCAGAAATAGTCTTCGGCTGTGGAACAAAAAATGGTGGG<br>AAATTTAATTCAAAAACTGCTGGCATCGTTGGCCTTGGGGGAGGTGATGGTTCCCTTATTTCTCAAATGAAAACTACAA<br>TTGCAGGTAAATTCTCCTACTGCTTGGTGCAACAAAGCTCAACCAAAATCAATTTTGGCACCAATGGAATTGTTTCAGGT<br>TCTGGCGTAGTTTCTACTCCCTTGCTTGCTAAGAATCCCAAAACCTTCTATTACCTCACACTCGATGCAATCAGTGTAGGA<br>GATCAAAGATTAGGTGTTATATCAGGAWCAACTACGGGTGGAGACTATGTCCTTGATTCTGGTACTACACTAACCTATT<br>TACCACCAGCTTATGCCTCAAAATTGCTTTCAGTAATGTCCAGTATGATAGCGGGACAACCCGTTGAAGGGCCATATGA<br>TCTYTGCTACAGWATTAGTTCACGACCTCAATTTCCTGAAGTCACAATACATTTCAGRGATGCAGATGTGAAGTTGAGC<br>CCTTCCAACKTTTTTATGAATATTTCAGAAGACCTTGTGTGCTCAGTTTTTAATGCTCGCGATGACATTCCACTTTATGGT<br>AACATTATGCAGACCAACTTTTTGATCGGCTATGACATTGAGGGACGCACAGTTTCGTTTAAACCAACTGACTGCTCCAA<br>GCAGTAG
PtCDR3_DPI-50-7	ATGGCAACCTTCTTGAGTTGTGCATTCATTCTCTTCTTCCTTTGTCTCTCAGTTCTCTCTCCAGCAGAGGCTCAAACTGGA<br>GGATTCAGTGTTGAGCTGATCCACCGTGACTCTCCAAAGTCTCCCTTCTATAACCCTAATGAAACTCCCTACCAACGGTT<br>GAGAAATGCTTTGAATCGTTCTGCCAATCGTCTCAGTCGGTTCAATAAAAACTCATCACTCTCTTCATCAAAAGTTTCCCA<br>AGCTGATTTAATACCTAATGTTGGTGACTATCTCATTCGTGTATCAATCGGTACTCCCCCTGTTGAAATACTAACTGTGG<br>CTGACACAGGAAGTGATCTCATATGGACACAGTGTCAACCCTGCCCACCATCACAATGTTACAAGCAAGACAATCCACT<br>TTTTGATCCTCAAAAGTCTTCCACTTACAAATATCTTTCTTGCTCATCGGGCCAATGCGCACCACCGATCAAAGATTCTTG<br>CTCTGCGGAGGGGAATTGCAAATATTCAGTCTCCTACGGAGACGATTCTTTCTCAAATGGCGATCTAGCTACCGAAACT<br>GTTACTGTGGGATCAACATCCGGACAGGCCGTGGCTCTGCCAGAAATAGTCTTCGGCTGTGGAACAAAAAATGGTGGG<br>AAATTTAATTCAAAAACTGCTGGCATCGTTGGCCTTGGGGGAGGTGATGGTTCCCTTATTTCTCAAATGAAAACTACAA<br>TTGCAGGTAAATTCTCCTACTGCTTGGTGCAACAAAGCTCAACCAAAATCAATTTTGGCACCAATGGAATTGTTTCAGGT<br>TCTGGCGTAGTTTCTACTCCCTTGCTTGTTAAGAATCCCAAAACCTTCTATTACCTCACACTCGATGCAATCAGTGTAGGA<br>GATCAAAGACTAGGTGTTATATCAGGATCAACTCCGGGAGGAGACATTGTAATTGATTCTGGTACTACACTTACCTATT<br>TACCACCAGCTTATGCCTCAAAATTGCTTTCAGTAATGTCCAGTATGATAGCGGGACAACCCGTTGAAGGGCCACATGA<br>TCTCTGCTACAGAATTAGTTCACGACCTCAATTTCCTGAAGTCACAATACATTTCAGGGATGCAGATGTGAAGTTGAGCC<br>CTTCCAACRTTTTTATGAATATTTCAGAAGACCTTGTGTGCTCAGTTTTTAATGCTCGCGATGACATTCCACTTTATGGTA<br>ACATTATGCAGACCAACTTTTTGATCGGCTATGACATTGAGGGACGCACAGTTTCGTTTAAACCAACTGACTGCTCCAA<br>GCAGTAG

PtCDR3_FD	ATGGCAACCTTCTTGAGTTGTGCATTCATTCTCTTCTTCCTTTGTCTCTCAGTTCTCTCTCCAGCAGAGGCTCAAACTGGA<br>GGATTCAGTGTTGAGCTGATCCACCGTGACTCTCCAAAGTCTCCCTTCTATAACCCTAATGAAACTCCCTACCAACGGTT<br>GAGAAATGCTTTGAATCGTTCTGCCAATCGTCTCAGTCGGTTCAATAAAAACTCATCACTCTCTTCATCAAAAGTTTCCCA<br>AGCTGATTTAATACCTAATGTTGGTGACTATCTCATTCGTGTATCAATCGGTACTCCCCCTGTTGAAATACTAACTGTGG<br>CTGACACAGGAAGTGATCTCATATGGACACAGTGTCAACCCTGCCCACCATCACAATGTTACAAGCAAGACAATCCACT<br>TTTTGATCCTCAAAAGTCTTCCACTTACAAATATCTTTCTTGCTCATCGGGCCAATGCGCACCACCGATCAAAGATTCTTG<br>CTCTGCGGAGGGGAATTGCAAATATTCAGTCTCCTACGGAGACGATTCTTTCTCAAATGGCGATCTAGCTACCGAAACT<br>GTTACTGTGGGATCAACATCCGGACAGGCCGTGGCTCTGCCAGAAATAGTCTTCGGCTGTGGAACAAAAAATGGTGGG<br>AAATTTAATTCAAAAACTGCTGGCATCGTTGGCCTTGGGGGAGGTGATGGTTCCCTTATTTCTCAAATGAAAACTACAA<br>TTGCAGGTAAATTCTCCTACTGCTTGGTGCAACAAAGCTCAACCAAAATCAATTTTGGCACCAATGGAATTGTTTCAGGT<br>TCTGGCGTAGTTTCTACTCCCTTGCTTATTAGTTCACGACCTCAATTTCCTGAAGTCACAATACATTTCAGGGATGCAGAT<br>GTGAAGTTGAGCCCTTCCAACGTTTTTATGAATATTTCAGAAGACCTTGTGTGCTCAGTTTTTAATGCTCGCGATGACAT<br>TCCACTTTATGGTAACATTATGCAGACCAACTTTTTGATCGGCTATGACATTGAGGGACGCACAGTTTCGTTTAAACCAA<br>CTGACTGCTCCAAGCAGTAG
PtCDR4_812	ATAATATTTATTGCAACTTATAAAAATTGAAACTAAAAACGCACATCCAACTTAATTACTACTGCTTGGAGCAGTCAGTT<br>GGTTTAAACGAAACYGTGCGTCCCTCAATGTCATAGCCGATCAAAAAGTTGGTCTGCATAATGTTACCATAAACTGGCG<br>TGCCATTGCTAGCACTAAAAACTGAACACACAAGGTCTTCTGAAATWTTCATAAAAAYGTTGGAAGGGCTCAACTTCAC<br>ATCTGCATCCCTGAAATGTATTGTGACTTCAGGAAATTGAGGTCGTGAACTAATTCTGTAGCAGAGATCATATGGCCCT<br>TCAACGGGTTGTSCCGCTATCATACTGGACATTACTGAAAGCAATTTTGAGGCATAAGCTGGTGGTAAATAGGTTAGTG<br>TAGTACCAGAATCAATTACAATGTCTCCTCCCGGAGTTGATCCTGATATAACACCTAGTCTTTGATCTCCTACACTGATTG<br>CATCGAGTGTGAGGTAATAGAAGGTTTTGGGATTCTTAACAAGCAAGGGAGTAGAAACTACGCCAGAACCTGAAACA<br>ATTCCATTGGTGCCAAAATTGATTTTGGTTGAGCTTTGTTGCACCAAGCAGTAGGAGAATTTACCTGCAATTGTAGTTCT<br>TATTTGAGAAATAAGGGAAGCATCACCTCCCCCAAGGCCAACGATGCCATCAGTTTTTGAATTAAATCTCCCACTATTTT<br>TTGTTCCGCAGCCAAAGACTATTTCTGGCAGAGCTACGGCCTGTCCGGATGTTGATCCCAAAGTAACAGTTTCGGTAGC<br>TAGATCGCCATTTGAGAAAGATTCTTGGTCTGCGTAGCCGACTAAATATTTGCAATTCCCCTCCCCAGAGCAAGATTCTT<br>TGATCGGTGGTGCGCATTGGCYCGATGAGCAAGAAAGATATTTGTAAGTGGAAGACTTTTGAGGATCAAAAAGTGGAT<br>TGTCTTGCTTGTAACATTGTGATGGTGGGCAGGGTTGACACTGTGTCCATATGAGATCACTTCCTGTGTCAGCCACAGC<br>TAGTATTTCAACAGGGGGAGTACCGATTGATATACGAATGAGATAGTCACCAACATTAGGTATTATATCAGCTTGGGAA<br>ACTTTTGATGAAGAGACTGATGAGTTTTTATTGAACTGACTGAGACGATTGGCAGAACGATTCAAAGCATTTCTCAACC<br>GTTGGTAGGGAGTTTCATTAGGGTTATAGAAGGGAGACTTTGGAGAGTCACGGTGGATCAGCTCAACACTGAACCCTC<br>CAGTTTGAGCCTCTGCTGGAGAGATAACTGAGAGACAAAGGAAGAAGAGAATGAATGCACAACTCAAGAAGGTTGCC<br>AT
PtCDR4_897	ATAATATTTATTGCAACTTATAAAAAWTGAAACTAAAAACGCACATCCAACTTAATTACTACTGCTTGGAGCAGTCAGT<br>TGGTTTAAACGAAACYGTGCGTCCCTCAATGTCATAGCCGATCAAAAAGTTGGTCTGCATAATGTTACCATAAAGTGGC<br>GTGCCATTGCTAGCACTAAAAACTGAACACACAAGGTCTTCTGAAATTTTCATAAAAATGTTGGAAGGGCTCAACTTCA<br>CATCTGCATCCCTGAAATGTATTGTGACTTCAGGAAATTGAGGTCGTGAACTAATTCTGTAGCARAGATCATATGGCCC<br>TTCAACRGGTTGTSCCGCTATCATACTGGACATTACTGAAAGCAATKTTGAGGCATAAGCTGGTGGTAAATAGGTTAGT<br>GTAGTACCAGAATCAATTACAATGTCTCCTCCCGGAGTTGATCCTGATATAACACCTAGTCTTTGATCTCCTACACTGATT<br>GCATCGAGTGTGAGGTAATAGAAGGTTTTGGGATTCTTAACAAGCAAGGGAGTAGAAACTACGCCAGAACCTGAAAC<br>AATTCCATTGGTGCCAAAATTGATTTTGGTTGAGCTTTGTTGCACCAAGCAGTAGGAGAATTTACCTGCAATTGTAGTTC<br>TTATTTGAGAAATAAGGGAAGCATCACCTCCCCCAAGGCCAACGATGCCATCAGTTTTTGAATTAAATCTCCCACTATTT<br>TTTGTTCCGCAGCCAAAGACTATTTCTGGCAGAGCCACGGCCTGTCCGGATGTTGATCCCAAAGTAACAGTTTCGGTAG<br>CTAGATCGCCATTTGAGAAAGATTCTTGGTCTCCGTAGCGGACTAAATATTTGCAATTCCCCTCCCCAGAGCAAGATTCT<br>TTGATCGGTGGGGCGCATTGGCTCGATGAGCAAGAAAGATATTTGTAAGTGGAAGACTTTTGAGGATCAAAAAGTGG<br>ATTGTCTTGCTTGTAACATTGTGATGGTGGGCAGGGTTGACACTGTGTCCATATGAGATCACTTCCTGTGTCAGCCACA<br>GCTAGTATTTCAACAGGGGGAGTACCGATTGATATACGAATGAGATAGTCACCAACATTAGGTATTATATCAGCTTGGG<br>AAACTTTTGMTGAAGAGACTGATGAGTTTTTATTGAAGTGACTGAGACGATTGGCAGAACGATTCAAAGCATTTCTCA<br>ACCGTTGGTAGGGAGTTTCATTAGGGTTATAGAAGGGAGACTTTGGAGAGTCACGGTGGATCAGCTCAACACTGAACC<br>CTCCAGTTTGAGCCTCTGCTKGAGAGATAACTGAGAGACAAAGGAAGAAGAGAATGAATGCACAACTCAAGAAGGTT<br>GCCAT

PtCDR4_DPI-50-7	ATAATATTTATTGCAACTTATAAAAATTGAAACTAAAAACGCACATCCAACTTAATTACTACTGCTTGGAGCAGTCAGTT<br>GGTTTAAACGAAACTGTGCGTCCCTCAATGTCATAGCCGATCAAAAAGTTGGTCTGCATAATGTTACCATAAAGTGGAA<br>TCGCGAGCATTAAAAACTGAGCACACAAGGTCTTCTGAAATATTCATAAAAATGTTGGAAGGGCTCAACTTCACATCTG<br>CATCCCTGAAATGTATTGTGACTTCAGGAAATTGAGGTCGTGAACTAATTCTGTAGCAGAGATCATATGGCCCTTCAAC<br>GGGTTGTCCCGCTATCATACTGGACATTACTGAAAGCAATTTTGAGGCATAAGCTGGTGGTAAATAGGTTAGTGTAGTA<br>CCAGAATCAATTACAATGTCTCCTCCCGGAGTTGATCCTGATATAACACCTAGTCTTTGATCTCCTACACTGATTGCATCG<br>AGTGTGAGGTAATAGAAGGTTTTGGGATTCTTAACAAGCAAGGGAGTAGAAACTACGCCAGAACCTGAAACAATTCCA<br>TTGGTGCCAAAATTGATTTTGGTTGAGCTTTGTTGCACCAAGCAGTAGGAGAATTTACCTGCAATTGTAGTTCTTATTTG<br>AGAAATAAGGGAAGCATCACCTCCCCCAAGGCCAACGATGCCATCAGTTTTTGAATTAAATTTCCCACCATTTTTTTTTC<br>CACAGCCGAAGACTATTTCTGGCAGAGCCACGGCCTGTCCAGATGTTGATCCCAAAGTAACAGTTTCGGTAGCTAGATC<br>GCCATTTGAGAAAGAATTGTCTCCGTAGTGGACTGAATATTTGCAATTCCCCTCCGCAGAGCAAGAATCTTTGATCGGT<br>GGTGCGCATTGGCCCGATGAGCAAGAAAGATATTTGTAAGTGGAAGACTTTTGAGGATCAAAAAGTGGATTGTCTTGC<br>TTGTAACATTGTGATGGTGGGCAGGGTTGACACTGTGTCCATATGAGATCACTTCCTGTGTCAGCCACAGCTAGTATTT<br>CAACAGGGGGAGTACCAATTGATATACGAATGAGATAGTCACCAACATTAGGTATTATATCAGCTTGGGAAACTTTTGA<br>TGAAGAGACTGATGAGTTTTTATTGAACTGACTGAGACGATTGGCAGAACGATTCAAAGCATTTCTCAACCGTTGGTAG<br>GGAGTTTCAATAGGGTTATAGAAGGGAGACTTTGGAGAGTCACGGTGGATCAGCTCAACACTGAACCCTCCAGTTTGG<br>GCCTCTGATGGAGAGATAACTGAGAGACAAAGGAAGAAGAGAATGAATGCACAACTCAAGAAGGTTGCCAT
PtCDR4_FD	ATAATATTTATTGCAACTTATAAAAATTGAAACTAAAAACGCACATCCAACTTAATTACTACTGCTTGGAGCAGTCAGTT<br>GGTTTAAACGAAACTGTGCGTCCCTCAATGTCATAGCCGATCAAAAAGTTGGTCTGCATAATGTTACCATAAAGTGGAG<br>CATTAAAAACTGAGCACACAAGGTCTTCTGAAATATTCATAAAAACGTTGGAAGGGCTCAACTTCACATCTGCATCCCT<br>GAAATGTATTGTGACTTCAGGAAATTGAGGTCGTGAACTAATTCTGTAGCAGAGATCATATGGCCCTTCAACGGGTTGT<br>CCCGCTATCATACTGGACATTACTGAAAGCAATTTTGAGGCATAAGCTGGTGGTAAATAGGTTAGTGTAGTACCAGAAT<br>CAATTACAATGTCTCCTCCCGGAGTTGATCCTGATATAACACCTAGTCTTTGATCTCCTACACTGATTGCATCGAGTGTG<br>AGGTAATAGAAGGTTTTGGGATTCTTAACAAGCAAGGGAGTAGAAACTACGCCAGAACCTGAAACAATTCCATTGGTG<br>CCAAAATTGATTTTGGTTGAGCTTTGTTGCACCAAGCAGTAGGAGAATTTACCTGCAATTGTAGTTTTGAATATTTGCAA<br>TTCCCCTCCGCAGAGCAAGAATCTTTGATCGGTGGTGCGCATTGGCCCGATGAGCAAGAAAGATATTTGTAAGTGGAA<br>GACTTTTGAGGATCAAAAAGTGGATTGTCTTGCTTGTAACATTGTGATGGTGGGCAGGGTTGACACTGTGTCCATATGA<br>GATCACTTCCTGTGTCAGCCACAGYTAGTATTTCAACAGGGGGAGTACCRATTGATAYACGAATGAGATAGTCACCAAC<br>ATTAGGTATTAWATCAGCTTGGGAAACTTTTGATGAAGAGASTGATGAGTTTTTATTGAACCGACTGAGACGATTGGC<br>AGAACGATTCAAAGCATTTCTCAACCGTTGGTAGGGAGTTTCATTAGGGTTATAGAAGGGAGACTTTGGAGAGTCACG<br>GTGGATCAGCTCAACACTGAACCCTCCAGTTTGAGCCTCTGATGGAGAGATAACTGAGAGACAAAGGAAGAAGAGAA<br>TGAATGCACAACTCAAGAAGGTTGCCAT
PtCDR5_812	GCAGTCAGTTGGTTTAAACGAAACCGTGCGTCCCTCAATGTCATAGCCGATCAAAAAGTTGGTCTGCATAATGTTACCA<br>TAAACTGGCGTGCCATTGCTAGCACTAAAAACTAAACACACAAGGTCTTCTGAAATATTCATAAAAAAGTTGGAAGGGC<br>TCAACTTTACATCTGCATCTCTGAAATGTATTGTGACTTCAGGAAATTGAGGTCGTGAACCTATACTGTAGCAAAGATCA<br>TATGGCCCTTCAACGGGTTGTGCCGCTATCATACTGGACATTACTGAAAGCAATKTTGMGGCATAAGCTGGTGGTAAA<br>TAGGTTAGTGTAGTACCAGAATCAATTACAATGTCTCCTCCCGGAGTTGATCCTGATATAACACCTAGTCTTTGATCTCC<br>TACACTGATTGCATCGAGTGTGAGGTAATAGAAGGTTTTGGGATTCTTAACAAGCAAGGGAGTAGAAACTACGCCAGA<br>ACCTGAAACAATTCCATTGGTGCCAAAATTGATTTTGGTTGAGCTTTGTTGCACCAAGCAGTAGGAGAATTTACCTGCA<br>ATTGTAGTTCTTATTTGAGAAATAAGGGAAGCATCACCTCCCCCAAGGCCAACGATGCCATCAGTTTTTGAATTAAATCT<br>CCCACCATTTTTTGTTCCGCAGCCAAAGACTATTTCTGGCAGAGCCACGGCCTGTCCGGATTTTGATCCCAAAGTAACAG<br>TTTCGGTAGCTAGATCGCCATTTGAGAAAGATTCTTGGTCTCCGTAGCGGACTAAATATTTGCAATTCCCCTCCCCCGAG<br>CAAGATTCTTTGATCGGTGGTGCGCATTGGCTCGATGAGCAAGAAAGATATTTGTAAGTGGAAGACTTTTGAGGATCA<br>AAAAGTGGATTGTCTTGCTTGTAACATTGTGATGGTGGGCAGGGTTGACACTGTGTCCATATGAGATCACTTCCTGTGT<br>CAGCCACAGCTAGTATTTCAACAGGGGGAGTACCGATTGATATACGAATGAGATAGTCACCAACATTAGGTATTATATC<br>AGCTTGGGAAACTTTTGMTGAAGAGACTGATGAGTTTTTATTGAASTGACTGAGACGATTGGCAGAACGATTCAAAGC<br>ATTTCTCAACCGTTGGTAGGGAGTTTCATTAGGGTTATAGAAGGGAGACTTTGGAGAGTCACGGTGGATCAGCTCAAC<br>ACTGAACCCTCCAGTTTGAGCCTCTGCTGGAGAGATAACTGAGAGACMAAGGAAGAAGAGAATGAATGCACAACTCA<br>AGAAGGTTGCCAT

PtCDR5_897	CTGCTTGGAGCAGTCAGTTGGTTTAAACGAAACCGTGCGTCCCTCAATGTCATAGCCGATCAAAAAGTTGGTCTGCATA<br>ATGTTACCATAAAGTGGCGTGCCATTGCTAGCACTAAAAACTGAACACACAAGGTCTTCTGAAATTTTCATAAAAATGTT<br>GGAAGGGCTCAACTTCACATCTGCATCCCTGAAATGTATTGTGACTTCAGGAAATTGAGGTCGTGAACTAATTCTGTAG<br>CAAAGATCATATGGCCCTTCAACAGGTTGTGCCGCTATCATACTGGACATTACTGAAAGCAATGTTGAGGCATAAGCTG<br>GTGGTAAATACCAGAATCAATTACAATGTCTCCTCCCGGAGTTGATCCTGATATAACACCTAGTCTTTGATCTCCTACAC<br>TGATTGCATCGAGTGTGAGGTAATAGAAGGTTTTGGGATTCTTAACAAGCAAGGGAGTAGAAACTACGCCAGAACCTG<br>AAACAATTCCATTGGTGCCAAAATTGATTTTGGTTGAGCTTTGTTGCACCAAGCAGTAGGAGAATTTACCTGCAATTGTA<br>GTTCTTATTTGAGAAATAAGGGAAGCATCACCTCCCCCAAGGCCAACGATGCCATCAGTTTTTGAATTAAATCTCCCACT<br>ATTTTTTGTTCCGCAGCCAAAGACTATTTCTGGCAGAGCCACGGCCTGTCCGGATGTTGATCCCAAAGTAACAGTTTCG<br>GTAGCTAGATCGCCATTTGAGAAAGATTCTTKGTCTCCGTAGCGGACTRAATATTTGCAAKTCCCCTCCSCAGAGCAAGA<br>WTCTTTGATCGGTGGTGCGCATTGGCTCGATGAGCAAGAAAGATATTTGTAAGTGGAAGACTTTTGAGGATCAAAAA<br>GTGGATTATCTTGCTTGTAACATTGTGATGGTGGGCAGGGTTGACACTGTGTCCATATGAGATCACTTCCTGTGTCAGC<br>CACAGCTAGTATTTCAACAGGGGGAGTACCGATTGATATACGAATGAGATAGTCACCAACATTAGGTATTATATCAGCT<br>TGGGAAACTTTTGATGAAGAGACTGATGAGTTTTTATTGAAGTGACTGAGACGATTGGCAGAACGATTCAAAGCATTTC<br>TCAACCGTTGGTAGGGAGTTTCATTAGGGTTATAGAAGGGAGACTTTGGAGAGTCACGGTGGATCAGCTCAACACTGA<br>ACCCTCCAGTTTGAGCCTCTGCTGGAGAGATAACTGAGAGACAAAGGAAGAAGAGAATGAATGCACAACTCAAGAAG<br>GTTGCCAT
PtCDR5_DPI-50-7	GCGAGCATTAAAAACTGAGCACACAAGGTCTTCTGAAATATTCATAAAAACGTTGGAAGGGCTCAACTTCACATCTGCA<br>TCCCTGAAATGTATTGTGACTTCAGGAAATTGAGGTCGTGAACTAATTCTGTAGCAGAGATCATATGGCCCTTCAACGG<br>GTTGTCCCGCTATCATACTGGACATTACTGAAAGCAATTTTGAGGCATAAGCTGGTGGTAAATAGGTAAGTGTAGTACC<br>AGAATCAATTACAATGTCTCCTCCCGGAGTTGATCCTGATATAACACCTAGTCTTTGATCTCCTACACTGATTGCATCGA<br>GTGTGAGGTAATAGAAGGTTTTGGGATTCTTAACAAGCAAGGGAGTAGAAACTACGCCAGAACCTGAAACAATTCCAT<br>TGGTGCCAAAATTGATTTTGGTTGAGCTTTGTTGCACCAAGCAGTAGGAGAATTTACCTGCAATTGTAGTTCTTATTTGA<br>GAAATAAGGGAAGCATCACCTCCCCCAAGGCCAACGATGCCATCAGTTTTTGAATTAAATTTCCCACCATTTTTTTTTCC<br>ACAGCCGAAGACTATTTCTGGCAGAGCCACGGCCTGTCCGGATGTTGATCCCAAAGTAACAGTTTCGGTAGCTAGATC<br>GCCATTTGAGAAAGATTCTTKGTCTCCGTAGTGGACTGAATATTTGCAATTCCCCTCCGCAGAGCAAGAATCTTTGATCG<br>GTGGTGCGCATTGGCTCGATGAGCAAGAAAGATAATTGTAAGTGGAAGACTTTTGAGGATCAAAAAGGGCATTATCTT<br>GCTTGTAACATTGTGATGGTGGGCAGGGTTGACACTGTGTCCATATGAGATCACTTCCTGTGTCAGCCACAGCTAGTAT<br>TTCAACAGGGGGAGTACCAATTGATATACGAATGAGATAGTCACCAACATTAGGTATTATATCAGCTTGGGAAACTTTT<br>GATGAAGAGACTGATGAGTTTTTATTGAACTGACTGAGACGATTGGCAGAACGATTCAAAGCATTTCTCAACCGTTGGT<br>AGGGAGTTTCAWTAGGGTTATAGAAGGGAGACTTTGGAGAGTCACGGTGGATCAGCTCAACACTGAACCCTCCAGTT<br>TGGGCCTCTGATGGAGAGATAACTGAGAGACAAAGGAAGAAGAGAATGAATGCACAACTCAAGAAGGTTGCCAT
PtCDR5_FD	CATTAAAAACTGAGCACACAAGGTCTTCTGAAATATTCATAAAAACGTTGGAAGGGCTCAACTTCACATCTGCATCCCT<br>GAAATGTATTGTGACTTCAGGAAATTGAGGTCGTGAACTAATTCTGTAGCAGAGATCATATGGCCCTTCAACCAGAATC<br>AATTACAATGTCTCCTCCCGGAGTTGATCCTGATATAACACCTAGTCTTTGATCTCCTACACTGATTGCATCGAGTGTGA<br>GGTAATAGAAGGTTTTGGGATTCTTAACAAGCAAGGGAGTAGAAACTACGCCAGAACCTGAAACAATTCCATTGGTGC<br>CAAAATTGATTTTGGTTGAGCTTTGTTGCACCAAGCAGTAGGAGAATTTACCTGCAATTGTAGTTTTCATTTGAGAAATA<br>AGGGAACCATCACCTCCCCCAAGGCCAACGATGCCAGCAGTTTTTGAATTAGATCGCCATTTGAGAAAGAATTGTCTCC<br>GTAGTGGACTGAATATTTGCAATTCCCCTCCGCAGAGCAAGAATCTTTGATCGGTGGTGCGCATTGGCTCGATGAGCAA<br>GAAAGATAATTGTAAGTGGAAGACTTTTGAGGATCAAAAAGGGCATTATCTTGCTTGTAACATTGTGATGGTGGGCAG<br>GGTTGACACTGTGTCCATATGAGATCACTTCCTGTGTCAGCCACAGCTAGTATTTCAACAGGGGGAGTACCAATTGATA<br>TACGAATGAGATAGTCACCAACATTAGGTATTATATCAGCTTGGGAAACTTTTGATGAAGAGACTGATGAGTTTTTATT<br>GAACTGACTGAGACGATTGGCAGAACGATTCAAAGCATTTCTCAACCGTTGGTAGGGAGTTTCATTAGGGTTATAGAA<br>GGGAGACTTTGGAGAGTCACGGTGGATCAGCTCAACACTGAACCCTCCAGTTTGRGCCTCTGATGGAGAGATAACTGA<br>GAGACAAAGGAAGAAGAGAATGAATGCACAACTCAAGAAGGTTGCCAT

PtCDR6_812	AAACACTCTGCTGTCCCTCAACTCTCCCTTCAATTTCAATGGAAGGGAAAGCAAGAAACCATCTTCTCCTTCTCTTCTCCT<br>TCTTCTTCACCGCCGCTGCCTCTCTCCAGTATCAAACCTTCGTCCTYAACTCTCTCCCTACCCCATCCACTCTCTCTTGGCCT<br>GAATCCGTATCCGTWTCCGAATCCGAATCTTCRCTCCCTCTACCAGCACCYGACGCAGAATCCTCGCTYTCCCTACGTTT<br>ACATCACGTAGACTCTCTGTCCTTCAACAGAACTCCAGAGCACCTCTTCAACCTCCGCATCCAACGCGACGTCTTACGTG<br>TCAAATCCTTAAMCGCCTTCGCTGAATCTGCCGTTCGTGYTCCCCCGCGCAACCGCTCYCGCGGGCGTGCCAACGGAGG<br>CTTTAGTAGCTCCGTCATATCAGGRCTCGCACAAGGCAGCGGCGAGTACTTCACGCGCCTCGGAGTTGGCACACCCCCT<br>AGGTATGTCTACATGGTCTTAGACACCGGTAGCGACGTCGTTTGGATACAGTGCGCTCCGTGCAAGAAATGCTACTCTC<br>AAACCGACCCTGTCTTCGACCCTGCCAAGTCCCGCTCGTTTGCTACCGTACCCTGTCGGTCCCCTTTGTGCCGCAAGCTC<br>GACTCTTCTGGCTGTAACCGCAGGAACACGTGTCTTTACCAAGTCTCYTACGGAGACGGTTCTATCACCGTCGGTGATTT<br>TTCCACTGAAACRCTGACGTTTCGKGGCACCAGAGTTGCGCGCGTGGCCCTCGGTTGTGGACACGACAATGAGGGCCT<br>CTTCGTTGCTGCTGCGGGCTTATTGGGGCTCGGTCGTGGGAGGTTGTCATTCCCAATACAAACCGGTCGCCGGTTCAAC<br>CGTAAGTTTTCTTACTGTTTAGTGGACCGATCCACTTCTGCTAAACCTTCATCGATGGTATTCGGTGACTCGGCCGTTTCA<br>CGAACCGCCCGGTTCACTCCGTTACTCACAAACCCTAAGCTCGACACATTCTACTATGTTGAACTAGTTGGAATCAGCGT<br>CGGTGGTGCTCATGTCCGTGGCATCACTGCTTCTTTGTTCAAACTCGATCCGGCCGGTAACGGTGGGGTCATCATAGAC<br>TCGGGTACGTCCGTGACTCGTTTGACCCGACCCGCTTACATCGCACTGCGTGATGCTTTCCGAGCTGGAGCATCCTCCTT<br>GAAGCGTGCGCCAGACTTTTCGCTGTTTGATACGTGCTTCGATTTGTCGGGAAAGACGGAAGTGAAGGTGCCCACGGT<br>GGTGTTGCATTTCAGAGGTGCTGATGTGTCGTTGCCGGCGACTAATTATCTAATACCCGTGGATAGTAGCGGAACATTT<br>TGCTTTGCATTTGCAGGAACCATGAGCGGGTTGTCGATAATTGGAAATATTCAGCAACAGGGCTTCCGGGTTGTTTATG<br>AYTTGGCCGCTTCACGGATCGGGTTTGCTCCGCGCGGGTGCGCTTGAGGTTGCATTGCGCGTGGGTGTAGTTGTAAGG<br>GAAAMACTCTCTTTTCCTGTTAATTATTCTGTCTCTGCGTGTTTTGTTTTATTTNATTTCTTTTTTTTTNGGGAATATGAA<br>AAAAAAATCTTTCCTGGATRYGAGTCTGAGAAARGAGATGGAGAAGAGAAGGGGGAAAAATGAWAAAATAATCAGA<br>GTGCAGGTGATMAGGTTTATAATTCACATATTTGTCTCTGTTAWATTATGAAATGYTTTTAATCAGTCTTCTAAAAGAA<br>AAAAAAA
PtCDR6_897	AAACACTCTGCTGTCCCTCAACTCTCCCTTCAATTTCAATGGAAGGGAAAGCAAGAAACCATCTTCTCCTTCTCTTCTCCT<br>TCTTCTTCACCGCCGCTGCCTCTCTCCAGTATCAAACCTTCGTCCTYAACTCTCTCCCTACCCCATCCACTCTCTCTTGGCCT<br>GAATCCGTATCCGTTTCCGAATCCGAATCTTCRCTCCCTCTACCAGCACCCGACGCAGAATCCTCGCTCTCCCTACGTTTA<br>CATCACGTAGACTCTCTGTCCTTCAACARAACTCCAGAGCACCTCTTCAACCTCCGCATCCAACGCGACGTCTTACGTGT<br>CAAATCCTTAACCGCCTTCGCTGAATCTGCCGTTCGTGCTCCCCCGCGCAACCGCTCTCGCGGGCGTGCCAACGGAGGC<br>TTTAGTAGCTCCGTCATATCAGGACTCGCACAAGGTAGCGGTGAGTACTTCACGCGCCTCGGAGTTGGCACACCCCCTA<br>GGTATGTCTACATGGTCTTAGACACCGGTAGCGACGTCGTTTGGATACAGTGCGCTCCATGCAAGAAATGCTACTCTCA<br>AACCGACCCTGTCTTCGACCCTGCCAAGTCCCGCTCGTTTGCTACCGTACCCTGTCGCTCCCCTTTGTGCCGCAAGCTCG<br>ACTCTTCTGGCTGTAACCGCAGGAACACGTGTCTTTACCAAGTCTCTTACGGAGACGGTTCTATCACCGTCGGTGATTTT<br>TCCACTGAAACACTGACGTTTCGGGGCACCAGAGTTGCGCGCGTGGCCCTCGGTTGTGGACACGACAATGAGGGCCTC<br>TTCGTTGCTGCTGCGGGCTTATTGGGGCTCGGTCGTGGGAGGTTGTCATTCCCAATACAAACCGGTCGCCGGTTCAACC<br>GTAAGTTTTCTTACTGTTTAGTGGACCGATCCACTTCTGCTAAACCTTCATCGATGGTATTCGGTGACTCGGCCGTTTCAC<br>GAACCGCCCGGTTCACTCCGTTACTCACAAACCCTAAGCTCGACACATTCTACTATGTTGAACTAGTTGGAATCAGCGTC<br>GGTGGTGCTCATGTCCGTGGCATCACTGCTTCTTTGTTCAAACTCGATCCGGCCGGTAACGGTGGGGTCATCATAGACT<br>CGGGTACGTCCGTGACTCGTTTGACCCGACCCGCTTACATCGCACTGCGTGATGCTTTCCGAGCTGGAGCATCCTCCTT<br>GAAGCGTGCGCCAGACTTTTCGCTGTTTGATACGTGCTTCGATTTGTCGGGAAAGACGGAAGTGAAGGTGCCCACGGT<br>GGTGTTGCATTTCAGAGGTGCTGATGTGTCGTTGCCGGCGACTAATTATCTAATACCCGTGGATAGTAGCGGAACATTT<br>TGCTTTGCATTTGCAGGAACCATGAGCGGGTTGTCGATAATTGGAAATATTCAGCAACAGGGCTTCCGGGTTGTTTATG<br>ATTTGGCCGCTTCACGGATCGGGTTTGCTCCGCGCGGGTGCGCTTGAGGTTGCATTGCGCGTGGGTGTAGTTGTAAGG<br>GAAAMACTCTCTTTTCCTGTTAATTATTCTGTCTCTGCGTGTTTTGTTTTATTTTATTTCTTTTTTTTTTGGGGAATATGAA<br>AAAAAAATCTTTCCTGGATRYGAGTCTGAGAAAAGAGATGGAGAAGAGAAGGGGGAAAAATGAWAAAATAATCAAG<br>AGTGCAGGTGATMAGGTTTATAATTCACATATTTGTCTCTGTTAWATTATGAAATGYTTTTAATCAGTCTTCTAAAAGA<br>AAAAAAAA

PtCDR6_DPI-50-7	AAACACTCTGCTGTCCCTCAACTCTCCCTTCAATTTCAATGGAAGGGAAAGCAAGAAACCATCTTCTCCTTCTCTTCTCCT<br>TCTTCTTCACCGCCGCTGCCTCTCTCCAGTATCAAACCTTCGTCCTCAACTCTCTCCCTACCCCATCCACTCTATCTTGGCC<br>TGAATCCGTATCCGTATCCGAATCCGAATCCGAATCTTCRCTCCCTCTACCAGCACCCGACGCAGAATCCTCGCTCTCCCT<br>ACGTTTACATCACGTAGACTCTCTGTCCTTCAACAAAACTCCAGAGCACCTCTTCAACCTCCGCATCCAACGCGACGTCTT<br>ACGTGTCAAATCCTTAACCGCCTTCGCTGAATCTGCCGTTCGTGCTCCCCCGCGCAACCGCTCTCGCGGGCGTGCCAAC<br>GGAGGCTTTAGTAGCTCCGTCATATCAGGACTCGCACAAGGCAGCGGCGAGTACTTCACGCGCCTCGGAGTTGGCACA<br>CCCCCTAGGTATGTCTACATGGTCTTAGACACCGGTAGCGACGTCGTTTGGATACAGTGCGCTCCATGCAAGAAATGCT<br>ACTCTCAAACCGACCCTGTCTTCGACCCTGCCAAGTCCCGCTCGTTTGCTACCGTACCCTGTCGCTCCCCTTTGTGCCGCA<br>AGCTCGACTCTTCTGGCTGTAACCGCAGGAACACGTGTCTTTACCAAGTCTCTTACGGAGACGGTTCTATCACCGTCGG<br>TGATTTTTCCACTGAAACACTGACGTTTCGGGGCACCAGAGTTGCGCGCGTGGCCCTCGGTTGTGGACACGACAATGA<br>GGGCCTCTTCGTTGCTGCTGCGGGCTTATTGGGGCTCGGTCGTGGGAGGTTGTCATTCCCAATACAAACCGGTCGCCG<br>GTTCAACCGTAAGTTTTCTTACTGTTTAGTGGACCGATCCACTTCTGCTAAACCTTCATCGATGGTATTCGGYGACTCGG<br>CCGTTTCACGAACCGCCCGGTTCACTCCGTTACTCACAAACCCTAAGCTCGACACATTCTACTATGTTGAACTAGTTGGA<br>ATCAGCGTCGGTGGTGCTCATGTCCGTGGCATCACTGCTTCTTTGTTCAAACTCGATCCGGCCGGTAACGGTGGGGTCA<br>TCATAGACTCGGGTACGTCCGTGACTCGTTTGACCCGACCCGCTTACATCGCACTGCGTGATGCTTTCCGAGCTGGAGC<br>ATCCTCCTTGAAGCGTGCGCCAGACTTTTCGCTGTTTGATACGTGCTTCGATTTGTCGGGAAAGACGGAAGTGAAGGTG<br>CCCACGGTGGTGTTGCATTTCAGAGGTGCTGATGTGTCGTTGCCGGCGACTAATTATCTAATACCCGTGGATAGTAGCG<br>GAACATTTTGCTTTGCATTTGCAGGAACCATGAGCGGGTTGTCGATAATTGGAAATATTCAGCAACAGGGCTTCCGGGT<br>TGTTTATGATTTGGCCGCTTCACGGATCGGGTTTGCTCCGCGCGGGTGCGCTTGAGGTTGCATTGCGCGTGGGTGTAGT<br>TGTAAGGGAAACACTCTTTTCCTGTTAATTATTCTGTCTCTGCGTGTTTTGTTTTATTTATTTCTTTTTTTTGGGAATATGA<br>AAAAAAAATCTTTCCTGGATGCGAGTCTGAGAAAAGAGATGGAGAAGAGAAGGGGGAAAAATGAAAAAATAATCAG<br>AGTGCAGGTGATCAGGTTTATAATTCACATATTTGTCTCTGTTATATTATGAAATGTTTTTAATCAGTCTTCTAAAAGAAA<br>AAAAAA
PtCDR6_FD	AAACACTCTGCTGTCCCTCAACTCTCCCTTCAGTTTCAATGGAAGGGAAAGCAAGAAACCATCTTCTCCTTCTCTTCTCCT<br>TCTTCTTCACCGCCGCTGCCTCTCTCCAGTATCAAACCTTCGTCCTCAACTCTCTCCCTACCCCCTCCACTCTCTCTTGGCCT<br>GAATCCGTATCCGNAATCCGAATCCGAATCTTCGCTCCCTCTACCAGCACCCGACGCAGAATCCTCGCTCTCCCTACGTT<br>TACATCACGTAGACTCTCTGTCCTTCAACAAAACTCCAGAGCACCTCTTCAACCTCCGCATCCAACGCGACGTCTTACGT<br>GTCAAATCCTTAACCGCCTTCGCTGAATCTGCCGTTCGTGCTCCCCCGCGCAACCGCTCTCGCGGGCGTGCCAACGGAG<br>GCTTTAGTAGCTCCGTCATATCAGGACTCGCACAAGGYAGCGGYGAGTACTTCACGCGCCTCGGAGTTGGCACACCCCC<br>TAGGTATGTCTACATGGTCTTAGACACCGGTAGCGACGTCGTTTGGATACAGTGCGCTCCATGCAAGAAATGCTACTCT<br>CAAACCGACCCTGTCTTCGACCCTGCCAAGTCCCGCTCGTTTGCTACCGTACCCTGTCGCTCCCCTTTGTGCCGCAAGCT<br>CGACTCTTCTGGCTGTAACCGCAGGAACACGTGTCTTTACCAAGTCTCTTACGGAGACGGTTCTATCACCGTCGGTGATT<br>TTTCCACTGAAACACTGACGTTTCGGGGCACCAGAGTTGCGCGCGTGGCCCTCGGTTGTGGACACGACAATGAGGGCC<br>TCTTCGTTGCTGCTGCGGGCTTATTGGGGCTCGGTCGTGGGAGGTTGTCATTCCCAATACAAACCGGTCGCCGGTTCAA<br>CCGTAAGTTTTCTTACTGTTTAGTGGACCGATCCACTTCTGCTAAACCTTCATCGATGGTATTCGGTGACTCGGCCGTTTC<br>ACGAACCGCCCGGTTCACTCCGTTACTCACAAACCCTAAGCTCGACACATTCTACTATGTTGAACTAGTTGGAATCAGCG<br>TCGGTGGTGCTCATGTCCGTGGCATCACTGCTTCTTTGTTCAAACTCGATCCGGCCGGTAACGGTGGGGTCATCATAGA<br>CTCGGGTACGTCCGTGACTCGTTTGACCCGACCCGCTTACATCGCACTGCGTGATGCTTTCCGAGCTGGAGCATCCTCCT<br>TGAAGCGTGCGCCAGACTTTTCGCTGTTTGATACGTGCTTCGATTTGTCGGGAAAGACGGAAGTGAAGGTGCCCACGG<br>TGGTGTTGCATTTCAGAGGTGCTGATGTGTCGTTGCCGGCGACTAATTATCTAATACCCGTGGATAGTAGCGGAACATT<br>TTGCTTTGCATTTGCAGGAACCATGAGCGGGTTGTCGATAATTGGAAATATTCAGCAACAGGGCTTCCGGGTTGTTTAT<br>GATTTGGCCGCTTCACGGATCGGGTTTGCTCCGCGCGGGTGCGCTTGAGGTTGCATTGCGCGTGGGTGTAGTTGTAAG<br>GGAAACACTCTTTTCCTGTTAATTATTCTGTCTCTGCGTGTTTTGTTTTATTTATTTCTTTTTTTTGGGAATATGAAAAAAA<br>AATCTTTCCTGGATGCGAGTCTGAGAAAAGAGATGGAGAAGAGAAGGGGGAAAAATGAAAAAATAATCAAGAGTGC<br>AGGTGATCAGGTTTATAATTCACATATTTGTCTCTGTTATATTATGAAATGTTTTTAATCAGTCTTCTAAAAGAAAAAAAA<br>A

PtCDR7_812	ATGCATTTATCAATTGGCAGTCCTCCTGTCGATATATATGGTTCAGTTGACACAGGAAGTGACTTAGTATGGACACAGT<br>GTGAGCCTTGCTCACAATCACAGTGTTTCCGGCAAGAGGCTCCACTCTTTGATCCTAAAAAGTCTTCCACATACAATTCT<br>ATTTCTTGCTTCTCATTCCAATGTGTCATAGCCGGAACGAATTGCTCCGAAGGAGATTGCATTTACTCCTTGGCATATGG<br>AAGAGGTTTACATGGATCGTTCTCAAGTGGCAATATTGCCACTGAAACTCTCACTTTCGCTTCAACATCCGGACAGCCCC<br>TTGAAATGCCAAAAATAATCTTTGGTTGCGGACACAACAACAGTGAAAGCCCAAACACTCCTTCAAATCAAACCGGCAC<br>CATTGGCCTTGGACCCGGCAGTTCTTCATTAATTTCTCAAATGGCAACATCAATTGCTGGTAAATTCTCCTATTGCCTGCC<br>CCATCAAGGTTCAAGCACAATAAACTTTGGTGGAATTGTTGCGGGTGCCGGGGTAGTTTCTACCCCTTTGATTATTAGG<br>GACCATTATTATCTCACCCTTGAAGCAATCAGTGTAGGCAACCAAAGGCTAGAATTTGTGTCCGGTGGAAAAAATTTTA<br>TTGATGCTGGAAACATTTTTGTTGATACTGGTGTTCAACGTACTTTATTACCACTAGAGTATTACTCAAATTTGAAGTTA<br>GTAATGTCCAACATGATAAGGGCACAACCCATTGAAATCAAAATAGGTGAGCCTGGCCTATCTGATATTCTCTGTTATA<br>ATATTAGTTCACAGCCTGAGCTTCCTGAAGTAACAATTCATTTTAAAGGCGCAGATGTGAAATTGAGCCCTTCCAACATT<br>TTTCAGAATATTTCAGATGATCTCATGTGTTCTGCTTTTAGAGGTGGTGATGCCAATACAATTTACGGGAACATAATGCA<br>GATAAACTTCTTAATCGGCTATGACATTGAGCAAAGGACAGTGTCGTTTAAGTCGACTCATTGCGCCAACAATTAATTA<br>AGTAACTGGAAAGCTTGGGAAGTGTGCTTTTGGTTAATGTTTAAACTGTTTTTTTCTTTATTTTAATTGATGTTGTTCTAT<br>TTGATTAGAAGTCTTATATCTAAAGCTTTCTCGTCCT
PtCDR7_897	ATGCATTTATCAATTGGCAGTCCTCCTGTCGATATATATGGTTCWRTTGACACAGGAAGTGACTTARTATGGACACAGT<br>GTGAGCCTTGCCCACAATCACARTGTTTCYRGCAAGAGGCTCCAYTCTTTGATCCTAAAAARTCTTCCACATACAATTCTA<br>TTTCTTGCTTCTCATTCCAATGTGTCATAGCCGGAACGAATTGCTCCGAAGGAGATTGCATTTACTCCTTGGCATATGGA<br>AGAGGTTTACATGGATCGTTCTCAAGTGGCAATATTGCCACTGAAACTCTCACTTTCGGTTCAACATCCGGACAGCCCCT<br>TGAAATGCCMAAAATAATCTTTGGTTGCGGACACAACAACAGTGAAAGCCCAAACACTCGTTCAAATCGAACCGGCAC<br>CATTGGCCTTGGACCCGGCAGTTCTTCATTAATTTCTCAAATGGCAACATCAATTGCTGGTAAATTCTCCTATTGCCTGCC<br>CCATCAAGGTTCAAGCACAATAAACTTTGGTGGAATTGTTGCGGGTGCCGGGGTAGTTTCTACCCCTTTGATTATTAGG<br>GACCATTATTATCTCACCCTTGAAGCAATCAGTGTAGGCAACCAAAGGCTAGAATTTGTGTCCGGTGGAAAATATTTTA<br>TTGATGCTGGAAACATTTTTGTTGATACTGGTGTTCAACGTACTTTATTACCACTAGAGTATKACTCAAATTTGAAGTTA<br>GTAATGTCCAACATGATAAGGGCACAACCCATTGAAATCAAAATAGGTGAGCCTGGCCTATCTGATATTCTCTGTTATA<br>ATATTAGTTCACAGCCTGAGCTTCCTGAAGTAACAATTCATTTTAAAGGCGCAGATGTGAAATTGAGCCCTTCCAACATT<br>TTTCAGAATTTTTCAGATGATCTCATGTGTTCTGCTTTTAGAGGTGGTGATGCCAATACAATTTACGGGAACATAATGCA<br>GATAAACTTCTTAATCGGCTATGACATTGAGCAAAGGACAGTGTCGTTTAAGTCGACTCATTGCGCCAACAATTAATTA<br>AGTAACTGGAAAGCTTGGGAAGTGTGCTTTTGGTTAATGTTTAAACTGTTTTTTNCTTTATTTTAATTGATGTTGTTCTAT<br>TTGATTAGAAGTCTTATATCTAAAGCTTTCTCGTCCT
PtCDR7_DPI-50-7	ATGCATTTATCAATTGGCAGTCCTCCTGTCGATATATATGGTTCAGTTGACACAGGAAGTGACTTAGTATGGACACAGT<br>GTGAGCCTTGCYCACAATCACAGTGTTTCCGGCAAGAGGCTCCACTCTTTGATCCTAAAAAGTCTTCCACATACAATTCT<br>ATTTCTTGCTTCTCATTCCAATGTGTCATAGCCGGAACGAATTGCTCCGAAGGAGATTGCATTTACTCCTTGGCATATGG<br>AAGAGGTTTACATGGATCGTTCTCAAGTGGCAATATTGCCACTGAAACTCTCACTTTCGCTTCAACATCCGGACAGCCCC<br>TTGAAATGCCAAAAATAATCTTTGGTTGCGGACACAACAACAGTGAAAGCCCAAACACTCCTTCAAATCAAACCGGCAC<br>CATTGGCCTTGGACCCGGCAGTTCTTCATTAATTTCTCAAATGGCAACATCAATTGCTGGTAAATTCTCCTATTGCCTGCC<br>CCATCAAGGTTCAAGCACAATAAACTTTGGTGGAATTGTTGCGGGTGCCGGGGTAGTTTCTACCCCTTTGATTATTAGG<br>GACCATTATTATCTCACCCTTGAAGCAATCAGTGTAGGCAACCAAAGGCTAGAATTTGTGTCCGGTGGAAAAAATTTTA<br>TTGATGCTGGAAACATTTTTGTTGATACTGGTGTTCAACGTACTTTATTACCACTAGAGTATTACTCAAATTTGAAGTTA<br>GTAATGTCCAACATGATAAGGGCACAACCCATTGAAATCAAAATAGGTGAGCCTGGCCTATCTGATATTCTCTGTTATA<br>ATATTAGTTCACAGCCTGAGCTTCCTGAAGTAACAATTCATTTTAAAGGCGCAGATGTGAAATTGAGCCCTTCCAACATT<br>TTTCAGAATATTTCAGATGATCTCATGTGTTCTGCTTTTAGAGGTGGTGATGCCAATACAATTTACGGGAACATAATGCA<br>GATAAACTTCTTAATCGGCTATGACATTGAGCAAAGGACAGTGTCGTTTAAGTCGACTCATTGCGCCAACAATTAATTA<br>AGTAACTGGAAAGCTTGGGAAGTGTGCTTTTGGTTAATGTTTAAACTGTTTTTTNCTTTATTTTAATTGATGTTGTTCTAT<br>TTGATTAGAAGTCTTATATCTAAAGCTTTCTCGTCCT

PtCDR7_FD	ATGCATTTATCAATTGGCAGTCCTCCTGTCGATATATATGGTTCAGTTGACACAGGAAGTGACTTAGTATGGACACAGT<br>GTGAGCCTTGCCCACAATCACAGTGTTTCCGGCAAGAGGCTCCACTCTTTGATCCTAAAAAGTCTTCCACATACAATTCT<br>ATTTCTTGCTTCTCATTCCAATGTGTCATAGCCGGAACGAATTGCTCCGAAGGAGATTGCATTTACTCCTTGGCATATGG<br>AAGAGGTTTACATGGATCGTTCTCAAGTGGCAATATTGCCACTGAAACTCTCACTTTCGSTTCAACATCCGGACAGCCCC<br>TTGAAATGCCMAAAATAATCTTYGGTTGCGGACACAACAACAGTGAAAGCCCAAACACTCCTTCAAATCAAACCGGCA<br>CCATTGGCCTTGGACCCGGCAGTTCTTCATTAATTTCTCAAATGGCAACATCAATTGCTGGTAAATTCTCCTATTGCCTGC<br>CCCATCAAGGTTCAAGCACAATAAACTTTGGTGGAATTGTTGCGGGTGCCGGGGTAGTTTCTACCCCTTTGATTATTAG<br>GGACCATTATTATCTCACCCTTGAAGCAATCAGTGTAGGCAACCAAAGGCTAGAATTTGTGTCCGGTGGAAAAAATTTT<br>ATTGATGCTGGAAACATTTTTGTTGATACTGGTGTTCAACGTACTTTATTACCACTAGAGTATTACTCAAATTTGAAGTTA<br>GTAATGTCCAACATGATAAGGGCACAACCCATTGAAATCAAAATAGGTGAGCCTGGCCTATCTGATATTCTCTGTTATA<br>ATATTAGTTCACAGCCTGAGCTTCCTGAAGTAACAATTCATTTTAAAGGCGCAGATGTGAAATTGAGCCCTTCCAACATT<br>TTTCAGAATATTTCAGATGATCTCATGTGTTCTGCTTTTAGAGGTGGTGATGCCAATACAATTTACGGGAACATAATGCA<br>GATAAACTTCTTAATCGGCTATGACATTGAGCAAAGGACAGTGTCGTTTAAGTCGACTCATTGCGCCAACAATTAATTA<br>AGTAACTGGAAAGCTTGGGAAGTGTGCTTTTGGTTAATGTTTAAACTGTTTTTTCTTTATTTTAATTGATGTTGTTCTATT<br>TGATTAGAAGTCTTATATCTAAAGCTTTCTCGTCCT
PtCDR8_812	ATGGCAACCTTCTTGAGTTGTGCATTCATTCTCTTCTTTCTTTTTTTTTATGTWGTCTCTCCAATAGAAGCTCAAACTGGA<br>GGTTTCAGTGTTGAGCTAATCCACCGTGACTCTCCCAAGTCTCCCTTTTACAACTCCAGTGAAACTCCCTACCAACGATT<br>GAGAAATGCTTTTACTCGTTCTACCAATCGTCCCAATCACTTTAATCAGAACTCATCAATATCTTCATCAAAAGCTTCCCA<br>AGCTGATATAATACCCAATATCGGAGACTATCTCATACGTATATCAATTGGTACTCCCGCGACTGAAATACTAGCAGTTG<br>CTGATACAGGAAGTGACCTCATATGGACGCAGTGCGAGCCTTGCCCACCATCACAATGTTACATGCAGGACTCTCCACT<br>TTTTGATCCTAAAAAGTCATCTACTTACAAATCTCTTCCATGCTCSTCAAGCCAATGTGCATCTCTTARCCAAAAATCTTGC<br>TCTGGGGTAAATTGTCAATACTCGGTCTCTTATGGAGATGGATCCTTCTCAAATGGCAATCTTGCTACCGAAACTGTTAC<br>TTTGGGTTCAACAACAGGACAGGCCGTTGCTCTGCCTGGAATAACCTTTGGTTGCGGAACAAACAATGGTGGCTTGTTT<br>AATTCAAAAACAACCGGAATTGTTGGCCTTGGAGGCGGCGATATTTCACTTATTTCTCAAATGCGAWCTACAATTGCTG<br>STAAATTCTCCTATTGTTTGGTTCAGGCAAGCTCAACCAAAATCAATTTTGGAACCAATGGAATTGTTTCAGGTCCAGGA<br>GTGGTTTCTACTCCCTTGATTGTTAAATCCAAARCCTTTTATGYTCTCACAATTGATGCAATCAGTGTAGGAAACCAAAG<br>ACTAGMAGTTATGTCTGGATCGACTCCAGGAGGAGACATTGTAATCGATTCTGGTACAACACTTACCTTATTGCCACAA<br>SGTTATAACTCAAAATTGTTGTCAGTAATGTCCAGTATGATTGAGGCACAACCTGTTGCAGACCCTAAAGGATCAYTTGA<br>ACTTTGCTACAGTTTTAATTCACTATCTCAAGTTCCTGAAGTTACAATACATTTCAGAGGTGCAGATGTGAAATTGAGTC<br>CTTCCAACTTTTTTGTGAAGGTTTCGGAGGATATTGTATGTTCTGTTTTTAAAGGTACCANNNATAGTCTGCCACTTTAT<br>GGTAATATAATGCAGACTAACTTTTTGGTCGGCTATGACATAGAACAASRAACAGTGTCATTTAAACCGACTGAYTGCA<br>CCAAGCAGTAATTAATTGGGAAGCTTATTCGATATTCTCCCCTCTACTAGAGTTTGATCGAGTGATTTTCAARCCACTTTC<br>TTTAGCMGATCAACCGATTTCAAATTCTAATGCGATTGGAGCTGAATAAAATCTATTTAAATAAATCTATTT
PtCDR8_897	ATGGCAACCTTCTTGAGTTGTGCATTCATTCTCTTCTTTCTTTTTTTTTATGTAGTCTCTCCAATAGAAGCTCAAACTGGAG<br>GTTTCAGTGTTGAGCTAATCCACCGTGACTCTCCCAAGTCTCCCTTTTACAACTCCAGTGAAACTCCCTACCAACGATTGA<br>GAAATGCTTTTACTCGTTCTACCAATCGTCCCAATCACTTTAATCAGAACTCATCAATATCTTCATCAAAAGCTTCCCAAG<br>CTGATATAATACCCAATATCGGAGACTATCTCATACGTATATCAATTGGTACTCCCGCGACTGAAATACTAGCAGTTGCT<br>GATACAGGAAGTGACCTCATATGGACGCAGTGCGAGCCTTGCCCACCATCACAATGTTACATGCAGGACTCTCCACTTT<br>TTGATCCTAAAAAGTCATCTACTTACAAATCTCTTCCATGCTCSTCAAGCCAATGTGCATCTCTTARCCAAAAATCTTGCT<br>CTGGGGTAAATTGTCAATACTCGGTCTCTTATGGAGATGGATCCTTCTCAAATGGCAATCTTGCTACCGAAACTGGCCG<br>TGGCTCTGCCTGGTATAACCTTTGGTTGCGGAACAAACAATGGTGGCTTGTTTAATTCAAAAACAACCGGAATTGTTGG<br>CCTTGGAGGCGGCGATATTTCACTTATTTCTCAAATGCGAWCTACAATTGCTGSTAAATTCTCCTATTGTTTGGTTCAGG<br>CAAGCTCAACCAAAATCAATTTTGGAACCAATGGAATTGTTTCAGGTCCAGGAGTGGTTTCTACTCCCTTGATTGTTAAA<br>TCCAAAACCTTTTATGYTCTCACAATTGATGCAATCAGTGTAGGAAACCAAAGACTAGMAGTTATGTCTGGATCGACTC<br>CAGGAGGAGACATTGTAATCGATTCTGGTACAACACTTACCTTATTGCCACAASGTTATAACTCAAAAYTGTTGTCAGTA<br>ATGTCCAGTATGATTGAGGCACAACCTGTTGCAGACCCTAAAGGATCAYTTGAACTTTGCTACAGTTTTAATTCACTATC<br>TCAAGTTCCTGAAGTTACAATACATTTCAGAGGTGCAGATGTGAAATTGAGTCCTTCCAACTTTTTTGTGAAGGTYTCGR<br>AGGATATTGTRTGTTCTGTTTTTAAAGGTACCAATAGTCTGCCACTTTATGGTAATATAATGCAGACTAACTTTTTGGTC<br>GGCTATGACATAGAACAASRAACWGTGTCATTTAAACCGACWGAYTGCACCAAGCARTAATTAATTGGGAAGCTTAW<br>TCGATATTCTCCMCTCTACTAGAGTTTGATCGAGTGATTTTCAAGCCACTTTCTTTAGCCGATCAACCGATTTCAAATTCT<br>AATGCGATTGGAGCTGAATAAAATCTATTTAAATAAATCTATTT

PtCDR8_DPI-50-7	ATGGCAACCTTCTTGAGTTGTGCATTCATTCTCTTCTTTTTTTTTTTTTATGTTGTCTCTCCAATAGAAGCTCAAACTGGAG<br>GTTTCAGTGTTGAGCTAATCCACCGTGACTCTCCCAAGTCTCCCTTTTACAACTCCAGTGAAACTCCCTACCAACGATTGA<br>GAAATGCTTTTACTCGTTCTACCAATCGTCCCAATCACTTTAATCAGAACTCATCAATATCTTCATCAAAAGCTTCCCAAG<br>CTGATATAATACCCAATATGGGAGACTATCTCATACGTATATCAATTGGTACTCCCGCGACTGAAATACTAGCAGTTGCT<br>GATACAGGAAGTGACCTCATATGGACGCAGTGCGAGCCTTGCCCACCATCACAATGTTACATGCAGGACTCTCCACTTT<br>TTTATCCTAAAAAGTCATCTACTTACAAATCTCTTCCATGCTCCTCAAGCCAATGTGCATCTCTTAGCCAAAAATCTTGCT<br>CTGGGGTAAATTGTCAATACTCGGTCTCTTATGGAGATGGATCCTTCTCAAATGGCAATCTTGCTACCGAAACTGTTACT<br>TTGGGTTCAACAACAGGACAGGCCGTGGCTCTGCCTGGAATAACCTTTGGTTGCGGAACAAACAATGGTGGCTTGTTT<br>AATTCAAAAACAACCGGAATTGTTGGCCTTGGAGGCGGCGGTATTTCACTTATTTCTCAAATGCGAACTACAATTGCTG<br>GTAAATTCTCCTATTGTTTGGTTCAGGCAAGCTCAACCAAAATCAATTTTGGAACCAATGGAATTGTTTCAGGTCCAGGA<br>GTGGTTTCTACTCCCTTGATTGTTAAATCCAAAACCTTTTATGCTCTCACAATTGATGCAATCAGTGTAGGAAACCAAAG<br>ACTAGAAGTTATGTCTGGATCGACTCCAGGAGGAGACATTGTAATCGATTCTGGTACAACACTTACCTTATTGCCACAA<br>CGTTATAACTCAAAATTGTTGTCAGTAATGTCCAGTATGATTGAGGCACAACCTGTTGCAGACCCTAAAGGATCACTTG<br>AACTTTGCTACAGTTTTAATTCACTATCTCAAGTTCCTGAAGTTACAATACATTTCAGAGGTGCAGATGTGAAATTGAGT<br>CCTTCCAACTTTTTTGTGAAGGTTTCGAAGGATATTGTATGTTCTGTTTTTAAAGGTACCAATMGTCTGCCACTTTATGG<br>TAATATAATGCAGACTAACTTTTTGGTCGGCTATGACATAGAACAAGGAACAGTGTCATTTAAACCGACTGATTGCACC<br>AAGCAATAATTAATTGGGAAGCTTAATCGATATTCTCCACTCTACTAGAGTTTGATCGAGTGATTTTCAAACCACTTTCTT<br>TAGCAGATCAACCGATTTCAAATTCTAATGCGATTGGAGCTGAATAAAATCTATTTAAATAAATGTA
PtCDR8_FD	ATGGCAACCTTCTTGAGTTGTGCATTCATTCTCTTCTTTTTTTTTTTTTATGTTGTCTCTCCAATAGAAGCTCAAACTGGAG<br>GTTTCAGTGTTGAGCTAATCCACCGTGACTCTCCCAAGTCTCCCTTTTACAACTCCAGTGAAACTCCCTACCAACGATTGA<br>GAAATGCTTTTACTCGTTCTACCAATCGTCCCAATCACTTTAATCAGAACTCATCAATATCTTCATCAAAAGCTTCCCAAG<br>CTGATATAATACCCAATATGGGAGACTATCTCATACGTATATCAATTGGTACTCCCGCGACTGAAATACTAGCAGTTGCT<br>GATACAGGAAGTGACCTCATATGGACGCAGTGCGAGCCTTGCCCACCATCACAATGTTACATGCAGGACTCTCCACTTT<br>TTTATCCTAAAAAGTCATCTACTTACAAATCTCTTCCATGCTCCTCAAGCCAATGTGCATCTCTTAGCCAAAAATCTTGCT<br>CTGGGGTAAATTGTCAATACTCGGTCTCTTATGGAGATGGATCCTTCTCAAATGGCAGTTCAACAGCAGGACAGGCCGT<br>GGCTCTGCCTGGAATAACCTTTGGTTGCGGAACAAACAATGGTGGCTTGTTTAATTCAAAAACAACCGGAATTGTTGGC<br>CTTGGAGGCGGCGGTATTTCACTTATTTCTCAAATGCGAACTACAATTGCTGGTAAATTCTCCTATTGTTTGGTTCAGGC<br>AAGCTCAACCAAAATCAATTTTGGAACCAATGGAATTGTTTCAGGTCCAGGAGTGGTTTCTACTCCCTTGATTGTTAAAT<br>CCAAAACCTTTTATGCTCTCACAATTGATGCAATCAGTGTAGGAAACCAAAGACTAGAAGTTATGTCTGGATCGACTCC<br>AGGAGGAGACATTGTAATCGATTCTGGTACAACACTTACCTTATTGCCACAACGTTATAACTCAAAATTGTTGTCAGTAA<br>TGTCCAGTATGATTGAGGCACAACCTGTTGCAGACCCTAAAGGATCACTTGAACTTTGCTACAGTTTTAATTCACTATCT<br>CAAGTTCCTGAAGTTACAATACATTTCAGAGGTGCAGATGTGAAATTGAGTCCTTCCAACTTTTTTGTGAAGGTTTCGAA<br>GGATATTGTATGTTCTGTTTTTAAAGGTACCAATAGTCTGCCACTTTATGGTAATATAATGCAGACTAACTTTTTGGTCG<br>GCTATGACATAGAACAAGGAACAGTGTCATTTAAACCGACTGATTGCACCAAGCAATAATTAATTGGGAAGCTTAATCG<br>ATATTCTCCACTCTACTAGAGTTTGATCGAGTGATTTTCAAACCACTTTCTTTAGCAGATCAACCGATTTCAAATTCTAAT<br>GCGATTGGAGCTGAATAAAATCTATTTAAATAAATGTAGTT
