# Supplementary material for: Genome resequencing and transcriptome profiling reveal structural diversity and expression patterns of constitutive disease resistance genes in Huanglongbing-tolerant Poncirus trifoliata and its hybrids
Source: Hortic Res. 2017 Nov 15;4:17064–. doi: 10.1038/hortres.2017.64 (PMC5686287; doi:10.1038/hortres.2017.64)
Supplement: Supplementary Table 6 [file hortres201764-s6.pdf]

Supplemental Table 6. Real-time PCR data. *CDR* gene expression values (fold changes) in *Poncirus* and *Citrus* genotypes before and after *C*las infection as detected by 14 pairs of primers designed from CDR gene sequences.

Primer_id	US-812	Std Deviation	Std Error	US-897	Std Deviation	Std Error	US-942	Std Deviation	Std Error	V	Std Deviation	Std Error	DG	Std Deviation	Std Error	RR	Std Deviation	Std Error
CDR1	5.37	3.80	2.19	2.45	1.91	1.10	1.84	0.85	0.49	1.48	1.34	0.78	1.82	2.52	1.45	1.97	0.98	0.56
CDR2	5.18	0.95	0.55	2.52	1.77	1.02	2.61	1.42	0.82	1.59	0.87	0.50	2.05	2.74	1.58	1.40	0.60	0.35
CDR5	5.83	7.05	4.07	2.65	2.55	1.47	0.85	0.69	0.40	1.57	1.53	0.88	2.02	2.35	1.36	1.91	1.05	0.61
CDR6	0.60	0.32	0.19	4.91	6.47	3.73	0.87	0.95	0.55	1.30	1.53	0.88	2.53	3.37	1.95	1.37	1.15	0.66
CDR7	6.24	3.64	2.10	3.41	1.36	0.79	1.29	0.75	0.43	0.95	0.82	0.47	1.51	1.47	0.85	2.47	1.35	0.78
CDR8	9.84	4.03	2.33	1.81	0.17	0.10	1.26	0.53	0.30	1.52	1.23	0.71	2.57	3.28	1.89	2.33	1.92	1.11
CDR9	5.26	3.79	2.19	2.24	1.79	1.04	1.76	0.67	0.39	2.88	-	-	1.67	1.79	1.03	1.52	0.55	0.32
CDR12	2.21	2.49	1.43	2.94	1.99	1.15	3.15	2.71	1.57	2.37	0.39	0.22	0.00	0.00	0.00	0.00	0.00	0.00
CDR13	5.07	3.15	1.82	2.46	1.41	0.82	1.84	1.21	0.70	1.27	1.08	0.63	1.14	1.04	0.60	1.71	1.01	0.58
CDR14	4.90	3.17	1.83	3.72	2.19	1.26	4.20	2.50	1.44	0.92	0.87	0.50	1.96	1.82	1.05	1.37	1.23	0.71
CDR15	4.85	2.72	1.57	1.76	1.02	0.59	2.53	0.22	0.13	0.76	0.74	0.43	0.64	0.49	0.28	1.33	0.14	0.08
CDR16	5.04	3.25	1.88	3.02	0.75	0.43	1.67	0.53	0.31	1.82	1.16	0.67	1.57	0.63	0.36	1.36	0.94	0.54
UaCDR1	5.47	2.91	1.68	2.17	0.95	0.55	2.02	1.34	0.78	1.61	1.60	0.93	1.52	1.11	0.64	2.22	0.91	0.53
UaCDR2	7.34	4.89	2.82	2.10	1.14	0.66	1.73	0.89	0.51	2.12	2.38	1.37	1.10	0.85	0.49	1.61	0.31	0.18

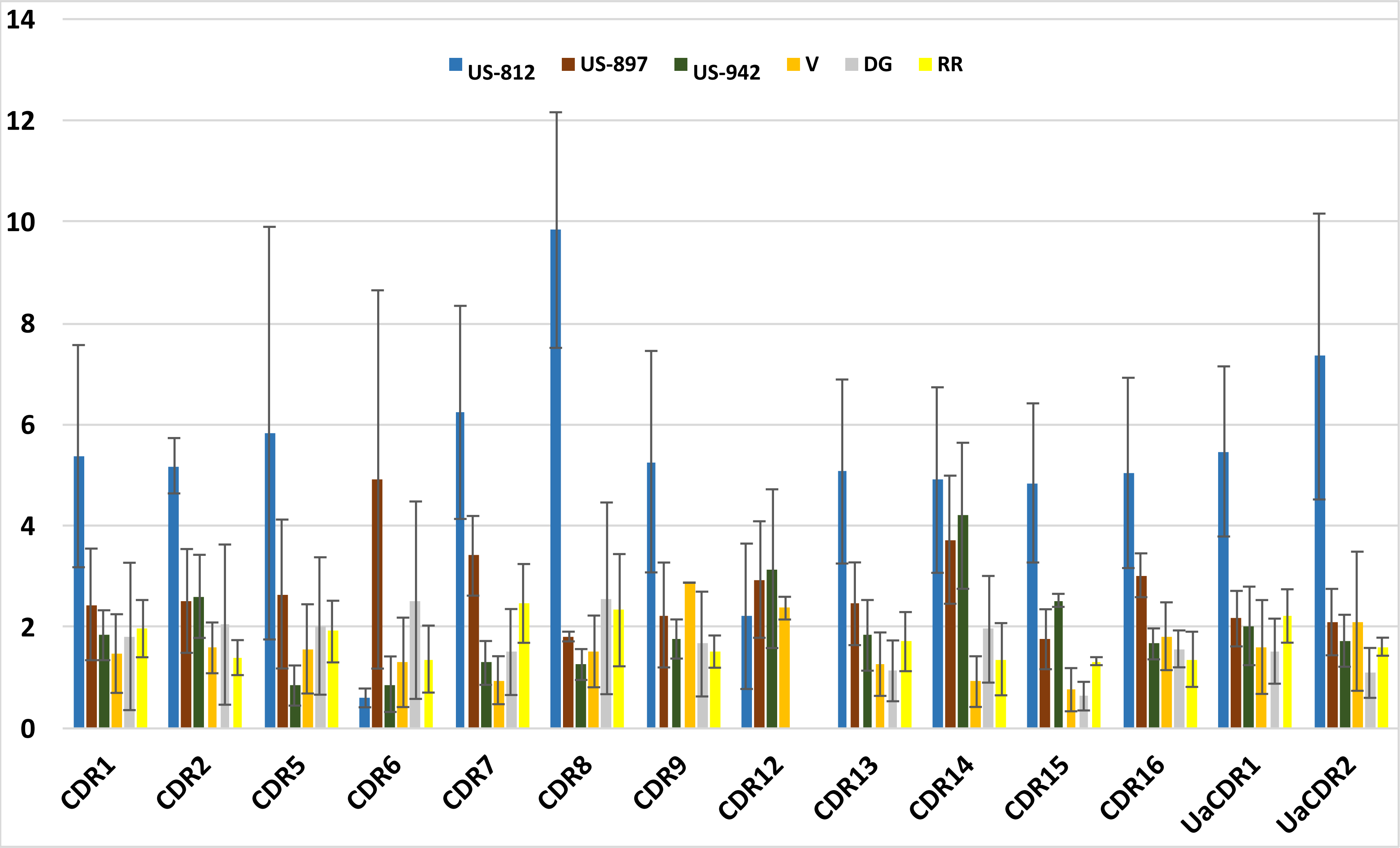

14
12
10
8
6
4
2
0
US-812
US-897
US-942
V
DG
RR
CDR1
CDR2
CDR5
CDR6
CDR7
CDR8
CDR9
CDR12
CDR13
CDR14
CDR15
CDR16
UaCDR1
UaCDR2

Supplemental Table 6. Real-time PCR data. Fold changes in three replicate leaf samples of *Poncirus* and *Citrus* genotypes before and after *C*las infection as detected by 14 pairs of primers designed from CDR gene sequences.

Primer_id	CDR1	CDR2	CDR5	CDR6	CDR7	CDR8	CDR_9	CDR_12	CDR_13	CDR_14	CDR_15	CDR_16	UteCDR_1	UteCDR_2
US812-1	5.46	0.00	10.82	0.00	8.00	6.99	6.08	5.05	6.54	5.33	7.92	6.59	7.39	9.75
US812-2	1.53	1.34	0.84	0.83	2.05	0.00	1.14	1.20	1.46	1.54	3.88	1.31	2.12	1.72
US812-3	9.13	9.03	0.00	0.38	8.66	12.68	8.57	0.40	7.21	7.84	2.74	7.24	6.89	10.56
**US-812**	**5.37**	**5.18**	**5.83**	**0.60**	**6.24**	**9.84**	**5.26**	**2.21**	**5.07**	**4.90**	**4.85**	**5.04**	**5.47**	**7.34**
**Std Deviation**	**3.80**	**0.95**	**7.05**	**0.32**	**3.64**	**4.03**	**3.79**	**2.49**	**3.15**	**3.17**	**2.72**	**3.25**	**2.91**	**4.89**
**Std Error**	**2.19**	**0.55**	**4.07**	**0.19**	**2.10**	**2.33**	**2.19**	**1.43**	**1.82**	**1.83**	**1.57**	**1.88**	**1.68**	**2.82**
US897-1	1.80	1.19	0.82	1.17	2.71	1.68	1.56	0.64	1.42	2.08	2.67	2.25	1.68	1.30
US897-2	4.59	4.53	5.56	12.38	4.98	2.00	4.27	3.96	4.07	6.21	1.97	3.75	3.26	3.40
US897-3	0.95	1.83	1.57	1.19	2.53	1.76	0.89	4.21	1.89	2.88	0.65	3.07	1.56	1.60
**US-897**	**2.45**	**2.52**	**2.65**	**4.91**	**3.41**	**1.81**	**2.24**	**2.94**	**2.46**	**3.72**	**1.76**	**3.02**	**2.17**	**2.10**
**Std Deviation**	**1.91**	**1.77**	**2.55**	**6.47**	**1.36**	**0.17**	**1.79**	**1.99**	**1.41**	**2.19**	**1.02**	**0.75**	**0.95**	**1.14**
**Std Error**	**1.10**	**1.02**	**1.47**	**3.73**	**0.79**	**0.10**	**1.04**	**1.15**	**0.82**	**1.26**	**0.59**	**0.43**	**0.55**	**0.66**
US942-1	0.87	1.15	1.06	0.17	0.51	0.81	1.01	2.21	0.55	7.06	0.00	1.06	0.52	0.72
US942-2	2.46	3.99	0.08	0.50	2.00	1.14	2.01	1.04	2.03	2.49	2.37	1.89	2.43	2.08
US942-3	2.19	2.69	1.40	1.95	1.37	1.84	2.27	6.21	2.94	3.04	2.69	2.05	3.12	2.39
**US-942**	**1.84**	**2.61**	**0.85**	**0.87**	**1.29**	**1.26**	**1.76**	**3.15**	**1.84**	**4.20**	**2.53**	**1.67**	**2.02**	**1.73**
**Std Deviation**	**0.85**	**1.42**	**0.69**	**0.95**	**0.75**	**0.53**	**0.67**	**2.71**	**1.21**	**2.50**	**0.22**	**0.53**	**1.34**	**0.89**
**Std Error**	**0.49**	**0.82**	**0.40**	**0.55**	**0.43**	**0.30**	**0.39**	**1.57**	**0.70**	**1.44**	**0.13**	**0.31**	**0.78**	**0.51**
Val-1	2.43	2.20	2.65	3.01	1.87	2.39	2.88	0.00	0.00	0.03	0.00	0.00	0.00	0.00
Val-2	0.00	0.00	0.00	0.06	0.28	0.00	0.00	2.10	2.03	1.76	1.47	2.64	2.74	3.80
Val-3	0.53	0.98	0.49	0.84	0.71	0.65	0.00	2.65	0.50	0.98	0.82	1.00	0.47	0.43
**V**	**1.48**	**1.59**	**1.57**	**1.30**	**0.95**	**1.52**	**2.88**	**2.37**	**1.27**	**0.92**	**0.76**	**1.82**	**1.61**	**2.12**
**Std Deviation**	**1.34**	**0.87**	**1.53**	**1.53**	**0.82**	**1.23**	**#DIV/0!**	**0.39**	**1.08**	**0.87**	**0.74**	**1.16**	**1.60**	**2.38**
**Std Error**	**0.78**	**0.50**	**0.88**	**0.88**	**0.47**	**0.71**	**#DIV/0!**	**0.22**	**0.63**	**0.50**	**0.43**	**0.67**	**0.93**	**1.37**
DG-1	0.32	0.31	0.21	0.26	0.43	0.27	0.43	0.00	0.32	1.05	0.98	1.78	0.89	0.37
DG-2	0.40	0.63	1.18	0.93	0.91	1.11	0.85	0.00	0.79	0.77	0.00	0.86	0.88	0.88
DG-3	4.72	5.21	4.68	6.41	3.18	6.32	3.72	0.00	2.31	4.06	0.29	2.07	2.81	2.03
**DG**	**1.82**	**2.05**	**2.02**	**2.53**	**1.51**	**2.57**	**1.67**	**0.00**	**1.14**	**1.96**	**0.64**	**1.57**	**1.52**	**1.10**
**Std Deviation**	**2.52**	**2.74**	**2.35**	**3.37**	**1.47**	**3.28**	**1.79**	**0.00**	**1.04**	**1.82**	**0.49**	**0.63**	**1.11**	**0.85**
**Std Error**	**1.45**	**1.58**	**1.36**	**1.95**	**0.85**	**1.89**	**1.03**	**0.00**	**0.60**	**1.05**	**0.28**	**0.36**	**0.64**	**0.49**
Rubyred-1	1.93	0.78	2.24	2.69	3.84	4.44	1.56	0.00	2.74	2.79	0.00	2.45	2.54	1.62
Rubyred-2	1.01	1.43	0.73	0.60	1.14	0.68	0.94	0.00	0.72	0.71	1.23	0.94	1.19	1.30
Rubyred-3	2.97	1.98	2.76	0.82	2.42	1.88	2.04	0.00	1.68	0.60	1.42	0.71	2.93	1.92
**RR**	**1.97**	**1.40**	**1.91**	**1.37**	**2.47**	**2.33**	**1.52**	**0.00**	**1.71**	**1.37**	**1.33**	**1.36**	**2.22**	**1.61**
**Std Deviation**	**0.98**	**0.60**	**1.05**	**1.15**	**1.35**	**1.92**	**0.55**	**0.00**	**1.01**	**1.23**	**0.14**	**0.94**	**0.91**	**0.31**
**Std Error**	**0.56**	**0.35**	**0.61**	**0.66**	**0.78**	**1.11**	**0.32**	**0.00**	**0.58**	**0.71**	**0.08**	**0.54**	**0.53**	**0.18**
